# Supplementary material for: The Mechanics of Bioinspired Stiff-to-Compliant Multi-Material 3D-Printed Interfaces
Source: Biomimetics (Basel). 2022 Oct 18;7(4):170. doi: 10.3390/biomimetics7040170 (PMC9624340; doi:10.3390/biomimetics7040170)
Supplement: Supplementary file 1 [file biomimetics-07-00170-s001.zip › biomimetics-1943057-supplementary.pdf]

```
; perimeters extrusion width = 0.45mm
; external perimeters extrusion width = 0.45mm
; infill extrusion width = 0.45mm
; solid infill extrusion width = 0.45mm
; top infill extrusion width = 0.40mm
; first layer extrusion width = 0.42mm

; external perimeters extrusion width = 0.45mm
; perimeters extrusion width = 0.45mm
; infill extrusion width = 0.45mm
; solid infill extrusion width = 0.45mm
; top infill extrusion width = 0.40mm
; first layer extrusion width = 0.42mm

M73 P0 R68
M201 X1250 Y1250 Z400 E5000 ; sets maximum accelerations, mm/sec^2
M203 X180 Y180 Z12 E80 ; sets maximum feedrates, mm/sec
M204 P1250 R1250 T1250 ; sets acceleration (P, T) and retract acceleration (R), mm/sec^2
M205 X8.00 Y8.00 Z2.00 E10.00 ; sets the jerk limits, mm/sec
M205 S0 T0 ; sets the minimum extruding and travel feed rate, mm/sec
M107
;TYPE:Custom
G90 ; use absolute coordinates
M83 ; extruder relative mode
M104 S170 ; set extruder temp for bed leveling
M140 S50 ; set bed temp
M109 R170 ; wait for bed leveling temp
M190 S50 ; wait for bed temp
```

```
G28 ; home all without mesh bed level
G29 ; mesh bed leveling
M104 S240 ; set extruder temp
G92 E0.0
G1 Y-2.0 X179 F2400
G1 Z3 F720
M109 S240 ; wait for extruder temp

; intro line
G1 X170 F1000
G1 Z0.2 F720
G1 X110.0 E8.0 F900
G1 X40.0 E10.0 F700
G92 E0.0

M221 S95 ; set flow
G21 ; set units to millimeters
G90 ; use absolute coordinates
M83 ; use relative distances for extrusion
M600
T0
M900 K0 ; Filament gcode
;LAYER_CHANGE
;Z:0.2
;HEIGHT:0.2
;BEFORE_LAYER_CHANGE
G92 E0.0
;0.2
```

```
G1 E-4.00000 F2400.000
G1 Z0.200 F9000.000
;AFTER_LAYER_CHANGE
;0.2
G1 X18.074 Y38.831
G1 E4.00000 F900.000
M204 S800
;TYPE:Skirt
;WIDTH:0.42
G1 F1074.044
G1 X18.424 Y38.262 E0.02409
G1 X18.918 Y37.812 E0.02409
G1 X19.518 Y37.518 E0.02409
G1 X20.176 Y37.402 E0.02409
G1 X62.258 Y37.401 E1.51738
G1 X163.727 Y38.157 E3.65881
G1 X164.613 Y38.341 E0.03262
G1 X165.363 Y38.841 E0.03249
G1 X165.877 Y39.594 E0.03289
G1 X166.070 Y40.486 E0.03289
G1 X166.078 Y75.483 E1.26190
G1 X166.019 Y137.174 E2.22441
G1 X165.837 Y138.056 E0.03249
G1 X165.338 Y138.810 E0.03262
G1 X164.568 Y139.334 E0.03357
G1 X163.656 Y139.522 E0.03357
G1 X119.638 Y139.522 E1.58717
G1 X68.980 Y139.347 E1.82663
```

```
G1 X20.144 Y138.765 E1.76103
G1 X19.274 Y138.577 E0.03209
G1 X18.534 Y138.082 E0.03209
G1 X18.012 Y137.312 E0.03357
G1 X17.825 Y136.399 E0.03357
G1 X17.877 Y39.772 E3.48416
M73 P1 R68
G1 X18.062 Y38.890 E0.03248
M204 S1250
; printing object tpu print.STL id:19 copy 0
; stop printing object tpu print.STL id:19 copy 0
; printing object Petg print.STL id:18 copy 0
G1 E-4.00000 F2400.000
G1 X40.041 Y45.565 F9000.000
G1 E4.00000 F900.000
M204 S800
;TYPE:Perimeter
M73 P1 R67
G1 F1074.044
G1 X48.074 Y45.565 E0.28963
G1 X48.928 Y45.512 E0.03085
G1 X49.792 Y45.350 E0.03172
G1 X50.631 Y45.083 E0.03172
G1 X51.430 Y44.715 E0.03172
G1 X52.178 Y44.252 E0.03172
G1 X52.863 Y43.700 E0.03172
G1 X53.475 Y43.068 E0.03172
G1 X54.004 Y42.365 E0.03172
G1 X54.443 Y41.603 E0.03172
```

```
G1 X54.785 Y40.792 E0.03172
G1 X54.849 Y40.565 E0.00851
G1 X61.467 Y40.565 E0.23862
G1 X61.467 Y53.991 E0.48410
G1 X54.849 Y53.991 E0.23862
G1 X54.785 Y53.764 E0.00851
G1 X54.443 Y52.953 E0.03172
G1 X54.004 Y52.191 E0.03172
G1 X53.475 Y51.488 E0.03172
G1 X52.863 Y50.856 E0.03172
G1 X52.178 Y50.304 E0.03172
G1 X51.430 Y49.840 E0.03172
G1 X50.631 Y49.472 E0.03172
G1 X49.792 Y49.205 E0.03172
G1 X48.928 Y49.043 E0.03172
G1 X48.074 Y48.991 E0.03085
G1 X40.041 Y48.991 E0.28963
G1 X40.041 Y45.625 E0.12136
M204 S1250
G1 X39.664 Y45.188 F9000.000
M204 S800
;TYPE:External perimeter
G1 F1074.044
G1 X48.062 Y45.188 E0.30280
G1 X48.881 Y45.137 E0.02959
G1 X49.700 Y44.984 E0.03004
G1 X50.494 Y44.731 E0.03004
G1 X51.251 Y44.383 E0.03004
G1 X51.959 Y43.944 E0.03004
```

```
G1 X52.608 Y43.421 E0.03004
G1 X53.187 Y42.822 E0.03004
G1 X53.689 Y42.157 E0.03004
G1 X54.105 Y41.435 E0.03004
G1 X54.429 Y40.667 E0.03004
G1 X54.564 Y40.188 E0.01795
G1 X61.844 Y40.188 E0.26250
G1 X61.844 Y54.368 E0.51129
G1 X54.564 Y54.368 E0.26250
G1 X54.429 Y53.889 E0.01795
G1 X54.105 Y53.121 E0.03004
G1 X53.689 Y52.399 E0.03004
G1 X53.187 Y51.734 E0.03004
G1 X52.608 Y51.135 E0.03004
G1 X51.959 Y50.612 E0.03004
G1 X51.251 Y50.173 E0.03004
G1 X50.494 Y49.825 E0.03004
G1 X49.700 Y49.572 E0.03004
G1 X48.881 Y49.418 E0.03004
G1 X48.062 Y49.368 E0.02959
G1 X39.664 Y49.368 E0.30280
G1 X39.664 Y45.248 E0.14856
M204 S1250
G1 X40.060 Y45.249 F9000.000
G1 E-4.00000 F2400.000
G1 X61.354 Y41.444 F9000.000
G1 E4.00000 F900.000
M204 S800
;TYPE:Solid infill
```

```
;WIDTH:0.42186
G1 F1068.771
G1 X60.758 Y40.848 E0.03057
G1 X60.222 Y40.848 E0.01942
G1 X61.184 Y41.810 E0.04934
G1 X61.184 Y42.346 E0.01942
G1 X59.686 Y40.848 E0.07680
G1 X59.150 Y40.848 E0.01942
G1 X61.184 Y42.882 E0.10426
G1 X61.184 Y43.418 E0.01942
G1 X58.614 Y40.848 E0.13172
G1 X58.078 Y40.848 E0.01942
G1 X61.184 Y43.954 E0.15918
G1 X61.184 Y44.490 E0.01942
G1 X57.542 Y40.848 E0.18665
G1 X57.006 Y40.848 E0.01942
G1 X61.184 Y45.026 E0.21411
G1 X61.184 Y45.562 E0.01942
G1 X56.470 Y40.848 E0.24157
G1 X55.934 Y40.848 E0.01942
G1 X61.184 Y46.098 E0.26903
G1 X61.184 Y46.634 E0.01942
G1 X55.399 Y40.848 E0.29649
G1 X55.063 Y40.848 E0.01215
G1 X55.008 Y40.993 E0.00563
G1 X61.184 Y47.169 E0.31653
G1 X61.184 Y47.705 E0.01942
G1 X54.849 Y41.369 E0.32468
G1 X54.688 Y41.745 E0.01479
```

G1 X61.184 Y48.241 E0.33292

G1 X61.184 Y48.777 E0.01942

G1 X54.492 Y42.085 E0.34295

G1 X54.296 Y42.425 E0.01422

G1 X61.184 Y49.313 E0.35299

G1 X61.184 Y49.849 E0.01942

G1 X54.076 Y42.740 E0.36429

G1 X53.845 Y43.046 E0.01387

G1 X61.184 Y50.385 E0.37609

G1 X61.184 Y50.921 E0.01942

G1 X53.604 Y43.340 E0.38845

G1 X53.340 Y43.613 E0.01373

G1 X61.184 Y51.457 E0.40196

G1 X61.184 Y51.993 E0.01942

G1 X53.077 Y43.885 E0.41547

G1 X52.783 Y44.127 E0.01380

G1 X61.184 Y52.528 E0.43053

G1 X61.184 Y53.064 E0.01942

G1 X52.486 Y44.366 E0.44573

G1 X52.172 Y44.588 E0.01393

G1 X61.184 Y53.600 E0.46184

G1 X61.184 Y53.708 E0.00390

G1 X60.756 Y53.708 E0.01552

G1 X51.841 Y44.793 E0.45684

G1 X51.505 Y44.992 E0.01418

G1 X60.220 Y53.708 E0.44663

G1 X59.684 Y53.708 E0.01942

G1 X51.138 Y45.161 E0.43797

G1 X50.771 Y45.330 E0.01464

```
G1 X59.148 Y53.708 E0.42931
G1 X58.613 Y53.708 E0.01942
G1 X50.368 Y45.464 E0.42247
G1 X49.962 Y45.593 E0.01546
G1 X58.077 Y53.708 E0.41584
G1 X57.541 Y53.708 E0.01942
G1 X49.522 Y45.689 E0.41094
G1 X49.070 Y45.773 E0.01664
G1 X57.005 Y53.708 E0.40661
G1 X56.469 Y53.708 E0.01942
G1 X48.578 Y45.817 E0.40436
G1 X48.073 Y45.848 E0.01834
G1 X55.933 Y53.708 E0.40279
G1 X55.397 Y53.708 E0.01942
G1 X54.481 Y52.792 E0.04696
M204 S1250
G1 X51.384 Y49.695 F9000.000
M204 S800
G1 F1068.771
G1 X47.537 Y45.848 E0.19713
G1 X47.001 Y45.848 E0.01942
G1 X50.188 Y49.034 E0.16330
G1 X49.862 Y48.931 E0.01240
G1 X49.476 Y48.858 E0.01422
G1 X46.465 Y45.848 E0.15428
G1 X45.929 Y45.848 E0.01942
G1 X48.836 Y48.754 E0.14896
G1 X48.265 Y48.719 E0.02074
G1 X45.393 Y45.848 E0.14715
```

G1 X44.857 Y45.848 E0.01942

G1 X47.718 Y48.708 E0.14657

G1 X47.182 Y48.708 E0.01942

G1 X44.321 Y45.848 E0.14657

G1 X43.786 Y45.848 E0.01942

G1 X46.646 Y48.708 E0.14657

G1 X46.110 Y48.708 E0.01942

G1 X43.250 Y45.848 E0.14657

G1 X42.714 Y45.848 E0.01942

G1 X45.574 Y48.708 E0.14657

G1 X45.038 Y48.708 E0.01942

G1 X42.178 Y45.848 E0.14657

G1 X41.642 Y45.848 E0.01942

G1 X44.502 Y48.708 E0.14657

G1 X43.966 Y48.708 E0.01942

G1 X41.106 Y45.848 E0.14657

G1 X40.570 Y45.848 E0.01942

G1 X43.430 Y48.708 E0.14657

G1 X42.895 Y48.708 E0.01942

G1 X40.324 Y46.138 E0.13172

G1 X40.324 Y46.674 E0.01942

G1 X42.359 Y48.708 E0.10425

G1 X41.823 Y48.708 E0.01942

G1 X40.324 Y47.209 E0.07679

G1 X40.324 Y47.745 E0.01942

G1 X41.287 Y48.708 E0.04933

G1 X40.751 Y48.708 E0.01942

G1 X40.154 Y48.112 E0.03056

M204 S1250

```
; stop printing object Petg print.STL id:18 copy 0
; printing object Petg print.STL id:14 copy 0
G1 E-4.00000 F2400.000
G1 X40.154 Y65.514 F9000.000
G1 E4.00000 F900.000
M204 S800
;TYPE:Perimeter
;WIDTH:0.42
G1 F1074.044
G1 X48.081 Y65.514 E0.28582
G1 X48.935 Y65.461 E0.03085
G1 X49.800 Y65.299 E0.03172
G1 X50.638 Y65.032 E0.03172
G1 X51.437 Y64.664 E0.03172
G1 X52.185 Y64.201 E0.03172
G1 X52.870 Y63.649 E0.03172
G1 X53.482 Y63.017 E0.03172
G1 X54.012 Y62.314 E0.03172
G1 X54.451 Y61.552 E0.03172
G1 X54.793 Y60.741 E0.03172
G1 X54.857 Y60.514 E0.00851
G1 X61.475 Y60.514 E0.23862
G1 X61.475 Y73.940 E0.48410
G1 X54.857 Y73.940 E0.23862
G1 X54.793 Y73.713 E0.00851
G1 X54.451 Y72.902 E0.03172
G1 X54.012 Y72.140 E0.03172
G1 X53.482 Y71.437 E0.03172
G1 X52.870 Y70.805 E0.03172
```

```
G1 X52.185 Y70.253 E0.03172
G1 X51.437 Y69.789 E0.03172
G1 X50.638 Y69.421 E0.03172
G1 X49.800 Y69.154 E0.03172
G1 X48.935 Y68.992 E0.03172
G1 X48.081 Y68.940 E0.03085
G1 X40.049 Y68.940 E0.28963
G1 X40.049 Y65.514 E0.12353
G1 X40.094 Y65.514 E0.00164
M204 S1250
G1 X39.672 Y65.137 F9000.000
M204 S800
;TYPE:External perimeter
G1 F1074.044
G1 X48.070 Y65.137 E0.30280
G1 X48.889 Y65.086 E0.02959
G1 X49.708 Y64.933 E0.03004
G1 X50.502 Y64.680 E0.03004
G1 X51.258 Y64.332 E0.03004
G1 X51.967 Y63.892 E0.03004
G1 X52.615 Y63.370 E0.03004
G1 X53.195 Y62.771 E0.03004
G1 X53.696 Y62.106 E0.03004
G1 X54.112 Y61.384 E0.03004
G1 X54.436 Y60.616 E0.03004
G1 X54.572 Y60.137 E0.01795
G1 X61.852 Y60.137 E0.26250
G1 X61.852 Y74.317 E0.51129
M73 P2 R67
```

```
G1 X54.572 Y74.317 E0.26250
G1 X54.436 Y73.838 E0.01795
G1 X54.112 Y73.070 E0.03004
G1 X53.696 Y72.348 E0.03004
G1 X53.195 Y71.683 E0.03004
G1 X52.615 Y71.084 E0.03004
G1 X51.967 Y70.561 E0.03004
G1 X51.258 Y70.122 E0.03004
G1 X50.502 Y69.773 E0.03004
G1 X49.708 Y69.521 E0.03004
G1 X48.889 Y69.367 E0.03004
G1 X48.070 Y69.317 E0.02959
G1 X39.672 Y69.317 E0.30280
G1 X39.672 Y65.197 E0.14856
M204 S1250
G1 X40.067 Y65.198 F9000.000
G1 E-4.00000 F2400.000
G1 X61.362 Y61.393 F9000.000
G1 E4.00000 F900.000
M204 S800
;TYPE:Solid infill
;WIDTH:0.42186
G1 F1068.771
G1 X60.765 Y60.797 E0.03057
G1 X60.229 Y60.797 E0.01942
G1 X61.192 Y61.759 E0.04934
G1 X61.192 Y62.295 E0.01942
G1 X59.693 Y60.797 E0.07680
G1 X59.157 Y60.797 E0.01942
```

```
G1 X61.192 Y62.831 E0.10426
G1 X61.192 Y63.367 E0.01942
G1 X58.621 Y60.797 E0.13172
G1 X58.086 Y60.797 E0.01942
G1 X61.192 Y63.903 E0.15918
G1 X61.192 Y64.439 E0.01942
G1 X57.550 Y60.797 E0.18665
G1 X57.014 Y60.797 E0.01942
G1 X61.192 Y64.975 E0.21411
G1 X61.192 Y65.511 E0.01942
G1 X56.478 Y60.797 E0.24157
G1 X55.942 Y60.797 E0.01942
G1 X61.192 Y66.047 E0.26903
G1 X61.192 Y66.583 E0.01942
G1 X55.406 Y60.797 E0.29649
G1 X55.071 Y60.797 E0.01215
G1 X55.015 Y60.942 E0.00563
G1 X61.192 Y67.118 E0.31653
G1 X61.192 Y67.654 E0.01942
G1 X54.856 Y61.318 E0.32468
G1 X54.695 Y61.694 E0.01479
G1 X61.192 Y68.190 E0.33292
G1 X61.192 Y68.726 E0.01942
G1 X54.499 Y62.034 E0.34295
G1 X54.304 Y62.374 E0.01422
G1 X61.192 Y69.262 E0.35299
G1 X61.192 Y69.798 E0.01942
G1 X54.083 Y62.689 E0.36429
G1 X53.853 Y62.995 E0.01387
```

```
G1 X61.192 Y70.334 E0.37609
G1 X61.192 Y70.870 E0.01942
G1 X53.612 Y63.289 E0.38845
G1 X53.348 Y63.562 E0.01373
G1 X61.192 Y71.406 E0.40196
G1 X61.192 Y71.942 E0.01942
G1 X53.084 Y63.834 E0.41547
G1 X52.790 Y64.076 E0.01380
G1 X61.192 Y72.477 E0.43053
G1 X61.192 Y73.013 E0.01942
G1 X52.494 Y64.315 E0.44573
G1 X52.180 Y64.537 E0.01393
G1 X61.192 Y73.549 E0.46184
G1 X61.192 Y73.657 E0.00390
G1 X60.764 Y73.657 E0.01552
G1 X51.849 Y64.742 E0.45684
G1 X51.512 Y64.941 E0.01418
G1 X60.228 Y73.657 E0.44663
G1 X59.692 Y73.657 E0.01942
G1 X51.145 Y65.110 E0.43797
G1 X50.778 Y65.279 E0.01464
G1 X59.156 Y73.657 E0.42931
G1 X58.620 Y73.657 E0.01942
G1 X50.376 Y65.413 E0.42247
G1 X49.969 Y65.542 E0.01546
G1 X58.084 Y73.657 E0.41584
G1 X57.548 Y73.657 E0.01942
G1 X49.529 Y65.638 E0.41094
G1 X49.078 Y65.722 E0.01664
```

```
G1 X57.012 Y73.657 E0.40661
G1 X56.476 Y73.657 E0.01942
G1 X48.586 Y65.766 E0.40436
G1 X48.080 Y65.797 E0.01834
G1 X55.941 Y73.657 E0.40279
G1 X55.405 Y73.657 E0.01942
G1 X54.488 Y72.740 E0.04696
M204 S1250
G1 X51.391 Y69.644 F9000.000
M204 S800
G1 F1068.771
G1 X47.544 Y65.797 E0.19713
G1 X47.008 Y65.797 E0.01942
G1 X50.195 Y68.983 E0.16330
G1 X49.869 Y68.879 E0.01240
G1 X49.483 Y68.807 E0.01422
G1 X46.473 Y65.797 E0.15428
G1 X45.937 Y65.797 E0.01942
G1 X48.843 Y68.703 E0.14896
G1 X48.272 Y68.668 E0.02074
G1 X45.401 Y65.797 E0.14715
G1 X44.865 Y65.797 E0.01942
G1 X47.725 Y68.657 E0.14657
M73 P2 R66
G1 X47.189 Y68.657 E0.01942
G1 X44.329 Y65.797 E0.14657
G1 X43.793 Y65.797 E0.01942
G1 X46.653 Y68.657 E0.14657
G1 X46.117 Y68.657 E0.01942
```

```
G1 X43.257 Y65.797 E0.14657
G1 X42.721 Y65.797 E0.01942
G1 X45.582 Y68.657 E0.14657
G1 X45.046 Y68.657 E0.01942
G1 X42.185 Y65.797 E0.14657
G1 X41.649 Y65.797 E0.01942
G1 X44.510 Y68.657 E0.14657
G1 X43.974 Y68.657 E0.01942
G1 X41.114 Y65.797 E0.14657
G1 X40.578 Y65.797 E0.01942
G1 X43.438 Y68.657 E0.14657
G1 X42.902 Y68.657 E0.01942
G1 X40.332 Y66.087 E0.13172
G1 X40.332 Y66.622 E0.01942
G1 X42.366 Y68.657 E0.10425
G1 X41.830 Y68.657 E0.01942
G1 X40.332 Y67.158 E0.07679
G1 X40.332 Y67.694 E0.01942
G1 X41.294 Y68.657 E0.04933
G1 X40.758 Y68.657 E0.01942
G1 X40.162 Y68.060 E0.03056
M204 S1250
; stop printing object Petg print.STL id:14 copy 0
; printing object tpu print.STL id:15 copy 0
; stop printing object tpu print.STL id:15 copy 0
; printing object Petg print.STL id:10 copy 0
G1 E-4.00000 F2400.000
G1 X40.162 Y87.706 F9000.000
G1 E4.00000 F900.000
```

```
M204 S800
;TYPE:Perimeter
;WIDTH:0.42
G1 F1074.044
G1 X48.030 Y87.706 E0.28370
G1 X48.884 Y87.653 E0.03085
G1 X49.749 Y87.492 E0.03172
G1 X50.587 Y87.225 E0.03172
G1 X51.386 Y86.857 E0.03172
G1 X52.134 Y86.393 E0.03172
G1 X52.819 Y85.841 E0.03172
G1 X53.431 Y85.209 E0.03172
G1 X53.960 Y84.506 E0.03172
G1 X54.400 Y83.744 E0.03172
G1 X54.742 Y82.933 E0.03172
G1 X54.806 Y82.706 E0.00851
G1 X61.424 Y82.706 E0.23862
G1 X61.424 Y96.132 E0.48410
G1 X54.806 Y96.132 E0.23862
G1 X54.742 Y95.905 E0.00851
G1 X54.400 Y95.094 E0.03172
G1 X53.960 Y94.332 E0.03172
G1 X53.431 Y93.629 E0.03172
G1 X52.819 Y92.997 E0.03172
G1 X52.134 Y92.445 E0.03172
G1 X51.386 Y91.982 E0.03172
G1 X50.587 Y91.614 E0.03172
G1 X49.749 Y91.347 E0.03172
G1 X48.884 Y91.185 E0.03172
```

```
G1 X48.030 Y91.132 E0.03085
G1 X39.998 Y91.132 E0.28963
G1 X39.998 Y87.706 E0.12353
G1 X40.102 Y87.706 E0.00376
M204 S1250
G1 X39.621 Y87.329 F9000.000
M204 S800
;TYPE:External perimeter
G1 F1074.044
G1 X48.018 Y87.329 E0.30280
G1 X48.837 Y87.279 E0.02959
G1 X49.656 Y87.125 E0.03004
G1 X50.450 Y86.872 E0.03004
G1 X51.207 Y86.524 E0.03004
G1 X51.915 Y86.085 E0.03004
G1 X52.564 Y85.562 E0.03004
G1 X53.144 Y84.963 E0.03004
G1 X53.645 Y84.298 E0.03004
G1 X54.061 Y83.576 E0.03004
G1 X54.385 Y82.808 E0.03004
G1 X54.521 Y82.329 E0.01795
G1 X61.801 Y82.329 E0.26250
G1 X61.801 Y96.509 E0.51129
G1 X54.521 Y96.509 E0.26250
G1 X54.385 Y96.030 E0.01795
G1 X54.061 Y95.262 E0.03004
G1 X53.645 Y94.540 E0.03004
G1 X53.144 Y93.875 E0.03004
G1 X52.564 Y93.276 E0.03004
```

```
G1 X51.915 Y92.753 E0.03004
G1 X51.207 Y92.314 E0.03004
G1 X50.450 Y91.966 E0.03004
G1 X49.656 Y91.713 E0.03004
G1 X48.837 Y91.560 E0.03004
G1 X48.018 Y91.509 E0.02959
G1 X39.621 Y91.509 E0.30280
G1 X39.621 Y87.389 E0.14856
M204 S1250
G1 X40.016 Y87.390 F9000.000
G1 E-4.00000 F2400.000
G1 X61.310 Y83.586 F9000.000
G1 E4.00000 F900.000
M204 S800
;TYPE:Solid infill
;WIDTH:0.42186
G1 F1068.771
G1 X60.714 Y82.989 E0.03057
G1 X60.178 Y82.989 E0.01942
G1 X61.141 Y83.952 E0.04934
G1 X61.141 Y84.488 E0.01942
G1 X59.642 Y82.989 E0.07680
G1 X59.106 Y82.989 E0.01942
G1 X61.141 Y85.024 E0.10426
G1 X61.141 Y85.559 E0.01942
G1 X58.570 Y82.989 E0.13172
G1 X58.034 Y82.989 E0.01942
G1 X61.141 Y86.095 E0.15918
G1 X61.141 Y86.631 E0.01942
```

```
G1 X57.498 Y82.989 E0.18665
G1 X56.963 Y82.989 E0.01942
G1 X61.141 Y87.167 E0.21411
G1 X61.141 Y87.703 E0.01942
G1 X56.427 Y82.989 E0.24157
G1 X55.891 Y82.989 E0.01942
G1 X61.141 Y88.239 E0.26903
G1 X61.141 Y88.775 E0.01942
G1 X55.355 Y82.989 E0.29649
G1 X55.020 Y82.989 E0.01215
G1 X54.964 Y83.134 E0.00563
G1 X61.141 Y89.311 E0.31653
G1 X61.141 Y89.847 E0.01942
G1 X54.805 Y83.511 E0.32468
G1 X54.644 Y83.886 E0.01479
G1 X61.141 Y90.383 E0.33292
G1 X61.141 Y90.918 E0.01942
G1 X54.448 Y84.226 E0.34295
G1 X54.252 Y84.566 E0.01422
G1 X61.141 Y91.454 E0.35299
G1 X61.141 Y91.990 E0.01942
G1 X54.032 Y84.881 E0.36429
G1 X53.802 Y85.187 E0.01387
M73 P3 R66
G1 X61.141 Y92.526 E0.37609
G1 X61.141 Y93.062 E0.01942
G1 X53.560 Y85.482 E0.38845
G1 X53.297 Y85.754 E0.01373
G1 X61.141 Y93.598 E0.40196
```

```
G1 X61.141 Y94.134 E0.01942
G1 X53.033 Y86.026 E0.41547
G1 X52.739 Y86.268 E0.01380
G1 X61.141 Y94.670 E0.43053
G1 X61.141 Y95.206 E0.01942
G1 X52.443 Y86.508 E0.44573
G1 X52.128 Y86.729 E0.01393
G1 X61.141 Y95.742 E0.46184
G1 X61.141 Y95.849 E0.00390
G1 X60.713 Y95.849 E0.01552
G1 X51.798 Y86.934 E0.45684
G1 X51.461 Y87.134 E0.01418
G1 X60.177 Y95.849 E0.44663
G1 X59.641 Y95.849 E0.01942
G1 X51.094 Y87.303 E0.43797
G1 X50.727 Y87.472 E0.01464
G1 X59.105 Y95.849 E0.42931
G1 X58.569 Y95.849 E0.01942
G1 X50.325 Y87.605 E0.42247
G1 X49.918 Y87.734 E0.01546
G1 X58.033 Y95.849 E0.41584
G1 X57.497 Y95.849 E0.01942
G1 X49.478 Y87.830 E0.41094
G1 X49.026 Y87.915 E0.01664
G1 X56.961 Y95.849 E0.40661
G1 X56.425 Y95.849 E0.01942
G1 X48.534 Y87.958 E0.40436
G1 X48.029 Y87.989 E0.01834
G1 X55.889 Y95.849 E0.40279
```

G1 X55.353 Y95.849 E0.01942

G1 X54.437 Y94.933 E0.04696

M204 S1250

G1 X51.340 Y91.836 F9000.000

M204 S800

G1 F1068.771

G1 X47.493 Y87.989 E0.19713

G1 X46.957 Y87.989 E0.01942

G1 X50.144 Y91.176 E0.16330

G1 X49.818 Y91.072 E0.01240

G1 X49.432 Y91.000 E0.01422

G1 X46.421 Y87.989 E0.15428

G1 X45.886 Y87.989 E0.01942

G1 X48.792 Y90.896 E0.14896

G1 X48.221 Y90.861 E0.02074

G1 X45.350 Y87.989 E0.14715

G1 X44.814 Y87.989 E0.01942

G1 X47.674 Y90.849 E0.14657

G1 X47.138 Y90.849 E0.01942

G1 X44.278 Y87.989 E0.14657

G1 X43.742 Y87.989 E0.01942

G1 X46.602 Y90.849 E0.14657

G1 X46.066 Y90.849 E0.01942

G1 X43.206 Y87.989 E0.14657

G1 X42.670 Y87.989 E0.01942

G1 X45.530 Y90.849 E0.14657

G1 X44.994 Y90.849 E0.01942

G1 X42.134 Y87.989 E0.14657

G1 X41.598 Y87.989 E0.01942

```
G1 X44.459 Y90.849 E0.14657
G1 X43.923 Y90.849 E0.01942
G1 X41.062 Y87.989 E0.14657
G1 X40.527 Y87.989 E0.01942
G1 X43.387 Y90.849 E0.14657
G1 X42.851 Y90.849 E0.01942
G1 X40.281 Y88.279 E0.13172
G1 X40.281 Y88.815 E0.01942
G1 X42.315 Y90.849 E0.10425
G1 X41.779 Y90.849 E0.01942
G1 X40.281 Y89.351 E0.07679
G1 X40.281 Y89.887 E0.01942
G1 X41.243 Y90.849 E0.04933
G1 X40.707 Y90.849 E0.01942
G1 X40.111 Y90.253 E0.03056
M204 S1250
; stop printing object Petg print.STL id:10 copy 0
; printing object tpu print.STL id:11 copy 0
; stop printing object tpu print.STL id:11 copy 0
; printing object tpu print.STL id:13 copy 0
; stop printing object tpu print.STL id:13 copy 0
; printing object tpu print.STL id:17 copy 0
; stop printing object tpu print.STL id:17 copy 0
; printing object Petg print.STL id:16 copy 0
G1 E-4.00000 F2400.000
G1 X54.798 Y122.176 F9000.000
G1 E4.00000 F900.000
M204 S800
;TYPE:Perimeter
```

```
;WIDTH:0.42
G1 F1074.044
G1 X61.415 Y122.176 E0.23862
G1 X61.415 Y135.602 E0.48410
G1 X54.798 Y135.602 E0.23862
G1 X54.733 Y135.374 E0.00851
G1 X54.391 Y134.564 E0.03172
G1 X53.952 Y133.801 E0.03172
G1 X53.423 Y133.099 E0.03172
G1 X52.811 Y132.467 E0.03172
G1 X52.126 Y131.915 E0.03172
G1 X51.378 Y131.451 E0.03172
G1 X50.579 Y131.083 E0.03172
G1 X49.740 Y130.816 E0.03172
G1 X48.876 Y130.654 E0.03172
G1 X48.022 Y130.602 E0.03085
G1 X39.989 Y130.602 E0.28963
G1 X39.989 Y127.176 E0.12353
G1 X48.022 Y127.176 E0.28963
G1 X48.876 Y127.123 E0.03085
G1 X49.740 Y126.961 E0.03172
G1 X50.579 Y126.694 E0.03172
G1 X51.378 Y126.326 E0.03172
G1 X52.126 Y125.863 E0.03172
G1 X52.811 Y125.310 E0.03172
G1 X53.423 Y124.678 E0.03172
G1 X53.952 Y123.976 E0.03172
G1 X54.391 Y123.213 E0.03172
G1 X54.733 Y122.403 E0.03172
```

```
G1 X54.781 Y122.233 E0.00635
M204 S1250
G1 X54.512 Y121.799 F9000.000
M204 S800
;TYPE:External perimeter
G1 F1074.044
G1 X61.792 Y121.799 E0.26250
G1 X61.792 Y135.979 E0.51129
G1 X54.512 Y135.979 E0.26250
G1 X54.377 Y135.500 E0.01795
G1 X54.053 Y134.732 E0.03004
G1 X53.637 Y134.010 E0.03004
G1 X53.136 Y133.344 E0.03004
G1 X52.556 Y132.746 E0.03004
G1 X51.907 Y132.223 E0.03004
G1 X51.199 Y131.784 E0.03004
G1 X50.442 Y131.435 E0.03004
G1 X49.648 Y131.182 E0.03004
G1 X48.829 Y131.029 E0.03004
G1 X48.010 Y130.979 E0.02959
G1 X39.612 Y130.979 E0.30280
G1 X39.612 Y126.799 E0.15072
G1 X48.010 Y126.799 E0.30280
G1 X48.829 Y126.748 E0.02959
G1 X49.648 Y126.595 E0.03004
G1 X50.442 Y126.342 E0.03004
G1 X51.199 Y125.993 E0.03004
G1 X51.907 Y125.554 E0.03004
G1 X52.556 Y125.031 E0.03004
```

```
G1 X53.136 Y124.433 E0.03004
G1 X53.637 Y123.767 E0.03004
G1 X54.053 Y123.045 E0.03004
G1 X54.377 Y122.278 E0.03004
G1 X54.496 Y121.856 E0.01579
M204 S1250
G1 X54.833 Y122.038 F9000.000
G1 X61.302 Y123.055
M204 S800
;TYPE:Solid infill
;WIDTH:0.42186
G1 F1068.771
G1 X60.706 Y122.458 E0.03057
G1 X60.170 Y122.458 E0.01942
G1 X61.132 Y123.421 E0.04934
G1 X61.132 Y123.957 E0.01942
G1 X59.634 Y122.458 E0.07680
G1 X59.098 Y122.458 E0.01942
G1 X61.132 Y124.493 E0.10426
G1 X61.132 Y125.029 E0.01942
G1 X58.562 Y122.458 E0.13172
G1 X58.026 Y122.458 E0.01942
G1 X61.132 Y125.565 E0.15918
G1 X61.132 Y126.101 E0.01942
G1 X57.490 Y122.458 E0.18665
G1 X56.954 Y122.458 E0.01942
G1 X61.132 Y126.637 E0.21411
G1 X61.132 Y127.173 E0.01942
G1 X56.418 Y122.458 E0.24157
```

```
G1 X55.883 Y122.458 E0.01942
G1 X61.132 Y127.708 E0.26903
G1 X61.132 Y128.244 E0.01942
G1 X55.347 Y122.458 E0.29649
G1 X55.011 Y122.458 E0.01215
G1 X54.956 Y122.603 E0.00563
G1 X61.132 Y128.780 E0.31653
G1 X61.132 Y129.316 E0.01942
G1 X54.797 Y122.980 E0.32468
G1 X54.636 Y123.355 E0.01479
G1 X61.132 Y129.852 E0.33292
G1 X61.132 Y130.388 E0.01942
G1 X54.440 Y123.695 E0.34295
G1 X54.244 Y124.036 E0.01422
G1 X61.132 Y130.924 E0.35299
G1 X61.132 Y131.460 E0.01942
G1 X54.024 Y124.351 E0.36429
G1 X53.793 Y124.656 E0.01387
G1 X61.132 Y131.996 E0.37609
G1 X61.132 Y132.532 E0.01942
G1 X53.552 Y124.951 E0.38845
G1 X53.289 Y125.223 E0.01373
G1 X61.132 Y133.067 E0.40196
G1 X61.132 Y133.603 E0.01942
G1 X53.025 Y125.496 E0.41547
G1 X52.731 Y125.738 E0.01380
G1 X61.132 Y134.139 E0.43053
G1 X61.132 Y134.675 E0.01942
G1 X52.434 Y125.977 E0.44573
```

```
G1 X52.120 Y126.199 E0.01393
G1 X61.132 Y135.211 E0.46184
G1 X61.132 Y135.319 E0.00390
G1 X60.704 Y135.319 E0.01552
G1 X51.789 Y126.404 E0.45684
G1 X51.453 Y126.603 E0.01418
G1 X60.168 Y135.319 E0.44663
G1 X59.632 Y135.319 E0.01942
G1 X51.086 Y126.772 E0.43797
G1 X50.719 Y126.941 E0.01464
G1 X59.097 Y135.319 E0.42931
G1 X58.561 Y135.319 E0.01942
G1 X50.316 Y127.075 E0.42247
G1 X49.910 Y127.204 E0.01546
G1 X58.025 Y135.319 E0.41584
G1 X57.489 Y135.319 E0.01942
G1 X49.470 Y127.300 E0.41094
M73 P4 R66
G1 X49.018 Y127.384 E0.01664
G1 X56.953 Y135.319 E0.40661
M73 P4 R65
G1 X56.417 Y135.319 E0.01942
G1 X48.526 Y127.428 E0.40436
G1 X48.021 Y127.458 E0.01834
G1 X55.881 Y135.319 E0.40279
G1 X55.345 Y135.319 E0.01942
G1 X54.429 Y134.402 E0.04696
M204 S1250
G1 X51.332 Y131.305 F9000.000
```

```
M204 S800
G1 F1068.771
G1 X47.485 Y127.458 E0.19713
G1 X46.949 Y127.458 E0.01942
G1 X50.136 Y130.645 E0.16330
G1 X49.810 Y130.541 E0.01240
G1 X49.424 Y130.469 E0.01422
G1 X46.413 Y127.458 E0.15428
G1 X45.877 Y127.458 E0.01942
G1 X48.784 Y130.365 E0.14896
G1 X48.213 Y130.330 E0.02074
G1 X45.341 Y127.458 E0.14715
G1 X44.805 Y127.458 E0.01942
G1 X47.666 Y130.319 E0.14657
G1 X47.130 Y130.319 E0.01942
G1 X44.270 Y127.458 E0.14657
G1 X43.734 Y127.458 E0.01942
G1 X46.594 Y130.319 E0.14657
G1 X46.058 Y130.319 E0.01942
G1 X43.198 Y127.458 E0.14657
G1 X42.662 Y127.458 E0.01942
G1 X45.522 Y130.319 E0.14657
G1 X44.986 Y130.319 E0.01942
G1 X42.126 Y127.458 E0.14657
G1 X41.590 Y127.458 E0.01942
G1 X44.450 Y130.319 E0.14657
G1 X43.914 Y130.319 E0.01942
G1 X41.054 Y127.458 E0.14657
G1 X40.518 Y127.458 E0.01942
```

```
G1 X43.378 Y130.319 E0.14657
G1 X42.843 Y130.319 E0.01942
G1 X40.272 Y127.748 E0.13172
G1 X40.272 Y128.284 E0.01942
G1 X42.307 Y130.319 E0.10425
G1 X41.771 Y130.319 E0.01942
G1 X40.272 Y128.820 E0.07679
G1 X40.272 Y129.356 E0.01942
G1 X41.235 Y130.319 E0.04933
G1 X40.699 Y130.319 E0.01942
G1 X40.103 Y129.722 E0.03056
M204 S1250
; stop printing object Petg print.STL id:16 copy 0
; printing object Petg print.STL id:12 copy 0
G1 E-4.00000 F2400.000
G1 X40.103 Y111.948 F9000.000
G1 E4.00000 F900.000
M204 S800
;TYPE:Perimeter
;WIDTH:0.42
G1 F1074.044
G1 X40.005 Y111.948 E0.00353
G1 X40.005 Y108.522 E0.12353
G1 X48.037 Y108.522 E0.28963
G1 X48.891 Y108.469 E0.03085
G1 X49.756 Y108.307 E0.03172
G1 X50.594 Y108.040 E0.03172
G1 X51.393 Y107.672 E0.03172
G1 X52.141 Y107.209 E0.03172
```

```
G1 X52.826 Y106.657 E0.03172
G1 X53.438 Y106.024 E0.03172
G1 X53.967 Y105.322 E0.03172
G1 X54.406 Y104.559 E0.03172
G1 X54.748 Y103.749 E0.03172
G1 X54.813 Y103.522 E0.00851
G1 X61.430 Y103.522 E0.23862
G1 X61.430 Y116.948 E0.48410
G1 X54.813 Y116.948 E0.23862
G1 X54.748 Y116.720 E0.00851
G1 X54.406 Y115.910 E0.03172
G1 X53.967 Y115.147 E0.03172
G1 X53.438 Y114.445 E0.03172
G1 X52.826 Y113.813 E0.03172
G1 X52.141 Y113.261 E0.03172
G1 X51.393 Y112.797 E0.03172
G1 X50.594 Y112.429 E0.03172
G1 X49.756 Y112.162 E0.03172
G1 X48.891 Y112.000 E0.03172
G1 X48.037 Y111.948 E0.03085
G1 X40.163 Y111.948 E0.28393
M204 S1250
G1 X39.627 Y112.325 F9000.000
M204 S800
;TYPE:External perimeter
G1 F1074.044
G1 X39.627 Y108.145 E0.15072
G1 X48.025 Y108.145 E0.30280
G1 X48.844 Y108.094 E0.02959
```

```
G1 X49.663 Y107.941 E0.03004
G1 X50.457 Y107.688 E0.03004
G1 X51.214 Y107.339 E0.03004
G1 X51.922 Y106.900 E0.03004
G1 X52.571 Y106.378 E0.03004
G1 X53.151 Y105.779 E0.03004
G1 X53.652 Y105.113 E0.03004
G1 X54.068 Y104.391 E0.03004
G1 X54.392 Y103.624 E0.03004
G1 X54.528 Y103.145 E0.01795
G1 X61.807 Y103.145 E0.26250
G1 X61.807 Y117.325 E0.51129
G1 X54.528 Y117.325 E0.26250
G1 X54.392 Y116.846 E0.01795
G1 X54.068 Y116.078 E0.03004
G1 X53.652 Y115.356 E0.03004
G1 X53.151 Y114.690 E0.03004
G1 X52.571 Y114.092 E0.03004
G1 X51.922 Y113.569 E0.03004
G1 X51.214 Y113.130 E0.03004
G1 X50.457 Y112.781 E0.03004
G1 X49.663 Y112.528 E0.03004
G1 X48.844 Y112.375 E0.03004
G1 X48.025 Y112.325 E0.02959
G1 X39.687 Y112.325 E0.30064
M204 S1250
G1 X39.828 Y111.979 F9000.000
G1 E-4.00000 F2400.000
G1 X61.317 Y104.401 F9000.000
```

```
G1 E4.00000 F900.000
M204 S800
;TYPE:Solid infill
;WIDTH:0.42186
G1 F1068.771
G1 X60.721 Y103.805 E0.03057
G1 X60.185 Y103.805 E0.01942
G1 X61.148 Y104.767 E0.04934
G1 X61.148 Y105.303 E0.01942
G1 X59.649 Y103.805 E0.07680
G1 X59.113 Y103.805 E0.01942
G1 X61.148 Y105.839 E0.10426
G1 X61.148 Y106.375 E0.01942
G1 X58.577 Y103.805 E0.13172
G1 X58.041 Y103.805 E0.01942
G1 X61.148 Y106.911 E0.15918
G1 X61.148 Y107.447 E0.01942
G1 X57.505 Y103.805 E0.18665
G1 X56.969 Y103.805 E0.01942
G1 X61.148 Y107.983 E0.21411
G1 X61.148 Y108.519 E0.01942
G1 X56.434 Y103.805 E0.24157
G1 X55.898 Y103.805 E0.01942
G1 X61.148 Y109.054 E0.26903
G1 X61.148 Y109.590 E0.01942
G1 X55.362 Y103.805 E0.29649
G1 X55.027 Y103.805 E0.01215
G1 X54.971 Y103.949 E0.00563
G1 X61.148 Y110.126 E0.31653
```

```
G1 X61.148 Y110.662 E0.01942
G1 X54.812 Y104.326 E0.32468
G1 X54.651 Y104.701 E0.01479
G1 X61.148 Y111.198 E0.33292
G1 X61.148 Y111.734 E0.01942
G1 X54.455 Y105.042 E0.34295
G1 X54.259 Y105.382 E0.01422
G1 X61.148 Y112.270 E0.35299
G1 X61.148 Y112.806 E0.01942
G1 X54.039 Y105.697 E0.36429
G1 X53.808 Y106.003 E0.01387
G1 X61.148 Y113.342 E0.37609
G1 X61.148 Y113.878 E0.01942
G1 X53.567 Y106.297 E0.38845
G1 X53.304 Y106.570 E0.01373
G1 X61.148 Y114.414 E0.40196
G1 X61.148 Y114.949 E0.01942
G1 X53.040 Y106.842 E0.41547
G1 X52.746 Y107.084 E0.01380
G1 X61.148 Y115.485 E0.43053
G1 X61.148 Y116.021 E0.01942
G1 X52.449 Y107.323 E0.44573
G1 X52.135 Y107.545 E0.01393
G1 X61.148 Y116.557 E0.46184
G1 X61.148 Y116.665 E0.00390
G1 X60.719 Y116.665 E0.01552
G1 X51.804 Y107.750 E0.45684
G1 X51.468 Y107.949 E0.01418
G1 X60.183 Y116.665 E0.44663
```

```
G1 X59.648 Y116.665 E0.01942
G1 X51.101 Y108.118 E0.43797
G1 X50.734 Y108.287 E0.01464
G1 X59.112 Y116.665 E0.42931
G1 X58.576 Y116.665 E0.01942
G1 X50.332 Y108.421 E0.42247
G1 X49.925 Y108.550 E0.01546
G1 X58.040 Y116.665 E0.41584
G1 X57.504 Y116.665 E0.01942
G1 X49.485 Y108.646 E0.41094
G1 X49.033 Y108.730 E0.01664
G1 X56.968 Y116.665 E0.40661
G1 X56.432 Y116.665 E0.01942
G1 X48.541 Y108.774 E0.40436
G1 X48.036 Y108.805 E0.01834
G1 X55.896 Y116.665 E0.40279
G1 X55.360 Y116.665 E0.01942
G1 X54.444 Y115.748 E0.04696
M204 S1250
G1 X51.347 Y112.651 F9000.000
M204 S800
G1 F1068.771
G1 X47.500 Y108.805 E0.19713
G1 X46.964 Y108.805 E0.01942
G1 X50.151 Y111.991 E0.16330
G1 X49.825 Y111.887 E0.01240
G1 X49.439 Y111.815 E0.01422
G1 X46.428 Y108.805 E0.15428
G1 X45.892 Y108.805 E0.01942
```

```
G1 X48.799 Y111.711 E0.14896
G1 X48.228 Y111.676 E0.02074
G1 X45.356 Y108.805 E0.14715
G1 X44.821 Y108.805 E0.01942
G1 X47.681 Y111.665 E0.14657
G1 X47.145 Y111.665 E0.01942
G1 X44.285 Y108.805 E0.14657
G1 X43.749 Y108.805 E0.01942
G1 X46.609 Y111.665 E0.14657
G1 X46.073 Y111.665 E0.01942
G1 X43.213 Y108.805 E0.14657
G1 X42.677 Y108.805 E0.01942
G1 X45.537 Y111.665 E0.14657
G1 X45.001 Y111.665 E0.01942
G1 X42.141 Y108.805 E0.14657
G1 X41.605 Y108.805 E0.01942
G1 X44.465 Y111.665 E0.14657
G1 X43.930 Y111.665 E0.01942
G1 X41.069 Y108.805 E0.14657
G1 X40.533 Y108.805 E0.01942
G1 X43.394 Y111.665 E0.14657
G1 X42.858 Y111.665 E0.01942
G1 X40.287 Y109.094 E0.13172
G1 X40.287 Y109.630 E0.01942
G1 X42.322 Y111.665 E0.10425
G1 X41.786 Y111.665 E0.01942
G1 X40.287 Y110.166 E0.07679
G1 X40.287 Y110.702 E0.01942
G1 X41.250 Y111.665 E0.04933
```

```
G1 X40.714 Y111.665 E0.01942
G1 X40.118 Y111.068 E0.03056
M204 S1250
; stop printing object Petg print.STL id:12 copy 0
; printing object tpu print.STL id:7 copy 0
; stop printing object tpu print.STL id:7 copy 0
; printing object tpu print.STL id:3 copy 0
; stop printing object tpu print.STL id:3 copy 0
; printing object Petg print.STL id:2 copy 0
G1 E-4.00000 F2400.000
G1 X89.794 Y112.528 F9000.000
G1 E4.00000 F900.000
M204 S800
;TYPE:Perimeter
;WIDTH:0.42
G1 F1074.044
G1 X89.794 Y109.103 E0.12353
G1 X97.826 Y109.103 E0.28963
G1 X98.680 Y109.050 E0.03085
G1 X99.545 Y108.888 E0.03172
G1 X100.383 Y108.621 E0.03172
G1 X101.182 Y108.253 E0.03172
G1 X101.930 Y107.789 E0.03172
G1 X102.615 Y107.237 E0.03172
G1 X103.227 Y106.605 E0.03172
G1 X103.757 Y105.903 E0.03172
G1 X104.196 Y105.140 E0.03172
G1 X104.538 Y104.330 E0.03172
G1 X104.602 Y104.103 E0.00851
```

```
G1 X111.220 Y104.103 E0.23862
M73 P5 R65
G1 X111.220 Y117.528 E0.48410
G1 X104.602 Y117.528 E0.23862
G1 X104.538 Y117.301 E0.00851
G1 X104.196 Y116.491 E0.03172
G1 X103.757 Y115.728 E0.03172
G1 X103.227 Y115.026 E0.03172
G1 X102.615 Y114.394 E0.03172
G1 X101.930 Y113.842 E0.03172
G1 X101.182 Y113.378 E0.03172
G1 X100.383 Y113.010 E0.03172
G1 X99.545 Y112.743 E0.03172
G1 X98.680 Y112.581 E0.03172
G1 X97.826 Y112.528 E0.03085
G1 X89.854 Y112.528 E0.28746
M204 S1250
G1 X89.417 Y112.906 F9000.000
M204 S800
;TYPE:External perimeter
G1 F1074.044
G1 X89.417 Y108.726 E0.15072
G1 X97.815 Y108.726 E0.30280
G1 X98.634 Y108.675 E0.02959
G1 X99.453 Y108.522 E0.03004
G1 X100.247 Y108.269 E0.03004
G1 X101.003 Y107.920 E0.03004
G1 X101.712 Y107.481 E0.03004
G1 X102.360 Y106.958 E0.03004
```

```
G1 X102.940 Y106.360 E0.03004
G1 X103.442 Y105.694 E0.03004
G1 X103.857 Y104.972 E0.03004
G1 X104.181 Y104.205 E0.03004
G1 X104.317 Y103.726 E0.01795
G1 X111.597 Y103.726 E0.26250
G1 X111.597 Y117.906 E0.51129
G1 X104.317 Y117.906 E0.26250
G1 X104.181 Y117.426 E0.01795
G1 X103.857 Y116.659 E0.03004
G1 X103.442 Y115.937 E0.03004
G1 X102.940 Y115.271 E0.03004
G1 X102.360 Y114.673 E0.03004
G1 X101.712 Y114.150 E0.03004
G1 X101.003 Y113.711 E0.03004
G1 X100.247 Y113.362 E0.03004
G1 X99.453 Y113.109 E0.03004
G1 X98.634 Y112.956 E0.03004
G1 X97.815 Y112.906 E0.02959
G1 X89.477 Y112.906 E0.30064
M204 S1250
G1 X89.617 Y112.559 F9000.000
G1 E-4.00000 F2400.000
G1 X111.107 Y104.982 F9000.000
G1 E4.00000 F900.000
M204 S800
;TYPE:Solid infill
;WIDTH:0.42186
G1 F1068.771
```

```
G1 X110.510 Y104.385 E0.03057
G1 X109.974 Y104.385 E0.01942
G1 X110.937 Y105.348 E0.04934
G1 X110.937 Y105.884 E0.01942
G1 X109.438 Y104.385 E0.07680
G1 X108.902 Y104.385 E0.01942
G1 X110.937 Y106.420 E0.10426
G1 X110.937 Y106.956 E0.01942
G1 X108.367 Y104.385 E0.13172
G1 X107.831 Y104.385 E0.01942
G1 X110.937 Y107.492 E0.15918
G1 X110.937 Y108.028 E0.01942
G1 X107.295 Y104.385 E0.18665
G1 X106.759 Y104.385 E0.01942
G1 X110.937 Y108.564 E0.21411
G1 X110.937 Y109.099 E0.01942
G1 X106.223 Y104.385 E0.24157
G1 X105.687 Y104.385 E0.01942
G1 X110.937 Y109.635 E0.26903
G1 X110.937 Y110.171 E0.01942
G1 X105.151 Y104.385 E0.29649
G1 X104.816 Y104.385 E0.01215
G1 X104.760 Y104.530 E0.00563
G1 X110.937 Y110.707 E0.31653
G1 X110.937 Y111.243 E0.01942
G1 X104.601 Y104.907 E0.32468
G1 X104.440 Y105.282 E0.01479
G1 X110.937 Y111.779 E0.33292
G1 X110.937 Y112.315 E0.01942
```

```
G1 X104.244 Y105.622 E0.34295
G1 X104.049 Y105.962 E0.01422
G1 X110.937 Y112.851 E0.35299
G1 X110.937 Y113.387 E0.01942
G1 X103.828 Y106.278 E0.36429
G1 X103.598 Y106.583 E0.01387
G1 X110.937 Y113.923 E0.37609
G1 X110.937 Y114.458 E0.01942
G1 X103.357 Y106.878 E0.38845
G1 X103.093 Y107.150 E0.01373
G1 X110.937 Y114.994 E0.40196
G1 X110.937 Y115.530 E0.01942
G1 X102.829 Y107.423 E0.41547
G1 X102.536 Y107.665 E0.01380
G1 X110.937 Y116.066 E0.43053
G1 X110.937 Y116.602 E0.01942
G1 X102.239 Y107.904 E0.44573
G1 X101.925 Y108.126 E0.01393
G1 X110.937 Y117.138 E0.46184
G1 X110.937 Y117.246 E0.00390
G1 X110.509 Y117.246 E0.01552
G1 X101.594 Y108.331 E0.45684
G1 X101.257 Y108.530 E0.01418
G1 X109.973 Y117.246 E0.44663
G1 X109.437 Y117.246 E0.01942
G1 X100.890 Y108.699 E0.43797
M73 P5 R64
G1 X100.523 Y108.868 E0.01464
G1 X108.901 Y117.246 E0.42931
```

```
G1 X108.365 Y117.246 E0.01942
G1 X100.121 Y109.001 E0.42247
G1 X99.714 Y109.131 E0.01546
G1 X107.829 Y117.246 E0.41584
G1 X107.293 Y117.246 E0.01942
G1 X99.274 Y109.226 E0.41094
G1 X98.823 Y109.311 E0.01664
G1 X106.757 Y117.246 E0.40661
G1 X106.221 Y117.246 E0.01942
G1 X98.331 Y109.355 E0.40436
G1 X97.825 Y109.385 E0.01834
G1 X105.686 Y117.246 E0.40279
G1 X105.150 Y117.246 E0.01942
G1 X104.233 Y116.329 E0.04696
M204 S1250
G1 X101.136 Y113.232 F9000.000
M204 S800
G1 F1068.771
G1 X97.289 Y109.385 E0.19713
G1 X96.754 Y109.385 E0.01942
G1 X99.940 Y112.572 E0.16330
G1 X99.614 Y112.468 E0.01240
G1 X99.228 Y112.396 E0.01422
G1 X96.218 Y109.385 E0.15428
G1 X95.682 Y109.385 E0.01942
G1 X98.589 Y112.292 E0.14896
G1 X98.017 Y112.257 E0.02074
G1 X95.146 Y109.385 E0.14715
G1 X94.610 Y109.385 E0.01942
```

```
G1 X97.470 Y112.246 E0.14657
G1 X96.934 Y112.246 E0.01942
G1 X94.074 Y109.385 E0.14657
G1 X93.538 Y109.385 E0.01942
G1 X96.398 Y112.246 E0.14657
G1 X95.862 Y112.246 E0.01942
G1 X93.002 Y109.385 E0.14657
G1 X92.466 Y109.385 E0.01942
G1 X95.327 Y112.246 E0.14657
G1 X94.791 Y112.246 E0.01942
G1 X91.930 Y109.385 E0.14657
G1 X91.395 Y109.385 E0.01942
G1 X94.255 Y112.246 E0.14657
G1 X93.719 Y112.246 E0.01942
G1 X90.859 Y109.385 E0.14657
G1 X90.323 Y109.385 E0.01942
G1 X93.183 Y112.246 E0.14657
G1 X92.647 Y112.246 E0.01942
G1 X90.077 Y109.675 E0.13172
G1 X90.077 Y110.211 E0.01942
G1 X92.111 Y112.246 E0.10425
G1 X91.575 Y112.246 E0.01942
G1 X90.077 Y110.747 E0.07679
G1 X90.077 Y111.283 E0.01942
G1 X91.039 Y112.246 E0.04933
G1 X90.503 Y112.246 E0.01942
G1 X89.907 Y111.649 E0.03056
M204 S1250
; stop printing object Petg print.STL id:2 copy 0
```

```
; printing object Petg print.STL id:6 copy 0
G1 E-4.00000 F2400.000
G1 X89.907 Y127.757 F9000.000
G1 E4.00000 F900.000
M204 S800
;TYPE:Perimeter
;WIDTH:0.42
G1 F1074.044
G1 X97.811 Y127.757 E0.28500
G1 X98.665 Y127.704 E0.03085
G1 X99.530 Y127.542 E0.03172
G1 X100.368 Y127.275 E0.03172
G1 X101.167 Y126.907 E0.03172
G1 X101.915 Y126.443 E0.03172
G1 X102.600 Y125.891 E0.03172
G1 X103.212 Y125.259 E0.03172
G1 X103.742 Y124.557 E0.03172
G1 X104.181 Y123.794 E0.03172
G1 X104.523 Y122.984 E0.03172
G1 X104.587 Y122.757 E0.00851
G1 X111.205 Y122.757 E0.23862
G1 X111.205 Y136.182 E0.48410
G1 X104.587 Y136.182 E0.23862
G1 X104.523 Y135.955 E0.00851
G1 X104.181 Y135.145 E0.03172
G1 X103.742 Y134.382 E0.03172
G1 X103.212 Y133.680 E0.03172
G1 X102.600 Y133.048 E0.03172
G1 X101.915 Y132.496 E0.03172
```

```
G1 X101.167 Y132.032 E0.03172
G1 X100.368 Y131.664 E0.03172
G1 X99.530 Y131.397 E0.03172
G1 X98.665 Y131.235 E0.03172
G1 X97.811 Y131.182 E0.03085
G1 X89.779 Y131.182 E0.28963
G1 X89.779 Y127.757 E0.12353
G1 X89.847 Y127.757 E0.00246
M204 S1250
G1 X89.402 Y127.379 F9000.000
M204 S800
;TYPE:External perimeter
G1 F1074.044
G1 X97.800 Y127.379 E0.30280
G1 X98.619 Y127.329 E0.02959
G1 X99.438 Y127.176 E0.03004
G1 X100.232 Y126.923 E0.03004
G1 X100.988 Y126.574 E0.03004
G1 X101.697 Y126.135 E0.03004
G1 X102.345 Y125.612 E0.03004
G1 X102.925 Y125.014 E0.03004
G1 X103.426 Y124.348 E0.03004
G1 X103.842 Y123.626 E0.03004
G1 X104.166 Y122.859 E0.03004
G1 X104.302 Y122.379 E0.01795
G1 X111.582 Y122.379 E0.26250
G1 X111.582 Y136.559 E0.51129
G1 X104.302 Y136.559 E0.26250
G1 X104.166 Y136.080 E0.01795
```

```
G1 X103.842 Y135.313 E0.03004
G1 X103.426 Y134.591 E0.03004
G1 X102.925 Y133.925 E0.03004
G1 X102.345 Y133.327 E0.03004
G1 X101.697 Y132.804 E0.03004
G1 X100.988 Y132.365 E0.03004
G1 X100.232 Y132.016 E0.03004
G1 X99.438 Y131.763 E0.03004
G1 X98.619 Y131.610 E0.03004
G1 X97.800 Y131.559 E0.02959
G1 X89.402 Y131.559 E0.30280
G1 X89.402 Y127.439 E0.14856
M204 S1250
G1 X89.797 Y127.441 F9000.000
G1 E-4.00000 F2400.000
G1 X111.092 Y123.636 F9000.000
G1 E4.00000 F900.000
M204 S800
;TYPE:Solid infill
;WIDTH:0.42186
G1 F1068.771
G1 X110.495 Y123.039 E0.03057
G1 X109.959 Y123.039 E0.01942
G1 X110.922 Y124.002 E0.04934
G1 X110.922 Y124.538 E0.01942
G1 X109.423 Y123.039 E0.07680
G1 X108.887 Y123.039 E0.01942
G1 X110.922 Y125.074 E0.10426
G1 X110.922 Y125.610 E0.01942
```

G1 X108.351 Y123.039 E0.13172

G1 X107.815 Y123.039 E0.01942

G1 X110.922 Y126.146 E0.15918

G1 X110.922 Y126.682 E0.01942

G1 X107.280 Y123.039 E0.18665

G1 X106.744 Y123.039 E0.01942

G1 X110.922 Y127.218 E0.21411

G1 X110.922 Y127.753 E0.01942

G1 X106.208 Y123.039 E0.24157

G1 X105.672 Y123.039 E0.01942

G1 X110.922 Y128.289 E0.26903

M73 P6 R64

G1 X110.922 Y128.825 E0.01942

G1 X105.136 Y123.039 E0.29649

G1 X104.801 Y123.039 E0.01215

G1 X104.745 Y123.184 E0.00563

G1 X110.922 Y129.361 E0.31653

G1 X110.922 Y129.897 E0.01942

G1 X104.586 Y123.561 E0.32468

G1 X104.425 Y123.936 E0.01479

G1 X110.922 Y130.433 E0.33292

G1 X110.922 Y130.969 E0.01942

G1 X104.229 Y124.276 E0.34295

G1 X104.033 Y124.616 E0.01422

G1 X110.922 Y131.505 E0.35299

G1 X110.922 Y132.041 E0.01942

G1 X103.813 Y124.932 E0.36429

G1 X103.583 Y125.237 E0.01387

G1 X110.922 Y132.577 E0.37609

```
G1 X110.922 Y133.112 E0.01942
G1 X103.341 Y125.532 E0.38845
G1 X103.078 Y125.804 E0.01373
G1 X110.922 Y133.648 E0.40196
G1 X110.922 Y134.184 E0.01942
G1 X102.814 Y126.077 E0.41547
G1 X102.520 Y126.319 E0.01380
G1 X110.922 Y134.720 E0.43053
G1 X110.922 Y135.256 E0.01942
G1 X102.224 Y126.558 E0.44573
G1 X101.909 Y126.780 E0.01393
G1 X110.922 Y135.792 E0.46184
G1 X110.922 Y135.900 E0.00390
G1 X110.494 Y135.900 E0.01552
G1 X101.579 Y126.985 E0.45684
G1 X101.242 Y127.184 E0.01418
G1 X109.958 Y135.900 E0.44663
G1 X109.422 Y135.900 E0.01942
G1 X100.875 Y127.353 E0.43797
G1 X100.508 Y127.522 E0.01464
G1 X108.886 Y135.900 E0.42931
G1 X108.350 Y135.900 E0.01942
G1 X100.106 Y127.655 E0.42247
G1 X99.699 Y127.785 E0.01546
G1 X107.814 Y135.900 E0.41584
G1 X107.278 Y135.900 E0.01942
G1 X99.259 Y127.880 E0.41094
G1 X98.808 Y127.965 E0.01664
G1 X106.742 Y135.900 E0.40661
```

```
G1 X106.206 Y135.900 E0.01942
G1 X98.316 Y128.009 E0.40436
G1 X97.810 Y128.039 E0.01834
G1 X105.670 Y135.900 E0.40279
G1 X105.135 Y135.900 E0.01942
G1 X104.218 Y134.983 E0.04696
M204 S1250
G1 X101.121 Y131.886 F9000.000
M204 S800
G1 F1068.771
G1 X97.274 Y128.039 E0.19713
G1 X96.738 Y128.039 E0.01942
G1 X99.925 Y131.226 E0.16330
G1 X99.599 Y131.122 E0.01240
G1 X99.213 Y131.050 E0.01422
G1 X96.203 Y128.039 E0.15428
G1 X95.667 Y128.039 E0.01942
G1 X98.573 Y130.946 E0.14896
G1 X98.002 Y130.911 E0.02074
G1 X95.131 Y128.039 E0.14715
G1 X94.595 Y128.039 E0.01942
G1 X97.455 Y130.900 E0.14657
G1 X96.919 Y130.900 E0.01942
G1 X94.059 Y128.039 E0.14657
G1 X93.523 Y128.039 E0.01942
G1 X96.383 Y130.900 E0.14657
G1 X95.847 Y130.900 E0.01942
G1 X92.987 Y128.039 E0.14657
G1 X92.451 Y128.039 E0.01942
```

```
G1 X95.311 Y130.900 E0.14657
G1 X94.776 Y130.900 E0.01942
G1 X91.915 Y128.039 E0.14657
G1 X91.379 Y128.039 E0.01942
G1 X94.240 Y130.900 E0.14657
G1 X93.704 Y130.900 E0.01942
G1 X90.844 Y128.039 E0.14657
G1 X90.308 Y128.039 E0.01942
G1 X93.168 Y130.900 E0.14657
G1 X92.632 Y130.900 E0.01942
G1 X90.062 Y128.329 E0.13172
G1 X90.062 Y128.865 E0.01942
G1 X92.096 Y130.900 E0.10425
G1 X91.560 Y130.900 E0.01942
G1 X90.062 Y129.401 E0.07679
G1 X90.062 Y129.937 E0.01942
G1 X91.024 Y130.900 E0.04933
G1 X90.488 Y130.900 E0.01942
G1 X89.892 Y130.303 E0.03056
M204 S1250
; stop printing object Petg print.STL id:6 copy 0
; printing object tpu print.STL id:27 copy 0
; stop printing object tpu print.STL id:27 copy 0
; printing object tpu print.STL id:23 copy 0
; stop printing object tpu print.STL id:23 copy 0
; printing object tpu print.STL id:21 copy 0
; stop printing object tpu print.STL id:21 copy 0
; printing object Petg print.STL id:20 copy 0
G1 E-4.00000 F2400.000
```

```
G1 X141.437 Y91.888 F9000.000
G1 E4.00000 F900.000
M204 S800
;TYPE:Perimeter
;WIDTH:0.42
G1 F1074.044
G1 X141.437 Y88.462 E0.12353
G1 X149.470 Y88.462 E0.28963
G1 X150.324 Y88.409 E0.03085
G1 X151.188 Y88.248 E0.03172
G1 X152.027 Y87.981 E0.03172
G1 X152.826 Y87.613 E0.03172
G1 X153.574 Y87.149 E0.03172
G1 X154.259 Y86.597 E0.03172
G1 X154.871 Y85.965 E0.03172
G1 X155.400 Y85.262 E0.03172
G1 X155.839 Y84.500 E0.03172
G1 X156.181 Y83.689 E0.03172
G1 X156.245 Y83.462 E0.00851
G1 X162.863 Y83.462 E0.23862
G1 X162.863 Y96.888 E0.48410
G1 X156.245 Y96.888 E0.23862
G1 X156.181 Y96.661 E0.00851
G1 X155.839 Y95.850 E0.03172
G1 X155.400 Y95.088 E0.03172
G1 X154.871 Y94.385 E0.03172
G1 X154.259 Y93.753 E0.03172
G1 X153.574 Y93.201 E0.03172
G1 X152.826 Y92.738 E0.03172
```

```
G1 X152.027 Y92.370 E0.03172
G1 X151.188 Y92.103 E0.03172
G1 X150.324 Y91.941 E0.03172
G1 X149.470 Y91.888 E0.03085
G1 X141.497 Y91.888 E0.28746
M204 S1250
G1 X141.060 Y92.265 F9000.000
M204 S800
;TYPE:External perimeter
G1 F1074.044
G1 X141.060 Y88.085 E0.15072
G1 X149.458 Y88.085 E0.30280
G1 X150.277 Y88.035 E0.02959
G1 X151.096 Y87.881 E0.03004
G1 X151.890 Y87.628 E0.03004
G1 X152.647 Y87.280 E0.03004
G1 X153.355 Y86.841 E0.03004
G1 X154.004 Y86.318 E0.03004
G1 X154.583 Y85.719 E0.03004
G1 X155.085 Y85.054 E0.03004
G1 X155.501 Y84.332 E0.03004
G1 X155.825 Y83.564 E0.03004
G1 X155.960 Y83.085 E0.01795
G1 X163.240 Y83.085 E0.26250
G1 X163.240 Y97.265 E0.51129
G1 X155.960 Y97.265 E0.26250
G1 X155.825 Y96.786 E0.01795
G1 X155.501 Y96.018 E0.03004
G1 X155.085 Y95.296 E0.03004
```

```
G1 X154.583 Y94.631 E0.03004
G1 X154.004 Y94.032 E0.03004
G1 X153.355 Y93.509 E0.03004
G1 X152.647 Y93.070 E0.03004
G1 X151.890 Y92.722 E0.03004
G1 X151.096 Y92.469 E0.03004
G1 X150.277 Y92.316 E0.03004
G1 X149.458 Y92.265 E0.02959
G1 X141.120 Y92.265 E0.30064
M204 S1250
G1 X141.261 Y91.919 F9000.000
G1 E-4.00000 F2400.000
G1 X162.750 Y84.341 F9000.000
G1 E4.00000 F900.000
M204 S800
;TYPE:Solid infill
;WIDTH:0.42186
G1 F1068.771
G1 X162.154 Y83.745 E0.03057
G1 X161.618 Y83.745 E0.01942
G1 X162.580 Y84.708 E0.04934
G1 X162.580 Y85.244 E0.01942
G1 X161.082 Y83.745 E0.07680
G1 X160.546 Y83.745 E0.01942
G1 X162.580 Y85.780 E0.10426
G1 X162.580 Y86.315 E0.01942
G1 X160.010 Y83.745 E0.13172
G1 X159.474 Y83.745 E0.01942
G1 X162.580 Y86.851 E0.15918
```

```
G1 X162.580 Y87.387 E0.01942
G1 X158.938 Y83.745 E0.18665
G1 X158.402 Y83.745 E0.01942
G1 X162.580 Y87.923 E0.21411
G1 X162.580 Y88.459 E0.01942
G1 X157.866 Y83.745 E0.24157
G1 X157.330 Y83.745 E0.01942
G1 X162.580 Y88.995 E0.26903
G1 X162.580 Y89.531 E0.01942
G1 X156.795 Y83.745 E0.29649
G1 X156.459 Y83.745 E0.01215
G1 X156.403 Y83.890 E0.00563
G1 X162.580 Y90.067 E0.31653
G1 X162.580 Y90.603 E0.01942
G1 X156.244 Y84.267 E0.32468
G1 X156.084 Y84.642 E0.01479
G1 X162.580 Y91.139 E0.33292
G1 X162.580 Y91.674 E0.01942
G1 X155.888 Y84.982 E0.34295
G1 X155.692 Y85.322 E0.01422
G1 X162.580 Y92.210 E0.35299
G1 X162.580 Y92.746 E0.01942
G1 X155.471 Y85.637 E0.36429
G1 X155.241 Y85.943 E0.01387
G1 X162.580 Y93.282 E0.37609
G1 X162.580 Y93.818 E0.01942
G1 X155.000 Y86.238 E0.38845
G1 X154.736 Y86.510 E0.01373
G1 X162.580 Y94.354 E0.40196
```

```
G1 X162.580 Y94.890 E0.01942
G1 X154.473 Y86.782 E0.41547
G1 X154.179 Y87.024 E0.01380
G1 X162.580 Y95.426 E0.43053
M73 P6 R63
G1 X162.580 Y95.962 E0.01942
G1 X153.882 Y87.263 E0.44573
G1 X153.568 Y87.485 E0.01393
G1 X162.580 Y96.498 E0.46184
G1 X162.580 Y96.605 E0.00390
G1 X162.152 Y96.605 E0.01552
G1 X153.237 Y87.690 E0.45684
M73 P7 R63
G1 X152.901 Y87.889 E0.01418
G1 X161.616 Y96.605 E0.44663
G1 X161.080 Y96.605 E0.01942
G1 X152.534 Y88.058 E0.43797
G1 X152.167 Y88.227 E0.01464
G1 X160.544 Y96.605 E0.42931
G1 X160.009 Y96.605 E0.01942
G1 X151.764 Y88.361 E0.42247
G1 X151.358 Y88.490 E0.01546
G1 X159.473 Y96.605 E0.41584
G1 X158.937 Y96.605 E0.01942
G1 X150.918 Y88.586 E0.41094
G1 X150.466 Y88.670 E0.01664
G1 X158.401 Y96.605 E0.40661
G1 X157.865 Y96.605 E0.01942
G1 X149.974 Y88.714 E0.40436
```

```
G1 X149.469 Y88.745 E0.01834
G1 X157.329 Y96.605 E0.40279
G1 X156.793 Y96.605 E0.01942
G1 X155.877 Y95.689 E0.04696
M204 S1250
G1 X152.780 Y92.592 F9000.000
M204 S800
G1 F1068.771
G1 X148.933 Y88.745 E0.19713
G1 X148.397 Y88.745 E0.01942
G1 X151.584 Y91.932 E0.16330
G1 X151.258 Y91.828 E0.01240
G1 X150.872 Y91.756 E0.01422
G1 X147.861 Y88.745 E0.15428
G1 X147.325 Y88.745 E0.01942
G1 X150.232 Y91.652 E0.14896
G1 X149.661 Y91.616 E0.02074
G1 X146.789 Y88.745 E0.14715
G1 X146.253 Y88.745 E0.01942
G1 X149.114 Y91.605 E0.14657
G1 X148.578 Y91.605 E0.01942
G1 X145.717 Y88.745 E0.14657
G1 X145.182 Y88.745 E0.01942
G1 X148.042 Y91.605 E0.14657
G1 X147.506 Y91.605 E0.01942
G1 X144.646 Y88.745 E0.14657
G1 X144.110 Y88.745 E0.01942
G1 X146.970 Y91.605 E0.14657
G1 X146.434 Y91.605 E0.01942
```

G1 X143.574 Y88.745 E0.14657

G1 X143.038 Y88.745 E0.01942

G1 X145.898 Y91.605 E0.14657

G1 X145.362 Y91.605 E0.01942

G1 X142.502 Y88.745 E0.14657

G1 X141.966 Y88.745 E0.01942

G1 X144.826 Y91.605 E0.14657

G1 X144.290 Y91.605 E0.01942

G1 X141.720 Y89.035 E0.13172

G1 X141.720 Y89.571 E0.01942

G1 X143.755 Y91.605 E0.10425

G1 X143.219 Y91.605 E0.01942

G1 X141.720 Y90.107 E0.07679

G1 X141.720 Y90.643 E0.01942

G1 X142.683 Y91.605 E0.04933

G1 X142.147 Y91.605 E0.01942

G1 X141.550 Y91.009 E0.03056

M204 S1250

; stop printing object Petg print.STL id:20 copy 0

; printing object Petg print.STL id:22 copy 0

G1 E-4.00000 F2400.000

G1 X141.550 Y109.278 F9000.000

G1 E4.00000 F900.000

M204 S800

;TYPE:Perimeter

;WIDTH:0.42

G1 F1074.044

G1 X149.477 Y109.278 E0.28579

G1 X150.330 Y109.225 E0.03085

G1 X151.195 Y109.063 E0.03172

G1 X152.033 Y108.796 E0.03172

G1 X152.833 Y108.428 E0.03172

G1 X153.580 Y107.965 E0.03172

G1 X154.265 Y107.412 E0.03172

G1 X154.877 Y106.780 E0.03172

G1 X155.407 Y106.078 E0.03172

G1 X155.846 Y105.315 E0.03172

G1 X156.188 Y104.505 E0.03172

G1 X156.252 Y104.278 E0.00851

G1 X162.870 Y104.278 E0.23862

G1 X162.870 Y117.704 E0.48410

G1 X156.252 Y117.704 E0.23862

G1 X156.188 Y117.476 E0.00851

G1 X155.846 Y116.666 E0.03172

G1 X155.407 Y115.903 E0.03172

G1 X154.877 Y115.201 E0.03172

G1 X154.265 Y114.569 E0.03172

G1 X153.580 Y114.017 E0.03172

G1 X152.833 Y113.553 E0.03172

G1 X152.033 Y113.185 E0.03172

G1 X151.195 Y112.918 E0.03172

G1 X150.330 Y112.756 E0.03172

G1 X149.477 Y112.704 E0.03085

G1 X141.444 Y112.704 E0.28963

G1 X141.444 Y109.278 E0.12353

G1 X141.490 Y109.278 E0.00167

M204 S1250

G1 X141.067 Y108.901 F9000.000

```
M204 S800
;TYPE:External perimeter
G1 F1074.044
G1 X149.465 Y108.901 E0.30280
G1 X150.284 Y108.850 E0.02959
G1 X151.103 Y108.697 E0.03004
G1 X151.897 Y108.444 E0.03004
G1 X152.654 Y108.095 E0.03004
G1 X153.362 Y107.656 E0.03004
G1 X154.011 Y107.133 E0.03004
G1 X154.590 Y106.535 E0.03004
G1 X155.092 Y105.869 E0.03004
G1 X155.508 Y105.147 E0.03004
G1 X155.832 Y104.380 E0.03004
G1 X155.967 Y103.901 E0.01795
G1 X163.247 Y103.901 E0.26250
G1 X163.247 Y118.081 E0.51129
G1 X155.967 Y118.081 E0.26250
G1 X155.832 Y117.602 E0.01795
G1 X155.508 Y116.834 E0.03004
G1 X155.092 Y116.112 E0.03004
G1 X154.590 Y115.446 E0.03004
G1 X154.011 Y114.848 E0.03004
G1 X153.362 Y114.325 E0.03004
G1 X152.654 Y113.886 E0.03004
G1 X151.897 Y113.537 E0.03004
G1 X151.103 Y113.284 E0.03004
G1 X150.284 Y113.131 E0.03004
G1 X149.465 Y113.081 E0.02959
```

G1 X141.067 Y113.081 E0.30280

G1 X141.067 Y108.961 E0.14856

M204 S1250

G1 X141.462 Y108.962 F9000.000

G1 E-4.00000 F2400.000

G1 X162.757 Y105.157 F9000.000

G1 E4.00000 F900.000

M204 S800

;TYPE:Solid infill

;WIDTH:0.42186

G1 F1068.771

G1 X162.160 Y104.560 E0.03057

G1 X161.624 Y104.560 E0.01942

G1 X162.587 Y105.523 E0.04934

G1 X162.587 Y106.059 E0.01942

G1 X161.089 Y104.560 E0.07680

G1 X160.553 Y104.560 E0.01942

G1 X162.587 Y106.595 E0.10426

G1 X162.587 Y107.131 E0.01942

G1 X160.017 Y104.560 E0.13172

G1 X159.481 Y104.560 E0.01942

G1 X162.587 Y107.667 E0.15918

G1 X162.587 Y108.203 E0.01942

G1 X158.945 Y104.560 E0.18665

G1 X158.409 Y104.560 E0.01942

G1 X162.587 Y108.739 E0.21411

G1 X162.587 Y109.275 E0.01942

G1 X157.873 Y104.560 E0.24157

G1 X157.337 Y104.560 E0.01942

G1 X162.587 Y109.810 E0.26903

G1 X162.587 Y110.346 E0.01942

G1 X156.801 Y104.560 E0.29649

G1 X156.466 Y104.560 E0.01215

G1 X156.410 Y104.705 E0.00563

G1 X162.587 Y110.882 E0.31653

G1 X162.587 Y111.418 E0.01942

G1 X156.251 Y105.082 E0.32468

G1 X156.091 Y105.457 E0.01479

G1 X162.587 Y111.954 E0.33292

G1 X162.587 Y112.490 E0.01942

G1 X155.895 Y105.797 E0.34295

G1 X155.699 Y106.138 E0.01422

G1 X162.587 Y113.026 E0.35299

G1 X162.587 Y113.562 E0.01942

G1 X155.478 Y106.453 E0.36429

G1 X155.248 Y106.758 E0.01387

G1 X162.587 Y114.098 E0.37609

G1 X162.587 Y114.634 E0.01942

G1 X155.007 Y107.053 E0.38845

G1 X154.743 Y107.325 E0.01373

G1 X162.587 Y115.169 E0.40196

G1 X162.587 Y115.705 E0.01942

G1 X154.480 Y107.598 E0.41547

G1 X154.186 Y107.840 E0.01380

G1 X162.587 Y116.241 E0.43053

G1 X162.587 Y116.777 E0.01942

G1 X153.889 Y108.079 E0.44573

G1 X153.575 Y108.301 E0.01393

G1 X162.587 Y117.313 E0.46184

G1 X162.587 Y117.421 E0.00390

G1 X162.159 Y117.421 E0.01552

G1 X153.244 Y108.506 E0.45684

G1 X152.907 Y108.705 E0.01418

G1 X161.623 Y117.421 E0.44663

G1 X161.087 Y117.421 E0.01942

G1 X152.540 Y108.874 E0.43797

G1 X152.174 Y109.043 E0.01464

G1 X160.551 Y117.421 E0.42931

G1 X160.015 Y117.421 E0.01942

G1 X151.771 Y109.177 E0.42247

G1 X151.365 Y109.306 E0.01546

G1 X159.479 Y117.421 E0.41584

G1 X158.944 Y117.421 E0.01942

G1 X150.924 Y109.402 E0.41094

G1 X150.473 Y109.486 E0.01664

G1 X158.408 Y117.421 E0.40661

G1 X157.872 Y117.421 E0.01942

G1 X149.981 Y109.530 E0.40436

G1 X149.476 Y109.560 E0.01834

G1 X157.336 Y117.421 E0.40279

G1 X156.800 Y117.421 E0.01942

G1 X155.884 Y116.504 E0.04696

M204 S1250

G1 X152.787 Y113.407 F9000.000

M204 S800

G1 F1068.771

G1 X148.940 Y109.560 E0.19713

```
G1 X148.404 Y109.560 E0.01942
G1 X151.590 Y112.747 E0.16330
G1 X151.264 Y112.643 E0.01240
G1 X150.879 Y112.571 E0.01422
G1 X147.868 Y109.560 E0.15428
G1 X147.332 Y109.560 E0.01942
G1 X150.239 Y112.467 E0.14896
G1 X149.668 Y112.432 E0.02074
G1 X146.796 Y109.560 E0.14715
G1 X146.260 Y109.560 E0.01942
G1 X149.120 Y112.421 E0.14657
G1 X148.585 Y112.421 E0.01942
G1 X145.724 Y109.560 E0.14657
G1 X145.188 Y109.560 E0.01942
G1 X148.049 Y112.421 E0.14657
M73 P8 R63
G1 X147.513 Y112.421 E0.01942
G1 X144.652 Y109.560 E0.14657
G1 X144.117 Y109.560 E0.01942
G1 X146.977 Y112.421 E0.14657
G1 X146.441 Y112.421 E0.01942
G1 X143.581 Y109.560 E0.14657
G1 X143.045 Y109.560 E0.01942
G1 X145.905 Y112.421 E0.14657
G1 X145.369 Y112.421 E0.01942
G1 X142.509 Y109.560 E0.14657
G1 X141.973 Y109.560 E0.01942
G1 X144.833 Y112.421 E0.14657
G1 X144.297 Y112.421 E0.01942
```

G1 X141.727 Y109.850 E0.13172

G1 X141.727 Y110.386 E0.01942

G1 X143.761 Y112.421 E0.10425

G1 X143.226 Y112.421 E0.01942

G1 X141.727 Y110.922 E0.07679

G1 X141.727 Y111.458 E0.01942

G1 X142.690 Y112.421 E0.04933

G1 X142.154 Y112.421 E0.01942

G1 X141.557 Y111.824 E0.03056

M204 S1250

; stop printing object Petg print.STL id:22 copy 0

; printing object Petg print.STL id:26 copy 0

G1 E-4.00000 F2400.000

G1 X141.557 Y127.932 F9000.000

G1 E4.00000 F900.000

M204 S800

;TYPE:Perimeter

;WIDTH:0.42

G1 F1074.044

G1 X149.461 Y127.932 E0.28500

G1 X150.315 Y127.879 E0.03085

G1 X151.180 Y127.717 E0.03172

G1 X152.018 Y127.450 E0.03172

G1 X152.817 Y127.082 E0.03172

G1 X153.565 Y126.618 E0.03172

G1 X154.250 Y126.066 E0.03172

G1 X154.862 Y125.434 E0.03172

G1 X155.392 Y124.732 E0.03172

G1 X155.831 Y123.969 E0.03172

```
G1 X156.173 Y123.159 E0.03172
G1 X156.237 Y122.932 E0.00851
G1 X162.855 Y122.932 E0.23862
G1 X162.855 Y136.357 E0.48410
G1 X156.237 Y136.357 E0.23862
G1 X156.173 Y136.130 E0.00851
G1 X155.831 Y135.320 E0.03172
G1 X155.392 Y134.557 E0.03172
G1 X154.862 Y133.855 E0.03172
G1 X154.250 Y133.223 E0.03172
G1 X153.565 Y132.671 E0.03172
G1 X152.817 Y132.207 E0.03172
G1 X152.018 Y131.839 E0.03172
G1 X151.180 Y131.572 E0.03172
G1 X150.315 Y131.410 E0.03172
G1 X149.461 Y131.357 E0.03085
G1 X141.429 Y131.357 E0.28963
G1 X141.429 Y127.932 E0.12353
G1 X141.497 Y127.932 E0.00246
M204 S1250
G1 X141.052 Y127.555 F9000.000
M204 S800
;TYPE:External perimeter
G1 F1074.044
G1 X149.450 Y127.555 E0.30280
G1 X150.269 Y127.504 E0.02959
G1 X151.088 Y127.351 E0.03004
G1 X151.882 Y127.098 E0.03004
G1 X152.639 Y126.749 E0.03004
```

```
G1 X153.347 Y126.310 E0.03004
G1 X153.996 Y125.787 E0.03004
G1 X154.575 Y125.189 E0.03004
G1 X155.077 Y124.523 E0.03004
G1 X155.493 Y123.801 E0.03004
G1 X155.816 Y123.034 E0.03004
G1 X155.952 Y122.555 E0.01795
G1 X163.232 Y122.555 E0.26250
G1 X163.232 Y136.735 E0.51129
G1 X155.952 Y136.735 E0.26250
G1 X155.816 Y136.255 E0.01795
G1 X155.493 Y135.488 E0.03004
G1 X155.077 Y134.766 E0.03004
G1 X154.575 Y134.100 E0.03004
G1 X153.996 Y133.502 E0.03004
G1 X153.347 Y132.979 E0.03004
G1 X152.639 Y132.540 E0.03004
G1 X151.882 Y132.191 E0.03004
G1 X151.088 Y131.938 E0.03004
G1 X150.269 Y131.785 E0.03004
G1 X149.450 Y131.735 E0.02959
G1 X141.052 Y131.735 E0.30280
G1 X141.052 Y127.615 E0.14856
M204 S1250
G1 X141.447 Y127.616 F9000.000
G1 E-4.00000 F2400.000
G1 X162.742 Y123.811 F9000.000
G1 E4.00000 F900.000
M204 S800
```

```
;TYPE:Solid infill
;WIDTH:0.42186
G1 F1068.771
G1 X162.145 Y123.214 E0.03057
G1 X161.609 Y123.214 E0.01942
G1 X162.572 Y124.177 E0.04934
G1 X162.572 Y124.713 E0.01942
G1 X161.073 Y123.214 E0.07680
G1 X160.538 Y123.214 E0.01942
G1 X162.572 Y125.249 E0.10426
G1 X162.572 Y125.785 E0.01942
G1 X160.002 Y123.214 E0.13172
G1 X159.466 Y123.214 E0.01942
G1 X162.572 Y126.321 E0.15918
G1 X162.572 Y126.857 E0.01942
G1 X158.930 Y123.214 E0.18665
G1 X158.394 Y123.214 E0.01942
G1 X162.572 Y127.393 E0.21411
G1 X162.572 Y127.928 E0.01942
G1 X157.858 Y123.214 E0.24157
G1 X157.322 Y123.214 E0.01942
G1 X162.572 Y128.464 E0.26903
G1 X162.572 Y129.000 E0.01942
G1 X156.786 Y123.214 E0.29649
G1 X156.451 Y123.214 E0.01215
G1 X156.395 Y123.359 E0.00563
G1 X162.572 Y129.536 E0.31653
G1 X162.572 Y130.072 E0.01942
G1 X156.236 Y123.736 E0.32468
```

G1 X156.075 Y124.111 E0.01479

G1 X162.572 Y130.608 E0.33292

G1 X162.572 Y131.144 E0.01942

G1 X155.880 Y124.451 E0.34295

G1 X155.684 Y124.791 E0.01422

G1 X162.572 Y131.680 E0.35299

M73 P8 R62

G1 X162.572 Y132.216 E0.01942

G1 X155.463 Y125.107 E0.36429

G1 X155.233 Y125.412 E0.01387

G1 X162.572 Y132.752 E0.37609

G1 X162.572 Y133.288 E0.01942

G1 X154.992 Y125.707 E0.38845

G1 X154.728 Y125.979 E0.01373

G1 X162.572 Y133.823 E0.40196

G1 X162.572 Y134.359 E0.01942

G1 X154.465 Y126.252 E0.41547

G1 X154.171 Y126.494 E0.01380

G1 X162.572 Y134.895 E0.43053

G1 X162.572 Y135.431 E0.01942

G1 X153.874 Y126.733 E0.44573

G1 X153.560 Y126.955 E0.01393

G1 X162.572 Y135.967 E0.46184

G1 X162.572 Y136.075 E0.00390

G1 X162.144 Y136.075 E0.01552

G1 X153.229 Y127.160 E0.45684

G1 X152.892 Y127.359 E0.01418

G1 X161.608 Y136.075 E0.44663

G1 X161.072 Y136.075 E0.01942

```
G1 X152.525 Y127.528 E0.43797
G1 X152.158 Y127.697 E0.01464
G1 X160.536 Y136.075 E0.42931
G1 X160.000 Y136.075 E0.01942
G1 X151.756 Y127.830 E0.42247
G1 X151.350 Y127.960 E0.01546
G1 X159.464 Y136.075 E0.41584
G1 X158.928 Y136.075 E0.01942
G1 X150.909 Y128.055 E0.41094
G1 X150.458 Y128.140 E0.01664
G1 X158.393 Y136.075 E0.40661
G1 X157.857 Y136.075 E0.01942
G1 X149.966 Y128.184 E0.40436
G1 X149.460 Y128.214 E0.01834
G1 X157.321 Y136.075 E0.40279
G1 X156.785 Y136.075 E0.01942
G1 X155.868 Y135.158 E0.04696
M204 S1250
G1 X152.771 Y132.061 F9000.000
M204 S800
G1 F1068.771
G1 X148.925 Y128.214 E0.19713
G1 X148.389 Y128.214 E0.01942
G1 X151.575 Y131.401 E0.16330
G1 X151.249 Y131.297 E0.01240
G1 X150.863 Y131.225 E0.01422
G1 X147.853 Y128.214 E0.15428
G1 X147.317 Y128.214 E0.01942
G1 X150.224 Y131.121 E0.14896
```

G1 X149.652 Y131.086 E0.02074

G1 X146.781 Y128.214 E0.14715

G1 X146.245 Y128.214 E0.01942

G1 X149.105 Y131.075 E0.14657

G1 X148.569 Y131.075 E0.01942

G1 X145.709 Y128.214 E0.14657

G1 X145.173 Y128.214 E0.01942

G1 X148.034 Y131.075 E0.14657

G1 X147.498 Y131.075 E0.01942

G1 X144.637 Y128.214 E0.14657

G1 X144.101 Y128.214 E0.01942

G1 X146.962 Y131.075 E0.14657

G1 X146.426 Y131.075 E0.01942

G1 X143.566 Y128.214 E0.14657

G1 X143.030 Y128.214 E0.01942

G1 X145.890 Y131.075 E0.14657

G1 X145.354 Y131.075 E0.01942

G1 X142.494 Y128.214 E0.14657

G1 X141.958 Y128.214 E0.01942

G1 X144.818 Y131.075 E0.14657

G1 X144.282 Y131.075 E0.01942

G1 X141.712 Y128.504 E0.13172

G1 X141.712 Y129.040 E0.01942

G1 X143.746 Y131.075 E0.10425

G1 X143.210 Y131.075 E0.01942

G1 X141.712 Y129.576 E0.07679

G1 X141.712 Y130.112 E0.01942

G1 X142.674 Y131.075 E0.04933

G1 X142.139 Y131.075 E0.01942

```
G1 X141.542 Y130.478 E0.03056
M204 S1250
; stop printing object Petg print.STL id:26 copy 0
; printing object Petg print.STL id:0 copy 0
G1 E-4.00000 F2400.000
G1 X111.213 Y96.713 F9000.000
G1 E4.00000 F900.000
M204 S800
;TYPE:Perimeter
;WIDTH:0.42
G1 F1074.044
G1 X104.595 Y96.713 E0.23862
G1 X104.531 Y96.486 E0.00851
G1 X104.189 Y95.675 E0.03172
G1 X103.750 Y94.913 E0.03172
G1 X103.220 Y94.210 E0.03172
G1 X102.608 Y93.578 E0.03172
G1 X101.923 Y93.026 E0.03172
G1 X101.176 Y92.563 E0.03172
G1 X100.376 Y92.194 E0.03172
G1 X99.538 Y91.927 E0.03172
G1 X98.673 Y91.766 E0.03172
G1 X97.819 Y91.713 E0.03085
G1 X89.787 Y91.713 E0.28963
G1 X89.787 Y88.287 E0.12353
G1 X97.819 Y88.287 E0.28963
G1 X98.673 Y88.234 E0.03085
G1 X99.538 Y88.073 E0.03172
G1 X100.376 Y87.806 E0.03172
```

```
G1 X101.176 Y87.437 E0.03172
G1 X101.923 Y86.974 E0.03172
G1 X102.608 Y86.422 E0.03172
G1 X103.220 Y85.790 E0.03172
G1 X103.750 Y85.087 E0.03172
G1 X104.189 Y84.325 E0.03172
G1 X104.531 Y83.514 E0.03172
G1 X104.595 Y83.287 E0.00851
G1 X111.213 Y83.287 E0.23862
G1 X111.213 Y96.653 E0.48194
M204 S1250
G1 X111.590 Y97.090 F9000.000
M204 S800
;TYPE:External perimeter
G1 F1074.044
G1 X104.310 Y97.090 E0.26250
G1 X104.174 Y96.611 E0.01795
G1 X103.851 Y95.843 E0.03004
G1 X103.435 Y95.121 E0.03004
G1 X102.933 Y94.456 E0.03004
G1 X102.354 Y93.857 E0.03004
G1 X101.705 Y93.334 E0.03004
G1 X100.997 Y92.895 E0.03004
G1 X100.240 Y92.547 E0.03004
G1 X99.446 Y92.294 E0.03004
G1 X98.627 Y92.141 E0.03004
G1 X97.808 Y92.090 E0.02959
G1 X89.410 Y92.090 E0.30280
G1 X89.410 Y87.910 E0.15072
```

```
G1 X97.808 Y87.910 E0.30280
G1 X98.627 Y87.859 E0.02959
G1 X99.446 Y87.706 E0.03004
G1 X100.240 Y87.453 E0.03004
M73 P9 R62
G1 X100.997 Y87.105 E0.03004
G1 X101.705 Y86.666 E0.03004
G1 X102.354 Y86.143 E0.03004
G1 X102.933 Y85.544 E0.03004
G1 X103.435 Y84.879 E0.03004
G1 X103.851 Y84.157 E0.03004
G1 X104.174 Y83.389 E0.03004
G1 X104.310 Y82.910 E0.01795
G1 X111.590 Y82.910 E0.26250
G1 X111.590 Y97.030 E0.50913
M204 S1250
G1 X111.216 Y96.947 F9000.000
G1 E-4.00000 F2400.000
G1 X111.100 Y84.166 F9000.000
G1 E4.00000 F900.000
M204 S800
;TYPE:Solid infill
;WIDTH:0.42186
G1 F1068.771
G1 X110.503 Y83.570 E0.03057
G1 X109.967 Y83.570 E0.01942
G1 X110.930 Y84.533 E0.04934
G1 X110.930 Y85.069 E0.01942
G1 X109.431 Y83.570 E0.07680
```

```
G1 X108.896 Y83.570 E0.01942
G1 X110.930 Y85.604 E0.10426
G1 X110.930 Y86.140 E0.01942
G1 X108.360 Y83.570 E0.13172
G1 X107.824 Y83.570 E0.01942
G1 X110.930 Y86.676 E0.15918
G1 X110.930 Y87.212 E0.01942
G1 X107.288 Y83.570 E0.18665
G1 X106.752 Y83.570 E0.01942
G1 X110.930 Y87.748 E0.21411
G1 X110.930 Y88.284 E0.01942
G1 X106.216 Y83.570 E0.24157
G1 X105.680 Y83.570 E0.01942
G1 X110.930 Y88.820 E0.26903
G1 X110.930 Y89.356 E0.01942
G1 X105.144 Y83.570 E0.29649
G1 X104.809 Y83.570 E0.01215
G1 X104.753 Y83.715 E0.00563
G1 X110.930 Y89.892 E0.31653
G1 X110.930 Y90.428 E0.01942
G1 X104.594 Y84.092 E0.32468
G1 X104.433 Y84.467 E0.01479
G1 X110.930 Y90.963 E0.33292
G1 X110.930 Y91.499 E0.01942
G1 X104.238 Y84.807 E0.34295
G1 X104.042 Y85.147 E0.01422
G1 X110.930 Y92.035 E0.35299
G1 X110.930 Y92.571 E0.01942
G1 X103.821 Y85.462 E0.36429
```

```
G1 X103.591 Y85.768 E0.01387
G1 X110.930 Y93.107 E0.37609
G1 X110.930 Y93.643 E0.01942
G1 X103.350 Y86.063 E0.38845
G1 X103.086 Y86.335 E0.01373
G1 X110.930 Y94.179 E0.40196
G1 X110.930 Y94.715 E0.01942
G1 X102.823 Y86.607 E0.41547
G1 X102.529 Y86.849 E0.01380
G1 X110.930 Y95.251 E0.43053
G1 X110.930 Y95.787 E0.01942
G1 X102.232 Y87.088 E0.44573
G1 X101.918 Y87.310 E0.01393
G1 X110.930 Y96.322 E0.46184
G1 X110.930 Y96.430 E0.00390
G1 X110.502 Y96.430 E0.01552
G1 X101.587 Y87.515 E0.45684
G1 X101.250 Y87.714 E0.01418
G1 X109.966 Y96.430 E0.44663
G1 X109.430 Y96.430 E0.01942
G1 X100.883 Y87.883 E0.43797
G1 X100.516 Y88.052 E0.01464
G1 X108.894 Y96.430 E0.42931
G1 X108.358 Y96.430 E0.01942
G1 X100.114 Y88.186 E0.42247
G1 X99.708 Y88.315 E0.01546
G1 X107.822 Y96.430 E0.41584
G1 X107.286 Y96.430 E0.01942
G1 X99.267 Y88.411 E0.41094
```

```
G1 X98.816 Y88.495 E0.01664
G1 X106.751 Y96.430 E0.40661
G1 X106.215 Y96.430 E0.01942
G1 X98.324 Y88.539 E0.40436
G1 X97.819 Y88.570 E0.01834
G1 X105.679 Y96.430 E0.40279
G1 X105.143 Y96.430 E0.01942
G1 X104.226 Y95.514 E0.04696
M204 S1250
G1 X101.130 Y92.417 F9000.000
M204 S800
G1 F1068.771
G1 X97.283 Y88.570 E0.19713
G1 X96.747 Y88.570 E0.01942
G1 X99.933 Y91.757 E0.16330
G1 X99.607 Y91.653 E0.01240
G1 X99.221 Y91.581 E0.01422
G1 X96.211 Y88.570 E0.15428
G1 X95.675 Y88.570 E0.01942
G1 X98.582 Y91.477 E0.14896
G1 X98.010 Y91.441 E0.02074
G1 X95.139 Y88.570 E0.14715
G1 X94.603 Y88.570 E0.01942
G1 X97.463 Y91.430 E0.14657
G1 X96.927 Y91.430 E0.01942
G1 X94.067 Y88.570 E0.14657
G1 X93.531 Y88.570 E0.01942
G1 X96.392 Y91.430 E0.14657
G1 X95.856 Y91.430 E0.01942
```

```
G1 X92.995 Y88.570 E0.14657
G1 X92.459 Y88.570 E0.01942
G1 X95.320 Y91.430 E0.14657
G1 X94.784 Y91.430 E0.01942
G1 X91.924 Y88.570 E0.14657
G1 X91.388 Y88.570 E0.01942
G1 X94.248 Y91.430 E0.14657
G1 X93.712 Y91.430 E0.01942
G1 X90.852 Y88.570 E0.14657
G1 X90.316 Y88.570 E0.01942
G1 X93.176 Y91.430 E0.14657
G1 X92.640 Y91.430 E0.01942
G1 X90.070 Y88.860 E0.13172
G1 X90.070 Y89.396 E0.01942
G1 X92.104 Y91.430 E0.10425
G1 X91.568 Y91.430 E0.01942
G1 X90.070 Y89.932 E0.07679
G1 X90.070 Y90.467 E0.01942
G1 X91.033 Y91.430 E0.04933
G1 X90.497 Y91.430 E0.01942
G1 X89.900 Y90.834 E0.03056
M204 S1250
; stop printing object Petg print.STL id:0 copy 0
; printing object tpu print.STL id:1 copy 0
; stop printing object tpu print.STL id:1 copy 0
; printing object tpu print.STL id:5 copy 0
; stop printing object tpu print.STL id:5 copy 0
; printing object tpu print.STL id:9 copy 0
; stop printing object tpu print.STL id:9 copy 0
```

; printing object Petg print.STL id:8 copy 0

G1 E-4.00000 F2400.000

G1 X104.639 Y54.572 F9000.000

G1 E4.00000 F900.000

M204 S800

;TYPE:Perimeter

;WIDTH:0.42

G1 F1074.044

G1 X104.575 Y54.345 E0.00851

G1 X104.233 Y53.534 E0.03172

G1 X103.793 Y52.772 E0.03172

G1 X103.264 Y52.069 E0.03172

G1 X102.652 Y51.437 E0.03172

G1 X101.967 Y50.885 E0.03172

G1 X101.219 Y50.421 E0.03172

G1 X100.420 Y50.053 E0.03172

G1 X99.582 Y49.786 E0.03172

G1 X98.717 Y49.624 E0.03172

G1 X97.863 Y49.572 E0.03085

G1 X89.831 Y49.572 E0.28963

G1 X89.831 Y46.146 E0.12353

G1 X97.863 Y46.146 E0.28963

G1 X98.717 Y46.093 E0.03085

G1 X99.582 Y45.931 E0.03172

G1 X100.420 Y45.664 E0.03172

G1 X101.219 Y45.296 E0.03172

G1 X101.967 Y44.833 E0.03172

G1 X102.652 Y44.281 E0.03172

G1 X103.264 Y43.648 E0.03172

G1 X103.793 Y42.946 E0.03172

G1 X104.233 Y42.183 E0.03172

G1 X104.575 Y41.373 E0.03172

G1 X104.639 Y41.146 E0.00851

G1 X111.257 Y41.146 E0.23862

G1 X111.257 Y54.572 E0.48410

G1 X104.699 Y54.572 E0.23646

M204 S1250

G1 X104.354 Y54.949 F9000.000

M204 S800

;TYPE:External perimeter

G1 F1074.044

G1 X104.218 Y54.470 E0.01795

G1 X103.894 Y53.702 E0.03004

G1 X103.478 Y52.980 E0.03004

G1 X102.977 Y52.314 E0.03004

G1 X102.397 Y51.716 E0.03004

G1 X101.748 Y51.193 E0.03004

G1 X101.040 Y50.754 E0.03004

G1 X100.283 Y50.405 E0.03004

G1 X99.489 Y50.153 E0.03004

G1 X98.670 Y49.999 E0.03004

G1 X97.851 Y49.949 E0.02959

G1 X89.454 Y49.949 E0.30280

G1 X89.454 Y45.769 E0.15072

G1 X97.851 Y45.769 E0.30280

G1 X98.670 Y45.718 E0.02959

G1 X99.489 Y45.565 E0.03004

G1 X100.283 Y45.312 E0.03004

```
G1 X101.040 Y44.963 E0.03004
G1 X101.748 Y44.524 E0.03004
G1 X102.397 Y44.002 E0.03004
G1 X102.977 Y43.403 E0.03004
G1 X103.478 Y42.738 E0.03004
G1 X103.894 Y42.015 E0.03004
G1 X104.218 Y41.248 E0.03004
G1 X104.354 Y40.769 E0.01795
G1 X111.634 Y40.769 E0.26250
G1 X111.634 Y54.949 E0.51129
G1 X104.414 Y54.949 E0.26033
M204 S1250
G1 X104.343 Y54.549 F9000.000
G1 E-4.00000 F2400.000
G1 X111.143 Y42.025 F9000.000
G1 E4.00000 F900.000
M204 S800
;TYPE:Solid infill
;WIDTH:0.42186
G1 F1068.771
G1 X110.547 Y41.429 E0.03057
G1 X110.011 Y41.429 E0.01942
G1 X110.974 Y42.391 E0.04934
G1 X110.974 Y42.927 E0.01942
G1 X109.475 Y41.429 E0.07680
G1 X108.939 Y41.429 E0.01942
G1 X110.974 Y43.463 E0.10426
G1 X110.974 Y43.999 E0.01942
G1 X108.403 Y41.429 E0.13172
```

G1 X107.867 Y41.429 E0.01942

G1 X110.974 Y44.535 E0.15918

G1 X110.974 Y45.071 E0.01942

G1 X107.332 Y41.429 E0.18665

G1 X106.796 Y41.429 E0.01942

G1 X110.974 Y45.607 E0.21411

G1 X110.974 Y46.143 E0.01942

G1 X106.260 Y41.429 E0.24157

M73 P9 R61

G1 X105.724 Y41.429 E0.01942

G1 X110.974 Y46.679 E0.26903

G1 X110.974 Y47.214 E0.01942

G1 X105.188 Y41.429 E0.29649

G1 X104.853 Y41.429 E0.01215

G1 X104.797 Y41.573 E0.00563

G1 X110.974 Y47.750 E0.31653

G1 X110.974 Y48.286 E0.01942

G1 X104.638 Y41.950 E0.32468

G1 X104.477 Y42.326 E0.01479

G1 X110.974 Y48.822 E0.33292

G1 X110.974 Y49.358 E0.01942

G1 X104.281 Y42.666 E0.34295

G1 X104.085 Y43.006 E0.01422

G1 X110.974 Y49.894 E0.35299

G1 X110.974 Y50.430 E0.01942

G1 X103.865 Y43.321 E0.36429

G1 X103.635 Y43.627 E0.01387

G1 X110.974 Y50.966 E0.37609

M73 P10 R61

```
G1 X110.974 Y51.502 E0.01942
G1 X103.393 Y43.921 E0.38845
G1 X103.130 Y44.194 E0.01373
G1 X110.974 Y52.038 E0.40196
G1 X110.974 Y52.573 E0.01942
G1 X102.866 Y44.466 E0.41547
G1 X102.572 Y44.708 E0.01380
G1 X110.974 Y53.109 E0.43053
G1 X110.974 Y53.645 E0.01942
G1 X102.276 Y44.947 E0.44573
G1 X101.961 Y45.169 E0.01393
G1 X110.974 Y54.181 E0.46184
G1 X110.974 Y54.289 E0.00390
G1 X110.546 Y54.289 E0.01552
G1 X101.631 Y45.374 E0.45684
G1 X101.294 Y45.573 E0.01418
G1 X110.010 Y54.289 E0.44663
G1 X109.474 Y54.289 E0.01942
G1 X100.927 Y45.742 E0.43797
G1 X100.560 Y45.911 E0.01464
G1 X108.938 Y54.289 E0.42931
G1 X108.402 Y54.289 E0.01942
G1 X100.158 Y46.045 E0.42247
G1 X99.751 Y46.174 E0.01546
G1 X107.866 Y54.289 E0.41584
G1 X107.330 Y54.289 E0.01942
G1 X99.311 Y46.270 E0.41094
G1 X98.859 Y46.354 E0.01664
G1 X106.794 Y54.289 E0.40661
```

```
G1 X106.258 Y54.289 E0.01942
G1 X98.367 Y46.398 E0.40436
G1 X97.862 Y46.429 E0.01834
G1 X105.722 Y54.289 E0.40279
G1 X105.187 Y54.289 E0.01942
G1 X104.270 Y53.372 E0.04696
M204 S1250
G1 X101.173 Y50.275 F9000.000
M204 S800
G1 F1068.771
G1 X97.326 Y46.429 E0.19713
G1 X96.790 Y46.429 E0.01942
G1 X99.977 Y49.615 E0.16330
G1 X99.651 Y49.511 E0.01240
G1 X99.265 Y49.439 E0.01422
G1 X96.254 Y46.429 E0.15428
G1 X95.719 Y46.429 E0.01942
G1 X98.625 Y49.335 E0.14896
G1 X98.054 Y49.300 E0.02074
G1 X95.183 Y46.429 E0.14715
G1 X94.647 Y46.429 E0.01942
G1 X97.507 Y49.289 E0.14657
G1 X96.971 Y49.289 E0.01942
G1 X94.111 Y46.429 E0.14657
G1 X93.575 Y46.429 E0.01942
G1 X96.435 Y49.289 E0.14657
G1 X95.899 Y49.289 E0.01942
G1 X93.039 Y46.429 E0.14657
G1 X92.503 Y46.429 E0.01942
```

```
G1 X95.363 Y49.289 E0.14657
G1 X94.827 Y49.289 E0.01942
G1 X91.967 Y46.429 E0.14657
G1 X91.431 Y46.429 E0.01942
G1 X94.292 Y49.289 E0.14657
G1 X93.756 Y49.289 E0.01942
G1 X90.895 Y46.429 E0.14657
G1 X90.360 Y46.429 E0.01942
G1 X93.220 Y49.289 E0.14657
G1 X92.684 Y49.289 E0.01942
G1 X90.114 Y46.718 E0.13172
G1 X90.114 Y47.254 E0.01942
G1 X92.148 Y49.289 E0.10425
G1 X91.612 Y49.289 E0.01942
G1 X90.114 Y47.790 E0.07679
G1 X90.114 Y48.326 E0.01942
G1 X91.076 Y49.289 E0.04933
G1 X90.540 Y49.289 E0.01942
G1 X89.944 Y48.692 E0.03056
M204 S1250
; stop printing object Petg print.STL id:8 copy 0
; printing object Petg print.STL id:4 copy 0
G1 E-4.00000 F2400.000
G1 X89.944 Y66.095 F9000.000
G1 E4.00000 F900.000
M204 S800
;TYPE:Perimeter
;WIDTH:0.42
G1 F1074.044
```

```
G1 X97.871 Y66.095 E0.28582
G1 X98.724 Y66.042 E0.03085
G1 X99.589 Y65.880 E0.03172
G1 X100.428 Y65.613 E0.03172
G1 X101.227 Y65.245 E0.03172
G1 X101.974 Y64.782 E0.03172
G1 X102.659 Y64.230 E0.03172
G1 X103.271 Y63.597 E0.03172
G1 X103.801 Y62.895 E0.03172
G1 X104.240 Y62.132 E0.03172
G1 X104.582 Y61.322 E0.03172
G1 X104.646 Y61.095 E0.00851
G1 X111.264 Y61.095 E0.23862
G1 X111.264 Y74.521 E0.48410
G1 X104.646 Y74.521 E0.23862
G1 X104.582 Y74.293 E0.00851
G1 X104.240 Y73.483 E0.03172
G1 X103.801 Y72.720 E0.03172
G1 X103.271 Y72.018 E0.03172
G1 X102.659 Y71.386 E0.03172
G1 X101.974 Y70.834 E0.03172
G1 X101.227 Y70.370 E0.03172
G1 X100.428 Y70.002 E0.03172
G1 X99.589 Y69.735 E0.03172
G1 X98.724 Y69.573 E0.03172
G1 X97.871 Y69.521 E0.03085
G1 X89.838 Y69.521 E0.28963
G1 X89.838 Y66.095 E0.12353
G1 X89.884 Y66.095 E0.00164
```

```
M204 S1250
G1 X89.461 Y65.718 F9000.000
M204 S800
;TYPE:External perimeter
G1 F1074.044
G1 X97.859 Y65.718 E0.30280
G1 X98.678 Y65.667 E0.02959
G1 X99.497 Y65.514 E0.03004
G1 X100.291 Y65.261 E0.03004
G1 X101.048 Y64.912 E0.03004
G1 X101.756 Y64.473 E0.03004
G1 X102.405 Y63.951 E0.03004
G1 X102.984 Y63.352 E0.03004
G1 X103.486 Y62.686 E0.03004
G1 X103.902 Y61.964 E0.03004
G1 X104.226 Y61.197 E0.03004
G1 X104.361 Y60.718 E0.01795
G1 X111.641 Y60.718 E0.26250
G1 X111.641 Y74.898 E0.51129
G1 X104.361 Y74.898 E0.26250
G1 X104.226 Y74.419 E0.01795
G1 X103.902 Y73.651 E0.03004
G1 X103.486 Y72.929 E0.03004
G1 X102.984 Y72.263 E0.03004
G1 X102.405 Y71.665 E0.03004
G1 X101.756 Y71.142 E0.03004
G1 X101.048 Y70.703 E0.03004
G1 X100.291 Y70.354 E0.03004
G1 X99.497 Y70.101 E0.03004
```

```
G1 X98.678 Y69.948 E0.03004
G1 X97.859 Y69.898 E0.02959
G1 X89.461 Y69.898 E0.30280
G1 X89.461 Y65.778 E0.14856
M204 S1250
G1 X89.856 Y65.779 F9000.000
G1 E-4.00000 F2400.000
G1 X111.151 Y61.974 F9000.000
G1 E4.00000 F900.000
M204 S800
;TYPE:Solid infill
;WIDTH:0.42186
G1 F1068.771
G1 X110.554 Y61.378 E0.03057
G1 X110.019 Y61.378 E0.01942
G1 X110.981 Y62.340 E0.04934
G1 X110.981 Y62.876 E0.01942
G1 X109.483 Y61.378 E0.07680
G1 X108.947 Y61.378 E0.01942
G1 X110.981 Y63.412 E0.10426
G1 X110.981 Y63.948 E0.01942
G1 X108.411 Y61.378 E0.13172
G1 X107.875 Y61.378 E0.01942
G1 X110.981 Y64.484 E0.15918
G1 X110.981 Y65.020 E0.01942
G1 X107.339 Y61.378 E0.18665
G1 X106.803 Y61.378 E0.01942
G1 X110.981 Y65.556 E0.21411
G1 X110.981 Y66.092 E0.01942
```

```
G1 X106.267 Y61.378 E0.24157
G1 X105.731 Y61.378 E0.01942
G1 X110.981 Y66.627 E0.26903
G1 X110.981 Y67.163 E0.01942
G1 X105.195 Y61.378 E0.29649
G1 X104.860 Y61.378 E0.01215
G1 X104.804 Y61.522 E0.00563
G1 X110.981 Y67.699 E0.31653
G1 X110.981 Y68.235 E0.01942
G1 X104.645 Y61.899 E0.32468
G1 X104.485 Y62.274 E0.01479
G1 X110.981 Y68.771 E0.33292
G1 X110.981 Y69.307 E0.01942
G1 X104.289 Y62.615 E0.34295
G1 X104.093 Y62.955 E0.01422
G1 X110.981 Y69.843 E0.35299
G1 X110.981 Y70.379 E0.01942
G1 X103.872 Y63.270 E0.36429
G1 X103.642 Y63.576 E0.01387
G1 X110.981 Y70.915 E0.37609
G1 X110.981 Y71.451 E0.01942
G1 X103.401 Y63.870 E0.38845
G1 X103.137 Y64.143 E0.01373
G1 X110.981 Y71.987 E0.40196
G1 X110.981 Y72.522 E0.01942
G1 X102.874 Y64.415 E0.41547
G1 X102.580 Y64.657 E0.01380
G1 X110.981 Y73.058 E0.43053
G1 X110.981 Y73.594 E0.01942
```

```
G1 X102.283 Y64.896 E0.44573
G1 X101.969 Y65.118 E0.01393
G1 X110.981 Y74.130 E0.46184
G1 X110.981 Y74.238 E0.00390
G1 X110.553 Y74.238 E0.01552
G1 X101.638 Y65.323 E0.45684
G1 X101.301 Y65.522 E0.01418
G1 X110.017 Y74.238 E0.44663
G1 X109.481 Y74.238 E0.01942
G1 X100.935 Y65.691 E0.43797
G1 X100.568 Y65.860 E0.01464
G1 X108.945 Y74.238 E0.42931
G1 X108.409 Y74.238 E0.01942
G1 X100.165 Y65.994 E0.42247
G1 X99.759 Y66.123 E0.01546
G1 X107.874 Y74.238 E0.41584
G1 X107.338 Y74.238 E0.01942
G1 X99.318 Y66.219 E0.41094
M73 P11 R61
G1 X98.867 Y66.303 E0.01664
G1 X106.802 Y74.238 E0.40661
G1 X106.266 Y74.238 E0.01942
G1 X98.375 Y66.347 E0.40436
G1 X97.870 Y66.378 E0.01834
G1 X105.730 Y74.238 E0.40279
G1 X105.194 Y74.238 E0.01942
G1 X104.278 Y73.321 E0.04696
M204 S1250
G1 X101.181 Y70.224 F9000.000
```

```
M204 S800
G1 F1068.771
G1 X97.334 Y66.378 E0.19713
G1 X96.798 Y66.378 E0.01942
G1 X99.985 Y69.564 E0.16330
G1 X99.658 Y69.460 E0.01240
G1 X99.273 Y69.388 E0.01422
G1 X96.262 Y66.378 E0.15428
G1 X95.726 Y66.378 E0.01942
G1 X98.633 Y69.284 E0.14896
G1 X98.062 Y69.249 E0.02074
G1 X95.190 Y66.378 E0.14715
G1 X94.654 Y66.378 E0.01942
G1 X97.514 Y69.238 E0.14657
G1 X96.979 Y69.238 E0.01942
G1 X94.118 Y66.378 E0.14657
G1 X93.582 Y66.378 E0.01942
G1 X96.443 Y69.238 E0.14657
G1 X95.907 Y69.238 E0.01942
G1 X93.047 Y66.378 E0.14657
G1 X92.511 Y66.378 E0.01942
G1 X95.371 Y69.238 E0.14657
G1 X94.835 Y69.238 E0.01942
G1 X91.975 Y66.378 E0.14657
G1 X91.439 Y66.378 E0.01942
G1 X94.299 Y69.238 E0.14657
G1 X93.763 Y69.238 E0.01942
G1 X90.903 Y66.378 E0.14657
G1 X90.367 Y66.378 E0.01942
```

```
G1 X93.227 Y69.238 E0.14657
G1 X92.691 Y69.238 E0.01942
G1 X90.121 Y66.667 E0.13172
G1 X90.121 Y67.203 E0.01942
G1 X92.155 Y69.238 E0.10425
G1 X91.620 Y69.238 E0.01942
G1 X90.121 Y67.739 E0.07679
G1 X90.121 Y68.275 E0.01942
G1 X91.084 Y69.238 E0.04933
G1 X90.548 Y69.238 E0.01942
G1 X89.951 Y68.641 E0.03056
M204 S1250
; stop printing object Petg print.STL id:4 copy 0
; printing object tpu print.STL id:29 copy 0
; stop printing object tpu print.STL id:29 copy 0
; printing object tpu print.STL id:25 copy 0
; stop printing object tpu print.STL id:25 copy 0
; printing object Petg print.STL id:24 copy 0
G1 E-4.00000 F2400.000
G1 X141.488 Y69.696 F9000.000
G1 E4.00000 F900.000
M204 S800
;TYPE:Perimeter
;WIDTH:0.42
G1 F1074.044
G1 X141.488 Y66.270 E0.12353
G1 X149.521 Y66.270 E0.28963
G1 X150.375 Y66.217 E0.03085
G1 X151.239 Y66.055 E0.03172
```

```
G1 X152.078 Y65.788 E0.03172
G1 X152.877 Y65.420 E0.03172
G1 X153.625 Y64.957 E0.03172
G1 X154.310 Y64.405 E0.03172
G1 X154.922 Y63.772 E0.03172
G1 X155.451 Y63.070 E0.03172
G1 X155.890 Y62.308 E0.03172
G1 X156.232 Y61.497 E0.03172
G1 X156.297 Y61.270 E0.00851
G1 X162.914 Y61.270 E0.23862
G1 X162.914 Y74.696 E0.48410
G1 X156.297 Y74.696 E0.23862
G1 X156.232 Y74.469 E0.00851
G1 X155.890 Y73.658 E0.03172
G1 X155.451 Y72.896 E0.03172
G1 X154.922 Y72.193 E0.03172
G1 X154.310 Y71.561 E0.03172
G1 X153.625 Y71.009 E0.03172
G1 X152.877 Y70.545 E0.03172
G1 X152.078 Y70.177 E0.03172
G1 X151.239 Y69.910 E0.03172
G1 X150.375 Y69.748 E0.03172
G1 X149.521 Y69.696 E0.03085
G1 X141.548 Y69.696 E0.28746
M204 S1250
G1 X141.111 Y70.073 F9000.000
M204 S800
;TYPE:External perimeter
G1 F1074.044
```

```
G1 X141.111 Y65.893 E0.15072
G1 X149.509 Y65.893 E0.30280
G1 X150.328 Y65.842 E0.02959
G1 X151.147 Y65.689 E0.03004
G1 X151.941 Y65.436 E0.03004
G1 X152.698 Y65.087 E0.03004
G1 X153.406 Y64.648 E0.03004
G1 X154.055 Y64.126 E0.03004
G1 X154.635 Y63.527 E0.03004
G1 X155.136 Y62.862 E0.03004
G1 X155.552 Y62.139 E0.03004
G1 X155.876 Y61.372 E0.03004
G1 X156.011 Y60.893 E0.01795
G1 X163.291 Y60.893 E0.26250
G1 X163.291 Y75.073 E0.51129
G1 X156.011 Y75.073 E0.26250
M73 P11 R60
G1 X155.876 Y74.594 E0.01795
G1 X155.552 Y73.826 E0.03004
G1 X155.136 Y73.104 E0.03004
G1 X154.635 Y72.439 E0.03004
G1 X154.055 Y71.840 E0.03004
G1 X153.406 Y71.317 E0.03004
G1 X152.698 Y70.878 E0.03004
G1 X151.941 Y70.529 E0.03004
G1 X151.147 Y70.277 E0.03004
G1 X150.328 Y70.123 E0.03004
G1 X149.509 Y70.073 E0.02959
G1 X141.171 Y70.073 E0.30064
```

```
M204 S1250
G1 X141.312 Y69.727 F9000.000
G1 E-4.00000 F2400.000
G1 X162.801 Y62.149 F9000.000
G1 E4.00000 F900.000
M204 S800
;TYPE:Solid infill
;WIDTH:0.42186
G1 F1068.771
G1 X162.205 Y61.553 E0.03057
G1 X161.669 Y61.553 E0.01942
G1 X162.632 Y62.515 E0.04934
G1 X162.632 Y63.051 E0.01942
G1 X161.133 Y61.553 E0.07680
G1 X160.597 Y61.553 E0.01942
G1 X162.632 Y63.587 E0.10426
G1 X162.632 Y64.123 E0.01942
G1 X160.061 Y61.553 E0.13172
G1 X159.525 Y61.553 E0.01942
G1 X162.632 Y64.659 E0.15918
G1 X162.632 Y65.195 E0.01942
G1 X158.989 Y61.553 E0.18665
G1 X158.453 Y61.553 E0.01942
G1 X162.632 Y65.731 E0.21411
G1 X162.632 Y66.267 E0.01942
G1 X157.917 Y61.553 E0.24157
G1 X157.382 Y61.553 E0.01942
G1 X162.632 Y66.803 E0.26903
G1 X162.632 Y67.338 E0.01942
```

```
G1 X156.846 Y61.553 E0.29649
G1 X156.510 Y61.553 E0.01215
G1 X156.455 Y61.697 E0.00563
G1 X162.632 Y67.874 E0.31653
G1 X162.632 Y68.410 E0.01942
G1 X156.296 Y62.074 E0.32468
G1 X156.135 Y62.450 E0.01479
G1 X162.632 Y68.946 E0.33292
G1 X162.632 Y69.482 E0.01942
G1 X155.939 Y62.790 E0.34295
G1 X155.743 Y63.130 E0.01422
G1 X162.632 Y70.018 E0.35299
G1 X162.632 Y70.554 E0.01942
G1 X155.523 Y63.445 E0.36429
G1 X155.292 Y63.751 E0.01387
G1 X162.632 Y71.090 E0.37609
G1 X162.632 Y71.626 E0.01942
G1 X155.051 Y64.045 E0.38845
G1 X154.788 Y64.318 E0.01373
G1 X162.632 Y72.162 E0.40196
G1 X162.632 Y72.697 E0.01942
G1 X154.524 Y64.590 E0.41547
G1 X154.230 Y64.832 E0.01380
G1 X162.632 Y73.233 E0.43053
G1 X162.632 Y73.769 E0.01942
G1 X153.933 Y65.071 E0.44573
G1 X153.619 Y65.293 E0.01393
G1 X162.632 Y74.305 E0.46184
G1 X162.632 Y74.413 E0.00390
```

```
G1 X162.203 Y74.413 E0.01552
G1 X153.288 Y65.498 E0.45684
G1 X152.952 Y65.697 E0.01418
G1 X161.667 Y74.413 E0.44663
G1 X161.131 Y74.413 E0.01942
G1 X152.585 Y65.866 E0.43797
G1 X152.218 Y66.035 E0.01464
G1 X160.596 Y74.413 E0.42931
G1 X160.060 Y74.413 E0.01942
G1 X151.815 Y66.169 E0.42247
G1 X151.409 Y66.298 E0.01546
G1 X159.524 Y74.413 E0.41584
G1 X158.988 Y74.413 E0.01942
G1 X150.969 Y66.394 E0.41094
G1 X150.517 Y66.478 E0.01664
G1 X158.452 Y74.413 E0.40661
G1 X157.916 Y74.413 E0.01942
G1 X150.025 Y66.522 E0.40436
G1 X149.520 Y66.553 E0.01834
G1 X157.380 Y74.413 E0.40279
G1 X156.844 Y74.413 E0.01942
G1 X155.928 Y73.496 E0.04696
M204 S1250
G1 X152.831 Y70.400 F9000.000
M204 S800
G1 F1068.771
G1 X148.984 Y66.553 E0.19713
G1 X148.448 Y66.553 E0.01942
G1 X151.635 Y69.739 E0.16330
```

G1 X151.309 Y69.635 E0.01240

G1 X150.923 Y69.563 E0.01422

G1 X147.912 Y66.553 E0.15428

G1 X147.376 Y66.553 E0.01942

G1 X150.283 Y69.459 E0.14896

G1 X149.712 Y69.424 E0.02074

G1 X146.840 Y66.553 E0.14715

G1 X146.304 Y66.553 E0.01942

G1 X149.165 Y69.413 E0.14657

G1 X148.629 Y69.413 E0.01942

G1 X145.769 Y66.553 E0.14657

G1 X145.233 Y66.553 E0.01942

G1 X148.093 Y69.413 E0.14657

G1 X147.557 Y69.413 E0.01942

G1 X144.697 Y66.553 E0.14657

G1 X144.161 Y66.553 E0.01942

G1 X147.021 Y69.413 E0.14657

G1 X146.485 Y69.413 E0.01942

G1 X143.625 Y66.553 E0.14657

G1 X143.089 Y66.553 E0.01942

G1 X145.949 Y69.413 E0.14657

G1 X145.413 Y69.413 E0.01942

G1 X142.553 Y66.553 E0.14657

G1 X142.017 Y66.553 E0.01942

G1 X144.878 Y69.413 E0.14657

G1 X144.342 Y69.413 E0.01942

G1 X141.771 Y66.843 E0.13172

G1 X141.771 Y67.378 E0.01942

G1 X143.806 Y69.413 E0.10425

G1 X143.270 Y69.413 E0.01942

G1 X141.771 Y67.914 E0.07679

G1 X141.771 Y68.450 E0.01942

G1 X142.734 Y69.413 E0.04933

G1 X142.198 Y69.413 E0.01942

G1 X141.602 Y68.816 E0.03056

M204 S1250

; stop printing object Petg print.STL id:24 copy 0

; printing object Petg print.STL id:28 copy 0

G1 E-4.00000 F2400.000

G1 X141.602 Y49.747 F9000.000

G1 E4.00000 F900.000

M204 S800

;TYPE:Perimeter

;WIDTH:0.42

G1 F1074.044

G1 X141.481 Y49.747 E0.00435

G1 X141.481 Y46.321 E0.12353

G1 X149.513 Y46.321 E0.28963

G1 X150.367 Y46.268 E0.03085

G1 X151.232 Y46.106 E0.03172

G1 X152.070 Y45.839 E0.03172

G1 X152.869 Y45.471 E0.03172

G1 X153.617 Y45.008 E0.03172

G1 X154.302 Y44.456 E0.03172

G1 X154.914 Y43.824 E0.03172

G1 X155.444 Y43.121 E0.03172

G1 X155.883 Y42.359 E0.03172

G1 X156.225 Y41.548 E0.03172

```
G1 X156.289 Y41.321 E0.00851
G1 X162.907 Y41.321 E0.23862
M73 P12 R60
G1 X162.907 Y54.747 E0.48410
G1 X156.289 Y54.747 E0.23862
G1 X156.225 Y54.520 E0.00851
G1 X155.883 Y53.709 E0.03172
G1 X155.444 Y52.947 E0.03172
G1 X154.914 Y52.244 E0.03172
G1 X154.302 Y51.612 E0.03172
G1 X153.617 Y51.060 E0.03172
G1 X152.869 Y50.596 E0.03172
G1 X152.070 Y50.228 E0.03172
G1 X151.232 Y49.961 E0.03172
G1 X150.367 Y49.799 E0.03172
G1 X149.513 Y49.747 E0.03085
G1 X141.662 Y49.747 E0.28311
M204 S1250
G1 X141.104 Y50.124 F9000.000
M204 S800
;TYPE:External perimeter
G1 F1074.044
G1 X141.104 Y45.944 E0.15072
G1 X149.502 Y45.944 E0.30280
G1 X150.321 Y45.893 E0.02959
G1 X151.140 Y45.740 E0.03004
G1 X151.934 Y45.487 E0.03004
G1 X152.691 Y45.139 E0.03004
G1 X153.399 Y44.699 E0.03004
```

```
G1 X154.047 Y44.177 E0.03004
G1 X154.627 Y43.578 E0.03004
G1 X155.129 Y42.913 E0.03004
G1 X155.544 Y42.191 E0.03004
G1 X155.868 Y41.423 E0.03004
G1 X156.004 Y40.944 E0.01795
G1 X163.284 Y40.944 E0.26250
G1 X163.284 Y55.124 E0.51129
G1 X156.004 Y55.124 E0.26250
G1 X155.868 Y54.645 E0.01795
G1 X155.544 Y53.877 E0.03004
G1 X155.129 Y53.155 E0.03004
G1 X154.627 Y52.490 E0.03004
G1 X154.047 Y51.891 E0.03004
G1 X153.399 Y51.368 E0.03004
G1 X152.691 Y50.929 E0.03004
G1 X151.934 Y50.580 E0.03004
G1 X151.140 Y50.328 E0.03004
G1 X150.321 Y50.174 E0.03004
G1 X149.502 Y50.124 E0.02959
G1 X141.164 Y50.124 E0.30064
M204 S1250
G1 X141.305 Y49.778 F9000.000
G1 E-4.00000 F2400.000
G1 X162.794 Y42.200 F9000.000
G1 E4.00000 F900.000
M204 S800
;TYPE:Solid infill
;WIDTH:0.42186
```

```
G1 F1068.771
G1 X162.197 Y41.604 E0.03057
G1 X161.661 Y41.604 E0.01942
G1 X162.624 Y42.566 E0.04934
G1 X162.624 Y43.102 E0.01942
G1 X161.125 Y41.604 E0.07680
G1 X160.589 Y41.604 E0.01942
G1 X162.624 Y43.638 E0.10426
G1 X162.624 Y44.174 E0.01942
G1 X160.054 Y41.604 E0.13172
G1 X159.518 Y41.604 E0.01942
G1 X162.624 Y44.710 E0.15918
G1 X162.624 Y45.246 E0.01942
G1 X158.982 Y41.604 E0.18665
G1 X158.446 Y41.604 E0.01942
G1 X162.624 Y45.782 E0.21411
G1 X162.624 Y46.318 E0.01942
G1 X157.910 Y41.604 E0.24157
G1 X157.374 Y41.604 E0.01942
G1 X162.624 Y46.854 E0.26903
G1 X162.624 Y47.390 E0.01942
G1 X156.838 Y41.604 E0.29649
G1 X156.503 Y41.604 E0.01215
G1 X156.447 Y41.749 E0.00563
G1 X162.624 Y47.925 E0.31653
G1 X162.624 Y48.461 E0.01942
G1 X156.288 Y42.125 E0.32468
G1 X156.127 Y42.501 E0.01479
G1 X162.624 Y48.997 E0.33292
```

```
G1 X162.624 Y49.533 E0.01942
G1 X155.932 Y42.841 E0.34295
G1 X155.736 Y43.181 E0.01422
G1 X162.624 Y50.069 E0.35299
G1 X162.624 Y50.605 E0.01942
G1 X155.515 Y43.496 E0.36429
G1 X155.285 Y43.802 E0.01387
G1 X162.624 Y51.141 E0.37609
G1 X162.624 Y51.677 E0.01942
G1 X155.044 Y44.096 E0.38845
G1 X154.780 Y44.369 E0.01373
G1 X162.624 Y52.213 E0.40196
G1 X162.624 Y52.749 E0.01942
G1 X154.516 Y44.641 E0.41547
G1 X154.223 Y44.883 E0.01380
G1 X162.624 Y53.284 E0.43053
G1 X162.624 Y53.820 E0.01942
G1 X153.926 Y45.122 E0.44573
G1 X153.612 Y45.344 E0.01393
G1 X162.624 Y54.356 E0.46184
G1 X162.624 Y54.464 E0.00390
G1 X162.196 Y54.464 E0.01552
G1 X153.281 Y45.549 E0.45684
G1 X152.944 Y45.748 E0.01418
G1 X161.660 Y54.464 E0.44663
G1 X161.124 Y54.464 E0.01942
G1 X152.577 Y45.917 E0.43797
G1 X152.210 Y46.086 E0.01464
G1 X160.588 Y54.464 E0.42931
```

```
G1 X160.052 Y54.464 E0.01942
G1 X151.808 Y46.220 E0.42247
G1 X151.401 Y46.349 E0.01546
G1 X159.516 Y54.464 E0.41584
G1 X158.980 Y54.464 E0.01942
G1 X150.961 Y46.445 E0.41094
G1 X150.510 Y46.529 E0.01664
G1 X158.444 Y54.464 E0.40661
G1 X157.909 Y54.464 E0.01942
G1 X150.018 Y46.573 E0.40436
G1 X149.512 Y46.604 E0.01834
G1 X157.373 Y54.464 E0.40279
G1 X156.837 Y54.464 E0.01942
G1 X155.920 Y53.547 E0.04696
M204 S1250
G1 X152.823 Y50.451 F9000.000
M204 S800
G1 F1068.771
G1 X148.977 Y46.604 E0.19713
G1 X148.441 Y46.604 E0.01942
G1 X151.627 Y49.790 E0.16330
G1 X151.301 Y49.686 E0.01240
G1 X150.915 Y49.614 E0.01422
G1 X147.905 Y46.604 E0.15428
G1 X147.369 Y46.604 E0.01942
G1 X150.276 Y49.510 E0.14896
G1 X149.704 Y49.475 E0.02074
G1 X146.833 Y46.604 E0.14715
G1 X146.297 Y46.604 E0.01942
```

```
G1 X149.157 Y49.464 E0.14657
G1 X148.621 Y49.464 E0.01942
G1 X145.761 Y46.604 E0.14657
G1 X145.225 Y46.604 E0.01942
G1 X148.085 Y49.464 E0.14657
G1 X147.550 Y49.464 E0.01942
G1 X144.689 Y46.604 E0.14657
G1 X144.153 Y46.604 E0.01942
G1 X147.014 Y49.464 E0.14657
G1 X146.478 Y49.464 E0.01942
G1 X143.617 Y46.604 E0.14657
G1 X143.082 Y46.604 E0.01942
G1 X145.942 Y49.464 E0.14657
G1 X145.406 Y49.464 E0.01942
G1 X142.546 Y46.604 E0.14657
G1 X142.010 Y46.604 E0.01942
G1 X144.870 Y49.464 E0.14657
G1 X144.334 Y49.464 E0.01942
G1 X141.764 Y46.894 E0.13172
G1 X141.764 Y47.429 E0.01942
G1 X143.798 Y49.464 E0.10425
G1 X143.262 Y49.464 E0.01942
G1 X141.764 Y47.965 E0.07679
G1 X141.764 Y48.501 E0.01942
G1 X142.726 Y49.464 E0.04933
G1 X142.191 Y49.464 E0.01942
G1 X141.594 Y48.867 E0.03056
M204 S1250
; stop printing object Petg print.STL id:28 copy 0
```

```
G1 E-4.00000 F2400.000
; Filament-specific end gcode
M600
T1
M900 K0.2 ; Filament gcode
; printing object tpu print.STL id:19 copy 0
G1 E-3.20000
G1 Z0.400 F9000.000
G1 X41.467 Y48.867
G1 Z0.200
G1 E3.20000 F1500.000
M204 S800
;TYPE:Perimeter
;WIDTH:0.42
G1 F1200.000
G1 X41.467 Y48.991 E0.00387
G1 X34.435 Y48.991 E0.22049
G1 X33.581 Y49.043 E0.02682
G1 X32.716 Y49.205 E0.02759
G1 X31.878 Y49.472 E0.02759
G1 X31.079 Y49.840 E0.02759
G1 X30.331 Y50.304 E0.02759
G1 X29.646 Y50.856 E0.02759
G1 X29.034 Y51.488 E0.02759
G1 X28.504 Y52.191 E0.02759
G1 X28.065 Y52.953 E0.02759
G1 X27.723 Y53.764 E0.02759
G1 X27.659 Y53.991 E0.00740
G1 X21.041 Y53.991 E0.20749
```

```
G1 X21.041 Y40.565 E0.42096
G1 X27.659 Y40.565 E0.20749
M73 P12 R59
G1 X27.723 Y40.792 E0.00740
G1 X28.065 Y41.603 E0.02759
G1 X28.504 Y42.365 E0.02759
G1 X29.034 Y43.068 E0.02759
G1 X29.646 Y43.700 E0.02759
G1 X30.331 Y44.252 E0.02759
G1 X31.079 Y44.715 E0.02759
G1 X31.878 Y45.083 E0.02759
G1 X32.716 Y45.350 E0.02759
G1 X33.581 Y45.512 E0.02759
G1 X34.435 Y45.565 E0.02682
G1 X41.467 Y45.565 E0.22049
G1 X41.467 Y48.807 E0.10167
M204 S1250
G1 X41.844 Y49.368 F9000.000
M204 S800
;TYPE:External perimeter
G1 F1200.000
G1 X34.446 Y49.368 E0.23195
G1 X33.627 Y49.418 E0.02573
G1 X32.808 Y49.572 E0.02613
G1 X32.015 Y49.825 E0.02613
G1 X31.258 Y50.173 E0.02613
G1 X30.550 Y50.612 E0.02613
G1 X29.901 Y51.135 E0.02613
G1 X29.321 Y51.734 E0.02613
```

```
G1 X28.820 Y52.399 E0.02613
G1 X28.404 Y53.121 E0.02613
G1 X28.080 Y53.889 E0.02613
G1 X27.944 Y54.368 E0.01561
G1 X20.664 Y54.368 E0.22826
G1 X20.664 Y40.188 E0.44460
G1 X27.944 Y40.188 E0.22826
G1 X28.080 Y40.667 E0.01561
G1 X28.404 Y41.435 E0.02613
G1 X28.820 Y42.157 E0.02613
G1 X29.321 Y42.822 E0.02613
G1 X29.901 Y43.421 E0.02613
G1 X30.550 Y43.944 E0.02613
G1 X31.258 Y44.383 E0.02613
G1 X32.015 Y44.731 E0.02613
G1 X32.808 Y44.984 E0.02613
G1 X33.627 Y45.137 E0.02613
G1 X34.446 Y45.188 E0.02573
G1 X41.844 Y45.188 E0.23195
G1 X41.844 Y49.308 E0.12918
M204 S1250
G1 X41.450 Y49.300 F9000.000
G1 E-2.24000 F2400.000
;WIPE_START
G1 F7200.000
G1 X38.964 Y49.331 E-0.91200
;WIPE_END
G1 E-0.04800 F2400.000
G1 Z0.400 F9000.000
```

```
G1 X41.354 Y46.446
G1 Z0.200
G1 E3.20000 F1500.000
M204 S800
;TYPE:Solid infill
;WIDTH:0.422818
G1 F1200.000
G1 X40.756 Y45.848 E0.02674
G1 X40.218 Y45.848 E0.01697
G1 X41.184 Y46.814 E0.04316
G1 X41.184 Y47.351 E0.01697
G1 X39.681 Y45.848 E0.06716
G1 X39.144 Y45.848 E0.01697
G1 X41.184 Y47.888 E0.09116
G1 X41.184 Y48.426 E0.01697
G1 X38.607 Y45.848 E0.11516
G1 X38.069 Y45.848 E0.01697
G1 X40.929 Y48.708 E0.12778
G1 X40.392 Y48.708 E0.01697
G1 X37.532 Y45.848 E0.12778
G1 X36.995 Y45.848 E0.01697
G1 X39.855 Y48.708 E0.12778
G1 X39.318 Y48.708 E0.01697
G1 X36.457 Y45.848 E0.12778
G1 X35.920 Y45.848 E0.01697
G1 X38.780 Y48.708 E0.12778
G1 X38.243 Y48.708 E0.01697
G1 X35.383 Y45.848 E0.12778
G1 X34.846 Y45.848 E0.01697
```

```
G1 X37.706 Y48.708 E0.12778
G1 X37.169 Y48.708 E0.01697
G1 X34.301 Y45.840 E0.12812
G1 X33.728 Y45.805 E0.01812
G1 X36.631 Y48.708 E0.12970
G1 X36.094 Y48.708 E0.01697
G1 X33.095 Y45.709 E0.13397
M73 P13 R59
G1 X32.647 Y45.625 E0.01440
G1 X32.393 Y45.544 E0.00841
G1 X35.557 Y48.708 E0.14133
G1 X35.020 Y48.708 E0.01697
G1 X31.214 Y44.902 E0.17002
M204 S1250
G1 E-2.24000 F2400.000
;WIPE_START
G1 F7200.000
G1 X33.250 Y46.938 E-0.91200
;WIPE_END
G1 E-0.04800 F2400.000
G1 Z0.400 F9000.000
G1 X27.963 Y41.652
G1 Z0.200
G1 E3.20000 F1500.000
M204 S800
G1 F1200.000
G1 X27.159 Y40.848 E0.03591
G1 X26.622 Y40.848 E0.01697
G1 X34.482 Y48.708 E0.35114
```

```
G1 X33.973 Y48.736 E0.01611
G1 X26.085 Y40.848 E0.35239
G1 X25.548 Y40.848 E0.01697
G1 X33.475 Y48.776 E0.35416
G1 X33.023 Y48.860 E0.01454
G1 X25.010 Y40.848 E0.35794
G1 X24.473 Y40.848 E0.01697
G1 X32.578 Y48.953 E0.36207
G1 X32.170 Y49.082 E0.01351
G1 X23.936 Y40.848 E0.36786
G1 X23.399 Y40.848 E0.01697
G1 X31.764 Y49.213 E0.37372
G1 X31.396 Y49.383 E0.01279
G1 X22.861 Y40.848 E0.38128
G1 X22.324 Y40.848 E0.01697
G1 X31.029 Y49.552 E0.38885
G1 X30.689 Y49.749 E0.01242
G1 X21.787 Y40.848 E0.39767
G1 X21.324 Y40.848 E0.01462
G1 X21.324 Y40.922 E0.00236
G1 X30.357 Y49.955 E0.40352
G1 X30.040 Y50.175 E0.01219
G1 X21.324 Y41.460 E0.38936
G1 X21.324 Y41.997 E0.01697
G1 X29.742 Y50.415 E0.37607
G1 X29.446 Y50.656 E0.01206
G1 X21.324 Y42.534 E0.36283
G1 X21.324 Y43.071 E0.01697
G1 X29.182 Y50.929 E0.35102
```

```
G1 X28.917 Y51.202 E0.01200
G1 X21.324 Y43.609 E0.33922
G1 X21.324 Y44.146 E0.01697
G1 X28.674 Y51.496 E0.32834
G1 X28.443 Y51.802 E0.01212
G1 X21.324 Y44.683 E0.31803
G1 X21.324 Y45.220 E0.01697
G1 X28.221 Y52.117 E0.30809
G1 X28.024 Y52.458 E0.01243
G1 X21.324 Y45.758 E0.29932
G1 X21.324 Y46.295 E0.01697
G1 X27.828 Y52.799 E0.29054
G1 X27.665 Y53.173 E0.01290
G1 X21.324 Y46.832 E0.28329
G1 X21.324 Y47.369 E0.01697
G1 X27.506 Y53.551 E0.27616
G1 X27.445 Y53.708 E0.00531
G1 X27.126 Y53.708 E0.01010
G1 X21.324 Y47.907 E0.25916
G1 X21.324 Y48.444 E0.01697
G1 X26.588 Y53.708 E0.23516
G1 X26.051 Y53.708 E0.01697
G1 X21.324 Y48.981 E0.21116
G1 X21.324 Y49.518 E0.01697
G1 X25.514 Y53.708 E0.18716
G1 X24.976 Y53.708 E0.01697
G1 X21.324 Y50.056 E0.16316
G1 X21.324 Y50.593 E0.01697
G1 X24.439 Y53.708 E0.13916
```

```
G1 X23.902 Y53.708 E0.01697
G1 X21.324 Y51.130 E0.11516
G1 X21.324 Y51.667 E0.01697
G1 X23.365 Y53.708 E0.09116
G1 X22.827 Y53.708 E0.01697
G1 X21.324 Y52.205 E0.06716
G1 X21.324 Y52.742 E0.01697
G1 X22.290 Y53.708 E0.04316
G1 X21.753 Y53.708 E0.01697
G1 X21.154 Y53.109 E0.02673
M204 S1250
; stop printing object tpu print.STL id:19 copy 0
; printing object Petg print.STL id:18 copy 0
; stop printing object Petg print.STL id:18 copy 0
; printing object Petg print.STL id:14 copy 0
; stop printing object Petg print.STL id:14 copy 0
; printing object tpu print.STL id:15 copy 0
G1 E-2.24000 F2400.000
;WIPE_START
G1 F7200.000
G1 X21.753 Y53.708 E-0.26801
G1 X22.290 Y53.708 E-0.17013
G1 X21.324 Y52.742 E-0.43262
G1 X21.324 Y52.612 E-0.04124
;WIPE_END
G1 E-0.04800 F2400.000
G1 Z0.400 F9000.000
G1 X21.154 Y60.514
G1 Z0.200
```

```
G1 E3.20000 F1500.000
M204 S800
;TYPE:Perimeter
;WIDTH:0.42
G1 F1200.000
G1 X27.667 Y60.514 E0.20418
G1 X27.731 Y60.741 E0.00740
G1 X28.073 Y61.552 E0.02759
G1 X28.512 Y62.314 E0.02759
G1 X29.042 Y63.017 E0.02759
G1 X29.653 Y63.649 E0.02759
G1 X30.339 Y64.201 E0.02759
G1 X31.086 Y64.664 E0.02759
G1 X31.885 Y65.032 E0.02759
G1 X32.724 Y65.299 E0.02759
G1 X33.588 Y65.461 E0.02759
G1 X34.442 Y65.514 E0.02682
G1 X41.475 Y65.514 E0.22049
G1 X41.475 Y68.940 E0.10741
G1 X34.442 Y68.940 E0.22049
G1 X33.588 Y68.992 E0.02682
G1 X32.724 Y69.154 E0.02759
G1 X31.885 Y69.421 E0.02759
G1 X31.086 Y69.789 E0.02759
G1 X30.339 Y70.253 E0.02759
G1 X29.653 Y70.805 E0.02759
G1 X29.042 Y71.437 E0.02759
G1 X28.512 Y72.140 E0.02759
G1 X28.073 Y72.902 E0.02759
```

```
G1 X27.731 Y73.713 E0.02759
G1 X27.667 Y73.940 E0.00740
G1 X21.049 Y73.940 E0.20749
G1 X21.049 Y60.514 E0.42096
G1 X21.094 Y60.514 E0.00143
M204 S1250
G1 X20.672 Y60.137 F9000.000
M204 S800
;TYPE:External perimeter
G1 F1200.000
G1 X27.952 Y60.137 E0.22826
G1 X28.087 Y60.616 E0.01561
G1 X28.411 Y61.384 E0.02613
G1 X28.827 Y62.106 E0.02613
G1 X29.329 Y62.771 E0.02613
G1 X29.908 Y63.370 E0.02613
G1 X30.557 Y63.892 E0.02613
G1 X31.265 Y64.332 E0.02613
G1 X32.022 Y64.680 E0.02613
G1 X32.816 Y64.933 E0.02613
G1 X33.635 Y65.086 E0.02613
G1 X34.454 Y65.137 E0.02573
G1 X41.852 Y65.137 E0.23195
G1 X41.852 Y69.317 E0.13106
G1 X34.454 Y69.317 E0.23195
G1 X33.635 Y69.367 E0.02573
G1 X32.816 Y69.521 E0.02613
G1 X32.022 Y69.773 E0.02613
G1 X31.265 Y70.122 E0.02613
```

```
G1 X30.557 Y70.561 E0.02613
G1 X29.908 Y71.084 E0.02613
G1 X29.329 Y71.683 E0.02613
G1 X28.827 Y72.348 E0.02613
G1 X28.411 Y73.070 E0.02613
G1 X28.087 Y73.838 E0.02613
G1 X27.952 Y74.317 E0.01561
G1 X20.672 Y74.317 E0.22826
G1 X20.672 Y60.197 E0.44272
M204 S1250
G1 X21.045 Y60.280 F9000.000
G1 E-2.24000 F2400.000
;WIPE_START
G1 F7200.000
G1 X23.552 Y60.173 E-0.91200
;WIPE_END
G1 E-0.04800 F2400.000
G1 Z0.400 F9000.000
G1 X41.362 Y66.395
G1 Z0.200
G1 E3.20000 F1500.000
M204 S800
;TYPE:Solid infill
;WIDTH:0.422818
G1 F1200.000
G1 X40.763 Y65.797 E0.02674
G1 X40.226 Y65.797 E0.01697
G1 X41.192 Y66.763 E0.04316
G1 X41.192 Y67.300 E0.01697
```

```
G1 X39.689 Y65.797 E0.06716
G1 X39.151 Y65.797 E0.01697
G1 X41.192 Y67.837 E0.09116
G1 X41.192 Y68.375 E0.01697
G1 X38.614 Y65.797 E0.11516
G1 X38.077 Y65.797 E0.01697
G1 X40.937 Y68.657 E0.12778
G1 X40.400 Y68.657 E0.01697
G1 X37.539 Y65.797 E0.12778
G1 X37.002 Y65.797 E0.01697
G1 X39.862 Y68.657 E0.12778
G1 X39.325 Y68.657 E0.01697
G1 X36.465 Y65.797 E0.12778
G1 X35.928 Y65.797 E0.01697
G1 X38.788 Y68.657 E0.12778
G1 X38.251 Y68.657 E0.01697
G1 X35.390 Y65.797 E0.12778
G1 X34.853 Y65.797 E0.01697
G1 X37.713 Y68.657 E0.12778
G1 X37.176 Y68.657 E0.01697
G1 X34.308 Y65.789 E0.12812
G1 X33.736 Y65.754 E0.01812
G1 X36.639 Y68.657 E0.12970
G1 X36.102 Y68.657 E0.01697
G1 X33.103 Y65.658 E0.13397
G1 X32.655 Y65.574 E0.01440
G1 X32.401 Y65.493 E0.00841
G1 X35.564 Y68.657 E0.14133
G1 X35.027 Y68.657 E0.01697
```

```
G1 X31.221 Y64.851 E0.17002
M204 S1250
G1 E-2.24000 F2400.000
;WIPE_START
G1 F7200.000
G1 X33.258 Y66.887 E-0.91200
;WIPE_END
G1 E-0.04800 F2400.000
G1 Z0.400 F9000.000
G1 X27.971 Y61.600
G1 Z0.200
G1 E3.20000 F1500.000
M204 S800
G1 F1200.000
G1 X27.167 Y60.797 E0.03591
G1 X26.630 Y60.797 E0.01697
G1 X34.490 Y68.657 E0.35114
G1 X33.981 Y68.685 E0.01611
G1 X26.092 Y60.797 E0.35239
G1 X25.555 Y60.797 E0.01697
G1 X33.483 Y68.724 E0.35416
G1 X33.030 Y68.809 E0.01454
G1 X25.018 Y60.797 E0.35794
G1 X24.481 Y60.797 E0.01697
G1 X32.585 Y68.901 E0.36207
G1 X32.178 Y69.031 E0.01351
G1 X23.943 Y60.797 E0.36786
G1 X23.406 Y60.797 E0.01697
G1 X31.772 Y69.162 E0.37372
```

G1 X31.404 Y69.332 E0.01279

G1 X22.869 Y60.797 E0.38128

G1 X22.332 Y60.797 E0.01697

G1 X31.036 Y69.501 E0.38885

G1 X30.696 Y69.698 E0.01242

G1 X21.794 Y60.797 E0.39767

G1 X21.332 Y60.797 E0.01462

G1 X21.332 Y60.871 E0.00236

G1 X30.364 Y69.904 E0.40352

G1 X30.047 Y70.124 E0.01219

G1 X21.332 Y61.408 E0.38936

G1 X21.332 Y61.946 E0.01697

G1 X29.750 Y70.364 E0.37607

G1 X29.454 Y70.605 E0.01206

G1 X21.332 Y62.483 E0.36283

G1 X21.332 Y63.020 E0.01697

G1 X29.189 Y70.878 E0.35102

G1 X28.925 Y71.151 E0.01200

G1 X21.332 Y63.558 E0.33922

G1 X21.332 Y64.095 E0.01697

G1 X28.682 Y71.445 E0.32834

G1 X28.451 Y71.751 E0.01212

G1 X21.332 Y64.632 E0.31803

G1 X21.332 Y65.169 E0.01697

G1 X28.228 Y72.066 E0.30809

M73 P14 R59

G1 X28.032 Y72.407 E0.01243

G1 X21.332 Y65.707 E0.29932

G1 X21.332 Y66.244 E0.01697

```
G1 X27.835 Y72.748 E0.29054
G1 X27.673 Y73.122 E0.01290
G1 X21.332 Y66.781 E0.28329
G1 X21.332 Y67.318 E0.01697
G1 X27.514 Y73.500 E0.27616
G1 X27.453 Y73.657 E0.00531
G1 X27.133 Y73.657 E0.01010
G1 X21.332 Y67.856 E0.25916
G1 X21.332 Y68.393 E0.01697
G1 X26.596 Y73.657 E0.23516
G1 X26.058 Y73.657 E0.01697
G1 X21.332 Y68.930 E0.21116
G1 X21.332 Y69.467 E0.01697
G1 X25.521 Y73.657 E0.18716
G1 X24.984 Y73.657 E0.01697
G1 X21.332 Y70.005 E0.16316
G1 X21.332 Y70.542 E0.01697
G1 X24.447 Y73.657 E0.13916
G1 X23.909 Y73.657 E0.01697
G1 X21.332 Y71.079 E0.11516
G1 X21.332 Y71.616 E0.01697
G1 X23.372 Y73.657 E0.09116
G1 X22.835 Y73.657 E0.01697
G1 X21.332 Y72.154 E0.06716
G1 X21.332 Y72.691 E0.01697
G1 X22.298 Y73.657 E0.04316
G1 X21.760 Y73.657 E0.01697
G1 X21.162 Y73.058 E0.02673
M204 S1250
```

```
; stop printing object tpu print.STL id:15 copy 0
; printing object Petg print.STL id:10 copy 0
; stop printing object Petg print.STL id:10 copy 0
; printing object tpu print.STL id:11 copy 0
G1 E-2.24000 F2400.000
;WIPE_START
G1 F7200.000
G1 X21.760 Y73.657 E-0.26801
G1 X22.298 Y73.657 E-0.17013
G1 X21.332 Y72.691 E-0.43262
G1 X21.332 Y72.561 E-0.04124
;WIPE_END
G1 E-0.04800 F2400.000
G1 Z0.400 F9000.000
G1 X21.162 Y82.706
G1 Z0.200
G1 E3.20000 F1500.000
M204 S800
;TYPE:Perimeter
;WIDTH:0.42
G1 F1200.000
G1 X27.615 Y82.706 E0.20234
G1 X27.680 Y82.933 E0.00740
G1 X28.022 Y83.744 E0.02759
G1 X28.461 Y84.506 E0.02759
G1 X28.990 Y85.209 E0.02759
G1 X29.602 Y85.841 E0.02759
G1 X30.287 Y86.393 E0.02759
G1 X31.035 Y86.857 E0.02759
```

```
G1 X31.834 Y87.225 E0.02759
G1 X32.673 Y87.492 E0.02759
G1 X33.537 Y87.653 E0.02759
G1 X34.391 Y87.706 E0.02682
G1 X41.424 Y87.706 E0.22049
G1 X41.424 Y91.132 E0.10741
G1 X34.391 Y91.132 E0.22049
G1 X33.537 Y91.185 E0.02682
G1 X32.673 Y91.347 E0.02759
G1 X31.834 Y91.614 E0.02759
G1 X31.035 Y91.982 E0.02759
G1 X30.287 Y92.445 E0.02759
G1 X29.602 Y92.997 E0.02759
G1 X28.990 Y93.629 E0.02759
G1 X28.461 Y94.332 E0.02759
G1 X28.022 Y95.094 E0.02759
G1 X27.680 Y95.905 E0.02759
G1 X27.615 Y96.132 E0.00740
G1 X20.998 Y96.132 E0.20749
G1 X20.998 Y82.706 E0.42096
G1 X21.102 Y82.706 E0.00327
M204 S1250
G1 X20.621 Y82.329 F9000.000
M204 S800
;TYPE:External perimeter
G1 F1200.000
G1 X27.901 Y82.329 E0.22826
G1 X28.036 Y82.808 E0.01561
G1 X28.360 Y83.576 E0.02613
```

```
G1 X28.776 Y84.298 E0.02613
G1 X29.277 Y84.963 E0.02613
G1 X29.857 Y85.562 E0.02613
G1 X30.506 Y86.085 E0.02613
G1 X31.214 Y86.524 E0.02613
G1 X31.971 Y86.872 E0.02613
G1 X32.765 Y87.125 E0.02613
G1 X33.584 Y87.279 E0.02613
G1 X34.403 Y87.329 E0.02573
G1 X41.801 Y87.329 E0.23195
G1 X41.801 Y91.509 E0.13106
G1 X34.403 Y91.509 E0.23195
G1 X33.584 Y91.560 E0.02573
G1 X32.765 Y91.713 E0.02613
G1 X31.971 Y91.966 E0.02613
G1 X31.214 Y92.314 E0.02613
G1 X30.506 Y92.753 E0.02613
G1 X29.857 Y93.276 E0.02613
G1 X29.277 Y93.875 E0.02613
G1 X28.776 Y94.540 E0.02613
G1 X28.360 Y95.262 E0.02613
G1 X28.036 Y96.030 E0.02613
G1 X27.901 Y96.509 E0.01561
G1 X20.621 Y96.509 E0.22826
M73 P14 R58
G1 X20.621 Y82.389 E0.44272
M204 S1250
G1 X20.994 Y82.472 F9000.000
G1 E-2.24000 F2400.000
```

```
;WIPE_START
G1 F7200.000
G1 X23.501 Y82.365 E-0.91200
;WIPE_END
G1 E-0.04800 F2400.000
G1 Z0.400 F9000.000
G1 X41.310 Y88.588
G1 Z0.200
G1 E3.20000 F1500.000
M204 S800
;TYPE:Solid infill
;WIDTH:0.422818
G1 F1200.000
G1 X40.712 Y87.989 E0.02674
G1 X40.175 Y87.989 E0.01697
G1 X41.141 Y88.955 E0.04316
G1 X41.141 Y89.492 E0.01697
G1 X39.637 Y87.989 E0.06716
G1 X39.100 Y87.989 E0.01697
G1 X41.141 Y90.030 E0.09116
G1 X41.141 Y90.567 E0.01697
G1 X38.563 Y87.989 E0.11516
G1 X38.026 Y87.989 E0.01697
G1 X40.886 Y90.849 E0.12778
G1 X40.349 Y90.849 E0.01697
G1 X37.488 Y87.989 E0.12778
G1 X36.951 Y87.989 E0.01697
G1 X39.811 Y90.849 E0.12778
G1 X39.274 Y90.849 E0.01697
```

```
G1 X36.414 Y87.989 E0.12778
G1 X35.877 Y87.989 E0.01697
G1 X38.737 Y90.849 E0.12778
G1 X38.200 Y90.849 E0.01697
G1 X35.339 Y87.989 E0.12778
G1 X34.802 Y87.989 E0.01697
G1 X37.662 Y90.849 E0.12778
G1 X37.125 Y90.849 E0.01697
G1 X34.257 Y87.981 E0.12812
G1 X33.684 Y87.946 E0.01812
G1 X36.588 Y90.849 E0.12970
G1 X36.051 Y90.849 E0.01697
G1 X33.052 Y87.850 E0.13397
G1 X32.603 Y87.766 E0.01440
G1 X32.350 Y87.686 E0.00841
G1 X35.513 Y90.849 E0.14133
G1 X34.976 Y90.849 E0.01697
G1 X31.170 Y87.043 E0.17002
M204 S1250
G1 E-2.24000 F2400.000
;WIPE_START
G1 F7200.000
G1 X33.207 Y89.080 E-0.91200
;WIPE_END
G1 E-0.04800 F2400.000
G1 Z0.400 F9000.000
G1 X27.920 Y83.793
G1 Z0.200
G1 E3.20000 F1500.000
```

```
M204 S800
G1 F1200.000
G1 X27.116 Y82.989 E0.03591
G1 X26.579 Y82.989 E0.01697
G1 X34.439 Y90.849 E0.35114
G1 X33.929 Y90.877 E0.01611
G1 X26.041 Y82.989 E0.35239
G1 X25.504 Y82.989 E0.01697
G1 X33.432 Y90.917 E0.35416
G1 X32.979 Y91.001 E0.01454
G1 X24.967 Y82.989 E0.35794
G1 X24.429 Y82.989 E0.01697
G1 X32.534 Y91.094 E0.36207
G1 X32.127 Y91.224 E0.01351
G1 X23.892 Y82.989 E0.36786
G1 X23.355 Y82.989 E0.01697
G1 X31.721 Y91.355 E0.37372
G1 X31.353 Y91.524 E0.01279
G1 X22.818 Y82.989 E0.38128
G1 X22.280 Y82.989 E0.01697
G1 X30.985 Y91.693 E0.38885
G1 X30.645 Y91.891 E0.01242
G1 X21.743 Y82.989 E0.39767
G1 X21.281 Y82.989 E0.01462
G1 X21.281 Y83.064 E0.00236
G1 X30.313 Y92.096 E0.40352
G1 X29.996 Y92.317 E0.01219
G1 X21.281 Y83.601 E0.38936
G1 X21.281 Y84.138 E0.01697
```

```
G1 X29.699 Y92.556 E0.37607
G1 X29.402 Y92.797 E0.01206
G1 X21.281 Y84.675 E0.36283
G1 X21.281 Y85.213 E0.01697
G1 X29.138 Y93.070 E0.35102
G1 X28.874 Y93.343 E0.01200
G1 X21.281 Y85.750 E0.33922
G1 X21.281 Y86.287 E0.01697
G1 X28.630 Y93.637 E0.32834
G1 X28.400 Y93.943 E0.01212
G1 X21.281 Y86.824 E0.31803
G1 X21.281 Y87.362 E0.01697
G1 X28.177 Y94.258 E0.30809
G1 X27.981 Y94.599 E0.01243
G1 X21.281 Y87.899 E0.29932
G1 X21.281 Y88.436 E0.01697
G1 X27.784 Y94.940 E0.29054
G1 X27.622 Y95.315 E0.01290
G1 X21.281 Y88.973 E0.28329
G1 X21.281 Y89.511 E0.01697
G1 X27.462 Y95.693 E0.27616
G1 X27.402 Y95.849 E0.00531
G1 X27.082 Y95.849 E0.01010
G1 X21.281 Y90.048 E0.25916
G1 X21.281 Y90.585 E0.01697
G1 X26.545 Y95.849 E0.23516
G1 X26.007 Y95.849 E0.01697
G1 X21.281 Y91.122 E0.21116
G1 X21.281 Y91.660 E0.01697
```

```
G1 X25.470 Y95.849 E0.18716
G1 X24.933 Y95.849 E0.01697
G1 X21.281 Y92.197 E0.16316
G1 X21.281 Y92.734 E0.01697
G1 X24.396 Y95.849 E0.13916
G1 X23.858 Y95.849 E0.01697
G1 X21.281 Y93.271 E0.11516
G1 X21.281 Y93.809 E0.01697
G1 X23.321 Y95.849 E0.09116
G1 X22.784 Y95.849 E0.01697
G1 X21.281 Y94.346 E0.06716
G1 X21.281 Y94.883 E0.01697
G1 X22.247 Y95.849 E0.04316
G1 X21.709 Y95.849 E0.01697
G1 X21.111 Y95.251 E0.02673
M204 S1250
; stop printing object tpu print.STL id:11 copy 0
; printing object tpu print.STL id:13 copy 0
G1 E-2.24000 F2400.000
;WIPE_START
G1 F7200.000
G1 X21.709 Y95.849 E-0.26801
G1 X22.247 Y95.849 E-0.17013
G1 X21.281 Y94.883 E-0.43262
G1 X21.281 Y94.753 E-0.04124
;WIPE_END
G1 E-0.04800 F2400.000
G1 Z0.400 F9000.000
G1 X21.111 Y103.522
```

```
G1 Z0.200
G1 E3.20000 F1500.000
M204 S800
;TYPE:Perimeter
;WIDTH:0.42
G1 F1200.000
G1 X27.622 Y103.522 E0.20416
G1 X27.687 Y103.749 E0.00740
G1 X28.029 Y104.559 E0.02759
G1 X28.468 Y105.322 E0.02759
G1 X28.997 Y106.024 E0.02759
G1 X29.609 Y106.657 E0.02759
G1 X30.294 Y107.209 E0.02759
G1 X31.042 Y107.672 E0.02759
G1 X31.841 Y108.040 E0.02759
G1 X32.679 Y108.307 E0.02759
G1 X33.544 Y108.469 E0.02759
G1 X34.398 Y108.522 E0.02682
G1 X41.430 Y108.522 E0.22049
G1 X41.430 Y111.948 E0.10741
G1 X34.398 Y111.948 E0.22049
G1 X33.544 Y112.000 E0.02682
G1 X32.679 Y112.162 E0.02759
G1 X31.841 Y112.429 E0.02759
G1 X31.042 Y112.797 E0.02759
G1 X30.294 Y113.261 E0.02759
G1 X29.609 Y113.813 E0.02759
G1 X28.997 Y114.445 E0.02759
G1 X28.468 Y115.147 E0.02759
```

```
G1 X28.029 Y115.910 E0.02759
G1 X27.687 Y116.720 E0.02759
G1 X27.622 Y116.948 E0.00740
G1 X21.005 Y116.948 E0.20749
G1 X21.005 Y103.522 E0.42096
G1 X21.051 Y103.522 E0.00145
M204 S1250
G1 X20.627 Y103.145 F9000.000
M204 S800
;TYPE:External perimeter
G1 F1200.000
G1 X27.907 Y103.145 E0.22826
G1 X28.043 Y103.624 E0.01561
G1 X28.367 Y104.391 E0.02613
G1 X28.783 Y105.113 E0.02613
G1 X29.284 Y105.779 E0.02613
G1 X29.864 Y106.378 E0.02613
G1 X30.513 Y106.900 E0.02613
G1 X31.221 Y107.339 E0.02613
G1 X31.978 Y107.688 E0.02613
G1 X32.772 Y107.941 E0.02613
G1 X33.591 Y108.094 E0.02613
G1 X34.410 Y108.145 E0.02573
G1 X41.807 Y108.145 E0.23195
G1 X41.807 Y112.325 E0.13106
G1 X34.410 Y112.325 E0.23195
G1 X33.591 Y112.375 E0.02573
G1 X32.772 Y112.528 E0.02613
G1 X31.978 Y112.781 E0.02613
```

```
G1 X31.221 Y113.130 E0.02613
G1 X30.513 Y113.569 E0.02613
G1 X29.864 Y114.092 E0.02613
G1 X29.284 Y114.690 E0.02613
G1 X28.783 Y115.356 E0.02613
G1 X28.367 Y116.078 E0.02613
G1 X28.043 Y116.846 E0.02613
G1 X27.907 Y117.325 E0.01561
G1 X20.627 Y117.325 E0.22826
G1 X20.627 Y103.205 E0.44272
M204 S1250
G1 X21.001 Y103.288 F9000.000
G1 E-2.24000 F2400.000
;WIPE_START
G1 F7200.000
G1 X23.507 Y103.181 E-0.91200
;WIPE_END
G1 E-0.04800 F2400.000
G1 Z0.400 F9000.000
G1 X41.317 Y109.403
M73 P15 R58
G1 Z0.200
G1 E3.20000 F1500.000
M204 S800
;TYPE:Solid infill
;WIDTH:0.422818
G1 F1200.000
G1 X40.719 Y108.805 E0.02674
G1 X40.181 Y108.805 E0.01697
```

G1 X41.148 Y109.771 E0.04316

G1 X41.148 Y110.308 E0.01697

G1 X39.644 Y108.805 E0.06716

G1 X39.107 Y108.805 E0.01697

G1 X41.148 Y110.845 E0.09116

G1 X41.148 Y111.382 E0.01697

G1 X38.570 Y108.805 E0.11516

G1 X38.032 Y108.805 E0.01697

G1 X40.893 Y111.665 E0.12778

G1 X40.355 Y111.665 E0.01697

G1 X37.495 Y108.805 E0.12778

G1 X36.958 Y108.805 E0.01697

G1 X39.818 Y111.665 E0.12778

G1 X39.281 Y111.665 E0.01697

G1 X36.421 Y108.805 E0.12778

G1 X35.883 Y108.805 E0.01697

G1 X38.744 Y111.665 E0.12778

G1 X38.206 Y111.665 E0.01697

G1 X35.346 Y108.805 E0.12778

G1 X34.809 Y108.805 E0.01697

G1 X37.669 Y111.665 E0.12778

G1 X37.132 Y111.665 E0.01697

G1 X34.264 Y108.797 E0.12812

G1 X33.691 Y108.761 E0.01812

G1 X36.595 Y111.665 E0.12970

G1 X36.057 Y111.665 E0.01697

G1 X33.058 Y108.666 E0.13397

G1 X32.610 Y108.582 E0.01440

G1 X32.356 Y108.501 E0.00841

```
G1 X35.520 Y111.665 E0.14133
G1 X34.983 Y111.665 E0.01697
G1 X31.177 Y107.859 E0.17002
M204 S1250
G1 E-2.24000 F2400.000
;WIPE_START
G1 F7200.000
G1 X33.213 Y109.895 E-0.91200
;WIPE_END
G1 E-0.04800 F2400.000
G1 Z0.400 F9000.000
G1 X27.926 Y104.608
G1 Z0.200
G1 E3.20000 F1500.000
M204 S800
G1 F1200.000
G1 X27.123 Y103.805 E0.03591
G1 X26.585 Y103.805 E0.01697
G1 X34.446 Y111.665 E0.35114
G1 X33.936 Y111.693 E0.01611
G1 X26.048 Y103.805 E0.35239
G1 X25.511 Y103.805 E0.01697
G1 X33.439 Y111.732 E0.35416
G1 X32.986 Y111.817 E0.01454
G1 X24.974 Y103.805 E0.35794
G1 X24.436 Y103.805 E0.01697
G1 X32.541 Y111.909 E0.36207
G1 X32.134 Y112.039 E0.01351
G1 X23.899 Y103.805 E0.36786
```

G1 X23.362 Y103.805 E0.01697

G1 X31.727 Y112.170 E0.37372

G1 X31.360 Y112.340 E0.01279

G1 X22.825 Y103.805 E0.38128

G1 X22.287 Y103.805 E0.01697

G1 X30.992 Y112.509 E0.38885

G1 X30.652 Y112.706 E0.01242

G1 X21.750 Y103.805 E0.39767

G1 X21.287 Y103.805 E0.01462

G1 X21.287 Y103.879 E0.00236

G1 X30.320 Y112.912 E0.40352

G1 X30.003 Y113.132 E0.01219

G1 X21.287 Y104.416 E0.38936

G1 X21.287 Y104.954 E0.01697

G1 X29.706 Y113.372 E0.37607

G1 X29.409 Y113.613 E0.01206

G1 X21.287 Y105.491 E0.36283

G1 X21.287 Y106.028 E0.01697

G1 X29.145 Y113.886 E0.35102

G1 X28.881 Y114.159 E0.01200

G1 X21.287 Y106.565 E0.33922

G1 X21.287 Y107.103 E0.01697

G1 X28.637 Y114.453 E0.32834

G1 X28.406 Y114.759 E0.01212

G1 X21.287 Y107.640 E0.31803

G1 X21.287 Y108.177 E0.01697

G1 X28.184 Y115.074 E0.30809

G1 X27.987 Y115.415 E0.01243

G1 X21.287 Y108.714 E0.29932

```
G1 X21.287 Y109.252 E0.01697
G1 X27.791 Y115.755 E0.29054
G1 X27.629 Y116.130 E0.01290
G1 X21.287 Y109.789 E0.28329
G1 X21.287 Y110.326 E0.01697
G1 X27.469 Y116.508 E0.27616
G1 X27.408 Y116.665 E0.00531
G1 X27.089 Y116.665 E0.01010
G1 X21.287 Y110.863 E0.25916
G1 X21.287 Y111.401 E0.01697
G1 X26.551 Y116.665 E0.23516
G1 X26.014 Y116.665 E0.01697
G1 X21.287 Y111.938 E0.21116
G1 X21.287 Y112.475 E0.01697
G1 X25.477 Y116.665 E0.18716
G1 X24.940 Y116.665 E0.01697
G1 X21.287 Y113.012 E0.16316
G1 X21.287 Y113.550 E0.01697
G1 X24.402 Y116.665 E0.13916
G1 X23.865 Y116.665 E0.01697
G1 X21.287 Y114.087 E0.11516
G1 X21.287 Y114.624 E0.01697
G1 X23.328 Y116.665 E0.09116
G1 X22.791 Y116.665 E0.01697
G1 X21.287 Y115.161 E0.06716
G1 X21.287 Y115.699 E0.01697
G1 X22.253 Y116.665 E0.04316
G1 X21.716 Y116.665 E0.01697
G1 X21.118 Y116.066 E0.02673
```

```
M204 S1250
; stop printing object tpu print.STL id:13 copy 0
; printing object tpu print.STL id:17 copy 0
G1 E-2.24000 F2400.000
;WIPE_START
G1 F7200.000
G1 X21.716 Y116.665 E-0.26801
G1 X22.253 Y116.665 E-0.17013
G1 X21.287 Y115.699 E-0.43262
G1 X21.287 Y115.569 E-0.04124
;WIPE_END
G1 E-0.04800 F2400.000
G1 Z0.400 F9000.000
G1 X21.118 Y122.176
G1 Z0.200
G1 E3.20000 F1500.000
M204 S800
;TYPE:Perimeter
;WIDTH:0.42
G1 F1200.000
G1 X27.607 Y122.176 E0.20347
G1 X27.671 Y122.403 E0.00740
G1 X28.013 Y123.213 E0.02759
G1 X28.453 Y123.976 E0.02759
G1 X28.982 Y124.678 E0.02759
G1 X29.594 Y125.310 E0.02759
G1 X30.279 Y125.863 E0.02759
G1 X31.027 Y126.326 E0.02759
G1 X31.826 Y126.694 E0.02759
```

```
G1 X32.664 Y126.961 E0.02759
G1 X33.529 Y127.123 E0.02759
G1 X34.383 Y127.176 E0.02682
G1 X41.415 Y127.176 E0.22049
G1 X41.415 Y130.602 E0.10741
G1 X34.383 Y130.602 E0.22049
G1 X33.529 Y130.654 E0.02682
G1 X32.664 Y130.816 E0.02759
G1 X31.826 Y131.083 E0.02759
G1 X31.027 Y131.451 E0.02759
G1 X30.279 Y131.915 E0.02759
G1 X29.594 Y132.467 E0.02759
G1 X28.982 Y133.099 E0.02759
G1 X28.453 Y133.801 E0.02759
G1 X28.013 Y134.564 E0.02759
G1 X27.671 Y135.374 E0.02759
G1 X27.607 Y135.602 E0.00740
G1 X20.989 Y135.602 E0.20749
G1 X20.989 Y122.176 E0.42096
G1 X21.058 Y122.176 E0.00214
M204 S1250
G1 X20.612 Y121.799 F9000.000
M204 S800
;TYPE:External perimeter
G1 F1200.000
G1 X27.892 Y121.799 E0.22826
G1 X28.028 Y122.278 E0.01561
G1 X28.352 Y123.045 E0.02613
G1 X28.768 Y123.767 E0.02613
```

```
G1 X29.269 Y124.433 E0.02613
G1 X29.849 Y125.031 E0.02613
G1 X30.498 Y125.554 E0.02613
G1 X31.206 Y125.993 E0.02613
G1 X31.963 Y126.342 E0.02613
G1 X32.757 Y126.595 E0.02613
G1 X33.576 Y126.748 E0.02613
G1 X34.395 Y126.799 E0.02573
G1 X41.792 Y126.799 E0.23195
G1 X41.792 Y130.979 E0.13106
G1 X34.395 Y130.979 E0.23195
G1 X33.576 Y131.029 E0.02573
G1 X32.757 Y131.182 E0.02613
G1 X31.963 Y131.435 E0.02613
G1 X31.206 Y131.784 E0.02613
G1 X30.498 Y132.223 E0.02613
G1 X29.849 Y132.746 E0.02613
G1 X29.269 Y133.344 E0.02613
G1 X28.768 Y134.010 E0.02613
G1 X28.352 Y134.732 E0.02613
G1 X28.028 Y135.500 E0.02613
G1 X27.892 Y135.979 E0.01561
G1 X20.612 Y135.979 E0.22826
G1 X20.612 Y121.859 E0.44272
M204 S1250
G1 X20.986 Y121.942 F9000.000
G1 E-2.24000 F2400.000
;WIPE_START
G1 F7200.000
```

```
G1 X23.492 Y121.835 E-0.91200
;WIPE_END
G1 E-0.04800 F2400.000
G1 Z0.400 F9000.000
G1 X41.302 Y128.057
G1 Z0.200
G1 E3.20000 F1500.000
M204 S800
;TYPE:Solid infill
;WIDTH:0.422818
G1 F1200.000
G1 X40.704 Y127.458 E0.02674
G1 X40.166 Y127.458 E0.01697
G1 X41.132 Y128.425 E0.04316
G1 X41.132 Y128.962 E0.01697
G1 X39.629 Y127.458 E0.06716
G1 X39.092 Y127.458 E0.01697
G1 X41.132 Y129.499 E0.09116
G1 X41.132 Y130.036 E0.01697
G1 X38.555 Y127.458 E0.11516
G1 X38.017 Y127.458 E0.01697
G1 X40.878 Y130.319 E0.12778
G1 X40.340 Y130.319 E0.01697
G1 X37.480 Y127.458 E0.12778
G1 X36.943 Y127.458 E0.01697
G1 X39.803 Y130.319 E0.12778
G1 X39.266 Y130.319 E0.01697
G1 X36.406 Y127.458 E0.12778
M73 P15 R57
```

```
G1 X35.868 Y127.458 E0.01697
G1 X38.729 Y130.319 E0.12778
G1 X38.191 Y130.319 E0.01697
G1 X35.331 Y127.458 E0.12778
G1 X34.794 Y127.458 E0.01697
G1 X37.654 Y130.319 E0.12778
G1 X37.117 Y130.319 E0.01697
G1 X34.249 Y127.451 E0.12812
G1 X33.676 Y127.415 E0.01812
G1 X36.580 Y130.319 E0.12970
G1 X36.042 Y130.319 E0.01697
G1 X33.043 Y127.320 E0.13397
G1 X32.595 Y127.236 E0.01440
G1 X32.341 Y127.155 E0.00841
G1 X35.505 Y130.319 E0.14133
G1 X34.968 Y130.319 E0.01697
G1 X31.162 Y126.513 E0.17002
M204 S1250
G1 E-2.24000 F2400.000
;WIPE_START
G1 F7200.000
G1 X33.198 Y128.549 E-0.91200
;WIPE_END
G1 E-0.04800 F2400.000
G1 Z0.400 F9000.000
G1 X27.911 Y123.262
G1 Z0.200
G1 E3.20000 F1500.000
M204 S800
```

```
G1 F1200.000
G1 X27.107 Y122.458 E0.03591
G1 X26.570 Y122.458 E0.01697
G1 X34.430 Y130.319 E0.35114
G1 X33.921 Y130.347 E0.01611
G1 X26.033 Y122.458 E0.35239
G1 X25.496 Y122.458 E0.01697
G1 X33.424 Y130.386 E0.35416
G1 X32.971 Y130.471 E0.01454
G1 X24.958 Y122.458 E0.35794
G1 X24.421 Y122.458 E0.01697
G1 X32.526 Y130.563 E0.36207
G1 X32.119 Y130.693 E0.01351
G1 X23.884 Y122.458 E0.36786
G1 X23.347 Y122.458 E0.01697
G1 X31.712 Y130.824 E0.37372
G1 X31.344 Y130.993 E0.01279
G1 X22.809 Y122.458 E0.38128
G1 X22.272 Y122.458 E0.01697
G1 X30.977 Y131.163 E0.38885
G1 X30.637 Y131.360 E0.01242
G1 X21.735 Y122.458 E0.39767
G1 X21.272 Y122.458 E0.01462
G1 X21.272 Y122.533 E0.00236
G1 X30.305 Y131.566 E0.40352
G1 X29.988 Y131.786 E0.01219
G1 X21.272 Y123.070 E0.38936
G1 X21.272 Y123.608 E0.01697
G1 X29.690 Y132.026 E0.37607
```

```
M73 P16 R57
G1 X29.394 Y132.267 E0.01206
G1 X21.272 Y124.145 E0.36283
G1 X21.272 Y124.682 E0.01697
G1 X29.130 Y132.540 E0.35102
G1 X28.866 Y132.813 E0.01200
G1 X21.272 Y125.219 E0.33922
G1 X21.272 Y125.757 E0.01697
G1 X28.622 Y133.107 E0.32834
G1 X28.391 Y133.413 E0.01212
G1 X21.272 Y126.294 E0.31803
G1 X21.272 Y126.831 E0.01697
G1 X28.169 Y133.728 E0.30809
G1 X27.972 Y134.069 E0.01243
G1 X21.272 Y127.368 E0.29932
G1 X21.272 Y127.906 E0.01697
G1 X27.776 Y134.409 E0.29054
G1 X27.614 Y134.784 E0.01290
G1 X21.272 Y128.443 E0.28329
G1 X21.272 Y128.980 E0.01697
G1 X27.454 Y135.162 E0.27616
G1 X27.393 Y135.319 E0.00531
G1 X27.074 Y135.319 E0.01010
G1 X21.272 Y129.517 E0.25916
G1 X21.272 Y130.055 E0.01697
G1 X26.536 Y135.319 E0.23516
G1 X25.999 Y135.319 E0.01697
G1 X21.272 Y130.592 E0.21116
G1 X21.272 Y131.129 E0.01697
```

G1 X25.462 Y135.319 E0.18716

G1 X24.925 Y135.319 E0.01697

G1 X21.272 Y131.666 E0.16316

G1 X21.272 Y132.204 E0.01697

G1 X24.387 Y135.319 E0.13916

G1 X23.850 Y135.319 E0.01697

G1 X21.272 Y132.741 E0.11516

G1 X21.272 Y133.278 E0.01697

G1 X23.313 Y135.319 E0.09116

G1 X22.776 Y135.319 E0.01697

G1 X21.272 Y133.815 E0.06716

G1 X21.272 Y134.353 E0.01697

G1 X22.238 Y135.319 E0.04316

G1 X21.701 Y135.319 E0.01697

G1 X21.103 Y134.720 E0.02673

M204 S1250

; stop printing object tpu print.STL id:17 copy 0

; printing object Petg print.STL id:16 copy 0

; stop printing object Petg print.STL id:16 copy 0

; printing object Petg print.STL id:12 copy 0

; stop printing object Petg print.STL id:12 copy 0

; printing object tpu print.STL id:7 copy 0

G1 E-2.24000 F2400.000

;WIPE_START

G1 F7200.000

G1 X21.701 Y135.319 E-0.26801

G1 X22.238 Y135.319 E-0.17013

G1 X21.272 Y134.353 E-0.43262

G1 X21.272 Y134.222 E-0.04124

```
;WIPE_END
G1 E-0.04800 F2400.000
G1 Z0.400 F9000.000
G1 X69.779 Y136.182
G1 Z0.200
G1 E3.20000 F1500.000
M204 S800
;TYPE:Perimeter
;WIDTH:0.42
G1 F1200.000
G1 X69.779 Y122.757 E0.42096
G1 X76.397 Y122.757 E0.20749
G1 X76.461 Y122.984 E0.00740
G1 X76.803 Y123.794 E0.02759
G1 X77.242 Y124.557 E0.02759
G1 X77.771 Y125.259 E0.02759
G1 X78.383 Y125.891 E0.02759
G1 X79.068 Y126.443 E0.02759
G1 X79.816 Y126.907 E0.02759
G1 X80.615 Y127.275 E0.02759
G1 X81.454 Y127.542 E0.02759
G1 X82.318 Y127.704 E0.02759
G1 X83.172 Y127.757 E0.02682
G1 X90.205 Y127.757 E0.22049
G1 X90.205 Y131.182 E0.10741
G1 X83.172 Y131.182 E0.22049
G1 X82.318 Y131.235 E0.02682
G1 X81.454 Y131.397 E0.02759
G1 X80.615 Y131.664 E0.02759
```

```
G1 X79.816 Y132.032 E0.02759
G1 X79.068 Y132.496 E0.02759
G1 X78.383 Y133.048 E0.02759
G1 X77.771 Y133.680 E0.02759
G1 X77.242 Y134.382 E0.02759
G1 X76.803 Y135.145 E0.02759
G1 X76.461 Y135.955 E0.02759
G1 X76.397 Y136.182 E0.00740
G1 X69.839 Y136.182 E0.20561
M204 S1250
G1 X69.402 Y136.559 F9000.000
M204 S800
;TYPE:External perimeter
G1 F1200.000
G1 X69.402 Y122.379 E0.44460
G1 X76.682 Y122.379 E0.22826
G1 X76.817 Y122.859 E0.01561
G1 X77.141 Y123.626 E0.02613
G1 X77.557 Y124.348 E0.02613
G1 X78.059 Y125.014 E0.02613
G1 X78.638 Y125.612 E0.02613
G1 X79.287 Y126.135 E0.02613
G1 X79.995 Y126.574 E0.02613
G1 X80.752 Y126.923 E0.02613
G1 X81.546 Y127.176 E0.02613
G1 X82.365 Y127.329 E0.02613
G1 X83.184 Y127.379 E0.02573
G1 X90.582 Y127.379 E0.23195
G1 X90.582 Y131.559 E0.13106
```

```
G1 X83.184 Y131.559 E0.23195
G1 X82.365 Y131.610 E0.02573
G1 X81.546 Y131.763 E0.02613
G1 X80.752 Y132.016 E0.02613
G1 X79.995 Y132.365 E0.02613
G1 X79.287 Y132.804 E0.02613
G1 X78.638 Y133.327 E0.02613
G1 X78.059 Y133.925 E0.02613
G1 X77.557 Y134.591 E0.02613
G1 X77.141 Y135.313 E0.02613
G1 X76.817 Y136.080 E0.02613
G1 X76.682 Y136.559 E0.01561
G1 X69.462 Y136.559 E0.22638
M204 S1250
G1 X69.594 Y136.209 F9000.000
G1 E-2.24000 F2400.000
;WIPE_START
G1 F7200.000
G1 X69.450 Y133.679 E-0.91200
;WIPE_END
G1 E-0.04800 F2400.000
G1 Z0.400 F9000.000
G1 X90.092 Y128.638
G1 Z0.200
G1 E3.20000 F1500.000
M204 S800
;TYPE:Solid infill
;WIDTH:0.422818
G1 F1200.000
```

G1 X89.493 Y128.039 E0.02674

G1 X88.956 Y128.039 E0.01697

G1 X89.922 Y129.005 E0.04316

G1 X89.922 Y129.543 E0.01697

G1 X88.418 Y128.039 E0.06716

G1 X87.881 Y128.039 E0.01697

G1 X89.922 Y130.080 E0.09116

G1 X89.922 Y130.617 E0.01697

G1 X87.344 Y128.039 E0.11516

G1 X86.807 Y128.039 E0.01697

G1 X89.667 Y130.900 E0.12778

G1 X89.130 Y130.900 E0.01697

G1 X86.269 Y128.039 E0.12778

G1 X85.732 Y128.039 E0.01697

G1 X88.592 Y130.900 E0.12778

G1 X88.055 Y130.900 E0.01697

G1 X85.195 Y128.039 E0.12778

G1 X84.658 Y128.039 E0.01697

G1 X87.518 Y130.900 E0.12778

G1 X86.981 Y130.900 E0.01697

G1 X84.120 Y128.039 E0.12778

G1 X83.583 Y128.039 E0.01697

G1 X86.443 Y130.900 E0.12778

G1 X85.906 Y130.900 E0.01697

G1 X83.038 Y128.032 E0.12812

G1 X82.466 Y127.996 E0.01812

G1 X85.369 Y130.900 E0.12970

G1 X84.832 Y130.900 E0.01697

G1 X81.833 Y127.901 E0.13397

```
G1 X81.384 Y127.817 E0.01440
G1 X81.131 Y127.736 E0.00841
G1 X84.294 Y130.900 E0.14133
G1 X83.757 Y130.900 E0.01697
G1 X79.951 Y127.094 E0.17002
M204 S1250
G1 E-2.24000 F2400.000
;WIPE_START
G1 F7200.000
G1 X81.988 Y129.130 E-0.91200
;WIPE_END
G1 E-0.04800 F2400.000
G1 Z0.400 F9000.000
G1 X76.701 Y123.843
G1 Z0.200
G1 E3.20000 F1500.000
M204 S800
G1 F1200.000
G1 X75.897 Y123.039 E0.03591
G1 X75.360 Y123.039 E0.01697
G1 X83.220 Y130.900 E0.35114
G1 X82.711 Y130.928 E0.01611
G1 X74.822 Y123.039 E0.35239
G1 X74.285 Y123.039 E0.01697
G1 X82.213 Y130.967 E0.35416
G1 X81.760 Y131.052 E0.01454
G1 X73.748 Y123.039 E0.35794
G1 X73.211 Y123.039 E0.01697
G1 X81.315 Y131.144 E0.36207
```

```
G1 X80.908 Y131.274 E0.01351
G1 X72.673 Y123.039 E0.36786
G1 X72.136 Y123.039 E0.01697
G1 X80.502 Y131.405 E0.37372
G1 X80.134 Y131.574 E0.01279
G1 X71.599 Y123.039 E0.38128
G1 X71.062 Y123.039 E0.01697
G1 X79.766 Y131.744 E0.38885
G1 X79.426 Y131.941 E0.01242
G1 X70.524 Y123.039 E0.39767
G1 X70.062 Y123.039 E0.01462
G1 X70.062 Y123.114 E0.00236
G1 X79.094 Y132.147 E0.40352
G1 X78.777 Y132.367 E0.01219
G1 X70.062 Y123.651 E0.38936
G1 X70.062 Y124.188 E0.01697
G1 X78.480 Y132.607 E0.37607
G1 X78.183 Y132.848 E0.01206
G1 X70.062 Y124.726 E0.36283
G1 X70.062 Y125.263 E0.01697
G1 X77.919 Y133.121 E0.35102
G1 X77.655 Y133.394 E0.01200
G1 X70.062 Y125.800 E0.33922
G1 X70.062 Y126.337 E0.01697
G1 X77.412 Y133.687 E0.32834
G1 X77.181 Y133.994 E0.01212
G1 X70.062 Y126.875 E0.31803
G1 X70.062 Y127.412 E0.01697
G1 X76.958 Y134.308 E0.30809
```

```
G1 X76.762 Y134.649 E0.01243
G1 X70.062 Y127.949 E0.29932
G1 X70.062 Y128.486 E0.01697
G1 X76.565 Y134.990 E0.29054
G1 X76.403 Y135.365 E0.01290
G1 X70.062 Y129.024 E0.28329
G1 X70.062 Y129.561 E0.01697
G1 X76.244 Y135.743 E0.27616
G1 X76.183 Y135.900 E0.00531
G1 X75.863 Y135.900 E0.01010
G1 X70.062 Y130.098 E0.25916
G1 X70.062 Y130.636 E0.01697
G1 X75.326 Y135.900 E0.23516
G1 X74.788 Y135.900 E0.01697
G1 X70.062 Y131.173 E0.21116
G1 X70.062 Y131.710 E0.01697
G1 X74.251 Y135.900 E0.18716
G1 X73.714 Y135.900 E0.01697
G1 X70.062 Y132.247 E0.16316
G1 X70.062 Y132.785 E0.01697
G1 X73.177 Y135.900 E0.13916
G1 X72.639 Y135.900 E0.01697
G1 X70.062 Y133.322 E0.11516
G1 X70.062 Y133.859 E0.01697
G1 X72.102 Y135.900 E0.09116
G1 X71.565 Y135.900 E0.01697
G1 X70.062 Y134.396 E0.06716
G1 X70.062 Y134.934 E0.01697
G1 X71.028 Y135.900 E0.04316
```

```
G1 X70.490 Y135.900 E0.01697
G1 X69.892 Y135.301 E0.02673
M204 S1250
; stop printing object tpu print.STL id:7 copy 0
; printing object tpu print.STL id:3 copy 0
G1 E-2.24000 F2400.000
;WIPE_START
G1 F7200.000
G1 X70.490 Y135.900 E-0.26801
G1 X71.028 Y135.900 E-0.17013
G1 X70.062 Y134.934 E-0.43262
G1 X70.062 Y134.803 E-0.04124
;WIPE_END
G1 E-0.04800 F2400.000
G1 Z0.400 F9000.000
G1 X69.892 Y117.528
G1 Z0.200
G1 E3.20000 F1500.000
M204 S800
;TYPE:Perimeter
;WIDTH:0.42
G1 F1200.000
G1 X69.794 Y117.528 E0.00307
G1 X69.794 Y104.103 E0.42096
G1 X76.412 Y104.103 E0.20749
G1 X76.476 Y104.330 E0.00740
G1 X76.818 Y105.140 E0.02759
G1 X77.257 Y105.903 E0.02759
G1 X77.787 Y106.605 E0.02759
```

```
G1 X78.399 Y107.237 E0.02759
G1 X79.084 Y107.789 E0.02759
G1 X79.831 Y108.253 E0.02759
G1 X80.630 Y108.621 E0.02759
G1 X81.469 Y108.888 E0.02759
G1 X82.334 Y109.050 E0.02759
G1 X83.187 Y109.103 E0.02682
G1 X90.220 Y109.103 E0.22049
G1 X90.220 Y112.528 E0.10741
G1 X83.187 Y112.528 E0.22049
G1 X82.334 Y112.581 E0.02682
G1 X81.469 Y112.743 E0.02759
G1 X80.630 Y113.010 E0.02759
G1 X79.831 Y113.378 E0.02759
G1 X79.084 Y113.842 E0.02759
G1 X78.399 Y114.394 E0.02759
G1 X77.787 Y115.026 E0.02759
G1 X77.257 Y115.728 E0.02759
G1 X76.818 Y116.491 E0.02759
G1 X76.476 Y117.301 E0.02759
G1 X76.412 Y117.528 E0.00740
G1 X69.952 Y117.528 E0.20254
M204 S1250
G1 X69.417 Y117.906 F9000.000
M204 S800
;TYPE:External perimeter
M73 P17 R57
G1 F1200.000
G1 X69.417 Y103.726 E0.44460
```

```
G1 X76.697 Y103.726 E0.22826
G1 X76.832 Y104.205 E0.01561
G1 X77.156 Y104.972 E0.02613
G1 X77.572 Y105.694 E0.02613
G1 X78.074 Y106.360 E0.02613
G1 X78.653 Y106.958 E0.02613
G1 X79.302 Y107.481 E0.02613
G1 X80.010 Y107.920 E0.02613
G1 X80.767 Y108.269 E0.02613
G1 X81.561 Y108.522 E0.02613
G1 X82.380 Y108.675 E0.02613
G1 X83.199 Y108.726 E0.02573
G1 X90.597 Y108.726 E0.23195
G1 X90.597 Y112.906 E0.13106
G1 X83.199 Y112.906 E0.23195
G1 X82.380 Y112.956 E0.02573
G1 X81.561 Y113.109 E0.02613
G1 X80.767 Y113.362 E0.02613
G1 X80.010 Y113.711 E0.02613
G1 X79.302 Y114.150 E0.02613
G1 X78.653 Y114.673 E0.02613
G1 X78.074 Y115.271 E0.02613
G1 X77.572 Y115.937 E0.02613
G1 X77.156 Y116.659 E0.02613
G1 X76.832 Y117.426 E0.02613
G1 X76.697 Y117.906 E0.01561
G1 X69.477 Y117.906 E0.22638
M204 S1250
G1 X69.609 Y117.555 F9000.000
```

```
G1 E-2.24000 F2400.000
;WIPE_START
G1 F7200.000
G1 X69.465 Y115.026 E-0.91200
;WIPE_END
G1 E-0.04800 F2400.000
G1 Z0.400 F9000.000
G1 X90.107 Y109.984
G1 Z0.200
G1 E3.20000 F1500.000
M204 S800
;TYPE:Solid infill
;WIDTH:0.422818
G1 F1200.000
G1 X89.508 Y109.385 E0.02674
G1 X88.971 Y109.385 E0.01697
G1 X89.937 Y110.352 E0.04316
G1 X89.937 Y110.889 E0.01697
G1 X88.434 Y109.385 E0.06716
G1 X87.896 Y109.385 E0.01697
G1 X89.937 Y111.426 E0.09116
G1 X89.937 Y111.963 E0.01697
G1 X87.359 Y109.385 E0.11516
G1 X86.822 Y109.385 E0.01697
G1 X89.682 Y112.246 E0.12778
G1 X89.145 Y112.246 E0.01697
G1 X86.285 Y109.385 E0.12778
G1 X85.747 Y109.385 E0.01697
G1 X88.608 Y112.246 E0.12778
```

```
G1 X88.070 Y112.246 E0.01697
G1 X85.210 Y109.385 E0.12778
G1 X84.673 Y109.385 E0.01697
G1 X87.533 Y112.246 E0.12778
G1 X86.996 Y112.246 E0.01697
G1 X84.136 Y109.385 E0.12778
G1 X83.598 Y109.385 E0.01697
G1 X86.459 Y112.246 E0.12778
G1 X85.921 Y112.246 E0.01697
G1 X83.053 Y109.378 E0.12812
G1 X82.481 Y109.342 E0.01812
G1 X85.384 Y112.246 E0.12970
G1 X84.847 Y112.246 E0.01697
G1 X81.848 Y109.247 E0.13397
G1 X81.400 Y109.163 E0.01440
G1 X81.146 Y109.082 E0.00841
G1 X84.309 Y112.246 E0.14133
G1 X83.772 Y112.246 E0.01697
G1 X79.966 Y108.440 E0.17002
M204 S1250
G1 E-2.24000 F2400.000
;WIPE_START
G1 F7200.000
G1 X82.003 Y110.476 E-0.91200
;WIPE_END
G1 E-0.04800 F2400.000
G1 Z0.400 F9000.000
G1 X76.716 Y105.189
G1 Z0.200
```

```
G1 E3.20000 F1500.000
M204 S800
G1 F1200.000
G1 X75.912 Y104.385 E0.03591
G1 X75.375 Y104.385 E0.01697
G1 X83.235 Y112.246 E0.35114
M73 P17 R56
G1 X82.726 Y112.274 E0.01611
G1 X74.837 Y104.385 E0.35239
G1 X74.300 Y104.385 E0.01697
G1 X82.228 Y112.313 E0.35416
G1 X81.775 Y112.398 E0.01454
G1 X73.763 Y104.385 E0.35794
G1 X73.226 Y104.385 E0.01697
G1 X81.331 Y112.490 E0.36207
G1 X80.923 Y112.620 E0.01351
G1 X72.688 Y104.385 E0.36786
G1 X72.151 Y104.385 E0.01697
G1 X80.517 Y112.751 E0.37372
G1 X80.149 Y112.920 E0.01279
G1 X71.614 Y104.385 E0.38128
G1 X71.077 Y104.385 E0.01697
G1 X79.781 Y113.090 E0.38885
G1 X79.441 Y113.287 E0.01242
G1 X70.539 Y104.385 E0.39767
G1 X70.077 Y104.385 E0.01462
G1 X70.077 Y104.460 E0.00236
G1 X79.110 Y113.493 E0.40352
G1 X78.792 Y113.713 E0.01219
```

```
G1 X70.077 Y104.997 E0.38936
G1 X70.077 Y105.534 E0.01697
G1 X78.495 Y113.953 E0.37607
G1 X78.199 Y114.194 E0.01206
G1 X70.077 Y106.072 E0.36283
G1 X70.077 Y106.609 E0.01697
G1 X77.934 Y114.467 E0.35102
G1 X77.670 Y114.740 E0.01200
G1 X70.077 Y107.146 E0.33922
G1 X70.077 Y107.684 E0.01697
G1 X77.427 Y115.033 E0.32834
G1 X77.196 Y115.340 E0.01212
G1 X70.077 Y108.221 E0.31803
G1 X70.077 Y108.758 E0.01697
G1 X76.973 Y115.655 E0.30809
G1 X76.777 Y115.995 E0.01243
G1 X70.077 Y109.295 E0.29932
G1 X70.077 Y109.833 E0.01697
G1 X76.581 Y116.336 E0.29054
G1 X76.418 Y116.711 E0.01290
G1 X70.077 Y110.370 E0.28329
G1 X70.077 Y110.907 E0.01697
G1 X76.259 Y117.089 E0.27616
G1 X76.198 Y117.246 E0.00531
G1 X75.878 Y117.246 E0.01010
G1 X70.077 Y111.444 E0.25916
G1 X70.077 Y111.982 E0.01697
G1 X75.341 Y117.246 E0.23516
G1 X74.804 Y117.246 E0.01697
```

G1 X70.077 Y112.519 E0.21116

G1 X70.077 Y113.056 E0.01697

G1 X74.266 Y117.246 E0.18716

G1 X73.729 Y117.246 E0.01697

G1 X70.077 Y113.593 E0.16316

G1 X70.077 Y114.131 E0.01697

G1 X73.192 Y117.246 E0.13916

G1 X72.655 Y117.246 E0.01697

G1 X70.077 Y114.668 E0.11516

G1 X70.077 Y115.205 E0.01697

G1 X72.117 Y117.246 E0.09116

G1 X71.580 Y117.246 E0.01697

G1 X70.077 Y115.742 E0.06716

G1 X70.077 Y116.280 E0.01697

G1 X71.043 Y117.246 E0.04316

G1 X70.505 Y117.246 E0.01697

G1 X69.907 Y116.647 E0.02673

M204 S1250

; stop printing object tpu print.STL id:3 copy 0

; printing object Petg print.STL id:2 copy 0

; stop printing object Petg print.STL id:2 copy 0

; printing object Petg print.STL id:6 copy 0

; stop printing object Petg print.STL id:6 copy 0

; printing object tpu print.STL id:27 copy 0

G1 E-2.24000 F2400.000

;WIPE_START

G1 F7200.000

G1 X70.505 Y117.246 E-0.26801

G1 X71.043 Y117.246 E-0.17013

```
G1 X70.077 Y116.280 E-0.43262
G1 X70.077 Y116.149 E-0.04124
;WIPE_END
G1 E-0.04800 F2400.000
G1 Z0.400 F9000.000
G1 X120.429 Y122.932
G1 Z0.200
G1 E3.20000 F1500.000
M204 S800
;TYPE:Perimeter
;WIDTH:0.42
G1 F1200.000
G1 X127.047 Y122.932 E0.20749
G1 X127.111 Y123.159 E0.00740
G1 X127.453 Y123.969 E0.02759
G1 X127.892 Y124.732 E0.02759
G1 X128.422 Y125.434 E0.02759
G1 X129.034 Y126.066 E0.02759
G1 X129.719 Y126.618 E0.02759
G1 X130.466 Y127.082 E0.02759
G1 X131.266 Y127.450 E0.02759
G1 X132.104 Y127.717 E0.02759
G1 X132.969 Y127.879 E0.02759
G1 X133.823 Y127.932 E0.02682
G1 X140.855 Y127.932 E0.22049
G1 X140.855 Y131.357 E0.10741
G1 X133.823 Y131.357 E0.22049
G1 X132.969 Y131.410 E0.02682
G1 X132.104 Y131.572 E0.02759
```

```
G1 X131.266 Y131.839 E0.02759
G1 X130.466 Y132.207 E0.02759
G1 X129.719 Y132.671 E0.02759
G1 X129.034 Y133.223 E0.02759
G1 X128.422 Y133.855 E0.02759
G1 X127.892 Y134.557 E0.02759
G1 X127.453 Y135.320 E0.02759
G1 X127.111 Y136.130 E0.02759
G1 X127.047 Y136.357 E0.00740
G1 X120.429 Y136.357 E0.20749
G1 X120.429 Y122.992 E0.41908
M204 S1250
G1 X120.052 Y122.555 F9000.000
M204 S800
;TYPE:External perimeter
G1 F1200.000
G1 X127.332 Y122.555 E0.22826
G1 X127.468 Y123.034 E0.01561
G1 X127.791 Y123.801 E0.02613
G1 X128.207 Y124.523 E0.02613
G1 X128.709 Y125.189 E0.02613
G1 X129.288 Y125.787 E0.02613
G1 X129.937 Y126.310 E0.02613
G1 X130.645 Y126.749 E0.02613
G1 X131.402 Y127.098 E0.02613
G1 X132.196 Y127.351 E0.02613
G1 X133.015 Y127.504 E0.02613
G1 X133.834 Y127.555 E0.02573
G1 X141.232 Y127.555 E0.23195
```

```
G1 X141.232 Y131.735 E0.13106
G1 X133.834 Y131.735 E0.23195
G1 X133.015 Y131.785 E0.02573
G1 X132.196 Y131.938 E0.02613
G1 X131.402 Y132.191 E0.02613
G1 X130.645 Y132.540 E0.02613
G1 X129.937 Y132.979 E0.02613
G1 X129.288 Y133.502 E0.02613
G1 X128.709 Y134.100 E0.02613
G1 X128.207 Y134.766 E0.02613
G1 X127.791 Y135.488 E0.02613
G1 X127.468 Y136.255 E0.02613
G1 X127.332 Y136.735 E0.01561
G1 X120.052 Y136.735 E0.22826
G1 X120.052 Y122.615 E0.44272
M204 S1250
G1 X120.426 Y122.698 F9000.000
G1 E-2.24000 F2400.000
;WIPE_START
G1 F7200.000
G1 X122.932 Y122.591 E-0.91200
;WIPE_END
G1 E-0.04800 F2400.000
G1 Z0.400 F9000.000
G1 X140.742 Y128.813
G1 Z0.200
G1 E3.20000 F1500.000
M204 S800
;TYPE:Solid infill
```

```
;WIDTH:0.422818
G1 F1200.000
G1 X140.143 Y128.214 E0.02674
G1 X139.606 Y128.214 E0.01697
G1 X140.572 Y129.181 E0.04316
G1 X140.572 Y129.718 E0.01697
G1 X139.069 Y128.214 E0.06716
G1 X138.531 Y128.214 E0.01697
G1 X140.572 Y130.255 E0.09116
G1 X140.572 Y130.792 E0.01697
G1 X137.994 Y128.214 E0.11516
G1 X137.457 Y128.214 E0.01697
G1 X140.317 Y131.075 E0.12778
G1 X139.780 Y131.075 E0.01697
G1 X136.920 Y128.214 E0.12778
G1 X136.382 Y128.214 E0.01697
G1 X139.243 Y131.075 E0.12778
G1 X138.705 Y131.075 E0.01697
G1 X135.845 Y128.214 E0.12778
G1 X135.308 Y128.214 E0.01697
G1 X138.168 Y131.075 E0.12778
G1 X137.631 Y131.075 E0.01697
G1 X134.771 Y128.214 E0.12778
G1 X134.233 Y128.214 E0.01697
G1 X137.094 Y131.075 E0.12778
G1 X136.556 Y131.075 E0.01697
G1 X133.688 Y128.207 E0.12812
G1 X133.116 Y128.171 E0.01812
G1 X136.019 Y131.075 E0.12970
```

G1 X135.482 Y131.075 E0.01697

G1 X132.483 Y128.076 E0.13397

G1 X132.035 Y127.992 E0.01440

G1 X131.781 Y127.911 E0.00841

G1 X134.945 Y131.075 E0.14133

G1 X134.407 Y131.075 E0.01697

G1 X130.601 Y127.269 E0.17002

M204 S1250

G1 E-2.24000 F2400.000

;WIPE_START

G1 F7200.000

G1 X132.638 Y129.305 E-0.91200

;WIPE_END

G1 E-0.04800 F2400.000

G1 Z0.400 F9000.000

G1 X127.351 Y124.018

G1 Z0.200

G1 E3.20000 F1500.000

M204 S800

G1 F1200.000

G1 X126.547 Y123.214 E0.03591

G1 X126.010 Y123.214 E0.01697

G1 X133.870 Y131.075 E0.35114

G1 X133.361 Y131.103 E0.01611

G1 X125.473 Y123.214 E0.35239

G1 X124.935 Y123.214 E0.01697

G1 X132.863 Y131.142 E0.35416

G1 X132.411 Y131.227 E0.01454

G1 X124.398 Y123.214 E0.35794

G1 X123.861 Y123.214 E0.01697

G1 X131.966 Y131.319 E0.36207

G1 X131.558 Y131.449 E0.01351

G1 X123.324 Y123.214 E0.36786

G1 X122.786 Y123.214 E0.01697

G1 X131.152 Y131.580 E0.37372

M73 P18 R56

G1 X130.784 Y131.749 E0.01279

G1 X122.249 Y123.214 E0.38128

G1 X121.712 Y123.214 E0.01697

G1 X130.416 Y131.919 E0.38885

G1 X130.076 Y132.116 E0.01242

G1 X121.175 Y123.214 E0.39767

G1 X120.712 Y123.214 E0.01462

G1 X120.712 Y123.289 E0.00236

G1 X129.745 Y132.322 E0.40352

G1 X129.428 Y132.542 E0.01219

G1 X120.712 Y123.826 E0.38936

G1 X120.712 Y124.364 E0.01697

G1 X129.130 Y132.782 E0.37607

G1 X128.834 Y133.023 E0.01206

G1 X120.712 Y124.901 E0.36283

G1 X120.712 Y125.438 E0.01697

G1 X128.569 Y133.296 E0.35102

G1 X128.305 Y133.569 E0.01200

G1 X120.712 Y125.975 E0.33922

G1 X120.712 Y126.513 E0.01697

G1 X128.062 Y133.862 E0.32834

G1 X127.831 Y134.169 E0.01212

```
G1 X120.712 Y127.050 E0.31803
G1 X120.712 Y127.587 E0.01697
G1 X127.608 Y134.484 E0.30809
G1 X127.412 Y134.824 E0.01243
G1 X120.712 Y128.124 E0.29932
G1 X120.712 Y128.662 E0.01697
G1 X127.216 Y135.165 E0.29054
G1 X127.053 Y135.540 E0.01290
G1 X120.712 Y129.199 E0.28329
G1 X120.712 Y129.736 E0.01697
G1 X126.894 Y135.918 E0.27616
G1 X126.833 Y136.075 E0.00531
G1 X126.513 Y136.075 E0.01010
G1 X120.712 Y130.273 E0.25916
G1 X120.712 Y130.811 E0.01697
G1 X125.976 Y136.075 E0.23516
G1 X125.439 Y136.075 E0.01697
G1 X120.712 Y131.348 E0.21116
G1 X120.712 Y131.885 E0.01697
G1 X124.901 Y136.075 E0.18716
G1 X124.364 Y136.075 E0.01697
G1 X120.712 Y132.422 E0.16316
G1 X120.712 Y132.960 E0.01697
G1 X123.827 Y136.075 E0.13916
G1 X123.290 Y136.075 E0.01697
G1 X120.712 Y133.497 E0.11516
G1 X120.712 Y134.034 E0.01697
G1 X122.752 Y136.075 E0.09116
G1 X122.215 Y136.075 E0.01697
```

```
G1 X120.712 Y134.571 E0.06716
G1 X120.712 Y135.109 E0.01697
G1 X121.678 Y136.075 E0.04316
G1 X121.141 Y136.075 E0.01697
G1 X120.542 Y135.476 E0.02673
M204 S1250
; stop printing object tpu print.STL id:27 copy 0
; printing object tpu print.STL id:23 copy 0
G1 E-2.24000 F2400.000
;WIPE_START
G1 F7200.000
G1 X121.141 Y136.075 E-0.26801
G1 X121.678 Y136.075 E-0.17013
G1 X120.712 Y135.109 E-0.43262
G1 X120.712 Y134.978 E-0.04124
;WIPE_END
G1 E-0.04800 F2400.000
G1 Z0.400 F9000.000
G1 X120.542 Y117.704
G1 Z0.200
G1 E3.20000 F1500.000
M204 S800
;TYPE:Perimeter
;WIDTH:0.42
G1 F1200.000
G1 X120.444 Y117.704 E0.00307
G1 X120.444 Y104.278 E0.42096
G1 X127.062 Y104.278 E0.20749
G1 X127.126 Y104.505 E0.00740
```

```
G1 X127.468 Y105.315 E0.02759
G1 X127.907 Y106.078 E0.02759
G1 X128.437 Y106.780 E0.02759
G1 X129.049 Y107.412 E0.02759
G1 X129.734 Y107.965 E0.02759
G1 X130.482 Y108.428 E0.02759
G1 X131.281 Y108.796 E0.02759
G1 X132.119 Y109.063 E0.02759
G1 X132.984 Y109.225 E0.02759
G1 X133.838 Y109.278 E0.02682
G1 X140.870 Y109.278 E0.22049
G1 X140.870 Y112.704 E0.10741
G1 X133.838 Y112.704 E0.22049
G1 X132.984 Y112.756 E0.02682
G1 X132.119 Y112.918 E0.02759
G1 X131.281 Y113.185 E0.02759
G1 X130.482 Y113.553 E0.02759
G1 X129.734 Y114.017 E0.02759
G1 X129.049 Y114.569 E0.02759
G1 X128.437 Y115.201 E0.02759
G1 X127.907 Y115.903 E0.02759
G1 X127.468 Y116.666 E0.02759
G1 X127.126 Y117.476 E0.02759
G1 X127.062 Y117.704 E0.00740
G1 X120.602 Y117.704 E0.20254
M204 S1250
G1 X120.067 Y118.081 F9000.000
M204 S800
;TYPE:External perimeter
```

```
G1 F1200.000
G1 X120.067 Y103.901 E0.44460
G1 X127.347 Y103.901 E0.22826
G1 X127.483 Y104.380 E0.01561
G1 X127.807 Y105.147 E0.02613
G1 X128.222 Y105.869 E0.02613
G1 X128.724 Y106.535 E0.02613
G1 X129.303 Y107.133 E0.02613
G1 X129.952 Y107.656 E0.02613
G1 X130.660 Y108.095 E0.02613
G1 X131.417 Y108.444 E0.02613
G1 X132.211 Y108.697 E0.02613
G1 X133.030 Y108.850 E0.02613
G1 X133.849 Y108.901 E0.02573
G1 X141.247 Y108.901 E0.23195
G1 X141.247 Y113.081 E0.13106
G1 X133.849 Y113.081 E0.23195
G1 X133.030 Y113.131 E0.02573
G1 X132.211 Y113.284 E0.02613
G1 X131.417 Y113.537 E0.02613
G1 X130.660 Y113.886 E0.02613
G1 X129.952 Y114.325 E0.02613
G1 X129.303 Y114.848 E0.02613
G1 X128.724 Y115.446 E0.02613
G1 X128.222 Y116.112 E0.02613
G1 X127.807 Y116.834 E0.02613
G1 X127.483 Y117.602 E0.02613
G1 X127.347 Y118.081 E0.01561
G1 X120.127 Y118.081 E0.22638
```

```
M204 S1250
G1 X120.260 Y117.730 F9000.000
G1 E-2.24000 F2400.000
;WIPE_START
G1 F7200.000
G1 X120.115 Y115.201 E-0.91200
;WIPE_END
G1 E-0.04800 F2400.000
G1 Z0.400 F9000.000
G1 X140.757 Y110.159
G1 Z0.200
G1 E3.20000 F1500.000
M204 S800
;TYPE:Solid infill
;WIDTH:0.422818
G1 F1200.000
G1 X140.158 Y109.560 E0.02674
G1 X139.621 Y109.560 E0.01697
G1 X140.587 Y110.527 E0.04316
G1 X140.587 Y111.064 E0.01697
G1 X139.084 Y109.560 E0.06716
G1 X138.547 Y109.560 E0.01697
G1 X140.587 Y111.601 E0.09116
G1 X140.587 Y112.138 E0.01697
G1 X138.009 Y109.560 E0.11516
G1 X137.472 Y109.560 E0.01697
G1 X140.332 Y112.421 E0.12778
G1 X139.795 Y112.421 E0.01697
G1 X136.935 Y109.560 E0.12778
```

```
G1 X136.398 Y109.560 E0.01697
G1 X139.258 Y112.421 E0.12778
G1 X138.721 Y112.421 E0.01697
G1 X135.860 Y109.560 E0.12778
G1 X135.323 Y109.560 E0.01697
G1 X138.183 Y112.421 E0.12778
G1 X137.646 Y112.421 E0.01697
G1 X134.786 Y109.560 E0.12778
G1 X134.249 Y109.560 E0.01697
G1 X137.109 Y112.421 E0.12778
G1 X136.571 Y112.421 E0.01697
G1 X133.704 Y109.553 E0.12812
G1 X133.131 Y109.517 E0.01812
G1 X136.034 Y112.421 E0.12970
G1 X135.497 Y112.421 E0.01697
G1 X132.498 Y109.422 E0.13397
G1 X132.050 Y109.338 E0.01440
G1 X131.796 Y109.257 E0.00841
G1 X134.960 Y112.421 E0.14133
G1 X134.422 Y112.421 E0.01697
G1 X130.617 Y108.615 E0.17002
M204 S1250
G1 E-2.24000 F2400.000
;WIPE_START
G1 F7200.000
G1 X132.653 Y110.651 E-0.91200
;WIPE_END
G1 E-0.04800 F2400.000
G1 Z0.400 F9000.000
```

```
G1 X127.366 Y105.364
G1 Z0.200
G1 E3.20000 F1500.000
M204 S800
G1 F1200.000
G1 X126.562 Y104.560 E0.03591
G1 X126.025 Y104.560 E0.01697
G1 X133.885 Y112.421 E0.35114
G1 X133.376 Y112.449 E0.01611
G1 X125.488 Y104.560 E0.35239
G1 X124.950 Y104.560 E0.01697
G1 X132.878 Y112.488 E0.35416
G1 X132.426 Y112.573 E0.01454
G1 X124.413 Y104.560 E0.35794
G1 X123.876 Y104.560 E0.01697
G1 X131.981 Y112.665 E0.36207
M73 P18 R55
G1 X131.573 Y112.795 E0.01351
G1 X123.339 Y104.560 E0.36786
G1 X122.801 Y104.560 E0.01697
G1 X131.167 Y112.926 E0.37372
G1 X130.799 Y113.095 E0.01279
G1 X122.264 Y104.560 E0.38128
G1 X121.727 Y104.560 E0.01697
G1 X130.431 Y113.265 E0.38885
G1 X130.091 Y113.462 E0.01242
G1 X121.190 Y104.560 E0.39767
G1 X120.727 Y104.560 E0.01462
G1 X120.727 Y104.635 E0.00236
```

```
G1 X129.760 Y113.668 E0.40352
G1 X129.443 Y113.888 E0.01219
G1 X120.727 Y105.172 E0.38936
G1 X120.727 Y105.710 E0.01697
G1 X129.145 Y114.128 E0.37607
G1 X128.849 Y114.369 E0.01206
G1 X120.727 Y106.247 E0.36283
G1 X120.727 Y106.784 E0.01697
G1 X128.585 Y114.642 E0.35102
G1 X128.320 Y114.915 E0.01200
G1 X120.727 Y107.321 E0.33922
G1 X120.727 Y107.859 E0.01697
G1 X128.077 Y115.209 E0.32834
G1 X127.846 Y115.515 E0.01212
G1 X120.727 Y108.396 E0.31803
G1 X120.727 Y108.933 E0.01697
G1 X127.623 Y115.830 E0.30809
G1 X127.427 Y116.170 E0.01243
G1 X120.727 Y109.470 E0.29932
G1 X120.727 Y110.008 E0.01697
G1 X127.231 Y116.511 E0.29054
G1 X127.068 Y116.886 E0.01290
G1 X120.727 Y110.545 E0.28329
G1 X120.727 Y111.082 E0.01697
G1 X126.909 Y117.264 E0.27616
G1 X126.848 Y117.421 E0.00531
G1 X126.528 Y117.421 E0.01010
G1 X120.727 Y111.619 E0.25916
G1 X120.727 Y112.157 E0.01697
```

```
G1 X125.991 Y117.421 E0.23516
G1 X125.454 Y117.421 E0.01697
G1 X120.727 Y112.694 E0.21116
G1 X120.727 Y113.231 E0.01697
G1 X124.917 Y117.421 E0.18716
G1 X124.379 Y117.421 E0.01697
G1 X120.727 Y113.768 E0.16316
G1 X120.727 Y114.306 E0.01697
G1 X123.842 Y117.421 E0.13916
G1 X123.305 Y117.421 E0.01697
G1 X120.727 Y114.843 E0.11516
G1 X120.727 Y115.380 E0.01697
G1 X122.768 Y117.421 E0.09116
G1 X122.230 Y117.421 E0.01697
G1 X120.727 Y115.917 E0.06716
G1 X120.727 Y116.455 E0.01697
G1 X121.693 Y117.421 E0.04316
G1 X121.156 Y117.421 E0.01697
G1 X120.557 Y116.822 E0.02673
M204 S1250
; stop printing object tpu print.STL id:23 copy 0
; printing object tpu print.STL id:21 copy 0
G1 E-2.24000 F2400.000
;WIPE_START
M73 P19 R55
G1 F7200.000
G1 X121.156 Y117.421 E-0.26801
G1 X121.693 Y117.421 E-0.17013
G1 X120.727 Y116.455 E-0.43262
```

```
G1 X120.727 Y116.324 E-0.04124
;WIPE_END
G1 E-0.04800 F2400.000
G1 Z0.400 F9000.000
G1 X120.557 Y96.888
G1 Z0.200
G1 E3.20000 F1500.000
M204 S800
;TYPE:Perimeter
;WIDTH:0.42
G1 F1200.000
G1 X120.437 Y96.888 E0.00376
G1 X120.437 Y83.462 E0.42096
G1 X127.055 Y83.462 E0.20749
G1 X127.119 Y83.689 E0.00740
G1 X127.461 Y84.500 E0.02759
G1 X127.900 Y85.262 E0.02759
G1 X128.430 Y85.965 E0.02759
G1 X129.042 Y86.597 E0.02759
G1 X129.727 Y87.149 E0.02759
G1 X130.475 Y87.613 E0.02759
G1 X131.274 Y87.981 E0.02759
G1 X132.112 Y88.248 E0.02759
G1 X132.977 Y88.409 E0.02759
G1 X133.831 Y88.462 E0.02682
G1 X140.863 Y88.462 E0.22049
G1 X140.863 Y91.888 E0.10741
G1 X133.831 Y91.888 E0.22049
G1 X132.977 Y91.941 E0.02682
```

```
G1 X132.112 Y92.103 E0.02759
G1 X131.274 Y92.370 E0.02759
G1 X130.475 Y92.738 E0.02759
G1 X129.727 Y93.201 E0.02759
G1 X129.042 Y93.753 E0.02759
G1 X128.430 Y94.385 E0.02759
G1 X127.900 Y95.088 E0.02759
G1 X127.461 Y95.850 E0.02759
G1 X127.119 Y96.661 E0.02759
G1 X127.055 Y96.888 E0.00740
G1 X120.617 Y96.888 E0.20185
M204 S1250
G1 X120.060 Y97.265 F9000.000
M204 S800
;TYPE:External perimeter
G1 F1200.000
G1 X120.060 Y83.085 E0.44460
G1 X127.340 Y83.085 E0.22826
G1 X127.476 Y83.564 E0.01561
G1 X127.800 Y84.332 E0.02613
G1 X128.216 Y85.054 E0.02613
G1 X128.717 Y85.719 E0.02613
G1 X129.297 Y86.318 E0.02613
G1 X129.945 Y86.841 E0.02613
G1 X130.654 Y87.280 E0.02613
G1 X131.410 Y87.628 E0.02613
G1 X132.204 Y87.881 E0.02613
G1 X133.023 Y88.035 E0.02613
G1 X133.842 Y88.085 E0.02573
```

G1 X141.240 Y88.085 E0.23195

G1 X141.240 Y92.265 E0.13106

G1 X133.842 Y92.265 E0.23195

G1 X133.023 Y92.316 E0.02573

G1 X132.204 Y92.469 E0.02613

G1 X131.410 Y92.722 E0.02613

G1 X130.654 Y93.070 E0.02613

G1 X129.945 Y93.509 E0.02613

G1 X129.297 Y94.032 E0.02613

G1 X128.717 Y94.631 E0.02613

G1 X128.216 Y95.296 E0.02613

G1 X127.800 Y96.018 E0.02613

G1 X127.476 Y96.786 E0.02613

G1 X127.340 Y97.265 E0.01561

G1 X120.120 Y97.265 E0.22638

M204 S1250

G1 X120.253 Y96.914 F9000.000

G1 E-2.24000 F2400.000

;WIPE_START

G1 F7200.000

G1 X120.108 Y94.385 E-0.91200

;WIPE_END

G1 E-0.04800 F2400.000

G1 Z0.400 F9000.000

G1 X140.750 Y89.344

G1 Z0.200

G1 E3.20000 F1500.000

M204 S800

;TYPE:Solid infill

```
;WIDTH:0.422818
G1 F1200.000
G1 X140.151 Y88.745 E0.02674
G1 X139.614 Y88.745 E0.01697
G1 X140.580 Y89.711 E0.04316
G1 X140.580 Y90.248 E0.01697
G1 X139.077 Y88.745 E0.06716
G1 X138.540 Y88.745 E0.01697
G1 X140.580 Y90.786 E0.09116
G1 X140.580 Y91.323 E0.01697
G1 X138.002 Y88.745 E0.11516
G1 X137.465 Y88.745 E0.01697
G1 X140.325 Y91.605 E0.12778
G1 X139.788 Y91.605 E0.01697
G1 X136.928 Y88.745 E0.12778
G1 X136.391 Y88.745 E0.01697
G1 X139.251 Y91.605 E0.12778
G1 X138.714 Y91.605 E0.01697
G1 X135.853 Y88.745 E0.12778
G1 X135.316 Y88.745 E0.01697
G1 X138.176 Y91.605 E0.12778
G1 X137.639 Y91.605 E0.01697
G1 X134.779 Y88.745 E0.12778
G1 X134.242 Y88.745 E0.01697
G1 X137.102 Y91.605 E0.12778
G1 X136.565 Y91.605 E0.01697
G1 X133.697 Y88.737 E0.12812
G1 X133.124 Y88.702 E0.01812
G1 X136.027 Y91.605 E0.12970
```

```
G1 X135.490 Y91.605 E0.01697
G1 X132.491 Y88.606 E0.13397
G1 X132.043 Y88.522 E0.01440
G1 X131.789 Y88.442 E0.00841
G1 X134.953 Y91.605 E0.14133
G1 X134.416 Y91.605 E0.01697
G1 X130.610 Y87.799 E0.17002
M204 S1250
G1 E-2.24000 F2400.000
;WIPE_START
G1 F7200.000
G1 X132.646 Y89.836 E-0.91200
;WIPE_END
G1 E-0.04800 F2400.000
G1 Z0.400 F9000.000
G1 X127.359 Y84.549
G1 Z0.200
G1 E3.20000 F1500.000
M204 S800
G1 F1200.000
G1 X126.555 Y83.745 E0.03591
G1 X126.018 Y83.745 E0.01697
G1 X133.878 Y91.605 E0.35114
G1 X133.369 Y91.633 E0.01611
G1 X125.481 Y83.745 E0.35239
G1 X124.944 Y83.745 E0.01697
G1 X132.871 Y91.673 E0.35416
G1 X132.419 Y91.757 E0.01454
G1 X124.406 Y83.745 E0.35794
```

G1 X123.869 Y83.745 E0.01697

G1 X131.974 Y91.850 E0.36207

G1 X131.566 Y91.980 E0.01351

G1 X123.332 Y83.745 E0.36786

G1 X122.795 Y83.745 E0.01697

G1 X131.160 Y92.111 E0.37372

G1 X130.792 Y92.280 E0.01279

G1 X122.257 Y83.745 E0.38128

G1 X121.720 Y83.745 E0.01697

G1 X130.424 Y92.449 E0.38885

G1 X130.085 Y92.647 E0.01242

G1 X121.183 Y83.745 E0.39767

G1 X120.720 Y83.745 E0.01462

G1 X120.720 Y83.820 E0.00236

G1 X129.753 Y92.852 E0.40352

G1 X129.436 Y93.073 E0.01219

G1 X120.720 Y84.357 E0.38936

G1 X120.720 Y84.894 E0.01697

G1 X129.138 Y93.312 E0.37607

G1 X128.842 Y93.553 E0.01206

G1 X120.720 Y85.431 E0.36283

G1 X120.720 Y85.969 E0.01697

G1 X128.578 Y93.826 E0.35102

G1 X128.313 Y94.099 E0.01200

G1 X120.720 Y86.506 E0.33922

G1 X120.720 Y87.043 E0.01697

G1 X128.070 Y94.393 E0.32834

G1 X127.839 Y94.699 E0.01212

G1 X120.720 Y87.580 E0.31803

G1 X120.720 Y88.118 E0.01697

G1 X127.617 Y95.014 E0.30809

G1 X127.420 Y95.355 E0.01243

G1 X120.720 Y88.655 E0.29932

G1 X120.720 Y89.192 E0.01697

G1 X127.224 Y95.696 E0.29054

G1 X127.061 Y96.071 E0.01290

G1 X120.720 Y89.729 E0.28329

G1 X120.720 Y90.267 E0.01697

G1 X126.902 Y96.449 E0.27616

G1 X126.841 Y96.605 E0.00531

G1 X126.521 Y96.605 E0.01010

G1 X120.720 Y90.804 E0.25916

G1 X120.720 Y91.341 E0.01697

G1 X125.984 Y96.605 E0.23516

G1 X125.447 Y96.605 E0.01697

G1 X120.720 Y91.878 E0.21116

G1 X120.720 Y92.416 E0.01697

G1 X124.910 Y96.605 E0.18716

G1 X124.372 Y96.605 E0.01697

G1 X120.720 Y92.953 E0.16316

G1 X120.720 Y93.490 E0.01697

G1 X123.835 Y96.605 E0.13916

G1 X123.298 Y96.605 E0.01697

G1 X120.720 Y94.027 E0.11516

G1 X120.720 Y94.565 E0.01697

G1 X122.761 Y96.605 E0.09116

G1 X122.223 Y96.605 E0.01697

G1 X120.720 Y95.102 E0.06716

```
G1 X120.720 Y95.639 E0.01697
G1 X121.686 Y96.605 E0.04316
G1 X121.149 Y96.605 E0.01697
G1 X120.550 Y96.007 E0.02673
M204 S1250
; stop printing object tpu print.STL id:21 copy 0
; printing object Petg print.STL id:20 copy 0
; stop printing object Petg print.STL id:20 copy 0
; printing object Petg print.STL id:22 copy 0
; stop printing object Petg print.STL id:22 copy 0
; printing object Petg print.STL id:26 copy 0
; stop printing object Petg print.STL id:26 copy 0
; printing object Petg print.STL id:0 copy 0
; stop printing object Petg print.STL id:0 copy 0
; printing object tpu print.STL id:1 copy 0
G1 E-2.24000 F2400.000
;WIPE_START
G1 F7200.000
G1 X121.149 Y96.605 E-0.26801
G1 X121.686 Y96.605 E-0.17013
G1 X120.720 Y95.639 E-0.43262
G1 X120.720 Y95.509 E-0.04124
;WIPE_END
G1 E-0.04800 F2400.000
G1 Z0.400 F9000.000
G1 X90.213 Y91.713
G1 Z0.200
G1 E3.20000 F1500.000
M204 S800
```

```
;TYPE:Perimeter
;WIDTH:0.42
G1 F1200.000
G1 X83.181 Y91.713 E0.22049
G1 X82.327 Y91.766 E0.02682
G1 X81.462 Y91.927 E0.02759
G1 X80.624 Y92.194 E0.02759
G1 X79.825 Y92.563 E0.02759
G1 X79.077 Y93.026 E0.02759
G1 X78.392 Y93.578 E0.02759
G1 X77.780 Y94.210 E0.02759
G1 X77.250 Y94.913 E0.02759
G1 X76.811 Y95.675 E0.02759
G1 X76.469 Y96.486 E0.02759
G1 X76.405 Y96.713 E0.00740
G1 X69.787 Y96.713 E0.20749
G1 X69.787 Y83.287 E0.42096
G1 X76.405 Y83.287 E0.20749
G1 X76.469 Y83.514 E0.00740
G1 X76.811 Y84.325 E0.02759
G1 X77.250 Y85.087 E0.02759
G1 X77.780 Y85.790 E0.02759
G1 X78.392 Y86.422 E0.02759
G1 X79.077 Y86.974 E0.02759
G1 X79.824 Y87.437 E0.02759
G1 X80.624 Y87.806 E0.02759
G1 X81.462 Y88.073 E0.02759
G1 X82.327 Y88.234 E0.02759
G1 X83.181 Y88.287 E0.02682
```

```
G1 X90.213 Y88.287 E0.22049
G1 X90.213 Y91.653 E0.10553
M204 S1250
G1 X90.590 Y92.090 F9000.000
M204 S800
;TYPE:External perimeter
G1 F1200.000
G1 X83.192 Y92.090 E0.23195
G1 X82.373 Y92.141 E0.02573
G1 X81.554 Y92.294 E0.02613
G1 X80.760 Y92.547 E0.02613
G1 X80.003 Y92.895 E0.02613
G1 X79.295 Y93.334 E0.02613
G1 X78.646 Y93.857 E0.02613
G1 X78.067 Y94.456 E0.02613
G1 X77.565 Y95.121 E0.02613
G1 X77.149 Y95.843 E0.02613
G1 X76.826 Y96.611 E0.02613
G1 X76.690 Y97.090 E0.01561
G1 X69.410 Y97.090 E0.22826
G1 X69.410 Y82.910 E0.44460
G1 X76.690 Y82.910 E0.22826
G1 X76.826 Y83.389 E0.01561
G1 X77.149 Y84.157 E0.02613
G1 X77.565 Y84.879 E0.02613
G1 X78.067 Y85.544 E0.02613
G1 X78.646 Y86.143 E0.02613
G1 X79.295 Y86.666 E0.02613
G1 X80.003 Y87.105 E0.02613
```

```
G1 X80.760 Y87.453 E0.02613
G1 X81.554 Y87.706 E0.02613
G1 X82.373 Y87.859 E0.02613
G1 X83.192 Y87.910 E0.02573
G1 X90.590 Y87.910 E0.23195
G1 X90.590 Y92.030 E0.12918
M204 S1250
G1 X90.196 Y92.022 F9000.000
G1 E-2.24000 F2400.000
;WIPE_START
G1 F7200.000
G1 X87.710 Y92.053 E-0.91200
;WIPE_END
G1 E-0.04800 F2400.000
G1 Z0.400 F9000.000
G1 X90.100 Y89.168
G1 Z0.200
G1 E3.20000 F1500.000
M204 S800
;TYPE:Solid infill
;WIDTH:0.422818
G1 F1200.000
G1 X89.501 Y88.570 E0.02674
G1 X88.964 Y88.570 E0.01697
G1 X89.930 Y89.536 E0.04316
G1 X89.930 Y90.073 E0.01697
G1 X88.427 Y88.570 E0.06716
G1 X87.889 Y88.570 E0.01697
G1 X89.930 Y90.611 E0.09116
```

```
G1 X89.930 Y91.148 E0.01697
G1 X87.352 Y88.570 E0.11516
G1 X86.815 Y88.570 E0.01697
G1 X89.675 Y91.430 E0.12778
G1 X89.138 Y91.430 E0.01697
G1 X86.278 Y88.570 E0.12778
G1 X85.740 Y88.570 E0.01697
G1 X88.601 Y91.430 E0.12778
G1 X88.063 Y91.430 E0.01697
G1 X85.203 Y88.570 E0.12778
G1 X84.666 Y88.570 E0.01697
G1 X87.526 Y91.430 E0.12778
G1 X86.989 Y91.430 E0.01697
G1 X84.129 Y88.570 E0.12778
G1 X83.591 Y88.570 E0.01697
G1 X86.452 Y91.430 E0.12778
G1 X85.914 Y91.430 E0.01697
G1 X83.046 Y88.562 E0.12812
G1 X82.474 Y88.527 E0.01812
G1 X85.377 Y91.430 E0.12970
G1 X84.840 Y91.430 E0.01697
G1 X81.841 Y88.431 E0.13397
G1 X81.393 Y88.347 E0.01440
G1 X81.139 Y88.266 E0.00841
G1 X84.303 Y91.430 E0.14133
G1 X83.765 Y91.430 E0.01697
G1 X79.959 Y87.624 E0.17002
M204 S1250
G1 E-2.24000 F2400.000
```

```
;WIPE_START
G1 F7200.000
G1 X81.996 Y89.661 E-0.91200
;WIPE_END
G1 E-0.04800 F2400.000
G1 Z0.400 F9000.000
G1 X76.709 Y84.374
G1 Z0.200
G1 E3.20000 F1500.000
M204 S800
G1 F1200.000
G1 X75.905 Y83.570 E0.03591
G1 X75.368 Y83.570 E0.01697
G1 X83.228 Y91.430 E0.35114
M73 P20 R55
G1 X82.719 Y91.458 E0.01611
G1 X74.831 Y83.570 E0.35239
G1 X74.293 Y83.570 E0.01697
G1 X82.221 Y91.498 E0.35416
G1 X81.769 Y91.582 E0.01454
G1 X73.756 Y83.570 E0.35794
G1 X73.219 Y83.570 E0.01697
G1 X81.324 Y91.675 E0.36207
G1 X80.916 Y91.804 E0.01351
G1 X72.682 Y83.570 E0.36786
G1 X72.144 Y83.570 E0.01697
G1 X80.510 Y91.935 E0.37372
G1 X80.142 Y92.105 E0.01279
G1 X71.607 Y83.570 E0.38128
```

```
G1 X71.070 Y83.570 E0.01697
G1 X79.774 Y92.274 E0.38885
G1 X79.434 Y92.472 E0.01242
G1 X70.533 Y83.570 E0.39767
M73 P20 R54
G1 X70.070 Y83.570 E0.01462
G1 X70.070 Y83.644 E0.00236
G1 X79.103 Y92.677 E0.40352
G1 X78.786 Y92.897 E0.01219
G1 X70.070 Y84.182 E0.38936
G1 X70.070 Y84.719 E0.01697
G1 X78.488 Y93.137 E0.37607
G1 X78.192 Y93.378 E0.01206
G1 X70.070 Y85.256 E0.36283
G1 X70.070 Y85.793 E0.01697
G1 X77.927 Y93.651 E0.35102
G1 X77.663 Y93.924 E0.01200
G1 X70.070 Y86.331 E0.33922
G1 X70.070 Y86.868 E0.01697
G1 X77.420 Y94.218 E0.32834
G1 X77.189 Y94.524 E0.01212
G1 X70.070 Y87.405 E0.31803
G1 X70.070 Y87.942 E0.01697
G1 X76.966 Y94.839 E0.30809
G1 X76.770 Y95.180 E0.01243
G1 X70.070 Y88.480 E0.29932
G1 X70.070 Y89.017 E0.01697
G1 X76.574 Y95.521 E0.29054
G1 X76.411 Y95.896 E0.01290
```

G1 X70.070 Y89.554 E0.28329

G1 X70.070 Y90.092 E0.01697

G1 X76.252 Y96.273 E0.27616

G1 X76.191 Y96.430 E0.00531

G1 X75.871 Y96.430 E0.01010

G1 X70.070 Y90.629 E0.25916

G1 X70.070 Y91.166 E0.01697

G1 X75.334 Y96.430 E0.23516

G1 X74.797 Y96.430 E0.01697

G1 X70.070 Y91.703 E0.21116

G1 X70.070 Y92.241 E0.01697

G1 X74.259 Y96.430 E0.18716

G1 X73.722 Y96.430 E0.01697

G1 X70.070 Y92.778 E0.16316

G1 X70.070 Y93.315 E0.01697

G1 X73.185 Y96.430 E0.13916

G1 X72.648 Y96.430 E0.01697

G1 X70.070 Y93.852 E0.11516

G1 X70.070 Y94.390 E0.01697

G1 X72.110 Y96.430 E0.09116

G1 X71.573 Y96.430 E0.01697

G1 X70.070 Y94.927 E0.06716

G1 X70.070 Y95.464 E0.01697

G1 X71.036 Y96.430 E0.04316

G1 X70.499 Y96.430 E0.01697

G1 X69.900 Y95.832 E0.02673

M204 S1250

; stop printing object tpu print.STL id:1 copy 0

; printing object tpu print.STL id:5 copy 0

```
G1 E-2.24000 F2400.000
;WIPE_START
G1 F7200.000
G1 X70.499 Y96.430 E-0.26801
G1 X71.036 Y96.430 E-0.17013
G1 X70.070 Y95.464 E-0.43262
G1 X70.070 Y95.334 E-0.04124
;WIPE_END
G1 E-0.04800 F2400.000
G1 Z0.400 F9000.000
G1 X69.900 Y74.521
G1 Z0.200
G1 E3.20000 F1500.000
M204 S800
;TYPE:Perimeter
;WIDTH:0.42
G1 F1200.000
G1 X69.838 Y74.521 E0.00194
G1 X69.838 Y61.095 E0.42096
G1 X76.456 Y61.095 E0.20749
G1 X76.520 Y61.322 E0.00740
G1 X76.862 Y62.132 E0.02759
G1 X77.301 Y62.895 E0.02759
G1 X77.831 Y63.597 E0.02759
G1 X78.443 Y64.230 E0.02759
G1 X79.128 Y64.782 E0.02759
G1 X79.876 Y65.245 E0.02759
G1 X80.675 Y65.613 E0.02759
G1 X81.513 Y65.880 E0.02759
```

```
G1 X82.378 Y66.042 E0.02759
G1 X83.232 Y66.095 E0.02682
G1 X90.264 Y66.095 E0.22049
G1 X90.264 Y69.521 E0.10741
G1 X83.232 Y69.521 E0.22049
G1 X82.378 Y69.573 E0.02682
G1 X81.513 Y69.735 E0.02759
G1 X80.675 Y70.002 E0.02759
G1 X79.876 Y70.370 E0.02759
G1 X79.128 Y70.834 E0.02759
G1 X78.443 Y71.386 E0.02759
G1 X77.831 Y72.018 E0.02759
G1 X77.301 Y72.720 E0.02759
G1 X76.862 Y73.483 E0.02759
G1 X76.520 Y74.293 E0.02759
G1 X76.456 Y74.521 E0.00740
G1 X69.960 Y74.521 E0.20367
M204 S1250
G1 X69.461 Y74.898 F9000.000
M204 S800
;TYPE:External perimeter
G1 F1200.000
G1 X69.461 Y60.718 E0.44460
G1 X76.741 Y60.718 E0.22826
G1 X76.877 Y61.197 E0.01561
G1 X77.201 Y61.964 E0.02613
G1 X77.616 Y62.686 E0.02613
G1 X78.118 Y63.352 E0.02613
G1 X78.698 Y63.951 E0.02613
```

```
G1 X79.346 Y64.473 E0.02613
G1 X80.055 Y64.912 E0.02613
G1 X80.811 Y65.261 E0.02613
G1 X81.605 Y65.514 E0.02613
G1 X82.424 Y65.667 E0.02613
G1 X83.243 Y65.718 E0.02573
G1 X90.641 Y65.718 E0.23195
G1 X90.641 Y69.898 E0.13106
G1 X83.243 Y69.898 E0.23195
G1 X82.424 Y69.948 E0.02573
G1 X81.605 Y70.101 E0.02613
G1 X80.811 Y70.354 E0.02613
G1 X80.055 Y70.703 E0.02613
G1 X79.346 Y71.142 E0.02613
G1 X78.698 Y71.665 E0.02613
G1 X78.118 Y72.263 E0.02613
G1 X77.616 Y72.929 E0.02613
G1 X77.201 Y73.651 E0.02613
G1 X76.877 Y74.419 E0.02613
G1 X76.741 Y74.898 E0.01561
G1 X69.521 Y74.898 E0.22638
M204 S1250
G1 X69.654 Y74.547 F9000.000
G1 E-2.24000 F2400.000
;WIPE_START
G1 F7200.000
G1 X69.509 Y72.018 E-0.91200
;WIPE_END
G1 E-0.04800 F2400.000
```

```
G1 Z0.400 F9000.000
G1 X90.151 Y66.976
G1 Z0.200
G1 E3.20000 F1500.000
M204 S800
;TYPE:Solid infill
;WIDTH:0.422818
G1 F1200.000
G1 X89.552 Y66.378 E0.02674
G1 X89.015 Y66.378 E0.01697
G1 X89.981 Y67.344 E0.04316
G1 X89.981 Y67.881 E0.01697
G1 X88.478 Y66.378 E0.06716
G1 X87.941 Y66.378 E0.01697
G1 X89.981 Y68.418 E0.09116
G1 X89.981 Y68.955 E0.01697
G1 X87.403 Y66.378 E0.11516
G1 X86.866 Y66.378 E0.01697
G1 X89.726 Y69.238 E0.12778
G1 X89.189 Y69.238 E0.01697
G1 X86.329 Y66.378 E0.12778
G1 X85.792 Y66.378 E0.01697
G1 X88.652 Y69.238 E0.12778
G1 X88.115 Y69.238 E0.01697
G1 X85.254 Y66.378 E0.12778
G1 X84.717 Y66.378 E0.01697
G1 X87.577 Y69.238 E0.12778
G1 X87.040 Y69.238 E0.01697
G1 X84.180 Y66.378 E0.12778
```

```
G1 X83.643 Y66.378 E0.01697
G1 X86.503 Y69.238 E0.12778
G1 X85.966 Y69.238 E0.01697
G1 X83.098 Y66.370 E0.12812
G1 X82.525 Y66.334 E0.01812
G1 X85.428 Y69.238 E0.12970
G1 X84.891 Y69.238 E0.01697
G1 X81.892 Y66.239 E0.13397
G1 X81.444 Y66.155 E0.01440
G1 X81.190 Y66.074 E0.00841
G1 X84.354 Y69.238 E0.14133
G1 X83.817 Y69.238 E0.01697
G1 X80.011 Y65.432 E0.17002
M204 S1250
G1 E-2.24000 F2400.000
;WIPE_START
G1 F7200.000
G1 X82.047 Y67.468 E-0.91200
;WIPE_END
G1 E-0.04800 F2400.000
G1 Z0.400 F9000.000
G1 X76.760 Y62.181
G1 Z0.200
G1 E3.20000 F1500.000
M204 S800
G1 F1200.000
G1 X75.956 Y61.378 E0.03591
G1 X75.419 Y61.378 E0.01697
G1 X83.279 Y69.238 E0.35114
```

```
G1 X82.770 Y69.266 E0.01611
G1 X74.882 Y61.378 E0.35239
G1 X74.345 Y61.378 E0.01697
G1 X82.272 Y69.305 E0.35416
G1 X81.820 Y69.390 E0.01454
G1 X73.807 Y61.378 E0.35794
G1 X73.270 Y61.378 E0.01697
G1 X81.375 Y69.482 E0.36207
G1 X80.967 Y69.612 E0.01351
G1 X72.733 Y61.378 E0.36786
G1 X72.195 Y61.378 E0.01697
G1 X80.561 Y69.743 E0.37372
G1 X80.193 Y69.913 E0.01279
G1 X71.658 Y61.378 E0.38128
G1 X71.121 Y61.378 E0.01697
G1 X79.825 Y70.082 E0.38885
G1 X79.485 Y70.279 E0.01242
G1 X70.584 Y61.378 E0.39767
G1 X70.121 Y61.378 E0.01462
G1 X70.121 Y61.452 E0.00236
G1 X79.154 Y70.485 E0.40352
G1 X78.837 Y70.705 E0.01219
G1 X70.121 Y61.989 E0.38936
G1 X70.121 Y62.527 E0.01697
G1 X78.539 Y70.945 E0.37607
G1 X78.243 Y71.186 E0.01206
G1 X70.121 Y63.064 E0.36283
G1 X70.121 Y63.601 E0.01697
G1 X77.979 Y71.459 E0.35102
```

```
G1 X77.714 Y71.732 E0.01200
G1 X70.121 Y64.138 E0.33922
G1 X70.121 Y64.676 E0.01697
G1 X77.471 Y72.026 E0.32834
G1 X77.240 Y72.332 E0.01212
G1 X70.121 Y65.213 E0.31803
G1 X70.121 Y65.750 E0.01697
G1 X77.018 Y72.647 E0.30809
G1 X76.821 Y72.988 E0.01243
G1 X70.121 Y66.287 E0.29932
G1 X70.121 Y66.825 E0.01697
G1 X76.625 Y73.328 E0.29054
G1 X76.462 Y73.703 E0.01290
G1 X70.121 Y67.362 E0.28329
M73 P21 R54
G1 X70.121 Y67.899 E0.01697
G1 X76.303 Y74.081 E0.27616
G1 X76.242 Y74.238 E0.00531
G1 X75.922 Y74.238 E0.01010
G1 X70.121 Y68.436 E0.25916
G1 X70.121 Y68.974 E0.01697
G1 X75.385 Y74.238 E0.23516
G1 X74.848 Y74.238 E0.01697
G1 X70.121 Y69.511 E0.21116
G1 X70.121 Y70.048 E0.01697
G1 X74.311 Y74.238 E0.18716
G1 X73.773 Y74.238 E0.01697
G1 X70.121 Y70.585 E0.16316
G1 X70.121 Y71.123 E0.01697
```

```
G1 X73.236 Y74.238 E0.13916
G1 X72.699 Y74.238 E0.01697
G1 X70.121 Y71.660 E0.11516
G1 X70.121 Y72.197 E0.01697
G1 X72.162 Y74.238 E0.09116
G1 X71.624 Y74.238 E0.01697
G1 X70.121 Y72.734 E0.06716
G1 X70.121 Y73.272 E0.01697
G1 X71.087 Y74.238 E0.04316
G1 X70.550 Y74.238 E0.01697
G1 X69.951 Y73.639 E0.02673
M204 S1250
; stop printing object tpu print.STL id:5 copy 0
; printing object tpu print.STL id:9 copy 0
G1 E-2.24000 F2400.000
;WIPE_START
G1 F7200.000
G1 X70.550 Y74.238 E-0.26801
G1 X71.087 Y74.238 E-0.17013
G1 X70.121 Y73.272 E-0.43262
G1 X70.121 Y73.142 E-0.04124
;WIPE_END
G1 E-0.04800 F2400.000
G1 Z0.400 F9000.000
G1 X69.951 Y54.572
G1 Z0.200
G1 E3.20000 F1500.000
M204 S800
;TYPE:Perimeter
```

```
;WIDTH:0.42
G1 F1200.000
G1 X69.831 Y54.572 E0.00378
G1 X69.831 Y41.146 E0.42096
G1 X76.448 Y41.146 E0.20749
G1 X76.513 Y41.373 E0.00740
G1 X76.855 Y42.183 E0.02759
G1 X77.294 Y42.946 E0.02759
G1 X77.823 Y43.648 E0.02759
G1 X78.435 Y44.281 E0.02759
G1 X79.120 Y44.833 E0.02759
G1 X79.868 Y45.296 E0.02759
G1 X80.667 Y45.664 E0.02759
G1 X81.506 Y45.931 E0.02759
G1 X82.370 Y46.093 E0.02759
G1 X83.224 Y46.146 E0.02682
G1 X90.257 Y46.146 E0.22049
G1 X90.257 Y49.572 E0.10741
G1 X83.224 Y49.572 E0.22049
G1 X82.370 Y49.624 E0.02682
G1 X81.506 Y49.786 E0.02759
G1 X80.667 Y50.053 E0.02759
G1 X79.868 Y50.421 E0.02759
G1 X79.120 Y50.885 E0.02759
G1 X78.435 Y51.437 E0.02759
G1 X77.823 Y52.069 E0.02759
G1 X77.294 Y52.772 E0.02759
G1 X76.855 Y53.534 E0.02759
G1 X76.513 Y54.345 E0.02759
```

```
G1 X76.448 Y54.572 E0.00740
G1 X70.011 Y54.572 E0.20183
M204 S1250
G1 X69.454 Y54.949 F9000.000
M204 S800
;TYPE:External perimeter
G1 F1200.000
G1 X69.454 Y40.769 E0.44460
G1 X76.734 Y40.769 E0.22826
G1 X76.869 Y41.248 E0.01561
G1 X77.193 Y42.015 E0.02613
G1 X77.609 Y42.738 E0.02613
G1 X78.110 Y43.403 E0.02613
G1 X78.690 Y44.002 E0.02613
G1 X79.339 Y44.524 E0.02613
G1 X80.047 Y44.963 E0.02613
G1 X80.804 Y45.312 E0.02613
G1 X81.598 Y45.565 E0.02613
G1 X82.417 Y45.718 E0.02613
G1 X83.236 Y45.769 E0.02573
G1 X90.634 Y45.769 E0.23195
G1 X90.634 Y49.949 E0.13106
G1 X83.236 Y49.949 E0.23195
G1 X82.417 Y49.999 E0.02573
G1 X81.598 Y50.153 E0.02613
G1 X80.804 Y50.405 E0.02613
G1 X80.047 Y50.754 E0.02613
G1 X79.339 Y51.193 E0.02613
G1 X78.690 Y51.716 E0.02613
```

```
G1 X78.110 Y52.314 E0.02613
G1 X77.609 Y52.980 E0.02613
G1 X77.193 Y53.702 E0.02613
G1 X76.869 Y54.470 E0.02613
G1 X76.734 Y54.949 E0.01561
G1 X69.514 Y54.949 E0.22638
M204 S1250
G1 X69.646 Y54.598 F9000.000
G1 E-2.24000 F2400.000
;WIPE_START
G1 F7200.000
G1 X69.501 Y52.069 E-0.91200
;WIPE_END
G1 E-0.04800 F2400.000
G1 Z0.400 F9000.000
G1 X90.143 Y47.027
G1 Z0.200
G1 E3.20000 F1500.000
M204 S800
;TYPE:Solid infill
;WIDTH:0.422818
G1 F1200.000
G1 X89.545 Y46.429 E0.02674
G1 X89.008 Y46.429 E0.01697
G1 X89.974 Y47.395 E0.04316
G1 X89.974 Y47.932 E0.01697
G1 X88.470 Y46.429 E0.06716
G1 X87.933 Y46.429 E0.01697
G1 X89.974 Y48.469 E0.09116
```

```
G1 X89.974 Y49.006 E0.01697
G1 X87.396 Y46.429 E0.11516
G1 X86.859 Y46.429 E0.01697
G1 X89.719 Y49.289 E0.12778
G1 X89.182 Y49.289 E0.01697
G1 X86.321 Y46.429 E0.12778
G1 X85.784 Y46.429 E0.01697
G1 X88.644 Y49.289 E0.12778
G1 X88.107 Y49.289 E0.01697
G1 X85.247 Y46.429 E0.12778
G1 X84.710 Y46.429 E0.01697
G1 X87.570 Y49.289 E0.12778
G1 X87.033 Y49.289 E0.01697
G1 X84.172 Y46.429 E0.12778
G1 X83.635 Y46.429 E0.01697
G1 X86.495 Y49.289 E0.12778
G1 X85.958 Y49.289 E0.01697
G1 X83.090 Y46.421 E0.12812
G1 X82.517 Y46.385 E0.01812
G1 X85.421 Y49.289 E0.12970
G1 X84.884 Y49.289 E0.01697
G1 X81.885 Y46.290 E0.13397
G1 X81.436 Y46.206 E0.01440
G1 X81.183 Y46.125 E0.00841
G1 X84.346 Y49.289 E0.14133
G1 X83.809 Y49.289 E0.01697
G1 X80.003 Y45.483 E0.17002
M204 S1250
G1 E-2.24000 F2400.000
```

```
;WIPE_START
G1 F7200.000
G1 X82.040 Y47.519 E-0.91200
;WIPE_END
G1 E-0.04800 F2400.000
G1 Z0.400 F9000.000
G1 X76.753 Y42.232
G1 Z0.200
G1 E3.20000 F1500.000
M204 S800
G1 F1200.000
G1 X75.949 Y41.429 E0.03591
G1 X75.412 Y41.429 E0.01697
G1 X83.272 Y49.289 E0.35114
G1 X82.762 Y49.317 E0.01611
G1 X74.874 Y41.429 E0.35239
G1 X74.337 Y41.429 E0.01697
G1 X82.265 Y49.356 E0.35416
G1 X81.812 Y49.441 E0.01454
G1 X73.800 Y41.429 E0.35794
G1 X73.263 Y41.429 E0.01697
G1 X81.367 Y49.533 E0.36207
G1 X80.960 Y49.663 E0.01351
G1 X72.725 Y41.429 E0.36786
G1 X72.188 Y41.429 E0.01697
G1 X80.554 Y49.794 E0.37372
G1 X80.186 Y49.964 E0.01279
G1 X71.651 Y41.429 E0.38128
G1 X71.113 Y41.429 E0.01697
```

```
G1 X79.818 Y50.133 E0.38885
G1 X79.478 Y50.330 E0.01242
G1 X70.576 Y41.429 E0.39767
G1 X70.114 Y41.429 E0.01462
G1 X70.114 Y41.503 E0.00236
G1 X79.146 Y50.536 E0.40352
G1 X78.829 Y50.756 E0.01219
G1 X70.114 Y42.040 E0.38936
G1 X70.114 Y42.578 E0.01697
G1 X78.532 Y50.996 E0.37607
G1 X78.235 Y51.237 E0.01206
G1 X70.114 Y43.115 E0.36283
G1 X70.114 Y43.652 E0.01697
G1 X77.971 Y51.510 E0.35102
M73 P21 R53
G1 X77.707 Y51.783 E0.01200
G1 X70.114 Y44.189 E0.33922
G1 X70.114 Y44.727 E0.01697
G1 X77.463 Y52.077 E0.32834
G1 X77.233 Y52.383 E0.01212
G1 X70.114 Y45.264 E0.31803
G1 X70.114 Y45.801 E0.01697
G1 X77.010 Y52.698 E0.30809
G1 X76.814 Y53.039 E0.01243
G1 X70.114 Y46.338 E0.29932
G1 X70.114 Y46.876 E0.01697
G1 X76.617 Y53.380 E0.29054
G1 X76.455 Y53.754 E0.01290
G1 X70.114 Y47.413 E0.28329
```

```
G1 X70.114 Y47.950 E0.01697
G1 X76.295 Y54.132 E0.27616
G1 X76.235 Y54.289 E0.00531
G1 X75.915 Y54.289 E0.01010
G1 X70.114 Y48.487 E0.25916
G1 X70.114 Y49.025 E0.01697
G1 X75.378 Y54.289 E0.23516
G1 X74.840 Y54.289 E0.01697
G1 X70.114 Y49.562 E0.21116
G1 X70.114 Y50.099 E0.01697
G1 X74.303 Y54.289 E0.18716
G1 X73.766 Y54.289 E0.01697
G1 X70.114 Y50.637 E0.16316
G1 X70.114 Y51.174 E0.01697
G1 X73.229 Y54.289 E0.13916
G1 X72.691 Y54.289 E0.01697
G1 X70.114 Y51.711 E0.11516
G1 X70.114 Y52.248 E0.01697
G1 X72.154 Y54.289 E0.09116
G1 X71.617 Y54.289 E0.01697
G1 X70.114 Y52.786 E0.06716
G1 X70.114 Y53.323 E0.01697
G1 X71.080 Y54.289 E0.04316
G1 X70.542 Y54.289 E0.01697
G1 X69.944 Y53.690 E0.02673
M204 S1250
; stop printing object tpu print.STL id:9 copy 0
; printing object Petg print.STL id:8 copy 0
; stop printing object Petg print.STL id:8 copy 0
```

```
; printing object Petg print.STL id:4 copy 0
; stop printing object Petg print.STL id:4 copy 0
; printing object tpu print.STL id:29 copy 0
G1 E-2.24000 F2400.000
;WIPE_START
G1 F7200.000
G1 X70.542 Y54.289 E-0.26801
G1 X71.080 Y54.289 E-0.17013
G1 X70.114 Y53.323 E-0.43262
G1 X70.114 Y53.193 E-0.04124
;WIPE_END
G1 E-0.04800 F2400.000
G1 Z0.400 F9000.000
G1 X120.481 Y54.747
G1 Z0.200
G1 E3.20000 F1500.000
M204 S800
;TYPE:Perimeter
;WIDTH:0.42
G1 F1200.000
G1 X120.481 Y41.321 E0.42096
G1 X127.099 Y41.321 E0.20749
G1 X127.163 Y41.548 E0.00740
G1 X127.505 Y42.359 E0.02759
G1 X127.944 Y43.121 E0.02759
G1 X128.474 Y43.824 E0.02759
G1 X129.086 Y44.456 E0.02759
G1 X129.771 Y45.008 E0.02759
G1 X130.518 Y45.471 E0.02759
```

```
G1 X131.318 Y45.839 E0.02759
G1 X132.156 Y46.106 E0.02759
G1 X133.021 Y46.268 E0.02759
G1 X133.874 Y46.321 E0.02682
G1 X140.907 Y46.321 E0.22049
G1 X140.907 Y49.747 E0.10741
G1 X133.874 Y49.747 E0.22049
G1 X133.021 Y49.799 E0.02682
G1 X132.156 Y49.961 E0.02759
G1 X131.318 Y50.228 E0.02759
G1 X130.518 Y50.596 E0.02759
G1 X129.771 Y51.060 E0.02759
G1 X129.086 Y51.612 E0.02759
G1 X128.474 Y52.244 E0.02759
G1 X127.944 Y52.947 E0.02759
G1 X127.505 Y53.709 E0.02759
G1 X127.163 Y54.520 E0.02759
G1 X127.099 Y54.747 E0.00740
G1 X120.541 Y54.747 E0.20561
M204 S1250
G1 X120.104 Y55.124 F9000.000
M204 S800
;TYPE:External perimeter
G1 F1200.000
G1 X120.104 Y40.944 E0.44460
G1 X127.384 Y40.944 E0.22826
G1 X127.519 Y41.423 E0.01561
G1 X127.843 Y42.191 E0.02613
G1 X128.259 Y42.913 E0.02613
```

```
G1 X128.761 Y43.578 E0.02613
G1 X129.340 Y44.177 E0.02613
G1 X129.989 Y44.699 E0.02613
G1 X130.697 Y45.139 E0.02613
G1 X131.454 Y45.487 E0.02613
G1 X132.248 Y45.740 E0.02613
G1 X133.067 Y45.893 E0.02613
G1 X133.886 Y45.944 E0.02573
G1 X141.284 Y45.944 E0.23195
G1 X141.284 Y50.124 E0.13106
G1 X133.886 Y50.124 E0.23195
G1 X133.067 Y50.174 E0.02573
G1 X132.248 Y50.328 E0.02613
G1 X131.454 Y50.580 E0.02613
G1 X130.697 Y50.929 E0.02613
G1 X129.989 Y51.368 E0.02613
G1 X129.340 Y51.891 E0.02613
G1 X128.761 Y52.490 E0.02613
G1 X128.259 Y53.155 E0.02613
G1 X127.843 Y53.877 E0.02613
G1 X127.519 Y54.645 E0.02613
G1 X127.384 Y55.124 E0.01561
G1 X120.164 Y55.124 E0.22638
M204 S1250
G1 X120.296 Y54.773 F9000.000
G1 E-2.24000 F2400.000
;WIPE_START
G1 F7200.000
G1 X120.152 Y52.244 E-0.91200
```

```
;WIPE_END
G1 E-0.04800 F2400.000
G1 Z0.400 F9000.000
G1 X140.794 Y47.202
G1 Z0.200
G1 E3.20000 F1500.000
M204 S800
;TYPE:Solid infill
;WIDTH:0.422818
G1 F1200.000
G1 X140.195 Y46.604 E0.02674
G1 X139.658 Y46.604 E0.01697
G1 X140.624 Y47.570 E0.04316
G1 X140.624 Y48.107 E0.01697
G1 X139.121 Y46.604 E0.06716
G1 X138.583 Y46.604 E0.01697
G1 X140.624 Y48.644 E0.09116
G1 X140.624 Y49.182 E0.01697
G1 X138.046 Y46.604 E0.11516
G1 X137.509 Y46.604 E0.01697
G1 X140.369 Y49.464 E0.12778
G1 X139.832 Y49.464 E0.01697
G1 X136.972 Y46.604 E0.12778
M73 P22 R53
G1 X136.434 Y46.604 E0.01697
G1 X139.295 Y49.464 E0.12778
G1 X138.757 Y49.464 E0.01697
G1 X135.897 Y46.604 E0.12778
G1 X135.360 Y46.604 E0.01697
```

```
G1 X138.220 Y49.464 E0.12778
G1 X137.683 Y49.464 E0.01697
G1 X134.823 Y46.604 E0.12778
G1 X134.285 Y46.604 E0.01697
G1 X137.146 Y49.464 E0.12778
G1 X136.608 Y49.464 E0.01697
G1 X133.740 Y46.596 E0.12812
G1 X133.168 Y46.561 E0.01812
G1 X136.071 Y49.464 E0.12970
G1 X135.534 Y49.464 E0.01697
G1 X132.535 Y46.465 E0.13397
G1 X132.087 Y46.381 E0.01440
G1 X131.833 Y46.300 E0.00841
G1 X134.997 Y49.464 E0.14133
G1 X134.459 Y49.464 E0.01697
G1 X130.653 Y45.658 E0.17002
M204 S1250
G1 E-2.24000 F2400.000
;WIPE_START
G1 F7200.000
G1 X132.690 Y47.694 E-0.91200
;WIPE_END
G1 E-0.04800 F2400.000
G1 Z0.400 F9000.000
G1 X127.403 Y42.407
G1 Z0.200
G1 E3.20000 F1500.000
M204 S800
G1 F1200.000
```

```
G1 X126.599 Y41.604 E0.03591
G1 X126.062 Y41.604 E0.01697
G1 X133.922 Y49.464 E0.35114
G1 X133.413 Y49.492 E0.01611
G1 X125.525 Y41.604 E0.35239
G1 X124.987 Y41.604 E0.01697
G1 X132.915 Y49.531 E0.35416
G1 X132.463 Y49.616 E0.01454
G1 X124.450 Y41.604 E0.35794
G1 X123.913 Y41.604 E0.01697
G1 X132.018 Y49.708 E0.36207
G1 X131.610 Y49.838 E0.01351
G1 X123.375 Y41.604 E0.36786
G1 X122.838 Y41.604 E0.01697
G1 X131.204 Y49.969 E0.37372
G1 X130.836 Y50.139 E0.01279
G1 X122.301 Y41.604 E0.38128
G1 X121.764 Y41.604 E0.01697
G1 X130.468 Y50.308 E0.38885
G1 X130.128 Y50.505 E0.01242
G1 X121.226 Y41.604 E0.39767
G1 X120.764 Y41.604 E0.01462
G1 X120.764 Y41.678 E0.00236
G1 X129.797 Y50.711 E0.40352
G1 X129.480 Y50.931 E0.01219
G1 X120.764 Y42.215 E0.38936
G1 X120.764 Y42.753 E0.01697
G1 X129.182 Y51.171 E0.37607
G1 X128.886 Y51.412 E0.01206
```

```
G1 X120.764 Y43.290 E0.36283
G1 X120.764 Y43.827 E0.01697
G1 X128.621 Y51.685 E0.35102
G1 X128.357 Y51.958 E0.01200
G1 X120.764 Y44.365 E0.33922
G1 X120.764 Y44.902 E0.01697
G1 X128.114 Y52.252 E0.32834
G1 X127.883 Y52.558 E0.01212
G1 X120.764 Y45.439 E0.31803
G1 X120.764 Y45.976 E0.01697
G1 X127.660 Y52.873 E0.30809
G1 X127.464 Y53.214 E0.01243
G1 X120.764 Y46.514 E0.29932
G1 X120.764 Y47.051 E0.01697
G1 X127.268 Y53.555 E0.29054
G1 X127.105 Y53.929 E0.01290
G1 X120.764 Y47.588 E0.28329
G1 X120.764 Y48.125 E0.01697
G1 X126.946 Y54.307 E0.27616
G1 X126.885 Y54.464 E0.00531
G1 X126.565 Y54.464 E0.01010
G1 X120.764 Y48.663 E0.25916
G1 X120.764 Y49.200 E0.01697
G1 X126.028 Y54.464 E0.23516
G1 X125.491 Y54.464 E0.01697
G1 X120.764 Y49.737 E0.21116
G1 X120.764 Y50.274 E0.01697
G1 X124.953 Y54.464 E0.18716
G1 X124.416 Y54.464 E0.01697
```

```
G1 X120.764 Y50.812 E0.16316
G1 X120.764 Y51.349 E0.01697
G1 X123.879 Y54.464 E0.13916
G1 X123.342 Y54.464 E0.01697
G1 X120.764 Y51.886 E0.11516
G1 X120.764 Y52.423 E0.01697
G1 X122.804 Y54.464 E0.09116
G1 X122.267 Y54.464 E0.01697
G1 X120.764 Y52.961 E0.06716
G1 X120.764 Y53.498 E0.01697
G1 X121.730 Y54.464 E0.04316
G1 X121.193 Y54.464 E0.01697
G1 X120.594 Y53.865 E0.02673
M204 S1250
; stop printing object tpu print.STL id:29 copy 0
; printing object tpu print.STL id:25 copy 0
G1 E-2.24000 F2400.000
;WIPE_START
G1 F7200.000
G1 X121.193 Y54.464 E-0.26801
G1 X121.730 Y54.464 E-0.17013
G1 X120.764 Y53.498 E-0.43262
G1 X120.764 Y53.368 E-0.04124
;WIPE_END
G1 E-0.04800 F2400.000
G1 Z0.400 F9000.000
G1 X120.594 Y61.270
G1 Z0.200
G1 E3.20000 F1500.000
```

```
M204 S800
;TYPE:Perimeter
;WIDTH:0.42
G1 F1200.000
G1 X127.106 Y61.270 E0.20418
G1 X127.171 Y61.497 E0.00740
G1 X127.512 Y62.308 E0.02759
G1 X127.952 Y63.070 E0.02759
G1 X128.481 Y63.772 E0.02759
G1 X129.093 Y64.405 E0.02759
G1 X129.778 Y64.957 E0.02759
G1 X130.526 Y65.420 E0.02759
G1 X131.325 Y65.788 E0.02759
G1 X132.163 Y66.055 E0.02759
G1 X133.028 Y66.217 E0.02759
G1 X133.882 Y66.270 E0.02682
G1 X140.914 Y66.270 E0.22049
G1 X140.914 Y69.696 E0.10741
G1 X133.882 Y69.696 E0.22049
G1 X133.028 Y69.748 E0.02682
G1 X132.163 Y69.910 E0.02759
G1 X131.325 Y70.177 E0.02759
G1 X130.526 Y70.545 E0.02759
G1 X129.778 Y71.009 E0.02759
G1 X129.093 Y71.561 E0.02759
G1 X128.481 Y72.193 E0.02759
G1 X127.952 Y72.896 E0.02759
G1 X127.512 Y73.658 E0.02759
G1 X127.171 Y74.469 E0.02759
```

```
G1 X127.106 Y74.696 E0.00740
G1 X120.488 Y74.696 E0.20749
G1 X120.488 Y61.270 E0.42096
G1 X120.534 Y61.270 E0.00143
M204 S1250
G1 X120.111 Y60.893 F9000.000
M204 S800
;TYPE:External perimeter
G1 F1200.000
G1 X127.391 Y60.893 E0.22826
G1 X127.527 Y61.372 E0.01561
G1 X127.851 Y62.139 E0.02613
G1 X128.267 Y62.862 E0.02613
G1 X128.768 Y63.527 E0.02613
G1 X129.348 Y64.126 E0.02613
G1 X129.997 Y64.648 E0.02613
G1 X130.705 Y65.087 E0.02613
G1 X131.462 Y65.436 E0.02613
G1 X132.256 Y65.689 E0.02613
G1 X133.075 Y65.842 E0.02613
G1 X133.894 Y65.893 E0.02573
G1 X141.291 Y65.893 E0.23195
G1 X141.291 Y70.073 E0.13106
G1 X133.894 Y70.073 E0.23195
G1 X133.075 Y70.123 E0.02573
G1 X132.256 Y70.277 E0.02613
G1 X131.462 Y70.529 E0.02613
G1 X130.705 Y70.878 E0.02613
G1 X129.997 Y71.317 E0.02613
```

```
G1 X129.348 Y71.840 E0.02613
G1 X128.768 Y72.439 E0.02613
G1 X128.267 Y73.104 E0.02613
G1 X127.851 Y73.826 E0.02613
G1 X127.527 Y74.594 E0.02613
G1 X127.391 Y75.073 E0.01561
G1 X120.111 Y75.073 E0.22826
G1 X120.111 Y60.953 E0.44272
M204 S1250
G1 X120.485 Y61.036 F9000.000
G1 E-2.24000 F2400.000
;WIPE_START
G1 F7200.000
G1 X122.991 Y60.929 E-0.91200
;WIPE_END
G1 E-0.04800 F2400.000
G1 Z0.400 F9000.000
G1 X140.801 Y67.151
G1 Z0.200
G1 E3.20000 F1500.000
M204 S800
;TYPE:Solid infill
;WIDTH:0.422818
G1 F1200.000
G1 X140.203 Y66.553 E0.02674
G1 X139.665 Y66.553 E0.01697
G1 X140.632 Y67.519 E0.04316
G1 X140.632 Y68.056 E0.01697
G1 X139.128 Y66.553 E0.06716
```

```
G1 X138.591 Y66.553 E0.01697
G1 X140.632 Y68.593 E0.09116
G1 X140.632 Y69.131 E0.01697
G1 X138.054 Y66.553 E0.11516
G1 X137.516 Y66.553 E0.01697
G1 X140.377 Y69.413 E0.12778
G1 X139.839 Y69.413 E0.01697
G1 X136.979 Y66.553 E0.12778
G1 X136.442 Y66.553 E0.01697
G1 X139.302 Y69.413 E0.12778
G1 X138.765 Y69.413 E0.01697
G1 X135.905 Y66.553 E0.12778
G1 X135.367 Y66.553 E0.01697
G1 X138.228 Y69.413 E0.12778
G1 X137.690 Y69.413 E0.01697
G1 X134.830 Y66.553 E0.12778
G1 X134.293 Y66.553 E0.01697
G1 X137.153 Y69.413 E0.12778
G1 X136.616 Y69.413 E0.01697
G1 X133.748 Y66.545 E0.12812
G1 X133.175 Y66.510 E0.01812
G1 X136.079 Y69.413 E0.12970
G1 X135.541 Y69.413 E0.01697
G1 X132.542 Y66.414 E0.13397
G1 X132.094 Y66.330 E0.01440
G1 X131.840 Y66.249 E0.00841
G1 X135.004 Y69.413 E0.14133
G1 X134.467 Y69.413 E0.01697
G1 X130.661 Y65.607 E0.17002
```

```
M204 S1250
G1 E-2.24000 F2400.000
;WIPE_START
G1 F7200.000
G1 X132.697 Y67.643 E-0.91200
;WIPE_END
G1 E-0.04800 F2400.000
G1 Z0.400 F9000.000
G1 X127.410 Y62.356
G1 Z0.200
G1 E3.20000 F1500.000
M204 S800
G1 F1200.000
G1 X126.607 Y61.553 E0.03591
G1 X126.069 Y61.553 E0.01697
G1 X133.930 Y69.413 E0.35114
G1 X133.420 Y69.441 E0.01611
G1 X125.532 Y61.553 E0.35239
G1 X124.995 Y61.553 E0.01697
G1 X132.923 Y69.480 E0.35416
G1 X132.470 Y69.565 E0.01454
G1 X124.457 Y61.553 E0.35794
G1 X123.920 Y61.553 E0.01697
G1 X132.025 Y69.657 E0.36207
G1 X131.618 Y69.787 E0.01351
G1 X123.383 Y61.553 E0.36786
G1 X122.846 Y61.553 E0.01697
G1 X131.211 Y69.918 E0.37372
G1 X130.843 Y70.088 E0.01279
```

G1 X122.308 Y61.553 E0.38128

G1 X121.771 Y61.553 E0.01697

G1 X130.476 Y70.257 E0.38885

G1 X130.136 Y70.454 E0.01242

G1 X121.234 Y61.553 E0.39767

G1 X120.771 Y61.553 E0.01462

G1 X120.771 Y61.627 E0.00236

G1 X129.804 Y70.660 E0.40352

G1 X129.487 Y70.880 E0.01219

G1 X120.771 Y62.164 E0.38936

G1 X120.771 Y62.702 E0.01697

G1 X129.190 Y71.120 E0.37607

G1 X128.893 Y71.361 E0.01206

G1 X120.771 Y63.239 E0.36283

G1 X120.771 Y63.776 E0.01697

G1 X128.629 Y71.634 E0.35102

M73 P23 R53

G1 X128.365 Y71.907 E0.01200

G1 X120.771 Y64.313 E0.33922

G1 X120.771 Y64.851 E0.01697

G1 X128.121 Y72.201 E0.32834

G1 X127.890 Y72.507 E0.01212

G1 X120.771 Y65.388 E0.31803

G1 X120.771 Y65.925 E0.01697

G1 X127.668 Y72.822 E0.30809

G1 X127.471 Y73.163 E0.01243

G1 X120.771 Y66.462 E0.29932

M73 P23 R52

G1 X120.771 Y67.000 E0.01697

```
G1 X127.275 Y73.504 E0.29054
G1 X127.113 Y73.878 E0.01290
G1 X120.771 Y67.537 E0.28329
G1 X120.771 Y68.074 E0.01697
G1 X126.953 Y74.256 E0.27616
G1 X126.892 Y74.413 E0.00531
G1 X126.573 Y74.413 E0.01010
G1 X120.771 Y68.612 E0.25916
G1 X120.771 Y69.149 E0.01697
G1 X126.035 Y74.413 E0.23516
G1 X125.498 Y74.413 E0.01697
G1 X120.771 Y69.686 E0.21116
G1 X120.771 Y70.223 E0.01697
G1 X124.961 Y74.413 E0.18716
G1 X124.424 Y74.413 E0.01697
G1 X120.771 Y70.761 E0.16316
G1 X120.771 Y71.298 E0.01697
G1 X123.886 Y74.413 E0.13916
G1 X123.349 Y74.413 E0.01697
G1 X120.771 Y71.835 E0.11516
G1 X120.771 Y72.372 E0.01697
G1 X122.812 Y74.413 E0.09116
G1 X122.275 Y74.413 E0.01697
G1 X120.771 Y72.910 E0.06716
G1 X120.771 Y73.447 E0.01697
G1 X121.737 Y74.413 E0.04316
G1 X121.200 Y74.413 E0.01697
G1 X120.602 Y73.814 E0.02673
M204 S1250
```

```
; stop printing object tpu print.STL id:25 copy 0
; printing object Petg print.STL id:24 copy 0
; stop printing object Petg print.STL id:24 copy 0
; printing object Petg print.STL id:28 copy 0
; stop printing object Petg print.STL id:28 copy 0
;LAYER_CHANGE
;Z:0.35
;HEIGHT:0.15
;BEFORE_LAYER_CHANGE
G92 E0.0
;0.35


G1 E-2.24000 F2400.000
;WIPE_START
G1 F7200.000
G1 X121.200 Y74.413 E-0.26801
G1 X121.737 Y74.413 E-0.17013
G1 X120.771 Y73.447 E-0.43262
G1 X120.771 Y73.317 E-0.04124
;WIPE_END
G1 E-0.04800 F2400.000
G1 Z0.400 F9000.000
;AFTER_LAYER_CHANGE
;0.35
M104 S240 T1 ; set temperature
M140 S90 ; set bed temperature
G1 X164.613 Y38.341
G1 Z0.350
```

```
G1 E3.20000 F1500.000
;TYPE:Skirt
;WIDTH:0.42
G1 F2400.000
G1 X165.363 Y38.841 E0.02179
G1 X165.877 Y39.594 E0.02206
G1 X166.070 Y40.486 E0.02206
G1 X166.078 Y75.483 E0.84640
G1 X166.019 Y137.174 E1.49198
G1 X165.837 Y138.056 E0.02179
G1 X165.338 Y138.810 E0.02188
G1 X164.568 Y139.334 E0.02252
G1 X163.656 Y139.522 E0.02252
G1 X119.638 Y139.522 E1.06457
G1 X68.980 Y139.347 E1.22518
G1 X20.144 Y138.765 E1.18118
G1 X19.274 Y138.577 E0.02152
G1 X18.534 Y138.082 E0.02152
G1 X18.012 Y137.312 E0.02252
G1 X17.825 Y136.399 E0.02252
G1 X17.877 Y39.772 E2.33694
G1 X18.074 Y38.831 E0.02324
G1 X18.424 Y38.262 E0.01616
G1 X18.918 Y37.812 E0.01616
G1 X19.518 Y37.518 E0.01616
G1 X20.176 Y37.402 E0.01616
G1 X62.258 Y37.401 E1.01776
G1 X163.727 Y38.157 E2.45408
G1 X164.554 Y38.329 E0.02043
```

```
; printing object tpu print.STL id:19 copy 0
G1 E-2.24000
;WIPE_START
G1 F7200.000
G1 X165.363 Y38.841 E-0.30305
G1 X165.877 Y39.594 E-0.28889
G1 X166.070 Y40.486 E-0.28889
G1 X166.071 Y40.584 E-0.03118
;WIPE_END
G1 E-0.04800 F2400.000
G1 Z0.550 F9000.000
G1 X41.611 Y45.421
G1 Z0.350
G1 E3.20000 F1500.000
M204 S800
;TYPE:Perimeter
;WIDTH:0.45
G1 F2400.000
G1 X41.611 Y49.135 E0.09678
G1 X34.439 Y49.135 E0.18688
G1 X33.599 Y49.187 E0.02194
G1 X32.752 Y49.345 E0.02246
G1 X31.930 Y49.607 E0.02246
G1 X31.147 Y49.968 E0.02246
G1 X30.415 Y50.422 E0.02246
G1 X29.743 Y50.963 E0.02246
G1 X29.144 Y51.582 E0.02246
G1 X28.625 Y52.270 E0.02246
G1 X28.195 Y53.017 E0.02246
```

```
G1 X27.860 Y53.812 E0.02246
G1 X27.768 Y54.135 E0.00876
G1 X20.897 Y54.135 E0.17903
G1 X20.897 Y40.421 E0.35734
G1 X27.768 Y40.421 E0.17903
G1 X27.860 Y40.744 E0.00876
G1 X28.195 Y41.538 E0.02246
G1 X28.625 Y42.285 E0.02246
G1 X29.144 Y42.974 E0.02246
G1 X29.743 Y43.593 E0.02246
G1 X30.415 Y44.134 E0.02246
G1 X31.147 Y44.588 E0.02246
G1 X31.930 Y44.949 E0.02246
G1 X32.752 Y45.210 E0.02246
G1 X33.599 Y45.369 E0.02246
G1 X34.439 Y45.421 E0.02194
G1 X41.551 Y45.421 E0.18531
M204 S1250
G1 X42.029 Y45.003 F9000.000
M204 S800
;TYPE:External perimeter
G1 F1800.000
G1 X42.029 Y49.553 E0.11855
G1 X34.452 Y49.553 E0.19743
G1 X33.650 Y49.602 E0.02093
G1 X32.854 Y49.751 E0.02112
G1 X32.082 Y49.997 E0.02112
G1 X31.345 Y50.336 E0.02112
G1 X30.657 Y50.763 E0.02112
```

```
G1 X30.026 Y51.272 E0.02112
G1 X29.462 Y51.854 E0.02112
G1 X28.974 Y52.501 E0.02112
G1 X28.570 Y53.203 E0.02112
G1 X28.255 Y53.950 E0.02112
G1 X28.084 Y54.553 E0.01632
G1 X20.479 Y54.553 E0.19815
G1 X20.479 Y40.003 E0.37911
G1 X28.084 Y40.003 E0.19815
G1 X28.255 Y40.606 E0.01632
G1 X28.570 Y41.352 E0.02112
G1 X28.974 Y42.054 E0.02112
G1 X29.462 Y42.702 E0.02112
G1 X30.026 Y43.284 E0.02112
G1 X30.657 Y43.792 E0.02112
G1 X31.345 Y44.219 E0.02112
G1 X32.082 Y44.558 E0.02112
G1 X32.854 Y44.804 E0.02112
G1 X33.650 Y44.953 E0.02112
G1 X34.452 Y45.003 E0.02093
G1 X41.969 Y45.003 E0.19586
M204 S1250
G1 X41.829 Y45.349 F9000.000
G1 E-2.24000 F2400.000
;WIPE_START
G1 F7200.000
G1 X42.007 Y47.883 E-0.91200
;WIPE_END
G1 E-0.04800 F2400.000
```

```
G1 Z0.550 F9000.000
G1 X21.869 Y40.546
G1 Z0.350
G1 E3.20000 F1500.000
M204 S1000
;TYPE:Solid infill
;WIDTH:0.450839
G1 F4800.000
G1 X21.210 Y41.204 E0.02431
G1 X21.210 Y41.796 E0.01546
G1 X22.273 Y40.734 E0.03922
G1 X22.865 Y40.734 E0.01546
G1 X21.210 Y42.388 E0.06108
G1 X21.210 Y42.980 E0.01546
G1 X23.457 Y40.734 E0.08294
G1 X24.049 Y40.734 E0.01546
G1 X21.210 Y43.573 E0.10480
G1 X21.210 Y44.165 E0.01546
G1 X24.641 Y40.734 E0.12666
G1 X25.233 Y40.734 E0.01546
G1 X21.210 Y44.757 E0.14853
G1 X21.210 Y45.349 E0.01546
G1 X25.825 Y40.734 E0.17039
G1 X26.417 Y40.734 E0.01546
G1 X21.210 Y45.941 E0.19225
G1 X21.210 Y46.533 E0.01546
G1 X27.009 Y40.734 E0.21411
G1 X27.531 Y40.734 E0.01363
G1 X27.547 Y40.789 E0.00148
```

```
G1 X21.210 Y47.125 E0.23395
G1 X21.210 Y47.717 E0.01546
G1 X27.717 Y41.211 E0.24022
G1 X27.892 Y41.627 E0.01180
G1 X21.210 Y48.309 E0.24671
G1 X21.210 Y48.901 E0.01546
G1 X28.104 Y42.008 E0.25451
G1 X28.320 Y42.384 E0.01132
G1 X21.210 Y49.493 E0.26250
G1 X21.210 Y50.085 E0.01546
G1 X28.567 Y42.729 E0.27162
G1 X28.821 Y43.066 E0.01104
G1 X21.210 Y50.677 E0.28101
G1 X21.210 Y51.269 E0.01546
G1 X29.100 Y43.379 E0.29132
G1 X29.392 Y43.680 E0.01093
G1 X21.210 Y51.861 E0.30207
G1 X21.210 Y52.453 E0.01546
G1 X29.702 Y43.962 E0.31352
G1 X30.030 Y44.226 E0.01099
G1 X21.210 Y53.045 E0.32563
G1 X21.210 Y53.638 E0.01546
G1 X30.372 Y44.476 E0.33826
G1 X30.737 Y44.703 E0.01123
G1 X21.618 Y53.822 E0.33670
G1 X22.210 Y53.822 E0.01546
G1 X31.114 Y44.918 E0.32875
G1 X31.520 Y45.105 E0.01165
G1 X22.802 Y53.822 E0.32186
```

```
G1 X23.395 Y53.822 E0.01546
G1 X31.937 Y45.280 E0.31540
G1 X32.386 Y45.423 E0.01230
G1 X23.987 Y53.822 E0.31012
G1 X24.579 Y53.822 E0.01546
G1 X32.852 Y45.548 E0.30549
G1 X33.351 Y45.641 E0.01325
G1 X25.171 Y53.822 E0.30204
G1 X25.763 Y53.822 E0.01546
G1 X33.884 Y45.700 E0.29986
G1 X34.443 Y45.734 E0.01460
G1 X26.355 Y53.822 E0.29862
G1 X26.947 Y53.822 E0.01546
G1 X27.945 Y52.824 E0.03684
G1 X28.363 Y52.097 E0.02190
G1 X28.905 Y51.378 E0.02352
G1 X29.532 Y50.731 E0.02351
G1 X30.233 Y50.166 E0.02351
G1 X30.999 Y49.691 E0.02352
G1 X31.145 Y49.624 E0.00420
G1 X35.035 Y45.734 E0.14362
G1 X35.627 Y45.734 E0.01546
G1 X32.154 Y49.207 E0.12823
G1 X32.675 Y49.041 E0.01428
G1 X32.966 Y48.987 E0.00774
G1 X36.219 Y45.734 E0.12009
G1 X36.811 Y45.734 E0.01546
G1 X33.677 Y48.868 E0.11572
G1 X34.308 Y48.829 E0.01651
```

```
G1 X37.403 Y45.734 E0.11428
G1 X37.995 Y45.734 E0.01546
G1 X34.907 Y48.822 E0.11400
G1 X35.499 Y48.822 E0.01546
G1 X38.587 Y45.734 E0.11400
G1 X39.179 Y45.734 E0.01546
G1 X36.091 Y48.822 E0.11400
G1 X36.683 Y48.822 E0.01546
G1 X39.771 Y45.734 E0.11400
G1 X40.363 Y45.734 E0.01546
G1 X37.275 Y48.822 E0.11400
G1 X37.867 Y48.822 E0.01546
G1 X40.955 Y45.734 E0.11400
G1 X41.298 Y45.734 E0.00895
G1 X41.298 Y45.983 E0.00650
G1 X38.460 Y48.822 E0.10481
G1 X39.052 Y48.822 E0.01546
G1 X41.298 Y46.575 E0.08295
G1 X41.298 Y47.167 E0.01546
G1 X39.644 Y48.822 E0.06109
G1 X40.236 Y48.822 E0.01546
G1 X41.298 Y47.759 E0.03923
G1 X41.298 Y48.351 E0.01546
G1 X40.640 Y49.010 E0.02431
M204 S1250
; stop printing object tpu print.STL id:19 copy 0
; printing object Petg print.STL id:18 copy 0
; stop printing object Petg print.STL id:18 copy 0
; printing object Petg print.STL id:14 copy 0
```

```
; stop printing object Petg print.STL id:14 copy 0
; printing object tpu print.STL id:15 copy 0
G1 E-2.24000 F2400.000
;WIPE_START
G1 F7200.000
G1 X41.298 Y48.351 E-0.29484
G1 X41.298 Y47.759 E-0.18749
G1 X40.339 Y48.719 E-0.42968
;WIPE_END
G1 E-0.04800 F2400.000
G1 Z0.550 F9000.000
G1 X41.619 Y65.370
G1 Z0.350
G1 E3.20000 F1500.000
M204 S800
;TYPE:Perimeter
;WIDTH:0.45
G1 F2400.000
G1 X41.619 Y69.084 E0.09678
G1 X34.447 Y69.084 E0.18688
G1 X33.606 Y69.136 E0.02194
G1 X32.759 Y69.294 E0.02246
G1 X31.938 Y69.556 E0.02246
G1 X31.155 Y69.917 E0.02246
G1 X30.422 Y70.371 E0.02246
G1 X29.751 Y70.912 E0.02246
G1 X29.151 Y71.531 E0.02246
G1 X28.633 Y72.219 E0.02246
G1 X28.202 Y72.966 E0.02246
```

```
G1 X27.867 Y73.760 E0.02246
G1 X27.776 Y74.084 E0.00876
G1 X20.905 Y74.084 E0.17903
G1 X20.905 Y60.370 E0.35734
G1 X27.776 Y60.370 E0.17903
G1 X27.867 Y60.693 E0.00876
G1 X28.202 Y61.487 E0.02246
G1 X28.633 Y62.234 E0.02246
G1 X29.151 Y62.923 E0.02246
G1 X29.751 Y63.542 E0.02246
G1 X30.422 Y64.083 E0.02246
G1 X31.155 Y64.537 E0.02246
G1 X31.938 Y64.898 E0.02246
G1 X32.759 Y65.159 E0.02246
G1 X33.606 Y65.318 E0.02246
G1 X34.447 Y65.370 E0.02194
G1 X41.559 Y65.370 E0.18531
M204 S1250
G1 X42.037 Y64.952 F9000.000
M204 S800
;TYPE:External perimeter
G1 F1800.000
G1 X42.037 Y69.502 E0.11855
G1 X34.460 Y69.502 E0.19743
G1 X33.658 Y69.551 E0.02093
G1 X32.861 Y69.700 E0.02112
G1 X32.089 Y69.946 E0.02112
G1 X31.353 Y70.285 E0.02112
G1 X30.664 Y70.712 E0.02112
```

```
G1 X30.033 Y71.221 E0.02112
G1 X29.469 Y71.803 E0.02112
G1 X28.982 Y72.450 E0.02112
G1 X28.577 Y73.152 E0.02112
G1 X28.262 Y73.899 E0.02112
G1 X28.092 Y74.502 E0.01632
G1 X20.487 Y74.502 E0.19815
G1 X20.487 Y59.952 E0.37911
G1 X28.092 Y59.952 E0.19815
G1 X28.262 Y60.554 E0.01632
G1 X28.577 Y61.301 E0.02112
G1 X28.982 Y62.003 E0.02112
G1 X29.469 Y62.651 E0.02112
G1 X30.033 Y63.233 E0.02112
G1 X30.664 Y63.741 E0.02112
G1 X31.353 Y64.168 E0.02112
G1 X32.089 Y64.507 E0.02112
G1 X32.861 Y64.753 E0.02112
G1 X33.658 Y64.902 E0.02112
G1 X34.460 Y64.952 E0.02093
G1 X41.977 Y64.952 E0.19586
M204 S1250
G1 X41.836 Y65.298 F9000.000
G1 E-2.24000 F2400.000
;WIPE_START
G1 F7200.000
G1 X42.015 Y67.832 E-0.91200
;WIPE_END
G1 E-0.04800 F2400.000
```

```
G1 Z0.550 F9000.000
G1 X21.876 Y60.495
G1 Z0.350
G1 E3.20000 F1500.000
M204 S1000
;TYPE:Solid infill
;WIDTH:0.450839
G1 F4800.000
G1 X21.218 Y61.153 E0.02431
G1 X21.218 Y61.745 E0.01546
G1 X22.280 Y60.683 E0.03922
G1 X22.872 Y60.683 E0.01546
G1 X21.218 Y62.337 E0.06108
G1 X21.218 Y62.929 E0.01546
G1 X23.464 Y60.683 E0.08294
G1 X24.056 Y60.683 E0.01546
M73 P24 R52
G1 X21.218 Y63.521 E0.10480
G1 X21.218 Y64.114 E0.01546
G1 X24.649 Y60.683 E0.12666
G1 X25.241 Y60.683 E0.01546
G1 X21.218 Y64.706 E0.14853
G1 X21.218 Y65.298 E0.01546
G1 X25.833 Y60.683 E0.17039
G1 X26.425 Y60.683 E0.01546
G1 X21.218 Y65.890 E0.19225
G1 X21.218 Y66.482 E0.01546
G1 X27.017 Y60.683 E0.21411
G1 X27.539 Y60.683 E0.01363
```

G1 X27.554 Y60.738 E0.00148

G1 X21.218 Y67.074 E0.23395

G1 X21.218 Y67.666 E0.01546

G1 X27.724 Y61.160 E0.24022

G1 X27.900 Y61.576 E0.01180

G1 X21.218 Y68.258 E0.24671

G1 X21.218 Y68.850 E0.01546

G1 X28.111 Y61.957 E0.25451

G1 X28.328 Y62.332 E0.01132

G1 X21.218 Y69.442 E0.26250

G1 X21.218 Y70.034 E0.01546

G1 X28.574 Y62.678 E0.27162

G1 X28.829 Y63.015 E0.01104

G1 X21.218 Y70.626 E0.28101

G1 X21.218 Y71.218 E0.01546

G1 X29.108 Y63.328 E0.29132

G1 X29.399 Y63.629 E0.01093

G1 X21.218 Y71.810 E0.30207

G1 X21.218 Y72.402 E0.01546

G1 X29.709 Y63.911 E0.31352

G1 X30.037 Y64.175 E0.01099

G1 X21.218 Y72.994 E0.32563

G1 X21.218 Y73.586 E0.01546

G1 X30.379 Y64.425 E0.33826

G1 X30.745 Y64.652 E0.01123

G1 X21.626 Y73.771 E0.33670

G1 X22.218 Y73.771 E0.01546

G1 X31.122 Y64.867 E0.32875

G1 X31.527 Y65.053 E0.01165

```
G1 X22.810 Y73.771 E0.32186
G1 X23.402 Y73.771 E0.01546
G1 X31.944 Y65.229 E0.31540
G1 X32.393 Y65.372 E0.01230
G1 X23.994 Y73.771 E0.31012
G1 X24.586 Y73.771 E0.01546
G1 X32.860 Y65.497 E0.30549
G1 X33.359 Y65.590 E0.01325
G1 X25.178 Y73.771 E0.30204
G1 X25.770 Y73.771 E0.01546
G1 X33.892 Y65.649 E0.29986
G1 X34.450 Y65.683 E0.01460
G1 X26.362 Y73.771 E0.29862
G1 X26.954 Y73.771 E0.01546
G1 X27.952 Y72.773 E0.03684
G1 X28.371 Y72.046 E0.02190
G1 X28.913 Y71.327 E0.02352
G1 X29.539 Y70.680 E0.02351
G1 X30.241 Y70.115 E0.02351
G1 X31.006 Y69.640 E0.02352
G1 X31.152 Y69.573 E0.00420
G1 X35.042 Y65.683 E0.14362
G1 X35.634 Y65.683 E0.01546
G1 X32.161 Y69.156 E0.12823
G1 X32.682 Y68.990 E0.01428
G1 X32.974 Y68.935 E0.00774
G1 X36.226 Y65.683 E0.12009
G1 X36.818 Y65.683 E0.01546
G1 X33.684 Y68.817 E0.11572
```

G1 X34.315 Y68.778 E0.01651

G1 X37.410 Y65.683 E0.11428

G1 X38.002 Y65.683 E0.01546

G1 X34.915 Y68.771 E0.11400

G1 X35.507 Y68.771 E0.01546

G1 X38.594 Y65.683 E0.11400

G1 X39.186 Y65.683 E0.01546

G1 X36.099 Y68.771 E0.11400

G1 X36.691 Y68.771 E0.01546

G1 X39.779 Y65.683 E0.11400

G1 X40.371 Y65.683 E0.01546

G1 X37.283 Y68.771 E0.11400

G1 X37.875 Y68.771 E0.01546

G1 X40.963 Y65.683 E0.11400

G1 X41.306 Y65.683 E0.00895

G1 X41.306 Y65.932 E0.00650

G1 X38.467 Y68.771 E0.10481

G1 X39.059 Y68.771 E0.01546

G1 X41.306 Y66.524 E0.08295

G1 X41.306 Y67.116 E0.01546

G1 X39.651 Y68.771 E0.06109

G1 X40.243 Y68.771 E0.01546

G1 X41.306 Y67.708 E0.03923

G1 X41.306 Y68.300 E0.01546

G1 X40.647 Y68.959 E0.02431

M204 S1250

; stop printing object tpu print.STL id:15 copy 0

; printing object Petg print.STL id:10 copy 0

; stop printing object Petg print.STL id:10 copy 0

```
; printing object tpu print.STL id:11 copy 0
G1 E-2.24000 F2400.000
;WIPE_START
G1 F7200.000
G1 X41.306 Y68.300 E-0.29484
G1 X41.306 Y67.708 E-0.18749
G1 X40.346 Y68.668 E-0.42968
;WIPE_END
G1 E-0.04800 F2400.000
G1 Z0.550 F9000.000
G1 X41.568 Y87.562
G1 Z0.350
G1 E3.20000 F1500.000
M204 S800
;TYPE:Perimeter
;WIDTH:0.45
G1 F2400.000
G1 X41.568 Y91.276 E0.09678
G1 X34.396 Y91.276 E0.18688
G1 X33.555 Y91.328 E0.02194
G1 X32.708 Y91.487 E0.02246
G1 X31.887 Y91.748 E0.02246
G1 X31.104 Y92.109 E0.02246
G1 X30.371 Y92.563 E0.02246
G1 X29.700 Y93.104 E0.02246
G1 X29.100 Y93.723 E0.02246
G1 X28.581 Y94.412 E0.02246
G1 X28.151 Y95.159 E0.02246
G1 X27.816 Y95.953 E0.02246
```

```
G1 X27.725 Y96.276 E0.00876
G1 X20.853 Y96.276 E0.17903
G1 X20.853 Y82.562 E0.35734
G1 X27.725 Y82.562 E0.17903
G1 X27.816 Y82.885 E0.00876
G1 X28.151 Y83.680 E0.02246
G1 X28.581 Y84.427 E0.02246
G1 X29.100 Y85.115 E0.02246
G1 X29.700 Y85.734 E0.02246
G1 X30.371 Y86.275 E0.02246
G1 X31.104 Y86.729 E0.02246
G1 X31.887 Y87.090 E0.02246
G1 X32.708 Y87.351 E0.02246
G1 X33.555 Y87.510 E0.02246
G1 X34.396 Y87.562 E0.02194
G1 X41.508 Y87.562 E0.18531
M204 S1250
G1 X41.986 Y87.144 F9000.000
M204 S800
;TYPE:External perimeter
G1 F1800.000
G1 X41.986 Y91.694 E0.11855
G1 X34.409 Y91.694 E0.19743
G1 X33.607 Y91.744 E0.02093
G1 X32.810 Y91.893 E0.02112
G1 X32.038 Y92.139 E0.02112
G1 X31.302 Y92.478 E0.02112
G1 X30.613 Y92.905 E0.02112
G1 X29.982 Y93.413 E0.02112
```

```
G1 X29.418 Y93.995 E0.02112
G1 X28.931 Y94.643 E0.02112
G1 X28.526 Y95.345 E0.02112
G1 X28.211 Y96.091 E0.02112
G1 X28.040 Y96.694 E0.01632
G1 X20.436 Y96.694 E0.19815
G1 X20.436 Y82.144 E0.37911
G1 X28.040 Y82.144 E0.19815
G1 X28.211 Y82.747 E0.01632
G1 X28.526 Y83.493 E0.02112
G1 X28.931 Y84.196 E0.02112
G1 X29.418 Y84.843 E0.02112
G1 X29.982 Y85.425 E0.02112
G1 X30.613 Y85.934 E0.02112
G1 X31.302 Y86.361 E0.02112
G1 X32.038 Y86.700 E0.02112
G1 X32.810 Y86.946 E0.02112
G1 X33.607 Y87.095 E0.02112
G1 X34.409 Y87.144 E0.02093
G1 X41.926 Y87.144 E0.19586
M204 S1250
G1 X41.785 Y87.490 F9000.000
G1 E-2.24000 F2400.000
;WIPE_START
G1 F7200.000
G1 X41.964 Y90.024 E-0.91200
;WIPE_END
G1 E-0.04800 F2400.000
G1 Z0.550 F9000.000
```

```
G1 X21.825 Y82.687
G1 Z0.350
G1 E3.20000 F1500.000
M204 S1000
;TYPE:Solid infill
;WIDTH:0.450839
G1 F4800.000
G1 X21.167 Y83.346 E0.02431
G1 X21.167 Y83.938 E0.01546
G1 X22.229 Y82.875 E0.03922
G1 X22.821 Y82.875 E0.01546
G1 X21.167 Y84.530 E0.06108
G1 X21.167 Y85.122 E0.01546
G1 X23.413 Y82.875 E0.08294
G1 X24.005 Y82.875 E0.01546
G1 X21.167 Y85.714 E0.10480
G1 X21.167 Y86.306 E0.01546
G1 X24.597 Y82.875 E0.12666
G1 X25.189 Y82.875 E0.01546
G1 X21.167 Y86.898 E0.14853
G1 X21.167 Y87.490 E0.01546
G1 X25.781 Y82.875 E0.17039
G1 X26.374 Y82.875 E0.01546
G1 X21.167 Y88.082 E0.19225
G1 X21.167 Y88.674 E0.01546
G1 X26.966 Y82.875 E0.21411
G1 X27.488 Y82.875 E0.01363
G1 X27.503 Y82.930 E0.00148
G1 X21.167 Y89.266 E0.23395
```

G1 X21.167 Y89.858 E0.01546

G1 X27.673 Y83.352 E0.24022

G1 X27.849 Y83.768 E0.01180

G1 X21.167 Y90.450 E0.24671

G1 X21.167 Y91.042 E0.01546

G1 X28.060 Y84.149 E0.25451

G1 X28.276 Y84.525 E0.01132

G1 X21.167 Y91.634 E0.26250

G1 X21.167 Y92.226 E0.01546

G1 X28.523 Y84.870 E0.27162

G1 X28.778 Y85.208 E0.01104

G1 X21.167 Y92.819 E0.28101

G1 X21.167 Y93.411 E0.01546

G1 X29.057 Y85.521 E0.29132

G1 X29.348 Y85.821 E0.01093

G1 X21.167 Y94.003 E0.30207

G1 X21.167 Y94.595 E0.01546

G1 X29.658 Y86.103 E0.31352

G1 X29.986 Y86.367 E0.01099

G1 X21.167 Y95.187 E0.32563

G1 X21.167 Y95.779 E0.01546

G1 X30.328 Y86.617 E0.33826

G1 X30.694 Y86.844 E0.01123

G1 X21.575 Y95.963 E0.33670

G1 X22.167 Y95.963 E0.01546

G1 X31.071 Y87.059 E0.32875

G1 X31.476 Y87.246 E0.01165

G1 X22.759 Y95.963 E0.32186

G1 X23.351 Y95.963 E0.01546

```
G1 X31.893 Y87.421 E0.31540
G1 X32.342 Y87.564 E0.01230
G1 X23.943 Y95.963 E0.31012
G1 X24.535 Y95.963 E0.01546
G1 X32.809 Y87.689 E0.30549
G1 X33.308 Y87.783 E0.01325
G1 X25.127 Y95.963 E0.30204
G1 X25.719 Y95.963 E0.01546
G1 X33.840 Y87.842 E0.29986
G1 X34.399 Y87.875 E0.01460
G1 X26.311 Y95.963 E0.29862
G1 X26.903 Y95.963 E0.01546
G1 X27.901 Y94.965 E0.03684
G1 X28.320 Y94.239 E0.02190
G1 X28.862 Y93.519 E0.02352
G1 X29.488 Y92.872 E0.02351
G1 X30.189 Y92.307 E0.02351
G1 X30.955 Y91.832 E0.02352
G1 X31.101 Y91.765 E0.00420
G1 X34.991 Y87.875 E0.14362
G1 X35.583 Y87.875 E0.01546
G1 X32.110 Y91.348 E0.12823
G1 X32.631 Y91.182 E0.01428
G1 X32.923 Y91.128 E0.00774
G1 X36.175 Y87.875 E0.12009
G1 X36.767 Y87.875 E0.01546
G1 X33.633 Y91.009 E0.11572
G1 X34.264 Y90.970 E0.01651
G1 X37.359 Y87.875 E0.11428
```

```
G1 X37.951 Y87.875 E0.01546
G1 X34.864 Y90.963 E0.11400
G1 X35.456 Y90.963 E0.01546
G1 X38.543 Y87.875 E0.11400
G1 X39.135 Y87.875 E0.01546
G1 X36.048 Y90.963 E0.11400
G1 X36.640 Y90.963 E0.01546
G1 X39.727 Y87.875 E0.11400
G1 X40.319 Y87.875 E0.01546
G1 X37.232 Y90.963 E0.11400
G1 X37.824 Y90.963 E0.01546
G1 X40.911 Y87.875 E0.11400
G1 X41.254 Y87.875 E0.00895
G1 X41.254 Y88.124 E0.00650
G1 X38.416 Y90.963 E0.10481
M73 P24 R51
G1 X39.008 Y90.963 E0.01546
G1 X41.254 Y88.716 E0.08295
G1 X41.254 Y89.308 E0.01546
G1 X39.600 Y90.963 E0.06109
G1 X40.192 Y90.963 E0.01546
G1 X41.254 Y89.901 E0.03923
G1 X41.254 Y90.493 E0.01546
G1 X40.596 Y91.151 E0.02431
M204 S1250
; stop printing object tpu print.STL id:11 copy 0
; printing object tpu print.STL id:13 copy 0
G1 E-2.24000 F2400.000
;WIPE_START
```

```
G1 F7200.000
G1 X41.254 Y90.493 E-0.29484
G1 X41.254 Y89.901 E-0.18749
G1 X40.295 Y90.860 E-0.42968
;WIPE_END
G1 E-0.04800 F2400.000
G1 Z0.550 F9000.000
G1 X41.575 Y108.377
G1 Z0.350
G1 E3.20000 F1500.000
M204 S800
;TYPE:Perimeter
;WIDTH:0.45
G1 F2400.000
G1 X41.575 Y112.092 E0.09678
G1 X34.402 Y112.092 E0.18688
G1 X33.562 Y112.144 E0.02194
G1 X32.715 Y112.302 E0.02246
G1 X31.893 Y112.564 E0.02246
G1 X31.110 Y112.924 E0.02246
G1 X30.378 Y113.379 E0.02246
G1 X29.707 Y113.920 E0.02246
G1 X29.107 Y114.539 E0.02246
G1 X28.588 Y115.227 E0.02246
G1 X28.158 Y115.974 E0.02246
G1 X27.823 Y116.768 E0.02246
G1 X27.731 Y117.092 E0.00876
G1 X20.860 Y117.092 E0.17903
G1 X20.860 Y103.377 E0.35734
```

```
G1 X27.731 Y103.377 E0.17903
G1 X27.823 Y103.701 E0.00876
G1 X28.158 Y104.495 E0.02246
G1 X28.588 Y105.242 E0.02246
G1 X29.107 Y105.930 E0.02246
G1 X29.707 Y106.550 E0.02246
G1 X30.378 Y107.091 E0.02246
G1 X31.110 Y107.545 E0.02246
G1 X31.893 Y107.905 E0.02246
G1 X32.715 Y108.167 E0.02246
G1 X33.562 Y108.326 E0.02246
G1 X34.402 Y108.377 E0.02194
G1 X41.515 Y108.377 E0.18531
M204 S1250
G1 X41.992 Y107.960 F9000.000
M204 S800
;TYPE:External perimeter
G1 F1800.000
G1 X41.992 Y112.510 E0.11855
G1 X34.415 Y112.510 E0.19743
G1 X33.613 Y112.559 E0.02093
G1 X32.817 Y112.708 E0.02112
G1 X32.045 Y112.954 E0.02112
G1 X31.309 Y113.293 E0.02112
G1 X30.620 Y113.720 E0.02112
G1 X29.989 Y114.229 E0.02112
G1 X29.425 Y114.811 E0.02112
G1 X28.937 Y115.458 E0.02112
G1 X28.533 Y116.160 E0.02112
```

```
G1 X28.218 Y116.907 E0.02112
G1 X28.047 Y117.510 E0.01632
G1 X20.442 Y117.510 E0.19815
G1 X20.442 Y102.960 E0.37911
G1 X28.047 Y102.960 E0.19815
G1 X28.218 Y103.562 E0.01632
G1 X28.533 Y104.309 E0.02112
G1 X28.937 Y105.011 E0.02112
G1 X29.425 Y105.658 E0.02112
G1 X29.989 Y106.241 E0.02112
G1 X30.620 Y106.749 E0.02112
G1 X31.309 Y107.176 E0.02112
G1 X32.045 Y107.515 E0.02112
G1 X32.817 Y107.761 E0.02112
G1 X33.613 Y107.910 E0.02112
G1 X34.415 Y107.960 E0.02093
G1 X41.932 Y107.960 E0.19586
M204 S1250
G1 X41.792 Y108.306 F9000.000
G1 E-2.24000 F2400.000
;WIPE_START
G1 F7200.000
G1 X41.970 Y110.839 E-0.91200
;WIPE_END
G1 E-0.04800 F2400.000
G1 Z0.550 F9000.000
G1 X21.832 Y103.503
G1 Z0.350
G1 E3.20000 F1500.000
```

```
M204 S1000
;TYPE:Solid infill
;WIDTH:0.450839
G1 F4800.000
G1 X21.174 Y104.161 E0.02431
G1 X21.174 Y104.753 E0.01546
G1 X22.236 Y103.691 E0.03922
G1 X22.828 Y103.691 E0.01546
G1 X21.174 Y105.345 E0.06108
G1 X21.174 Y105.937 E0.01546
G1 X23.420 Y103.691 E0.08294
G1 X24.012 Y103.691 E0.01546
G1 X21.174 Y106.529 E0.10480
G1 X21.174 Y107.121 E0.01546
G1 X24.604 Y103.691 E0.12666
G1 X25.196 Y103.691 E0.01546
G1 X21.174 Y107.713 E0.14853
G1 X21.174 Y108.306 E0.01546
G1 X25.788 Y103.691 E0.17039
G1 X26.380 Y103.691 E0.01546
G1 X21.174 Y108.898 E0.19225
G1 X21.174 Y109.490 E0.01546
G1 X26.972 Y103.691 E0.21411
G1 X27.494 Y103.691 E0.01363
G1 X27.510 Y103.745 E0.00148
G1 X21.174 Y110.082 E0.23395
G1 X21.174 Y110.674 E0.01546
G1 X27.680 Y104.168 E0.24022
G1 X27.855 Y104.584 E0.01180
```

```
G1 X21.174 Y111.266 E0.24671
G1 X21.174 Y111.858 E0.01546
G1 X28.067 Y104.965 E0.25451
G1 X28.283 Y105.340 E0.01132
G1 X21.174 Y112.450 E0.26250
G1 X21.174 Y113.042 E0.01546
G1 X28.530 Y105.686 E0.27162
G1 X28.785 Y106.023 E0.01104
G1 X21.174 Y113.634 E0.28101
G1 X21.174 Y114.226 E0.01546
G1 X29.064 Y106.336 E0.29132
G1 X29.355 Y106.637 E0.01093
G1 X21.174 Y114.818 E0.30207
G1 X21.174 Y115.410 E0.01546
G1 X29.665 Y106.919 E0.31352
G1 X29.993 Y107.183 E0.01099
G1 X21.174 Y116.002 E0.32563
G1 X21.174 Y116.594 E0.01546
G1 X30.335 Y107.433 E0.33826
G1 X30.701 Y107.659 E0.01123
G1 X21.582 Y116.778 E0.33670
G1 X22.174 Y116.778 E0.01546
G1 X31.077 Y107.875 E0.32875
G1 X31.483 Y108.061 E0.01165
G1 X22.766 Y116.778 E0.32186
G1 X23.358 Y116.778 E0.01546
G1 X31.900 Y108.236 E0.31540
G1 X32.349 Y108.379 E0.01230
G1 X23.950 Y116.778 E0.31012
```

G1 X24.542 Y116.778 E0.01546

G1 X32.816 Y108.505 E0.30549

G1 X33.314 Y108.598 E0.01325

G1 X25.134 Y116.778 E0.30204

G1 X25.726 Y116.778 E0.01546

G1 X33.847 Y108.657 E0.29986

G1 X34.406 Y108.691 E0.01460

G1 X26.318 Y116.778 E0.29862

G1 X26.910 Y116.778 E0.01546

G1 X27.908 Y115.781 E0.03684

G1 X28.326 Y115.054 E0.02190

G1 X28.868 Y114.335 E0.02352

G1 X29.495 Y113.688 E0.02351

G1 X30.196 Y113.123 E0.02351

G1 X30.962 Y112.648 E0.02352

G1 X31.108 Y112.581 E0.00420

G1 X34.998 Y108.691 E0.14362

G1 X35.590 Y108.691 E0.01546

G1 X32.117 Y112.164 E0.12823

G1 X32.638 Y111.998 E0.01428

G1 X32.929 Y111.943 E0.00774

G1 X36.182 Y108.691 E0.12009

G1 X36.774 Y108.691 E0.01546

G1 X33.640 Y111.825 E0.11572

G1 X34.271 Y111.786 E0.01651

G1 X37.366 Y108.691 E0.11428

G1 X37.958 Y108.691 E0.01546

G1 X34.870 Y111.778 E0.11400

G1 X35.462 Y111.778 E0.01546

```
G1 X38.550 Y108.691 E0.11400
G1 X39.142 Y108.691 E0.01546
G1 X36.054 Y111.778 E0.11400
G1 X36.647 Y111.778 E0.01546
G1 X39.734 Y108.691 E0.11400
G1 X40.326 Y108.691 E0.01546
G1 X37.239 Y111.778 E0.11400
G1 X37.831 Y111.778 E0.01546
G1 X40.918 Y108.691 E0.11400
G1 X41.261 Y108.691 E0.00895
G1 X41.261 Y108.940 E0.00650
G1 X38.423 Y111.778 E0.10481
G1 X39.015 Y111.778 E0.01546
G1 X41.261 Y109.532 E0.08295
G1 X41.261 Y110.124 E0.01546
G1 X39.607 Y111.778 E0.06109
G1 X40.199 Y111.778 E0.01546
G1 X41.261 Y110.716 E0.03923
G1 X41.261 Y111.308 E0.01546
G1 X40.603 Y111.967 E0.02431
M204 S1250
; stop printing object tpu print.STL id:13 copy 0
; printing object tpu print.STL id:17 copy 0
G1 E-2.24000 F2400.000
;WIPE_START
G1 F7200.000
G1 X41.261 Y111.308 E-0.29484
G1 X41.261 Y110.716 E-0.18749
G1 X40.302 Y111.676 E-0.42968
```

```
;WIPE_END
G1 E-0.04800 F2400.000
G1 Z0.550 F9000.000
G1 X41.560 Y127.031
G1 Z0.350
G1 E3.20000 F1500.000
M204 S800
;TYPE:Perimeter
;WIDTH:0.45
G1 F2400.000
G1 X41.560 Y130.746 E0.09678
G1 X34.387 Y130.746 E0.18688
G1 X33.547 Y130.798 E0.02194
G1 X32.700 Y130.956 E0.02246
G1 X31.878 Y131.218 E0.02246
G1 X31.095 Y131.578 E0.02246
G1 X30.363 Y132.033 E0.02246
G1 X29.692 Y132.573 E0.02246
G1 X29.092 Y133.193 E0.02246
G1 X28.573 Y133.881 E0.02246
G1 X28.143 Y134.628 E0.02246
G1 X27.808 Y135.422 E0.02246
G1 X27.716 Y135.746 E0.00876
G1 X20.845 Y135.746 E0.17903
G1 X20.845 Y122.031 E0.35734
G1 X27.716 Y122.031 E0.17903
G1 X27.808 Y122.355 E0.00876
G1 X28.143 Y123.149 E0.02246
G1 X28.573 Y123.896 E0.02246
```

```
G1 X29.092 Y124.584 E0.02246
G1 X29.692 Y125.204 E0.02246
G1 X30.363 Y125.745 E0.02246
G1 X31.095 Y126.199 E0.02246
G1 X31.878 Y126.559 E0.02246
G1 X32.700 Y126.821 E0.02246
G1 X33.547 Y126.980 E0.02246
G1 X34.387 Y127.031 E0.02194
G1 X41.500 Y127.031 E0.18531
M204 S1250
G1 X41.977 Y126.614 F9000.000
M204 S800
;TYPE:External perimeter
G1 F1800.000
G1 X41.977 Y131.164 E0.11855
G1 X34.400 Y131.164 E0.19743
G1 X33.598 Y131.213 E0.02093
G1 X32.802 Y131.362 E0.02112
G1 X32.030 Y131.608 E0.02112
G1 X31.294 Y131.947 E0.02112
G1 X30.605 Y132.374 E0.02112
G1 X29.974 Y132.883 E0.02112
G1 X29.410 Y133.465 E0.02112
G1 X28.922 Y134.112 E0.02112
G1 X28.518 Y134.814 E0.02112
G1 X28.203 Y135.561 E0.02112
G1 X28.032 Y136.164 E0.01632
G1 X20.427 Y136.164 E0.19815
G1 X20.427 Y121.614 E0.37911
```

```
G1 X28.032 Y121.614 E0.19815
G1 X28.203 Y122.216 E0.01632
G1 X28.518 Y122.963 E0.02112
G1 X28.922 Y123.665 E0.02112
G1 X29.410 Y124.312 E0.02112
G1 X29.974 Y124.895 E0.02112
G1 X30.605 Y125.403 E0.02112
G1 X31.294 Y125.830 E0.02112
G1 X32.030 Y126.169 E0.02112
G1 X32.802 Y126.415 E0.02112
G1 X33.598 Y126.564 E0.02112
G1 X34.400 Y126.614 E0.02093
G1 X41.917 Y126.614 E0.19586
M204 S1250
M73 P25 R51
G1 X41.777 Y126.960 F9000.000
G1 E-2.24000 F2400.000
;WIPE_START
G1 F7200.000
G1 X41.955 Y129.493 E-0.91200
;WIPE_END
G1 E-0.04800 F2400.000
G1 Z0.550 F9000.000
G1 X21.817 Y122.157
G1 Z0.350
G1 E3.20000 F1500.000
M204 S1000
;TYPE:Solid infill
;WIDTH:0.450839
```

```
G1 F4800.000
G1 X21.159 Y122.815 E0.02431
G1 X21.159 Y123.407 E0.01546
G1 X22.221 Y122.345 E0.03922
G1 X22.813 Y122.345 E0.01546
G1 X21.159 Y123.999 E0.06108
G1 X21.159 Y124.591 E0.01546
G1 X23.405 Y122.345 E0.08294
G1 X23.997 Y122.345 E0.01546
G1 X21.159 Y125.183 E0.10480
G1 X21.159 Y125.775 E0.01546
G1 X24.589 Y122.345 E0.12666
G1 X25.181 Y122.345 E0.01546
G1 X21.159 Y126.367 E0.14853
G1 X21.159 Y126.959 E0.01546
G1 X25.773 Y122.345 E0.17039
G1 X26.365 Y122.345 E0.01546
G1 X21.159 Y127.552 E0.19225
G1 X21.159 Y128.144 E0.01546
G1 X26.957 Y122.345 E0.21411
G1 X27.479 Y122.345 E0.01363
G1 X27.495 Y122.399 E0.00148
G1 X21.159 Y128.736 E0.23395
G1 X21.159 Y129.328 E0.01546
G1 X27.665 Y122.822 E0.24022
G1 X27.840 Y123.238 E0.01180
G1 X21.159 Y129.920 E0.24671
G1 X21.159 Y130.512 E0.01546
G1 X28.052 Y123.619 E0.25451
```

```
G1 X28.268 Y123.994 E0.01132
G1 X21.159 Y131.104 E0.26250
G1 X21.159 Y131.696 E0.01546
G1 X28.515 Y124.340 E0.27162
G1 X28.769 Y124.677 E0.01104
G1 X21.159 Y132.288 E0.28101
G1 X21.159 Y132.880 E0.01546
G1 X29.049 Y124.990 E0.29132
G1 X29.340 Y125.291 E0.01093
G1 X21.159 Y133.472 E0.30207
G1 X21.159 Y134.064 E0.01546
G1 X29.650 Y125.573 E0.31352
G1 X29.978 Y125.837 E0.01099
G1 X21.159 Y134.656 E0.32563
G1 X21.159 Y135.248 E0.01546
G1 X30.320 Y126.087 E0.33826
G1 X30.685 Y126.313 E0.01123
G1 X21.566 Y135.432 E0.33670
G1 X22.158 Y135.432 E0.01546
G1 X31.062 Y126.529 E0.32875
G1 X31.468 Y126.715 E0.01165
G1 X22.751 Y135.432 E0.32186
G1 X23.343 Y135.432 E0.01546
G1 X31.885 Y126.890 E0.31540
G1 X32.334 Y127.033 E0.01230
G1 X23.935 Y135.432 E0.31012
G1 X24.527 Y135.432 E0.01546
G1 X32.801 Y127.159 E0.30549
G1 X33.299 Y127.252 E0.01325
```

```
G1 X25.119 Y135.432 E0.30204
G1 X25.711 Y135.432 E0.01546
G1 X33.832 Y127.311 E0.29986
G1 X34.391 Y127.345 E0.01460
G1 X26.303 Y135.432 E0.29862
G1 X26.895 Y135.432 E0.01546
G1 X27.893 Y134.435 E0.03684
G1 X28.311 Y133.708 E0.02190
G1 X28.853 Y132.989 E0.02352
G1 X29.480 Y132.342 E0.02351
G1 X30.181 Y131.776 E0.02351
G1 X30.947 Y131.302 E0.02352
G1 X31.093 Y131.235 E0.00420
G1 X34.983 Y127.345 E0.14362
G1 X35.575 Y127.345 E0.01546
G1 X32.102 Y130.818 E0.12823
G1 X32.623 Y130.652 E0.01428
G1 X32.914 Y130.597 E0.00774
G1 X36.167 Y127.345 E0.12009
G1 X36.759 Y127.345 E0.01546
G1 X33.625 Y130.479 E0.11572
G1 X34.256 Y130.440 E0.01651
G1 X37.351 Y127.345 E0.11428
G1 X37.943 Y127.345 E0.01546
G1 X34.855 Y130.432 E0.11400
G1 X35.447 Y130.432 E0.01546
G1 X38.535 Y127.345 E0.11400
G1 X39.127 Y127.345 E0.01546
G1 X36.039 Y130.432 E0.11400
```

```
G1 X36.631 Y130.432 E0.01546
G1 X39.719 Y127.345 E0.11400
G1 X40.311 Y127.345 E0.01546
G1 X37.223 Y130.432 E0.11400
G1 X37.816 Y130.432 E0.01546
G1 X40.903 Y127.345 E0.11400
G1 X41.246 Y127.345 E0.00895
G1 X41.246 Y127.594 E0.00650
G1 X38.408 Y130.432 E0.10481
G1 X39.000 Y130.432 E0.01546
G1 X41.246 Y128.186 E0.08295
G1 X41.246 Y128.778 E0.01546
G1 X39.592 Y130.432 E0.06109
G1 X40.184 Y130.432 E0.01546
G1 X41.246 Y129.370 E0.03923
G1 X41.246 Y129.962 E0.01546
G1 X40.588 Y130.620 E0.02431
M204 S1250
; stop printing object tpu print.STL id:17 copy 0
; printing object Petg print.STL id:16 copy 0
; stop printing object Petg print.STL id:16 copy 0
; printing object Petg print.STL id:12 copy 0
; stop printing object Petg print.STL id:12 copy 0
; printing object tpu print.STL id:7 copy 0
G1 E-2.24000 F2400.000
;WIPE_START
G1 F7200.000
G1 X41.246 Y129.962 E-0.29484
G1 X41.246 Y129.370 E-0.18749
```

```
G1 X40.287 Y130.329 E-0.42968
;WIPE_END
G1 E-0.04800 F2400.000
G1 Z0.550 F9000.000
G1 X69.635 Y136.327
G1 Z0.350
G1 E3.20000 F1500.000
M204 S800
;TYPE:Perimeter
;WIDTH:0.45
G1 F2400.000
G1 X69.635 Y122.612 E0.35734
G1 X76.506 Y122.612 E0.17903
G1 X76.597 Y122.936 E0.00876
G1 X76.932 Y123.730 E0.02246
G1 X77.362 Y124.477 E0.02246
G1 X77.881 Y125.165 E0.02246
G1 X78.481 Y125.785 E0.02246
G1 X79.152 Y126.325 E0.02246
G1 X79.885 Y126.780 E0.02246
G1 X80.668 Y127.140 E0.02246
G1 X81.489 Y127.402 E0.02246
G1 X82.336 Y127.560 E0.02246
G1 X83.177 Y127.612 E0.02194
G1 X90.349 Y127.612 E0.18688
G1 X90.349 Y131.327 E0.09678
G1 X83.177 Y131.327 E0.18688
G1 X82.336 Y131.379 E0.02194
G1 X81.489 Y131.537 E0.02246
```

```
G1 X80.668 Y131.799 E0.02246
G1 X79.885 Y132.159 E0.02246
G1 X79.152 Y132.613 E0.02246
G1 X78.481 Y133.154 E0.02246
G1 X77.881 Y133.774 E0.02246
G1 X77.362 Y134.462 E0.02246
G1 X76.932 Y135.209 E0.02246
G1 X76.597 Y136.003 E0.02246
G1 X76.506 Y136.327 E0.00876
G1 X69.695 Y136.327 E0.17747
M204 S1250
G1 X69.217 Y136.744 F9000.000
M204 S800
;TYPE:External perimeter
G1 F1800.000
G1 X69.217 Y122.194 E0.37911
G1 X76.822 Y122.194 E0.19815
G1 X76.992 Y122.797 E0.01632
G1 X77.307 Y123.544 E0.02112
G1 X77.712 Y124.246 E0.02112
G1 X78.199 Y124.893 E0.02112
G1 X78.763 Y125.475 E0.02112
G1 X79.394 Y125.984 E0.02112
G1 X80.083 Y126.411 E0.02112
G1 X80.819 Y126.750 E0.02112
G1 X81.591 Y126.996 E0.02112
G1 X82.388 Y127.145 E0.02112
G1 X83.190 Y127.194 E0.02093
G1 X90.767 Y127.194 E0.19743
```

```
G1 X90.767 Y131.744 E0.11855
G1 X83.190 Y131.744 E0.19743
G1 X82.388 Y131.794 E0.02093
G1 X81.591 Y131.943 E0.02112
G1 X80.819 Y132.189 E0.02112
G1 X80.083 Y132.528 E0.02112
G1 X79.394 Y132.955 E0.02112
G1 X78.763 Y133.463 E0.02112
G1 X78.199 Y134.046 E0.02112
G1 X77.712 Y134.693 E0.02112
G1 X77.307 Y135.395 E0.02112
G1 X76.992 Y136.142 E0.02112
G1 X76.822 Y136.744 E0.01632
G1 X69.277 Y136.744 E0.19659
M204 S1250
G1 X69.408 Y136.393 F9000.000
G1 E-2.24000 F2400.000
;WIPE_START
G1 F7200.000
G1 X69.265 Y133.864 E-0.91200
;WIPE_END
G1 E-0.04800 F2400.000
G1 Z0.550 F9000.000
G1 X89.377 Y131.201
G1 Z0.350
G1 E3.20000 F1500.000
M204 S1000
;TYPE:Solid infill
;WIDTH:0.450839
```

```
G1 F4800.000
G1 X90.036 Y130.543 E0.02431
G1 X90.036 Y129.951 E0.01546
G1 X88.973 Y131.013 E0.03923
G1 X88.381 Y131.013 E0.01546
G1 X90.036 Y129.359 E0.06109
G1 X90.036 Y128.767 E0.01546
G1 X87.789 Y131.013 E0.08295
G1 X87.197 Y131.013 E0.01546
G1 X90.036 Y128.175 E0.10481
G1 X90.036 Y127.926 E0.00650
G1 X89.693 Y127.926 E0.00895
G1 X86.605 Y131.013 E0.11400
G1 X86.013 Y131.013 E0.01546
G1 X89.101 Y127.926 E0.11400
G1 X88.508 Y127.926 E0.01546
G1 X85.421 Y131.013 E0.11400
G1 X84.829 Y131.013 E0.01546
G1 X87.916 Y127.926 E0.11400
G1 X87.324 Y127.926 E0.01546
G1 X84.237 Y131.013 E0.11400
G1 X83.645 Y131.013 E0.01546
G1 X86.732 Y127.926 E0.11400
G1 X86.140 Y127.926 E0.01546
G1 X83.045 Y131.021 E0.11428
G1 X82.414 Y131.060 E0.01651
G1 X85.548 Y127.926 E0.11572
G1 X84.956 Y127.926 E0.01546
G1 X81.704 Y131.178 E0.12009
```

```
G1 X81.412 Y131.233 E0.00774
G1 X80.891 Y131.399 E0.01428
G1 X84.364 Y127.926 E0.12823
G1 X83.772 Y127.926 E0.01546
G1 X79.882 Y131.815 E0.14362
G1 X79.736 Y131.883 E0.00420
G1 X78.970 Y132.357 E0.02352
G1 X78.269 Y132.922 E0.02351
G1 X77.643 Y133.570 E0.02351
G1 X77.101 Y134.289 E0.02352
G1 X76.682 Y135.016 E0.02190
G1 X75.684 Y136.013 E0.03684
G1 X75.092 Y136.013 E0.01546
G1 X83.180 Y127.926 E0.29862
G1 X82.622 Y127.892 E0.01460
G1 X74.500 Y136.013 E0.29986
G1 X73.908 Y136.013 E0.01546
G1 X82.089 Y127.833 E0.30204
G1 X81.590 Y127.740 E0.01325
G1 X73.316 Y136.013 E0.30549
G1 X72.724 Y136.013 E0.01546
G1 X81.123 Y127.614 E0.31012
G1 X80.674 Y127.471 E0.01230
G1 X72.132 Y136.013 E0.31540
G1 X71.540 Y136.013 E0.01546
G1 X80.257 Y127.296 E0.32186
G1 X79.852 Y127.109 E0.01165
G1 X70.948 Y136.013 E0.32875
G1 X70.356 Y136.013 E0.01546
```

```
G1 X79.475 Y126.894 E0.33670
G1 X79.109 Y126.668 E0.01123
G1 X69.948 Y135.829 E0.33826
G1 X69.948 Y135.237 E0.01546
G1 X78.767 Y126.418 E0.32563
G1 X78.439 Y126.154 E0.01099
G1 X69.948 Y134.645 E0.31352
G1 X69.948 Y134.053 E0.01546
G1 X78.129 Y125.872 E0.30207
G1 X77.838 Y125.571 E0.01093
G1 X69.948 Y133.461 E0.29132
G1 X69.948 Y132.869 E0.01546
G1 X77.559 Y125.258 E0.28101
G1 X77.304 Y124.920 E0.01104
G1 X69.948 Y132.277 E0.27162
G1 X69.948 Y131.685 E0.01546
G1 X77.057 Y124.575 E0.26250
G1 X76.841 Y124.199 E0.01132
G1 X69.948 Y131.093 E0.25451
G1 X69.948 Y130.501 E0.01546
G1 X76.630 Y123.819 E0.24671
G1 X76.454 Y123.402 E0.01180
G1 X69.948 Y129.909 E0.24022
G1 X69.948 Y129.316 E0.01546
G1 X76.284 Y122.980 E0.23395
G1 X76.269 Y122.926 E0.00148
G1 X75.747 Y122.926 E0.01363
G1 X69.948 Y128.724 E0.21411
G1 X69.948 Y128.132 E0.01546
```

```
G1 X75.155 Y122.926 E0.19225
G1 X74.563 Y122.926 E0.01546
G1 X69.948 Y127.540 E0.17039
G1 X69.948 Y126.948 E0.01546
G1 X73.971 Y122.926 E0.14853
G1 X73.378 Y122.926 E0.01546
G1 X69.948 Y126.356 E0.12666
G1 X69.948 Y125.764 E0.01546
G1 X72.786 Y122.926 E0.10480
G1 X72.194 Y122.926 E0.01546
G1 X69.948 Y125.172 E0.08294
G1 X69.948 Y124.580 E0.01546
G1 X71.602 Y122.926 E0.06108
G1 X71.010 Y122.926 E0.01546
G1 X69.948 Y123.988 E0.03922
G1 X69.948 Y123.396 E0.01546
G1 X70.606 Y122.738 E0.02431
M204 S1250
; stop printing object tpu print.STL id:7 copy 0
; printing object tpu print.STL id:3 copy 0
G1 E-2.24000 F2400.000
;WIPE_START
G1 F7200.000
G1 X69.948 Y123.396 E-0.29480
G1 X69.948 Y123.988 E-0.18749
G1 X70.907 Y123.028 E-0.42971
;WIPE_END
G1 E-0.04800 F2400.000
G1 Z0.550 F9000.000
```

```
G1 X69.650 Y117.673
G1 Z0.350
G1 E3.20000 F1500.000
M204 S800
;TYPE:Perimeter
;WIDTH:0.45
G1 F2400.000
G1 X69.650 Y103.958 E0.35734
G1 X76.521 Y103.958 E0.17903
G1 X76.612 Y104.282 E0.00876
G1 X76.947 Y105.076 E0.02246
G1 X77.378 Y105.823 E0.02246
G1 X77.896 Y106.511 E0.02246
G1 X78.496 Y107.131 E0.02246
G1 X79.167 Y107.672 E0.02246
G1 X79.900 Y108.126 E0.02246
G1 X80.683 Y108.486 E0.02246
G1 X81.504 Y108.748 E0.02246
G1 X82.351 Y108.906 E0.02246
G1 X83.192 Y108.958 E0.02194
G1 X90.364 Y108.958 E0.18688
G1 X90.364 Y112.673 E0.09678
G1 X83.192 Y112.673 E0.18688
G1 X82.351 Y112.725 E0.02194
G1 X81.504 Y112.883 E0.02246
G1 X80.683 Y113.145 E0.02246
G1 X79.900 Y113.505 E0.02246
G1 X79.167 Y113.960 E0.02246
G1 X78.496 Y114.500 E0.02246
```

```
G1 X77.896 Y115.120 E0.02246
G1 X77.378 Y115.808 E0.02246
G1 X76.947 Y116.555 E0.02246
G1 X76.612 Y117.349 E0.02246
G1 X76.521 Y117.673 E0.00876
G1 X69.710 Y117.673 E0.17747
M204 S1250
G1 X69.232 Y118.091 F9000.000
M204 S800
;TYPE:External perimeter
G1 F1800.000
G1 X69.232 Y103.541 E0.37911
G1 X76.837 Y103.541 E0.19815
G1 X77.007 Y104.143 E0.01632
G1 X77.322 Y104.890 E0.02112
G1 X77.727 Y105.592 E0.02112
G1 X78.215 Y106.239 E0.02112
G1 X78.778 Y106.822 E0.02112
G1 X79.409 Y107.330 E0.02112
G1 X80.098 Y107.757 E0.02112
G1 X80.834 Y108.096 E0.02112
G1 X81.606 Y108.342 E0.02112
G1 X82.403 Y108.491 E0.02112
G1 X83.205 Y108.541 E0.02093
G1 X90.782 Y108.541 E0.19743
G1 X90.782 Y113.091 E0.11855
G1 X83.205 Y113.091 E0.19743
G1 X82.403 Y113.140 E0.02093
G1 X81.606 Y113.289 E0.02112
```

```
G1 X80.834 Y113.535 E0.02112
G1 X80.098 Y113.874 E0.02112
G1 X79.409 Y114.301 E0.02112
G1 X78.778 Y114.810 E0.02112
G1 X78.215 Y115.392 E0.02112
G1 X77.727 Y116.039 E0.02112
G1 X77.322 Y116.741 E0.02112
G1 X77.007 Y117.488 E0.02112
G1 X76.837 Y118.091 E0.01632
G1 X69.292 Y118.091 E0.19659
M204 S1250
G1 X69.423 Y117.739 F9000.000
G1 E-2.24000 F2400.000
;WIPE_START
G1 F7200.000
G1 X69.280 Y115.211 E-0.91200
;WIPE_END
G1 E-0.04800 F2400.000
G1 Z0.550 F9000.000
G1 X89.392 Y112.547
G1 Z0.350
G1 E3.20000 F1500.000
M204 S1000
;TYPE:Solid infill
;WIDTH:0.450839
G1 F4800.000
G1 X90.051 Y111.889 E0.02431
G1 X90.051 Y111.297 E0.01546
G1 X88.988 Y112.359 E0.03923
```

G1 X88.396 Y112.359 E0.01546

G1 X90.051 Y110.705 E0.06109

G1 X90.051 Y110.113 E0.01546

G1 X87.804 Y112.359 E0.08295

G1 X87.212 Y112.359 E0.01546

G1 X90.051 Y109.521 E0.10481

G1 X90.051 Y109.272 E0.00650

G1 X89.708 Y109.272 E0.00895

G1 X86.620 Y112.359 E0.11400

G1 X86.028 Y112.359 E0.01546

G1 X89.116 Y109.272 E0.11400

G1 X88.524 Y109.272 E0.01546

G1 X85.436 Y112.359 E0.11400

G1 X84.844 Y112.359 E0.01546

G1 X87.932 Y109.272 E0.11400

G1 X87.339 Y109.272 E0.01546

G1 X84.252 Y112.359 E0.11400

G1 X83.660 Y112.359 E0.01546

G1 X86.747 Y109.272 E0.11400

G1 X86.155 Y109.272 E0.01546

G1 X83.060 Y112.367 E0.11428

G1 X82.429 Y112.406 E0.01651

G1 X85.563 Y109.272 E0.11572

G1 X84.971 Y109.272 E0.01546

G1 X81.719 Y112.524 E0.12009

G1 X81.427 Y112.579 E0.00774

G1 X80.906 Y112.745 E0.01428

G1 X84.379 Y109.272 E0.12823

G1 X83.787 Y109.272 E0.01546

```
G1 X79.897 Y113.161 E0.14362
G1 X79.751 Y113.229 E0.00420
G1 X78.986 Y113.703 E0.02352
G1 X78.284 Y114.269 E0.02351
G1 X77.658 Y114.916 E0.02351
G1 X77.116 Y115.635 E0.02352
G1 X76.697 Y116.362 E0.02190
G1 X75.699 Y117.359 E0.03684
G1 X75.107 Y117.359 E0.01546
G1 X83.195 Y109.272 E0.29862
G1 X82.637 Y109.238 E0.01460
G1 X74.515 Y117.359 E0.29986
G1 X73.923 Y117.359 E0.01546
G1 X82.104 Y109.179 E0.30204
G1 X81.605 Y109.086 E0.01325
G1 X73.331 Y117.359 E0.30549
G1 X72.739 Y117.359 E0.01546
G1 X81.138 Y108.960 E0.31012
G1 X80.689 Y108.817 E0.01230
G1 X72.147 Y117.359 E0.31540
G1 X71.555 Y117.359 E0.01546
G1 X80.272 Y108.642 E0.32186
G1 X79.867 Y108.456 E0.01165
G1 X70.963 Y117.359 E0.32875
G1 X70.371 Y117.359 E0.01546
G1 X79.490 Y108.240 E0.33670
G1 X79.124 Y108.014 E0.01123
G1 X69.963 Y117.175 E0.33826
G1 X69.963 Y116.583 E0.01546
```

```
G1 X78.782 Y107.764 E0.32563
G1 X78.454 Y107.500 E0.01099
G1 X69.963 Y115.991 E0.31352
G1 X69.963 Y115.399 E0.01546
G1 X78.144 Y107.218 E0.30207
G1 X77.853 Y106.917 E0.01093
G1 X69.963 Y114.807 E0.29132
G1 X69.963 Y114.215 E0.01546
G1 X77.574 Y106.604 E0.28101
G1 X77.319 Y106.266 E0.01104
G1 X69.963 Y113.623 E0.27162
G1 X69.963 Y113.031 E0.01546
G1 X77.073 Y105.921 E0.26250
G1 X76.856 Y105.546 E0.01132
G1 X69.963 Y112.439 E0.25451
G1 X69.963 Y111.847 E0.01546
G1 X76.645 Y105.165 E0.24671
G1 X76.469 Y104.748 E0.01180
G1 X69.963 Y111.255 E0.24022
G1 X69.963 Y110.663 E0.01546
G1 X76.299 Y104.326 E0.23395
G1 X76.284 Y104.272 E0.00148
G1 X75.762 Y104.272 E0.01363
G1 X69.963 Y110.070 E0.21411
G1 X69.963 Y109.478 E0.01546
G1 X75.170 Y104.272 E0.19225
G1 X74.578 Y104.272 E0.01546
G1 X69.963 Y108.886 E0.17039
G1 X69.963 Y108.294 E0.01546
```

```
G1 X73.986 Y104.272 E0.14853
G1 X73.394 Y104.272 E0.01546
G1 X69.963 Y107.702 E0.12666
G1 X69.963 Y107.110 E0.01546
G1 X72.802 Y104.272 E0.10480
G1 X72.209 Y104.272 E0.01546
G1 X69.963 Y106.518 E0.08294
G1 X69.963 Y105.926 E0.01546
G1 X71.617 Y104.272 E0.06108
G1 X71.025 Y104.272 E0.01546
G1 X69.963 Y105.334 E0.03922
G1 X69.963 Y104.742 E0.01546
G1 X70.621 Y104.084 E0.02431
M204 S1250
; stop printing object tpu print.STL id:3 copy 0
; printing object Petg print.STL id:2 copy 0
; stop printing object Petg print.STL id:2 copy 0
; printing object Petg print.STL id:6 copy 0
; stop printing object Petg print.STL id:6 copy 0
; printing object tpu print.STL id:27 copy 0
G1 E-2.24000 F2400.000
;WIPE_START
G1 F7200.000
G1 X69.963 Y104.742 E-0.29480
G1 X69.963 Y105.334 E-0.18749
G1 X70.923 Y104.374 E-0.42971
;WIPE_END
G1 E-0.04800 F2400.000
G1 Z0.550 F9000.000
```

```
G1 X120.285 Y122.787
G1 Z0.350
G1 E3.20000 F1500.000
M204 S800
;TYPE:Perimeter
;WIDTH:0.45
G1 F2400.000
G1 X127.156 Y122.787 E0.17903
G1 X127.247 Y123.111 E0.00876
G1 X127.583 Y123.905 E0.02246
G1 X128.013 Y124.652 E0.02246
G1 X128.532 Y125.340 E0.02246
G1 X129.131 Y125.960 E0.02246
G1 X129.802 Y126.501 E0.02246
G1 X130.535 Y126.955 E0.02246
G1 X131.318 Y127.315 E0.02246
G1 X132.139 Y127.577 E0.02246
G1 X132.986 Y127.735 E0.02246
G1 X133.827 Y127.787 E0.02194
G1 X140.999 Y127.787 E0.18688
G1 X140.999 Y131.502 E0.09678
G1 X133.827 Y131.502 E0.18688
G1 X132.986 Y131.554 E0.02194
G1 X132.139 Y131.712 E0.02246
G1 X131.318 Y131.974 E0.02246
G1 X130.535 Y132.334 E0.02246
G1 X129.802 Y132.789 E0.02246
G1 X129.131 Y133.329 E0.02246
G1 X128.532 Y133.949 E0.02246
```

```
G1 X128.013 Y134.637 E0.02246
G1 X127.583 Y135.384 E0.02246
G1 X127.247 Y136.178 E0.02246
G1 X127.156 Y136.502 E0.00876
G1 X120.285 Y136.502 E0.17903
G1 X120.285 Y122.847 E0.35578
M204 S1250
G1 X119.867 Y122.370 F9000.000
M204 S800
;TYPE:External perimeter
G1 F1800.000
G1 X127.472 Y122.370 E0.19815
G1 X127.642 Y122.972 E0.01632
G1 X127.957 Y123.719 E0.02112
G1 X128.362 Y124.421 E0.02112
G1 X128.850 Y125.068 E0.02112
G1 X129.413 Y125.651 E0.02112
G1 X130.044 Y126.159 E0.02112
G1 X130.733 Y126.586 E0.02112
G1 X131.469 Y126.925 E0.02112
G1 X132.241 Y127.171 E0.02112
G1 X133.038 Y127.320 E0.02112
G1 X133.840 Y127.370 E0.02093
G1 X141.417 Y127.370 E0.19743
G1 X141.417 Y131.920 E0.11855
M73 P26 R50
G1 X133.840 Y131.920 E0.19743
G1 X133.038 Y131.969 E0.02093
G1 X132.241 Y132.118 E0.02112
```

```
G1 X131.469 Y132.364 E0.02112
G1 X130.733 Y132.703 E0.02112
G1 X130.044 Y133.130 E0.02112
G1 X129.413 Y133.639 E0.02112
G1 X128.850 Y134.221 E0.02112
G1 X128.362 Y134.868 E0.02112
G1 X127.957 Y135.570 E0.02112
G1 X127.642 Y136.317 E0.02112
G1 X127.472 Y136.920 E0.01632
G1 X119.867 Y136.920 E0.19815
G1 X119.867 Y122.430 E0.37755
M204 S1250
G1 X120.241 Y122.512 F9000.000
G1 E-2.24000 F2400.000
;WIPE_START
G1 F7200.000
G1 X122.747 Y122.407 E-0.91200
;WIPE_END
G1 E-0.04800 F2400.000
G1 Z0.550 F9000.000
G1 X140.027 Y131.376
G1 Z0.350
G1 E3.20000 F1500.000
M204 S1000
;TYPE:Solid infill
;WIDTH:0.450839
G1 F4800.000
G1 X140.686 Y130.718 E0.02431
G1 X140.686 Y130.126 E0.01546
```

```
G1 X139.623 Y131.188 E0.03923
G1 X139.031 Y131.188 E0.01546
G1 X140.686 Y129.534 E0.06109
G1 X140.686 Y128.942 E0.01546
G1 X138.439 Y131.188 E0.08295
G1 X137.847 Y131.188 E0.01546
G1 X140.686 Y128.350 E0.10481
G1 X140.686 Y128.101 E0.00650
G1 X140.343 Y128.101 E0.00895
G1 X137.255 Y131.188 E0.11400
G1 X136.663 Y131.188 E0.01546
G1 X139.751 Y128.101 E0.11400
G1 X139.159 Y128.101 E0.01546
G1 X136.071 Y131.188 E0.11400
G1 X135.479 Y131.188 E0.01546
G1 X138.567 Y128.101 E0.11400
G1 X137.975 Y128.101 E0.01546
G1 X134.887 Y131.188 E0.11400
G1 X134.295 Y131.188 E0.01546
G1 X137.383 Y128.101 E0.11400
G1 X136.790 Y128.101 E0.01546
G1 X133.695 Y131.196 E0.11428
G1 X133.064 Y131.235 E0.01651
G1 X136.198 Y128.101 E0.11572
G1 X135.606 Y128.101 E0.01546
G1 X132.354 Y131.353 E0.12009
G1 X132.063 Y131.408 E0.00774
G1 X131.541 Y131.574 E0.01428
G1 X135.014 Y128.101 E0.12823
```

```
G1 X134.422 Y128.101 E0.01546
G1 X130.532 Y131.991 E0.14362
G1 X130.386 Y132.058 E0.00420
G1 X129.621 Y132.532 E0.02352
G1 X128.919 Y133.098 E0.02351
G1 X128.293 Y133.745 E0.02351
G1 X127.751 Y134.464 E0.02352
G1 X127.332 Y135.191 E0.02190
G1 X126.335 Y136.188 E0.03684
G1 X125.743 Y136.188 E0.01546
G1 X133.830 Y128.101 E0.29862
G1 X133.272 Y128.067 E0.01460
G1 X125.150 Y136.188 E0.29986
G1 X124.558 Y136.188 E0.01546
G1 X132.739 Y128.008 E0.30204
G1 X132.240 Y127.915 E0.01325
G1 X123.966 Y136.188 E0.30549
G1 X123.374 Y136.188 E0.01546
G1 X131.773 Y127.789 E0.31012
G1 X131.324 Y127.646 E0.01230
G1 X122.782 Y136.188 E0.31540
G1 X122.190 Y136.188 E0.01546
G1 X130.907 Y127.471 E0.32186
G1 X130.502 Y127.285 E0.01165
G1 X121.598 Y136.188 E0.32875
G1 X121.006 Y136.188 E0.01546
G1 X130.125 Y127.069 E0.33670
G1 X129.760 Y126.843 E0.01123
G1 X120.598 Y136.004 E0.33826
```

G1 X120.598 Y135.412 E0.01546

G1 X129.417 Y126.593 E0.32563

G1 X129.090 Y126.329 E0.01099

G1 X120.598 Y134.820 E0.31352

G1 X120.598 Y134.228 E0.01546

G1 X128.779 Y126.047 E0.30207

G1 X128.488 Y125.746 E0.01093

G1 X120.598 Y133.636 E0.29132

G1 X120.598 Y133.044 E0.01546

G1 X128.209 Y125.433 E0.28101

G1 X127.955 Y125.095 E0.01104

G1 X120.598 Y132.452 E0.27162

G1 X120.598 Y131.860 E0.01546

G1 X127.708 Y124.750 E0.26250

G1 X127.491 Y124.375 E0.01132

G1 X120.598 Y131.268 E0.25451

G1 X120.598 Y130.676 E0.01546

G1 X127.280 Y123.994 E0.24671

G1 X127.104 Y123.578 E0.01180

G1 X120.598 Y130.084 E0.24022

G1 X120.598 Y129.492 E0.01546

G1 X126.934 Y123.155 E0.23395

G1 X126.919 Y123.101 E0.00148

G1 X126.397 Y123.101 E0.01363

G1 X120.598 Y128.900 E0.21411

G1 X120.598 Y128.307 E0.01546

G1 X125.805 Y123.101 E0.19225

G1 X125.213 Y123.101 E0.01546

G1 X120.598 Y127.715 E0.17039

```
G1 X120.598 Y127.123 E0.01546
G1 X124.621 Y123.101 E0.14853
G1 X124.029 Y123.101 E0.01546
G1 X120.598 Y126.531 E0.12666
G1 X120.598 Y125.939 E0.01546
G1 X123.437 Y123.101 E0.10480
G1 X122.845 Y123.101 E0.01546
G1 X120.598 Y125.347 E0.08294
G1 X120.598 Y124.755 E0.01546
G1 X122.253 Y123.101 E0.06108
G1 X121.660 Y123.101 E0.01546
G1 X120.598 Y124.163 E0.03922
G1 X120.598 Y123.571 E0.01546
G1 X121.256 Y122.913 E0.02431
M204 S1250
; stop printing object tpu print.STL id:27 copy 0
; printing object tpu print.STL id:23 copy 0
G1 E-2.24000 F2400.000
;WIPE_START
G1 F7200.000
G1 X120.598 Y123.571 E-0.29480
G1 X120.598 Y124.163 E-0.18749
G1 X121.558 Y123.204 E-0.42971
;WIPE_END
G1 E-0.04800 F2400.000
G1 Z0.550 F9000.000
G1 X120.300 Y117.848
G1 Z0.350
G1 E3.20000 F1500.000
```

```
M204 S800
;TYPE:Perimeter
;WIDTH:0.45
G1 F2400.000
G1 X120.300 Y104.133 E0.35734
G1 X127.171 Y104.133 E0.17903
G1 X127.263 Y104.457 E0.00876
G1 X127.598 Y105.251 E0.02246
G1 X128.028 Y105.998 E0.02246
G1 X128.547 Y106.686 E0.02246
G1 X129.146 Y107.306 E0.02246
G1 X129.817 Y107.847 E0.02246
G1 X130.550 Y108.301 E0.02246
G1 X131.333 Y108.661 E0.02246
G1 X132.154 Y108.923 E0.02246
G1 X133.002 Y109.082 E0.02246
G1 X133.842 Y109.133 E0.02194
G1 X141.014 Y109.133 E0.18688
G1 X141.014 Y112.848 E0.09678
G1 X133.842 Y112.848 E0.18688
G1 X133.002 Y112.900 E0.02194
G1 X132.154 Y113.058 E0.02246
G1 X131.333 Y113.320 E0.02246
G1 X130.550 Y113.680 E0.02246
G1 X129.817 Y114.135 E0.02246
G1 X129.146 Y114.675 E0.02246
G1 X128.547 Y115.295 E0.02246
G1 X128.028 Y115.983 E0.02246
G1 X127.598 Y116.730 E0.02246
```

```
G1 X127.263 Y117.524 E0.02246
G1 X127.171 Y117.848 E0.00876
G1 X120.360 Y117.848 E0.17747
M204 S1250
G1 X119.882 Y118.266 F9000.000
M204 S800
;TYPE:External perimeter
G1 F1800.000
G1 X119.882 Y103.716 E0.37911
G1 X127.487 Y103.716 E0.19815
G1 X127.658 Y104.318 E0.01632
G1 X127.973 Y105.065 E0.02112
G1 X128.377 Y105.767 E0.02112
G1 X128.865 Y106.414 E0.02112
G1 X129.428 Y106.997 E0.02112
G1 X130.059 Y107.505 E0.02112
G1 X130.748 Y107.932 E0.02112
G1 X131.484 Y108.271 E0.02112
G1 X132.257 Y108.517 E0.02112
G1 X133.053 Y108.666 E0.02112
G1 X133.855 Y108.716 E0.02093
G1 X141.432 Y108.716 E0.19743
G1 X141.432 Y113.266 E0.11855
G1 X133.855 Y113.266 E0.19743
G1 X133.053 Y113.315 E0.02093
G1 X132.257 Y113.464 E0.02112
G1 X131.484 Y113.710 E0.02112
G1 X130.748 Y114.049 E0.02112
G1 X130.059 Y114.476 E0.02112
```

```
G1 X129.428 Y114.985 E0.02112
G1 X128.865 Y115.567 E0.02112
G1 X128.377 Y116.214 E0.02112
G1 X127.973 Y116.916 E0.02112
G1 X127.658 Y117.663 E0.02112
G1 X127.487 Y118.266 E0.01632
G1 X119.942 Y118.266 E0.19659
M204 S1250
G1 X120.073 Y117.914 F9000.000
G1 E-2.24000 F2400.000
;WIPE_START
G1 F7200.000
G1 X119.930 Y115.386 E-0.91200
;WIPE_END
G1 E-0.04800 F2400.000
G1 Z0.550 F9000.000
G1 X140.043 Y112.722
G1 Z0.350
G1 E3.20000 F1500.000
M204 S1000
;TYPE:Solid infill
;WIDTH:0.450839
G1 F4800.000
G1 X140.701 Y112.064 E0.02431
G1 X140.701 Y111.472 E0.01546
G1 X139.639 Y112.534 E0.03923
G1 X139.046 Y112.534 E0.01546
G1 X140.701 Y110.880 E0.06109
G1 X140.701 Y110.288 E0.01546
```

```
G1 X138.454 Y112.534 E0.08295
G1 X137.862 Y112.534 E0.01546
G1 X140.701 Y109.696 E0.10481
G1 X140.701 Y109.447 E0.00650
G1 X140.358 Y109.447 E0.00895
G1 X137.270 Y112.534 E0.11400
G1 X136.678 Y112.534 E0.01546
G1 X139.766 Y109.447 E0.11400
G1 X139.174 Y109.447 E0.01546
G1 X136.086 Y112.534 E0.11400
G1 X135.494 Y112.534 E0.01546
G1 X138.582 Y109.447 E0.11400
G1 X137.990 Y109.447 E0.01546
G1 X134.902 Y112.534 E0.11400
G1 X134.310 Y112.534 E0.01546
G1 X137.398 Y109.447 E0.11400
G1 X136.806 Y109.447 E0.01546
G1 X133.710 Y112.542 E0.11428
G1 X133.079 Y112.581 E0.01651
G1 X136.214 Y109.447 E0.11572
G1 X135.621 Y109.447 E0.01546
G1 X132.369 Y112.699 E0.12009
G1 X132.078 Y112.754 E0.00774
G1 X131.556 Y112.920 E0.01428
G1 X135.029 Y109.447 E0.12823
G1 X134.437 Y109.447 E0.01546
G1 X130.548 Y113.337 E0.14362
G1 X130.401 Y113.404 E0.00420
G1 X129.636 Y113.878 E0.02352
```

```
G1 X128.935 Y114.444 E0.02351
G1 X128.308 Y115.091 E0.02351
G1 X127.766 Y115.810 E0.02352
G1 X127.347 Y116.537 E0.02190
G1 X126.350 Y117.534 E0.03684
G1 X125.758 Y117.534 E0.01546
G1 X133.845 Y109.447 E0.29862
G1 X133.287 Y109.413 E0.01460
G1 X125.166 Y117.534 E0.29986
G1 X124.574 Y117.534 E0.01546
G1 X132.754 Y109.354 E0.30204
G1 X132.255 Y109.261 E0.01325
G1 X123.981 Y117.534 E0.30549
G1 X123.389 Y117.534 E0.01546
G1 X131.788 Y109.135 E0.31012
G1 X131.339 Y108.992 E0.01230
G1 X122.797 Y117.534 E0.31540
G1 X122.205 Y117.534 E0.01546
G1 X130.922 Y108.817 E0.32186
G1 X130.517 Y108.631 E0.01165
G1 X121.613 Y117.534 E0.32875
G1 X121.021 Y117.534 E0.01546
G1 X130.140 Y108.415 E0.33670
G1 X129.775 Y108.189 E0.01123
G1 X120.613 Y117.350 E0.33826
G1 X120.613 Y116.758 E0.01546
G1 X129.433 Y107.939 E0.32563
G1 X129.105 Y107.675 E0.01099
G1 X120.613 Y116.166 E0.31352
```

```
G1 X120.613 Y115.574 E0.01546
G1 X128.794 Y107.393 E0.30207
G1 X128.503 Y107.092 E0.01093
G1 X120.613 Y114.982 E0.29132
G1 X120.613 Y114.390 E0.01546
G1 X128.224 Y106.779 E0.28101
G1 X127.970 Y106.441 E0.01104
G1 X120.613 Y113.798 E0.27162
G1 X120.613 Y113.206 E0.01546
G1 X127.723 Y106.096 E0.26250
G1 X127.506 Y105.721 E0.01132
G1 X120.613 Y112.614 E0.25451
G1 X120.613 Y112.022 E0.01546
G1 X127.295 Y105.340 E0.24671
G1 X127.119 Y104.924 E0.01180
G1 X120.613 Y111.430 E0.24022
G1 X120.613 Y110.838 E0.01546
G1 X126.950 Y104.501 E0.23395
G1 X126.934 Y104.447 E0.00148
G1 X126.412 Y104.447 E0.01363
G1 X120.613 Y110.246 E0.21411
G1 X120.613 Y109.654 E0.01546
G1 X125.820 Y104.447 E0.19225
G1 X125.228 Y104.447 E0.01546
G1 X120.613 Y109.061 E0.17039
G1 X120.613 Y108.469 E0.01546
G1 X124.636 Y104.447 E0.14853
G1 X124.044 Y104.447 E0.01546
G1 X120.613 Y107.877 E0.12666
```

```
G1 X120.613 Y107.285 E0.01546
G1 X123.452 Y104.447 E0.10480
G1 X122.860 Y104.447 E0.01546
G1 X120.613 Y106.693 E0.08294
G1 X120.613 Y106.101 E0.01546
G1 X122.268 Y104.447 E0.06108
G1 X121.676 Y104.447 E0.01546
G1 X120.613 Y105.509 E0.03922
G1 X120.613 Y104.917 E0.01546
G1 X121.272 Y104.259 E0.02431
M204 S1250
; stop printing object tpu print.STL id:23 copy 0
; printing object tpu print.STL id:21 copy 0
G1 E-2.24000 F2400.000
;WIPE_START
G1 F7200.000
G1 X120.613 Y104.917 E-0.29480
G1 X120.613 Y105.509 E-0.18749
G1 X121.573 Y104.550 E-0.42971
;WIPE_END
G1 E-0.04800 F2400.000
G1 Z0.550 F9000.000
G1 X120.293 Y97.032
G1 Z0.350
G1 E3.20000 F1500.000
M204 S800
;TYPE:Perimeter
;WIDTH:0.45
G1 F2400.000
```

```
G1 X120.293 Y83.318 E0.35734
G1 X127.164 Y83.318 E0.17903
G1 X127.256 Y83.641 E0.00876
G1 X127.591 Y84.436 E0.02246
G1 X128.021 Y85.183 E0.02246
G1 X128.540 Y85.871 E0.02246
G1 X129.139 Y86.490 E0.02246
G1 X129.811 Y87.031 E0.02246
G1 X130.543 Y87.485 E0.02246
G1 X131.326 Y87.846 E0.02246
G1 X132.147 Y88.107 E0.02246
G1 X132.995 Y88.266 E0.02246
G1 X133.835 Y88.318 E0.02194
G1 X141.007 Y88.318 E0.18688
G1 X141.007 Y92.032 E0.09678
G1 X133.835 Y92.032 E0.18688
G1 X132.995 Y92.084 E0.02194
G1 X132.147 Y92.243 E0.02246
G1 X131.326 Y92.504 E0.02246
G1 X130.543 Y92.865 E0.02246
G1 X129.811 Y93.319 E0.02246
G1 X129.139 Y93.860 E0.02246
G1 X128.540 Y94.479 E0.02246
G1 X128.021 Y95.168 E0.02246
G1 X127.591 Y95.915 E0.02246
G1 X127.256 Y96.709 E0.02246
G1 X127.164 Y97.032 E0.00876
G1 X120.353 Y97.032 E0.17747
M204 S1250
```

G1 X119.875 Y97.450 F9000.000

M204 S800

;TYPE:External perimeter

G1 F1800.000

G1 X119.875 Y82.900 E0.37911

G1 X127.480 Y82.900 E0.19815

G1 X127.651 Y83.503 E0.01632

G1 X127.966 Y84.249 E0.02112

G1 X128.370 Y84.952 E0.02112

G1 X128.858 Y85.599 E0.02112

G1 X129.422 Y86.181 E0.02112

G1 X130.053 Y86.690 E0.02112

G1 X130.741 Y87.117 E0.02112

G1 X131.477 Y87.456 E0.02112

G1 X132.250 Y87.702 E0.02112

G1 X133.046 Y87.851 E0.02112

G1 X133.848 Y87.900 E0.02093

G1 X141.425 Y87.900 E0.19743

G1 X141.425 Y92.450 E0.11855

G1 X133.848 Y92.450 E0.19743

G1 X133.046 Y92.500 E0.02093

G1 X132.250 Y92.649 E0.02112

G1 X131.477 Y92.895 E0.02112

G1 X130.741 Y93.234 E0.02112

G1 X130.053 Y93.661 E0.02112

G1 X129.422 Y94.169 E0.02112

G1 X128.858 Y94.751 E0.02112

G1 X128.370 Y95.398 E0.02112

G1 X127.966 Y96.101 E0.02112

```
G1 X127.651 Y96.847 E0.02112
G1 X127.480 Y97.450 E0.01632
G1 X119.935 Y97.450 E0.19659
M204 S1250
G1 X120.066 Y97.099 F9000.000
G1 E-2.24000 F2400.000
;WIPE_START
G1 F7200.000
G1 X119.923 Y94.570 E-0.91200
;WIPE_END
G1 E-0.04800 F2400.000
G1 Z0.550 F9000.000
G1 X140.036 Y91.907
G1 Z0.350
G1 E3.20000 F1500.000
M204 S1000
;TYPE:Solid infill
;WIDTH:0.450839
G1 F4800.000
G1 X140.694 Y91.249 E0.02431
G1 X140.694 Y90.656 E0.01546
G1 X139.632 Y91.719 E0.03923
G1 X139.040 Y91.719 E0.01546
G1 X140.694 Y90.064 E0.06109
G1 X140.694 Y89.472 E0.01546
G1 X138.448 Y91.719 E0.08295
G1 X137.855 Y91.719 E0.01546
G1 X140.694 Y88.880 E0.10481
G1 X140.694 Y88.631 E0.00650
```

G1 X140.351 Y88.631 E0.00895

G1 X137.263 Y91.719 E0.11400

G1 X136.671 Y91.719 E0.01546

G1 X139.759 Y88.631 E0.11400

G1 X139.167 Y88.631 E0.01546

G1 X136.079 Y91.719 E0.11400

G1 X135.487 Y91.719 E0.01546

G1 X138.575 Y88.631 E0.11400

G1 X137.983 Y88.631 E0.01546

G1 X134.895 Y91.719 E0.11400

G1 X134.303 Y91.719 E0.01546

G1 X137.391 Y88.631 E0.11400

G1 X136.799 Y88.631 E0.01546

G1 X133.704 Y91.726 E0.11428

G1 X133.073 Y91.765 E0.01651

G1 X136.207 Y88.631 E0.11572

G1 X135.615 Y88.631 E0.01546

G1 X132.362 Y91.884 E0.12009

G1 X132.071 Y91.938 E0.00774

G1 X131.550 Y92.104 E0.01428

G1 X135.023 Y88.631 E0.12823

G1 X134.431 Y88.631 E0.01546

G1 X130.541 Y92.521 E0.14362

G1 X130.395 Y92.588 E0.00420

G1 X129.629 Y93.063 E0.02352

G1 X128.928 Y93.628 E0.02351

G1 X128.301 Y94.275 E0.02351

G1 X127.759 Y94.994 E0.02352

G1 X127.341 Y95.721 E0.02190

```
G1 X126.343 Y96.719 E0.03684
G1 X125.751 Y96.719 E0.01546
G1 X133.838 Y88.631 E0.29862
G1 X133.280 Y88.598 E0.01460
G1 X125.159 Y96.719 E0.29986
G1 X124.567 Y96.719 E0.01546
G1 X132.747 Y88.538 E0.30204
G1 X132.248 Y88.445 E0.01325
G1 X123.975 Y96.719 E0.30549
G1 X123.383 Y96.719 E0.01546
G1 X131.782 Y88.320 E0.31012
G1 X131.333 Y88.177 E0.01230
G1 X122.790 Y96.719 E0.31540
G1 X122.198 Y96.719 E0.01546
G1 X130.916 Y88.002 E0.32186
G1 X130.510 Y87.815 E0.01165
G1 X121.606 Y96.719 E0.32875
G1 X121.014 Y96.719 E0.01546
G1 X130.133 Y87.600 E0.33670
G1 X129.768 Y87.373 E0.01123
G1 X120.606 Y96.535 E0.33826
G1 X120.606 Y95.943 E0.01546
G1 X129.426 Y87.123 E0.32563
G1 X129.098 Y86.859 E0.01099
G1 X120.606 Y95.351 E0.31352
G1 X120.606 Y94.759 E0.01546
G1 X128.788 Y86.577 E0.30207
G1 X128.496 Y86.277 E0.01093
G1 X120.606 Y94.167 E0.29132
```

G1 X120.606 Y93.574 E0.01546

G1 X128.217 Y85.964 E0.28101

G1 X127.963 Y85.626 E0.01104

G1 X120.606 Y92.982 E0.27162

G1 X120.606 Y92.390 E0.01546

G1 X127.716 Y85.281 E0.26250

G1 X127.500 Y84.905 E0.01132

G1 X120.606 Y91.798 E0.25451

G1 X120.606 Y91.206 E0.01546

G1 X127.288 Y84.524 E0.24671

G1 X127.113 Y84.108 E0.01180

G1 X120.606 Y90.614 E0.24022

G1 X120.606 Y90.022 E0.01546

G1 X126.943 Y83.686 E0.23395

G1 X126.927 Y83.631 E0.00148

G1 X126.405 Y83.631 E0.01363

G1 X120.606 Y89.430 E0.21411

G1 X120.606 Y88.838 E0.01546

G1 X125.813 Y83.631 E0.19225

G1 X125.221 Y83.631 E0.01546

G1 X120.606 Y88.246 E0.17039

G1 X120.606 Y87.654 E0.01546

G1 X124.629 Y83.631 E0.14853

G1 X124.037 Y83.631 E0.01546

G1 X120.606 Y87.062 E0.12666

G1 X120.606 Y86.470 E0.01546

G1 X123.445 Y83.631 E0.10480

G1 X122.853 Y83.631 E0.01546

G1 X120.606 Y85.878 E0.08294

G1 X120.606 Y85.286 E0.01546

G1 X122.261 Y83.631 E0.06108

G1 X121.669 Y83.631 E0.01546

G1 X120.606 Y84.694 E0.03922

G1 X120.606 Y84.102 E0.01546

G1 X121.265 Y83.443 E0.02431

M204 S1250

; stop printing object tpu print.STL id:21 copy 0

; printing object Petg print.STL id:20 copy 0

; stop printing object Petg print.STL id:20 copy 0

; printing object Petg print.STL id:22 copy 0

; stop printing object Petg print.STL id:22 copy 0

; printing object Petg print.STL id:26 copy 0

; stop printing object Petg print.STL id:26 copy 0

; printing object Petg print.STL id:0 copy 0

; stop printing object Petg print.STL id:0 copy 0

; printing object tpu print.STL id:1 copy 0

G1 E-2.24000 F2400.000

;WIPE_START

G1 F7200.000

G1 X120.606 Y84.102 E-0.29480

G1 X120.606 Y84.694 E-0.18749

G1 X121.566 Y83.734 E-0.42971

;WIPE_END

G1 E-0.04800 F2400.000

G1 Z0.550 F9000.000

G1 X90.357 Y88.143

G1 Z0.350

G1 E3.20000 F1500.000

```
M204 S800
;TYPE:Perimeter
;WIDTH:0.45
G1 F2400.000
G1 X90.357 Y91.857 E0.09678
G1 X83.185 Y91.857 E0.18688
G1 X82.344 Y91.909 E0.02194
G1 X81.497 Y92.068 E0.02246
G1 X80.676 Y92.329 E0.02246
G1 X79.893 Y92.690 E0.02246
G1 X79.160 Y93.144 E0.02246
G1 X78.489 Y93.685 E0.02246
G1 X77.890 Y94.304 E0.02246
G1 X77.371 Y94.993 E0.02246
G1 X76.941 Y95.739 E0.02246
G1 X76.606 Y96.534 E0.02246
G1 X76.514 Y96.857 E0.00876
G1 X69.643 Y96.857 E0.17903
G1 X69.643 Y83.143 E0.35734
G1 X76.514 Y83.143 E0.17903
G1 X76.606 Y83.466 E0.00876
G1 X76.941 Y84.261 E0.02246
G1 X77.371 Y85.007 E0.02246
G1 X77.890 Y85.696 E0.02246
G1 X78.489 Y86.315 E0.02246
G1 X79.160 Y86.856 E0.02246
G1 X79.893 Y87.310 E0.02246
G1 X80.676 Y87.671 E0.02246
G1 X81.497 Y87.932 E0.02246
```

```
G1 X82.344 Y88.091 E0.02246
G1 X83.185 Y88.143 E0.02194
G1 X90.297 Y88.143 E0.18531
M204 S1250
G1 X90.775 Y87.725 F9000.000
M204 S800
;TYPE:External perimeter
M73 P27 R50
G1 F1800.000
G1 X90.775 Y92.275 E0.11855
G1 X83.198 Y92.275 E0.19743
G1 X82.396 Y92.325 E0.02093
G1 X81.599 Y92.474 E0.02112
G1 X80.827 Y92.719 E0.02112
G1 X80.091 Y93.059 E0.02112
G1 X79.402 Y93.486 E0.02112
G1 X78.771 Y93.994 E0.02112
G1 X78.208 Y94.576 E0.02112
G1 X77.720 Y95.223 E0.02112
G1 X77.315 Y95.926 E0.02112
G1 X77.000 Y96.672 E0.02112
G1 X76.830 Y97.275 E0.01632
G1 X69.225 Y97.275 E0.19815
G1 X69.225 Y82.725 E0.37911
G1 X76.830 Y82.725 E0.19815
G1 X77.000 Y83.328 E0.01632
G1 X77.315 Y84.074 E0.02112
G1 X77.720 Y84.777 E0.02112
G1 X78.208 Y85.424 E0.02112
```

```
G1 X78.771 Y86.006 E0.02112
G1 X79.402 Y86.514 E0.02112
G1 X80.091 Y86.941 E0.02112
G1 X80.827 Y87.281 E0.02112
G1 X81.599 Y87.526 E0.02112
G1 X82.396 Y87.675 E0.02112
G1 X83.198 Y87.725 E0.02093
G1 X90.715 Y87.725 E0.19586
M204 S1250
G1 X90.574 Y88.071 F9000.000
G1 E-2.24000 F2400.000
;WIPE_START
G1 F7200.000
G1 X90.753 Y90.605 E-0.91200
;WIPE_END
G1 E-0.04800 F2400.000
G1 Z0.550 F9000.000
G1 X70.614 Y83.268
G1 Z0.350
G1 E3.20000 F1500.000
M204 S1000
;TYPE:Solid infill
;WIDTH:0.450839
G1 F4800.000
G1 X69.956 Y83.926 E0.02431
G1 X69.956 Y84.518 E0.01546
G1 X71.018 Y83.456 E0.03922
G1 X71.611 Y83.456 E0.01546
G1 X69.956 Y85.111 E0.06108
```

```
G1 X69.956 Y85.703 E0.01546
G1 X72.203 Y83.456 E0.08294
G1 X72.795 Y83.456 E0.01546
G1 X69.956 Y86.295 E0.10480
G1 X69.956 Y86.887 E0.01546
G1 X73.387 Y83.456 E0.12666
G1 X73.979 Y83.456 E0.01546
G1 X69.956 Y87.479 E0.14853
G1 X69.956 Y88.071 E0.01546
G1 X74.571 Y83.456 E0.17039
G1 X75.163 Y83.456 E0.01546
G1 X69.956 Y88.663 E0.19225
G1 X69.956 Y89.255 E0.01546
G1 X75.755 Y83.456 E0.21411
G1 X76.277 Y83.456 E0.01363
G1 X76.292 Y83.511 E0.00148
G1 X69.956 Y89.847 E0.23395
G1 X69.956 Y90.439 E0.01546
G1 X76.462 Y83.933 E0.24022
G1 X76.638 Y84.349 E0.01180
G1 X69.956 Y91.031 E0.24671
G1 X69.956 Y91.623 E0.01546
G1 X76.849 Y84.730 E0.25451
G1 X77.066 Y85.106 E0.01132
G1 X69.956 Y92.215 E0.26250
G1 X69.956 Y92.807 E0.01546
G1 X77.313 Y85.451 E0.27162
G1 X77.567 Y85.789 E0.01104
G1 X69.956 Y93.399 E0.28101
```

```
G1 X69.956 Y93.991 E0.01546
G1 X77.846 Y86.101 E0.29132
G1 X78.137 Y86.402 E0.01093
G1 X69.956 Y94.583 E0.30207
G1 X69.956 Y95.176 E0.01546
G1 X78.448 Y86.684 E0.31352
G1 X78.775 Y86.948 E0.01099
G1 X69.956 Y95.768 E0.32563
G1 X69.956 Y96.360 E0.01546
G1 X79.118 Y87.198 E0.33826
G1 X79.483 Y87.425 E0.01123
G1 X70.364 Y96.544 E0.33670
G1 X70.956 Y96.544 E0.01546
G1 X79.860 Y87.640 E0.32875
G1 X80.265 Y87.827 E0.01165
G1 X71.548 Y96.544 E0.32186
G1 X72.140 Y96.544 E0.01546
G1 X80.682 Y88.002 E0.31540
G1 X81.131 Y88.145 E0.01230
G1 X72.732 Y96.544 E0.31012
G1 X73.324 Y96.544 E0.01546
G1 X81.598 Y88.270 E0.30549
G1 X82.097 Y88.363 E0.01325
G1 X73.916 Y96.544 E0.30204
G1 X74.508 Y96.544 E0.01546
G1 X82.630 Y88.422 E0.29986
G1 X83.188 Y88.456 E0.01460
G1 X75.101 Y96.544 E0.29862
G1 X75.693 Y96.544 E0.01546
```

```
G1 X76.690 Y95.546 E0.03684
G1 X77.109 Y94.819 E0.02190
G1 X77.651 Y94.100 E0.02352
G1 X78.277 Y93.453 E0.02351
G1 X78.979 Y92.888 E0.02351
G1 X79.744 Y92.413 E0.02352
G1 X79.890 Y92.346 E0.00420
G1 X83.780 Y88.456 E0.14362
G1 X84.372 Y88.456 E0.01546
G1 X80.899 Y91.929 E0.12823
G1 X81.421 Y91.763 E0.01428
G1 X81.712 Y91.709 E0.00774
G1 X84.964 Y88.456 E0.12009
G1 X85.556 Y88.456 E0.01546
G1 X82.422 Y91.590 E0.11572
G1 X83.053 Y91.551 E0.01651
G1 X86.149 Y88.456 E0.11428
G1 X86.741 Y88.456 E0.01546
G1 X83.653 Y91.544 E0.11400
G1 X84.245 Y91.544 E0.01546
G1 X87.333 Y88.456 E0.11400
G1 X87.925 Y88.456 E0.01546
G1 X84.837 Y91.544 E0.11400
G1 X85.429 Y91.544 E0.01546
G1 X88.517 Y88.456 E0.11400
G1 X89.109 Y88.456 E0.01546
G1 X86.021 Y91.544 E0.11400
G1 X86.613 Y91.544 E0.01546
G1 X89.701 Y88.456 E0.11400
```

G1 X90.044 Y88.456 E0.00895

G1 X90.044 Y88.705 E0.00650

G1 X87.205 Y91.544 E0.10481

G1 X87.797 Y91.544 E0.01546

G1 X90.044 Y89.297 E0.08295

G1 X90.044 Y89.889 E0.01546

G1 X88.389 Y91.544 E0.06109

G1 X88.981 Y91.544 E0.01546

G1 X90.044 Y90.481 E0.03923

G1 X90.044 Y91.073 E0.01546

G1 X89.385 Y91.732 E0.02431

M204 S1250

; stop printing object tpu print.STL id:1 copy 0

; printing object tpu print.STL id:5 copy 0

G1 E-2.24000 F2400.000

;WIPE_START

G1 F7200.000

G1 X90.044 Y91.073 E-0.29484

G1 X90.044 Y90.481 E-0.18749

G1 X89.084 Y91.441 E-0.42968

;WIPE_END

G1 E-0.04800 F2400.000

G1 Z0.550 F9000.000

G1 X76.565 Y74.665

G1 Z0.350

G1 E3.20000 F1500.000

M204 S800

;TYPE:Perimeter

;WIDTH:0.45

```
G1 F2400.000
G1 X69.694 Y74.665 E0.17903
G1 X69.694 Y60.950 E0.35734
G1 X76.565 Y60.950 E0.17903
G1 X76.657 Y61.274 E0.00876
G1 X76.992 Y62.068 E0.02246
G1 X77.422 Y62.815 E0.02246
G1 X77.941 Y63.503 E0.02246
G1 X78.540 Y64.123 E0.02246
G1 X79.211 Y64.664 E0.02246
G1 X79.944 Y65.118 E0.02246
G1 X80.727 Y65.478 E0.02246
G1 X81.548 Y65.740 E0.02246
G1 X82.396 Y65.899 E0.02246
G1 X83.236 Y65.950 E0.02194
G1 X90.408 Y65.950 E0.18688
G1 X90.408 Y69.665 E0.09678
G1 X83.236 Y69.665 E0.18688
G1 X82.396 Y69.717 E0.02194
G1 X81.548 Y69.875 E0.02246
G1 X80.727 Y70.137 E0.02246
G1 X79.944 Y70.497 E0.02246
G1 X79.211 Y70.952 E0.02246
G1 X78.540 Y71.493 E0.02246
G1 X77.941 Y72.112 E0.02246
G1 X77.422 Y72.800 E0.02246
G1 X76.992 Y73.547 E0.02246
G1 X76.657 Y74.341 E0.02246
G1 X76.581 Y74.607 E0.00720
```

```
M204 S1250
G1 X76.881 Y75.083 F9000.000
M204 S800
;TYPE:External perimeter
G1 F1800.000
G1 X69.276 Y75.083 E0.19815
G1 X69.276 Y60.533 E0.37911
G1 X76.881 Y60.533 E0.19815
G1 X77.052 Y61.135 E0.01632
G1 X77.367 Y61.882 E0.02112
G1 X77.771 Y62.584 E0.02112
G1 X78.259 Y63.231 E0.02112
G1 X78.823 Y63.814 E0.02112
G1 X79.454 Y64.322 E0.02112
G1 X80.142 Y64.749 E0.02112
G1 X80.878 Y65.088 E0.02112
G1 X81.651 Y65.334 E0.02112
G1 X82.447 Y65.483 E0.02112
G1 X83.249 Y65.533 E0.02093
G1 X90.826 Y65.533 E0.19743
G1 X90.826 Y70.083 E0.11855
G1 X83.249 Y70.083 E0.19743
G1 X82.447 Y70.132 E0.02093
G1 X81.651 Y70.281 E0.02112
G1 X80.878 Y70.527 E0.02112
G1 X80.142 Y70.866 E0.02112
G1 X79.454 Y71.293 E0.02112
G1 X78.823 Y71.802 E0.02112
G1 X78.259 Y72.384 E0.02112
```

```
G1 X77.771 Y73.031 E0.02112
G1 X77.367 Y73.733 E0.02112
G1 X77.052 Y74.480 E0.02112
G1 X76.897 Y75.025 E0.01476
M204 S1250
G1 X76.560 Y74.844 F9000.000
G1 E-2.24000 F2400.000
;WIPE_START
G1 F7200.000
G1 X74.017 Y75.047 E-0.91200
;WIPE_END
G1 E-0.04800 F2400.000
G1 Z0.550 F9000.000
G1 X70.666 Y61.076
G1 Z0.350
G1 E3.20000 F1500.000
M204 S1000
;TYPE:Solid infill
;WIDTH:0.450839
G1 F4800.000
G1 X70.007 Y61.734 E0.02431
G1 X70.007 Y62.326 E0.01546
G1 X71.070 Y61.264 E0.03922
G1 X71.662 Y61.264 E0.01546
G1 X70.007 Y62.918 E0.06108
G1 X70.007 Y63.510 E0.01546
G1 X72.254 Y61.264 E0.08294
G1 X72.846 Y61.264 E0.01546
G1 X70.007 Y64.102 E0.10480
```

```
G1 X70.007 Y64.694 E0.01546
G1 X73.438 Y61.264 E0.12666
G1 X74.030 Y61.264 E0.01546
G1 X70.007 Y65.286 E0.14853
G1 X70.007 Y65.878 E0.01546
G1 X74.622 Y61.264 E0.17039
G1 X75.214 Y61.264 E0.01546
G1 X70.007 Y66.471 E0.19225
G1 X70.007 Y67.063 E0.01546
G1 X75.806 Y61.264 E0.21411
G1 X76.328 Y61.264 E0.01363
G1 X76.344 Y61.318 E0.00148
G1 X70.007 Y67.655 E0.23395
G1 X70.007 Y68.247 E0.01546
G1 X76.513 Y61.741 E0.24022
G1 X76.689 Y62.157 E0.01180
G1 X70.007 Y68.839 E0.24671
M73 P27 R49
G1 X70.007 Y69.431 E0.01546
G1 X76.900 Y62.538 E0.25451
G1 X77.117 Y62.913 E0.01132
G1 X70.007 Y70.023 E0.26250
G1 X70.007 Y70.615 E0.01546
G1 X77.364 Y63.259 E0.27162
G1 X77.618 Y63.596 E0.01104
G1 X70.007 Y71.207 E0.28101
G1 X70.007 Y71.799 E0.01546
G1 X77.897 Y63.909 E0.29132
G1 X78.189 Y64.210 E0.01093
```

G1 X70.007 Y72.391 E0.30207

G1 X70.007 Y72.983 E0.01546

G1 X78.499 Y64.492 E0.31352

G1 X78.827 Y64.756 E0.01099

G1 X70.007 Y73.575 E0.32563

G1 X70.007 Y74.167 E0.01546

G1 X79.169 Y65.006 E0.33826

G1 X79.534 Y65.232 E0.01123

G1 X70.415 Y74.351 E0.33670

G1 X71.007 Y74.351 E0.01546

G1 X79.911 Y65.448 E0.32875

G1 X80.316 Y65.634 E0.01165

G1 X71.599 Y74.351 E0.32186

G1 X72.191 Y74.351 E0.01546

G1 X80.733 Y65.809 E0.31540

G1 X81.183 Y65.952 E0.01230

G1 X72.783 Y74.351 E0.31012

G1 X73.376 Y74.351 E0.01546

G1 X81.649 Y66.078 E0.30549

G1 X82.148 Y66.171 E0.01325

G1 X73.968 Y74.351 E0.30204

G1 X74.560 Y74.351 E0.01546

G1 X82.681 Y66.230 E0.29986

G1 X83.239 Y66.264 E0.01460

G1 X75.152 Y74.351 E0.29862

G1 X75.744 Y74.351 E0.01546

G1 X76.741 Y73.354 E0.03684

G1 X77.160 Y72.627 E0.02190

G1 X77.702 Y71.908 E0.02352

G1 X78.329 Y71.261 E0.02351

G1 X79.030 Y70.696 E0.02351

G1 X79.795 Y70.221 E0.02352

G1 X79.942 Y70.154 E0.00420

G1 X83.831 Y66.264 E0.14362

G1 X84.423 Y66.264 E0.01546

G1 X80.950 Y69.737 E0.12823

G1 X81.472 Y69.571 E0.01428

G1 X81.763 Y69.516 E0.00774

G1 X85.016 Y66.264 E0.12009

G1 X85.608 Y66.264 E0.01546

G1 X82.473 Y69.398 E0.11572

G1 X83.104 Y69.359 E0.01651

G1 X86.200 Y66.264 E0.11428

G1 X86.792 Y66.264 E0.01546

G1 X83.704 Y69.351 E0.11400

G1 X84.296 Y69.351 E0.01546

G1 X87.384 Y66.264 E0.11400

G1 X87.976 Y66.264 E0.01546

G1 X84.888 Y69.351 E0.11400

G1 X85.480 Y69.351 E0.01546

G1 X88.568 Y66.264 E0.11400

G1 X89.160 Y66.264 E0.01546

G1 X86.072 Y69.351 E0.11400

G1 X86.664 Y69.351 E0.01546

G1 X89.752 Y66.264 E0.11400

G1 X90.095 Y66.264 E0.00895

G1 X90.095 Y66.513 E0.00650

G1 X87.256 Y69.351 E0.10481

```
G1 X87.848 Y69.351 E0.01546
G1 X90.095 Y67.105 E0.08295
G1 X90.095 Y67.697 E0.01546
G1 X88.441 Y69.351 E0.06109
G1 X89.033 Y69.351 E0.01546
G1 X90.095 Y68.289 E0.03923
G1 X90.095 Y68.881 E0.01546
G1 X89.437 Y69.539 E0.02431
M204 S1250
; stop printing object tpu print.STL id:5 copy 0
; printing object tpu print.STL id:9 copy 0
G1 E-2.24000 F2400.000
;WIPE_START
G1 F7200.000
G1 X90.095 Y68.881 E-0.29484
G1 X90.095 Y68.289 E-0.18749
G1 X89.136 Y69.249 E-0.42968
;WIPE_END
G1 E-0.04800 F2400.000
G1 Z0.550 F9000.000
G1 X76.558 Y54.716
G1 Z0.350
G1 E3.20000 F1500.000
M204 S800
;TYPE:Perimeter
;WIDTH:0.45
G1 F2400.000
G1 X69.686 Y54.716 E0.17903
G1 X69.686 Y41.002 E0.35734
```

```
G1 X76.558 Y41.002 E0.17903
G1 X76.649 Y41.325 E0.00876
G1 X76.984 Y42.119 E0.02246
G1 X77.414 Y42.866 E0.02246
G1 X77.933 Y43.555 E0.02246
G1 X78.533 Y44.174 E0.02246
G1 X79.204 Y44.715 E0.02246
G1 X79.937 Y45.169 E0.02246
G1 X80.720 Y45.530 E0.02246
G1 X81.541 Y45.791 E0.02246
G1 X82.388 Y45.950 E0.02246
G1 X83.229 Y46.002 E0.02194
G1 X90.401 Y46.002 E0.18688
G1 X90.401 Y49.716 E0.09678
G1 X83.229 Y49.716 E0.18688
G1 X82.388 Y49.768 E0.02194
G1 X81.541 Y49.926 E0.02246
G1 X80.720 Y50.188 E0.02246
G1 X79.937 Y50.549 E0.02246
G1 X79.204 Y51.003 E0.02246
G1 X78.533 Y51.544 E0.02246
G1 X77.933 Y52.163 E0.02246
G1 X77.414 Y52.851 E0.02246
G1 X76.984 Y53.598 E0.02246
G1 X76.649 Y54.392 E0.02246
G1 X76.574 Y54.658 E0.00720
M204 S1250
G1 X76.874 Y55.134 F9000.000
M204 S800
```

```
;TYPE:External perimeter
G1 F1800.000
G1 X69.269 Y55.134 E0.19815
G1 X69.269 Y40.584 E0.37911
G1 X76.874 Y40.584 E0.19815
G1 X77.044 Y41.186 E0.01632
G1 X77.359 Y41.933 E0.02112
G1 X77.764 Y42.635 E0.02112
G1 X78.251 Y43.282 E0.02112
G1 X78.815 Y43.865 E0.02112
G1 X79.446 Y44.373 E0.02112
G1 X80.135 Y44.800 E0.02112
G1 X80.871 Y45.139 E0.02112
G1 X81.643 Y45.385 E0.02112
G1 X82.440 Y45.534 E0.02112
G1 X83.242 Y45.584 E0.02093
G1 X90.819 Y45.584 E0.19743
G1 X90.819 Y50.134 E0.11855
G1 X83.242 Y50.134 E0.19743
G1 X82.440 Y50.183 E0.02093
G1 X81.643 Y50.332 E0.02112
G1 X80.871 Y50.578 E0.02112
G1 X80.135 Y50.917 E0.02112
G1 X79.446 Y51.344 E0.02112
G1 X78.815 Y51.853 E0.02112
G1 X78.251 Y52.435 E0.02112
G1 X77.764 Y53.082 E0.02112
G1 X77.359 Y53.784 E0.02112
G1 X77.044 Y54.531 E0.02112
```

```
G1 X76.890 Y55.076 E0.01476
M204 S1250
G1 X76.552 Y54.895 F9000.000
G1 E-2.24000 F2400.000
;WIPE_START
G1 F7200.000
G1 X74.010 Y55.098 E-0.91200
;WIPE_END
G1 E-0.04800 F2400.000
G1 Z0.550 F9000.000
G1 X70.658 Y41.127
G1 Z0.350
G1 E3.20000 F1500.000
M204 S1000
;TYPE:Solid infill
;WIDTH:0.450839
G1 F4800.000
G1 X70.000 Y41.785 E0.02431
G1 X70.000 Y42.377 E0.01546
G1 X71.062 Y41.315 E0.03922
G1 X71.654 Y41.315 E0.01546
G1 X70.000 Y42.969 E0.06108
G1 X70.000 Y43.561 E0.01546
G1 X72.246 Y41.315 E0.08294
G1 X72.838 Y41.315 E0.01546
G1 X70.000 Y44.153 E0.10480
G1 X70.000 Y44.745 E0.01546
G1 X73.430 Y41.315 E0.12666
G1 X74.022 Y41.315 E0.01546
```

G1 X70.000 Y45.337 E0.14853

G1 X70.000 Y45.930 E0.01546

G1 X74.615 Y41.315 E0.17039

G1 X75.207 Y41.315 E0.01546

G1 X70.000 Y46.522 E0.19225

G1 X70.000 Y47.114 E0.01546

G1 X75.799 Y41.315 E0.21411

G1 X76.321 Y41.315 E0.01363

G1 X76.336 Y41.369 E0.00148

G1 X70.000 Y47.706 E0.23395

G1 X70.000 Y48.298 E0.01546

G1 X76.506 Y41.792 E0.24022

G1 X76.682 Y42.208 E0.01180

G1 X70.000 Y48.890 E0.24671

G1 X70.000 Y49.482 E0.01546

G1 X76.893 Y42.589 E0.25451

G1 X77.109 Y42.964 E0.01132

G1 X70.000 Y50.074 E0.26250

G1 X70.000 Y50.666 E0.01546

G1 X77.356 Y43.310 E0.27162

G1 X77.611 Y43.647 E0.01104

G1 X70.000 Y51.258 E0.28101

G1 X70.000 Y51.850 E0.01546

G1 X77.890 Y43.960 E0.29132

G1 X78.181 Y44.261 E0.01093

G1 X70.000 Y52.442 E0.30207

G1 X70.000 Y53.034 E0.01546

G1 X78.491 Y44.543 E0.31352

G1 X78.819 Y44.807 E0.01099

```
G1 X70.000 Y53.626 E0.32563
G1 X70.000 Y54.218 E0.01546
G1 X79.161 Y45.057 E0.33826
G1 X79.527 Y45.283 E0.01123
G1 X70.408 Y54.403 E0.33670
G1 X71.000 Y54.403 E0.01546
G1 X79.904 Y45.499 E0.32875
G1 X80.309 Y45.685 E0.01165
G1 X71.592 Y54.403 E0.32186
G1 X72.184 Y54.403 E0.01546
G1 X80.726 Y45.860 E0.31540
G1 X81.175 Y46.003 E0.01230
G1 X72.776 Y54.403 E0.31012
G1 X73.368 Y54.403 E0.01546
G1 X81.642 Y46.129 E0.30549
G1 X82.141 Y46.222 E0.01325
G1 X73.960 Y54.403 E0.30204
G1 X74.552 Y54.403 E0.01546
G1 X82.673 Y46.281 E0.29986
G1 X83.232 Y46.315 E0.01460
G1 X75.144 Y54.403 E0.29862
G1 X75.736 Y54.403 E0.01546
G1 X76.734 Y53.405 E0.03684
G1 X77.153 Y52.678 E0.02190
G1 X77.695 Y51.959 E0.02352
G1 X78.321 Y51.312 E0.02351
G1 X79.022 Y50.747 E0.02351
G1 X79.788 Y50.272 E0.02352
G1 X79.934 Y50.205 E0.00420
```

```
G1 X83.824 Y46.315 E0.14362
G1 X84.416 Y46.315 E0.01546
G1 X80.943 Y49.788 E0.12823
G1 X81.464 Y49.622 E0.01428
G1 X81.756 Y49.567 E0.00774
G1 X85.008 Y46.315 E0.12009
G1 X85.600 Y46.315 E0.01546
G1 X82.466 Y49.449 E0.11572
G1 X83.097 Y49.410 E0.01651
G1 X86.192 Y46.315 E0.11428
G1 X86.784 Y46.315 E0.01546
G1 X83.697 Y49.403 E0.11400
G1 X84.289 Y49.403 E0.01546
G1 X87.376 Y46.315 E0.11400
G1 X87.968 Y46.315 E0.01546
G1 X84.881 Y49.403 E0.11400
G1 X85.473 Y49.403 E0.01546
G1 X88.560 Y46.315 E0.11400
G1 X89.152 Y46.315 E0.01546
G1 X86.065 Y49.403 E0.11400
G1 X86.657 Y49.403 E0.01546
G1 X89.745 Y46.315 E0.11400
G1 X90.087 Y46.315 E0.00895
G1 X90.087 Y46.564 E0.00650
G1 X87.249 Y49.403 E0.10481
G1 X87.841 Y49.403 E0.01546
G1 X90.087 Y47.156 E0.08295
G1 X90.087 Y47.748 E0.01546
G1 X88.433 Y49.403 E0.06109
```

```
G1 X89.025 Y49.403 E0.01546
G1 X90.087 Y48.340 E0.03923
G1 X90.087 Y48.932 E0.01546
G1 X89.429 Y49.591 E0.02431
M204 S1250
; stop printing object tpu print.STL id:9 copy 0
; printing object Petg print.STL id:8 copy 0
; stop printing object Petg print.STL id:8 copy 0
; printing object Petg print.STL id:4 copy 0
; stop printing object Petg print.STL id:4 copy 0
; printing object tpu print.STL id:29 copy 0
G1 E-2.24000 F2400.000
;WIPE_START
G1 F7200.000
G1 X90.087 Y48.932 E-0.29484
G1 X90.087 Y48.340 E-0.18749
G1 X89.128 Y49.300 E-0.42968
;WIPE_END
G1 E-0.04800 F2400.000
G1 Z0.550 F9000.000
G1 X120.337 Y54.891
G1 Z0.350
G1 E3.20000 F1500.000
M204 S800
;TYPE:Perimeter
;WIDTH:0.45
G1 F2400.000
G1 X120.337 Y41.177 E0.35734
G1 X127.208 Y41.177 E0.17903
```

G1 X127.299 Y41.500 E0.00876

G1 X127.634 Y42.294 E0.02246

G1 X128.065 Y43.041 E0.02246

M73 P28 R49

G1 X128.583 Y43.730 E0.02246

G1 X129.183 Y44.349 E0.02246

G1 X129.854 Y44.890 E0.02246

G1 X130.587 Y45.344 E0.02246

G1 X131.370 Y45.705 E0.02246

G1 X132.191 Y45.966 E0.02246

G1 X133.038 Y46.125 E0.02246

G1 X133.879 Y46.177 E0.02194

G1 X141.051 Y46.177 E0.18688

G1 X141.051 Y49.891 E0.09678

G1 X133.879 Y49.891 E0.18688

G1 X133.038 Y49.943 E0.02194

G1 X132.191 Y50.101 E0.02246

G1 X131.370 Y50.363 E0.02246

G1 X130.587 Y50.724 E0.02246

G1 X129.854 Y51.178 E0.02246

G1 X129.183 Y51.719 E0.02246

G1 X128.583 Y52.338 E0.02246

G1 X128.065 Y53.026 E0.02246

G1 X127.634 Y53.773 E0.02246

G1 X127.299 Y54.567 E0.02246

G1 X127.208 Y54.891 E0.00876

G1 X120.397 Y54.891 E0.17747

M204 S1250

G1 X119.919 Y55.309 F9000.000

```
M204 S800
;TYPE:External perimeter
G1 F1800.000
G1 X119.919 Y40.759 E0.37911
G1 X127.524 Y40.759 E0.19815
G1 X127.694 Y41.361 E0.01632
G1 X128.009 Y42.108 E0.02112
G1 X128.414 Y42.810 E0.02112
G1 X128.902 Y43.458 E0.02112
G1 X129.465 Y44.040 E0.02112
G1 X130.096 Y44.548 E0.02112
G1 X130.785 Y44.975 E0.02112
G1 X131.521 Y45.314 E0.02112
G1 X132.293 Y45.560 E0.02112
G1 X133.090 Y45.709 E0.02112
G1 X133.892 Y45.759 E0.02093
G1 X141.469 Y45.759 E0.19743
G1 X141.469 Y50.309 E0.11855
G1 X133.892 Y50.309 E0.19743
G1 X133.090 Y50.358 E0.02093
G1 X132.293 Y50.507 E0.02112
G1 X131.521 Y50.753 E0.02112
G1 X130.785 Y51.092 E0.02112
G1 X130.096 Y51.519 E0.02112
G1 X129.465 Y52.028 E0.02112
G1 X128.902 Y52.610 E0.02112
G1 X128.414 Y53.257 E0.02112
G1 X128.009 Y53.959 E0.02112
G1 X127.694 Y54.706 E0.02112
```

```
G1 X127.524 Y55.309 E0.01632
G1 X119.979 Y55.309 E0.19659
M204 S1250
G1 X120.110 Y54.957 F9000.000
G1 E-2.24000 F2400.000
;WIPE_START
G1 F7200.000
G1 X119.967 Y52.429 E-0.91200
;WIPE_END
G1 E-0.04800 F2400.000
G1 Z0.550 F9000.000
G1 X140.079 Y49.766
G1 Z0.350
G1 E3.20000 F1500.000
M204 S1000
;TYPE:Solid infill
;WIDTH:0.450839
G1 F4800.000
G1 X140.738 Y49.107 E0.02431
G1 X140.738 Y48.515 E0.01546
G1 X139.675 Y49.578 E0.03923
G1 X139.083 Y49.578 E0.01546
G1 X140.738 Y47.923 E0.06109
G1 X140.738 Y47.331 E0.01546
G1 X138.491 Y49.578 E0.08295
G1 X137.899 Y49.578 E0.01546
G1 X140.738 Y46.739 E0.10481
G1 X140.738 Y46.490 E0.00650
G1 X140.395 Y46.490 E0.00895
```

G1 X137.307 Y49.578 E0.11400

G1 X136.715 Y49.578 E0.01546

G1 X139.803 Y46.490 E0.11400

G1 X139.211 Y46.490 E0.01546

G1 X136.123 Y49.578 E0.11400

G1 X135.531 Y49.578 E0.01546

G1 X138.619 Y46.490 E0.11400

G1 X138.027 Y46.490 E0.01546

G1 X134.939 Y49.578 E0.11400

G1 X134.347 Y49.578 E0.01546

G1 X137.434 Y46.490 E0.11400

G1 X136.842 Y46.490 E0.01546

G1 X133.747 Y49.585 E0.11428

G1 X133.116 Y49.624 E0.01651

G1 X136.250 Y46.490 E0.11572

G1 X135.658 Y46.490 E0.01546

G1 X132.406 Y49.742 E0.12009

G1 X132.114 Y49.797 E0.00774

G1 X131.593 Y49.963 E0.01428

G1 X135.066 Y46.490 E0.12823

G1 X134.474 Y46.490 E0.01546

G1 X130.584 Y50.380 E0.14362

G1 X130.438 Y50.447 E0.00420

G1 X129.673 Y50.922 E0.02352

G1 X128.971 Y51.487 E0.02351

G1 X128.345 Y52.134 E0.02351

G1 X127.803 Y52.853 E0.02352

G1 X127.384 Y53.580 E0.02190

G1 X126.386 Y54.578 E0.03684

G1 X125.794 Y54.578 E0.01546

G1 X133.882 Y46.490 E0.29862

G1 X133.324 Y46.456 E0.01460

G1 X125.202 Y54.578 E0.29986

G1 X124.610 Y54.578 E0.01546

G1 X132.791 Y46.397 E0.30204

G1 X132.292 Y46.304 E0.01325

G1 X124.018 Y54.578 E0.30549

G1 X123.426 Y54.578 E0.01546

G1 X131.825 Y46.179 E0.31012

G1 X131.376 Y46.036 E0.01230

G1 X122.834 Y54.578 E0.31540

G1 X122.242 Y54.578 E0.01546

G1 X130.959 Y45.860 E0.32186

G1 X130.554 Y45.674 E0.01165

G1 X121.650 Y54.578 E0.32875

G1 X121.058 Y54.578 E0.01546

G1 X130.177 Y45.459 E0.33670

G1 X129.812 Y45.232 E0.01123

G1 X120.650 Y54.393 E0.33826

G1 X120.650 Y53.801 E0.01546

G1 X129.469 Y44.982 E0.32563

G1 X129.142 Y44.718 E0.01099

G1 X120.650 Y53.209 E0.31352

G1 X120.650 Y52.617 E0.01546

G1 X128.831 Y44.436 E0.30207

G1 X128.540 Y44.135 E0.01093

G1 X120.650 Y52.025 E0.29132

G1 X120.650 Y51.433 E0.01546

```
G1 X128.261 Y43.822 E0.28101
G1 X128.006 Y43.485 E0.01104
G1 X120.650 Y50.841 E0.27162
G1 X120.650 Y50.249 E0.01546
G1 X127.760 Y43.139 E0.26250
G1 X127.543 Y42.764 E0.01132
G1 X120.650 Y49.657 E0.25451
G1 X120.650 Y49.065 E0.01546
G1 X127.332 Y42.383 E0.24671
G1 X127.156 Y41.967 E0.01180
G1 X120.650 Y48.473 E0.24022
G1 X120.650 Y47.881 E0.01546
G1 X126.986 Y41.545 E0.23395
G1 X126.971 Y41.490 E0.00148
G1 X126.449 Y41.490 E0.01363
G1 X120.650 Y47.289 E0.21411
G1 X120.650 Y46.697 E0.01546
G1 X125.857 Y41.490 E0.19225
G1 X125.265 Y41.490 E0.01546
G1 X120.650 Y46.105 E0.17039
G1 X120.650 Y45.513 E0.01546
G1 X124.673 Y41.490 E0.14853
G1 X124.081 Y41.490 E0.01546
G1 X120.650 Y44.921 E0.12666
G1 X120.650 Y44.328 E0.01546
G1 X123.489 Y41.490 E0.10480
G1 X122.897 Y41.490 E0.01546
G1 X120.650 Y43.736 E0.08294
G1 X120.650 Y43.144 E0.01546
```

```
G1 X122.304 Y41.490 E0.06108
G1 X121.712 Y41.490 E0.01546
G1 X120.650 Y42.552 E0.03922
G1 X120.650 Y41.960 E0.01546
G1 X121.308 Y41.302 E0.02431
M204 S1250
; stop printing object tpu print.STL id:29 copy 0
; printing object tpu print.STL id:25 copy 0
G1 E-2.24000 F2400.000
;WIPE_START
G1 F7200.000
G1 X120.650 Y41.960 E-0.29480
G1 X120.650 Y42.552 E-0.18749
G1 X121.610 Y41.593 E-0.42971
;WIPE_END
G1 E-0.04800 F2400.000
G1 Z0.550 F9000.000
G1 X120.344 Y61.126
G1 Z0.350
G1 E3.20000 F1500.000
M204 S800
;TYPE:Perimeter
;WIDTH:0.45
G1 F2400.000
G1 X127.215 Y61.126 E0.17903
G1 X127.307 Y61.449 E0.00876
G1 X127.642 Y62.243 E0.02246
G1 X128.072 Y62.990 E0.02246
G1 X128.591 Y63.679 E0.02246
```

```
G1 X129.191 Y64.298 E0.02246
G1 X129.862 Y64.839 E0.02246
G1 X130.594 Y65.293 E0.02246
G1 X131.377 Y65.654 E0.02246
G1 X132.199 Y65.915 E0.02246
G1 X133.046 Y66.074 E0.02246
G1 X133.886 Y66.126 E0.02194
G1 X141.059 Y66.126 E0.18688
G1 X141.059 Y69.840 E0.09678
G1 X133.886 Y69.840 E0.18688
G1 X133.046 Y69.892 E0.02194
G1 X132.199 Y70.050 E0.02246
G1 X131.377 Y70.312 E0.02246
G1 X130.594 Y70.673 E0.02246
G1 X129.862 Y71.127 E0.02246
G1 X129.191 Y71.668 E0.02246
G1 X128.591 Y72.287 E0.02246
G1 X128.072 Y72.975 E0.02246
G1 X127.642 Y73.722 E0.02246
G1 X127.307 Y74.516 E0.02246
G1 X127.215 Y74.840 E0.00876
G1 X120.344 Y74.840 E0.17903
G1 X120.344 Y61.186 E0.35578
M204 S1250
G1 X119.926 Y60.708 F9000.000
M204 S800
;TYPE:External perimeter
G1 F1800.000
G1 X127.531 Y60.708 E0.19815
```

```
G1 X127.702 Y61.310 E0.01632
G1 X128.017 Y62.057 E0.02112
G1 X128.421 Y62.759 E0.02112
G1 X128.909 Y63.406 E0.02112
G1 X129.473 Y63.989 E0.02112
G1 X130.104 Y64.497 E0.02112
G1 X130.793 Y64.924 E0.02112
G1 X131.529 Y65.263 E0.02112
G1 X132.301 Y65.509 E0.02112
G1 X133.097 Y65.658 E0.02112
G1 X133.899 Y65.708 E0.02093
G1 X141.476 Y65.708 E0.19743
G1 X141.476 Y70.258 E0.11855
G1 X133.899 Y70.258 E0.19743
G1 X133.097 Y70.307 E0.02093
G1 X132.301 Y70.456 E0.02112
G1 X131.529 Y70.702 E0.02112
G1 X130.793 Y71.041 E0.02112
G1 X130.104 Y71.468 E0.02112
G1 X129.473 Y71.977 E0.02112
G1 X128.909 Y72.559 E0.02112
G1 X128.421 Y73.206 E0.02112
G1 X128.017 Y73.908 E0.02112
G1 X127.702 Y74.655 E0.02112
G1 X127.531 Y75.258 E0.01632
G1 X119.926 Y75.258 E0.19815
G1 X119.926 Y60.768 E0.37755
M204 S1250
G1 X120.300 Y60.850 F9000.000
```

```
G1 E-2.24000 F2400.000
;WIPE_START
G1 F7200.000
G1 X122.806 Y60.745 E-0.91200
;WIPE_END
G1 E-0.04800 F2400.000
G1 Z0.550 F9000.000
G1 X140.087 Y69.715
G1 Z0.350
G1 E3.20000 F1500.000
M204 S1000
;TYPE:Solid infill
;WIDTH:0.450839
G1 F4800.000
G1 X140.745 Y69.056 E0.02431
G1 X140.745 Y68.464 E0.01546
G1 X139.683 Y69.527 E0.03923
G1 X139.091 Y69.527 E0.01546
G1 X140.745 Y67.872 E0.06109
G1 X140.745 Y67.280 E0.01546
G1 X138.499 Y69.527 E0.08295
G1 X137.907 Y69.527 E0.01546
G1 X140.745 Y66.688 E0.10481
G1 X140.745 Y66.439 E0.00650
G1 X140.402 Y66.439 E0.00895
G1 X137.315 Y69.527 E0.11400
G1 X136.723 Y69.527 E0.01546
G1 X139.810 Y66.439 E0.11400
G1 X139.218 Y66.439 E0.01546
```

```
G1 X136.130 Y69.527 E0.11400
G1 X135.538 Y69.527 E0.01546
G1 X138.626 Y66.439 E0.11400
G1 X138.034 Y66.439 E0.01546
G1 X134.946 Y69.527 E0.11400
G1 X134.354 Y69.527 E0.01546
G1 X137.442 Y66.439 E0.11400
G1 X136.850 Y66.439 E0.01546
G1 X133.755 Y69.534 E0.11428
G1 X133.124 Y69.573 E0.01651
G1 X136.258 Y66.439 E0.11572
G1 X135.666 Y66.439 E0.01546
G1 X132.413 Y69.691 E0.12009
G1 X132.122 Y69.746 E0.00774
G1 X131.601 Y69.912 E0.01428
G1 X135.074 Y66.439 E0.12823
G1 X134.482 Y66.439 E0.01546
G1 X130.592 Y70.329 E0.14362
G1 X130.446 Y70.396 E0.00420
G1 X129.680 Y70.871 E0.02352
G1 X128.979 Y71.436 E0.02351
G1 X128.352 Y72.083 E0.02351
G1 X127.810 Y72.802 E0.02352
G1 X127.392 Y73.529 E0.02190
G1 X126.394 Y74.527 E0.03684
G1 X125.802 Y74.527 E0.01546
G1 X133.890 Y66.439 E0.29862
G1 X133.331 Y66.405 E0.01460
G1 X125.210 Y74.527 E0.29986
```

```
G1 X124.618 Y74.527 E0.01546
G1 X132.798 Y66.346 E0.30204
G1 X132.300 Y66.253 E0.01325
G1 X124.026 Y74.527 E0.30549
G1 X123.434 Y74.527 E0.01546
G1 X131.833 Y66.127 E0.31012
G1 X131.384 Y65.984 E0.01230
G1 X122.842 Y74.527 E0.31540
G1 X122.250 Y74.527 E0.01546
G1 X130.967 Y65.809 E0.32186
G1 X130.561 Y65.623 E0.01165
G1 X121.658 Y74.527 E0.32875
G1 X121.065 Y74.527 E0.01546
G1 X130.185 Y65.408 E0.33670
G1 X129.819 Y65.181 E0.01123
G1 X120.658 Y74.342 E0.33826
G1 X120.658 Y73.750 E0.01546
G1 X129.477 Y64.931 E0.32563
G1 X129.149 Y64.667 E0.01099
G1 X120.658 Y73.158 E0.31352
G1 X120.658 Y72.566 E0.01546
G1 X128.839 Y64.385 E0.30207
G1 X128.548 Y64.084 E0.01093
G1 X120.658 Y71.974 E0.29132
G1 X120.658 Y71.382 E0.01546
G1 X128.268 Y63.771 E0.28101
G1 X128.014 Y63.434 E0.01104
G1 X120.658 Y70.790 E0.27162
G1 X120.658 Y70.198 E0.01546
```

```
G1 X127.767 Y63.088 E0.26250
G1 X127.551 Y62.713 E0.01132
G1 X120.658 Y69.606 E0.25451
G1 X120.658 Y69.014 E0.01546
G1 X127.339 Y62.332 E0.24671
G1 X127.164 Y61.916 E0.01180
G1 X120.658 Y68.422 E0.24022
G1 X120.658 Y67.830 E0.01546
G1 X126.994 Y61.493 E0.23395
G1 X126.978 Y61.439 E0.00148
G1 X126.456 Y61.439 E0.01363
G1 X120.658 Y67.238 E0.21411
G1 X120.658 Y66.646 E0.01546
G1 X125.864 Y61.439 E0.19225
G1 X125.272 Y61.439 E0.01546
G1 X120.658 Y66.054 E0.17039
G1 X120.658 Y65.462 E0.01546
G1 X124.680 Y61.439 E0.14853
G1 X124.088 Y61.439 E0.01546
G1 X120.658 Y64.869 E0.12666
G1 X120.658 Y64.277 E0.01546
G1 X123.496 Y61.439 E0.10480
G1 X122.904 Y61.439 E0.01546
G1 X120.658 Y63.685 E0.08294
G1 X120.658 Y63.093 E0.01546
G1 X122.312 Y61.439 E0.06108
G1 X121.720 Y61.439 E0.01546
G1 X120.658 Y62.501 E0.03922
G1 X120.658 Y61.909 E0.01546
```

```
G1 X121.316 Y61.251 E0.02431
M204 S1250
; stop printing object tpu print.STL id:25 copy 0
; printing object Petg print.STL id:24 copy 0
; stop printing object Petg print.STL id:24 copy 0
; printing object Petg print.STL id:28 copy 0
; stop printing object Petg print.STL id:28 copy 0
G1 E-2.80000 F2400.000
;WIPE_START
G1 F7200.000
G1 X120.658 Y61.909 E-0.29480
G1 X120.658 Y62.501 E-0.18749
G1 X121.720 Y61.439 E-0.47575
G1 X122.295 Y61.439 E-0.18197
;WIPE_END
G1 E-0.06000 F2400.000
G1 Z0.550 F9000.000
; Filament-specific end gcode
M600
T0
M900 K0 ; Filament gcode
; printing object tpu print.STL id:19 copy 0
; stop printing object tpu print.STL id:19 copy 0
; printing object Petg print.STL id:18 copy 0
G1 X61.611 Y54.135
G1 Z0.350
G1 E4.00000 F900.000
M204 S800
;TYPE:Perimeter
```

```
;WIDTH:0.45
G1 F1292.454
G1 X54.740 Y54.135 E0.20589
G1 X54.649 Y53.812 E0.01007
G1 X54.314 Y53.017 E0.02583
G1 X53.884 Y52.270 E0.02583
G1 X53.365 Y51.582 E0.02583
G1 X52.765 Y50.963 E0.02583
G1 X52.094 Y50.422 E0.02583
G1 X51.361 Y49.968 E0.02583
G1 X50.578 Y49.607 E0.02583
G1 X49.757 Y49.345 E0.02583
G1 X48.910 Y49.187 E0.02583
G1 X48.069 Y49.135 E0.02523
G1 X39.897 Y49.135 E0.24487
G1 X39.897 Y45.421 E0.11130
G1 X48.069 Y45.421 E0.24487
G1 X48.910 Y45.369 E0.02523
G1 X49.757 Y45.210 E0.02583
G1 X50.578 Y44.949 E0.02583
G1 X51.361 Y44.588 E0.02583
G1 X52.094 Y44.134 E0.02583
G1 X52.765 Y43.593 E0.02583
G1 X53.365 Y42.974 E0.02583
G1 X53.884 Y42.285 E0.02583
G1 X54.314 Y41.538 E0.02583
G1 X54.649 Y40.744 E0.02583
G1 X54.740 Y40.421 E0.01007
G1 X61.611 Y40.421 E0.20589
```

```
G1 X61.611 Y54.075 E0.40914
M204 S1250
G1 X62.029 Y54.553 F9000.000
M204 S800
;TYPE:External perimeter
G1 F1292.454
G1 X54.424 Y54.553 E0.22787
G1 X54.254 Y53.950 E0.01877
G1 X53.939 Y53.203 E0.02428
G1 X53.534 Y52.501 E0.02428
G1 X53.047 Y51.854 E0.02428
G1 X52.483 Y51.272 E0.02428
G1 X51.852 Y50.763 E0.02428
G1 X51.163 Y50.336 E0.02428
G1 X50.427 Y49.997 E0.02428
G1 X49.655 Y49.751 E0.02428
G1 X48.858 Y49.602 E0.02428
G1 X48.056 Y49.553 E0.02407
G1 X39.479 Y49.553 E0.25701
G1 X39.479 Y45.003 E0.13634
G1 X48.056 Y45.003 E0.25701
G1 X48.858 Y44.953 E0.02407
G1 X49.655 Y44.804 E0.02428
G1 X50.427 Y44.558 E0.02428
G1 X51.163 Y44.219 E0.02428
G1 X51.852 Y43.792 E0.02428
G1 X52.483 Y43.284 E0.02428
G1 X53.047 Y42.702 E0.02428
G1 X53.534 Y42.054 E0.02428
```

```
G1 X53.939 Y41.352 E0.02428
G1 X54.254 Y40.606 E0.02428
G1 X54.424 Y40.003 E0.01877
G1 X62.029 Y40.003 E0.22787
G1 X62.029 Y54.493 E0.43418
M204 S1250
G1 X61.655 Y54.411 F9000.000
G1 X60.628 Y54.010
M204 S1000
;TYPE:Solid infill
;WIDTH:0.456608
G1 F1272.331
G1 X61.298 Y53.339 E0.02887
G1 X61.298 Y52.739 E0.01827
G1 X60.215 Y53.822 E0.04661
G1 X59.615 Y53.822 E0.01827
G1 X61.298 Y52.139 E0.07245
G1 X61.298 Y51.538 E0.01827
G1 X59.015 Y53.822 E0.09828
G1 X58.415 Y53.822 E0.01827
G1 X61.298 Y50.938 E0.12412
G1 X61.298 Y50.338 E0.01827
G1 X57.814 Y53.822 E0.14996
G1 X57.214 Y53.822 E0.01827
G1 X61.298 Y49.738 E0.17580
G1 X61.298 Y49.138 E0.01827
G1 X56.614 Y53.822 E0.20163
G1 X56.014 Y53.822 E0.01827
G1 X61.298 Y48.537 E0.22747
```

```
G1 X61.298 Y47.937 E0.01827
G1 X55.414 Y53.822 E0.25331
G1 X54.977 Y53.822 E0.01328
G1 X54.940 Y53.695 E0.00402
G1 X61.298 Y47.337 E0.27370
G1 X61.298 Y46.737 E0.01827
G1 X54.762 Y53.273 E0.28136
G1 X54.581 Y52.854 E0.01390
G1 X61.298 Y46.136 E0.28914
M73 P28 R48
G1 X61.298 Y45.536 E0.01827
G1 X54.362 Y52.473 E0.29859
G1 X54.142 Y52.092 E0.01337
G1 X61.298 Y44.936 E0.30805
G1 X61.298 Y44.336 E0.01827
G1 X53.884 Y51.750 E0.31916
G1 X53.626 Y51.408 E0.01305
G1 X61.298 Y43.736 E0.33026
G1 X61.298 Y43.135 E0.01827
G1 X53.334 Y51.100 E0.34283
G1 X53.039 Y50.795 E0.01292
G1 X61.298 Y42.535 E0.35554
M73 P29 R48
G1 X61.298 Y41.935 E0.01827
G1 X52.714 Y50.519 E0.36951
G1 X52.382 Y50.251 E0.01299
G1 X61.298 Y41.335 E0.38382
G1 X61.298 Y40.735 E0.01827
G1 X52.023 Y50.009 E0.39924
```

```
G1 X51.653 Y49.780 E0.01327
G1 X60.698 Y40.734 E0.38938
G1 X60.098 Y40.734 E0.01827
G1 X51.257 Y49.575 E0.38056
G1 X50.846 Y49.385 E0.01377
G1 X59.498 Y40.734 E0.37241
G1 X58.898 Y40.734 E0.01827
G1 X50.408 Y49.224 E0.36545
G1 X49.953 Y49.079 E0.01454
G1 X58.298 Y40.734 E0.35921
G1 X57.697 Y40.734 E0.01827
G1 X49.460 Y48.971 E0.35457
G1 X48.955 Y48.877 E0.01566
G1 X57.097 Y40.734 E0.35050
G1 X56.497 Y40.734 E0.01827
G1 X48.390 Y48.841 E0.34897
G1 X47.809 Y48.822 E0.01769
G1 X55.897 Y40.734 E0.34814
G1 X55.296 Y40.734 E0.01827
G1 X54.413 Y41.618 E0.03805
M204 S1250
G1 E-4.00000 F2400.000
G1 X47.021 Y49.010 F9000.000
G1 E4.00000 F900.000
M204 S1000
G1 F1272.331
G1 X50.872 Y45.158 E0.16578
G1 X50.692 Y45.241 E0.00604
G1 X49.954 Y45.476 E0.02358
```

```
G1 X46.609 Y48.822 E0.14400
G1 X46.008 Y48.822 E0.01827
G1 X49.196 Y45.634 E0.13722
G1 X48.948 Y45.680 E0.00767
G1 X48.523 Y45.707 E0.01297
G1 X45.408 Y48.822 E0.13409
G1 X44.808 Y48.822 E0.01827
G1 X47.896 Y45.734 E0.13291
G1 X47.295 Y45.734 E0.01827
G1 X44.208 Y48.822 E0.13291
G1 X43.607 Y48.822 E0.01827
G1 X46.695 Y45.734 E0.13291
G1 X46.095 Y45.734 E0.01827
G1 X43.007 Y48.822 E0.13291
G1 X42.407 Y48.822 E0.01827
G1 X45.495 Y45.734 E0.13291
G1 X44.894 Y45.734 E0.01827
G1 X41.807 Y48.822 E0.13291
G1 X41.207 Y48.822 E0.01827
G1 X44.294 Y45.734 E0.13291
G1 X43.694 Y45.734 E0.01827
G1 X40.606 Y48.822 E0.13291
G1 X40.210 Y48.822 E0.01205
G1 X40.210 Y48.617 E0.00622
G1 X43.094 Y45.734 E0.12412
G1 X42.494 Y45.734 E0.01827
G1 X40.210 Y48.017 E0.09828
G1 X40.210 Y47.417 E0.01827
G1 X41.893 Y45.734 E0.07244
```

```
G1 X41.293 Y45.734 E0.01827
G1 X40.210 Y46.817 E0.04661
G1 X40.210 Y46.217 E0.01827
G1 X40.881 Y45.546 E0.02886
M204 S1250
; stop printing object Petg print.STL id:18 copy 0
; printing object Petg print.STL id:14 copy 0
G1 E-4.00000 F2400.000
G1 X39.905 Y65.370 F9000.000
G1 E4.00000 F900.000
M204 S800
;TYPE:Perimeter
;WIDTH:0.45
G1 F1292.454
G1 X48.077 Y65.370 E0.24487
G1 X48.917 Y65.318 E0.02523
G1 X49.765 Y65.159 E0.02583
G1 X50.586 Y64.898 E0.02583
G1 X51.369 Y64.537 E0.02583
G1 X52.101 Y64.083 E0.02583
G1 X52.773 Y63.542 E0.02583
G1 X53.372 Y62.923 E0.02583
G1 X53.891 Y62.234 E0.02583
G1 X54.321 Y61.487 E0.02583
G1 X54.656 Y60.693 E0.02583
G1 X54.748 Y60.370 E0.01007
G1 X61.619 Y60.370 E0.20589
G1 X61.619 Y74.084 E0.41094
G1 X54.748 Y74.084 E0.20589
```

```
G1 X54.656 Y73.760 E0.01007
G1 X54.321 Y72.966 E0.02583
G1 X53.891 Y72.219 E0.02583
G1 X53.372 Y71.531 E0.02583
G1 X52.773 Y70.912 E0.02583
G1 X52.101 Y70.371 E0.02583
G1 X51.369 Y69.917 E0.02583
G1 X50.586 Y69.556 E0.02583
G1 X49.765 Y69.294 E0.02583
G1 X48.917 Y69.136 E0.02583
G1 X48.077 Y69.084 E0.02523
G1 X39.905 Y69.084 E0.24487
G1 X39.905 Y65.430 E0.10950
M204 S1250
G1 X39.487 Y64.952 F9000.000
M204 S800
;TYPE:External perimeter
G1 F1292.454
G1 X48.064 Y64.952 E0.25701
G1 X48.866 Y64.902 E0.02407
G1 X49.662 Y64.753 E0.02428
G1 X50.435 Y64.507 E0.02428
G1 X51.171 Y64.168 E0.02428
G1 X51.859 Y63.741 E0.02428
G1 X52.490 Y63.233 E0.02428
G1 X53.054 Y62.651 E0.02428
G1 X53.542 Y62.003 E0.02428
G1 X53.946 Y61.301 E0.02428
G1 X54.261 Y60.554 E0.02428
```

```
G1 X54.432 Y59.952 E0.01877
G1 X62.037 Y59.952 E0.22787
G1 X62.037 Y74.502 E0.43598
G1 X54.432 Y74.502 E0.22787
G1 X54.261 Y73.899 E0.01877
G1 X53.946 Y73.152 E0.02428
G1 X53.542 Y72.450 E0.02428
G1 X53.054 Y71.803 E0.02428
G1 X52.490 Y71.221 E0.02428
G1 X51.859 Y70.712 E0.02428
G1 X51.171 Y70.285 E0.02428
G1 X50.435 Y69.946 E0.02428
G1 X49.662 Y69.700 E0.02428
G1 X48.866 Y69.551 E0.02428
G1 X48.064 Y69.502 E0.02407
G1 X39.487 Y69.502 E0.25701
G1 X39.487 Y65.012 E0.13454
M204 S1250
G1 X39.882 Y65.017 F9000.000
G1 X40.888 Y65.495
M204 S1000
;TYPE:Solid infill
;WIDTH:0.456608
G1 F1272.331
G1 X40.218 Y66.165 E0.02886
G1 X40.218 Y66.766 E0.01827
G1 X41.301 Y65.683 E0.04661
G1 X41.901 Y65.683 E0.01827
G1 X40.218 Y67.366 E0.07244
```

```
G1 X40.218 Y67.966 E0.01827
G1 X42.501 Y65.683 E0.09828
G1 X43.101 Y65.683 E0.01827
G1 X40.218 Y68.566 E0.12412
G1 X40.218 Y68.771 E0.00622
G1 X40.614 Y68.771 E0.01205
G1 X43.702 Y65.683 E0.13291
G1 X44.302 Y65.683 E0.01827
G1 X41.214 Y68.771 E0.13291
G1 X41.814 Y68.771 E0.01827
G1 X44.902 Y65.683 E0.13291
G1 X45.502 Y65.683 E0.01827
G1 X42.415 Y68.771 E0.13291
G1 X43.015 Y68.771 E0.01827
G1 X46.102 Y65.683 E0.13291
G1 X46.703 Y65.683 E0.01827
G1 X43.615 Y68.771 E0.13291
G1 X44.215 Y68.771 E0.01827
G1 X47.303 Y65.683 E0.13291
G1 X47.903 Y65.683 E0.01827
G1 X44.815 Y68.771 E0.13291
G1 X45.416 Y68.771 E0.01827
G1 X48.531 Y65.656 E0.13409
G1 X48.956 Y65.629 E0.01297
G1 X49.204 Y65.583 E0.00767
G1 X46.016 Y68.771 E0.13722
G1 X46.616 Y68.771 E0.01827
G1 X49.961 Y65.425 E0.14400
G1 X50.699 Y65.190 E0.02358
```

```
G1 X50.880 Y65.107 E0.00604
G1 X47.028 Y68.959 E0.16578
M204 S1250
G1 E-4.00000 F2400.000
G1 X54.420 Y61.567 F9000.000
G1 E4.00000 F900.000
M204 S1000
G1 F1272.331
G1 X55.304 Y60.683 E0.03805
G1 X55.904 Y60.683 E0.01827
G1 X47.816 Y68.771 E0.34814
G1 X48.398 Y68.790 E0.01769
G1 X56.504 Y60.683 E0.34897
G1 X57.105 Y60.683 E0.01827
G1 X48.962 Y68.825 E0.35050
G1 X49.468 Y68.920 E0.01566
G1 X57.705 Y60.683 E0.35457
G1 X58.305 Y60.683 E0.01827
G1 X49.960 Y69.028 E0.35921
G1 X50.415 Y69.173 E0.01454
G1 X58.905 Y60.683 E0.36545
G1 X59.505 Y60.683 E0.01827
G1 X50.854 Y69.334 E0.37241
G1 X51.265 Y69.524 E0.01377
G1 X60.106 Y60.683 E0.38056
G1 X60.706 Y60.683 E0.01827
G1 X51.660 Y69.729 E0.38938
G1 X52.031 Y69.958 E0.01327
G1 X61.306 Y60.683 E0.39924
```

```
G1 X61.306 Y61.284 E0.01827
G1 X52.389 Y70.200 E0.38382
G1 X52.722 Y70.468 E0.01299
G1 X61.306 Y61.884 E0.36951
G1 X61.306 Y62.484 E0.01827
G1 X53.046 Y70.744 E0.35554
G1 X53.341 Y71.049 E0.01292
G1 X61.306 Y63.084 E0.34283
G1 X61.306 Y63.685 E0.01827
G1 X53.633 Y71.357 E0.33026
G1 X53.891 Y71.699 E0.01305
G1 X61.306 Y64.285 E0.31916
G1 X61.306 Y64.885 E0.01827
G1 X54.149 Y72.041 E0.30805
G1 X54.369 Y72.422 E0.01337
G1 X61.306 Y65.485 E0.29859
G1 X61.306 Y66.085 E0.01827
G1 X54.589 Y72.803 E0.28914
G1 X54.769 Y73.222 E0.01390
G1 X61.306 Y66.686 E0.28136
G1 X61.306 Y67.286 E0.01827
G1 X54.947 Y73.644 E0.27370
G1 X54.985 Y73.771 E0.00402
G1 X55.421 Y73.771 E0.01328
G1 X61.306 Y67.886 E0.25331
G1 X61.306 Y68.486 E0.01827
G1 X56.021 Y73.771 E0.22747
G1 X56.622 Y73.771 E0.01827
G1 X61.306 Y69.087 E0.20163
```

```
G1 X61.306 Y69.687 E0.01827
G1 X57.222 Y73.771 E0.17580
G1 X57.822 Y73.771 E0.01827
G1 X61.306 Y70.287 E0.14996
G1 X61.306 Y70.887 E0.01827
G1 X58.422 Y73.771 E0.12412
G1 X59.022 Y73.771 E0.01827
G1 X61.306 Y71.487 E0.09828
G1 X61.306 Y72.088 E0.01827
G1 X59.623 Y73.771 E0.07245
G1 X60.223 Y73.771 E0.01827
G1 X61.306 Y72.688 E0.04661
G1 X61.306 Y73.288 E0.01827
G1 X60.635 Y73.959 E0.02887
M204 S1250
; stop printing object Petg print.STL id:14 copy 0
; printing object tpu print.STL id:15 copy 0
; stop printing object tpu print.STL id:15 copy 0
; printing object Petg print.STL id:10 copy 0
G1 E-4.00000 F2400.000
G1 X61.568 Y82.562 F9000.000
G1 E4.00000 F900.000
M204 S800
;TYPE:Perimeter
;WIDTH:0.45
G1 F1292.454
G1 X61.568 Y96.276 E0.41094
G1 X54.697 Y96.276 E0.20589
G1 X54.605 Y95.953 E0.01007
```

```
G1 X54.270 Y95.159 E0.02583
G1 X53.840 Y94.412 E0.02583
G1 X53.321 Y93.723 E0.02583
G1 X52.722 Y93.104 E0.02583
G1 X52.050 Y92.563 E0.02583
G1 X51.318 Y92.109 E0.02583
G1 X50.535 Y91.748 E0.02583
G1 X49.713 Y91.487 E0.02583
G1 X48.866 Y91.328 E0.02583
G1 X48.026 Y91.276 E0.02523
G1 X39.853 Y91.276 E0.24487
G1 X39.853 Y87.562 E0.11130
G1 X48.026 Y87.562 E0.24487
M73 P30 R48
G1 X48.866 Y87.510 E0.02523
G1 X49.713 Y87.351 E0.02583
G1 X50.535 Y87.090 E0.02583
G1 X51.318 Y86.729 E0.02583
G1 X52.050 Y86.275 E0.02583
G1 X52.722 Y85.734 E0.02583
G1 X53.321 Y85.115 E0.02583
G1 X53.840 Y84.427 E0.02583
G1 X54.270 Y83.680 E0.02583
G1 X54.605 Y82.885 E0.02583
G1 X54.697 Y82.562 E0.01007
G1 X61.508 Y82.562 E0.20409
M204 S1250
G1 X61.986 Y82.144 F9000.000
M204 S800
```

```
;TYPE:External perimeter
G1 F1292.454
G1 X61.986 Y96.694 E0.43598
G1 X54.381 Y96.694 E0.22787
G1 X54.210 Y96.091 E0.01877
G1 X53.895 Y95.345 E0.02428
G1 X53.491 Y94.643 E0.02428
G1 X53.003 Y93.995 E0.02428
G1 X52.439 Y93.413 E0.02428
G1 X51.808 Y92.905 E0.02428
G1 X51.119 Y92.478 E0.02428
G1 X50.383 Y92.139 E0.02428
G1 X49.611 Y91.893 E0.02428
G1 X48.815 Y91.744 E0.02428
G1 X48.013 Y91.694 E0.02407
G1 X39.436 Y91.694 E0.25701
G1 X39.436 Y87.144 E0.13634
G1 X48.013 Y87.144 E0.25701
G1 X48.815 Y87.095 E0.02407
G1 X49.611 Y86.946 E0.02428
G1 X50.383 Y86.700 E0.02428
G1 X51.119 Y86.361 E0.02428
G1 X51.808 Y85.934 E0.02428
G1 X52.439 Y85.425 E0.02428
G1 X53.003 Y84.843 E0.02428
G1 X53.491 Y84.196 E0.02428
G1 X53.895 Y83.493 E0.02428
G1 X54.210 Y82.747 E0.02428
G1 X54.381 Y82.144 E0.01877
```

```
G1 X61.926 Y82.144 E0.22608
M204 S1250
G1 X61.795 Y82.496 F9000.000
G1 E-4.00000 F2400.000
G1 X54.369 Y83.759 F9000.000
G1 E4.00000 F900.000
M204 S1000
;TYPE:Solid infill
;WIDTH:0.456608
G1 F1272.331
G1 X55.253 Y82.875 E0.03805
G1 X55.853 Y82.875 E0.01827
G1 X47.765 Y90.963 E0.34814
G1 X48.346 Y90.982 E0.01769
G1 X56.453 Y82.875 E0.34897
G1 X57.053 Y82.875 E0.01827
G1 X48.911 Y91.018 E0.35050
G1 X49.417 Y91.112 E0.01566
G1 X57.654 Y82.875 E0.35457
G1 X58.254 Y82.875 E0.01827
G1 X49.909 Y91.220 E0.35921
G1 X50.364 Y91.365 E0.01454
G1 X58.854 Y82.875 E0.36545
G1 X59.454 Y82.875 E0.01827
G1 X50.803 Y91.527 E0.37241
G1 X51.214 Y91.716 E0.01377
G1 X60.055 Y82.875 E0.38056
G1 X60.655 Y82.875 E0.01827
G1 X51.609 Y91.921 E0.38938
```

```
G1 X51.980 Y92.151 E0.01327
G1 X61.254 Y82.876 E0.39924
G1 X61.254 Y83.476 E0.01827
G1 X52.338 Y92.393 E0.38382
G1 X52.670 Y92.660 E0.01299
G1 X61.254 Y84.076 E0.36951
G1 X61.254 Y84.676 E0.01827
G1 X52.995 Y92.936 E0.35554
G1 X53.290 Y93.241 E0.01292
G1 X61.254 Y85.277 E0.34283
G1 X61.254 Y85.877 E0.01827
G1 X53.582 Y93.549 E0.33026
G1 X53.840 Y93.891 E0.01305
G1 X61.254 Y86.477 E0.31916
G1 X61.254 Y87.077 E0.01827
G1 X54.098 Y94.234 E0.30805
G1 X54.318 Y94.614 E0.01337
G1 X61.254 Y87.678 E0.29859
G1 X61.254 Y88.278 E0.01827
G1 X54.537 Y94.995 E0.28914
G1 X54.718 Y95.414 E0.01390
G1 X61.254 Y88.878 E0.28136
M73 P30 R47
G1 X61.254 Y89.478 E0.01827
G1 X54.896 Y95.837 E0.27370
G1 X54.934 Y95.963 E0.00402
G1 X55.370 Y95.963 E0.01328
G1 X61.254 Y90.078 E0.25331
G1 X61.254 Y90.679 E0.01827
```

```
G1 X55.970 Y95.963 E0.22747
G1 X56.570 Y95.963 E0.01827
G1 X61.254 Y91.279 E0.20163
G1 X61.254 Y91.879 E0.01827
G1 X57.171 Y95.963 E0.17580
G1 X57.771 Y95.963 E0.01827
G1 X61.254 Y92.479 E0.14996
G1 X61.254 Y93.080 E0.01827
G1 X58.371 Y95.963 E0.12412
G1 X58.971 Y95.963 E0.01827
G1 X61.254 Y93.680 E0.09828
G1 X61.254 Y94.280 E0.01827
G1 X59.571 Y95.963 E0.07245
G1 X60.172 Y95.963 E0.01827
G1 X61.254 Y94.880 E0.04661
G1 X61.254 Y95.480 E0.01827
G1 X60.584 Y96.151 E0.02887
M204 S1250
G1 E-4.00000 F2400.000
G1 X46.977 Y91.151 F9000.000
G1 E4.00000 F900.000
M204 S1000
G1 F1272.331
G1 X50.828 Y87.300 E0.16578
G1 X50.648 Y87.383 E0.00604
G1 X49.910 Y87.618 E0.02358
G1 X46.565 Y90.963 E0.14400
G1 X45.965 Y90.963 E0.01827
G1 X49.152 Y87.775 E0.13722
```

G1 X48.905 Y87.822 E0.00767

G1 X48.480 Y87.848 E0.01297

G1 X45.364 Y90.963 E0.13409

G1 X44.764 Y90.963 E0.01827

G1 X47.852 Y87.875 E0.13291

G1 X47.252 Y87.875 E0.01827

G1 X44.164 Y90.963 E0.13291

G1 X43.564 Y90.963 E0.01827

G1 X46.651 Y87.875 E0.13291

G1 X46.051 Y87.875 E0.01827

G1 X42.964 Y90.963 E0.13291

G1 X42.363 Y90.963 E0.01827

G1 X45.451 Y87.875 E0.13291

G1 X44.851 Y87.875 E0.01827

G1 X41.763 Y90.963 E0.13291

G1 X41.163 Y90.963 E0.01827

G1 X44.251 Y87.875 E0.13291

G1 X43.650 Y87.875 E0.01827

G1 X40.563 Y90.963 E0.13291

G1 X40.167 Y90.963 E0.01205

G1 X40.167 Y90.759 E0.00622

G1 X43.050 Y87.875 E0.12412

G1 X42.450 Y87.875 E0.01827

G1 X40.167 Y90.158 E0.09828

G1 X40.167 Y89.558 E0.01827

G1 X41.850 Y87.875 E0.07244

G1 X41.250 Y87.875 E0.01827

G1 X40.167 Y88.958 E0.04661

G1 X40.167 Y88.358 E0.01827

```
G1 X40.837 Y87.687 E0.02886
M204 S1250
; stop printing object Petg print.STL id:10 copy 0
; printing object tpu print.STL id:11 copy 0
; stop printing object tpu print.STL id:11 copy 0
; printing object tpu print.STL id:13 copy 0
; stop printing object tpu print.STL id:13 copy 0
; printing object tpu print.STL id:17 copy 0
; stop printing object tpu print.STL id:17 copy 0
; printing object Petg print.STL id:16 copy 0
G1 E-4.00000 F2400.000
G1 X54.688 Y122.031 F9000.000
G1 E4.00000 F900.000
M204 S800
;TYPE:Perimeter
;WIDTH:0.45
G1 F1292.454
G1 X61.560 Y122.031 E0.20589
G1 X61.560 Y135.746 E0.41094
G1 X54.688 Y135.746 E0.20589
G1 X54.597 Y135.422 E0.01007
G1 X54.262 Y134.628 E0.02583
G1 X53.832 Y133.881 E0.02583
G1 X53.313 Y133.193 E0.02583
G1 X52.713 Y132.573 E0.02583
G1 X52.042 Y132.033 E0.02583
G1 X51.309 Y131.578 E0.02583
G1 X50.526 Y131.218 E0.02583
G1 X49.705 Y130.956 E0.02583
```

```
G1 X48.858 Y130.798 E0.02583
G1 X48.017 Y130.746 E0.02523
G1 X39.845 Y130.746 E0.24487
G1 X39.845 Y127.031 E0.11130
G1 X48.017 Y127.031 E0.24487
G1 X48.858 Y126.980 E0.02523
G1 X49.705 Y126.821 E0.02583
G1 X50.526 Y126.559 E0.02583
G1 X51.309 Y126.199 E0.02583
G1 X52.042 Y125.745 E0.02583
G1 X52.713 Y125.204 E0.02583
G1 X53.313 Y124.584 E0.02583
G1 X53.832 Y123.896 E0.02583
G1 X54.262 Y123.149 E0.02583
G1 X54.597 Y122.355 E0.02583
G1 X54.672 Y122.089 E0.00828
M204 S1250
G1 X54.373 Y121.614 F9000.000
M204 S800
;TYPE:External perimeter
G1 F1292.454
G1 X61.977 Y121.614 E0.22787
G1 X61.977 Y136.164 E0.43598
G1 X54.373 Y136.164 E0.22787
G1 X54.202 Y135.561 E0.01877
G1 X53.887 Y134.814 E0.02428
G1 X53.482 Y134.112 E0.02428
G1 X52.995 Y133.465 E0.02428
G1 X52.431 Y132.883 E0.02428
```

```
G1 X51.800 Y132.374 E0.02428
G1 X51.111 Y131.947 E0.02428
G1 X50.375 Y131.608 E0.02428
G1 X49.603 Y131.362 E0.02428
G1 X48.806 Y131.213 E0.02428
G1 X48.004 Y131.164 E0.02407
G1 X39.427 Y131.164 E0.25701
G1 X39.427 Y126.614 E0.13634
G1 X48.004 Y126.614 E0.25701
G1 X48.806 Y126.564 E0.02407
G1 X49.603 Y126.415 E0.02428
G1 X50.375 Y126.169 E0.02428
G1 X51.111 Y125.830 E0.02428
G1 X51.800 Y125.403 E0.02428
G1 X52.431 Y124.895 E0.02428
G1 X52.995 Y124.312 E0.02428
G1 X53.482 Y123.665 E0.02428
G1 X53.887 Y122.963 E0.02428
G1 X54.202 Y122.216 E0.02428
G1 X54.356 Y121.671 E0.01697
M204 S1250
G1 X54.694 Y121.852 F9000.000
G1 X54.361 Y123.229
M204 S1000
;TYPE:Solid infill
;WIDTH:0.456608
G1 F1272.331
G1 X55.245 Y122.345 E0.03805
G1 X55.845 Y122.345 E0.01827
```

G1 X47.757 Y130.432 E0.34814

G1 X48.338 Y130.452 E0.01769

G1 X56.445 Y122.345 E0.34897

G1 X57.045 Y122.345 E0.01827

G1 X48.903 Y130.487 E0.35050

G1 X49.408 Y130.582 E0.01566

G1 X57.645 Y122.345 E0.35457

G1 X58.246 Y122.345 E0.01827

G1 X49.901 Y130.690 E0.35921

G1 X50.356 Y130.835 E0.01454

G1 X58.846 Y122.345 E0.36545

G1 X59.446 Y122.345 E0.01827

G1 X50.795 Y130.996 E0.37241

G1 X51.205 Y131.186 E0.01377

G1 X60.046 Y122.345 E0.38056

G1 X60.646 Y122.345 E0.01827

G1 X51.601 Y131.390 E0.38938

G1 X51.971 Y131.620 E0.01327

G1 X61.246 Y122.345 E0.39924

G1 X61.246 Y122.945 E0.01827

G1 X52.330 Y131.862 E0.38382

G1 X52.662 Y132.130 E0.01299

G1 X61.246 Y123.546 E0.36951

G1 X61.246 Y124.146 E0.01827

G1 X52.987 Y132.405 E0.35554

G1 X53.282 Y132.710 E0.01292

G1 X61.246 Y124.746 E0.34283

G1 X61.246 Y125.346 E0.01827

G1 X53.574 Y133.019 E0.33026

G1 X53.832 Y133.361 E0.01305

G1 X61.246 Y125.947 E0.31916

G1 X61.246 Y126.547 E0.01827

G1 X54.090 Y133.703 E0.30805

M73 P31 R47

G1 X54.310 Y134.083 E0.01337

G1 X61.246 Y127.147 E0.29859

G1 X61.246 Y127.747 E0.01827

G1 X54.529 Y134.464 E0.28914

G1 X54.710 Y134.884 E0.01390

G1 X61.246 Y128.347 E0.28136

G1 X61.246 Y128.948 E0.01827

G1 X54.888 Y135.306 E0.27370

G1 X54.925 Y135.432 E0.00402

G1 X55.362 Y135.432 E0.01328

G1 X61.246 Y129.548 E0.25331

G1 X61.246 Y130.148 E0.01827

G1 X55.962 Y135.432 E0.22747

G1 X56.562 Y135.432 E0.01827

G1 X61.246 Y130.748 E0.20163

G1 X61.246 Y131.349 E0.01827

G1 X57.162 Y135.432 E0.17580

G1 X57.763 Y135.432 E0.01827

G1 X61.246 Y131.949 E0.14996

G1 X61.246 Y132.549 E0.01827

G1 X58.363 Y135.432 E0.12412

G1 X58.963 Y135.432 E0.01827

G1 X61.246 Y133.149 E0.09828

G1 X61.246 Y133.749 E0.01827

```
G1 X59.563 Y135.432 E0.07245
G1 X60.163 Y135.432 E0.01827
G1 X61.246 Y134.350 E0.04661
G1 X61.246 Y134.950 E0.01827
G1 X60.576 Y135.620 E0.02887
M204 S1250
G1 E-4.00000 F2400.000
G1 X46.969 Y130.620 F9000.000
G1 E4.00000 F900.000
M204 S1000
G1 F1272.331
G1 X50.820 Y126.769 E0.16578
G1 X50.640 Y126.852 E0.00604
G1 X49.902 Y127.087 E0.02358
G1 X46.557 Y130.432 E0.14400
G1 X45.956 Y130.432 E0.01827
G1 X49.144 Y127.245 E0.13722
G1 X48.896 Y127.291 E0.00767
G1 X48.471 Y127.317 E0.01297
G1 X45.356 Y130.432 E0.13409
G1 X44.756 Y130.432 E0.01827
G1 X47.844 Y127.345 E0.13291
G1 X47.243 Y127.345 E0.01827
G1 X44.156 Y130.432 E0.13291
G1 X43.556 Y130.432 E0.01827
G1 X46.643 Y127.345 E0.13291
G1 X46.043 Y127.345 E0.01827
G1 X42.955 Y130.432 E0.13291
G1 X42.355 Y130.432 E0.01827
```

```
G1 X45.443 Y127.345 E0.13291
G1 X44.843 Y127.345 E0.01827
G1 X41.755 Y130.432 E0.13291
G1 X41.155 Y130.432 E0.01827
G1 X44.242 Y127.345 E0.13291
G1 X43.642 Y127.345 E0.01827
G1 X40.554 Y130.432 E0.13291
G1 X40.159 Y130.432 E0.01205
G1 X40.159 Y130.228 E0.00622
G1 X43.042 Y127.345 E0.12412
G1 X42.442 Y127.345 E0.01827
G1 X40.159 Y129.628 E0.09828
G1 X40.159 Y129.028 E0.01827
G1 X41.841 Y127.345 E0.07244
G1 X41.241 Y127.345 E0.01827
G1 X40.159 Y128.428 E0.04661
G1 X40.159 Y127.827 E0.01827
G1 X40.829 Y127.157 E0.02886
M204 S1250
; stop printing object Petg print.STL id:16 copy 0
; printing object Petg print.STL id:12 copy 0
G1 E-4.00000 F2400.000
G1 X39.860 Y112.092 F9000.000
G1 E4.00000 F900.000
M204 S800
;TYPE:Perimeter
;WIDTH:0.45
G1 F1292.454
G1 X39.860 Y108.377 E0.11130
```

```
G1 X48.032 Y108.377 E0.24487
G1 X48.873 Y108.326 E0.02523
G1 X49.720 Y108.167 E0.02583
G1 X50.542 Y107.905 E0.02583
G1 X51.325 Y107.545 E0.02583
G1 X52.057 Y107.091 E0.02583
G1 X52.728 Y106.550 E0.02583
G1 X53.328 Y105.930 E0.02583
G1 X53.847 Y105.242 E0.02583
G1 X54.277 Y104.495 E0.02583
G1 X54.612 Y103.701 E0.02583
G1 X54.704 Y103.377 E0.01007
G1 X61.575 Y103.377 E0.20589
G1 X61.575 Y117.092 E0.41094
G1 X54.704 Y117.092 E0.20589
G1 X54.612 Y116.768 E0.01007
G1 X54.277 Y115.974 E0.02583
G1 X53.847 Y115.227 E0.02583
G1 X53.328 Y114.539 E0.02583
G1 X52.728 Y113.920 E0.02583
G1 X52.057 Y113.379 E0.02583
G1 X51.325 Y112.924 E0.02583
G1 X50.542 Y112.564 E0.02583
G1 X49.720 Y112.302 E0.02583
G1 X48.873 Y112.144 E0.02583
G1 X48.032 Y112.092 E0.02523
G1 X39.920 Y112.092 E0.24307
M204 S1250
G1 X39.442 Y112.510 F9000.000
```

```
M204 S800
;TYPE:External perimeter
G1 F1292.454
G1 X39.442 Y107.960 E0.13634
G1 X48.020 Y107.960 E0.25701
G1 X48.821 Y107.910 E0.02407
G1 X49.618 Y107.761 E0.02428
G1 X50.390 Y107.515 E0.02428
G1 X51.126 Y107.176 E0.02428
G1 X51.815 Y106.749 E0.02428
G1 X52.446 Y106.241 E0.02428
G1 X53.010 Y105.658 E0.02428
G1 X53.498 Y105.011 E0.02428
G1 X53.902 Y104.309 E0.02428
G1 X54.217 Y103.562 E0.02428
G1 X54.388 Y102.960 E0.01877
G1 X61.992 Y102.960 E0.22787
G1 X61.992 Y117.510 E0.43598
G1 X54.388 Y117.510 E0.22787
G1 X54.217 Y116.907 E0.01877
G1 X53.902 Y116.160 E0.02428
G1 X53.498 Y115.458 E0.02428
G1 X53.010 Y114.811 E0.02428
G1 X52.446 Y114.229 E0.02428
G1 X51.815 Y113.720 E0.02428
G1 X51.126 Y113.293 E0.02428
G1 X50.390 Y112.954 E0.02428
G1 X49.618 Y112.708 E0.02428
G1 X48.821 Y112.559 E0.02428
```

```
G1 X48.020 Y112.510 E0.02407
G1 X39.502 Y112.510 E0.25521
M204 S1250
G1 X39.643 Y112.164 F9000.000
G1 X40.844 Y108.503
M204 S1000
;TYPE:Solid infill
;WIDTH:0.456608
G1 F1272.331
G1 X40.174 Y109.173 E0.02886
G1 X40.174 Y109.774 E0.01827
G1 X41.256 Y108.691 E0.04661
G1 X41.857 Y108.691 E0.01827
G1 X40.174 Y110.374 E0.07244
G1 X40.174 Y110.974 E0.01827
G1 X42.457 Y108.691 E0.09828
G1 X43.057 Y108.691 E0.01827
G1 X40.174 Y111.574 E0.12412
G1 X40.174 Y111.778 E0.00622
G1 X40.570 Y111.778 E0.01205
G1 X43.657 Y108.691 E0.13291
G1 X44.257 Y108.691 E0.01827
G1 X41.170 Y111.778 E0.13291
G1 X41.770 Y111.778 E0.01827
G1 X44.858 Y108.691 E0.13291
G1 X45.458 Y108.691 E0.01827
G1 X42.370 Y111.778 E0.13291
G1 X42.970 Y111.778 E0.01827
G1 X46.058 Y108.691 E0.13291
```

```
G1 X46.658 Y108.691 E0.01827
G1 X43.571 Y111.778 E0.13291
G1 X44.171 Y111.778 E0.01827
G1 X47.259 Y108.691 E0.13291
G1 X47.859 Y108.691 E0.01827
G1 X44.771 Y111.778 E0.13291
G1 X45.371 Y111.778 E0.01827
G1 X48.486 Y108.663 E0.13409
G1 X48.912 Y108.637 E0.01297
G1 X49.159 Y108.591 E0.00767
G1 X45.972 Y111.778 E0.13722
G1 X46.572 Y111.778 E0.01827
G1 X49.917 Y108.433 E0.14400
G1 X50.655 Y108.198 E0.02358
G1 X50.835 Y108.115 E0.00604
G1 X46.984 Y111.967 E0.16578
M204 S1250
G1 E-4.00000 F2400.000
G1 X54.376 Y104.575 F9000.000
G1 E4.00000 F900.000
M204 S1000
G1 F1272.331
G1 X55.260 Y103.691 E0.03805
G1 X55.860 Y103.691 E0.01827
G1 X47.772 Y111.778 E0.34814
G1 X48.353 Y111.798 E0.01769
G1 X56.460 Y103.691 E0.34897
G1 X57.060 Y103.691 E0.01827
G1 X48.918 Y111.833 E0.35050
```

```
G1 X49.423 Y111.928 E0.01566
G1 X57.661 Y103.691 E0.35457
G1 X58.261 Y103.691 E0.01827
G1 X49.916 Y112.036 E0.35921
G1 X50.371 Y112.181 E0.01454
G1 X58.861 Y103.691 E0.36545
G1 X59.461 Y103.691 E0.01827
G1 X50.810 Y112.342 E0.37241
G1 X51.221 Y112.532 E0.01377
G1 X60.061 Y103.691 E0.38056
G1 X60.662 Y103.691 E0.01827
G1 X51.616 Y112.736 E0.38938
G1 X51.986 Y112.966 E0.01327
G1 X61.261 Y103.691 E0.39924
G1 X61.261 Y104.292 E0.01827
G1 X52.345 Y113.208 E0.38382
G1 X52.677 Y113.476 E0.01299
G1 X61.261 Y104.892 E0.36951
G1 X61.261 Y105.492 E0.01827
G1 X53.002 Y113.751 E0.35554
G1 X53.297 Y114.056 E0.01292
G1 X61.261 Y106.092 E0.34283
G1 X61.261 Y106.692 E0.01827
G1 X53.589 Y114.365 E0.33026
G1 X53.847 Y114.707 E0.01305
G1 X61.261 Y107.293 E0.31916
G1 X61.261 Y107.893 E0.01827
G1 X54.105 Y115.049 E0.30805
G1 X54.325 Y115.430 E0.01337
```

```
G1 X61.261 Y108.493 E0.29859
G1 X61.261 Y109.093 E0.01827
G1 X54.544 Y115.810 E0.28914
G1 X54.725 Y116.230 E0.01390
G1 X61.261 Y109.694 E0.28136
G1 X61.261 Y110.294 E0.01827
G1 X54.903 Y116.652 E0.27370
G1 X54.941 Y116.778 E0.00402
G1 X55.377 Y116.778 E0.01328
G1 X61.261 Y110.894 E0.25331
G1 X61.261 Y111.494 E0.01827
G1 X55.977 Y116.778 E0.22747
G1 X56.577 Y116.778 E0.01827
G1 X61.261 Y112.094 E0.20163
G1 X61.261 Y112.695 E0.01827
G1 X57.177 Y116.778 E0.17580
G1 X57.778 Y116.778 E0.01827
G1 X61.261 Y113.295 E0.14996
G1 X61.261 Y113.895 E0.01827
G1 X58.378 Y116.778 E0.12412
G1 X58.978 Y116.778 E0.01827
G1 X61.261 Y114.495 E0.09828
G1 X61.261 Y115.095 E0.01827
G1 X59.578 Y116.778 E0.07245
G1 X60.179 Y116.778 E0.01827
G1 X61.261 Y115.696 E0.04661
G1 X61.261 Y116.296 E0.01827
G1 X60.591 Y116.967 E0.02887
M204 S1250
```

```
; stop printing object Petg print.STL id:12 copy 0
; printing object tpu print.STL id:7 copy 0
; stop printing object tpu print.STL id:7 copy 0
; printing object tpu print.STL id:3 copy 0
; stop printing object tpu print.STL id:3 copy 0
; printing object Petg print.STL id:2 copy 0
G1 E-4.00000 F2400.000
M73 P31 R46
G1 X89.650 Y112.673 F9000.000
G1 E4.00000 F900.000
M204 S800
;TYPE:Perimeter
;WIDTH:0.45
G1 F1292.454
G1 X89.650 Y108.958 E0.11130
G1 X97.822 Y108.958 E0.24487
G1 X98.662 Y108.906 E0.02523
G1 X99.510 Y108.748 E0.02583
G1 X100.331 Y108.486 E0.02583
G1 X101.114 Y108.126 E0.02583
G1 X101.847 Y107.672 E0.02583
G1 X102.518 Y107.131 E0.02583
G1 X103.117 Y106.511 E0.02583
G1 X103.636 Y105.823 E0.02583
G1 X104.066 Y105.076 E0.02583
G1 X104.401 Y104.282 E0.02583
G1 X104.493 Y103.958 E0.01007
G1 X111.364 Y103.958 E0.20589
G1 X111.364 Y117.673 E0.41094
```

```
G1 X104.493 Y117.673 E0.20589
G1 X104.401 Y117.349 E0.01007
G1 X104.066 Y116.555 E0.02583
G1 X103.636 Y115.808 E0.02583
G1 X103.117 Y115.120 E0.02583
G1 X102.518 Y114.500 E0.02583
G1 X101.847 Y113.960 E0.02583
G1 X101.114 Y113.505 E0.02583
G1 X100.331 Y113.145 E0.02583
G1 X99.510 Y112.883 E0.02583
G1 X98.662 Y112.725 E0.02583
G1 X97.822 Y112.673 E0.02523
G1 X89.710 Y112.673 E0.24307
M204 S1250
G1 X89.232 Y113.091 F9000.000
M204 S800
;TYPE:External perimeter
G1 F1292.454
G1 X89.232 Y108.541 E0.13634
G1 X97.809 Y108.541 E0.25701
G1 X98.611 Y108.491 E0.02407
G1 X99.407 Y108.342 E0.02428
G1 X100.180 Y108.096 E0.02428
G1 X100.916 Y107.757 E0.02428
G1 X101.604 Y107.330 E0.02428
G1 X102.235 Y106.822 E0.02428
G1 X102.799 Y106.239 E0.02428
G1 X103.287 Y105.592 E0.02428
G1 X103.691 Y104.890 E0.02428
```

```
G1 X104.006 Y104.143 E0.02428
G1 X104.177 Y103.541 E0.01877
G1 X111.782 Y103.541 E0.22787
G1 X111.782 Y118.091 E0.43598
M73 P32 R46
G1 X104.177 Y118.091 E0.22787
G1 X104.006 Y117.488 E0.01877
G1 X103.691 Y116.741 E0.02428
G1 X103.287 Y116.039 E0.02428
G1 X102.799 Y115.392 E0.02428
G1 X102.235 Y114.810 E0.02428
G1 X101.604 Y114.301 E0.02428
G1 X100.916 Y113.874 E0.02428
G1 X100.180 Y113.535 E0.02428
G1 X99.407 Y113.289 E0.02428
G1 X98.611 Y113.140 E0.02428
G1 X97.809 Y113.091 E0.02407
G1 X89.292 Y113.091 E0.25521
M204 S1250
G1 X89.432 Y112.744 F9000.000
G1 X90.634 Y109.084
M204 S1000
;TYPE:Solid infill
;WIDTH:0.456608
G1 F1272.331
G1 X89.963 Y109.754 E0.02886
G1 X89.963 Y110.354 E0.01827
G1 X91.046 Y109.272 E0.04661
G1 X91.646 Y109.272 E0.01827
```

G1 X89.963 Y110.955 E0.07244

G1 X89.963 Y111.555 E0.01827

G1 X92.246 Y109.272 E0.09828

G1 X92.846 Y109.272 E0.01827

G1 X89.963 Y112.155 E0.12412

G1 X89.963 Y112.359 E0.00622

G1 X90.359 Y112.359 E0.01205

G1 X93.447 Y109.272 E0.13291

G1 X94.047 Y109.272 E0.01827

G1 X90.959 Y112.359 E0.13291

G1 X91.559 Y112.359 E0.01827

G1 X94.647 Y109.272 E0.13291

G1 X95.247 Y109.272 E0.01827

G1 X92.160 Y112.359 E0.13291

G1 X92.760 Y112.359 E0.01827

G1 X95.847 Y109.272 E0.13291

G1 X96.448 Y109.272 E0.01827

G1 X93.360 Y112.359 E0.13291

G1 X93.960 Y112.359 E0.01827

G1 X97.048 Y109.272 E0.13291

G1 X97.648 Y109.272 E0.01827

G1 X94.560 Y112.359 E0.13291

G1 X95.161 Y112.359 E0.01827

G1 X98.276 Y109.244 E0.13409

G1 X98.701 Y109.218 E0.01297

G1 X98.949 Y109.172 E0.00767

G1 X95.761 Y112.359 E0.13722

G1 X96.361 Y112.359 E0.01827

G1 X99.706 Y109.014 E0.14400

```
G1 X100.444 Y108.779 E0.02358
G1 X100.625 Y108.696 E0.00604
G1 X96.773 Y112.547 E0.16578
M204 S1250
G1 E-4.00000 F2400.000
G1 X104.165 Y105.156 F9000.000
G1 E4.00000 F900.000
M204 S1000
G1 F1272.331
G1 X105.049 Y104.272 E0.03805
G1 X105.649 Y104.272 E0.01827
G1 X97.562 Y112.359 E0.34814
G1 X98.143 Y112.379 E0.01769
G1 X106.249 Y104.272 E0.34897
G1 X106.850 Y104.272 E0.01827
G1 X98.707 Y112.414 E0.35050
G1 X99.213 Y112.509 E0.01566
G1 X107.450 Y104.272 E0.35457
G1 X108.050 Y104.272 E0.01827
G1 X99.705 Y112.617 E0.35921
G1 X100.160 Y112.762 E0.01454
G1 X108.650 Y104.272 E0.36545
G1 X109.251 Y104.272 E0.01827
G1 X100.599 Y112.923 E0.37241
G1 X101.010 Y113.112 E0.01377
G1 X109.851 Y104.272 E0.38056
G1 X110.451 Y104.272 E0.01827
G1 X101.405 Y113.317 E0.38938
G1 X101.776 Y113.547 E0.01327
```

```
G1 X111.051 Y104.272 E0.39924
G1 X111.051 Y104.872 E0.01827
G1 X102.134 Y113.789 E0.38382
G1 X102.467 Y114.057 E0.01299
G1 X111.051 Y105.473 E0.36951
G1 X111.051 Y106.073 E0.01827
G1 X102.791 Y114.332 E0.35554
G1 X103.086 Y114.637 E0.01292
G1 X111.051 Y106.673 E0.34283
G1 X111.051 Y107.273 E0.01827
G1 X103.378 Y114.946 E0.33026
G1 X103.636 Y115.288 E0.01305
G1 X111.051 Y107.874 E0.31916
G1 X111.051 Y108.474 E0.01827
G1 X103.894 Y115.630 E0.30805
G1 X104.114 Y116.010 E0.01337
G1 X111.051 Y109.074 E0.29859
G1 X111.051 Y109.674 E0.01827
G1 X104.334 Y116.391 E0.28914
G1 X104.514 Y116.811 E0.01390
G1 X111.051 Y110.274 E0.28136
G1 X111.051 Y110.875 E0.01827
G1 X104.692 Y117.233 E0.27370
G1 X104.730 Y117.359 E0.00402
G1 X105.166 Y117.359 E0.01328
G1 X111.051 Y111.475 E0.25331
G1 X111.051 Y112.075 E0.01827
G1 X105.766 Y117.359 E0.22747
G1 X106.367 Y117.359 E0.01827
```

```
G1 X111.051 Y112.675 E0.20163
G1 X111.051 Y113.275 E0.01827
G1 X106.967 Y117.359 E0.17580
G1 X107.567 Y117.359 E0.01827
G1 X111.051 Y113.876 E0.14996
G1 X111.051 Y114.476 E0.01827
G1 X108.167 Y117.359 E0.12412
G1 X108.767 Y117.359 E0.01827
G1 X111.051 Y115.076 E0.09828
G1 X111.051 Y115.676 E0.01827
G1 X109.368 Y117.359 E0.07245
G1 X109.968 Y117.359 E0.01827
G1 X111.051 Y116.277 E0.04661
G1 X111.051 Y116.877 E0.01827
G1 X110.380 Y117.547 E0.02887
M204 S1250
; stop printing object Petg print.STL id:2 copy 0
; printing object Petg print.STL id:6 copy 0
G1 X111.349 Y122.612 F9000.000
M204 S800
;TYPE:Perimeter
;WIDTH:0.45
G1 F1292.454
G1 X111.349 Y136.327 E0.41094
G1 X104.478 Y136.327 E0.20589
G1 X104.386 Y136.003 E0.01007
G1 X104.051 Y135.209 E0.02583
G1 X103.621 Y134.462 E0.02583
G1 X103.102 Y133.774 E0.02583
```

```
G1 X102.503 Y133.154 E0.02583
G1 X101.831 Y132.613 E0.02583
G1 X101.099 Y132.159 E0.02583
G1 X100.316 Y131.799 E0.02583
G1 X99.495 Y131.537 E0.02583
G1 X98.647 Y131.379 E0.02583
G1 X97.807 Y131.327 E0.02523
G1 X89.635 Y131.327 E0.24487
G1 X89.635 Y127.612 E0.11130
G1 X97.807 Y127.612 E0.24487
G1 X98.647 Y127.560 E0.02523
G1 X99.495 Y127.402 E0.02583
G1 X100.316 Y127.140 E0.02583
G1 X101.099 Y126.780 E0.02583
G1 X101.831 Y126.325 E0.02583
G1 X102.503 Y125.785 E0.02583
G1 X103.102 Y125.165 E0.02583
G1 X103.621 Y124.477 E0.02583
G1 X104.051 Y123.730 E0.02583
G1 X104.386 Y122.936 E0.02583
G1 X104.478 Y122.612 E0.01007
G1 X111.289 Y122.612 E0.20409
M204 S1250
G1 X111.767 Y122.194 F9000.000
M204 S800
;TYPE:External perimeter
G1 F1292.454
G1 X111.767 Y136.744 E0.43598
G1 X104.162 Y136.744 E0.22787
```

```
G1 X103.991 Y136.142 E0.01877
G1 X103.676 Y135.395 E0.02428
G1 X103.272 Y134.693 E0.02428
G1 X102.784 Y134.046 E0.02428
G1 X102.220 Y133.463 E0.02428
G1 X101.589 Y132.955 E0.02428
G1 X100.901 Y132.528 E0.02428
G1 X100.164 Y132.189 E0.02428
G1 X99.392 Y131.943 E0.02428
G1 X98.596 Y131.794 E0.02428
G1 X97.794 Y131.744 E0.02407
G1 X89.217 Y131.744 E0.25701
G1 X89.217 Y127.194 E0.13634
G1 X97.794 Y127.194 E0.25701
G1 X98.596 Y127.145 E0.02407
G1 X99.392 Y126.996 E0.02428
G1 X100.164 Y126.750 E0.02428
G1 X100.901 Y126.411 E0.02428
G1 X101.589 Y125.984 E0.02428
G1 X102.220 Y125.475 E0.02428
G1 X102.784 Y124.893 E0.02428
G1 X103.272 Y124.246 E0.02428
G1 X103.676 Y123.544 E0.02428
G1 X103.991 Y122.797 E0.02428
G1 X104.162 Y122.194 E0.01877
G1 X111.707 Y122.194 E0.22608
M204 S1250
G1 X111.576 Y122.546 F9000.000
G1 E-4.00000 F2400.000
```

```
G1 X104.150 Y123.810 F9000.000
G1 E4.00000 F900.000
M204 S1000
;TYPE:Solid infill
;WIDTH:0.456608
G1 F1272.331
G1 X105.034 Y122.926 E0.03805
G1 X105.634 Y122.926 E0.01827
G1 X97.546 Y131.013 E0.34814
G1 X98.127 Y131.033 E0.01769
G1 X106.234 Y122.926 E0.34897
G1 X106.835 Y122.926 E0.01827
G1 X98.692 Y131.068 E0.35050
G1 X99.198 Y131.163 E0.01566
G1 X107.435 Y122.926 E0.35457
G1 X108.035 Y122.926 E0.01827
G1 X99.690 Y131.271 E0.35921
G1 X100.145 Y131.416 E0.01454
G1 X108.635 Y122.926 E0.36545
G1 X109.235 Y122.926 E0.01827
G1 X100.584 Y131.577 E0.37241
G1 X100.995 Y131.766 E0.01377
G1 X109.836 Y122.926 E0.38056
G1 X110.436 Y122.926 E0.01827
G1 X101.390 Y131.971 E0.38938
G1 X101.761 Y132.201 E0.01327
G1 X111.036 Y122.926 E0.39924
G1 X111.036 Y123.526 E0.01827
G1 X102.119 Y132.443 E0.38382
```

```
G1 X102.451 Y132.711 E0.01299
G1 X111.036 Y124.127 E0.36951
G1 X111.036 Y124.727 E0.01827
G1 X102.776 Y132.986 E0.35554
G1 X103.071 Y133.291 E0.01292
G1 X111.036 Y125.327 E0.34283
G1 X111.036 Y125.927 E0.01827
G1 X103.363 Y133.600 E0.33026
G1 X103.621 Y133.942 E0.01305
G1 X111.036 Y126.527 E0.31916
G1 X111.036 Y127.128 E0.01827
G1 X103.879 Y134.284 E0.30805
G1 X104.099 Y134.664 E0.01337
G1 X111.036 Y127.728 E0.29859
G1 X111.036 Y128.328 E0.01827
G1 X104.318 Y135.045 E0.28914
G1 X104.499 Y135.465 E0.01390
G1 X111.036 Y128.928 E0.28136
G1 X111.036 Y129.529 E0.01827
G1 X104.677 Y135.887 E0.27370
G1 X104.715 Y136.013 E0.00402
G1 X105.151 Y136.013 E0.01328
G1 X111.036 Y130.129 E0.25331
G1 X111.036 Y130.729 E0.01827
G1 X105.751 Y136.013 E0.22747
G1 X106.351 Y136.013 E0.01827
G1 X111.036 Y131.329 E0.20163
G1 X111.036 Y131.929 E0.01827
G1 X106.952 Y136.013 E0.17580
```

```
G1 X107.552 Y136.013 E0.01827
G1 X111.036 Y132.530 E0.14996
M73 P33 R46
G1 X111.036 Y133.130 E0.01827
G1 X108.152 Y136.013 E0.12412
G1 X108.752 Y136.013 E0.01827
G1 X111.036 Y133.730 E0.09828
G1 X111.036 Y134.330 E0.01827
G1 X109.353 Y136.013 E0.07245
G1 X109.953 Y136.013 E0.01827
G1 X111.036 Y134.931 E0.04661
G1 X111.036 Y135.531 E0.01827
G1 X110.365 Y136.201 E0.02887
M204 S1250
G1 E-4.00000 F2400.000
G1 X96.758 Y131.201 F9000.000
G1 E4.00000 F900.000
M204 S1000
G1 F1272.331
G1 X100.610 Y127.350 E0.16578
G1 X100.429 Y127.433 E0.00604
G1 X99.691 Y127.668 E0.02358
G1 X96.346 Y131.013 E0.14400
G1 X95.746 Y131.013 E0.01827
G1 X98.933 Y127.826 E0.13722
G1 X98.686 Y127.872 E0.00767
G1 X98.261 Y127.898 E0.01297
G1 X95.146 Y131.013 E0.13409
G1 X94.545 Y131.013 E0.01827
```

```
G1 X97.633 Y127.926 E0.13291
G1 X97.033 Y127.926 E0.01827
G1 X93.945 Y131.013 E0.13291
G1 X93.345 Y131.013 E0.01827
G1 X96.433 Y127.926 E0.13291
G1 X95.832 Y127.926 E0.01827
G1 X92.745 Y131.013 E0.13291
G1 X92.144 Y131.013 E0.01827
G1 X95.232 Y127.926 E0.13291
G1 X94.632 Y127.926 E0.01827
G1 X91.544 Y131.013 E0.13291
G1 X90.944 Y131.013 E0.01827
G1 X94.032 Y127.926 E0.13291
G1 X93.431 Y127.926 E0.01827
G1 X90.344 Y131.013 E0.13291
G1 X89.948 Y131.013 E0.01205
G1 X89.948 Y130.809 E0.00622
G1 X92.831 Y127.926 E0.12412
G1 X92.231 Y127.926 E0.01827
G1 X89.948 Y130.209 E0.09828
G1 X89.948 Y129.609 E0.01827
G1 X91.631 Y127.926 E0.07244
G1 X91.031 Y127.926 E0.01827
G1 X89.948 Y129.008 E0.04661
G1 X89.948 Y128.408 E0.01827
G1 X90.618 Y127.738 E0.02886
M204 S1250
; stop printing object Petg print.STL id:6 copy 0
; printing object tpu print.STL id:27 copy 0
```

; stop printing object tpu print.STL id:27 copy 0

; printing object tpu print.STL id:23 copy 0

; stop printing object tpu print.STL id:23 copy 0

; printing object tpu print.STL id:21 copy 0

; stop printing object tpu print.STL id:21 copy 0

; printing object Petg print.STL id:20 copy 0

G1 E-4.00000 F2400.000

G1 X141.293 Y92.032 F9000.000

G1 E4.00000 F900.000

M204 S800

;TYPE:Perimeter

;WIDTH:0.45

G1 F1292.454

G1 X141.293 Y88.318 E0.11130

G1 X149.465 Y88.318 E0.24487

G1 X150.306 Y88.266 E0.02523

G1 X151.153 Y88.107 E0.02583

G1 X151.974 Y87.846 E0.02583

G1 X152.757 Y87.485 E0.02583

G1 X153.490 Y87.031 E0.02583

G1 X154.161 Y86.490 E0.02583

G1 X154.761 Y85.871 E0.02583

G1 X155.279 Y85.183 E0.02583

G1 X155.710 Y84.436 E0.02583

G1 X156.045 Y83.641 E0.02583

G1 X156.136 Y83.318 E0.01007

G1 X163.007 Y83.318 E0.20589

G1 X163.007 Y97.032 E0.41094

G1 X156.136 Y97.032 E0.20589

```
G1 X156.045 Y96.709 E0.01007
G1 X155.710 Y95.915 E0.02583
G1 X155.279 Y95.168 E0.02583
G1 X154.761 Y94.479 E0.02583
G1 X154.161 Y93.860 E0.02583
G1 X153.490 Y93.319 E0.02583
G1 X152.757 Y92.865 E0.02583
G1 X151.974 Y92.504 E0.02583
G1 X151.153 Y92.243 E0.02583
G1 X150.306 Y92.084 E0.02583
G1 X149.465 Y92.032 E0.02523
G1 X141.353 Y92.032 E0.24307
M204 S1250
G1 X140.875 Y92.450 F9000.000
M204 S800
;TYPE:External perimeter
G1 F1292.454
G1 X140.875 Y87.900 E0.13634
G1 X149.452 Y87.900 E0.25701
G1 X150.254 Y87.851 E0.02407
G1 X151.051 Y87.702 E0.02428
G1 X151.823 Y87.456 E0.02428
G1 X152.559 Y87.117 E0.02428
G1 X153.248 Y86.690 E0.02428
G1 X153.879 Y86.181 E0.02428
G1 X154.443 Y85.599 E0.02428
G1 X154.930 Y84.952 E0.02428
G1 X155.335 Y84.249 E0.02428
G1 X155.650 Y83.503 E0.02428
```

```
G1 X155.820 Y82.900 E0.01877
G1 X163.425 Y82.900 E0.22787
G1 X163.425 Y97.450 E0.43598
G1 X155.820 Y97.450 E0.22787
G1 X155.650 Y96.847 E0.01877
G1 X155.335 Y96.101 E0.02428
G1 X154.930 Y95.398 E0.02428
G1 X154.443 Y94.751 E0.02428
G1 X153.879 Y94.169 E0.02428
G1 X153.248 Y93.661 E0.02428
G1 X152.559 Y93.234 E0.02428
G1 X151.823 Y92.895 E0.02428
G1 X151.051 Y92.649 E0.02428
G1 X150.254 Y92.500 E0.02428
G1 X149.452 Y92.450 E0.02407
G1 X140.935 Y92.450 E0.25521
M204 S1250
G1 X141.076 Y92.104 F9000.000
G1 X142.277 Y88.443
M204 S1000
;TYPE:Solid infill
;WIDTH:0.456608
G1 F1272.331
G1 X141.606 Y89.114 E0.02886
G1 X141.606 Y89.714 E0.01827
G1 X142.689 Y88.631 E0.04661
G1 X143.289 Y88.631 E0.01827
G1 X141.606 Y90.314 E0.07244
G1 X141.606 Y90.914 E0.01827
```

```
G1 X143.890 Y88.631 E0.09828
G1 X144.490 Y88.631 E0.01827
M73 P33 R45
G1 X141.606 Y91.515 E0.12412
G1 X141.606 Y91.719 E0.00622
G1 X142.002 Y91.719 E0.01205
G1 X145.090 Y88.631 E0.13291
G1 X145.690 Y88.631 E0.01827
G1 X142.603 Y91.719 E0.13291
G1 X143.203 Y91.719 E0.01827
G1 X146.290 Y88.631 E0.13291
G1 X146.891 Y88.631 E0.01827
G1 X143.803 Y91.719 E0.13291
G1 X144.403 Y91.719 E0.01827
G1 X147.491 Y88.631 E0.13291
G1 X148.091 Y88.631 E0.01827
G1 X145.003 Y91.719 E0.13291
G1 X145.604 Y91.719 E0.01827
G1 X148.691 Y88.631 E0.13291
G1 X149.292 Y88.631 E0.01827
G1 X146.204 Y91.719 E0.13291
G1 X146.804 Y91.719 E0.01827
G1 X149.919 Y88.604 E0.13409
G1 X150.344 Y88.578 E0.01297
G1 X150.592 Y88.531 E0.00767
G1 X147.404 Y91.719 E0.13722
G1 X148.005 Y91.719 E0.01827
G1 X151.350 Y88.374 E0.14400
G1 X152.088 Y88.139 E0.02358
```

G1 X152.268 Y88.056 E0.00604

G1 X148.417 Y91.907 E0.16578

M204 S1250

G1 E-4.00000 F2400.000

G1 X155.809 Y84.515 F9000.000

G1 E4.00000 F900.000

M204 S1000

G1 F1272.331

G1 X156.692 Y83.631 E0.03805

G1 X157.293 Y83.631 E0.01827

G1 X149.205 Y91.719 E0.34814

G1 X149.786 Y91.738 E0.01769

G1 X157.893 Y83.631 E0.34897

G1 X158.493 Y83.631 E0.01827

G1 X150.351 Y91.774 E0.35050

G1 X150.856 Y91.868 E0.01566

G1 X159.093 Y83.631 E0.35457

G1 X159.694 Y83.631 E0.01827

G1 X151.349 Y91.976 E0.35921

G1 X151.804 Y92.121 E0.01454

G1 X160.294 Y83.631 E0.36545

G1 X160.894 Y83.631 E0.01827

G1 X152.242 Y92.283 E0.37241

G1 X152.653 Y92.472 E0.01377

G1 X161.494 Y83.631 E0.38056

G1 X162.094 Y83.631 E0.01827

G1 X153.049 Y92.677 E0.38938

G1 X153.419 Y92.907 E0.01327

G1 X162.694 Y83.632 E0.39924

```
G1 X162.694 Y84.232 E0.01827
G1 X153.778 Y93.148 E0.38382
G1 X154.110 Y93.416 E0.01299
G1 X162.694 Y84.832 E0.36951
G1 X162.694 Y85.432 E0.01827
G1 X154.435 Y93.692 E0.35554
G1 X154.730 Y93.997 E0.01292
G1 X162.694 Y86.033 E0.34283
G1 X162.694 Y86.633 E0.01827
G1 X155.022 Y94.305 E0.33026
G1 X155.280 Y94.647 E0.01305
G1 X162.694 Y87.233 E0.31916
G1 X162.694 Y87.833 E0.01827
G1 X155.538 Y94.990 E0.30805
G1 X155.758 Y95.370 E0.01337
G1 X162.694 Y88.433 E0.29859
G1 X162.694 Y89.034 E0.01827
G1 X155.977 Y95.751 E0.28914
G1 X156.158 Y96.170 E0.01390
G1 X162.694 Y89.634 E0.28136
G1 X162.694 Y90.234 E0.01827
G1 X156.336 Y96.592 E0.27370
G1 X156.373 Y96.719 E0.00402
G1 X156.810 Y96.719 E0.01328
G1 X162.694 Y90.834 E0.25331
G1 X162.694 Y91.435 E0.01827
G1 X157.410 Y96.719 E0.22747
G1 X158.010 Y96.719 E0.01827
G1 X162.694 Y92.035 E0.20163
```

```
G1 X162.694 Y92.635 E0.01827
G1 X158.610 Y96.719 E0.17580
G1 X159.210 Y96.719 E0.01827
G1 X162.694 Y93.235 E0.14996
G1 X162.694 Y93.835 E0.01827
G1 X159.811 Y96.719 E0.12412
G1 X160.411 Y96.719 E0.01827
G1 X162.694 Y94.436 E0.09828
G1 X162.694 Y95.036 E0.01827
G1 X161.011 Y96.719 E0.07245
G1 X161.611 Y96.719 E0.01827
G1 X162.694 Y95.636 E0.04661
G1 X162.694 Y96.236 E0.01827
G1 X162.023 Y96.907 E0.02887
M204 S1250
; stop printing object Petg print.STL id:20 copy 0
; printing object Petg print.STL id:22 copy 0
G1 E-4.00000 F2400.000
G1 X163.014 Y104.133 F9000.000
G1 E4.00000 F900.000
M204 S800
;TYPE:Perimeter
;WIDTH:0.45
G1 F1292.454
G1 X163.014 Y117.848 E0.41094
G1 X156.143 Y117.848 E0.20589
G1 X156.052 Y117.524 E0.01007
G1 X155.717 Y116.730 E0.02583
G1 X155.286 Y115.983 E0.02583
```

```
G1 X154.768 Y115.295 E0.02583
G1 X154.168 Y114.675 E0.02583
G1 X153.497 Y114.135 E0.02583
G1 X152.764 Y113.680 E0.02583
G1 X151.981 Y113.320 E0.02583
G1 X151.160 Y113.058 E0.02583
G1 X150.313 Y112.900 E0.02583
G1 X149.472 Y112.848 E0.02523
G1 X141.300 Y112.848 E0.24487
G1 X141.300 Y109.133 E0.11130
G1 X149.472 Y109.133 E0.24487
G1 X150.313 Y109.082 E0.02523
G1 X151.160 Y108.923 E0.02583
G1 X151.981 Y108.661 E0.02583
G1 X152.764 Y108.301 E0.02583
G1 X153.497 Y107.847 E0.02583
G1 X154.168 Y107.306 E0.02583
G1 X154.768 Y106.686 E0.02583
G1 X155.286 Y105.998 E0.02583
G1 X155.717 Y105.251 E0.02583
G1 X156.052 Y104.457 E0.02583
G1 X156.143 Y104.133 E0.01007
G1 X162.954 Y104.133 E0.20409
M204 S1250
G1 X163.432 Y103.716 F9000.000
M204 S800
;TYPE:External perimeter
G1 F1292.454
G1 X163.432 Y118.266 E0.43598
```

```
G1 X155.827 Y118.266 E0.22787
G1 X155.657 Y117.663 E0.01877
G1 X155.342 Y116.916 E0.02428
G1 X154.937 Y116.214 E0.02428
G1 X154.449 Y115.567 E0.02428
G1 X153.886 Y114.985 E0.02428
G1 X153.255 Y114.476 E0.02428
G1 X152.566 Y114.049 E0.02428
G1 X151.830 Y113.710 E0.02428
G1 X151.058 Y113.464 E0.02428
G1 X150.261 Y113.315 E0.02428
G1 X149.459 Y113.266 E0.02407
G1 X140.882 Y113.266 E0.25701
G1 X140.882 Y108.716 E0.13634
G1 X149.459 Y108.716 E0.25701
G1 X150.261 Y108.666 E0.02407
G1 X151.058 Y108.517 E0.02428
G1 X151.830 Y108.271 E0.02428
G1 X152.566 Y107.932 E0.02428
G1 X153.255 Y107.505 E0.02428
G1 X153.886 Y106.997 E0.02428
G1 X154.449 Y106.414 E0.02428
G1 X154.937 Y105.767 E0.02428
G1 X155.342 Y105.065 E0.02428
G1 X155.657 Y104.318 E0.02428
G1 X155.827 Y103.716 E0.01877
G1 X163.372 Y103.716 E0.22608
M204 S1250
G1 X163.241 Y104.067 F9000.000
```

```
G1 E-4.00000 F2400.000
G1 X155.815 Y105.331 F9000.000
G1 E4.00000 F900.000
M204 S1000
;TYPE:Solid infill
;WIDTH:0.456608
G1 F1272.331
G1 X156.699 Y104.447 E0.03805
G1 X157.299 Y104.447 E0.01827
G1 X149.212 Y112.534 E0.34814
G1 X149.793 Y112.554 E0.01769
G1 X157.900 Y104.447 E0.34897
G1 X158.500 Y104.447 E0.01827
G1 X150.357 Y112.589 E0.35050
M73 P34 R45
G1 X150.863 Y112.684 E0.01566
G1 X159.100 Y104.447 E0.35457
G1 X159.700 Y104.447 E0.01827
G1 X151.355 Y112.792 E0.35921
G1 X151.811 Y112.937 E0.01454
G1 X160.301 Y104.447 E0.36545
G1 X160.901 Y104.447 E0.01827
G1 X152.249 Y113.098 E0.37241
G1 X152.660 Y113.288 E0.01377
G1 X161.501 Y104.447 E0.38056
G1 X162.101 Y104.447 E0.01827
G1 X153.056 Y113.492 E0.38938
G1 X153.426 Y113.722 E0.01327
G1 X162.701 Y104.447 E0.39924
```

```
G1 X162.701 Y105.047 E0.01827
G1 X153.784 Y113.964 E0.38382
G1 X154.117 Y114.232 E0.01299
G1 X162.701 Y105.648 E0.36951
G1 X162.701 Y106.248 E0.01827
G1 X154.441 Y114.507 E0.35554
G1 X154.737 Y114.812 E0.01292
G1 X162.701 Y106.848 E0.34283
G1 X162.701 Y107.448 E0.01827
G1 X155.029 Y115.121 E0.33026
G1 X155.287 Y115.463 E0.01305
G1 X162.701 Y108.049 E0.31916
G1 X162.701 Y108.649 E0.01827
G1 X155.545 Y115.805 E0.30805
G1 X155.764 Y116.185 E0.01337
G1 X162.701 Y109.249 E0.29859
G1 X162.701 Y109.849 E0.01827
G1 X155.984 Y116.566 E0.28914
G1 X156.165 Y116.986 E0.01390
G1 X162.701 Y110.449 E0.28136
G1 X162.701 Y111.050 E0.01827
G1 X156.343 Y117.408 E0.27370
G1 X156.380 Y117.534 E0.00402
G1 X156.816 Y117.534 E0.01328
G1 X162.701 Y111.650 E0.25331
G1 X162.701 Y112.250 E0.01827
G1 X157.417 Y117.534 E0.22747
G1 X158.017 Y117.534 E0.01827
G1 X162.701 Y112.850 E0.20163
```

```
G1 X162.701 Y113.451 E0.01827
G1 X158.617 Y117.534 E0.17580
G1 X159.217 Y117.534 E0.01827
G1 X162.701 Y114.051 E0.14996
G1 X162.701 Y114.651 E0.01827
G1 X159.817 Y117.534 E0.12412
G1 X160.418 Y117.534 E0.01827
G1 X162.701 Y115.251 E0.09828
G1 X162.701 Y115.851 E0.01827
G1 X161.018 Y117.534 E0.07245
G1 X161.618 Y117.534 E0.01827
G1 X162.701 Y116.452 E0.04661
G1 X162.701 Y117.052 E0.01827
G1 X162.030 Y117.722 E0.02887
M204 S1250
G1 E-4.00000 F2400.000
G1 X148.424 Y112.722 F9000.000
G1 E4.00000 F900.000
M204 S1000
G1 F1272.331
G1 X152.275 Y108.871 E0.16578
G1 X152.095 Y108.954 E0.00604
G1 X151.357 Y109.189 E0.02358
G1 X148.011 Y112.534 E0.14400
G1 X147.411 Y112.534 E0.01827
G1 X150.599 Y109.347 E0.13722
G1 X150.351 Y109.393 E0.00767
G1 X149.926 Y109.419 E0.01297
G1 X146.811 Y112.534 E0.13409
```

```
G1 X146.211 Y112.534 E0.01827
G1 X149.298 Y109.447 E0.13291
G1 X148.698 Y109.447 E0.01827
G1 X145.610 Y112.534 E0.13291
G1 X145.010 Y112.534 E0.01827
G1 X148.098 Y109.447 E0.13291
G1 X147.498 Y109.447 E0.01827
G1 X144.410 Y112.534 E0.13291
G1 X143.810 Y112.534 E0.01827
G1 X146.898 Y109.447 E0.13291
G1 X146.297 Y109.447 E0.01827
G1 X143.210 Y112.534 E0.13291
G1 X142.609 Y112.534 E0.01827
G1 X145.697 Y109.447 E0.13291
G1 X145.097 Y109.447 E0.01827
G1 X142.009 Y112.534 E0.13291
G1 X141.613 Y112.534 E0.01205
G1 X141.613 Y112.330 E0.00622
G1 X144.497 Y109.447 E0.12412
G1 X143.896 Y109.447 E0.01827
G1 X141.613 Y111.730 E0.09828
G1 X141.613 Y111.130 E0.01827
G1 X143.296 Y109.447 E0.07244
G1 X142.696 Y109.447 E0.01827
G1 X141.613 Y110.530 E0.04661
G1 X141.613 Y109.929 E0.01827
G1 X142.284 Y109.259 E0.02886
M204 S1250
; stop printing object Petg print.STL id:22 copy 0
```

```
; printing object Petg print.STL id:26 copy 0
G1 E-4.00000 F2400.000
G1 X141.285 Y127.787 F9000.000
G1 E4.00000 F900.000
M204 S800
;TYPE:Perimeter
;WIDTH:0.45
G1 F1292.454
G1 X149.457 Y127.787 E0.24487
G1 X150.297 Y127.735 E0.02523
G1 X151.145 Y127.577 E0.02583
G1 X151.966 Y127.315 E0.02583
G1 X152.749 Y126.955 E0.02583
G1 X153.482 Y126.501 E0.02583
G1 X154.153 Y125.960 E0.02583
G1 X154.752 Y125.340 E0.02583
G1 X155.271 Y124.652 E0.02583
G1 X155.701 Y123.905 E0.02583
G1 X156.036 Y123.111 E0.02583
G1 X156.128 Y122.787 E0.01007
G1 X162.999 Y122.787 E0.20589
G1 X162.999 Y136.502 E0.41094
G1 X156.128 Y136.502 E0.20589
G1 X156.036 Y136.178 E0.01007
G1 X155.701 Y135.384 E0.02583
G1 X155.271 Y134.637 E0.02583
G1 X154.752 Y133.949 E0.02583
G1 X154.153 Y133.329 E0.02583
G1 X153.482 Y132.789 E0.02583
```

```
G1 X152.749 Y132.334 E0.02583
G1 X151.966 Y131.974 E0.02583
G1 X151.145 Y131.712 E0.02583
G1 X150.297 Y131.554 E0.02583
G1 X149.457 Y131.502 E0.02523
G1 X141.285 Y131.502 E0.24487
G1 X141.285 Y127.847 E0.10950
M204 S1250
G1 X140.867 Y127.370 F9000.000
M204 S800
;TYPE:External perimeter
G1 F1292.454
G1 X149.444 Y127.370 E0.25701
G1 X150.246 Y127.320 E0.02407
G1 X151.043 Y127.171 E0.02428
G1 X151.815 Y126.925 E0.02428
G1 X152.551 Y126.586 E0.02428
G1 X153.240 Y126.159 E0.02428
G1 X153.871 Y125.651 E0.02428
G1 X154.434 Y125.068 E0.02428
G1 X154.922 Y124.421 E0.02428
G1 X155.326 Y123.719 E0.02428
G1 X155.641 Y122.972 E0.02428
G1 X155.812 Y122.370 E0.01877
G1 X163.417 Y122.370 E0.22787
G1 X163.417 Y136.920 E0.43598
G1 X155.812 Y136.920 E0.22787
G1 X155.641 Y136.317 E0.01877
G1 X155.326 Y135.570 E0.02428
```

```
G1 X154.922 Y134.868 E0.02428
G1 X154.434 Y134.221 E0.02428
G1 X153.871 Y133.639 E0.02428
G1 X153.240 Y133.130 E0.02428
G1 X152.551 Y132.703 E0.02428
G1 X151.815 Y132.364 E0.02428
G1 X151.043 Y132.118 E0.02428
G1 X150.246 Y131.969 E0.02428
G1 X149.444 Y131.920 E0.02407
G1 X140.867 Y131.920 E0.25701
G1 X140.867 Y127.430 E0.13454
M204 S1250
G1 X141.262 Y127.434 F9000.000
G1 X142.269 Y127.913
M204 S1000
;TYPE:Solid infill
;WIDTH:0.456608
G1 F1272.331
G1 X141.598 Y128.583 E0.02886
G1 X141.598 Y129.183 E0.01827
G1 X142.681 Y128.101 E0.04661
G1 X143.281 Y128.101 E0.01827
G1 X141.598 Y129.784 E0.07244
G1 X141.598 Y130.384 E0.01827
G1 X143.881 Y128.101 E0.09828
G1 X144.482 Y128.101 E0.01827
G1 X141.598 Y130.984 E0.12412
G1 X141.598 Y131.188 E0.00622
G1 X141.994 Y131.188 E0.01205
```

```
G1 X145.082 Y128.101 E0.13291
G1 X145.682 Y128.101 E0.01827
G1 X142.594 Y131.188 E0.13291
G1 X143.195 Y131.188 E0.01827
G1 X146.282 Y128.101 E0.13291
G1 X146.882 Y128.101 E0.01827
G1 X143.795 Y131.188 E0.13291
G1 X144.395 Y131.188 E0.01827
G1 X147.483 Y128.101 E0.13291
G1 X148.083 Y128.101 E0.01827
G1 X144.995 Y131.188 E0.13291
G1 X145.595 Y131.188 E0.01827
G1 X148.683 Y128.101 E0.13291
G1 X149.283 Y128.101 E0.01827
G1 X146.196 Y131.188 E0.13291
G1 X146.796 Y131.188 E0.01827
G1 X149.911 Y128.073 E0.13409
G1 X150.336 Y128.047 E0.01297
G1 X150.584 Y128.001 E0.00767
G1 X147.396 Y131.188 E0.13722
G1 X147.996 Y131.188 E0.01827
G1 X151.342 Y127.843 E0.14400
G1 X152.080 Y127.608 E0.02358
G1 X152.260 Y127.525 E0.00604
G1 X148.408 Y131.376 E0.16578
M204 S1250
G1 E-4.00000 F2400.000
G1 X155.800 Y123.985 F9000.000
G1 E4.00000 F900.000
```

```
M204 S1000
G1 F1272.331
G1 X156.684 Y123.101 E0.03805
G1 X157.284 Y123.101 E0.01827
G1 X149.197 Y131.188 E0.34814
G1 X149.778 Y131.208 E0.01769
G1 X157.885 Y123.101 E0.34897
G1 X158.485 Y123.101 E0.01827
G1 X150.342 Y131.243 E0.35050
G1 X150.848 Y131.338 E0.01566
G1 X159.085 Y123.101 E0.35457
G1 X159.685 Y123.101 E0.01827
G1 X151.340 Y131.446 E0.35921
G1 X151.796 Y131.591 E0.01454
G1 X160.285 Y123.101 E0.36545
G1 X160.886 Y123.101 E0.01827
G1 X152.234 Y131.752 E0.37241
M73 P34 R44
G1 X152.645 Y131.941 E0.01377
G1 X161.486 Y123.101 E0.38056
G1 X162.086 Y123.101 E0.01827
G1 X153.040 Y132.146 E0.38938
G1 X153.411 Y132.376 E0.01327
G1 X162.686 Y123.101 E0.39924
G1 X162.686 Y123.701 E0.01827
G1 X153.769 Y132.618 E0.38382
G1 X154.102 Y132.886 E0.01299
G1 X162.686 Y124.302 E0.36951
G1 X162.686 Y124.902 E0.01827
```

G1 X154.426 Y133.161 E0.35554

G1 X154.722 Y133.466 E0.01292

G1 X162.686 Y125.502 E0.34283

G1 X162.686 Y126.102 E0.01827

G1 X155.014 Y133.775 E0.33026

G1 X155.271 Y134.117 E0.01305

G1 X162.686 Y126.703 E0.31916

G1 X162.686 Y127.303 E0.01827

G1 X155.529 Y134.459 E0.30805

G1 X155.749 Y134.839 E0.01337

G1 X162.686 Y127.903 E0.29859

G1 X162.686 Y128.503 E0.01827

G1 X155.969 Y135.220 E0.28914

G1 X156.149 Y135.640 E0.01390

G1 X162.686 Y129.103 E0.28136

G1 X162.686 Y129.704 E0.01827

G1 X156.328 Y136.062 E0.27370

G1 X156.365 Y136.188 E0.00402

G1 X156.801 Y136.188 E0.01328

G1 X162.686 Y130.304 E0.25331

M73 P35 R44

G1 X162.686 Y130.904 E0.01827

G1 X157.401 Y136.188 E0.22747

G1 X158.002 Y136.188 E0.01827

G1 X162.686 Y131.504 E0.20163

G1 X162.686 Y132.104 E0.01827

G1 X158.602 Y136.188 E0.17580

G1 X159.202 Y136.188 E0.01827

G1 X162.686 Y132.705 E0.14996

G1 X162.686 Y133.305 E0.01827

G1 X159.802 Y136.188 E0.12412

G1 X160.403 Y136.188 E0.01827

G1 X162.686 Y133.905 E0.09828

G1 X162.686 Y134.505 E0.01827

G1 X161.003 Y136.188 E0.07245

G1 X161.603 Y136.188 E0.01827

G1 X162.686 Y135.106 E0.04661

G1 X162.686 Y135.706 E0.01827

G1 X162.015 Y136.376 E0.02887

M204 S1250

; stop printing object Petg print.STL id:26 copy 0

; printing object Petg print.STL id:0 copy 0

G1 E-4.00000 F2400.000

G1 X111.357 Y96.857 F9000.000

G1 E4.00000 F900.000

M204 S800

;TYPE:Perimeter

;WIDTH:0.45

G1 F1292.454

G1 X104.486 Y96.857 E0.20589

G1 X104.394 Y96.534 E0.01007

G1 X104.059 Y95.739 E0.02583

G1 X103.629 Y94.993 E0.02583

G1 X103.110 Y94.304 E0.02583

G1 X102.511 Y93.685 E0.02583

G1 X101.840 Y93.144 E0.02583

G1 X101.107 Y92.690 E0.02583

G1 X100.324 Y92.329 E0.02583

```
G1 X99.503 Y92.068 E0.02583
G1 X98.656 Y91.909 E0.02583
G1 X97.815 Y91.857 E0.02523
G1 X89.643 Y91.857 E0.24487
G1 X89.643 Y88.143 E0.11130
G1 X97.815 Y88.143 E0.24487
G1 X98.656 Y88.091 E0.02523
G1 X99.503 Y87.932 E0.02583
G1 X100.324 Y87.671 E0.02583
G1 X101.107 Y87.310 E0.02583
G1 X101.840 Y86.856 E0.02583
G1 X102.511 Y86.315 E0.02583
G1 X103.110 Y85.696 E0.02583
G1 X103.629 Y85.007 E0.02583
G1 X104.059 Y84.261 E0.02583
G1 X104.394 Y83.466 E0.02583
G1 X104.486 Y83.143 E0.01007
G1 X111.357 Y83.143 E0.20589
G1 X111.357 Y96.797 E0.40914
M204 S1250
G1 X111.775 Y97.275 F9000.000
M204 S800
;TYPE:External perimeter
G1 F1292.454
G1 X104.170 Y97.275 E0.22787
G1 X104.000 Y96.672 E0.01877
G1 X103.685 Y95.926 E0.02428
G1 X103.280 Y95.223 E0.02428
G1 X102.792 Y94.576 E0.02428
```

```
G1 X102.229 Y93.994 E0.02428
G1 X101.598 Y93.486 E0.02428
G1 X100.909 Y93.059 E0.02428
G1 X100.173 Y92.719 E0.02428
G1 X99.401 Y92.474 E0.02428
G1 X98.604 Y92.325 E0.02428
G1 X97.802 Y92.275 E0.02407
G1 X89.225 Y92.275 E0.25701
G1 X89.225 Y87.725 E0.13634
G1 X97.802 Y87.725 E0.25701
G1 X98.604 Y87.675 E0.02407
G1 X99.401 Y87.526 E0.02428
G1 X100.173 Y87.281 E0.02428
G1 X100.909 Y86.941 E0.02428
G1 X101.598 Y86.514 E0.02428
G1 X102.229 Y86.006 E0.02428
G1 X102.792 Y85.424 E0.02428
G1 X103.280 Y84.777 E0.02428
G1 X103.685 Y84.074 E0.02428
G1 X104.000 Y83.328 E0.02428
G1 X104.170 Y82.725 E0.01877
G1 X111.775 Y82.725 E0.22787
G1 X111.775 Y97.215 E0.43418
M204 S1250
G1 X111.401 Y97.133 F9000.000
G1 X110.373 Y96.732
M204 S1000
;TYPE:Solid infill
;WIDTH:0.456608
```

```
G1 F1272.331
G1 X111.044 Y96.061 E0.02887
G1 X111.044 Y95.461 E0.01827
G1 X109.961 Y96.544 E0.04661
G1 X109.361 Y96.544 E0.01827
G1 X111.044 Y94.861 E0.07245
G1 X111.044 Y94.261 E0.01827
G1 X108.761 Y96.544 E0.09828
G1 X108.160 Y96.544 E0.01827
G1 X111.044 Y93.660 E0.12412
G1 X111.044 Y93.060 E0.01827
G1 X107.560 Y96.544 E0.14996
G1 X106.960 Y96.544 E0.01827
G1 X111.044 Y92.460 E0.17580
G1 X111.044 Y91.860 E0.01827
G1 X106.360 Y96.544 E0.20163
G1 X105.760 Y96.544 E0.01827
G1 X111.044 Y91.260 E0.22747
G1 X111.044 Y90.659 E0.01827
G1 X105.159 Y96.544 E0.25331
G1 X104.723 Y96.544 E0.01328
G1 X104.686 Y96.417 E0.00402
G1 X111.044 Y90.059 E0.27370
G1 X111.044 Y89.459 E0.01827
G1 X104.507 Y95.995 E0.28136
G1 X104.327 Y95.576 E0.01390
G1 X111.044 Y88.859 E0.28914
G1 X111.044 Y88.258 E0.01827
G1 X104.107 Y95.195 E0.29859
```

G1 X103.887 Y94.815 E0.01337

G1 X111.044 Y87.658 E0.30805

G1 X111.044 Y87.058 E0.01827

G1 X103.630 Y94.472 E0.31916

G1 X103.372 Y94.130 E0.01305

G1 X111.044 Y86.458 E0.33026

G1 X111.044 Y85.858 E0.01827

G1 X103.080 Y93.822 E0.34283

G1 X102.784 Y93.517 E0.01292

G1 X111.044 Y85.257 E0.35554

G1 X111.044 Y84.657 E0.01827

G1 X102.460 Y93.241 E0.36951

G1 X102.127 Y92.973 E0.01299

G1 X111.044 Y84.057 E0.38382

G1 X111.044 Y83.457 E0.01827

G1 X101.769 Y92.732 E0.39924

G1 X101.398 Y92.502 E0.01327

G1 X110.444 Y83.456 E0.38938

G1 X109.844 Y83.456 E0.01827

G1 X101.003 Y92.297 E0.38056

G1 X100.592 Y92.108 E0.01377

G1 X109.244 Y83.456 E0.37241

G1 X108.643 Y83.456 E0.01827

G1 X100.154 Y91.946 E0.36545

G1 X99.698 Y91.801 E0.01454

G1 X108.043 Y83.456 E0.35921

G1 X107.443 Y83.456 E0.01827

G1 X99.206 Y91.693 E0.35457

G1 X98.700 Y91.599 E0.01566

G1 X106.843 Y83.456 E0.35050

G1 X106.243 Y83.456 E0.01827

G1 X98.136 Y91.563 E0.34897

G1 X97.555 Y91.544 E0.01769

G1 X105.642 Y83.456 E0.34814

G1 X105.042 Y83.456 E0.01827

G1 X104.158 Y84.340 E0.03805

M204 S1250

G1 E-4.00000 F2400.000

G1 X96.766 Y91.732 F9000.000

G1 E4.00000 F900.000

M204 S1000

G1 F1272.331

G1 X100.618 Y87.881 E0.16578

G1 X100.438 Y87.964 E0.00604

G1 X99.700 Y88.199 E0.02358

G1 X96.354 Y91.544 E0.14400

G1 X95.754 Y91.544 E0.01827

G1 X98.942 Y88.356 E0.13722

G1 X98.694 Y88.402 E0.00767

G1 X98.269 Y88.429 E0.01297

G1 X95.154 Y91.544 E0.13409

G1 X94.554 Y91.544 E0.01827

G1 X97.641 Y88.456 E0.13291

G1 X97.041 Y88.456 E0.01827

G1 X93.953 Y91.544 E0.13291

G1 X93.353 Y91.544 E0.01827

G1 X96.441 Y88.456 E0.13291

G1 X95.841 Y88.456 E0.01827

```
G1 X92.753 Y91.544 E0.13291
G1 X92.153 Y91.544 E0.01827
G1 X95.240 Y88.456 E0.13291
G1 X94.640 Y88.456 E0.01827
G1 X91.553 Y91.544 E0.13291
G1 X90.952 Y91.544 E0.01827
G1 X94.040 Y88.456 E0.13291
G1 X93.440 Y88.456 E0.01827
G1 X90.352 Y91.544 E0.13291
G1 X89.956 Y91.544 E0.01205
G1 X89.956 Y91.340 E0.00622
G1 X92.840 Y88.456 E0.12412
G1 X92.239 Y88.456 E0.01827
G1 X89.956 Y90.739 E0.09828
G1 X89.956 Y90.139 E0.01827
G1 X91.639 Y88.456 E0.07244
G1 X91.039 Y88.456 E0.01827
G1 X89.956 Y89.539 E0.04661
G1 X89.956 Y88.939 E0.01827
G1 X90.627 Y88.268 E0.02886
M204 S1250
; stop printing object Petg print.STL id:0 copy 0
; printing object tpu print.STL id:1 copy 0
; stop printing object tpu print.STL id:1 copy 0
; printing object tpu print.STL id:5 copy 0
; stop printing object tpu print.STL id:5 copy 0
; printing object tpu print.STL id:9 copy 0
; stop printing object tpu print.STL id:9 copy 0
; printing object Petg print.STL id:8 copy 0
```

```
G1 E-4.00000 F2400.000
G1 X104.530 Y54.716 F9000.000
G1 E4.00000 F900.000
M204 S800
;TYPE:Perimeter
;WIDTH:0.45
G1 F1292.454
G1 X104.438 Y54.392 E0.01007
G1 X104.103 Y53.598 E0.02583
G1 X103.673 Y52.851 E0.02583
G1 X103.154 Y52.163 E0.02583
G1 X102.555 Y51.544 E0.02583
G1 X101.883 Y51.003 E0.02583
G1 X101.151 Y50.549 E0.02583
G1 X100.368 Y50.188 E0.02583
G1 X99.546 Y49.926 E0.02583
G1 X98.699 Y49.768 E0.02583
G1 X97.859 Y49.716 E0.02523
G1 X89.686 Y49.716 E0.24487
G1 X89.686 Y46.002 E0.11130
G1 X97.859 Y46.002 E0.24487
G1 X98.699 Y45.950 E0.02523
G1 X99.546 Y45.791 E0.02583
G1 X100.368 Y45.530 E0.02583
G1 X101.151 Y45.169 E0.02583
G1 X101.883 Y44.715 E0.02583
G1 X102.555 Y44.174 E0.02583
G1 X103.154 Y43.555 E0.02583
G1 X103.673 Y42.866 E0.02583
```

```
G1 X104.103 Y42.119 E0.02583
G1 X104.438 Y41.325 E0.02583
G1 X104.530 Y41.002 E0.01007
G1 X111.401 Y41.002 E0.20589
G1 X111.401 Y54.716 E0.41094
G1 X104.590 Y54.716 E0.20409
M204 S1250
G1 X104.214 Y55.134 F9000.000
M204 S800
;TYPE:External perimeter
G1 F1292.454
G1 X104.043 Y54.531 E0.01877
G1 X103.728 Y53.784 E0.02428
G1 X103.324 Y53.082 E0.02428
G1 X102.836 Y52.435 E0.02428
G1 X102.272 Y51.853 E0.02428
G1 X101.641 Y51.344 E0.02428
G1 X100.952 Y50.917 E0.02428
G1 X100.216 Y50.578 E0.02428
G1 X99.444 Y50.332 E0.02428
G1 X98.648 Y50.183 E0.02428
G1 X97.846 Y50.134 E0.02407
G1 X89.269 Y50.134 E0.25701
G1 X89.269 Y45.584 E0.13634
G1 X97.846 Y45.584 E0.25701
G1 X98.648 Y45.534 E0.02407
G1 X99.444 Y45.385 E0.02428
G1 X100.216 Y45.139 E0.02428
G1 X100.952 Y44.800 E0.02428
```

```
G1 X101.641 Y44.373 E0.02428
G1 X102.272 Y43.865 E0.02428
G1 X102.836 Y43.282 E0.02428
G1 X103.324 Y42.635 E0.02428
G1 X103.728 Y41.933 E0.02428
G1 X104.043 Y41.186 E0.02428
G1 X104.214 Y40.584 E0.01877
G1 X111.819 Y40.584 E0.22787
G1 X111.819 Y55.134 E0.43598
G1 X104.274 Y55.134 E0.22608
M204 S1250
G1 X104.204 Y54.734 F9000.000
G1 X110.417 Y54.591
M204 S1000
;TYPE:Solid infill
;WIDTH:0.456608
G1 F1272.331
G1 X111.087 Y53.920 E0.02887
G1 X111.087 Y53.320 E0.01827
G1 X110.005 Y54.403 E0.04661
G1 X109.404 Y54.403 E0.01827
G1 X111.087 Y52.720 E0.07245
G1 X111.087 Y52.119 E0.01827
G1 X108.804 Y54.403 E0.09828
G1 X108.204 Y54.403 E0.01827
G1 X111.087 Y51.519 E0.12412
G1 X111.087 Y50.919 E0.01827
G1 X107.604 Y54.403 E0.14996
G1 X107.004 Y54.403 E0.01827
```

```
G1 X111.087 Y50.319 E0.17580
G1 X111.087 Y49.718 E0.01827
G1 X106.403 Y54.403 E0.20163
G1 X105.803 Y54.403 E0.01827
G1 X111.087 Y49.118 E0.22747
G1 X111.087 Y48.518 E0.01827
G1 X105.203 Y54.403 E0.25331
G1 X104.767 Y54.403 E0.01328
G1 X104.729 Y54.276 E0.00402
G1 X111.087 Y47.918 E0.27370
G1 X111.087 Y47.318 E0.01827
G1 X104.551 Y53.854 E0.28136
G1 X104.370 Y53.434 E0.01390
G1 X111.087 Y46.717 E0.28914
G1 X111.087 Y46.117 E0.01827
G1 X104.151 Y53.054 E0.29859
G1 X103.931 Y52.673 E0.01337
G1 X111.087 Y45.517 E0.30805
G1 X111.087 Y44.917 E0.01827
G1 X103.673 Y52.331 E0.31916
M73 P36 R44
G1 X103.415 Y51.989 E0.01305
G1 X111.087 Y44.316 E0.33026
G1 X111.087 Y43.716 E0.01827
G1 X103.123 Y51.681 E0.34283
G1 X102.828 Y51.376 E0.01292
G1 X111.087 Y43.116 E0.35554
G1 X111.087 Y42.516 E0.01827
G1 X102.503 Y51.100 E0.36951
```

```
G1 X102.171 Y50.832 E0.01299
G1 X111.087 Y41.916 E0.38382
G1 X111.087 Y41.315 E0.01827
G1 X101.813 Y50.590 E0.39924
G1 X101.442 Y50.361 E0.01327
G1 X110.488 Y41.315 E0.38938
G1 X109.888 Y41.315 E0.01827
G1 X101.047 Y50.156 E0.38056
G1 X100.636 Y49.966 E0.01377
G1 X109.287 Y41.315 E0.37241
G1 X108.687 Y41.315 E0.01827
G1 X100.197 Y49.805 E0.36545
G1 X99.742 Y49.660 E0.01454
G1 X108.087 Y41.315 E0.35921
G1 X107.487 Y41.315 E0.01827
G1 X99.250 Y49.552 E0.35457
G1 X98.744 Y49.457 E0.01566
G1 X106.886 Y41.315 E0.35050
G1 X106.286 Y41.315 E0.01827
G1 X98.179 Y49.422 E0.34897
G1 X97.598 Y49.403 E0.01769
G1 X105.686 Y41.315 E0.34814
G1 X105.086 Y41.315 E0.01827
G1 X104.202 Y42.199 E0.03805
M204 S1250
G1 E-4.00000 F2400.000
G1 X96.810 Y49.591 F9000.000
G1 E4.00000 F900.000
M204 S1000
```

```
G1 F1272.331
G1 X100.661 Y45.739 E0.16578
G1 X100.481 Y45.822 E0.00604
G1 X99.743 Y46.057 E0.02358
G1 X96.398 Y49.403 E0.14400
M73 P36 R43
G1 X95.798 Y49.403 E0.01827
G1 X98.985 Y46.215 E0.13722
G1 X98.738 Y46.261 E0.00767
G1 X98.313 Y46.287 E0.01297
G1 X95.197 Y49.403 E0.13409
G1 X94.597 Y49.403 E0.01827
G1 X97.685 Y46.315 E0.13291
G1 X97.085 Y46.315 E0.01827
G1 X93.997 Y49.403 E0.13291
G1 X93.397 Y49.403 E0.01827
G1 X96.485 Y46.315 E0.13291
G1 X95.884 Y46.315 E0.01827
G1 X92.797 Y49.403 E0.13291
G1 X92.196 Y49.403 E0.01827
G1 X95.284 Y46.315 E0.13291
G1 X94.684 Y46.315 E0.01827
G1 X91.596 Y49.403 E0.13291
G1 X90.996 Y49.403 E0.01827
G1 X94.084 Y46.315 E0.13291
G1 X93.483 Y46.315 E0.01827
G1 X90.396 Y49.403 E0.13291
G1 X90.000 Y49.403 E0.01205
G1 X90.000 Y49.198 E0.00622
```

```
G1 X92.883 Y46.315 E0.12412
G1 X92.283 Y46.315 E0.01827
G1 X90.000 Y48.598 E0.09828
G1 X90.000 Y47.998 E0.01827
G1 X91.683 Y46.315 E0.07244
G1 X91.083 Y46.315 E0.01827
G1 X90.000 Y47.398 E0.04661
G1 X90.000 Y46.797 E0.01827
G1 X90.670 Y46.127 E0.02886
M204 S1250
; stop printing object Petg print.STL id:8 copy 0
; printing object Petg print.STL id:4 copy 0
G1 E-4.00000 F2400.000
G1 X89.694 Y65.950 F9000.000
G1 E4.00000 F900.000
M204 S800
;TYPE:Perimeter
;WIDTH:0.45
G1 F1292.454
G1 X97.866 Y65.950 E0.24487
G1 X98.707 Y65.899 E0.02523
G1 X99.554 Y65.740 E0.02583
G1 X100.375 Y65.478 E0.02583
G1 X101.158 Y65.118 E0.02583
G1 X101.891 Y64.664 E0.02583
G1 X102.562 Y64.123 E0.02583
G1 X103.162 Y63.503 E0.02583
G1 X103.680 Y62.815 E0.02583
G1 X104.111 Y62.068 E0.02583
```

```
G1 X104.446 Y61.274 E0.02583
G1 X104.537 Y60.950 E0.01007
G1 X111.408 Y60.950 E0.20589
G1 X111.408 Y74.665 E0.41094
G1 X104.537 Y74.665 E0.20589
G1 X104.446 Y74.341 E0.01007
G1 X104.111 Y73.547 E0.02583
G1 X103.680 Y72.800 E0.02583
G1 X103.162 Y72.112 E0.02583
G1 X102.562 Y71.493 E0.02583
G1 X101.891 Y70.952 E0.02583
G1 X101.158 Y70.497 E0.02583
G1 X100.375 Y70.137 E0.02583
G1 X99.554 Y69.875 E0.02583
G1 X98.707 Y69.717 E0.02583
G1 X97.866 Y69.665 E0.02523
G1 X89.694 Y69.665 E0.24487
G1 X89.694 Y66.010 E0.10950
M204 S1250
G1 X89.276 Y65.533 F9000.000
M204 S800
;TYPE:External perimeter
G1 F1292.454
G1 X97.853 Y65.533 E0.25701
G1 X98.655 Y65.483 E0.02407
G1 X99.452 Y65.334 E0.02428
G1 X100.224 Y65.088 E0.02428
G1 X100.960 Y64.749 E0.02428
G1 X101.649 Y64.322 E0.02428
```

```
G1 X102.280 Y63.814 E0.02428
G1 X102.843 Y63.231 E0.02428
G1 X103.331 Y62.584 E0.02428
G1 X103.736 Y61.882 E0.02428
G1 X104.051 Y61.135 E0.02428
G1 X104.221 Y60.533 E0.01877
G1 X111.826 Y60.533 E0.22787
G1 X111.826 Y75.083 E0.43598
G1 X104.221 Y75.083 E0.22787
G1 X104.051 Y74.480 E0.01877
G1 X103.736 Y73.733 E0.02428
G1 X103.331 Y73.031 E0.02428
G1 X102.843 Y72.384 E0.02428
G1 X102.280 Y71.802 E0.02428
G1 X101.649 Y71.293 E0.02428
G1 X100.960 Y70.866 E0.02428
G1 X100.224 Y70.527 E0.02428
G1 X99.452 Y70.281 E0.02428
G1 X98.655 Y70.132 E0.02428
G1 X97.853 Y70.083 E0.02407
G1 X89.276 Y70.083 E0.25701
G1 X89.276 Y65.593 E0.13454
M204 S1250
G1 X89.671 Y65.597 F9000.000
G1 X90.678 Y66.076
M204 S1000
;TYPE:Solid infill
;WIDTH:0.456608
G1 F1272.331
```

G1 X90.007 Y66.746 E0.02886

G1 X90.007 Y67.347 E0.01827

G1 X91.090 Y66.264 E0.04661

G1 X91.690 Y66.264 E0.01827

G1 X90.007 Y67.947 E0.07244

G1 X90.007 Y68.547 E0.01827

G1 X92.290 Y66.264 E0.09828

G1 X92.891 Y66.264 E0.01827

G1 X90.007 Y69.147 E0.12412

G1 X90.007 Y69.351 E0.00622

G1 X90.403 Y69.351 E0.01205

G1 X93.491 Y66.264 E0.13291

G1 X94.091 Y66.264 E0.01827

G1 X91.003 Y69.351 E0.13291

G1 X91.604 Y69.351 E0.01827

G1 X94.691 Y66.264 E0.13291

G1 X95.292 Y66.264 E0.01827

G1 X92.204 Y69.351 E0.13291

G1 X92.804 Y69.351 E0.01827

G1 X95.892 Y66.264 E0.13291

G1 X96.492 Y66.264 E0.01827

G1 X93.404 Y69.351 E0.13291

G1 X94.005 Y69.351 E0.01827

G1 X97.092 Y66.264 E0.13291

G1 X97.692 Y66.264 E0.01827

G1 X94.605 Y69.351 E0.13291

G1 X95.205 Y69.351 E0.01827

G1 X98.320 Y66.236 E0.13409

G1 X98.745 Y66.210 E0.01297

```
G1 X98.993 Y66.164 E0.00767
G1 X95.805 Y69.351 E0.13722
G1 X96.405 Y69.351 E0.01827
G1 X99.751 Y66.006 E0.14400
G1 X100.489 Y65.771 E0.02358
G1 X100.669 Y65.688 E0.00604
G1 X96.818 Y69.539 E0.16578
M204 S1250
G1 E-4.00000 F2400.000
G1 X104.209 Y62.148 F9000.000
G1 E4.00000 F900.000
M204 S1000
G1 F1272.331
G1 X105.093 Y61.264 E0.03805
G1 X105.694 Y61.264 E0.01827
G1 X97.606 Y69.351 E0.34814
G1 X98.187 Y69.371 E0.01769
G1 X106.294 Y61.264 E0.34897
G1 X106.894 Y61.264 E0.01827
G1 X98.751 Y69.406 E0.35050
G1 X99.257 Y69.501 E0.01566
G1 X107.494 Y61.264 E0.35457
G1 X108.094 Y61.264 E0.01827
G1 X99.750 Y69.609 E0.35921
G1 X100.205 Y69.754 E0.01454
G1 X108.695 Y61.264 E0.36545
G1 X109.295 Y61.264 E0.01827
G1 X100.643 Y69.915 E0.37241
G1 X101.054 Y70.105 E0.01377
```

```
G1 X109.895 Y61.264 E0.38056
G1 X110.495 Y61.264 E0.01827
G1 X101.450 Y70.309 E0.38938
G1 X101.820 Y70.539 E0.01327
G1 X111.095 Y61.264 E0.39924
G1 X111.095 Y61.865 E0.01827
G1 X102.178 Y70.781 E0.38382
G1 X102.511 Y71.049 E0.01299
G1 X111.095 Y62.465 E0.36951
G1 X111.095 Y63.065 E0.01827
G1 X102.835 Y71.324 E0.35554
G1 X103.131 Y71.629 E0.01292
G1 X111.095 Y63.665 E0.34283
G1 X111.095 Y64.265 E0.01827
G1 X103.423 Y71.938 E0.33026
G1 X103.681 Y72.280 E0.01305
G1 X111.095 Y64.866 E0.31916
G1 X111.095 Y65.466 E0.01827
G1 X103.939 Y72.622 E0.30805
G1 X104.159 Y73.003 E0.01337
G1 X111.095 Y66.066 E0.29859
G1 X111.095 Y66.666 E0.01827
G1 X104.378 Y73.383 E0.28914
G1 X104.559 Y73.803 E0.01390
G1 X111.095 Y67.267 E0.28136
G1 X111.095 Y67.867 E0.01827
G1 X104.737 Y74.225 E0.27370
G1 X104.774 Y74.351 E0.00402
G1 X105.210 Y74.351 E0.01328
```

G1 X111.095 Y68.467 E0.25331

G1 X111.095 Y69.067 E0.01827

G1 X105.811 Y74.351 E0.22747

G1 X106.411 Y74.351 E0.01827

G1 X111.095 Y69.667 E0.20163

G1 X111.095 Y70.268 E0.01827

G1 X107.011 Y74.351 E0.17580

G1 X107.611 Y74.351 E0.01827

G1 X111.095 Y70.868 E0.14996

G1 X111.095 Y71.468 E0.01827

G1 X108.212 Y74.351 E0.12412

G1 X108.812 Y74.351 E0.01827

G1 X111.095 Y72.068 E0.09828

G1 X111.095 Y72.668 E0.01827

G1 X109.412 Y74.351 E0.07245

G1 X110.012 Y74.351 E0.01827

G1 X111.095 Y73.269 E0.04661

G1 X111.095 Y73.869 E0.01827

G1 X110.424 Y74.539 E0.02887

M204 S1250

; stop printing object Petg print.STL id:4 copy 0

; printing object tpu print.STL id:29 copy 0

; stop printing object tpu print.STL id:29 copy 0

; printing object tpu print.STL id:25 copy 0

; stop printing object tpu print.STL id:25 copy 0

; printing object Petg print.STL id:24 copy 0

G1 E-4.00000 F2400.000

G1 X141.344 Y69.840 F9000.000

G1 E4.00000 F900.000

```
M204 S800
;TYPE:Perimeter
;WIDTH:0.45
G1 F1292.454
G1 X141.344 Y66.126 E0.11130
G1 X149.516 Y66.126 E0.24487
M73 P37 R43
G1 X150.357 Y66.074 E0.02523
G1 X151.204 Y65.915 E0.02583
G1 X152.026 Y65.654 E0.02583
G1 X152.808 Y65.293 E0.02583
G1 X153.541 Y64.839 E0.02583
G1 X154.212 Y64.298 E0.02583
G1 X154.812 Y63.679 E0.02583
G1 X155.331 Y62.990 E0.02583
G1 X155.761 Y62.243 E0.02583
G1 X156.096 Y61.449 E0.02583
G1 X156.187 Y61.126 E0.01007
G1 X163.059 Y61.126 E0.20589
G1 X163.059 Y74.840 E0.41094
G1 X156.187 Y74.840 E0.20589
G1 X156.096 Y74.516 E0.01007
G1 X155.761 Y73.722 E0.02583
G1 X155.331 Y72.975 E0.02583
G1 X154.812 Y72.287 E0.02583
G1 X154.212 Y71.668 E0.02583
G1 X153.541 Y71.127 E0.02583
G1 X152.808 Y70.673 E0.02583
G1 X152.026 Y70.312 E0.02583
```

```
G1 X151.204 Y70.050 E0.02583
G1 X150.357 Y69.892 E0.02583
G1 X149.516 Y69.840 E0.02523
G1 X141.404 Y69.840 E0.24307
M204 S1250
G1 X140.926 Y70.258 F9000.000
M204 S800
;TYPE:External perimeter
G1 F1292.454
G1 X140.926 Y65.708 E0.13634
G1 X149.503 Y65.708 E0.25701
G1 X150.305 Y65.658 E0.02407
G1 X151.102 Y65.509 E0.02428
G1 X151.874 Y65.263 E0.02428
G1 X152.610 Y64.924 E0.02428
G1 X153.299 Y64.497 E0.02428
G1 X153.930 Y63.989 E0.02428
G1 X154.494 Y63.406 E0.02428
G1 X154.981 Y62.759 E0.02428
G1 X155.386 Y62.057 E0.02428
G1 X155.701 Y61.310 E0.02428
G1 X155.872 Y60.708 E0.01877
G1 X163.476 Y60.708 E0.22787
G1 X163.476 Y75.258 E0.43598
G1 X155.872 Y75.258 E0.22787
G1 X155.701 Y74.655 E0.01877
G1 X155.386 Y73.908 E0.02428
G1 X154.981 Y73.206 E0.02428
G1 X154.494 Y72.559 E0.02428
```

```
G1 X153.930 Y71.977 E0.02428
G1 X153.299 Y71.468 E0.02428
G1 X152.610 Y71.041 E0.02428
G1 X151.874 Y70.702 E0.02428
G1 X151.102 Y70.456 E0.02428
G1 X150.305 Y70.307 E0.02428
G1 X149.503 Y70.258 E0.02407
G1 X140.986 Y70.258 E0.25521
M204 S1250
G1 X141.127 Y69.912 F9000.000
G1 X142.328 Y66.251
M204 S1000
;TYPE:Solid infill
;WIDTH:0.456608
G1 F1272.331
G1 X141.658 Y66.921 E0.02886
G1 X141.658 Y67.522 E0.01827
G1 X142.740 Y66.439 E0.04661
G1 X143.341 Y66.439 E0.01827
G1 X141.658 Y68.122 E0.07244
G1 X141.658 Y68.722 E0.01827
G1 X143.941 Y66.439 E0.09828
G1 X144.541 Y66.439 E0.01827
G1 X141.658 Y69.322 E0.12412
G1 X141.658 Y69.527 E0.00622
G1 X142.053 Y69.527 E0.01205
G1 X145.141 Y66.439 E0.13291
G1 X145.741 Y66.439 E0.01827
G1 X142.654 Y69.527 E0.13291
```

```
G1 X143.254 Y69.527 E0.01827
G1 X146.342 Y66.439 E0.13291
G1 X146.942 Y66.439 E0.01827
G1 X143.854 Y69.527 E0.13291
G1 X144.454 Y69.527 E0.01827
G1 X147.542 Y66.439 E0.13291
G1 X148.142 Y66.439 E0.01827
G1 X145.055 Y69.527 E0.13291
G1 X145.655 Y69.527 E0.01827
G1 X148.742 Y66.439 E0.13291
G1 X149.343 Y66.439 E0.01827
G1 X146.255 Y69.527 E0.13291
G1 X146.855 Y69.527 E0.01827
G1 X149.970 Y66.411 E0.13409
G1 X150.396 Y66.385 E0.01297
G1 X150.643 Y66.339 E0.00767
G1 X147.455 Y69.527 E0.13722
G1 X148.056 Y69.527 E0.01827
G1 X151.401 Y66.181 E0.14400
G1 X152.139 Y65.946 E0.02358
G1 X152.319 Y65.863 E0.00604
G1 X148.468 Y69.715 E0.16578
M204 S1250
G1 E-4.00000 F2400.000
G1 X155.860 Y62.323 F9000.000
G1 E4.00000 F900.000
M204 S1000
G1 F1272.331
G1 X156.744 Y61.439 E0.03805
```

```
G1 X157.344 Y61.439 E0.01827
G1 X149.256 Y69.527 E0.34814
G1 X149.837 Y69.546 E0.01769
G1 X157.944 Y61.439 E0.34897
G1 X158.544 Y61.439 E0.01827
G1 X150.402 Y69.581 E0.35050
G1 X150.907 Y69.676 E0.01566
G1 X159.144 Y61.439 E0.35457
G1 X159.745 Y61.439 E0.01827
G1 X151.400 Y69.784 E0.35921
G1 X151.855 Y69.929 E0.01454
G1 X160.345 Y61.439 E0.36545
G1 X160.945 Y61.439 E0.01827
G1 X152.294 Y70.090 E0.37241
G1 X152.705 Y70.280 E0.01377
G1 X161.545 Y61.439 E0.38056
G1 X162.146 Y61.439 E0.01827
G1 X153.100 Y70.485 E0.38938
G1 X153.470 Y70.714 E0.01327
G1 X162.745 Y61.439 E0.39924
G1 X162.745 Y62.040 E0.01827
G1 X153.829 Y70.956 E0.38382
G1 X154.161 Y71.224 E0.01299
G1 X162.745 Y62.640 E0.36951
G1 X162.745 Y63.240 E0.01827
G1 X154.486 Y71.500 E0.35554
G1 X154.781 Y71.805 E0.01292
G1 X162.745 Y63.840 E0.34283
G1 X162.745 Y64.440 E0.01827
```

```
G1 X155.073 Y72.113 E0.33026
G1 X155.331 Y72.455 E0.01305
G1 X162.745 Y65.041 E0.31916
G1 X162.745 Y65.641 E0.01827
G1 X155.589 Y72.797 E0.30805
G1 X155.809 Y73.178 E0.01337
G1 X162.745 Y66.241 E0.29859
G1 X162.745 Y66.841 E0.01827
G1 X156.028 Y73.558 E0.28914
G1 X156.209 Y73.978 E0.01390
G1 X162.745 Y67.442 E0.28136
G1 X162.745 Y68.042 E0.01827
G1 X156.387 Y74.400 E0.27370
G1 X156.424 Y74.527 E0.00402
G1 X156.861 Y74.527 E0.01328
G1 X162.745 Y68.642 E0.25331
G1 X162.745 Y69.242 E0.01827
G1 X157.461 Y74.527 E0.22747
G1 X158.061 Y74.527 E0.01827
G1 X162.745 Y69.842 E0.20163
G1 X162.745 Y70.443 E0.01827
G1 X158.661 Y74.527 E0.17580
G1 X159.262 Y74.527 E0.01827
G1 X162.745 Y71.043 E0.14996
G1 X162.745 Y71.643 E0.01827
G1 X159.862 Y74.527 E0.12412
G1 X160.462 Y74.527 E0.01827
G1 X162.745 Y72.243 E0.09828
G1 X162.745 Y72.844 E0.01827
```

G1 X161.062 Y74.527 E0.07245

G1 X161.662 Y74.527 E0.01827

G1 X162.745 Y73.444 E0.04661

G1 X162.745 Y74.044 E0.01827

G1 X162.075 Y74.715 E0.02887

M204 S1250

; stop printing object Petg print.STL id:24 copy 0

; printing object Petg print.STL id:28 copy 0

G1 E-4.00000 F2400.000

G1 X163.051 Y54.891 F9000.000

G1 E4.00000 F900.000

M204 S800

;TYPE:Perimeter

;WIDTH:0.45

G1 F1292.454

G1 X156.180 Y54.891 E0.20589

G1 X156.088 Y54.567 E0.01007

G1 X155.753 Y53.773 E0.02583

G1 X155.323 Y53.026 E0.02583

G1 X154.804 Y52.338 E0.02583

G1 X154.205 Y51.719 E0.02583

G1 X153.534 Y51.178 E0.02583

G1 X152.801 Y50.724 E0.02583

G1 X152.018 Y50.363 E0.02583

G1 X151.197 Y50.101 E0.02583

G1 X150.349 Y49.943 E0.02583

G1 X149.509 Y49.891 E0.02523

G1 X141.337 Y49.891 E0.24487

G1 X141.337 Y46.177 E0.11130

```
G1 X149.509 Y46.177 E0.24487
G1 X150.349 Y46.125 E0.02523
G1 X151.197 Y45.966 E0.02583
G1 X152.018 Y45.705 E0.02583
G1 X152.801 Y45.344 E0.02583
G1 X153.534 Y44.890 E0.02583
G1 X154.205 Y44.349 E0.02583
G1 X154.804 Y43.730 E0.02583
G1 X155.323 Y43.041 E0.02583
G1 X155.753 Y42.294 E0.02583
G1 X156.088 Y41.500 E0.02583
G1 X156.180 Y41.177 E0.01007
G1 X163.051 Y41.177 E0.20589
G1 X163.051 Y54.831 E0.40914
M204 S1250
G1 X163.469 Y55.309 F9000.000
M204 S800
;TYPE:External perimeter
G1 F1292.454
G1 X155.864 Y55.309 E0.22787
G1 X155.693 Y54.706 E0.01877
G1 X155.378 Y53.959 E0.02428
G1 X154.974 Y53.257 E0.02428
G1 X154.486 Y52.610 E0.02428
G1 X153.923 Y52.028 E0.02428
G1 X153.292 Y51.519 E0.02428
G1 X152.603 Y51.092 E0.02428
G1 X151.867 Y50.753 E0.02428
M73 P37 R42
```

```
G1 X151.094 Y50.507 E0.02428
G1 X150.298 Y50.358 E0.02428
G1 X149.496 Y50.309 E0.02407
G1 X140.919 Y50.309 E0.25701
G1 X140.919 Y45.759 E0.13634
G1 X149.496 Y45.759 E0.25701
G1 X150.298 Y45.709 E0.02407
G1 X151.094 Y45.560 E0.02428
G1 X151.867 Y45.314 E0.02428
G1 X152.603 Y44.975 E0.02428
G1 X153.292 Y44.548 E0.02428
G1 X153.923 Y44.040 E0.02428
G1 X154.486 Y43.458 E0.02428
G1 X154.974 Y42.810 E0.02428
G1 X155.378 Y42.108 E0.02428
G1 X155.693 Y41.361 E0.02428
G1 X155.864 Y40.759 E0.01877
G1 X163.469 Y40.759 E0.22787
G1 X163.469 Y55.249 E0.43418
M204 S1250
G1 X163.095 Y55.167 F9000.000
G1 X162.067 Y54.766
M204 S1000
;TYPE:Solid infill
;WIDTH:0.456608
G1 F1272.331
G1 X162.738 Y54.095 E0.02887
G1 X162.738 Y53.495 E0.01827
G1 X161.655 Y54.578 E0.04661
```

```
G1 X161.055 Y54.578 E0.01827
G1 X162.738 Y52.895 E0.07245
G1 X162.738 Y52.294 E0.01827
G1 X160.454 Y54.578 E0.09828
G1 X159.854 Y54.578 E0.01827
G1 X162.738 Y51.694 E0.12412
G1 X162.738 Y51.094 E0.01827
G1 X159.254 Y54.578 E0.14996
G1 X158.654 Y54.578 E0.01827
G1 X162.738 Y50.494 E0.17580
G1 X162.738 Y49.893 E0.01827
G1 X158.054 Y54.578 E0.20163
G1 X157.453 Y54.578 E0.01827
G1 X162.738 Y49.293 E0.22747
G1 X162.738 Y48.693 E0.01827
G1 X156.853 Y54.578 E0.25331
G1 X156.417 Y54.578 E0.01328
G1 X156.379 Y54.451 E0.00402
G1 X162.738 Y48.093 E0.27370
G1 X162.738 Y47.493 E0.01827
G1 X156.201 Y54.029 E0.28136
G1 X156.021 Y53.610 E0.01390
G1 X162.738 Y46.892 E0.28914
G1 X162.738 Y46.292 E0.01827
G1 X155.801 Y53.229 E0.29859
G1 X155.581 Y52.848 E0.01337
G1 X162.738 Y45.692 E0.30805
G1 X162.738 Y45.092 E0.01827
G1 X155.323 Y52.506 E0.31916
```

```
G1 X155.065 Y52.164 E0.01305
G1 X162.738 Y44.492 E0.33026
G1 X162.738 Y43.891 E0.01827
G1 X154.773 Y51.856 E0.34283
G1 X154.478 Y51.551 E0.01292
G1 X162.738 Y43.291 E0.35554
G1 X162.738 Y42.691 E0.01827
G1 X154.154 Y51.275 E0.36951
G1 X153.821 Y51.007 E0.01299
G1 X162.738 Y42.091 E0.38382
G1 X162.738 Y41.490 E0.01827
G1 X153.463 Y50.765 E0.39924
M73 P38 R42
G1 X153.092 Y50.536 E0.01327
G1 X162.138 Y41.490 E0.38938
G1 X161.538 Y41.490 E0.01827
G1 X152.697 Y50.331 E0.38056
G1 X152.286 Y50.141 E0.01377
G1 X160.938 Y41.490 E0.37241
G1 X160.337 Y41.490 E0.01827
G1 X151.848 Y49.980 E0.36545
G1 X151.392 Y49.835 E0.01454
G1 X159.737 Y41.490 E0.35921
G1 X159.137 Y41.490 E0.01827
G1 X150.900 Y49.727 E0.35457
G1 X150.394 Y49.632 E0.01566
G1 X158.537 Y41.490 E0.35050
G1 X157.937 Y41.490 E0.01827
G1 X149.830 Y49.597 E0.34897
```

```
G1 X149.249 Y49.578 E0.01769
G1 X157.336 Y41.490 E0.34814
G1 X156.736 Y41.490 E0.01827
G1 X155.852 Y42.374 E0.03805
M204 S1250
G1 E-4.00000 F2400.000
G1 X148.460 Y49.766 F9000.000
G1 E4.00000 F900.000
M204 S1000
G1 F1272.331
G1 X152.312 Y45.914 E0.16578
G1 X152.132 Y45.997 E0.00604
G1 X151.393 Y46.232 E0.02358
G1 X148.048 Y49.578 E0.14400
G1 X147.448 Y49.578 E0.01827
G1 X150.636 Y46.390 E0.13722
G1 X150.388 Y46.436 E0.00767
G1 X149.963 Y46.463 E0.01297
G1 X146.848 Y49.578 E0.13409
G1 X146.248 Y49.578 E0.01827
G1 X149.335 Y46.490 E0.13291
G1 X148.735 Y46.490 E0.01827
G1 X145.647 Y49.578 E0.13291
G1 X145.047 Y49.578 E0.01827
G1 X148.135 Y46.490 E0.13291
G1 X147.535 Y46.490 E0.01827
G1 X144.447 Y49.578 E0.13291
G1 X143.847 Y49.578 E0.01827
G1 X146.934 Y46.490 E0.13291
```

```
G1 X146.334 Y46.490 E0.01827
G1 X143.246 Y49.578 E0.13291
G1 X142.646 Y49.578 E0.01827
G1 X145.734 Y46.490 E0.13291
G1 X145.134 Y46.490 E0.01827
G1 X142.046 Y49.578 E0.13291
G1 X141.650 Y49.578 E0.01205
G1 X141.650 Y49.373 E0.00622
G1 X144.533 Y46.490 E0.12412
G1 X143.933 Y46.490 E0.01827
G1 X141.650 Y48.773 E0.09828
G1 X141.650 Y48.173 E0.01827
G1 X143.333 Y46.490 E0.07244
G1 X142.733 Y46.490 E0.01827
G1 X141.650 Y47.573 E0.04661
G1 X141.650 Y46.972 E0.01827
G1 X142.321 Y46.302 E0.02886
M204 S1250
; stop printing object Petg print.STL id:28 copy 0
;LAYER_CHANGE
;Z:0.5
;HEIGHT:0.15
;BEFORE_LAYER_CHANGE
G92 E0.0
;0.5


G1 E-4.00000 F2400.000
G1 Z0.500 F9000.000
```

```
;AFTER_LAYER_CHANGE
;0.5
G1 X164.613 Y38.341
G1 E4.00000 F900.000
;TYPE:Skirt
;WIDTH:0.42
G1 F1392.435
G1 X165.363 Y38.841 E0.02506
G1 X165.877 Y39.594 E0.02537
G1 X166.070 Y40.486 E0.02537
G1 X166.078 Y75.483 E0.97336
G1 X166.019 Y137.174 E1.71578
G1 X165.837 Y138.056 E0.02506
G1 X165.338 Y138.810 E0.02516
G1 X164.568 Y139.334 E0.02589
G1 X163.656 Y139.522 E0.02589
G1 X119.638 Y139.522 E1.22425
G1 X68.980 Y139.347 E1.40895
G1 X20.144 Y138.765 E1.35836
G1 X19.274 Y138.577 E0.02475
G1 X18.534 Y138.082 E0.02475
G1 X18.012 Y137.312 E0.02589
G1 X17.825 Y136.399 E0.02589
G1 X17.877 Y39.772 E2.68748
G1 X18.074 Y38.831 E0.02672
G1 X18.424 Y38.262 E0.01858
G1 X18.918 Y37.812 E0.01858
G1 X19.518 Y37.518 E0.01858
G1 X20.176 Y37.402 E0.01858
```

```
G1 X62.258 Y37.401 E1.17042
G1 X163.727 Y38.157 E2.82219
G1 X164.554 Y38.329 E0.02350
; printing object tpu print.STL id:19 copy 0
; stop printing object tpu print.STL id:19 copy 0
; printing object Petg print.STL id:18 copy 0
G1 E-4.00000 F2400.000
G1 X61.611 Y40.421 F9000.000
G1 E4.00000 F900.000
M204 S800
;TYPE:Perimeter
;WIDTH:0.45
G1 F1292.454
G1 X61.611 Y54.135 E0.41094
G1 X54.740 Y54.135 E0.20589
G1 X54.649 Y53.812 E0.01007
G1 X54.314 Y53.017 E0.02583
G1 X53.884 Y52.270 E0.02583
G1 X53.365 Y51.582 E0.02583
G1 X52.765 Y50.963 E0.02583
G1 X52.094 Y50.422 E0.02583
G1 X51.361 Y49.968 E0.02583
G1 X50.578 Y49.607 E0.02583
G1 X49.757 Y49.345 E0.02583
G1 X48.910 Y49.187 E0.02583
G1 X48.069 Y49.135 E0.02523
G1 X39.897 Y49.135 E0.24487
G1 X39.897 Y45.421 E0.11130
G1 X48.069 Y45.421 E0.24487
```

```
G1 X48.910 Y45.369 E0.02523
G1 X49.757 Y45.210 E0.02583
G1 X50.578 Y44.949 E0.02583
G1 X51.361 Y44.588 E0.02583
G1 X52.094 Y44.134 E0.02583
G1 X52.765 Y43.593 E0.02583
G1 X53.365 Y42.974 E0.02583
G1 X53.884 Y42.285 E0.02583
G1 X54.314 Y41.538 E0.02583
G1 X54.649 Y40.744 E0.02583
G1 X54.740 Y40.421 E0.01007
G1 X61.551 Y40.421 E0.20409
M204 S1250
G1 X62.029 Y40.003 F9000.000
M204 S800
;TYPE:External perimeter
G1 F1292.454
G1 X62.029 Y54.553 E0.43598
G1 X54.424 Y54.553 E0.22787
G1 X54.254 Y53.950 E0.01877
G1 X53.939 Y53.203 E0.02428
G1 X53.534 Y52.501 E0.02428
G1 X53.047 Y51.854 E0.02428
G1 X52.483 Y51.272 E0.02428
G1 X51.852 Y50.763 E0.02428
G1 X51.163 Y50.336 E0.02428
G1 X50.427 Y49.997 E0.02428
G1 X49.655 Y49.751 E0.02428
G1 X48.858 Y49.602 E0.02428
```

```
G1 X48.056 Y49.553 E0.02407
G1 X39.479 Y49.553 E0.25701
G1 X39.479 Y45.003 E0.13634
G1 X48.056 Y45.003 E0.25701
G1 X48.858 Y44.953 E0.02407
G1 X49.655 Y44.804 E0.02428
G1 X50.427 Y44.558 E0.02428
G1 X51.163 Y44.219 E0.02428
G1 X51.852 Y43.792 E0.02428
G1 X52.483 Y43.284 E0.02428
G1 X53.047 Y42.702 E0.02428
G1 X53.534 Y42.054 E0.02428
G1 X53.939 Y41.352 E0.02428
G1 X54.254 Y40.606 E0.02428
G1 X54.424 Y40.003 E0.01877
G1 X61.969 Y40.003 E0.22608
M204 S1250
G1 X61.838 Y40.354 F9000.000
G1 X61.486 Y41.405
M204 S1000
;TYPE:Solid infill
;WIDTH:0.456609
G1 F1272.328
G1 X60.816 Y40.734 E0.02887
G1 X60.215 Y40.734 E0.01827
G1 X61.298 Y41.817 E0.04661
G1 X61.298 Y42.417 E0.01827
G1 X59.615 Y40.734 E0.07245
G1 X59.015 Y40.734 E0.01827
```

```
G1 X61.298 Y43.017 E0.09828
G1 X61.298 Y43.617 E0.01827
G1 X58.415 Y40.734 E0.12412
G1 X57.814 Y40.734 E0.01827
G1 X61.298 Y44.218 E0.14996
G1 X61.298 Y44.818 E0.01827
G1 X57.214 Y40.734 E0.17580
M73 P39 R42
G1 X56.614 Y40.734 E0.01827
G1 X61.298 Y45.418 E0.20163
G1 X61.298 Y46.018 E0.01827
G1 X56.014 Y40.734 E0.22747
G1 X55.414 Y40.734 E0.01827
G1 X61.298 Y46.619 E0.25331
G1 X61.298 Y47.219 E0.01827
G1 X54.940 Y40.860 E0.27370
G1 X54.762 Y41.283 E0.01395
G1 X61.298 Y47.819 E0.28137
G1 X61.298 Y48.419 E0.01827
G1 X54.581 Y41.702 E0.28914
G1 X54.362 Y42.083 E0.01338
G1 X61.298 Y49.019 E0.29859
G1 X61.298 Y49.620 E0.01827
G1 X54.142 Y42.463 E0.30805
G1 X53.884 Y42.806 E0.01305
G1 X61.298 Y50.220 E0.31916
G1 X61.298 Y50.820 E0.01827
G1 X53.626 Y43.148 E0.33026
G1 X53.334 Y43.456 E0.01292
```

```
G1 X61.298 Y51.420 E0.34283
G1 X61.298 Y52.021 E0.01827
G1 X53.039 Y43.761 E0.35554
G1 X52.714 Y44.037 E0.01296
G1 X61.298 Y52.621 E0.36951
G1 X61.298 Y53.221 E0.01827
G1 X52.382 Y44.304 E0.38382
G1 X52.023 Y44.546 E0.01316
G1 X61.298 Y53.822 E0.39926
M73 P39 R41
G1 X60.698 Y53.822 E0.01825
G1 X51.653 Y44.776 E0.38938
G1 X51.510 Y44.865 E0.00511
G1 X51.257 Y44.981 E0.00847
G1 X60.098 Y53.822 E0.38056
G1 X59.498 Y53.822 E0.01827
G1 X50.846 Y45.170 E0.37241
G1 X50.692 Y45.241 E0.00518
G1 X50.408 Y45.332 E0.00907
G1 X58.898 Y53.822 E0.36545
G1 X58.298 Y53.822 E0.01827
G1 X49.953 Y45.477 E0.35921
G1 X49.460 Y45.585 E0.01534
G1 X57.697 Y53.822 E0.35457
G1 X57.097 Y53.822 E0.01827
G1 X48.955 Y45.679 E0.35050
G1 X48.390 Y45.715 E0.01722
G1 X56.497 Y53.822 E0.34897
G1 X55.897 Y53.822 E0.01827
```

G1 X47.809 Y45.734 E0.34814

G1 X47.209 Y45.734 E0.01827

G1 X50.872 Y49.397 E0.15769

G1 X50.692 Y49.314 E0.00603

G1 X49.954 Y49.079 E0.02358

G1 X46.609 Y45.734 E0.14400

G1 X46.008 Y45.734 E0.01827

G1 X49.196 Y48.922 E0.13722

G1 X48.948 Y48.875 E0.00767

G1 X48.523 Y48.849 E0.01297

G1 X45.408 Y45.734 E0.13409

G1 X44.808 Y45.734 E0.01827

G1 X47.896 Y48.822 E0.13291

G1 X47.295 Y48.822 E0.01827

G1 X44.208 Y45.734 E0.13291

G1 X43.607 Y45.734 E0.01827

G1 X46.695 Y48.822 E0.13291

G1 X46.095 Y48.822 E0.01827

G1 X43.007 Y45.734 E0.13291

G1 X42.407 Y45.734 E0.01827

G1 X45.495 Y48.822 E0.13291

G1 X44.894 Y48.822 E0.01827

G1 X41.807 Y45.734 E0.13291

G1 X41.207 Y45.734 E0.01827

G1 X44.294 Y48.822 E0.13291

G1 X43.694 Y48.822 E0.01827

G1 X40.606 Y45.734 E0.13291

G1 X40.210 Y45.734 E0.01205

G1 X40.210 Y45.938 E0.00622

```
G1 X43.094 Y48.822 E0.12412
G1 X42.494 Y48.822 E0.01827
G1 X40.210 Y46.539 E0.09828
G1 X40.210 Y47.139 E0.01827
G1 X41.893 Y48.822 E0.07244
G1 X41.293 Y48.822 E0.01827
G1 X40.210 Y47.739 E0.04660
G1 X40.210 Y48.339 E0.01827
G1 X40.881 Y49.010 E0.02886
M204 S1250
G1 E-4.00000 F2400.000
G1 X54.413 Y52.938 F9000.000
G1 E4.00000 F900.000
M204 S1000
G1 F1272.328
G1 X55.484 Y54.010 E0.04614
M204 S1250
; stop printing object Petg print.STL id:18 copy 0
; printing object Petg print.STL id:14 copy 0
G1 X54.748 Y60.370 F9000.000
M204 S800
;TYPE:Perimeter
;WIDTH:0.45
G1 F1292.454
G1 X61.619 Y60.370 E0.20589
G1 X61.619 Y74.084 E0.41094
G1 X54.748 Y74.084 E0.20589
G1 X54.656 Y73.760 E0.01007
G1 X54.321 Y72.966 E0.02583
```

```
G1 X53.891 Y72.219 E0.02583
G1 X53.372 Y71.531 E0.02583
G1 X52.773 Y70.912 E0.02583
G1 X52.101 Y70.371 E0.02583
G1 X51.369 Y69.917 E0.02583
G1 X50.586 Y69.556 E0.02583
G1 X49.765 Y69.294 E0.02583
G1 X48.917 Y69.136 E0.02583
G1 X48.077 Y69.084 E0.02523
G1 X39.905 Y69.084 E0.24487
G1 X39.905 Y65.370 E0.11130
G1 X48.077 Y65.370 E0.24487
G1 X48.917 Y65.318 E0.02523
G1 X49.765 Y65.159 E0.02583
G1 X50.586 Y64.898 E0.02583
G1 X51.369 Y64.537 E0.02583
G1 X52.101 Y64.083 E0.02583
G1 X52.773 Y63.542 E0.02583
G1 X53.372 Y62.923 E0.02583
G1 X53.891 Y62.234 E0.02583
G1 X54.321 Y61.487 E0.02583
G1 X54.656 Y60.693 E0.02583
G1 X54.732 Y60.427 E0.00828
M204 S1250
G1 X54.432 Y59.952 F9000.000
M204 S800
;TYPE:External perimeter
G1 F1292.454
G1 X62.037 Y59.952 E0.22787
```

```
G1 X62.037 Y74.502 E0.43598
G1 X54.432 Y74.502 E0.22787
G1 X54.261 Y73.899 E0.01877
G1 X53.946 Y73.152 E0.02428
G1 X53.542 Y72.450 E0.02428
G1 X53.054 Y71.803 E0.02428
G1 X52.490 Y71.221 E0.02428
G1 X51.859 Y70.712 E0.02428
G1 X51.171 Y70.285 E0.02428
G1 X50.435 Y69.946 E0.02428
G1 X49.662 Y69.700 E0.02428
G1 X48.866 Y69.551 E0.02428
G1 X48.064 Y69.502 E0.02407
G1 X39.487 Y69.502 E0.25701
G1 X39.487 Y64.952 E0.13634
G1 X48.064 Y64.952 E0.25701
G1 X48.866 Y64.902 E0.02407
G1 X49.662 Y64.753 E0.02428
G1 X50.435 Y64.507 E0.02428
G1 X51.171 Y64.168 E0.02428
G1 X51.859 Y63.741 E0.02428
G1 X52.490 Y63.233 E0.02428
G1 X53.054 Y62.651 E0.02428
G1 X53.542 Y62.003 E0.02428
G1 X53.946 Y61.301 E0.02428
G1 X54.261 Y60.554 E0.02428
G1 X54.416 Y60.010 E0.01697
M204 S1250
G1 X54.753 Y60.190 F9000.000
```

```
G1 E-4.00000 F2400.000
G1 X61.494 Y61.354 F9000.000
G1 E4.00000 F900.000
M204 S1000
;TYPE:Solid infill
;WIDTH:0.456609
G1 F1272.328
G1 X60.823 Y60.683 E0.02887
G1 X60.223 Y60.683 E0.01827
G1 X61.306 Y61.766 E0.04661
G1 X61.306 Y62.366 E0.01827
G1 X59.623 Y60.683 E0.07245
G1 X59.022 Y60.683 E0.01827
G1 X61.306 Y62.966 E0.09828
G1 X61.306 Y63.566 E0.01827
G1 X58.422 Y60.683 E0.12412
G1 X57.822 Y60.683 E0.01827
G1 X61.306 Y64.167 E0.14996
G1 X61.306 Y64.767 E0.01827
G1 X57.222 Y60.683 E0.17580
G1 X56.621 Y60.683 E0.01827
G1 X61.306 Y65.367 E0.20163
G1 X61.306 Y65.967 E0.01827
G1 X56.021 Y60.683 E0.22747
G1 X55.421 Y60.683 E0.01827
G1 X61.306 Y66.568 E0.25331
G1 X61.306 Y67.168 E0.01827
G1 X54.947 Y60.809 E0.27370
G1 X54.769 Y61.232 E0.01395
```

```
G1 X61.306 Y67.768 E0.28137
G1 X61.306 Y68.368 E0.01827
G1 X54.589 Y61.651 E0.28914
G1 X54.369 Y62.032 E0.01338
G1 X61.306 Y68.968 E0.29859
G1 X61.306 Y69.569 E0.01827
G1 X54.149 Y62.412 E0.30805
G1 X53.891 Y62.755 E0.01305
G1 X61.306 Y70.169 E0.31916
G1 X61.306 Y70.769 E0.01827
G1 X53.633 Y63.097 E0.33026
G1 X53.341 Y63.405 E0.01292
G1 X61.306 Y71.369 E0.34283
G1 X61.306 Y71.969 E0.01827
G1 X53.046 Y63.710 E0.35554
G1 X52.721 Y63.986 E0.01296
G1 X61.306 Y72.570 E0.36951
G1 X61.306 Y73.170 E0.01827
G1 X52.389 Y64.253 E0.38382
G1 X52.031 Y64.495 E0.01316
G1 X61.306 Y73.771 E0.39926
G1 X60.706 Y73.771 E0.01825
G1 X51.660 Y64.725 E0.38938
G1 X51.518 Y64.813 E0.00511
G1 X51.265 Y64.930 E0.00847
G1 X60.106 Y73.771 E0.38056
G1 X59.505 Y73.771 E0.01827
G1 X50.854 Y65.119 E0.37241
G1 X50.699 Y65.190 E0.00518
```

```
G1 X50.415 Y65.281 E0.00907
G1 X58.905 Y73.771 E0.36545
G1 X58.305 Y73.771 E0.01827
G1 X49.960 Y65.426 E0.35921
G1 X49.468 Y65.534 E0.01534
G1 X57.705 Y73.771 E0.35457
G1 X57.105 Y73.771 E0.01827
G1 X48.962 Y65.628 E0.35050
G1 X48.397 Y65.664 E0.01722
G1 X56.504 Y73.771 E0.34897
G1 X55.904 Y73.771 E0.01827
G1 X47.816 Y65.683 E0.34814
G1 X47.216 Y65.683 E0.01827
G1 X50.880 Y69.346 E0.15769
G1 X50.699 Y69.263 E0.00603
G1 X49.961 Y69.028 E0.02358
G1 X46.616 Y65.683 E0.14400
G1 X46.016 Y65.683 E0.01827
G1 X49.203 Y68.871 E0.13722
G1 X48.956 Y68.824 E0.00767
G1 X48.531 Y68.798 E0.01297
G1 X45.416 Y65.683 E0.13409
G1 X44.815 Y65.683 E0.01827
G1 X47.903 Y68.771 E0.13291
G1 X47.303 Y68.771 E0.01827
G1 X44.215 Y65.683 E0.13291
M73 P40 R41
G1 X43.615 Y65.683 E0.01827
G1 X46.703 Y68.771 E0.13291
```

```
G1 X46.102 Y68.771 E0.01827
G1 X43.015 Y65.683 E0.13291
G1 X42.414 Y65.683 E0.01827
G1 X45.502 Y68.771 E0.13291
G1 X44.902 Y68.771 E0.01827
G1 X41.814 Y65.683 E0.13291
G1 X41.214 Y65.683 E0.01827
G1 X44.302 Y68.771 E0.13291
G1 X43.701 Y68.771 E0.01827
G1 X40.614 Y65.683 E0.13291
G1 X40.218 Y65.683 E0.01205
G1 X40.218 Y65.887 E0.00622
G1 X43.101 Y68.771 E0.12412
G1 X42.501 Y68.771 E0.01827
G1 X40.218 Y66.488 E0.09828
G1 X40.218 Y67.088 E0.01827
G1 X41.901 Y68.771 E0.07244
G1 X41.301 Y68.771 E0.01827
G1 X40.218 Y67.688 E0.04660
G1 X40.218 Y68.288 E0.01827
G1 X40.888 Y68.959 E0.02886
M204 S1250
G1 E-4.00000 F2400.000
G1 X54.420 Y72.887 F9000.000
G1 E4.00000 F900.000
M204 S1000
G1 F1272.328
G1 X55.492 Y73.959 E0.04614
M204 S1250
```

```
; stop printing object Petg print.STL id:14 copy 0
; printing object tpu print.STL id:15 copy 0
; stop printing object tpu print.STL id:15 copy 0
; printing object Petg print.STL id:10 copy 0
G1 E-4.00000 F2400.000
G1 X54.697 Y82.562 F9000.000
G1 E4.00000 F900.000
M204 S800
;TYPE:Perimeter
;WIDTH:0.45
G1 F1292.454
G1 X61.568 Y82.562 E0.20589
G1 X61.568 Y96.276 E0.41094
G1 X54.697 Y96.276 E0.20589
G1 X54.605 Y95.953 E0.01007
G1 X54.270 Y95.159 E0.02583
G1 X53.840 Y94.412 E0.02583
G1 X53.321 Y93.723 E0.02583
G1 X52.722 Y93.104 E0.02583
G1 X52.050 Y92.563 E0.02583
G1 X51.318 Y92.109 E0.02583
G1 X50.535 Y91.748 E0.02583
G1 X49.713 Y91.487 E0.02583
G1 X48.866 Y91.328 E0.02583
G1 X48.026 Y91.276 E0.02523
G1 X39.853 Y91.276 E0.24487
G1 X39.853 Y87.562 E0.11130
G1 X48.026 Y87.562 E0.24487
G1 X48.866 Y87.510 E0.02523
```

```
G1 X49.713 Y87.351 E0.02583
G1 X50.535 Y87.090 E0.02583
G1 X51.318 Y86.729 E0.02583
G1 X52.050 Y86.275 E0.02583
G1 X52.722 Y85.734 E0.02583
G1 X53.321 Y85.115 E0.02583
G1 X53.840 Y84.427 E0.02583
G1 X54.270 Y83.680 E0.02583
G1 X54.605 Y82.885 E0.02583
G1 X54.680 Y82.620 E0.00828
M204 S1250
G1 X54.381 Y82.144 F9000.000
M204 S800
;TYPE:External perimeter
G1 F1292.454
G1 X61.986 Y82.144 E0.22787
G1 X61.986 Y96.694 E0.43598
G1 X54.381 Y96.694 E0.22787
G1 X54.210 Y96.091 E0.01877
G1 X53.895 Y95.345 E0.02428
G1 X53.491 Y94.643 E0.02428
G1 X53.003 Y93.995 E0.02428
G1 X52.439 Y93.413 E0.02428
G1 X51.808 Y92.905 E0.02428
G1 X51.119 Y92.478 E0.02428
G1 X50.383 Y92.139 E0.02428
G1 X49.611 Y91.893 E0.02428
G1 X48.815 Y91.744 E0.02428
G1 X48.013 Y91.694 E0.02407
```

```
G1 X39.436 Y91.694 E0.25701
G1 X39.436 Y87.144 E0.13634
G1 X48.013 Y87.144 E0.25701
G1 X48.815 Y87.095 E0.02407
G1 X49.611 Y86.946 E0.02428
G1 X50.383 Y86.700 E0.02428
G1 X51.119 Y86.361 E0.02428
G1 X51.808 Y85.934 E0.02428
G1 X52.439 Y85.425 E0.02428
G1 X53.003 Y84.843 E0.02428
G1 X53.491 Y84.196 E0.02428
G1 X53.895 Y83.493 E0.02428
G1 X54.210 Y82.747 E0.02428
G1 X54.364 Y82.202 E0.01697
M204 S1250
G1 X54.702 Y82.383 F9000.000
G1 E-4.00000 F2400.000
G1 X61.442 Y83.546 F9000.000
G1 E4.00000 F900.000
M204 S1000
;TYPE:Solid infill
;WIDTH:0.456609
G1 F1272.328
G1 X60.772 Y82.875 E0.02887
G1 X60.172 Y82.875 E0.01827
G1 X61.254 Y83.958 E0.04661
G1 X61.254 Y84.558 E0.01827
G1 X59.571 Y82.875 E0.07245
G1 X58.971 Y82.875 E0.01827
```

```
G1 X61.254 Y85.159 E0.09828
G1 X61.254 Y85.759 E0.01827
G1 X58.371 Y82.875 E0.12412
G1 X57.771 Y82.875 E0.01827
G1 X61.254 Y86.359 E0.14996
G1 X61.254 Y86.959 E0.01827
G1 X57.171 Y82.875 E0.17580
G1 X56.570 Y82.875 E0.01827
G1 X61.254 Y87.559 E0.20163
G1 X61.254 Y88.160 E0.01827
G1 X55.970 Y82.875 E0.22747
G1 X55.370 Y82.875 E0.01827
G1 X61.254 Y88.760 E0.25331
G1 X61.254 Y89.360 E0.01827
G1 X54.896 Y83.002 E0.27370
G1 X54.718 Y83.424 E0.01395
G1 X61.254 Y89.960 E0.28137
G1 X61.254 Y90.561 E0.01827
G1 X54.537 Y83.843 E0.28914
G1 X54.318 Y84.224 E0.01338
G1 X61.254 Y91.161 E0.29859
G1 X61.254 Y91.761 E0.01827
G1 X54.098 Y84.605 E0.30805
G1 X53.840 Y84.947 E0.01305
G1 X61.254 Y92.361 E0.31916
G1 X61.254 Y92.961 E0.01827
G1 X53.582 Y85.289 E0.33026
G1 X53.290 Y85.597 E0.01292
G1 X61.254 Y93.562 E0.34283
```

G1 X61.254 Y94.162 E0.01827

G1 X52.995 Y85.902 E0.35554

G1 X52.670 Y86.178 E0.01296

G1 X61.254 Y94.762 E0.36951

G1 X61.254 Y95.362 E0.01827

G1 X52.338 Y86.446 E0.38382

G1 X51.980 Y86.688 E0.01316

G1 X61.254 Y95.963 E0.39926

G1 X60.655 Y95.963 E0.01825

G1 X51.609 Y86.917 E0.38938

G1 X51.466 Y87.006 E0.00511

G1 X51.214 Y87.122 E0.00847

G1 X60.055 Y95.963 E0.38056

G1 X59.454 Y95.963 E0.01827

G1 X50.803 Y87.311 E0.37241

G1 X50.648 Y87.383 E0.00518

G1 X50.364 Y87.473 E0.00907

G1 X58.854 Y95.963 E0.36545

G1 X58.254 Y95.963 E0.01827

G1 X49.909 Y87.618 E0.35921

G1 X49.417 Y87.726 E0.01534

G1 X57.654 Y95.963 E0.35457

G1 X57.053 Y95.963 E0.01827

G1 X48.911 Y87.820 E0.35050

G1 X48.346 Y87.856 E0.01722

G1 X56.453 Y95.963 E0.34897

G1 X55.853 Y95.963 E0.01827

G1 X47.765 Y87.875 E0.34814

G1 X47.165 Y87.875 E0.01827

```
G1 X50.828 Y91.539 E0.15769
G1 X50.648 Y91.456 E0.00603
G1 X49.910 Y91.221 E0.02358
G1 X46.565 Y87.875 E0.14400
G1 X45.965 Y87.875 E0.01827
G1 X49.152 Y91.063 E0.13722
G1 X48.905 Y91.017 E0.00767
G1 X48.480 Y90.990 E0.01297
G1 X45.364 Y87.875 E0.13409
G1 X44.764 Y87.875 E0.01827
G1 X47.852 Y90.963 E0.13291
G1 X47.252 Y90.963 E0.01827
G1 X44.164 Y87.875 E0.13291
G1 X43.564 Y87.875 E0.01827
G1 X46.651 Y90.963 E0.13291
M73 P40 R40
G1 X46.051 Y90.963 E0.01827
G1 X42.964 Y87.875 E0.13291
G1 X42.363 Y87.875 E0.01827
G1 X45.451 Y90.963 E0.13291
G1 X44.851 Y90.963 E0.01827
G1 X41.763 Y87.875 E0.13291
G1 X41.163 Y87.875 E0.01827
G1 X44.251 Y90.963 E0.13291
G1 X43.650 Y90.963 E0.01827
G1 X40.563 Y87.875 E0.13291
G1 X40.167 Y87.875 E0.01205
G1 X40.167 Y88.080 E0.00622
G1 X43.050 Y90.963 E0.12412
```

```
G1 X42.450 Y90.963 E0.01827
G1 X40.167 Y88.680 E0.09828
G1 X40.167 Y89.280 E0.01827
G1 X41.850 Y90.963 E0.07244
G1 X41.249 Y90.963 E0.01827
G1 X40.167 Y89.880 E0.04660
G1 X40.167 Y90.481 E0.01827
G1 X40.837 Y91.151 E0.02886
M204 S1250
G1 E-4.00000 F2400.000
G1 X54.369 Y95.079 F9000.000
G1 E4.00000 F900.000
M204 S1000
G1 F1272.328
G1 X55.441 Y96.151 E0.04614
M204 S1250
; stop printing object Petg print.STL id:10 copy 0
; printing object tpu print.STL id:11 copy 0
; stop printing object tpu print.STL id:11 copy 0
; printing object tpu print.STL id:13 copy 0
; stop printing object tpu print.STL id:13 copy 0
; printing object tpu print.STL id:17 copy 0
; stop printing object tpu print.STL id:17 copy 0
; printing object Petg print.STL id:16 copy 0
G1 E-4.00000 F2400.000
G1 X54.688 Y122.031 F9000.000
G1 E4.00000 F900.000
M204 S800
;TYPE:Perimeter
```

```
;WIDTH:0.45
G1 F1292.454
G1 X61.560 Y122.031 E0.20589
G1 X61.560 Y135.746 E0.41094
G1 X54.688 Y135.746 E0.20589
G1 X54.597 Y135.422 E0.01007
G1 X54.262 Y134.628 E0.02583
G1 X53.832 Y133.881 E0.02583
G1 X53.313 Y133.193 E0.02583
G1 X52.713 Y132.573 E0.02583
G1 X52.042 Y132.033 E0.02583
G1 X51.309 Y131.578 E0.02583
G1 X50.526 Y131.218 E0.02583
G1 X49.705 Y130.956 E0.02583
G1 X48.858 Y130.798 E0.02583
G1 X48.017 Y130.746 E0.02523
G1 X39.845 Y130.746 E0.24487
G1 X39.845 Y127.031 E0.11130
G1 X48.017 Y127.031 E0.24487
G1 X48.858 Y126.980 E0.02523
G1 X49.705 Y126.821 E0.02583
G1 X50.526 Y126.559 E0.02583
G1 X51.309 Y126.199 E0.02583
G1 X52.042 Y125.745 E0.02583
G1 X52.713 Y125.204 E0.02583
G1 X53.313 Y124.584 E0.02583
G1 X53.832 Y123.896 E0.02583
G1 X54.262 Y123.149 E0.02583
G1 X54.597 Y122.355 E0.02583
```

```
G1 X54.672 Y122.089 E0.00828
M204 S1250
G1 X54.373 Y121.614 F9000.000
M204 S800
;TYPE:External perimeter
G1 F1292.454
G1 X61.977 Y121.614 E0.22787
G1 X61.977 Y136.164 E0.43598
G1 X54.373 Y136.164 E0.22787
G1 X54.202 Y135.561 E0.01877
G1 X53.887 Y134.814 E0.02428
G1 X53.482 Y134.112 E0.02428
G1 X52.995 Y133.465 E0.02428
G1 X52.431 Y132.883 E0.02428
G1 X51.800 Y132.374 E0.02428
G1 X51.111 Y131.947 E0.02428
G1 X50.375 Y131.608 E0.02428
G1 X49.603 Y131.362 E0.02428
G1 X48.806 Y131.213 E0.02428
G1 X48.004 Y131.164 E0.02407
G1 X39.427 Y131.164 E0.25701
G1 X39.427 Y126.614 E0.13634
G1 X48.004 Y126.614 E0.25701
G1 X48.806 Y126.564 E0.02407
G1 X49.603 Y126.415 E0.02428
G1 X50.375 Y126.169 E0.02428
G1 X51.111 Y125.830 E0.02428
G1 X51.800 Y125.403 E0.02428
G1 X52.431 Y124.895 E0.02428
```

```
G1 X52.995 Y124.312 E0.02428
G1 X53.482 Y123.665 E0.02428
G1 X53.887 Y122.963 E0.02428
G1 X54.202 Y122.216 E0.02428
G1 X54.356 Y121.671 E0.01697
M204 S1250
G1 X54.694 Y121.852 F9000.000
G1 E-4.00000 F2400.000
G1 X61.434 Y123.015 F9000.000
G1 E4.00000 F900.000
M204 S1000
;TYPE:Solid infill
;WIDTH:0.456609
G1 F1272.328
G1 X60.764 Y122.345 E0.02887
G1 X60.163 Y122.345 E0.01827
G1 X61.246 Y123.428 E0.04661
G1 X61.246 Y124.028 E0.01827
G1 X59.563 Y122.345 E0.07245
G1 X58.963 Y122.345 E0.01827
G1 X61.246 Y124.628 E0.09828
G1 X61.246 Y125.228 E0.01827
G1 X58.363 Y122.345 E0.12412
G1 X57.763 Y122.345 E0.01827
G1 X61.246 Y125.828 E0.14996
G1 X61.246 Y126.429 E0.01827
G1 X57.162 Y122.345 E0.17580
G1 X56.562 Y122.345 E0.01827
G1 X61.246 Y127.029 E0.20163
```

G1 X61.246 Y127.629 E0.01827

G1 X55.962 Y122.345 E0.22747

G1 X55.362 Y122.345 E0.01827

G1 X61.246 Y128.229 E0.25331

G1 X61.246 Y128.830 E0.01827

G1 X54.888 Y122.471 E0.27370

G1 X54.710 Y122.893 E0.01395

G1 X61.246 Y129.430 E0.28137

G1 X61.246 Y130.030 E0.01827

G1 X54.529 Y123.313 E0.28914

G1 X54.310 Y123.694 E0.01338

M73 P41 R40

G1 X61.246 Y130.630 E0.29859

G1 X61.246 Y131.230 E0.01827

G1 X54.090 Y124.074 E0.30805

G1 X53.832 Y124.416 E0.01305

G1 X61.246 Y131.831 E0.31916

G1 X61.246 Y132.431 E0.01827

G1 X53.574 Y124.759 E0.33026

G1 X53.282 Y125.067 E0.01292

G1 X61.246 Y133.031 E0.34283

G1 X61.246 Y133.631 E0.01827

G1 X52.987 Y125.372 E0.35554

G1 X52.662 Y125.647 E0.01296

G1 X61.246 Y134.232 E0.36951

G1 X61.246 Y134.832 E0.01827

G1 X52.330 Y125.915 E0.38382

G1 X51.971 Y126.157 E0.01316

G1 X61.246 Y135.432 E0.39926

```
G1 X60.646 Y135.432 E0.01825
G1 X51.601 Y126.387 E0.38938
G1 X51.458 Y126.475 E0.00511
G1 X51.205 Y126.592 E0.00847
G1 X60.046 Y135.432 E0.38056
G1 X59.446 Y135.432 E0.01827
G1 X50.795 Y126.781 E0.37241
G1 X50.640 Y126.852 E0.00518
G1 X50.356 Y126.943 E0.00907
G1 X58.846 Y135.432 E0.36545
G1 X58.246 Y135.432 E0.01827
G1 X49.901 Y127.088 E0.35921
G1 X49.408 Y127.195 E0.01534
G1 X57.645 Y135.432 E0.35457
G1 X57.045 Y135.432 E0.01827
G1 X48.903 Y127.290 E0.35050
G1 X48.338 Y127.326 E0.01722
G1 X56.445 Y135.432 E0.34897
G1 X55.845 Y135.432 E0.01827
G1 X47.757 Y127.345 E0.34814
G1 X47.157 Y127.345 E0.01827
G1 X50.820 Y131.008 E0.15769
G1 X50.640 Y130.925 E0.00603
G1 X49.902 Y130.690 E0.02358
G1 X46.557 Y127.345 E0.14400
G1 X45.956 Y127.345 E0.01827
G1 X49.144 Y130.532 E0.13722
G1 X48.896 Y130.486 E0.00767
G1 X48.471 Y130.460 E0.01297
```

```
G1 X45.356 Y127.345 E0.13409
G1 X44.756 Y127.345 E0.01827
G1 X47.844 Y130.432 E0.13291
G1 X47.243 Y130.432 E0.01827
G1 X44.156 Y127.345 E0.13291
G1 X43.556 Y127.345 E0.01827
G1 X46.643 Y130.432 E0.13291
G1 X46.043 Y130.432 E0.01827
G1 X42.955 Y127.345 E0.13291
G1 X42.355 Y127.345 E0.01827
G1 X45.443 Y130.432 E0.13291
G1 X44.843 Y130.432 E0.01827
G1 X41.755 Y127.345 E0.13291
G1 X41.155 Y127.345 E0.01827
G1 X44.242 Y130.432 E0.13291
G1 X43.642 Y130.432 E0.01827
G1 X40.554 Y127.345 E0.13291
G1 X40.159 Y127.345 E0.01205
G1 X40.159 Y127.549 E0.00622
G1 X43.042 Y130.432 E0.12412
G1 X42.442 Y130.432 E0.01827
G1 X40.159 Y128.149 E0.09828
G1 X40.159 Y128.750 E0.01827
G1 X41.841 Y130.432 E0.07244
G1 X41.241 Y130.432 E0.01827
G1 X40.159 Y129.350 E0.04660
G1 X40.159 Y129.950 E0.01827
G1 X40.829 Y130.620 E0.02886
M204 S1250
```

```
G1 E-4.00000 F2400.000
G1 X54.361 Y134.549 F9000.000
G1 E4.00000 F900.000
M204 S1000
G1 F1272.328
G1 X55.433 Y135.620 E0.04614
M204 S1250
; stop printing object Petg print.STL id:16 copy 0
; printing object Petg print.STL id:12 copy 0
G1 E-4.00000 F2400.000
G1 X54.704 Y117.092 F9000.000
G1 E4.00000 F900.000
M204 S800
;TYPE:Perimeter
;WIDTH:0.45
G1 F1292.454
G1 X54.612 Y116.768 E0.01007
G1 X54.277 Y115.974 E0.02583
G1 X53.847 Y115.227 E0.02583
G1 X53.328 Y114.539 E0.02583
G1 X52.728 Y113.920 E0.02583
G1 X52.057 Y113.379 E0.02583
G1 X51.325 Y112.924 E0.02583
G1 X50.542 Y112.564 E0.02583
G1 X49.720 Y112.302 E0.02583
G1 X48.873 Y112.144 E0.02583
G1 X48.032 Y112.092 E0.02523
G1 X39.860 Y112.092 E0.24487
G1 X39.860 Y108.377 E0.11130
```

```
G1 X48.032 Y108.377 E0.24487
G1 X48.873 Y108.326 E0.02523
G1 X49.720 Y108.167 E0.02583
G1 X50.542 Y107.905 E0.02583
G1 X51.325 Y107.545 E0.02583
G1 X52.057 Y107.091 E0.02583
G1 X52.728 Y106.550 E0.02583
G1 X53.328 Y105.930 E0.02583
G1 X53.847 Y105.242 E0.02583
G1 X54.277 Y104.495 E0.02583
G1 X54.612 Y103.701 E0.02583
G1 X54.704 Y103.377 E0.01007
G1 X61.575 Y103.377 E0.20589
G1 X61.575 Y117.092 E0.41094
G1 X54.764 Y117.092 E0.20409
M204 S1250
G1 X54.388 Y117.510 F9000.000
M204 S800
;TYPE:External perimeter
G1 F1292.454
G1 X54.217 Y116.907 E0.01877
G1 X53.902 Y116.160 E0.02428
G1 X53.498 Y115.458 E0.02428
G1 X53.010 Y114.811 E0.02428
G1 X52.446 Y114.229 E0.02428
G1 X51.815 Y113.720 E0.02428
G1 X51.126 Y113.293 E0.02428
G1 X50.390 Y112.954 E0.02428
G1 X49.618 Y112.708 E0.02428
```

```
G1 X48.821 Y112.559 E0.02428
G1 X48.020 Y112.510 E0.02407
G1 X39.442 Y112.510 E0.25701
G1 X39.442 Y107.960 E0.13634
G1 X48.020 Y107.960 E0.25701
G1 X48.821 Y107.910 E0.02407
G1 X49.618 Y107.761 E0.02428
G1 X50.390 Y107.515 E0.02428
G1 X51.126 Y107.176 E0.02428
G1 X51.815 Y106.749 E0.02428
G1 X52.446 Y106.241 E0.02428
G1 X53.010 Y105.658 E0.02428
G1 X53.498 Y105.011 E0.02428
G1 X53.902 Y104.309 E0.02428
G1 X54.217 Y103.562 E0.02428
G1 X54.388 Y102.960 E0.01877
G1 X61.992 Y102.960 E0.22787
G1 X61.992 Y117.510 E0.43598
G1 X54.448 Y117.510 E0.22608
M204 S1250
G1 X54.378 Y117.110 F9000.000
G1 X55.448 Y116.967
M204 S1000
;TYPE:Solid infill
;WIDTH:0.456609
G1 F1272.328
G1 X54.376 Y115.895 E0.04614
M204 S1250
G1 E-4.00000 F2400.000
```

```
G1 X61.449 Y104.361 F9000.000
G1 E4.00000 F900.000
M204 S1000
G1 F1272.328
G1 X60.779 Y103.691 E0.02887
G1 X60.179 Y103.691 E0.01827
G1 X61.261 Y104.774 E0.04661
G1 X61.261 Y105.374 E0.01827
G1 X59.578 Y103.691 E0.07245
G1 X58.978 Y103.691 E0.01827
G1 X61.261 Y105.974 E0.09828
G1 X61.261 Y106.574 E0.01827
G1 X58.378 Y103.691 E0.12412
G1 X57.778 Y103.691 E0.01827
G1 X61.261 Y107.175 E0.14996
G1 X61.261 Y107.775 E0.01827
G1 X57.177 Y103.691 E0.17580
G1 X56.577 Y103.691 E0.01827
G1 X61.261 Y108.375 E0.20163
G1 X61.261 Y108.975 E0.01827
G1 X55.977 Y103.691 E0.22747
G1 X55.377 Y103.691 E0.01827
G1 X61.261 Y109.575 E0.25331
G1 X61.261 Y110.176 E0.01827
G1 X54.903 Y103.817 E0.27370
G1 X54.725 Y104.239 E0.01395
G1 X61.261 Y110.776 E0.28137
G1 X61.261 Y111.376 E0.01827
G1 X54.544 Y104.659 E0.28914
```

```
G1 X54.325 Y105.040 E0.01338
G1 X61.261 Y111.976 E0.29859
G1 X61.261 Y112.576 E0.01827
G1 X54.105 Y105.420 E0.30805
G1 X53.847 Y105.762 E0.01305
G1 X61.261 Y113.177 E0.31916
G1 X61.261 Y113.777 E0.01827
G1 X53.589 Y106.105 E0.33026
G1 X53.297 Y106.413 E0.01292
G1 X61.261 Y114.377 E0.34283
G1 X61.261 Y114.977 E0.01827
G1 X53.002 Y106.718 E0.35554
G1 X52.677 Y106.993 E0.01296
G1 X61.261 Y115.578 E0.36951
G1 X61.261 Y116.178 E0.01827
G1 X52.345 Y107.261 E0.38382
G1 X51.986 Y107.503 E0.01316
G1 X61.261 Y116.778 E0.39926
G1 X60.662 Y116.778 E0.01825
G1 X51.616 Y107.733 E0.38938
G1 X51.473 Y107.821 E0.00511
G1 X51.221 Y107.938 E0.00847
G1 X60.061 Y116.778 E0.38056
G1 X59.461 Y116.778 E0.01827
G1 X50.810 Y108.127 E0.37241
G1 X50.655 Y108.198 E0.00518
G1 X50.371 Y108.289 E0.00907
G1 X58.861 Y116.778 E0.36545
G1 X58.261 Y116.778 E0.01827
```

```
G1 X49.916 Y108.434 E0.35921
G1 X49.423 Y108.541 E0.01534
G1 X57.660 Y116.778 E0.35457
G1 X57.060 Y116.778 E0.01827
G1 X48.918 Y108.636 E0.35050
G1 X48.353 Y108.672 E0.01722
G1 X56.460 Y116.778 E0.34897
G1 X55.860 Y116.778 E0.01827
G1 X47.772 Y108.691 E0.34814
G1 X47.172 Y108.691 E0.01827
G1 X50.835 Y112.354 E0.15769
G1 X50.655 Y112.271 E0.00603
G1 X49.917 Y112.036 E0.02358
G1 X46.572 Y108.691 E0.14400
G1 X45.971 Y108.691 E0.01827
G1 X49.159 Y111.879 E0.13722
G1 X48.912 Y111.832 E0.00767
G1 X48.486 Y111.806 E0.01297
G1 X45.371 Y108.691 E0.13409
G1 X44.771 Y108.691 E0.01827
G1 X47.859 Y111.778 E0.13291
G1 X47.259 Y111.778 E0.01827
G1 X44.171 Y108.691 E0.13291
G1 X43.571 Y108.691 E0.01827
G1 X46.658 Y111.778 E0.13291
G1 X46.058 Y111.778 E0.01827
G1 X42.970 Y108.691 E0.13291
G1 X42.370 Y108.691 E0.01827
G1 X45.458 Y111.778 E0.13291
```

```
G1 X44.858 Y111.778 E0.01827
G1 X41.770 Y108.691 E0.13291
G1 X41.170 Y108.691 E0.01827
G1 X44.257 Y111.778 E0.13291
G1 X43.657 Y111.778 E0.01827
G1 X40.570 Y108.691 E0.13291
G1 X40.174 Y108.691 E0.01205
G1 X40.174 Y108.895 E0.00622
G1 X43.057 Y111.778 E0.12412
G1 X42.457 Y111.778 E0.01827
G1 X40.174 Y109.495 E0.09828
G1 X40.174 Y110.096 E0.01827
G1 X41.857 Y111.778 E0.07244
G1 X41.256 Y111.778 E0.01827
G1 X40.174 Y110.696 E0.04660
M73 P42 R40
G1 X40.174 Y111.296 E0.01827
G1 X40.844 Y111.967 E0.02886
M204 S1250
; stop printing object Petg print.STL id:12 copy 0
; printing object tpu print.STL id:7 copy 0
; stop printing object tpu print.STL id:7 copy 0
; printing object tpu print.STL id:3 copy 0
; stop printing object tpu print.STL id:3 copy 0
; printing object Petg print.STL id:2 copy 0
G1 E-4.00000 F2400.000
G1 X89.650 Y112.673 F9000.000
G1 E4.00000 F900.000
M204 S800
```

```
;TYPE:Perimeter
;WIDTH:0.45
G1 F1292.454
G1 X89.650 Y108.958 E0.11130
G1 X97.822 Y108.958 E0.24487
G1 X98.662 Y108.906 E0.02523
G1 X99.510 Y108.748 E0.02583
G1 X100.331 Y108.486 E0.02583
G1 X101.114 Y108.126 E0.02583
G1 X101.847 Y107.672 E0.02583
G1 X102.518 Y107.131 E0.02583
G1 X103.117 Y106.511 E0.02583
G1 X103.636 Y105.823 E0.02583
G1 X104.066 Y105.076 E0.02583
G1 X104.401 Y104.282 E0.02583
G1 X104.493 Y103.958 E0.01007
G1 X111.364 Y103.958 E0.20589
G1 X111.364 Y117.673 E0.41094
G1 X104.493 Y117.673 E0.20589
G1 X104.401 Y117.349 E0.01007
G1 X104.066 Y116.555 E0.02583
G1 X103.636 Y115.808 E0.02583
G1 X103.117 Y115.120 E0.02583
G1 X102.518 Y114.500 E0.02583
G1 X101.847 Y113.960 E0.02583
G1 X101.114 Y113.505 E0.02583
G1 X100.331 Y113.145 E0.02583
G1 X99.510 Y112.883 E0.02583
G1 X98.662 Y112.725 E0.02583
```

```
G1 X97.822 Y112.673 E0.02523
G1 X89.710 Y112.673 E0.24307
M204 S1250
G1 X89.232 Y113.091 F9000.000
M204 S800
;TYPE:External perimeter
G1 F1292.454
G1 X89.232 Y108.541 E0.13634
G1 X97.809 Y108.541 E0.25701
G1 X98.611 Y108.491 E0.02407
G1 X99.407 Y108.342 E0.02428
G1 X100.180 Y108.096 E0.02428
M73 P42 R39
G1 X100.916 Y107.757 E0.02428
G1 X101.604 Y107.330 E0.02428
G1 X102.235 Y106.822 E0.02428
G1 X102.799 Y106.239 E0.02428
G1 X103.287 Y105.592 E0.02428
G1 X103.691 Y104.890 E0.02428
G1 X104.006 Y104.143 E0.02428
G1 X104.177 Y103.541 E0.01877
G1 X111.782 Y103.541 E0.22787
G1 X111.782 Y118.091 E0.43598
G1 X104.177 Y118.091 E0.22787
G1 X104.006 Y117.488 E0.01877
G1 X103.691 Y116.741 E0.02428
G1 X103.287 Y116.039 E0.02428
G1 X102.799 Y115.392 E0.02428
G1 X102.235 Y114.810 E0.02428
```

```
G1 X101.604 Y114.301 E0.02428
G1 X100.916 Y113.874 E0.02428
G1 X100.180 Y113.535 E0.02428
G1 X99.407 Y113.289 E0.02428
G1 X98.611 Y113.140 E0.02428
G1 X97.809 Y113.091 E0.02407
G1 X89.292 Y113.091 E0.25521
M204 S1250
G1 X89.432 Y112.744 F9000.000
G1 X90.633 Y112.547
M204 S1000
;TYPE:Solid infill
;WIDTH:0.456609
G1 F1272.328
G1 X89.963 Y111.877 E0.02886
G1 X89.963 Y111.277 E0.01827
G1 X91.046 Y112.359 E0.04660
G1 X91.646 Y112.359 E0.01827
G1 X89.963 Y110.676 E0.07244
G1 X89.963 Y110.076 E0.01827
G1 X92.246 Y112.359 E0.09828
G1 X92.846 Y112.359 E0.01827
G1 X89.963 Y109.476 E0.12412
G1 X89.963 Y109.272 E0.00622
G1 X90.359 Y109.272 E0.01205
G1 X93.447 Y112.359 E0.13291
G1 X94.047 Y112.359 E0.01827
G1 X90.959 Y109.272 E0.13291
G1 X91.559 Y109.272 E0.01827
```

```
G1 X94.647 Y112.359 E0.13291
G1 X95.247 Y112.359 E0.01827
G1 X92.160 Y109.272 E0.13291
G1 X92.760 Y109.272 E0.01827
G1 X95.847 Y112.359 E0.13291
G1 X96.448 Y112.359 E0.01827
G1 X93.360 Y109.272 E0.13291
G1 X93.960 Y109.272 E0.01827
G1 X97.048 Y112.359 E0.13291
G1 X97.648 Y112.359 E0.01827
G1 X94.560 Y109.272 E0.13291
G1 X95.161 Y109.272 E0.01827
G1 X98.276 Y112.387 E0.13409
G1 X98.701 Y112.413 E0.01297
G1 X98.949 Y112.459 E0.00767
G1 X95.761 Y109.272 E0.13722
G1 X96.361 Y109.272 E0.01827
G1 X99.706 Y112.617 E0.14400
G1 X100.444 Y112.852 E0.02358
G1 X100.625 Y112.935 E0.00603
G1 X96.961 Y109.272 E0.15769
G1 X97.562 Y109.272 E0.01827
G1 X105.649 Y117.359 E0.34814
G1 X106.249 Y117.359 E0.01827
G1 X98.143 Y109.252 E0.34897
G1 X98.707 Y109.217 E0.01722
G1 X106.850 Y117.359 E0.35050
G1 X107.450 Y117.359 E0.01827
G1 X99.213 Y109.122 E0.35457
```

```
G1 X99.705 Y109.014 E0.01534
G1 X108.050 Y117.359 E0.35921
G1 X108.650 Y117.359 E0.01827
G1 X100.160 Y108.870 E0.36545
G1 X100.444 Y108.779 E0.00907
G1 X100.599 Y108.708 E0.00518
G1 X109.250 Y117.359 E0.37241
G1 X109.851 Y117.359 E0.01827
G1 X101.010 Y108.519 E0.38056
G1 X101.263 Y108.402 E0.00847
G1 X101.405 Y108.314 E0.00511
G1 X110.451 Y117.359 E0.38938
G1 X111.051 Y117.359 E0.01825
G1 X101.776 Y108.084 E0.39926
G1 X102.134 Y107.842 E0.01316
G1 X111.051 Y116.759 E0.38382
G1 X111.051 Y116.158 E0.01827
G1 X102.467 Y107.574 E0.36951
G1 X102.791 Y107.299 E0.01296
G1 X111.051 Y115.558 E0.35554
G1 X111.051 Y114.958 E0.01827
G1 X103.086 Y106.994 E0.34283
G1 X103.378 Y106.686 E0.01292
G1 X111.051 Y114.358 E0.33026
G1 X111.051 Y113.758 E0.01827
G1 X103.636 Y106.343 E0.31916
G1 X103.894 Y106.001 E0.01305
G1 X111.051 Y113.157 E0.30805
G1 X111.051 Y112.557 E0.01827
```

```
G1 X104.114 Y105.621 E0.29859
G1 X104.334 Y105.240 E0.01338
G1 X111.051 Y111.957 E0.28914
G1 X111.051 Y111.357 E0.01827
G1 X104.514 Y104.820 E0.28137
G1 X104.692 Y104.398 E0.01395
G1 X111.051 Y110.756 E0.27370
G1 X111.051 Y110.156 E0.01827
G1 X105.166 Y104.272 E0.25331
G1 X105.766 Y104.272 E0.01827
G1 X111.051 Y109.556 E0.22747
G1 X111.051 Y108.956 E0.01827
G1 X106.367 Y104.272 E0.20163
G1 X106.967 Y104.272 E0.01827
G1 X111.051 Y108.356 E0.17580
G1 X111.051 Y107.755 E0.01827
G1 X107.567 Y104.272 E0.14996
G1 X108.167 Y104.272 E0.01827
G1 X111.051 Y107.155 E0.12412
G1 X111.051 Y106.555 E0.01827
G1 X108.767 Y104.272 E0.09828
G1 X109.368 Y104.272 E0.01827
G1 X111.051 Y105.955 E0.07245
G1 X111.051 Y105.355 E0.01827
G1 X109.968 Y104.272 E0.04661
G1 X110.568 Y104.272 E0.01827
G1 X111.239 Y104.942 E0.02887
M204 S1250
G1 E-4.00000 F2400.000
```

G1 X104.165 Y116.476 F9000.000

G1 E4.00000 F900.000

M204 S1000

G1 F1272.328

G1 X105.237 Y117.547 E0.04614

M204 S1250

; stop printing object Petg print.STL id:2 copy 0

; printing object Petg print.STL id:6 copy 0

G1 X104.478 Y122.612 F9000.000

M204 S800

;TYPE:Perimeter

;WIDTH:0.45

G1 F1292.454

G1 X111.349 Y122.612 E0.20589

G1 X111.349 Y136.327 E0.41094

G1 X104.478 Y136.327 E0.20589

G1 X104.386 Y136.003 E0.01007

G1 X104.051 Y135.209 E0.02583

G1 X103.621 Y134.462 E0.02583

G1 X103.102 Y133.774 E0.02583

G1 X102.503 Y133.154 E0.02583

G1 X101.831 Y132.613 E0.02583

G1 X101.099 Y132.159 E0.02583

G1 X100.316 Y131.799 E0.02583

G1 X99.495 Y131.537 E0.02583

G1 X98.647 Y131.379 E0.02583

G1 X97.807 Y131.327 E0.02523

G1 X89.635 Y131.327 E0.24487

G1 X89.635 Y127.612 E0.11130

```
G1 X97.807 Y127.612 E0.24487
G1 X98.647 Y127.560 E0.02523
G1 X99.495 Y127.402 E0.02583
G1 X100.316 Y127.140 E0.02583
G1 X101.099 Y126.780 E0.02583
G1 X101.831 Y126.325 E0.02583
G1 X102.503 Y125.785 E0.02583
G1 X103.102 Y125.165 E0.02583
G1 X103.621 Y124.477 E0.02583
G1 X104.051 Y123.730 E0.02583
G1 X104.386 Y122.936 E0.02583
G1 X104.461 Y122.670 E0.00828
M204 S1250
G1 X104.162 Y122.194 F9000.000
M204 S800
;TYPE:External perimeter
G1 F1292.454
G1 X111.767 Y122.194 E0.22787
G1 X111.767 Y136.744 E0.43598
G1 X104.162 Y136.744 E0.22787
G1 X103.991 Y136.142 E0.01877
G1 X103.676 Y135.395 E0.02428
G1 X103.272 Y134.693 E0.02428
G1 X102.784 Y134.046 E0.02428
G1 X102.220 Y133.463 E0.02428
G1 X101.589 Y132.955 E0.02428
G1 X100.901 Y132.528 E0.02428
G1 X100.164 Y132.189 E0.02428
G1 X99.392 Y131.943 E0.02428
```

```
G1 X98.596 Y131.794 E0.02428
G1 X97.794 Y131.744 E0.02407
G1 X89.217 Y131.744 E0.25701
G1 X89.217 Y127.194 E0.13634
G1 X97.794 Y127.194 E0.25701
G1 X98.596 Y127.145 E0.02407
G1 X99.392 Y126.996 E0.02428
G1 X100.164 Y126.750 E0.02428
G1 X100.901 Y126.411 E0.02428
G1 X101.589 Y125.984 E0.02428
G1 X102.220 Y125.475 E0.02428
G1 X102.784 Y124.893 E0.02428
G1 X103.272 Y124.246 E0.02428
G1 X103.676 Y123.544 E0.02428
G1 X103.991 Y122.797 E0.02428
G1 X104.146 Y122.252 E0.01697
M204 S1250
G1 X104.483 Y122.433 F9000.000
G1 E-4.00000 F2400.000
G1 X111.224 Y123.596 F9000.000
G1 E4.00000 F900.000
M204 S1000
;TYPE:Solid infill
;WIDTH:0.456609
G1 F1272.328
G1 X110.553 Y122.926 E0.02887
G1 X109.953 Y122.926 E0.01827
G1 X111.036 Y124.008 E0.04661
G1 X111.036 Y124.609 E0.01827
```

```
G1 X109.353 Y122.926 E0.07245
G1 X108.752 Y122.926 E0.01827
G1 X111.036 Y125.209 E0.09828
G1 X111.036 Y125.809 E0.01827
G1 X108.152 Y122.926 E0.12412
G1 X107.552 Y122.926 E0.01827
G1 X111.036 Y126.409 E0.14996
G1 X111.036 Y127.010 E0.01827
G1 X106.952 Y122.926 E0.17580
G1 X106.351 Y122.926 E0.01827
G1 X111.036 Y127.610 E0.20163
G1 X111.036 Y128.210 E0.01827
G1 X105.751 Y122.926 E0.22747
G1 X105.151 Y122.926 E0.01827
G1 X111.036 Y128.810 E0.25331
G1 X111.036 Y129.410 E0.01827
G1 X104.677 Y123.052 E0.27370
G1 X104.499 Y123.474 E0.01395
G1 X111.036 Y130.011 E0.28137
G1 X111.036 Y130.611 E0.01827
G1 X104.318 Y123.894 E0.28914
G1 X104.099 Y124.275 E0.01338
G1 X111.036 Y131.211 E0.29859
G1 X111.036 Y131.811 E0.01827
G1 X103.879 Y124.655 E0.30805
G1 X103.621 Y124.997 E0.01305
G1 X111.036 Y132.412 E0.31916
G1 X111.036 Y133.012 E0.01827
G1 X103.363 Y125.339 E0.33026
```

```
G1 X103.071 Y125.648 E0.01292
G1 X111.036 Y133.612 E0.34283
G1 X111.036 Y134.212 E0.01827
G1 X102.776 Y125.953 E0.35554
G1 X102.451 Y126.228 E0.01296
G1 X111.036 Y134.812 E0.36951
M73 P43 R39
G1 X111.036 Y135.413 E0.01827
G1 X102.119 Y126.496 E0.38382
G1 X101.761 Y126.738 E0.01316
G1 X111.036 Y136.013 E0.39926
G1 X110.436 Y136.013 E0.01825
G1 X101.390 Y126.968 E0.38938
G1 X101.247 Y127.056 E0.00511
G1 X100.995 Y127.173 E0.00847
G1 X109.836 Y136.013 E0.38056
G1 X109.235 Y136.013 E0.01827
G1 X100.584 Y127.362 E0.37241
G1 X100.429 Y127.433 E0.00518
G1 X100.145 Y127.523 E0.00907
G1 X108.635 Y136.013 E0.36545
G1 X108.035 Y136.013 E0.01827
G1 X99.690 Y127.668 E0.35921
G1 X99.198 Y127.776 E0.01534
G1 X107.435 Y136.013 E0.35457
G1 X106.835 Y136.013 E0.01827
G1 X98.692 Y127.871 E0.35050
G1 X98.127 Y127.906 E0.01722
G1 X106.234 Y136.013 E0.34897
```

G1 X105.634 Y136.013 E0.01827

G1 X97.546 Y127.926 E0.34814

G1 X96.946 Y127.926 E0.01827

G1 X100.609 Y131.589 E0.15769

G1 X100.429 Y131.506 E0.00603

G1 X99.691 Y131.271 E0.02358

G1 X96.346 Y127.926 E0.14400

G1 X95.746 Y127.926 E0.01827

G1 X98.933 Y131.113 E0.13722

G1 X98.686 Y131.067 E0.00767

G1 X98.261 Y131.041 E0.01297

G1 X95.146 Y127.926 E0.13409

G1 X94.545 Y127.926 E0.01827

G1 X97.633 Y131.013 E0.13291

G1 X97.033 Y131.013 E0.01827

G1 X93.945 Y127.926 E0.13291

G1 X93.345 Y127.926 E0.01827

G1 X96.433 Y131.013 E0.13291

G1 X95.832 Y131.013 E0.01827

G1 X92.745 Y127.926 E0.13291

G1 X92.144 Y127.926 E0.01827

G1 X95.232 Y131.013 E0.13291

G1 X94.632 Y131.013 E0.01827

G1 X91.544 Y127.926 E0.13291

G1 X90.944 Y127.926 E0.01827

G1 X94.032 Y131.013 E0.13291

G1 X93.431 Y131.013 E0.01827

G1 X90.344 Y127.926 E0.13291

G1 X89.948 Y127.926 E0.01205

```
G1 X89.948 Y128.130 E0.00622
G1 X92.831 Y131.013 E0.12412
G1 X92.231 Y131.013 E0.01827
G1 X89.948 Y128.730 E0.09828
G1 X89.948 Y129.330 E0.01827
G1 X91.631 Y131.013 E0.07244
G1 X91.031 Y131.013 E0.01827
G1 X89.948 Y129.931 E0.04660
G1 X89.948 Y130.531 E0.01827
G1 X90.618 Y131.201 E0.02886
M204 S1250
G1 E-4.00000 F2400.000
G1 X104.150 Y135.130 F9000.000
G1 E4.00000 F900.000
M204 S1000
G1 F1272.328
G1 X105.222 Y136.201 E0.04614
M204 S1250
; stop printing object Petg print.STL id:6 copy 0
; printing object tpu print.STL id:27 copy 0
; stop printing object tpu print.STL id:27 copy 0
; printing object tpu print.STL id:23 copy 0
; stop printing object tpu print.STL id:23 copy 0
; printing object tpu print.STL id:21 copy 0
; stop printing object tpu print.STL id:21 copy 0
; printing object Petg print.STL id:20 copy 0
G1 E-4.00000 F2400.000
G1 X141.293 Y92.032 F9000.000
G1 E4.00000 F900.000
```

```
M204 S800
;TYPE:Perimeter
;WIDTH:0.45
G1 F1292.454
G1 X141.293 Y88.318 E0.11130
G1 X149.465 Y88.318 E0.24487
G1 X150.306 Y88.266 E0.02523
G1 X151.153 Y88.107 E0.02583
G1 X151.974 Y87.846 E0.02583
G1 X152.757 Y87.485 E0.02583
G1 X153.490 Y87.031 E0.02583
G1 X154.161 Y86.490 E0.02583
G1 X154.761 Y85.871 E0.02583
G1 X155.279 Y85.183 E0.02583
G1 X155.710 Y84.436 E0.02583
G1 X156.045 Y83.641 E0.02583
G1 X156.136 Y83.318 E0.01007
G1 X163.007 Y83.318 E0.20589
G1 X163.007 Y97.032 E0.41094
G1 X156.136 Y97.032 E0.20589
G1 X156.045 Y96.709 E0.01007
G1 X155.710 Y95.915 E0.02583
G1 X155.279 Y95.168 E0.02583
G1 X154.761 Y94.479 E0.02583
G1 X154.161 Y93.860 E0.02583
G1 X153.490 Y93.319 E0.02583
G1 X152.757 Y92.865 E0.02583
G1 X151.974 Y92.504 E0.02583
G1 X151.153 Y92.243 E0.02583
```

```
G1 X150.306 Y92.084 E0.02583
G1 X149.465 Y92.032 E0.02523
G1 X141.353 Y92.032 E0.24307
M204 S1250
G1 X140.875 Y92.450 F9000.000
M204 S800
;TYPE:External perimeter
G1 F1292.454
G1 X140.875 Y87.900 E0.13634
G1 X149.452 Y87.900 E0.25701
G1 X150.254 Y87.851 E0.02407
G1 X151.051 Y87.702 E0.02428
G1 X151.823 Y87.456 E0.02428
G1 X152.559 Y87.117 E0.02428
G1 X153.248 Y86.690 E0.02428
G1 X153.879 Y86.181 E0.02428
G1 X154.443 Y85.599 E0.02428
G1 X154.930 Y84.952 E0.02428
G1 X155.335 Y84.249 E0.02428
G1 X155.650 Y83.503 E0.02428
G1 X155.820 Y82.900 E0.01877
G1 X163.425 Y82.900 E0.22787
G1 X163.425 Y97.450 E0.43598
G1 X155.820 Y97.450 E0.22787
G1 X155.650 Y96.847 E0.01877
G1 X155.335 Y96.101 E0.02428
G1 X154.930 Y95.398 E0.02428
G1 X154.443 Y94.751 E0.02428
G1 X153.879 Y94.169 E0.02428
```

```
G1 X153.248 Y93.661 E0.02428
G1 X152.559 Y93.234 E0.02428
G1 X151.823 Y92.895 E0.02428
G1 X151.051 Y92.649 E0.02428
G1 X150.254 Y92.500 E0.02428
G1 X149.452 Y92.450 E0.02407
G1 X140.935 Y92.450 E0.25521
M204 S1250
G1 X141.076 Y92.104 F9000.000
G1 X142.277 Y91.907
M204 S1000
;TYPE:Solid infill
;WIDTH:0.456609
G1 F1272.328
G1 X141.606 Y91.236 E0.02886
G1 X141.606 Y90.636 E0.01827
G1 X142.689 Y91.719 E0.04660
G1 X143.289 Y91.719 E0.01827
G1 X141.606 Y90.036 E0.07244
G1 X141.606 Y89.436 E0.01827
G1 X143.890 Y91.719 E0.09828
G1 X144.490 Y91.719 E0.01827
G1 X141.606 Y88.836 E0.12412
G1 X141.606 Y88.631 E0.00622
G1 X142.002 Y88.631 E0.01205
G1 X145.090 Y91.719 E0.13291
G1 X145.690 Y91.719 E0.01827
G1 X142.603 Y88.631 E0.13291
G1 X143.203 Y88.631 E0.01827
```

G1 X146.290 Y91.719 E0.13291

G1 X146.891 Y91.719 E0.01827

G1 X143.803 Y88.631 E0.13291

G1 X144.403 Y88.631 E0.01827

G1 X147.491 Y91.719 E0.13291

G1 X148.091 Y91.719 E0.01827

G1 X145.003 Y88.631 E0.13291

G1 X145.604 Y88.631 E0.01827

G1 X148.691 Y91.719 E0.13291

G1 X149.291 Y91.719 E0.01827

G1 X146.204 Y88.631 E0.13291

G1 X146.804 Y88.631 E0.01827

G1 X149.919 Y91.746 E0.13409

G1 X150.344 Y91.773 E0.01297

G1 X150.592 Y91.819 E0.00767

G1 X147.404 Y88.631 E0.13722

G1 X148.004 Y88.631 E0.01827

G1 X151.350 Y91.976 E0.14400

G1 X152.088 Y92.212 E0.02358

G1 X152.268 Y92.295 E0.00603

G1 X148.605 Y88.631 E0.15769

G1 X149.205 Y88.631 E0.01827

G1 X157.293 Y96.719 E0.34814

G1 X157.893 Y96.719 E0.01827

G1 X149.786 Y88.612 E0.34897

G1 X150.351 Y88.576 E0.01722

G1 X158.493 Y96.719 E0.35050

M73 P43 R38

G1 X159.093 Y96.719 E0.01827

```
G1 X150.856 Y88.482 E0.35457
G1 X151.349 Y88.374 E0.01534
G1 X159.693 Y96.719 E0.35921
G1 X160.294 Y96.719 E0.01827
G1 X151.804 Y88.229 E0.36545
G1 X152.088 Y88.139 E0.00907
G1 X152.242 Y88.067 E0.00518
G1 X160.894 Y96.719 E0.37241
G1 X161.494 Y96.719 E0.01827
G1 X152.653 Y87.878 E0.38056
G1 X152.906 Y87.762 E0.00847
G1 X153.049 Y87.673 E0.00511
G1 X162.094 Y96.719 E0.38938
G1 X162.694 Y96.719 E0.01825
G1 X153.419 Y87.444 E0.39926
G1 X153.778 Y87.202 E0.01316
G1 X162.694 Y96.118 E0.38382
G1 X162.694 Y95.518 E0.01827
G1 X154.110 Y86.934 E0.36951
G1 X154.435 Y86.658 E0.01296
G1 X162.694 Y94.918 E0.35554
G1 X162.694 Y94.318 E0.01827
G1 X154.730 Y86.353 E0.34283
G1 X155.022 Y86.045 E0.01292
G1 X162.694 Y93.717 E0.33026
G1 X162.694 Y93.117 E0.01827
G1 X155.280 Y85.703 E0.31916
G1 X155.538 Y85.361 E0.01305
G1 X162.694 Y92.517 E0.30805
```

```
G1 X162.694 Y91.917 E0.01827
G1 X155.758 Y84.980 E0.29859
G1 X155.977 Y84.599 E0.01338
G1 X162.694 Y91.316 E0.28914
G1 X162.694 Y90.716 E0.01827
G1 X156.158 Y84.180 E0.28137
G1 X156.336 Y83.758 E0.01395
G1 X162.694 Y90.116 E0.27370
G1 X162.694 Y89.516 E0.01827
G1 X156.810 Y83.631 E0.25331
G1 X157.410 Y83.631 E0.01827
G1 X162.694 Y88.916 E0.22747
G1 X162.694 Y88.315 E0.01827
G1 X158.010 Y83.631 E0.20163
G1 X158.610 Y83.631 E0.01827
G1 X162.694 Y87.715 E0.17580
G1 X162.694 Y87.115 E0.01827
G1 X159.210 Y83.631 E0.14996
G1 X159.811 Y83.631 E0.01827
G1 X162.694 Y86.515 E0.12412
G1 X162.694 Y85.914 E0.01827
G1 X160.411 Y83.631 E0.09828
G1 X161.011 Y83.631 E0.01827
G1 X162.694 Y85.314 E0.07245
G1 X162.694 Y84.714 E0.01827
G1 X161.611 Y83.631 E0.04661
G1 X162.211 Y83.631 E0.01827
G1 X162.882 Y84.302 E0.02887
M204 S1250
```

G1 E-4.00000 F2400.000

G1 X155.809 Y95.835 F9000.000

G1 E4.00000 F900.000

M204 S1000

G1 F1272.328

G1 X156.880 Y96.907 E0.04614

M204 S1250

; stop printing object Petg print.STL id:20 copy 0

; printing object Petg print.STL id:22 copy 0

G1 E-4.00000 F2400.000

G1 X156.143 Y104.133 F9000.000

G1 E4.00000 F900.000

M204 S800

;TYPE:Perimeter

;WIDTH:0.45

G1 F1292.454

G1 X163.014 Y104.133 E0.20589

G1 X163.014 Y117.848 E0.41094

G1 X156.143 Y117.848 E0.20589

G1 X156.052 Y117.524 E0.01007

G1 X155.717 Y116.730 E0.02583

G1 X155.286 Y115.983 E0.02583

G1 X154.768 Y115.295 E0.02583

G1 X154.168 Y114.675 E0.02583

G1 X153.497 Y114.135 E0.02583

G1 X152.764 Y113.680 E0.02583

G1 X151.981 Y113.320 E0.02583

G1 X151.160 Y113.058 E0.02583

G1 X150.313 Y112.900 E0.02583

```
G1 X149.472 Y112.848 E0.02523
G1 X141.300 Y112.848 E0.24487
G1 X141.300 Y109.133 E0.11130
G1 X149.472 Y109.133 E0.24487
M73 P44 R38
G1 X150.313 Y109.082 E0.02523
G1 X151.160 Y108.923 E0.02583
G1 X151.981 Y108.661 E0.02583
G1 X152.764 Y108.301 E0.02583
G1 X153.497 Y107.847 E0.02583
G1 X154.168 Y107.306 E0.02583
G1 X154.768 Y106.686 E0.02583
G1 X155.286 Y105.998 E0.02583
G1 X155.717 Y105.251 E0.02583
G1 X156.052 Y104.457 E0.02583
G1 X156.127 Y104.191 E0.00828
M204 S1250
G1 X155.827 Y103.716 F9000.000
M204 S800
;TYPE:External perimeter
G1 F1292.454
G1 X163.432 Y103.716 E0.22787
G1 X163.432 Y118.266 E0.43598
G1 X155.827 Y118.266 E0.22787
G1 X155.657 Y117.663 E0.01877
G1 X155.342 Y116.916 E0.02428
G1 X154.937 Y116.214 E0.02428
G1 X154.449 Y115.567 E0.02428
G1 X153.886 Y114.985 E0.02428
```

G1 X153.255 Y114.476 E0.02428

G1 X152.566 Y114.049 E0.02428

G1 X151.830 Y113.710 E0.02428

G1 X151.058 Y113.464 E0.02428

G1 X150.261 Y113.315 E0.02428

G1 X149.459 Y113.266 E0.02407

G1 X140.882 Y113.266 E0.25701

G1 X140.882 Y108.716 E0.13634

G1 X149.459 Y108.716 E0.25701

G1 X150.261 Y108.666 E0.02407

G1 X151.058 Y108.517 E0.02428

G1 X151.830 Y108.271 E0.02428

G1 X152.566 Y107.932 E0.02428

G1 X153.255 Y107.505 E0.02428

G1 X153.886 Y106.997 E0.02428

G1 X154.449 Y106.414 E0.02428

G1 X154.937 Y105.767 E0.02428

G1 X155.342 Y105.065 E0.02428

G1 X155.657 Y104.318 E0.02428

G1 X155.811 Y103.773 E0.01697

M204 S1250

G1 X156.148 Y103.954 F9000.000

G1 E-4.00000 F2400.000

G1 X162.889 Y105.117 F9000.000

G1 E4.00000 F900.000

M204 S1000

;TYPE:Solid infill

;WIDTH:0.456609

G1 F1272.328

G1 X162.218 Y104.447 E0.02887

G1 X161.618 Y104.447 E0.01827

G1 X162.701 Y105.530 E0.04661

G1 X162.701 Y106.130 E0.01827

G1 X161.018 Y104.447 E0.07245

G1 X160.418 Y104.447 E0.01827

G1 X162.701 Y106.730 E0.09828

G1 X162.701 Y107.330 E0.01827

G1 X159.817 Y104.447 E0.12412

G1 X159.217 Y104.447 E0.01827

G1 X162.701 Y107.930 E0.14996

G1 X162.701 Y108.531 E0.01827

G1 X158.617 Y104.447 E0.17580

G1 X158.017 Y104.447 E0.01827

G1 X162.701 Y109.131 E0.20163

G1 X162.701 Y109.731 E0.01827

G1 X157.417 Y104.447 E0.22747

G1 X156.816 Y104.447 E0.01827

G1 X162.701 Y110.331 E0.25331

G1 X162.701 Y110.932 E0.01827

G1 X156.343 Y104.573 E0.27370

G1 X156.165 Y104.995 E0.01395

G1 X162.701 Y111.532 E0.28137

G1 X162.701 Y112.132 E0.01827

G1 X155.984 Y105.415 E0.28914

G1 X155.764 Y105.796 E0.01338

G1 X162.701 Y112.732 E0.29859

G1 X162.701 Y113.332 E0.01827

G1 X155.545 Y106.176 E0.30805

```
G1 X155.287 Y106.518 E0.01305
G1 X162.701 Y113.933 E0.31916
G1 X162.701 Y114.533 E0.01827
G1 X155.029 Y106.861 E0.33026
G1 X154.737 Y107.169 E0.01292
G1 X162.701 Y115.133 E0.34283
G1 X162.701 Y115.733 E0.01827
G1 X154.441 Y107.474 E0.35554
G1 X154.117 Y107.749 E0.01296
G1 X162.701 Y116.334 E0.36951
G1 X162.701 Y116.934 E0.01827
G1 X153.784 Y108.017 E0.38382
G1 X153.426 Y108.259 E0.01316
G1 X162.701 Y117.534 E0.39926
G1 X162.101 Y117.534 E0.01825
G1 X153.056 Y108.489 E0.38938
G1 X152.913 Y108.577 E0.00511
G1 X152.660 Y108.694 E0.00847
G1 X161.501 Y117.534 E0.38056
G1 X160.901 Y117.534 E0.01827
G1 X152.249 Y108.883 E0.37241
G1 X152.095 Y108.954 E0.00518
G1 X151.811 Y109.045 E0.00907
G1 X160.301 Y117.534 E0.36545
G1 X159.700 Y117.534 E0.01827
G1 X151.355 Y109.190 E0.35921
G1 X150.863 Y109.297 E0.01534
G1 X159.100 Y117.534 E0.35457
G1 X158.500 Y117.534 E0.01827
```

G1 X150.357 Y109.392 E0.35050

G1 X149.793 Y109.428 E0.01722

G1 X157.900 Y117.534 E0.34897

G1 X157.299 Y117.534 E0.01827

G1 X149.212 Y109.447 E0.34814

G1 X148.612 Y109.447 E0.01827

G1 X152.275 Y113.110 E0.15769

G1 X152.095 Y113.027 E0.00603

G1 X151.357 Y112.792 E0.02358

G1 X148.011 Y109.447 E0.14400

G1 X147.411 Y109.447 E0.01827

G1 X150.599 Y112.634 E0.13722

G1 X150.351 Y112.588 E0.00767

G1 X149.926 Y112.562 E0.01297

G1 X146.811 Y109.447 E0.13409

G1 X146.211 Y109.447 E0.01827

G1 X149.298 Y112.534 E0.13291

G1 X148.698 Y112.534 E0.01827

G1 X145.610 Y109.447 E0.13291

G1 X145.010 Y109.447 E0.01827

G1 X148.098 Y112.534 E0.13291

G1 X147.498 Y112.534 E0.01827

G1 X144.410 Y109.447 E0.13291

G1 X143.810 Y109.447 E0.01827

G1 X146.897 Y112.534 E0.13291

G1 X146.297 Y112.534 E0.01827

G1 X143.210 Y109.447 E0.13291

G1 X142.609 Y109.447 E0.01827

G1 X145.697 Y112.534 E0.13291

```
G1 X145.097 Y112.534 E0.01827
G1 X142.009 Y109.447 E0.13291
G1 X141.613 Y109.447 E0.01205
G1 X141.613 Y109.651 E0.00622
G1 X144.497 Y112.534 E0.12412
G1 X143.896 Y112.534 E0.01827
G1 X141.613 Y110.251 E0.09828
G1 X141.613 Y110.852 E0.01827
G1 X143.296 Y112.534 E0.07244
G1 X142.696 Y112.534 E0.01827
G1 X141.613 Y111.452 E0.04660
G1 X141.613 Y112.052 E0.01827
G1 X142.284 Y112.722 E0.02886
M204 S1250
G1 E-4.00000 F2400.000
G1 X155.815 Y116.651 F9000.000
G1 E4.00000 F900.000
M204 S1000
G1 F1272.328
G1 X156.887 Y117.722 E0.04614
M204 S1250
; stop printing object Petg print.STL id:22 copy 0
; printing object Petg print.STL id:26 copy 0
G1 X156.128 Y122.787 F9000.000
M204 S800
;TYPE:Perimeter
;WIDTH:0.45
G1 F1292.454
G1 X162.999 Y122.787 E0.20589
```

```
G1 X162.999 Y136.502 E0.41094
G1 X156.128 Y136.502 E0.20589
G1 X156.036 Y136.178 E0.01007
G1 X155.701 Y135.384 E0.02583
G1 X155.271 Y134.637 E0.02583
G1 X154.752 Y133.949 E0.02583
G1 X154.153 Y133.329 E0.02583
G1 X153.482 Y132.789 E0.02583
G1 X152.749 Y132.334 E0.02583
G1 X151.966 Y131.974 E0.02583
G1 X151.145 Y131.712 E0.02583
G1 X150.297 Y131.554 E0.02583
G1 X149.457 Y131.502 E0.02523
G1 X141.285 Y131.502 E0.24487
G1 X141.285 Y127.787 E0.11130
G1 X149.457 Y127.787 E0.24487
G1 X150.297 Y127.735 E0.02523
G1 X151.145 Y127.577 E0.02583
G1 X151.966 Y127.315 E0.02583
G1 X152.749 Y126.955 E0.02583
G1 X153.482 Y126.501 E0.02583
G1 X154.153 Y125.960 E0.02583
G1 X154.752 Y125.340 E0.02583
G1 X155.271 Y124.652 E0.02583
G1 X155.701 Y123.905 E0.02583
G1 X156.036 Y123.111 E0.02583
G1 X156.112 Y122.845 E0.00828
M204 S1250
G1 X155.812 Y122.370 F9000.000
```

```
M204 S800
;TYPE:External perimeter
G1 F1292.454
G1 X163.417 Y122.370 E0.22787
G1 X163.417 Y136.920 E0.43598
G1 X155.812 Y136.920 E0.22787
G1 X155.641 Y136.317 E0.01877
G1 X155.326 Y135.570 E0.02428
G1 X154.922 Y134.868 E0.02428
G1 X154.434 Y134.221 E0.02428
G1 X153.871 Y133.639 E0.02428
G1 X153.240 Y133.130 E0.02428
G1 X152.551 Y132.703 E0.02428
G1 X151.815 Y132.364 E0.02428
G1 X151.043 Y132.118 E0.02428
G1 X150.246 Y131.969 E0.02428
G1 X149.444 Y131.920 E0.02407
G1 X140.867 Y131.920 E0.25701
G1 X140.867 Y127.370 E0.13634
G1 X149.444 Y127.370 E0.25701
G1 X150.246 Y127.320 E0.02407
G1 X151.043 Y127.171 E0.02428
G1 X151.815 Y126.925 E0.02428
G1 X152.551 Y126.586 E0.02428
G1 X153.240 Y126.159 E0.02428
G1 X153.871 Y125.651 E0.02428
G1 X154.434 Y125.068 E0.02428
G1 X154.922 Y124.421 E0.02428
G1 X155.326 Y123.719 E0.02428
```

```
G1 X155.641 Y122.972 E0.02428
G1 X155.796 Y122.427 E0.01697
M204 S1250
G1 X156.133 Y122.608 F9000.000
G1 E-4.00000 F2400.000
G1 X162.874 Y123.771 F9000.000
G1 E4.00000 F900.000
M204 S1000
;TYPE:Solid infill
;WIDTH:0.456609
G1 F1272.328
G1 X162.203 Y123.101 E0.02887
G1 X161.603 Y123.101 E0.01827
G1 X162.686 Y124.184 E0.04661
G1 X162.686 Y124.784 E0.01827
G1 X161.003 Y123.101 E0.07245
G1 X160.403 Y123.101 E0.01827
G1 X162.686 Y125.384 E0.09828
G1 X162.686 Y125.984 E0.01827
G1 X159.802 Y123.101 E0.12412
G1 X159.202 Y123.101 E0.01827
G1 X162.686 Y126.584 E0.14996
G1 X162.686 Y127.185 E0.01827
G1 X158.602 Y123.101 E0.17580
G1 X158.002 Y123.101 E0.01827
G1 X162.686 Y127.785 E0.20163
G1 X162.686 Y128.385 E0.01827
G1 X157.401 Y123.101 E0.22747
G1 X156.801 Y123.101 E0.01827
```

```
G1 X162.686 Y128.985 E0.25331
G1 X162.686 Y129.585 E0.01827
G1 X156.327 Y123.227 E0.27370
G1 X156.149 Y123.649 E0.01395
G1 X162.686 Y130.186 E0.28137
G1 X162.686 Y130.786 E0.01827
G1 X155.969 Y124.069 E0.28914
G1 X155.749 Y124.450 E0.01338
G1 X162.686 Y131.386 E0.29859
G1 X162.686 Y131.986 E0.01827
G1 X155.529 Y124.830 E0.30805
G1 X155.271 Y125.172 E0.01305
G1 X162.686 Y132.587 E0.31916
G1 X162.686 Y133.187 E0.01827
G1 X155.014 Y125.515 E0.33026
G1 X154.722 Y125.823 E0.01292
G1 X162.686 Y133.787 E0.34283
G1 X162.686 Y134.387 E0.01827
G1 X154.426 Y126.128 E0.35554
G1 X154.102 Y126.403 E0.01296
G1 X162.686 Y134.987 E0.36951
G1 X162.686 Y135.588 E0.01827
G1 X153.769 Y126.671 E0.38382
G1 X153.411 Y126.913 E0.01316
G1 X162.686 Y136.188 E0.39926
G1 X162.086 Y136.188 E0.01825
G1 X153.040 Y127.143 E0.38938
G1 X152.898 Y127.231 E0.00511
G1 X152.645 Y127.348 E0.00847
```

```
G1 X161.486 Y136.188 E0.38056
G1 X160.886 Y136.188 E0.01827
G1 X152.234 Y127.537 E0.37241
M73 P45 R38
G1 X152.080 Y127.608 E0.00518
G1 X151.796 Y127.699 E0.00907
G1 X160.285 Y136.188 E0.36545
G1 X159.685 Y136.188 E0.01827
G1 X151.340 Y127.844 E0.35921
G1 X150.848 Y127.951 E0.01534
G1 X159.085 Y136.188 E0.35457
M73 P45 R37
G1 X158.485 Y136.188 E0.01827
G1 X150.342 Y128.046 E0.35050
G1 X149.778 Y128.082 E0.01722
G1 X157.885 Y136.188 E0.34897
G1 X157.284 Y136.188 E0.01827
G1 X149.197 Y128.101 E0.34814
G1 X148.596 Y128.101 E0.01827
G1 X152.260 Y131.764 E0.15769
G1 X152.080 Y131.681 E0.00603
G1 X151.341 Y131.446 E0.02358
G1 X147.996 Y128.101 E0.14400
G1 X147.396 Y128.101 E0.01827
G1 X150.584 Y131.288 E0.13722
G1 X150.336 Y131.242 E0.00767
G1 X149.911 Y131.216 E0.01297
G1 X146.796 Y128.101 E0.13409
G1 X146.196 Y128.101 E0.01827
```

```
G1 X149.283 Y131.188 E0.13291
G1 X148.683 Y131.188 E0.01827
G1 X145.595 Y128.101 E0.13291
G1 X144.995 Y128.101 E0.01827
G1 X148.083 Y131.188 E0.13291
G1 X147.483 Y131.188 E0.01827
G1 X144.395 Y128.101 E0.13291
G1 X143.795 Y128.101 E0.01827
G1 X146.882 Y131.188 E0.13291
G1 X146.282 Y131.188 E0.01827
G1 X143.194 Y128.101 E0.13291
G1 X142.594 Y128.101 E0.01827
G1 X145.682 Y131.188 E0.13291
G1 X145.082 Y131.188 E0.01827
G1 X141.994 Y128.101 E0.13291
G1 X141.598 Y128.101 E0.01205
G1 X141.598 Y128.305 E0.00622
G1 X144.481 Y131.188 E0.12412
G1 X143.881 Y131.188 E0.01827
G1 X141.598 Y128.905 E0.09828
G1 X141.598 Y129.505 E0.01827
G1 X143.281 Y131.188 E0.07244
G1 X142.681 Y131.188 E0.01827
G1 X141.598 Y130.106 E0.04660
G1 X141.598 Y130.706 E0.01827
G1 X142.269 Y131.376 E0.02886
M204 S1250
G1 E-4.00000 F2400.000
G1 X155.800 Y135.305 F9000.000
```

```
G1 E4.00000 F900.000
M204 S1000
G1 F1272.328
G1 X156.872 Y136.376 E0.04614
M204 S1250
; stop printing object Petg print.STL id:26 copy 0
; printing object Petg print.STL id:0 copy 0
G1 E-4.00000 F2400.000
G1 X111.357 Y96.857 F9000.000
G1 E4.00000 F900.000
M204 S800
;TYPE:Perimeter
;WIDTH:0.45
G1 F1292.454
G1 X104.486 Y96.857 E0.20589
G1 X104.394 Y96.534 E0.01007
G1 X104.059 Y95.739 E0.02583
G1 X103.629 Y94.993 E0.02583
G1 X103.110 Y94.304 E0.02583
G1 X102.511 Y93.685 E0.02583
G1 X101.840 Y93.144 E0.02583
G1 X101.107 Y92.690 E0.02583
G1 X100.324 Y92.329 E0.02583
G1 X99.503 Y92.068 E0.02583
G1 X98.656 Y91.909 E0.02583
G1 X97.815 Y91.857 E0.02523
G1 X89.643 Y91.857 E0.24487
G1 X89.643 Y88.143 E0.11130
G1 X97.815 Y88.143 E0.24487
```

```
G1 X98.656 Y88.091 E0.02523
G1 X99.503 Y87.932 E0.02583
G1 X100.324 Y87.671 E0.02583
G1 X101.107 Y87.310 E0.02583
G1 X101.840 Y86.856 E0.02583
G1 X102.511 Y86.315 E0.02583
G1 X103.110 Y85.696 E0.02583
G1 X103.629 Y85.007 E0.02583
G1 X104.059 Y84.261 E0.02583
G1 X104.394 Y83.466 E0.02583
G1 X104.486 Y83.143 E0.01007
G1 X111.357 Y83.143 E0.20589
G1 X111.357 Y96.797 E0.40914
M204 S1250
G1 X111.775 Y97.275 F9000.000
M204 S800
;TYPE:External perimeter
G1 F1292.454
G1 X104.170 Y97.275 E0.22787
G1 X104.000 Y96.672 E0.01877
G1 X103.685 Y95.926 E0.02428
G1 X103.280 Y95.223 E0.02428
G1 X102.792 Y94.576 E0.02428
G1 X102.229 Y93.994 E0.02428
G1 X101.598 Y93.486 E0.02428
G1 X100.909 Y93.059 E0.02428
G1 X100.173 Y92.719 E0.02428
G1 X99.401 Y92.474 E0.02428
G1 X98.604 Y92.325 E0.02428
```

```
G1 X97.802 Y92.275 E0.02407
G1 X89.225 Y92.275 E0.25701
G1 X89.225 Y87.725 E0.13634
G1 X97.802 Y87.725 E0.25701
G1 X98.604 Y87.675 E0.02407
G1 X99.401 Y87.526 E0.02428
G1 X100.173 Y87.281 E0.02428
G1 X100.909 Y86.941 E0.02428
G1 X101.598 Y86.514 E0.02428
G1 X102.229 Y86.006 E0.02428
G1 X102.792 Y85.424 E0.02428
G1 X103.280 Y84.777 E0.02428
G1 X103.685 Y84.074 E0.02428
G1 X104.000 Y83.328 E0.02428
G1 X104.170 Y82.725 E0.01877
G1 X111.775 Y82.725 E0.22787
G1 X111.775 Y97.215 E0.43418
M204 S1250
G1 X111.401 Y97.133 F9000.000
G1 X105.230 Y96.732
M204 S1000
;TYPE:Solid infill
;WIDTH:0.456609
G1 F1272.328
G1 X104.158 Y95.660 E0.04614
M204 S1250
G1 E-4.00000 F2400.000
G1 X111.232 Y84.127 F9000.000
G1 E4.00000 F900.000
```

```
M204 S1000
G1 F1272.328
G1 X110.561 Y83.456 E0.02887
G1 X109.961 Y83.456 E0.01827
G1 X111.044 Y84.539 E0.04661
G1 X111.044 Y85.139 E0.01827
G1 X109.361 Y83.456 E0.07245
G1 X108.761 Y83.456 E0.01827
G1 X111.044 Y85.739 E0.09828
G1 X111.044 Y86.340 E0.01827
G1 X108.160 Y83.456 E0.12412
G1 X107.560 Y83.456 E0.01827
G1 X111.044 Y86.940 E0.14996
G1 X111.044 Y87.540 E0.01827
G1 X106.960 Y83.456 E0.17580
G1 X106.360 Y83.456 E0.01827
G1 X111.044 Y88.140 E0.20163
G1 X111.044 Y88.741 E0.01827
G1 X105.759 Y83.456 E0.22747
G1 X105.159 Y83.456 E0.01827
G1 X111.044 Y89.341 E0.25331
G1 X111.044 Y89.941 E0.01827
G1 X104.686 Y83.583 E0.27370
G1 X104.507 Y84.005 E0.01395
G1 X111.044 Y90.541 E0.28137
G1 X111.044 Y91.141 E0.01827
G1 X104.327 Y84.424 E0.28914
G1 X104.107 Y84.805 E0.01338
G1 X111.044 Y91.742 E0.29859
```

G1 X111.044 Y92.342 E0.01827

G1 X103.887 Y85.185 E0.30805

G1 X103.630 Y85.528 E0.01305

G1 X111.044 Y92.942 E0.31916

G1 X111.044 Y93.542 E0.01827

G1 X103.372 Y85.870 E0.33026

G1 X103.080 Y86.178 E0.01292

G1 X111.044 Y94.142 E0.34283

G1 X111.044 Y94.743 E0.01827

G1 X102.784 Y86.483 E0.35554

G1 X102.460 Y86.759 E0.01296

G1 X111.044 Y95.343 E0.36951

G1 X111.044 Y95.943 E0.01827

G1 X102.127 Y87.027 E0.38382

G1 X101.769 Y87.269 E0.01316

G1 X111.044 Y96.544 E0.39926

G1 X110.444 Y96.544 E0.01825

G1 X101.398 Y87.498 E0.38938

G1 X101.256 Y87.587 E0.00511

G1 X101.003 Y87.703 E0.00847

G1 X109.844 Y96.544 E0.38056

G1 X109.244 Y96.544 E0.01827

G1 X100.592 Y87.892 E0.37241

G1 X100.438 Y87.964 E0.00518

G1 X100.154 Y88.054 E0.00907

G1 X108.643 Y96.544 E0.36545

G1 X108.043 Y96.544 E0.01827

G1 X99.698 Y88.199 E0.35921

G1 X99.206 Y88.307 E0.01534

```
G1 X107.443 Y96.544 E0.35457
G1 X106.843 Y96.544 E0.01827
G1 X98.700 Y88.401 E0.35050
G1 X98.136 Y88.437 E0.01722
G1 X106.243 Y96.544 E0.34897
G1 X105.642 Y96.544 E0.01827
G1 X97.555 Y88.456 E0.34814
G1 X96.954 Y88.456 E0.01827
G1 X100.618 Y92.119 E0.15769
G1 X100.438 Y92.036 E0.00603
G1 X99.699 Y91.801 E0.02358
G1 X96.354 Y88.456 E0.14400
G1 X95.754 Y88.456 E0.01827
G1 X98.942 Y91.644 E0.13722
G1 X98.694 Y91.598 E0.00767
G1 X98.269 Y91.571 E0.01297
G1 X95.154 Y88.456 E0.13409
G1 X94.554 Y88.456 E0.01827
G1 X97.641 Y91.544 E0.13291
G1 X97.041 Y91.544 E0.01827
G1 X93.953 Y88.456 E0.13291
G1 X93.353 Y88.456 E0.01827
G1 X96.441 Y91.544 E0.13291
G1 X95.841 Y91.544 E0.01827
G1 X92.753 Y88.456 E0.13291
G1 X92.153 Y88.456 E0.01827
G1 X95.240 Y91.544 E0.13291
G1 X94.640 Y91.544 E0.01827
G1 X91.552 Y88.456 E0.13291
```

```
G1 X90.952 Y88.456 E0.01827
G1 X94.040 Y91.544 E0.13291
G1 X93.440 Y91.544 E0.01827
G1 X90.352 Y88.456 E0.13291
G1 X89.956 Y88.456 E0.01205
G1 X89.956 Y88.661 E0.00622
G1 X92.839 Y91.544 E0.12412
G1 X92.239 Y91.544 E0.01827
G1 X89.956 Y89.261 E0.09828
G1 X89.956 Y89.861 E0.01827
G1 X91.639 Y91.544 E0.07244
G1 X91.039 Y91.544 E0.01827
G1 X89.956 Y90.461 E0.04660
G1 X89.956 Y91.061 E0.01827
G1 X90.627 Y91.732 E0.02886
M204 S1250
; stop printing object Petg print.STL id:0 copy 0
; printing object tpu print.STL id:1 copy 0
; stop printing object tpu print.STL id:1 copy 0
; printing object tpu print.STL id:5 copy 0
; stop printing object tpu print.STL id:5 copy 0
; printing object tpu print.STL id:9 copy 0
; stop printing object tpu print.STL id:9 copy 0
; printing object Petg print.STL id:8 copy 0
G1 E-4.00000 F2400.000
G1 X104.530 Y54.716 F9000.000
G1 E4.00000 F900.000
M204 S800
;TYPE:Perimeter
```

```
;WIDTH:0.45
G1 F1292.454
G1 X104.438 Y54.392 E0.01007
G1 X104.103 Y53.598 E0.02583
G1 X103.673 Y52.851 E0.02583
G1 X103.154 Y52.163 E0.02583
G1 X102.555 Y51.544 E0.02583
G1 X101.883 Y51.003 E0.02583
G1 X101.151 Y50.549 E0.02583
G1 X100.368 Y50.188 E0.02583
G1 X99.546 Y49.926 E0.02583
G1 X98.699 Y49.768 E0.02583
G1 X97.859 Y49.716 E0.02523
G1 X89.686 Y49.716 E0.24487
G1 X89.686 Y46.002 E0.11130
G1 X97.859 Y46.002 E0.24487
G1 X98.699 Y45.950 E0.02523
G1 X99.546 Y45.791 E0.02583
G1 X100.368 Y45.530 E0.02583
G1 X101.151 Y45.169 E0.02583
G1 X101.883 Y44.715 E0.02583
G1 X102.555 Y44.174 E0.02583
G1 X103.154 Y43.555 E0.02583
G1 X103.673 Y42.866 E0.02583
G1 X104.103 Y42.119 E0.02583
G1 X104.438 Y41.325 E0.02583
G1 X104.530 Y41.002 E0.01007
G1 X111.401 Y41.002 E0.20589
G1 X111.401 Y54.716 E0.41094
```

```
G1 X104.590 Y54.716 E0.20409
M204 S1250
G1 X104.214 Y55.134 F9000.000
M204 S800
;TYPE:External perimeter
G1 F1292.454
G1 X104.043 Y54.531 E0.01877
G1 X103.728 Y53.784 E0.02428
G1 X103.324 Y53.082 E0.02428
G1 X102.836 Y52.435 E0.02428
G1 X102.272 Y51.853 E0.02428
G1 X101.641 Y51.344 E0.02428
G1 X100.952 Y50.917 E0.02428
G1 X100.216 Y50.578 E0.02428
G1 X99.444 Y50.332 E0.02428
G1 X98.648 Y50.183 E0.02428
G1 X97.846 Y50.134 E0.02407
G1 X89.269 Y50.134 E0.25701
G1 X89.269 Y45.584 E0.13634
G1 X97.846 Y45.584 E0.25701
G1 X98.648 Y45.534 E0.02407
G1 X99.444 Y45.385 E0.02428
G1 X100.216 Y45.139 E0.02428
G1 X100.952 Y44.800 E0.02428
G1 X101.641 Y44.373 E0.02428
G1 X102.272 Y43.865 E0.02428
G1 X102.836 Y43.282 E0.02428
G1 X103.324 Y42.635 E0.02428
G1 X103.728 Y41.933 E0.02428
```

```
G1 X104.043 Y41.186 E0.02428
G1 X104.214 Y40.584 E0.01877
G1 X111.819 Y40.584 E0.22787
G1 X111.819 Y55.134 E0.43598
M73 P46 R37
G1 X104.274 Y55.134 E0.22608
M204 S1250
G1 X104.204 Y54.734 F9000.000
G1 X105.274 Y54.591
M204 S1000
;TYPE:Solid infill
;WIDTH:0.456609
G1 F1272.328
G1 X104.202 Y53.519 E0.04614
M204 S1250
G1 E-4.00000 F2400.000
G1 X111.276 Y41.985 F9000.000
G1 E4.00000 F900.000
M204 S1000
G1 F1272.328
G1 X110.605 Y41.315 E0.02887
G1 X110.005 Y41.315 E0.01827
G1 X111.087 Y42.398 E0.04661
G1 X111.087 Y42.998 E0.01827
G1 X109.404 Y41.315 E0.07245
G1 X108.804 Y41.315 E0.01827
G1 X111.087 Y43.598 E0.09828
G1 X111.087 Y44.198 E0.01827
G1 X108.204 Y41.315 E0.12412
```

G1 X107.604 Y41.315 E0.01827

G1 X111.087 Y44.799 E0.14996

G1 X111.087 Y45.399 E0.01827

G1 X107.004 Y41.315 E0.17580

G1 X106.403 Y41.315 E0.01827

G1 X111.087 Y45.999 E0.20163

G1 X111.087 Y46.599 E0.01827

G1 X105.803 Y41.315 E0.22747

G1 X105.203 Y41.315 E0.01827

G1 X111.087 Y47.199 E0.25331

G1 X111.087 Y47.800 E0.01827

G1 X104.729 Y41.441 E0.27370

G1 X104.551 Y41.863 E0.01395

G1 X111.087 Y48.400 E0.28137

G1 X111.087 Y49.000 E0.01827

G1 X104.370 Y42.283 E0.28914

G1 X104.151 Y42.664 E0.01338

G1 X111.087 Y49.600 E0.29859

G1 X111.087 Y50.201 E0.01827

G1 X103.931 Y43.044 E0.30805

G1 X103.673 Y43.386 E0.01305

G1 X111.087 Y50.801 E0.31916

G1 X111.087 Y51.401 E0.01827

G1 X103.415 Y43.729 E0.33026

G1 X103.123 Y44.037 E0.01292

G1 X111.087 Y52.001 E0.34283

G1 X111.087 Y52.601 E0.01827

G1 X102.828 Y44.342 E0.35554

G1 X102.503 Y44.617 E0.01296

```
G1 X111.087 Y53.202 E0.36951
G1 X111.087 Y53.802 E0.01827
G1 X102.171 Y44.885 E0.38382
G1 X101.813 Y45.127 E0.01316
G1 X111.087 Y54.403 E0.39926
G1 X110.488 Y54.403 E0.01825
G1 X101.442 Y45.357 E0.38938
G1 X101.299 Y45.445 E0.00511
G1 X101.047 Y45.562 E0.00847
G1 X109.888 Y54.403 E0.38056
G1 X109.287 Y54.403 E0.01827
G1 X100.636 Y45.751 E0.37241
G1 X100.481 Y45.822 E0.00518
G1 X100.197 Y45.913 E0.00907
G1 X108.687 Y54.403 E0.36545
G1 X108.087 Y54.403 E0.01827
G1 X99.742 Y46.058 E0.35921
G1 X99.250 Y46.165 E0.01534
G1 X107.487 Y54.403 E0.35457
G1 X106.886 Y54.403 E0.01827
G1 X98.744 Y46.260 E0.35050
G1 X98.179 Y46.296 E0.01722
G1 X106.286 Y54.403 E0.34897
G1 X105.686 Y54.403 E0.01827
G1 X97.598 Y46.315 E0.34814
G1 X96.998 Y46.315 E0.01827
G1 X100.661 Y49.978 E0.15769
G1 X100.481 Y49.895 E0.00603
G1 X99.743 Y49.660 E0.02358
```

```
G1 X96.398 Y46.315 E0.14400
G1 X95.798 Y46.315 E0.01827
G1 X98.985 Y49.503 E0.13722
G1 X98.738 Y49.456 E0.00767
G1 X98.313 Y49.430 E0.01297
G1 X95.197 Y46.315 E0.13409
G1 X94.597 Y46.315 E0.01827
G1 X97.685 Y49.403 E0.13291
G1 X97.085 Y49.403 E0.01827
G1 X93.997 Y46.315 E0.13291
G1 X93.397 Y46.315 E0.01827
G1 X96.484 Y49.403 E0.13291
G1 X95.884 Y49.403 E0.01827
G1 X92.797 Y46.315 E0.13291
G1 X92.196 Y46.315 E0.01827
G1 X95.284 Y49.403 E0.13291
G1 X94.684 Y49.403 E0.01827
G1 X91.596 Y46.315 E0.13291
G1 X90.996 Y46.315 E0.01827
G1 X94.084 Y49.403 E0.13291
G1 X93.483 Y49.403 E0.01827
G1 X90.396 Y46.315 E0.13291
G1 X90.000 Y46.315 E0.01205
G1 X90.000 Y46.519 E0.00622
G1 X92.883 Y49.403 E0.12412
G1 X92.283 Y49.403 E0.01827
G1 X90.000 Y47.119 E0.09828
G1 X90.000 Y47.720 E0.01827
G1 X91.683 Y49.403 E0.07244
```

```
G1 X91.082 Y49.403 E0.01827
G1 X90.000 Y48.320 E0.04660
G1 X90.000 Y48.920 E0.01827
G1 X90.670 Y49.591 E0.02886
M204 S1250
; stop printing object Petg print.STL id:8 copy 0
; printing object Petg print.STL id:4 copy 0
G1 E-4.00000 F2400.000
G1 X89.694 Y65.950 F9000.000
G1 E4.00000 F900.000
M204 S800
;TYPE:Perimeter
;WIDTH:0.45
G1 F1292.454
G1 X97.866 Y65.950 E0.24487
G1 X98.707 Y65.899 E0.02523
G1 X99.554 Y65.740 E0.02583
M73 P46 R36
G1 X100.375 Y65.478 E0.02583
G1 X101.158 Y65.118 E0.02583
G1 X101.891 Y64.664 E0.02583
G1 X102.562 Y64.123 E0.02583
G1 X103.162 Y63.503 E0.02583
G1 X103.680 Y62.815 E0.02583
G1 X104.111 Y62.068 E0.02583
G1 X104.446 Y61.274 E0.02583
G1 X104.537 Y60.950 E0.01007
G1 X111.408 Y60.950 E0.20589
G1 X111.408 Y74.665 E0.41094
```

```
G1 X104.537 Y74.665 E0.20589
G1 X104.446 Y74.341 E0.01007
G1 X104.111 Y73.547 E0.02583
G1 X103.680 Y72.800 E0.02583
G1 X103.162 Y72.112 E0.02583
G1 X102.562 Y71.493 E0.02583
G1 X101.891 Y70.952 E0.02583
G1 X101.158 Y70.497 E0.02583
G1 X100.375 Y70.137 E0.02583
G1 X99.554 Y69.875 E0.02583
G1 X98.707 Y69.717 E0.02583
G1 X97.866 Y69.665 E0.02523
G1 X89.694 Y69.665 E0.24487
G1 X89.694 Y66.010 E0.10950
M204 S1250
G1 X89.276 Y65.533 F9000.000
M204 S800
;TYPE:External perimeter
G1 F1292.454
G1 X97.853 Y65.533 E0.25701
G1 X98.655 Y65.483 E0.02407
G1 X99.452 Y65.334 E0.02428
G1 X100.224 Y65.088 E0.02428
G1 X100.960 Y64.749 E0.02428
G1 X101.649 Y64.322 E0.02428
G1 X102.280 Y63.814 E0.02428
G1 X102.843 Y63.231 E0.02428
G1 X103.331 Y62.584 E0.02428
G1 X103.736 Y61.882 E0.02428
```

```
G1 X104.051 Y61.135 E0.02428
G1 X104.221 Y60.533 E0.01877
G1 X111.826 Y60.533 E0.22787
G1 X111.826 Y75.083 E0.43598
G1 X104.221 Y75.083 E0.22787
G1 X104.051 Y74.480 E0.01877
G1 X103.736 Y73.733 E0.02428
G1 X103.331 Y73.031 E0.02428
G1 X102.843 Y72.384 E0.02428
G1 X102.280 Y71.802 E0.02428
G1 X101.649 Y71.293 E0.02428
G1 X100.960 Y70.866 E0.02428
G1 X100.224 Y70.527 E0.02428
G1 X99.452 Y70.281 E0.02428
G1 X98.655 Y70.132 E0.02428
G1 X97.853 Y70.083 E0.02407
G1 X89.276 Y70.083 E0.25701
G1 X89.276 Y65.593 E0.13454
M204 S1250
G1 X89.671 Y65.597 F9000.000
G1 X90.678 Y69.539
M204 S1000
;TYPE:Solid infill
;WIDTH:0.456609
G1 F1272.328
G1 X90.007 Y68.869 E0.02886
G1 X90.007 Y68.269 E0.01827
G1 X91.090 Y69.351 E0.04660
G1 X91.690 Y69.351 E0.01827
```

G1 X90.007 Y67.669 E0.07244

G1 X90.007 Y67.068 E0.01827

G1 X92.290 Y69.351 E0.09828

G1 X92.891 Y69.351 E0.01827

G1 X90.007 Y66.468 E0.12412

G1 X90.007 Y66.264 E0.00622

G1 X90.403 Y66.264 E0.01205

G1 X93.491 Y69.351 E0.13291

G1 X94.091 Y69.351 E0.01827

G1 X91.003 Y66.264 E0.13291

G1 X91.604 Y66.264 E0.01827

G1 X94.691 Y69.351 E0.13291

G1 X95.292 Y69.351 E0.01827

G1 X92.204 Y66.264 E0.13291

G1 X92.804 Y66.264 E0.01827

G1 X95.892 Y69.351 E0.13291

G1 X96.492 Y69.351 E0.01827

G1 X93.404 Y66.264 E0.13291

G1 X94.005 Y66.264 E0.01827

G1 X97.092 Y69.351 E0.13291

G1 X97.692 Y69.351 E0.01827

G1 X94.605 Y66.264 E0.13291

G1 X95.205 Y66.264 E0.01827

G1 X98.320 Y69.379 E0.13409

G1 X98.745 Y69.405 E0.01297

G1 X98.993 Y69.451 E0.00767

G1 X95.805 Y66.264 E0.13722

G1 X96.405 Y66.264 E0.01827

G1 X99.751 Y69.609 E0.14400

G1 X100.489 Y69.844 E0.02358

G1 X100.669 Y69.927 E0.00603

G1 X97.006 Y66.264 E0.15769

G1 X97.606 Y66.264 E0.01827

G1 X105.693 Y74.351 E0.34814

G1 X106.294 Y74.351 E0.01827

G1 X98.187 Y66.245 E0.34897

G1 X98.751 Y66.209 E0.01722

G1 X106.894 Y74.351 E0.35050

G1 X107.494 Y74.351 E0.01827

G1 X99.257 Y66.114 E0.35457

G1 X99.749 Y66.007 E0.01534

G1 X108.094 Y74.351 E0.35921

G1 X108.695 Y74.351 E0.01827

G1 X100.205 Y65.862 E0.36545

G1 X100.489 Y65.771 E0.00907

G1 X100.643 Y65.700 E0.00518

G1 X109.295 Y74.351 E0.37241

G1 X109.895 Y74.351 E0.01827

G1 X101.054 Y65.511 E0.38056

G1 X101.307 Y65.394 E0.00847

G1 X101.450 Y65.306 E0.00511

G1 X110.495 Y74.351 E0.38938

G1 X111.095 Y74.351 E0.01825

G1 X101.820 Y65.076 E0.39926

G1 X102.178 Y64.834 E0.01316

G1 X111.095 Y73.751 E0.38382

G1 X111.095 Y73.151 E0.01827

G1 X102.511 Y64.566 E0.36951

```
G1 X102.835 Y64.291 E0.01296
G1 X111.095 Y72.550 E0.35554
G1 X111.095 Y71.950 E0.01827
G1 X103.131 Y63.986 E0.34283
G1 X103.423 Y63.678 E0.01292
G1 X111.095 Y71.350 E0.33026
G1 X111.095 Y70.750 E0.01827
G1 X103.681 Y63.335 E0.31916
G1 X103.939 Y62.993 E0.01305
G1 X111.095 Y70.149 E0.30805
G1 X111.095 Y69.549 E0.01827
M73 P47 R36
G1 X104.159 Y62.613 E0.29859
G1 X104.378 Y62.232 E0.01338
G1 X111.095 Y68.949 E0.28914
G1 X111.095 Y68.349 E0.01827
G1 X104.559 Y61.812 E0.28137
G1 X104.737 Y61.390 E0.01395
G1 X111.095 Y67.749 E0.27370
G1 X111.095 Y67.148 E0.01827
G1 X105.210 Y61.264 E0.25331
G1 X105.811 Y61.264 E0.01827
G1 X111.095 Y66.548 E0.22747
G1 X111.095 Y65.948 E0.01827
G1 X106.411 Y61.264 E0.20163
G1 X107.011 Y61.264 E0.01827
G1 X111.095 Y65.348 E0.17580
G1 X111.095 Y64.747 E0.01827
G1 X107.611 Y61.264 E0.14996
```

```
G1 X108.212 Y61.264 E0.01827
G1 X111.095 Y64.147 E0.12412
G1 X111.095 Y63.547 E0.01827
G1 X108.812 Y61.264 E0.09828
G1 X109.412 Y61.264 E0.01827
G1 X111.095 Y62.947 E0.07245
G1 X111.095 Y62.347 E0.01827
G1 X110.012 Y61.264 E0.04661
G1 X110.612 Y61.264 E0.01827
G1 X111.283 Y61.934 E0.02887
M204 S1250
G1 E-4.00000 F2400.000
G1 X104.209 Y73.468 F9000.000
G1 E4.00000 F900.000
M204 S1000
G1 F1272.328
G1 X105.281 Y74.539 E0.04614
M204 S1250
; stop printing object Petg print.STL id:4 copy 0
; printing object tpu print.STL id:29 copy 0
; stop printing object tpu print.STL id:29 copy 0
; printing object tpu print.STL id:25 copy 0
; stop printing object tpu print.STL id:25 copy 0
; printing object Petg print.STL id:24 copy 0
G1 E-4.00000 F2400.000
G1 X141.344 Y69.840 F9000.000
G1 E4.00000 F900.000
M204 S800
;TYPE:Perimeter
```

```
;WIDTH:0.45
G1 F1292.454
G1 X141.344 Y66.126 E0.11130
G1 X149.516 Y66.126 E0.24487
G1 X150.357 Y66.074 E0.02523
G1 X151.204 Y65.915 E0.02583
G1 X152.026 Y65.654 E0.02583
G1 X152.808 Y65.293 E0.02583
G1 X153.541 Y64.839 E0.02583
G1 X154.212 Y64.298 E0.02583
G1 X154.812 Y63.679 E0.02583
G1 X155.331 Y62.990 E0.02583
G1 X155.761 Y62.243 E0.02583
G1 X156.096 Y61.449 E0.02583
G1 X156.187 Y61.126 E0.01007
G1 X163.059 Y61.126 E0.20589
G1 X163.059 Y74.840 E0.41094
G1 X156.187 Y74.840 E0.20589
G1 X156.096 Y74.516 E0.01007
G1 X155.761 Y73.722 E0.02583
G1 X155.331 Y72.975 E0.02583
G1 X154.812 Y72.287 E0.02583
G1 X154.212 Y71.668 E0.02583
G1 X153.541 Y71.127 E0.02583
G1 X152.808 Y70.673 E0.02583
G1 X152.026 Y70.312 E0.02583
G1 X151.204 Y70.050 E0.02583
G1 X150.357 Y69.892 E0.02583
G1 X149.516 Y69.840 E0.02523
```

G1 X141.404 Y69.840 E0.24307

M204 S1250

G1 X140.926 Y70.258 F9000.000

M204 S800

;TYPE:External perimeter

G1 F1292.454

G1 X140.926 Y65.708 E0.13634

G1 X149.503 Y65.708 E0.25701

G1 X150.305 Y65.658 E0.02407

G1 X151.102 Y65.509 E0.02428

G1 X151.874 Y65.263 E0.02428

G1 X152.610 Y64.924 E0.02428

G1 X153.299 Y64.497 E0.02428

G1 X153.930 Y63.989 E0.02428

G1 X154.494 Y63.406 E0.02428

G1 X154.981 Y62.759 E0.02428

G1 X155.386 Y62.057 E0.02428

G1 X155.701 Y61.310 E0.02428

G1 X155.872 Y60.708 E0.01877

G1 X163.476 Y60.708 E0.22787

G1 X163.476 Y75.258 E0.43598

G1 X155.872 Y75.258 E0.22787

G1 X155.701 Y74.655 E0.01877

G1 X155.386 Y73.908 E0.02428

G1 X154.981 Y73.206 E0.02428

G1 X154.494 Y72.559 E0.02428

G1 X153.930 Y71.977 E0.02428

G1 X153.299 Y71.468 E0.02428

G1 X152.610 Y71.041 E0.02428

```
G1 X151.874 Y70.702 E0.02428
G1 X151.102 Y70.456 E0.02428
G1 X150.305 Y70.307 E0.02428
G1 X149.503 Y70.258 E0.02407
G1 X140.986 Y70.258 E0.25521
M204 S1250
G1 X141.127 Y69.912 F9000.000
G1 X142.328 Y69.715
M204 S1000
;TYPE:Solid infill
;WIDTH:0.456609
G1 F1272.328
G1 X141.658 Y69.044 E0.02886
G1 X141.658 Y68.444 E0.01827
G1 X142.740 Y69.527 E0.04660
G1 X143.340 Y69.527 E0.01827
G1 X141.658 Y67.844 E0.07244
G1 X141.658 Y67.243 E0.01827
G1 X143.941 Y69.527 E0.09828
G1 X144.541 Y69.527 E0.01827
G1 X141.658 Y66.643 E0.12412
G1 X141.658 Y66.439 E0.00622
G1 X142.053 Y66.439 E0.01205
G1 X145.141 Y69.527 E0.13291
G1 X145.741 Y69.527 E0.01827
G1 X142.654 Y66.439 E0.13291
G1 X143.254 Y66.439 E0.01827
G1 X146.342 Y69.527 E0.13291
G1 X146.942 Y69.527 E0.01827
```

```
G1 X143.854 Y66.439 E0.13291
G1 X144.454 Y66.439 E0.01827
G1 X147.542 Y69.527 E0.13291
G1 X148.142 Y69.527 E0.01827
G1 X145.055 Y66.439 E0.13291
G1 X145.655 Y66.439 E0.01827
G1 X148.742 Y69.527 E0.13291
G1 X149.343 Y69.527 E0.01827
G1 X146.255 Y66.439 E0.13291
G1 X146.855 Y66.439 E0.01827
G1 X149.970 Y69.554 E0.13409
G1 X150.396 Y69.580 E0.01297
G1 X150.643 Y69.627 E0.00767
G1 X147.455 Y66.439 E0.13722
G1 X148.056 Y66.439 E0.01827
G1 X151.401 Y69.784 E0.14400
G1 X152.139 Y70.019 E0.02358
G1 X152.319 Y70.102 E0.00603
G1 X148.656 Y66.439 E0.15769
G1 X149.256 Y66.439 E0.01827
G1 X157.344 Y74.527 E0.34814
G1 X157.944 Y74.527 E0.01827
G1 X149.837 Y66.420 E0.34897
G1 X150.402 Y66.384 E0.01722
G1 X158.544 Y74.527 E0.35050
G1 X159.144 Y74.527 E0.01827
G1 X150.907 Y66.289 E0.35457
G1 X151.400 Y66.182 E0.01534
G1 X159.745 Y74.527 E0.35921
```

```
G1 X160.345 Y74.527 E0.01827
G1 X151.855 Y66.037 E0.36545
G1 X152.139 Y65.946 E0.00907
G1 X152.294 Y65.875 E0.00518
G1 X160.945 Y74.527 E0.37241
G1 X161.545 Y74.527 E0.01827
G1 X152.704 Y65.686 E0.38056
G1 X152.957 Y65.569 E0.00847
G1 X153.100 Y65.481 E0.00511
G1 X162.145 Y74.527 E0.38938
G1 X162.745 Y74.527 E0.01825
G1 X153.470 Y65.251 E0.39926
G1 X153.829 Y65.009 E0.01316
G1 X162.745 Y73.926 E0.38382
G1 X162.745 Y73.326 E0.01827
G1 X154.161 Y64.742 E0.36951
G1 X154.486 Y64.466 E0.01296
G1 X162.745 Y72.725 E0.35554
G1 X162.745 Y72.125 E0.01827
G1 X154.781 Y64.161 E0.34283
G1 X155.073 Y63.853 E0.01292
G1 X162.745 Y71.525 E0.33026
G1 X162.745 Y70.925 E0.01827
G1 X155.331 Y63.510 E0.31916
G1 X155.589 Y63.168 E0.01305
G1 X162.745 Y70.325 E0.30805
G1 X162.745 Y69.724 E0.01827
G1 X155.809 Y62.788 E0.29859
G1 X156.028 Y62.407 E0.01338
```

```
G1 X162.745 Y69.124 E0.28914
G1 X162.745 Y68.524 E0.01827
G1 X156.209 Y61.987 E0.28137
G1 X156.387 Y61.565 E0.01395
G1 X162.745 Y67.924 E0.27370
G1 X162.745 Y67.323 E0.01827
G1 X156.861 Y61.439 E0.25331
G1 X157.461 Y61.439 E0.01827
G1 X162.745 Y66.723 E0.22747
G1 X162.745 Y66.123 E0.01827
G1 X158.061 Y61.439 E0.20163
G1 X158.661 Y61.439 E0.01827
G1 X162.745 Y65.523 E0.17580
G1 X162.745 Y64.923 E0.01827
G1 X159.262 Y61.439 E0.14996
G1 X159.862 Y61.439 E0.01827
G1 X162.745 Y64.322 E0.12412
G1 X162.745 Y63.722 E0.01827
G1 X160.462 Y61.439 E0.09828
G1 X161.062 Y61.439 E0.01827
G1 X162.745 Y63.122 E0.07245
G1 X162.745 Y62.522 E0.01827
G1 X161.662 Y61.439 E0.04661
G1 X162.263 Y61.439 E0.01827
G1 X162.933 Y62.109 E0.02887
M204 S1250
G1 E-4.00000 F2400.000
G1 X155.860 Y73.643 F9000.000
G1 E4.00000 F900.000
```

M204 S1000

G1 F1272.328

G1 X156.932 Y74.715 E0.04614

M204 S1250

; stop printing object Petg print.STL id:24 copy 0

; printing object Petg print.STL id:28 copy 0

G1 E-4.00000 F2400.000

G1 X156.180 Y54.891 F9000.000

G1 E4.00000 F900.000

M204 S800

;TYPE:Perimeter

;WIDTH:0.45

G1 F1292.454

G1 X156.088 Y54.567 E0.01007

G1 X155.753 Y53.773 E0.02583

G1 X155.323 Y53.026 E0.02583

G1 X154.804 Y52.338 E0.02583

G1 X154.205 Y51.719 E0.02583

G1 X153.534 Y51.178 E0.02583

G1 X152.801 Y50.724 E0.02583

G1 X152.018 Y50.363 E0.02583

G1 X151.197 Y50.101 E0.02583

G1 X150.349 Y49.943 E0.02583

G1 X149.509 Y49.891 E0.02523

G1 X141.337 Y49.891 E0.24487

G1 X141.337 Y46.177 E0.11130

G1 X149.509 Y46.177 E0.24487

G1 X150.349 Y46.125 E0.02523

G1 X151.197 Y45.966 E0.02583

```
G1 X152.018 Y45.705 E0.02583
G1 X152.801 Y45.344 E0.02583
G1 X153.534 Y44.890 E0.02583
G1 X154.205 Y44.349 E0.02583
G1 X154.804 Y43.730 E0.02583
G1 X155.323 Y43.041 E0.02583
G1 X155.753 Y42.294 E0.02583
G1 X156.088 Y41.500 E0.02583
G1 X156.180 Y41.177 E0.01007
G1 X163.051 Y41.177 E0.20589
G1 X163.051 Y54.891 E0.41094
G1 X156.240 Y54.891 E0.20409
M204 S1250
G1 X155.864 Y55.309 F9000.000
M204 S800
;TYPE:External perimeter
G1 F1292.454
G1 X155.693 Y54.706 E0.01877
G1 X155.378 Y53.959 E0.02428
G1 X154.974 Y53.257 E0.02428
G1 X154.486 Y52.610 E0.02428
G1 X153.923 Y52.028 E0.02428
G1 X153.292 Y51.519 E0.02428
G1 X152.603 Y51.092 E0.02428
G1 X151.867 Y50.753 E0.02428
G1 X151.094 Y50.507 E0.02428
G1 X150.298 Y50.358 E0.02428
G1 X149.496 Y50.309 E0.02407
G1 X140.919 Y50.309 E0.25701
```

```
G1 X140.919 Y45.759 E0.13634
G1 X149.496 Y45.759 E0.25701
G1 X150.298 Y45.709 E0.02407
G1 X151.094 Y45.560 E0.02428
G1 X151.867 Y45.314 E0.02428
G1 X152.603 Y44.975 E0.02428
G1 X153.292 Y44.548 E0.02428
G1 X153.923 Y44.040 E0.02428
G1 X154.486 Y43.458 E0.02428
G1 X154.974 Y42.810 E0.02428
G1 X155.378 Y42.108 E0.02428
G1 X155.693 Y41.361 E0.02428
G1 X155.864 Y40.759 E0.01877
G1 X163.469 Y40.759 E0.22787
G1 X163.469 Y55.309 E0.43598
G1 X155.924 Y55.309 E0.22608
M204 S1250
G1 X155.855 Y54.909 F9000.000
G1 X156.924 Y54.766
M204 S1000
;TYPE:Solid infill
;WIDTH:0.456609
G1 F1272.328
G1 X155.852 Y53.694 E0.04614
M204 S1250
G1 E-4.00000 F2400.000
G1 X162.926 Y42.161 F9000.000
G1 E4.00000 F900.000
M204 S1000
```

```
G1 F1272.328
G1 X162.255 Y41.490 E0.02887
G1 X161.655 Y41.490 E0.01827
G1 X162.738 Y42.573 E0.04661
G1 X162.738 Y43.173 E0.01827
G1 X161.055 Y41.490 E0.07245
G1 X160.454 Y41.490 E0.01827
G1 X162.738 Y43.773 E0.09828
G1 X162.738 Y44.373 E0.01827
G1 X159.854 Y41.490 E0.12412
G1 X159.254 Y41.490 E0.01827
G1 X162.738 Y44.974 E0.14996
G1 X162.738 Y45.574 E0.01827
M73 P47 R35
G1 X158.654 Y41.490 E0.17580
G1 X158.054 Y41.490 E0.01827
G1 X162.738 Y46.174 E0.20163
G1 X162.738 Y46.774 E0.01827
G1 X157.453 Y41.490 E0.22747
M73 P48 R35
G1 X156.853 Y41.490 E0.01827
G1 X162.738 Y47.375 E0.25331
G1 X162.738 Y47.975 E0.01827
G1 X156.379 Y41.616 E0.27370
G1 X156.201 Y42.039 E0.01395
G1 X162.738 Y48.575 E0.28137
G1 X162.738 Y49.175 E0.01827
G1 X156.021 Y42.458 E0.28914
G1 X155.801 Y42.839 E0.01338
```

```
G1 X162.738 Y49.775 E0.29859
G1 X162.738 Y50.376 E0.01827
G1 X155.581 Y43.219 E0.30805
G1 X155.323 Y43.561 E0.01305
G1 X162.738 Y50.976 E0.31916
G1 X162.738 Y51.576 E0.01827
G1 X155.065 Y43.904 E0.33026
G1 X154.773 Y44.212 E0.01292
G1 X162.738 Y52.176 E0.34283
G1 X162.738 Y52.776 E0.01827
G1 X154.478 Y44.517 E0.35554
G1 X154.154 Y44.793 E0.01296
G1 X162.738 Y53.377 E0.36951
G1 X162.738 Y53.977 E0.01827
G1 X153.821 Y45.060 E0.38382
G1 X153.463 Y45.302 E0.01316
G1 X162.738 Y54.578 E0.39926
G1 X162.138 Y54.578 E0.01825
G1 X153.092 Y45.532 E0.38938
G1 X152.950 Y45.620 E0.00511
G1 X152.697 Y45.737 E0.00847
G1 X161.538 Y54.578 E0.38056
G1 X160.938 Y54.578 E0.01827
G1 X152.286 Y45.926 E0.37241
G1 X152.132 Y45.997 E0.00518
G1 X151.847 Y46.088 E0.00907
G1 X160.337 Y54.578 E0.36545
G1 X159.737 Y54.578 E0.01827
G1 X151.392 Y46.233 E0.35921
```

```
G1 X150.900 Y46.340 E0.01534
G1 X159.137 Y54.578 E0.35457
G1 X158.537 Y54.578 E0.01827
G1 X150.394 Y46.435 E0.35050
G1 X149.830 Y46.471 E0.01722
G1 X157.936 Y54.578 E0.34897
G1 X157.336 Y54.578 E0.01827
G1 X149.249 Y46.490 E0.34814
G1 X148.648 Y46.490 E0.01827
G1 X152.312 Y50.153 E0.15769
G1 X152.132 Y50.070 E0.00603
G1 X151.393 Y49.835 E0.02358
G1 X148.048 Y46.490 E0.14400
G1 X147.448 Y46.490 E0.01827
G1 X150.636 Y49.678 E0.13722
G1 X150.388 Y49.631 E0.00767
G1 X149.963 Y49.605 E0.01297
G1 X146.848 Y46.490 E0.13409
G1 X146.247 Y46.490 E0.01827
G1 X149.335 Y49.578 E0.13291
G1 X148.735 Y49.578 E0.01827
G1 X145.647 Y46.490 E0.13291
G1 X145.047 Y46.490 E0.01827
G1 X148.135 Y49.578 E0.13291
G1 X147.534 Y49.578 E0.01827
G1 X144.447 Y46.490 E0.13291
G1 X143.847 Y46.490 E0.01827
G1 X146.934 Y49.578 E0.13291
G1 X146.334 Y49.578 E0.01827
```

```
G1 X143.246 Y46.490 E0.13291
G1 X142.646 Y46.490 E0.01827
G1 X145.734 Y49.578 E0.13291
G1 X145.134 Y49.578 E0.01827
G1 X142.046 Y46.490 E0.13291
G1 X141.650 Y46.490 E0.01205
G1 X141.650 Y46.694 E0.00622
G1 X144.533 Y49.578 E0.12412
G1 X143.933 Y49.578 E0.01827
G1 X141.650 Y47.294 E0.09828
G1 X141.650 Y47.895 E0.01827
G1 X143.333 Y49.578 E0.07244
G1 X142.733 Y49.578 E0.01827
G1 X141.650 Y48.495 E0.04660
G1 X141.650 Y49.095 E0.01827
G1 X142.321 Y49.766 E0.02886
M204 S1250
; stop printing object Petg print.STL id:28 copy 0
G1 E-4.00000 F2400.000
; Filament-specific end gcode
M600
T1
M900 K0.2 ; Filament gcode
; printing object tpu print.STL id:19 copy 0
G1 Z0.700 F9000.000
G1 X41.611 Y49.135
G1 Z0.500
G1 E4.00000 F1500.000
M204 S800
```

```
;TYPE:Perimeter
;WIDTH:0.45
G1 F2400.000
G1 X34.439 Y49.135 E0.18688
G1 X33.599 Y49.187 E0.02194
G1 X32.752 Y49.345 E0.02246
G1 X31.930 Y49.607 E0.02246
G1 X31.147 Y49.968 E0.02246
G1 X30.415 Y50.422 E0.02246
G1 X29.743 Y50.963 E0.02246
G1 X29.144 Y51.582 E0.02246
G1 X28.625 Y52.270 E0.02246
G1 X28.195 Y53.017 E0.02246
G1 X27.860 Y53.812 E0.02246
G1 X27.768 Y54.135 E0.00876
G1 X20.897 Y54.135 E0.17903
G1 X20.897 Y40.421 E0.35734
G1 X27.768 Y40.421 E0.17903
G1 X27.860 Y40.744 E0.00876
G1 X28.195 Y41.538 E0.02246
G1 X28.625 Y42.285 E0.02246
G1 X29.144 Y42.974 E0.02246
G1 X29.743 Y43.593 E0.02246
G1 X30.415 Y44.134 E0.02246
G1 X31.147 Y44.588 E0.02246
G1 X31.930 Y44.949 E0.02246
G1 X32.752 Y45.210 E0.02246
G1 X33.599 Y45.369 E0.02246
G1 X34.439 Y45.421 E0.02194
```

```
G1 X41.611 Y45.421 E0.18688
G1 X41.611 Y49.075 E0.09522
M204 S1250
G1 X42.029 Y49.553 F9000.000
M204 S800
;TYPE:External perimeter
G1 F1800.000
G1 X34.452 Y49.553 E0.19743
G1 X33.650 Y49.602 E0.02093
G1 X32.854 Y49.751 E0.02112
G1 X32.082 Y49.997 E0.02112
G1 X31.345 Y50.336 E0.02112
G1 X30.657 Y50.763 E0.02112
G1 X30.026 Y51.272 E0.02112
G1 X29.462 Y51.854 E0.02112
G1 X28.974 Y52.501 E0.02112
G1 X28.570 Y53.203 E0.02112
G1 X28.255 Y53.950 E0.02112
G1 X28.084 Y54.553 E0.01632
G1 X20.479 Y54.553 E0.19815
G1 X20.479 Y40.003 E0.37911
G1 X28.084 Y40.003 E0.19815
G1 X28.255 Y40.606 E0.01632
G1 X28.570 Y41.352 E0.02112
G1 X28.974 Y42.054 E0.02112
G1 X29.462 Y42.702 E0.02112
G1 X30.026 Y43.284 E0.02112
G1 X30.657 Y43.792 E0.02112
G1 X31.345 Y44.219 E0.02112
```

```
G1 X32.082 Y44.558 E0.02112
G1 X32.854 Y44.804 E0.02112
G1 X33.650 Y44.953 E0.02112
G1 X34.452 Y45.003 E0.02093
G1 X42.029 Y45.003 E0.19743
G1 X42.029 Y49.493 E0.11699
M204 S1250
G1 X41.636 Y49.481 F9000.000
G1 E-2.24000 F2400.000
;WIPE_START
G1 F7200.000
G1 X39.149 Y49.516 E-0.91200
;WIPE_END
G1 E-0.04800 F2400.000
G1 Z0.700 F9000.000
G1 X41.486 Y46.392
G1 Z0.500
G1 E3.20000 F1500.000
M204 S1000
;TYPE:Solid infill
;WIDTH:0.450839
G1 F4800.000
G1 X40.828 Y45.734 E0.02431
G1 X40.236 Y45.734 E0.01546
G1 X41.298 Y46.796 E0.03923
G1 X41.298 Y47.388 E0.01546
G1 X39.644 Y45.734 E0.06109
G1 X39.052 Y45.734 E0.01546
G1 X41.298 Y47.981 E0.08295
```

```
G1 X41.298 Y48.573 E0.01546
G1 X38.460 Y45.734 E0.10481
G1 X37.867 Y45.734 E0.01546
G1 X40.955 Y48.822 E0.11400
G1 X40.363 Y48.822 E0.01546
G1 X37.275 Y45.734 E0.11400
G1 X36.683 Y45.734 E0.01546
G1 X39.771 Y48.822 E0.11400
G1 X39.179 Y48.822 E0.01546
G1 X36.091 Y45.734 E0.11400
G1 X35.499 Y45.734 E0.01546
G1 X38.587 Y48.822 E0.11400
G1 X37.995 Y48.822 E0.01546
G1 X34.907 Y45.734 E0.11400
G1 X34.308 Y45.726 E0.01566
G1 X37.403 Y48.822 E0.11428
G1 X36.811 Y48.822 E0.01546
G1 X33.677 Y45.688 E0.11572
G1 X32.966 Y45.569 E0.01880
G1 X36.219 Y48.822 E0.12009
G1 X35.627 Y48.822 E0.01546
G1 X32.154 Y45.349 E0.12823
G1 X31.817 Y45.241 E0.00923
G1 X31.145 Y44.932 E0.01931
G1 X35.035 Y48.822 E0.14362
G1 X34.443 Y48.822 E0.01546
G1 X26.355 Y40.734 E0.29862
G1 X26.947 Y40.734 E0.01546
G1 X28.456 Y42.243 E0.05573
```

```
M204 S1250
G1 E-2.24000 F2400.000
;WIPE_START
G1 F7200.000
G1 X26.947 Y40.734 E-0.67595
G1 X26.355 Y40.734 E-0.18749
G1 X26.463 Y40.842 E-0.04857
;WIPE_END
G1 E-0.04800 F2400.000
G1 Z0.700 F9000.000
G1 X34.062 Y49.033
G1 Z0.500
G1 E3.20000 F1500.000
M204 S1000
G1 F4800.000
G1 X25.763 Y40.734 E0.30641
G1 X25.171 Y40.734 E0.01546
G1 X33.351 Y48.914 E0.30204
G1 X32.852 Y49.008 E0.01325
G1 X24.579 Y40.734 E0.30549
G1 X23.987 Y40.734 E0.01546
G1 X32.386 Y49.133 E0.31012
G1 X31.937 Y49.276 E0.01230
G1 X23.395 Y40.734 E0.31540
G1 X22.802 Y40.734 E0.01546
G1 X31.520 Y49.451 E0.32186
G1 X31.114 Y49.638 E0.01165
G1 X22.210 Y40.734 E0.32875
G1 X21.618 Y40.734 E0.01546
```

```
G1 X30.737 Y49.853 E0.33670
G1 X30.372 Y50.080 E0.01123
G1 X21.210 Y40.918 E0.33826
G1 X21.210 Y41.510 E0.01546
G1 X30.030 Y50.330 E0.32563
G1 X29.702 Y50.594 E0.01099
G1 X21.210 Y42.102 E0.31353
G1 X21.210 Y42.694 E0.01546
G1 X29.392 Y50.876 E0.30207
G1 X29.100 Y51.176 E0.01093
G1 X21.210 Y43.286 E0.29132
G1 X21.210 Y43.878 E0.01546
G1 X28.821 Y51.489 E0.28101
G1 X28.567 Y51.827 E0.01104
G1 X21.210 Y44.471 E0.27162
G1 X21.210 Y45.063 E0.01546
G1 X28.320 Y52.172 E0.26250
G1 X28.104 Y52.548 E0.01132
G1 X21.210 Y45.655 E0.25451
G1 X21.210 Y46.247 E0.01546
G1 X27.892 Y52.928 E0.24671
G1 X27.717 Y53.345 E0.01180
G1 X21.210 Y46.839 E0.24022
G1 X21.210 Y47.431 E0.01546
G1 X27.547 Y53.767 E0.23395
G1 X27.531 Y53.822 E0.00148
G1 X27.009 Y53.822 E0.01363
G1 X21.210 Y48.023 E0.21411
G1 X21.210 Y48.615 E0.01546
```

G1 X26.417 Y53.822 E0.19225

G1 X25.825 Y53.822 E0.01546

G1 X21.210 Y49.207 E0.17039

G1 X21.210 Y49.799 E0.01546

G1 X25.233 Y53.822 E0.14853

G1 X24.641 Y53.822 E0.01546

G1 X21.210 Y50.391 E0.12666

G1 X21.210 Y50.983 E0.01546

G1 X24.049 Y53.822 E0.10480

G1 X23.457 Y53.822 E0.01546

G1 X21.210 Y51.575 E0.08294

G1 X21.210 Y52.167 E0.01546

G1 X22.865 Y53.822 E0.06108

G1 X22.273 Y53.822 E0.01546

G1 X21.210 Y52.759 E0.03922

G1 X21.210 Y53.351 E0.01546

G1 X21.869 Y54.010 E0.02431

M204 S1250

; stop printing object tpu print.STL id:19 copy 0

; printing object Petg print.STL id:18 copy 0

; stop printing object Petg print.STL id:18 copy 0

; printing object Petg print.STL id:14 copy 0

; stop printing object Petg print.STL id:14 copy 0

; printing object tpu print.STL id:15 copy 0

G1 E-2.24000 F2400.000

;WIPE_START

G1 F7200.000

G1 X21.210 Y53.351 E-0.29480

G1 X21.210 Y52.759 E-0.18749

```
G1 X22.170 Y53.719 E-0.42971
;WIPE_END
G1 E-0.04800 F2400.000
G1 Z0.700 F9000.000
G1 X20.905 Y60.370
G1 Z0.500
G1 E3.20000 F1500.000
M204 S800
;TYPE:Perimeter
;WIDTH:0.45
G1 F2400.000
G1 X27.776 Y60.370 E0.17903
G1 X27.867 Y60.693 E0.00876
G1 X28.202 Y61.487 E0.02246
G1 X28.633 Y62.234 E0.02246
G1 X29.151 Y62.923 E0.02246
G1 X29.751 Y63.542 E0.02246
G1 X30.422 Y64.083 E0.02246
G1 X31.155 Y64.537 E0.02246
G1 X31.938 Y64.898 E0.02246
G1 X32.759 Y65.159 E0.02246
G1 X33.606 Y65.318 E0.02246
G1 X34.447 Y65.370 E0.02194
G1 X41.619 Y65.370 E0.18688
G1 X41.619 Y69.084 E0.09678
G1 X34.447 Y69.084 E0.18688
G1 X33.606 Y69.136 E0.02194
G1 X32.759 Y69.294 E0.02246
G1 X31.938 Y69.556 E0.02246
```

```
G1 X31.155 Y69.917 E0.02246
G1 X30.422 Y70.371 E0.02246
G1 X29.751 Y70.912 E0.02246
G1 X29.151 Y71.531 E0.02246
G1 X28.633 Y72.219 E0.02246
G1 X28.202 Y72.966 E0.02246
G1 X27.867 Y73.760 E0.02246
G1 X27.776 Y74.084 E0.00876
G1 X20.905 Y74.084 E0.17903
G1 X20.905 Y60.430 E0.35578
M204 S1250
G1 X20.487 Y59.952 F9000.000
M204 S800
;TYPE:External perimeter
G1 F1800.000
G1 X28.092 Y59.952 E0.19815
G1 X28.262 Y60.554 E0.01632
G1 X28.577 Y61.301 E0.02112
G1 X28.982 Y62.003 E0.02112
G1 X29.469 Y62.651 E0.02112
G1 X30.033 Y63.233 E0.02112
G1 X30.664 Y63.741 E0.02112
G1 X31.353 Y64.168 E0.02112
G1 X32.089 Y64.507 E0.02112
G1 X32.861 Y64.753 E0.02112
G1 X33.658 Y64.902 E0.02112
G1 X34.460 Y64.952 E0.02093
G1 X42.037 Y64.952 E0.19743
G1 X42.037 Y69.502 E0.11855
```

```
G1 X34.460 Y69.502 E0.19743
G1 X33.658 Y69.551 E0.02093
G1 X32.861 Y69.700 E0.02112
G1 X32.089 Y69.946 E0.02112
G1 X31.353 Y70.285 E0.02112
G1 X30.664 Y70.712 E0.02112
G1 X30.033 Y71.221 E0.02112
G1 X29.469 Y71.803 E0.02112
G1 X28.982 Y72.450 E0.02112
G1 X28.577 Y73.152 E0.02112
G1 X28.262 Y73.899 E0.02112
G1 X28.092 Y74.502 E0.01632
G1 X20.487 Y74.502 E0.19815
G1 X20.487 Y60.012 E0.37755
M204 S1250
G1 X20.861 Y60.094 F9000.000
G1 E-2.24000 F2400.000
;WIPE_START
G1 F7200.000
G1 X23.367 Y59.989 E-0.91200
;WIPE_END
G1 E-0.04800 F2400.000
G1 Z0.700 F9000.000
G1 X28.464 Y62.192
G1 Z0.500
G1 E3.20000 F1500.000
M204 S1000
;TYPE:Solid infill
;WIDTH:0.450839
```

```
G1 F4800.000
G1 X26.954 Y60.683 E0.05573
G1 X26.362 Y60.683 E0.01546
G1 X34.450 Y68.771 E0.29862
G1 X35.042 Y68.771 E0.01546
G1 X31.152 Y64.881 E0.14362
G1 X31.824 Y65.190 E0.01931
G1 X32.161 Y65.298 E0.00923
G1 X35.634 Y68.771 E0.12823
G1 X36.226 Y68.771 E0.01546
G1 X32.974 Y65.518 E0.12009
G1 X33.684 Y65.636 E0.01880
G1 X36.818 Y68.771 E0.11572
G1 X37.410 Y68.771 E0.01546
G1 X34.315 Y65.675 E0.11428
G1 X34.915 Y65.683 E0.01566
G1 X38.002 Y68.771 E0.11400
G1 X38.594 Y68.771 E0.01546
G1 X35.507 Y65.683 E0.11400
G1 X36.099 Y65.683 E0.01546
G1 X39.186 Y68.771 E0.11400
G1 X39.779 Y68.771 E0.01546
G1 X36.691 Y65.683 E0.11400
G1 X37.283 Y65.683 E0.01546
G1 X40.371 Y68.771 E0.11400
G1 X40.963 Y68.771 E0.01546
G1 X37.875 Y65.683 E0.11400
G1 X38.467 Y65.683 E0.01546
G1 X41.306 Y68.522 E0.10481
```

```
G1 X41.306 Y67.929 E0.01546
G1 X39.059 Y65.683 E0.08295
G1 X39.651 Y65.683 E0.01546
G1 X41.306 Y67.337 E0.06109
G1 X41.306 Y66.745 E0.01546
G1 X40.243 Y65.683 E0.03923
G1 X40.835 Y65.683 E0.01546
G1 X41.494 Y66.341 E0.02431
M204 S1250
G1 E-2.24000 F2400.000
;WIPE_START
G1 F7200.000
G1 X40.835 Y65.683 E-0.29484
G1 X40.243 Y65.683 E-0.18749
G1 X41.203 Y66.642 E-0.42968
;WIPE_END
G1 E-0.04800 F2400.000
G1 Z0.700 F9000.000
G1 X34.069 Y68.982
G1 Z0.500
G1 E3.20000 F1500.000
M204 S1000
G1 F4800.000
G1 X25.770 Y60.683 E0.30641
G1 X25.178 Y60.683 E0.01546
G1 X33.359 Y68.863 E0.30204
G1 X32.860 Y68.957 E0.01325
G1 X24.586 Y60.683 E0.30549
G1 X23.994 Y60.683 E0.01546
```

```
G1 X32.393 Y69.082 E0.31012
G1 X31.944 Y69.225 E0.01230
G1 X23.402 Y60.683 E0.31540
G1 X22.810 Y60.683 E0.01546
G1 X31.527 Y69.400 E0.32186
M73 P49 R35
G1 X31.122 Y69.587 E0.01165
G1 X22.218 Y60.683 E0.32875
G1 X21.626 Y60.683 E0.01546
G1 X30.745 Y69.802 E0.33670
G1 X30.379 Y70.029 E0.01123
G1 X21.218 Y60.867 E0.33826
G1 X21.218 Y61.459 E0.01546
G1 X30.037 Y70.278 E0.32563
G1 X29.709 Y70.543 E0.01099
G1 X21.218 Y62.051 E0.31353
G1 X21.218 Y62.643 E0.01546
G1 X29.399 Y70.824 E0.30207
G1 X29.108 Y71.125 E0.01093
G1 X21.218 Y63.235 E0.29132
G1 X21.218 Y63.827 E0.01546
G1 X28.829 Y71.438 E0.28101
G1 X28.574 Y71.776 E0.01104
G1 X21.218 Y64.419 E0.27162
G1 X21.218 Y65.012 E0.01546
G1 X28.328 Y72.121 E0.26250
G1 X28.111 Y72.497 E0.01132
G1 X21.218 Y65.604 E0.25451
G1 X21.218 Y66.196 E0.01546
```

```
G1 X27.900 Y72.877 E0.24671
G1 X27.724 Y73.294 E0.01180
G1 X21.218 Y66.788 E0.24022
G1 X21.218 Y67.380 E0.01546
G1 X27.554 Y73.716 E0.23395
G1 X27.539 Y73.771 E0.00148
G1 X27.017 Y73.771 E0.01363
G1 X21.218 Y67.972 E0.21411
G1 X21.218 Y68.564 E0.01546
G1 X26.425 Y73.771 E0.19225
G1 X25.833 Y73.771 E0.01546
G1 X21.218 Y69.156 E0.17039
G1 X21.218 Y69.748 E0.01546
G1 X25.241 Y73.771 E0.14853
G1 X24.649 Y73.771 E0.01546
G1 X21.218 Y70.340 E0.12666
G1 X21.218 Y70.932 E0.01546
G1 X24.056 Y73.771 E0.10480
G1 X23.464 Y73.771 E0.01546
G1 X21.218 Y71.524 E0.08294
G1 X21.218 Y72.116 E0.01546
G1 X22.872 Y73.771 E0.06108
G1 X22.280 Y73.771 E0.01546
G1 X21.218 Y72.708 E0.03922
G1 X21.218 Y73.300 E0.01546
G1 X21.876 Y73.959 E0.02431
M204 S1250
; stop printing object tpu print.STL id:15 copy 0
; printing object Petg print.STL id:10 copy 0
```

```
; stop printing object Petg print.STL id:10 copy 0
; printing object tpu print.STL id:11 copy 0
G1 E-2.24000 F2400.000
;WIPE_START
G1 F7200.000
G1 X21.218 Y73.300 E-0.29480
G1 X21.218 Y72.708 E-0.18749
G1 X22.177 Y73.668 E-0.42971
;WIPE_END
G1 E-0.04800 F2400.000
G1 Z0.700 F9000.000
G1 X20.853 Y82.562
G1 Z0.500
G1 E3.20000 F1500.000
M204 S800
;TYPE:Perimeter
;WIDTH:0.45
G1 F2400.000
G1 X27.725 Y82.562 E0.17903
G1 X27.816 Y82.885 E0.00876
G1 X28.151 Y83.680 E0.02246
G1 X28.581 Y84.427 E0.02246
G1 X29.100 Y85.115 E0.02246
G1 X29.700 Y85.734 E0.02246
G1 X30.371 Y86.275 E0.02246
G1 X31.104 Y86.729 E0.02246
G1 X31.887 Y87.090 E0.02246
G1 X32.708 Y87.351 E0.02246
G1 X33.555 Y87.510 E0.02246
```

```
G1 X34.396 Y87.562 E0.02194
G1 X41.568 Y87.562 E0.18688
G1 X41.568 Y91.276 E0.09678
G1 X34.396 Y91.276 E0.18688
G1 X33.555 Y91.328 E0.02194
G1 X32.708 Y91.487 E0.02246
G1 X31.887 Y91.748 E0.02246
G1 X31.104 Y92.109 E0.02246
G1 X30.371 Y92.563 E0.02246
G1 X29.700 Y93.104 E0.02246
G1 X29.100 Y93.723 E0.02246
G1 X28.581 Y94.412 E0.02246
G1 X28.151 Y95.159 E0.02246
G1 X27.816 Y95.953 E0.02246
G1 X27.725 Y96.276 E0.00876
G1 X20.853 Y96.276 E0.17903
G1 X20.853 Y82.622 E0.35578
M204 S1250
G1 X20.436 Y82.144 F9000.000
M204 S800
;TYPE:External perimeter
G1 F1800.000
G1 X28.040 Y82.144 E0.19815
G1 X28.211 Y82.747 E0.01632
G1 X28.526 Y83.493 E0.02112
G1 X28.931 Y84.196 E0.02112
G1 X29.418 Y84.843 E0.02112
G1 X29.982 Y85.425 E0.02112
G1 X30.613 Y85.934 E0.02112
```

```
G1 X31.302 Y86.361 E0.02112
G1 X32.038 Y86.700 E0.02112
G1 X32.810 Y86.946 E0.02112
G1 X33.607 Y87.095 E0.02112
G1 X34.409 Y87.144 E0.02093
G1 X41.986 Y87.144 E0.19743
G1 X41.986 Y91.694 E0.11855
G1 X34.409 Y91.694 E0.19743
G1 X33.607 Y91.744 E0.02093
G1 X32.810 Y91.893 E0.02112
G1 X32.038 Y92.139 E0.02112
G1 X31.302 Y92.478 E0.02112
G1 X30.613 Y92.905 E0.02112
G1 X29.982 Y93.413 E0.02112
G1 X29.418 Y93.995 E0.02112
G1 X28.931 Y94.643 E0.02112
G1 X28.526 Y95.345 E0.02112
G1 X28.211 Y96.091 E0.02112
G1 X28.040 Y96.694 E0.01632
G1 X20.436 Y96.694 E0.19815
G1 X20.436 Y82.204 E0.37755
M204 S1250
G1 X20.810 Y82.286 F9000.000
G1 E-2.24000 F2400.000
;WIPE_START
G1 F7200.000
G1 X23.316 Y82.181 E-0.91200
;WIPE_END
G1 E-0.04800 F2400.000
```

```
G1 Z0.700 F9000.000
G1 X28.413 Y84.385
G1 Z0.500
G1 E3.20000 F1500.000
M204 S1000
;TYPE:Solid infill
;WIDTH:0.450839
G1 F4800.000
G1 X26.903 Y82.875 E0.05573
G1 X26.311 Y82.875 E0.01546
G1 X34.399 Y90.963 E0.29862
G1 X34.991 Y90.963 E0.01546
G1 X31.101 Y87.073 E0.14362
G1 X31.773 Y87.383 E0.01931
G1 X32.110 Y87.490 E0.00923
G1 X35.583 Y90.963 E0.12823
G1 X36.175 Y90.963 E0.01546
G1 X32.923 Y87.710 E0.12009
G1 X33.633 Y87.829 E0.01880
G1 X36.767 Y90.963 E0.11572
G1 X37.359 Y90.963 E0.01546
G1 X34.264 Y87.868 E0.11428
G1 X34.864 Y87.875 E0.01566
G1 X37.951 Y90.963 E0.11400
G1 X38.543 Y90.963 E0.01546
G1 X35.456 Y87.875 E0.11400
G1 X36.048 Y87.875 E0.01546
G1 X39.135 Y90.963 E0.11400
G1 X39.727 Y90.963 E0.01546
```

```
G1 X36.640 Y87.875 E0.11400
G1 X37.232 Y87.875 E0.01546
G1 X40.319 Y90.963 E0.11400
G1 X40.911 Y90.963 E0.01546
G1 X37.824 Y87.875 E0.11400
G1 X38.416 Y87.875 E0.01546
G1 X41.254 Y90.714 E0.10481
G1 X41.254 Y90.122 E0.01546
G1 X39.008 Y87.875 E0.08295
G1 X39.600 Y87.875 E0.01546
G1 X41.254 Y89.530 E0.06109
G1 X41.254 Y88.938 E0.01546
G1 X40.192 Y87.875 E0.03923
G1 X40.784 Y87.875 E0.01546
G1 X41.442 Y88.534 E0.02431
M204 S1250
G1 E-2.24000 F2400.000
;WIPE_START
G1 F7200.000
G1 X40.784 Y87.875 E-0.29484
G1 X40.192 Y87.875 E-0.18749
G1 X41.152 Y88.835 E-0.42968
;WIPE_END
G1 E-0.04800 F2400.000
G1 Z0.700 F9000.000
G1 X34.018 Y91.174
G1 Z0.500
G1 E3.20000 F1500.000
M204 S1000
```

```
G1 F4800.000
G1 X25.719 Y82.875 E0.30641
G1 X25.127 Y82.875 E0.01546
G1 X33.308 Y91.056 E0.30204
G1 X32.809 Y91.149 E0.01325
G1 X24.535 Y82.875 E0.30549
G1 X23.943 Y82.875 E0.01546
G1 X32.342 Y91.274 E0.31012
G1 X31.893 Y91.417 E0.01230
G1 X23.351 Y82.875 E0.31540
G1 X22.759 Y82.875 E0.01546
G1 X31.476 Y91.592 E0.32186
G1 X31.071 Y91.779 E0.01165
G1 X22.167 Y82.875 E0.32875
G1 X21.575 Y82.875 E0.01546
G1 X30.694 Y91.994 E0.33670
G1 X30.328 Y92.221 E0.01123
G1 X21.167 Y83.059 E0.33826
G1 X21.167 Y83.652 E0.01546
G1 X29.986 Y92.471 E0.32563
G1 X29.658 Y92.735 E0.01099
G1 X21.167 Y84.244 E0.31353
G1 X21.167 Y84.836 E0.01546
G1 X29.348 Y93.017 E0.30207
G1 X29.057 Y93.318 E0.01093
G1 X21.167 Y85.428 E0.29132
G1 X21.167 Y86.020 E0.01546
G1 X28.778 Y93.631 E0.28101
G1 X28.523 Y93.968 E0.01104
```

```
G1 X21.167 Y86.612 E0.27162
G1 X21.167 Y87.204 E0.01546
G1 X28.276 Y94.313 E0.26250
G1 X28.060 Y94.689 E0.01132
G1 X21.167 Y87.796 E0.25451
G1 X21.167 Y88.388 E0.01546
G1 X27.849 Y95.070 E0.24671
G1 X27.673 Y95.486 E0.01180
G1 X21.167 Y88.980 E0.24022
G1 X21.167 Y89.572 E0.01546
G1 X27.503 Y95.908 E0.23395
G1 X27.488 Y95.963 E0.00148
G1 X26.966 Y95.963 E0.01363
G1 X21.167 Y90.164 E0.21411
G1 X21.167 Y90.756 E0.01546
G1 X26.374 Y95.963 E0.19225
G1 X25.781 Y95.963 E0.01546
G1 X21.167 Y91.348 E0.17039
G1 X21.167 Y91.940 E0.01546
G1 X25.189 Y95.963 E0.14853
M73 P49 R34
G1 X24.597 Y95.963 E0.01546
G1 X21.167 Y92.532 E0.12666
G1 X21.167 Y93.124 E0.01546
G1 X24.005 Y95.963 E0.10480
G1 X23.413 Y95.963 E0.01546
G1 X21.167 Y93.717 E0.08294
G1 X21.167 Y94.309 E0.01546
G1 X22.821 Y95.963 E0.06108
```

```
G1 X22.229 Y95.963 E0.01546
G1 X21.167 Y94.901 E0.03922
G1 X21.167 Y95.493 E0.01546
G1 X21.825 Y96.151 E0.02431
M204 S1250
; stop printing object tpu print.STL id:11 copy 0
; printing object tpu print.STL id:13 copy 0
G1 E-2.24000 F2400.000
;WIPE_START
G1 F7200.000
G1 X21.167 Y95.493 E-0.29480
G1 X21.167 Y94.901 E-0.18749
G1 X22.126 Y95.860 E-0.42971
;WIPE_END
G1 E-0.04800 F2400.000
G1 Z0.700 F9000.000
G1 X20.860 Y103.377
G1 Z0.500
G1 E3.20000 F1500.000
M204 S800
;TYPE:Perimeter
;WIDTH:0.45
G1 F2400.000
G1 X27.731 Y103.377 E0.17903
G1 X27.823 Y103.701 E0.00876
G1 X28.158 Y104.495 E0.02246
G1 X28.588 Y105.242 E0.02246
G1 X29.107 Y105.930 E0.02246
G1 X29.707 Y106.550 E0.02246
```

G1 X30.378 Y107.091 E0.02246

G1 X31.110 Y107.545 E0.02246

G1 X31.893 Y107.905 E0.02246

G1 X32.715 Y108.167 E0.02246

G1 X33.562 Y108.326 E0.02246

G1 X34.402 Y108.377 E0.02194

G1 X41.575 Y108.377 E0.18688

G1 X41.575 Y112.092 E0.09678

G1 X34.402 Y112.092 E0.18688

G1 X33.562 Y112.144 E0.02194

G1 X32.715 Y112.302 E0.02246

G1 X31.893 Y112.564 E0.02246

G1 X31.110 Y112.924 E0.02246

G1 X30.378 Y113.379 E0.02246

G1 X29.707 Y113.920 E0.02246

G1 X29.107 Y114.539 E0.02246

G1 X28.588 Y115.227 E0.02246

G1 X28.158 Y115.974 E0.02246

G1 X27.823 Y116.768 E0.02246

G1 X27.731 Y117.092 E0.00876

G1 X20.860 Y117.092 E0.17903

G1 X20.860 Y103.437 E0.35578

M204 S1250

G1 X20.442 Y102.960 F9000.000

M204 S800

;TYPE:External perimeter

G1 F1800.000

G1 X28.047 Y102.960 E0.19815

G1 X28.218 Y103.562 E0.01632

```
G1 X28.533 Y104.309 E0.02112
G1 X28.937 Y105.011 E0.02112
G1 X29.425 Y105.658 E0.02112
G1 X29.989 Y106.241 E0.02112
G1 X30.620 Y106.749 E0.02112
G1 X31.309 Y107.176 E0.02112
G1 X32.045 Y107.515 E0.02112
G1 X32.817 Y107.761 E0.02112
G1 X33.613 Y107.910 E0.02112
G1 X34.415 Y107.960 E0.02093
G1 X41.992 Y107.960 E0.19743
G1 X41.992 Y112.510 E0.11855
G1 X34.415 Y112.510 E0.19743
G1 X33.613 Y112.559 E0.02093
G1 X32.817 Y112.708 E0.02112
G1 X32.045 Y112.954 E0.02112
G1 X31.309 Y113.293 E0.02112
G1 X30.620 Y113.720 E0.02112
G1 X29.989 Y114.229 E0.02112
G1 X29.425 Y114.811 E0.02112
G1 X28.937 Y115.458 E0.02112
G1 X28.533 Y116.160 E0.02112
G1 X28.218 Y116.907 E0.02112
G1 X28.047 Y117.510 E0.01632
G1 X20.442 Y117.510 E0.19815
G1 X20.442 Y103.020 E0.37755
M204 S1250
G1 X20.816 Y103.102 F9000.000
G1 E-2.24000 F2400.000
```

```
;WIPE_START
G1 F7200.000
G1 X23.322 Y102.997 E-0.91200
;WIPE_END
G1 E-0.04800 F2400.000
G1 Z0.700 F9000.000
G1 X28.419 Y105.200
G1 Z0.500
G1 E3.20000 F1500.000
M204 S1000
;TYPE:Solid infill
;WIDTH:0.450839
G1 F4800.000
G1 X26.910 Y103.691 E0.05573
G1 X26.318 Y103.691 E0.01546
G1 X34.406 Y111.778 E0.29862
G1 X34.998 Y111.778 E0.01546
G1 X31.108 Y107.889 E0.14362
G1 X31.780 Y108.198 E0.01931
G1 X32.117 Y108.305 E0.00923
G1 X35.590 Y111.778 E0.12823
G1 X36.182 Y111.778 E0.01546
G1 X32.929 Y108.526 E0.12009
G1 X33.640 Y108.644 E0.01880
G1 X36.774 Y111.778 E0.11572
G1 X37.366 Y111.778 E0.01546
G1 X34.271 Y108.683 E0.11428
G1 X34.870 Y108.691 E0.01566
G1 X37.958 Y111.778 E0.11400
```

```
G1 X38.550 Y111.778 E0.01546
G1 X35.462 Y108.691 E0.11400
G1 X36.054 Y108.691 E0.01546
G1 X39.142 Y111.778 E0.11400
G1 X39.734 Y111.778 E0.01546
G1 X36.647 Y108.691 E0.11400
G1 X37.239 Y108.691 E0.01546
G1 X40.326 Y111.778 E0.11400
G1 X40.918 Y111.778 E0.01546
G1 X37.831 Y108.691 E0.11400
G1 X38.423 Y108.691 E0.01546
G1 X41.261 Y111.529 E0.10481
G1 X41.261 Y110.937 E0.01546
G1 X39.015 Y108.691 E0.08295
G1 X39.607 Y108.691 E0.01546
G1 X41.261 Y110.345 E0.06109
G1 X41.261 Y109.753 E0.01546
G1 X40.199 Y108.691 E0.03923
G1 X40.791 Y108.691 E0.01546
G1 X41.449 Y109.349 E0.02431
M204 S1250
G1 E-2.24000 F2400.000
;WIPE_START
G1 F7200.000
G1 X40.791 Y108.691 E-0.29484
G1 X40.199 Y108.691 E-0.18749
G1 X41.158 Y109.650 E-0.42968
;WIPE_END
G1 E-0.04800 F2400.000
```

```
G1 Z0.700 F9000.000
G1 X34.025 Y111.990
G1 Z0.500
G1 E3.20000 F1500.000
M204 S1000
G1 F4800.000
G1 X25.726 Y103.691 E0.30641
G1 X25.134 Y103.691 E0.01546
G1 X33.314 Y111.871 E0.30204
G1 X32.816 Y111.965 E0.01325
G1 X24.542 Y103.691 E0.30549
G1 X23.950 Y103.691 E0.01546
G1 X32.349 Y112.090 E0.31012
G1 X31.900 Y112.233 E0.01230
G1 X23.358 Y103.691 E0.31540
G1 X22.766 Y103.691 E0.01546
G1 X31.483 Y112.408 E0.32186
G1 X31.077 Y112.595 E0.01165
G1 X22.174 Y103.691 E0.32875
G1 X21.582 Y103.691 E0.01546
G1 X30.701 Y112.810 E0.33670
G1 X30.335 Y113.036 E0.01123
G1 X21.174 Y103.875 E0.33826
G1 X21.174 Y104.467 E0.01546
G1 X29.993 Y113.286 E0.32563
G1 X29.665 Y113.551 E0.01099
G1 X21.174 Y105.059 E0.31353
G1 X21.174 Y105.651 E0.01546
G1 X29.355 Y113.832 E0.30207
```

G1 X29.064 Y114.133 E0.01093

G1 X21.174 Y106.243 E0.29132

G1 X21.174 Y106.835 E0.01546

G1 X28.785 Y114.446 E0.28101

G1 X28.530 Y114.784 E0.01104

G1 X21.174 Y107.427 E0.27162

G1 X21.174 Y108.019 E0.01546

G1 X28.283 Y115.129 E0.26250

G1 X28.067 Y115.505 E0.01132

G1 X21.174 Y108.611 E0.25451

G1 X21.174 Y109.204 E0.01546

G1 X27.855 Y115.885 E0.24671

G1 X27.680 Y116.302 E0.01180

G1 X21.174 Y109.796 E0.24022

G1 X21.174 Y110.388 E0.01546

G1 X27.510 Y116.724 E0.23395

G1 X27.494 Y116.778 E0.00148

G1 X26.972 Y116.778 E0.01363

G1 X21.174 Y110.980 E0.21411

G1 X21.174 Y111.572 E0.01546

G1 X26.380 Y116.778 E0.19225

G1 X25.788 Y116.778 E0.01546

G1 X21.174 Y112.164 E0.17039

G1 X21.174 Y112.756 E0.01546

G1 X25.196 Y116.778 E0.14853

G1 X24.604 Y116.778 E0.01546

G1 X21.174 Y113.348 E0.12666

G1 X21.174 Y113.940 E0.01546

G1 X24.012 Y116.778 E0.10480

```
G1 X23.420 Y116.778 E0.01546
G1 X21.174 Y114.532 E0.08294
G1 X21.174 Y115.124 E0.01546
G1 X22.828 Y116.778 E0.06108
G1 X22.236 Y116.778 E0.01546
G1 X21.174 Y115.716 E0.03922
G1 X21.174 Y116.308 E0.01546
G1 X21.832 Y116.967 E0.02431
M204 S1250
; stop printing object tpu print.STL id:13 copy 0
; printing object tpu print.STL id:17 copy 0
G1 E-2.24000 F2400.000
;WIPE_START
G1 F7200.000
G1 X21.174 Y116.308 E-0.29480
G1 X21.174 Y115.716 E-0.18749
G1 X22.133 Y116.676 E-0.42971
;WIPE_END
G1 E-0.04800 F2400.000
G1 Z0.700 F9000.000
G1 X20.845 Y122.031
G1 Z0.500
G1 E3.20000 F1500.000
M204 S800
;TYPE:Perimeter
;WIDTH:0.45
G1 F2400.000
G1 X27.716 Y122.031 E0.17903
G1 X27.808 Y122.355 E0.00876
```

```
G1 X28.143 Y123.149 E0.02246
G1 X28.573 Y123.896 E0.02246
G1 X29.092 Y124.584 E0.02246
G1 X29.692 Y125.204 E0.02246
G1 X30.363 Y125.745 E0.02246
G1 X31.095 Y126.199 E0.02246
G1 X31.878 Y126.559 E0.02246
G1 X32.700 Y126.821 E0.02246
G1 X33.547 Y126.980 E0.02246
G1 X34.387 Y127.031 E0.02194
G1 X41.560 Y127.031 E0.18688
G1 X41.560 Y130.746 E0.09678
G1 X34.387 Y130.746 E0.18688
G1 X33.547 Y130.798 E0.02194
G1 X32.700 Y130.956 E0.02246
G1 X31.878 Y131.218 E0.02246
G1 X31.095 Y131.578 E0.02246
G1 X30.363 Y132.033 E0.02246
G1 X29.692 Y132.573 E0.02246
G1 X29.092 Y133.193 E0.02246
G1 X28.573 Y133.881 E0.02246
G1 X28.143 Y134.628 E0.02246
G1 X27.808 Y135.422 E0.02246
G1 X27.716 Y135.746 E0.00876
G1 X20.845 Y135.746 E0.17903
G1 X20.845 Y122.091 E0.35578
M204 S1250
G1 X20.427 Y121.614 F9000.000
M204 S800
```

```
;TYPE:External perimeter
G1 F1800.000
G1 X28.032 Y121.614 E0.19815
G1 X28.203 Y122.216 E0.01632
G1 X28.518 Y122.963 E0.02112
G1 X28.922 Y123.665 E0.02112
G1 X29.410 Y124.312 E0.02112
G1 X29.974 Y124.895 E0.02112
G1 X30.605 Y125.403 E0.02112
G1 X31.294 Y125.830 E0.02112
G1 X32.030 Y126.169 E0.02112
G1 X32.802 Y126.415 E0.02112
G1 X33.598 Y126.564 E0.02112
G1 X34.400 Y126.614 E0.02093
G1 X41.977 Y126.614 E0.19743
G1 X41.977 Y131.164 E0.11855
G1 X34.400 Y131.164 E0.19743
G1 X33.598 Y131.213 E0.02093
G1 X32.802 Y131.362 E0.02112
G1 X32.030 Y131.608 E0.02112
G1 X31.294 Y131.947 E0.02112
G1 X30.605 Y132.374 E0.02112
G1 X29.974 Y132.883 E0.02112
G1 X29.410 Y133.465 E0.02112
G1 X28.922 Y134.112 E0.02112
G1 X28.518 Y134.814 E0.02112
G1 X28.203 Y135.561 E0.02112
G1 X28.032 Y136.164 E0.01632
G1 X20.427 Y136.164 E0.19815
```

```
G1 X20.427 Y121.674 E0.37755
M204 S1250
G1 X20.801 Y121.756 F9000.000
G1 E-2.24000 F2400.000
;WIPE_START
G1 F7200.000
G1 X23.307 Y121.651 E-0.91200
;WIPE_END
G1 E-0.04800 F2400.000
G1 Z0.700 F9000.000
G1 X28.404 Y123.854
G1 Z0.500
G1 E3.20000 F1500.000
M204 S1000
;TYPE:Solid infill
;WIDTH:0.450839
G1 F4800.000
G1 X26.895 Y122.345 E0.05573
G1 X26.303 Y122.345 E0.01546
G1 X34.391 Y130.432 E0.29862
G1 X34.983 Y130.432 E0.01546
G1 X31.093 Y126.543 E0.14362
G1 X31.765 Y126.852 E0.01931
G1 X32.102 Y126.959 E0.00923
G1 X35.575 Y130.432 E0.12823
G1 X36.167 Y130.432 E0.01546
G1 X32.914 Y127.180 E0.12009
G1 X33.625 Y127.298 E0.01880
G1 X36.759 Y130.432 E0.11572
```

```
G1 X37.351 Y130.432 E0.01546
G1 X34.256 Y127.337 E0.11428
G1 X34.855 Y127.345 E0.01566
G1 X37.943 Y130.432 E0.11400
G1 X38.535 Y130.432 E0.01546
G1 X35.447 Y127.345 E0.11400
G1 X36.039 Y127.345 E0.01546
G1 X39.127 Y130.432 E0.11400
G1 X39.719 Y130.432 E0.01546
G1 X36.631 Y127.345 E0.11400
G1 X37.223 Y127.345 E0.01546
G1 X40.311 Y130.432 E0.11400
G1 X40.903 Y130.432 E0.01546
G1 X37.816 Y127.345 E0.11400
G1 X38.408 Y127.345 E0.01546
G1 X41.246 Y130.183 E0.10481
G1 X41.246 Y129.591 E0.01546
G1 X39.000 Y127.345 E0.08295
G1 X39.592 Y127.345 E0.01546
G1 X41.246 Y128.999 E0.06109
G1 X41.246 Y128.407 E0.01546
G1 X40.184 Y127.345 E0.03923
G1 X40.776 Y127.345 E0.01546
M73 P50 R34
G1 X41.434 Y128.003 E0.02431
M204 S1250
G1 E-2.24000 F2400.000
;WIPE_START
G1 F7200.000
```

```
G1 X40.776 Y127.345 E-0.29484
G1 X40.184 Y127.345 E-0.18749
G1 X41.143 Y128.304 E-0.42968
;WIPE_END
G1 E-0.04800 F2400.000
G1 Z0.700 F9000.000
G1 X34.010 Y130.644
G1 Z0.500
G1 E3.20000 F1500.000
M204 S1000
G1 F4800.000
G1 X25.711 Y122.345 E0.30641
G1 X25.119 Y122.345 E0.01546
G1 X33.299 Y130.525 E0.30204
G1 X32.801 Y130.619 E0.01325
G1 X24.527 Y122.345 E0.30549
G1 X23.935 Y122.345 E0.01546
G1 X32.334 Y130.744 E0.31012
G1 X31.885 Y130.887 E0.01230
G1 X23.343 Y122.345 E0.31540
G1 X22.751 Y122.345 E0.01546
G1 X31.468 Y131.062 E0.32186
G1 X31.062 Y131.249 E0.01165
G1 X22.158 Y122.345 E0.32875
G1 X21.566 Y122.345 E0.01546
G1 X30.685 Y131.464 E0.33670
G1 X30.320 Y131.690 E0.01123
G1 X21.159 Y122.529 E0.33826
G1 X21.159 Y123.121 E0.01546
```

```
G1 X29.978 Y131.940 E0.32563
G1 X29.650 Y132.204 E0.01099
G1 X21.159 Y123.713 E0.31353
G1 X21.159 Y124.305 E0.01546
G1 X29.340 Y132.486 E0.30207
G1 X29.049 Y132.787 E0.01093
G1 X21.159 Y124.897 E0.29132
G1 X21.159 Y125.489 E0.01546
G1 X28.769 Y133.100 E0.28101
G1 X28.515 Y133.438 E0.01104
G1 X21.159 Y126.081 E0.27162
G1 X21.159 Y126.673 E0.01546
G1 X28.268 Y133.783 E0.26250
G1 X28.052 Y134.159 E0.01132
G1 X21.159 Y127.265 E0.25451
G1 X21.159 Y127.857 E0.01546
G1 X27.840 Y134.539 E0.24671
G1 X27.665 Y134.956 E0.01180
G1 X21.159 Y128.450 E0.24022
G1 X21.159 Y129.042 E0.01546
G1 X27.495 Y135.378 E0.23395
G1 X27.479 Y135.432 E0.00148
G1 X26.957 Y135.432 E0.01363
G1 X21.159 Y129.634 E0.21411
G1 X21.159 Y130.226 E0.01546
G1 X26.365 Y135.432 E0.19225
G1 X25.773 Y135.432 E0.01546
G1 X21.159 Y130.818 E0.17039
G1 X21.159 Y131.410 E0.01546
```

G1 X25.181 Y135.432 E0.14853

G1 X24.589 Y135.432 E0.01546

G1 X21.159 Y132.002 E0.12666

G1 X21.159 Y132.594 E0.01546

G1 X23.997 Y135.432 E0.10480

G1 X23.405 Y135.432 E0.01546

G1 X21.159 Y133.186 E0.08294

G1 X21.159 Y133.778 E0.01546

G1 X22.813 Y135.432 E0.06108

G1 X22.221 Y135.432 E0.01546

G1 X21.159 Y134.370 E0.03922

G1 X21.159 Y134.962 E0.01546

G1 X21.817 Y135.620 E0.02431

M204 S1250

; stop printing object tpu print.STL id:17 copy 0

; printing object Petg print.STL id:16 copy 0

; stop printing object Petg print.STL id:16 copy 0

; printing object Petg print.STL id:12 copy 0

; stop printing object Petg print.STL id:12 copy 0

; printing object tpu print.STL id:7 copy 0

G1 E-2.24000 F2400.000

;WIPE_START

G1 F7200.000

G1 X21.159 Y134.962 E-0.29480

G1 X21.159 Y134.370 E-0.18749

G1 X22.118 Y135.330 E-0.42971

;WIPE_END

G1 E-0.04800 F2400.000

G1 Z0.700 F9000.000

```
G1 X69.635 Y136.327

G1 Z0.500

G1 E3.20000 F1500.000

M204 S800

;TYPE:Perimeter

;WIDTH:0.45

G1 F2400.000

G1 X69.635 Y122.612 E0.35734

G1 X76.506 Y122.612 E0.17903

G1 X76.597 Y122.936 E0.00876

G1 X76.932 Y123.730 E0.02246

G1 X77.362 Y124.477 E0.02246

G1 X77.881 Y125.165 E0.02246

G1 X78.481 Y125.785 E0.02246

G1 X79.152 Y126.325 E0.02246

G1 X79.885 Y126.780 E0.02246

G1 X80.668 Y127.140 E0.02246

G1 X81.489 Y127.402 E0.02246

G1 X82.336 Y127.560 E0.02246

G1 X83.177 Y127.612 E0.02194

G1 X90.349 Y127.612 E0.18688

G1 X90.349 Y131.327 E0.09678

G1 X83.177 Y131.327 E0.18688

G1 X82.336 Y131.379 E0.02194

G1 X81.489 Y131.537 E0.02246

G1 X80.668 Y131.799 E0.02246

G1 X79.885 Y132.159 E0.02246

G1 X79.152 Y132.613 E0.02246

G1 X78.481 Y133.154 E0.02246
```

```
G1 X77.881 Y133.774 E0.02246
G1 X77.362 Y134.462 E0.02246
G1 X76.932 Y135.209 E0.02246
G1 X76.597 Y136.003 E0.02246
G1 X76.506 Y136.327 E0.00876
G1 X69.695 Y136.327 E0.17747
M204 S1250
G1 X69.217 Y136.744 F9000.000
M204 S800
;TYPE:External perimeter
G1 F1800.000
G1 X69.217 Y122.194 E0.37911
G1 X76.822 Y122.194 E0.19815
G1 X76.992 Y122.797 E0.01632
G1 X77.307 Y123.544 E0.02112
G1 X77.712 Y124.246 E0.02112
G1 X78.199 Y124.893 E0.02112
G1 X78.763 Y125.475 E0.02112
G1 X79.394 Y125.984 E0.02112
G1 X80.083 Y126.411 E0.02112
G1 X80.819 Y126.750 E0.02112
G1 X81.591 Y126.996 E0.02112
G1 X82.388 Y127.145 E0.02112
G1 X83.190 Y127.194 E0.02093
G1 X90.767 Y127.194 E0.19743
G1 X90.767 Y131.744 E0.11855
G1 X83.190 Y131.744 E0.19743
G1 X82.388 Y131.794 E0.02093
G1 X81.591 Y131.943 E0.02112
```

```
G1 X80.819 Y132.189 E0.02112
G1 X80.083 Y132.528 E0.02112
G1 X79.394 Y132.955 E0.02112
G1 X78.763 Y133.463 E0.02112
G1 X78.199 Y134.046 E0.02112
G1 X77.712 Y134.693 E0.02112
G1 X77.307 Y135.395 E0.02112
G1 X76.992 Y136.142 E0.02112
G1 X76.822 Y136.744 E0.01632
G1 X69.277 Y136.744 E0.19659
M204 S1250
G1 X69.408 Y136.393 F9000.000
G1 E-2.24000 F2400.000
;WIPE_START
G1 F7200.000
G1 X69.265 Y133.864 E-0.91200
;WIPE_END
G1 E-0.04800 F2400.000
G1 Z0.700 F9000.000
G1 X70.606 Y136.201
G1 Z0.500
G1 E3.20000 F1500.000
M204 S1000
;TYPE:Solid infill
;WIDTH:0.450839
G1 F4800.000
G1 X69.948 Y135.543 E0.02431
G1 X69.948 Y134.951 E0.01546
G1 X71.010 Y136.013 E0.03922
```

```
G1 X71.602 Y136.013 E0.01546
G1 X69.948 Y134.359 E0.06108
G1 X69.948 Y133.767 E0.01546
G1 X72.194 Y136.013 E0.08294
G1 X72.786 Y136.013 E0.01546
G1 X69.948 Y133.175 E0.10480
G1 X69.948 Y132.583 E0.01546
G1 X73.378 Y136.013 E0.12666
G1 X73.971 Y136.013 E0.01546
G1 X69.948 Y131.991 E0.14853
G1 X69.948 Y131.399 E0.01546
G1 X74.563 Y136.013 E0.17039
G1 X75.155 Y136.013 E0.01546
G1 X69.948 Y130.807 E0.19225
G1 X69.948 Y130.215 E0.01546
G1 X75.747 Y136.013 E0.21411
G1 X76.269 Y136.013 E0.01363
G1 X76.284 Y135.959 E0.00148
G1 X69.948 Y129.622 E0.23395
G1 X69.948 Y129.030 E0.01546
G1 X76.454 Y135.536 E0.24022
G1 X76.630 Y135.120 E0.01180
G1 X69.948 Y128.438 E0.24671
G1 X69.948 Y127.846 E0.01546
G1 X76.841 Y134.739 E0.25451
G1 X77.057 Y134.364 E0.01132
G1 X69.948 Y127.254 E0.26250
G1 X69.948 Y126.662 E0.01546
G1 X77.304 Y134.019 E0.27162
```

```
G1 X77.559 Y133.681 E0.01104
G1 X69.948 Y126.070 E0.28101
G1 X69.948 Y125.478 E0.01546
G1 X77.838 Y133.368 E0.29132
G1 X78.129 Y133.067 E0.01093
G1 X69.948 Y124.886 E0.30207
G1 X69.948 Y124.294 E0.01546
G1 X78.439 Y132.785 E0.31353
G1 X78.767 Y132.521 E0.01099
G1 X69.948 Y123.702 E0.32563
G1 X69.948 Y123.110 E0.01546
G1 X79.109 Y132.271 E0.33826
G1 X79.475 Y132.045 E0.01123
G1 X70.356 Y122.926 E0.33670
G1 X70.948 Y122.926 E0.01546
G1 X79.852 Y131.829 E0.32875
G1 X80.257 Y131.643 E0.01165
G1 X71.540 Y122.926 E0.32186
G1 X72.132 Y122.926 E0.01546
G1 X80.674 Y131.468 E0.31540
G1 X81.123 Y131.325 E0.01230
G1 X72.724 Y122.926 E0.31012
G1 X73.316 Y122.926 E0.01546
G1 X81.590 Y131.199 E0.30549
G1 X82.089 Y131.106 E0.01325
G1 X73.908 Y122.926 E0.30204
G1 X74.500 Y122.926 E0.01546
G1 X82.799 Y131.224 E0.30641
M204 S1250
```

```
G1 E-2.24000 F2400.000
;WIPE_START
G1 F7200.000
G1 X80.763 Y129.188 E-0.91200
;WIPE_END
G1 E-0.04800 F2400.000
G1 Z0.700 F9000.000
G1 X90.224 Y128.584
G1 Z0.500
G1 E3.20000 F1500.000
M204 S1000
G1 F4800.000
G1 X89.565 Y127.926 E0.02431
G1 X88.973 Y127.926 E0.01546
G1 X90.036 Y128.988 E0.03923
G1 X90.036 Y129.580 E0.01546
G1 X88.381 Y127.926 E0.06109
G1 X87.789 Y127.926 E0.01546
G1 X90.036 Y130.172 E0.08295
G1 X90.036 Y130.764 E0.01546
G1 X87.197 Y127.926 E0.10481
G1 X86.605 Y127.926 E0.01546
G1 X89.693 Y131.013 E0.11400
G1 X89.101 Y131.013 E0.01546
G1 X86.013 Y127.926 E0.11400
G1 X85.421 Y127.926 E0.01546
G1 X88.508 Y131.013 E0.11400
G1 X87.916 Y131.013 E0.01546
G1 X84.829 Y127.926 E0.11400
```

```
G1 X84.237 Y127.926 E0.01546
G1 X87.324 Y131.013 E0.11400
G1 X86.732 Y131.013 E0.01546
G1 X83.645 Y127.926 E0.11400
G1 X83.045 Y127.918 E0.01566
G1 X86.140 Y131.013 E0.11428
G1 X85.548 Y131.013 E0.01546
G1 X82.414 Y127.879 E0.11572
G1 X81.704 Y127.761 E0.01880
G1 X84.956 Y131.013 E0.12009
G1 X84.364 Y131.013 E0.01546
G1 X80.891 Y127.540 E0.12823
G1 X80.554 Y127.433 E0.00923
G1 X79.882 Y127.124 E0.01931
G1 X83.772 Y131.013 E0.14362
G1 X83.180 Y131.013 E0.01546
G1 X75.092 Y122.926 E0.29862
G1 X75.684 Y122.926 E0.01546
G1 X77.194 Y124.435 E0.05573
M204 S1250
; stop printing object tpu print.STL id:7 copy 0
; printing object tpu print.STL id:3 copy 0
G1 E-2.24000 F2400.000
;WIPE_START
G1 F7200.000
G1 X75.684 Y122.926 E-0.67595
G1 X75.092 Y122.926 E-0.18749
G1 X75.201 Y123.034 E-0.04857
;WIPE_END
```

```
G1 E-0.04800 F2400.000
G1 Z0.700 F9000.000
G1 X76.521 Y117.673
G1 Z0.500
G1 E3.20000 F1500.000
M204 S800
;TYPE:Perimeter
;WIDTH:0.45
G1 F2400.000
G1 X69.650 Y117.673 E0.17903
G1 X69.650 Y103.958 E0.35734
G1 X76.521 Y103.958 E0.17903
G1 X76.612 Y104.282 E0.00876
G1 X76.947 Y105.076 E0.02246
G1 X77.378 Y105.823 E0.02246
G1 X77.896 Y106.511 E0.02246
G1 X78.496 Y107.131 E0.02246
G1 X79.167 Y107.672 E0.02246
G1 X79.900 Y108.126 E0.02246
G1 X80.683 Y108.486 E0.02246
G1 X81.504 Y108.748 E0.02246
G1 X82.351 Y108.906 E0.02246
G1 X83.192 Y108.958 E0.02194
G1 X90.364 Y108.958 E0.18688
G1 X90.364 Y112.673 E0.09678
G1 X83.192 Y112.673 E0.18688
G1 X82.351 Y112.725 E0.02194
G1 X81.504 Y112.883 E0.02246
G1 X80.683 Y113.145 E0.02246
```

```
G1 X79.900 Y113.505 E0.02246
G1 X79.167 Y113.960 E0.02246
G1 X78.496 Y114.500 E0.02246
G1 X77.896 Y115.120 E0.02246
G1 X77.378 Y115.808 E0.02246
G1 X76.947 Y116.555 E0.02246
G1 X76.612 Y117.349 E0.02246
G1 X76.537 Y117.615 E0.00720
M204 S1250
G1 X76.837 Y118.091 F9000.000
M204 S800
;TYPE:External perimeter
G1 F1800.000
G1 X69.232 Y118.091 E0.19815
G1 X69.232 Y103.541 E0.37911
G1 X76.837 Y103.541 E0.19815
G1 X77.007 Y104.143 E0.01632
G1 X77.322 Y104.890 E0.02112
G1 X77.727 Y105.592 E0.02112
G1 X78.215 Y106.239 E0.02112
G1 X78.778 Y106.822 E0.02112
G1 X79.409 Y107.330 E0.02112
G1 X80.098 Y107.757 E0.02112
G1 X80.834 Y108.096 E0.02112
G1 X81.606 Y108.342 E0.02112
G1 X82.403 Y108.491 E0.02112
G1 X83.205 Y108.541 E0.02093
G1 X90.782 Y108.541 E0.19743
G1 X90.782 Y113.091 E0.11855
```

```
G1 X83.205 Y113.091 E0.19743
G1 X82.403 Y113.140 E0.02093
G1 X81.606 Y113.289 E0.02112
G1 X80.834 Y113.535 E0.02112
G1 X80.098 Y113.874 E0.02112
G1 X79.409 Y114.301 E0.02112
G1 X78.778 Y114.810 E0.02112
G1 X78.215 Y115.392 E0.02112
G1 X77.727 Y116.039 E0.02112
G1 X77.322 Y116.741 E0.02112
G1 X77.007 Y117.488 E0.02112
G1 X76.853 Y118.033 E0.01476
M204 S1250
G1 X76.516 Y117.852 F9000.000
G1 E-2.24000 F2400.000
;WIPE_START
G1 F7200.000
G1 X73.973 Y118.055 E-0.91200
;WIPE_END
G1 E-0.04800 F2400.000
G1 Z0.700 F9000.000
G1 X70.621 Y117.547
G1 Z0.500
G1 E3.20000 F1500.000
M204 S1000
;TYPE:Solid infill
;WIDTH:0.450839
G1 F4800.000
G1 X69.963 Y116.889 E0.02431
```

```
G1 X69.963 Y116.297 E0.01546
G1 X71.025 Y117.359 E0.03922
G1 X71.617 Y117.359 E0.01546
G1 X69.963 Y115.705 E0.06108
G1 X69.963 Y115.113 E0.01546
G1 X72.209 Y117.359 E0.08294
G1 X72.802 Y117.359 E0.01546
G1 X69.963 Y114.521 E0.10480
G1 X69.963 Y113.929 E0.01546
G1 X73.394 Y117.359 E0.12666
G1 X73.986 Y117.359 E0.01546
G1 X69.963 Y113.337 E0.14853
G1 X69.963 Y112.745 E0.01546
G1 X74.578 Y117.359 E0.17039
G1 X75.170 Y117.359 E0.01546
G1 X69.963 Y112.153 E0.19225
G1 X69.963 Y111.561 E0.01546
G1 X75.762 Y117.359 E0.21411
G1 X76.284 Y117.359 E0.01363
G1 X76.299 Y117.305 E0.00148
G1 X69.963 Y110.968 E0.23395
G1 X69.963 Y110.376 E0.01546
G1 X76.469 Y116.883 E0.24022
G1 X76.645 Y116.466 E0.01180
G1 X69.963 Y109.784 E0.24671
G1 X69.963 Y109.192 E0.01546
G1 X76.856 Y116.086 E0.25451
G1 X77.073 Y115.710 E0.01132
G1 X69.963 Y108.600 E0.26250
```

```
G1 X69.963 Y108.008 E0.01546
G1 X77.319 Y115.365 E0.27162
G1 X77.574 Y115.027 E0.01104
G1 X69.963 Y107.416 E0.28101
G1 X69.963 Y106.824 E0.01546
G1 X77.853 Y114.714 E0.29132
G1 X78.144 Y114.413 E0.01093
G1 X69.963 Y106.232 E0.30207
G1 X69.963 Y105.640 E0.01546
G1 X78.454 Y114.131 E0.31353
G1 X78.782 Y113.867 E0.01099
G1 X69.963 Y105.048 E0.32563
G1 X69.963 Y104.456 E0.01546
G1 X79.124 Y113.617 E0.33826
G1 X79.490 Y113.391 E0.01123
G1 X70.371 Y104.272 E0.33670
G1 X70.963 Y104.272 E0.01546
G1 X79.867 Y113.176 E0.32875
G1 X80.272 Y112.989 E0.01165
G1 X71.555 Y104.272 E0.32186
G1 X72.147 Y104.272 E0.01546
G1 X80.689 Y112.814 E0.31540
G1 X81.138 Y112.671 E0.01230
G1 X72.739 Y104.272 E0.31012
G1 X73.331 Y104.272 E0.01546
G1 X81.605 Y112.545 E0.30549
G1 X82.104 Y112.452 E0.01325
G1 X73.923 Y104.272 E0.30204
G1 X74.515 Y104.272 E0.01546
```

```
G1 X82.814 Y112.570 E0.30641
M204 S1250
G1 E-2.24000 F2400.000
;WIPE_START
G1 F7200.000
G1 X80.778 Y110.534 E-0.91200
;WIPE_END
G1 E-0.04800 F2400.000
G1 Z0.700 F9000.000
G1 X90.239 Y109.930
G1 Z0.500
G1 E3.20000 F1500.000
M204 S1000
G1 F4800.000
G1 X89.580 Y109.272 E0.02431
G1 X88.988 Y109.272 E0.01546
G1 X90.051 Y110.334 E0.03923
G1 X90.051 Y110.926 E0.01546
G1 X88.396 Y109.272 E0.06109
G1 X87.804 Y109.272 E0.01546
G1 X90.051 Y111.518 E0.08295
G1 X90.051 Y112.110 E0.01546
G1 X87.212 Y109.272 E0.10481
G1 X86.620 Y109.272 E0.01546
G1 X89.708 Y112.359 E0.11400
G1 X89.116 Y112.359 E0.01546
G1 X86.028 Y109.272 E0.11400
G1 X85.436 Y109.272 E0.01546
G1 X88.524 Y112.359 E0.11400
```

G1 X87.932 Y112.359 E0.01546

G1 X84.844 Y109.272 E0.11400

G1 X84.252 Y109.272 E0.01546

G1 X87.339 Y112.359 E0.11400

G1 X86.747 Y112.359 E0.01546

G1 X83.660 Y109.272 E0.11400

G1 X83.060 Y109.264 E0.01566

G1 X86.155 Y112.359 E0.11428

G1 X85.563 Y112.359 E0.01546

G1 X82.429 Y109.225 E0.11572

G1 X81.719 Y109.107 E0.01880

G1 X84.971 Y112.359 E0.12009

G1 X84.379 Y112.359 E0.01546

G1 X80.906 Y108.886 E0.12823

G1 X80.569 Y108.779 E0.00923

G1 X79.897 Y108.470 E0.01931

G1 X83.787 Y112.359 E0.14362

G1 X83.195 Y112.359 E0.01546

G1 X75.107 Y104.272 E0.29862

G1 X75.699 Y104.272 E0.01546

G1 X77.209 Y105.781 E0.05573

M204 S1250

; stop printing object tpu print.STL id:3 copy 0

; printing object Petg print.STL id:2 copy 0

; stop printing object Petg print.STL id:2 copy 0

; printing object Petg print.STL id:6 copy 0

; stop printing object Petg print.STL id:6 copy 0

; printing object tpu print.STL id:27 copy 0

G1 E-2.24000 F2400.000

```
;WIPE_START
G1 F7200.000
G1 X75.699 Y104.272 E-0.67595
G1 X75.107 Y104.272 E-0.18749
G1 X75.216 Y104.380 E-0.04857
;WIPE_END
G1 E-0.04800 F2400.000
G1 Z0.700 F9000.000
G1 X120.285 Y122.787
G1 Z0.500
G1 E3.20000 F1500.000
M204 S800
;TYPE:Perimeter
;WIDTH:0.45
G1 F2400.000
G1 X127.156 Y122.787 E0.17903
G1 X127.247 Y123.111 E0.00876
G1 X127.583 Y123.905 E0.02246
G1 X128.013 Y124.652 E0.02246
G1 X128.532 Y125.340 E0.02246
G1 X129.131 Y125.960 E0.02246
G1 X129.802 Y126.501 E0.02246
G1 X130.535 Y126.955 E0.02246
G1 X131.318 Y127.315 E0.02246
G1 X132.139 Y127.577 E0.02246
G1 X132.986 Y127.735 E0.02246
G1 X133.827 Y127.787 E0.02194
G1 X140.999 Y127.787 E0.18688
G1 X140.999 Y131.502 E0.09678
```

```
G1 X133.827 Y131.502 E0.18688
G1 X132.986 Y131.554 E0.02194
G1 X132.139 Y131.712 E0.02246
G1 X131.318 Y131.974 E0.02246
M73 P50 R33
G1 X130.535 Y132.334 E0.02246
G1 X129.802 Y132.789 E0.02246
G1 X129.131 Y133.329 E0.02246
G1 X128.532 Y133.949 E0.02246
G1 X128.013 Y134.637 E0.02246
G1 X127.583 Y135.384 E0.02246
G1 X127.247 Y136.178 E0.02246
G1 X127.156 Y136.502 E0.00876
G1 X120.285 Y136.502 E0.17903
G1 X120.285 Y122.847 E0.35578
M204 S1250
G1 X119.867 Y122.370 F9000.000
M204 S800
;TYPE:External perimeter
G1 F1800.000
G1 X127.472 Y122.370 E0.19815
G1 X127.642 Y122.972 E0.01632
G1 X127.957 Y123.719 E0.02112
G1 X128.362 Y124.421 E0.02112
G1 X128.850 Y125.068 E0.02112
G1 X129.413 Y125.651 E0.02112
G1 X130.044 Y126.159 E0.02112
G1 X130.733 Y126.586 E0.02112
G1 X131.469 Y126.925 E0.02112
```

```
G1 X132.241 Y127.171 E0.02112
G1 X133.038 Y127.320 E0.02112
G1 X133.840 Y127.370 E0.02093
G1 X141.417 Y127.370 E0.19743
G1 X141.417 Y131.920 E0.11855
G1 X133.840 Y131.920 E0.19743
G1 X133.038 Y131.969 E0.02093
G1 X132.241 Y132.118 E0.02112
G1 X131.469 Y132.364 E0.02112
G1 X130.733 Y132.703 E0.02112
G1 X130.044 Y133.130 E0.02112
G1 X129.413 Y133.639 E0.02112
G1 X128.850 Y134.221 E0.02112
G1 X128.362 Y134.868 E0.02112
G1 X127.957 Y135.570 E0.02112
G1 X127.642 Y136.317 E0.02112
G1 X127.472 Y136.920 E0.01632
G1 X119.867 Y136.920 E0.19815
G1 X119.867 Y122.430 E0.37755
M204 S1250
G1 X120.241 Y122.512 F9000.000
G1 E-2.24000 F2400.000
;WIPE_START
G1 F7200.000
G1 X122.747 Y122.407 E-0.91200
;WIPE_END
G1 E-0.04800 F2400.000
G1 Z0.700 F9000.000
G1 X127.844 Y124.610
```

G1 Z0.500

G1 E3.20000 F1500.000

M204 S1000

;TYPE:Solid infill

;WIDTH:0.450839

G1 F4800.000

G1 X126.335 Y123.101 E0.05573

M73 P51 R33

G1 X125.743 Y123.101 E0.01546

G1 X133.830 Y131.188 E0.29862

G1 X134.422 Y131.188 E0.01546

G1 X130.532 Y127.299 E0.14362

G1 X131.204 Y127.608 E0.01931

G1 X131.541 Y127.715 E0.00923

G1 X135.014 Y131.188 E0.12823

G1 X135.606 Y131.188 E0.01546

G1 X132.354 Y127.936 E0.12009

G1 X133.064 Y128.054 E0.01880

G1 X136.198 Y131.188 E0.11572

G1 X136.790 Y131.188 E0.01546

G1 X133.695 Y128.093 E0.11428

G1 X134.295 Y128.101 E0.01566

G1 X137.383 Y131.188 E0.11400

G1 X137.975 Y131.188 E0.01546

G1 X134.887 Y128.101 E0.11400

G1 X135.479 Y128.101 E0.01546

G1 X138.567 Y131.188 E0.11400

G1 X139.159 Y131.188 E0.01546

G1 X136.071 Y128.101 E0.11400

```
G1 X136.663 Y128.101 E0.01546
G1 X139.751 Y131.188 E0.11400
G1 X140.343 Y131.188 E0.01546
G1 X137.255 Y128.101 E0.11400
G1 X137.847 Y128.101 E0.01546
G1 X140.686 Y130.939 E0.10481
G1 X140.686 Y130.347 E0.01546
G1 X138.439 Y128.101 E0.08295
G1 X139.031 Y128.101 E0.01546
G1 X140.686 Y129.755 E0.06109
G1 X140.686 Y129.163 E0.01546
G1 X139.623 Y128.101 E0.03923
G1 X140.215 Y128.101 E0.01546
G1 X140.874 Y128.759 E0.02431
M204 S1250
G1 E-2.24000 F2400.000
;WIPE_START
G1 F7200.000
G1 X140.215 Y128.101 E-0.29484
G1 X139.623 Y128.101 E-0.18749
G1 X140.583 Y129.060 E-0.42968
;WIPE_END
G1 E-0.04800 F2400.000
G1 Z0.700 F9000.000
G1 X133.449 Y131.399
G1 Z0.500
G1 E3.20000 F1500.000
M204 S1000
G1 F4800.000
```

```
G1 X125.150 Y123.101 E0.30641
G1 X124.558 Y123.101 E0.01546
G1 X132.739 Y131.281 E0.30204
G1 X132.240 Y131.374 E0.01325
G1 X123.966 Y123.101 E0.30549
G1 X123.374 Y123.101 E0.01546
G1 X131.773 Y131.500 E0.31012
G1 X131.324 Y131.643 E0.01230
G1 X122.782 Y123.101 E0.31540
G1 X122.190 Y123.101 E0.01546
G1 X130.907 Y131.818 E0.32186
G1 X130.502 Y132.005 E0.01165
G1 X121.598 Y123.101 E0.32875
G1 X121.006 Y123.101 E0.01546
G1 X130.125 Y132.220 E0.33670
G1 X129.760 Y132.446 E0.01123
G1 X120.598 Y123.285 E0.33826
G1 X120.598 Y123.877 E0.01546
G1 X129.417 Y132.696 E0.32563
G1 X129.090 Y132.960 E0.01099
G1 X120.598 Y124.469 E0.31353
G1 X120.598 Y125.061 E0.01546
G1 X128.779 Y133.242 E0.30207
G1 X128.488 Y133.543 E0.01093
G1 X120.598 Y125.653 E0.29132
G1 X120.598 Y126.245 E0.01546
G1 X128.209 Y133.856 E0.28101
G1 X127.955 Y134.194 E0.01104
G1 X120.598 Y126.837 E0.27162
```

```
G1 X120.598 Y127.429 E0.01546
G1 X127.708 Y134.539 E0.26250
G1 X127.491 Y134.915 E0.01132
G1 X120.598 Y128.021 E0.25451
G1 X120.598 Y128.613 E0.01546
G1 X127.280 Y135.295 E0.24671
G1 X127.104 Y135.712 E0.01180
G1 X120.598 Y129.205 E0.24022
G1 X120.598 Y129.798 E0.01546
G1 X126.934 Y136.134 E0.23395
G1 X126.919 Y136.188 E0.00148
G1 X126.397 Y136.188 E0.01363
G1 X120.598 Y130.390 E0.21411
G1 X120.598 Y130.982 E0.01546
G1 X125.805 Y136.188 E0.19225
G1 X125.213 Y136.188 E0.01546
G1 X120.598 Y131.574 E0.17039
G1 X120.598 Y132.166 E0.01546
G1 X124.621 Y136.188 E0.14853
G1 X124.029 Y136.188 E0.01546
G1 X120.598 Y132.758 E0.12666
G1 X120.598 Y133.350 E0.01546
G1 X123.437 Y136.188 E0.10480
G1 X122.845 Y136.188 E0.01546
G1 X120.598 Y133.942 E0.08294
G1 X120.598 Y134.534 E0.01546
G1 X122.253 Y136.188 E0.06108
G1 X121.660 Y136.188 E0.01546
G1 X120.598 Y135.126 E0.03922
```

```
G1 X120.598 Y135.718 E0.01546
G1 X121.256 Y136.376 E0.02431
M204 S1250
; stop printing object tpu print.STL id:27 copy 0
; printing object tpu print.STL id:23 copy 0
G1 E-2.24000 F2400.000
;WIPE_START
G1 F7200.000
G1 X120.598 Y135.718 E-0.29480
G1 X120.598 Y135.126 E-0.18749
G1 X121.558 Y136.086 E-0.42971
;WIPE_END
G1 E-0.04800 F2400.000
G1 Z0.700 F9000.000
G1 X120.300 Y117.848
G1 Z0.500
G1 E3.20000 F1500.000
M204 S800
;TYPE:Perimeter
;WIDTH:0.45
G1 F2400.000
G1 X120.300 Y104.133 E0.35734
G1 X127.171 Y104.133 E0.17903
G1 X127.263 Y104.457 E0.00876
G1 X127.598 Y105.251 E0.02246
G1 X128.028 Y105.998 E0.02246
G1 X128.547 Y106.686 E0.02246
G1 X129.146 Y107.306 E0.02246
G1 X129.817 Y107.847 E0.02246
```

```
G1 X130.550 Y108.301 E0.02246
G1 X131.333 Y108.661 E0.02246
G1 X132.154 Y108.923 E0.02246
G1 X133.002 Y109.082 E0.02246
G1 X133.842 Y109.133 E0.02194
G1 X141.014 Y109.133 E0.18688
G1 X141.014 Y112.848 E0.09678
G1 X133.842 Y112.848 E0.18688
G1 X133.002 Y112.900 E0.02194
G1 X132.154 Y113.058 E0.02246
G1 X131.333 Y113.320 E0.02246
G1 X130.550 Y113.680 E0.02246
G1 X129.817 Y114.135 E0.02246
G1 X129.146 Y114.675 E0.02246
G1 X128.547 Y115.295 E0.02246
G1 X128.028 Y115.983 E0.02246
G1 X127.598 Y116.730 E0.02246
G1 X127.263 Y117.524 E0.02246
G1 X127.171 Y117.848 E0.00876
G1 X120.360 Y117.848 E0.17747
M204 S1250
G1 X119.882 Y118.266 F9000.000
M204 S800
;TYPE:External perimeter
G1 F1800.000
G1 X119.882 Y103.716 E0.37911
G1 X127.487 Y103.716 E0.19815
G1 X127.658 Y104.318 E0.01632
G1 X127.973 Y105.065 E0.02112
```

```
G1 X128.377 Y105.767 E0.02112
G1 X128.865 Y106.414 E0.02112
G1 X129.428 Y106.997 E0.02112
G1 X130.059 Y107.505 E0.02112
G1 X130.748 Y107.932 E0.02112
G1 X131.484 Y108.271 E0.02112
G1 X132.257 Y108.517 E0.02112
G1 X133.053 Y108.666 E0.02112
G1 X133.855 Y108.716 E0.02093
G1 X141.432 Y108.716 E0.19743
G1 X141.432 Y113.266 E0.11855
G1 X133.855 Y113.266 E0.19743
G1 X133.053 Y113.315 E0.02093
G1 X132.257 Y113.464 E0.02112
G1 X131.484 Y113.710 E0.02112
G1 X130.748 Y114.049 E0.02112
G1 X130.059 Y114.476 E0.02112
G1 X129.428 Y114.985 E0.02112
G1 X128.865 Y115.567 E0.02112
G1 X128.377 Y116.214 E0.02112
G1 X127.973 Y116.916 E0.02112
G1 X127.658 Y117.663 E0.02112
G1 X127.487 Y118.266 E0.01632
G1 X119.942 Y118.266 E0.19659
M204 S1250
G1 X120.073 Y117.914 F9000.000
G1 E-2.24000 F2400.000
;WIPE_START
G1 F7200.000
```

```
G1 X119.930 Y115.386 E-0.91200
;WIPE_END
G1 E-0.04800 F2400.000
G1 Z0.700 F9000.000
G1 X121.272 Y117.722
G1 Z0.500
G1 E3.20000 F1500.000
M204 S1000
;TYPE:Solid infill
;WIDTH:0.450839
G1 F4800.000
G1 X120.613 Y117.064 E0.02431
G1 X120.613 Y116.472 E0.01546
G1 X121.676 Y117.534 E0.03922
G1 X122.268 Y117.534 E0.01546
G1 X120.613 Y115.880 E0.06108
G1 X120.613 Y115.288 E0.01546
G1 X122.860 Y117.534 E0.08294
G1 X123.452 Y117.534 E0.01546
G1 X120.613 Y114.696 E0.10480
G1 X120.613 Y114.104 E0.01546
G1 X124.044 Y117.534 E0.12666
G1 X124.636 Y117.534 E0.01546
G1 X120.613 Y113.512 E0.14853
G1 X120.613 Y112.920 E0.01546
G1 X125.228 Y117.534 E0.17039
G1 X125.820 Y117.534 E0.01546
G1 X120.613 Y112.328 E0.19225
G1 X120.613 Y111.736 E0.01546
```

G1 X126.412 Y117.534 E0.21411

G1 X126.934 Y117.534 E0.01363

G1 X126.950 Y117.480 E0.00148

G1 X120.613 Y111.144 E0.23395

G1 X120.613 Y110.552 E0.01546

G1 X127.119 Y117.058 E0.24022

G1 X127.295 Y116.641 E0.01180

G1 X120.613 Y109.959 E0.24671

G1 X120.613 Y109.367 E0.01546

G1 X127.506 Y116.261 E0.25451

G1 X127.723 Y115.885 E0.01132

G1 X120.613 Y108.775 E0.26250

G1 X120.613 Y108.183 E0.01546

G1 X127.970 Y115.540 E0.27162

G1 X128.224 Y115.202 E0.01104

G1 X120.613 Y107.591 E0.28101

G1 X120.613 Y106.999 E0.01546

G1 X128.503 Y114.889 E0.29132

G1 X128.794 Y114.588 E0.01093

G1 X120.613 Y106.407 E0.30207

G1 X120.613 Y105.815 E0.01546

G1 X129.105 Y114.306 E0.31353

G1 X129.433 Y114.042 E0.01099

G1 X120.613 Y105.223 E0.32563

G1 X120.613 Y104.631 E0.01546

G1 X129.775 Y113.792 E0.33826

G1 X130.140 Y113.566 E0.01123

G1 X121.021 Y104.447 E0.33670

G1 X121.613 Y104.447 E0.01546

```
G1 X130.517 Y113.351 E0.32875
G1 X130.922 Y113.164 E0.01165
G1 X122.205 Y104.447 E0.32186
G1 X122.797 Y104.447 E0.01546
G1 X131.339 Y112.989 E0.31540
G1 X131.788 Y112.846 E0.01230
G1 X123.389 Y104.447 E0.31012
G1 X123.981 Y104.447 E0.01546
G1 X132.255 Y112.721 E0.30549
G1 X132.754 Y112.627 E0.01325
G1 X124.574 Y104.447 E0.30204
G1 X125.166 Y104.447 E0.01546
G1 X133.464 Y112.746 E0.30641
M204 S1250
G1 E-2.24000 F2400.000
;WIPE_START
G1 F7200.000
G1 X131.428 Y110.709 E-0.91200
;WIPE_END
G1 E-0.04800 F2400.000
G1 Z0.700 F9000.000
G1 X140.889 Y110.105
G1 Z0.500
G1 E3.20000 F1500.000
M204 S1000
G1 F4800.000
G1 X140.231 Y109.447 E0.02431
G1 X139.639 Y109.447 E0.01546
G1 X140.701 Y110.509 E0.03923
```

```
G1 X140.701 Y111.101 E0.01546
G1 X139.046 Y109.447 E0.06109
G1 X138.454 Y109.447 E0.01546
G1 X140.701 Y111.693 E0.08295
G1 X140.701 Y112.285 E0.01546
G1 X137.862 Y109.447 E0.10481
G1 X137.270 Y109.447 E0.01546
G1 X140.358 Y112.534 E0.11400
G1 X139.766 Y112.534 E0.01546
G1 X136.678 Y109.447 E0.11400
G1 X136.086 Y109.447 E0.01546
G1 X139.174 Y112.534 E0.11400
G1 X138.582 Y112.534 E0.01546
G1 X135.494 Y109.447 E0.11400
G1 X134.902 Y109.447 E0.01546
G1 X137.990 Y112.534 E0.11400
G1 X137.398 Y112.534 E0.01546
G1 X134.310 Y109.447 E0.11400
G1 X133.710 Y109.439 E0.01566
G1 X136.806 Y112.534 E0.11428
G1 X136.214 Y112.534 E0.01546
G1 X133.079 Y109.400 E0.11572
G1 X132.369 Y109.282 E0.01880
G1 X135.621 Y112.534 E0.12009
G1 X135.029 Y112.534 E0.01546
G1 X131.556 Y109.061 E0.12823
G1 X131.219 Y108.954 E0.00923
G1 X130.548 Y108.645 E0.01931
G1 X134.437 Y112.534 E0.14362
```

```
G1 X133.845 Y112.534 E0.01546
G1 X125.758 Y104.447 E0.29862
G1 X126.350 Y104.447 E0.01546
G1 X127.859 Y105.956 E0.05573
M204 S1250
; stop printing object tpu print.STL id:23 copy 0
; printing object tpu print.STL id:21 copy 0
G1 E-2.24000 F2400.000
;WIPE_START
G1 F7200.000
G1 X126.350 Y104.447 E-0.67595
G1 X125.758 Y104.447 E-0.18749
G1 X125.866 Y104.555 E-0.04857
;WIPE_END
G1 E-0.04800 F2400.000
G1 Z0.700 F9000.000
G1 X127.164 Y97.032
G1 Z0.500
G1 E3.20000 F1500.000
M204 S800
;TYPE:Perimeter
;WIDTH:0.45
G1 F2400.000
G1 X120.293 Y97.032 E0.17903
G1 X120.293 Y83.318 E0.35734
G1 X127.164 Y83.318 E0.17903
G1 X127.256 Y83.641 E0.00876
G1 X127.591 Y84.436 E0.02246
G1 X128.021 Y85.183 E0.02246
```

```
G1 X128.540 Y85.871 E0.02246
G1 X129.139 Y86.490 E0.02246
G1 X129.811 Y87.031 E0.02246
G1 X130.543 Y87.485 E0.02246
G1 X131.326 Y87.846 E0.02246
G1 X132.147 Y88.107 E0.02246
G1 X132.995 Y88.266 E0.02246
G1 X133.835 Y88.318 E0.02194
G1 X141.007 Y88.318 E0.18688
G1 X141.007 Y92.032 E0.09678
G1 X133.835 Y92.032 E0.18688
G1 X132.995 Y92.084 E0.02194
G1 X132.147 Y92.243 E0.02246
G1 X131.326 Y92.504 E0.02246
G1 X130.543 Y92.865 E0.02246
G1 X129.811 Y93.319 E0.02246
G1 X129.139 Y93.860 E0.02246
G1 X128.540 Y94.479 E0.02246
G1 X128.021 Y95.168 E0.02246
G1 X127.591 Y95.915 E0.02246
G1 X127.256 Y96.709 E0.02246
G1 X127.181 Y96.975 E0.00720
M204 S1250
G1 X127.480 Y97.450 F9000.000
M204 S800
;TYPE:External perimeter
G1 F1800.000
G1 X119.875 Y97.450 E0.19815
G1 X119.875 Y82.900 E0.37911
```

```
G1 X127.480 Y82.900 E0.19815
G1 X127.651 Y83.503 E0.01632
G1 X127.966 Y84.249 E0.02112
G1 X128.370 Y84.952 E0.02112
G1 X128.858 Y85.599 E0.02112
G1 X129.422 Y86.181 E0.02112
G1 X130.053 Y86.690 E0.02112
G1 X130.741 Y87.117 E0.02112
G1 X131.477 Y87.456 E0.02112
G1 X132.250 Y87.702 E0.02112
G1 X133.046 Y87.851 E0.02112
G1 X133.848 Y87.900 E0.02093
G1 X141.425 Y87.900 E0.19743
G1 X141.425 Y92.450 E0.11855
G1 X133.848 Y92.450 E0.19743
G1 X133.046 Y92.500 E0.02093
G1 X132.250 Y92.649 E0.02112
G1 X131.477 Y92.895 E0.02112
G1 X130.741 Y93.234 E0.02112
G1 X130.053 Y93.661 E0.02112
G1 X129.422 Y94.169 E0.02112
G1 X128.858 Y94.751 E0.02112
G1 X128.370 Y95.398 E0.02112
G1 X127.966 Y96.101 E0.02112
G1 X127.651 Y96.847 E0.02112
G1 X127.496 Y97.392 E0.01476
M204 S1250
G1 X127.159 Y97.212 F9000.000
G1 E-2.24000 F2400.000
```

```
;WIPE_START
G1 F7200.000
G1 X124.617 Y97.414 E-0.91200
;WIPE_END
G1 E-0.04800 F2400.000
G1 Z0.700 F9000.000
G1 X121.265 Y96.907
G1 Z0.500
G1 E3.20000 F1500.000
M204 S1000
;TYPE:Solid infill
;WIDTH:0.450839
G1 F4800.000
G1 X120.606 Y96.249 E0.02431
G1 X120.606 Y95.657 E0.01546
G1 X121.669 Y96.719 E0.03922
G1 X122.261 Y96.719 E0.01546
G1 X120.606 Y95.065 E0.06108
G1 X120.606 Y94.472 E0.01546
G1 X122.853 Y96.719 E0.08294
G1 X123.445 Y96.719 E0.01546
G1 X120.606 Y93.880 E0.10480
G1 X120.606 Y93.288 E0.01546
G1 X124.037 Y96.719 E0.12666
G1 X124.629 Y96.719 E0.01546
G1 X120.606 Y92.696 E0.14853
G1 X120.606 Y92.104 E0.01546
G1 X125.221 Y96.719 E0.17039
G1 X125.813 Y96.719 E0.01546
```

```
G1 X120.606 Y91.512 E0.19225
G1 X120.606 Y90.920 E0.01546
G1 X126.405 Y96.719 E0.21411
G1 X126.927 Y96.719 E0.01363
G1 X126.943 Y96.664 E0.00148
G1 X120.606 Y90.328 E0.23395
G1 X120.606 Y89.736 E0.01546
G1 X127.113 Y96.242 E0.24022
G1 X127.288 Y95.826 E0.01180
G1 X120.606 Y89.144 E0.24671
G1 X120.606 Y88.552 E0.01546
G1 X127.500 Y95.445 E0.25451
G1 X127.716 Y95.069 E0.01132
G1 X120.606 Y87.960 E0.26250
G1 X120.606 Y87.368 E0.01546
G1 X127.963 Y94.724 E0.27162
G1 X128.217 Y94.387 E0.01104
G1 X120.606 Y86.776 E0.28101
G1 X120.606 Y86.184 E0.01546
G1 X128.496 Y94.074 E0.29132
G1 X128.788 Y93.773 E0.01093
G1 X120.606 Y85.592 E0.30207
G1 X120.606 Y85.000 E0.01546
G1 X129.098 Y93.491 E0.31353
G1 X129.426 Y93.227 E0.01099
G1 X120.606 Y84.407 E0.32563
G1 X120.606 Y83.815 E0.01546
G1 X129.768 Y92.977 E0.33826
G1 X130.133 Y92.750 E0.01123
```

```
G1 X121.014 Y83.631 E0.33670
G1 X121.606 Y83.631 E0.01546
G1 X130.510 Y92.535 E0.32875
G1 X130.916 Y92.348 E0.01165
G1 X122.198 Y83.631 E0.32186
G1 X122.790 Y83.631 E0.01546
G1 X131.333 Y92.173 E0.31540
G1 X131.782 Y92.030 E0.01230
G1 X123.383 Y83.631 E0.31012
G1 X123.975 Y83.631 E0.01546
G1 X132.248 Y91.905 E0.30549
G1 X132.747 Y91.812 E0.01325
G1 X124.567 Y83.631 E0.30204
G1 X125.159 Y83.631 E0.01546
G1 X133.457 Y91.930 E0.30641
M204 S1250
G1 E-2.24000 F2400.000
;WIPE_START
G1 F7200.000
G1 X131.421 Y89.894 E-0.91200
;WIPE_END
G1 E-0.04800 F2400.000
G1 Z0.700 F9000.000
G1 X140.882 Y89.290
G1 Z0.500
G1 E3.20000 F1500.000
M204 S1000
G1 F4800.000
G1 X140.224 Y88.631 E0.02431
```

```
G1 X139.632 Y88.631 E0.01546
G1 X140.694 Y89.694 E0.03923
G1 X140.694 Y90.286 E0.01546
G1 X139.040 Y88.631 E0.06109
G1 X138.448 Y88.631 E0.01546
G1 X140.694 Y90.878 E0.08295
G1 X140.694 Y91.470 E0.01546
G1 X137.855 Y88.631 E0.10481
G1 X137.263 Y88.631 E0.01546
G1 X140.351 Y91.719 E0.11400
G1 X139.759 Y91.719 E0.01546
G1 X136.671 Y88.631 E0.11400
G1 X136.079 Y88.631 E0.01546
G1 X139.167 Y91.719 E0.11400
G1 X138.575 Y91.719 E0.01546
G1 X135.487 Y88.631 E0.11400
G1 X134.895 Y88.631 E0.01546
G1 X137.983 Y91.719 E0.11400
G1 X137.391 Y91.719 E0.01546
G1 X134.303 Y88.631 E0.11400
G1 X133.704 Y88.624 E0.01566
G1 X136.799 Y91.719 E0.11428
G1 X136.207 Y91.719 E0.01546
G1 X133.073 Y88.585 E0.11572
G1 X132.362 Y88.466 E0.01880
G1 X135.615 Y91.719 E0.12009
G1 X135.023 Y91.719 E0.01546
G1 X131.550 Y88.246 E0.12823
G1 X131.213 Y88.139 E0.00923
```

```
G1 X130.541 Y87.829 E0.01931
G1 X134.431 Y91.719 E0.14362
G1 X133.838 Y91.719 E0.01546
G1 X125.751 Y83.631 E0.29862
G1 X126.343 Y83.631 E0.01546
G1 X127.852 Y85.141 E0.05573
M204 S1250
; stop printing object tpu print.STL id:21 copy 0
; printing object Petg print.STL id:20 copy 0
; stop printing object Petg print.STL id:20 copy 0
; printing object Petg print.STL id:22 copy 0
; stop printing object Petg print.STL id:22 copy 0
; printing object Petg print.STL id:26 copy 0
; stop printing object Petg print.STL id:26 copy 0
; printing object Petg print.STL id:0 copy 0
; stop printing object Petg print.STL id:0 copy 0
; printing object tpu print.STL id:1 copy 0
G1 E-2.24000 F2400.000
;WIPE_START
G1 F7200.000
G1 X126.343 Y83.631 E-0.67595
G1 X125.751 Y83.631 E-0.18749
G1 X125.859 Y83.740 E-0.04857
;WIPE_END
G1 E-0.04800 F2400.000
G1 Z0.700 F9000.000
G1 X90.357 Y88.143
G1 Z0.500
G1 E3.20000 F1500.000
```

```
M204 S800
;TYPE:Perimeter
;WIDTH:0.45
G1 F2400.000
G1 X90.357 Y91.857 E0.09678
G1 X83.185 Y91.857 E0.18688
G1 X82.344 Y91.909 E0.02194
G1 X81.497 Y92.068 E0.02246
G1 X80.676 Y92.329 E0.02246
G1 X79.893 Y92.690 E0.02246
G1 X79.160 Y93.144 E0.02246
G1 X78.489 Y93.685 E0.02246
G1 X77.890 Y94.304 E0.02246
G1 X77.371 Y94.993 E0.02246
G1 X76.941 Y95.739 E0.02246
G1 X76.606 Y96.534 E0.02246
G1 X76.514 Y96.857 E0.00876
G1 X69.643 Y96.857 E0.17903
G1 X69.643 Y83.143 E0.35734
G1 X76.514 Y83.143 E0.17903
G1 X76.606 Y83.466 E0.00876
G1 X76.941 Y84.261 E0.02246
G1 X77.371 Y85.007 E0.02246
G1 X77.890 Y85.696 E0.02246
G1 X78.489 Y86.315 E0.02246
G1 X79.160 Y86.856 E0.02246
G1 X79.893 Y87.310 E0.02246
G1 X80.676 Y87.671 E0.02246
G1 X81.497 Y87.932 E0.02246
```

```
G1 X82.344 Y88.091 E0.02246
G1 X83.185 Y88.143 E0.02194
G1 X90.297 Y88.143 E0.18531
M204 S1250
G1 X90.775 Y87.725 F9000.000
M204 S800
;TYPE:External perimeter
G1 F1800.000
G1 X90.775 Y92.275 E0.11855
G1 X83.198 Y92.275 E0.19743
G1 X82.396 Y92.325 E0.02093
G1 X81.599 Y92.474 E0.02112
G1 X80.827 Y92.719 E0.02112
G1 X80.091 Y93.059 E0.02112
G1 X79.402 Y93.486 E0.02112
G1 X78.771 Y93.994 E0.02112
G1 X78.208 Y94.576 E0.02112
G1 X77.720 Y95.223 E0.02112
G1 X77.315 Y95.926 E0.02112
G1 X77.000 Y96.672 E0.02112
G1 X76.830 Y97.275 E0.01632
G1 X69.225 Y97.275 E0.19815
M73 P52 R33
G1 X69.225 Y82.725 E0.37911
G1 X76.830 Y82.725 E0.19815
G1 X77.000 Y83.328 E0.01632
G1 X77.315 Y84.074 E0.02112
G1 X77.720 Y84.777 E0.02112
G1 X78.208 Y85.424 E0.02112
```

```
G1 X78.771 Y86.006 E0.02112
G1 X79.402 Y86.514 E0.02112
G1 X80.091 Y86.941 E0.02112
G1 X80.827 Y87.281 E0.02112
G1 X81.599 Y87.526 E0.02112
G1 X82.396 Y87.675 E0.02112
G1 X83.198 Y87.725 E0.02093
G1 X90.715 Y87.725 E0.19586
M204 S1250
G1 X90.574 Y88.071 F9000.000
G1 E-2.24000 F2400.000
;WIPE_START
G1 F7200.000
G1 X90.753 Y90.605 E-0.91200
;WIPE_END
G1 E-0.04800 F2400.000
G1 Z0.700 F9000.000
G1 X90.232 Y89.115
G1 Z0.500
G1 E3.20000 F1500.000
M204 S1000
;TYPE:Solid infill
;WIDTH:0.450839
G1 F4800.000
G1 X89.573 Y88.456 E0.02431
G1 X88.981 Y88.456 E0.01546
G1 X90.044 Y89.519 E0.03923
G1 X90.044 Y90.111 E0.01546
G1 X88.389 Y88.456 E0.06109
```

```
G1 X87.797 Y88.456 E0.01546
G1 X90.044 Y90.703 E0.08295
G1 X90.044 Y91.295 E0.01546
G1 X87.205 Y88.456 E0.10481
G1 X86.613 Y88.456 E0.01546
G1 X89.701 Y91.544 E0.11400
G1 X89.109 Y91.544 E0.01546
G1 X86.021 Y88.456 E0.11400
G1 X85.429 Y88.456 E0.01546
G1 X88.517 Y91.544 E0.11400
G1 X87.925 Y91.544 E0.01546
G1 X84.837 Y88.456 E0.11400
G1 X84.245 Y88.456 E0.01546
G1 X87.333 Y91.544 E0.11400
G1 X86.741 Y91.544 E0.01546
G1 X83.653 Y88.456 E0.11400
G1 X83.053 Y88.449 E0.01566
G1 X86.149 Y91.544 E0.11428
G1 X85.556 Y91.544 E0.01546
G1 X82.422 Y88.410 E0.11572
G1 X81.712 Y88.291 E0.01880
G1 X84.964 Y91.544 E0.12009
G1 X84.372 Y91.544 E0.01546
G1 X80.899 Y88.071 E0.12823
G1 X80.562 Y87.964 E0.00923
G1 X79.890 Y87.654 E0.01931
G1 X83.780 Y91.544 E0.14362
G1 X83.188 Y91.544 E0.01546
G1 X75.101 Y83.456 E0.29862
```

```
G1 X75.693 Y83.456 E0.01546
G1 X77.202 Y84.966 E0.05573
M204 S1250
G1 E-2.24000 F2400.000
;WIPE_START
G1 F7200.000
G1 X75.693 Y83.456 E-0.67595
G1 X75.101 Y83.456 E-0.18749
G1 X75.209 Y83.565 E-0.04857
;WIPE_END
G1 E-0.04800 F2400.000
G1 Z0.700 F9000.000
G1 X82.807 Y91.755
G1 Z0.500
G1 E3.20000 F1500.000
M204 S1000
G1 F4800.000
G1 X74.508 Y83.456 E0.30641
G1 X73.916 Y83.456 E0.01546
G1 X82.097 Y91.637 E0.30204
G1 X81.598 Y91.730 E0.01325
G1 X73.324 Y83.456 E0.30549
G1 X72.732 Y83.456 E0.01546
G1 X81.131 Y91.855 E0.31012
G1 X80.682 Y91.998 E0.01230
G1 X72.140 Y83.456 E0.31540
G1 X71.548 Y83.456 E0.01546
G1 X80.265 Y92.173 E0.32186
G1 X79.860 Y92.360 E0.01165
```

```
G1 X70.956 Y83.456 E0.32875
G1 X70.364 Y83.456 E0.01546
G1 X79.483 Y92.575 E0.33670
G1 X79.118 Y92.802 E0.01123
G1 X69.956 Y83.640 E0.33826
G1 X69.956 Y84.232 E0.01546
G1 X78.775 Y93.052 E0.32563
G1 X78.448 Y93.316 E0.01099
G1 X69.956 Y84.824 E0.31353
G1 X69.956 Y85.416 E0.01546
G1 X78.137 Y93.598 E0.30207
G1 X77.846 Y93.899 E0.01093
G1 X69.956 Y86.009 E0.29132
G1 X69.956 Y86.601 E0.01546
G1 X77.567 Y94.211 E0.28101
G1 X77.313 Y94.549 E0.01104
G1 X69.956 Y87.193 E0.27162
G1 X69.956 Y87.785 E0.01546
G1 X77.066 Y94.894 E0.26250
G1 X76.849 Y95.270 E0.01132
G1 X69.956 Y88.377 E0.25451
G1 X69.956 Y88.969 E0.01546
G1 X76.638 Y95.651 E0.24671
G1 X76.462 Y96.067 E0.01180
G1 X69.956 Y89.561 E0.24022
G1 X69.956 Y90.153 E0.01546
G1 X76.292 Y96.489 E0.23395
G1 X76.277 Y96.544 E0.00148
G1 X75.755 Y96.544 E0.01363
```

```
G1 X69.956 Y90.745 E0.21411
G1 X69.956 Y91.337 E0.01546
G1 X75.163 Y96.544 E0.19225
G1 X74.571 Y96.544 E0.01546
G1 X69.956 Y91.929 E0.17039
G1 X69.956 Y92.521 E0.01546
G1 X73.979 Y96.544 E0.14853
G1 X73.387 Y96.544 E0.01546
G1 X69.956 Y93.113 E0.12666
G1 X69.956 Y93.705 E0.01546
G1 X72.795 Y96.544 E0.10480
G1 X72.203 Y96.544 E0.01546
G1 X69.956 Y94.297 E0.08294
G1 X69.956 Y94.889 E0.01546
G1 X71.611 Y96.544 E0.06108
G1 X71.018 Y96.544 E0.01546
G1 X69.956 Y95.482 E0.03922
G1 X69.956 Y96.074 E0.01546
G1 X70.614 Y96.732 E0.02431
M204 S1250
; stop printing object tpu print.STL id:1 copy 0
; printing object tpu print.STL id:5 copy 0
G1 E-2.24000 F2400.000
;WIPE_START
G1 F7200.000
G1 X69.956 Y96.074 E-0.29480
G1 X69.956 Y95.482 E-0.18749
G1 X70.916 Y96.441 E-0.42971
;WIPE_END
```

```
G1 E-0.04800 F2400.000
G1 Z0.700 F9000.000
G1 X69.694 Y74.665
G1 Z0.500
G1 E3.20000 F1500.000
M204 S800
;TYPE:Perimeter
;WIDTH:0.45
G1 F2400.000
G1 X69.694 Y60.950 E0.35734
G1 X76.565 Y60.950 E0.17903
G1 X76.657 Y61.274 E0.00876
G1 X76.992 Y62.068 E0.02246
G1 X77.422 Y62.815 E0.02246
G1 X77.941 Y63.503 E0.02246
G1 X78.540 Y64.123 E0.02246
G1 X79.211 Y64.664 E0.02246
G1 X79.944 Y65.118 E0.02246
G1 X80.727 Y65.478 E0.02246
G1 X81.548 Y65.740 E0.02246
G1 X82.396 Y65.899 E0.02246
G1 X83.236 Y65.950 E0.02194
G1 X90.408 Y65.950 E0.18688
G1 X90.408 Y69.665 E0.09678
G1 X83.236 Y69.665 E0.18688
G1 X82.396 Y69.717 E0.02194
G1 X81.548 Y69.875 E0.02246
G1 X80.727 Y70.137 E0.02246
G1 X79.944 Y70.497 E0.02246
```

```
G1 X79.211 Y70.952 E0.02246
G1 X78.540 Y71.493 E0.02246
G1 X77.941 Y72.112 E0.02246
G1 X77.422 Y72.800 E0.02246
G1 X76.992 Y73.547 E0.02246
G1 X76.657 Y74.341 E0.02246
G1 X76.565 Y74.665 E0.00876
G1 X69.754 Y74.665 E0.17747
M204 S1250
G1 X69.276 Y75.083 F9000.000
M204 S800
;TYPE:External perimeter
G1 F1800.000
G1 X69.276 Y60.533 E0.37911
G1 X76.881 Y60.533 E0.19815
G1 X77.052 Y61.135 E0.01632
G1 X77.367 Y61.882 E0.02112
G1 X77.771 Y62.584 E0.02112
G1 X78.259 Y63.231 E0.02112
G1 X78.823 Y63.814 E0.02112
G1 X79.454 Y64.322 E0.02112
G1 X80.142 Y64.749 E0.02112
G1 X80.878 Y65.088 E0.02112
G1 X81.651 Y65.334 E0.02112
G1 X82.447 Y65.483 E0.02112
G1 X83.249 Y65.533 E0.02093
G1 X90.826 Y65.533 E0.19743
G1 X90.826 Y70.083 E0.11855
G1 X83.249 Y70.083 E0.19743
```

```
M73 P52 R32
G1 X82.447 Y70.132 E0.02093
G1 X81.651 Y70.281 E0.02112
G1 X80.878 Y70.527 E0.02112
G1 X80.142 Y70.866 E0.02112
G1 X79.454 Y71.293 E0.02112
G1 X78.823 Y71.802 E0.02112
G1 X78.259 Y72.384 E0.02112
G1 X77.771 Y73.031 E0.02112
G1 X77.367 Y73.733 E0.02112
G1 X77.052 Y74.480 E0.02112
G1 X76.881 Y75.083 E0.01632
G1 X69.336 Y75.083 E0.19659
M204 S1250
G1 X69.467 Y74.731 F9000.000
G1 E-2.24000 F2400.000
;WIPE_START
G1 F7200.000
G1 X69.324 Y72.203 E-0.91200
;WIPE_END
G1 E-0.04800 F2400.000
G1 Z0.700 F9000.000
G1 X70.666 Y74.539
G1 Z0.500
G1 E3.20000 F1500.000
M204 S1000
;TYPE:Solid infill
;WIDTH:0.450839
G1 F4800.000
```

```
G1 X70.007 Y73.881 E0.02431
G1 X70.007 Y73.289 E0.01546
G1 X71.070 Y74.351 E0.03922
G1 X71.662 Y74.351 E0.01546
G1 X70.007 Y72.697 E0.06108
G1 X70.007 Y72.105 E0.01546
G1 X72.254 Y74.351 E0.08294
G1 X72.846 Y74.351 E0.01546
G1 X70.007 Y71.513 E0.10480
G1 X70.007 Y70.921 E0.01546
G1 X73.438 Y74.351 E0.12666
G1 X74.030 Y74.351 E0.01546
G1 X70.007 Y70.329 E0.14853
G1 X70.007 Y69.737 E0.01546
G1 X74.622 Y74.351 E0.17039
G1 X75.214 Y74.351 E0.01546
G1 X70.007 Y69.145 E0.19225
G1 X70.007 Y68.553 E0.01546
G1 X75.806 Y74.351 E0.21411
G1 X76.328 Y74.351 E0.01363
G1 X76.344 Y74.297 E0.00148
G1 X70.007 Y67.961 E0.23395
G1 X70.007 Y67.369 E0.01546
G1 X76.513 Y73.875 E0.24022
G1 X76.689 Y73.458 E0.01180
G1 X70.007 Y66.777 E0.24671
G1 X70.007 Y66.184 E0.01546
G1 X76.900 Y73.078 E0.25451
G1 X77.117 Y72.702 E0.01132
```

```
G1 X70.007 Y65.592 E0.26250
G1 X70.007 Y65.000 E0.01546
G1 X77.364 Y72.357 E0.27162
G1 X77.618 Y72.019 E0.01104
G1 X70.007 Y64.408 E0.28101
G1 X70.007 Y63.816 E0.01546
G1 X77.897 Y71.706 E0.29132
G1 X78.189 Y71.405 E0.01093
G1 X70.007 Y63.224 E0.30207
G1 X70.007 Y62.632 E0.01546
G1 X78.499 Y71.124 E0.31353
G1 X78.827 Y70.859 E0.01099
G1 X70.007 Y62.040 E0.32563
G1 X70.007 Y61.448 E0.01546
G1 X79.169 Y70.609 E0.33826
G1 X79.534 Y70.383 E0.01123
G1 X70.415 Y61.264 E0.33670
G1 X71.007 Y61.264 E0.01546
G1 X79.911 Y70.168 E0.32875
G1 X80.316 Y69.981 E0.01165
G1 X71.599 Y61.264 E0.32186
G1 X72.191 Y61.264 E0.01546
G1 X80.733 Y69.806 E0.31540
G1 X81.183 Y69.663 E0.01230
G1 X72.783 Y61.264 E0.31012
G1 X73.376 Y61.264 E0.01546
G1 X81.649 Y69.538 E0.30549
G1 X82.148 Y69.444 E0.01325
G1 X73.968 Y61.264 E0.30204
```

```
G1 X74.560 Y61.264 E0.01546
G1 X82.858 Y69.563 E0.30641
M204 S1250
G1 E-2.24000 F2400.000
;WIPE_START
G1 F7200.000
G1 X80.822 Y67.526 E-0.91200
;WIPE_END
G1 E-0.04800 F2400.000
G1 Z0.700 F9000.000
G1 X90.283 Y66.922
G1 Z0.500
G1 E3.20000 F1500.000
M204 S1000
G1 F4800.000
G1 X89.625 Y66.264 E0.02431
G1 X89.033 Y66.264 E0.01546
G1 X90.095 Y67.326 E0.03923
G1 X90.095 Y67.918 E0.01546
G1 X88.441 Y66.264 E0.06109
G1 X87.848 Y66.264 E0.01546
G1 X90.095 Y68.510 E0.08295
G1 X90.095 Y69.102 E0.01546
G1 X87.256 Y66.264 E0.10481
G1 X86.664 Y66.264 E0.01546
G1 X89.752 Y69.351 E0.11400
G1 X89.160 Y69.351 E0.01546
G1 X86.072 Y66.264 E0.11400
G1 X85.480 Y66.264 E0.01546
```

```
G1 X88.568 Y69.351 E0.11400
G1 X87.976 Y69.351 E0.01546
G1 X84.888 Y66.264 E0.11400
G1 X84.296 Y66.264 E0.01546
G1 X87.384 Y69.351 E0.11400
G1 X86.792 Y69.351 E0.01546
G1 X83.704 Y66.264 E0.11400
G1 X83.104 Y66.256 E0.01566
G1 X86.200 Y69.351 E0.11428
G1 X85.608 Y69.351 E0.01546
G1 X82.473 Y66.217 E0.11572
G1 X81.763 Y66.099 E0.01880
G1 X85.016 Y69.351 E0.12009
G1 X84.423 Y69.351 E0.01546
G1 X80.950 Y65.878 E0.12823
G1 X80.613 Y65.771 E0.00923
G1 X79.942 Y65.462 E0.01931
G1 X83.831 Y69.351 E0.14362
G1 X83.239 Y69.351 E0.01546
G1 X75.152 Y61.264 E0.29862
G1 X75.744 Y61.264 E0.01546
G1 X77.253 Y62.773 E0.05573
M204 S1250
; stop printing object tpu print.STL id:5 copy 0
; printing object tpu print.STL id:9 copy 0
G1 E-2.24000 F2400.000
;WIPE_START
G1 F7200.000
G1 X75.744 Y61.264 E-0.67595
```

```
G1 X75.152 Y61.264 E-0.18749
G1 X75.260 Y61.372 E-0.04857
;WIPE_END
G1 E-0.04800 F2400.000
G1 Z0.700 F9000.000
G1 X76.558 Y54.716
G1 Z0.500
G1 E3.20000 F1500.000
M204 S800
;TYPE:Perimeter
;WIDTH:0.45
G1 F2400.000
G1 X69.686 Y54.716 E0.17903
G1 X69.686 Y41.002 E0.35734
G1 X76.558 Y41.002 E0.17903
G1 X76.649 Y41.325 E0.00876
G1 X76.984 Y42.119 E0.02246
G1 X77.414 Y42.866 E0.02246
G1 X77.933 Y43.555 E0.02246
G1 X78.533 Y44.174 E0.02246
G1 X79.204 Y44.715 E0.02246
G1 X79.937 Y45.169 E0.02246
G1 X80.720 Y45.530 E0.02246
G1 X81.541 Y45.791 E0.02246
G1 X82.388 Y45.950 E0.02246
G1 X83.229 Y46.002 E0.02194
G1 X90.401 Y46.002 E0.18688
G1 X90.401 Y49.716 E0.09678
G1 X83.229 Y49.716 E0.18688
```

```
G1 X82.388 Y49.768 E0.02194
G1 X81.541 Y49.926 E0.02246
G1 X80.720 Y50.188 E0.02246
G1 X79.937 Y50.549 E0.02246
G1 X79.204 Y51.003 E0.02246
G1 X78.533 Y51.544 E0.02246
G1 X77.933 Y52.163 E0.02246
G1 X77.414 Y52.851 E0.02246
G1 X76.984 Y53.598 E0.02246
G1 X76.649 Y54.392 E0.02246
G1 X76.574 Y54.658 E0.00720
M204 S1250
G1 X76.874 Y55.134 F9000.000
M204 S800
;TYPE:External perimeter
G1 F1800.000
G1 X69.269 Y55.134 E0.19815
G1 X69.269 Y40.584 E0.37911
G1 X76.874 Y40.584 E0.19815
G1 X77.044 Y41.186 E0.01632
G1 X77.359 Y41.933 E0.02112
G1 X77.764 Y42.635 E0.02112
G1 X78.251 Y43.282 E0.02112
G1 X78.815 Y43.865 E0.02112
G1 X79.446 Y44.373 E0.02112
G1 X80.135 Y44.800 E0.02112
G1 X80.871 Y45.139 E0.02112
G1 X81.643 Y45.385 E0.02112
G1 X82.440 Y45.534 E0.02112
```

```
G1 X83.242 Y45.584 E0.02093
G1 X90.819 Y45.584 E0.19743
G1 X90.819 Y50.134 E0.11855
G1 X83.242 Y50.134 E0.19743
G1 X82.440 Y50.183 E0.02093
G1 X81.643 Y50.332 E0.02112
G1 X80.871 Y50.578 E0.02112
G1 X80.135 Y50.917 E0.02112
G1 X79.446 Y51.344 E0.02112
G1 X78.815 Y51.853 E0.02112
G1 X78.251 Y52.435 E0.02112
G1 X77.764 Y53.082 E0.02112
G1 X77.359 Y53.784 E0.02112
G1 X77.044 Y54.531 E0.02112
G1 X76.890 Y55.076 E0.01476
M204 S1250
G1 X76.552 Y54.895 F9000.000
G1 E-2.24000 F2400.000
;WIPE_START
G1 F7200.000
G1 X74.010 Y55.098 E-0.91200
;WIPE_END
G1 E-0.04800 F2400.000
G1 Z0.700 F9000.000
G1 X70.658 Y54.591
G1 Z0.500
G1 E3.20000 F1500.000
M204 S1000
;TYPE:Solid infill
```

```
;WIDTH:0.450839
G1 F4800.000
G1 X70.000 Y53.932 E0.02431
G1 X70.000 Y53.340 E0.01546
G1 X71.062 Y54.403 E0.03922
G1 X71.654 Y54.403 E0.01546
G1 X70.000 Y52.748 E0.06108
G1 X70.000 Y52.156 E0.01546
G1 X72.246 Y54.403 E0.08294
G1 X72.838 Y54.403 E0.01546
G1 X70.000 Y51.564 E0.10480
G1 X70.000 Y50.972 E0.01546
G1 X73.430 Y54.403 E0.12666
G1 X74.022 Y54.403 E0.01546
G1 X70.000 Y50.380 E0.14853
G1 X70.000 Y49.788 E0.01546
G1 X74.615 Y54.403 E0.17039
G1 X75.207 Y54.403 E0.01546
G1 X70.000 Y49.196 E0.19225
G1 X70.000 Y48.604 E0.01546
G1 X75.799 Y54.403 E0.21411
G1 X76.321 Y54.403 E0.01363
G1 X76.336 Y54.348 E0.00148
G1 X70.000 Y48.012 E0.23395
G1 X70.000 Y47.420 E0.01546
G1 X76.506 Y53.926 E0.24022
G1 X76.682 Y53.509 E0.01180
G1 X70.000 Y46.828 E0.24671
G1 X70.000 Y46.235 E0.01546
```

```
G1 X76.893 Y53.129 E0.25451
G1 X77.109 Y52.753 E0.01132
G1 X70.000 Y45.643 E0.26250
G1 X70.000 Y45.051 E0.01546
G1 X77.356 Y52.408 E0.27162
G1 X77.611 Y52.070 E0.01104
G1 X70.000 Y44.459 E0.28101
G1 X70.000 Y43.867 E0.01546
G1 X77.890 Y51.757 E0.29132
G1 X78.181 Y51.456 E0.01093
G1 X70.000 Y43.275 E0.30207
G1 X70.000 Y42.683 E0.01546
G1 X78.491 Y51.175 E0.31353
G1 X78.819 Y50.910 E0.01099
G1 X70.000 Y42.091 E0.32563
G1 X70.000 Y41.499 E0.01546
G1 X79.161 Y50.660 E0.33826
G1 X79.527 Y50.434 E0.01123
G1 X70.408 Y41.315 E0.33670
G1 X71.000 Y41.315 E0.01546
G1 X79.904 Y50.219 E0.32875
G1 X80.309 Y50.032 E0.01165
G1 X71.592 Y41.315 E0.32186
G1 X72.184 Y41.315 E0.01546
G1 X80.726 Y49.857 E0.31540
G1 X81.175 Y49.714 E0.01230
G1 X72.776 Y41.315 E0.31012
G1 X73.368 Y41.315 E0.01546
G1 X81.642 Y49.589 E0.30549
```

```
G1 X82.141 Y49.495 E0.01325
G1 X73.960 Y41.315 E0.30204
G1 X74.552 Y41.315 E0.01546
G1 X82.851 Y49.614 E0.30641
M204 S1250
G1 E-2.24000 F2400.000
;WIPE_START
G1 F7200.000
G1 X80.814 Y47.577 E-0.91200
;WIPE_END
G1 E-0.04800 F2400.000
G1 Z0.700 F9000.000
G1 X90.276 Y46.973
G1 Z0.500
G1 E3.20000 F1500.000
M204 S1000
G1 F4800.000
G1 X89.617 Y46.315 E0.02431
G1 X89.025 Y46.315 E0.01546
G1 X90.087 Y47.377 E0.03923
G1 X90.087 Y47.969 E0.01546
G1 X88.433 Y46.315 E0.06109
G1 X87.841 Y46.315 E0.01546
G1 X90.087 Y48.561 E0.08295
G1 X90.087 Y49.153 E0.01546
G1 X87.249 Y46.315 E0.10481
G1 X86.657 Y46.315 E0.01546
G1 X89.745 Y49.403 E0.11400
G1 X89.152 Y49.403 E0.01546
```

```
G1 X86.065 Y46.315 E0.11400
G1 X85.473 Y46.315 E0.01546
G1 X88.560 Y49.403 E0.11400
G1 X87.968 Y49.403 E0.01546
G1 X84.881 Y46.315 E0.11400
G1 X84.289 Y46.315 E0.01546
G1 X87.376 Y49.403 E0.11400
G1 X86.784 Y49.403 E0.01546
G1 X83.697 Y46.315 E0.11400
G1 X83.097 Y46.307 E0.01566
G1 X86.192 Y49.403 E0.11428
G1 X85.600 Y49.403 E0.01546
G1 X82.466 Y46.268 E0.11572
G1 X81.756 Y46.150 E0.01880
G1 X85.008 Y49.403 E0.12009
G1 X84.416 Y49.403 E0.01546
G1 X80.943 Y45.930 E0.12823
G1 X80.606 Y45.822 E0.00923
G1 X79.934 Y45.513 E0.01931
G1 X83.824 Y49.403 E0.14362
G1 X83.232 Y49.403 E0.01546
G1 X75.144 Y41.315 E0.29862
G1 X75.736 Y41.315 E0.01546
G1 X77.246 Y42.824 E0.05573
M204 S1250
; stop printing object tpu print.STL id:9 copy 0
; printing object Petg print.STL id:8 copy 0
; stop printing object Petg print.STL id:8 copy 0
; printing object Petg print.STL id:4 copy 0
```

```
; stop printing object Petg print.STL id:4 copy 0
; printing object tpu print.STL id:29 copy 0
G1 E-2.24000 F2400.000
;WIPE_START
G1 F7200.000
G1 X75.736 Y41.315 E-0.67595
G1 X75.144 Y41.315 E-0.18749
G1 X75.253 Y41.423 E-0.04857
;WIPE_END
G1 E-0.04800 F2400.000
G1 Z0.700 F9000.000
G1 X120.337 Y41.177
G1 Z0.500
G1 E3.20000 F1500.000
M204 S800
;TYPE:Perimeter
;WIDTH:0.45
G1 F2400.000
G1 X127.208 Y41.177 E0.17903
G1 X127.299 Y41.500 E0.00876
G1 X127.634 Y42.294 E0.02246
G1 X128.065 Y43.041 E0.02246
G1 X128.583 Y43.730 E0.02246
G1 X129.183 Y44.349 E0.02246
G1 X129.854 Y44.890 E0.02246
G1 X130.587 Y45.344 E0.02246
G1 X131.370 Y45.705 E0.02246
M73 P53 R32
G1 X132.191 Y45.966 E0.02246
```

```
G1 X133.038 Y46.125 E0.02246
G1 X133.879 Y46.177 E0.02194
G1 X141.051 Y46.177 E0.18688
G1 X141.051 Y49.891 E0.09678
G1 X133.879 Y49.891 E0.18688
G1 X133.038 Y49.943 E0.02194
G1 X132.191 Y50.101 E0.02246
G1 X131.370 Y50.363 E0.02246
G1 X130.587 Y50.724 E0.02246
G1 X129.854 Y51.178 E0.02246
G1 X129.183 Y51.719 E0.02246
G1 X128.583 Y52.338 E0.02246
G1 X128.065 Y53.026 E0.02246
G1 X127.634 Y53.773 E0.02246
G1 X127.299 Y54.567 E0.02246
G1 X127.208 Y54.891 E0.00876
G1 X120.337 Y54.891 E0.17903
G1 X120.337 Y41.237 E0.35578
M204 S1250
G1 X119.919 Y40.759 F9000.000
M204 S800
;TYPE:External perimeter
G1 F1800.000
G1 X127.524 Y40.759 E0.19815
G1 X127.694 Y41.361 E0.01632
G1 X128.009 Y42.108 E0.02112
G1 X128.414 Y42.810 E0.02112
G1 X128.902 Y43.458 E0.02112
G1 X129.465 Y44.040 E0.02112
```

```
G1 X130.096 Y44.548 E0.02112
G1 X130.785 Y44.975 E0.02112
G1 X131.521 Y45.314 E0.02112
G1 X132.293 Y45.560 E0.02112
G1 X133.090 Y45.709 E0.02112
G1 X133.892 Y45.759 E0.02093
G1 X141.469 Y45.759 E0.19743
G1 X141.469 Y50.309 E0.11855
G1 X133.892 Y50.309 E0.19743
G1 X133.090 Y50.358 E0.02093
G1 X132.293 Y50.507 E0.02112
G1 X131.521 Y50.753 E0.02112
G1 X130.785 Y51.092 E0.02112
G1 X130.096 Y51.519 E0.02112
G1 X129.465 Y52.028 E0.02112
G1 X128.902 Y52.610 E0.02112
G1 X128.414 Y53.257 E0.02112
G1 X128.009 Y53.959 E0.02112
G1 X127.694 Y54.706 E0.02112
G1 X127.524 Y55.309 E0.01632
G1 X119.919 Y55.309 E0.19815
G1 X119.919 Y40.819 E0.37755
M204 S1250
G1 X120.293 Y40.901 F9000.000
G1 E-2.24000 F2400.000
;WIPE_START
G1 F7200.000
G1 X122.799 Y40.796 E-0.91200
;WIPE_END
```

```
G1 E-0.04800 F2400.000
G1 Z0.700 F9000.000
G1 X127.896 Y42.999
G1 Z0.500
G1 E3.20000 F1500.000
M204 S1000
;TYPE:Solid infill
;WIDTH:0.450839
G1 F4800.000
G1 X126.387 Y41.490 E0.05573
G1 X125.794 Y41.490 E0.01546
G1 X133.882 Y49.578 E0.29862
G1 X134.474 Y49.578 E0.01546
G1 X130.584 Y45.688 E0.14362
G1 X131.256 Y45.997 E0.01931
G1 X131.593 Y46.105 E0.00923
G1 X135.066 Y49.578 E0.12823
G1 X135.658 Y49.578 E0.01546
G1 X132.406 Y46.325 E0.12009
G1 X133.116 Y46.443 E0.01880
G1 X136.250 Y49.578 E0.11572
G1 X136.842 Y49.578 E0.01546
G1 X133.747 Y46.482 E0.11428
G1 X134.347 Y46.490 E0.01566
G1 X137.434 Y49.578 E0.11400
G1 X138.027 Y49.578 E0.01546
G1 X134.939 Y46.490 E0.11400
G1 X135.531 Y46.490 E0.01546
G1 X138.619 Y49.578 E0.11400
```

```
G1 X139.211 Y49.578 E0.01546
G1 X136.123 Y46.490 E0.11400
G1 X136.715 Y46.490 E0.01546
G1 X139.803 Y49.578 E0.11400
G1 X140.395 Y49.578 E0.01546
G1 X137.307 Y46.490 E0.11400
G1 X137.899 Y46.490 E0.01546
G1 X140.738 Y49.329 E0.10481
G1 X140.738 Y48.736 E0.01546
G1 X138.491 Y46.490 E0.08295
G1 X139.083 Y46.490 E0.01546
G1 X140.738 Y48.144 E0.06109
G1 X140.738 Y47.552 E0.01546
G1 X139.675 Y46.490 E0.03923
G1 X140.267 Y46.490 E0.01546
G1 X140.926 Y47.148 E0.02431
M204 S1250
G1 E-2.24000 F2400.000
;WIPE_START
G1 F7200.000
G1 X140.267 Y46.490 E-0.29484
G1 X139.675 Y46.490 E-0.18749
G1 X140.635 Y47.449 E-0.42968
;WIPE_END
G1 E-0.04800 F2400.000
G1 Z0.700 F9000.000
G1 X133.501 Y49.789
G1 Z0.500
G1 E3.20000 F1500.000
```

```
M204 S1000
G1 F4800.000
G1 X125.202 Y41.490 E0.30641
G1 X124.610 Y41.490 E0.01546
G1 X132.791 Y49.670 E0.30204
G1 X132.292 Y49.764 E0.01325
G1 X124.018 Y41.490 E0.30549
G1 X123.426 Y41.490 E0.01546
G1 X131.825 Y49.889 E0.31012
G1 X131.376 Y50.032 E0.01230
G1 X122.834 Y41.490 E0.31540
G1 X122.242 Y41.490 E0.01546
G1 X130.959 Y50.207 E0.32186
G1 X130.554 Y50.394 E0.01165
G1 X121.650 Y41.490 E0.32875
G1 X121.058 Y41.490 E0.01546
G1 X130.177 Y50.609 E0.33670
G1 X129.812 Y50.836 E0.01123
G1 X120.650 Y41.674 E0.33826
G1 X120.650 Y42.266 E0.01546
G1 X129.469 Y51.085 E0.32563
G1 X129.142 Y51.350 E0.01099
G1 X120.650 Y42.858 E0.31353
G1 X120.650 Y43.450 E0.01546
G1 X128.831 Y51.631 E0.30207
G1 X128.540 Y51.932 E0.01093
G1 X120.650 Y44.042 E0.29132
G1 X120.650 Y44.634 E0.01546
G1 X128.261 Y52.245 E0.28101
```

```
G1 X128.006 Y52.583 E0.01104
G1 X120.650 Y45.226 E0.27162
G1 X120.650 Y45.819 E0.01546
G1 X127.760 Y52.928 E0.26250
G1 X127.543 Y53.304 E0.01132
G1 X120.650 Y46.411 E0.25451
G1 X120.650 Y47.003 E0.01546
G1 X127.332 Y53.684 E0.24671
G1 X127.156 Y54.101 E0.01180
G1 X120.650 Y47.595 E0.24022
G1 X120.650 Y48.187 E0.01546
G1 X126.986 Y54.523 E0.23395
G1 X126.971 Y54.578 E0.00148
G1 X126.449 Y54.578 E0.01363
G1 X120.650 Y48.779 E0.21411
G1 X120.650 Y49.371 E0.01546
G1 X125.857 Y54.578 E0.19225
G1 X125.265 Y54.578 E0.01546
G1 X120.650 Y49.963 E0.17039
G1 X120.650 Y50.555 E0.01546
G1 X124.673 Y54.578 E0.14853
G1 X124.081 Y54.578 E0.01546
G1 X120.650 Y51.147 E0.12666
G1 X120.650 Y51.739 E0.01546
G1 X123.489 Y54.578 E0.10480
G1 X122.897 Y54.578 E0.01546
G1 X120.650 Y52.331 E0.08294
G1 X120.650 Y52.923 E0.01546
G1 X122.304 Y54.578 E0.06108
```

```
G1 X121.712 Y54.578 E0.01546
G1 X120.650 Y53.515 E0.03922
G1 X120.650 Y54.107 E0.01546
G1 X121.308 Y54.766 E0.02431
M204 S1250
; stop printing object tpu print.STL id:29 copy 0
; printing object tpu print.STL id:25 copy 0
G1 E-2.24000 F2400.000
;WIPE_START
G1 F7200.000
G1 X120.650 Y54.107 E-0.29480
G1 X120.650 Y53.515 E-0.18749
G1 X121.610 Y54.475 E-0.42971
;WIPE_END
G1 E-0.04800 F2400.000
G1 Z0.700 F9000.000
G1 X120.344 Y61.126
G1 Z0.500
G1 E3.20000 F1500.000
M204 S800
;TYPE:Perimeter
;WIDTH:0.45
G1 F2400.000
G1 X127.215 Y61.126 E0.17903
G1 X127.307 Y61.449 E0.00876
G1 X127.642 Y62.243 E0.02246
G1 X128.072 Y62.990 E0.02246
G1 X128.591 Y63.679 E0.02246
G1 X129.191 Y64.298 E0.02246
```

```
G1 X129.862 Y64.839 E0.02246
G1 X130.594 Y65.293 E0.02246
G1 X131.377 Y65.654 E0.02246
G1 X132.199 Y65.915 E0.02246
G1 X133.046 Y66.074 E0.02246
G1 X133.886 Y66.126 E0.02194
G1 X141.059 Y66.126 E0.18688
G1 X141.059 Y69.840 E0.09678
G1 X133.886 Y69.840 E0.18688
G1 X133.046 Y69.892 E0.02194
G1 X132.199 Y70.050 E0.02246
G1 X131.377 Y70.312 E0.02246
G1 X130.594 Y70.673 E0.02246
G1 X129.862 Y71.127 E0.02246
G1 X129.191 Y71.668 E0.02246
G1 X128.591 Y72.287 E0.02246
G1 X128.072 Y72.975 E0.02246
G1 X127.642 Y73.722 E0.02246
G1 X127.307 Y74.516 E0.02246
G1 X127.215 Y74.840 E0.00876
G1 X120.344 Y74.840 E0.17903
G1 X120.344 Y61.186 E0.35578
M204 S1250
G1 X119.926 Y60.708 F9000.000
M204 S800
;TYPE:External perimeter
G1 F1800.000
G1 X127.531 Y60.708 E0.19815
G1 X127.702 Y61.310 E0.01632
```

```
G1 X128.017 Y62.057 E0.02112
G1 X128.421 Y62.759 E0.02112
G1 X128.909 Y63.406 E0.02112
G1 X129.473 Y63.989 E0.02112
G1 X130.104 Y64.497 E0.02112
G1 X130.793 Y64.924 E0.02112
G1 X131.529 Y65.263 E0.02112
G1 X132.301 Y65.509 E0.02112
G1 X133.097 Y65.658 E0.02112
G1 X133.899 Y65.708 E0.02093
G1 X141.476 Y65.708 E0.19743
G1 X141.476 Y70.258 E0.11855
G1 X133.899 Y70.258 E0.19743
G1 X133.097 Y70.307 E0.02093
G1 X132.301 Y70.456 E0.02112
G1 X131.529 Y70.702 E0.02112
G1 X130.793 Y71.041 E0.02112
G1 X130.104 Y71.468 E0.02112
G1 X129.473 Y71.977 E0.02112
G1 X128.909 Y72.559 E0.02112
G1 X128.421 Y73.206 E0.02112
G1 X128.017 Y73.908 E0.02112
G1 X127.702 Y74.655 E0.02112
G1 X127.531 Y75.258 E0.01632
G1 X119.926 Y75.258 E0.19815
G1 X119.926 Y60.768 E0.37755
M204 S1250
G1 X120.300 Y60.850 F9000.000
G1 E-2.24000 F2400.000
```

```
;WIPE_START
G1 F7200.000
G1 X122.806 Y60.745 E-0.91200
;WIPE_END
G1 E-0.04800 F2400.000
G1 Z0.700 F9000.000
G1 X127.903 Y62.948
G1 Z0.500
G1 E3.20000 F1500.000
M204 S1000
;TYPE:Solid infill
;WIDTH:0.450839
G1 F4800.000
G1 X126.394 Y61.439 E0.05573
G1 X125.802 Y61.439 E0.01546
G1 X133.890 Y69.527 E0.29862
G1 X134.482 Y69.527 E0.01546
G1 X130.592 Y65.637 E0.14362
G1 X131.264 Y65.946 E0.01931
G1 X131.601 Y66.054 E0.00923
G1 X135.074 Y69.527 E0.12823
G1 X135.666 Y69.527 E0.01546
G1 X132.413 Y66.274 E0.12009
G1 X133.124 Y66.392 E0.01880
G1 X136.258 Y69.527 E0.11572
G1 X136.850 Y69.527 E0.01546
G1 X133.755 Y66.431 E0.11428
G1 X134.354 Y66.439 E0.01566
G1 X137.442 Y69.527 E0.11400
```

```
G1 X138.034 Y69.527 E0.01546
G1 X134.946 Y66.439 E0.11400
G1 X135.538 Y66.439 E0.01546
G1 X138.626 Y69.527 E0.11400
G1 X139.218 Y69.527 E0.01546
G1 X136.130 Y66.439 E0.11400
G1 X136.723 Y66.439 E0.01546
G1 X139.810 Y69.527 E0.11400
G1 X140.402 Y69.527 E0.01546
G1 X137.315 Y66.439 E0.11400
G1 X137.907 Y66.439 E0.01546
G1 X140.745 Y69.277 E0.10481
G1 X140.745 Y68.685 E0.01546
G1 X138.499 Y66.439 E0.08295
G1 X139.091 Y66.439 E0.01546
G1 X140.745 Y68.093 E0.06109
G1 X140.745 Y67.501 E0.01546
G1 X139.683 Y66.439 E0.03923
G1 X140.275 Y66.439 E0.01546
G1 X140.933 Y67.097 E0.02431
M204 S1250
G1 E-2.24000 F2400.000
;WIPE_START
G1 F7200.000
G1 X140.275 Y66.439 E-0.29484
G1 X139.683 Y66.439 E-0.18749
G1 X140.642 Y67.398 E-0.42968
;WIPE_END
G1 E-0.04800 F2400.000
```

```
G1 Z0.700 F9000.000
G1 X133.509 Y69.738
G1 Z0.500
G1 E3.20000 F1500.000
M204 S1000
G1 F4800.000
G1 X125.210 Y61.439 E0.30641
G1 X124.618 Y61.439 E0.01546
G1 X132.798 Y69.619 E0.30204
G1 X132.300 Y69.713 E0.01325
G1 X124.026 Y61.439 E0.30549
G1 X123.434 Y61.439 E0.01546
G1 X131.833 Y69.838 E0.31012
G1 X131.384 Y69.981 E0.01230
G1 X122.842 Y61.439 E0.31540
G1 X122.250 Y61.439 E0.01546
G1 X130.967 Y70.156 E0.32186
G1 X130.561 Y70.343 E0.01165
G1 X121.658 Y61.439 E0.32875
G1 X121.065 Y61.439 E0.01546
G1 X130.185 Y70.558 E0.33670
G1 X129.819 Y70.785 E0.01123
G1 X120.658 Y61.623 E0.33826
G1 X120.658 Y62.215 E0.01546
G1 X129.477 Y71.034 E0.32563
G1 X129.149 Y71.299 E0.01099
G1 X120.658 Y62.807 E0.31353
G1 X120.658 Y63.399 E0.01546
G1 X128.839 Y71.580 E0.30207
```

```
G1 X128.548 Y71.881 E0.01093
G1 X120.658 Y63.991 E0.29132
G1 X120.658 Y64.583 E0.01546
G1 X128.268 Y72.194 E0.28101
G1 X128.014 Y72.532 E0.01104
G1 X120.658 Y65.175 E0.27162
G1 X120.658 Y65.767 E0.01546
G1 X127.767 Y72.877 E0.26250
G1 X127.551 Y73.253 E0.01132
G1 X120.658 Y66.360 E0.25451
G1 X120.658 Y66.952 E0.01546
G1 X127.339 Y73.633 E0.24671
G1 X127.164 Y74.050 E0.01180
G1 X120.658 Y67.544 E0.24022
G1 X120.658 Y68.136 E0.01546
G1 X126.994 Y74.472 E0.23395
G1 X126.978 Y74.527 E0.00148
G1 X126.456 Y74.527 E0.01363
G1 X120.658 Y68.728 E0.21411
G1 X120.658 Y69.320 E0.01546
G1 X125.864 Y74.527 E0.19225
G1 X125.272 Y74.527 E0.01546
G1 X120.658 Y69.912 E0.17039
G1 X120.658 Y70.504 E0.01546
G1 X124.680 Y74.527 E0.14853
G1 X124.088 Y74.527 E0.01546
G1 X120.658 Y71.096 E0.12666
G1 X120.658 Y71.688 E0.01546
G1 X123.496 Y74.527 E0.10480
```

```
G1 X122.904 Y74.527 E0.01546
G1 X120.658 Y72.280 E0.08294
G1 X120.658 Y72.872 E0.01546
G1 X122.312 Y74.527 E0.06108
G1 X121.720 Y74.527 E0.01546
G1 X120.658 Y73.464 E0.03922
G1 X120.658 Y74.056 E0.01546
G1 X121.316 Y74.715 E0.02431
M204 S1250
; stop printing object tpu print.STL id:25 copy 0
; printing object Petg print.STL id:24 copy 0
; stop printing object Petg print.STL id:24 copy 0
; printing object Petg print.STL id:28 copy 0
; stop printing object Petg print.STL id:28 copy 0
M106 S38.25
;LAYER_CHANGE
;Z:0.65
;HEIGHT:0.15
;BEFORE_LAYER_CHANGE
G92 E0.0
;0.65


G1 E-2.24000 F2400.000
;WIPE_START
G1 F7200.000
G1 X120.658 Y74.056 E-0.29480
G1 X120.658 Y73.464 E-0.18749
G1 X121.617 Y74.424 E-0.42971
```

```
;WIPE_END
G1 E-0.04800 F2400.000
G1 Z0.700 F9000.000
;AFTER_LAYER_CHANGE
;0.65
; printing object tpu print.STL id:19 copy 0
G1 X41.611 Y49.135
G1 Z0.650
G1 E3.20000 F1500.000
M204 S800
;TYPE:Perimeter
;WIDTH:0.45
G1 F2400.000
G1 X34.439 Y49.135 E0.18688
G1 X33.599 Y49.187 E0.02194
G1 X32.752 Y49.345 E0.02246
G1 X31.930 Y49.607 E0.02246
G1 X31.147 Y49.968 E0.02246
G1 X30.415 Y50.422 E0.02246
G1 X29.743 Y50.963 E0.02246
G1 X29.144 Y51.582 E0.02246
G1 X28.625 Y52.270 E0.02246
G1 X28.195 Y53.017 E0.02246
G1 X27.860 Y53.812 E0.02246
G1 X27.768 Y54.135 E0.00876
G1 X20.897 Y54.135 E0.17903
G1 X20.897 Y40.421 E0.35734
G1 X27.768 Y40.421 E0.17903
G1 X27.860 Y40.744 E0.00876
```

```
G1 X28.195 Y41.538 E0.02246
G1 X28.625 Y42.285 E0.02246
G1 X29.144 Y42.974 E0.02246
G1 X29.743 Y43.593 E0.02246
G1 X30.415 Y44.134 E0.02246
G1 X31.147 Y44.588 E0.02246
G1 X31.930 Y44.949 E0.02246
G1 X32.752 Y45.210 E0.02246
G1 X33.599 Y45.369 E0.02246
G1 X34.439 Y45.421 E0.02194
G1 X41.611 Y45.421 E0.18688
G1 X41.611 Y49.075 E0.09522
M204 S1250
G1 X42.029 Y49.553 F9000.000
M204 S800
;TYPE:External perimeter
G1 F1800.000
G1 X34.452 Y49.553 E0.19743
G1 X33.650 Y49.602 E0.02093
G1 X32.854 Y49.751 E0.02112
G1 X32.082 Y49.997 E0.02112
G1 X31.345 Y50.336 E0.02112
G1 X30.657 Y50.763 E0.02112
G1 X30.026 Y51.272 E0.02112
G1 X29.462 Y51.854 E0.02112
G1 X28.974 Y52.501 E0.02112
G1 X28.570 Y53.203 E0.02112
G1 X28.255 Y53.950 E0.02112
G1 X28.084 Y54.553 E0.01632
```

```
G1 X20.479 Y54.553 E0.19815
G1 X20.479 Y40.003 E0.37911
G1 X28.084 Y40.003 E0.19815
G1 X28.255 Y40.606 E0.01632
G1 X28.570 Y41.352 E0.02112
G1 X28.974 Y42.054 E0.02112
G1 X29.462 Y42.702 E0.02112
G1 X30.026 Y43.284 E0.02112
G1 X30.657 Y43.792 E0.02112
G1 X31.345 Y44.219 E0.02112
G1 X32.082 Y44.558 E0.02112
G1 X32.854 Y44.804 E0.02112
G1 X33.650 Y44.953 E0.02112
G1 X34.452 Y45.003 E0.02093
G1 X42.029 Y45.003 E0.19743
G1 X42.029 Y49.493 E0.11699
M204 S1250
G1 X41.636 Y49.481 F9000.000
G1 E-2.24000 F2400.000
;WIPE_START
G1 F7200.000
G1 X39.149 Y49.516 E-0.91200
;WIPE_END
G1 E-0.04800 F2400.000
G1 Z0.850 F9000.000
G1 X21.869 Y40.546
G1 Z0.650
G1 E3.20000 F1500.000
M204 S1000
```

```
;TYPE:Solid infill
;WIDTH:0.450839
G1 F4800.000
G1 X21.210 Y41.204 E0.02431
G1 X21.210 Y41.796 E0.01546
G1 X22.273 Y40.734 E0.03922
G1 X22.865 Y40.734 E0.01546
G1 X21.210 Y42.388 E0.06108
G1 X21.210 Y42.980 E0.01546
G1 X23.457 Y40.734 E0.08294
G1 X24.049 Y40.734 E0.01546
G1 X21.210 Y43.573 E0.10480
G1 X21.210 Y44.165 E0.01546
G1 X24.641 Y40.734 E0.12666
G1 X25.233 Y40.734 E0.01546
G1 X21.210 Y44.757 E0.14853
G1 X21.210 Y45.349 E0.01546
M73 P53 R31
G1 X25.825 Y40.734 E0.17039
G1 X26.417 Y40.734 E0.01546
G1 X21.210 Y45.941 E0.19225
G1 X21.210 Y46.533 E0.01546
G1 X27.009 Y40.734 E0.21411
G1 X27.531 Y40.734 E0.01363
G1 X27.547 Y40.789 E0.00148
G1 X21.210 Y47.125 E0.23395
G1 X21.210 Y47.717 E0.01546
G1 X27.717 Y41.211 E0.24022
G1 X27.892 Y41.627 E0.01180
```

G1 X21.210 Y48.309 E0.24671

G1 X21.210 Y48.901 E0.01546

G1 X28.104 Y42.008 E0.25451

G1 X28.320 Y42.384 E0.01132

G1 X21.210 Y49.493 E0.26250

G1 X21.210 Y50.085 E0.01546

G1 X28.567 Y42.729 E0.27162

G1 X28.821 Y43.066 E0.01104

G1 X21.210 Y50.677 E0.28101

G1 X21.210 Y51.269 E0.01546

G1 X29.100 Y43.379 E0.29132

G1 X29.392 Y43.680 E0.01093

G1 X21.210 Y51.861 E0.30207

G1 X21.210 Y52.453 E0.01546

G1 X29.702 Y43.962 E0.31352

G1 X30.030 Y44.226 E0.01099

G1 X21.210 Y53.045 E0.32563

G1 X21.210 Y53.638 E0.01546

G1 X30.372 Y44.476 E0.33826

G1 X30.737 Y44.703 E0.01123

G1 X21.618 Y53.822 E0.33670

G1 X22.210 Y53.822 E0.01546

G1 X31.114 Y44.918 E0.32875

G1 X31.520 Y45.105 E0.01165

G1 X22.802 Y53.822 E0.32186

G1 X23.395 Y53.822 E0.01546

G1 X31.937 Y45.280 E0.31540

G1 X32.386 Y45.423 E0.01230

G1 X23.987 Y53.822 E0.31012

G1 X24.579 Y53.822 E0.01546

G1 X32.852 Y45.548 E0.30549

G1 X33.351 Y45.641 E0.01325

G1 X25.171 Y53.822 E0.30204

G1 X25.763 Y53.822 E0.01546

G1 X33.884 Y45.700 E0.29986

G1 X34.443 Y45.734 E0.01460

G1 X26.355 Y53.822 E0.29862

G1 X26.947 Y53.822 E0.01546

G1 X27.945 Y52.824 E0.03684

G1 X28.363 Y52.097 E0.02190

G1 X28.905 Y51.378 E0.02352

G1 X29.532 Y50.731 E0.02351

G1 X30.233 Y50.166 E0.02351

G1 X30.999 Y49.691 E0.02352

G1 X31.145 Y49.624 E0.00420

G1 X35.035 Y45.734 E0.14362

G1 X35.627 Y45.734 E0.01546

G1 X32.154 Y49.207 E0.12823

G1 X32.675 Y49.041 E0.01428

G1 X32.966 Y48.987 E0.00774

G1 X36.219 Y45.734 E0.12009

G1 X36.811 Y45.734 E0.01546

G1 X33.677 Y48.868 E0.11572

G1 X34.308 Y48.829 E0.01651

G1 X37.403 Y45.734 E0.11428

G1 X37.995 Y45.734 E0.01546

G1 X34.907 Y48.822 E0.11400

G1 X35.499 Y48.822 E0.01546

G1 X38.587 Y45.734 E0.11400

M73 P54 R31

G1 X39.179 Y45.734 E0.01546

G1 X36.091 Y48.822 E0.11400

G1 X36.683 Y48.822 E0.01546

G1 X39.771 Y45.734 E0.11400

G1 X40.363 Y45.734 E0.01546

G1 X37.275 Y48.822 E0.11400

G1 X37.867 Y48.822 E0.01546

G1 X40.955 Y45.734 E0.11400

G1 X41.298 Y45.734 E0.00895

G1 X41.298 Y45.983 E0.00650

G1 X38.460 Y48.822 E0.10481

G1 X39.052 Y48.822 E0.01546

G1 X41.298 Y46.575 E0.08295

G1 X41.298 Y47.167 E0.01546

G1 X39.644 Y48.822 E0.06109

G1 X40.236 Y48.822 E0.01546

G1 X41.298 Y47.759 E0.03923

G1 X41.298 Y48.351 E0.01546

G1 X40.640 Y49.010 E0.02431

M204 S1250

; stop printing object tpu print.STL id:19 copy 0

; printing object Petg print.STL id:18 copy 0

; stop printing object Petg print.STL id:18 copy 0

; printing object Petg print.STL id:14 copy 0

; stop printing object Petg print.STL id:14 copy 0

; printing object tpu print.STL id:15 copy 0

G1 E-2.24000 F2400.000

```
;WIPE_START
G1 F7200.000
G1 X41.298 Y48.351 E-0.29484
G1 X41.298 Y47.759 E-0.18749
G1 X40.339 Y48.719 E-0.42968
;WIPE_END
G1 E-0.04800 F2400.000
G1 Z0.850 F9000.000
G1 X41.619 Y65.370
G1 Z0.650
G1 E3.20000 F1500.000
M204 S800
;TYPE:Perimeter
;WIDTH:0.45
G1 F2400.000
G1 X41.619 Y69.084 E0.09678
G1 X34.447 Y69.084 E0.18688
G1 X33.606 Y69.136 E0.02194
G1 X32.759 Y69.294 E0.02246
G1 X31.938 Y69.556 E0.02246
G1 X31.155 Y69.917 E0.02246
G1 X30.422 Y70.371 E0.02246
G1 X29.751 Y70.912 E0.02246
G1 X29.151 Y71.531 E0.02246
G1 X28.633 Y72.219 E0.02246
G1 X28.202 Y72.966 E0.02246
G1 X27.867 Y73.760 E0.02246
G1 X27.776 Y74.084 E0.00876
G1 X20.905 Y74.084 E0.17903
```

```
G1 X20.905 Y60.370 E0.35734
G1 X27.776 Y60.370 E0.17903
G1 X27.867 Y60.693 E0.00876
G1 X28.202 Y61.487 E0.02246
G1 X28.633 Y62.234 E0.02246
G1 X29.151 Y62.923 E0.02246
G1 X29.751 Y63.542 E0.02246
G1 X30.422 Y64.083 E0.02246
G1 X31.155 Y64.537 E0.02246
G1 X31.938 Y64.898 E0.02246
G1 X32.759 Y65.159 E0.02246
G1 X33.606 Y65.318 E0.02246
G1 X34.447 Y65.370 E0.02194
G1 X41.559 Y65.370 E0.18531
M204 S1250
G1 X42.037 Y64.952 F9000.000
M204 S800
;TYPE:External perimeter
G1 F1800.000
G1 X42.037 Y69.502 E0.11855
G1 X34.460 Y69.502 E0.19743
G1 X33.658 Y69.551 E0.02093
G1 X32.861 Y69.700 E0.02112
G1 X32.089 Y69.946 E0.02112
G1 X31.353 Y70.285 E0.02112
G1 X30.664 Y70.712 E0.02112
G1 X30.033 Y71.221 E0.02112
G1 X29.469 Y71.803 E0.02112
G1 X28.982 Y72.450 E0.02112
```

```
G1 X28.577 Y73.152 E0.02112
G1 X28.262 Y73.899 E0.02112
G1 X28.092 Y74.502 E0.01632
G1 X20.487 Y74.502 E0.19815
G1 X20.487 Y59.952 E0.37911
G1 X28.092 Y59.952 E0.19815
G1 X28.262 Y60.554 E0.01632
G1 X28.577 Y61.301 E0.02112
G1 X28.982 Y62.003 E0.02112
G1 X29.469 Y62.651 E0.02112
G1 X30.033 Y63.233 E0.02112
G1 X30.664 Y63.741 E0.02112
G1 X31.353 Y64.168 E0.02112
G1 X32.089 Y64.507 E0.02112
G1 X32.861 Y64.753 E0.02112
G1 X33.658 Y64.902 E0.02112
G1 X34.460 Y64.952 E0.02093
G1 X41.977 Y64.952 E0.19586
M204 S1250
G1 X41.836 Y65.298 F9000.000
G1 E-2.24000 F2400.000
;WIPE_START
G1 F7200.000
G1 X42.015 Y67.832 E-0.91200
;WIPE_END
G1 E-0.04800 F2400.000
G1 Z0.850 F9000.000
G1 X21.876 Y60.495
G1 Z0.650
```

```
G1 E3.20000 F1500.000
M204 S1000
;TYPE:Solid infill
;WIDTH:0.450839
G1 F4800.000
G1 X21.218 Y61.153 E0.02431
G1 X21.218 Y61.745 E0.01546
G1 X22.280 Y60.683 E0.03922
G1 X22.872 Y60.683 E0.01546
G1 X21.218 Y62.337 E0.06108
G1 X21.218 Y62.929 E0.01546
G1 X23.464 Y60.683 E0.08294
G1 X24.056 Y60.683 E0.01546
G1 X21.218 Y63.521 E0.10480
G1 X21.218 Y64.114 E0.01546
G1 X24.649 Y60.683 E0.12666
G1 X25.241 Y60.683 E0.01546
G1 X21.218 Y64.706 E0.14853
G1 X21.218 Y65.298 E0.01546
G1 X25.833 Y60.683 E0.17039
G1 X26.425 Y60.683 E0.01546
G1 X21.218 Y65.890 E0.19225
G1 X21.218 Y66.482 E0.01546
G1 X27.017 Y60.683 E0.21411
G1 X27.539 Y60.683 E0.01363
G1 X27.554 Y60.738 E0.00148
G1 X21.218 Y67.074 E0.23395
G1 X21.218 Y67.666 E0.01546
G1 X27.724 Y61.160 E0.24022
```

```
G1 X27.900 Y61.576 E0.01180
G1 X21.218 Y68.258 E0.24671
G1 X21.218 Y68.850 E0.01546
G1 X28.111 Y61.957 E0.25451
G1 X28.328 Y62.332 E0.01132
G1 X21.218 Y69.442 E0.26250
G1 X21.218 Y70.034 E0.01546
G1 X28.574 Y62.678 E0.27162
G1 X28.829 Y63.015 E0.01104
G1 X21.218 Y70.626 E0.28101
G1 X21.218 Y71.218 E0.01546
G1 X29.108 Y63.328 E0.29132
G1 X29.399 Y63.629 E0.01093
G1 X21.218 Y71.810 E0.30207
G1 X21.218 Y72.402 E0.01546
G1 X29.709 Y63.911 E0.31352
G1 X30.037 Y64.175 E0.01099
G1 X21.218 Y72.994 E0.32563
G1 X21.218 Y73.586 E0.01546
G1 X30.379 Y64.425 E0.33826
G1 X30.745 Y64.652 E0.01123
G1 X21.626 Y73.771 E0.33670
G1 X22.218 Y73.771 E0.01546
G1 X31.122 Y64.867 E0.32875
G1 X31.527 Y65.053 E0.01165
G1 X22.810 Y73.771 E0.32186
G1 X23.402 Y73.771 E0.01546
G1 X31.944 Y65.229 E0.31540
G1 X32.393 Y65.372 E0.01230
```

```
G1 X23.994 Y73.771 E0.31012
G1 X24.586 Y73.771 E0.01546
G1 X32.860 Y65.497 E0.30549
G1 X33.359 Y65.590 E0.01325
G1 X25.178 Y73.771 E0.30204
G1 X25.770 Y73.771 E0.01546
G1 X33.892 Y65.649 E0.29986
G1 X34.450 Y65.683 E0.01460
G1 X26.362 Y73.771 E0.29862
G1 X26.954 Y73.771 E0.01546
G1 X27.952 Y72.773 E0.03684
G1 X28.371 Y72.046 E0.02190
G1 X28.913 Y71.327 E0.02352
G1 X29.539 Y70.680 E0.02351
G1 X30.241 Y70.115 E0.02351
G1 X31.006 Y69.640 E0.02352
G1 X31.152 Y69.573 E0.00420
G1 X35.042 Y65.683 E0.14362
G1 X35.634 Y65.683 E0.01546
G1 X32.161 Y69.156 E0.12823
G1 X32.682 Y68.990 E0.01428
G1 X32.974 Y68.935 E0.00774
G1 X36.226 Y65.683 E0.12009
G1 X36.818 Y65.683 E0.01546
G1 X33.684 Y68.817 E0.11572
G1 X34.315 Y68.778 E0.01651
G1 X37.410 Y65.683 E0.11428
G1 X38.002 Y65.683 E0.01546
G1 X34.915 Y68.771 E0.11400
```

G1 X35.507 Y68.771 E0.01546

G1 X38.594 Y65.683 E0.11400

G1 X39.186 Y65.683 E0.01546

G1 X36.099 Y68.771 E0.11400

G1 X36.691 Y68.771 E0.01546

G1 X39.779 Y65.683 E0.11400

G1 X40.371 Y65.683 E0.01546

G1 X37.283 Y68.771 E0.11400

G1 X37.875 Y68.771 E0.01546

G1 X40.963 Y65.683 E0.11400

G1 X41.306 Y65.683 E0.00895

G1 X41.306 Y65.932 E0.00650

G1 X38.467 Y68.771 E0.10481

G1 X39.059 Y68.771 E0.01546

G1 X41.306 Y66.524 E0.08295

G1 X41.306 Y67.116 E0.01546

G1 X39.651 Y68.771 E0.06109

G1 X40.243 Y68.771 E0.01546

G1 X41.306 Y67.708 E0.03923

G1 X41.306 Y68.300 E0.01546

G1 X40.647 Y68.959 E0.02431

M204 S1250

; stop printing object tpu print.STL id:15 copy 0

; printing object Petg print.STL id:10 copy 0

; stop printing object Petg print.STL id:10 copy 0

; printing object tpu print.STL id:11 copy 0

G1 E-2.24000 F2400.000

;WIPE_START

G1 F7200.000

```
G1 X41.306 Y68.300 E-0.29484
G1 X41.306 Y67.708 E-0.18749
G1 X40.346 Y68.668 E-0.42968
;WIPE_END
G1 E-0.04800 F2400.000
G1 Z0.850 F9000.000
G1 X41.568 Y87.562
G1 Z0.650
G1 E3.20000 F1500.000
M204 S800
;TYPE:Perimeter
;WIDTH:0.45
G1 F2400.000
G1 X41.568 Y91.276 E0.09678
G1 X34.396 Y91.276 E0.18688
G1 X33.555 Y91.328 E0.02194
G1 X32.708 Y91.487 E0.02246
G1 X31.887 Y91.748 E0.02246
G1 X31.104 Y92.109 E0.02246
G1 X30.371 Y92.563 E0.02246
G1 X29.700 Y93.104 E0.02246
G1 X29.100 Y93.723 E0.02246
G1 X28.581 Y94.412 E0.02246
G1 X28.151 Y95.159 E0.02246
G1 X27.816 Y95.953 E0.02246
G1 X27.725 Y96.276 E0.00876
G1 X20.853 Y96.276 E0.17903
G1 X20.853 Y82.562 E0.35734
G1 X27.725 Y82.562 E0.17903
```

```
G1 X27.816 Y82.885 E0.00876
G1 X28.151 Y83.680 E0.02246
G1 X28.581 Y84.427 E0.02246
G1 X29.100 Y85.115 E0.02246
G1 X29.700 Y85.734 E0.02246
G1 X30.371 Y86.275 E0.02246
G1 X31.104 Y86.729 E0.02246
G1 X31.887 Y87.090 E0.02246
G1 X32.708 Y87.351 E0.02246
G1 X33.555 Y87.510 E0.02246
G1 X34.396 Y87.562 E0.02194
G1 X41.508 Y87.562 E0.18531
M204 S1250
G1 X41.986 Y87.144 F9000.000
M204 S800
;TYPE:External perimeter
G1 F1800.000
G1 X41.986 Y91.694 E0.11855
G1 X34.409 Y91.694 E0.19743
G1 X33.607 Y91.744 E0.02093
G1 X32.810 Y91.893 E0.02112
G1 X32.038 Y92.139 E0.02112
G1 X31.302 Y92.478 E0.02112
G1 X30.613 Y92.905 E0.02112
G1 X29.982 Y93.413 E0.02112
G1 X29.418 Y93.995 E0.02112
G1 X28.931 Y94.643 E0.02112
G1 X28.526 Y95.345 E0.02112
G1 X28.211 Y96.091 E0.02112
```

```
G1 X28.040 Y96.694 E0.01632
G1 X20.436 Y96.694 E0.19815
G1 X20.436 Y82.144 E0.37911
G1 X28.040 Y82.144 E0.19815
G1 X28.211 Y82.747 E0.01632
G1 X28.526 Y83.493 E0.02112
G1 X28.931 Y84.196 E0.02112
G1 X29.418 Y84.843 E0.02112
G1 X29.982 Y85.425 E0.02112
G1 X30.613 Y85.934 E0.02112
G1 X31.302 Y86.361 E0.02112
G1 X32.038 Y86.700 E0.02112
G1 X32.810 Y86.946 E0.02112
G1 X33.607 Y87.095 E0.02112
G1 X34.409 Y87.144 E0.02093
G1 X41.926 Y87.144 E0.19586
M204 S1250
G1 X41.785 Y87.490 F9000.000
G1 E-2.24000 F2400.000
;WIPE_START
G1 F7200.000
G1 X41.964 Y90.024 E-0.91200
;WIPE_END
G1 E-0.04800 F2400.000
G1 Z0.850 F9000.000
G1 X21.825 Y82.687
G1 Z0.650
G1 E3.20000 F1500.000
M204 S1000
```

```
;TYPE:Solid infill
;WIDTH:0.450839
G1 F4800.000
G1 X21.167 Y83.346 E0.02431
G1 X21.167 Y83.938 E0.01546
G1 X22.229 Y82.875 E0.03922
G1 X22.821 Y82.875 E0.01546
G1 X21.167 Y84.530 E0.06108
G1 X21.167 Y85.122 E0.01546
G1 X23.413 Y82.875 E0.08294
G1 X24.005 Y82.875 E0.01546
G1 X21.167 Y85.714 E0.10480
G1 X21.167 Y86.306 E0.01546
G1 X24.597 Y82.875 E0.12666
G1 X25.189 Y82.875 E0.01546
G1 X21.167 Y86.898 E0.14853
G1 X21.167 Y87.490 E0.01546
G1 X25.781 Y82.875 E0.17039
G1 X26.374 Y82.875 E0.01546
G1 X21.167 Y88.082 E0.19225
G1 X21.167 Y88.674 E0.01546
G1 X26.966 Y82.875 E0.21411
G1 X27.488 Y82.875 E0.01363
G1 X27.503 Y82.930 E0.00148
G1 X21.167 Y89.266 E0.23395
G1 X21.167 Y89.858 E0.01546
G1 X27.673 Y83.352 E0.24022
G1 X27.849 Y83.768 E0.01180
G1 X21.167 Y90.450 E0.24671
```

```
G1 X21.167 Y91.042 E0.01546
G1 X28.060 Y84.149 E0.25451
G1 X28.276 Y84.525 E0.01132
G1 X21.167 Y91.634 E0.26250
G1 X21.167 Y92.226 E0.01546
G1 X28.523 Y84.870 E0.27162
G1 X28.778 Y85.208 E0.01104
G1 X21.167 Y92.819 E0.28101
G1 X21.167 Y93.411 E0.01546
G1 X29.057 Y85.521 E0.29132
G1 X29.348 Y85.821 E0.01093
G1 X21.167 Y94.003 E0.30207
G1 X21.167 Y94.595 E0.01546
G1 X29.658 Y86.103 E0.31352
G1 X29.986 Y86.367 E0.01099
G1 X21.167 Y95.187 E0.32563
G1 X21.167 Y95.779 E0.01546
G1 X30.328 Y86.617 E0.33826
G1 X30.694 Y86.844 E0.01123
G1 X21.575 Y95.963 E0.33670
G1 X22.167 Y95.963 E0.01546
G1 X31.071 Y87.059 E0.32875
G1 X31.476 Y87.246 E0.01165
G1 X22.759 Y95.963 E0.32186
G1 X23.351 Y95.963 E0.01546
G1 X31.893 Y87.421 E0.31540
G1 X32.342 Y87.564 E0.01230
G1 X23.943 Y95.963 E0.31012
G1 X24.535 Y95.963 E0.01546
```

G1 X32.809 Y87.689 E0.30549

G1 X33.308 Y87.783 E0.01325

G1 X25.127 Y95.963 E0.30204

G1 X25.719 Y95.963 E0.01546

G1 X33.840 Y87.842 E0.29986

G1 X34.399 Y87.875 E0.01460

G1 X26.311 Y95.963 E0.29862

G1 X26.903 Y95.963 E0.01546

G1 X27.901 Y94.965 E0.03684

G1 X28.320 Y94.239 E0.02190

G1 X28.862 Y93.519 E0.02352

G1 X29.488 Y92.872 E0.02351

G1 X30.189 Y92.307 E0.02351

G1 X30.955 Y91.832 E0.02352

G1 X31.101 Y91.765 E0.00420

G1 X34.991 Y87.875 E0.14362

G1 X35.583 Y87.875 E0.01546

G1 X32.110 Y91.348 E0.12823

G1 X32.631 Y91.182 E0.01428

G1 X32.923 Y91.128 E0.00774

G1 X36.175 Y87.875 E0.12009

G1 X36.767 Y87.875 E0.01546

G1 X33.633 Y91.009 E0.11572

G1 X34.264 Y90.970 E0.01651

G1 X37.359 Y87.875 E0.11428

G1 X37.951 Y87.875 E0.01546

G1 X34.864 Y90.963 E0.11400

G1 X35.456 Y90.963 E0.01546

G1 X38.543 Y87.875 E0.11400

G1 X39.135 Y87.875 E0.01546

G1 X36.048 Y90.963 E0.11400

G1 X36.640 Y90.963 E0.01546

G1 X39.727 Y87.875 E0.11400

G1 X40.319 Y87.875 E0.01546

G1 X37.232 Y90.963 E0.11400

G1 X37.824 Y90.963 E0.01546

G1 X40.911 Y87.875 E0.11400

G1 X41.254 Y87.875 E0.00895

G1 X41.254 Y88.124 E0.00650

G1 X38.416 Y90.963 E0.10481

G1 X39.008 Y90.963 E0.01546

G1 X41.254 Y88.716 E0.08295

G1 X41.254 Y89.308 E0.01546

G1 X39.600 Y90.963 E0.06109

G1 X40.192 Y90.963 E0.01546

G1 X41.254 Y89.901 E0.03923

G1 X41.254 Y90.493 E0.01546

G1 X40.596 Y91.151 E0.02431

M204 S1250

; stop printing object tpu print.STL id:11 copy 0

; printing object tpu print.STL id:13 copy 0

G1 E-2.24000 F2400.000

;WIPE_START

G1 F7200.000

G1 X41.254 Y90.493 E-0.29484

G1 X41.254 Y89.901 E-0.18749

G1 X40.295 Y90.860 E-0.42968

;WIPE_END

```
G1 E-0.04800 F2400.000
G1 Z0.850 F9000.000
G1 X41.575 Y108.377
G1 Z0.650
G1 E3.20000 F1500.000
M204 S800
;TYPE:Perimeter
;WIDTH:0.45
G1 F2400.000
G1 X41.575 Y112.092 E0.09678
G1 X34.402 Y112.092 E0.18688
G1 X33.562 Y112.144 E0.02194
G1 X32.715 Y112.302 E0.02246
G1 X31.893 Y112.564 E0.02246
G1 X31.110 Y112.924 E0.02246
G1 X30.378 Y113.379 E0.02246
G1 X29.707 Y113.920 E0.02246
G1 X29.107 Y114.539 E0.02246
G1 X28.588 Y115.227 E0.02246
G1 X28.158 Y115.974 E0.02246
G1 X27.823 Y116.768 E0.02246
G1 X27.731 Y117.092 E0.00876
G1 X20.860 Y117.092 E0.17903
G1 X20.860 Y103.377 E0.35734
G1 X27.731 Y103.377 E0.17903
G1 X27.823 Y103.701 E0.00876
G1 X28.158 Y104.495 E0.02246
G1 X28.588 Y105.242 E0.02246
G1 X29.107 Y105.930 E0.02246
```

```
G1 X29.707 Y106.550 E0.02246
G1 X30.378 Y107.091 E0.02246
G1 X31.110 Y107.545 E0.02246
G1 X31.893 Y107.905 E0.02246
G1 X32.715 Y108.167 E0.02246
G1 X33.562 Y108.326 E0.02246
G1 X34.402 Y108.377 E0.02194
G1 X41.515 Y108.377 E0.18531
M204 S1250
G1 X41.992 Y107.960 F9000.000
M204 S800
;TYPE:External perimeter
G1 F1800.000
G1 X41.992 Y112.510 E0.11855
G1 X34.415 Y112.510 E0.19743
G1 X33.613 Y112.559 E0.02093
G1 X32.817 Y112.708 E0.02112
G1 X32.045 Y112.954 E0.02112
G1 X31.309 Y113.293 E0.02112
G1 X30.620 Y113.720 E0.02112
G1 X29.989 Y114.229 E0.02112
G1 X29.425 Y114.811 E0.02112
G1 X28.937 Y115.458 E0.02112
G1 X28.533 Y116.160 E0.02112
G1 X28.218 Y116.907 E0.02112
G1 X28.047 Y117.510 E0.01632
G1 X20.442 Y117.510 E0.19815
G1 X20.442 Y102.960 E0.37911
G1 X28.047 Y102.960 E0.19815
```

```
G1 X28.218 Y103.562 E0.01632
G1 X28.533 Y104.309 E0.02112
G1 X28.937 Y105.011 E0.02112
G1 X29.425 Y105.658 E0.02112
G1 X29.989 Y106.241 E0.02112
G1 X30.620 Y106.749 E0.02112
G1 X31.309 Y107.176 E0.02112
G1 X32.045 Y107.515 E0.02112
G1 X32.817 Y107.761 E0.02112
G1 X33.613 Y107.910 E0.02112
G1 X34.415 Y107.960 E0.02093
G1 X41.932 Y107.960 E0.19586
M204 S1250
G1 X41.792 Y108.306 F9000.000
G1 E-2.24000 F2400.000
;WIPE_START
G1 F7200.000
G1 X41.970 Y110.839 E-0.91200
;WIPE_END
G1 E-0.04800 F2400.000
G1 Z0.850 F9000.000
G1 X21.832 Y103.503
G1 Z0.650
G1 E3.20000 F1500.000
M204 S1000
;TYPE:Solid infill
;WIDTH:0.450839
G1 F4800.000
G1 X21.174 Y104.161 E0.02431
```

```
G1 X21.174 Y104.753 E0.01546
G1 X22.236 Y103.691 E0.03922
G1 X22.828 Y103.691 E0.01546
G1 X21.174 Y105.345 E0.06108
G1 X21.174 Y105.937 E0.01546
G1 X23.420 Y103.691 E0.08294
G1 X24.012 Y103.691 E0.01546
G1 X21.174 Y106.529 E0.10480
G1 X21.174 Y107.121 E0.01546
G1 X24.604 Y103.691 E0.12666
G1 X25.196 Y103.691 E0.01546
G1 X21.174 Y107.713 E0.14853
G1 X21.174 Y108.306 E0.01546
G1 X25.788 Y103.691 E0.17039
G1 X26.380 Y103.691 E0.01546
G1 X21.174 Y108.898 E0.19225
G1 X21.174 Y109.490 E0.01546
G1 X26.972 Y103.691 E0.21411
G1 X27.494 Y103.691 E0.01363
G1 X27.510 Y103.745 E0.00148
G1 X21.174 Y110.082 E0.23395
G1 X21.174 Y110.674 E0.01546
G1 X27.680 Y104.168 E0.24022
G1 X27.855 Y104.584 E0.01180
G1 X21.174 Y111.266 E0.24671
G1 X21.174 Y111.858 E0.01546
G1 X28.067 Y104.965 E0.25451
G1 X28.283 Y105.340 E0.01132
G1 X21.174 Y112.450 E0.26250
```

G1 X21.174 Y113.042 E0.01546

G1 X28.530 Y105.686 E0.27162

G1 X28.785 Y106.023 E0.01104

G1 X21.174 Y113.634 E0.28101

G1 X21.174 Y114.226 E0.01546

G1 X29.064 Y106.336 E0.29132

G1 X29.355 Y106.637 E0.01093

G1 X21.174 Y114.818 E0.30207

G1 X21.174 Y115.410 E0.01546

G1 X29.665 Y106.919 E0.31352

G1 X29.993 Y107.183 E0.01099

G1 X21.174 Y116.002 E0.32563

G1 X21.174 Y116.594 E0.01546

G1 X30.335 Y107.433 E0.33826

G1 X30.701 Y107.659 E0.01123

G1 X21.582 Y116.778 E0.33670

G1 X22.174 Y116.778 E0.01546

G1 X31.077 Y107.875 E0.32875

G1 X31.483 Y108.061 E0.01165

G1 X22.766 Y116.778 E0.32186

G1 X23.358 Y116.778 E0.01546

G1 X31.900 Y108.236 E0.31540

G1 X32.349 Y108.379 E0.01230

G1 X23.950 Y116.778 E0.31012

G1 X24.542 Y116.778 E0.01546

G1 X32.816 Y108.505 E0.30549

G1 X33.314 Y108.598 E0.01325

G1 X25.134 Y116.778 E0.30204

G1 X25.726 Y116.778 E0.01546

G1 X33.847 Y108.657 E0.29986

G1 X34.406 Y108.691 E0.01460

G1 X26.318 Y116.778 E0.29862

M73 P55 R31

G1 X26.910 Y116.778 E0.01546

G1 X27.908 Y115.781 E0.03684

G1 X28.326 Y115.054 E0.02190

G1 X28.868 Y114.335 E0.02352

G1 X29.495 Y113.688 E0.02351

G1 X30.196 Y113.123 E0.02351

G1 X30.962 Y112.648 E0.02352

G1 X31.108 Y112.581 E0.00420

G1 X34.998 Y108.691 E0.14362

G1 X35.590 Y108.691 E0.01546

G1 X32.117 Y112.164 E0.12823

G1 X32.638 Y111.998 E0.01428

G1 X32.929 Y111.943 E0.00774

G1 X36.182 Y108.691 E0.12009

G1 X36.774 Y108.691 E0.01546

G1 X33.640 Y111.825 E0.11572

G1 X34.271 Y111.786 E0.01651

G1 X37.366 Y108.691 E0.11428

G1 X37.958 Y108.691 E0.01546

G1 X34.870 Y111.778 E0.11400

G1 X35.462 Y111.778 E0.01546

G1 X38.550 Y108.691 E0.11400

G1 X39.142 Y108.691 E0.01546

G1 X36.054 Y111.778 E0.11400

G1 X36.647 Y111.778 E0.01546

```
G1 X39.734 Y108.691 E0.11400
G1 X40.326 Y108.691 E0.01546
G1 X37.239 Y111.778 E0.11400
G1 X37.831 Y111.778 E0.01546
G1 X40.918 Y108.691 E0.11400
G1 X41.261 Y108.691 E0.00895
G1 X41.261 Y108.940 E0.00650
G1 X38.423 Y111.778 E0.10481
G1 X39.015 Y111.778 E0.01546
G1 X41.261 Y109.532 E0.08295
G1 X41.261 Y110.124 E0.01546
G1 X39.607 Y111.778 E0.06109
G1 X40.199 Y111.778 E0.01546
G1 X41.261 Y110.716 E0.03923
G1 X41.261 Y111.308 E0.01546
G1 X40.603 Y111.967 E0.02431
M204 S1250
; stop printing object tpu print.STL id:13 copy 0
; printing object tpu print.STL id:17 copy 0
G1 E-2.24000 F2400.000
;WIPE_START
G1 F7200.000
G1 X41.261 Y111.308 E-0.29484
G1 X41.261 Y110.716 E-0.18749
G1 X40.302 Y111.676 E-0.42968
;WIPE_END
G1 E-0.04800 F2400.000
G1 Z0.850 F9000.000
G1 X41.560 Y127.031
```

```
G1 Z0.650
G1 E3.20000 F1500.000
M204 S800
;TYPE:Perimeter
;WIDTH:0.45
G1 F2400.000
G1 X41.560 Y130.746 E0.09678
G1 X34.387 Y130.746 E0.18688
G1 X33.547 Y130.798 E0.02194
G1 X32.700 Y130.956 E0.02246
G1 X31.878 Y131.218 E0.02246
G1 X31.095 Y131.578 E0.02246
G1 X30.363 Y132.033 E0.02246
G1 X29.692 Y132.573 E0.02246
G1 X29.092 Y133.193 E0.02246
G1 X28.573 Y133.881 E0.02246
G1 X28.143 Y134.628 E0.02246
G1 X27.808 Y135.422 E0.02246
G1 X27.716 Y135.746 E0.00876
G1 X20.845 Y135.746 E0.17903
G1 X20.845 Y122.031 E0.35734
G1 X27.716 Y122.031 E0.17903
G1 X27.808 Y122.355 E0.00876
G1 X28.143 Y123.149 E0.02246
G1 X28.573 Y123.896 E0.02246
G1 X29.092 Y124.584 E0.02246
G1 X29.692 Y125.204 E0.02246
G1 X30.363 Y125.745 E0.02246
G1 X31.095 Y126.199 E0.02246
```

```
G1 X31.878 Y126.559 E0.02246
G1 X32.700 Y126.821 E0.02246
G1 X33.547 Y126.980 E0.02246
G1 X34.387 Y127.031 E0.02194
G1 X41.500 Y127.031 E0.18531
M204 S1250
G1 X41.977 Y126.614 F9000.000
M204 S800
;TYPE:External perimeter
G1 F1800.000
G1 X41.977 Y131.164 E0.11855
G1 X34.400 Y131.164 E0.19743
G1 X33.598 Y131.213 E0.02093
G1 X32.802 Y131.362 E0.02112
G1 X32.030 Y131.608 E0.02112
G1 X31.294 Y131.947 E0.02112
G1 X30.605 Y132.374 E0.02112
G1 X29.974 Y132.883 E0.02112
G1 X29.410 Y133.465 E0.02112
G1 X28.922 Y134.112 E0.02112
G1 X28.518 Y134.814 E0.02112
G1 X28.203 Y135.561 E0.02112
G1 X28.032 Y136.164 E0.01632
G1 X20.427 Y136.164 E0.19815
G1 X20.427 Y121.614 E0.37911
G1 X28.032 Y121.614 E0.19815
G1 X28.203 Y122.216 E0.01632
G1 X28.518 Y122.963 E0.02112
G1 X28.922 Y123.665 E0.02112
```

```
G1 X29.410 Y124.312 E0.02112
G1 X29.974 Y124.895 E0.02112
G1 X30.605 Y125.403 E0.02112
G1 X31.294 Y125.830 E0.02112
G1 X32.030 Y126.169 E0.02112
G1 X32.802 Y126.415 E0.02112
G1 X33.598 Y126.564 E0.02112
G1 X34.400 Y126.614 E0.02093
G1 X41.917 Y126.614 E0.19586
M204 S1250
G1 X41.777 Y126.960 F9000.000
G1 E-2.24000 F2400.000
;WIPE_START
G1 F7200.000
G1 X41.955 Y129.493 E-0.91200
;WIPE_END
G1 E-0.04800 F2400.000
G1 Z0.850 F9000.000
G1 X21.817 Y122.157
G1 Z0.650
G1 E3.20000 F1500.000
M204 S1000
;TYPE:Solid infill
;WIDTH:0.450839
G1 F4800.000
G1 X21.159 Y122.815 E0.02431
G1 X21.159 Y123.407 E0.01546
G1 X22.221 Y122.345 E0.03922
G1 X22.813 Y122.345 E0.01546
```

```
G1 X21.159 Y123.999 E0.06108
G1 X21.159 Y124.591 E0.01546
G1 X23.405 Y122.345 E0.08294
G1 X23.997 Y122.345 E0.01546
G1 X21.159 Y125.183 E0.10480
G1 X21.159 Y125.775 E0.01546
G1 X24.589 Y122.345 E0.12666
G1 X25.181 Y122.345 E0.01546
G1 X21.159 Y126.367 E0.14853
G1 X21.159 Y126.959 E0.01546
G1 X25.773 Y122.345 E0.17039
G1 X26.365 Y122.345 E0.01546
G1 X21.159 Y127.552 E0.19225
G1 X21.159 Y128.144 E0.01546
G1 X26.957 Y122.345 E0.21411
G1 X27.479 Y122.345 E0.01363
G1 X27.495 Y122.399 E0.00148
G1 X21.159 Y128.736 E0.23395
G1 X21.159 Y129.328 E0.01546
G1 X27.665 Y122.822 E0.24022
G1 X27.840 Y123.238 E0.01180
G1 X21.159 Y129.920 E0.24671
G1 X21.159 Y130.512 E0.01546
G1 X28.052 Y123.619 E0.25451
G1 X28.268 Y123.994 E0.01132
G1 X21.159 Y131.104 E0.26250
G1 X21.159 Y131.696 E0.01546
G1 X28.515 Y124.340 E0.27162
G1 X28.769 Y124.677 E0.01104
```

```
G1 X21.159 Y132.288 E0.28101
G1 X21.159 Y132.880 E0.01546
G1 X29.049 Y124.990 E0.29132
G1 X29.340 Y125.291 E0.01093
G1 X21.159 Y133.472 E0.30207
G1 X21.159 Y134.064 E0.01546
G1 X29.650 Y125.573 E0.31352
G1 X29.978 Y125.837 E0.01099
G1 X21.159 Y134.656 E0.32563
G1 X21.159 Y135.248 E0.01546
G1 X30.320 Y126.087 E0.33826
G1 X30.685 Y126.313 E0.01123
G1 X21.566 Y135.432 E0.33670
G1 X22.158 Y135.432 E0.01546
G1 X31.062 Y126.529 E0.32875
G1 X31.468 Y126.715 E0.01165
G1 X22.751 Y135.432 E0.32186
M73 P55 R30
G1 X23.343 Y135.432 E0.01546
G1 X31.885 Y126.890 E0.31540
G1 X32.334 Y127.033 E0.01230
G1 X23.935 Y135.432 E0.31012
G1 X24.527 Y135.432 E0.01546
G1 X32.801 Y127.159 E0.30549
G1 X33.299 Y127.252 E0.01325
G1 X25.119 Y135.432 E0.30204
G1 X25.711 Y135.432 E0.01546
G1 X33.832 Y127.311 E0.29986
G1 X34.391 Y127.345 E0.01460
```

```
G1 X26.303 Y135.432 E0.29862
G1 X26.895 Y135.432 E0.01546
G1 X27.893 Y134.435 E0.03684
G1 X28.311 Y133.708 E0.02190
G1 X28.853 Y132.989 E0.02352
G1 X29.480 Y132.342 E0.02351
G1 X30.181 Y131.776 E0.02351
G1 X30.947 Y131.302 E0.02352
G1 X31.093 Y131.235 E0.00420
G1 X34.983 Y127.345 E0.14362
G1 X35.575 Y127.345 E0.01546
G1 X32.102 Y130.818 E0.12823
G1 X32.623 Y130.652 E0.01428
G1 X32.914 Y130.597 E0.00774
G1 X36.167 Y127.345 E0.12009
G1 X36.759 Y127.345 E0.01546
G1 X33.625 Y130.479 E0.11572
G1 X34.256 Y130.440 E0.01651
G1 X37.351 Y127.345 E0.11428
G1 X37.943 Y127.345 E0.01546
G1 X34.855 Y130.432 E0.11400
G1 X35.447 Y130.432 E0.01546
G1 X38.535 Y127.345 E0.11400
G1 X39.127 Y127.345 E0.01546
G1 X36.039 Y130.432 E0.11400
G1 X36.631 Y130.432 E0.01546
G1 X39.719 Y127.345 E0.11400
G1 X40.311 Y127.345 E0.01546
G1 X37.223 Y130.432 E0.11400
```

```
G1 X37.816 Y130.432 E0.01546
G1 X40.903 Y127.345 E0.11400
G1 X41.246 Y127.345 E0.00895
G1 X41.246 Y127.594 E0.00650
G1 X38.408 Y130.432 E0.10481
G1 X39.000 Y130.432 E0.01546
G1 X41.246 Y128.186 E0.08295
G1 X41.246 Y128.778 E0.01546
G1 X39.592 Y130.432 E0.06109
G1 X40.184 Y130.432 E0.01546
G1 X41.246 Y129.370 E0.03923
G1 X41.246 Y129.962 E0.01546
G1 X40.588 Y130.620 E0.02431
M204 S1250
; stop printing object tpu print.STL id:17 copy 0
; printing object Petg print.STL id:16 copy 0
; stop printing object Petg print.STL id:16 copy 0
; printing object Petg print.STL id:12 copy 0
; stop printing object Petg print.STL id:12 copy 0
; printing object tpu print.STL id:7 copy 0
G1 E-2.24000 F2400.000
;WIPE_START
G1 F7200.000
G1 X41.246 Y129.962 E-0.29484
G1 X41.246 Y129.370 E-0.18749
G1 X40.287 Y130.329 E-0.42968
;WIPE_END
G1 E-0.04800 F2400.000
G1 Z0.850 F9000.000
```

```
G1 X69.635 Y136.327

G1 Z0.650

G1 E3.20000 F1500.000

M204 S800

;TYPE:Perimeter

;WIDTH:0.45

G1 F2400.000

G1 X69.635 Y122.612 E0.35734

G1 X76.506 Y122.612 E0.17903

G1 X76.597 Y122.936 E0.00876

G1 X76.932 Y123.730 E0.02246

G1 X77.362 Y124.477 E0.02246

G1 X77.881 Y125.165 E0.02246

G1 X78.481 Y125.785 E0.02246

G1 X79.152 Y126.325 E0.02246

G1 X79.885 Y126.780 E0.02246

G1 X80.668 Y127.140 E0.02246

G1 X81.489 Y127.402 E0.02246

G1 X82.336 Y127.560 E0.02246

G1 X83.177 Y127.612 E0.02194

G1 X90.349 Y127.612 E0.18688

G1 X90.349 Y131.327 E0.09678

G1 X83.177 Y131.327 E0.18688

G1 X82.336 Y131.379 E0.02194

G1 X81.489 Y131.537 E0.02246

G1 X80.668 Y131.799 E0.02246

G1 X79.885 Y132.159 E0.02246

G1 X79.152 Y132.613 E0.02246

G1 X78.481 Y133.154 E0.02246
```

```
G1 X77.881 Y133.774 E0.02246
G1 X77.362 Y134.462 E0.02246
G1 X76.932 Y135.209 E0.02246
G1 X76.597 Y136.003 E0.02246
G1 X76.506 Y136.327 E0.00876
G1 X69.695 Y136.327 E0.17747
M204 S1250
G1 X69.217 Y136.744 F9000.000
M204 S800
;TYPE:External perimeter
G1 F1800.000
G1 X69.217 Y122.194 E0.37911
G1 X76.822 Y122.194 E0.19815
G1 X76.992 Y122.797 E0.01632
G1 X77.307 Y123.544 E0.02112
G1 X77.712 Y124.246 E0.02112
G1 X78.199 Y124.893 E0.02112
G1 X78.763 Y125.475 E0.02112
G1 X79.394 Y125.984 E0.02112
G1 X80.083 Y126.411 E0.02112
G1 X80.819 Y126.750 E0.02112
G1 X81.591 Y126.996 E0.02112
G1 X82.388 Y127.145 E0.02112
G1 X83.190 Y127.194 E0.02093
G1 X90.767 Y127.194 E0.19743
G1 X90.767 Y131.744 E0.11855
G1 X83.190 Y131.744 E0.19743
G1 X82.388 Y131.794 E0.02093
G1 X81.591 Y131.943 E0.02112
```

```
G1 X80.819 Y132.189 E0.02112
G1 X80.083 Y132.528 E0.02112
G1 X79.394 Y132.955 E0.02112
G1 X78.763 Y133.463 E0.02112
G1 X78.199 Y134.046 E0.02112
G1 X77.712 Y134.693 E0.02112
G1 X77.307 Y135.395 E0.02112
G1 X76.992 Y136.142 E0.02112
G1 X76.822 Y136.744 E0.01632
G1 X69.277 Y136.744 E0.19659
M204 S1250
G1 X69.408 Y136.393 F9000.000
G1 E-2.24000 F2400.000
;WIPE_START
G1 F7200.000
G1 X69.265 Y133.864 E-0.91200
;WIPE_END
G1 E-0.04800 F2400.000
G1 Z0.850 F9000.000
G1 X89.377 Y131.201
G1 Z0.650
G1 E3.20000 F1500.000
M204 S1000
;TYPE:Solid infill
;WIDTH:0.450839
G1 F4800.000
G1 X90.036 Y130.543 E0.02431
G1 X90.036 Y129.951 E0.01546
G1 X88.973 Y131.013 E0.03923
```

```
G1 X88.381 Y131.013 E0.01546
G1 X90.036 Y129.359 E0.06109
G1 X90.036 Y128.767 E0.01546
G1 X87.789 Y131.013 E0.08295
G1 X87.197 Y131.013 E0.01546
G1 X90.036 Y128.175 E0.10481
G1 X90.036 Y127.926 E0.00650
G1 X89.693 Y127.926 E0.00895
G1 X86.605 Y131.013 E0.11400
G1 X86.013 Y131.013 E0.01546
G1 X89.101 Y127.926 E0.11400
G1 X88.508 Y127.926 E0.01546
G1 X85.421 Y131.013 E0.11400
G1 X84.829 Y131.013 E0.01546
G1 X87.916 Y127.926 E0.11400
G1 X87.324 Y127.926 E0.01546
G1 X84.237 Y131.013 E0.11400
G1 X83.645 Y131.013 E0.01546
G1 X86.732 Y127.926 E0.11400
G1 X86.140 Y127.926 E0.01546
G1 X83.045 Y131.021 E0.11428
G1 X82.414 Y131.060 E0.01651
G1 X85.548 Y127.926 E0.11572
G1 X84.956 Y127.926 E0.01546
G1 X81.704 Y131.178 E0.12009
G1 X81.412 Y131.233 E0.00774
G1 X80.891 Y131.399 E0.01428
G1 X84.364 Y127.926 E0.12823
G1 X83.772 Y127.926 E0.01546
```

```
G1 X79.882 Y131.815 E0.14362
G1 X79.736 Y131.883 E0.00420
G1 X78.970 Y132.357 E0.02352
G1 X78.269 Y132.922 E0.02351
G1 X77.643 Y133.570 E0.02351
G1 X77.101 Y134.289 E0.02352
G1 X76.682 Y135.016 E0.02190
G1 X75.684 Y136.013 E0.03684
G1 X75.092 Y136.013 E0.01546
G1 X83.180 Y127.926 E0.29862
G1 X82.622 Y127.892 E0.01460
G1 X74.500 Y136.013 E0.29986
G1 X73.908 Y136.013 E0.01546
G1 X82.089 Y127.833 E0.30204
G1 X81.590 Y127.740 E0.01325
G1 X73.316 Y136.013 E0.30549
G1 X72.724 Y136.013 E0.01546
G1 X81.123 Y127.614 E0.31012
G1 X80.674 Y127.471 E0.01230
G1 X72.132 Y136.013 E0.31540
G1 X71.540 Y136.013 E0.01546
G1 X80.257 Y127.296 E0.32186
G1 X79.852 Y127.109 E0.01165
G1 X70.948 Y136.013 E0.32875
G1 X70.356 Y136.013 E0.01546
G1 X79.475 Y126.894 E0.33670
G1 X79.109 Y126.668 E0.01123
G1 X69.948 Y135.829 E0.33826
G1 X69.948 Y135.237 E0.01546
```

```
G1 X78.767 Y126.418 E0.32563
G1 X78.439 Y126.154 E0.01099
G1 X69.948 Y134.645 E0.31352
G1 X69.948 Y134.053 E0.01546
G1 X78.129 Y125.872 E0.30207
G1 X77.838 Y125.571 E0.01093
G1 X69.948 Y133.461 E0.29132
G1 X69.948 Y132.869 E0.01546
G1 X77.559 Y125.258 E0.28101
G1 X77.304 Y124.920 E0.01104
G1 X69.948 Y132.277 E0.27162
G1 X69.948 Y131.685 E0.01546
G1 X77.057 Y124.575 E0.26250
G1 X76.841 Y124.199 E0.01132
G1 X69.948 Y131.093 E0.25451
G1 X69.948 Y130.501 E0.01546
G1 X76.630 Y123.819 E0.24671
G1 X76.454 Y123.402 E0.01180
G1 X69.948 Y129.909 E0.24022
G1 X69.948 Y129.316 E0.01546
G1 X76.284 Y122.980 E0.23395
G1 X76.269 Y122.926 E0.00148
G1 X75.747 Y122.926 E0.01363
G1 X69.948 Y128.724 E0.21411
G1 X69.948 Y128.132 E0.01546
G1 X75.155 Y122.926 E0.19225
G1 X74.563 Y122.926 E0.01546
G1 X69.948 Y127.540 E0.17039
G1 X69.948 Y126.948 E0.01546
```

```
G1 X73.971 Y122.926 E0.14853
G1 X73.378 Y122.926 E0.01546
G1 X69.948 Y126.356 E0.12666
G1 X69.948 Y125.764 E0.01546
G1 X72.786 Y122.926 E0.10480
G1 X72.194 Y122.926 E0.01546
G1 X69.948 Y125.172 E0.08294
G1 X69.948 Y124.580 E0.01546
G1 X71.602 Y122.926 E0.06108
G1 X71.010 Y122.926 E0.01546
G1 X69.948 Y123.988 E0.03922
G1 X69.948 Y123.396 E0.01546
G1 X70.606 Y122.738 E0.02431
M204 S1250
; stop printing object tpu print.STL id:7 copy 0
; printing object tpu print.STL id:3 copy 0
G1 E-2.24000 F2400.000
;WIPE_START
G1 F7200.000
G1 X69.948 Y123.396 E-0.29480
G1 X69.948 Y123.988 E-0.18749
G1 X70.907 Y123.028 E-0.42971
;WIPE_END
G1 E-0.04800 F2400.000
G1 Z0.850 F9000.000
G1 X69.650 Y117.673
G1 Z0.650
G1 E3.20000 F1500.000
M204 S800
```

```
;TYPE:Perimeter
;WIDTH:0.45
G1 F2400.000
G1 X69.650 Y103.958 E0.35734
G1 X76.521 Y103.958 E0.17903
G1 X76.612 Y104.282 E0.00876
G1 X76.947 Y105.076 E0.02246
G1 X77.378 Y105.823 E0.02246
G1 X77.896 Y106.511 E0.02246
G1 X78.496 Y107.131 E0.02246
G1 X79.167 Y107.672 E0.02246
G1 X79.900 Y108.126 E0.02246
G1 X80.683 Y108.486 E0.02246
G1 X81.504 Y108.748 E0.02246
G1 X82.351 Y108.906 E0.02246
G1 X83.192 Y108.958 E0.02194
G1 X90.364 Y108.958 E0.18688
G1 X90.364 Y112.673 E0.09678
G1 X83.192 Y112.673 E0.18688
G1 X82.351 Y112.725 E0.02194
G1 X81.504 Y112.883 E0.02246
G1 X80.683 Y113.145 E0.02246
G1 X79.900 Y113.505 E0.02246
G1 X79.167 Y113.960 E0.02246
G1 X78.496 Y114.500 E0.02246
G1 X77.896 Y115.120 E0.02246
G1 X77.378 Y115.808 E0.02246
G1 X76.947 Y116.555 E0.02246
G1 X76.612 Y117.349 E0.02246
```

```
G1 X76.521 Y117.673 E0.00876
G1 X69.710 Y117.673 E0.17747
M204 S1250
G1 X69.232 Y118.091 F9000.000
M204 S800
;TYPE:External perimeter
G1 F1800.000
G1 X69.232 Y103.541 E0.37911
G1 X76.837 Y103.541 E0.19815
G1 X77.007 Y104.143 E0.01632
G1 X77.322 Y104.890 E0.02112
G1 X77.727 Y105.592 E0.02112
G1 X78.215 Y106.239 E0.02112
G1 X78.778 Y106.822 E0.02112
G1 X79.409 Y107.330 E0.02112
G1 X80.098 Y107.757 E0.02112
G1 X80.834 Y108.096 E0.02112
G1 X81.606 Y108.342 E0.02112
G1 X82.403 Y108.491 E0.02112
G1 X83.205 Y108.541 E0.02093
G1 X90.782 Y108.541 E0.19743
G1 X90.782 Y113.091 E0.11855
G1 X83.205 Y113.091 E0.19743
G1 X82.403 Y113.140 E0.02093
G1 X81.606 Y113.289 E0.02112
G1 X80.834 Y113.535 E0.02112
G1 X80.098 Y113.874 E0.02112
G1 X79.409 Y114.301 E0.02112
G1 X78.778 Y114.810 E0.02112
```

```
G1 X78.215 Y115.392 E0.02112
G1 X77.727 Y116.039 E0.02112
G1 X77.322 Y116.741 E0.02112
G1 X77.007 Y117.488 E0.02112
G1 X76.837 Y118.091 E0.01632
G1 X69.292 Y118.091 E0.19659
M204 S1250
G1 X69.423 Y117.739 F9000.000
G1 E-2.24000 F2400.000
;WIPE_START
G1 F7200.000
G1 X69.280 Y115.211 E-0.91200
;WIPE_END
G1 E-0.04800 F2400.000
G1 Z0.850 F9000.000
G1 X89.392 Y112.547
G1 Z0.650
G1 E3.20000 F1500.000
M204 S1000
;TYPE:Solid infill
;WIDTH:0.450839
G1 F4800.000
G1 X90.051 Y111.889 E0.02431
G1 X90.051 Y111.297 E0.01546
G1 X88.988 Y112.359 E0.03923
G1 X88.396 Y112.359 E0.01546
G1 X90.051 Y110.705 E0.06109
G1 X90.051 Y110.113 E0.01546
G1 X87.804 Y112.359 E0.08295
```

G1 X87.212 Y112.359 E0.01546

G1 X90.051 Y109.521 E0.10481

G1 X90.051 Y109.272 E0.00650

G1 X89.708 Y109.272 E0.00895

G1 X86.620 Y112.359 E0.11400

G1 X86.028 Y112.359 E0.01546

G1 X89.116 Y109.272 E0.11400

G1 X88.524 Y109.272 E0.01546

G1 X85.436 Y112.359 E0.11400

G1 X84.844 Y112.359 E0.01546

G1 X87.932 Y109.272 E0.11400

G1 X87.339 Y109.272 E0.01546

G1 X84.252 Y112.359 E0.11400

G1 X83.660 Y112.359 E0.01546

G1 X86.747 Y109.272 E0.11400

G1 X86.155 Y109.272 E0.01546

G1 X83.060 Y112.367 E0.11428

G1 X82.429 Y112.406 E0.01651

G1 X85.563 Y109.272 E0.11572

G1 X84.971 Y109.272 E0.01546

G1 X81.719 Y112.524 E0.12009

G1 X81.427 Y112.579 E0.00774

G1 X80.906 Y112.745 E0.01428

G1 X84.379 Y109.272 E0.12823

G1 X83.787 Y109.272 E0.01546

G1 X79.897 Y113.161 E0.14362

G1 X79.751 Y113.229 E0.00420

G1 X78.986 Y113.703 E0.02352

G1 X78.284 Y114.269 E0.02351

G1 X77.658 Y114.916 E0.02351

G1 X77.116 Y115.635 E0.02352

G1 X76.697 Y116.362 E0.02190

G1 X75.699 Y117.359 E0.03684

G1 X75.107 Y117.359 E0.01546

G1 X83.195 Y109.272 E0.29862

G1 X82.637 Y109.238 E0.01460

G1 X74.515 Y117.359 E0.29986

G1 X73.923 Y117.359 E0.01546

G1 X82.104 Y109.179 E0.30204

G1 X81.605 Y109.086 E0.01325

G1 X73.331 Y117.359 E0.30549

G1 X72.739 Y117.359 E0.01546

G1 X81.138 Y108.960 E0.31012

G1 X80.689 Y108.817 E0.01230

G1 X72.147 Y117.359 E0.31540

G1 X71.555 Y117.359 E0.01546

G1 X80.272 Y108.642 E0.32186

G1 X79.867 Y108.456 E0.01165

G1 X70.963 Y117.359 E0.32875

G1 X70.371 Y117.359 E0.01546

G1 X79.490 Y108.240 E0.33670

G1 X79.124 Y108.014 E0.01123

G1 X69.963 Y117.175 E0.33826

G1 X69.963 Y116.583 E0.01546

G1 X78.782 Y107.764 E0.32563

G1 X78.454 Y107.500 E0.01099

G1 X69.963 Y115.991 E0.31352

M73 P56 R30

```
G1 X69.963 Y115.399 E0.01546
G1 X78.144 Y107.218 E0.30207
G1 X77.853 Y106.917 E0.01093
G1 X69.963 Y114.807 E0.29132
G1 X69.963 Y114.215 E0.01546
G1 X77.574 Y106.604 E0.28101
G1 X77.319 Y106.266 E0.01104
G1 X69.963 Y113.623 E0.27162
G1 X69.963 Y113.031 E0.01546
G1 X77.073 Y105.921 E0.26250
G1 X76.856 Y105.546 E0.01132
G1 X69.963 Y112.439 E0.25451
G1 X69.963 Y111.847 E0.01546
G1 X76.645 Y105.165 E0.24671
G1 X76.469 Y104.748 E0.01180
G1 X69.963 Y111.255 E0.24022
G1 X69.963 Y110.663 E0.01546
G1 X76.299 Y104.326 E0.23395
G1 X76.284 Y104.272 E0.00148
G1 X75.762 Y104.272 E0.01363
G1 X69.963 Y110.070 E0.21411
G1 X69.963 Y109.478 E0.01546
G1 X75.170 Y104.272 E0.19225
G1 X74.578 Y104.272 E0.01546
G1 X69.963 Y108.886 E0.17039
G1 X69.963 Y108.294 E0.01546
G1 X73.986 Y104.272 E0.14853
G1 X73.394 Y104.272 E0.01546
G1 X69.963 Y107.702 E0.12666
```

```
G1 X69.963 Y107.110 E0.01546
G1 X72.802 Y104.272 E0.10480
G1 X72.209 Y104.272 E0.01546
G1 X69.963 Y106.518 E0.08294
G1 X69.963 Y105.926 E0.01546
G1 X71.617 Y104.272 E0.06108
G1 X71.025 Y104.272 E0.01546
G1 X69.963 Y105.334 E0.03922
G1 X69.963 Y104.742 E0.01546
G1 X70.621 Y104.084 E0.02431
M204 S1250
; stop printing object tpu print.STL id:3 copy 0
; printing object Petg print.STL id:2 copy 0
; stop printing object Petg print.STL id:2 copy 0
; printing object Petg print.STL id:6 copy 0
; stop printing object Petg print.STL id:6 copy 0
; printing object tpu print.STL id:27 copy 0
G1 E-2.24000 F2400.000
;WIPE_START
G1 F7200.000
G1 X69.963 Y104.742 E-0.29480
G1 X69.963 Y105.334 E-0.18749
G1 X70.923 Y104.374 E-0.42971
;WIPE_END
G1 E-0.04800 F2400.000
G1 Z0.850 F9000.000
G1 X120.285 Y122.787
G1 Z0.650
G1 E3.20000 F1500.000
```

```
M204 S800
;TYPE:Perimeter
;WIDTH:0.45
G1 F2400.000
G1 X127.156 Y122.787 E0.17903
G1 X127.247 Y123.111 E0.00876
G1 X127.583 Y123.905 E0.02246
G1 X128.013 Y124.652 E0.02246
G1 X128.532 Y125.340 E0.02246
G1 X129.131 Y125.960 E0.02246
G1 X129.802 Y126.501 E0.02246
G1 X130.535 Y126.955 E0.02246
G1 X131.318 Y127.315 E0.02246
G1 X132.139 Y127.577 E0.02246
G1 X132.986 Y127.735 E0.02246
G1 X133.827 Y127.787 E0.02194
G1 X140.999 Y127.787 E0.18688
G1 X140.999 Y131.502 E0.09678
G1 X133.827 Y131.502 E0.18688
G1 X132.986 Y131.554 E0.02194
G1 X132.139 Y131.712 E0.02246
G1 X131.318 Y131.974 E0.02246
G1 X130.535 Y132.334 E0.02246
G1 X129.802 Y132.789 E0.02246
G1 X129.131 Y133.329 E0.02246
G1 X128.532 Y133.949 E0.02246
G1 X128.013 Y134.637 E0.02246
G1 X127.583 Y135.384 E0.02246
G1 X127.247 Y136.178 E0.02246
```

```
G1 X127.156 Y136.502 E0.00876
G1 X120.285 Y136.502 E0.17903
G1 X120.285 Y122.847 E0.35578
M204 S1250
G1 X119.867 Y122.370 F9000.000
M204 S800
;TYPE:External perimeter
G1 F1800.000
G1 X127.472 Y122.370 E0.19815
G1 X127.642 Y122.972 E0.01632
G1 X127.957 Y123.719 E0.02112
G1 X128.362 Y124.421 E0.02112
G1 X128.850 Y125.068 E0.02112
G1 X129.413 Y125.651 E0.02112
G1 X130.044 Y126.159 E0.02112
G1 X130.733 Y126.586 E0.02112
G1 X131.469 Y126.925 E0.02112
G1 X132.241 Y127.171 E0.02112
G1 X133.038 Y127.320 E0.02112
G1 X133.840 Y127.370 E0.02093
G1 X141.417 Y127.370 E0.19743
G1 X141.417 Y131.920 E0.11855
G1 X133.840 Y131.920 E0.19743
G1 X133.038 Y131.969 E0.02093
G1 X132.241 Y132.118 E0.02112
G1 X131.469 Y132.364 E0.02112
G1 X130.733 Y132.703 E0.02112
G1 X130.044 Y133.130 E0.02112
G1 X129.413 Y133.639 E0.02112
```

```
G1 X128.850 Y134.221 E0.02112
G1 X128.362 Y134.868 E0.02112
G1 X127.957 Y135.570 E0.02112
G1 X127.642 Y136.317 E0.02112
G1 X127.472 Y136.920 E0.01632
G1 X119.867 Y136.920 E0.19815
G1 X119.867 Y122.430 E0.37755
M204 S1250
G1 X120.241 Y122.512 F9000.000
G1 E-2.24000 F2400.000
;WIPE_START
G1 F7200.000
G1 X122.747 Y122.407 E-0.91200
;WIPE_END
G1 E-0.04800 F2400.000
G1 Z0.850 F9000.000
G1 X140.027 Y131.376
G1 Z0.650
G1 E3.20000 F1500.000
M204 S1000
;TYPE:Solid infill
;WIDTH:0.450839
G1 F4800.000
G1 X140.686 Y130.718 E0.02431
G1 X140.686 Y130.126 E0.01546
G1 X139.623 Y131.188 E0.03923
G1 X139.031 Y131.188 E0.01546
G1 X140.686 Y129.534 E0.06109
G1 X140.686 Y128.942 E0.01546
```

```
G1 X138.439 Y131.188 E0.08295
G1 X137.847 Y131.188 E0.01546
G1 X140.686 Y128.350 E0.10481
G1 X140.686 Y128.101 E0.00650
G1 X140.343 Y128.101 E0.00895
G1 X137.255 Y131.188 E0.11400
G1 X136.663 Y131.188 E0.01546
G1 X139.751 Y128.101 E0.11400
G1 X139.159 Y128.101 E0.01546
G1 X136.071 Y131.188 E0.11400
G1 X135.479 Y131.188 E0.01546
G1 X138.567 Y128.101 E0.11400
G1 X137.975 Y128.101 E0.01546
G1 X134.887 Y131.188 E0.11400
G1 X134.295 Y131.188 E0.01546
G1 X137.383 Y128.101 E0.11400
G1 X136.790 Y128.101 E0.01546
G1 X133.695 Y131.196 E0.11428
G1 X133.064 Y131.235 E0.01651
G1 X136.198 Y128.101 E0.11572
G1 X135.606 Y128.101 E0.01546
G1 X132.354 Y131.353 E0.12009
G1 X132.063 Y131.408 E0.00774
G1 X131.541 Y131.574 E0.01428
G1 X135.014 Y128.101 E0.12823
G1 X134.422 Y128.101 E0.01546
G1 X130.532 Y131.991 E0.14362
G1 X130.386 Y132.058 E0.00420
G1 X129.621 Y132.532 E0.02352
```

```
G1 X128.919 Y133.098 E0.02351
G1 X128.293 Y133.745 E0.02351
G1 X127.751 Y134.464 E0.02352
G1 X127.332 Y135.191 E0.02190
G1 X126.335 Y136.188 E0.03684
G1 X125.743 Y136.188 E0.01546
G1 X133.830 Y128.101 E0.29862
G1 X133.272 Y128.067 E0.01460
G1 X125.150 Y136.188 E0.29986
G1 X124.558 Y136.188 E0.01546
G1 X132.739 Y128.008 E0.30204
G1 X132.240 Y127.915 E0.01325
G1 X123.966 Y136.188 E0.30549
G1 X123.374 Y136.188 E0.01546
G1 X131.773 Y127.789 E0.31012
G1 X131.324 Y127.646 E0.01230
G1 X122.782 Y136.188 E0.31540
G1 X122.190 Y136.188 E0.01546
G1 X130.907 Y127.471 E0.32186
G1 X130.502 Y127.285 E0.01165
G1 X121.598 Y136.188 E0.32875
G1 X121.006 Y136.188 E0.01546
G1 X130.125 Y127.069 E0.33670
G1 X129.760 Y126.843 E0.01123
G1 X120.598 Y136.004 E0.33826
G1 X120.598 Y135.412 E0.01546
G1 X129.417 Y126.593 E0.32563
G1 X129.090 Y126.329 E0.01099
G1 X120.598 Y134.820 E0.31352
```

G1 X120.598 Y134.228 E0.01546

G1 X128.779 Y126.047 E0.30207

G1 X128.488 Y125.746 E0.01093

G1 X120.598 Y133.636 E0.29132

G1 X120.598 Y133.044 E0.01546

G1 X128.209 Y125.433 E0.28101

G1 X127.955 Y125.095 E0.01104

G1 X120.598 Y132.452 E0.27162

G1 X120.598 Y131.860 E0.01546

G1 X127.708 Y124.750 E0.26250

G1 X127.491 Y124.375 E0.01132

G1 X120.598 Y131.268 E0.25451

G1 X120.598 Y130.676 E0.01546

G1 X127.280 Y123.994 E0.24671

G1 X127.104 Y123.578 E0.01180

G1 X120.598 Y130.084 E0.24022

G1 X120.598 Y129.492 E0.01546

G1 X126.934 Y123.155 E0.23395

G1 X126.919 Y123.101 E0.00148

G1 X126.397 Y123.101 E0.01363

G1 X120.598 Y128.900 E0.21411

G1 X120.598 Y128.307 E0.01546

G1 X125.805 Y123.101 E0.19225

G1 X125.213 Y123.101 E0.01546

G1 X120.598 Y127.715 E0.17039

G1 X120.598 Y127.123 E0.01546

G1 X124.621 Y123.101 E0.14853

G1 X124.029 Y123.101 E0.01546

G1 X120.598 Y126.531 E0.12666

```
G1 X120.598 Y125.939 E0.01546
G1 X123.437 Y123.101 E0.10480
G1 X122.845 Y123.101 E0.01546
G1 X120.598 Y125.347 E0.08294
G1 X120.598 Y124.755 E0.01546
G1 X122.253 Y123.101 E0.06108
G1 X121.660 Y123.101 E0.01546
G1 X120.598 Y124.163 E0.03922
G1 X120.598 Y123.571 E0.01546
G1 X121.256 Y122.913 E0.02431
M204 S1250
; stop printing object tpu print.STL id:27 copy 0
; printing object tpu print.STL id:23 copy 0
G1 E-2.24000 F2400.000
;WIPE_START
G1 F7200.000
G1 X120.598 Y123.571 E-0.29480
G1 X120.598 Y124.163 E-0.18749
G1 X121.558 Y123.204 E-0.42971
;WIPE_END
G1 E-0.04800 F2400.000
G1 Z0.850 F9000.000
G1 X120.300 Y117.848
G1 Z0.650
G1 E3.20000 F1500.000
M204 S800
;TYPE:Perimeter
;WIDTH:0.45
G1 F2400.000
```

```
G1 X120.300 Y104.133 E0.35734
G1 X127.171 Y104.133 E0.17903
G1 X127.263 Y104.457 E0.00876
G1 X127.598 Y105.251 E0.02246
G1 X128.028 Y105.998 E0.02246
G1 X128.547 Y106.686 E0.02246
G1 X129.146 Y107.306 E0.02246
G1 X129.817 Y107.847 E0.02246
G1 X130.550 Y108.301 E0.02246
G1 X131.333 Y108.661 E0.02246
G1 X132.154 Y108.923 E0.02246
G1 X133.002 Y109.082 E0.02246
G1 X133.842 Y109.133 E0.02194
G1 X141.014 Y109.133 E0.18688
G1 X141.014 Y112.848 E0.09678
G1 X133.842 Y112.848 E0.18688
G1 X133.002 Y112.900 E0.02194
G1 X132.154 Y113.058 E0.02246
G1 X131.333 Y113.320 E0.02246
G1 X130.550 Y113.680 E0.02246
G1 X129.817 Y114.135 E0.02246
G1 X129.146 Y114.675 E0.02246
G1 X128.547 Y115.295 E0.02246
G1 X128.028 Y115.983 E0.02246
G1 X127.598 Y116.730 E0.02246
G1 X127.263 Y117.524 E0.02246
G1 X127.171 Y117.848 E0.00876
G1 X120.360 Y117.848 E0.17747
M204 S1250
```

G1 X119.882 Y118.266 F9000.000

M204 S800

;TYPE:External perimeter

G1 F1800.000

G1 X119.882 Y103.716 E0.37911

G1 X127.487 Y103.716 E0.19815

G1 X127.658 Y104.318 E0.01632

G1 X127.973 Y105.065 E0.02112

G1 X128.377 Y105.767 E0.02112

G1 X128.865 Y106.414 E0.02112

G1 X129.428 Y106.997 E0.02112

G1 X130.059 Y107.505 E0.02112

G1 X130.748 Y107.932 E0.02112

G1 X131.484 Y108.271 E0.02112

G1 X132.257 Y108.517 E0.02112

G1 X133.053 Y108.666 E0.02112

G1 X133.855 Y108.716 E0.02093

G1 X141.432 Y108.716 E0.19743

G1 X141.432 Y113.266 E0.11855

G1 X133.855 Y113.266 E0.19743

G1 X133.053 Y113.315 E0.02093

G1 X132.257 Y113.464 E0.02112

G1 X131.484 Y113.710 E0.02112

G1 X130.748 Y114.049 E0.02112

G1 X130.059 Y114.476 E0.02112

G1 X129.428 Y114.985 E0.02112

G1 X128.865 Y115.567 E0.02112

G1 X128.377 Y116.214 E0.02112

G1 X127.973 Y116.916 E0.02112

```
G1 X127.658 Y117.663 E0.02112
G1 X127.487 Y118.266 E0.01632
G1 X119.942 Y118.266 E0.19659
M204 S1250
G1 X120.073 Y117.914 F9000.000
G1 E-2.24000 F2400.000
;WIPE_START
G1 F7200.000
G1 X119.930 Y115.386 E-0.91200
;WIPE_END
G1 E-0.04800 F2400.000
G1 Z0.850 F9000.000
G1 X140.043 Y112.722
G1 Z0.650
G1 E3.20000 F1500.000
M204 S1000
;TYPE:Solid infill
;WIDTH:0.450839
G1 F4800.000
G1 X140.701 Y112.064 E0.02431
G1 X140.701 Y111.472 E0.01546
G1 X139.639 Y112.534 E0.03923
G1 X139.046 Y112.534 E0.01546
G1 X140.701 Y110.880 E0.06109
G1 X140.701 Y110.288 E0.01546
G1 X138.454 Y112.534 E0.08295
G1 X137.862 Y112.534 E0.01546
G1 X140.701 Y109.696 E0.10481
G1 X140.701 Y109.447 E0.00650
```

G1 X140.358 Y109.447 E0.00895

G1 X137.270 Y112.534 E0.11400

G1 X136.678 Y112.534 E0.01546

G1 X139.766 Y109.447 E0.11400

G1 X139.174 Y109.447 E0.01546

G1 X136.086 Y112.534 E0.11400

G1 X135.494 Y112.534 E0.01546

G1 X138.582 Y109.447 E0.11400

G1 X137.990 Y109.447 E0.01546

G1 X134.902 Y112.534 E0.11400

G1 X134.310 Y112.534 E0.01546

G1 X137.398 Y109.447 E0.11400

G1 X136.806 Y109.447 E0.01546

G1 X133.710 Y112.542 E0.11428

G1 X133.079 Y112.581 E0.01651

G1 X136.214 Y109.447 E0.11572

G1 X135.621 Y109.447 E0.01546

G1 X132.369 Y112.699 E0.12009

G1 X132.078 Y112.754 E0.00774

G1 X131.556 Y112.920 E0.01428

G1 X135.029 Y109.447 E0.12823

G1 X134.437 Y109.447 E0.01546

G1 X130.548 Y113.337 E0.14362

G1 X130.401 Y113.404 E0.00420

G1 X129.636 Y113.878 E0.02352

G1 X128.935 Y114.444 E0.02351

G1 X128.308 Y115.091 E0.02351

G1 X127.766 Y115.810 E0.02352

G1 X127.347 Y116.537 E0.02190

G1 X126.350 Y117.534 E0.03684

G1 X125.758 Y117.534 E0.01546

G1 X133.845 Y109.447 E0.29862

G1 X133.287 Y109.413 E0.01460

G1 X125.166 Y117.534 E0.29986

G1 X124.574 Y117.534 E0.01546

G1 X132.754 Y109.354 E0.30204

G1 X132.255 Y109.261 E0.01325

G1 X123.981 Y117.534 E0.30549

G1 X123.389 Y117.534 E0.01546

G1 X131.788 Y109.135 E0.31012

G1 X131.339 Y108.992 E0.01230

G1 X122.797 Y117.534 E0.31540

G1 X122.205 Y117.534 E0.01546

G1 X130.922 Y108.817 E0.32186

G1 X130.517 Y108.631 E0.01165

G1 X121.613 Y117.534 E0.32875

G1 X121.021 Y117.534 E0.01546

G1 X130.140 Y108.415 E0.33670

G1 X129.775 Y108.189 E0.01123

G1 X120.613 Y117.350 E0.33826

G1 X120.613 Y116.758 E0.01546

G1 X129.433 Y107.939 E0.32563

G1 X129.105 Y107.675 E0.01099

G1 X120.613 Y116.166 E0.31352

G1 X120.613 Y115.574 E0.01546

G1 X128.794 Y107.393 E0.30207

G1 X128.503 Y107.092 E0.01093

G1 X120.613 Y114.982 E0.29132

```
G1 X120.613 Y114.390 E0.01546
G1 X128.224 Y106.779 E0.28101
G1 X127.970 Y106.441 E0.01104
G1 X120.613 Y113.798 E0.27162
G1 X120.613 Y113.206 E0.01546
G1 X127.723 Y106.096 E0.26250
G1 X127.506 Y105.721 E0.01132
G1 X120.613 Y112.614 E0.25451
G1 X120.613 Y112.022 E0.01546
G1 X127.295 Y105.340 E0.24671
G1 X127.119 Y104.924 E0.01180
G1 X120.613 Y111.430 E0.24022
G1 X120.613 Y110.838 E0.01546
G1 X126.950 Y104.501 E0.23395
G1 X126.934 Y104.447 E0.00148
G1 X126.412 Y104.447 E0.01363
G1 X120.613 Y110.246 E0.21411
G1 X120.613 Y109.654 E0.01546
G1 X125.820 Y104.447 E0.19225
G1 X125.228 Y104.447 E0.01546
G1 X120.613 Y109.061 E0.17039
G1 X120.613 Y108.469 E0.01546
G1 X124.636 Y104.447 E0.14853
G1 X124.044 Y104.447 E0.01546
G1 X120.613 Y107.877 E0.12666
G1 X120.613 Y107.285 E0.01546
G1 X123.452 Y104.447 E0.10480
G1 X122.860 Y104.447 E0.01546
G1 X120.613 Y106.693 E0.08294
```

```
G1 X120.613 Y106.101 E0.01546
G1 X122.268 Y104.447 E0.06108
G1 X121.676 Y104.447 E0.01546
G1 X120.613 Y105.509 E0.03922
G1 X120.613 Y104.917 E0.01546
G1 X121.272 Y104.259 E0.02431
M204 S1250
; stop printing object tpu print.STL id:23 copy 0
; printing object tpu print.STL id:21 copy 0
G1 E-2.24000 F2400.000
;WIPE_START
M73 P56 R29
G1 F7200.000
G1 X120.613 Y104.917 E-0.29480
G1 X120.613 Y105.509 E-0.18749
G1 X121.573 Y104.550 E-0.42971
;WIPE_END
G1 E-0.04800 F2400.000
G1 Z0.850 F9000.000
G1 X120.293 Y97.032
G1 Z0.650
G1 E3.20000 F1500.000
M204 S800
;TYPE:Perimeter
;WIDTH:0.45
G1 F2400.000
G1 X120.293 Y83.318 E0.35734
G1 X127.164 Y83.318 E0.17903
G1 X127.256 Y83.641 E0.00876
```

```
G1 X127.591 Y84.436 E0.02246
G1 X128.021 Y85.183 E0.02246
G1 X128.540 Y85.871 E0.02246
G1 X129.139 Y86.490 E0.02246
G1 X129.811 Y87.031 E0.02246
G1 X130.543 Y87.485 E0.02246
G1 X131.326 Y87.846 E0.02246
G1 X132.147 Y88.107 E0.02246
G1 X132.995 Y88.266 E0.02246
G1 X133.835 Y88.318 E0.02194
G1 X141.007 Y88.318 E0.18688
G1 X141.007 Y92.032 E0.09678
G1 X133.835 Y92.032 E0.18688
G1 X132.995 Y92.084 E0.02194
G1 X132.147 Y92.243 E0.02246
G1 X131.326 Y92.504 E0.02246
G1 X130.543 Y92.865 E0.02246
G1 X129.811 Y93.319 E0.02246
G1 X129.139 Y93.860 E0.02246
G1 X128.540 Y94.479 E0.02246
G1 X128.021 Y95.168 E0.02246
G1 X127.591 Y95.915 E0.02246
G1 X127.256 Y96.709 E0.02246
G1 X127.164 Y97.032 E0.00876
G1 X120.353 Y97.032 E0.17747
M204 S1250
G1 X119.875 Y97.450 F9000.000
M204 S800
;TYPE:External perimeter
```

```
G1 F1800.000
G1 X119.875 Y82.900 E0.37911
G1 X127.480 Y82.900 E0.19815
G1 X127.651 Y83.503 E0.01632
G1 X127.966 Y84.249 E0.02112
G1 X128.370 Y84.952 E0.02112
G1 X128.858 Y85.599 E0.02112
G1 X129.422 Y86.181 E0.02112
G1 X130.053 Y86.690 E0.02112
G1 X130.741 Y87.117 E0.02112
G1 X131.477 Y87.456 E0.02112
G1 X132.250 Y87.702 E0.02112
G1 X133.046 Y87.851 E0.02112
G1 X133.848 Y87.900 E0.02093
G1 X141.425 Y87.900 E0.19743
G1 X141.425 Y92.450 E0.11855
G1 X133.848 Y92.450 E0.19743
G1 X133.046 Y92.500 E0.02093
G1 X132.250 Y92.649 E0.02112
G1 X131.477 Y92.895 E0.02112
G1 X130.741 Y93.234 E0.02112
G1 X130.053 Y93.661 E0.02112
G1 X129.422 Y94.169 E0.02112
G1 X128.858 Y94.751 E0.02112
G1 X128.370 Y95.398 E0.02112
G1 X127.966 Y96.101 E0.02112
G1 X127.651 Y96.847 E0.02112
G1 X127.480 Y97.450 E0.01632
G1 X119.935 Y97.450 E0.19659
```

```
M204 S1250
G1 X120.066 Y97.099 F9000.000
G1 E-2.24000 F2400.000
;WIPE_START
G1 F7200.000
G1 X119.923 Y94.570 E-0.91200
;WIPE_END
G1 E-0.04800 F2400.000
G1 Z0.850 F9000.000
G1 X140.036 Y91.907
G1 Z0.650
G1 E3.20000 F1500.000
M204 S1000
;TYPE:Solid infill
;WIDTH:0.450839
G1 F4800.000
G1 X140.694 Y91.249 E0.02431
G1 X140.694 Y90.656 E0.01546
G1 X139.632 Y91.719 E0.03923
G1 X139.040 Y91.719 E0.01546
G1 X140.694 Y90.064 E0.06109
G1 X140.694 Y89.472 E0.01546
G1 X138.448 Y91.719 E0.08295
G1 X137.855 Y91.719 E0.01546
G1 X140.694 Y88.880 E0.10481
G1 X140.694 Y88.631 E0.00650
G1 X140.351 Y88.631 E0.00895
G1 X137.263 Y91.719 E0.11400
G1 X136.671 Y91.719 E0.01546
```

```
G1 X139.759 Y88.631 E0.11400
G1 X139.167 Y88.631 E0.01546
G1 X136.079 Y91.719 E0.11400
G1 X135.487 Y91.719 E0.01546
G1 X138.575 Y88.631 E0.11400
G1 X137.983 Y88.631 E0.01546
G1 X134.895 Y91.719 E0.11400
G1 X134.303 Y91.719 E0.01546
G1 X137.391 Y88.631 E0.11400
G1 X136.799 Y88.631 E0.01546
G1 X133.704 Y91.726 E0.11428
G1 X133.073 Y91.765 E0.01651
G1 X136.207 Y88.631 E0.11572
G1 X135.615 Y88.631 E0.01546
G1 X132.362 Y91.884 E0.12009
G1 X132.071 Y91.938 E0.00774
G1 X131.550 Y92.104 E0.01428
G1 X135.023 Y88.631 E0.12823
G1 X134.431 Y88.631 E0.01546
G1 X130.541 Y92.521 E0.14362
G1 X130.395 Y92.588 E0.00420
G1 X129.629 Y93.063 E0.02352
G1 X128.928 Y93.628 E0.02351
G1 X128.301 Y94.275 E0.02351
G1 X127.759 Y94.994 E0.02352
G1 X127.341 Y95.721 E0.02190
G1 X126.343 Y96.719 E0.03684
G1 X125.751 Y96.719 E0.01546
G1 X133.838 Y88.631 E0.29862
```

```
G1 X133.280 Y88.598 E0.01460
G1 X125.159 Y96.719 E0.29986
G1 X124.567 Y96.719 E0.01546
G1 X132.747 Y88.538 E0.30204
G1 X132.248 Y88.445 E0.01325
G1 X123.975 Y96.719 E0.30549
G1 X123.383 Y96.719 E0.01546
G1 X131.782 Y88.320 E0.31012
G1 X131.333 Y88.177 E0.01230
G1 X122.790 Y96.719 E0.31540
G1 X122.198 Y96.719 E0.01546
G1 X130.916 Y88.002 E0.32186
G1 X130.510 Y87.815 E0.01165
G1 X121.606 Y96.719 E0.32875
M73 P57 R29
G1 X121.014 Y96.719 E0.01546
G1 X130.133 Y87.600 E0.33670
G1 X129.768 Y87.373 E0.01123
G1 X120.606 Y96.535 E0.33826
G1 X120.606 Y95.943 E0.01546
G1 X129.426 Y87.123 E0.32563
G1 X129.098 Y86.859 E0.01099
G1 X120.606 Y95.351 E0.31352
G1 X120.606 Y94.759 E0.01546
G1 X128.788 Y86.577 E0.30207
G1 X128.496 Y86.277 E0.01093
G1 X120.606 Y94.167 E0.29132
G1 X120.606 Y93.574 E0.01546
G1 X128.217 Y85.964 E0.28101
```

```
G1 X127.963 Y85.626 E0.01104
G1 X120.606 Y92.982 E0.27162
G1 X120.606 Y92.390 E0.01546
G1 X127.716 Y85.281 E0.26250
G1 X127.500 Y84.905 E0.01132
G1 X120.606 Y91.798 E0.25451
G1 X120.606 Y91.206 E0.01546
G1 X127.288 Y84.524 E0.24671
G1 X127.113 Y84.108 E0.01180
G1 X120.606 Y90.614 E0.24022
G1 X120.606 Y90.022 E0.01546
G1 X126.943 Y83.686 E0.23395
G1 X126.927 Y83.631 E0.00148
G1 X126.405 Y83.631 E0.01363
G1 X120.606 Y89.430 E0.21411
G1 X120.606 Y88.838 E0.01546
G1 X125.813 Y83.631 E0.19225
G1 X125.221 Y83.631 E0.01546
G1 X120.606 Y88.246 E0.17039
G1 X120.606 Y87.654 E0.01546
G1 X124.629 Y83.631 E0.14853
G1 X124.037 Y83.631 E0.01546
G1 X120.606 Y87.062 E0.12666
G1 X120.606 Y86.470 E0.01546
G1 X123.445 Y83.631 E0.10480
G1 X122.853 Y83.631 E0.01546
G1 X120.606 Y85.878 E0.08294
G1 X120.606 Y85.286 E0.01546
G1 X122.261 Y83.631 E0.06108
```

```
G1 X121.669 Y83.631 E0.01546
G1 X120.606 Y84.694 E0.03922
G1 X120.606 Y84.102 E0.01546
G1 X121.265 Y83.443 E0.02431
M204 S1250
; stop printing object tpu print.STL id:21 copy 0
; printing object Petg print.STL id:20 copy 0
; stop printing object Petg print.STL id:20 copy 0
; printing object Petg print.STL id:22 copy 0
; stop printing object Petg print.STL id:22 copy 0
; printing object Petg print.STL id:26 copy 0
; stop printing object Petg print.STL id:26 copy 0
; printing object Petg print.STL id:0 copy 0
; stop printing object Petg print.STL id:0 copy 0
; printing object tpu print.STL id:1 copy 0
G1 E-2.24000 F2400.000
;WIPE_START
G1 F7200.000
G1 X120.606 Y84.102 E-0.29480
G1 X120.606 Y84.694 E-0.18749
G1 X121.566 Y83.734 E-0.42971
;WIPE_END
G1 E-0.04800 F2400.000
G1 Z0.850 F9000.000
G1 X90.357 Y88.143
G1 Z0.650
G1 E3.20000 F1500.000
M204 S800
;TYPE:Perimeter
```

```
;WIDTH:0.45
G1 F2400.000
G1 X90.357 Y91.857 E0.09678
G1 X83.185 Y91.857 E0.18688
G1 X82.344 Y91.909 E0.02194
G1 X81.497 Y92.068 E0.02246
G1 X80.676 Y92.329 E0.02246
G1 X79.893 Y92.690 E0.02246
G1 X79.160 Y93.144 E0.02246
G1 X78.489 Y93.685 E0.02246
G1 X77.890 Y94.304 E0.02246
G1 X77.371 Y94.993 E0.02246
G1 X76.941 Y95.739 E0.02246
G1 X76.606 Y96.534 E0.02246
G1 X76.514 Y96.857 E0.00876
G1 X69.643 Y96.857 E0.17903
G1 X69.643 Y83.143 E0.35734
G1 X76.514 Y83.143 E0.17903
G1 X76.606 Y83.466 E0.00876
G1 X76.941 Y84.261 E0.02246
G1 X77.371 Y85.007 E0.02246
G1 X77.890 Y85.696 E0.02246
G1 X78.489 Y86.315 E0.02246
G1 X79.160 Y86.856 E0.02246
G1 X79.893 Y87.310 E0.02246
G1 X80.676 Y87.671 E0.02246
G1 X81.497 Y87.932 E0.02246
G1 X82.344 Y88.091 E0.02246
G1 X83.185 Y88.143 E0.02194
```

```
G1 X90.297 Y88.143 E0.18531
M204 S1250
G1 X90.775 Y87.725 F9000.000
M204 S800
;TYPE:External perimeter
G1 F1800.000
G1 X90.775 Y92.275 E0.11855
G1 X83.198 Y92.275 E0.19743
G1 X82.396 Y92.325 E0.02093
G1 X81.599 Y92.474 E0.02112
G1 X80.827 Y92.719 E0.02112
G1 X80.091 Y93.059 E0.02112
G1 X79.402 Y93.486 E0.02112
G1 X78.771 Y93.994 E0.02112
G1 X78.208 Y94.576 E0.02112
G1 X77.720 Y95.223 E0.02112
G1 X77.315 Y95.926 E0.02112
G1 X77.000 Y96.672 E0.02112
G1 X76.830 Y97.275 E0.01632
G1 X69.225 Y97.275 E0.19815
G1 X69.225 Y82.725 E0.37911
G1 X76.830 Y82.725 E0.19815
G1 X77.000 Y83.328 E0.01632
G1 X77.315 Y84.074 E0.02112
G1 X77.720 Y84.777 E0.02112
G1 X78.208 Y85.424 E0.02112
G1 X78.771 Y86.006 E0.02112
G1 X79.402 Y86.514 E0.02112
G1 X80.091 Y86.941 E0.02112
```

```
G1 X80.827 Y87.281 E0.02112
G1 X81.599 Y87.526 E0.02112
G1 X82.396 Y87.675 E0.02112
G1 X83.198 Y87.725 E0.02093
G1 X90.715 Y87.725 E0.19586
M204 S1250
G1 X90.574 Y88.071 F9000.000
G1 E-2.24000 F2400.000
;WIPE_START
G1 F7200.000
G1 X90.753 Y90.605 E-0.91200
;WIPE_END
G1 E-0.04800 F2400.000
G1 Z0.850 F9000.000
G1 X70.614 Y83.268
G1 Z0.650
G1 E3.20000 F1500.000
M204 S1000
;TYPE:Solid infill
;WIDTH:0.450839
G1 F4800.000
G1 X69.956 Y83.926 E0.02431
G1 X69.956 Y84.518 E0.01546
G1 X71.018 Y83.456 E0.03922
G1 X71.611 Y83.456 E0.01546
G1 X69.956 Y85.111 E0.06108
G1 X69.956 Y85.703 E0.01546
G1 X72.203 Y83.456 E0.08294
G1 X72.795 Y83.456 E0.01546
```

```
G1 X69.956 Y86.295 E0.10480
G1 X69.956 Y86.887 E0.01546
G1 X73.387 Y83.456 E0.12666
G1 X73.979 Y83.456 E0.01546
G1 X69.956 Y87.479 E0.14853
G1 X69.956 Y88.071 E0.01546
G1 X74.571 Y83.456 E0.17039
G1 X75.163 Y83.456 E0.01546
G1 X69.956 Y88.663 E0.19225
G1 X69.956 Y89.255 E0.01546
G1 X75.755 Y83.456 E0.21411
G1 X76.277 Y83.456 E0.01363
G1 X76.292 Y83.511 E0.00148
G1 X69.956 Y89.847 E0.23395
G1 X69.956 Y90.439 E0.01546
G1 X76.462 Y83.933 E0.24022
G1 X76.638 Y84.349 E0.01180
G1 X69.956 Y91.031 E0.24671
G1 X69.956 Y91.623 E0.01546
G1 X76.849 Y84.730 E0.25451
G1 X77.066 Y85.106 E0.01132
G1 X69.956 Y92.215 E0.26250
G1 X69.956 Y92.807 E0.01546
G1 X77.313 Y85.451 E0.27162
G1 X77.567 Y85.789 E0.01104
G1 X69.956 Y93.399 E0.28101
G1 X69.956 Y93.991 E0.01546
G1 X77.846 Y86.101 E0.29132
G1 X78.137 Y86.402 E0.01093
```

G1 X69.956 Y94.583 E0.30207

G1 X69.956 Y95.176 E0.01546

G1 X78.448 Y86.684 E0.31352

G1 X78.775 Y86.948 E0.01099

G1 X69.956 Y95.768 E0.32563

G1 X69.956 Y96.360 E0.01546

G1 X79.118 Y87.198 E0.33826

G1 X79.483 Y87.425 E0.01123

G1 X70.364 Y96.544 E0.33670

G1 X70.956 Y96.544 E0.01546

G1 X79.860 Y87.640 E0.32875

G1 X80.265 Y87.827 E0.01165

G1 X71.548 Y96.544 E0.32186

G1 X72.140 Y96.544 E0.01546

G1 X80.682 Y88.002 E0.31540

G1 X81.131 Y88.145 E0.01230

G1 X72.732 Y96.544 E0.31012

G1 X73.324 Y96.544 E0.01546

G1 X81.598 Y88.270 E0.30549

G1 X82.097 Y88.363 E0.01325

G1 X73.916 Y96.544 E0.30204

G1 X74.508 Y96.544 E0.01546

G1 X82.630 Y88.422 E0.29986

G1 X83.188 Y88.456 E0.01460

G1 X75.101 Y96.544 E0.29862

G1 X75.693 Y96.544 E0.01546

G1 X76.690 Y95.546 E0.03684

G1 X77.109 Y94.819 E0.02190

G1 X77.651 Y94.100 E0.02352

```
G1 X78.277 Y93.453 E0.02351
G1 X78.979 Y92.888 E0.02351
G1 X79.744 Y92.413 E0.02352
G1 X79.890 Y92.346 E0.00420
G1 X83.780 Y88.456 E0.14362
G1 X84.372 Y88.456 E0.01546
G1 X80.899 Y91.929 E0.12823
G1 X81.421 Y91.763 E0.01428
G1 X81.712 Y91.709 E0.00774
G1 X84.964 Y88.456 E0.12009
G1 X85.556 Y88.456 E0.01546
G1 X82.422 Y91.590 E0.11572
G1 X83.053 Y91.551 E0.01651
G1 X86.149 Y88.456 E0.11428
G1 X86.741 Y88.456 E0.01546
G1 X83.653 Y91.544 E0.11400
G1 X84.245 Y91.544 E0.01546
G1 X87.333 Y88.456 E0.11400
G1 X87.925 Y88.456 E0.01546
G1 X84.837 Y91.544 E0.11400
G1 X85.429 Y91.544 E0.01546
G1 X88.517 Y88.456 E0.11400
G1 X89.109 Y88.456 E0.01546
G1 X86.021 Y91.544 E0.11400
G1 X86.613 Y91.544 E0.01546
G1 X89.701 Y88.456 E0.11400
G1 X90.044 Y88.456 E0.00895
G1 X90.044 Y88.705 E0.00650
G1 X87.205 Y91.544 E0.10481
```

```
G1 X87.797 Y91.544 E0.01546
G1 X90.044 Y89.297 E0.08295
G1 X90.044 Y89.889 E0.01546
G1 X88.389 Y91.544 E0.06109
G1 X88.981 Y91.544 E0.01546
G1 X90.044 Y90.481 E0.03923
G1 X90.044 Y91.073 E0.01546
G1 X89.385 Y91.732 E0.02431
M204 S1250
; stop printing object tpu print.STL id:1 copy 0
; printing object tpu print.STL id:5 copy 0
G1 E-2.24000 F2400.000
;WIPE_START
G1 F7200.000
G1 X90.044 Y91.073 E-0.29484
G1 X90.044 Y90.481 E-0.18749
G1 X89.084 Y91.441 E-0.42968
;WIPE_END
G1 E-0.04800 F2400.000
G1 Z0.850 F9000.000
G1 X76.565 Y74.665
G1 Z0.650
G1 E3.20000 F1500.000
M204 S800
;TYPE:Perimeter
;WIDTH:0.45
G1 F2400.000
G1 X69.694 Y74.665 E0.17903
G1 X69.694 Y60.950 E0.35734
```

```
G1 X76.565 Y60.950 E0.17903
G1 X76.657 Y61.274 E0.00876
G1 X76.992 Y62.068 E0.02246
G1 X77.422 Y62.815 E0.02246
G1 X77.941 Y63.503 E0.02246
G1 X78.540 Y64.123 E0.02246
G1 X79.211 Y64.664 E0.02246
G1 X79.944 Y65.118 E0.02246
G1 X80.727 Y65.478 E0.02246
G1 X81.548 Y65.740 E0.02246
G1 X82.396 Y65.899 E0.02246
G1 X83.236 Y65.950 E0.02194
G1 X90.408 Y65.950 E0.18688
G1 X90.408 Y69.665 E0.09678
G1 X83.236 Y69.665 E0.18688
G1 X82.396 Y69.717 E0.02194
G1 X81.548 Y69.875 E0.02246
G1 X80.727 Y70.137 E0.02246
G1 X79.944 Y70.497 E0.02246
G1 X79.211 Y70.952 E0.02246
G1 X78.540 Y71.493 E0.02246
G1 X77.941 Y72.112 E0.02246
G1 X77.422 Y72.800 E0.02246
G1 X76.992 Y73.547 E0.02246
G1 X76.657 Y74.341 E0.02246
G1 X76.581 Y74.607 E0.00720
M204 S1250
G1 X76.881 Y75.083 F9000.000
M204 S800
```

```
;TYPE:External perimeter
G1 F1800.000
G1 X69.276 Y75.083 E0.19815
G1 X69.276 Y60.533 E0.37911
G1 X76.881 Y60.533 E0.19815
G1 X77.052 Y61.135 E0.01632
G1 X77.367 Y61.882 E0.02112
G1 X77.771 Y62.584 E0.02112
G1 X78.259 Y63.231 E0.02112
G1 X78.823 Y63.814 E0.02112
G1 X79.454 Y64.322 E0.02112
G1 X80.142 Y64.749 E0.02112
G1 X80.878 Y65.088 E0.02112
G1 X81.651 Y65.334 E0.02112
G1 X82.447 Y65.483 E0.02112
G1 X83.249 Y65.533 E0.02093
G1 X90.826 Y65.533 E0.19743
G1 X90.826 Y70.083 E0.11855
G1 X83.249 Y70.083 E0.19743
G1 X82.447 Y70.132 E0.02093
G1 X81.651 Y70.281 E0.02112
G1 X80.878 Y70.527 E0.02112
G1 X80.142 Y70.866 E0.02112
G1 X79.454 Y71.293 E0.02112
G1 X78.823 Y71.802 E0.02112
G1 X78.259 Y72.384 E0.02112
G1 X77.771 Y73.031 E0.02112
G1 X77.367 Y73.733 E0.02112
G1 X77.052 Y74.480 E0.02112
```

```
G1 X76.897 Y75.025 E0.01476
M204 S1250
G1 X76.560 Y74.844 F9000.000
G1 E-2.24000 F2400.000
;WIPE_START
G1 F7200.000
G1 X74.017 Y75.047 E-0.91200
;WIPE_END
G1 E-0.04800 F2400.000
G1 Z0.850 F9000.000
G1 X70.666 Y61.076
G1 Z0.650
G1 E3.20000 F1500.000
M204 S1000
;TYPE:Solid infill
;WIDTH:0.450839
G1 F4800.000
G1 X70.007 Y61.734 E0.02431
G1 X70.007 Y62.326 E0.01546
G1 X71.070 Y61.264 E0.03922
G1 X71.662 Y61.264 E0.01546
G1 X70.007 Y62.918 E0.06108
G1 X70.007 Y63.510 E0.01546
G1 X72.254 Y61.264 E0.08294
G1 X72.846 Y61.264 E0.01546
G1 X70.007 Y64.102 E0.10480
G1 X70.007 Y64.694 E0.01546
G1 X73.438 Y61.264 E0.12666
G1 X74.030 Y61.264 E0.01546
```

```
G1 X70.007 Y65.286 E0.14853
G1 X70.007 Y65.878 E0.01546
G1 X74.622 Y61.264 E0.17039
G1 X75.214 Y61.264 E0.01546
G1 X70.007 Y66.471 E0.19225
G1 X70.007 Y67.063 E0.01546
G1 X75.806 Y61.264 E0.21411
G1 X76.328 Y61.264 E0.01363
G1 X76.344 Y61.318 E0.00148
G1 X70.007 Y67.655 E0.23395
G1 X70.007 Y68.247 E0.01546
G1 X76.513 Y61.741 E0.24022
G1 X76.689 Y62.157 E0.01180
G1 X70.007 Y68.839 E0.24671
G1 X70.007 Y69.431 E0.01546
G1 X76.900 Y62.538 E0.25451
G1 X77.117 Y62.913 E0.01132
G1 X70.007 Y70.023 E0.26250
G1 X70.007 Y70.615 E0.01546
G1 X77.364 Y63.259 E0.27162
G1 X77.618 Y63.596 E0.01104
G1 X70.007 Y71.207 E0.28101
G1 X70.007 Y71.799 E0.01546
G1 X77.897 Y63.909 E0.29132
G1 X78.189 Y64.210 E0.01093
G1 X70.007 Y72.391 E0.30207
G1 X70.007 Y72.983 E0.01546
G1 X78.499 Y64.492 E0.31352
G1 X78.827 Y64.756 E0.01099
```

G1 X70.007 Y73.575 E0.32563

G1 X70.007 Y74.167 E0.01546

G1 X79.169 Y65.006 E0.33826

G1 X79.534 Y65.232 E0.01123

G1 X70.415 Y74.351 E0.33670

G1 X71.007 Y74.351 E0.01546

G1 X79.911 Y65.448 E0.32875

G1 X80.316 Y65.634 E0.01165

G1 X71.599 Y74.351 E0.32186

G1 X72.191 Y74.351 E0.01546

G1 X80.733 Y65.809 E0.31540

G1 X81.183 Y65.952 E0.01230

G1 X72.783 Y74.351 E0.31012

G1 X73.376 Y74.351 E0.01546

G1 X81.649 Y66.078 E0.30549

G1 X82.148 Y66.171 E0.01325

G1 X73.968 Y74.351 E0.30204

G1 X74.560 Y74.351 E0.01546

G1 X82.681 Y66.230 E0.29986

G1 X83.239 Y66.264 E0.01460

G1 X75.152 Y74.351 E0.29862

G1 X75.744 Y74.351 E0.01546

G1 X76.741 Y73.354 E0.03684

G1 X77.160 Y72.627 E0.02190

G1 X77.702 Y71.908 E0.02352

G1 X78.329 Y71.261 E0.02351

G1 X79.030 Y70.696 E0.02351

G1 X79.795 Y70.221 E0.02352

G1 X79.942 Y70.154 E0.00420

G1 X83.831 Y66.264 E0.14362

G1 X84.423 Y66.264 E0.01546

G1 X80.950 Y69.737 E0.12823

G1 X81.472 Y69.571 E0.01428

G1 X81.763 Y69.516 E0.00774

G1 X85.016 Y66.264 E0.12009

G1 X85.608 Y66.264 E0.01546

G1 X82.473 Y69.398 E0.11572

G1 X83.104 Y69.359 E0.01651

G1 X86.200 Y66.264 E0.11428

G1 X86.792 Y66.264 E0.01546

G1 X83.704 Y69.351 E0.11400

G1 X84.296 Y69.351 E0.01546

G1 X87.384 Y66.264 E0.11400

G1 X87.976 Y66.264 E0.01546

G1 X84.888 Y69.351 E0.11400

G1 X85.480 Y69.351 E0.01546

G1 X88.568 Y66.264 E0.11400

G1 X89.160 Y66.264 E0.01546

G1 X86.072 Y69.351 E0.11400

G1 X86.664 Y69.351 E0.01546

G1 X89.752 Y66.264 E0.11400

G1 X90.095 Y66.264 E0.00895

G1 X90.095 Y66.513 E0.00650

G1 X87.256 Y69.351 E0.10481

G1 X87.848 Y69.351 E0.01546

G1 X90.095 Y67.105 E0.08295

G1 X90.095 Y67.697 E0.01546

G1 X88.441 Y69.351 E0.06109

```
G1 X89.033 Y69.351 E0.01546
G1 X90.095 Y68.289 E0.03923
G1 X90.095 Y68.881 E0.01546
G1 X89.437 Y69.539 E0.02431
M204 S1250
; stop printing object tpu print.STL id:5 copy 0
; printing object tpu print.STL id:9 copy 0
G1 E-2.24000 F2400.000
;WIPE_START
G1 F7200.000
G1 X90.095 Y68.881 E-0.29484
G1 X90.095 Y68.289 E-0.18749
G1 X89.136 Y69.249 E-0.42968
;WIPE_END
G1 E-0.04800 F2400.000
G1 Z0.850 F9000.000
G1 X76.558 Y54.716
G1 Z0.650
G1 E3.20000 F1500.000
M204 S800
;TYPE:Perimeter
;WIDTH:0.45
G1 F2400.000
G1 X69.686 Y54.716 E0.17903
G1 X69.686 Y41.002 E0.35734
G1 X76.558 Y41.002 E0.17903
G1 X76.649 Y41.325 E0.00876
G1 X76.984 Y42.119 E0.02246
G1 X77.414 Y42.866 E0.02246
```

G1 X77.933 Y43.555 E0.02246

G1 X78.533 Y44.174 E0.02246

G1 X79.204 Y44.715 E0.02246

G1 X79.937 Y45.169 E0.02246

G1 X80.720 Y45.530 E0.02246

G1 X81.541 Y45.791 E0.02246

G1 X82.388 Y45.950 E0.02246

G1 X83.229 Y46.002 E0.02194

G1 X90.401 Y46.002 E0.18688

G1 X90.401 Y49.716 E0.09678

G1 X83.229 Y49.716 E0.18688

G1 X82.388 Y49.768 E0.02194

G1 X81.541 Y49.926 E0.02246

G1 X80.720 Y50.188 E0.02246

G1 X79.937 Y50.549 E0.02246

G1 X79.204 Y51.003 E0.02246

G1 X78.533 Y51.544 E0.02246

G1 X77.933 Y52.163 E0.02246

G1 X77.414 Y52.851 E0.02246

G1 X76.984 Y53.598 E0.02246

G1 X76.649 Y54.392 E0.02246

G1 X76.574 Y54.658 E0.00720

M204 S1250

G1 X76.874 Y55.134 F9000.000

M204 S800

;TYPE:External perimeter

G1 F1800.000

G1 X69.269 Y55.134 E0.19815

G1 X69.269 Y40.584 E0.37911

```
G1 X76.874 Y40.584 E0.19815
G1 X77.044 Y41.186 E0.01632
G1 X77.359 Y41.933 E0.02112
G1 X77.764 Y42.635 E0.02112
G1 X78.251 Y43.282 E0.02112
G1 X78.815 Y43.865 E0.02112
G1 X79.446 Y44.373 E0.02112
G1 X80.135 Y44.800 E0.02112
G1 X80.871 Y45.139 E0.02112
G1 X81.643 Y45.385 E0.02112
G1 X82.440 Y45.534 E0.02112
G1 X83.242 Y45.584 E0.02093
G1 X90.819 Y45.584 E0.19743
G1 X90.819 Y50.134 E0.11855
G1 X83.242 Y50.134 E0.19743
G1 X82.440 Y50.183 E0.02093
G1 X81.643 Y50.332 E0.02112
G1 X80.871 Y50.578 E0.02112
G1 X80.135 Y50.917 E0.02112
G1 X79.446 Y51.344 E0.02112
G1 X78.815 Y51.853 E0.02112
G1 X78.251 Y52.435 E0.02112
G1 X77.764 Y53.082 E0.02112
G1 X77.359 Y53.784 E0.02112
G1 X77.044 Y54.531 E0.02112
G1 X76.890 Y55.076 E0.01476
M204 S1250
G1 X76.552 Y54.895 F9000.000
G1 E-2.24000 F2400.000
```

```
;WIPE_START
G1 F7200.000
G1 X74.010 Y55.098 E-0.91200
;WIPE_END
G1 E-0.04800 F2400.000
G1 Z0.850 F9000.000
G1 X70.658 Y41.127
G1 Z0.650
G1 E3.20000 F1500.000
M204 S1000
;TYPE:Solid infill
;WIDTH:0.450839
G1 F4800.000
G1 X70.000 Y41.785 E0.02431
G1 X70.000 Y42.377 E0.01546
G1 X71.062 Y41.315 E0.03922
G1 X71.654 Y41.315 E0.01546
G1 X70.000 Y42.969 E0.06108
G1 X70.000 Y43.561 E0.01546
G1 X72.246 Y41.315 E0.08294
G1 X72.838 Y41.315 E0.01546
G1 X70.000 Y44.153 E0.10480
G1 X70.000 Y44.745 E0.01546
G1 X73.430 Y41.315 E0.12666
G1 X74.022 Y41.315 E0.01546
G1 X70.000 Y45.337 E0.14853
G1 X70.000 Y45.930 E0.01546
G1 X74.615 Y41.315 E0.17039
G1 X75.207 Y41.315 E0.01546
```

```
G1 X70.000 Y46.522 E0.19225
G1 X70.000 Y47.114 E0.01546
G1 X75.799 Y41.315 E0.21411
G1 X76.321 Y41.315 E0.01363
G1 X76.336 Y41.369 E0.00148
G1 X70.000 Y47.706 E0.23395
G1 X70.000 Y48.298 E0.01546
G1 X76.506 Y41.792 E0.24022
G1 X76.682 Y42.208 E0.01180
G1 X70.000 Y48.890 E0.24671
G1 X70.000 Y49.482 E0.01546
G1 X76.893 Y42.589 E0.25451
G1 X77.109 Y42.964 E0.01132
G1 X70.000 Y50.074 E0.26250
G1 X70.000 Y50.666 E0.01546
G1 X77.356 Y43.310 E0.27162
G1 X77.611 Y43.647 E0.01104
G1 X70.000 Y51.258 E0.28101
G1 X70.000 Y51.850 E0.01546
G1 X77.890 Y43.960 E0.29132
M73 P58 R29
G1 X78.181 Y44.261 E0.01093
G1 X70.000 Y52.442 E0.30207
G1 X70.000 Y53.034 E0.01546
G1 X78.491 Y44.543 E0.31352
G1 X78.819 Y44.807 E0.01099
G1 X70.000 Y53.626 E0.32563
G1 X70.000 Y54.218 E0.01546
G1 X79.161 Y45.057 E0.33826
```

```
G1 X79.527 Y45.283 E0.01123
G1 X70.408 Y54.403 E0.33670
G1 X71.000 Y54.403 E0.01546
G1 X79.904 Y45.499 E0.32875
G1 X80.309 Y45.685 E0.01165
G1 X71.592 Y54.403 E0.32186
G1 X72.184 Y54.403 E0.01546
G1 X80.726 Y45.860 E0.31540
G1 X81.175 Y46.003 E0.01230
G1 X72.776 Y54.403 E0.31012
G1 X73.368 Y54.403 E0.01546
G1 X81.642 Y46.129 E0.30549
G1 X82.141 Y46.222 E0.01325
G1 X73.960 Y54.403 E0.30204
G1 X74.552 Y54.403 E0.01546
G1 X82.673 Y46.281 E0.29986
G1 X83.232 Y46.315 E0.01460
G1 X75.144 Y54.403 E0.29862
G1 X75.736 Y54.403 E0.01546
G1 X76.734 Y53.405 E0.03684
G1 X77.153 Y52.678 E0.02190
G1 X77.695 Y51.959 E0.02352
G1 X78.321 Y51.312 E0.02351
G1 X79.022 Y50.747 E0.02351
G1 X79.788 Y50.272 E0.02352
G1 X79.934 Y50.205 E0.00420
G1 X83.824 Y46.315 E0.14362
G1 X84.416 Y46.315 E0.01546
G1 X80.943 Y49.788 E0.12823
```

```
G1 X81.464 Y49.622 E0.01428
G1 X81.756 Y49.567 E0.00774
G1 X85.008 Y46.315 E0.12009
G1 X85.600 Y46.315 E0.01546
G1 X82.466 Y49.449 E0.11572
G1 X83.097 Y49.410 E0.01651
G1 X86.192 Y46.315 E0.11428
G1 X86.784 Y46.315 E0.01546
G1 X83.697 Y49.403 E0.11400
G1 X84.289 Y49.403 E0.01546
G1 X87.376 Y46.315 E0.11400
G1 X87.968 Y46.315 E0.01546
G1 X84.881 Y49.403 E0.11400
G1 X85.473 Y49.403 E0.01546
G1 X88.560 Y46.315 E0.11400
G1 X89.152 Y46.315 E0.01546
G1 X86.065 Y49.403 E0.11400
G1 X86.657 Y49.403 E0.01546
G1 X89.745 Y46.315 E0.11400
G1 X90.087 Y46.315 E0.00895
G1 X90.087 Y46.564 E0.00650
G1 X87.249 Y49.403 E0.10481
G1 X87.841 Y49.403 E0.01546
G1 X90.087 Y47.156 E0.08295
G1 X90.087 Y47.748 E0.01546
G1 X88.433 Y49.403 E0.06109
G1 X89.025 Y49.403 E0.01546
G1 X90.087 Y48.340 E0.03923
G1 X90.087 Y48.932 E0.01546
```

```
G1 X89.429 Y49.591 E0.02431
M204 S1250
; stop printing object tpu print.STL id:9 copy 0
; printing object Petg print.STL id:8 copy 0
; stop printing object Petg print.STL id:8 copy 0
; printing object Petg print.STL id:4 copy 0
; stop printing object Petg print.STL id:4 copy 0
; printing object tpu print.STL id:29 copy 0
G1 E-2.24000 F2400.000
;WIPE_START
G1 F7200.000
G1 X90.087 Y48.932 E-0.29484
G1 X90.087 Y48.340 E-0.18749
G1 X89.128 Y49.300 E-0.42968
;WIPE_END
G1 E-0.04800 F2400.000
G1 Z0.850 F9000.000
G1 X120.337 Y54.891
G1 Z0.650
G1 E3.20000 F1500.000
M204 S800
;TYPE:Perimeter
;WIDTH:0.45
G1 F2400.000
G1 X120.337 Y41.177 E0.35734
G1 X127.208 Y41.177 E0.17903
G1 X127.299 Y41.500 E0.00876
G1 X127.634 Y42.294 E0.02246
G1 X128.065 Y43.041 E0.02246
```

```
G1 X128.583 Y43.730 E0.02246
G1 X129.183 Y44.349 E0.02246
G1 X129.854 Y44.890 E0.02246
G1 X130.587 Y45.344 E0.02246
G1 X131.370 Y45.705 E0.02246
G1 X132.191 Y45.966 E0.02246
G1 X133.038 Y46.125 E0.02246
G1 X133.879 Y46.177 E0.02194
G1 X141.051 Y46.177 E0.18688
G1 X141.051 Y49.891 E0.09678
G1 X133.879 Y49.891 E0.18688
G1 X133.038 Y49.943 E0.02194
G1 X132.191 Y50.101 E0.02246
G1 X131.370 Y50.363 E0.02246
G1 X130.587 Y50.724 E0.02246
G1 X129.854 Y51.178 E0.02246
G1 X129.183 Y51.719 E0.02246
G1 X128.583 Y52.338 E0.02246
G1 X128.065 Y53.026 E0.02246
G1 X127.634 Y53.773 E0.02246
G1 X127.299 Y54.567 E0.02246
G1 X127.208 Y54.891 E0.00876
G1 X120.397 Y54.891 E0.17747
M204 S1250
G1 X119.919 Y55.309 F9000.000
M204 S800
;TYPE:External perimeter
G1 F1800.000
G1 X119.919 Y40.759 E0.37911
```

```
G1 X127.524 Y40.759 E0.19815
G1 X127.694 Y41.361 E0.01632
G1 X128.009 Y42.108 E0.02112
G1 X128.414 Y42.810 E0.02112
G1 X128.902 Y43.458 E0.02112
G1 X129.465 Y44.040 E0.02112
G1 X130.096 Y44.548 E0.02112
G1 X130.785 Y44.975 E0.02112
G1 X131.521 Y45.314 E0.02112
G1 X132.293 Y45.560 E0.02112
G1 X133.090 Y45.709 E0.02112
G1 X133.892 Y45.759 E0.02093
G1 X141.469 Y45.759 E0.19743
G1 X141.469 Y50.309 E0.11855
M73 P58 R28
G1 X133.892 Y50.309 E0.19743
G1 X133.090 Y50.358 E0.02093
G1 X132.293 Y50.507 E0.02112
G1 X131.521 Y50.753 E0.02112
G1 X130.785 Y51.092 E0.02112
G1 X130.096 Y51.519 E0.02112
G1 X129.465 Y52.028 E0.02112
G1 X128.902 Y52.610 E0.02112
G1 X128.414 Y53.257 E0.02112
G1 X128.009 Y53.959 E0.02112
G1 X127.694 Y54.706 E0.02112
G1 X127.524 Y55.309 E0.01632
G1 X119.979 Y55.309 E0.19659
M204 S1250
```

```
G1 X120.110 Y54.957 F9000.000
G1 E-2.24000 F2400.000
;WIPE_START
G1 F7200.000
G1 X119.967 Y52.429 E-0.91200
;WIPE_END
G1 E-0.04800 F2400.000
G1 Z0.850 F9000.000
G1 X140.079 Y49.766
G1 Z0.650
G1 E3.20000 F1500.000
M204 S1000
;TYPE:Solid infill
;WIDTH:0.450839
G1 F4800.000
G1 X140.738 Y49.107 E0.02431
G1 X140.738 Y48.515 E0.01546
G1 X139.675 Y49.578 E0.03923
G1 X139.083 Y49.578 E0.01546
G1 X140.738 Y47.923 E0.06109
G1 X140.738 Y47.331 E0.01546
G1 X138.491 Y49.578 E0.08295
G1 X137.899 Y49.578 E0.01546
G1 X140.738 Y46.739 E0.10481
G1 X140.738 Y46.490 E0.00650
G1 X140.395 Y46.490 E0.00895
G1 X137.307 Y49.578 E0.11400
G1 X136.715 Y49.578 E0.01546
G1 X139.803 Y46.490 E0.11400
```

```
G1 X139.211 Y46.490 E0.01546
G1 X136.123 Y49.578 E0.11400
G1 X135.531 Y49.578 E0.01546
G1 X138.619 Y46.490 E0.11400
G1 X138.027 Y46.490 E0.01546
G1 X134.939 Y49.578 E0.11400
G1 X134.347 Y49.578 E0.01546
G1 X137.434 Y46.490 E0.11400
G1 X136.842 Y46.490 E0.01546
G1 X133.747 Y49.585 E0.11428
G1 X133.116 Y49.624 E0.01651
G1 X136.250 Y46.490 E0.11572
G1 X135.658 Y46.490 E0.01546
G1 X132.406 Y49.742 E0.12009
G1 X132.114 Y49.797 E0.00774
G1 X131.593 Y49.963 E0.01428
G1 X135.066 Y46.490 E0.12823
G1 X134.474 Y46.490 E0.01546
G1 X130.584 Y50.380 E0.14362
G1 X130.438 Y50.447 E0.00420
G1 X129.673 Y50.922 E0.02352
G1 X128.971 Y51.487 E0.02351
G1 X128.345 Y52.134 E0.02351
G1 X127.803 Y52.853 E0.02352
G1 X127.384 Y53.580 E0.02190
G1 X126.386 Y54.578 E0.03684
G1 X125.794 Y54.578 E0.01546
G1 X133.882 Y46.490 E0.29862
G1 X133.324 Y46.456 E0.01460
```

```
G1 X125.202 Y54.578 E0.29986
G1 X124.610 Y54.578 E0.01546
G1 X132.791 Y46.397 E0.30204
G1 X132.292 Y46.304 E0.01325
G1 X124.018 Y54.578 E0.30549
G1 X123.426 Y54.578 E0.01546
G1 X131.825 Y46.179 E0.31012
G1 X131.376 Y46.036 E0.01230
G1 X122.834 Y54.578 E0.31540
G1 X122.242 Y54.578 E0.01546
G1 X130.959 Y45.860 E0.32186
G1 X130.554 Y45.674 E0.01165
G1 X121.650 Y54.578 E0.32875
G1 X121.058 Y54.578 E0.01546
G1 X130.177 Y45.459 E0.33670
G1 X129.812 Y45.232 E0.01123
G1 X120.650 Y54.393 E0.33826
G1 X120.650 Y53.801 E0.01546
G1 X129.469 Y44.982 E0.32563
G1 X129.142 Y44.718 E0.01099
G1 X120.650 Y53.209 E0.31352
G1 X120.650 Y52.617 E0.01546
G1 X128.831 Y44.436 E0.30207
G1 X128.540 Y44.135 E0.01093
G1 X120.650 Y52.025 E0.29132
G1 X120.650 Y51.433 E0.01546
G1 X128.261 Y43.822 E0.28101
G1 X128.006 Y43.485 E0.01104
G1 X120.650 Y50.841 E0.27162
```

G1 X120.650 Y50.249 E0.01546

G1 X127.760 Y43.139 E0.26250

G1 X127.543 Y42.764 E0.01132

G1 X120.650 Y49.657 E0.25451

G1 X120.650 Y49.065 E0.01546

G1 X127.332 Y42.383 E0.24671

G1 X127.156 Y41.967 E0.01180

G1 X120.650 Y48.473 E0.24022

G1 X120.650 Y47.881 E0.01546

G1 X126.986 Y41.545 E0.23395

G1 X126.971 Y41.490 E0.00148

G1 X126.449 Y41.490 E0.01363

G1 X120.650 Y47.289 E0.21411

G1 X120.650 Y46.697 E0.01546

G1 X125.857 Y41.490 E0.19225

G1 X125.265 Y41.490 E0.01546

G1 X120.650 Y46.105 E0.17039

G1 X120.650 Y45.513 E0.01546

G1 X124.673 Y41.490 E0.14853

G1 X124.081 Y41.490 E0.01546

G1 X120.650 Y44.921 E0.12666

G1 X120.650 Y44.328 E0.01546

G1 X123.489 Y41.490 E0.10480

G1 X122.897 Y41.490 E0.01546

G1 X120.650 Y43.736 E0.08294

G1 X120.650 Y43.144 E0.01546

G1 X122.304 Y41.490 E0.06108

G1 X121.712 Y41.490 E0.01546

G1 X120.650 Y42.552 E0.03922

```
G1 X120.650 Y41.960 E0.01546
G1 X121.308 Y41.302 E0.02431
M204 S1250
; stop printing object tpu print.STL id:29 copy 0
; printing object tpu print.STL id:25 copy 0
G1 E-2.24000 F2400.000
;WIPE_START
G1 F7200.000
G1 X120.650 Y41.960 E-0.29480
G1 X120.650 Y42.552 E-0.18749
G1 X121.610 Y41.593 E-0.42971
;WIPE_END
G1 E-0.04800 F2400.000
G1 Z0.850 F9000.000
G1 X120.344 Y61.126
G1 Z0.650
G1 E3.20000 F1500.000
M204 S800
;TYPE:Perimeter
;WIDTH:0.45
G1 F2400.000
G1 X127.215 Y61.126 E0.17903
G1 X127.307 Y61.449 E0.00876
G1 X127.642 Y62.243 E0.02246
G1 X128.072 Y62.990 E0.02246
G1 X128.591 Y63.679 E0.02246
G1 X129.191 Y64.298 E0.02246
G1 X129.862 Y64.839 E0.02246
G1 X130.594 Y65.293 E0.02246
```

```
G1 X131.377 Y65.654 E0.02246
G1 X132.199 Y65.915 E0.02246
G1 X133.046 Y66.074 E0.02246
G1 X133.886 Y66.126 E0.02194
G1 X141.059 Y66.126 E0.18688
G1 X141.059 Y69.840 E0.09678
G1 X133.886 Y69.840 E0.18688
G1 X133.046 Y69.892 E0.02194
G1 X132.199 Y70.050 E0.02246
G1 X131.377 Y70.312 E0.02246
G1 X130.594 Y70.673 E0.02246
G1 X129.862 Y71.127 E0.02246
G1 X129.191 Y71.668 E0.02246
G1 X128.591 Y72.287 E0.02246
G1 X128.072 Y72.975 E0.02246
G1 X127.642 Y73.722 E0.02246
G1 X127.307 Y74.516 E0.02246
G1 X127.215 Y74.840 E0.00876
G1 X120.344 Y74.840 E0.17903
G1 X120.344 Y61.186 E0.35578
M204 S1250
G1 X119.926 Y60.708 F9000.000
M204 S800
;TYPE:External perimeter
G1 F1800.000
G1 X127.531 Y60.708 E0.19815
G1 X127.702 Y61.310 E0.01632
G1 X128.017 Y62.057 E0.02112
G1 X128.421 Y62.759 E0.02112
```

```
G1 X128.909 Y63.406 E0.02112
G1 X129.473 Y63.989 E0.02112
G1 X130.104 Y64.497 E0.02112
G1 X130.793 Y64.924 E0.02112
G1 X131.529 Y65.263 E0.02112
G1 X132.301 Y65.509 E0.02112
G1 X133.097 Y65.658 E0.02112
G1 X133.899 Y65.708 E0.02093
G1 X141.476 Y65.708 E0.19743
G1 X141.476 Y70.258 E0.11855
G1 X133.899 Y70.258 E0.19743
G1 X133.097 Y70.307 E0.02093
G1 X132.301 Y70.456 E0.02112
G1 X131.529 Y70.702 E0.02112
G1 X130.793 Y71.041 E0.02112
G1 X130.104 Y71.468 E0.02112
G1 X129.473 Y71.977 E0.02112
G1 X128.909 Y72.559 E0.02112
G1 X128.421 Y73.206 E0.02112
G1 X128.017 Y73.908 E0.02112
G1 X127.702 Y74.655 E0.02112
G1 X127.531 Y75.258 E0.01632
G1 X119.926 Y75.258 E0.19815
G1 X119.926 Y60.768 E0.37755
M204 S1250
G1 X120.300 Y60.850 F9000.000
G1 E-2.24000 F2400.000
;WIPE_START
G1 F7200.000
```

```
G1 X122.806 Y60.745 E-0.91200
;WIPE_END
G1 E-0.04800 F2400.000
G1 Z0.850 F9000.000
G1 X140.087 Y69.715
G1 Z0.650
G1 E3.20000 F1500.000
M204 S1000
;TYPE:Solid infill
;WIDTH:0.450839
G1 F4800.000
G1 X140.745 Y69.056 E0.02431
G1 X140.745 Y68.464 E0.01546
G1 X139.683 Y69.527 E0.03923
G1 X139.091 Y69.527 E0.01546
G1 X140.745 Y67.872 E0.06109
G1 X140.745 Y67.280 E0.01546
G1 X138.499 Y69.527 E0.08295
G1 X137.907 Y69.527 E0.01546
G1 X140.745 Y66.688 E0.10481
G1 X140.745 Y66.439 E0.00650
G1 X140.402 Y66.439 E0.00895
G1 X137.315 Y69.527 E0.11400
G1 X136.723 Y69.527 E0.01546
G1 X139.810 Y66.439 E0.11400
G1 X139.218 Y66.439 E0.01546
G1 X136.130 Y69.527 E0.11400
G1 X135.538 Y69.527 E0.01546
G1 X138.626 Y66.439 E0.11400
```

G1 X138.034 Y66.439 E0.01546

G1 X134.946 Y69.527 E0.11400

G1 X134.354 Y69.527 E0.01546

G1 X137.442 Y66.439 E0.11400

G1 X136.850 Y66.439 E0.01546

G1 X133.755 Y69.534 E0.11428

G1 X133.124 Y69.573 E0.01651

G1 X136.258 Y66.439 E0.11572

G1 X135.666 Y66.439 E0.01546

G1 X132.413 Y69.691 E0.12009

G1 X132.122 Y69.746 E0.00774

G1 X131.601 Y69.912 E0.01428

G1 X135.074 Y66.439 E0.12823

G1 X134.482 Y66.439 E0.01546

G1 X130.592 Y70.329 E0.14362

G1 X130.446 Y70.396 E0.00420

G1 X129.680 Y70.871 E0.02352

G1 X128.979 Y71.436 E0.02351

G1 X128.352 Y72.083 E0.02351

G1 X127.810 Y72.802 E0.02352

G1 X127.392 Y73.529 E0.02190

G1 X126.394 Y74.527 E0.03684

G1 X125.802 Y74.527 E0.01546

G1 X133.890 Y66.439 E0.29862

G1 X133.331 Y66.405 E0.01460

G1 X125.210 Y74.527 E0.29986

G1 X124.618 Y74.527 E0.01546

G1 X132.798 Y66.346 E0.30204

G1 X132.300 Y66.253 E0.01325

```
G1 X124.026 Y74.527 E0.30549
G1 X123.434 Y74.527 E0.01546
G1 X131.833 Y66.127 E0.31012
G1 X131.384 Y65.984 E0.01230
G1 X122.842 Y74.527 E0.31540
G1 X122.250 Y74.527 E0.01546
G1 X130.967 Y65.809 E0.32186
G1 X130.561 Y65.623 E0.01165
G1 X121.658 Y74.527 E0.32875
G1 X121.065 Y74.527 E0.01546
G1 X130.185 Y65.408 E0.33670
G1 X129.819 Y65.181 E0.01123
G1 X120.658 Y74.342 E0.33826
G1 X120.658 Y73.750 E0.01546
G1 X129.477 Y64.931 E0.32563
G1 X129.149 Y64.667 E0.01099
G1 X120.658 Y73.158 E0.31352
G1 X120.658 Y72.566 E0.01546
G1 X128.839 Y64.385 E0.30207
G1 X128.548 Y64.084 E0.01093
G1 X120.658 Y71.974 E0.29132
G1 X120.658 Y71.382 E0.01546
G1 X128.268 Y63.771 E0.28101
G1 X128.014 Y63.434 E0.01104
G1 X120.658 Y70.790 E0.27162
G1 X120.658 Y70.198 E0.01546
G1 X127.767 Y63.088 E0.26250
G1 X127.551 Y62.713 E0.01132
G1 X120.658 Y69.606 E0.25451
```

G1 X120.658 Y69.014 E0.01546

G1 X127.339 Y62.332 E0.24671

G1 X127.164 Y61.916 E0.01180

G1 X120.658 Y68.422 E0.24022

G1 X120.658 Y67.830 E0.01546

G1 X126.994 Y61.493 E0.23395

G1 X126.978 Y61.439 E0.00148

G1 X126.456 Y61.439 E0.01363

G1 X120.658 Y67.238 E0.21411

G1 X120.658 Y66.646 E0.01546

G1 X125.864 Y61.439 E0.19225

G1 X125.272 Y61.439 E0.01546

G1 X120.658 Y66.054 E0.17039

G1 X120.658 Y65.462 E0.01546

G1 X124.680 Y61.439 E0.14853

G1 X124.088 Y61.439 E0.01546

G1 X120.658 Y64.869 E0.12666

G1 X120.658 Y64.277 E0.01546

G1 X123.496 Y61.439 E0.10480

G1 X122.904 Y61.439 E0.01546

G1 X120.658 Y63.685 E0.08294

G1 X120.658 Y63.093 E0.01546

G1 X122.312 Y61.439 E0.06108

G1 X121.720 Y61.439 E0.01546

G1 X120.658 Y62.501 E0.03922

G1 X120.658 Y61.909 E0.01546

G1 X121.316 Y61.251 E0.02431

M204 S1250

; stop printing object tpu print.STL id:25 copy 0

```
; printing object Petg print.STL id:24 copy 0
; stop printing object Petg print.STL id:24 copy 0
; printing object Petg print.STL id:28 copy 0
; stop printing object Petg print.STL id:28 copy 0
G1 E-2.80000 F2400.000
;WIPE_START
G1 F7200.000
G1 X120.658 Y61.909 E-0.29480
G1 X120.658 Y62.501 E-0.18749
G1 X121.720 Y61.439 E-0.47575
G1 X122.295 Y61.439 E-0.18197
;WIPE_END
G1 E-0.06000 F2400.000
G1 Z0.850 F9000.000
; Filament-specific end gcode
M600
M107
T0
M900 K0 ; Filament gcode
; printing object tpu print.STL id:19 copy 0
; stop printing object tpu print.STL id:19 copy 0
; printing object Petg print.STL id:18 copy 0
G1 X61.611 Y54.135
G1 Z0.650
G1 E4.00000 F900.000
M204 S800
;TYPE:Perimeter
;WIDTH:0.45
G1 F1292.454
```

G1 X54.740 Y54.135 E0.20589

G1 X54.649 Y53.812 E0.01007

G1 X54.314 Y53.017 E0.02583

G1 X53.884 Y52.270 E0.02583

G1 X53.365 Y51.582 E0.02583

G1 X52.765 Y50.963 E0.02583

G1 X52.094 Y50.422 E0.02583

G1 X51.361 Y49.968 E0.02583

G1 X50.578 Y49.607 E0.02583

G1 X49.757 Y49.345 E0.02583

G1 X48.910 Y49.187 E0.02583

G1 X48.069 Y49.135 E0.02523

G1 X39.897 Y49.135 E0.24487

G1 X39.897 Y45.421 E0.11130

G1 X48.069 Y45.421 E0.24487

G1 X48.910 Y45.369 E0.02523

G1 X49.757 Y45.210 E0.02583

G1 X50.578 Y44.949 E0.02583

G1 X51.361 Y44.588 E0.02583

G1 X52.094 Y44.134 E0.02583

G1 X52.765 Y43.593 E0.02583

G1 X53.365 Y42.974 E0.02583

G1 X53.884 Y42.285 E0.02583

G1 X54.314 Y41.538 E0.02583

G1 X54.649 Y40.744 E0.02583

G1 X54.740 Y40.421 E0.01007

G1 X61.611 Y40.421 E0.20589

G1 X61.611 Y54.075 E0.40914

M204 S1250

```
G1 X62.029 Y54.553 F9000.000
M204 S800
;TYPE:External perimeter
G1 F1292.454
G1 X54.424 Y54.553 E0.22787
G1 X54.254 Y53.950 E0.01877
G1 X53.939 Y53.203 E0.02428
G1 X53.534 Y52.501 E0.02428
G1 X53.047 Y51.854 E0.02428
G1 X52.483 Y51.272 E0.02428
G1 X51.852 Y50.763 E0.02428
G1 X51.163 Y50.336 E0.02428
G1 X50.427 Y49.997 E0.02428
G1 X49.655 Y49.751 E0.02428
G1 X48.858 Y49.602 E0.02428
G1 X48.056 Y49.553 E0.02407
G1 X39.479 Y49.553 E0.25701
G1 X39.479 Y45.003 E0.13634
G1 X48.056 Y45.003 E0.25701
G1 X48.858 Y44.953 E0.02407
G1 X49.655 Y44.804 E0.02428
G1 X50.427 Y44.558 E0.02428
G1 X51.163 Y44.219 E0.02428
G1 X51.852 Y43.792 E0.02428
G1 X52.483 Y43.284 E0.02428
G1 X53.047 Y42.702 E0.02428
G1 X53.534 Y42.054 E0.02428
G1 X53.939 Y41.352 E0.02428
G1 X54.254 Y40.606 E0.02428
```

```
G1 X54.424 Y40.003 E0.01877
G1 X62.029 Y40.003 E0.22787
G1 X62.029 Y54.493 E0.43418
M204 S1250
M73 P59 R28
G1 X61.655 Y54.411 F9000.000
G1 X60.628 Y54.010
M204 S1000
;TYPE:Solid infill
;WIDTH:0.456608
G1 F1272.331
G1 X61.298 Y53.339 E0.02887
G1 X61.298 Y52.739 E0.01827
G1 X60.215 Y53.822 E0.04661
G1 X59.615 Y53.822 E0.01827
G1 X61.298 Y52.139 E0.07245
G1 X61.298 Y51.538 E0.01827
G1 X59.015 Y53.822 E0.09828
G1 X58.415 Y53.822 E0.01827
G1 X61.298 Y50.938 E0.12412
G1 X61.298 Y50.338 E0.01827
G1 X57.814 Y53.822 E0.14996
G1 X57.214 Y53.822 E0.01827
G1 X61.298 Y49.738 E0.17580
G1 X61.298 Y49.138 E0.01827
G1 X56.614 Y53.822 E0.20163
G1 X56.014 Y53.822 E0.01827
G1 X61.298 Y48.537 E0.22747
G1 X61.298 Y47.937 E0.01827
```

```
G1 X55.414 Y53.822 E0.25331

G1 X54.977 Y53.822 E0.01328

G1 X54.940 Y53.695 E0.00402

G1 X61.298 Y47.337 E0.27370

G1 X61.298 Y46.737 E0.01827

G1 X54.762 Y53.273 E0.28136

G1 X54.581 Y52.854 E0.01390

G1 X61.298 Y46.136 E0.28914

G1 X61.298 Y45.536 E0.01827

G1 X54.362 Y52.473 E0.29859

G1 X54.142 Y52.092 E0.01337

G1 X61.298 Y44.936 E0.30805

G1 X61.298 Y44.336 E0.01827

G1 X53.884 Y51.750 E0.31916

G1 X53.626 Y51.408 E0.01305

G1 X61.298 Y43.736 E0.33026

G1 X61.298 Y43.135 E0.01827

G1 X53.334 Y51.100 E0.34283

G1 X53.039 Y50.795 E0.01292

G1 X61.298 Y42.535 E0.35554

G1 X61.298 Y41.935 E0.01827

G1 X52.714 Y50.519 E0.36951

G1 X52.382 Y50.251 E0.01299

G1 X61.298 Y41.335 E0.38382

G1 X61.298 Y40.735 E0.01827

G1 X52.023 Y50.009 E0.39924

G1 X51.653 Y49.780 E0.01327

G1 X60.698 Y40.734 E0.38938

G1 X60.098 Y40.734 E0.01827
```

```
G1 X51.257 Y49.575 E0.38056
G1 X50.846 Y49.385 E0.01377
G1 X59.498 Y40.734 E0.37241
G1 X58.898 Y40.734 E0.01827
G1 X50.408 Y49.224 E0.36545
G1 X49.953 Y49.079 E0.01454
G1 X58.298 Y40.734 E0.35921
G1 X57.697 Y40.734 E0.01827
G1 X49.460 Y48.971 E0.35457
G1 X48.955 Y48.877 E0.01566
G1 X57.097 Y40.734 E0.35050
G1 X56.497 Y40.734 E0.01827
G1 X48.390 Y48.841 E0.34897
G1 X47.809 Y48.822 E0.01769
G1 X55.897 Y40.734 E0.34814
G1 X55.296 Y40.734 E0.01827
G1 X54.413 Y41.618 E0.03805
M204 S1250
G1 E-4.00000 F2400.000
G1 X47.021 Y49.010 F9000.000
G1 E4.00000 F900.000
M204 S1000
G1 F1272.331
G1 X50.872 Y45.158 E0.16578
G1 X50.692 Y45.241 E0.00604
G1 X49.954 Y45.476 E0.02358
G1 X46.609 Y48.822 E0.14400
G1 X46.008 Y48.822 E0.01827
G1 X49.196 Y45.634 E0.13722
```

G1 X48.948 Y45.680 E0.00767

G1 X48.523 Y45.707 E0.01297

G1 X45.408 Y48.822 E0.13409

G1 X44.808 Y48.822 E0.01827

G1 X47.896 Y45.734 E0.13291

G1 X47.295 Y45.734 E0.01827

G1 X44.208 Y48.822 E0.13291

G1 X43.607 Y48.822 E0.01827

G1 X46.695 Y45.734 E0.13291

G1 X46.095 Y45.734 E0.01827

G1 X43.007 Y48.822 E0.13291

G1 X42.407 Y48.822 E0.01827

G1 X45.495 Y45.734 E0.13291

G1 X44.894 Y45.734 E0.01827

G1 X41.807 Y48.822 E0.13291

G1 X41.207 Y48.822 E0.01827

G1 X44.294 Y45.734 E0.13291

G1 X43.694 Y45.734 E0.01827

G1 X40.606 Y48.822 E0.13291

G1 X40.210 Y48.822 E0.01205

G1 X40.210 Y48.617 E0.00622

G1 X43.094 Y45.734 E0.12412

G1 X42.494 Y45.734 E0.01827

G1 X40.210 Y48.017 E0.09828

G1 X40.210 Y47.417 E0.01827

G1 X41.893 Y45.734 E0.07244

G1 X41.293 Y45.734 E0.01827

G1 X40.210 Y46.817 E0.04661

G1 X40.210 Y46.217 E0.01827

```
G1 X40.881 Y45.546 E0.02886
M204 S1250
; stop printing object Petg print.STL id:18 copy 0
; printing object Petg print.STL id:14 copy 0
G1 E-4.00000 F2400.000
G1 X39.905 Y65.370 F9000.000
G1 E4.00000 F900.000
M204 S800
;TYPE:Perimeter
;WIDTH:0.45
G1 F1292.454
G1 X48.077 Y65.370 E0.24487
G1 X48.917 Y65.318 E0.02523
G1 X49.765 Y65.159 E0.02583
G1 X50.586 Y64.898 E0.02583
G1 X51.369 Y64.537 E0.02583
G1 X52.101 Y64.083 E0.02583
G1 X52.773 Y63.542 E0.02583
G1 X53.372 Y62.923 E0.02583
G1 X53.891 Y62.234 E0.02583
G1 X54.321 Y61.487 E0.02583
G1 X54.656 Y60.693 E0.02583
G1 X54.748 Y60.370 E0.01007
G1 X61.619 Y60.370 E0.20589
G1 X61.619 Y74.084 E0.41094
G1 X54.748 Y74.084 E0.20589
G1 X54.656 Y73.760 E0.01007
G1 X54.321 Y72.966 E0.02583
G1 X53.891 Y72.219 E0.02583
```

```
G1 X53.372 Y71.531 E0.02583
G1 X52.773 Y70.912 E0.02583
G1 X52.101 Y70.371 E0.02583
G1 X51.369 Y69.917 E0.02583
G1 X50.586 Y69.556 E0.02583
G1 X49.765 Y69.294 E0.02583
G1 X48.917 Y69.136 E0.02583
G1 X48.077 Y69.084 E0.02523
G1 X39.905 Y69.084 E0.24487
G1 X39.905 Y65.430 E0.10950
M204 S1250
G1 X39.487 Y64.952 F9000.000
M204 S800
;TYPE:External perimeter
G1 F1292.454
G1 X48.064 Y64.952 E0.25701
G1 X48.866 Y64.902 E0.02407
G1 X49.662 Y64.753 E0.02428
G1 X50.435 Y64.507 E0.02428
G1 X51.171 Y64.168 E0.02428
G1 X51.859 Y63.741 E0.02428
G1 X52.490 Y63.233 E0.02428
G1 X53.054 Y62.651 E0.02428
G1 X53.542 Y62.003 E0.02428
G1 X53.946 Y61.301 E0.02428
G1 X54.261 Y60.554 E0.02428
G1 X54.432 Y59.952 E0.01877
G1 X62.037 Y59.952 E0.22787
G1 X62.037 Y74.502 E0.43598
```

```
G1 X54.432 Y74.502 E0.22787
G1 X54.261 Y73.899 E0.01877
G1 X53.946 Y73.152 E0.02428
G1 X53.542 Y72.450 E0.02428
G1 X53.054 Y71.803 E0.02428
G1 X52.490 Y71.221 E0.02428
G1 X51.859 Y70.712 E0.02428
G1 X51.171 Y70.285 E0.02428
G1 X50.435 Y69.946 E0.02428
G1 X49.662 Y69.700 E0.02428
G1 X48.866 Y69.551 E0.02428
G1 X48.064 Y69.502 E0.02407
G1 X39.487 Y69.502 E0.25701
G1 X39.487 Y65.012 E0.13454
M204 S1250
G1 X39.882 Y65.017 F9000.000
G1 X40.888 Y65.495
M204 S1000
;TYPE:Solid infill
;WIDTH:0.456608
G1 F1272.331
G1 X40.218 Y66.165 E0.02886
G1 X40.218 Y66.766 E0.01827
G1 X41.301 Y65.683 E0.04661
G1 X41.901 Y65.683 E0.01827
G1 X40.218 Y67.366 E0.07244
G1 X40.218 Y67.966 E0.01827
G1 X42.501 Y65.683 E0.09828
G1 X43.101 Y65.683 E0.01827
```

```
G1 X40.218 Y68.566 E0.12412
G1 X40.218 Y68.771 E0.00622
G1 X40.614 Y68.771 E0.01205
G1 X43.702 Y65.683 E0.13291
G1 X44.302 Y65.683 E0.01827
G1 X41.214 Y68.771 E0.13291
G1 X41.814 Y68.771 E0.01827
G1 X44.902 Y65.683 E0.13291
G1 X45.502 Y65.683 E0.01827
G1 X42.415 Y68.771 E0.13291
G1 X43.015 Y68.771 E0.01827
G1 X46.102 Y65.683 E0.13291
G1 X46.703 Y65.683 E0.01827
G1 X43.615 Y68.771 E0.13291
G1 X44.215 Y68.771 E0.01827
G1 X47.303 Y65.683 E0.13291
M73 P59 R27
G1 X47.903 Y65.683 E0.01827
G1 X44.815 Y68.771 E0.13291
G1 X45.416 Y68.771 E0.01827
G1 X48.531 Y65.656 E0.13409
G1 X48.956 Y65.629 E0.01297
G1 X49.204 Y65.583 E0.00767
G1 X46.016 Y68.771 E0.13722
G1 X46.616 Y68.771 E0.01827
G1 X49.961 Y65.425 E0.14400
G1 X50.699 Y65.190 E0.02358
G1 X50.880 Y65.107 E0.00604
G1 X47.028 Y68.959 E0.16578
```

```
M204 S1250
G1 E-4.00000 F2400.000
G1 X54.420 Y61.567 F9000.000
G1 E4.00000 F900.000
M204 S1000
G1 F1272.331
G1 X55.304 Y60.683 E0.03805
G1 X55.904 Y60.683 E0.01827
G1 X47.816 Y68.771 E0.34814
G1 X48.398 Y68.790 E0.01769
G1 X56.504 Y60.683 E0.34897
G1 X57.105 Y60.683 E0.01827
G1 X48.962 Y68.825 E0.35050
G1 X49.468 Y68.920 E0.01566
G1 X57.705 Y60.683 E0.35457
G1 X58.305 Y60.683 E0.01827
G1 X49.960 Y69.028 E0.35921
G1 X50.415 Y69.173 E0.01454
G1 X58.905 Y60.683 E0.36545
G1 X59.505 Y60.683 E0.01827
G1 X50.854 Y69.334 E0.37241
G1 X51.265 Y69.524 E0.01377
G1 X60.106 Y60.683 E0.38056
G1 X60.706 Y60.683 E0.01827
G1 X51.660 Y69.729 E0.38938
G1 X52.031 Y69.958 E0.01327
G1 X61.306 Y60.683 E0.39924
G1 X61.306 Y61.284 E0.01827
G1 X52.389 Y70.200 E0.38382
```

G1 X52.722 Y70.468 E0.01299

G1 X61.306 Y61.884 E0.36951

G1 X61.306 Y62.484 E0.01827

G1 X53.046 Y70.744 E0.35554

G1 X53.341 Y71.049 E0.01292

G1 X61.306 Y63.084 E0.34283

G1 X61.306 Y63.685 E0.01827

G1 X53.633 Y71.357 E0.33026

G1 X53.891 Y71.699 E0.01305

G1 X61.306 Y64.285 E0.31916

G1 X61.306 Y64.885 E0.01827

G1 X54.149 Y72.041 E0.30805

G1 X54.369 Y72.422 E0.01337

G1 X61.306 Y65.485 E0.29859

M73 P60 R27

G1 X61.306 Y66.085 E0.01827

G1 X54.589 Y72.803 E0.28914

G1 X54.769 Y73.222 E0.01390

G1 X61.306 Y66.686 E0.28136

G1 X61.306 Y67.286 E0.01827

G1 X54.947 Y73.644 E0.27370

G1 X54.985 Y73.771 E0.00402

G1 X55.421 Y73.771 E0.01328

G1 X61.306 Y67.886 E0.25331

G1 X61.306 Y68.486 E0.01827

G1 X56.021 Y73.771 E0.22747

G1 X56.622 Y73.771 E0.01827

G1 X61.306 Y69.087 E0.20163

G1 X61.306 Y69.687 E0.01827

G1 X57.222 Y73.771 E0.17580

G1 X57.822 Y73.771 E0.01827

G1 X61.306 Y70.287 E0.14996

G1 X61.306 Y70.887 E0.01827

G1 X58.422 Y73.771 E0.12412

G1 X59.022 Y73.771 E0.01827

G1 X61.306 Y71.487 E0.09828

G1 X61.306 Y72.088 E0.01827

G1 X59.623 Y73.771 E0.07245

G1 X60.223 Y73.771 E0.01827

G1 X61.306 Y72.688 E0.04661

G1 X61.306 Y73.288 E0.01827

G1 X60.635 Y73.959 E0.02887

M204 S1250

; stop printing object Petg print.STL id:14 copy 0

; printing object tpu print.STL id:15 copy 0

; stop printing object tpu print.STL id:15 copy 0

; printing object Petg print.STL id:10 copy 0

G1 E-4.00000 F2400.000

G1 X61.568 Y82.562 F9000.000

G1 E4.00000 F900.000

M204 S800

;TYPE:Perimeter

;WIDTH:0.45

G1 F1292.454

G1 X61.568 Y96.276 E0.41094

G1 X54.697 Y96.276 E0.20589

G1 X54.605 Y95.953 E0.01007

G1 X54.270 Y95.159 E0.02583

```
G1 X53.840 Y94.412 E0.02583
G1 X53.321 Y93.723 E0.02583
G1 X52.722 Y93.104 E0.02583
G1 X52.050 Y92.563 E0.02583
G1 X51.318 Y92.109 E0.02583
G1 X50.535 Y91.748 E0.02583
G1 X49.713 Y91.487 E0.02583
G1 X48.866 Y91.328 E0.02583
G1 X48.026 Y91.276 E0.02523
G1 X39.853 Y91.276 E0.24487
G1 X39.853 Y87.562 E0.11130
G1 X48.026 Y87.562 E0.24487
G1 X48.866 Y87.510 E0.02523
G1 X49.713 Y87.351 E0.02583
G1 X50.535 Y87.090 E0.02583
G1 X51.318 Y86.729 E0.02583
G1 X52.050 Y86.275 E0.02583
G1 X52.722 Y85.734 E0.02583
G1 X53.321 Y85.115 E0.02583
G1 X53.840 Y84.427 E0.02583
G1 X54.270 Y83.680 E0.02583
G1 X54.605 Y82.885 E0.02583
G1 X54.697 Y82.562 E0.01007
G1 X61.508 Y82.562 E0.20409
M204 S1250
G1 X61.986 Y82.144 F9000.000
M204 S800
;TYPE:External perimeter
G1 F1292.454
```

```
G1 X61.986 Y96.694 E0.43598
G1 X54.381 Y96.694 E0.22787
G1 X54.210 Y96.091 E0.01877
G1 X53.895 Y95.345 E0.02428
G1 X53.491 Y94.643 E0.02428
G1 X53.003 Y93.995 E0.02428
G1 X52.439 Y93.413 E0.02428
G1 X51.808 Y92.905 E0.02428
G1 X51.119 Y92.478 E0.02428
G1 X50.383 Y92.139 E0.02428
G1 X49.611 Y91.893 E0.02428
G1 X48.815 Y91.744 E0.02428
G1 X48.013 Y91.694 E0.02407
G1 X39.436 Y91.694 E0.25701
G1 X39.436 Y87.144 E0.13634
G1 X48.013 Y87.144 E0.25701
G1 X48.815 Y87.095 E0.02407
G1 X49.611 Y86.946 E0.02428
G1 X50.383 Y86.700 E0.02428
G1 X51.119 Y86.361 E0.02428
G1 X51.808 Y85.934 E0.02428
G1 X52.439 Y85.425 E0.02428
G1 X53.003 Y84.843 E0.02428
G1 X53.491 Y84.196 E0.02428
G1 X53.895 Y83.493 E0.02428
G1 X54.210 Y82.747 E0.02428
G1 X54.381 Y82.144 E0.01877
G1 X61.926 Y82.144 E0.22608
M204 S1250
```

```
G1 X61.795 Y82.496 F9000.000
G1 E-4.00000 F2400.000
G1 X54.369 Y83.759 F9000.000
G1 E4.00000 F900.000
M204 S1000
;TYPE:Solid infill
;WIDTH:0.456608
G1 F1272.331
G1 X55.253 Y82.875 E0.03805
G1 X55.853 Y82.875 E0.01827
G1 X47.765 Y90.963 E0.34814
G1 X48.346 Y90.982 E0.01769
G1 X56.453 Y82.875 E0.34897
G1 X57.053 Y82.875 E0.01827
G1 X48.911 Y91.018 E0.35050
G1 X49.417 Y91.112 E0.01566
G1 X57.654 Y82.875 E0.35457
G1 X58.254 Y82.875 E0.01827
G1 X49.909 Y91.220 E0.35921
G1 X50.364 Y91.365 E0.01454
G1 X58.854 Y82.875 E0.36545
G1 X59.454 Y82.875 E0.01827
G1 X50.803 Y91.527 E0.37241
G1 X51.214 Y91.716 E0.01377
G1 X60.055 Y82.875 E0.38056
G1 X60.655 Y82.875 E0.01827
G1 X51.609 Y91.921 E0.38938
G1 X51.980 Y92.151 E0.01327
G1 X61.254 Y82.876 E0.39924
```

```
G1 X61.254 Y83.476 E0.01827
G1 X52.338 Y92.393 E0.38382
G1 X52.670 Y92.660 E0.01299
G1 X61.254 Y84.076 E0.36951
G1 X61.254 Y84.676 E0.01827
G1 X52.995 Y92.936 E0.35554
G1 X53.290 Y93.241 E0.01292
G1 X61.254 Y85.277 E0.34283
G1 X61.254 Y85.877 E0.01827
G1 X53.582 Y93.549 E0.33026
G1 X53.840 Y93.891 E0.01305
G1 X61.254 Y86.477 E0.31916
G1 X61.254 Y87.077 E0.01827
G1 X54.098 Y94.234 E0.30805
G1 X54.318 Y94.614 E0.01337
G1 X61.254 Y87.678 E0.29859
G1 X61.254 Y88.278 E0.01827
G1 X54.537 Y94.995 E0.28914
G1 X54.718 Y95.414 E0.01390
G1 X61.254 Y88.878 E0.28136
G1 X61.254 Y89.478 E0.01827
G1 X54.896 Y95.837 E0.27370
G1 X54.934 Y95.963 E0.00402
G1 X55.370 Y95.963 E0.01328
G1 X61.254 Y90.078 E0.25331
G1 X61.254 Y90.679 E0.01827
G1 X55.970 Y95.963 E0.22747
G1 X56.570 Y95.963 E0.01827
G1 X61.254 Y91.279 E0.20163
```

```
G1 X61.254 Y91.879 E0.01827
G1 X57.171 Y95.963 E0.17580
G1 X57.771 Y95.963 E0.01827
G1 X61.254 Y92.479 E0.14996
G1 X61.254 Y93.080 E0.01827
G1 X58.371 Y95.963 E0.12412
G1 X58.971 Y95.963 E0.01827
G1 X61.254 Y93.680 E0.09828
G1 X61.254 Y94.280 E0.01827
G1 X59.571 Y95.963 E0.07245
G1 X60.172 Y95.963 E0.01827
G1 X61.254 Y94.880 E0.04661
G1 X61.254 Y95.480 E0.01827
G1 X60.584 Y96.151 E0.02887
M204 S1250
G1 E-4.00000 F2400.000
G1 X46.977 Y91.151 F9000.000
G1 E4.00000 F900.000
M204 S1000
G1 F1272.331
G1 X50.828 Y87.300 E0.16578
G1 X50.648 Y87.383 E0.00604
G1 X49.910 Y87.618 E0.02358
G1 X46.565 Y90.963 E0.14400
G1 X45.965 Y90.963 E0.01827
G1 X49.152 Y87.775 E0.13722
G1 X48.905 Y87.822 E0.00767
G1 X48.480 Y87.848 E0.01297
G1 X45.364 Y90.963 E0.13409
```

G1 X44.764 Y90.963 E0.01827

G1 X47.852 Y87.875 E0.13291

G1 X47.252 Y87.875 E0.01827

G1 X44.164 Y90.963 E0.13291

G1 X43.564 Y90.963 E0.01827

G1 X46.651 Y87.875 E0.13291

G1 X46.051 Y87.875 E0.01827

G1 X42.964 Y90.963 E0.13291

G1 X42.363 Y90.963 E0.01827

G1 X45.451 Y87.875 E0.13291

G1 X44.851 Y87.875 E0.01827

G1 X41.763 Y90.963 E0.13291

G1 X41.163 Y90.963 E0.01827

G1 X44.251 Y87.875 E0.13291

G1 X43.650 Y87.875 E0.01827

G1 X40.563 Y90.963 E0.13291

G1 X40.167 Y90.963 E0.01205

G1 X40.167 Y90.759 E0.00622

G1 X43.050 Y87.875 E0.12412

G1 X42.450 Y87.875 E0.01827

G1 X40.167 Y90.158 E0.09828

G1 X40.167 Y89.558 E0.01827

G1 X41.850 Y87.875 E0.07244

G1 X41.250 Y87.875 E0.01827

G1 X40.167 Y88.958 E0.04661

G1 X40.167 Y88.358 E0.01827

G1 X40.837 Y87.687 E0.02886

M204 S1250

; stop printing object Petg print.STL id:10 copy 0

```
; printing object tpu print.STL id:11 copy 0
; stop printing object tpu print.STL id:11 copy 0
; printing object tpu print.STL id:13 copy 0
; stop printing object tpu print.STL id:13 copy 0
; printing object tpu print.STL id:17 copy 0
; stop printing object tpu print.STL id:17 copy 0
; printing object Petg print.STL id:16 copy 0
G1 E-4.00000 F2400.000
G1 X54.688 Y122.031 F9000.000
G1 E4.00000 F900.000
M204 S800
;TYPE:Perimeter
;WIDTH:0.45
G1 F1292.454
G1 X61.560 Y122.031 E0.20589
G1 X61.560 Y135.746 E0.41094
G1 X54.688 Y135.746 E0.20589
G1 X54.597 Y135.422 E0.01007
G1 X54.262 Y134.628 E0.02583
G1 X53.832 Y133.881 E0.02583
G1 X53.313 Y133.193 E0.02583
G1 X52.713 Y132.573 E0.02583
G1 X52.042 Y132.033 E0.02583
G1 X51.309 Y131.578 E0.02583
G1 X50.526 Y131.218 E0.02583
G1 X49.705 Y130.956 E0.02583
G1 X48.858 Y130.798 E0.02583
G1 X48.017 Y130.746 E0.02523
G1 X39.845 Y130.746 E0.24487
```

```
G1 X39.845 Y127.031 E0.11130
G1 X48.017 Y127.031 E0.24487
G1 X48.858 Y126.980 E0.02523
G1 X49.705 Y126.821 E0.02583
G1 X50.526 Y126.559 E0.02583
G1 X51.309 Y126.199 E0.02583
G1 X52.042 Y125.745 E0.02583
G1 X52.713 Y125.204 E0.02583
G1 X53.313 Y124.584 E0.02583
G1 X53.832 Y123.896 E0.02583
G1 X54.262 Y123.149 E0.02583
G1 X54.597 Y122.355 E0.02583
G1 X54.672 Y122.089 E0.00828
M204 S1250
G1 X54.373 Y121.614 F9000.000
M204 S800
;TYPE:External perimeter
G1 F1292.454
G1 X61.977 Y121.614 E0.22787
G1 X61.977 Y136.164 E0.43598
G1 X54.373 Y136.164 E0.22787
G1 X54.202 Y135.561 E0.01877
G1 X53.887 Y134.814 E0.02428
G1 X53.482 Y134.112 E0.02428
G1 X52.995 Y133.465 E0.02428
G1 X52.431 Y132.883 E0.02428
G1 X51.800 Y132.374 E0.02428
G1 X51.111 Y131.947 E0.02428
G1 X50.375 Y131.608 E0.02428
```

```
G1 X49.603 Y131.362 E0.02428
G1 X48.806 Y131.213 E0.02428
G1 X48.004 Y131.164 E0.02407
G1 X39.427 Y131.164 E0.25701
G1 X39.427 Y126.614 E0.13634
G1 X48.004 Y126.614 E0.25701
G1 X48.806 Y126.564 E0.02407
G1 X49.603 Y126.415 E0.02428
G1 X50.375 Y126.169 E0.02428
G1 X51.111 Y125.830 E0.02428
G1 X51.800 Y125.403 E0.02428
G1 X52.431 Y124.895 E0.02428
G1 X52.995 Y124.312 E0.02428
G1 X53.482 Y123.665 E0.02428
G1 X53.887 Y122.963 E0.02428
G1 X54.202 Y122.216 E0.02428
G1 X54.356 Y121.671 E0.01697
M204 S1250
G1 X54.694 Y121.852 F9000.000
G1 X54.361 Y123.229
M204 S1000
;TYPE:Solid infill
;WIDTH:0.456608
G1 F1272.331
G1 X55.245 Y122.345 E0.03805
G1 X55.845 Y122.345 E0.01827
G1 X47.757 Y130.432 E0.34814
G1 X48.338 Y130.452 E0.01769
G1 X56.445 Y122.345 E0.34897
```

G1 X57.045 Y122.345 E0.01827

G1 X48.903 Y130.487 E0.35050

G1 X49.408 Y130.582 E0.01566

G1 X57.645 Y122.345 E0.35457

G1 X58.246 Y122.345 E0.01827

G1 X49.901 Y130.690 E0.35921

M73 P61 R27

G1 X50.356 Y130.835 E0.01454

G1 X58.846 Y122.345 E0.36545

G1 X59.446 Y122.345 E0.01827

G1 X50.795 Y130.996 E0.37241

G1 X51.205 Y131.186 E0.01377

G1 X60.046 Y122.345 E0.38056

G1 X60.646 Y122.345 E0.01827

G1 X51.601 Y131.390 E0.38938

G1 X51.971 Y131.620 E0.01327

G1 X61.246 Y122.345 E0.39924

G1 X61.246 Y122.945 E0.01827

G1 X52.330 Y131.862 E0.38382

G1 X52.662 Y132.130 E0.01299

G1 X61.246 Y123.546 E0.36951

G1 X61.246 Y124.146 E0.01827

G1 X52.987 Y132.405 E0.35554

G1 X53.282 Y132.710 E0.01292

G1 X61.246 Y124.746 E0.34283

G1 X61.246 Y125.346 E0.01827

G1 X53.574 Y133.019 E0.33026

G1 X53.832 Y133.361 E0.01305

G1 X61.246 Y125.947 E0.31916

```
G1 X61.246 Y126.547 E0.01827
G1 X54.090 Y133.703 E0.30805
M73 P61 R26
G1 X54.310 Y134.083 E0.01337
G1 X61.246 Y127.147 E0.29859
G1 X61.246 Y127.747 E0.01827
G1 X54.529 Y134.464 E0.28914
G1 X54.710 Y134.884 E0.01390
G1 X61.246 Y128.347 E0.28136
G1 X61.246 Y128.948 E0.01827
G1 X54.888 Y135.306 E0.27370
G1 X54.925 Y135.432 E0.00402
G1 X55.362 Y135.432 E0.01328
G1 X61.246 Y129.548 E0.25331
G1 X61.246 Y130.148 E0.01827
G1 X55.962 Y135.432 E0.22747
G1 X56.562 Y135.432 E0.01827
G1 X61.246 Y130.748 E0.20163
G1 X61.246 Y131.349 E0.01827
G1 X57.162 Y135.432 E0.17580
G1 X57.763 Y135.432 E0.01827
G1 X61.246 Y131.949 E0.14996
G1 X61.246 Y132.549 E0.01827
G1 X58.363 Y135.432 E0.12412
G1 X58.963 Y135.432 E0.01827
G1 X61.246 Y133.149 E0.09828
G1 X61.246 Y133.749 E0.01827
G1 X59.563 Y135.432 E0.07245
G1 X60.163 Y135.432 E0.01827
```

```
G1 X61.246 Y134.350 E0.04661
G1 X61.246 Y134.950 E0.01827
G1 X60.576 Y135.620 E0.02887
M204 S1250
G1 E-4.00000 F2400.000
G1 X46.969 Y130.620 F9000.000
G1 E4.00000 F900.000
M204 S1000
G1 F1272.331
G1 X50.820 Y126.769 E0.16578
G1 X50.640 Y126.852 E0.00604
G1 X49.902 Y127.087 E0.02358
G1 X46.557 Y130.432 E0.14400
G1 X45.956 Y130.432 E0.01827
G1 X49.144 Y127.245 E0.13722
G1 X48.896 Y127.291 E0.00767
G1 X48.471 Y127.317 E0.01297
G1 X45.356 Y130.432 E0.13409
G1 X44.756 Y130.432 E0.01827
G1 X47.844 Y127.345 E0.13291
G1 X47.243 Y127.345 E0.01827
G1 X44.156 Y130.432 E0.13291
G1 X43.556 Y130.432 E0.01827
G1 X46.643 Y127.345 E0.13291
G1 X46.043 Y127.345 E0.01827
G1 X42.955 Y130.432 E0.13291
G1 X42.355 Y130.432 E0.01827
G1 X45.443 Y127.345 E0.13291
G1 X44.843 Y127.345 E0.01827
```

```
G1 X41.755 Y130.432 E0.13291
G1 X41.155 Y130.432 E0.01827
G1 X44.242 Y127.345 E0.13291
G1 X43.642 Y127.345 E0.01827
G1 X40.554 Y130.432 E0.13291
G1 X40.159 Y130.432 E0.01205
G1 X40.159 Y130.228 E0.00622
G1 X43.042 Y127.345 E0.12412
G1 X42.442 Y127.345 E0.01827
G1 X40.159 Y129.628 E0.09828
G1 X40.159 Y129.028 E0.01827
G1 X41.841 Y127.345 E0.07244
G1 X41.241 Y127.345 E0.01827
G1 X40.159 Y128.428 E0.04661
G1 X40.159 Y127.827 E0.01827
G1 X40.829 Y127.157 E0.02886
M204 S1250
; stop printing object Petg print.STL id:16 copy 0
; printing object Petg print.STL id:12 copy 0
G1 E-4.00000 F2400.000
G1 X39.860 Y112.092 F9000.000
G1 E4.00000 F900.000
M204 S800
;TYPE:Perimeter
;WIDTH:0.45
G1 F1292.454
G1 X39.860 Y108.377 E0.11130
G1 X48.032 Y108.377 E0.24487
G1 X48.873 Y108.326 E0.02523
```

G1 X49.720 Y108.167 E0.02583

G1 X50.542 Y107.905 E0.02583

G1 X51.325 Y107.545 E0.02583

G1 X52.057 Y107.091 E0.02583

G1 X52.728 Y106.550 E0.02583

G1 X53.328 Y105.930 E0.02583

G1 X53.847 Y105.242 E0.02583

G1 X54.277 Y104.495 E0.02583

G1 X54.612 Y103.701 E0.02583

G1 X54.704 Y103.377 E0.01007

G1 X61.575 Y103.377 E0.20589

G1 X61.575 Y117.092 E0.41094

G1 X54.704 Y117.092 E0.20589

G1 X54.612 Y116.768 E0.01007

G1 X54.277 Y115.974 E0.02583

G1 X53.847 Y115.227 E0.02583

G1 X53.328 Y114.539 E0.02583

G1 X52.728 Y113.920 E0.02583

G1 X52.057 Y113.379 E0.02583

G1 X51.325 Y112.924 E0.02583

G1 X50.542 Y112.564 E0.02583

G1 X49.720 Y112.302 E0.02583

G1 X48.873 Y112.144 E0.02583

G1 X48.032 Y112.092 E0.02523

G1 X39.920 Y112.092 E0.24307

M204 S1250

G1 X39.442 Y112.510 F9000.000

M204 S800

;TYPE:External perimeter

```
G1 F1292.454
G1 X39.442 Y107.960 E0.13634
G1 X48.020 Y107.960 E0.25701
G1 X48.821 Y107.910 E0.02407
G1 X49.618 Y107.761 E0.02428
G1 X50.390 Y107.515 E0.02428
G1 X51.126 Y107.176 E0.02428
G1 X51.815 Y106.749 E0.02428
G1 X52.446 Y106.241 E0.02428
G1 X53.010 Y105.658 E0.02428
G1 X53.498 Y105.011 E0.02428
G1 X53.902 Y104.309 E0.02428
G1 X54.217 Y103.562 E0.02428
G1 X54.388 Y102.960 E0.01877
G1 X61.992 Y102.960 E0.22787
G1 X61.992 Y117.510 E0.43598
G1 X54.388 Y117.510 E0.22787
G1 X54.217 Y116.907 E0.01877
G1 X53.902 Y116.160 E0.02428
G1 X53.498 Y115.458 E0.02428
G1 X53.010 Y114.811 E0.02428
G1 X52.446 Y114.229 E0.02428
G1 X51.815 Y113.720 E0.02428
G1 X51.126 Y113.293 E0.02428
G1 X50.390 Y112.954 E0.02428
G1 X49.618 Y112.708 E0.02428
G1 X48.821 Y112.559 E0.02428
G1 X48.020 Y112.510 E0.02407
G1 X39.502 Y112.510 E0.25521
```

```
M204 S1250
G1 X39.643 Y112.164 F9000.000
G1 X40.844 Y108.503
M204 S1000
;TYPE:Solid infill
;WIDTH:0.456608
G1 F1272.331
G1 X40.174 Y109.173 E0.02886
G1 X40.174 Y109.774 E0.01827
G1 X41.256 Y108.691 E0.04661
G1 X41.857 Y108.691 E0.01827
G1 X40.174 Y110.374 E0.07244
G1 X40.174 Y110.974 E0.01827
G1 X42.457 Y108.691 E0.09828
G1 X43.057 Y108.691 E0.01827
G1 X40.174 Y111.574 E0.12412
G1 X40.174 Y111.778 E0.00622
G1 X40.570 Y111.778 E0.01205
G1 X43.657 Y108.691 E0.13291
G1 X44.257 Y108.691 E0.01827
G1 X41.170 Y111.778 E0.13291
G1 X41.770 Y111.778 E0.01827
G1 X44.858 Y108.691 E0.13291
G1 X45.458 Y108.691 E0.01827
G1 X42.370 Y111.778 E0.13291
G1 X42.970 Y111.778 E0.01827
G1 X46.058 Y108.691 E0.13291
G1 X46.658 Y108.691 E0.01827
G1 X43.571 Y111.778 E0.13291
```

```
G1 X44.171 Y111.778 E0.01827
G1 X47.259 Y108.691 E0.13291
G1 X47.859 Y108.691 E0.01827
G1 X44.771 Y111.778 E0.13291
G1 X45.371 Y111.778 E0.01827
G1 X48.486 Y108.663 E0.13409
G1 X48.912 Y108.637 E0.01297
G1 X49.159 Y108.591 E0.00767
G1 X45.972 Y111.778 E0.13722
G1 X46.572 Y111.778 E0.01827
G1 X49.917 Y108.433 E0.14400
G1 X50.655 Y108.198 E0.02358
G1 X50.835 Y108.115 E0.00604
G1 X46.984 Y111.967 E0.16578
M204 S1250
G1 E-4.00000 F2400.000
G1 X54.376 Y104.575 F9000.000
G1 E4.00000 F900.000
M204 S1000
G1 F1272.331
G1 X55.260 Y103.691 E0.03805
G1 X55.860 Y103.691 E0.01827
G1 X47.772 Y111.778 E0.34814
G1 X48.353 Y111.798 E0.01769
G1 X56.460 Y103.691 E0.34897
G1 X57.060 Y103.691 E0.01827
G1 X48.918 Y111.833 E0.35050
G1 X49.423 Y111.928 E0.01566
G1 X57.661 Y103.691 E0.35457
```

```
G1 X58.261 Y103.691 E0.01827
G1 X49.916 Y112.036 E0.35921
G1 X50.371 Y112.181 E0.01454
G1 X58.861 Y103.691 E0.36545
G1 X59.461 Y103.691 E0.01827
G1 X50.810 Y112.342 E0.37241
G1 X51.221 Y112.532 E0.01377
G1 X60.061 Y103.691 E0.38056
G1 X60.662 Y103.691 E0.01827
G1 X51.616 Y112.736 E0.38938
G1 X51.986 Y112.966 E0.01327
G1 X61.261 Y103.691 E0.39924
G1 X61.261 Y104.292 E0.01827
G1 X52.345 Y113.208 E0.38382
G1 X52.677 Y113.476 E0.01299
G1 X61.261 Y104.892 E0.36951
G1 X61.261 Y105.492 E0.01827
G1 X53.002 Y113.751 E0.35554
G1 X53.297 Y114.056 E0.01292
G1 X61.261 Y106.092 E0.34283
G1 X61.261 Y106.692 E0.01827
G1 X53.589 Y114.365 E0.33026
G1 X53.847 Y114.707 E0.01305
G1 X61.261 Y107.293 E0.31916
G1 X61.261 Y107.893 E0.01827
G1 X54.105 Y115.049 E0.30805
G1 X54.325 Y115.430 E0.01337
G1 X61.261 Y108.493 E0.29859
G1 X61.261 Y109.093 E0.01827
```

G1 X54.544 Y115.810 E0.28914

G1 X54.725 Y116.230 E0.01390

G1 X61.261 Y109.694 E0.28136

G1 X61.261 Y110.294 E0.01827

G1 X54.903 Y116.652 E0.27370

G1 X54.941 Y116.778 E0.00402

G1 X55.377 Y116.778 E0.01328

G1 X61.261 Y110.894 E0.25331

G1 X61.261 Y111.494 E0.01827

G1 X55.977 Y116.778 E0.22747

G1 X56.577 Y116.778 E0.01827

G1 X61.261 Y112.094 E0.20163

G1 X61.261 Y112.695 E0.01827

G1 X57.177 Y116.778 E0.17580

M73 P62 R26

G1 X57.778 Y116.778 E0.01827

G1 X61.261 Y113.295 E0.14996

G1 X61.261 Y113.895 E0.01827

G1 X58.378 Y116.778 E0.12412

G1 X58.978 Y116.778 E0.01827

G1 X61.261 Y114.495 E0.09828

G1 X61.261 Y115.095 E0.01827

G1 X59.578 Y116.778 E0.07245

G1 X60.179 Y116.778 E0.01827

G1 X61.261 Y115.696 E0.04661

G1 X61.261 Y116.296 E0.01827

G1 X60.591 Y116.967 E0.02887

M204 S1250

; stop printing object Petg print.STL id:12 copy 0

```
; printing object tpu print.STL id:7 copy 0
; stop printing object tpu print.STL id:7 copy 0
; printing object tpu print.STL id:3 copy 0
; stop printing object tpu print.STL id:3 copy 0
; printing object Petg print.STL id:2 copy 0
G1 E-4.00000 F2400.000
G1 X89.650 Y112.673 F9000.000
G1 E4.00000 F900.000
M204 S800
;TYPE:Perimeter
;WIDTH:0.45
G1 F1292.454
G1 X89.650 Y108.958 E0.11130
G1 X97.822 Y108.958 E0.24487
G1 X98.662 Y108.906 E0.02523
G1 X99.510 Y108.748 E0.02583
G1 X100.331 Y108.486 E0.02583
G1 X101.114 Y108.126 E0.02583
G1 X101.847 Y107.672 E0.02583
G1 X102.518 Y107.131 E0.02583
G1 X103.117 Y106.511 E0.02583
G1 X103.636 Y105.823 E0.02583
G1 X104.066 Y105.076 E0.02583
G1 X104.401 Y104.282 E0.02583
G1 X104.493 Y103.958 E0.01007
G1 X111.364 Y103.958 E0.20589
G1 X111.364 Y117.673 E0.41094
G1 X104.493 Y117.673 E0.20589
G1 X104.401 Y117.349 E0.01007
```

G1 X104.066 Y116.555 E0.02583

G1 X103.636 Y115.808 E0.02583

G1 X103.117 Y115.120 E0.02583

G1 X102.518 Y114.500 E0.02583

G1 X101.847 Y113.960 E0.02583

G1 X101.114 Y113.505 E0.02583

G1 X100.331 Y113.145 E0.02583

G1 X99.510 Y112.883 E0.02583

G1 X98.662 Y112.725 E0.02583

G1 X97.822 Y112.673 E0.02523

G1 X89.710 Y112.673 E0.24307

M204 S1250

G1 X89.232 Y113.091 F9000.000

M204 S800

;TYPE:External perimeter

G1 F1292.454

G1 X89.232 Y108.541 E0.13634

G1 X97.809 Y108.541 E0.25701

G1 X98.611 Y108.491 E0.02407

G1 X99.407 Y108.342 E0.02428

G1 X100.180 Y108.096 E0.02428

G1 X100.916 Y107.757 E0.02428

G1 X101.604 Y107.330 E0.02428

G1 X102.235 Y106.822 E0.02428

G1 X102.799 Y106.239 E0.02428

G1 X103.287 Y105.592 E0.02428

G1 X103.691 Y104.890 E0.02428

G1 X104.006 Y104.143 E0.02428

G1 X104.177 Y103.541 E0.01877

```
G1 X111.782 Y103.541 E0.22787
G1 X111.782 Y118.091 E0.43598
G1 X104.177 Y118.091 E0.22787
G1 X104.006 Y117.488 E0.01877
G1 X103.691 Y116.741 E0.02428
G1 X103.287 Y116.039 E0.02428
G1 X102.799 Y115.392 E0.02428
G1 X102.235 Y114.810 E0.02428
G1 X101.604 Y114.301 E0.02428
G1 X100.916 Y113.874 E0.02428
G1 X100.180 Y113.535 E0.02428
G1 X99.407 Y113.289 E0.02428
G1 X98.611 Y113.140 E0.02428
G1 X97.809 Y113.091 E0.02407
G1 X89.292 Y113.091 E0.25521
M204 S1250
G1 X89.432 Y112.744 F9000.000
G1 X90.634 Y109.084
M204 S1000
;TYPE:Solid infill
;WIDTH:0.456608
G1 F1272.331
G1 X89.963 Y109.754 E0.02886
G1 X89.963 Y110.354 E0.01827
G1 X91.046 Y109.272 E0.04661
G1 X91.646 Y109.272 E0.01827
G1 X89.963 Y110.955 E0.07244
G1 X89.963 Y111.555 E0.01827
G1 X92.246 Y109.272 E0.09828
```

G1 X92.846 Y109.272 E0.01827

G1 X89.963 Y112.155 E0.12412

G1 X89.963 Y112.359 E0.00622

G1 X90.359 Y112.359 E0.01205

G1 X93.447 Y109.272 E0.13291

G1 X94.047 Y109.272 E0.01827

G1 X90.959 Y112.359 E0.13291

G1 X91.559 Y112.359 E0.01827

G1 X94.647 Y109.272 E0.13291

G1 X95.247 Y109.272 E0.01827

G1 X92.160 Y112.359 E0.13291

G1 X92.760 Y112.359 E0.01827

G1 X95.847 Y109.272 E0.13291

G1 X96.448 Y109.272 E0.01827

G1 X93.360 Y112.359 E0.13291

G1 X93.960 Y112.359 E0.01827

G1 X97.048 Y109.272 E0.13291

G1 X97.648 Y109.272 E0.01827

G1 X94.560 Y112.359 E0.13291

G1 X95.161 Y112.359 E0.01827

G1 X98.276 Y109.244 E0.13409

G1 X98.701 Y109.218 E0.01297

G1 X98.949 Y109.172 E0.00767

G1 X95.761 Y112.359 E0.13722

G1 X96.361 Y112.359 E0.01827

G1 X99.706 Y109.014 E0.14400

G1 X100.444 Y108.779 E0.02358

G1 X100.625 Y108.696 E0.00604

G1 X96.773 Y112.547 E0.16578

```
M204 S1250
G1 E-4.00000 F2400.000
G1 X104.165 Y105.156 F9000.000
G1 E4.00000 F900.000
M204 S1000
G1 F1272.331
G1 X105.049 Y104.272 E0.03805
G1 X105.649 Y104.272 E0.01827
G1 X97.562 Y112.359 E0.34814
G1 X98.143 Y112.379 E0.01769
G1 X106.249 Y104.272 E0.34897
G1 X106.850 Y104.272 E0.01827
G1 X98.707 Y112.414 E0.35050
G1 X99.213 Y112.509 E0.01566
G1 X107.450 Y104.272 E0.35457
G1 X108.050 Y104.272 E0.01827
G1 X99.705 Y112.617 E0.35921
G1 X100.160 Y112.762 E0.01454
G1 X108.650 Y104.272 E0.36545
G1 X109.251 Y104.272 E0.01827
G1 X100.599 Y112.923 E0.37241
G1 X101.010 Y113.112 E0.01377
G1 X109.851 Y104.272 E0.38056
G1 X110.451 Y104.272 E0.01827
G1 X101.405 Y113.317 E0.38938
G1 X101.776 Y113.547 E0.01327
G1 X111.051 Y104.272 E0.39924
G1 X111.051 Y104.872 E0.01827
G1 X102.134 Y113.789 E0.38382
```

G1 X102.467 Y114.057 E0.01299

G1 X111.051 Y105.473 E0.36951

G1 X111.051 Y106.073 E0.01827

G1 X102.791 Y114.332 E0.35554

G1 X103.086 Y114.637 E0.01292

G1 X111.051 Y106.673 E0.34283

G1 X111.051 Y107.273 E0.01827

G1 X103.378 Y114.946 E0.33026

G1 X103.636 Y115.288 E0.01305

G1 X111.051 Y107.874 E0.31916

G1 X111.051 Y108.474 E0.01827

G1 X103.894 Y115.630 E0.30805

G1 X104.114 Y116.010 E0.01337

G1 X111.051 Y109.074 E0.29859

G1 X111.051 Y109.674 E0.01827

G1 X104.334 Y116.391 E0.28914

G1 X104.514 Y116.811 E0.01390

G1 X111.051 Y110.274 E0.28136

G1 X111.051 Y110.875 E0.01827

G1 X104.692 Y117.233 E0.27370

G1 X104.730 Y117.359 E0.00402

G1 X105.166 Y117.359 E0.01328

G1 X111.051 Y111.475 E0.25331

G1 X111.051 Y112.075 E0.01827

G1 X105.766 Y117.359 E0.22747

G1 X106.367 Y117.359 E0.01827

G1 X111.051 Y112.675 E0.20163

M73 P62 R25

G1 X111.051 Y113.275 E0.01827

```
G1 X106.967 Y117.359 E0.17580
G1 X107.567 Y117.359 E0.01827
G1 X111.051 Y113.876 E0.14996
G1 X111.051 Y114.476 E0.01827
G1 X108.167 Y117.359 E0.12412
G1 X108.767 Y117.359 E0.01827
G1 X111.051 Y115.076 E0.09828
G1 X111.051 Y115.676 E0.01827
G1 X109.368 Y117.359 E0.07245
G1 X109.968 Y117.359 E0.01827
G1 X111.051 Y116.277 E0.04661
G1 X111.051 Y116.877 E0.01827
G1 X110.380 Y117.547 E0.02887
M204 S1250
; stop printing object Petg print.STL id:2 copy 0
; printing object Petg print.STL id:6 copy 0
G1 X111.349 Y122.612 F9000.000
M204 S800
;TYPE:Perimeter
;WIDTH:0.45
G1 F1292.454
G1 X111.349 Y136.327 E0.41094
G1 X104.478 Y136.327 E0.20589
G1 X104.386 Y136.003 E0.01007
G1 X104.051 Y135.209 E0.02583
G1 X103.621 Y134.462 E0.02583
G1 X103.102 Y133.774 E0.02583
G1 X102.503 Y133.154 E0.02583
G1 X101.831 Y132.613 E0.02583
```

G1 X101.099 Y132.159 E0.02583

G1 X100.316 Y131.799 E0.02583

G1 X99.495 Y131.537 E0.02583

G1 X98.647 Y131.379 E0.02583

G1 X97.807 Y131.327 E0.02523

G1 X89.635 Y131.327 E0.24487

G1 X89.635 Y127.612 E0.11130

G1 X97.807 Y127.612 E0.24487

G1 X98.647 Y127.560 E0.02523

G1 X99.495 Y127.402 E0.02583

G1 X100.316 Y127.140 E0.02583

G1 X101.099 Y126.780 E0.02583

G1 X101.831 Y126.325 E0.02583

G1 X102.503 Y125.785 E0.02583

G1 X103.102 Y125.165 E0.02583

G1 X103.621 Y124.477 E0.02583

G1 X104.051 Y123.730 E0.02583

G1 X104.386 Y122.936 E0.02583

G1 X104.478 Y122.612 E0.01007

G1 X111.289 Y122.612 E0.20409

M204 S1250

G1 X111.767 Y122.194 F9000.000

M204 S800

;TYPE:External perimeter

G1 F1292.454

G1 X111.767 Y136.744 E0.43598

G1 X104.162 Y136.744 E0.22787

G1 X103.991 Y136.142 E0.01877

G1 X103.676 Y135.395 E0.02428

```
G1 X103.272 Y134.693 E0.02428
G1 X102.784 Y134.046 E0.02428
G1 X102.220 Y133.463 E0.02428
G1 X101.589 Y132.955 E0.02428
G1 X100.901 Y132.528 E0.02428
G1 X100.164 Y132.189 E0.02428
G1 X99.392 Y131.943 E0.02428
G1 X98.596 Y131.794 E0.02428
G1 X97.794 Y131.744 E0.02407
G1 X89.217 Y131.744 E0.25701
G1 X89.217 Y127.194 E0.13634
G1 X97.794 Y127.194 E0.25701
G1 X98.596 Y127.145 E0.02407
G1 X99.392 Y126.996 E0.02428
G1 X100.164 Y126.750 E0.02428
G1 X100.901 Y126.411 E0.02428
G1 X101.589 Y125.984 E0.02428
G1 X102.220 Y125.475 E0.02428
G1 X102.784 Y124.893 E0.02428
G1 X103.272 Y124.246 E0.02428
G1 X103.676 Y123.544 E0.02428
G1 X103.991 Y122.797 E0.02428
G1 X104.162 Y122.194 E0.01877
G1 X111.707 Y122.194 E0.22608
M204 S1250
G1 X111.576 Y122.546 F9000.000
G1 E-4.00000 F2400.000
G1 X104.150 Y123.810 F9000.000
G1 E4.00000 F900.000
```

```
M204 S1000
;TYPE:Solid infill
;WIDTH:0.456608
G1 F1272.331
G1 X105.034 Y122.926 E0.03805
G1 X105.634 Y122.926 E0.01827
G1 X97.546 Y131.013 E0.34814
G1 X98.127 Y131.033 E0.01769
G1 X106.234 Y122.926 E0.34897
G1 X106.835 Y122.926 E0.01827
G1 X98.692 Y131.068 E0.35050
G1 X99.198 Y131.163 E0.01566
G1 X107.435 Y122.926 E0.35457
G1 X108.035 Y122.926 E0.01827
G1 X99.690 Y131.271 E0.35921
G1 X100.145 Y131.416 E0.01454
G1 X108.635 Y122.926 E0.36545
G1 X109.235 Y122.926 E0.01827
G1 X100.584 Y131.577 E0.37241
G1 X100.995 Y131.766 E0.01377
G1 X109.836 Y122.926 E0.38056
G1 X110.436 Y122.926 E0.01827
G1 X101.390 Y131.971 E0.38938
G1 X101.761 Y132.201 E0.01327
G1 X111.036 Y122.926 E0.39924
G1 X111.036 Y123.526 E0.01827
G1 X102.119 Y132.443 E0.38382
M73 P63 R25
G1 X102.451 Y132.711 E0.01299
```

```
G1 X111.036 Y124.127 E0.36951
G1 X111.036 Y124.727 E0.01827
G1 X102.776 Y132.986 E0.35554
G1 X103.071 Y133.291 E0.01292
G1 X111.036 Y125.327 E0.34283
G1 X111.036 Y125.927 E0.01827
G1 X103.363 Y133.600 E0.33026
G1 X103.621 Y133.942 E0.01305
G1 X111.036 Y126.527 E0.31916
G1 X111.036 Y127.128 E0.01827
G1 X103.879 Y134.284 E0.30805
G1 X104.099 Y134.664 E0.01337
G1 X111.036 Y127.728 E0.29859
G1 X111.036 Y128.328 E0.01827
G1 X104.318 Y135.045 E0.28914
G1 X104.499 Y135.465 E0.01390
G1 X111.036 Y128.928 E0.28136
G1 X111.036 Y129.529 E0.01827
G1 X104.677 Y135.887 E0.27370
G1 X104.715 Y136.013 E0.00402
G1 X105.151 Y136.013 E0.01328
G1 X111.036 Y130.129 E0.25331
G1 X111.036 Y130.729 E0.01827
G1 X105.751 Y136.013 E0.22747
G1 X106.351 Y136.013 E0.01827
G1 X111.036 Y131.329 E0.20163
G1 X111.036 Y131.929 E0.01827
G1 X106.952 Y136.013 E0.17580
G1 X107.552 Y136.013 E0.01827
```

```
G1 X111.036 Y132.530 E0.14996
G1 X111.036 Y133.130 E0.01827
G1 X108.152 Y136.013 E0.12412
G1 X108.752 Y136.013 E0.01827
G1 X111.036 Y133.730 E0.09828
G1 X111.036 Y134.330 E0.01827
G1 X109.353 Y136.013 E0.07245
G1 X109.953 Y136.013 E0.01827
G1 X111.036 Y134.931 E0.04661
G1 X111.036 Y135.531 E0.01827
G1 X110.365 Y136.201 E0.02887
M204 S1250
G1 E-4.00000 F2400.000
G1 X96.758 Y131.201 F9000.000
G1 E4.00000 F900.000
M204 S1000
G1 F1272.331
G1 X100.610 Y127.350 E0.16578
G1 X100.429 Y127.433 E0.00604
G1 X99.691 Y127.668 E0.02358
G1 X96.346 Y131.013 E0.14400
G1 X95.746 Y131.013 E0.01827
G1 X98.933 Y127.826 E0.13722
G1 X98.686 Y127.872 E0.00767
G1 X98.261 Y127.898 E0.01297
G1 X95.146 Y131.013 E0.13409
G1 X94.545 Y131.013 E0.01827
G1 X97.633 Y127.926 E0.13291
G1 X97.033 Y127.926 E0.01827
```

G1 X93.945 Y131.013 E0.13291

G1 X93.345 Y131.013 E0.01827

G1 X96.433 Y127.926 E0.13291

G1 X95.832 Y127.926 E0.01827

G1 X92.745 Y131.013 E0.13291

G1 X92.144 Y131.013 E0.01827

G1 X95.232 Y127.926 E0.13291

G1 X94.632 Y127.926 E0.01827

G1 X91.544 Y131.013 E0.13291

G1 X90.944 Y131.013 E0.01827

G1 X94.032 Y127.926 E0.13291

G1 X93.431 Y127.926 E0.01827

G1 X90.344 Y131.013 E0.13291

G1 X89.948 Y131.013 E0.01205

G1 X89.948 Y130.809 E0.00622

G1 X92.831 Y127.926 E0.12412

G1 X92.231 Y127.926 E0.01827

G1 X89.948 Y130.209 E0.09828

G1 X89.948 Y129.609 E0.01827

G1 X91.631 Y127.926 E0.07244

G1 X91.031 Y127.926 E0.01827

G1 X89.948 Y129.008 E0.04661

G1 X89.948 Y128.408 E0.01827

G1 X90.618 Y127.738 E0.02886

M204 S1250

; stop printing object Petg print.STL id:6 copy 0

; printing object tpu print.STL id:27 copy 0

; stop printing object tpu print.STL id:27 copy 0

; printing object tpu print.STL id:23 copy 0

```
; stop printing object tpu print.STL id:23 copy 0
; printing object tpu print.STL id:21 copy 0
; stop printing object tpu print.STL id:21 copy 0
; printing object Petg print.STL id:20 copy 0
G1 E-4.00000 F2400.000
G1 X141.293 Y92.032 F9000.000
G1 E4.00000 F900.000
M204 S800
;TYPE:Perimeter
;WIDTH:0.45
G1 F1292.454
G1 X141.293 Y88.318 E0.11130
G1 X149.465 Y88.318 E0.24487
G1 X150.306 Y88.266 E0.02523
G1 X151.153 Y88.107 E0.02583
G1 X151.974 Y87.846 E0.02583
G1 X152.757 Y87.485 E0.02583
G1 X153.490 Y87.031 E0.02583
G1 X154.161 Y86.490 E0.02583
G1 X154.761 Y85.871 E0.02583
G1 X155.279 Y85.183 E0.02583
G1 X155.710 Y84.436 E0.02583
G1 X156.045 Y83.641 E0.02583
G1 X156.136 Y83.318 E0.01007
G1 X163.007 Y83.318 E0.20589
G1 X163.007 Y97.032 E0.41094
G1 X156.136 Y97.032 E0.20589
G1 X156.045 Y96.709 E0.01007
G1 X155.710 Y95.915 E0.02583
```

```
G1 X155.279 Y95.168 E0.02583
G1 X154.761 Y94.479 E0.02583
G1 X154.161 Y93.860 E0.02583
G1 X153.490 Y93.319 E0.02583
G1 X152.757 Y92.865 E0.02583
G1 X151.974 Y92.504 E0.02583
G1 X151.153 Y92.243 E0.02583
G1 X150.306 Y92.084 E0.02583
G1 X149.465 Y92.032 E0.02523
G1 X141.353 Y92.032 E0.24307
M204 S1250
G1 X140.875 Y92.450 F9000.000
M204 S800
;TYPE:External perimeter
G1 F1292.454
G1 X140.875 Y87.900 E0.13634
G1 X149.452 Y87.900 E0.25701
G1 X150.254 Y87.851 E0.02407
G1 X151.051 Y87.702 E0.02428
G1 X151.823 Y87.456 E0.02428
G1 X152.559 Y87.117 E0.02428
G1 X153.248 Y86.690 E0.02428
G1 X153.879 Y86.181 E0.02428
G1 X154.443 Y85.599 E0.02428
G1 X154.930 Y84.952 E0.02428
G1 X155.335 Y84.249 E0.02428
G1 X155.650 Y83.503 E0.02428
G1 X155.820 Y82.900 E0.01877
G1 X163.425 Y82.900 E0.22787
```

```
G1 X163.425 Y97.450 E0.43598
G1 X155.820 Y97.450 E0.22787
G1 X155.650 Y96.847 E0.01877
G1 X155.335 Y96.101 E0.02428
G1 X154.930 Y95.398 E0.02428
G1 X154.443 Y94.751 E0.02428
G1 X153.879 Y94.169 E0.02428
G1 X153.248 Y93.661 E0.02428
G1 X152.559 Y93.234 E0.02428
G1 X151.823 Y92.895 E0.02428
G1 X151.051 Y92.649 E0.02428
G1 X150.254 Y92.500 E0.02428
G1 X149.452 Y92.450 E0.02407
G1 X140.935 Y92.450 E0.25521
M204 S1250
G1 X141.076 Y92.104 F9000.000
G1 X142.277 Y88.443
M204 S1000
;TYPE:Solid infill
;WIDTH:0.456608
G1 F1272.331
G1 X141.606 Y89.114 E0.02886
G1 X141.606 Y89.714 E0.01827
G1 X142.689 Y88.631 E0.04661
G1 X143.289 Y88.631 E0.01827
G1 X141.606 Y90.314 E0.07244
G1 X141.606 Y90.914 E0.01827
G1 X143.890 Y88.631 E0.09828
G1 X144.490 Y88.631 E0.01827
```

```
G1 X141.606 Y91.515 E0.12412
G1 X141.606 Y91.719 E0.00622
G1 X142.002 Y91.719 E0.01205
G1 X145.090 Y88.631 E0.13291
G1 X145.690 Y88.631 E0.01827
G1 X142.603 Y91.719 E0.13291
G1 X143.203 Y91.719 E0.01827
G1 X146.290 Y88.631 E0.13291
G1 X146.891 Y88.631 E0.01827
G1 X143.803 Y91.719 E0.13291
G1 X144.403 Y91.719 E0.01827
G1 X147.491 Y88.631 E0.13291
G1 X148.091 Y88.631 E0.01827
G1 X145.003 Y91.719 E0.13291
G1 X145.604 Y91.719 E0.01827
G1 X148.691 Y88.631 E0.13291
G1 X149.292 Y88.631 E0.01827
G1 X146.204 Y91.719 E0.13291
G1 X146.804 Y91.719 E0.01827
G1 X149.919 Y88.604 E0.13409
G1 X150.344 Y88.578 E0.01297
G1 X150.592 Y88.531 E0.00767
G1 X147.404 Y91.719 E0.13722
G1 X148.005 Y91.719 E0.01827
G1 X151.350 Y88.374 E0.14400
G1 X152.088 Y88.139 E0.02358
G1 X152.268 Y88.056 E0.00604
G1 X148.417 Y91.907 E0.16578
M204 S1250
```

```
G1 E-4.00000 F2400.000
G1 X155.809 Y84.515 F9000.000
G1 E4.00000 F900.000
M204 S1000
G1 F1272.331
G1 X156.692 Y83.631 E0.03805
G1 X157.293 Y83.631 E0.01827
G1 X149.205 Y91.719 E0.34814
G1 X149.786 Y91.738 E0.01769
G1 X157.893 Y83.631 E0.34897
G1 X158.493 Y83.631 E0.01827
G1 X150.351 Y91.774 E0.35050
G1 X150.856 Y91.868 E0.01566
G1 X159.093 Y83.631 E0.35457
G1 X159.694 Y83.631 E0.01827
G1 X151.349 Y91.976 E0.35921
G1 X151.804 Y92.121 E0.01454
G1 X160.294 Y83.631 E0.36545
G1 X160.894 Y83.631 E0.01827
G1 X152.242 Y92.283 E0.37241
G1 X152.653 Y92.472 E0.01377
G1 X161.494 Y83.631 E0.38056
G1 X162.094 Y83.631 E0.01827
G1 X153.049 Y92.677 E0.38938
G1 X153.419 Y92.907 E0.01327
G1 X162.694 Y83.632 E0.39924
G1 X162.694 Y84.232 E0.01827
G1 X153.778 Y93.148 E0.38382
G1 X154.110 Y93.416 E0.01299
```

```
G1 X162.694 Y84.832 E0.36951
G1 X162.694 Y85.432 E0.01827
G1 X154.435 Y93.692 E0.35554
G1 X154.730 Y93.997 E0.01292
G1 X162.694 Y86.033 E0.34283
G1 X162.694 Y86.633 E0.01827
G1 X155.022 Y94.305 E0.33026
G1 X155.280 Y94.647 E0.01305
G1 X162.694 Y87.233 E0.31916
G1 X162.694 Y87.833 E0.01827
G1 X155.538 Y94.990 E0.30805
G1 X155.758 Y95.370 E0.01337
G1 X162.694 Y88.433 E0.29859
G1 X162.694 Y89.034 E0.01827
G1 X155.977 Y95.751 E0.28914
G1 X156.158 Y96.170 E0.01390
G1 X162.694 Y89.634 E0.28136
G1 X162.694 Y90.234 E0.01827
G1 X156.336 Y96.592 E0.27370
G1 X156.373 Y96.719 E0.00402
G1 X156.810 Y96.719 E0.01328
G1 X162.694 Y90.834 E0.25331
G1 X162.694 Y91.435 E0.01827
G1 X157.410 Y96.719 E0.22747
G1 X158.010 Y96.719 E0.01827
G1 X162.694 Y92.035 E0.20163
G1 X162.694 Y92.635 E0.01827
G1 X158.610 Y96.719 E0.17580
G1 X159.210 Y96.719 E0.01827
```

```
G1 X162.694 Y93.235 E0.14996
G1 X162.694 Y93.835 E0.01827
G1 X159.811 Y96.719 E0.12412
G1 X160.411 Y96.719 E0.01827
G1 X162.694 Y94.436 E0.09828
G1 X162.694 Y95.036 E0.01827
G1 X161.011 Y96.719 E0.07245
G1 X161.611 Y96.719 E0.01827
G1 X162.694 Y95.636 E0.04661
G1 X162.694 Y96.236 E0.01827
G1 X162.023 Y96.907 E0.02887
M204 S1250
; stop printing object Petg print.STL id:20 copy 0
; printing object Petg print.STL id:22 copy 0
G1 E-4.00000 F2400.000
G1 X163.014 Y104.133 F9000.000
G1 E4.00000 F900.000
M204 S800
;TYPE:Perimeter
;WIDTH:0.45
G1 F1292.454
G1 X163.014 Y117.848 E0.41094
G1 X156.143 Y117.848 E0.20589
G1 X156.052 Y117.524 E0.01007
G1 X155.717 Y116.730 E0.02583
G1 X155.286 Y115.983 E0.02583
G1 X154.768 Y115.295 E0.02583
G1 X154.168 Y114.675 E0.02583
G1 X153.497 Y114.135 E0.02583
```

```
G1 X152.764 Y113.680 E0.02583
G1 X151.981 Y113.320 E0.02583
G1 X151.160 Y113.058 E0.02583
G1 X150.313 Y112.900 E0.02583
G1 X149.472 Y112.848 E0.02523
G1 X141.300 Y112.848 E0.24487
M73 P64 R25
G1 X141.300 Y109.133 E0.11130
G1 X149.472 Y109.133 E0.24487
G1 X150.313 Y109.082 E0.02523
G1 X151.160 Y108.923 E0.02583
G1 X151.981 Y108.661 E0.02583
G1 X152.764 Y108.301 E0.02583
G1 X153.497 Y107.847 E0.02583
G1 X154.168 Y107.306 E0.02583
G1 X154.768 Y106.686 E0.02583
G1 X155.286 Y105.998 E0.02583
G1 X155.717 Y105.251 E0.02583
G1 X156.052 Y104.457 E0.02583
G1 X156.143 Y104.133 E0.01007
G1 X162.954 Y104.133 E0.20409
M204 S1250
G1 X163.432 Y103.716 F9000.000
M204 S800
;TYPE:External perimeter
G1 F1292.454
G1 X163.432 Y118.266 E0.43598
G1 X155.827 Y118.266 E0.22787
G1 X155.657 Y117.663 E0.01877
```

```
G1 X155.342 Y116.916 E0.02428
G1 X154.937 Y116.214 E0.02428
G1 X154.449 Y115.567 E0.02428
G1 X153.886 Y114.985 E0.02428
G1 X153.255 Y114.476 E0.02428
G1 X152.566 Y114.049 E0.02428
G1 X151.830 Y113.710 E0.02428
G1 X151.058 Y113.464 E0.02428
G1 X150.261 Y113.315 E0.02428
G1 X149.459 Y113.266 E0.02407
G1 X140.882 Y113.266 E0.25701
G1 X140.882 Y108.716 E0.13634
G1 X149.459 Y108.716 E0.25701
G1 X150.261 Y108.666 E0.02407
G1 X151.058 Y108.517 E0.02428
M73 P64 R24
G1 X151.830 Y108.271 E0.02428
G1 X152.566 Y107.932 E0.02428
G1 X153.255 Y107.505 E0.02428
G1 X153.886 Y106.997 E0.02428
G1 X154.449 Y106.414 E0.02428
G1 X154.937 Y105.767 E0.02428
G1 X155.342 Y105.065 E0.02428
G1 X155.657 Y104.318 E0.02428
G1 X155.827 Y103.716 E0.01877
G1 X163.372 Y103.716 E0.22608
M204 S1250
G1 X163.241 Y104.067 F9000.000
G1 E-4.00000 F2400.000
```

```
G1 X155.815 Y105.331 F9000.000
G1 E4.00000 F900.000
M204 S1000
;TYPE:Solid infill
;WIDTH:0.456608
G1 F1272.331
G1 X156.699 Y104.447 E0.03805
G1 X157.299 Y104.447 E0.01827
G1 X149.212 Y112.534 E0.34814
G1 X149.793 Y112.554 E0.01769
G1 X157.900 Y104.447 E0.34897
G1 X158.500 Y104.447 E0.01827
G1 X150.357 Y112.589 E0.35050
G1 X150.863 Y112.684 E0.01566
G1 X159.100 Y104.447 E0.35457
G1 X159.700 Y104.447 E0.01827
G1 X151.355 Y112.792 E0.35921
G1 X151.811 Y112.937 E0.01454
G1 X160.301 Y104.447 E0.36545
G1 X160.901 Y104.447 E0.01827
G1 X152.249 Y113.098 E0.37241
G1 X152.660 Y113.288 E0.01377
G1 X161.501 Y104.447 E0.38056
G1 X162.101 Y104.447 E0.01827
G1 X153.056 Y113.492 E0.38938
G1 X153.426 Y113.722 E0.01327
G1 X162.701 Y104.447 E0.39924
G1 X162.701 Y105.047 E0.01827
G1 X153.784 Y113.964 E0.38382
```

```
G1 X154.117 Y114.232 E0.01299
G1 X162.701 Y105.648 E0.36951
G1 X162.701 Y106.248 E0.01827
G1 X154.441 Y114.507 E0.35554
G1 X154.737 Y114.812 E0.01292
G1 X162.701 Y106.848 E0.34283
G1 X162.701 Y107.448 E0.01827
G1 X155.029 Y115.121 E0.33026
G1 X155.287 Y115.463 E0.01305
G1 X162.701 Y108.049 E0.31916
G1 X162.701 Y108.649 E0.01827
G1 X155.545 Y115.805 E0.30805
G1 X155.764 Y116.185 E0.01337
G1 X162.701 Y109.249 E0.29859
G1 X162.701 Y109.849 E0.01827
G1 X155.984 Y116.566 E0.28914
G1 X156.165 Y116.986 E0.01390
G1 X162.701 Y110.449 E0.28136
G1 X162.701 Y111.050 E0.01827
G1 X156.343 Y117.408 E0.27370
G1 X156.380 Y117.534 E0.00402
G1 X156.816 Y117.534 E0.01328
G1 X162.701 Y111.650 E0.25331
G1 X162.701 Y112.250 E0.01827
G1 X157.417 Y117.534 E0.22747
G1 X158.017 Y117.534 E0.01827
G1 X162.701 Y112.850 E0.20163
G1 X162.701 Y113.451 E0.01827
G1 X158.617 Y117.534 E0.17580
```

```
G1 X159.217 Y117.534 E0.01827
G1 X162.701 Y114.051 E0.14996
G1 X162.701 Y114.651 E0.01827
G1 X159.817 Y117.534 E0.12412
G1 X160.418 Y117.534 E0.01827
G1 X162.701 Y115.251 E0.09828
G1 X162.701 Y115.851 E0.01827
G1 X161.018 Y117.534 E0.07245
G1 X161.618 Y117.534 E0.01827
G1 X162.701 Y116.452 E0.04661
G1 X162.701 Y117.052 E0.01827
G1 X162.030 Y117.722 E0.02887
M204 S1250
G1 E-4.00000 F2400.000
G1 X148.424 Y112.722 F9000.000
G1 E4.00000 F900.000
M204 S1000
G1 F1272.331
G1 X152.275 Y108.871 E0.16578
G1 X152.095 Y108.954 E0.00604
G1 X151.357 Y109.189 E0.02358
G1 X148.011 Y112.534 E0.14400
G1 X147.411 Y112.534 E0.01827
G1 X150.599 Y109.347 E0.13722
G1 X150.351 Y109.393 E0.00767
G1 X149.926 Y109.419 E0.01297
G1 X146.811 Y112.534 E0.13409
G1 X146.211 Y112.534 E0.01827
G1 X149.298 Y109.447 E0.13291
```

G1 X148.698 Y109.447 E0.01827

G1 X145.610 Y112.534 E0.13291

G1 X145.010 Y112.534 E0.01827

G1 X148.098 Y109.447 E0.13291

G1 X147.498 Y109.447 E0.01827

G1 X144.410 Y112.534 E0.13291

G1 X143.810 Y112.534 E0.01827

G1 X146.898 Y109.447 E0.13291

G1 X146.297 Y109.447 E0.01827

G1 X143.210 Y112.534 E0.13291

G1 X142.609 Y112.534 E0.01827

G1 X145.697 Y109.447 E0.13291

G1 X145.097 Y109.447 E0.01827

G1 X142.009 Y112.534 E0.13291

G1 X141.613 Y112.534 E0.01205

G1 X141.613 Y112.330 E0.00622

G1 X144.497 Y109.447 E0.12412

G1 X143.896 Y109.447 E0.01827

G1 X141.613 Y111.730 E0.09828

G1 X141.613 Y111.130 E0.01827

G1 X143.296 Y109.447 E0.07244

G1 X142.696 Y109.447 E0.01827

G1 X141.613 Y110.530 E0.04661

G1 X141.613 Y109.929 E0.01827

G1 X142.284 Y109.259 E0.02886

M204 S1250

; stop printing object Petg print.STL id:22 copy 0

; printing object Petg print.STL id:26 copy 0

G1 E-4.00000 F2400.000

```
G1 X141.285 Y127.787 F9000.000
G1 E4.00000 F900.000
M204 S800
;TYPE:Perimeter
;WIDTH:0.45
G1 F1292.454
G1 X149.457 Y127.787 E0.24487
G1 X150.297 Y127.735 E0.02523
G1 X151.145 Y127.577 E0.02583
G1 X151.966 Y127.315 E0.02583
G1 X152.749 Y126.955 E0.02583
G1 X153.482 Y126.501 E0.02583
G1 X154.153 Y125.960 E0.02583
G1 X154.752 Y125.340 E0.02583
G1 X155.271 Y124.652 E0.02583
G1 X155.701 Y123.905 E0.02583
G1 X156.036 Y123.111 E0.02583
G1 X156.128 Y122.787 E0.01007
G1 X162.999 Y122.787 E0.20589
G1 X162.999 Y136.502 E0.41094
G1 X156.128 Y136.502 E0.20589
G1 X156.036 Y136.178 E0.01007
G1 X155.701 Y135.384 E0.02583
G1 X155.271 Y134.637 E0.02583
G1 X154.752 Y133.949 E0.02583
G1 X154.153 Y133.329 E0.02583
G1 X153.482 Y132.789 E0.02583
G1 X152.749 Y132.334 E0.02583
G1 X151.966 Y131.974 E0.02583
```

```
G1 X151.145 Y131.712 E0.02583
G1 X150.297 Y131.554 E0.02583
G1 X149.457 Y131.502 E0.02523
G1 X141.285 Y131.502 E0.24487
G1 X141.285 Y127.847 E0.10950
M204 S1250
G1 X140.867 Y127.370 F9000.000
M204 S800
;TYPE:External perimeter
G1 F1292.454
G1 X149.444 Y127.370 E0.25701
G1 X150.246 Y127.320 E0.02407
G1 X151.043 Y127.171 E0.02428
G1 X151.815 Y126.925 E0.02428
G1 X152.551 Y126.586 E0.02428
G1 X153.240 Y126.159 E0.02428
G1 X153.871 Y125.651 E0.02428
G1 X154.434 Y125.068 E0.02428
G1 X154.922 Y124.421 E0.02428
G1 X155.326 Y123.719 E0.02428
G1 X155.641 Y122.972 E0.02428
G1 X155.812 Y122.370 E0.01877
G1 X163.417 Y122.370 E0.22787
G1 X163.417 Y136.920 E0.43598
G1 X155.812 Y136.920 E0.22787
G1 X155.641 Y136.317 E0.01877
G1 X155.326 Y135.570 E0.02428
G1 X154.922 Y134.868 E0.02428
G1 X154.434 Y134.221 E0.02428
```

```
G1 X153.871 Y133.639 E0.02428
G1 X153.240 Y133.130 E0.02428
G1 X152.551 Y132.703 E0.02428
G1 X151.815 Y132.364 E0.02428
G1 X151.043 Y132.118 E0.02428
G1 X150.246 Y131.969 E0.02428
G1 X149.444 Y131.920 E0.02407
G1 X140.867 Y131.920 E0.25701
G1 X140.867 Y127.430 E0.13454
M204 S1250
G1 X141.262 Y127.434 F9000.000
G1 X142.269 Y127.913
M204 S1000
;TYPE:Solid infill
;WIDTH:0.456608
G1 F1272.331
G1 X141.598 Y128.583 E0.02886
G1 X141.598 Y129.183 E0.01827
G1 X142.681 Y128.101 E0.04661
G1 X143.281 Y128.101 E0.01827
G1 X141.598 Y129.784 E0.07244
G1 X141.598 Y130.384 E0.01827
G1 X143.881 Y128.101 E0.09828
G1 X144.482 Y128.101 E0.01827
G1 X141.598 Y130.984 E0.12412
G1 X141.598 Y131.188 E0.00622
G1 X141.994 Y131.188 E0.01205
G1 X145.082 Y128.101 E0.13291
G1 X145.682 Y128.101 E0.01827
```

```
G1 X142.594 Y131.188 E0.13291
G1 X143.195 Y131.188 E0.01827
G1 X146.282 Y128.101 E0.13291
G1 X146.882 Y128.101 E0.01827
G1 X143.795 Y131.188 E0.13291
G1 X144.395 Y131.188 E0.01827
G1 X147.483 Y128.101 E0.13291
G1 X148.083 Y128.101 E0.01827
G1 X144.995 Y131.188 E0.13291
G1 X145.595 Y131.188 E0.01827
G1 X148.683 Y128.101 E0.13291
G1 X149.283 Y128.101 E0.01827
G1 X146.196 Y131.188 E0.13291
G1 X146.796 Y131.188 E0.01827
G1 X149.911 Y128.073 E0.13409
G1 X150.336 Y128.047 E0.01297
G1 X150.584 Y128.001 E0.00767
G1 X147.396 Y131.188 E0.13722
G1 X147.996 Y131.188 E0.01827
G1 X151.342 Y127.843 E0.14400
G1 X152.080 Y127.608 E0.02358
G1 X152.260 Y127.525 E0.00604
G1 X148.408 Y131.376 E0.16578
M204 S1250
G1 E-4.00000 F2400.000
G1 X155.800 Y123.985 F9000.000
G1 E4.00000 F900.000
M204 S1000
G1 F1272.331
```

```
G1 X156.684 Y123.101 E0.03805
G1 X157.284 Y123.101 E0.01827
G1 X149.197 Y131.188 E0.34814
G1 X149.778 Y131.208 E0.01769
G1 X157.885 Y123.101 E0.34897
G1 X158.485 Y123.101 E0.01827
G1 X150.342 Y131.243 E0.35050
G1 X150.848 Y131.338 E0.01566
G1 X159.085 Y123.101 E0.35457
G1 X159.685 Y123.101 E0.01827
G1 X151.340 Y131.446 E0.35921
G1 X151.796 Y131.591 E0.01454
G1 X160.285 Y123.101 E0.36545
G1 X160.886 Y123.101 E0.01827
G1 X152.234 Y131.752 E0.37241
G1 X152.645 Y131.941 E0.01377
G1 X161.486 Y123.101 E0.38056
G1 X162.086 Y123.101 E0.01827
G1 X153.040 Y132.146 E0.38938
M73 P65 R24
G1 X153.411 Y132.376 E0.01327
G1 X162.686 Y123.101 E0.39924
G1 X162.686 Y123.701 E0.01827
G1 X153.769 Y132.618 E0.38382
G1 X154.102 Y132.886 E0.01299
G1 X162.686 Y124.302 E0.36951
G1 X162.686 Y124.902 E0.01827
G1 X154.426 Y133.161 E0.35554
G1 X154.722 Y133.466 E0.01292
```

G1 X162.686 Y125.502 E0.34283

G1 X162.686 Y126.102 E0.01827

G1 X155.014 Y133.775 E0.33026

G1 X155.271 Y134.117 E0.01305

G1 X162.686 Y126.703 E0.31916

G1 X162.686 Y127.303 E0.01827

G1 X155.529 Y134.459 E0.30805

G1 X155.749 Y134.839 E0.01337

G1 X162.686 Y127.903 E0.29859

G1 X162.686 Y128.503 E0.01827

G1 X155.969 Y135.220 E0.28914

G1 X156.149 Y135.640 E0.01390

G1 X162.686 Y129.103 E0.28136

G1 X162.686 Y129.704 E0.01827

G1 X156.328 Y136.062 E0.27370

G1 X156.365 Y136.188 E0.00402

G1 X156.801 Y136.188 E0.01328

G1 X162.686 Y130.304 E0.25331

G1 X162.686 Y130.904 E0.01827

G1 X157.401 Y136.188 E0.22747

G1 X158.002 Y136.188 E0.01827

G1 X162.686 Y131.504 E0.20163

G1 X162.686 Y132.104 E0.01827

G1 X158.602 Y136.188 E0.17580

G1 X159.202 Y136.188 E0.01827

G1 X162.686 Y132.705 E0.14996

G1 X162.686 Y133.305 E0.01827

G1 X159.802 Y136.188 E0.12412

G1 X160.403 Y136.188 E0.01827

```
G1 X162.686 Y133.905 E0.09828
G1 X162.686 Y134.505 E0.01827
G1 X161.003 Y136.188 E0.07245
G1 X161.603 Y136.188 E0.01827
G1 X162.686 Y135.106 E0.04661
G1 X162.686 Y135.706 E0.01827
G1 X162.015 Y136.376 E0.02887
M204 S1250
; stop printing object Petg print.STL id:26 copy 0
; printing object Petg print.STL id:0 copy 0
G1 E-4.00000 F2400.000
G1 X111.357 Y96.857 F9000.000
G1 E4.00000 F900.000
M204 S800
;TYPE:Perimeter
;WIDTH:0.45
G1 F1292.454
G1 X104.486 Y96.857 E0.20589
G1 X104.394 Y96.534 E0.01007
G1 X104.059 Y95.739 E0.02583
G1 X103.629 Y94.993 E0.02583
G1 X103.110 Y94.304 E0.02583
G1 X102.511 Y93.685 E0.02583
G1 X101.840 Y93.144 E0.02583
G1 X101.107 Y92.690 E0.02583
G1 X100.324 Y92.329 E0.02583
G1 X99.503 Y92.068 E0.02583
G1 X98.656 Y91.909 E0.02583
G1 X97.815 Y91.857 E0.02523
```

```
G1 X89.643 Y91.857 E0.24487
G1 X89.643 Y88.143 E0.11130
G1 X97.815 Y88.143 E0.24487
G1 X98.656 Y88.091 E0.02523
G1 X99.503 Y87.932 E0.02583
G1 X100.324 Y87.671 E0.02583
G1 X101.107 Y87.310 E0.02583
G1 X101.840 Y86.856 E0.02583
G1 X102.511 Y86.315 E0.02583
G1 X103.110 Y85.696 E0.02583
G1 X103.629 Y85.007 E0.02583
G1 X104.059 Y84.261 E0.02583
G1 X104.394 Y83.466 E0.02583
G1 X104.486 Y83.143 E0.01007
G1 X111.357 Y83.143 E0.20589
G1 X111.357 Y96.797 E0.40914
M204 S1250
G1 X111.775 Y97.275 F9000.000
M204 S800
;TYPE:External perimeter
G1 F1292.454
G1 X104.170 Y97.275 E0.22787
G1 X104.000 Y96.672 E0.01877
G1 X103.685 Y95.926 E0.02428
G1 X103.280 Y95.223 E0.02428
G1 X102.792 Y94.576 E0.02428
G1 X102.229 Y93.994 E0.02428
G1 X101.598 Y93.486 E0.02428
G1 X100.909 Y93.059 E0.02428
```

```
G1 X100.173 Y92.719 E0.02428
G1 X99.401 Y92.474 E0.02428
G1 X98.604 Y92.325 E0.02428
G1 X97.802 Y92.275 E0.02407
G1 X89.225 Y92.275 E0.25701
G1 X89.225 Y87.725 E0.13634
G1 X97.802 Y87.725 E0.25701
G1 X98.604 Y87.675 E0.02407
G1 X99.401 Y87.526 E0.02428
G1 X100.173 Y87.281 E0.02428
G1 X100.909 Y86.941 E0.02428
G1 X101.598 Y86.514 E0.02428
G1 X102.229 Y86.006 E0.02428
G1 X102.792 Y85.424 E0.02428
G1 X103.280 Y84.777 E0.02428
G1 X103.685 Y84.074 E0.02428
G1 X104.000 Y83.328 E0.02428
G1 X104.170 Y82.725 E0.01877
G1 X111.775 Y82.725 E0.22787
G1 X111.775 Y97.215 E0.43418
M204 S1250
G1 X111.401 Y97.133 F9000.000
G1 X110.373 Y96.732
M204 S1000
;TYPE:Solid infill
;WIDTH:0.456608
G1 F1272.331
G1 X111.044 Y96.061 E0.02887
G1 X111.044 Y95.461 E0.01827
```

```
G1 X109.961 Y96.544 E0.04661
G1 X109.361 Y96.544 E0.01827
G1 X111.044 Y94.861 E0.07245
G1 X111.044 Y94.261 E0.01827
G1 X108.761 Y96.544 E0.09828
G1 X108.160 Y96.544 E0.01827
G1 X111.044 Y93.660 E0.12412
G1 X111.044 Y93.060 E0.01827
G1 X107.560 Y96.544 E0.14996
G1 X106.960 Y96.544 E0.01827
G1 X111.044 Y92.460 E0.17580
G1 X111.044 Y91.860 E0.01827
G1 X106.360 Y96.544 E0.20163
G1 X105.760 Y96.544 E0.01827
G1 X111.044 Y91.260 E0.22747
G1 X111.044 Y90.659 E0.01827
G1 X105.159 Y96.544 E0.25331
G1 X104.723 Y96.544 E0.01328
G1 X104.686 Y96.417 E0.00402
G1 X111.044 Y90.059 E0.27370
G1 X111.044 Y89.459 E0.01827
G1 X104.507 Y95.995 E0.28136
G1 X104.327 Y95.576 E0.01390
G1 X111.044 Y88.859 E0.28914
G1 X111.044 Y88.258 E0.01827
G1 X104.107 Y95.195 E0.29859
G1 X103.887 Y94.815 E0.01337
G1 X111.044 Y87.658 E0.30805
G1 X111.044 Y87.058 E0.01827
```

G1 X103.630 Y94.472 E0.31916

G1 X103.372 Y94.130 E0.01305

G1 X111.044 Y86.458 E0.33026

G1 X111.044 Y85.858 E0.01827

G1 X103.080 Y93.822 E0.34283

M73 P65 R23

G1 X102.784 Y93.517 E0.01292

G1 X111.044 Y85.257 E0.35554

G1 X111.044 Y84.657 E0.01827

G1 X102.460 Y93.241 E0.36951

G1 X102.127 Y92.973 E0.01299

G1 X111.044 Y84.057 E0.38382

G1 X111.044 Y83.457 E0.01827

G1 X101.769 Y92.732 E0.39924

G1 X101.398 Y92.502 E0.01327

G1 X110.444 Y83.456 E0.38938

G1 X109.844 Y83.456 E0.01827

G1 X101.003 Y92.297 E0.38056

G1 X100.592 Y92.108 E0.01377

G1 X109.244 Y83.456 E0.37241

G1 X108.643 Y83.456 E0.01827

G1 X100.154 Y91.946 E0.36545

G1 X99.698 Y91.801 E0.01454

G1 X108.043 Y83.456 E0.35921

G1 X107.443 Y83.456 E0.01827

G1 X99.206 Y91.693 E0.35457

G1 X98.700 Y91.599 E0.01566

G1 X106.843 Y83.456 E0.35050

G1 X106.243 Y83.456 E0.01827

```
G1 X98.136 Y91.563 E0.34897
G1 X97.555 Y91.544 E0.01769
G1 X105.642 Y83.456 E0.34814
G1 X105.042 Y83.456 E0.01827
G1 X104.158 Y84.340 E0.03805
M204 S1250
G1 E-4.00000 F2400.000
G1 X96.766 Y91.732 F9000.000
G1 E4.00000 F900.000
M204 S1000
G1 F1272.331
G1 X100.618 Y87.881 E0.16578
G1 X100.438 Y87.964 E0.00604
G1 X99.700 Y88.199 E0.02358
G1 X96.354 Y91.544 E0.14400
G1 X95.754 Y91.544 E0.01827
G1 X98.942 Y88.356 E0.13722
G1 X98.694 Y88.402 E0.00767
G1 X98.269 Y88.429 E0.01297
G1 X95.154 Y91.544 E0.13409
G1 X94.554 Y91.544 E0.01827
G1 X97.641 Y88.456 E0.13291
G1 X97.041 Y88.456 E0.01827
G1 X93.953 Y91.544 E0.13291
G1 X93.353 Y91.544 E0.01827
G1 X96.441 Y88.456 E0.13291
G1 X95.841 Y88.456 E0.01827
G1 X92.753 Y91.544 E0.13291
G1 X92.153 Y91.544 E0.01827
```

G1 X95.240 Y88.456 E0.13291

G1 X94.640 Y88.456 E0.01827

G1 X91.553 Y91.544 E0.13291

G1 X90.952 Y91.544 E0.01827

G1 X94.040 Y88.456 E0.13291

G1 X93.440 Y88.456 E0.01827

G1 X90.352 Y91.544 E0.13291

G1 X89.956 Y91.544 E0.01205

G1 X89.956 Y91.340 E0.00622

G1 X92.840 Y88.456 E0.12412

G1 X92.239 Y88.456 E0.01827

G1 X89.956 Y90.739 E0.09828

G1 X89.956 Y90.139 E0.01827

G1 X91.639 Y88.456 E0.07244

G1 X91.039 Y88.456 E0.01827

G1 X89.956 Y89.539 E0.04661

G1 X89.956 Y88.939 E0.01827

G1 X90.627 Y88.268 E0.02886

M204 S1250

; stop printing object Petg print.STL id:0 copy 0

; printing object tpu print.STL id:1 copy 0

; stop printing object tpu print.STL id:1 copy 0

; printing object tpu print.STL id:5 copy 0

; stop printing object tpu print.STL id:5 copy 0

; printing object tpu print.STL id:9 copy 0

; stop printing object tpu print.STL id:9 copy 0

; printing object Petg print.STL id:8 copy 0

G1 E-4.00000 F2400.000

G1 X104.530 Y54.716 F9000.000

```
G1 E4.00000 F900.000
M204 S800
;TYPE:Perimeter
;WIDTH:0.45
G1 F1292.454
G1 X104.438 Y54.392 E0.01007
G1 X104.103 Y53.598 E0.02583
G1 X103.673 Y52.851 E0.02583
G1 X103.154 Y52.163 E0.02583
G1 X102.555 Y51.544 E0.02583
G1 X101.883 Y51.003 E0.02583
G1 X101.151 Y50.549 E0.02583
G1 X100.368 Y50.188 E0.02583
G1 X99.546 Y49.926 E0.02583
G1 X98.699 Y49.768 E0.02583
G1 X97.859 Y49.716 E0.02523
G1 X89.686 Y49.716 E0.24487
G1 X89.686 Y46.002 E0.11130
G1 X97.859 Y46.002 E0.24487
G1 X98.699 Y45.950 E0.02523
G1 X99.546 Y45.791 E0.02583
G1 X100.368 Y45.530 E0.02583
G1 X101.151 Y45.169 E0.02583
G1 X101.883 Y44.715 E0.02583
G1 X102.555 Y44.174 E0.02583
G1 X103.154 Y43.555 E0.02583
G1 X103.673 Y42.866 E0.02583
G1 X104.103 Y42.119 E0.02583
G1 X104.438 Y41.325 E0.02583
```

```
G1 X104.530 Y41.002 E0.01007
G1 X111.401 Y41.002 E0.20589
G1 X111.401 Y54.716 E0.41094
G1 X104.590 Y54.716 E0.20409
M204 S1250
G1 X104.214 Y55.134 F9000.000
M204 S800
;TYPE:External perimeter
G1 F1292.454
G1 X104.043 Y54.531 E0.01877
G1 X103.728 Y53.784 E0.02428
G1 X103.324 Y53.082 E0.02428
G1 X102.836 Y52.435 E0.02428
G1 X102.272 Y51.853 E0.02428
G1 X101.641 Y51.344 E0.02428
G1 X100.952 Y50.917 E0.02428
G1 X100.216 Y50.578 E0.02428
G1 X99.444 Y50.332 E0.02428
G1 X98.648 Y50.183 E0.02428
G1 X97.846 Y50.134 E0.02407
G1 X89.269 Y50.134 E0.25701
G1 X89.269 Y45.584 E0.13634
G1 X97.846 Y45.584 E0.25701
G1 X98.648 Y45.534 E0.02407
M73 P66 R23
G1 X99.444 Y45.385 E0.02428
G1 X100.216 Y45.139 E0.02428
G1 X100.952 Y44.800 E0.02428
G1 X101.641 Y44.373 E0.02428
```

G1 X102.272 Y43.865 E0.02428

G1 X102.836 Y43.282 E0.02428

G1 X103.324 Y42.635 E0.02428

G1 X103.728 Y41.933 E0.02428

G1 X104.043 Y41.186 E0.02428

G1 X104.214 Y40.584 E0.01877

G1 X111.819 Y40.584 E0.22787

G1 X111.819 Y55.134 E0.43598

G1 X104.274 Y55.134 E0.22608

M204 S1250

G1 X104.204 Y54.734 F9000.000

G1 X110.417 Y54.591

M204 S1000

;TYPE:Solid infill

;WIDTH:0.456608

G1 F1272.331

G1 X111.087 Y53.920 E0.02887

G1 X111.087 Y53.320 E0.01827

G1 X110.005 Y54.403 E0.04661

G1 X109.404 Y54.403 E0.01827

G1 X111.087 Y52.720 E0.07245

G1 X111.087 Y52.119 E0.01827

G1 X108.804 Y54.403 E0.09828

G1 X108.204 Y54.403 E0.01827

G1 X111.087 Y51.519 E0.12412

G1 X111.087 Y50.919 E0.01827

G1 X107.604 Y54.403 E0.14996

G1 X107.004 Y54.403 E0.01827

G1 X111.087 Y50.319 E0.17580

G1 X111.087 Y49.718 E0.01827

G1 X106.403 Y54.403 E0.20163

G1 X105.803 Y54.403 E0.01827

G1 X111.087 Y49.118 E0.22747

G1 X111.087 Y48.518 E0.01827

G1 X105.203 Y54.403 E0.25331

G1 X104.767 Y54.403 E0.01328

G1 X104.729 Y54.276 E0.00402

G1 X111.087 Y47.918 E0.27370

G1 X111.087 Y47.318 E0.01827

G1 X104.551 Y53.854 E0.28136

G1 X104.370 Y53.434 E0.01390

G1 X111.087 Y46.717 E0.28914

G1 X111.087 Y46.117 E0.01827

G1 X104.151 Y53.054 E0.29859

G1 X103.931 Y52.673 E0.01337

G1 X111.087 Y45.517 E0.30805

G1 X111.087 Y44.917 E0.01827

G1 X103.673 Y52.331 E0.31916

G1 X103.415 Y51.989 E0.01305

G1 X111.087 Y44.316 E0.33026

G1 X111.087 Y43.716 E0.01827

G1 X103.123 Y51.681 E0.34283

G1 X102.828 Y51.376 E0.01292

G1 X111.087 Y43.116 E0.35554

G1 X111.087 Y42.516 E0.01827

G1 X102.503 Y51.100 E0.36951

G1 X102.171 Y50.832 E0.01299

G1 X111.087 Y41.916 E0.38382

```
G1 X111.087 Y41.315 E0.01827
G1 X101.813 Y50.590 E0.39924
G1 X101.442 Y50.361 E0.01327
G1 X110.488 Y41.315 E0.38938
G1 X109.888 Y41.315 E0.01827
G1 X101.047 Y50.156 E0.38056
G1 X100.636 Y49.966 E0.01377
G1 X109.287 Y41.315 E0.37241
G1 X108.687 Y41.315 E0.01827
G1 X100.197 Y49.805 E0.36545
G1 X99.742 Y49.660 E0.01454
G1 X108.087 Y41.315 E0.35921
G1 X107.487 Y41.315 E0.01827
G1 X99.250 Y49.552 E0.35457
G1 X98.744 Y49.457 E0.01566
G1 X106.886 Y41.315 E0.35050
G1 X106.286 Y41.315 E0.01827
G1 X98.179 Y49.422 E0.34897
G1 X97.598 Y49.403 E0.01769
G1 X105.686 Y41.315 E0.34814
G1 X105.086 Y41.315 E0.01827
G1 X104.202 Y42.199 E0.03805
M204 S1250
G1 E-4.00000 F2400.000
G1 X96.810 Y49.591 F9000.000
G1 E4.00000 F900.000
M204 S1000
G1 F1272.331
G1 X100.661 Y45.739 E0.16578
```

G1 X100.481 Y45.822 E0.00604

G1 X99.743 Y46.057 E0.02358

G1 X96.398 Y49.403 E0.14400

G1 X95.798 Y49.403 E0.01827

G1 X98.985 Y46.215 E0.13722

G1 X98.738 Y46.261 E0.00767

G1 X98.313 Y46.287 E0.01297

G1 X95.197 Y49.403 E0.13409

G1 X94.597 Y49.403 E0.01827

G1 X97.685 Y46.315 E0.13291

G1 X97.085 Y46.315 E0.01827

G1 X93.997 Y49.403 E0.13291

G1 X93.397 Y49.403 E0.01827

G1 X96.485 Y46.315 E0.13291

G1 X95.884 Y46.315 E0.01827

G1 X92.797 Y49.403 E0.13291

G1 X92.196 Y49.403 E0.01827

G1 X95.284 Y46.315 E0.13291

G1 X94.684 Y46.315 E0.01827

G1 X91.596 Y49.403 E0.13291

G1 X90.996 Y49.403 E0.01827

G1 X94.084 Y46.315 E0.13291

G1 X93.483 Y46.315 E0.01827

G1 X90.396 Y49.403 E0.13291

G1 X90.000 Y49.403 E0.01205

G1 X90.000 Y49.198 E0.00622

G1 X92.883 Y46.315 E0.12412

G1 X92.283 Y46.315 E0.01827

G1 X90.000 Y48.598 E0.09828

```
G1 X90.000 Y47.998 E0.01827
G1 X91.683 Y46.315 E0.07244
G1 X91.083 Y46.315 E0.01827
G1 X90.000 Y47.398 E0.04661
G1 X90.000 Y46.797 E0.01827
G1 X90.670 Y46.127 E0.02886
M204 S1250
; stop printing object Petg print.STL id:8 copy 0
; printing object Petg print.STL id:4 copy 0
G1 E-4.00000 F2400.000
G1 X89.694 Y65.950 F9000.000
G1 E4.00000 F900.000
M204 S800
;TYPE:Perimeter
;WIDTH:0.45
G1 F1292.454
G1 X97.866 Y65.950 E0.24487
G1 X98.707 Y65.899 E0.02523
G1 X99.554 Y65.740 E0.02583
G1 X100.375 Y65.478 E0.02583
G1 X101.158 Y65.118 E0.02583
G1 X101.891 Y64.664 E0.02583
G1 X102.562 Y64.123 E0.02583
G1 X103.162 Y63.503 E0.02583
G1 X103.680 Y62.815 E0.02583
G1 X104.111 Y62.068 E0.02583
G1 X104.446 Y61.274 E0.02583
G1 X104.537 Y60.950 E0.01007
G1 X111.408 Y60.950 E0.20589
```

```
G1 X111.408 Y74.665 E0.41094
G1 X104.537 Y74.665 E0.20589
G1 X104.446 Y74.341 E0.01007
G1 X104.111 Y73.547 E0.02583
G1 X103.680 Y72.800 E0.02583
G1 X103.162 Y72.112 E0.02583
G1 X102.562 Y71.493 E0.02583
G1 X101.891 Y70.952 E0.02583
G1 X101.158 Y70.497 E0.02583
G1 X100.375 Y70.137 E0.02583
G1 X99.554 Y69.875 E0.02583
G1 X98.707 Y69.717 E0.02583
G1 X97.866 Y69.665 E0.02523
G1 X89.694 Y69.665 E0.24487
G1 X89.694 Y66.010 E0.10950
M204 S1250
G1 X89.276 Y65.533 F9000.000
M204 S800
;TYPE:External perimeter
G1 F1292.454
G1 X97.853 Y65.533 E0.25701
G1 X98.655 Y65.483 E0.02407
G1 X99.452 Y65.334 E0.02428
G1 X100.224 Y65.088 E0.02428
G1 X100.960 Y64.749 E0.02428
G1 X101.649 Y64.322 E0.02428
G1 X102.280 Y63.814 E0.02428
G1 X102.843 Y63.231 E0.02428
G1 X103.331 Y62.584 E0.02428
```

```
G1 X103.736 Y61.882 E0.02428
G1 X104.051 Y61.135 E0.02428
G1 X104.221 Y60.533 E0.01877
G1 X111.826 Y60.533 E0.22787
G1 X111.826 Y75.083 E0.43598
G1 X104.221 Y75.083 E0.22787
G1 X104.051 Y74.480 E0.01877
G1 X103.736 Y73.733 E0.02428
G1 X103.331 Y73.031 E0.02428
G1 X102.843 Y72.384 E0.02428
G1 X102.280 Y71.802 E0.02428
G1 X101.649 Y71.293 E0.02428
G1 X100.960 Y70.866 E0.02428
G1 X100.224 Y70.527 E0.02428
G1 X99.452 Y70.281 E0.02428
G1 X98.655 Y70.132 E0.02428
G1 X97.853 Y70.083 E0.02407
G1 X89.276 Y70.083 E0.25701
G1 X89.276 Y65.593 E0.13454
M204 S1250
G1 X89.671 Y65.597 F9000.000
G1 X90.678 Y66.076
M204 S1000
;TYPE:Solid infill
;WIDTH:0.456608
G1 F1272.331
G1 X90.007 Y66.746 E0.02886
G1 X90.007 Y67.347 E0.01827
G1 X91.090 Y66.264 E0.04661
```

```
G1 X91.690 Y66.264 E0.01827
G1 X90.007 Y67.947 E0.07244
G1 X90.007 Y68.547 E0.01827
G1 X92.290 Y66.264 E0.09828
G1 X92.891 Y66.264 E0.01827
G1 X90.007 Y69.147 E0.12412
G1 X90.007 Y69.351 E0.00622
G1 X90.403 Y69.351 E0.01205
G1 X93.491 Y66.264 E0.13291
G1 X94.091 Y66.264 E0.01827
G1 X91.003 Y69.351 E0.13291
G1 X91.604 Y69.351 E0.01827
G1 X94.691 Y66.264 E0.13291
G1 X95.292 Y66.264 E0.01827
G1 X92.204 Y69.351 E0.13291
G1 X92.804 Y69.351 E0.01827
G1 X95.892 Y66.264 E0.13291
G1 X96.492 Y66.264 E0.01827
G1 X93.404 Y69.351 E0.13291
G1 X94.005 Y69.351 E0.01827
G1 X97.092 Y66.264 E0.13291
G1 X97.692 Y66.264 E0.01827
G1 X94.605 Y69.351 E0.13291
G1 X95.205 Y69.351 E0.01827
G1 X98.320 Y66.236 E0.13409
G1 X98.745 Y66.210 E0.01297
G1 X98.993 Y66.164 E0.00767
G1 X95.805 Y69.351 E0.13722
G1 X96.405 Y69.351 E0.01827
```

```
G1 X99.751 Y66.006 E0.14400
G1 X100.489 Y65.771 E0.02358
G1 X100.669 Y65.688 E0.00604
G1 X96.818 Y69.539 E0.16578
M204 S1250
G1 E-4.00000 F2400.000
G1 X104.209 Y62.148 F9000.000
G1 E4.00000 F900.000
M204 S1000
G1 F1272.331
G1 X105.093 Y61.264 E0.03805
G1 X105.694 Y61.264 E0.01827
G1 X97.606 Y69.351 E0.34814
G1 X98.187 Y69.371 E0.01769
G1 X106.294 Y61.264 E0.34897
G1 X106.894 Y61.264 E0.01827
G1 X98.751 Y69.406 E0.35050
G1 X99.257 Y69.501 E0.01566
G1 X107.494 Y61.264 E0.35457
G1 X108.094 Y61.264 E0.01827
G1 X99.750 Y69.609 E0.35921
G1 X100.205 Y69.754 E0.01454
G1 X108.695 Y61.264 E0.36545
G1 X109.295 Y61.264 E0.01827
G1 X100.643 Y69.915 E0.37241
G1 X101.054 Y70.105 E0.01377
G1 X109.895 Y61.264 E0.38056
G1 X110.495 Y61.264 E0.01827
G1 X101.450 Y70.309 E0.38938
```

G1 X101.820 Y70.539 E0.01327

G1 X111.095 Y61.264 E0.39924

G1 X111.095 Y61.865 E0.01827

G1 X102.178 Y70.781 E0.38382

G1 X102.511 Y71.049 E0.01299

G1 X111.095 Y62.465 E0.36951

G1 X111.095 Y63.065 E0.01827

G1 X102.835 Y71.324 E0.35554

G1 X103.131 Y71.629 E0.01292

G1 X111.095 Y63.665 E0.34283

G1 X111.095 Y64.265 E0.01827

G1 X103.423 Y71.938 E0.33026

M73 P67 R23

G1 X103.681 Y72.280 E0.01305

G1 X111.095 Y64.866 E0.31916

G1 X111.095 Y65.466 E0.01827

G1 X103.939 Y72.622 E0.30805

M73 P67 R22

G1 X104.159 Y73.003 E0.01337

G1 X111.095 Y66.066 E0.29859

G1 X111.095 Y66.666 E0.01827

G1 X104.378 Y73.383 E0.28914

G1 X104.559 Y73.803 E0.01390

G1 X111.095 Y67.267 E0.28136

G1 X111.095 Y67.867 E0.01827

G1 X104.737 Y74.225 E0.27370

G1 X104.774 Y74.351 E0.00402

G1 X105.210 Y74.351 E0.01328

G1 X111.095 Y68.467 E0.25331

```
G1 X111.095 Y69.067 E0.01827
G1 X105.811 Y74.351 E0.22747
G1 X106.411 Y74.351 E0.01827
G1 X111.095 Y69.667 E0.20163
G1 X111.095 Y70.268 E0.01827
G1 X107.011 Y74.351 E0.17580
G1 X107.611 Y74.351 E0.01827
G1 X111.095 Y70.868 E0.14996
G1 X111.095 Y71.468 E0.01827
G1 X108.212 Y74.351 E0.12412
G1 X108.812 Y74.351 E0.01827
G1 X111.095 Y72.068 E0.09828
G1 X111.095 Y72.668 E0.01827
G1 X109.412 Y74.351 E0.07245
G1 X110.012 Y74.351 E0.01827
G1 X111.095 Y73.269 E0.04661
G1 X111.095 Y73.869 E0.01827
G1 X110.424 Y74.539 E0.02887
M204 S1250
; stop printing object Petg print.STL id:4 copy 0
; printing object tpu print.STL id:29 copy 0
; stop printing object tpu print.STL id:29 copy 0
; printing object tpu print.STL id:25 copy 0
; stop printing object tpu print.STL id:25 copy 0
; printing object Petg print.STL id:24 copy 0
G1 E-4.00000 F2400.000
G1 X141.344 Y69.840 F9000.000
G1 E4.00000 F900.000
M204 S800
```

```
;TYPE:Perimeter
;WIDTH:0.45
G1 F1292.454
G1 X141.344 Y66.126 E0.11130
G1 X149.516 Y66.126 E0.24487
G1 X150.357 Y66.074 E0.02523
G1 X151.204 Y65.915 E0.02583
G1 X152.026 Y65.654 E0.02583
G1 X152.808 Y65.293 E0.02583
G1 X153.541 Y64.839 E0.02583
G1 X154.212 Y64.298 E0.02583
G1 X154.812 Y63.679 E0.02583
G1 X155.331 Y62.990 E0.02583
G1 X155.761 Y62.243 E0.02583
G1 X156.096 Y61.449 E0.02583
G1 X156.187 Y61.126 E0.01007
G1 X163.059 Y61.126 E0.20589
G1 X163.059 Y74.840 E0.41094
G1 X156.187 Y74.840 E0.20589
G1 X156.096 Y74.516 E0.01007
G1 X155.761 Y73.722 E0.02583
G1 X155.331 Y72.975 E0.02583
G1 X154.812 Y72.287 E0.02583
G1 X154.212 Y71.668 E0.02583
G1 X153.541 Y71.127 E0.02583
G1 X152.808 Y70.673 E0.02583
G1 X152.026 Y70.312 E0.02583
G1 X151.204 Y70.050 E0.02583
G1 X150.357 Y69.892 E0.02583
```

```
G1 X149.516 Y69.840 E0.02523
G1 X141.404 Y69.840 E0.24307
M204 S1250
G1 X140.926 Y70.258 F9000.000
M204 S800
;TYPE:External perimeter
G1 F1292.454
G1 X140.926 Y65.708 E0.13634
G1 X149.503 Y65.708 E0.25701
G1 X150.305 Y65.658 E0.02407
G1 X151.102 Y65.509 E0.02428
G1 X151.874 Y65.263 E0.02428
G1 X152.610 Y64.924 E0.02428
G1 X153.299 Y64.497 E0.02428
G1 X153.930 Y63.989 E0.02428
G1 X154.494 Y63.406 E0.02428
G1 X154.981 Y62.759 E0.02428
G1 X155.386 Y62.057 E0.02428
G1 X155.701 Y61.310 E0.02428
G1 X155.872 Y60.708 E0.01877
G1 X163.476 Y60.708 E0.22787
G1 X163.476 Y75.258 E0.43598
G1 X155.872 Y75.258 E0.22787
G1 X155.701 Y74.655 E0.01877
G1 X155.386 Y73.908 E0.02428
G1 X154.981 Y73.206 E0.02428
G1 X154.494 Y72.559 E0.02428
G1 X153.930 Y71.977 E0.02428
G1 X153.299 Y71.468 E0.02428
```

G1 X152.610 Y71.041 E0.02428

G1 X151.874 Y70.702 E0.02428

G1 X151.102 Y70.456 E0.02428

G1 X150.305 Y70.307 E0.02428

G1 X149.503 Y70.258 E0.02407

G1 X140.986 Y70.258 E0.25521

M204 S1250

G1 X141.127 Y69.912 F9000.000

G1 X142.328 Y66.251

M204 S1000

;TYPE:Solid infill

;WIDTH:0.456608

G1 F1272.331

G1 X141.658 Y66.921 E0.02886

G1 X141.658 Y67.522 E0.01827

G1 X142.740 Y66.439 E0.04661

G1 X143.341 Y66.439 E0.01827

G1 X141.658 Y68.122 E0.07244

G1 X141.658 Y68.722 E0.01827

G1 X143.941 Y66.439 E0.09828

G1 X144.541 Y66.439 E0.01827

G1 X141.658 Y69.322 E0.12412

G1 X141.658 Y69.527 E0.00622

G1 X142.053 Y69.527 E0.01205

G1 X145.141 Y66.439 E0.13291

G1 X145.741 Y66.439 E0.01827

G1 X142.654 Y69.527 E0.13291

G1 X143.254 Y69.527 E0.01827

G1 X146.342 Y66.439 E0.13291

```
G1 X146.942 Y66.439 E0.01827
G1 X143.854 Y69.527 E0.13291
G1 X144.454 Y69.527 E0.01827
G1 X147.542 Y66.439 E0.13291
G1 X148.142 Y66.439 E0.01827
G1 X145.055 Y69.527 E0.13291
G1 X145.655 Y69.527 E0.01827
G1 X148.742 Y66.439 E0.13291
G1 X149.343 Y66.439 E0.01827
G1 X146.255 Y69.527 E0.13291
G1 X146.855 Y69.527 E0.01827
G1 X149.970 Y66.411 E0.13409
G1 X150.396 Y66.385 E0.01297
G1 X150.643 Y66.339 E0.00767
G1 X147.455 Y69.527 E0.13722
G1 X148.056 Y69.527 E0.01827
G1 X151.401 Y66.181 E0.14400
G1 X152.139 Y65.946 E0.02358
G1 X152.319 Y65.863 E0.00604
G1 X148.468 Y69.715 E0.16578
M204 S1250
G1 E-4.00000 F2400.000
G1 X155.860 Y62.323 F9000.000
G1 E4.00000 F900.000
M204 S1000
G1 F1272.331
G1 X156.744 Y61.439 E0.03805
G1 X157.344 Y61.439 E0.01827
G1 X149.256 Y69.527 E0.34814
```

```
G1 X149.837 Y69.546 E0.01769
G1 X157.944 Y61.439 E0.34897
G1 X158.544 Y61.439 E0.01827
G1 X150.402 Y69.581 E0.35050
G1 X150.907 Y69.676 E0.01566
G1 X159.144 Y61.439 E0.35457
G1 X159.745 Y61.439 E0.01827
G1 X151.400 Y69.784 E0.35921
G1 X151.855 Y69.929 E0.01454
G1 X160.345 Y61.439 E0.36545
G1 X160.945 Y61.439 E0.01827
G1 X152.294 Y70.090 E0.37241
G1 X152.705 Y70.280 E0.01377
G1 X161.545 Y61.439 E0.38056
G1 X162.146 Y61.439 E0.01827
G1 X153.100 Y70.485 E0.38938
G1 X153.470 Y70.714 E0.01327
G1 X162.745 Y61.439 E0.39924
G1 X162.745 Y62.040 E0.01827
G1 X153.829 Y70.956 E0.38382
G1 X154.161 Y71.224 E0.01299
G1 X162.745 Y62.640 E0.36951
G1 X162.745 Y63.240 E0.01827
G1 X154.486 Y71.500 E0.35554
G1 X154.781 Y71.805 E0.01292
G1 X162.745 Y63.840 E0.34283
G1 X162.745 Y64.440 E0.01827
G1 X155.073 Y72.113 E0.33026
G1 X155.331 Y72.455 E0.01305
```

```
G1 X162.745 Y65.041 E0.31916
G1 X162.745 Y65.641 E0.01827
G1 X155.589 Y72.797 E0.30805
G1 X155.809 Y73.178 E0.01337
G1 X162.745 Y66.241 E0.29859
G1 X162.745 Y66.841 E0.01827
G1 X156.028 Y73.558 E0.28914
G1 X156.209 Y73.978 E0.01390
G1 X162.745 Y67.442 E0.28136
G1 X162.745 Y68.042 E0.01827
G1 X156.387 Y74.400 E0.27370
G1 X156.424 Y74.527 E0.00402
G1 X156.861 Y74.527 E0.01328
G1 X162.745 Y68.642 E0.25331
G1 X162.745 Y69.242 E0.01827
G1 X157.461 Y74.527 E0.22747
G1 X158.061 Y74.527 E0.01827
G1 X162.745 Y69.842 E0.20163
G1 X162.745 Y70.443 E0.01827
G1 X158.661 Y74.527 E0.17580
G1 X159.262 Y74.527 E0.01827
G1 X162.745 Y71.043 E0.14996
G1 X162.745 Y71.643 E0.01827
G1 X159.862 Y74.527 E0.12412
G1 X160.462 Y74.527 E0.01827
G1 X162.745 Y72.243 E0.09828
G1 X162.745 Y72.844 E0.01827
G1 X161.062 Y74.527 E0.07245
G1 X161.662 Y74.527 E0.01827
```

```
G1 X162.745 Y73.444 E0.04661
G1 X162.745 Y74.044 E0.01827
G1 X162.075 Y74.715 E0.02887
M204 S1250
; stop printing object Petg print.STL id:24 copy 0
; printing object Petg print.STL id:28 copy 0
G1 E-4.00000 F2400.000
G1 X163.051 Y54.891 F9000.000
G1 E4.00000 F900.000
M204 S800
;TYPE:Perimeter
;WIDTH:0.45
G1 F1292.454
G1 X156.180 Y54.891 E0.20589
G1 X156.088 Y54.567 E0.01007
G1 X155.753 Y53.773 E0.02583
G1 X155.323 Y53.026 E0.02583
G1 X154.804 Y52.338 E0.02583
G1 X154.205 Y51.719 E0.02583
G1 X153.534 Y51.178 E0.02583
G1 X152.801 Y50.724 E0.02583
G1 X152.018 Y50.363 E0.02583
G1 X151.197 Y50.101 E0.02583
G1 X150.349 Y49.943 E0.02583
G1 X149.509 Y49.891 E0.02523
G1 X141.337 Y49.891 E0.24487
G1 X141.337 Y46.177 E0.11130
G1 X149.509 Y46.177 E0.24487
G1 X150.349 Y46.125 E0.02523
```

G1 X151.197 Y45.966 E0.02583

G1 X152.018 Y45.705 E0.02583

G1 X152.801 Y45.344 E0.02583

G1 X153.534 Y44.890 E0.02583

G1 X154.205 Y44.349 E0.02583

G1 X154.804 Y43.730 E0.02583

G1 X155.323 Y43.041 E0.02583

G1 X155.753 Y42.294 E0.02583

G1 X156.088 Y41.500 E0.02583

G1 X156.180 Y41.177 E0.01007

G1 X163.051 Y41.177 E0.20589

G1 X163.051 Y54.831 E0.40914

M204 S1250

G1 X163.469 Y55.309 F9000.000

M204 S800

;TYPE:External perimeter

G1 F1292.454

G1 X155.864 Y55.309 E0.22787

G1 X155.693 Y54.706 E0.01877

G1 X155.378 Y53.959 E0.02428

G1 X154.974 Y53.257 E0.02428

G1 X154.486 Y52.610 E0.02428

G1 X153.923 Y52.028 E0.02428

G1 X153.292 Y51.519 E0.02428

G1 X152.603 Y51.092 E0.02428

G1 X151.867 Y50.753 E0.02428

G1 X151.094 Y50.507 E0.02428

G1 X150.298 Y50.358 E0.02428

G1 X149.496 Y50.309 E0.02407

```
G1 X140.919 Y50.309 E0.25701
G1 X140.919 Y45.759 E0.13634
G1 X149.496 Y45.759 E0.25701
G1 X150.298 Y45.709 E0.02407
G1 X151.094 Y45.560 E0.02428
G1 X151.867 Y45.314 E0.02428
G1 X152.603 Y44.975 E0.02428
G1 X153.292 Y44.548 E0.02428
G1 X153.923 Y44.040 E0.02428
G1 X154.486 Y43.458 E0.02428
G1 X154.974 Y42.810 E0.02428
G1 X155.378 Y42.108 E0.02428
G1 X155.693 Y41.361 E0.02428
G1 X155.864 Y40.759 E0.01877
G1 X163.469 Y40.759 E0.22787
G1 X163.469 Y55.249 E0.43418
M204 S1250
G1 X163.095 Y55.167 F9000.000
G1 X162.067 Y54.766
M204 S1000
;TYPE:Solid infill
;WIDTH:0.456608
G1 F1272.331
G1 X162.738 Y54.095 E0.02887
G1 X162.738 Y53.495 E0.01827
G1 X161.655 Y54.578 E0.04661
G1 X161.055 Y54.578 E0.01827
G1 X162.738 Y52.895 E0.07245
G1 X162.738 Y52.294 E0.01827
```

```
G1 X160.454 Y54.578 E0.09828
G1 X159.854 Y54.578 E0.01827
G1 X162.738 Y51.694 E0.12412
G1 X162.738 Y51.094 E0.01827
G1 X159.254 Y54.578 E0.14996
G1 X158.654 Y54.578 E0.01827
G1 X162.738 Y50.494 E0.17580
G1 X162.738 Y49.893 E0.01827
G1 X158.054 Y54.578 E0.20163
G1 X157.453 Y54.578 E0.01827
G1 X162.738 Y49.293 E0.22747
M73 P68 R22
G1 X162.738 Y48.693 E0.01827
G1 X156.853 Y54.578 E0.25331
G1 X156.417 Y54.578 E0.01328
G1 X156.379 Y54.451 E0.00402
G1 X162.738 Y48.093 E0.27370
G1 X162.738 Y47.493 E0.01827
G1 X156.201 Y54.029 E0.28136
G1 X156.021 Y53.610 E0.01390
G1 X162.738 Y46.892 E0.28914
G1 X162.738 Y46.292 E0.01827
G1 X155.801 Y53.229 E0.29859
G1 X155.581 Y52.848 E0.01337
G1 X162.738 Y45.692 E0.30805
G1 X162.738 Y45.092 E0.01827
G1 X155.323 Y52.506 E0.31916
G1 X155.065 Y52.164 E0.01305
G1 X162.738 Y44.492 E0.33026
```

```
G1 X162.738 Y43.891 E0.01827
G1 X154.773 Y51.856 E0.34283
G1 X154.478 Y51.551 E0.01292
G1 X162.738 Y43.291 E0.35554
G1 X162.738 Y42.691 E0.01827
G1 X154.154 Y51.275 E0.36951
G1 X153.821 Y51.007 E0.01299
G1 X162.738 Y42.091 E0.38382
G1 X162.738 Y41.490 E0.01827
G1 X153.463 Y50.765 E0.39924
G1 X153.092 Y50.536 E0.01327
G1 X162.138 Y41.490 E0.38938
G1 X161.538 Y41.490 E0.01827
G1 X152.697 Y50.331 E0.38056
G1 X152.286 Y50.141 E0.01377
G1 X160.938 Y41.490 E0.37241
G1 X160.337 Y41.490 E0.01827
G1 X151.848 Y49.980 E0.36545
G1 X151.392 Y49.835 E0.01454
G1 X159.737 Y41.490 E0.35921
G1 X159.137 Y41.490 E0.01827
G1 X150.900 Y49.727 E0.35457
G1 X150.394 Y49.632 E0.01566
G1 X158.537 Y41.490 E0.35050
G1 X157.937 Y41.490 E0.01827
G1 X149.830 Y49.597 E0.34897
G1 X149.249 Y49.578 E0.01769
G1 X157.336 Y41.490 E0.34814
G1 X156.736 Y41.490 E0.01827
```

```
G1 X155.852 Y42.374 E0.03805
M204 S1250
G1 E-4.00000 F2400.000
G1 X148.460 Y49.766 F9000.000
G1 E4.00000 F900.000
M204 S1000
G1 F1272.331
G1 X152.312 Y45.914 E0.16578
G1 X152.132 Y45.997 E0.00604
G1 X151.393 Y46.232 E0.02358
G1 X148.048 Y49.578 E0.14400
G1 X147.448 Y49.578 E0.01827
G1 X150.636 Y46.390 E0.13722
G1 X150.388 Y46.436 E0.00767
G1 X149.963 Y46.463 E0.01297
G1 X146.848 Y49.578 E0.13409
G1 X146.248 Y49.578 E0.01827
G1 X149.335 Y46.490 E0.13291
G1 X148.735 Y46.490 E0.01827
G1 X145.647 Y49.578 E0.13291
G1 X145.047 Y49.578 E0.01827
G1 X148.135 Y46.490 E0.13291
G1 X147.535 Y46.490 E0.01827
G1 X144.447 Y49.578 E0.13291
G1 X143.847 Y49.578 E0.01827
G1 X146.934 Y46.490 E0.13291
G1 X146.334 Y46.490 E0.01827
G1 X143.246 Y49.578 E0.13291
G1 X142.646 Y49.578 E0.01827
```

```
G1 X145.734 Y46.490 E0.13291
G1 X145.134 Y46.490 E0.01827
G1 X142.046 Y49.578 E0.13291
G1 X141.650 Y49.578 E0.01205
G1 X141.650 Y49.373 E0.00622
G1 X144.533 Y46.490 E0.12412
G1 X143.933 Y46.490 E0.01827
G1 X141.650 Y48.773 E0.09828
G1 X141.650 Y48.173 E0.01827
G1 X143.333 Y46.490 E0.07244
G1 X142.733 Y46.490 E0.01827
G1 X141.650 Y47.573 E0.04661
G1 X141.650 Y46.972 E0.01827
G1 X142.321 Y46.302 E0.02886
M204 S1250
; stop printing object Petg print.STL id:28 copy 0
M106 S63.75
;LAYER_CHANGE
;Z:0.8
;HEIGHT:0.15
;BEFORE_LAYER_CHANGE
G92 E0.0
;0.8


G1 E-4.00000 F2400.000
G1 Z0.800 F9000.000
;AFTER_LAYER_CHANGE
;0.8
```

```
; printing object tpu print.STL id:19 copy 0
; stop printing object tpu print.STL id:19 copy 0
; printing object Petg print.STL id:18 copy 0
G1 X61.611 Y40.421
G1 E4.00000 F900.000
M204 S800
;TYPE:Perimeter
;WIDTH:0.45
G1 F1292.454
G1 X61.611 Y54.135 E0.41094
G1 X54.740 Y54.135 E0.20589
G1 X54.649 Y53.812 E0.01007
G1 X54.314 Y53.017 E0.02583
G1 X53.884 Y52.270 E0.02583
G1 X53.365 Y51.582 E0.02583
G1 X52.765 Y50.963 E0.02583
G1 X52.094 Y50.422 E0.02583
G1 X51.361 Y49.968 E0.02583
G1 X50.578 Y49.607 E0.02583
G1 X49.757 Y49.345 E0.02583
G1 X48.910 Y49.187 E0.02583
G1 X48.069 Y49.135 E0.02523
G1 X39.897 Y49.135 E0.24487
G1 X39.897 Y45.421 E0.11130
G1 X48.069 Y45.421 E0.24487
M73 P68 R21
G1 X48.910 Y45.369 E0.02523
G1 X49.757 Y45.210 E0.02583
G1 X50.578 Y44.949 E0.02583
```

G1 X51.361 Y44.588 E0.02583

G1 X52.094 Y44.134 E0.02583

G1 X52.765 Y43.593 E0.02583

G1 X53.365 Y42.974 E0.02583

G1 X53.884 Y42.285 E0.02583

G1 X54.314 Y41.538 E0.02583

G1 X54.649 Y40.744 E0.02583

G1 X54.740 Y40.421 E0.01007

G1 X61.551 Y40.421 E0.20409

M204 S1250

G1 X62.029 Y40.003 F9000.000

M204 S800

;TYPE:External perimeter

G1 F1292.454

G1 X62.029 Y54.553 E0.43598

G1 X54.424 Y54.553 E0.22787

G1 X54.254 Y53.950 E0.01877

G1 X53.939 Y53.203 E0.02428

G1 X53.534 Y52.501 E0.02428

G1 X53.047 Y51.854 E0.02428

G1 X52.483 Y51.272 E0.02428

G1 X51.852 Y50.763 E0.02428

G1 X51.163 Y50.336 E0.02428

G1 X50.427 Y49.997 E0.02428

G1 X49.655 Y49.751 E0.02428

G1 X48.858 Y49.602 E0.02428

G1 X48.056 Y49.553 E0.02407

G1 X39.479 Y49.553 E0.25701

G1 X39.479 Y45.003 E0.13634

```
G1 X48.056 Y45.003 E0.25701
G1 X48.858 Y44.953 E0.02407
G1 X49.655 Y44.804 E0.02428
G1 X50.427 Y44.558 E0.02428
G1 X51.163 Y44.219 E0.02428
G1 X51.852 Y43.792 E0.02428
G1 X52.483 Y43.284 E0.02428
G1 X53.047 Y42.702 E0.02428
G1 X53.534 Y42.054 E0.02428
G1 X53.939 Y41.352 E0.02428
G1 X54.254 Y40.606 E0.02428
G1 X54.424 Y40.003 E0.01877
G1 X61.969 Y40.003 E0.22608
M204 S1250
G1 X61.838 Y40.354 F9000.000
G1 X61.486 Y41.405
M204 S1000
;TYPE:Solid infill
;WIDTH:0.456609
G1 F1272.328
G1 X60.816 Y40.734 E0.02887
G1 X60.215 Y40.734 E0.01827
G1 X61.298 Y41.817 E0.04661
G1 X61.298 Y42.417 E0.01827
G1 X59.615 Y40.734 E0.07245
G1 X59.015 Y40.734 E0.01827
G1 X61.298 Y43.017 E0.09828
G1 X61.298 Y43.617 E0.01827
G1 X58.415 Y40.734 E0.12412
```

G1 X57.814 Y40.734 E0.01827

G1 X61.298 Y44.218 E0.14996

G1 X61.298 Y44.818 E0.01827

G1 X57.214 Y40.734 E0.17580

G1 X56.614 Y40.734 E0.01827

G1 X61.298 Y45.418 E0.20163

G1 X61.298 Y46.018 E0.01827

G1 X56.014 Y40.734 E0.22747

G1 X55.414 Y40.734 E0.01827

G1 X61.298 Y46.619 E0.25331

G1 X61.298 Y47.219 E0.01827

G1 X54.940 Y40.860 E0.27370

G1 X54.762 Y41.283 E0.01395

G1 X61.298 Y47.819 E0.28137

G1 X61.298 Y48.419 E0.01827

G1 X54.581 Y41.702 E0.28914

G1 X54.362 Y42.083 E0.01338

G1 X61.298 Y49.019 E0.29859

G1 X61.298 Y49.620 E0.01827

G1 X54.142 Y42.463 E0.30805

G1 X53.884 Y42.806 E0.01305

G1 X61.298 Y50.220 E0.31916

G1 X61.298 Y50.820 E0.01827

G1 X53.626 Y43.148 E0.33026

G1 X53.334 Y43.456 E0.01292

G1 X61.298 Y51.420 E0.34283

G1 X61.298 Y52.021 E0.01827

G1 X53.039 Y43.761 E0.35554

G1 X52.714 Y44.037 E0.01296

```
G1 X61.298 Y52.621 E0.36951
G1 X61.298 Y53.221 E0.01827
G1 X52.382 Y44.304 E0.38382
G1 X52.023 Y44.546 E0.01316
G1 X61.298 Y53.822 E0.39926
G1 X60.698 Y53.822 E0.01825
G1 X51.653 Y44.776 E0.38938
G1 X51.510 Y44.865 E0.00511
G1 X51.257 Y44.981 E0.00847
G1 X60.098 Y53.822 E0.38056
G1 X59.498 Y53.822 E0.01827
G1 X50.846 Y45.170 E0.37241
G1 X50.692 Y45.241 E0.00518
G1 X50.408 Y45.332 E0.00907
G1 X58.898 Y53.822 E0.36545
G1 X58.298 Y53.822 E0.01827
G1 X49.953 Y45.477 E0.35921
G1 X49.460 Y45.585 E0.01534
G1 X57.697 Y53.822 E0.35457
G1 X57.097 Y53.822 E0.01827
G1 X48.955 Y45.679 E0.35050
G1 X48.390 Y45.715 E0.01722
G1 X56.497 Y53.822 E0.34897
G1 X55.897 Y53.822 E0.01827
G1 X47.809 Y45.734 E0.34814
G1 X47.209 Y45.734 E0.01827
G1 X50.872 Y49.397 E0.15769
G1 X50.692 Y49.314 E0.00603
G1 X49.954 Y49.079 E0.02358
```

```
G1 X46.609 Y45.734 E0.14400
G1 X46.008 Y45.734 E0.01827
G1 X49.196 Y48.922 E0.13722
G1 X48.948 Y48.875 E0.00767
G1 X48.523 Y48.849 E0.01297
G1 X45.408 Y45.734 E0.13409
G1 X44.808 Y45.734 E0.01827
G1 X47.896 Y48.822 E0.13291
G1 X47.295 Y48.822 E0.01827
G1 X44.208 Y45.734 E0.13291
G1 X43.607 Y45.734 E0.01827
G1 X46.695 Y48.822 E0.13291
M73 P69 R21
G1 X46.095 Y48.822 E0.01827
G1 X43.007 Y45.734 E0.13291
G1 X42.407 Y45.734 E0.01827
G1 X45.495 Y48.822 E0.13291
G1 X44.894 Y48.822 E0.01827
G1 X41.807 Y45.734 E0.13291
G1 X41.207 Y45.734 E0.01827
G1 X44.294 Y48.822 E0.13291
G1 X43.694 Y48.822 E0.01827
G1 X40.606 Y45.734 E0.13291
G1 X40.210 Y45.734 E0.01205
G1 X40.210 Y45.938 E0.00622
G1 X43.094 Y48.822 E0.12412
G1 X42.494 Y48.822 E0.01827
G1 X40.210 Y46.539 E0.09828
G1 X40.210 Y47.139 E0.01827
```

```
G1 X41.893 Y48.822 E0.07244
G1 X41.293 Y48.822 E0.01827
G1 X40.210 Y47.739 E0.04660
G1 X40.210 Y48.339 E0.01827
G1 X40.881 Y49.010 E0.02886
M204 S1250
G1 E-4.00000 F2400.000
G1 X54.413 Y52.938 F9000.000
G1 E4.00000 F900.000
M204 S1000
G1 F1272.328
G1 X55.484 Y54.010 E0.04614
M204 S1250
; stop printing object Petg print.STL id:18 copy 0
; printing object Petg print.STL id:14 copy 0
G1 X54.748 Y60.370 F9000.000
M204 S800
;TYPE:Perimeter
;WIDTH:0.45
G1 F1292.454
G1 X61.619 Y60.370 E0.20589
G1 X61.619 Y74.084 E0.41094
G1 X54.748 Y74.084 E0.20589
G1 X54.656 Y73.760 E0.01007
G1 X54.321 Y72.966 E0.02583
G1 X53.891 Y72.219 E0.02583
G1 X53.372 Y71.531 E0.02583
G1 X52.773 Y70.912 E0.02583
G1 X52.101 Y70.371 E0.02583
```

```
G1 X51.369 Y69.917 E0.02583
G1 X50.586 Y69.556 E0.02583
G1 X49.765 Y69.294 E0.02583
G1 X48.917 Y69.136 E0.02583
G1 X48.077 Y69.084 E0.02523
G1 X39.905 Y69.084 E0.24487
G1 X39.905 Y65.370 E0.11130
G1 X48.077 Y65.370 E0.24487
G1 X48.917 Y65.318 E0.02523
G1 X49.765 Y65.159 E0.02583
G1 X50.586 Y64.898 E0.02583
G1 X51.369 Y64.537 E0.02583
G1 X52.101 Y64.083 E0.02583
G1 X52.773 Y63.542 E0.02583
G1 X53.372 Y62.923 E0.02583
G1 X53.891 Y62.234 E0.02583
G1 X54.321 Y61.487 E0.02583
G1 X54.656 Y60.693 E0.02583
G1 X54.732 Y60.427 E0.00828
M204 S1250
G1 X54.432 Y59.952 F9000.000
M204 S800
;TYPE:External perimeter
G1 F1292.454
G1 X62.037 Y59.952 E0.22787
G1 X62.037 Y74.502 E0.43598
G1 X54.432 Y74.502 E0.22787
G1 X54.261 Y73.899 E0.01877
G1 X53.946 Y73.152 E0.02428
```

```
G1 X53.542 Y72.450 E0.02428
G1 X53.054 Y71.803 E0.02428
G1 X52.490 Y71.221 E0.02428
G1 X51.859 Y70.712 E0.02428
G1 X51.171 Y70.285 E0.02428
G1 X50.435 Y69.946 E0.02428
G1 X49.662 Y69.700 E0.02428
G1 X48.866 Y69.551 E0.02428
G1 X48.064 Y69.502 E0.02407
G1 X39.487 Y69.502 E0.25701
G1 X39.487 Y64.952 E0.13634
G1 X48.064 Y64.952 E0.25701
G1 X48.866 Y64.902 E0.02407
G1 X49.662 Y64.753 E0.02428
G1 X50.435 Y64.507 E0.02428
G1 X51.171 Y64.168 E0.02428
G1 X51.859 Y63.741 E0.02428
G1 X52.490 Y63.233 E0.02428
G1 X53.054 Y62.651 E0.02428
G1 X53.542 Y62.003 E0.02428
G1 X53.946 Y61.301 E0.02428
G1 X54.261 Y60.554 E0.02428
G1 X54.416 Y60.010 E0.01697
M204 S1250
G1 X54.753 Y60.190 F9000.000
G1 E-4.00000 F2400.000
G1 X61.494 Y61.354 F9000.000
G1 E4.00000 F900.000
M204 S1000
```

```
;TYPE:Solid infill
;WIDTH:0.456609
G1 F1272.328
G1 X60.823 Y60.683 E0.02887
G1 X60.223 Y60.683 E0.01827
G1 X61.306 Y61.766 E0.04661
G1 X61.306 Y62.366 E0.01827
G1 X59.623 Y60.683 E0.07245
G1 X59.022 Y60.683 E0.01827
G1 X61.306 Y62.966 E0.09828
G1 X61.306 Y63.566 E0.01827
G1 X58.422 Y60.683 E0.12412
G1 X57.822 Y60.683 E0.01827
G1 X61.306 Y64.167 E0.14996
G1 X61.306 Y64.767 E0.01827
G1 X57.222 Y60.683 E0.17580
G1 X56.621 Y60.683 E0.01827
G1 X61.306 Y65.367 E0.20163
G1 X61.306 Y65.967 E0.01827
G1 X56.021 Y60.683 E0.22747
G1 X55.421 Y60.683 E0.01827
G1 X61.306 Y66.568 E0.25331
G1 X61.306 Y67.168 E0.01827
G1 X54.947 Y60.809 E0.27370
G1 X54.769 Y61.232 E0.01395
G1 X61.306 Y67.768 E0.28137
G1 X61.306 Y68.368 E0.01827
G1 X54.589 Y61.651 E0.28914
G1 X54.369 Y62.032 E0.01338
```

G1 X61.306 Y68.968 E0.29859

G1 X61.306 Y69.569 E0.01827

G1 X54.149 Y62.412 E0.30805

G1 X53.891 Y62.755 E0.01305

G1 X61.306 Y70.169 E0.31916

G1 X61.306 Y70.769 E0.01827

G1 X53.633 Y63.097 E0.33026

G1 X53.341 Y63.405 E0.01292

G1 X61.306 Y71.369 E0.34283

G1 X61.306 Y71.969 E0.01827

G1 X53.046 Y63.710 E0.35554

G1 X52.721 Y63.986 E0.01296

G1 X61.306 Y72.570 E0.36951

G1 X61.306 Y73.170 E0.01827

G1 X52.389 Y64.253 E0.38382

G1 X52.031 Y64.495 E0.01316

G1 X61.306 Y73.771 E0.39926

G1 X60.706 Y73.771 E0.01825

G1 X51.660 Y64.725 E0.38938

G1 X51.518 Y64.813 E0.00511

G1 X51.265 Y64.930 E0.00847

G1 X60.106 Y73.771 E0.38056

G1 X59.505 Y73.771 E0.01827

G1 X50.854 Y65.119 E0.37241

G1 X50.699 Y65.190 E0.00518

G1 X50.415 Y65.281 E0.00907

G1 X58.905 Y73.771 E0.36545

G1 X58.305 Y73.771 E0.01827

G1 X49.960 Y65.426 E0.35921

G1 X49.468 Y65.534 E0.01534

G1 X57.705 Y73.771 E0.35457

G1 X57.105 Y73.771 E0.01827

G1 X48.962 Y65.628 E0.35050

G1 X48.397 Y65.664 E0.01722

G1 X56.504 Y73.771 E0.34897

G1 X55.904 Y73.771 E0.01827

G1 X47.816 Y65.683 E0.34814

G1 X47.216 Y65.683 E0.01827

G1 X50.880 Y69.346 E0.15769

G1 X50.699 Y69.263 E0.00603

G1 X49.961 Y69.028 E0.02358

G1 X46.616 Y65.683 E0.14400

G1 X46.016 Y65.683 E0.01827

G1 X49.203 Y68.871 E0.13722

G1 X48.956 Y68.824 E0.00767

G1 X48.531 Y68.798 E0.01297

G1 X45.416 Y65.683 E0.13409

G1 X44.815 Y65.683 E0.01827

G1 X47.903 Y68.771 E0.13291

G1 X47.303 Y68.771 E0.01827

G1 X44.215 Y65.683 E0.13291

G1 X43.615 Y65.683 E0.01827

G1 X46.703 Y68.771 E0.13291

G1 X46.102 Y68.771 E0.01827

G1 X43.015 Y65.683 E0.13291

G1 X42.414 Y65.683 E0.01827

G1 X45.502 Y68.771 E0.13291

G1 X44.902 Y68.771 E0.01827

```
G1 X41.814 Y65.683 E0.13291
G1 X41.214 Y65.683 E0.01827
G1 X44.302 Y68.771 E0.13291
G1 X43.701 Y68.771 E0.01827
G1 X40.614 Y65.683 E0.13291
G1 X40.218 Y65.683 E0.01205
G1 X40.218 Y65.887 E0.00622
G1 X43.101 Y68.771 E0.12412
G1 X42.501 Y68.771 E0.01827
G1 X40.218 Y66.488 E0.09828
G1 X40.218 Y67.088 E0.01827
G1 X41.901 Y68.771 E0.07244
G1 X41.301 Y68.771 E0.01827
G1 X40.218 Y67.688 E0.04660
G1 X40.218 Y68.288 E0.01827
G1 X40.888 Y68.959 E0.02886
M204 S1250
G1 E-4.00000 F2400.000
G1 X54.420 Y72.887 F9000.000
G1 E4.00000 F900.000
M204 S1000
G1 F1272.328
G1 X55.492 Y73.959 E0.04614
M204 S1250
; stop printing object Petg print.STL id:14 copy 0
; printing object tpu print.STL id:15 copy 0
; stop printing object tpu print.STL id:15 copy 0
; printing object Petg print.STL id:10 copy 0
G1 E-4.00000 F2400.000
```

```
G1 X54.697 Y82.562 F9000.000
G1 E4.00000 F900.000
M204 S800
;TYPE:Perimeter
;WIDTH:0.45
G1 F1292.454
G1 X61.568 Y82.562 E0.20589
G1 X61.568 Y96.276 E0.41094
G1 X54.697 Y96.276 E0.20589
G1 X54.605 Y95.953 E0.01007
G1 X54.270 Y95.159 E0.02583
G1 X53.840 Y94.412 E0.02583
G1 X53.321 Y93.723 E0.02583
G1 X52.722 Y93.104 E0.02583
G1 X52.050 Y92.563 E0.02583
G1 X51.318 Y92.109 E0.02583
G1 X50.535 Y91.748 E0.02583
G1 X49.713 Y91.487 E0.02583
G1 X48.866 Y91.328 E0.02583
G1 X48.026 Y91.276 E0.02523
G1 X39.853 Y91.276 E0.24487
G1 X39.853 Y87.562 E0.11130
G1 X48.026 Y87.562 E0.24487
G1 X48.866 Y87.510 E0.02523
G1 X49.713 Y87.351 E0.02583
G1 X50.535 Y87.090 E0.02583
G1 X51.318 Y86.729 E0.02583
G1 X52.050 Y86.275 E0.02583
G1 X52.722 Y85.734 E0.02583
```

```
G1 X53.321 Y85.115 E0.02583
G1 X53.840 Y84.427 E0.02583
G1 X54.270 Y83.680 E0.02583
G1 X54.605 Y82.885 E0.02583
G1 X54.680 Y82.620 E0.00828
M204 S1250
G1 X54.381 Y82.144 F9000.000
M204 S800
;TYPE:External perimeter
G1 F1292.454
G1 X61.986 Y82.144 E0.22787
G1 X61.986 Y96.694 E0.43598
G1 X54.381 Y96.694 E0.22787
G1 X54.210 Y96.091 E0.01877
G1 X53.895 Y95.345 E0.02428
G1 X53.491 Y94.643 E0.02428
G1 X53.003 Y93.995 E0.02428
G1 X52.439 Y93.413 E0.02428
G1 X51.808 Y92.905 E0.02428
G1 X51.119 Y92.478 E0.02428
G1 X50.383 Y92.139 E0.02428
G1 X49.611 Y91.893 E0.02428
G1 X48.815 Y91.744 E0.02428
G1 X48.013 Y91.694 E0.02407
G1 X39.436 Y91.694 E0.25701
G1 X39.436 Y87.144 E0.13634
G1 X48.013 Y87.144 E0.25701
G1 X48.815 Y87.095 E0.02407
G1 X49.611 Y86.946 E0.02428
```

```
G1 X50.383 Y86.700 E0.02428
G1 X51.119 Y86.361 E0.02428
G1 X51.808 Y85.934 E0.02428
G1 X52.439 Y85.425 E0.02428
G1 X53.003 Y84.843 E0.02428
G1 X53.491 Y84.196 E0.02428
G1 X53.895 Y83.493 E0.02428
G1 X54.210 Y82.747 E0.02428
G1 X54.364 Y82.202 E0.01697
M204 S1250
G1 X54.702 Y82.383 F9000.000
G1 E-4.00000 F2400.000
G1 X61.442 Y83.546 F9000.000
G1 E4.00000 F900.000
M204 S1000
;TYPE:Solid infill
;WIDTH:0.456609
G1 F1272.328
G1 X60.772 Y82.875 E0.02887
G1 X60.172 Y82.875 E0.01827
G1 X61.254 Y83.958 E0.04661
G1 X61.254 Y84.558 E0.01827
G1 X59.571 Y82.875 E0.07245
G1 X58.971 Y82.875 E0.01827
G1 X61.254 Y85.159 E0.09828
G1 X61.254 Y85.759 E0.01827
G1 X58.371 Y82.875 E0.12412
G1 X57.771 Y82.875 E0.01827
G1 X61.254 Y86.359 E0.14996
```

```
G1 X61.254 Y86.959 E0.01827
G1 X57.171 Y82.875 E0.17580
G1 X56.570 Y82.875 E0.01827
G1 X61.254 Y87.559 E0.20163
G1 X61.254 Y88.160 E0.01827
G1 X55.970 Y82.875 E0.22747
G1 X55.370 Y82.875 E0.01827
G1 X61.254 Y88.760 E0.25331
G1 X61.254 Y89.360 E0.01827
G1 X54.896 Y83.002 E0.27370
G1 X54.718 Y83.424 E0.01395
G1 X61.254 Y89.960 E0.28137
M73 P69 R20
G1 X61.254 Y90.561 E0.01827
G1 X54.537 Y83.843 E0.28914
G1 X54.318 Y84.224 E0.01338
G1 X61.254 Y91.161 E0.29859
G1 X61.254 Y91.761 E0.01827
G1 X54.098 Y84.605 E0.30805
M73 P70 R20
G1 X53.840 Y84.947 E0.01305
G1 X61.254 Y92.361 E0.31916
G1 X61.254 Y92.961 E0.01827
G1 X53.582 Y85.289 E0.33026
G1 X53.290 Y85.597 E0.01292
G1 X61.254 Y93.562 E0.34283
G1 X61.254 Y94.162 E0.01827
G1 X52.995 Y85.902 E0.35554
G1 X52.670 Y86.178 E0.01296
```

```
G1 X61.254 Y94.762 E0.36951
G1 X61.254 Y95.362 E0.01827
G1 X52.338 Y86.446 E0.38382
G1 X51.980 Y86.688 E0.01316
G1 X61.254 Y95.963 E0.39926
G1 X60.655 Y95.963 E0.01825
G1 X51.609 Y86.917 E0.38938
G1 X51.466 Y87.006 E0.00511
G1 X51.214 Y87.122 E0.00847
G1 X60.055 Y95.963 E0.38056
G1 X59.454 Y95.963 E0.01827
G1 X50.803 Y87.311 E0.37241
G1 X50.648 Y87.383 E0.00518
G1 X50.364 Y87.473 E0.00907
G1 X58.854 Y95.963 E0.36545
G1 X58.254 Y95.963 E0.01827
G1 X49.909 Y87.618 E0.35921
G1 X49.417 Y87.726 E0.01534
G1 X57.654 Y95.963 E0.35457
G1 X57.053 Y95.963 E0.01827
G1 X48.911 Y87.820 E0.35050
G1 X48.346 Y87.856 E0.01722
G1 X56.453 Y95.963 E0.34897
G1 X55.853 Y95.963 E0.01827
G1 X47.765 Y87.875 E0.34814
G1 X47.165 Y87.875 E0.01827
G1 X50.828 Y91.539 E0.15769
G1 X50.648 Y91.456 E0.00603
G1 X49.910 Y91.221 E0.02358
```

G1 X46.565 Y87.875 E0.14400

G1 X45.965 Y87.875 E0.01827

G1 X49.152 Y91.063 E0.13722

G1 X48.905 Y91.017 E0.00767

G1 X48.480 Y90.990 E0.01297

G1 X45.364 Y87.875 E0.13409

G1 X44.764 Y87.875 E0.01827

G1 X47.852 Y90.963 E0.13291

G1 X47.252 Y90.963 E0.01827

G1 X44.164 Y87.875 E0.13291

G1 X43.564 Y87.875 E0.01827

G1 X46.651 Y90.963 E0.13291

G1 X46.051 Y90.963 E0.01827

G1 X42.964 Y87.875 E0.13291

G1 X42.363 Y87.875 E0.01827

G1 X45.451 Y90.963 E0.13291

G1 X44.851 Y90.963 E0.01827

G1 X41.763 Y87.875 E0.13291

G1 X41.163 Y87.875 E0.01827

G1 X44.251 Y90.963 E0.13291

G1 X43.650 Y90.963 E0.01827

G1 X40.563 Y87.875 E0.13291

G1 X40.167 Y87.875 E0.01205

G1 X40.167 Y88.080 E0.00622

G1 X43.050 Y90.963 E0.12412

G1 X42.450 Y90.963 E0.01827

G1 X40.167 Y88.680 E0.09828

G1 X40.167 Y89.280 E0.01827

G1 X41.850 Y90.963 E0.07244

```
G1 X41.249 Y90.963 E0.01827
G1 X40.167 Y89.880 E0.04660
G1 X40.167 Y90.481 E0.01827
G1 X40.837 Y91.151 E0.02886
M204 S1250
G1 E-4.00000 F2400.000
G1 X54.369 Y95.079 F9000.000
G1 E4.00000 F900.000
M204 S1000
G1 F1272.328
G1 X55.441 Y96.151 E0.04614
M204 S1250
; stop printing object Petg print.STL id:10 copy 0
; printing object tpu print.STL id:11 copy 0
; stop printing object tpu print.STL id:11 copy 0
; printing object tpu print.STL id:13 copy 0
; stop printing object tpu print.STL id:13 copy 0
; printing object tpu print.STL id:17 copy 0
; stop printing object tpu print.STL id:17 copy 0
; printing object Petg print.STL id:16 copy 0
G1 E-4.00000 F2400.000
G1 X54.688 Y122.031 F9000.000
G1 E4.00000 F900.000
M204 S800
;TYPE:Perimeter
;WIDTH:0.45
G1 F1292.454
G1 X61.560 Y122.031 E0.20589
G1 X61.560 Y135.746 E0.41094
```

```
G1 X54.688 Y135.746 E0.20589
G1 X54.597 Y135.422 E0.01007
G1 X54.262 Y134.628 E0.02583
G1 X53.832 Y133.881 E0.02583
G1 X53.313 Y133.193 E0.02583
G1 X52.713 Y132.573 E0.02583
G1 X52.042 Y132.033 E0.02583
G1 X51.309 Y131.578 E0.02583
G1 X50.526 Y131.218 E0.02583
G1 X49.705 Y130.956 E0.02583
G1 X48.858 Y130.798 E0.02583
G1 X48.017 Y130.746 E0.02523
G1 X39.845 Y130.746 E0.24487
G1 X39.845 Y127.031 E0.11130
G1 X48.017 Y127.031 E0.24487
G1 X48.858 Y126.980 E0.02523
G1 X49.705 Y126.821 E0.02583
G1 X50.526 Y126.559 E0.02583
G1 X51.309 Y126.199 E0.02583
G1 X52.042 Y125.745 E0.02583
G1 X52.713 Y125.204 E0.02583
G1 X53.313 Y124.584 E0.02583
G1 X53.832 Y123.896 E0.02583
G1 X54.262 Y123.149 E0.02583
G1 X54.597 Y122.355 E0.02583
G1 X54.672 Y122.089 E0.00828
M204 S1250
G1 X54.373 Y121.614 F9000.000
M204 S800
```

```
;TYPE:External perimeter
G1 F1292.454
G1 X61.977 Y121.614 E0.22787
G1 X61.977 Y136.164 E0.43598
G1 X54.373 Y136.164 E0.22787
G1 X54.202 Y135.561 E0.01877
G1 X53.887 Y134.814 E0.02428
G1 X53.482 Y134.112 E0.02428
G1 X52.995 Y133.465 E0.02428
G1 X52.431 Y132.883 E0.02428
G1 X51.800 Y132.374 E0.02428
G1 X51.111 Y131.947 E0.02428
G1 X50.375 Y131.608 E0.02428
G1 X49.603 Y131.362 E0.02428
G1 X48.806 Y131.213 E0.02428
G1 X48.004 Y131.164 E0.02407
G1 X39.427 Y131.164 E0.25701
G1 X39.427 Y126.614 E0.13634
G1 X48.004 Y126.614 E0.25701
G1 X48.806 Y126.564 E0.02407
G1 X49.603 Y126.415 E0.02428
G1 X50.375 Y126.169 E0.02428
G1 X51.111 Y125.830 E0.02428
G1 X51.800 Y125.403 E0.02428
G1 X52.431 Y124.895 E0.02428
G1 X52.995 Y124.312 E0.02428
G1 X53.482 Y123.665 E0.02428
G1 X53.887 Y122.963 E0.02428
G1 X54.202 Y122.216 E0.02428
```

```
G1 X54.356 Y121.671 E0.01697
M204 S1250
G1 X54.694 Y121.852 F9000.000
G1 E-4.00000 F2400.000
G1 X61.434 Y123.015 F9000.000
G1 E4.00000 F900.000
M204 S1000
;TYPE:Solid infill
;WIDTH:0.456609
G1 F1272.328
G1 X60.764 Y122.345 E0.02887
G1 X60.163 Y122.345 E0.01827
G1 X61.246 Y123.428 E0.04661
G1 X61.246 Y124.028 E0.01827
G1 X59.563 Y122.345 E0.07245
G1 X58.963 Y122.345 E0.01827
G1 X61.246 Y124.628 E0.09828
G1 X61.246 Y125.228 E0.01827
G1 X58.363 Y122.345 E0.12412
G1 X57.763 Y122.345 E0.01827
G1 X61.246 Y125.828 E0.14996
G1 X61.246 Y126.429 E0.01827
G1 X57.162 Y122.345 E0.17580
G1 X56.562 Y122.345 E0.01827
G1 X61.246 Y127.029 E0.20163
G1 X61.246 Y127.629 E0.01827
G1 X55.962 Y122.345 E0.22747
G1 X55.362 Y122.345 E0.01827
G1 X61.246 Y128.229 E0.25331
```

```
G1 X61.246 Y128.830 E0.01827
G1 X54.888 Y122.471 E0.27370
G1 X54.710 Y122.893 E0.01395
G1 X61.246 Y129.430 E0.28137
G1 X61.246 Y130.030 E0.01827
G1 X54.529 Y123.313 E0.28914
G1 X54.310 Y123.694 E0.01338
G1 X61.246 Y130.630 E0.29859
G1 X61.246 Y131.230 E0.01827
G1 X54.090 Y124.074 E0.30805
G1 X53.832 Y124.416 E0.01305
G1 X61.246 Y131.831 E0.31916
G1 X61.246 Y132.431 E0.01827
G1 X53.574 Y124.759 E0.33026
G1 X53.282 Y125.067 E0.01292
G1 X61.246 Y133.031 E0.34283
G1 X61.246 Y133.631 E0.01827
G1 X52.987 Y125.372 E0.35554
G1 X52.662 Y125.647 E0.01296
G1 X61.246 Y134.232 E0.36951
G1 X61.246 Y134.832 E0.01827
G1 X52.330 Y125.915 E0.38382
G1 X51.971 Y126.157 E0.01316
G1 X61.246 Y135.432 E0.39926
G1 X60.646 Y135.432 E0.01825
G1 X51.601 Y126.387 E0.38938
G1 X51.458 Y126.475 E0.00511
G1 X51.205 Y126.592 E0.00847
G1 X60.046 Y135.432 E0.38056
```

```
G1 X59.446 Y135.432 E0.01827
G1 X50.795 Y126.781 E0.37241
G1 X50.640 Y126.852 E0.00518
G1 X50.356 Y126.943 E0.00907
G1 X58.846 Y135.432 E0.36545
G1 X58.246 Y135.432 E0.01827
G1 X49.901 Y127.088 E0.35921
G1 X49.408 Y127.195 E0.01534
G1 X57.645 Y135.432 E0.35457
G1 X57.045 Y135.432 E0.01827
G1 X48.903 Y127.290 E0.35050
G1 X48.338 Y127.326 E0.01722
G1 X56.445 Y135.432 E0.34897
G1 X55.845 Y135.432 E0.01827
G1 X47.757 Y127.345 E0.34814
G1 X47.157 Y127.345 E0.01827
G1 X50.820 Y131.008 E0.15769
G1 X50.640 Y130.925 E0.00603
G1 X49.902 Y130.690 E0.02358
G1 X46.557 Y127.345 E0.14400
G1 X45.956 Y127.345 E0.01827
G1 X49.144 Y130.532 E0.13722
G1 X48.896 Y130.486 E0.00767
G1 X48.471 Y130.460 E0.01297
G1 X45.356 Y127.345 E0.13409
G1 X44.756 Y127.345 E0.01827
G1 X47.844 Y130.432 E0.13291
G1 X47.243 Y130.432 E0.01827
G1 X44.156 Y127.345 E0.13291
```

```
G1 X43.556 Y127.345 E0.01827
G1 X46.643 Y130.432 E0.13291
G1 X46.043 Y130.432 E0.01827
G1 X42.955 Y127.345 E0.13291
G1 X42.355 Y127.345 E0.01827
G1 X45.443 Y130.432 E0.13291
G1 X44.843 Y130.432 E0.01827
G1 X41.755 Y127.345 E0.13291
G1 X41.155 Y127.345 E0.01827
G1 X44.242 Y130.432 E0.13291
G1 X43.642 Y130.432 E0.01827
G1 X40.554 Y127.345 E0.13291
G1 X40.159 Y127.345 E0.01205
G1 X40.159 Y127.549 E0.00622
G1 X43.042 Y130.432 E0.12412
G1 X42.442 Y130.432 E0.01827
G1 X40.159 Y128.149 E0.09828
G1 X40.159 Y128.750 E0.01827
G1 X41.841 Y130.432 E0.07244
G1 X41.241 Y130.432 E0.01827
G1 X40.159 Y129.350 E0.04660
G1 X40.159 Y129.950 E0.01827
G1 X40.829 Y130.620 E0.02886
M204 S1250
G1 E-4.00000 F2400.000
G1 X54.361 Y134.549 F9000.000
G1 E4.00000 F900.000
M204 S1000
G1 F1272.328
```

```
G1 X55.433 Y135.620 E0.04614
M204 S1250
; stop printing object Petg print.STL id:16 copy 0
; printing object Petg print.STL id:12 copy 0
G1 E-4.00000 F2400.000
M73 P71 R20
G1 X54.704 Y117.092 F9000.000
G1 E4.00000 F900.000
M204 S800
;TYPE:Perimeter
;WIDTH:0.45
G1 F1292.454
G1 X54.612 Y116.768 E0.01007
G1 X54.277 Y115.974 E0.02583
G1 X53.847 Y115.227 E0.02583
G1 X53.328 Y114.539 E0.02583
G1 X52.728 Y113.920 E0.02583
G1 X52.057 Y113.379 E0.02583
G1 X51.325 Y112.924 E0.02583
G1 X50.542 Y112.564 E0.02583
G1 X49.720 Y112.302 E0.02583
G1 X48.873 Y112.144 E0.02583
G1 X48.032 Y112.092 E0.02523
G1 X39.860 Y112.092 E0.24487
G1 X39.860 Y108.377 E0.11130
G1 X48.032 Y108.377 E0.24487
G1 X48.873 Y108.326 E0.02523
G1 X49.720 Y108.167 E0.02583
G1 X50.542 Y107.905 E0.02583
```

G1 X51.325 Y107.545 E0.02583

G1 X52.057 Y107.091 E0.02583

G1 X52.728 Y106.550 E0.02583

G1 X53.328 Y105.930 E0.02583

G1 X53.847 Y105.242 E0.02583

G1 X54.277 Y104.495 E0.02583

G1 X54.612 Y103.701 E0.02583

G1 X54.704 Y103.377 E0.01007

G1 X61.575 Y103.377 E0.20589

G1 X61.575 Y117.092 E0.41094

G1 X54.764 Y117.092 E0.20409

M204 S1250

G1 X54.388 Y117.510 F9000.000

M204 S800

;TYPE:External perimeter

G1 F1292.454

G1 X54.217 Y116.907 E0.01877

G1 X53.902 Y116.160 E0.02428

G1 X53.498 Y115.458 E0.02428

G1 X53.010 Y114.811 E0.02428

G1 X52.446 Y114.229 E0.02428

G1 X51.815 Y113.720 E0.02428

G1 X51.126 Y113.293 E0.02428

G1 X50.390 Y112.954 E0.02428

G1 X49.618 Y112.708 E0.02428

G1 X48.821 Y112.559 E0.02428

G1 X48.020 Y112.510 E0.02407

G1 X39.442 Y112.510 E0.25701

G1 X39.442 Y107.960 E0.13634

```
G1 X48.020 Y107.960 E0.25701
G1 X48.821 Y107.910 E0.02407
G1 X49.618 Y107.761 E0.02428
G1 X50.390 Y107.515 E0.02428
G1 X51.126 Y107.176 E0.02428
G1 X51.815 Y106.749 E0.02428
G1 X52.446 Y106.241 E0.02428
G1 X53.010 Y105.658 E0.02428
G1 X53.498 Y105.011 E0.02428
G1 X53.902 Y104.309 E0.02428
G1 X54.217 Y103.562 E0.02428
G1 X54.388 Y102.960 E0.01877
G1 X61.992 Y102.960 E0.22787
G1 X61.992 Y117.510 E0.43598
G1 X54.448 Y117.510 E0.22608
M204 S1250
G1 X54.378 Y117.110 F9000.000
G1 X55.448 Y116.967
M204 S1000
;TYPE:Solid infill
;WIDTH:0.456609
G1 F1272.328
G1 X54.376 Y115.895 E0.04614
M204 S1250
G1 E-4.00000 F2400.000
G1 X61.449 Y104.361 F9000.000
G1 E4.00000 F900.000
M204 S1000
G1 F1272.328
```

```
G1 X60.779 Y103.691 E0.02887
G1 X60.179 Y103.691 E0.01827
G1 X61.261 Y104.774 E0.04661
G1 X61.261 Y105.374 E0.01827
G1 X59.578 Y103.691 E0.07245
G1 X58.978 Y103.691 E0.01827
G1 X61.261 Y105.974 E0.09828
G1 X61.261 Y106.574 E0.01827
G1 X58.378 Y103.691 E0.12412
G1 X57.778 Y103.691 E0.01827
G1 X61.261 Y107.175 E0.14996
G1 X61.261 Y107.775 E0.01827
G1 X57.177 Y103.691 E0.17580
G1 X56.577 Y103.691 E0.01827
G1 X61.261 Y108.375 E0.20163
G1 X61.261 Y108.975 E0.01827
G1 X55.977 Y103.691 E0.22747
G1 X55.377 Y103.691 E0.01827
G1 X61.261 Y109.575 E0.25331
G1 X61.261 Y110.176 E0.01827
G1 X54.903 Y103.817 E0.27370
G1 X54.725 Y104.239 E0.01395
G1 X61.261 Y110.776 E0.28137
G1 X61.261 Y111.376 E0.01827
G1 X54.544 Y104.659 E0.28914
G1 X54.325 Y105.040 E0.01338
G1 X61.261 Y111.976 E0.29859
G1 X61.261 Y112.576 E0.01827
G1 X54.105 Y105.420 E0.30805
```

```
G1 X53.847 Y105.762 E0.01305
G1 X61.261 Y113.177 E0.31916
G1 X61.261 Y113.777 E0.01827
G1 X53.589 Y106.105 E0.33026
G1 X53.297 Y106.413 E0.01292
G1 X61.261 Y114.377 E0.34283
G1 X61.261 Y114.977 E0.01827
G1 X53.002 Y106.718 E0.35554
G1 X52.677 Y106.993 E0.01296
G1 X61.261 Y115.578 E0.36951
G1 X61.261 Y116.178 E0.01827
G1 X52.345 Y107.261 E0.38382
G1 X51.986 Y107.503 E0.01316
G1 X61.261 Y116.778 E0.39926
G1 X60.662 Y116.778 E0.01825
G1 X51.616 Y107.733 E0.38938
G1 X51.473 Y107.821 E0.00511
G1 X51.221 Y107.938 E0.00847
G1 X60.061 Y116.778 E0.38056
M73 P71 R19
G1 X59.461 Y116.778 E0.01827
G1 X50.810 Y108.127 E0.37241
G1 X50.655 Y108.198 E0.00518
G1 X50.371 Y108.289 E0.00907
G1 X58.861 Y116.778 E0.36545
G1 X58.261 Y116.778 E0.01827
G1 X49.916 Y108.434 E0.35921
G1 X49.423 Y108.541 E0.01534
G1 X57.660 Y116.778 E0.35457
```

```
G1 X57.060 Y116.778 E0.01827
G1 X48.918 Y108.636 E0.35050
G1 X48.353 Y108.672 E0.01722
G1 X56.460 Y116.778 E0.34897
G1 X55.860 Y116.778 E0.01827
G1 X47.772 Y108.691 E0.34814
G1 X47.172 Y108.691 E0.01827
G1 X50.835 Y112.354 E0.15769
G1 X50.655 Y112.271 E0.00603
G1 X49.917 Y112.036 E0.02358
G1 X46.572 Y108.691 E0.14400
G1 X45.971 Y108.691 E0.01827
G1 X49.159 Y111.879 E0.13722
G1 X48.912 Y111.832 E0.00767
G1 X48.486 Y111.806 E0.01297
G1 X45.371 Y108.691 E0.13409
G1 X44.771 Y108.691 E0.01827
G1 X47.859 Y111.778 E0.13291
G1 X47.259 Y111.778 E0.01827
G1 X44.171 Y108.691 E0.13291
G1 X43.571 Y108.691 E0.01827
G1 X46.658 Y111.778 E0.13291
G1 X46.058 Y111.778 E0.01827
G1 X42.970 Y108.691 E0.13291
G1 X42.370 Y108.691 E0.01827
G1 X45.458 Y111.778 E0.13291
G1 X44.858 Y111.778 E0.01827
G1 X41.770 Y108.691 E0.13291
G1 X41.170 Y108.691 E0.01827
```

```
G1 X44.257 Y111.778 E0.13291
G1 X43.657 Y111.778 E0.01827
G1 X40.570 Y108.691 E0.13291
G1 X40.174 Y108.691 E0.01205
G1 X40.174 Y108.895 E0.00622
G1 X43.057 Y111.778 E0.12412
G1 X42.457 Y111.778 E0.01827
G1 X40.174 Y109.495 E0.09828
G1 X40.174 Y110.096 E0.01827
G1 X41.857 Y111.778 E0.07244
G1 X41.256 Y111.778 E0.01827
G1 X40.174 Y110.696 E0.04660
G1 X40.174 Y111.296 E0.01827
G1 X40.844 Y111.967 E0.02886
M204 S1250
; stop printing object Petg print.STL id:12 copy 0
; printing object tpu print.STL id:7 copy 0
; stop printing object tpu print.STL id:7 copy 0
; printing object tpu print.STL id:3 copy 0
; stop printing object tpu print.STL id:3 copy 0
; printing object Petg print.STL id:2 copy 0
G1 E-4.00000 F2400.000
G1 X89.650 Y112.673 F9000.000
G1 E4.00000 F900.000
M204 S800
;TYPE:Perimeter
;WIDTH:0.45
G1 F1292.454
G1 X89.650 Y108.958 E0.11130
```

```
G1 X97.822 Y108.958 E0.24487
G1 X98.662 Y108.906 E0.02523
G1 X99.510 Y108.748 E0.02583
G1 X100.331 Y108.486 E0.02583
G1 X101.114 Y108.126 E0.02583
G1 X101.847 Y107.672 E0.02583
G1 X102.518 Y107.131 E0.02583
G1 X103.117 Y106.511 E0.02583
G1 X103.636 Y105.823 E0.02583
G1 X104.066 Y105.076 E0.02583
G1 X104.401 Y104.282 E0.02583
G1 X104.493 Y103.958 E0.01007
G1 X111.364 Y103.958 E0.20589
G1 X111.364 Y117.673 E0.41094
G1 X104.493 Y117.673 E0.20589
G1 X104.401 Y117.349 E0.01007
G1 X104.066 Y116.555 E0.02583
G1 X103.636 Y115.808 E0.02583
G1 X103.117 Y115.120 E0.02583
G1 X102.518 Y114.500 E0.02583
G1 X101.847 Y113.960 E0.02583
G1 X101.114 Y113.505 E0.02583
G1 X100.331 Y113.145 E0.02583
G1 X99.510 Y112.883 E0.02583
G1 X98.662 Y112.725 E0.02583
G1 X97.822 Y112.673 E0.02523
G1 X89.710 Y112.673 E0.24307
M204 S1250
G1 X89.232 Y113.091 F9000.000
```

```
M204 S800
;TYPE:External perimeter
G1 F1292.454
G1 X89.232 Y108.541 E0.13634
G1 X97.809 Y108.541 E0.25701
G1 X98.611 Y108.491 E0.02407
G1 X99.407 Y108.342 E0.02428
G1 X100.180 Y108.096 E0.02428
G1 X100.916 Y107.757 E0.02428
G1 X101.604 Y107.330 E0.02428
G1 X102.235 Y106.822 E0.02428
G1 X102.799 Y106.239 E0.02428
G1 X103.287 Y105.592 E0.02428
G1 X103.691 Y104.890 E0.02428
G1 X104.006 Y104.143 E0.02428
G1 X104.177 Y103.541 E0.01877
G1 X111.782 Y103.541 E0.22787
G1 X111.782 Y118.091 E0.43598
G1 X104.177 Y118.091 E0.22787
G1 X104.006 Y117.488 E0.01877
G1 X103.691 Y116.741 E0.02428
G1 X103.287 Y116.039 E0.02428
G1 X102.799 Y115.392 E0.02428
G1 X102.235 Y114.810 E0.02428
G1 X101.604 Y114.301 E0.02428
G1 X100.916 Y113.874 E0.02428
G1 X100.180 Y113.535 E0.02428
G1 X99.407 Y113.289 E0.02428
G1 X98.611 Y113.140 E0.02428
```

```
G1 X97.809 Y113.091 E0.02407
G1 X89.292 Y113.091 E0.25521
M204 S1250
G1 X89.432 Y112.744 F9000.000
G1 X90.633 Y112.547
M204 S1000
;TYPE:Solid infill
;WIDTH:0.456609
G1 F1272.328
G1 X89.963 Y111.877 E0.02886
G1 X89.963 Y111.277 E0.01827
G1 X91.046 Y112.359 E0.04660
G1 X91.646 Y112.359 E0.01827
G1 X89.963 Y110.676 E0.07244
G1 X89.963 Y110.076 E0.01827
G1 X92.246 Y112.359 E0.09828
G1 X92.846 Y112.359 E0.01827
G1 X89.963 Y109.476 E0.12412
G1 X89.963 Y109.272 E0.00622
G1 X90.359 Y109.272 E0.01205
G1 X93.447 Y112.359 E0.13291
G1 X94.047 Y112.359 E0.01827
G1 X90.959 Y109.272 E0.13291
G1 X91.559 Y109.272 E0.01827
G1 X94.647 Y112.359 E0.13291
G1 X95.247 Y112.359 E0.01827
G1 X92.160 Y109.272 E0.13291
G1 X92.760 Y109.272 E0.01827
G1 X95.847 Y112.359 E0.13291
```

G1 X96.448 Y112.359 E0.01827

G1 X93.360 Y109.272 E0.13291

G1 X93.960 Y109.272 E0.01827

G1 X97.048 Y112.359 E0.13291

G1 X97.648 Y112.359 E0.01827

G1 X94.560 Y109.272 E0.13291

G1 X95.161 Y109.272 E0.01827

G1 X98.276 Y112.387 E0.13409

G1 X98.701 Y112.413 E0.01297

G1 X98.949 Y112.459 E0.00767

G1 X95.761 Y109.272 E0.13722

G1 X96.361 Y109.272 E0.01827

G1 X99.706 Y112.617 E0.14400

G1 X100.444 Y112.852 E0.02358

G1 X100.625 Y112.935 E0.00603

G1 X96.961 Y109.272 E0.15769

G1 X97.562 Y109.272 E0.01827

G1 X105.649 Y117.359 E0.34814

G1 X106.249 Y117.359 E0.01827

G1 X98.143 Y109.252 E0.34897

G1 X98.707 Y109.217 E0.01722

G1 X106.850 Y117.359 E0.35050

G1 X107.450 Y117.359 E0.01827

G1 X99.213 Y109.122 E0.35457

G1 X99.705 Y109.014 E0.01534

G1 X108.050 Y117.359 E0.35921

G1 X108.650 Y117.359 E0.01827

G1 X100.160 Y108.870 E0.36545

G1 X100.444 Y108.779 E0.00907

G1 X100.599 Y108.708 E0.00518

G1 X109.250 Y117.359 E0.37241

M73 P72 R19

G1 X109.851 Y117.359 E0.01827

G1 X101.010 Y108.519 E0.38056

G1 X101.263 Y108.402 E0.00847

G1 X101.405 Y108.314 E0.00511

G1 X110.451 Y117.359 E0.38938

G1 X111.051 Y117.359 E0.01825

G1 X101.776 Y108.084 E0.39926

G1 X102.134 Y107.842 E0.01316

G1 X111.051 Y116.759 E0.38382

G1 X111.051 Y116.158 E0.01827

G1 X102.467 Y107.574 E0.36951

G1 X102.791 Y107.299 E0.01296

G1 X111.051 Y115.558 E0.35554

G1 X111.051 Y114.958 E0.01827

G1 X103.086 Y106.994 E0.34283

G1 X103.378 Y106.686 E0.01292

G1 X111.051 Y114.358 E0.33026

G1 X111.051 Y113.758 E0.01827

G1 X103.636 Y106.343 E0.31916

G1 X103.894 Y106.001 E0.01305

G1 X111.051 Y113.157 E0.30805

G1 X111.051 Y112.557 E0.01827

G1 X104.114 Y105.621 E0.29859

G1 X104.334 Y105.240 E0.01338

G1 X111.051 Y111.957 E0.28914

G1 X111.051 Y111.357 E0.01827

```
G1 X104.514 Y104.820 E0.28137
G1 X104.692 Y104.398 E0.01395
G1 X111.051 Y110.756 E0.27370
G1 X111.051 Y110.156 E0.01827
G1 X105.166 Y104.272 E0.25331
G1 X105.766 Y104.272 E0.01827
G1 X111.051 Y109.556 E0.22747
G1 X111.051 Y108.956 E0.01827
G1 X106.367 Y104.272 E0.20163
G1 X106.967 Y104.272 E0.01827
G1 X111.051 Y108.356 E0.17580
G1 X111.051 Y107.755 E0.01827
G1 X107.567 Y104.272 E0.14996
G1 X108.167 Y104.272 E0.01827
G1 X111.051 Y107.155 E0.12412
G1 X111.051 Y106.555 E0.01827
G1 X108.767 Y104.272 E0.09828
G1 X109.368 Y104.272 E0.01827
G1 X111.051 Y105.955 E0.07245
G1 X111.051 Y105.355 E0.01827
G1 X109.968 Y104.272 E0.04661
G1 X110.568 Y104.272 E0.01827
G1 X111.239 Y104.942 E0.02887
M204 S1250
G1 E-4.00000 F2400.000
G1 X104.165 Y116.476 F9000.000
G1 E4.00000 F900.000
M204 S1000
G1 F1272.328
```

```
G1 X105.237 Y117.547 E0.04614
M204 S1250
; stop printing object Petg print.STL id:2 copy 0
; printing object Petg print.STL id:6 copy 0
G1 X104.478 Y122.612 F9000.000
M204 S800
;TYPE:Perimeter
;WIDTH:0.45
G1 F1292.454
G1 X111.349 Y122.612 E0.20589
G1 X111.349 Y136.327 E0.41094
G1 X104.478 Y136.327 E0.20589
G1 X104.386 Y136.003 E0.01007
G1 X104.051 Y135.209 E0.02583
G1 X103.621 Y134.462 E0.02583
G1 X103.102 Y133.774 E0.02583
G1 X102.503 Y133.154 E0.02583
G1 X101.831 Y132.613 E0.02583
G1 X101.099 Y132.159 E0.02583
G1 X100.316 Y131.799 E0.02583
G1 X99.495 Y131.537 E0.02583
G1 X98.647 Y131.379 E0.02583
G1 X97.807 Y131.327 E0.02523
G1 X89.635 Y131.327 E0.24487
G1 X89.635 Y127.612 E0.11130
G1 X97.807 Y127.612 E0.24487
G1 X98.647 Y127.560 E0.02523
G1 X99.495 Y127.402 E0.02583
G1 X100.316 Y127.140 E0.02583
```

```
G1 X101.099 Y126.780 E0.02583
G1 X101.831 Y126.325 E0.02583
G1 X102.503 Y125.785 E0.02583
G1 X103.102 Y125.165 E0.02583
G1 X103.621 Y124.477 E0.02583
G1 X104.051 Y123.730 E0.02583
G1 X104.386 Y122.936 E0.02583
G1 X104.461 Y122.670 E0.00828
M204 S1250
G1 X104.162 Y122.194 F9000.000
M204 S800
;TYPE:External perimeter
G1 F1292.454
G1 X111.767 Y122.194 E0.22787
G1 X111.767 Y136.744 E0.43598
G1 X104.162 Y136.744 E0.22787
G1 X103.991 Y136.142 E0.01877
G1 X103.676 Y135.395 E0.02428
G1 X103.272 Y134.693 E0.02428
G1 X102.784 Y134.046 E0.02428
G1 X102.220 Y133.463 E0.02428
G1 X101.589 Y132.955 E0.02428
G1 X100.901 Y132.528 E0.02428
G1 X100.164 Y132.189 E0.02428
G1 X99.392 Y131.943 E0.02428
G1 X98.596 Y131.794 E0.02428
G1 X97.794 Y131.744 E0.02407
G1 X89.217 Y131.744 E0.25701
G1 X89.217 Y127.194 E0.13634
```

```
G1 X97.794 Y127.194 E0.25701
G1 X98.596 Y127.145 E0.02407
G1 X99.392 Y126.996 E0.02428
G1 X100.164 Y126.750 E0.02428
G1 X100.901 Y126.411 E0.02428
G1 X101.589 Y125.984 E0.02428
G1 X102.220 Y125.475 E0.02428
G1 X102.784 Y124.893 E0.02428
G1 X103.272 Y124.246 E0.02428
G1 X103.676 Y123.544 E0.02428
G1 X103.991 Y122.797 E0.02428
G1 X104.146 Y122.252 E0.01697
M204 S1250
G1 X104.483 Y122.433 F9000.000
G1 E-4.00000 F2400.000
G1 X111.224 Y123.596 F9000.000
G1 E4.00000 F900.000
M204 S1000
;TYPE:Solid infill
;WIDTH:0.456609
G1 F1272.328
G1 X110.553 Y122.926 E0.02887
G1 X109.953 Y122.926 E0.01827
G1 X111.036 Y124.008 E0.04661
G1 X111.036 Y124.609 E0.01827
G1 X109.353 Y122.926 E0.07245
G1 X108.752 Y122.926 E0.01827
G1 X111.036 Y125.209 E0.09828
G1 X111.036 Y125.809 E0.01827
```

```
G1 X108.152 Y122.926 E0.12412
G1 X107.552 Y122.926 E0.01827
G1 X111.036 Y126.409 E0.14996
G1 X111.036 Y127.010 E0.01827
G1 X106.952 Y122.926 E0.17580
G1 X106.351 Y122.926 E0.01827
G1 X111.036 Y127.610 E0.20163
G1 X111.036 Y128.210 E0.01827
G1 X105.751 Y122.926 E0.22747
G1 X105.151 Y122.926 E0.01827
G1 X111.036 Y128.810 E0.25331
G1 X111.036 Y129.410 E0.01827
G1 X104.677 Y123.052 E0.27370
G1 X104.499 Y123.474 E0.01395
G1 X111.036 Y130.011 E0.28137
G1 X111.036 Y130.611 E0.01827
G1 X104.318 Y123.894 E0.28914
G1 X104.099 Y124.275 E0.01338
G1 X111.036 Y131.211 E0.29859
G1 X111.036 Y131.811 E0.01827
G1 X103.879 Y124.655 E0.30805
G1 X103.621 Y124.997 E0.01305
G1 X111.036 Y132.412 E0.31916
G1 X111.036 Y133.012 E0.01827
G1 X103.363 Y125.339 E0.33026
G1 X103.071 Y125.648 E0.01292
G1 X111.036 Y133.612 E0.34283
G1 X111.036 Y134.212 E0.01827
G1 X102.776 Y125.953 E0.35554
```

G1 X102.451 Y126.228 E0.01296

G1 X111.036 Y134.812 E0.36951

G1 X111.036 Y135.413 E0.01827

G1 X102.119 Y126.496 E0.38382

G1 X101.761 Y126.738 E0.01316

G1 X111.036 Y136.013 E0.39926

G1 X110.436 Y136.013 E0.01825

G1 X101.390 Y126.968 E0.38938

G1 X101.247 Y127.056 E0.00511

G1 X100.995 Y127.173 E0.00847

G1 X109.836 Y136.013 E0.38056

G1 X109.235 Y136.013 E0.01827

G1 X100.584 Y127.362 E0.37241

G1 X100.429 Y127.433 E0.00518

G1 X100.145 Y127.523 E0.00907

G1 X108.635 Y136.013 E0.36545

G1 X108.035 Y136.013 E0.01827

G1 X99.690 Y127.668 E0.35921

G1 X99.198 Y127.776 E0.01534

G1 X107.435 Y136.013 E0.35457

G1 X106.835 Y136.013 E0.01827

G1 X98.692 Y127.871 E0.35050

G1 X98.127 Y127.906 E0.01722

G1 X106.234 Y136.013 E0.34897

G1 X105.634 Y136.013 E0.01827

G1 X97.546 Y127.926 E0.34814

G1 X96.946 Y127.926 E0.01827

G1 X100.609 Y131.589 E0.15769

G1 X100.429 Y131.506 E0.00603

G1 X99.691 Y131.271 E0.02358

G1 X96.346 Y127.926 E0.14400

G1 X95.746 Y127.926 E0.01827

G1 X98.933 Y131.113 E0.13722

G1 X98.686 Y131.067 E0.00767

G1 X98.261 Y131.041 E0.01297

G1 X95.146 Y127.926 E0.13409

G1 X94.545 Y127.926 E0.01827

G1 X97.633 Y131.013 E0.13291

G1 X97.033 Y131.013 E0.01827

G1 X93.945 Y127.926 E0.13291

G1 X93.345 Y127.926 E0.01827

G1 X96.433 Y131.013 E0.13291

G1 X95.832 Y131.013 E0.01827

G1 X92.745 Y127.926 E0.13291

G1 X92.144 Y127.926 E0.01827

G1 X95.232 Y131.013 E0.13291

G1 X94.632 Y131.013 E0.01827

G1 X91.544 Y127.926 E0.13291

G1 X90.944 Y127.926 E0.01827

G1 X94.032 Y131.013 E0.13291

G1 X93.431 Y131.013 E0.01827

G1 X90.344 Y127.926 E0.13291

G1 X89.948 Y127.926 E0.01205

G1 X89.948 Y128.130 E0.00622

G1 X92.831 Y131.013 E0.12412

G1 X92.231 Y131.013 E0.01827

G1 X89.948 Y128.730 E0.09828

G1 X89.948 Y129.330 E0.01827

```
G1 X91.631 Y131.013 E0.07244
G1 X91.031 Y131.013 E0.01827
G1 X89.948 Y129.931 E0.04660
M73 P72 R18
G1 X89.948 Y130.531 E0.01827
G1 X90.618 Y131.201 E0.02886
M204 S1250
G1 E-4.00000 F2400.000
G1 X104.150 Y135.130 F9000.000
G1 E4.00000 F900.000
M204 S1000
G1 F1272.328
G1 X105.222 Y136.201 E0.04614
M204 S1250
; stop printing object Petg print.STL id:6 copy 0
; printing object tpu print.STL id:27 copy 0
; stop printing object tpu print.STL id:27 copy 0
; printing object tpu print.STL id:23 copy 0
; stop printing object tpu print.STL id:23 copy 0
; printing object tpu print.STL id:21 copy 0
; stop printing object tpu print.STL id:21 copy 0
; printing object Petg print.STL id:20 copy 0
G1 E-4.00000 F2400.000
G1 X141.293 Y92.032 F9000.000
G1 E4.00000 F900.000
M204 S800
;TYPE:Perimeter
;WIDTH:0.45
G1 F1292.454
```

```
G1 X141.293 Y88.318 E0.11130
G1 X149.465 Y88.318 E0.24487
G1 X150.306 Y88.266 E0.02523
G1 X151.153 Y88.107 E0.02583
G1 X151.974 Y87.846 E0.02583
G1 X152.757 Y87.485 E0.02583
G1 X153.490 Y87.031 E0.02583
G1 X154.161 Y86.490 E0.02583
G1 X154.761 Y85.871 E0.02583
G1 X155.279 Y85.183 E0.02583
G1 X155.710 Y84.436 E0.02583
G1 X156.045 Y83.641 E0.02583
G1 X156.136 Y83.318 E0.01007
G1 X163.007 Y83.318 E0.20589
G1 X163.007 Y97.032 E0.41094
G1 X156.136 Y97.032 E0.20589
G1 X156.045 Y96.709 E0.01007
G1 X155.710 Y95.915 E0.02583
G1 X155.279 Y95.168 E0.02583
G1 X154.761 Y94.479 E0.02583
G1 X154.161 Y93.860 E0.02583
G1 X153.490 Y93.319 E0.02583
G1 X152.757 Y92.865 E0.02583
G1 X151.974 Y92.504 E0.02583
G1 X151.153 Y92.243 E0.02583
G1 X150.306 Y92.084 E0.02583
G1 X149.465 Y92.032 E0.02523
G1 X141.353 Y92.032 E0.24307
M204 S1250
```

```
M73 P73 R18
G1 X140.875 Y92.450 F9000.000
M204 S800
;TYPE:External perimeter
G1 F1292.454
G1 X140.875 Y87.900 E0.13634
G1 X149.452 Y87.900 E0.25701
G1 X150.254 Y87.851 E0.02407
G1 X151.051 Y87.702 E0.02428
G1 X151.823 Y87.456 E0.02428
G1 X152.559 Y87.117 E0.02428
G1 X153.248 Y86.690 E0.02428
G1 X153.879 Y86.181 E0.02428
G1 X154.443 Y85.599 E0.02428
G1 X154.930 Y84.952 E0.02428
G1 X155.335 Y84.249 E0.02428
G1 X155.650 Y83.503 E0.02428
G1 X155.820 Y82.900 E0.01877
G1 X163.425 Y82.900 E0.22787
G1 X163.425 Y97.450 E0.43598
G1 X155.820 Y97.450 E0.22787
G1 X155.650 Y96.847 E0.01877
G1 X155.335 Y96.101 E0.02428
G1 X154.930 Y95.398 E0.02428
G1 X154.443 Y94.751 E0.02428
G1 X153.879 Y94.169 E0.02428
G1 X153.248 Y93.661 E0.02428
G1 X152.559 Y93.234 E0.02428
G1 X151.823 Y92.895 E0.02428
```

```
G1 X151.051 Y92.649 E0.02428
G1 X150.254 Y92.500 E0.02428
G1 X149.452 Y92.450 E0.02407
G1 X140.935 Y92.450 E0.25521
M204 S1250
G1 X141.076 Y92.104 F9000.000
G1 X142.277 Y91.907
M204 S1000
;TYPE:Solid infill
;WIDTH:0.456609
G1 F1272.328
G1 X141.606 Y91.236 E0.02886
G1 X141.606 Y90.636 E0.01827
G1 X142.689 Y91.719 E0.04660
G1 X143.289 Y91.719 E0.01827
G1 X141.606 Y90.036 E0.07244
G1 X141.606 Y89.436 E0.01827
G1 X143.890 Y91.719 E0.09828
G1 X144.490 Y91.719 E0.01827
G1 X141.606 Y88.836 E0.12412
G1 X141.606 Y88.631 E0.00622
G1 X142.002 Y88.631 E0.01205
G1 X145.090 Y91.719 E0.13291
G1 X145.690 Y91.719 E0.01827
G1 X142.603 Y88.631 E0.13291
G1 X143.203 Y88.631 E0.01827
G1 X146.290 Y91.719 E0.13291
G1 X146.891 Y91.719 E0.01827
G1 X143.803 Y88.631 E0.13291
```

```
G1 X144.403 Y88.631 E0.01827
G1 X147.491 Y91.719 E0.13291
G1 X148.091 Y91.719 E0.01827
G1 X145.003 Y88.631 E0.13291
G1 X145.604 Y88.631 E0.01827
G1 X148.691 Y91.719 E0.13291
G1 X149.291 Y91.719 E0.01827
G1 X146.204 Y88.631 E0.13291
G1 X146.804 Y88.631 E0.01827
G1 X149.919 Y91.746 E0.13409
G1 X150.344 Y91.773 E0.01297
G1 X150.592 Y91.819 E0.00767
G1 X147.404 Y88.631 E0.13722
G1 X148.004 Y88.631 E0.01827
G1 X151.350 Y91.976 E0.14400
G1 X152.088 Y92.212 E0.02358
G1 X152.268 Y92.295 E0.00603
G1 X148.605 Y88.631 E0.15769
G1 X149.205 Y88.631 E0.01827
G1 X157.293 Y96.719 E0.34814
G1 X157.893 Y96.719 E0.01827
G1 X149.786 Y88.612 E0.34897
G1 X150.351 Y88.576 E0.01722
G1 X158.493 Y96.719 E0.35050
G1 X159.093 Y96.719 E0.01827
G1 X150.856 Y88.482 E0.35457
G1 X151.349 Y88.374 E0.01534
G1 X159.693 Y96.719 E0.35921
G1 X160.294 Y96.719 E0.01827
```

```
G1 X151.804 Y88.229 E0.36545
G1 X152.088 Y88.139 E0.00907
G1 X152.242 Y88.067 E0.00518
G1 X160.894 Y96.719 E0.37241
G1 X161.494 Y96.719 E0.01827
G1 X152.653 Y87.878 E0.38056
G1 X152.906 Y87.762 E0.00847
G1 X153.049 Y87.673 E0.00511
G1 X162.094 Y96.719 E0.38938
G1 X162.694 Y96.719 E0.01825
G1 X153.419 Y87.444 E0.39926
G1 X153.778 Y87.202 E0.01316
G1 X162.694 Y96.118 E0.38382
G1 X162.694 Y95.518 E0.01827
G1 X154.110 Y86.934 E0.36951
G1 X154.435 Y86.658 E0.01296
G1 X162.694 Y94.918 E0.35554
G1 X162.694 Y94.318 E0.01827
G1 X154.730 Y86.353 E0.34283
G1 X155.022 Y86.045 E0.01292
G1 X162.694 Y93.717 E0.33026
G1 X162.694 Y93.117 E0.01827
G1 X155.280 Y85.703 E0.31916
G1 X155.538 Y85.361 E0.01305
G1 X162.694 Y92.517 E0.30805
G1 X162.694 Y91.917 E0.01827
G1 X155.758 Y84.980 E0.29859
G1 X155.977 Y84.599 E0.01338
G1 X162.694 Y91.316 E0.28914
```

```
G1 X162.694 Y90.716 E0.01827
G1 X156.158 Y84.180 E0.28137
G1 X156.336 Y83.758 E0.01395
G1 X162.694 Y90.116 E0.27370
G1 X162.694 Y89.516 E0.01827
G1 X156.810 Y83.631 E0.25331
G1 X157.410 Y83.631 E0.01827
G1 X162.694 Y88.916 E0.22747
G1 X162.694 Y88.315 E0.01827
G1 X158.010 Y83.631 E0.20163
G1 X158.610 Y83.631 E0.01827
G1 X162.694 Y87.715 E0.17580
G1 X162.694 Y87.115 E0.01827
G1 X159.210 Y83.631 E0.14996
G1 X159.811 Y83.631 E0.01827
G1 X162.694 Y86.515 E0.12412
G1 X162.694 Y85.914 E0.01827
G1 X160.411 Y83.631 E0.09828
G1 X161.011 Y83.631 E0.01827
G1 X162.694 Y85.314 E0.07245
G1 X162.694 Y84.714 E0.01827
G1 X161.611 Y83.631 E0.04661
G1 X162.211 Y83.631 E0.01827
G1 X162.882 Y84.302 E0.02887
M204 S1250
G1 E-4.00000 F2400.000
G1 X155.809 Y95.835 F9000.000
G1 E4.00000 F900.000
M204 S1000
```

```
G1 F1272.328
G1 X156.880 Y96.907 E0.04614
M204 S1250
; stop printing object Petg print.STL id:20 copy 0
; printing object Petg print.STL id:22 copy 0
G1 E-4.00000 F2400.000
G1 X156.143 Y104.133 F9000.000
G1 E4.00000 F900.000
M204 S800
;TYPE:Perimeter
;WIDTH:0.45
G1 F1292.454
G1 X163.014 Y104.133 E0.20589
G1 X163.014 Y117.848 E0.41094
G1 X156.143 Y117.848 E0.20589
G1 X156.052 Y117.524 E0.01007
G1 X155.717 Y116.730 E0.02583
G1 X155.286 Y115.983 E0.02583
G1 X154.768 Y115.295 E0.02583
G1 X154.168 Y114.675 E0.02583
G1 X153.497 Y114.135 E0.02583
G1 X152.764 Y113.680 E0.02583
G1 X151.981 Y113.320 E0.02583
G1 X151.160 Y113.058 E0.02583
G1 X150.313 Y112.900 E0.02583
G1 X149.472 Y112.848 E0.02523
G1 X141.300 Y112.848 E0.24487
G1 X141.300 Y109.133 E0.11130
G1 X149.472 Y109.133 E0.24487
```

```
G1 X150.313 Y109.082 E0.02523
G1 X151.160 Y108.923 E0.02583
G1 X151.981 Y108.661 E0.02583
G1 X152.764 Y108.301 E0.02583
G1 X153.497 Y107.847 E0.02583
G1 X154.168 Y107.306 E0.02583
G1 X154.768 Y106.686 E0.02583
G1 X155.286 Y105.998 E0.02583
G1 X155.717 Y105.251 E0.02583
G1 X156.052 Y104.457 E0.02583
G1 X156.127 Y104.191 E0.00828
M204 S1250
G1 X155.827 Y103.716 F9000.000
M204 S800
;TYPE:External perimeter
G1 F1292.454
G1 X163.432 Y103.716 E0.22787
G1 X163.432 Y118.266 E0.43598
G1 X155.827 Y118.266 E0.22787
G1 X155.657 Y117.663 E0.01877
G1 X155.342 Y116.916 E0.02428
G1 X154.937 Y116.214 E0.02428
G1 X154.449 Y115.567 E0.02428
G1 X153.886 Y114.985 E0.02428
G1 X153.255 Y114.476 E0.02428
G1 X152.566 Y114.049 E0.02428
G1 X151.830 Y113.710 E0.02428
G1 X151.058 Y113.464 E0.02428
G1 X150.261 Y113.315 E0.02428
```

```
G1 X149.459 Y113.266 E0.02407
G1 X140.882 Y113.266 E0.25701
G1 X140.882 Y108.716 E0.13634
G1 X149.459 Y108.716 E0.25701
G1 X150.261 Y108.666 E0.02407
G1 X151.058 Y108.517 E0.02428
G1 X151.830 Y108.271 E0.02428
G1 X152.566 Y107.932 E0.02428
G1 X153.255 Y107.505 E0.02428
G1 X153.886 Y106.997 E0.02428
G1 X154.449 Y106.414 E0.02428
G1 X154.937 Y105.767 E0.02428
G1 X155.342 Y105.065 E0.02428
G1 X155.657 Y104.318 E0.02428
G1 X155.811 Y103.773 E0.01697
M204 S1250
G1 X156.148 Y103.954 F9000.000
G1 E-4.00000 F2400.000
G1 X162.889 Y105.117 F9000.000
G1 E4.00000 F900.000
M204 S1000
;TYPE:Solid infill
;WIDTH:0.456609
G1 F1272.328
G1 X162.218 Y104.447 E0.02887
G1 X161.618 Y104.447 E0.01827
G1 X162.701 Y105.530 E0.04661
G1 X162.701 Y106.130 E0.01827
G1 X161.018 Y104.447 E0.07245
```

G1 X160.418 Y104.447 E0.01827

G1 X162.701 Y106.730 E0.09828

G1 X162.701 Y107.330 E0.01827

G1 X159.817 Y104.447 E0.12412

G1 X159.217 Y104.447 E0.01827

G1 X162.701 Y107.930 E0.14996

G1 X162.701 Y108.531 E0.01827

G1 X158.617 Y104.447 E0.17580

G1 X158.017 Y104.447 E0.01827

G1 X162.701 Y109.131 E0.20163

G1 X162.701 Y109.731 E0.01827

G1 X157.417 Y104.447 E0.22747

G1 X156.816 Y104.447 E0.01827

G1 X162.701 Y110.331 E0.25331

G1 X162.701 Y110.932 E0.01827

G1 X156.343 Y104.573 E0.27370

G1 X156.165 Y104.995 E0.01395

G1 X162.701 Y111.532 E0.28137

G1 X162.701 Y112.132 E0.01827

G1 X155.984 Y105.415 E0.28914

G1 X155.764 Y105.796 E0.01338

G1 X162.701 Y112.732 E0.29859

G1 X162.701 Y113.332 E0.01827

G1 X155.545 Y106.176 E0.30805

G1 X155.287 Y106.518 E0.01305

G1 X162.701 Y113.933 E0.31916

G1 X162.701 Y114.533 E0.01827

G1 X155.029 Y106.861 E0.33026

G1 X154.737 Y107.169 E0.01292

```
G1 X162.701 Y115.133 E0.34283
G1 X162.701 Y115.733 E0.01827
G1 X154.441 Y107.474 E0.35554
G1 X154.117 Y107.749 E0.01296
G1 X162.701 Y116.334 E0.36951
G1 X162.701 Y116.934 E0.01827
G1 X153.784 Y108.017 E0.38382
G1 X153.426 Y108.259 E0.01316
G1 X162.701 Y117.534 E0.39926
G1 X162.101 Y117.534 E0.01825
G1 X153.056 Y108.489 E0.38938
G1 X152.913 Y108.577 E0.00511
G1 X152.660 Y108.694 E0.00847
G1 X161.501 Y117.534 E0.38056
G1 X160.901 Y117.534 E0.01827
G1 X152.249 Y108.883 E0.37241
M73 P74 R18
G1 X152.095 Y108.954 E0.00518
G1 X151.811 Y109.045 E0.00907
G1 X160.301 Y117.534 E0.36545
G1 X159.700 Y117.534 E0.01827
G1 X151.355 Y109.190 E0.35921
G1 X150.863 Y109.297 E0.01534
G1 X159.100 Y117.534 E0.35457
G1 X158.500 Y117.534 E0.01827
G1 X150.357 Y109.392 E0.35050
G1 X149.793 Y109.428 E0.01722
G1 X157.900 Y117.534 E0.34897
G1 X157.299 Y117.534 E0.01827
```

```
G1 X149.212 Y109.447 E0.34814
G1 X148.612 Y109.447 E0.01827
G1 X152.275 Y113.110 E0.15769
G1 X152.095 Y113.027 E0.00603
G1 X151.357 Y112.792 E0.02358
G1 X148.011 Y109.447 E0.14400
G1 X147.411 Y109.447 E0.01827
G1 X150.599 Y112.634 E0.13722
G1 X150.351 Y112.588 E0.00767
G1 X149.926 Y112.562 E0.01297
G1 X146.811 Y109.447 E0.13409
G1 X146.211 Y109.447 E0.01827
G1 X149.298 Y112.534 E0.13291
G1 X148.698 Y112.534 E0.01827
G1 X145.610 Y109.447 E0.13291
G1 X145.010 Y109.447 E0.01827
G1 X148.098 Y112.534 E0.13291
G1 X147.498 Y112.534 E0.01827
G1 X144.410 Y109.447 E0.13291
G1 X143.810 Y109.447 E0.01827
G1 X146.897 Y112.534 E0.13291
G1 X146.297 Y112.534 E0.01827
G1 X143.210 Y109.447 E0.13291
G1 X142.609 Y109.447 E0.01827
G1 X145.697 Y112.534 E0.13291
G1 X145.097 Y112.534 E0.01827
G1 X142.009 Y109.447 E0.13291
G1 X141.613 Y109.447 E0.01205
G1 X141.613 Y109.651 E0.00622
```

```
G1 X144.497 Y112.534 E0.12412
G1 X143.896 Y112.534 E0.01827
G1 X141.613 Y110.251 E0.09828
G1 X141.613 Y110.852 E0.01827
G1 X143.296 Y112.534 E0.07244
G1 X142.696 Y112.534 E0.01827
G1 X141.613 Y111.452 E0.04660
G1 X141.613 Y112.052 E0.01827
G1 X142.284 Y112.722 E0.02886
M204 S1250
G1 E-4.00000 F2400.000
G1 X155.815 Y116.651 F9000.000
G1 E4.00000 F900.000
M204 S1000
G1 F1272.328
G1 X156.887 Y117.722 E0.04614
M204 S1250
; stop printing object Petg print.STL id:22 copy 0
; printing object Petg print.STL id:26 copy 0
G1 X156.128 Y122.787 F9000.000
M204 S800
;TYPE:Perimeter
;WIDTH:0.45
G1 F1292.454
G1 X162.999 Y122.787 E0.20589
G1 X162.999 Y136.502 E0.41094
G1 X156.128 Y136.502 E0.20589
G1 X156.036 Y136.178 E0.01007
G1 X155.701 Y135.384 E0.02583
```

```
G1 X155.271 Y134.637 E0.02583
G1 X154.752 Y133.949 E0.02583
G1 X154.153 Y133.329 E0.02583
G1 X153.482 Y132.789 E0.02583
G1 X152.749 Y132.334 E0.02583
G1 X151.966 Y131.974 E0.02583
G1 X151.145 Y131.712 E0.02583
G1 X150.297 Y131.554 E0.02583
G1 X149.457 Y131.502 E0.02523
G1 X141.285 Y131.502 E0.24487
G1 X141.285 Y127.787 E0.11130
G1 X149.457 Y127.787 E0.24487
G1 X150.297 Y127.735 E0.02523
G1 X151.145 Y127.577 E0.02583
G1 X151.966 Y127.315 E0.02583
G1 X152.749 Y126.955 E0.02583
G1 X153.482 Y126.501 E0.02583
G1 X154.153 Y125.960 E0.02583
G1 X154.752 Y125.340 E0.02583
G1 X155.271 Y124.652 E0.02583
G1 X155.701 Y123.905 E0.02583
G1 X156.036 Y123.111 E0.02583
G1 X156.112 Y122.845 E0.00828
M204 S1250
G1 X155.812 Y122.370 F9000.000
M204 S800
;TYPE:External perimeter
G1 F1292.454
G1 X163.417 Y122.370 E0.22787
```

```
G1 X163.417 Y136.920 E0.43598
G1 X155.812 Y136.920 E0.22787
G1 X155.641 Y136.317 E0.01877
G1 X155.326 Y135.570 E0.02428
G1 X154.922 Y134.868 E0.02428
G1 X154.434 Y134.221 E0.02428
G1 X153.871 Y133.639 E0.02428
G1 X153.240 Y133.130 E0.02428
G1 X152.551 Y132.703 E0.02428
G1 X151.815 Y132.364 E0.02428
G1 X151.043 Y132.118 E0.02428
G1 X150.246 Y131.969 E0.02428
G1 X149.444 Y131.920 E0.02407
G1 X140.867 Y131.920 E0.25701
G1 X140.867 Y127.370 E0.13634
G1 X149.444 Y127.370 E0.25701
G1 X150.246 Y127.320 E0.02407
G1 X151.043 Y127.171 E0.02428
G1 X151.815 Y126.925 E0.02428
G1 X152.551 Y126.586 E0.02428
G1 X153.240 Y126.159 E0.02428
G1 X153.871 Y125.651 E0.02428
G1 X154.434 Y125.068 E0.02428
G1 X154.922 Y124.421 E0.02428
G1 X155.326 Y123.719 E0.02428
G1 X155.641 Y122.972 E0.02428
M73 P74 R17
G1 X155.796 Y122.427 E0.01697
M204 S1250
```

```
G1 X156.133 Y122.608 F9000.000
G1 E-4.00000 F2400.000
G1 X162.874 Y123.771 F9000.000
G1 E4.00000 F900.000
M204 S1000
;TYPE:Solid infill
;WIDTH:0.456609
G1 F1272.328
G1 X162.203 Y123.101 E0.02887
G1 X161.603 Y123.101 E0.01827
G1 X162.686 Y124.184 E0.04661
G1 X162.686 Y124.784 E0.01827
G1 X161.003 Y123.101 E0.07245
G1 X160.403 Y123.101 E0.01827
G1 X162.686 Y125.384 E0.09828
G1 X162.686 Y125.984 E0.01827
G1 X159.802 Y123.101 E0.12412
G1 X159.202 Y123.101 E0.01827
G1 X162.686 Y126.584 E0.14996
G1 X162.686 Y127.185 E0.01827
G1 X158.602 Y123.101 E0.17580
G1 X158.002 Y123.101 E0.01827
G1 X162.686 Y127.785 E0.20163
G1 X162.686 Y128.385 E0.01827
G1 X157.401 Y123.101 E0.22747
G1 X156.801 Y123.101 E0.01827
G1 X162.686 Y128.985 E0.25331
G1 X162.686 Y129.585 E0.01827
G1 X156.327 Y123.227 E0.27370
```

```
G1 X156.149 Y123.649 E0.01395
G1 X162.686 Y130.186 E0.28137
G1 X162.686 Y130.786 E0.01827
G1 X155.969 Y124.069 E0.28914
G1 X155.749 Y124.450 E0.01338
G1 X162.686 Y131.386 E0.29859
G1 X162.686 Y131.986 E0.01827
G1 X155.529 Y124.830 E0.30805
G1 X155.271 Y125.172 E0.01305
G1 X162.686 Y132.587 E0.31916
G1 X162.686 Y133.187 E0.01827
G1 X155.014 Y125.515 E0.33026
G1 X154.722 Y125.823 E0.01292
G1 X162.686 Y133.787 E0.34283
G1 X162.686 Y134.387 E0.01827
G1 X154.426 Y126.128 E0.35554
G1 X154.102 Y126.403 E0.01296
G1 X162.686 Y134.987 E0.36951
G1 X162.686 Y135.588 E0.01827
G1 X153.769 Y126.671 E0.38382
G1 X153.411 Y126.913 E0.01316
G1 X162.686 Y136.188 E0.39926
G1 X162.086 Y136.188 E0.01825
G1 X153.040 Y127.143 E0.38938
G1 X152.898 Y127.231 E0.00511
G1 X152.645 Y127.348 E0.00847
G1 X161.486 Y136.188 E0.38056
G1 X160.886 Y136.188 E0.01827
G1 X152.234 Y127.537 E0.37241
```

```
G1 X152.080 Y127.608 E0.00518
G1 X151.796 Y127.699 E0.00907
G1 X160.285 Y136.188 E0.36545
G1 X159.685 Y136.188 E0.01827
G1 X151.340 Y127.844 E0.35921
G1 X150.848 Y127.951 E0.01534
G1 X159.085 Y136.188 E0.35457
G1 X158.485 Y136.188 E0.01827
G1 X150.342 Y128.046 E0.35050
G1 X149.778 Y128.082 E0.01722
G1 X157.885 Y136.188 E0.34897
G1 X157.284 Y136.188 E0.01827
G1 X149.197 Y128.101 E0.34814
G1 X148.596 Y128.101 E0.01827
G1 X152.260 Y131.764 E0.15769
G1 X152.080 Y131.681 E0.00603
G1 X151.341 Y131.446 E0.02358
G1 X147.996 Y128.101 E0.14400
G1 X147.396 Y128.101 E0.01827
G1 X150.584 Y131.288 E0.13722
G1 X150.336 Y131.242 E0.00767
G1 X149.911 Y131.216 E0.01297
G1 X146.796 Y128.101 E0.13409
G1 X146.196 Y128.101 E0.01827
G1 X149.283 Y131.188 E0.13291
G1 X148.683 Y131.188 E0.01827
G1 X145.595 Y128.101 E0.13291
G1 X144.995 Y128.101 E0.01827
G1 X148.083 Y131.188 E0.13291
```

```
G1 X147.483 Y131.188 E0.01827
G1 X144.395 Y128.101 E0.13291
G1 X143.795 Y128.101 E0.01827
G1 X146.882 Y131.188 E0.13291
G1 X146.282 Y131.188 E0.01827
G1 X143.194 Y128.101 E0.13291
G1 X142.594 Y128.101 E0.01827
G1 X145.682 Y131.188 E0.13291
G1 X145.082 Y131.188 E0.01827
G1 X141.994 Y128.101 E0.13291
G1 X141.598 Y128.101 E0.01205
G1 X141.598 Y128.305 E0.00622
G1 X144.481 Y131.188 E0.12412
G1 X143.881 Y131.188 E0.01827
G1 X141.598 Y128.905 E0.09828
G1 X141.598 Y129.505 E0.01827
G1 X143.281 Y131.188 E0.07244
G1 X142.681 Y131.188 E0.01827
G1 X141.598 Y130.106 E0.04660
G1 X141.598 Y130.706 E0.01827
G1 X142.269 Y131.376 E0.02886
M204 S1250
G1 E-4.00000 F2400.000
G1 X155.800 Y135.305 F9000.000
G1 E4.00000 F900.000
M204 S1000
G1 F1272.328
G1 X156.872 Y136.376 E0.04614
M204 S1250
```

```
; stop printing object Petg print.STL id:26 copy 0
; printing object Petg print.STL id:0 copy 0
G1 E-4.00000 F2400.000
G1 X111.357 Y96.857 F9000.000
G1 E4.00000 F900.000
M204 S800
;TYPE:Perimeter
;WIDTH:0.45
G1 F1292.454
G1 X104.486 Y96.857 E0.20589
G1 X104.394 Y96.534 E0.01007
G1 X104.059 Y95.739 E0.02583
G1 X103.629 Y94.993 E0.02583
G1 X103.110 Y94.304 E0.02583
G1 X102.511 Y93.685 E0.02583
G1 X101.840 Y93.144 E0.02583
G1 X101.107 Y92.690 E0.02583
G1 X100.324 Y92.329 E0.02583
G1 X99.503 Y92.068 E0.02583
G1 X98.656 Y91.909 E0.02583
G1 X97.815 Y91.857 E0.02523
G1 X89.643 Y91.857 E0.24487
G1 X89.643 Y88.143 E0.11130
G1 X97.815 Y88.143 E0.24487
G1 X98.656 Y88.091 E0.02523
G1 X99.503 Y87.932 E0.02583
G1 X100.324 Y87.671 E0.02583
G1 X101.107 Y87.310 E0.02583
G1 X101.840 Y86.856 E0.02583
```

```
G1 X102.511 Y86.315 E0.02583
G1 X103.110 Y85.696 E0.02583
G1 X103.629 Y85.007 E0.02583
G1 X104.059 Y84.261 E0.02583
G1 X104.394 Y83.466 E0.02583
G1 X104.486 Y83.143 E0.01007
G1 X111.357 Y83.143 E0.20589
G1 X111.357 Y96.797 E0.40914
M204 S1250
G1 X111.775 Y97.275 F9000.000
M204 S800
;TYPE:External perimeter
G1 F1292.454
G1 X104.170 Y97.275 E0.22787
G1 X104.000 Y96.672 E0.01877
G1 X103.685 Y95.926 E0.02428
G1 X103.280 Y95.223 E0.02428
G1 X102.792 Y94.576 E0.02428
G1 X102.229 Y93.994 E0.02428
G1 X101.598 Y93.486 E0.02428
G1 X100.909 Y93.059 E0.02428
G1 X100.173 Y92.719 E0.02428
G1 X99.401 Y92.474 E0.02428
G1 X98.604 Y92.325 E0.02428
G1 X97.802 Y92.275 E0.02407
G1 X89.225 Y92.275 E0.25701
G1 X89.225 Y87.725 E0.13634
G1 X97.802 Y87.725 E0.25701
G1 X98.604 Y87.675 E0.02407
```

```
G1 X99.401 Y87.526 E0.02428
G1 X100.173 Y87.281 E0.02428
G1 X100.909 Y86.941 E0.02428
G1 X101.598 Y86.514 E0.02428
G1 X102.229 Y86.006 E0.02428
G1 X102.792 Y85.424 E0.02428
G1 X103.280 Y84.777 E0.02428
G1 X103.685 Y84.074 E0.02428
G1 X104.000 Y83.328 E0.02428
G1 X104.170 Y82.725 E0.01877
G1 X111.775 Y82.725 E0.22787
G1 X111.775 Y97.215 E0.43418
M204 S1250
M73 P75 R17
G1 X111.401 Y97.133 F9000.000
G1 X105.230 Y96.732
M204 S1000
;TYPE:Solid infill
;WIDTH:0.456609
G1 F1272.328
G1 X104.158 Y95.660 E0.04614
M204 S1250
G1 E-4.00000 F2400.000
G1 X111.232 Y84.127 F9000.000
G1 E4.00000 F900.000
M204 S1000
G1 F1272.328
G1 X110.561 Y83.456 E0.02887
G1 X109.961 Y83.456 E0.01827
```

G1 X111.044 Y84.539 E0.04661

G1 X111.044 Y85.139 E0.01827

G1 X109.361 Y83.456 E0.07245

G1 X108.761 Y83.456 E0.01827

G1 X111.044 Y85.739 E0.09828

G1 X111.044 Y86.340 E0.01827

G1 X108.160 Y83.456 E0.12412

G1 X107.560 Y83.456 E0.01827

G1 X111.044 Y86.940 E0.14996

G1 X111.044 Y87.540 E0.01827

G1 X106.960 Y83.456 E0.17580

G1 X106.360 Y83.456 E0.01827

G1 X111.044 Y88.140 E0.20163

G1 X111.044 Y88.741 E0.01827

G1 X105.759 Y83.456 E0.22747

G1 X105.159 Y83.456 E0.01827

G1 X111.044 Y89.341 E0.25331

G1 X111.044 Y89.941 E0.01827

G1 X104.686 Y83.583 E0.27370

G1 X104.507 Y84.005 E0.01395

G1 X111.044 Y90.541 E0.28137

G1 X111.044 Y91.141 E0.01827

G1 X104.327 Y84.424 E0.28914

G1 X104.107 Y84.805 E0.01338

G1 X111.044 Y91.742 E0.29859

G1 X111.044 Y92.342 E0.01827

G1 X103.887 Y85.185 E0.30805

G1 X103.630 Y85.528 E0.01305

G1 X111.044 Y92.942 E0.31916

G1 X111.044 Y93.542 E0.01827

G1 X103.372 Y85.870 E0.33026

G1 X103.080 Y86.178 E0.01292

G1 X111.044 Y94.142 E0.34283

G1 X111.044 Y94.743 E0.01827

G1 X102.784 Y86.483 E0.35554

G1 X102.460 Y86.759 E0.01296

G1 X111.044 Y95.343 E0.36951

G1 X111.044 Y95.943 E0.01827

G1 X102.127 Y87.027 E0.38382

G1 X101.769 Y87.269 E0.01316

G1 X111.044 Y96.544 E0.39926

G1 X110.444 Y96.544 E0.01825

G1 X101.398 Y87.498 E0.38938

G1 X101.256 Y87.587 E0.00511

G1 X101.003 Y87.703 E0.00847

G1 X109.844 Y96.544 E0.38056

G1 X109.244 Y96.544 E0.01827

G1 X100.592 Y87.892 E0.37241

G1 X100.438 Y87.964 E0.00518

G1 X100.154 Y88.054 E0.00907

G1 X108.643 Y96.544 E0.36545

G1 X108.043 Y96.544 E0.01827

G1 X99.698 Y88.199 E0.35921

G1 X99.206 Y88.307 E0.01534

G1 X107.443 Y96.544 E0.35457

G1 X106.843 Y96.544 E0.01827

G1 X98.700 Y88.401 E0.35050

G1 X98.136 Y88.437 E0.01722

G1 X106.243 Y96.544 E0.34897

G1 X105.642 Y96.544 E0.01827

G1 X97.555 Y88.456 E0.34814

G1 X96.954 Y88.456 E0.01827

G1 X100.618 Y92.119 E0.15769

G1 X100.438 Y92.036 E0.00603

G1 X99.699 Y91.801 E0.02358

G1 X96.354 Y88.456 E0.14400

G1 X95.754 Y88.456 E0.01827

G1 X98.942 Y91.644 E0.13722

G1 X98.694 Y91.598 E0.00767

G1 X98.269 Y91.571 E0.01297

G1 X95.154 Y88.456 E0.13409

G1 X94.554 Y88.456 E0.01827

G1 X97.641 Y91.544 E0.13291

G1 X97.041 Y91.544 E0.01827

G1 X93.953 Y88.456 E0.13291

G1 X93.353 Y88.456 E0.01827

G1 X96.441 Y91.544 E0.13291

G1 X95.841 Y91.544 E0.01827

G1 X92.753 Y88.456 E0.13291

G1 X92.153 Y88.456 E0.01827

G1 X95.240 Y91.544 E0.13291

G1 X94.640 Y91.544 E0.01827

G1 X91.552 Y88.456 E0.13291

G1 X90.952 Y88.456 E0.01827

G1 X94.040 Y91.544 E0.13291

G1 X93.440 Y91.544 E0.01827

G1 X90.352 Y88.456 E0.13291

```
G1 X89.956 Y88.456 E0.01205
G1 X89.956 Y88.661 E0.00622
G1 X92.839 Y91.544 E0.12412
G1 X92.239 Y91.544 E0.01827
G1 X89.956 Y89.261 E0.09828
G1 X89.956 Y89.861 E0.01827
G1 X91.639 Y91.544 E0.07244
G1 X91.039 Y91.544 E0.01827
G1 X89.956 Y90.461 E0.04660
G1 X89.956 Y91.061 E0.01827
G1 X90.627 Y91.732 E0.02886
M204 S1250
; stop printing object Petg print.STL id:0 copy 0
; printing object tpu print.STL id:1 copy 0
; stop printing object tpu print.STL id:1 copy 0
; printing object tpu print.STL id:5 copy 0
; stop printing object tpu print.STL id:5 copy 0
; printing object tpu print.STL id:9 copy 0
; stop printing object tpu print.STL id:9 copy 0
; printing object Petg print.STL id:8 copy 0
G1 E-4.00000 F2400.000
G1 X104.530 Y54.716 F9000.000
G1 E4.00000 F900.000
M204 S800
;TYPE:Perimeter
;WIDTH:0.45
G1 F1292.454
G1 X104.438 Y54.392 E0.01007
G1 X104.103 Y53.598 E0.02583
```

```
G1 X103.673 Y52.851 E0.02583
G1 X103.154 Y52.163 E0.02583
G1 X102.555 Y51.544 E0.02583
G1 X101.883 Y51.003 E0.02583
G1 X101.151 Y50.549 E0.02583
G1 X100.368 Y50.188 E0.02583
G1 X99.546 Y49.926 E0.02583
G1 X98.699 Y49.768 E0.02583
G1 X97.859 Y49.716 E0.02523
G1 X89.686 Y49.716 E0.24487
G1 X89.686 Y46.002 E0.11130
G1 X97.859 Y46.002 E0.24487
G1 X98.699 Y45.950 E0.02523
G1 X99.546 Y45.791 E0.02583
G1 X100.368 Y45.530 E0.02583
G1 X101.151 Y45.169 E0.02583
G1 X101.883 Y44.715 E0.02583
G1 X102.555 Y44.174 E0.02583
G1 X103.154 Y43.555 E0.02583
G1 X103.673 Y42.866 E0.02583
G1 X104.103 Y42.119 E0.02583
G1 X104.438 Y41.325 E0.02583
G1 X104.530 Y41.002 E0.01007
G1 X111.401 Y41.002 E0.20589
G1 X111.401 Y54.716 E0.41094
G1 X104.590 Y54.716 E0.20409
M204 S1250
G1 X104.214 Y55.134 F9000.000
M204 S800
```

```
;TYPE:External perimeter
G1 F1292.454
G1 X104.043 Y54.531 E0.01877
G1 X103.728 Y53.784 E0.02428
G1 X103.324 Y53.082 E0.02428
G1 X102.836 Y52.435 E0.02428
G1 X102.272 Y51.853 E0.02428
G1 X101.641 Y51.344 E0.02428
G1 X100.952 Y50.917 E0.02428
G1 X100.216 Y50.578 E0.02428
G1 X99.444 Y50.332 E0.02428
G1 X98.648 Y50.183 E0.02428
G1 X97.846 Y50.134 E0.02407
G1 X89.269 Y50.134 E0.25701
G1 X89.269 Y45.584 E0.13634
G1 X97.846 Y45.584 E0.25701
G1 X98.648 Y45.534 E0.02407
G1 X99.444 Y45.385 E0.02428
G1 X100.216 Y45.139 E0.02428
G1 X100.952 Y44.800 E0.02428
G1 X101.641 Y44.373 E0.02428
G1 X102.272 Y43.865 E0.02428
G1 X102.836 Y43.282 E0.02428
G1 X103.324 Y42.635 E0.02428
G1 X103.728 Y41.933 E0.02428
G1 X104.043 Y41.186 E0.02428
G1 X104.214 Y40.584 E0.01877
G1 X111.819 Y40.584 E0.22787
G1 X111.819 Y55.134 E0.43598
```

```
G1 X104.274 Y55.134 E0.22608
M204 S1250
G1 X104.204 Y54.734 F9000.000
G1 X105.274 Y54.591
M204 S1000
;TYPE:Solid infill
;WIDTH:0.456609
G1 F1272.328
G1 X104.202 Y53.519 E0.04614
M204 S1250
G1 E-4.00000 F2400.000
G1 X111.276 Y41.985 F9000.000
G1 E4.00000 F900.000
M204 S1000
G1 F1272.328
G1 X110.605 Y41.315 E0.02887
G1 X110.005 Y41.315 E0.01827
G1 X111.087 Y42.398 E0.04661
G1 X111.087 Y42.998 E0.01827
G1 X109.404 Y41.315 E0.07245
G1 X108.804 Y41.315 E0.01827
G1 X111.087 Y43.598 E0.09828
G1 X111.087 Y44.198 E0.01827
G1 X108.204 Y41.315 E0.12412
G1 X107.604 Y41.315 E0.01827
G1 X111.087 Y44.799 E0.14996
G1 X111.087 Y45.399 E0.01827
G1 X107.004 Y41.315 E0.17580
G1 X106.403 Y41.315 E0.01827
```

G1 X111.087 Y45.999 E0.20163

G1 X111.087 Y46.599 E0.01827

G1 X105.803 Y41.315 E0.22747

G1 X105.203 Y41.315 E0.01827

G1 X111.087 Y47.199 E0.25331

G1 X111.087 Y47.800 E0.01827

G1 X104.729 Y41.441 E0.27370

G1 X104.551 Y41.863 E0.01395

G1 X111.087 Y48.400 E0.28137

G1 X111.087 Y49.000 E0.01827

G1 X104.370 Y42.283 E0.28914

G1 X104.151 Y42.664 E0.01338

G1 X111.087 Y49.600 E0.29859

G1 X111.087 Y50.201 E0.01827

G1 X103.931 Y43.044 E0.30805

G1 X103.673 Y43.386 E0.01305

G1 X111.087 Y50.801 E0.31916

G1 X111.087 Y51.401 E0.01827

G1 X103.415 Y43.729 E0.33026

G1 X103.123 Y44.037 E0.01292

G1 X111.087 Y52.001 E0.34283

M73 P75 R16

G1 X111.087 Y52.601 E0.01827

G1 X102.828 Y44.342 E0.35554

G1 X102.503 Y44.617 E0.01296

G1 X111.087 Y53.202 E0.36951

G1 X111.087 Y53.802 E0.01827

G1 X102.171 Y44.885 E0.38382

G1 X101.813 Y45.127 E0.01316

G1 X111.087 Y54.403 E0.39926

G1 X110.488 Y54.403 E0.01825

G1 X101.442 Y45.357 E0.38938

G1 X101.299 Y45.445 E0.00511

G1 X101.047 Y45.562 E0.00847

G1 X109.888 Y54.403 E0.38056

G1 X109.287 Y54.403 E0.01827

G1 X100.636 Y45.751 E0.37241

G1 X100.481 Y45.822 E0.00518

G1 X100.197 Y45.913 E0.00907

G1 X108.687 Y54.403 E0.36545

G1 X108.087 Y54.403 E0.01827

G1 X99.742 Y46.058 E0.35921

G1 X99.250 Y46.165 E0.01534

G1 X107.487 Y54.403 E0.35457

G1 X106.886 Y54.403 E0.01827

G1 X98.744 Y46.260 E0.35050

G1 X98.179 Y46.296 E0.01722

G1 X106.286 Y54.403 E0.34897

M73 P76 R16

G1 X105.686 Y54.403 E0.01827

G1 X97.598 Y46.315 E0.34814

G1 X96.998 Y46.315 E0.01827

G1 X100.661 Y49.978 E0.15769

G1 X100.481 Y49.895 E0.00603

G1 X99.743 Y49.660 E0.02358

G1 X96.398 Y46.315 E0.14400

G1 X95.798 Y46.315 E0.01827

G1 X98.985 Y49.503 E0.13722

```
G1 X98.738 Y49.456 E0.00767
G1 X98.313 Y49.430 E0.01297
G1 X95.197 Y46.315 E0.13409
G1 X94.597 Y46.315 E0.01827
G1 X97.685 Y49.403 E0.13291
G1 X97.085 Y49.403 E0.01827
G1 X93.997 Y46.315 E0.13291
G1 X93.397 Y46.315 E0.01827
G1 X96.484 Y49.403 E0.13291
G1 X95.884 Y49.403 E0.01827
G1 X92.797 Y46.315 E0.13291
G1 X92.196 Y46.315 E0.01827
G1 X95.284 Y49.403 E0.13291
G1 X94.684 Y49.403 E0.01827
G1 X91.596 Y46.315 E0.13291
G1 X90.996 Y46.315 E0.01827
G1 X94.084 Y49.403 E0.13291
G1 X93.483 Y49.403 E0.01827
G1 X90.396 Y46.315 E0.13291
G1 X90.000 Y46.315 E0.01205
G1 X90.000 Y46.519 E0.00622
G1 X92.883 Y49.403 E0.12412
G1 X92.283 Y49.403 E0.01827
G1 X90.000 Y47.119 E0.09828
G1 X90.000 Y47.720 E0.01827
G1 X91.683 Y49.403 E0.07244
G1 X91.082 Y49.403 E0.01827
G1 X90.000 Y48.320 E0.04660
G1 X90.000 Y48.920 E0.01827
```

G1 X90.670 Y49.591 E0.02886

M204 S1250

; stop printing object Petg print.STL id:8 copy 0

; printing object Petg print.STL id:4 copy 0

G1 E-4.00000 F2400.000

G1 X89.694 Y65.950 F9000.000

G1 E4.00000 F900.000

M204 S800

;TYPE:Perimeter

;WIDTH:0.45

G1 F1292.454

G1 X97.866 Y65.950 E0.24487

G1 X98.707 Y65.899 E0.02523

G1 X99.554 Y65.740 E0.02583

G1 X100.375 Y65.478 E0.02583

G1 X101.158 Y65.118 E0.02583

G1 X101.891 Y64.664 E0.02583

G1 X102.562 Y64.123 E0.02583

G1 X103.162 Y63.503 E0.02583

G1 X103.680 Y62.815 E0.02583

G1 X104.111 Y62.068 E0.02583

G1 X104.446 Y61.274 E0.02583

G1 X104.537 Y60.950 E0.01007

G1 X111.408 Y60.950 E0.20589

G1 X111.408 Y74.665 E0.41094

G1 X104.537 Y74.665 E0.20589

G1 X104.446 Y74.341 E0.01007

G1 X104.111 Y73.547 E0.02583

G1 X103.680 Y72.800 E0.02583

G1 X103.162 Y72.112 E0.02583

G1 X102.562 Y71.493 E0.02583

G1 X101.891 Y70.952 E0.02583

G1 X101.158 Y70.497 E0.02583

G1 X100.375 Y70.137 E0.02583

G1 X99.554 Y69.875 E0.02583

G1 X98.707 Y69.717 E0.02583

G1 X97.866 Y69.665 E0.02523

G1 X89.694 Y69.665 E0.24487

G1 X89.694 Y66.010 E0.10950

M204 S1250

G1 X89.276 Y65.533 F9000.000

M204 S800

;TYPE:External perimeter

G1 F1292.454

G1 X97.853 Y65.533 E0.25701

G1 X98.655 Y65.483 E0.02407

G1 X99.452 Y65.334 E0.02428

G1 X100.224 Y65.088 E0.02428

G1 X100.960 Y64.749 E0.02428

G1 X101.649 Y64.322 E0.02428

G1 X102.280 Y63.814 E0.02428

G1 X102.843 Y63.231 E0.02428

G1 X103.331 Y62.584 E0.02428

G1 X103.736 Y61.882 E0.02428

G1 X104.051 Y61.135 E0.02428

G1 X104.221 Y60.533 E0.01877

G1 X111.826 Y60.533 E0.22787

G1 X111.826 Y75.083 E0.43598

G1 X104.221 Y75.083 E0.22787

G1 X104.051 Y74.480 E0.01877

G1 X103.736 Y73.733 E0.02428

G1 X103.331 Y73.031 E0.02428

G1 X102.843 Y72.384 E0.02428

G1 X102.280 Y71.802 E0.02428

G1 X101.649 Y71.293 E0.02428

G1 X100.960 Y70.866 E0.02428

G1 X100.224 Y70.527 E0.02428

G1 X99.452 Y70.281 E0.02428

G1 X98.655 Y70.132 E0.02428

G1 X97.853 Y70.083 E0.02407

G1 X89.276 Y70.083 E0.25701

G1 X89.276 Y65.593 E0.13454

M204 S1250

G1 X89.671 Y65.597 F9000.000

G1 X90.678 Y69.539

M204 S1000

;TYPE:Solid infill

;WIDTH:0.456609

G1 F1272.328

G1 X90.007 Y68.869 E0.02886

G1 X90.007 Y68.269 E0.01827

G1 X91.090 Y69.351 E0.04660

G1 X91.690 Y69.351 E0.01827

G1 X90.007 Y67.669 E0.07244

G1 X90.007 Y67.068 E0.01827

G1 X92.290 Y69.351 E0.09828

G1 X92.891 Y69.351 E0.01827

```
G1 X90.007 Y66.468 E0.12412
G1 X90.007 Y66.264 E0.00622
G1 X90.403 Y66.264 E0.01205
G1 X93.491 Y69.351 E0.13291
G1 X94.091 Y69.351 E0.01827
G1 X91.003 Y66.264 E0.13291
G1 X91.604 Y66.264 E0.01827
G1 X94.691 Y69.351 E0.13291
G1 X95.292 Y69.351 E0.01827
G1 X92.204 Y66.264 E0.13291
G1 X92.804 Y66.264 E0.01827
G1 X95.892 Y69.351 E0.13291
G1 X96.492 Y69.351 E0.01827
G1 X93.404 Y66.264 E0.13291
G1 X94.005 Y66.264 E0.01827
G1 X97.092 Y69.351 E0.13291
G1 X97.692 Y69.351 E0.01827
G1 X94.605 Y66.264 E0.13291
G1 X95.205 Y66.264 E0.01827
G1 X98.320 Y69.379 E0.13409
G1 X98.745 Y69.405 E0.01297
G1 X98.993 Y69.451 E0.00767
G1 X95.805 Y66.264 E0.13722
G1 X96.405 Y66.264 E0.01827
G1 X99.751 Y69.609 E0.14400
G1 X100.489 Y69.844 E0.02358
G1 X100.669 Y69.927 E0.00603
G1 X97.006 Y66.264 E0.15769
G1 X97.606 Y66.264 E0.01827
```

```
G1 X105.693 Y74.351 E0.34814
G1 X106.294 Y74.351 E0.01827
G1 X98.187 Y66.245 E0.34897
G1 X98.751 Y66.209 E0.01722
G1 X106.894 Y74.351 E0.35050
G1 X107.494 Y74.351 E0.01827
G1 X99.257 Y66.114 E0.35457
G1 X99.749 Y66.007 E0.01534
G1 X108.094 Y74.351 E0.35921
G1 X108.695 Y74.351 E0.01827
G1 X100.205 Y65.862 E0.36545
G1 X100.489 Y65.771 E0.00907
G1 X100.643 Y65.700 E0.00518
G1 X109.295 Y74.351 E0.37241
G1 X109.895 Y74.351 E0.01827
G1 X101.054 Y65.511 E0.38056
G1 X101.307 Y65.394 E0.00847
G1 X101.450 Y65.306 E0.00511
G1 X110.495 Y74.351 E0.38938
G1 X111.095 Y74.351 E0.01825
G1 X101.820 Y65.076 E0.39926
G1 X102.178 Y64.834 E0.01316
G1 X111.095 Y73.751 E0.38382
G1 X111.095 Y73.151 E0.01827
G1 X102.511 Y64.566 E0.36951
G1 X102.835 Y64.291 E0.01296
G1 X111.095 Y72.550 E0.35554
G1 X111.095 Y71.950 E0.01827
G1 X103.131 Y63.986 E0.34283
```

```
G1 X103.423 Y63.678 E0.01292
G1 X111.095 Y71.350 E0.33026
G1 X111.095 Y70.750 E0.01827
G1 X103.681 Y63.335 E0.31916
G1 X103.939 Y62.993 E0.01305
G1 X111.095 Y70.149 E0.30805
G1 X111.095 Y69.549 E0.01827
G1 X104.159 Y62.613 E0.29859
G1 X104.378 Y62.232 E0.01338
G1 X111.095 Y68.949 E0.28914
G1 X111.095 Y68.349 E0.01827
G1 X104.559 Y61.812 E0.28137
G1 X104.737 Y61.390 E0.01395
G1 X111.095 Y67.749 E0.27370
G1 X111.095 Y67.148 E0.01827
G1 X105.210 Y61.264 E0.25331
G1 X105.811 Y61.264 E0.01827
G1 X111.095 Y66.548 E0.22747
G1 X111.095 Y65.948 E0.01827
G1 X106.411 Y61.264 E0.20163
G1 X107.011 Y61.264 E0.01827
G1 X111.095 Y65.348 E0.17580
G1 X111.095 Y64.747 E0.01827
G1 X107.611 Y61.264 E0.14996
G1 X108.212 Y61.264 E0.01827
G1 X111.095 Y64.147 E0.12412
G1 X111.095 Y63.547 E0.01827
G1 X108.812 Y61.264 E0.09828
G1 X109.412 Y61.264 E0.01827
```

```
G1 X111.095 Y62.947 E0.07245
G1 X111.095 Y62.347 E0.01827
G1 X110.012 Y61.264 E0.04661
G1 X110.612 Y61.264 E0.01827
G1 X111.283 Y61.934 E0.02887
M204 S1250
G1 E-4.00000 F2400.000
G1 X104.209 Y73.468 F9000.000
G1 E4.00000 F900.000
M204 S1000
G1 F1272.328
G1 X105.281 Y74.539 E0.04614
M204 S1250
; stop printing object Petg print.STL id:4 copy 0
; printing object tpu print.STL id:29 copy 0
; stop printing object tpu print.STL id:29 copy 0
; printing object tpu print.STL id:25 copy 0
; stop printing object tpu print.STL id:25 copy 0
; printing object Petg print.STL id:24 copy 0
G1 E-4.00000 F2400.000
G1 X141.344 Y69.840 F9000.000
G1 E4.00000 F900.000
M204 S800
;TYPE:Perimeter
;WIDTH:0.45
G1 F1292.454
G1 X141.344 Y66.126 E0.11130
G1 X149.516 Y66.126 E0.24487
G1 X150.357 Y66.074 E0.02523
```

```
G1 X151.204 Y65.915 E0.02583
G1 X152.026 Y65.654 E0.02583
G1 X152.808 Y65.293 E0.02583
G1 X153.541 Y64.839 E0.02583
G1 X154.212 Y64.298 E0.02583
G1 X154.812 Y63.679 E0.02583
G1 X155.331 Y62.990 E0.02583
G1 X155.761 Y62.243 E0.02583
G1 X156.096 Y61.449 E0.02583
G1 X156.187 Y61.126 E0.01007
G1 X163.059 Y61.126 E0.20589
G1 X163.059 Y74.840 E0.41094
G1 X156.187 Y74.840 E0.20589
G1 X156.096 Y74.516 E0.01007
G1 X155.761 Y73.722 E0.02583
G1 X155.331 Y72.975 E0.02583
G1 X154.812 Y72.287 E0.02583
G1 X154.212 Y71.668 E0.02583
G1 X153.541 Y71.127 E0.02583
G1 X152.808 Y70.673 E0.02583
G1 X152.026 Y70.312 E0.02583
G1 X151.204 Y70.050 E0.02583
G1 X150.357 Y69.892 E0.02583
G1 X149.516 Y69.840 E0.02523
G1 X141.404 Y69.840 E0.24307
M204 S1250
G1 X140.926 Y70.258 F9000.000
M204 S800
;TYPE:External perimeter
```

```
G1 F1292.454
G1 X140.926 Y65.708 E0.13634
G1 X149.503 Y65.708 E0.25701
G1 X150.305 Y65.658 E0.02407
G1 X151.102 Y65.509 E0.02428
G1 X151.874 Y65.263 E0.02428
G1 X152.610 Y64.924 E0.02428
G1 X153.299 Y64.497 E0.02428
G1 X153.930 Y63.989 E0.02428
G1 X154.494 Y63.406 E0.02428
G1 X154.981 Y62.759 E0.02428
G1 X155.386 Y62.057 E0.02428
G1 X155.701 Y61.310 E0.02428
G1 X155.872 Y60.708 E0.01877
G1 X163.476 Y60.708 E0.22787
G1 X163.476 Y75.258 E0.43598
G1 X155.872 Y75.258 E0.22787
G1 X155.701 Y74.655 E0.01877
G1 X155.386 Y73.908 E0.02428
G1 X154.981 Y73.206 E0.02428
G1 X154.494 Y72.559 E0.02428
G1 X153.930 Y71.977 E0.02428
G1 X153.299 Y71.468 E0.02428
G1 X152.610 Y71.041 E0.02428
G1 X151.874 Y70.702 E0.02428
G1 X151.102 Y70.456 E0.02428
G1 X150.305 Y70.307 E0.02428
G1 X149.503 Y70.258 E0.02407
G1 X140.986 Y70.258 E0.25521
```

```
M204 S1250
G1 X141.127 Y69.912 F9000.000
G1 X142.328 Y69.715
M204 S1000
;TYPE:Solid infill
;WIDTH:0.456609
G1 F1272.328
G1 X141.658 Y69.044 E0.02886
G1 X141.658 Y68.444 E0.01827
G1 X142.740 Y69.527 E0.04660
G1 X143.340 Y69.527 E0.01827
G1 X141.658 Y67.844 E0.07244
G1 X141.658 Y67.243 E0.01827
G1 X143.941 Y69.527 E0.09828
G1 X144.541 Y69.527 E0.01827
G1 X141.658 Y66.643 E0.12412
G1 X141.658 Y66.439 E0.00622
G1 X142.053 Y66.439 E0.01205
G1 X145.141 Y69.527 E0.13291
G1 X145.741 Y69.527 E0.01827
G1 X142.654 Y66.439 E0.13291
G1 X143.254 Y66.439 E0.01827
G1 X146.342 Y69.527 E0.13291
G1 X146.942 Y69.527 E0.01827
G1 X143.854 Y66.439 E0.13291
G1 X144.454 Y66.439 E0.01827
G1 X147.542 Y69.527 E0.13291
G1 X148.142 Y69.527 E0.01827
G1 X145.055 Y66.439 E0.13291
```

```
G1 X145.655 Y66.439 E0.01827
G1 X148.742 Y69.527 E0.13291
G1 X149.343 Y69.527 E0.01827
G1 X146.255 Y66.439 E0.13291
G1 X146.855 Y66.439 E0.01827
G1 X149.970 Y69.554 E0.13409
M73 P77 R16
G1 X150.396 Y69.580 E0.01297
G1 X150.643 Y69.627 E0.00767
G1 X147.455 Y66.439 E0.13722
G1 X148.056 Y66.439 E0.01827
G1 X151.401 Y69.784 E0.14400
G1 X152.139 Y70.019 E0.02358
G1 X152.319 Y70.102 E0.00603
G1 X148.656 Y66.439 E0.15769
G1 X149.256 Y66.439 E0.01827
G1 X157.344 Y74.527 E0.34814
G1 X157.944 Y74.527 E0.01827
G1 X149.837 Y66.420 E0.34897
G1 X150.402 Y66.384 E0.01722
G1 X158.544 Y74.527 E0.35050
G1 X159.144 Y74.527 E0.01827
G1 X150.907 Y66.289 E0.35457
G1 X151.400 Y66.182 E0.01534
G1 X159.745 Y74.527 E0.35921
G1 X160.345 Y74.527 E0.01827
G1 X151.855 Y66.037 E0.36545
G1 X152.139 Y65.946 E0.00907
G1 X152.294 Y65.875 E0.00518
```

```
G1 X160.945 Y74.527 E0.37241
G1 X161.545 Y74.527 E0.01827
G1 X152.704 Y65.686 E0.38056
G1 X152.957 Y65.569 E0.00847
G1 X153.100 Y65.481 E0.00511
G1 X162.145 Y74.527 E0.38938
G1 X162.745 Y74.527 E0.01825
G1 X153.470 Y65.251 E0.39926
G1 X153.829 Y65.009 E0.01316
G1 X162.745 Y73.926 E0.38382
G1 X162.745 Y73.326 E0.01827
G1 X154.161 Y64.742 E0.36951
G1 X154.486 Y64.466 E0.01296
G1 X162.745 Y72.725 E0.35554
G1 X162.745 Y72.125 E0.01827
G1 X154.781 Y64.161 E0.34283
G1 X155.073 Y63.853 E0.01292
G1 X162.745 Y71.525 E0.33026
G1 X162.745 Y70.925 E0.01827
G1 X155.331 Y63.510 E0.31916
G1 X155.589 Y63.168 E0.01305
G1 X162.745 Y70.325 E0.30805
G1 X162.745 Y69.724 E0.01827
G1 X155.809 Y62.788 E0.29859
G1 X156.028 Y62.407 E0.01338
G1 X162.745 Y69.124 E0.28914
M73 P77 R15
G1 X162.745 Y68.524 E0.01827
G1 X156.209 Y61.987 E0.28137
```

```
G1 X156.387 Y61.565 E0.01395
G1 X162.745 Y67.924 E0.27370
G1 X162.745 Y67.323 E0.01827
G1 X156.861 Y61.439 E0.25331
G1 X157.461 Y61.439 E0.01827
G1 X162.745 Y66.723 E0.22747
G1 X162.745 Y66.123 E0.01827
G1 X158.061 Y61.439 E0.20163
G1 X158.661 Y61.439 E0.01827
G1 X162.745 Y65.523 E0.17580
G1 X162.745 Y64.923 E0.01827
G1 X159.262 Y61.439 E0.14996
G1 X159.862 Y61.439 E0.01827
G1 X162.745 Y64.322 E0.12412
G1 X162.745 Y63.722 E0.01827
G1 X160.462 Y61.439 E0.09828
G1 X161.062 Y61.439 E0.01827
G1 X162.745 Y63.122 E0.07245
G1 X162.745 Y62.522 E0.01827
G1 X161.662 Y61.439 E0.04661
G1 X162.263 Y61.439 E0.01827
G1 X162.933 Y62.109 E0.02887
M204 S1250
G1 E-4.00000 F2400.000
G1 X155.860 Y73.643 F9000.000
G1 E4.00000 F900.000
M204 S1000
G1 F1272.328
G1 X156.932 Y74.715 E0.04614
```

```
M204 S1250
; stop printing object Petg print.STL id:24 copy 0
; printing object Petg print.STL id:28 copy 0
G1 E-4.00000 F2400.000
G1 X156.180 Y54.891 F9000.000
G1 E4.00000 F900.000
M204 S800
;TYPE:Perimeter
;WIDTH:0.45
G1 F1292.454
G1 X156.088 Y54.567 E0.01007
G1 X155.753 Y53.773 E0.02583
G1 X155.323 Y53.026 E0.02583
G1 X154.804 Y52.338 E0.02583
G1 X154.205 Y51.719 E0.02583
G1 X153.534 Y51.178 E0.02583
G1 X152.801 Y50.724 E0.02583
G1 X152.018 Y50.363 E0.02583
G1 X151.197 Y50.101 E0.02583
G1 X150.349 Y49.943 E0.02583
G1 X149.509 Y49.891 E0.02523
G1 X141.337 Y49.891 E0.24487
G1 X141.337 Y46.177 E0.11130
G1 X149.509 Y46.177 E0.24487
G1 X150.349 Y46.125 E0.02523
G1 X151.197 Y45.966 E0.02583
G1 X152.018 Y45.705 E0.02583
G1 X152.801 Y45.344 E0.02583
G1 X153.534 Y44.890 E0.02583
```

```
G1 X154.205 Y44.349 E0.02583
G1 X154.804 Y43.730 E0.02583
G1 X155.323 Y43.041 E0.02583
G1 X155.753 Y42.294 E0.02583
G1 X156.088 Y41.500 E0.02583
G1 X156.180 Y41.177 E0.01007
G1 X163.051 Y41.177 E0.20589
G1 X163.051 Y54.891 E0.41094
G1 X156.240 Y54.891 E0.20409
M204 S1250
G1 X155.864 Y55.309 F9000.000
M204 S800
;TYPE:External perimeter
G1 F1292.454
G1 X155.693 Y54.706 E0.01877
G1 X155.378 Y53.959 E0.02428
G1 X154.974 Y53.257 E0.02428
G1 X154.486 Y52.610 E0.02428
G1 X153.923 Y52.028 E0.02428
G1 X153.292 Y51.519 E0.02428
G1 X152.603 Y51.092 E0.02428
G1 X151.867 Y50.753 E0.02428
G1 X151.094 Y50.507 E0.02428
G1 X150.298 Y50.358 E0.02428
G1 X149.496 Y50.309 E0.02407
G1 X140.919 Y50.309 E0.25701
G1 X140.919 Y45.759 E0.13634
G1 X149.496 Y45.759 E0.25701
G1 X150.298 Y45.709 E0.02407
```

```
G1 X151.094 Y45.560 E0.02428
G1 X151.867 Y45.314 E0.02428
G1 X152.603 Y44.975 E0.02428
G1 X153.292 Y44.548 E0.02428
G1 X153.923 Y44.040 E0.02428
G1 X154.486 Y43.458 E0.02428
G1 X154.974 Y42.810 E0.02428
G1 X155.378 Y42.108 E0.02428
G1 X155.693 Y41.361 E0.02428
G1 X155.864 Y40.759 E0.01877
G1 X163.469 Y40.759 E0.22787
G1 X163.469 Y55.309 E0.43598
G1 X155.924 Y55.309 E0.22608
M204 S1250
G1 X155.855 Y54.909 F9000.000
G1 X156.924 Y54.766
M204 S1000
;TYPE:Solid infill
;WIDTH:0.456609
G1 F1272.328
G1 X155.852 Y53.694 E0.04614
M204 S1250
G1 E-4.00000 F2400.000
G1 X162.926 Y42.161 F9000.000
G1 E4.00000 F900.000
M204 S1000
G1 F1272.328
G1 X162.255 Y41.490 E0.02887
G1 X161.655 Y41.490 E0.01827
```

```
G1 X162.738 Y42.573 E0.04661
G1 X162.738 Y43.173 E0.01827
G1 X161.055 Y41.490 E0.07245
G1 X160.454 Y41.490 E0.01827
G1 X162.738 Y43.773 E0.09828
G1 X162.738 Y44.373 E0.01827
G1 X159.854 Y41.490 E0.12412
G1 X159.254 Y41.490 E0.01827
G1 X162.738 Y44.974 E0.14996
G1 X162.738 Y45.574 E0.01827
G1 X158.654 Y41.490 E0.17580
G1 X158.054 Y41.490 E0.01827
G1 X162.738 Y46.174 E0.20163
G1 X162.738 Y46.774 E0.01827
G1 X157.453 Y41.490 E0.22747
G1 X156.853 Y41.490 E0.01827
G1 X162.738 Y47.375 E0.25331
G1 X162.738 Y47.975 E0.01827
G1 X156.379 Y41.616 E0.27370
G1 X156.201 Y42.039 E0.01395
G1 X162.738 Y48.575 E0.28137
G1 X162.738 Y49.175 E0.01827
G1 X156.021 Y42.458 E0.28914
G1 X155.801 Y42.839 E0.01338
G1 X162.738 Y49.775 E0.29859
G1 X162.738 Y50.376 E0.01827
G1 X155.581 Y43.219 E0.30805
G1 X155.323 Y43.561 E0.01305
G1 X162.738 Y50.976 E0.31916
```

```
G1 X162.738 Y51.576 E0.01827
G1 X155.065 Y43.904 E0.33026
G1 X154.773 Y44.212 E0.01292
G1 X162.738 Y52.176 E0.34283
G1 X162.738 Y52.776 E0.01827
G1 X154.478 Y44.517 E0.35554
G1 X154.154 Y44.793 E0.01296
G1 X162.738 Y53.377 E0.36951
G1 X162.738 Y53.977 E0.01827
G1 X153.821 Y45.060 E0.38382
G1 X153.463 Y45.302 E0.01316
G1 X162.738 Y54.578 E0.39926
G1 X162.138 Y54.578 E0.01825
G1 X153.092 Y45.532 E0.38938
G1 X152.950 Y45.620 E0.00511
G1 X152.697 Y45.737 E0.00847
G1 X161.538 Y54.578 E0.38056
G1 X160.938 Y54.578 E0.01827
G1 X152.286 Y45.926 E0.37241
G1 X152.132 Y45.997 E0.00518
G1 X151.847 Y46.088 E0.00907
G1 X160.337 Y54.578 E0.36545
G1 X159.737 Y54.578 E0.01827
G1 X151.392 Y46.233 E0.35921
G1 X150.900 Y46.340 E0.01534
G1 X159.137 Y54.578 E0.35457
G1 X158.537 Y54.578 E0.01827
G1 X150.394 Y46.435 E0.35050
G1 X149.830 Y46.471 E0.01722
```

```
G1 X157.936 Y54.578 E0.34897
G1 X157.336 Y54.578 E0.01827
G1 X149.249 Y46.490 E0.34814
G1 X148.648 Y46.490 E0.01827
G1 X152.312 Y50.153 E0.15769
G1 X152.132 Y50.070 E0.00603
G1 X151.393 Y49.835 E0.02358
G1 X148.048 Y46.490 E0.14400
G1 X147.448 Y46.490 E0.01827
G1 X150.636 Y49.678 E0.13722
G1 X150.388 Y49.631 E0.00767
G1 X149.963 Y49.605 E0.01297
G1 X146.848 Y46.490 E0.13409
G1 X146.247 Y46.490 E0.01827
G1 X149.335 Y49.578 E0.13291
G1 X148.735 Y49.578 E0.01827
G1 X145.647 Y46.490 E0.13291
G1 X145.047 Y46.490 E0.01827
G1 X148.135 Y49.578 E0.13291
G1 X147.534 Y49.578 E0.01827
G1 X144.447 Y46.490 E0.13291
G1 X143.847 Y46.490 E0.01827
G1 X146.934 Y49.578 E0.13291
G1 X146.334 Y49.578 E0.01827
G1 X143.246 Y46.490 E0.13291
G1 X142.646 Y46.490 E0.01827
G1 X145.734 Y49.578 E0.13291
M73 P78 R15
G1 X145.134 Y49.578 E0.01827
```

```
G1 X142.046 Y46.490 E0.13291
G1 X141.650 Y46.490 E0.01205
G1 X141.650 Y46.694 E0.00622
G1 X144.533 Y49.578 E0.12412
G1 X143.933 Y49.578 E0.01827
G1 X141.650 Y47.294 E0.09828
G1 X141.650 Y47.895 E0.01827
G1 X143.333 Y49.578 E0.07244
G1 X142.733 Y49.578 E0.01827
G1 X141.650 Y48.495 E0.04660
G1 X141.650 Y49.095 E0.01827
G1 X142.321 Y49.766 E0.02886
M204 S1250
; stop printing object Petg print.STL id:28 copy 0
G1 E-4.00000 F2400.000
; Filament-specific end gcode
M600
M106 S76.5
T1
M900 K0.2 ; Filament gcode
; printing object tpu print.STL id:19 copy 0
G1 Z1.000 F9000.000
G1 X41.611 Y49.135
G1 Z0.800
G1 E4.00000 F1500.000
M204 S800
;TYPE:Perimeter
;WIDTH:0.45
G1 F2400.000
```

```
G1 X34.439 Y49.135 E0.18688
G1 X33.599 Y49.187 E0.02194
G1 X32.752 Y49.345 E0.02246
G1 X31.930 Y49.607 E0.02246
G1 X31.147 Y49.968 E0.02246
G1 X30.415 Y50.422 E0.02246
G1 X29.743 Y50.963 E0.02246
G1 X29.144 Y51.582 E0.02246
G1 X28.625 Y52.270 E0.02246
G1 X28.195 Y53.017 E0.02246
G1 X27.860 Y53.812 E0.02246
G1 X27.768 Y54.135 E0.00876
G1 X20.897 Y54.135 E0.17903
G1 X20.897 Y40.421 E0.35734
G1 X27.768 Y40.421 E0.17903
G1 X27.860 Y40.744 E0.00876
G1 X28.195 Y41.538 E0.02246
G1 X28.625 Y42.285 E0.02246
G1 X29.144 Y42.974 E0.02246
G1 X29.743 Y43.593 E0.02246
G1 X30.415 Y44.134 E0.02246
G1 X31.147 Y44.588 E0.02246
G1 X31.930 Y44.949 E0.02246
G1 X32.752 Y45.210 E0.02246
G1 X33.599 Y45.369 E0.02246
G1 X34.439 Y45.421 E0.02194
G1 X41.611 Y45.421 E0.18688
G1 X41.611 Y49.075 E0.09522
M204 S1250
```

```
G1 X42.029 Y49.553 F9000.000
M204 S800
;TYPE:External perimeter
G1 F1800.000
G1 X34.452 Y49.553 E0.19743
G1 X33.650 Y49.602 E0.02093
G1 X32.854 Y49.751 E0.02112
G1 X32.082 Y49.997 E0.02112
G1 X31.345 Y50.336 E0.02112
G1 X30.657 Y50.763 E0.02112
G1 X30.026 Y51.272 E0.02112
G1 X29.462 Y51.854 E0.02112
G1 X28.974 Y52.501 E0.02112
G1 X28.570 Y53.203 E0.02112
G1 X28.255 Y53.950 E0.02112
G1 X28.084 Y54.553 E0.01632
G1 X20.479 Y54.553 E0.19815
G1 X20.479 Y40.003 E0.37911
G1 X28.084 Y40.003 E0.19815
G1 X28.255 Y40.606 E0.01632
G1 X28.570 Y41.352 E0.02112
G1 X28.974 Y42.054 E0.02112
G1 X29.462 Y42.702 E0.02112
G1 X30.026 Y43.284 E0.02112
G1 X30.657 Y43.792 E0.02112
G1 X31.345 Y44.219 E0.02112
G1 X32.082 Y44.558 E0.02112
G1 X32.854 Y44.804 E0.02112
G1 X33.650 Y44.953 E0.02112
```

```
G1 X34.452 Y45.003 E0.02093
G1 X42.029 Y45.003 E0.19743
G1 X42.029 Y49.493 E0.11699
M204 S1250
G1 X41.636 Y49.481 F9000.000
G1 E-2.24000 F2400.000
;WIPE_START
G1 F7200.000
G1 X39.149 Y49.516 E-0.91200
;WIPE_END
G1 E-0.04800 F2400.000
G1 Z1.000 F9000.000
G1 X41.486 Y46.392
G1 Z0.800
G1 E3.20000 F1500.000
M204 S1000
;TYPE:Solid infill
;WIDTH:0.450839
G1 F4800.000
G1 X40.828 Y45.734 E0.02431
G1 X40.236 Y45.734 E0.01546
G1 X41.298 Y46.796 E0.03923
G1 X41.298 Y47.388 E0.01546
G1 X39.644 Y45.734 E0.06109
G1 X39.052 Y45.734 E0.01546
G1 X41.298 Y47.981 E0.08295
G1 X41.298 Y48.573 E0.01546
G1 X38.460 Y45.734 E0.10481
G1 X37.867 Y45.734 E0.01546
```

```
G1 X40.955 Y48.822 E0.11400
G1 X40.363 Y48.822 E0.01546
G1 X37.275 Y45.734 E0.11400
G1 X36.683 Y45.734 E0.01546
G1 X39.771 Y48.822 E0.11400
G1 X39.179 Y48.822 E0.01546
G1 X36.091 Y45.734 E0.11400
G1 X35.499 Y45.734 E0.01546
G1 X38.587 Y48.822 E0.11400
G1 X37.995 Y48.822 E0.01546
G1 X34.907 Y45.734 E0.11400
G1 X34.308 Y45.726 E0.01566
G1 X37.403 Y48.822 E0.11428
G1 X36.811 Y48.822 E0.01546
G1 X33.677 Y45.688 E0.11572
G1 X32.966 Y45.569 E0.01880
G1 X36.219 Y48.822 E0.12009
G1 X35.627 Y48.822 E0.01546
G1 X32.154 Y45.349 E0.12823
G1 X31.817 Y45.241 E0.00923
G1 X31.145 Y44.932 E0.01931
G1 X35.035 Y48.822 E0.14362
G1 X34.443 Y48.822 E0.01546
G1 X26.355 Y40.734 E0.29862
G1 X26.947 Y40.734 E0.01546
G1 X28.456 Y42.243 E0.05573
M204 S1250
G1 E-2.24000 F2400.000
;WIPE_START
```

```
G1 F7200.000
G1 X26.947 Y40.734 E-0.67595
G1 X26.355 Y40.734 E-0.18749
G1 X26.463 Y40.842 E-0.04857
;WIPE_END
G1 E-0.04800 F2400.000
G1 Z1.000 F9000.000
G1 X34.062 Y49.033
G1 Z0.800
G1 E3.20000 F1500.000
M204 S1000
G1 F4800.000
G1 X25.763 Y40.734 E0.30641
G1 X25.171 Y40.734 E0.01546
G1 X33.351 Y48.914 E0.30204
G1 X32.852 Y49.008 E0.01325
G1 X24.579 Y40.734 E0.30549
G1 X23.987 Y40.734 E0.01546
G1 X32.386 Y49.133 E0.31012
G1 X31.937 Y49.276 E0.01230
G1 X23.395 Y40.734 E0.31540
G1 X22.802 Y40.734 E0.01546
G1 X31.520 Y49.451 E0.32186
G1 X31.114 Y49.638 E0.01165
G1 X22.210 Y40.734 E0.32875
G1 X21.618 Y40.734 E0.01546
G1 X30.737 Y49.853 E0.33670
G1 X30.372 Y50.080 E0.01123
G1 X21.210 Y40.918 E0.33826
```

G1 X21.210 Y41.510 E0.01546

G1 X30.030 Y50.330 E0.32563

G1 X29.702 Y50.594 E0.01099

G1 X21.210 Y42.102 E0.31353

G1 X21.210 Y42.694 E0.01546

G1 X29.392 Y50.876 E0.30207

G1 X29.100 Y51.176 E0.01093

G1 X21.210 Y43.286 E0.29132

G1 X21.210 Y43.878 E0.01546

G1 X28.821 Y51.489 E0.28101

G1 X28.567 Y51.827 E0.01104

G1 X21.210 Y44.471 E0.27162

G1 X21.210 Y45.063 E0.01546

G1 X28.320 Y52.172 E0.26250

G1 X28.104 Y52.548 E0.01132

G1 X21.210 Y45.655 E0.25451

G1 X21.210 Y46.247 E0.01546

G1 X27.892 Y52.928 E0.24671

G1 X27.717 Y53.345 E0.01180

G1 X21.210 Y46.839 E0.24022

G1 X21.210 Y47.431 E0.01546

G1 X27.547 Y53.767 E0.23395

G1 X27.531 Y53.822 E0.00148

G1 X27.009 Y53.822 E0.01363

G1 X21.210 Y48.023 E0.21411

G1 X21.210 Y48.615 E0.01546

G1 X26.417 Y53.822 E0.19225

G1 X25.825 Y53.822 E0.01546

G1 X21.210 Y49.207 E0.17039

```
G1 X21.210 Y49.799 E0.01546
G1 X25.233 Y53.822 E0.14853
G1 X24.641 Y53.822 E0.01546
G1 X21.210 Y50.391 E0.12666
G1 X21.210 Y50.983 E0.01546
G1 X24.049 Y53.822 E0.10480
G1 X23.457 Y53.822 E0.01546
G1 X21.210 Y51.575 E0.08294
G1 X21.210 Y52.167 E0.01546
G1 X22.865 Y53.822 E0.06108
G1 X22.273 Y53.822 E0.01546
G1 X21.210 Y52.759 E0.03922
G1 X21.210 Y53.351 E0.01546
G1 X21.869 Y54.010 E0.02431
M204 S1250
; stop printing object tpu print.STL id:19 copy 0
; printing object Petg print.STL id:18 copy 0
; stop printing object Petg print.STL id:18 copy 0
; printing object Petg print.STL id:14 copy 0
; stop printing object Petg print.STL id:14 copy 0
; printing object tpu print.STL id:15 copy 0
G1 E-2.24000 F2400.000
;WIPE_START
G1 F7200.000
G1 X21.210 Y53.351 E-0.29480
G1 X21.210 Y52.759 E-0.18749
G1 X22.170 Y53.719 E-0.42971
;WIPE_END
G1 E-0.04800 F2400.000
```

G1 Z1.000 F9000.000

G1 X20.905 Y60.370

G1 Z0.800

G1 E3.20000 F1500.000

M204 S800

;TYPE:Perimeter

;WIDTH:0.45

G1 F2400.000

G1 X27.776 Y60.370 E0.17903

G1 X27.867 Y60.693 E0.00876

G1 X28.202 Y61.487 E0.02246

G1 X28.633 Y62.234 E0.02246

G1 X29.151 Y62.923 E0.02246

G1 X29.751 Y63.542 E0.02246

G1 X30.422 Y64.083 E0.02246

G1 X31.155 Y64.537 E0.02246

G1 X31.938 Y64.898 E0.02246

G1 X32.759 Y65.159 E0.02246

G1 X33.606 Y65.318 E0.02246

G1 X34.447 Y65.370 E0.02194

G1 X41.619 Y65.370 E0.18688

G1 X41.619 Y69.084 E0.09678

G1 X34.447 Y69.084 E0.18688

G1 X33.606 Y69.136 E0.02194

G1 X32.759 Y69.294 E0.02246

G1 X31.938 Y69.556 E0.02246

G1 X31.155 Y69.917 E0.02246

G1 X30.422 Y70.371 E0.02246

G1 X29.751 Y70.912 E0.02246

```
G1 X29.151 Y71.531 E0.02246
G1 X28.633 Y72.219 E0.02246
G1 X28.202 Y72.966 E0.02246
G1 X27.867 Y73.760 E0.02246
G1 X27.776 Y74.084 E0.00876
G1 X20.905 Y74.084 E0.17903
G1 X20.905 Y60.430 E0.35578
M204 S1250
G1 X20.487 Y59.952 F9000.000
M204 S800
;TYPE:External perimeter
G1 F1800.000
G1 X28.092 Y59.952 E0.19815
G1 X28.262 Y60.554 E0.01632
G1 X28.577 Y61.301 E0.02112
G1 X28.982 Y62.003 E0.02112
G1 X29.469 Y62.651 E0.02112
G1 X30.033 Y63.233 E0.02112
G1 X30.664 Y63.741 E0.02112
G1 X31.353 Y64.168 E0.02112
G1 X32.089 Y64.507 E0.02112
G1 X32.861 Y64.753 E0.02112
G1 X33.658 Y64.902 E0.02112
G1 X34.460 Y64.952 E0.02093
G1 X42.037 Y64.952 E0.19743
G1 X42.037 Y69.502 E0.11855
G1 X34.460 Y69.502 E0.19743
G1 X33.658 Y69.551 E0.02093
G1 X32.861 Y69.700 E0.02112
```

```
G1 X32.089 Y69.946 E0.02112
G1 X31.353 Y70.285 E0.02112
G1 X30.664 Y70.712 E0.02112
G1 X30.033 Y71.221 E0.02112
G1 X29.469 Y71.803 E0.02112
G1 X28.982 Y72.450 E0.02112
G1 X28.577 Y73.152 E0.02112
G1 X28.262 Y73.899 E0.02112
G1 X28.092 Y74.502 E0.01632
G1 X20.487 Y74.502 E0.19815
G1 X20.487 Y60.012 E0.37755
M204 S1250
G1 X20.861 Y60.094 F9000.000
G1 E-2.24000 F2400.000
;WIPE_START
G1 F7200.000
G1 X23.367 Y59.989 E-0.91200
;WIPE_END
G1 E-0.04800 F2400.000
G1 Z1.000 F9000.000
G1 X28.464 Y62.192
G1 Z0.800
G1 E3.20000 F1500.000
M204 S1000
;TYPE:Solid infill
;WIDTH:0.450839
G1 F4800.000
G1 X26.954 Y60.683 E0.05573
G1 X26.362 Y60.683 E0.01546
```

G1 X34.450 Y68.771 E0.29862

G1 X35.042 Y68.771 E0.01546

G1 X31.152 Y64.881 E0.14362

G1 X31.824 Y65.190 E0.01931

G1 X32.161 Y65.298 E0.00923

G1 X35.634 Y68.771 E0.12823

G1 X36.226 Y68.771 E0.01546

G1 X32.974 Y65.518 E0.12009

G1 X33.684 Y65.636 E0.01880

G1 X36.818 Y68.771 E0.11572

G1 X37.410 Y68.771 E0.01546

G1 X34.315 Y65.675 E0.11428

G1 X34.915 Y65.683 E0.01566

G1 X38.002 Y68.771 E0.11400

G1 X38.594 Y68.771 E0.01546

G1 X35.507 Y65.683 E0.11400

G1 X36.099 Y65.683 E0.01546

G1 X39.186 Y68.771 E0.11400

G1 X39.779 Y68.771 E0.01546

G1 X36.691 Y65.683 E0.11400

G1 X37.283 Y65.683 E0.01546

G1 X40.371 Y68.771 E0.11400

G1 X40.963 Y68.771 E0.01546

G1 X37.875 Y65.683 E0.11400

G1 X38.467 Y65.683 E0.01546

G1 X41.306 Y68.522 E0.10481

G1 X41.306 Y67.929 E0.01546

G1 X39.059 Y65.683 E0.08295

G1 X39.651 Y65.683 E0.01546

```
G1 X41.306 Y67.337 E0.06109
G1 X41.306 Y66.745 E0.01546
G1 X40.243 Y65.683 E0.03923
G1 X40.835 Y65.683 E0.01546
G1 X41.494 Y66.341 E0.02431
M204 S1250
G1 E-2.24000 F2400.000
;WIPE_START
G1 F7200.000
G1 X40.835 Y65.683 E-0.29484
G1 X40.243 Y65.683 E-0.18749
G1 X41.203 Y66.642 E-0.42968
;WIPE_END
G1 E-0.04800 F2400.000
G1 Z1.000 F9000.000
G1 X34.069 Y68.982
G1 Z0.800
G1 E3.20000 F1500.000
M204 S1000
G1 F4800.000
G1 X25.770 Y60.683 E0.30641
G1 X25.178 Y60.683 E0.01546
G1 X33.359 Y68.863 E0.30204
G1 X32.860 Y68.957 E0.01325
G1 X24.586 Y60.683 E0.30549
G1 X23.994 Y60.683 E0.01546
G1 X32.393 Y69.082 E0.31012
G1 X31.944 Y69.225 E0.01230
G1 X23.402 Y60.683 E0.31540
```

```
G1 X22.810 Y60.683 E0.01546
G1 X31.527 Y69.400 E0.32186
G1 X31.122 Y69.587 E0.01165
G1 X22.218 Y60.683 E0.32875
G1 X21.626 Y60.683 E0.01546
G1 X30.745 Y69.802 E0.33670
G1 X30.379 Y70.029 E0.01123
G1 X21.218 Y60.867 E0.33826
G1 X21.218 Y61.459 E0.01546
G1 X30.037 Y70.278 E0.32563
G1 X29.709 Y70.543 E0.01099
G1 X21.218 Y62.051 E0.31353
G1 X21.218 Y62.643 E0.01546
G1 X29.399 Y70.824 E0.30207
G1 X29.108 Y71.125 E0.01093
G1 X21.218 Y63.235 E0.29132
G1 X21.218 Y63.827 E0.01546
G1 X28.829 Y71.438 E0.28101
G1 X28.574 Y71.776 E0.01104
G1 X21.218 Y64.419 E0.27162
G1 X21.218 Y65.012 E0.01546
G1 X28.328 Y72.121 E0.26250
G1 X28.111 Y72.497 E0.01132
G1 X21.218 Y65.604 E0.25451
G1 X21.218 Y66.196 E0.01546
G1 X27.900 Y72.877 E0.24671
G1 X27.724 Y73.294 E0.01180
G1 X21.218 Y66.788 E0.24022
G1 X21.218 Y67.380 E0.01546
```

```
G1 X27.554 Y73.716 E0.23395
G1 X27.539 Y73.771 E0.00148
G1 X27.017 Y73.771 E0.01363
G1 X21.218 Y67.972 E0.21411
G1 X21.218 Y68.564 E0.01546
G1 X26.425 Y73.771 E0.19225
G1 X25.833 Y73.771 E0.01546
G1 X21.218 Y69.156 E0.17039
G1 X21.218 Y69.748 E0.01546
G1 X25.241 Y73.771 E0.14853
G1 X24.649 Y73.771 E0.01546
G1 X21.218 Y70.340 E0.12666
G1 X21.218 Y70.932 E0.01546
G1 X24.056 Y73.771 E0.10480
G1 X23.464 Y73.771 E0.01546
G1 X21.218 Y71.524 E0.08294
G1 X21.218 Y72.116 E0.01546
G1 X22.872 Y73.771 E0.06108
G1 X22.280 Y73.771 E0.01546
G1 X21.218 Y72.708 E0.03922
G1 X21.218 Y73.300 E0.01546
G1 X21.876 Y73.959 E0.02431
M204 S1250
; stop printing object tpu print.STL id:15 copy 0
; printing object Petg print.STL id:10 copy 0
; stop printing object Petg print.STL id:10 copy 0
; printing object tpu print.STL id:11 copy 0
G1 E-2.24000 F2400.000
;WIPE_START
```

```
G1 F7200.000
G1 X21.218 Y73.300 E-0.29480
G1 X21.218 Y72.708 E-0.18749
G1 X22.177 Y73.668 E-0.42971
;WIPE_END
G1 E-0.04800 F2400.000
G1 Z1.000 F9000.000
G1 X20.853 Y82.562
M73 P78 R14
G1 Z0.800
G1 E3.20000 F1500.000
M204 S800
;TYPE:Perimeter
;WIDTH:0.45
G1 F2400.000
G1 X27.725 Y82.562 E0.17903
G1 X27.816 Y82.885 E0.00876
G1 X28.151 Y83.680 E0.02246
G1 X28.581 Y84.427 E0.02246
G1 X29.100 Y85.115 E0.02246
G1 X29.700 Y85.734 E0.02246
G1 X30.371 Y86.275 E0.02246
G1 X31.104 Y86.729 E0.02246
G1 X31.887 Y87.090 E0.02246
G1 X32.708 Y87.351 E0.02246
G1 X33.555 Y87.510 E0.02246
G1 X34.396 Y87.562 E0.02194
G1 X41.568 Y87.562 E0.18688
G1 X41.568 Y91.276 E0.09678
```

```
G1 X34.396 Y91.276 E0.18688
G1 X33.555 Y91.328 E0.02194
G1 X32.708 Y91.487 E0.02246
G1 X31.887 Y91.748 E0.02246
G1 X31.104 Y92.109 E0.02246
G1 X30.371 Y92.563 E0.02246
G1 X29.700 Y93.104 E0.02246
G1 X29.100 Y93.723 E0.02246
G1 X28.581 Y94.412 E0.02246
G1 X28.151 Y95.159 E0.02246
G1 X27.816 Y95.953 E0.02246
G1 X27.725 Y96.276 E0.00876
G1 X20.853 Y96.276 E0.17903
G1 X20.853 Y82.622 E0.35578
M204 S1250
G1 X20.436 Y82.144 F9000.000
M204 S800
;TYPE:External perimeter
G1 F1800.000
G1 X28.040 Y82.144 E0.19815
G1 X28.211 Y82.747 E0.01632
G1 X28.526 Y83.493 E0.02112
G1 X28.931 Y84.196 E0.02112
G1 X29.418 Y84.843 E0.02112
G1 X29.982 Y85.425 E0.02112
G1 X30.613 Y85.934 E0.02112
G1 X31.302 Y86.361 E0.02112
G1 X32.038 Y86.700 E0.02112
G1 X32.810 Y86.946 E0.02112
```

```
G1 X33.607 Y87.095 E0.02112
G1 X34.409 Y87.144 E0.02093
G1 X41.986 Y87.144 E0.19743
G1 X41.986 Y91.694 E0.11855
G1 X34.409 Y91.694 E0.19743
G1 X33.607 Y91.744 E0.02093
G1 X32.810 Y91.893 E0.02112
G1 X32.038 Y92.139 E0.02112
G1 X31.302 Y92.478 E0.02112
G1 X30.613 Y92.905 E0.02112
G1 X29.982 Y93.413 E0.02112
G1 X29.418 Y93.995 E0.02112
G1 X28.931 Y94.643 E0.02112
G1 X28.526 Y95.345 E0.02112
G1 X28.211 Y96.091 E0.02112
G1 X28.040 Y96.694 E0.01632
G1 X20.436 Y96.694 E0.19815
G1 X20.436 Y82.204 E0.37755
M204 S1250
G1 X20.810 Y82.286 F9000.000
G1 E-2.24000 F2400.000
;WIPE_START
G1 F7200.000
G1 X23.316 Y82.181 E-0.91200
;WIPE_END
G1 E-0.04800 F2400.000
G1 Z1.000 F9000.000
G1 X28.413 Y84.385
G1 Z0.800
```

```
G1 E3.20000 F1500.000
M204 S1000
;TYPE:Solid infill
;WIDTH:0.450839
G1 F4800.000
G1 X26.903 Y82.875 E0.05573
G1 X26.311 Y82.875 E0.01546
G1 X34.399 Y90.963 E0.29862
G1 X34.991 Y90.963 E0.01546
G1 X31.101 Y87.073 E0.14362
G1 X31.773 Y87.383 E0.01931
G1 X32.110 Y87.490 E0.00923
G1 X35.583 Y90.963 E0.12823
G1 X36.175 Y90.963 E0.01546
G1 X32.923 Y87.710 E0.12009
G1 X33.633 Y87.829 E0.01880
G1 X36.767 Y90.963 E0.11572
G1 X37.359 Y90.963 E0.01546
G1 X34.264 Y87.868 E0.11428
G1 X34.864 Y87.875 E0.01566
G1 X37.951 Y90.963 E0.11400
G1 X38.543 Y90.963 E0.01546
G1 X35.456 Y87.875 E0.11400
G1 X36.048 Y87.875 E0.01546
G1 X39.135 Y90.963 E0.11400
G1 X39.727 Y90.963 E0.01546
G1 X36.640 Y87.875 E0.11400
G1 X37.232 Y87.875 E0.01546
G1 X40.319 Y90.963 E0.11400
```

```
G1 X40.911 Y90.963 E0.01546
G1 X37.824 Y87.875 E0.11400
G1 X38.416 Y87.875 E0.01546
G1 X41.254 Y90.714 E0.10481
G1 X41.254 Y90.122 E0.01546
G1 X39.008 Y87.875 E0.08295
G1 X39.600 Y87.875 E0.01546
G1 X41.254 Y89.530 E0.06109
G1 X41.254 Y88.938 E0.01546
G1 X40.192 Y87.875 E0.03923
G1 X40.784 Y87.875 E0.01546
G1 X41.442 Y88.534 E0.02431
M204 S1250
G1 E-2.24000 F2400.000
;WIPE_START
G1 F7200.000
G1 X40.784 Y87.875 E-0.29484
G1 X40.192 Y87.875 E-0.18749
G1 X41.152 Y88.835 E-0.42968
;WIPE_END
G1 E-0.04800 F2400.000
G1 Z1.000 F9000.000
G1 X34.018 Y91.174
G1 Z0.800
G1 E3.20000 F1500.000
M204 S1000
G1 F4800.000
G1 X25.719 Y82.875 E0.30641
G1 X25.127 Y82.875 E0.01546
```

```
G1 X33.308 Y91.056 E0.30204
G1 X32.809 Y91.149 E0.01325
G1 X24.535 Y82.875 E0.30549
G1 X23.943 Y82.875 E0.01546
G1 X32.342 Y91.274 E0.31012
G1 X31.893 Y91.417 E0.01230
G1 X23.351 Y82.875 E0.31540
G1 X22.759 Y82.875 E0.01546
G1 X31.476 Y91.592 E0.32186
G1 X31.071 Y91.779 E0.01165
G1 X22.167 Y82.875 E0.32875
G1 X21.575 Y82.875 E0.01546
G1 X30.694 Y91.994 E0.33670
G1 X30.328 Y92.221 E0.01123
G1 X21.167 Y83.059 E0.33826
G1 X21.167 Y83.652 E0.01546
G1 X29.986 Y92.471 E0.32563
M73 P79 R14
G1 X29.658 Y92.735 E0.01099
G1 X21.167 Y84.244 E0.31353
G1 X21.167 Y84.836 E0.01546
G1 X29.348 Y93.017 E0.30207
G1 X29.057 Y93.318 E0.01093
G1 X21.167 Y85.428 E0.29132
G1 X21.167 Y86.020 E0.01546
G1 X28.778 Y93.631 E0.28101
G1 X28.523 Y93.968 E0.01104
G1 X21.167 Y86.612 E0.27162
G1 X21.167 Y87.204 E0.01546
```

G1 X28.276 Y94.313 E0.26250

G1 X28.060 Y94.689 E0.01132

G1 X21.167 Y87.796 E0.25451

G1 X21.167 Y88.388 E0.01546

G1 X27.849 Y95.070 E0.24671

G1 X27.673 Y95.486 E0.01180

G1 X21.167 Y88.980 E0.24022

G1 X21.167 Y89.572 E0.01546

G1 X27.503 Y95.908 E0.23395

G1 X27.488 Y95.963 E0.00148

G1 X26.966 Y95.963 E0.01363

G1 X21.167 Y90.164 E0.21411

G1 X21.167 Y90.756 E0.01546

G1 X26.374 Y95.963 E0.19225

G1 X25.781 Y95.963 E0.01546

G1 X21.167 Y91.348 E0.17039

G1 X21.167 Y91.940 E0.01546

G1 X25.189 Y95.963 E0.14853

G1 X24.597 Y95.963 E0.01546

G1 X21.167 Y92.532 E0.12666

G1 X21.167 Y93.124 E0.01546

G1 X24.005 Y95.963 E0.10480

G1 X23.413 Y95.963 E0.01546

G1 X21.167 Y93.717 E0.08294

G1 X21.167 Y94.309 E0.01546

G1 X22.821 Y95.963 E0.06108

G1 X22.229 Y95.963 E0.01546

G1 X21.167 Y94.901 E0.03922

G1 X21.167 Y95.493 E0.01546

```
G1 X21.825 Y96.151 E0.02431
M204 S1250
; stop printing object tpu print.STL id:11 copy 0
; printing object tpu print.STL id:13 copy 0
G1 E-2.24000 F2400.000
;WIPE_START
G1 F7200.000
G1 X21.167 Y95.493 E-0.29480
G1 X21.167 Y94.901 E-0.18749
G1 X22.126 Y95.860 E-0.42971
;WIPE_END
G1 E-0.04800 F2400.000
G1 Z1.000 F9000.000
G1 X20.860 Y103.377
G1 Z0.800
G1 E3.20000 F1500.000
M204 S800
;TYPE:Perimeter
;WIDTH:0.45
G1 F2400.000
G1 X27.731 Y103.377 E0.17903
G1 X27.823 Y103.701 E0.00876
G1 X28.158 Y104.495 E0.02246
G1 X28.588 Y105.242 E0.02246
G1 X29.107 Y105.930 E0.02246
G1 X29.707 Y106.550 E0.02246
G1 X30.378 Y107.091 E0.02246
G1 X31.110 Y107.545 E0.02246
G1 X31.893 Y107.905 E0.02246
```

```
G1 X32.715 Y108.167 E0.02246
G1 X33.562 Y108.326 E0.02246
G1 X34.402 Y108.377 E0.02194
G1 X41.575 Y108.377 E0.18688
G1 X41.575 Y112.092 E0.09678
G1 X34.402 Y112.092 E0.18688
G1 X33.562 Y112.144 E0.02194
G1 X32.715 Y112.302 E0.02246
G1 X31.893 Y112.564 E0.02246
G1 X31.110 Y112.924 E0.02246
G1 X30.378 Y113.379 E0.02246
G1 X29.707 Y113.920 E0.02246
G1 X29.107 Y114.539 E0.02246
G1 X28.588 Y115.227 E0.02246
G1 X28.158 Y115.974 E0.02246
G1 X27.823 Y116.768 E0.02246
G1 X27.731 Y117.092 E0.00876
G1 X20.860 Y117.092 E0.17903
G1 X20.860 Y103.437 E0.35578
M204 S1250
G1 X20.442 Y102.960 F9000.000
M204 S800
;TYPE:External perimeter
G1 F1800.000
G1 X28.047 Y102.960 E0.19815
G1 X28.218 Y103.562 E0.01632
G1 X28.533 Y104.309 E0.02112
G1 X28.937 Y105.011 E0.02112
G1 X29.425 Y105.658 E0.02112
```

```
G1 X29.989 Y106.241 E0.02112
G1 X30.620 Y106.749 E0.02112
G1 X31.309 Y107.176 E0.02112
G1 X32.045 Y107.515 E0.02112
G1 X32.817 Y107.761 E0.02112
G1 X33.613 Y107.910 E0.02112
G1 X34.415 Y107.960 E0.02093
G1 X41.992 Y107.960 E0.19743
G1 X41.992 Y112.510 E0.11855
G1 X34.415 Y112.510 E0.19743
G1 X33.613 Y112.559 E0.02093
G1 X32.817 Y112.708 E0.02112
G1 X32.045 Y112.954 E0.02112
G1 X31.309 Y113.293 E0.02112
G1 X30.620 Y113.720 E0.02112
G1 X29.989 Y114.229 E0.02112
G1 X29.425 Y114.811 E0.02112
G1 X28.937 Y115.458 E0.02112
G1 X28.533 Y116.160 E0.02112
G1 X28.218 Y116.907 E0.02112
G1 X28.047 Y117.510 E0.01632
G1 X20.442 Y117.510 E0.19815
G1 X20.442 Y103.020 E0.37755
M204 S1250
G1 X20.816 Y103.102 F9000.000
G1 E-2.24000 F2400.000
;WIPE_START
G1 F7200.000
G1 X23.322 Y102.997 E-0.91200
```

```
;WIPE_END
G1 E-0.04800 F2400.000
G1 Z1.000 F9000.000
G1 X28.419 Y105.200
G1 Z0.800
G1 E3.20000 F1500.000
M204 S1000
;TYPE:Solid infill
;WIDTH:0.450839
G1 F4800.000
G1 X26.910 Y103.691 E0.05573
G1 X26.318 Y103.691 E0.01546
G1 X34.406 Y111.778 E0.29862
G1 X34.998 Y111.778 E0.01546
G1 X31.108 Y107.889 E0.14362
G1 X31.780 Y108.198 E0.01931
G1 X32.117 Y108.305 E0.00923
G1 X35.590 Y111.778 E0.12823
G1 X36.182 Y111.778 E0.01546
G1 X32.929 Y108.526 E0.12009
G1 X33.640 Y108.644 E0.01880
G1 X36.774 Y111.778 E0.11572
G1 X37.366 Y111.778 E0.01546
G1 X34.271 Y108.683 E0.11428
G1 X34.870 Y108.691 E0.01566
G1 X37.958 Y111.778 E0.11400
G1 X38.550 Y111.778 E0.01546
G1 X35.462 Y108.691 E0.11400
G1 X36.054 Y108.691 E0.01546
```

```
G1 X39.142 Y111.778 E0.11400
G1 X39.734 Y111.778 E0.01546
G1 X36.647 Y108.691 E0.11400
G1 X37.239 Y108.691 E0.01546
G1 X40.326 Y111.778 E0.11400
G1 X40.918 Y111.778 E0.01546
G1 X37.831 Y108.691 E0.11400
G1 X38.423 Y108.691 E0.01546
G1 X41.261 Y111.529 E0.10481
G1 X41.261 Y110.937 E0.01546
G1 X39.015 Y108.691 E0.08295
G1 X39.607 Y108.691 E0.01546
G1 X41.261 Y110.345 E0.06109
G1 X41.261 Y109.753 E0.01546
G1 X40.199 Y108.691 E0.03923
G1 X40.791 Y108.691 E0.01546
G1 X41.449 Y109.349 E0.02431
M204 S1250
G1 E-2.24000 F2400.000
;WIPE_START
G1 F7200.000
G1 X40.791 Y108.691 E-0.29484
G1 X40.199 Y108.691 E-0.18749
G1 X41.158 Y109.650 E-0.42968
;WIPE_END
G1 E-0.04800 F2400.000
G1 Z1.000 F9000.000
G1 X34.025 Y111.990
G1 Z0.800
```

```
G1 E3.20000 F1500.000
M204 S1000
G1 F4800.000
G1 X25.726 Y103.691 E0.30641
G1 X25.134 Y103.691 E0.01546
G1 X33.314 Y111.871 E0.30204
G1 X32.816 Y111.965 E0.01325
G1 X24.542 Y103.691 E0.30549
G1 X23.950 Y103.691 E0.01546
G1 X32.349 Y112.090 E0.31012
G1 X31.900 Y112.233 E0.01230
G1 X23.358 Y103.691 E0.31540
G1 X22.766 Y103.691 E0.01546
G1 X31.483 Y112.408 E0.32186
G1 X31.077 Y112.595 E0.01165
G1 X22.174 Y103.691 E0.32875
G1 X21.582 Y103.691 E0.01546
G1 X30.701 Y112.810 E0.33670
G1 X30.335 Y113.036 E0.01123
G1 X21.174 Y103.875 E0.33826
G1 X21.174 Y104.467 E0.01546
G1 X29.993 Y113.286 E0.32563
G1 X29.665 Y113.551 E0.01099
G1 X21.174 Y105.059 E0.31353
G1 X21.174 Y105.651 E0.01546
G1 X29.355 Y113.832 E0.30207
G1 X29.064 Y114.133 E0.01093
G1 X21.174 Y106.243 E0.29132
G1 X21.174 Y106.835 E0.01546
```

```
G1 X28.785 Y114.446 E0.28101
G1 X28.530 Y114.784 E0.01104
G1 X21.174 Y107.427 E0.27162
G1 X21.174 Y108.019 E0.01546
G1 X28.283 Y115.129 E0.26250
G1 X28.067 Y115.505 E0.01132
G1 X21.174 Y108.611 E0.25451
G1 X21.174 Y109.204 E0.01546
G1 X27.855 Y115.885 E0.24671
G1 X27.680 Y116.302 E0.01180
G1 X21.174 Y109.796 E0.24022
G1 X21.174 Y110.388 E0.01546
G1 X27.510 Y116.724 E0.23395
G1 X27.494 Y116.778 E0.00148
G1 X26.972 Y116.778 E0.01363
G1 X21.174 Y110.980 E0.21411
G1 X21.174 Y111.572 E0.01546
G1 X26.380 Y116.778 E0.19225
G1 X25.788 Y116.778 E0.01546
G1 X21.174 Y112.164 E0.17039
G1 X21.174 Y112.756 E0.01546
G1 X25.196 Y116.778 E0.14853
G1 X24.604 Y116.778 E0.01546
G1 X21.174 Y113.348 E0.12666
G1 X21.174 Y113.940 E0.01546
G1 X24.012 Y116.778 E0.10480
G1 X23.420 Y116.778 E0.01546
G1 X21.174 Y114.532 E0.08294
G1 X21.174 Y115.124 E0.01546
```

```
G1 X22.828 Y116.778 E0.06108
G1 X22.236 Y116.778 E0.01546
G1 X21.174 Y115.716 E0.03922
G1 X21.174 Y116.308 E0.01546
G1 X21.832 Y116.967 E0.02431
M204 S1250
; stop printing object tpu print.STL id:13 copy 0
; printing object tpu print.STL id:17 copy 0
G1 E-2.24000 F2400.000
;WIPE_START
G1 F7200.000
G1 X21.174 Y116.308 E-0.29480
G1 X21.174 Y115.716 E-0.18749
G1 X22.133 Y116.676 E-0.42971
;WIPE_END
G1 E-0.04800 F2400.000
G1 Z1.000 F9000.000
G1 X20.845 Y122.031
G1 Z0.800
G1 E3.20000 F1500.000
M204 S800
;TYPE:Perimeter
;WIDTH:0.45
G1 F2400.000
G1 X27.716 Y122.031 E0.17903
G1 X27.808 Y122.355 E0.00876
G1 X28.143 Y123.149 E0.02246
G1 X28.573 Y123.896 E0.02246
G1 X29.092 Y124.584 E0.02246
```

```
G1 X29.692 Y125.204 E0.02246
G1 X30.363 Y125.745 E0.02246
G1 X31.095 Y126.199 E0.02246
G1 X31.878 Y126.559 E0.02246
G1 X32.700 Y126.821 E0.02246
G1 X33.547 Y126.980 E0.02246
G1 X34.387 Y127.031 E0.02194
G1 X41.560 Y127.031 E0.18688
G1 X41.560 Y130.746 E0.09678
G1 X34.387 Y130.746 E0.18688
G1 X33.547 Y130.798 E0.02194
G1 X32.700 Y130.956 E0.02246
G1 X31.878 Y131.218 E0.02246
G1 X31.095 Y131.578 E0.02246
G1 X30.363 Y132.033 E0.02246
G1 X29.692 Y132.573 E0.02246
G1 X29.092 Y133.193 E0.02246
G1 X28.573 Y133.881 E0.02246
G1 X28.143 Y134.628 E0.02246
G1 X27.808 Y135.422 E0.02246
G1 X27.716 Y135.746 E0.00876
G1 X20.845 Y135.746 E0.17903
G1 X20.845 Y122.091 E0.35578
M204 S1250
G1 X20.427 Y121.614 F9000.000
M204 S800
;TYPE:External perimeter
G1 F1800.000
G1 X28.032 Y121.614 E0.19815
```

```
G1 X28.203 Y122.216 E0.01632
G1 X28.518 Y122.963 E0.02112
G1 X28.922 Y123.665 E0.02112
G1 X29.410 Y124.312 E0.02112
G1 X29.974 Y124.895 E0.02112
G1 X30.605 Y125.403 E0.02112
G1 X31.294 Y125.830 E0.02112
G1 X32.030 Y126.169 E0.02112
G1 X32.802 Y126.415 E0.02112
G1 X33.598 Y126.564 E0.02112
G1 X34.400 Y126.614 E0.02093
G1 X41.977 Y126.614 E0.19743
G1 X41.977 Y131.164 E0.11855
G1 X34.400 Y131.164 E0.19743
G1 X33.598 Y131.213 E0.02093
G1 X32.802 Y131.362 E0.02112
G1 X32.030 Y131.608 E0.02112
G1 X31.294 Y131.947 E0.02112
G1 X30.605 Y132.374 E0.02112
G1 X29.974 Y132.883 E0.02112
G1 X29.410 Y133.465 E0.02112
G1 X28.922 Y134.112 E0.02112
G1 X28.518 Y134.814 E0.02112
G1 X28.203 Y135.561 E0.02112
G1 X28.032 Y136.164 E0.01632
G1 X20.427 Y136.164 E0.19815
G1 X20.427 Y121.674 E0.37755
M204 S1250
G1 X20.801 Y121.756 F9000.000
```

```
G1 E-2.24000 F2400.000
;WIPE_START
G1 F7200.000
G1 X23.307 Y121.651 E-0.91200
;WIPE_END
G1 E-0.04800 F2400.000
G1 Z1.000 F9000.000
G1 X28.404 Y123.854
G1 Z0.800
G1 E3.20000 F1500.000
M204 S1000
;TYPE:Solid infill
;WIDTH:0.450839
G1 F4800.000
G1 X26.895 Y122.345 E0.05573
G1 X26.303 Y122.345 E0.01546
G1 X34.391 Y130.432 E0.29862
G1 X34.983 Y130.432 E0.01546
G1 X31.093 Y126.543 E0.14362
G1 X31.765 Y126.852 E0.01931
G1 X32.102 Y126.959 E0.00923
G1 X35.575 Y130.432 E0.12823
G1 X36.167 Y130.432 E0.01546
G1 X32.914 Y127.180 E0.12009
G1 X33.625 Y127.298 E0.01880
G1 X36.759 Y130.432 E0.11572
G1 X37.351 Y130.432 E0.01546
G1 X34.256 Y127.337 E0.11428
G1 X34.855 Y127.345 E0.01566
```

```
G1 X37.943 Y130.432 E0.11400
G1 X38.535 Y130.432 E0.01546
G1 X35.447 Y127.345 E0.11400
G1 X36.039 Y127.345 E0.01546
G1 X39.127 Y130.432 E0.11400
G1 X39.719 Y130.432 E0.01546
G1 X36.631 Y127.345 E0.11400
G1 X37.223 Y127.345 E0.01546
G1 X40.311 Y130.432 E0.11400
G1 X40.903 Y130.432 E0.01546
G1 X37.816 Y127.345 E0.11400
G1 X38.408 Y127.345 E0.01546
G1 X41.246 Y130.183 E0.10481
G1 X41.246 Y129.591 E0.01546
G1 X39.000 Y127.345 E0.08295
G1 X39.592 Y127.345 E0.01546
G1 X41.246 Y128.999 E0.06109
G1 X41.246 Y128.407 E0.01546
G1 X40.184 Y127.345 E0.03923
G1 X40.776 Y127.345 E0.01546
G1 X41.434 Y128.003 E0.02431
M204 S1250
G1 E-2.24000 F2400.000
;WIPE_START
G1 F7200.000
G1 X40.776 Y127.345 E-0.29484
G1 X40.184 Y127.345 E-0.18749
G1 X41.143 Y128.304 E-0.42968
;WIPE_END
```

```
G1 E-0.04800 F2400.000
G1 Z1.000 F9000.000
G1 X34.010 Y130.644
G1 Z0.800
G1 E3.20000 F1500.000
M204 S1000
G1 F4800.000
G1 X25.711 Y122.345 E0.30641
G1 X25.119 Y122.345 E0.01546
G1 X33.299 Y130.525 E0.30204
G1 X32.801 Y130.619 E0.01325
G1 X24.527 Y122.345 E0.30549
G1 X23.935 Y122.345 E0.01546
G1 X32.334 Y130.744 E0.31012
G1 X31.885 Y130.887 E0.01230
G1 X23.343 Y122.345 E0.31540
G1 X22.751 Y122.345 E0.01546
G1 X31.468 Y131.062 E0.32186
G1 X31.062 Y131.249 E0.01165
G1 X22.158 Y122.345 E0.32875
G1 X21.566 Y122.345 E0.01546
G1 X30.685 Y131.464 E0.33670
G1 X30.320 Y131.690 E0.01123
G1 X21.159 Y122.529 E0.33826
G1 X21.159 Y123.121 E0.01546
G1 X29.978 Y131.940 E0.32563
G1 X29.650 Y132.204 E0.01099
G1 X21.159 Y123.713 E0.31353
G1 X21.159 Y124.305 E0.01546
```

```
G1 X29.340 Y132.486 E0.30207
G1 X29.049 Y132.787 E0.01093
G1 X21.159 Y124.897 E0.29132
G1 X21.159 Y125.489 E0.01546
G1 X28.769 Y133.100 E0.28101
G1 X28.515 Y133.438 E0.01104
G1 X21.159 Y126.081 E0.27162
G1 X21.159 Y126.673 E0.01546
G1 X28.268 Y133.783 E0.26250
G1 X28.052 Y134.159 E0.01132
G1 X21.159 Y127.265 E0.25451
G1 X21.159 Y127.857 E0.01546
G1 X27.840 Y134.539 E0.24671
G1 X27.665 Y134.956 E0.01180
G1 X21.159 Y128.450 E0.24022
G1 X21.159 Y129.042 E0.01546
G1 X27.495 Y135.378 E0.23395
G1 X27.479 Y135.432 E0.00148
G1 X26.957 Y135.432 E0.01363
G1 X21.159 Y129.634 E0.21411
G1 X21.159 Y130.226 E0.01546
G1 X26.365 Y135.432 E0.19225
G1 X25.773 Y135.432 E0.01546
G1 X21.159 Y130.818 E0.17039
G1 X21.159 Y131.410 E0.01546
G1 X25.181 Y135.432 E0.14853
G1 X24.589 Y135.432 E0.01546
G1 X21.159 Y132.002 E0.12666
G1 X21.159 Y132.594 E0.01546
```

```
G1 X23.997 Y135.432 E0.10480
G1 X23.405 Y135.432 E0.01546
G1 X21.159 Y133.186 E0.08294
G1 X21.159 Y133.778 E0.01546
G1 X22.813 Y135.432 E0.06108
G1 X22.221 Y135.432 E0.01546
G1 X21.159 Y134.370 E0.03922
G1 X21.159 Y134.962 E0.01546
G1 X21.817 Y135.620 E0.02431
M204 S1250
; stop printing object tpu print.STL id:17 copy 0
; printing object Petg print.STL id:16 copy 0
; stop printing object Petg print.STL id:16 copy 0
; printing object Petg print.STL id:12 copy 0
; stop printing object Petg print.STL id:12 copy 0
; printing object tpu print.STL id:7 copy 0
G1 E-2.24000 F2400.000
;WIPE_START
G1 F7200.000
G1 X21.159 Y134.962 E-0.29480
G1 X21.159 Y134.370 E-0.18749
G1 X22.118 Y135.330 E-0.42971
;WIPE_END
G1 E-0.04800 F2400.000
G1 Z1.000 F9000.000
G1 X69.635 Y136.327
G1 Z0.800
G1 E3.20000 F1500.000
M204 S800
```

```
;TYPE:Perimeter
;WIDTH:0.45
G1 F2400.000
G1 X69.635 Y122.612 E0.35734
G1 X76.506 Y122.612 E0.17903
G1 X76.597 Y122.936 E0.00876
G1 X76.932 Y123.730 E0.02246
G1 X77.362 Y124.477 E0.02246
G1 X77.881 Y125.165 E0.02246
G1 X78.481 Y125.785 E0.02246
G1 X79.152 Y126.325 E0.02246
G1 X79.885 Y126.780 E0.02246
G1 X80.668 Y127.140 E0.02246
G1 X81.489 Y127.402 E0.02246
G1 X82.336 Y127.560 E0.02246
G1 X83.177 Y127.612 E0.02194
G1 X90.349 Y127.612 E0.18688
G1 X90.349 Y131.327 E0.09678
G1 X83.177 Y131.327 E0.18688
G1 X82.336 Y131.379 E0.02194
G1 X81.489 Y131.537 E0.02246
G1 X80.668 Y131.799 E0.02246
G1 X79.885 Y132.159 E0.02246
G1 X79.152 Y132.613 E0.02246
G1 X78.481 Y133.154 E0.02246
G1 X77.881 Y133.774 E0.02246
G1 X77.362 Y134.462 E0.02246
G1 X76.932 Y135.209 E0.02246
G1 X76.597 Y136.003 E0.02246
```

```
G1 X76.506 Y136.327 E0.00876
G1 X69.695 Y136.327 E0.17747
M204 S1250
G1 X69.217 Y136.744 F9000.000
M204 S800
;TYPE:External perimeter
G1 F1800.000
G1 X69.217 Y122.194 E0.37911
G1 X76.822 Y122.194 E0.19815
G1 X76.992 Y122.797 E0.01632
G1 X77.307 Y123.544 E0.02112
G1 X77.712 Y124.246 E0.02112
G1 X78.199 Y124.893 E0.02112
G1 X78.763 Y125.475 E0.02112
G1 X79.394 Y125.984 E0.02112
G1 X80.083 Y126.411 E0.02112
G1 X80.819 Y126.750 E0.02112
G1 X81.591 Y126.996 E0.02112
G1 X82.388 Y127.145 E0.02112
G1 X83.190 Y127.194 E0.02093
G1 X90.767 Y127.194 E0.19743
G1 X90.767 Y131.744 E0.11855
G1 X83.190 Y131.744 E0.19743
G1 X82.388 Y131.794 E0.02093
G1 X81.591 Y131.943 E0.02112
G1 X80.819 Y132.189 E0.02112
G1 X80.083 Y132.528 E0.02112
G1 X79.394 Y132.955 E0.02112
G1 X78.763 Y133.463 E0.02112
```

```
G1 X78.199 Y134.046 E0.02112
G1 X77.712 Y134.693 E0.02112
G1 X77.307 Y135.395 E0.02112
G1 X76.992 Y136.142 E0.02112
G1 X76.822 Y136.744 E0.01632
G1 X69.277 Y136.744 E0.19659
M204 S1250
G1 X69.408 Y136.393 F9000.000
G1 E-2.24000 F2400.000
;WIPE_START
G1 F7200.000
G1 X69.265 Y133.864 E-0.91200
;WIPE_END
G1 E-0.04800 F2400.000
G1 Z1.000 F9000.000
G1 X70.606 Y136.201
G1 Z0.800
G1 E3.20000 F1500.000
M204 S1000
;TYPE:Solid infill
;WIDTH:0.450839
G1 F4800.000
G1 X69.948 Y135.543 E0.02431
G1 X69.948 Y134.951 E0.01546
G1 X71.010 Y136.013 E0.03922
G1 X71.602 Y136.013 E0.01546
G1 X69.948 Y134.359 E0.06108
G1 X69.948 Y133.767 E0.01546
G1 X72.194 Y136.013 E0.08294
```

```
G1 X72.786 Y136.013 E0.01546
G1 X69.948 Y133.175 E0.10480
G1 X69.948 Y132.583 E0.01546
G1 X73.378 Y136.013 E0.12666
G1 X73.971 Y136.013 E0.01546
G1 X69.948 Y131.991 E0.14853
G1 X69.948 Y131.399 E0.01546
G1 X74.563 Y136.013 E0.17039
G1 X75.155 Y136.013 E0.01546
G1 X69.948 Y130.807 E0.19225
G1 X69.948 Y130.215 E0.01546
G1 X75.747 Y136.013 E0.21411
G1 X76.269 Y136.013 E0.01363
G1 X76.284 Y135.959 E0.00148
G1 X69.948 Y129.622 E0.23395
G1 X69.948 Y129.030 E0.01546
G1 X76.454 Y135.536 E0.24022
G1 X76.630 Y135.120 E0.01180
G1 X69.948 Y128.438 E0.24671
G1 X69.948 Y127.846 E0.01546
G1 X76.841 Y134.739 E0.25451
G1 X77.057 Y134.364 E0.01132
G1 X69.948 Y127.254 E0.26250
G1 X69.948 Y126.662 E0.01546
G1 X77.304 Y134.019 E0.27162
G1 X77.559 Y133.681 E0.01104
G1 X69.948 Y126.070 E0.28101
G1 X69.948 Y125.478 E0.01546
G1 X77.838 Y133.368 E0.29132
```

```
M73 P80 R14
G1 X78.129 Y133.067 E0.01093
G1 X69.948 Y124.886 E0.30207
G1 X69.948 Y124.294 E0.01546
G1 X78.439 Y132.785 E0.31353
G1 X78.767 Y132.521 E0.01099
G1 X69.948 Y123.702 E0.32563
G1 X69.948 Y123.110 E0.01546
G1 X79.109 Y132.271 E0.33826
G1 X79.475 Y132.045 E0.01123
G1 X70.356 Y122.926 E0.33670
G1 X70.948 Y122.926 E0.01546
G1 X79.852 Y131.829 E0.32875
G1 X80.257 Y131.643 E0.01165
G1 X71.540 Y122.926 E0.32186
G1 X72.132 Y122.926 E0.01546
G1 X80.674 Y131.468 E0.31540
G1 X81.123 Y131.325 E0.01230
G1 X72.724 Y122.926 E0.31012
G1 X73.316 Y122.926 E0.01546
G1 X81.590 Y131.199 E0.30549
G1 X82.089 Y131.106 E0.01325
G1 X73.908 Y122.926 E0.30204
G1 X74.500 Y122.926 E0.01546
G1 X82.799 Y131.224 E0.30641
M204 S1250
G1 E-2.24000 F2400.000
;WIPE_START
G1 F7200.000
```

```
G1 X80.763 Y129.188 E-0.91200
;WIPE_END
G1 E-0.04800 F2400.000
G1 Z1.000 F9000.000
G1 X90.224 Y128.584
G1 Z0.800
G1 E3.20000 F1500.000
M204 S1000
G1 F4800.000
G1 X89.565 Y127.926 E0.02431
G1 X88.973 Y127.926 E0.01546
G1 X90.036 Y128.988 E0.03923
G1 X90.036 Y129.580 E0.01546
G1 X88.381 Y127.926 E0.06109
G1 X87.789 Y127.926 E0.01546
G1 X90.036 Y130.172 E0.08295
G1 X90.036 Y130.764 E0.01546
G1 X87.197 Y127.926 E0.10481
G1 X86.605 Y127.926 E0.01546
G1 X89.693 Y131.013 E0.11400
G1 X89.101 Y131.013 E0.01546
G1 X86.013 Y127.926 E0.11400
G1 X85.421 Y127.926 E0.01546
G1 X88.508 Y131.013 E0.11400
G1 X87.916 Y131.013 E0.01546
G1 X84.829 Y127.926 E0.11400
G1 X84.237 Y127.926 E0.01546
G1 X87.324 Y131.013 E0.11400
G1 X86.732 Y131.013 E0.01546
```

G1 X83.645 Y127.926 E0.11400

G1 X83.045 Y127.918 E0.01566

G1 X86.140 Y131.013 E0.11428

G1 X85.548 Y131.013 E0.01546

G1 X82.414 Y127.879 E0.11572

G1 X81.704 Y127.761 E0.01880

G1 X84.956 Y131.013 E0.12009

G1 X84.364 Y131.013 E0.01546

G1 X80.891 Y127.540 E0.12823

G1 X80.554 Y127.433 E0.00923

G1 X79.882 Y127.124 E0.01931

G1 X83.772 Y131.013 E0.14362

G1 X83.180 Y131.013 E0.01546

G1 X75.092 Y122.926 E0.29862

G1 X75.684 Y122.926 E0.01546

G1 X77.194 Y124.435 E0.05573

M204 S1250

; stop printing object tpu print.STL id:7 copy 0

; printing object tpu print.STL id:3 copy 0

G1 E-2.24000 F2400.000

;WIPE_START

G1 F7200.000

G1 X75.684 Y122.926 E-0.67595

G1 X75.092 Y122.926 E-0.18749

G1 X75.201 Y123.034 E-0.04857

;WIPE_END

G1 E-0.04800 F2400.000

G1 Z1.000 F9000.000

G1 X76.521 Y117.673

```
G1 Z0.800
G1 E3.20000 F1500.000
M204 S800
;TYPE:Perimeter
;WIDTH:0.45
G1 F2400.000
G1 X69.650 Y117.673 E0.17903
G1 X69.650 Y103.958 E0.35734
G1 X76.521 Y103.958 E0.17903
G1 X76.612 Y104.282 E0.00876
G1 X76.947 Y105.076 E0.02246
G1 X77.378 Y105.823 E0.02246
G1 X77.896 Y106.511 E0.02246
G1 X78.496 Y107.131 E0.02246
G1 X79.167 Y107.672 E0.02246
G1 X79.900 Y108.126 E0.02246
G1 X80.683 Y108.486 E0.02246
G1 X81.504 Y108.748 E0.02246
G1 X82.351 Y108.906 E0.02246
G1 X83.192 Y108.958 E0.02194
G1 X90.364 Y108.958 E0.18688
G1 X90.364 Y112.673 E0.09678
G1 X83.192 Y112.673 E0.18688
G1 X82.351 Y112.725 E0.02194
G1 X81.504 Y112.883 E0.02246
G1 X80.683 Y113.145 E0.02246
G1 X79.900 Y113.505 E0.02246
G1 X79.167 Y113.960 E0.02246
G1 X78.496 Y114.500 E0.02246
```

```
G1 X77.896 Y115.120 E0.02246
G1 X77.378 Y115.808 E0.02246
G1 X76.947 Y116.555 E0.02246
G1 X76.612 Y117.349 E0.02246
G1 X76.537 Y117.615 E0.00720
M204 S1250
G1 X76.837 Y118.091 F9000.000
M204 S800
;TYPE:External perimeter
G1 F1800.000
G1 X69.232 Y118.091 E0.19815
G1 X69.232 Y103.541 E0.37911
M73 P80 R13
G1 X76.837 Y103.541 E0.19815
G1 X77.007 Y104.143 E0.01632
G1 X77.322 Y104.890 E0.02112
G1 X77.727 Y105.592 E0.02112
G1 X78.215 Y106.239 E0.02112
G1 X78.778 Y106.822 E0.02112
G1 X79.409 Y107.330 E0.02112
G1 X80.098 Y107.757 E0.02112
G1 X80.834 Y108.096 E0.02112
G1 X81.606 Y108.342 E0.02112
G1 X82.403 Y108.491 E0.02112
G1 X83.205 Y108.541 E0.02093
G1 X90.782 Y108.541 E0.19743
G1 X90.782 Y113.091 E0.11855
G1 X83.205 Y113.091 E0.19743
G1 X82.403 Y113.140 E0.02093
```

```
G1 X81.606 Y113.289 E0.02112
G1 X80.834 Y113.535 E0.02112
G1 X80.098 Y113.874 E0.02112
G1 X79.409 Y114.301 E0.02112
G1 X78.778 Y114.810 E0.02112
G1 X78.215 Y115.392 E0.02112
G1 X77.727 Y116.039 E0.02112
G1 X77.322 Y116.741 E0.02112
G1 X77.007 Y117.488 E0.02112
G1 X76.853 Y118.033 E0.01476
M204 S1250
G1 X76.516 Y117.852 F9000.000
G1 E-2.24000 F2400.000
;WIPE_START
G1 F7200.000
G1 X73.973 Y118.055 E-0.91200
;WIPE_END
G1 E-0.04800 F2400.000
G1 Z1.000 F9000.000
G1 X70.621 Y117.547
G1 Z0.800
G1 E3.20000 F1500.000
M204 S1000
;TYPE:Solid infill
;WIDTH:0.450839
G1 F4800.000
G1 X69.963 Y116.889 E0.02431
G1 X69.963 Y116.297 E0.01546
G1 X71.025 Y117.359 E0.03922
```

```
G1 X71.617 Y117.359 E0.01546
G1 X69.963 Y115.705 E0.06108
G1 X69.963 Y115.113 E0.01546
G1 X72.209 Y117.359 E0.08294
G1 X72.802 Y117.359 E0.01546
G1 X69.963 Y114.521 E0.10480
G1 X69.963 Y113.929 E0.01546
G1 X73.394 Y117.359 E0.12666
G1 X73.986 Y117.359 E0.01546
G1 X69.963 Y113.337 E0.14853
G1 X69.963 Y112.745 E0.01546
G1 X74.578 Y117.359 E0.17039
G1 X75.170 Y117.359 E0.01546
G1 X69.963 Y112.153 E0.19225
G1 X69.963 Y111.561 E0.01546
G1 X75.762 Y117.359 E0.21411
G1 X76.284 Y117.359 E0.01363
G1 X76.299 Y117.305 E0.00148
G1 X69.963 Y110.968 E0.23395
G1 X69.963 Y110.376 E0.01546
G1 X76.469 Y116.883 E0.24022
G1 X76.645 Y116.466 E0.01180
G1 X69.963 Y109.784 E0.24671
G1 X69.963 Y109.192 E0.01546
G1 X76.856 Y116.086 E0.25451
G1 X77.073 Y115.710 E0.01132
G1 X69.963 Y108.600 E0.26250
G1 X69.963 Y108.008 E0.01546
G1 X77.319 Y115.365 E0.27162
```

G1 X77.574 Y115.027 E0.01104

G1 X69.963 Y107.416 E0.28101

G1 X69.963 Y106.824 E0.01546

G1 X77.853 Y114.714 E0.29132

G1 X78.144 Y114.413 E0.01093

G1 X69.963 Y106.232 E0.30207

G1 X69.963 Y105.640 E0.01546

G1 X78.454 Y114.131 E0.31353

G1 X78.782 Y113.867 E0.01099

G1 X69.963 Y105.048 E0.32563

G1 X69.963 Y104.456 E0.01546

G1 X79.124 Y113.617 E0.33826

G1 X79.490 Y113.391 E0.01123

G1 X70.371 Y104.272 E0.33670

G1 X70.963 Y104.272 E0.01546

G1 X79.867 Y113.176 E0.32875

G1 X80.272 Y112.989 E0.01165

G1 X71.555 Y104.272 E0.32186

G1 X72.147 Y104.272 E0.01546

G1 X80.689 Y112.814 E0.31540

G1 X81.138 Y112.671 E0.01230

G1 X72.739 Y104.272 E0.31012

G1 X73.331 Y104.272 E0.01546

G1 X81.605 Y112.545 E0.30549

G1 X82.104 Y112.452 E0.01325

G1 X73.923 Y104.272 E0.30204

G1 X74.515 Y104.272 E0.01546

G1 X82.814 Y112.570 E0.30641

M204 S1250

```
G1 E-2.24000 F2400.000
;WIPE_START
G1 F7200.000
G1 X80.778 Y110.534 E-0.91200
;WIPE_END
G1 E-0.04800 F2400.000
G1 Z1.000 F9000.000
G1 X90.239 Y109.930
G1 Z0.800
G1 E3.20000 F1500.000
M204 S1000
G1 F4800.000
G1 X89.580 Y109.272 E0.02431
G1 X88.988 Y109.272 E0.01546
G1 X90.051 Y110.334 E0.03923
G1 X90.051 Y110.926 E0.01546
G1 X88.396 Y109.272 E0.06109
G1 X87.804 Y109.272 E0.01546
G1 X90.051 Y111.518 E0.08295
G1 X90.051 Y112.110 E0.01546
G1 X87.212 Y109.272 E0.10481
G1 X86.620 Y109.272 E0.01546
G1 X89.708 Y112.359 E0.11400
G1 X89.116 Y112.359 E0.01546
G1 X86.028 Y109.272 E0.11400
G1 X85.436 Y109.272 E0.01546
G1 X88.524 Y112.359 E0.11400
G1 X87.932 Y112.359 E0.01546
G1 X84.844 Y109.272 E0.11400
```

```
G1 X84.252 Y109.272 E0.01546
G1 X87.339 Y112.359 E0.11400
G1 X86.747 Y112.359 E0.01546
G1 X83.660 Y109.272 E0.11400
G1 X83.060 Y109.264 E0.01566
G1 X86.155 Y112.359 E0.11428
G1 X85.563 Y112.359 E0.01546
G1 X82.429 Y109.225 E0.11572
G1 X81.719 Y109.107 E0.01880
G1 X84.971 Y112.359 E0.12009
G1 X84.379 Y112.359 E0.01546
G1 X80.906 Y108.886 E0.12823
G1 X80.569 Y108.779 E0.00923
G1 X79.897 Y108.470 E0.01931
G1 X83.787 Y112.359 E0.14362
G1 X83.195 Y112.359 E0.01546
G1 X75.107 Y104.272 E0.29862
G1 X75.699 Y104.272 E0.01546
G1 X77.209 Y105.781 E0.05573
M204 S1250
; stop printing object tpu print.STL id:3 copy 0
; printing object Petg print.STL id:2 copy 0
; stop printing object Petg print.STL id:2 copy 0
; printing object Petg print.STL id:6 copy 0
; stop printing object Petg print.STL id:6 copy 0
; printing object tpu print.STL id:27 copy 0
G1 E-2.24000 F2400.000
;WIPE_START
G1 F7200.000
```

```
G1 X75.699 Y104.272 E-0.67595
G1 X75.107 Y104.272 E-0.18749
G1 X75.216 Y104.380 E-0.04857
;WIPE_END
G1 E-0.04800 F2400.000
G1 Z1.000 F9000.000
G1 X120.285 Y122.787
G1 Z0.800
G1 E3.20000 F1500.000
M204 S800
;TYPE:Perimeter
;WIDTH:0.45
G1 F2400.000
G1 X127.156 Y122.787 E0.17903
G1 X127.247 Y123.111 E0.00876
G1 X127.583 Y123.905 E0.02246
G1 X128.013 Y124.652 E0.02246
G1 X128.532 Y125.340 E0.02246
G1 X129.131 Y125.960 E0.02246
G1 X129.802 Y126.501 E0.02246
G1 X130.535 Y126.955 E0.02246
G1 X131.318 Y127.315 E0.02246
G1 X132.139 Y127.577 E0.02246
G1 X132.986 Y127.735 E0.02246
G1 X133.827 Y127.787 E0.02194
G1 X140.999 Y127.787 E0.18688
G1 X140.999 Y131.502 E0.09678
G1 X133.827 Y131.502 E0.18688
G1 X132.986 Y131.554 E0.02194
```

```
G1 X132.139 Y131.712 E0.02246
G1 X131.318 Y131.974 E0.02246
G1 X130.535 Y132.334 E0.02246
G1 X129.802 Y132.789 E0.02246
G1 X129.131 Y133.329 E0.02246
G1 X128.532 Y133.949 E0.02246
G1 X128.013 Y134.637 E0.02246
G1 X127.583 Y135.384 E0.02246
G1 X127.247 Y136.178 E0.02246
G1 X127.156 Y136.502 E0.00876
G1 X120.285 Y136.502 E0.17903
G1 X120.285 Y122.847 E0.35578
M204 S1250
G1 X119.867 Y122.370 F9000.000
M204 S800
;TYPE:External perimeter
G1 F1800.000
G1 X127.472 Y122.370 E0.19815
G1 X127.642 Y122.972 E0.01632
G1 X127.957 Y123.719 E0.02112
G1 X128.362 Y124.421 E0.02112
G1 X128.850 Y125.068 E0.02112
G1 X129.413 Y125.651 E0.02112
G1 X130.044 Y126.159 E0.02112
G1 X130.733 Y126.586 E0.02112
G1 X131.469 Y126.925 E0.02112
G1 X132.241 Y127.171 E0.02112
G1 X133.038 Y127.320 E0.02112
G1 X133.840 Y127.370 E0.02093
```

```
G1 X141.417 Y127.370 E0.19743
G1 X141.417 Y131.920 E0.11855
G1 X133.840 Y131.920 E0.19743
G1 X133.038 Y131.969 E0.02093
G1 X132.241 Y132.118 E0.02112
G1 X131.469 Y132.364 E0.02112
G1 X130.733 Y132.703 E0.02112
G1 X130.044 Y133.130 E0.02112
G1 X129.413 Y133.639 E0.02112
G1 X128.850 Y134.221 E0.02112
G1 X128.362 Y134.868 E0.02112
G1 X127.957 Y135.570 E0.02112
G1 X127.642 Y136.317 E0.02112
G1 X127.472 Y136.920 E0.01632
G1 X119.867 Y136.920 E0.19815
G1 X119.867 Y122.430 E0.37755
M204 S1250
G1 X120.241 Y122.512 F9000.000
G1 E-2.24000 F2400.000
;WIPE_START
G1 F7200.000
G1 X122.747 Y122.407 E-0.91200
;WIPE_END
G1 E-0.04800 F2400.000
G1 Z1.000 F9000.000
G1 X127.844 Y124.610
G1 Z0.800
G1 E3.20000 F1500.000
M204 S1000
```

```
;TYPE:Solid infill
;WIDTH:0.450839
G1 F4800.000
G1 X126.335 Y123.101 E0.05573
G1 X125.743 Y123.101 E0.01546
G1 X133.830 Y131.188 E0.29862
G1 X134.422 Y131.188 E0.01546
G1 X130.532 Y127.299 E0.14362
G1 X131.204 Y127.608 E0.01931
G1 X131.541 Y127.715 E0.00923
G1 X135.014 Y131.188 E0.12823
G1 X135.606 Y131.188 E0.01546
G1 X132.354 Y127.936 E0.12009
G1 X133.064 Y128.054 E0.01880
G1 X136.198 Y131.188 E0.11572
G1 X136.790 Y131.188 E0.01546
G1 X133.695 Y128.093 E0.11428
G1 X134.295 Y128.101 E0.01566
G1 X137.383 Y131.188 E0.11400
G1 X137.975 Y131.188 E0.01546
G1 X134.887 Y128.101 E0.11400
G1 X135.479 Y128.101 E0.01546
G1 X138.567 Y131.188 E0.11400
G1 X139.159 Y131.188 E0.01546
G1 X136.071 Y128.101 E0.11400
G1 X136.663 Y128.101 E0.01546
G1 X139.751 Y131.188 E0.11400
G1 X140.343 Y131.188 E0.01546
G1 X137.255 Y128.101 E0.11400
```

```
G1 X137.847 Y128.101 E0.01546
G1 X140.686 Y130.939 E0.10481
G1 X140.686 Y130.347 E0.01546
G1 X138.439 Y128.101 E0.08295
G1 X139.031 Y128.101 E0.01546
G1 X140.686 Y129.755 E0.06109
G1 X140.686 Y129.163 E0.01546
G1 X139.623 Y128.101 E0.03923
G1 X140.215 Y128.101 E0.01546
G1 X140.874 Y128.759 E0.02431
M204 S1250
G1 E-2.24000 F2400.000
;WIPE_START
G1 F7200.000
G1 X140.215 Y128.101 E-0.29484
G1 X139.623 Y128.101 E-0.18749
G1 X140.583 Y129.060 E-0.42968
;WIPE_END
G1 E-0.04800 F2400.000
G1 Z1.000 F9000.000
G1 X133.449 Y131.399
G1 Z0.800
G1 E3.20000 F1500.000
M204 S1000
G1 F4800.000
G1 X125.150 Y123.101 E0.30641
G1 X124.558 Y123.101 E0.01546
G1 X132.739 Y131.281 E0.30204
G1 X132.240 Y131.374 E0.01325
```

G1 X123.966 Y123.101 E0.30549

G1 X123.374 Y123.101 E0.01546

G1 X131.773 Y131.500 E0.31012

G1 X131.324 Y131.643 E0.01230

G1 X122.782 Y123.101 E0.31540

G1 X122.190 Y123.101 E0.01546

G1 X130.907 Y131.818 E0.32186

G1 X130.502 Y132.005 E0.01165

G1 X121.598 Y123.101 E0.32875

G1 X121.006 Y123.101 E0.01546

G1 X130.125 Y132.220 E0.33670

G1 X129.760 Y132.446 E0.01123

G1 X120.598 Y123.285 E0.33826

G1 X120.598 Y123.877 E0.01546

G1 X129.417 Y132.696 E0.32563

G1 X129.090 Y132.960 E0.01099

G1 X120.598 Y124.469 E0.31353

G1 X120.598 Y125.061 E0.01546

G1 X128.779 Y133.242 E0.30207

G1 X128.488 Y133.543 E0.01093

G1 X120.598 Y125.653 E0.29132

G1 X120.598 Y126.245 E0.01546

G1 X128.209 Y133.856 E0.28101

G1 X127.955 Y134.194 E0.01104

G1 X120.598 Y126.837 E0.27162

G1 X120.598 Y127.429 E0.01546

G1 X127.708 Y134.539 E0.26250

G1 X127.491 Y134.915 E0.01132

G1 X120.598 Y128.021 E0.25451

```
G1 X120.598 Y128.613 E0.01546
G1 X127.280 Y135.295 E0.24671
G1 X127.104 Y135.712 E0.01180
G1 X120.598 Y129.205 E0.24022
G1 X120.598 Y129.798 E0.01546
G1 X126.934 Y136.134 E0.23395
G1 X126.919 Y136.188 E0.00148
G1 X126.397 Y136.188 E0.01363
G1 X120.598 Y130.390 E0.21411
G1 X120.598 Y130.982 E0.01546
G1 X125.805 Y136.188 E0.19225
G1 X125.213 Y136.188 E0.01546
G1 X120.598 Y131.574 E0.17039
G1 X120.598 Y132.166 E0.01546
G1 X124.621 Y136.188 E0.14853
G1 X124.029 Y136.188 E0.01546
G1 X120.598 Y132.758 E0.12666
G1 X120.598 Y133.350 E0.01546
G1 X123.437 Y136.188 E0.10480
G1 X122.845 Y136.188 E0.01546
G1 X120.598 Y133.942 E0.08294
G1 X120.598 Y134.534 E0.01546
G1 X122.253 Y136.188 E0.06108
G1 X121.660 Y136.188 E0.01546
G1 X120.598 Y135.126 E0.03922
G1 X120.598 Y135.718 E0.01546
G1 X121.256 Y136.376 E0.02431
M204 S1250
; stop printing object tpu print.STL id:27 copy 0
```

```
; printing object tpu print.STL id:23 copy 0
G1 E-2.24000 F2400.000
;WIPE_START
G1 F7200.000
G1 X120.598 Y135.718 E-0.29480
G1 X120.598 Y135.126 E-0.18749
G1 X121.558 Y136.086 E-0.42971
;WIPE_END
G1 E-0.04800 F2400.000
G1 Z1.000 F9000.000
G1 X120.300 Y117.848
G1 Z0.800
G1 E3.20000 F1500.000
M204 S800
;TYPE:Perimeter
;WIDTH:0.45
G1 F2400.000
G1 X120.300 Y104.133 E0.35734
G1 X127.171 Y104.133 E0.17903
G1 X127.263 Y104.457 E0.00876
G1 X127.598 Y105.251 E0.02246
G1 X128.028 Y105.998 E0.02246
G1 X128.547 Y106.686 E0.02246
G1 X129.146 Y107.306 E0.02246
G1 X129.817 Y107.847 E0.02246
G1 X130.550 Y108.301 E0.02246
G1 X131.333 Y108.661 E0.02246
G1 X132.154 Y108.923 E0.02246
G1 X133.002 Y109.082 E0.02246
```

```
G1 X133.842 Y109.133 E0.02194
G1 X141.014 Y109.133 E0.18688
G1 X141.014 Y112.848 E0.09678
G1 X133.842 Y112.848 E0.18688
G1 X133.002 Y112.900 E0.02194
G1 X132.154 Y113.058 E0.02246
G1 X131.333 Y113.320 E0.02246
G1 X130.550 Y113.680 E0.02246
G1 X129.817 Y114.135 E0.02246
G1 X129.146 Y114.675 E0.02246
G1 X128.547 Y115.295 E0.02246
G1 X128.028 Y115.983 E0.02246
G1 X127.598 Y116.730 E0.02246
G1 X127.263 Y117.524 E0.02246
G1 X127.171 Y117.848 E0.00876
G1 X120.360 Y117.848 E0.17747
M204 S1250
G1 X119.882 Y118.266 F9000.000
M204 S800
;TYPE:External perimeter
G1 F1800.000
G1 X119.882 Y103.716 E0.37911
G1 X127.487 Y103.716 E0.19815
G1 X127.658 Y104.318 E0.01632
G1 X127.973 Y105.065 E0.02112
G1 X128.377 Y105.767 E0.02112
G1 X128.865 Y106.414 E0.02112
G1 X129.428 Y106.997 E0.02112
G1 X130.059 Y107.505 E0.02112
```

```
G1 X130.748 Y107.932 E0.02112
G1 X131.484 Y108.271 E0.02112
G1 X132.257 Y108.517 E0.02112
G1 X133.053 Y108.666 E0.02112
G1 X133.855 Y108.716 E0.02093
G1 X141.432 Y108.716 E0.19743
G1 X141.432 Y113.266 E0.11855
G1 X133.855 Y113.266 E0.19743
G1 X133.053 Y113.315 E0.02093
G1 X132.257 Y113.464 E0.02112
G1 X131.484 Y113.710 E0.02112
G1 X130.748 Y114.049 E0.02112
G1 X130.059 Y114.476 E0.02112
G1 X129.428 Y114.985 E0.02112
G1 X128.865 Y115.567 E0.02112
G1 X128.377 Y116.214 E0.02112
G1 X127.973 Y116.916 E0.02112
G1 X127.658 Y117.663 E0.02112
G1 X127.487 Y118.266 E0.01632
G1 X119.942 Y118.266 E0.19659
M204 S1250
G1 X120.073 Y117.914 F9000.000
G1 E-2.24000 F2400.000
;WIPE_START
G1 F7200.000
G1 X119.930 Y115.386 E-0.91200
;WIPE_END
G1 E-0.04800 F2400.000
G1 Z1.000 F9000.000
```

```
G1 X121.272 Y117.722
G1 Z0.800
G1 E3.20000 F1500.000
M204 S1000
;TYPE:Solid infill
;WIDTH:0.450839
G1 F4800.000
G1 X120.613 Y117.064 E0.02431
G1 X120.613 Y116.472 E0.01546
G1 X121.676 Y117.534 E0.03922
G1 X122.268 Y117.534 E0.01546
G1 X120.613 Y115.880 E0.06108
G1 X120.613 Y115.288 E0.01546
G1 X122.860 Y117.534 E0.08294
G1 X123.452 Y117.534 E0.01546
G1 X120.613 Y114.696 E0.10480
G1 X120.613 Y114.104 E0.01546
G1 X124.044 Y117.534 E0.12666
G1 X124.636 Y117.534 E0.01546
G1 X120.613 Y113.512 E0.14853
M73 P81 R13
G1 X120.613 Y112.920 E0.01546
G1 X125.228 Y117.534 E0.17039
G1 X125.820 Y117.534 E0.01546
G1 X120.613 Y112.328 E0.19225
G1 X120.613 Y111.736 E0.01546
G1 X126.412 Y117.534 E0.21411
G1 X126.934 Y117.534 E0.01363
G1 X126.950 Y117.480 E0.00148
```

G1 X120.613 Y111.144 E0.23395

G1 X120.613 Y110.552 E0.01546

G1 X127.119 Y117.058 E0.24022

G1 X127.295 Y116.641 E0.01180

G1 X120.613 Y109.959 E0.24671

G1 X120.613 Y109.367 E0.01546

G1 X127.506 Y116.261 E0.25451

G1 X127.723 Y115.885 E0.01132

G1 X120.613 Y108.775 E0.26250

G1 X120.613 Y108.183 E0.01546

G1 X127.970 Y115.540 E0.27162

G1 X128.224 Y115.202 E0.01104

G1 X120.613 Y107.591 E0.28101

G1 X120.613 Y106.999 E0.01546

G1 X128.503 Y114.889 E0.29132

G1 X128.794 Y114.588 E0.01093

G1 X120.613 Y106.407 E0.30207

G1 X120.613 Y105.815 E0.01546

G1 X129.105 Y114.306 E0.31353

G1 X129.433 Y114.042 E0.01099

G1 X120.613 Y105.223 E0.32563

G1 X120.613 Y104.631 E0.01546

G1 X129.775 Y113.792 E0.33826

G1 X130.140 Y113.566 E0.01123

G1 X121.021 Y104.447 E0.33670

G1 X121.613 Y104.447 E0.01546

G1 X130.517 Y113.351 E0.32875

G1 X130.922 Y113.164 E0.01165

G1 X122.205 Y104.447 E0.32186

```
G1 X122.797 Y104.447 E0.01546
G1 X131.339 Y112.989 E0.31540
G1 X131.788 Y112.846 E0.01230
G1 X123.389 Y104.447 E0.31012
G1 X123.981 Y104.447 E0.01546
G1 X132.255 Y112.721 E0.30549
G1 X132.754 Y112.627 E0.01325
G1 X124.574 Y104.447 E0.30204
G1 X125.166 Y104.447 E0.01546
G1 X133.464 Y112.746 E0.30641
M204 S1250
G1 E-2.24000 F2400.000
;WIPE_START
G1 F7200.000
G1 X131.428 Y110.709 E-0.91200
;WIPE_END
G1 E-0.04800 F2400.000
G1 Z1.000 F9000.000
G1 X140.889 Y110.105
G1 Z0.800
G1 E3.20000 F1500.000
M204 S1000
G1 F4800.000
G1 X140.231 Y109.447 E0.02431
G1 X139.639 Y109.447 E0.01546
G1 X140.701 Y110.509 E0.03923
G1 X140.701 Y111.101 E0.01546
G1 X139.046 Y109.447 E0.06109
G1 X138.454 Y109.447 E0.01546
```

G1 X140.701 Y111.693 E0.08295

G1 X140.701 Y112.285 E0.01546

G1 X137.862 Y109.447 E0.10481

G1 X137.270 Y109.447 E0.01546

G1 X140.358 Y112.534 E0.11400

G1 X139.766 Y112.534 E0.01546

G1 X136.678 Y109.447 E0.11400

G1 X136.086 Y109.447 E0.01546

G1 X139.174 Y112.534 E0.11400

G1 X138.582 Y112.534 E0.01546

G1 X135.494 Y109.447 E0.11400

G1 X134.902 Y109.447 E0.01546

G1 X137.990 Y112.534 E0.11400

G1 X137.398 Y112.534 E0.01546

G1 X134.310 Y109.447 E0.11400

G1 X133.710 Y109.439 E0.01566

G1 X136.806 Y112.534 E0.11428

G1 X136.214 Y112.534 E0.01546

G1 X133.079 Y109.400 E0.11572

G1 X132.369 Y109.282 E0.01880

G1 X135.621 Y112.534 E0.12009

G1 X135.029 Y112.534 E0.01546

G1 X131.556 Y109.061 E0.12823

G1 X131.219 Y108.954 E0.00923

G1 X130.548 Y108.645 E0.01931

G1 X134.437 Y112.534 E0.14362

G1 X133.845 Y112.534 E0.01546

G1 X125.758 Y104.447 E0.29862

G1 X126.350 Y104.447 E0.01546

G1 X127.859 Y105.956 E0.05573

M204 S1250

; stop printing object tpu print.STL id:23 copy 0

; printing object tpu print.STL id:21 copy 0

G1 E-2.24000 F2400.000

;WIPE_START

G1 F7200.000

G1 X126.350 Y104.447 E-0.67595

G1 X125.758 Y104.447 E-0.18749

G1 X125.866 Y104.555 E-0.04857

;WIPE_END

G1 E-0.04800 F2400.000

G1 Z1.000 F9000.000

G1 X127.164 Y97.032

G1 Z0.800

G1 E3.20000 F1500.000

M204 S800

;TYPE:Perimeter

;WIDTH:0.45

G1 F2400.000

G1 X120.293 Y97.032 E0.17903

G1 X120.293 Y83.318 E0.35734

G1 X127.164 Y83.318 E0.17903

G1 X127.256 Y83.641 E0.00876

G1 X127.591 Y84.436 E0.02246

G1 X128.021 Y85.183 E0.02246

G1 X128.540 Y85.871 E0.02246

G1 X129.139 Y86.490 E0.02246

G1 X129.811 Y87.031 E0.02246

```
G1 X130.543 Y87.485 E0.02246
G1 X131.326 Y87.846 E0.02246
G1 X132.147 Y88.107 E0.02246
G1 X132.995 Y88.266 E0.02246
G1 X133.835 Y88.318 E0.02194
G1 X141.007 Y88.318 E0.18688
G1 X141.007 Y92.032 E0.09678
G1 X133.835 Y92.032 E0.18688
G1 X132.995 Y92.084 E0.02194
G1 X132.147 Y92.243 E0.02246
G1 X131.326 Y92.504 E0.02246
G1 X130.543 Y92.865 E0.02246
G1 X129.811 Y93.319 E0.02246
G1 X129.139 Y93.860 E0.02246
G1 X128.540 Y94.479 E0.02246
G1 X128.021 Y95.168 E0.02246
G1 X127.591 Y95.915 E0.02246
G1 X127.256 Y96.709 E0.02246
G1 X127.181 Y96.975 E0.00720
M204 S1250
G1 X127.480 Y97.450 F9000.000
M204 S800
;TYPE:External perimeter
G1 F1800.000
G1 X119.875 Y97.450 E0.19815
G1 X119.875 Y82.900 E0.37911
G1 X127.480 Y82.900 E0.19815
G1 X127.651 Y83.503 E0.01632
G1 X127.966 Y84.249 E0.02112
```

```
G1 X128.370 Y84.952 E0.02112
G1 X128.858 Y85.599 E0.02112
G1 X129.422 Y86.181 E0.02112
G1 X130.053 Y86.690 E0.02112
G1 X130.741 Y87.117 E0.02112
G1 X131.477 Y87.456 E0.02112
G1 X132.250 Y87.702 E0.02112
G1 X133.046 Y87.851 E0.02112
G1 X133.848 Y87.900 E0.02093
G1 X141.425 Y87.900 E0.19743
G1 X141.425 Y92.450 E0.11855
G1 X133.848 Y92.450 E0.19743
G1 X133.046 Y92.500 E0.02093
G1 X132.250 Y92.649 E0.02112
G1 X131.477 Y92.895 E0.02112
G1 X130.741 Y93.234 E0.02112
G1 X130.053 Y93.661 E0.02112
G1 X129.422 Y94.169 E0.02112
G1 X128.858 Y94.751 E0.02112
G1 X128.370 Y95.398 E0.02112
G1 X127.966 Y96.101 E0.02112
G1 X127.651 Y96.847 E0.02112
G1 X127.496 Y97.392 E0.01476
M204 S1250
G1 X127.159 Y97.212 F9000.000
G1 E-2.24000 F2400.000
;WIPE_START
G1 F7200.000
G1 X124.617 Y97.414 E-0.91200
```

```
;WIPE_END
G1 E-0.04800 F2400.000
G1 Z1.000 F9000.000
G1 X121.265 Y96.907
G1 Z0.800
G1 E3.20000 F1500.000
M204 S1000
;TYPE:Solid infill
;WIDTH:0.450839
G1 F4800.000
G1 X120.606 Y96.249 E0.02431
G1 X120.606 Y95.657 E0.01546
G1 X121.669 Y96.719 E0.03922
G1 X122.261 Y96.719 E0.01546
G1 X120.606 Y95.065 E0.06108
G1 X120.606 Y94.472 E0.01546
G1 X122.853 Y96.719 E0.08294
G1 X123.445 Y96.719 E0.01546
G1 X120.606 Y93.880 E0.10480
G1 X120.606 Y93.288 E0.01546
G1 X124.037 Y96.719 E0.12666
G1 X124.629 Y96.719 E0.01546
G1 X120.606 Y92.696 E0.14853
G1 X120.606 Y92.104 E0.01546
G1 X125.221 Y96.719 E0.17039
G1 X125.813 Y96.719 E0.01546
G1 X120.606 Y91.512 E0.19225
G1 X120.606 Y90.920 E0.01546
G1 X126.405 Y96.719 E0.21411
```

```
G1 X126.927 Y96.719 E0.01363
G1 X126.943 Y96.664 E0.00148
G1 X120.606 Y90.328 E0.23395
G1 X120.606 Y89.736 E0.01546
G1 X127.113 Y96.242 E0.24022
G1 X127.288 Y95.826 E0.01180
G1 X120.606 Y89.144 E0.24671
G1 X120.606 Y88.552 E0.01546
G1 X127.500 Y95.445 E0.25451
G1 X127.716 Y95.069 E0.01132
G1 X120.606 Y87.960 E0.26250
G1 X120.606 Y87.368 E0.01546
G1 X127.963 Y94.724 E0.27162
G1 X128.217 Y94.387 E0.01104
G1 X120.606 Y86.776 E0.28101
G1 X120.606 Y86.184 E0.01546
G1 X128.496 Y94.074 E0.29132
G1 X128.788 Y93.773 E0.01093
G1 X120.606 Y85.592 E0.30207
G1 X120.606 Y85.000 E0.01546
G1 X129.098 Y93.491 E0.31353
G1 X129.426 Y93.227 E0.01099
G1 X120.606 Y84.407 E0.32563
G1 X120.606 Y83.815 E0.01546
G1 X129.768 Y92.977 E0.33826
G1 X130.133 Y92.750 E0.01123
G1 X121.014 Y83.631 E0.33670
G1 X121.606 Y83.631 E0.01546
G1 X130.510 Y92.535 E0.32875
```

```
G1 X130.916 Y92.348 E0.01165
G1 X122.198 Y83.631 E0.32186
G1 X122.790 Y83.631 E0.01546
G1 X131.333 Y92.173 E0.31540
G1 X131.782 Y92.030 E0.01230
G1 X123.383 Y83.631 E0.31012
G1 X123.975 Y83.631 E0.01546
G1 X132.248 Y91.905 E0.30549
G1 X132.747 Y91.812 E0.01325
G1 X124.567 Y83.631 E0.30204
G1 X125.159 Y83.631 E0.01546
G1 X133.457 Y91.930 E0.30641
M204 S1250
G1 E-2.24000 F2400.000
;WIPE_START
G1 F7200.000
G1 X131.421 Y89.894 E-0.91200
;WIPE_END
G1 E-0.04800 F2400.000
G1 Z1.000 F9000.000
G1 X140.882 Y89.290
G1 Z0.800
G1 E3.20000 F1500.000
M204 S1000
G1 F4800.000
G1 X140.224 Y88.631 E0.02431
G1 X139.632 Y88.631 E0.01546
G1 X140.694 Y89.694 E0.03923
G1 X140.694 Y90.286 E0.01546
```

G1 X139.040 Y88.631 E0.06109

G1 X138.448 Y88.631 E0.01546

G1 X140.694 Y90.878 E0.08295

G1 X140.694 Y91.470 E0.01546

G1 X137.855 Y88.631 E0.10481

G1 X137.263 Y88.631 E0.01546

G1 X140.351 Y91.719 E0.11400

G1 X139.759 Y91.719 E0.01546

G1 X136.671 Y88.631 E0.11400

G1 X136.079 Y88.631 E0.01546

G1 X139.167 Y91.719 E0.11400

G1 X138.575 Y91.719 E0.01546

G1 X135.487 Y88.631 E0.11400

G1 X134.895 Y88.631 E0.01546

G1 X137.983 Y91.719 E0.11400

G1 X137.391 Y91.719 E0.01546

G1 X134.303 Y88.631 E0.11400

G1 X133.704 Y88.624 E0.01566

G1 X136.799 Y91.719 E0.11428

G1 X136.207 Y91.719 E0.01546

G1 X133.073 Y88.585 E0.11572

G1 X132.362 Y88.466 E0.01880

G1 X135.615 Y91.719 E0.12009

G1 X135.023 Y91.719 E0.01546

G1 X131.550 Y88.246 E0.12823

G1 X131.213 Y88.139 E0.00923

G1 X130.541 Y87.829 E0.01931

G1 X134.431 Y91.719 E0.14362

G1 X133.838 Y91.719 E0.01546

```
G1 X125.751 Y83.631 E0.29862
G1 X126.343 Y83.631 E0.01546
G1 X127.852 Y85.141 E0.05573
M204 S1250
; stop printing object tpu print.STL id:21 copy 0
; printing object Petg print.STL id:20 copy 0
; stop printing object Petg print.STL id:20 copy 0
; printing object Petg print.STL id:22 copy 0
; stop printing object Petg print.STL id:22 copy 0
; printing object Petg print.STL id:26 copy 0
; stop printing object Petg print.STL id:26 copy 0
; printing object Petg print.STL id:0 copy 0
; stop printing object Petg print.STL id:0 copy 0
; printing object tpu print.STL id:1 copy 0
G1 E-2.24000 F2400.000
;WIPE_START
G1 F7200.000
G1 X126.343 Y83.631 E-0.67595
G1 X125.751 Y83.631 E-0.18749
G1 X125.859 Y83.740 E-0.04857
;WIPE_END
G1 E-0.04800 F2400.000
G1 Z1.000 F9000.000
G1 X90.357 Y88.143
G1 Z0.800
G1 E3.20000 F1500.000
M204 S800
;TYPE:Perimeter
;WIDTH:0.45
```

```
G1 F2400.000
G1 X90.357 Y91.857 E0.09678
G1 X83.185 Y91.857 E0.18688
G1 X82.344 Y91.909 E0.02194
G1 X81.497 Y92.068 E0.02246
G1 X80.676 Y92.329 E0.02246
G1 X79.893 Y92.690 E0.02246
G1 X79.160 Y93.144 E0.02246
G1 X78.489 Y93.685 E0.02246
G1 X77.890 Y94.304 E0.02246
G1 X77.371 Y94.993 E0.02246
G1 X76.941 Y95.739 E0.02246
G1 X76.606 Y96.534 E0.02246
G1 X76.514 Y96.857 E0.00876
G1 X69.643 Y96.857 E0.17903
G1 X69.643 Y83.143 E0.35734
G1 X76.514 Y83.143 E0.17903
G1 X76.606 Y83.466 E0.00876
G1 X76.941 Y84.261 E0.02246
G1 X77.371 Y85.007 E0.02246
G1 X77.890 Y85.696 E0.02246
G1 X78.489 Y86.315 E0.02246
G1 X79.160 Y86.856 E0.02246
G1 X79.893 Y87.310 E0.02246
G1 X80.676 Y87.671 E0.02246
G1 X81.497 Y87.932 E0.02246
G1 X82.344 Y88.091 E0.02246
G1 X83.185 Y88.143 E0.02194
G1 X90.297 Y88.143 E0.18531
```

```
M204 S1250
G1 X90.775 Y87.725 F9000.000
M204 S800
;TYPE:External perimeter
G1 F1800.000
G1 X90.775 Y92.275 E0.11855
G1 X83.198 Y92.275 E0.19743
G1 X82.396 Y92.325 E0.02093
G1 X81.599 Y92.474 E0.02112
G1 X80.827 Y92.719 E0.02112
G1 X80.091 Y93.059 E0.02112
G1 X79.402 Y93.486 E0.02112
G1 X78.771 Y93.994 E0.02112
G1 X78.208 Y94.576 E0.02112
G1 X77.720 Y95.223 E0.02112
G1 X77.315 Y95.926 E0.02112
G1 X77.000 Y96.672 E0.02112
G1 X76.830 Y97.275 E0.01632
G1 X69.225 Y97.275 E0.19815
G1 X69.225 Y82.725 E0.37911
G1 X76.830 Y82.725 E0.19815
G1 X77.000 Y83.328 E0.01632
G1 X77.315 Y84.074 E0.02112
G1 X77.720 Y84.777 E0.02112
G1 X78.208 Y85.424 E0.02112
G1 X78.771 Y86.006 E0.02112
G1 X79.402 Y86.514 E0.02112
G1 X80.091 Y86.941 E0.02112
G1 X80.827 Y87.281 E0.02112
```

```
G1 X81.599 Y87.526 E0.02112
G1 X82.396 Y87.675 E0.02112
G1 X83.198 Y87.725 E0.02093
G1 X90.715 Y87.725 E0.19586
M204 S1250
G1 X90.574 Y88.071 F9000.000
G1 E-2.24000 F2400.000
;WIPE_START
G1 F7200.000
G1 X90.753 Y90.605 E-0.91200
;WIPE_END
G1 E-0.04800 F2400.000
G1 Z1.000 F9000.000
G1 X90.232 Y89.115
G1 Z0.800
G1 E3.20000 F1500.000
M204 S1000
;TYPE:Solid infill
;WIDTH:0.450839
G1 F4800.000
G1 X89.573 Y88.456 E0.02431
G1 X88.981 Y88.456 E0.01546
G1 X90.044 Y89.519 E0.03923
M73 P81 R12
G1 X90.044 Y90.111 E0.01546
G1 X88.389 Y88.456 E0.06109
G1 X87.797 Y88.456 E0.01546
G1 X90.044 Y90.703 E0.08295
G1 X90.044 Y91.295 E0.01546
```

```
G1 X87.205 Y88.456 E0.10481
G1 X86.613 Y88.456 E0.01546
G1 X89.701 Y91.544 E0.11400
G1 X89.109 Y91.544 E0.01546
G1 X86.021 Y88.456 E0.11400
G1 X85.429 Y88.456 E0.01546
G1 X88.517 Y91.544 E0.11400
G1 X87.925 Y91.544 E0.01546
G1 X84.837 Y88.456 E0.11400
G1 X84.245 Y88.456 E0.01546
G1 X87.333 Y91.544 E0.11400
G1 X86.741 Y91.544 E0.01546
G1 X83.653 Y88.456 E0.11400
G1 X83.053 Y88.449 E0.01566
G1 X86.149 Y91.544 E0.11428
G1 X85.556 Y91.544 E0.01546
G1 X82.422 Y88.410 E0.11572
G1 X81.712 Y88.291 E0.01880
G1 X84.964 Y91.544 E0.12009
G1 X84.372 Y91.544 E0.01546
G1 X80.899 Y88.071 E0.12823
G1 X80.562 Y87.964 E0.00923
G1 X79.890 Y87.654 E0.01931
G1 X83.780 Y91.544 E0.14362
G1 X83.188 Y91.544 E0.01546
G1 X75.101 Y83.456 E0.29862
G1 X75.693 Y83.456 E0.01546
G1 X77.202 Y84.966 E0.05573
M204 S1250
```

```
G1 E-2.24000 F2400.000
;WIPE_START
G1 F7200.000
G1 X75.693 Y83.456 E-0.67595
G1 X75.101 Y83.456 E-0.18749
G1 X75.209 Y83.565 E-0.04857
;WIPE_END
G1 E-0.04800 F2400.000
G1 Z1.000 F9000.000
G1 X82.807 Y91.755
G1 Z0.800
G1 E3.20000 F1500.000
M204 S1000
G1 F4800.000
G1 X74.508 Y83.456 E0.30641
G1 X73.916 Y83.456 E0.01546
G1 X82.097 Y91.637 E0.30204
G1 X81.598 Y91.730 E0.01325
G1 X73.324 Y83.456 E0.30549
G1 X72.732 Y83.456 E0.01546
G1 X81.131 Y91.855 E0.31012
G1 X80.682 Y91.998 E0.01230
G1 X72.140 Y83.456 E0.31540
G1 X71.548 Y83.456 E0.01546
G1 X80.265 Y92.173 E0.32186
G1 X79.860 Y92.360 E0.01165
G1 X70.956 Y83.456 E0.32875
G1 X70.364 Y83.456 E0.01546
G1 X79.483 Y92.575 E0.33670
```

G1 X79.118 Y92.802 E0.01123

G1 X69.956 Y83.640 E0.33826

G1 X69.956 Y84.232 E0.01546

G1 X78.775 Y93.052 E0.32563

G1 X78.448 Y93.316 E0.01099

G1 X69.956 Y84.824 E0.31353

G1 X69.956 Y85.416 E0.01546

G1 X78.137 Y93.598 E0.30207

G1 X77.846 Y93.899 E0.01093

G1 X69.956 Y86.009 E0.29132

G1 X69.956 Y86.601 E0.01546

G1 X77.567 Y94.211 E0.28101

G1 X77.313 Y94.549 E0.01104

G1 X69.956 Y87.193 E0.27162

G1 X69.956 Y87.785 E0.01546

G1 X77.066 Y94.894 E0.26250

G1 X76.849 Y95.270 E0.01132

G1 X69.956 Y88.377 E0.25451

G1 X69.956 Y88.969 E0.01546

G1 X76.638 Y95.651 E0.24671

G1 X76.462 Y96.067 E0.01180

G1 X69.956 Y89.561 E0.24022

G1 X69.956 Y90.153 E0.01546

G1 X76.292 Y96.489 E0.23395

G1 X76.277 Y96.544 E0.00148

G1 X75.755 Y96.544 E0.01363

G1 X69.956 Y90.745 E0.21411

G1 X69.956 Y91.337 E0.01546

G1 X75.163 Y96.544 E0.19225

```
G1 X74.571 Y96.544 E0.01546
G1 X69.956 Y91.929 E0.17039
G1 X69.956 Y92.521 E0.01546
G1 X73.979 Y96.544 E0.14853
G1 X73.387 Y96.544 E0.01546
G1 X69.956 Y93.113 E0.12666
G1 X69.956 Y93.705 E0.01546
G1 X72.795 Y96.544 E0.10480
G1 X72.203 Y96.544 E0.01546
G1 X69.956 Y94.297 E0.08294
G1 X69.956 Y94.889 E0.01546
G1 X71.611 Y96.544 E0.06108
G1 X71.018 Y96.544 E0.01546
G1 X69.956 Y95.482 E0.03922
G1 X69.956 Y96.074 E0.01546
G1 X70.614 Y96.732 E0.02431
M204 S1250
; stop printing object tpu print.STL id:1 copy 0
; printing object tpu print.STL id:5 copy 0
G1 E-2.24000 F2400.000
;WIPE_START
G1 F7200.000
G1 X69.956 Y96.074 E-0.29480
G1 X69.956 Y95.482 E-0.18749
G1 X70.916 Y96.441 E-0.42971
;WIPE_END
G1 E-0.04800 F2400.000
G1 Z1.000 F9000.000
G1 X69.694 Y74.665
```

```
G1 Z0.800
G1 E3.20000 F1500.000
M204 S800
;TYPE:Perimeter
;WIDTH:0.45
G1 F2400.000
G1 X69.694 Y60.950 E0.35734
G1 X76.565 Y60.950 E0.17903
G1 X76.657 Y61.274 E0.00876
G1 X76.992 Y62.068 E0.02246
G1 X77.422 Y62.815 E0.02246
G1 X77.941 Y63.503 E0.02246
G1 X78.540 Y64.123 E0.02246
G1 X79.211 Y64.664 E0.02246
G1 X79.944 Y65.118 E0.02246
G1 X80.727 Y65.478 E0.02246
G1 X81.548 Y65.740 E0.02246
G1 X82.396 Y65.899 E0.02246
G1 X83.236 Y65.950 E0.02194
G1 X90.408 Y65.950 E0.18688
G1 X90.408 Y69.665 E0.09678
G1 X83.236 Y69.665 E0.18688
G1 X82.396 Y69.717 E0.02194
G1 X81.548 Y69.875 E0.02246
G1 X80.727 Y70.137 E0.02246
G1 X79.944 Y70.497 E0.02246
G1 X79.211 Y70.952 E0.02246
G1 X78.540 Y71.493 E0.02246
G1 X77.941 Y72.112 E0.02246
```

```
G1 X77.422 Y72.800 E0.02246
G1 X76.992 Y73.547 E0.02246
G1 X76.657 Y74.341 E0.02246
G1 X76.565 Y74.665 E0.00876
G1 X69.754 Y74.665 E0.17747
M204 S1250
G1 X69.276 Y75.083 F9000.000
M204 S800
;TYPE:External perimeter
G1 F1800.000
G1 X69.276 Y60.533 E0.37911
G1 X76.881 Y60.533 E0.19815
G1 X77.052 Y61.135 E0.01632
G1 X77.367 Y61.882 E0.02112
G1 X77.771 Y62.584 E0.02112
G1 X78.259 Y63.231 E0.02112
G1 X78.823 Y63.814 E0.02112
G1 X79.454 Y64.322 E0.02112
G1 X80.142 Y64.749 E0.02112
G1 X80.878 Y65.088 E0.02112
G1 X81.651 Y65.334 E0.02112
G1 X82.447 Y65.483 E0.02112
G1 X83.249 Y65.533 E0.02093
G1 X90.826 Y65.533 E0.19743
G1 X90.826 Y70.083 E0.11855
M73 P82 R12
G1 X83.249 Y70.083 E0.19743
G1 X82.447 Y70.132 E0.02093
G1 X81.651 Y70.281 E0.02112
```

```
G1 X80.878 Y70.527 E0.02112
G1 X80.142 Y70.866 E0.02112
G1 X79.454 Y71.293 E0.02112
G1 X78.823 Y71.802 E0.02112
G1 X78.259 Y72.384 E0.02112
G1 X77.771 Y73.031 E0.02112
G1 X77.367 Y73.733 E0.02112
G1 X77.052 Y74.480 E0.02112
G1 X76.881 Y75.083 E0.01632
G1 X69.336 Y75.083 E0.19659
M204 S1250
G1 X69.467 Y74.731 F9000.000
G1 E-2.24000 F2400.000
;WIPE_START
G1 F7200.000
G1 X69.324 Y72.203 E-0.91200
;WIPE_END
G1 E-0.04800 F2400.000
G1 Z1.000 F9000.000
G1 X70.666 Y74.539
G1 Z0.800
G1 E3.20000 F1500.000
M204 S1000
;TYPE:Solid infill
;WIDTH:0.450839
G1 F4800.000
G1 X70.007 Y73.881 E0.02431
G1 X70.007 Y73.289 E0.01546
G1 X71.070 Y74.351 E0.03922
```

G1 X71.662 Y74.351 E0.01546

G1 X70.007 Y72.697 E0.06108

G1 X70.007 Y72.105 E0.01546

G1 X72.254 Y74.351 E0.08294

G1 X72.846 Y74.351 E0.01546

G1 X70.007 Y71.513 E0.10480

G1 X70.007 Y70.921 E0.01546

G1 X73.438 Y74.351 E0.12666

G1 X74.030 Y74.351 E0.01546

G1 X70.007 Y70.329 E0.14853

G1 X70.007 Y69.737 E0.01546

G1 X74.622 Y74.351 E0.17039

G1 X75.214 Y74.351 E0.01546

G1 X70.007 Y69.145 E0.19225

G1 X70.007 Y68.553 E0.01546

G1 X75.806 Y74.351 E0.21411

G1 X76.328 Y74.351 E0.01363

G1 X76.344 Y74.297 E0.00148

G1 X70.007 Y67.961 E0.23395

G1 X70.007 Y67.369 E0.01546

G1 X76.513 Y73.875 E0.24022

G1 X76.689 Y73.458 E0.01180

G1 X70.007 Y66.777 E0.24671

G1 X70.007 Y66.184 E0.01546

G1 X76.900 Y73.078 E0.25451

G1 X77.117 Y72.702 E0.01132

G1 X70.007 Y65.592 E0.26250

G1 X70.007 Y65.000 E0.01546

G1 X77.364 Y72.357 E0.27162

G1 X77.618 Y72.019 E0.01104

G1 X70.007 Y64.408 E0.28101

G1 X70.007 Y63.816 E0.01546

G1 X77.897 Y71.706 E0.29132

G1 X78.189 Y71.405 E0.01093

G1 X70.007 Y63.224 E0.30207

G1 X70.007 Y62.632 E0.01546

G1 X78.499 Y71.124 E0.31353

G1 X78.827 Y70.859 E0.01099

G1 X70.007 Y62.040 E0.32563

G1 X70.007 Y61.448 E0.01546

G1 X79.169 Y70.609 E0.33826

G1 X79.534 Y70.383 E0.01123

G1 X70.415 Y61.264 E0.33670

G1 X71.007 Y61.264 E0.01546

G1 X79.911 Y70.168 E0.32875

G1 X80.316 Y69.981 E0.01165

G1 X71.599 Y61.264 E0.32186

G1 X72.191 Y61.264 E0.01546

G1 X80.733 Y69.806 E0.31540

G1 X81.183 Y69.663 E0.01230

G1 X72.783 Y61.264 E0.31012

G1 X73.376 Y61.264 E0.01546

G1 X81.649 Y69.538 E0.30549

G1 X82.148 Y69.444 E0.01325

G1 X73.968 Y61.264 E0.30204

G1 X74.560 Y61.264 E0.01546

G1 X82.858 Y69.563 E0.30641

M204 S1250

```
G1 E-2.24000 F2400.000
;WIPE_START
G1 F7200.000
G1 X80.822 Y67.526 E-0.91200
;WIPE_END
G1 E-0.04800 F2400.000
G1 Z1.000 F9000.000
G1 X90.283 Y66.922
G1 Z0.800
G1 E3.20000 F1500.000
M204 S1000
G1 F4800.000
G1 X89.625 Y66.264 E0.02431
G1 X89.033 Y66.264 E0.01546
G1 X90.095 Y67.326 E0.03923
G1 X90.095 Y67.918 E0.01546
G1 X88.441 Y66.264 E0.06109
G1 X87.848 Y66.264 E0.01546
G1 X90.095 Y68.510 E0.08295
G1 X90.095 Y69.102 E0.01546
G1 X87.256 Y66.264 E0.10481
G1 X86.664 Y66.264 E0.01546
G1 X89.752 Y69.351 E0.11400
G1 X89.160 Y69.351 E0.01546
G1 X86.072 Y66.264 E0.11400
G1 X85.480 Y66.264 E0.01546
G1 X88.568 Y69.351 E0.11400
G1 X87.976 Y69.351 E0.01546
G1 X84.888 Y66.264 E0.11400
```

```
G1 X84.296 Y66.264 E0.01546
G1 X87.384 Y69.351 E0.11400
G1 X86.792 Y69.351 E0.01546
G1 X83.704 Y66.264 E0.11400
G1 X83.104 Y66.256 E0.01566
G1 X86.200 Y69.351 E0.11428
G1 X85.608 Y69.351 E0.01546
G1 X82.473 Y66.217 E0.11572
G1 X81.763 Y66.099 E0.01880
G1 X85.016 Y69.351 E0.12009
G1 X84.423 Y69.351 E0.01546
G1 X80.950 Y65.878 E0.12823
G1 X80.613 Y65.771 E0.00923
G1 X79.942 Y65.462 E0.01931
G1 X83.831 Y69.351 E0.14362
G1 X83.239 Y69.351 E0.01546
G1 X75.152 Y61.264 E0.29862
G1 X75.744 Y61.264 E0.01546
G1 X77.253 Y62.773 E0.05573
M204 S1250
; stop printing object tpu print.STL id:5 copy 0
; printing object tpu print.STL id:9 copy 0
G1 E-2.24000 F2400.000
;WIPE_START
G1 F7200.000
G1 X75.744 Y61.264 E-0.67595
G1 X75.152 Y61.264 E-0.18749
G1 X75.260 Y61.372 E-0.04857
;WIPE_END
```

G1 E-0.04800 F2400.000

G1 Z1.000 F9000.000

G1 X76.558 Y54.716

G1 Z0.800

G1 E3.20000 F1500.000

M204 S800

;TYPE:Perimeter

;WIDTH:0.45

G1 F2400.000

G1 X69.686 Y54.716 E0.17903

G1 X69.686 Y41.002 E0.35734

G1 X76.558 Y41.002 E0.17903

G1 X76.649 Y41.325 E0.00876

G1 X76.984 Y42.119 E0.02246

G1 X77.414 Y42.866 E0.02246

G1 X77.933 Y43.555 E0.02246

G1 X78.533 Y44.174 E0.02246

G1 X79.204 Y44.715 E0.02246

G1 X79.937 Y45.169 E0.02246

G1 X80.720 Y45.530 E0.02246

G1 X81.541 Y45.791 E0.02246

G1 X82.388 Y45.950 E0.02246

G1 X83.229 Y46.002 E0.02194

G1 X90.401 Y46.002 E0.18688

G1 X90.401 Y49.716 E0.09678

G1 X83.229 Y49.716 E0.18688

G1 X82.388 Y49.768 E0.02194

G1 X81.541 Y49.926 E0.02246

G1 X80.720 Y50.188 E0.02246

```
G1 X79.937 Y50.549 E0.02246
G1 X79.204 Y51.003 E0.02246
G1 X78.533 Y51.544 E0.02246
G1 X77.933 Y52.163 E0.02246
G1 X77.414 Y52.851 E0.02246
G1 X76.984 Y53.598 E0.02246
G1 X76.649 Y54.392 E0.02246
G1 X76.574 Y54.658 E0.00720
M204 S1250
G1 X76.874 Y55.134 F9000.000
M204 S800
;TYPE:External perimeter
G1 F1800.000
G1 X69.269 Y55.134 E0.19815
G1 X69.269 Y40.584 E0.37911
G1 X76.874 Y40.584 E0.19815
G1 X77.044 Y41.186 E0.01632
G1 X77.359 Y41.933 E0.02112
G1 X77.764 Y42.635 E0.02112
G1 X78.251 Y43.282 E0.02112
G1 X78.815 Y43.865 E0.02112
G1 X79.446 Y44.373 E0.02112
G1 X80.135 Y44.800 E0.02112
G1 X80.871 Y45.139 E0.02112
G1 X81.643 Y45.385 E0.02112
G1 X82.440 Y45.534 E0.02112
G1 X83.242 Y45.584 E0.02093
G1 X90.819 Y45.584 E0.19743
G1 X90.819 Y50.134 E0.11855
```

```
G1 X83.242 Y50.134 E0.19743
G1 X82.440 Y50.183 E0.02093
G1 X81.643 Y50.332 E0.02112
G1 X80.871 Y50.578 E0.02112
G1 X80.135 Y50.917 E0.02112
G1 X79.446 Y51.344 E0.02112
G1 X78.815 Y51.853 E0.02112
G1 X78.251 Y52.435 E0.02112
G1 X77.764 Y53.082 E0.02112
G1 X77.359 Y53.784 E0.02112
G1 X77.044 Y54.531 E0.02112
G1 X76.890 Y55.076 E0.01476
M204 S1250
G1 X76.552 Y54.895 F9000.000
G1 E-2.24000 F2400.000
;WIPE_START
G1 F7200.000
G1 X74.010 Y55.098 E-0.91200
;WIPE_END
G1 E-0.04800 F2400.000
G1 Z1.000 F9000.000
G1 X70.658 Y54.591
G1 Z0.800
G1 E3.20000 F1500.000
M204 S1000
;TYPE:Solid infill
;WIDTH:0.450839
G1 F4800.000
G1 X70.000 Y53.932 E0.02431
```

```
G1 X70.000 Y53.340 E0.01546
G1 X71.062 Y54.403 E0.03922
G1 X71.654 Y54.403 E0.01546
G1 X70.000 Y52.748 E0.06108
G1 X70.000 Y52.156 E0.01546
G1 X72.246 Y54.403 E0.08294
G1 X72.838 Y54.403 E0.01546
G1 X70.000 Y51.564 E0.10480
G1 X70.000 Y50.972 E0.01546
G1 X73.430 Y54.403 E0.12666
G1 X74.022 Y54.403 E0.01546
G1 X70.000 Y50.380 E0.14853
G1 X70.000 Y49.788 E0.01546
G1 X74.615 Y54.403 E0.17039
G1 X75.207 Y54.403 E0.01546
G1 X70.000 Y49.196 E0.19225
G1 X70.000 Y48.604 E0.01546
G1 X75.799 Y54.403 E0.21411
G1 X76.321 Y54.403 E0.01363
G1 X76.336 Y54.348 E0.00148
G1 X70.000 Y48.012 E0.23395
G1 X70.000 Y47.420 E0.01546
G1 X76.506 Y53.926 E0.24022
G1 X76.682 Y53.509 E0.01180
G1 X70.000 Y46.828 E0.24671
G1 X70.000 Y46.235 E0.01546
G1 X76.893 Y53.129 E0.25451
G1 X77.109 Y52.753 E0.01132
G1 X70.000 Y45.643 E0.26250
```

G1 X70.000 Y45.051 E0.01546

G1 X77.356 Y52.408 E0.27162

G1 X77.611 Y52.070 E0.01104

G1 X70.000 Y44.459 E0.28101

G1 X70.000 Y43.867 E0.01546

G1 X77.890 Y51.757 E0.29132

G1 X78.181 Y51.456 E0.01093

G1 X70.000 Y43.275 E0.30207

G1 X70.000 Y42.683 E0.01546

G1 X78.491 Y51.175 E0.31353

G1 X78.819 Y50.910 E0.01099

G1 X70.000 Y42.091 E0.32563

G1 X70.000 Y41.499 E0.01546

G1 X79.161 Y50.660 E0.33826

G1 X79.527 Y50.434 E0.01123

G1 X70.408 Y41.315 E0.33670

G1 X71.000 Y41.315 E0.01546

G1 X79.904 Y50.219 E0.32875

G1 X80.309 Y50.032 E0.01165

G1 X71.592 Y41.315 E0.32186

G1 X72.184 Y41.315 E0.01546

G1 X80.726 Y49.857 E0.31540

G1 X81.175 Y49.714 E0.01230

G1 X72.776 Y41.315 E0.31012

G1 X73.368 Y41.315 E0.01546

G1 X81.642 Y49.589 E0.30549

G1 X82.141 Y49.495 E0.01325

G1 X73.960 Y41.315 E0.30204

G1 X74.552 Y41.315 E0.01546

```
G1 X82.851 Y49.614 E0.30641
M204 S1250
G1 E-2.24000 F2400.000
;WIPE_START
G1 F7200.000
G1 X80.814 Y47.577 E-0.91200
;WIPE_END
G1 E-0.04800 F2400.000
G1 Z1.000 F9000.000
G1 X90.276 Y46.973
G1 Z0.800
G1 E3.20000 F1500.000
M204 S1000
G1 F4800.000
G1 X89.617 Y46.315 E0.02431
G1 X89.025 Y46.315 E0.01546
G1 X90.087 Y47.377 E0.03923
G1 X90.087 Y47.969 E0.01546
G1 X88.433 Y46.315 E0.06109
G1 X87.841 Y46.315 E0.01546
G1 X90.087 Y48.561 E0.08295
G1 X90.087 Y49.153 E0.01546
G1 X87.249 Y46.315 E0.10481
G1 X86.657 Y46.315 E0.01546
G1 X89.745 Y49.403 E0.11400
G1 X89.152 Y49.403 E0.01546
G1 X86.065 Y46.315 E0.11400
G1 X85.473 Y46.315 E0.01546
G1 X88.560 Y49.403 E0.11400
```

G1 X87.968 Y49.403 E0.01546

G1 X84.881 Y46.315 E0.11400

G1 X84.289 Y46.315 E0.01546

G1 X87.376 Y49.403 E0.11400

G1 X86.784 Y49.403 E0.01546

G1 X83.697 Y46.315 E0.11400

G1 X83.097 Y46.307 E0.01566

G1 X86.192 Y49.403 E0.11428

G1 X85.600 Y49.403 E0.01546

G1 X82.466 Y46.268 E0.11572

G1 X81.756 Y46.150 E0.01880

G1 X85.008 Y49.403 E0.12009

G1 X84.416 Y49.403 E0.01546

G1 X80.943 Y45.930 E0.12823

G1 X80.606 Y45.822 E0.00923

G1 X79.934 Y45.513 E0.01931

G1 X83.824 Y49.403 E0.14362

G1 X83.232 Y49.403 E0.01546

G1 X75.144 Y41.315 E0.29862

G1 X75.736 Y41.315 E0.01546

G1 X77.246 Y42.824 E0.05573

M204 S1250

; stop printing object tpu print.STL id:9 copy 0

; printing object Petg print.STL id:8 copy 0

; stop printing object Petg print.STL id:8 copy 0

; printing object Petg print.STL id:4 copy 0

; stop printing object Petg print.STL id:4 copy 0

; printing object tpu print.STL id:29 copy 0

G1 E-2.24000 F2400.000

```
;WIPE_START
G1 F7200.000
G1 X75.736 Y41.315 E-0.67595
G1 X75.144 Y41.315 E-0.18749
G1 X75.253 Y41.423 E-0.04857
;WIPE_END
G1 E-0.04800 F2400.000
G1 Z1.000 F9000.000
G1 X120.337 Y41.177
G1 Z0.800
G1 E3.20000 F1500.000
M204 S800
;TYPE:Perimeter
;WIDTH:0.45
G1 F2400.000
G1 X127.208 Y41.177 E0.17903
G1 X127.299 Y41.500 E0.00876
G1 X127.634 Y42.294 E0.02246
G1 X128.065 Y43.041 E0.02246
G1 X128.583 Y43.730 E0.02246
G1 X129.183 Y44.349 E0.02246
G1 X129.854 Y44.890 E0.02246
G1 X130.587 Y45.344 E0.02246
G1 X131.370 Y45.705 E0.02246
G1 X132.191 Y45.966 E0.02246
G1 X133.038 Y46.125 E0.02246
G1 X133.879 Y46.177 E0.02194
G1 X141.051 Y46.177 E0.18688
G1 X141.051 Y49.891 E0.09678
```

```
G1 X133.879 Y49.891 E0.18688
G1 X133.038 Y49.943 E0.02194
G1 X132.191 Y50.101 E0.02246
G1 X131.370 Y50.363 E0.02246
G1 X130.587 Y50.724 E0.02246
G1 X129.854 Y51.178 E0.02246
G1 X129.183 Y51.719 E0.02246
G1 X128.583 Y52.338 E0.02246
G1 X128.065 Y53.026 E0.02246
G1 X127.634 Y53.773 E0.02246
G1 X127.299 Y54.567 E0.02246
G1 X127.208 Y54.891 E0.00876
G1 X120.337 Y54.891 E0.17903
G1 X120.337 Y41.237 E0.35578
M204 S1250
G1 X119.919 Y40.759 F9000.000
M204 S800
;TYPE:External perimeter
G1 F1800.000
G1 X127.524 Y40.759 E0.19815
G1 X127.694 Y41.361 E0.01632
G1 X128.009 Y42.108 E0.02112
G1 X128.414 Y42.810 E0.02112
G1 X128.902 Y43.458 E0.02112
G1 X129.465 Y44.040 E0.02112
G1 X130.096 Y44.548 E0.02112
G1 X130.785 Y44.975 E0.02112
G1 X131.521 Y45.314 E0.02112
G1 X132.293 Y45.560 E0.02112
```

```
G1 X133.090 Y45.709 E0.02112
G1 X133.892 Y45.759 E0.02093
G1 X141.469 Y45.759 E0.19743
G1 X141.469 Y50.309 E0.11855
G1 X133.892 Y50.309 E0.19743
G1 X133.090 Y50.358 E0.02093
G1 X132.293 Y50.507 E0.02112
G1 X131.521 Y50.753 E0.02112
G1 X130.785 Y51.092 E0.02112
G1 X130.096 Y51.519 E0.02112
G1 X129.465 Y52.028 E0.02112
G1 X128.902 Y52.610 E0.02112
G1 X128.414 Y53.257 E0.02112
G1 X128.009 Y53.959 E0.02112
G1 X127.694 Y54.706 E0.02112
G1 X127.524 Y55.309 E0.01632
G1 X119.919 Y55.309 E0.19815
G1 X119.919 Y40.819 E0.37755
M204 S1250
G1 X120.293 Y40.901 F9000.000
G1 E-2.24000 F2400.000
;WIPE_START
G1 F7200.000
G1 X122.799 Y40.796 E-0.91200
;WIPE_END
G1 E-0.04800 F2400.000
G1 Z1.000 F9000.000
G1 X127.896 Y42.999
G1 Z0.800
```

```
G1 E3.20000 F1500.000
M204 S1000
;TYPE:Solid infill
;WIDTH:0.450839
G1 F4800.000
G1 X126.387 Y41.490 E0.05573
G1 X125.794 Y41.490 E0.01546
G1 X133.882 Y49.578 E0.29862
G1 X134.474 Y49.578 E0.01546
G1 X130.584 Y45.688 E0.14362
G1 X131.256 Y45.997 E0.01931
G1 X131.593 Y46.105 E0.00923
G1 X135.066 Y49.578 E0.12823
G1 X135.658 Y49.578 E0.01546
G1 X132.406 Y46.325 E0.12009
G1 X133.116 Y46.443 E0.01880
G1 X136.250 Y49.578 E0.11572
G1 X136.842 Y49.578 E0.01546
G1 X133.747 Y46.482 E0.11428
G1 X134.347 Y46.490 E0.01566
G1 X137.434 Y49.578 E0.11400
G1 X138.027 Y49.578 E0.01546
G1 X134.939 Y46.490 E0.11400
G1 X135.531 Y46.490 E0.01546
G1 X138.619 Y49.578 E0.11400
G1 X139.211 Y49.578 E0.01546
G1 X136.123 Y46.490 E0.11400
G1 X136.715 Y46.490 E0.01546
G1 X139.803 Y49.578 E0.11400
```

```
G1 X140.395 Y49.578 E0.01546
G1 X137.307 Y46.490 E0.11400
G1 X137.899 Y46.490 E0.01546
G1 X140.738 Y49.329 E0.10481
G1 X140.738 Y48.736 E0.01546
G1 X138.491 Y46.490 E0.08295
G1 X139.083 Y46.490 E0.01546
G1 X140.738 Y48.144 E0.06109
G1 X140.738 Y47.552 E0.01546
G1 X139.675 Y46.490 E0.03923
G1 X140.267 Y46.490 E0.01546
G1 X140.926 Y47.148 E0.02431
M204 S1250
G1 E-2.24000 F2400.000
;WIPE_START
G1 F7200.000
G1 X140.267 Y46.490 E-0.29484
G1 X139.675 Y46.490 E-0.18749
G1 X140.635 Y47.449 E-0.42968
;WIPE_END
G1 E-0.04800 F2400.000
G1 Z1.000 F9000.000
G1 X133.501 Y49.789
G1 Z0.800
G1 E3.20000 F1500.000
M204 S1000
G1 F4800.000
G1 X125.202 Y41.490 E0.30641
G1 X124.610 Y41.490 E0.01546
```

```
G1 X132.791 Y49.670 E0.30204
G1 X132.292 Y49.764 E0.01325
G1 X124.018 Y41.490 E0.30549
G1 X123.426 Y41.490 E0.01546
G1 X131.825 Y49.889 E0.31012
G1 X131.376 Y50.032 E0.01230
G1 X122.834 Y41.490 E0.31540
G1 X122.242 Y41.490 E0.01546
G1 X130.959 Y50.207 E0.32186
G1 X130.554 Y50.394 E0.01165
G1 X121.650 Y41.490 E0.32875
G1 X121.058 Y41.490 E0.01546
G1 X130.177 Y50.609 E0.33670
G1 X129.812 Y50.836 E0.01123
G1 X120.650 Y41.674 E0.33826
G1 X120.650 Y42.266 E0.01546
G1 X129.469 Y51.085 E0.32563
G1 X129.142 Y51.350 E0.01099
G1 X120.650 Y42.858 E0.31353
G1 X120.650 Y43.450 E0.01546
G1 X128.831 Y51.631 E0.30207
G1 X128.540 Y51.932 E0.01093
G1 X120.650 Y44.042 E0.29132
G1 X120.650 Y44.634 E0.01546
G1 X128.261 Y52.245 E0.28101
G1 X128.006 Y52.583 E0.01104
G1 X120.650 Y45.226 E0.27162
G1 X120.650 Y45.819 E0.01546
G1 X127.760 Y52.928 E0.26250
```

```
G1 X127.543 Y53.304 E0.01132
G1 X120.650 Y46.411 E0.25451
G1 X120.650 Y47.003 E0.01546
G1 X127.332 Y53.684 E0.24671
G1 X127.156 Y54.101 E0.01180
G1 X120.650 Y47.595 E0.24022
G1 X120.650 Y48.187 E0.01546
G1 X126.986 Y54.523 E0.23395
G1 X126.971 Y54.578 E0.00148
G1 X126.449 Y54.578 E0.01363
G1 X120.650 Y48.779 E0.21411
G1 X120.650 Y49.371 E0.01546
G1 X125.857 Y54.578 E0.19225
G1 X125.265 Y54.578 E0.01546
G1 X120.650 Y49.963 E0.17039
G1 X120.650 Y50.555 E0.01546
G1 X124.673 Y54.578 E0.14853
G1 X124.081 Y54.578 E0.01546
G1 X120.650 Y51.147 E0.12666
G1 X120.650 Y51.739 E0.01546
G1 X123.489 Y54.578 E0.10480
G1 X122.897 Y54.578 E0.01546
G1 X120.650 Y52.331 E0.08294
G1 X120.650 Y52.923 E0.01546
G1 X122.304 Y54.578 E0.06108
G1 X121.712 Y54.578 E0.01546
G1 X120.650 Y53.515 E0.03922
G1 X120.650 Y54.107 E0.01546
G1 X121.308 Y54.766 E0.02431
```

```
M204 S1250
; stop printing object tpu print.STL id:29 copy 0
; printing object tpu print.STL id:25 copy 0
G1 E-2.24000 F2400.000
;WIPE_START
G1 F7200.000
G1 X120.650 Y54.107 E-0.29480
G1 X120.650 Y53.515 E-0.18749
G1 X121.610 Y54.475 E-0.42971
;WIPE_END
G1 E-0.04800 F2400.000
G1 Z1.000 F9000.000
G1 X120.344 Y61.126
G1 Z0.800
G1 E3.20000 F1500.000
M204 S800
;TYPE:Perimeter
;WIDTH:0.45
G1 F2400.000
G1 X127.215 Y61.126 E0.17903
G1 X127.307 Y61.449 E0.00876
G1 X127.642 Y62.243 E0.02246
G1 X128.072 Y62.990 E0.02246
G1 X128.591 Y63.679 E0.02246
G1 X129.191 Y64.298 E0.02246
G1 X129.862 Y64.839 E0.02246
G1 X130.594 Y65.293 E0.02246
G1 X131.377 Y65.654 E0.02246
G1 X132.199 Y65.915 E0.02246
```

```
G1 X133.046 Y66.074 E0.02246
G1 X133.886 Y66.126 E0.02194
G1 X141.059 Y66.126 E0.18688
G1 X141.059 Y69.840 E0.09678
G1 X133.886 Y69.840 E0.18688
G1 X133.046 Y69.892 E0.02194
G1 X132.199 Y70.050 E0.02246
G1 X131.377 Y70.312 E0.02246
G1 X130.594 Y70.673 E0.02246
G1 X129.862 Y71.127 E0.02246
G1 X129.191 Y71.668 E0.02246
G1 X128.591 Y72.287 E0.02246
G1 X128.072 Y72.975 E0.02246
G1 X127.642 Y73.722 E0.02246
G1 X127.307 Y74.516 E0.02246
G1 X127.215 Y74.840 E0.00876
G1 X120.344 Y74.840 E0.17903
M73 P83 R12
G1 X120.344 Y61.186 E0.35578
M204 S1250
G1 X119.926 Y60.708 F9000.000
M204 S800
;TYPE:External perimeter
G1 F1800.000
G1 X127.531 Y60.708 E0.19815
G1 X127.702 Y61.310 E0.01632
G1 X128.017 Y62.057 E0.02112
G1 X128.421 Y62.759 E0.02112
G1 X128.909 Y63.406 E0.02112
```

```
G1 X129.473 Y63.989 E0.02112
G1 X130.104 Y64.497 E0.02112
G1 X130.793 Y64.924 E0.02112
G1 X131.529 Y65.263 E0.02112
G1 X132.301 Y65.509 E0.02112
G1 X133.097 Y65.658 E0.02112
G1 X133.899 Y65.708 E0.02093
G1 X141.476 Y65.708 E0.19743
G1 X141.476 Y70.258 E0.11855
G1 X133.899 Y70.258 E0.19743
G1 X133.097 Y70.307 E0.02093
G1 X132.301 Y70.456 E0.02112
G1 X131.529 Y70.702 E0.02112
G1 X130.793 Y71.041 E0.02112
G1 X130.104 Y71.468 E0.02112
G1 X129.473 Y71.977 E0.02112
G1 X128.909 Y72.559 E0.02112
G1 X128.421 Y73.206 E0.02112
G1 X128.017 Y73.908 E0.02112
G1 X127.702 Y74.655 E0.02112
G1 X127.531 Y75.258 E0.01632
G1 X119.926 Y75.258 E0.19815
G1 X119.926 Y60.768 E0.37755
M204 S1250
G1 X120.300 Y60.850 F9000.000
G1 E-2.24000 F2400.000
;WIPE_START
G1 F7200.000
G1 X122.806 Y60.745 E-0.91200
```

```
;WIPE_END
G1 E-0.04800 F2400.000
G1 Z1.000 F9000.000
G1 X127.903 Y62.948
G1 Z0.800
G1 E3.20000 F1500.000
M204 S1000
;TYPE:Solid infill
;WIDTH:0.450839
G1 F4800.000
G1 X126.394 Y61.439 E0.05573
G1 X125.802 Y61.439 E0.01546
G1 X133.890 Y69.527 E0.29862
G1 X134.482 Y69.527 E0.01546
G1 X130.592 Y65.637 E0.14362
G1 X131.264 Y65.946 E0.01931
G1 X131.601 Y66.054 E0.00923
G1 X135.074 Y69.527 E0.12823
G1 X135.666 Y69.527 E0.01546
G1 X132.413 Y66.274 E0.12009
G1 X133.124 Y66.392 E0.01880
G1 X136.258 Y69.527 E0.11572
G1 X136.850 Y69.527 E0.01546
G1 X133.755 Y66.431 E0.11428
G1 X134.354 Y66.439 E0.01566
G1 X137.442 Y69.527 E0.11400
G1 X138.034 Y69.527 E0.01546
G1 X134.946 Y66.439 E0.11400
G1 X135.538 Y66.439 E0.01546
```

```
G1 X138.626 Y69.527 E0.11400
G1 X139.218 Y69.527 E0.01546
G1 X136.130 Y66.439 E0.11400
G1 X136.723 Y66.439 E0.01546
G1 X139.810 Y69.527 E0.11400
G1 X140.402 Y69.527 E0.01546
G1 X137.315 Y66.439 E0.11400
G1 X137.907 Y66.439 E0.01546
G1 X140.745 Y69.277 E0.10481
G1 X140.745 Y68.685 E0.01546
G1 X138.499 Y66.439 E0.08295
G1 X139.091 Y66.439 E0.01546
G1 X140.745 Y68.093 E0.06109
G1 X140.745 Y67.501 E0.01546
G1 X139.683 Y66.439 E0.03923
G1 X140.275 Y66.439 E0.01546
G1 X140.933 Y67.097 E0.02431
M204 S1250
G1 E-2.24000 F2400.000
;WIPE_START
M73 P83 R11
G1 F7200.000
G1 X140.275 Y66.439 E-0.29484
G1 X139.683 Y66.439 E-0.18749
G1 X140.642 Y67.398 E-0.42968
;WIPE_END
G1 E-0.04800 F2400.000
G1 Z1.000 F9000.000
G1 X133.509 Y69.738
```

```
G1 Z0.800
G1 E3.20000 F1500.000
M204 S1000
G1 F4800.000
G1 X125.210 Y61.439 E0.30641
G1 X124.618 Y61.439 E0.01546
G1 X132.798 Y69.619 E0.30204
G1 X132.300 Y69.713 E0.01325
G1 X124.026 Y61.439 E0.30549
G1 X123.434 Y61.439 E0.01546
G1 X131.833 Y69.838 E0.31012
G1 X131.384 Y69.981 E0.01230
G1 X122.842 Y61.439 E0.31540
G1 X122.250 Y61.439 E0.01546
G1 X130.967 Y70.156 E0.32186
G1 X130.561 Y70.343 E0.01165
G1 X121.658 Y61.439 E0.32875
G1 X121.065 Y61.439 E0.01546
G1 X130.185 Y70.558 E0.33670
G1 X129.819 Y70.785 E0.01123
G1 X120.658 Y61.623 E0.33826
G1 X120.658 Y62.215 E0.01546
G1 X129.477 Y71.034 E0.32563
G1 X129.149 Y71.299 E0.01099
G1 X120.658 Y62.807 E0.31353
G1 X120.658 Y63.399 E0.01546
G1 X128.839 Y71.580 E0.30207
G1 X128.548 Y71.881 E0.01093
G1 X120.658 Y63.991 E0.29132
```

```
G1 X120.658 Y64.583 E0.01546
G1 X128.268 Y72.194 E0.28101
G1 X128.014 Y72.532 E0.01104
G1 X120.658 Y65.175 E0.27162
G1 X120.658 Y65.767 E0.01546
G1 X127.767 Y72.877 E0.26250
G1 X127.551 Y73.253 E0.01132
G1 X120.658 Y66.360 E0.25451
G1 X120.658 Y66.952 E0.01546
G1 X127.339 Y73.633 E0.24671
G1 X127.164 Y74.050 E0.01180
G1 X120.658 Y67.544 E0.24022
G1 X120.658 Y68.136 E0.01546
G1 X126.994 Y74.472 E0.23395
G1 X126.978 Y74.527 E0.00148
G1 X126.456 Y74.527 E0.01363
G1 X120.658 Y68.728 E0.21411
G1 X120.658 Y69.320 E0.01546
G1 X125.864 Y74.527 E0.19225
G1 X125.272 Y74.527 E0.01546
G1 X120.658 Y69.912 E0.17039
G1 X120.658 Y70.504 E0.01546
G1 X124.680 Y74.527 E0.14853
G1 X124.088 Y74.527 E0.01546
G1 X120.658 Y71.096 E0.12666
G1 X120.658 Y71.688 E0.01546
G1 X123.496 Y74.527 E0.10480
G1 X122.904 Y74.527 E0.01546
G1 X120.658 Y72.280 E0.08294
```

G1 X120.658 Y72.872 E0.01546

G1 X122.312 Y74.527 E0.06108

G1 X121.720 Y74.527 E0.01546

G1 X120.658 Y73.464 E0.03922

G1 X120.658 Y74.056 E0.01546

G1 X121.316 Y74.715 E0.02431

M204 S1250

; stop printing object tpu print.STL id:25 copy 0

; printing object Petg print.STL id:24 copy 0

; stop printing object Petg print.STL id:24 copy 0

; printing object Petg print.STL id:28 copy 0

; stop printing object Petg print.STL id:28 copy 0

;LAYER_CHANGE

;Z:0.95

;HEIGHT:0.15

;BEFORE_LAYER_CHANGE

G92 E0.0

;0.95

G1 E-2.24000 F2400.000

;WIPE_START

G1 F7200.000

G1 X120.658 Y74.056 E-0.29480

G1 X120.658 Y73.464 E-0.18749

G1 X121.617 Y74.424 E-0.42971

;WIPE_END

G1 E-0.04800 F2400.000

G1 Z1.000 F9000.000

;AFTER_LAYER_CHANGE

;0.95

; printing object tpu print.STL id:19 copy 0

G1 X41.611 Y49.135

G1 Z0.950

G1 E3.20000 F1500.000

M204 S800

;TYPE:Perimeter

;WIDTH:0.45

G1 F2400.000

G1 X34.439 Y49.135 E0.18688

G1 X33.599 Y49.187 E0.02194

G1 X32.752 Y49.345 E0.02246

G1 X31.930 Y49.607 E0.02246

G1 X31.147 Y49.968 E0.02246

G1 X30.415 Y50.422 E0.02246

G1 X29.743 Y50.963 E0.02246

G1 X29.144 Y51.582 E0.02246

G1 X28.625 Y52.270 E0.02246

G1 X28.195 Y53.017 E0.02246

G1 X27.860 Y53.812 E0.02246

G1 X27.768 Y54.135 E0.00876

G1 X20.897 Y54.135 E0.17903

G1 X20.897 Y40.421 E0.35734

G1 X27.768 Y40.421 E0.17903

G1 X27.832 Y40.646 E0.00610

G1 X28.193 Y41.535 E0.02499

G1 X28.625 Y42.285 E0.02257

G1 X29.144 Y42.974 E0.02246

```
G1 X29.743 Y43.593 E0.02246
G1 X30.415 Y44.134 E0.02246
G1 X31.147 Y44.588 E0.02246
G1 X31.930 Y44.949 E0.02246
G1 X32.752 Y45.210 E0.02246
G1 X33.599 Y45.369 E0.02246
G1 X34.439 Y45.421 E0.02194
G1 X41.611 Y45.421 E0.18688
G1 X41.611 Y49.075 E0.09522
M204 S1250
G1 X42.029 Y49.553 F9000.000
M204 S800
;TYPE:External perimeter
G1 F1800.000
G1 X34.452 Y49.553 E0.19743
G1 X33.650 Y49.602 E0.02093
G1 X32.854 Y49.751 E0.02112
G1 X32.082 Y49.997 E0.02112
G1 X31.345 Y50.336 E0.02112
G1 X30.657 Y50.763 E0.02112
G1 X30.026 Y51.272 E0.02112
G1 X29.462 Y51.854 E0.02112
G1 X28.974 Y52.501 E0.02112
G1 X28.570 Y53.203 E0.02112
G1 X28.255 Y53.950 E0.02112
G1 X28.084 Y54.553 E0.01632
G1 X20.479 Y54.553 E0.19815
G1 X20.479 Y40.003 E0.37911
G1 X28.084 Y40.003 E0.19815
```

```
G1 X28.228 Y40.510 E0.01373
G1 X28.569 Y41.351 E0.02365
G1 X28.974 Y42.054 E0.02116
G1 X29.462 Y42.702 E0.02112
G1 X30.026 Y43.284 E0.02112
G1 X30.657 Y43.792 E0.02112
G1 X31.345 Y44.219 E0.02112
G1 X32.082 Y44.558 E0.02112
G1 X32.854 Y44.804 E0.02112
G1 X33.650 Y44.953 E0.02112
G1 X34.452 Y45.003 E0.02093
G1 X42.029 Y45.003 E0.19743
G1 X42.029 Y49.493 E0.11699
M204 S1250
G1 X41.636 Y49.481 F9000.000
G1 E-2.24000 F2400.000
;WIPE_START
G1 F7200.000
G1 X39.149 Y49.516 E-0.91200
;WIPE_END
G1 E-0.04800 F2400.000
G1 Z1.150 F9000.000
G1 X41.489 Y48.258
G1 Z0.950
G1 E3.20000 F1500.000
M204 S1000
;TYPE:Top solid infill
;WIDTH:0.405169
G1 F2400.000
```

G1 X40.900 Y48.847 E0.01937

G1 X40.372 Y48.847 E0.01227

G1 X41.323 Y47.896 E0.03128

G1 X41.323 Y47.368 E0.01227

G1 X39.845 Y48.847 E0.04863

G1 X39.317 Y48.847 E0.01227

G1 X41.323 Y46.841 E0.06598

G1 X41.323 Y46.313 E0.01227

G1 X38.790 Y48.847 E0.08333

G1 X38.262 Y48.847 E0.01227

G1 X41.323 Y45.786 E0.10068

G1 X41.323 Y45.709 E0.00179

G1 X40.873 Y45.709 E0.01048

G1 X37.735 Y48.847 E0.10321

G1 X37.207 Y48.847 E0.01227

G1 X40.345 Y45.709 E0.10321

G1 X39.818 Y45.709 E0.01227

G1 X36.680 Y48.847 E0.10321

G1 X36.152 Y48.847 E0.01227

G1 X39.290 Y45.709 E0.10321

G1 X38.763 Y45.709 E0.01227

G1 X35.625 Y48.847 E0.10321

G1 X35.097 Y48.847 E0.01227

G1 X38.235 Y45.709 E0.10321

G1 X37.708 Y45.709 E0.01227

G1 X34.570 Y48.847 E0.10321

G1 X34.017 Y48.872 E0.01288

G1 X37.180 Y45.709 E0.10405

G1 X36.653 Y45.709 E0.01227

```
G1 X33.438 Y48.924 E0.10574
G1 X32.789 Y49.045 E0.01536
G1 X36.125 Y45.709 E0.10974
G1 X35.598 Y45.709 E0.01227
G1 X31.754 Y49.553 E0.12645
M204 S1250
G1 E-2.24000
;WIPE_START
G1 F7200.000
G1 X33.790 Y47.517 E-0.91200
;WIPE_END
G1 E-0.04800 F2400.000
G1 Z1.150 F9000.000
G1 X27.895 Y53.412
G1 Z0.950
G1 E3.20000 F1500.000
M204 S1000
G1 F2400.000
G1 X27.460 Y53.847 E0.01431
G1 X26.933 Y53.847 E0.01227
G1 X27.998 Y52.781 E0.03505
G1 X28.384 Y52.111 E0.01799
G1 X28.924 Y51.394 E0.02088
G1 X29.549 Y50.749 E0.02088
G1 X30.248 Y50.186 E0.02088
G1 X31.114 Y49.666 E0.02350
G1 X35.070 Y45.709 E0.13015
G1 X34.543 Y45.709 E0.01227
G1 X26.405 Y53.847 E0.26769
```

G1 X25.878 Y53.847 E0.01227

G1 X34.040 Y45.685 E0.26848

G1 X33.545 Y45.652 E0.01153

G1 X25.350 Y53.847 E0.26956

G1 X24.823 Y53.847 E0.01227

G1 X33.101 Y45.569 E0.27230

G1 X32.659 Y45.483 E0.01047

G1 X24.295 Y53.847 E0.27511

G1 X23.768 Y53.847 E0.01227

G1 X32.259 Y45.356 E0.27930

G1 X31.859 Y45.228 E0.00977

G1 X23.240 Y53.847 E0.28349

G1 X22.713 Y53.847 E0.01227

G1 X31.494 Y45.065 E0.28886

G1 X31.133 Y44.899 E0.00925

G1 X22.185 Y53.847 E0.29433

G1 X21.658 Y53.847 E0.01227

G1 X30.795 Y44.709 E0.30057

G1 X30.470 Y44.507 E0.00891

G1 X21.185 Y53.792 E0.30541

G1 X21.185 Y53.264 E0.01227

G1 X30.155 Y44.295 E0.29504

G1 X29.863 Y44.059 E0.00873

G1 X21.185 Y52.737 E0.28544

G1 X21.185 Y52.209 E0.01227

G1 X29.571 Y43.824 E0.27583

G1 X29.309 Y43.559 E0.00868

G1 X21.185 Y51.682 E0.26721

G1 X21.185 Y51.154 E0.01227

G1 X29.049 Y43.290 E0.25868

G1 X28.807 Y43.005 E0.00870

G1 X21.185 Y50.627 E0.25070

G1 X21.185 Y50.099 E0.01227

G1 X28.580 Y42.705 E0.24324

G1 X28.358 Y42.399 E0.00878

G1 X21.185 Y49.572 E0.23594

G1 X21.185 Y49.044 E0.01227

G1 X28.165 Y42.065 E0.22960

G1 X27.972 Y41.730 E0.00898

G1 X21.185 Y48.517 E0.22326

G1 X21.185 Y47.989 E0.01227

G1 X27.812 Y41.363 E0.21797

G1 X27.660 Y40.988 E0.00942

G1 X21.185 Y47.462 E0.21296

G1 X21.185 Y46.935 E0.01227

G1 X27.411 Y40.709 E0.20479

G1 X26.884 Y40.709 E0.01227

G1 X21.185 Y46.407 E0.18744

G1 X21.185 Y45.880 E0.01227

G1 X26.356 Y40.709 E0.17009

G1 X25.829 Y40.709 E0.01227

G1 X21.185 Y45.352 E0.15273

G1 X21.185 Y44.825 E0.01227

G1 X25.301 Y40.709 E0.13538

G1 X24.774 Y40.709 E0.01227

G1 X21.185 Y44.297 E0.11803

G1 X21.185 Y43.770 E0.01227

G1 X24.246 Y40.709 E0.10068

G1 X23.719 Y40.709 E0.01227

G1 X21.185 Y43.242 E0.08333

G1 X21.185 Y42.715 E0.01227

G1 X23.191 Y40.709 E0.06598

G1 X22.664 Y40.709 E0.01227

G1 X21.185 Y42.187 E0.04863

G1 X21.185 Y41.660 E0.01227

G1 X22.136 Y40.709 E0.03128

G1 X21.609 Y40.709 E0.01227

G1 X21.020 Y41.298 E0.01937

M204 S1250

; stop printing object tpu print.STL id:19 copy 0

; printing object Petg print.STL id:18 copy 0

; stop printing object Petg print.STL id:18 copy 0

; printing object Petg print.STL id:14 copy 0

; stop printing object Petg print.STL id:14 copy 0

; printing object tpu print.STL id:15 copy 0

G1 E-2.24000

;WIPE_START

G1 F7200.000

G1 X21.609 Y40.709 E-0.26372

G1 X22.136 Y40.709 E-0.16703

G1 X21.185 Y41.660 E-0.42582

G1 X21.185 Y41.835 E-0.05543

;WIPE_END

G1 E-0.04800 F2400.000

G1 Z1.150 F9000.000

G1 X21.020 Y60.370

G1 Z0.950

```
G1 E3.20000 F1500.000
M204 S800
;TYPE:Perimeter
;WIDTH:0.45
G1 F2400.000
G1 X27.776 Y60.370 E0.17603
G1 X27.839 Y60.595 E0.00610
G1 X28.200 Y61.483 E0.02499
G1 X28.633 Y62.234 E0.02257
G1 X29.151 Y62.923 E0.02246
G1 X29.751 Y63.542 E0.02246
G1 X30.422 Y64.083 E0.02246
G1 X31.155 Y64.537 E0.02246
G1 X31.938 Y64.898 E0.02246
G1 X32.759 Y65.159 E0.02246
G1 X33.606 Y65.318 E0.02246
G1 X34.447 Y65.370 E0.02194
G1 X41.619 Y65.370 E0.18688
G1 X41.619 Y69.084 E0.09678
G1 X34.447 Y69.084 E0.18688
G1 X33.606 Y69.136 E0.02194
G1 X32.759 Y69.294 E0.02246
G1 X31.938 Y69.556 E0.02246
G1 X31.155 Y69.917 E0.02246
G1 X30.422 Y70.371 E0.02246
G1 X29.751 Y70.912 E0.02246
G1 X29.151 Y71.531 E0.02246
G1 X28.633 Y72.219 E0.02246
G1 X28.202 Y72.966 E0.02246
```

```
G1 X27.867 Y73.760 E0.02246
G1 X27.776 Y74.084 E0.00876
G1 X20.905 Y74.084 E0.17903
G1 X20.905 Y60.370 E0.35734
G1 X20.960 Y60.370 E0.00144
M204 S1250
G1 X20.487 Y59.952 F9000.000
M204 S800
;TYPE:External perimeter
G1 F1800.000
G1 X28.092 Y59.952 E0.19815
G1 X28.235 Y60.459 E0.01373
G1 X28.576 Y61.300 E0.02365
G1 X28.982 Y62.003 E0.02116
G1 X29.469 Y62.651 E0.02112
G1 X30.033 Y63.233 E0.02112
G1 X30.664 Y63.741 E0.02112
G1 X31.353 Y64.168 E0.02112
G1 X32.089 Y64.507 E0.02112
G1 X32.861 Y64.753 E0.02112
G1 X33.658 Y64.902 E0.02112
G1 X34.460 Y64.952 E0.02093
G1 X42.037 Y64.952 E0.19743
G1 X42.037 Y69.502 E0.11855
G1 X34.460 Y69.502 E0.19743
G1 X33.658 Y69.551 E0.02093
G1 X32.861 Y69.700 E0.02112
G1 X32.089 Y69.946 E0.02112
G1 X31.353 Y70.285 E0.02112
```

```
G1 X30.664 Y70.712 E0.02112
G1 X30.033 Y71.221 E0.02112
G1 X29.469 Y71.803 E0.02112
G1 X28.982 Y72.450 E0.02112
G1 X28.577 Y73.152 E0.02112
G1 X28.262 Y73.899 E0.02112
G1 X28.092 Y74.502 E0.01632
G1 X20.487 Y74.502 E0.19815
G1 X20.487 Y60.012 E0.37755
M204 S1250
G1 X20.861 Y60.094 F9000.000
G1 E-2.24000 F2400.000
;WIPE_START
G1 F7200.000
G1 X23.367 Y59.989 E-0.91200
;WIPE_END
G1 E-0.04800 F2400.000
G1 Z1.150 F9000.000
G1 X41.496 Y68.207
G1 Z0.950
G1 E3.20000 F1500.000
M204 S1000
;TYPE:Top solid infill
;WIDTH:0.405169
G1 F2400.000
G1 X40.907 Y68.796 E0.01937
G1 X40.380 Y68.796 E0.01227
G1 X41.331 Y67.845 E0.03128
G1 X41.331 Y67.317 E0.01227
```

```
G1 X39.852 Y68.796 E0.04863
G1 X39.325 Y68.796 E0.01227
G1 X41.331 Y66.790 E0.06598
G1 X41.331 Y66.262 E0.01227
G1 X38.797 Y68.796 E0.08333
G1 X38.270 Y68.796 E0.01227
G1 X41.331 Y65.735 E0.10068
G1 X41.331 Y65.658 E0.00179
G1 X40.880 Y65.658 E0.01048
G1 X37.742 Y68.796 E0.10321
G1 X37.215 Y68.796 E0.01227
G1 X40.353 Y65.658 E0.10321
G1 X39.825 Y65.658 E0.01227
G1 X36.687 Y68.796 E0.10321
G1 X36.160 Y68.796 E0.01227
G1 X39.298 Y65.658 E0.10321
G1 X38.770 Y65.658 E0.01227
G1 X35.632 Y68.796 E0.10321
G1 X35.105 Y68.796 E0.01227
G1 X38.243 Y65.658 E0.10321
G1 X37.715 Y65.658 E0.01227
G1 X34.577 Y68.796 E0.10321
G1 X34.024 Y68.821 E0.01288
G1 X37.188 Y65.658 E0.10405
G1 X36.660 Y65.658 E0.01227
G1 X33.446 Y68.873 E0.10574
G1 X32.797 Y68.994 E0.01536
G1 X36.133 Y65.658 E0.10974
G1 X35.605 Y65.658 E0.01227
```

```
G1 X31.761 Y69.502 E0.12645
M204 S1250
G1 E-2.24000
;WIPE_START
G1 F7200.000
G1 X33.798 Y67.466 E-0.91200
;WIPE_END
G1 E-0.04800 F2400.000
G1 Z1.150 F9000.000
G1 X27.903 Y73.361
G1 Z0.950
G1 E3.20000 F1500.000
M204 S1000
G1 F2400.000
G1 X27.468 Y73.796 E0.01431
G1 X26.940 Y73.796 E0.01227
G1 X28.006 Y72.730 E0.03505
G1 X28.392 Y72.060 E0.01799
G1 X28.932 Y71.343 E0.02088
G1 X29.556 Y70.698 E0.02088
G1 X30.255 Y70.135 E0.02088
G1 X31.121 Y69.615 E0.02350
G1 X35.078 Y65.658 E0.13015
G1 X34.550 Y65.658 E0.01227
G1 X26.413 Y73.796 E0.26769
G1 X25.885 Y73.796 E0.01227
G1 X34.047 Y65.634 E0.26848
G1 X33.552 Y65.601 E0.01153
M73 P84 R11
```

G1 X25.358 Y73.796 E0.26956

G1 X24.830 Y73.796 E0.01227

G1 X33.108 Y65.518 E0.27230

G1 X32.666 Y65.432 E0.01047

G1 X24.303 Y73.796 E0.27511

G1 X23.775 Y73.796 E0.01227

G1 X32.266 Y65.305 E0.27930

G1 X31.866 Y65.177 E0.00977

G1 X23.248 Y73.796 E0.28349

G1 X22.720 Y73.796 E0.01227

G1 X31.502 Y65.014 E0.28886

G1 X31.141 Y64.848 E0.00925

G1 X22.193 Y73.796 E0.29433

G1 X21.665 Y73.796 E0.01227

G1 X30.803 Y64.658 E0.30057

G1 X30.477 Y64.456 E0.00891

G1 X21.193 Y73.741 E0.30541

G1 X21.193 Y73.213 E0.01227

G1 X30.162 Y64.244 E0.29504

G1 X29.870 Y64.008 E0.00873

G1 X21.193 Y72.686 E0.28544

G1 X21.193 Y72.158 E0.01227

G1 X29.578 Y63.773 E0.27583

G1 X29.316 Y63.507 E0.00868

G1 X21.193 Y71.631 E0.26721

G1 X21.193 Y71.103 E0.01227

G1 X29.057 Y63.239 E0.25868

G1 X28.814 Y62.954 E0.00870

G1 X21.193 Y70.576 E0.25070

```
G1 X21.193 Y70.048 E0.01227
G1 X28.588 Y62.654 E0.24324
G1 X28.365 Y62.348 E0.00878
G1 X21.193 Y69.521 E0.23594
G1 X21.193 Y68.993 E0.01227
G1 X28.173 Y62.014 E0.22960
G1 X27.980 Y61.679 E0.00898
G1 X21.193 Y68.466 E0.22326
G1 X21.193 Y67.938 E0.01227
G1 X27.819 Y61.312 E0.21797
G1 X27.667 Y60.937 E0.00942
G1 X21.193 Y67.411 E0.21296
G1 X21.193 Y66.883 E0.01227
G1 X27.418 Y60.658 E0.20479
G1 X26.891 Y60.658 E0.01227
G1 X21.193 Y66.356 E0.18744
G1 X21.193 Y65.829 E0.01227
G1 X26.364 Y60.658 E0.17009
G1 X25.836 Y60.658 E0.01227
G1 X21.193 Y65.301 E0.15273
G1 X21.193 Y64.774 E0.01227
G1 X25.309 Y60.658 E0.13538
G1 X24.781 Y60.658 E0.01227
G1 X21.193 Y64.246 E0.11803
G1 X21.193 Y63.719 E0.01227
G1 X24.254 Y60.658 E0.10068
G1 X23.726 Y60.658 E0.01227
G1 X21.193 Y63.191 E0.08333
G1 X21.193 Y62.664 E0.01227
```

```
G1 X23.199 Y60.658 E0.06598
G1 X22.671 Y60.658 E0.01227
G1 X21.193 Y62.136 E0.04863
G1 X21.193 Y61.609 E0.01227
G1 X22.144 Y60.658 E0.03128
G1 X21.616 Y60.658 E0.01227
G1 X21.027 Y61.247 E0.01937
M204 S1250
; stop printing object tpu print.STL id:15 copy 0
; printing object Petg print.STL id:10 copy 0
; stop printing object Petg print.STL id:10 copy 0
; printing object tpu print.STL id:11 copy 0
G1 E-2.24000
;WIPE_START
G1 F7200.000
G1 X21.616 Y60.658 E-0.26372
G1 X22.144 Y60.658 E-0.16703
G1 X21.193 Y61.609 E-0.42582
G1 X21.193 Y61.784 E-0.05543
;WIPE_END
G1 E-0.04800 F2400.000
G1 Z1.150 F9000.000
G1 X21.027 Y82.562
G1 Z0.950
G1 E3.20000 F1500.000
M204 S800
;TYPE:Perimeter
;WIDTH:0.45
G1 F2400.000
```

```
G1 X27.725 Y82.562 E0.17450
G1 X27.788 Y82.787 E0.00610
G1 X28.149 Y83.676 E0.02499
G1 X28.581 Y84.427 E0.02257
G1 X29.100 Y85.115 E0.02246
G1 X29.700 Y85.734 E0.02246
G1 X30.371 Y86.275 E0.02246
G1 X31.104 Y86.729 E0.02246
G1 X31.887 Y87.090 E0.02246
G1 X32.708 Y87.351 E0.02246
G1 X33.555 Y87.510 E0.02246
G1 X34.396 Y87.562 E0.02194
G1 X41.568 Y87.562 E0.18688
G1 X41.568 Y91.276 E0.09678
G1 X34.396 Y91.276 E0.18688
G1 X33.555 Y91.328 E0.02194
G1 X32.708 Y91.487 E0.02246
G1 X31.887 Y91.748 E0.02246
G1 X31.104 Y92.109 E0.02246
G1 X30.371 Y92.563 E0.02246
G1 X29.700 Y93.104 E0.02246
G1 X29.100 Y93.723 E0.02246
G1 X28.581 Y94.412 E0.02246
G1 X28.151 Y95.159 E0.02246
G1 X27.816 Y95.953 E0.02246
G1 X27.725 Y96.276 E0.00876
G1 X20.853 Y96.276 E0.17903
G1 X20.853 Y82.562 E0.35734
G1 X20.967 Y82.562 E0.00297
```

```
M204 S1250
G1 X20.436 Y82.144 F9000.000
M204 S800
;TYPE:External perimeter
G1 F1800.000
G1 X28.040 Y82.144 E0.19815
G1 X28.184 Y82.651 E0.01373
G1 X28.525 Y83.492 E0.02365
G1 X28.931 Y84.196 E0.02116
G1 X29.418 Y84.843 E0.02112
G1 X29.982 Y85.425 E0.02112
G1 X30.613 Y85.934 E0.02112
G1 X31.302 Y86.361 E0.02112
G1 X32.038 Y86.700 E0.02112
G1 X32.810 Y86.946 E0.02112
G1 X33.607 Y87.095 E0.02112
G1 X34.409 Y87.144 E0.02093
G1 X41.986 Y87.144 E0.19743
G1 X41.986 Y91.694 E0.11855
G1 X34.409 Y91.694 E0.19743
G1 X33.607 Y91.744 E0.02093
G1 X32.810 Y91.893 E0.02112
G1 X32.038 Y92.139 E0.02112
G1 X31.302 Y92.478 E0.02112
G1 X30.613 Y92.905 E0.02112
G1 X29.982 Y93.413 E0.02112
G1 X29.418 Y93.995 E0.02112
G1 X28.931 Y94.643 E0.02112
G1 X28.526 Y95.345 E0.02112
```

```
G1 X28.211 Y96.091 E0.02112
G1 X28.040 Y96.694 E0.01632
G1 X20.436 Y96.694 E0.19815
G1 X20.436 Y82.204 E0.37755
M204 S1250
G1 X20.810 Y82.286 F9000.000
G1 E-2.24000 F2400.000
;WIPE_START
G1 F7200.000
G1 X23.316 Y82.181 E-0.91200
;WIPE_END
G1 E-0.04800 F2400.000
G1 Z1.150 F9000.000
G1 X41.445 Y90.399
G1 Z0.950
G1 E3.20000 F1500.000
M204 S1000
;TYPE:Top solid infill
;WIDTH:0.405169
G1 F2400.000
G1 X40.856 Y90.988 E0.01937
G1 X40.329 Y90.988 E0.01227
G1 X41.279 Y90.037 E0.03128
G1 X41.279 Y89.510 E0.01227
G1 X39.801 Y90.988 E0.04863
G1 X39.274 Y90.988 E0.01227
G1 X41.279 Y88.982 E0.06598
G1 X41.279 Y88.455 E0.01227
G1 X38.746 Y90.988 E0.08333
```

```
G1 X38.219 Y90.988 E0.01227
G1 X41.279 Y87.927 E0.10068
G1 X41.279 Y87.850 E0.00179
G1 X40.829 Y87.850 E0.01048
G1 X37.691 Y90.988 E0.10321
G1 X37.164 Y90.988 E0.01227
G1 X40.301 Y87.850 E0.10321
G1 X39.774 Y87.850 E0.01227
G1 X36.636 Y90.988 E0.10321
G1 X36.109 Y90.988 E0.01227
G1 X39.246 Y87.850 E0.10321
G1 X38.719 Y87.850 E0.01227
G1 X35.581 Y90.988 E0.10321
G1 X35.054 Y90.988 E0.01227
G1 X38.192 Y87.850 E0.10321
G1 X37.664 Y87.850 E0.01227
G1 X34.526 Y90.988 E0.10321
G1 X33.973 Y91.013 E0.01288
G1 X37.137 Y87.850 E0.10405
G1 X36.609 Y87.850 E0.01227
G1 X33.394 Y91.065 E0.10574
G1 X32.746 Y91.186 E0.01536
G1 X36.082 Y87.850 E0.10974
G1 X35.554 Y87.850 E0.01227
G1 X31.710 Y91.694 E0.12645
M204 S1250
G1 E-2.24000
;WIPE_START
G1 F7200.000
```

```
G1 X33.747 Y89.658 E-0.91200
;WIPE_END
G1 E-0.04800 F2400.000
G1 Z1.150 F9000.000
G1 X27.852 Y95.553
G1 Z0.950
G1 E3.20000 F1500.000
M204 S1000
G1 F2400.000
G1 X27.416 Y95.988 E0.01431
G1 X26.889 Y95.988 E0.01227
G1 X27.954 Y94.923 E0.03505
G1 X28.340 Y94.252 E0.01799
G1 X28.881 Y93.536 E0.02088
G1 X29.505 Y92.891 E0.02088
G1 X30.204 Y92.327 E0.02088
G1 X31.070 Y91.807 E0.02350
G1 X35.027 Y87.850 E0.13015
G1 X34.499 Y87.850 E0.01227
G1 X26.362 Y95.988 E0.26769
G1 X25.834 Y95.988 E0.01227
G1 X33.996 Y87.826 E0.26848
G1 X33.501 Y87.793 E0.01153
G1 X25.307 Y95.988 E0.26956
G1 X24.779 Y95.988 E0.01227
G1 X33.057 Y87.710 E0.27230
G1 X32.615 Y87.625 E0.01047
G1 X24.252 Y95.988 E0.27511
G1 X23.724 Y95.988 E0.01227
```

```
G1 X32.215 Y87.497 E0.27930
G1 X31.815 Y87.370 E0.00977
G1 X23.197 Y95.988 E0.28349
G1 X22.669 Y95.988 E0.01227
G1 X31.451 Y87.207 E0.28886
G1 X31.089 Y87.040 E0.00925
G1 X22.142 Y95.988 E0.29433
G1 X21.614 Y95.988 E0.01227
G1 X30.752 Y86.850 E0.30057
G1 X30.426 Y86.649 E0.00891
G1 X21.142 Y95.933 E0.30541
G1 X21.142 Y95.406 E0.01227
G1 X30.111 Y86.436 E0.29504
G1 X29.819 Y86.201 E0.00873
G1 X21.142 Y94.878 E0.28544
G1 X21.142 Y94.351 E0.01227
G1 X29.527 Y85.965 E0.27583
G1 X29.265 Y85.700 E0.00868
G1 X21.142 Y93.823 E0.26721
G1 X21.142 Y93.296 E0.01227
G1 X29.006 Y85.432 E0.25868
G1 X28.763 Y85.147 E0.00870
G1 X21.142 Y92.768 E0.25070
G1 X21.142 Y92.241 E0.01227
G1 X28.536 Y84.846 E0.24324
G1 X28.314 Y84.541 E0.00878
G1 X21.142 Y91.713 E0.23594
G1 X21.142 Y91.186 E0.01227
G1 X28.122 Y84.206 E0.22960
```

```
G1 X27.929 Y83.871 E0.00898
G1 X21.142 Y90.658 E0.22326
G1 X21.142 Y90.131 E0.01227
G1 X27.768 Y83.504 E0.21797
M73 P84 R10
G1 X27.616 Y83.129 E0.00942
G1 X21.142 Y89.603 E0.21296
G1 X21.142 Y89.076 E0.01227
G1 X27.367 Y82.850 E0.20479
G1 X26.840 Y82.850 E0.01227
G1 X21.142 Y88.548 E0.18744
G1 X21.142 Y88.021 E0.01227
G1 X26.312 Y82.850 E0.17009
G1 X25.785 Y82.850 E0.01227
G1 X21.142 Y87.493 E0.15273
G1 X21.142 Y86.966 E0.01227
G1 X25.257 Y82.850 E0.13538
G1 X24.730 Y82.850 E0.01227
G1 X21.142 Y86.438 E0.11803
G1 X21.142 Y85.911 E0.01227
G1 X24.203 Y82.850 E0.10068
G1 X23.675 Y82.850 E0.01227
G1 X21.142 Y85.384 E0.08333
G1 X21.142 Y84.856 E0.01227
G1 X23.148 Y82.850 E0.06598
G1 X22.620 Y82.850 E0.01227
G1 X21.142 Y84.329 E0.04863
G1 X21.142 Y83.801 E0.01227
G1 X22.093 Y82.850 E0.03128
```

```
G1 X21.565 Y82.850 E0.01227
G1 X20.976 Y83.439 E0.01937
M204 S1250
; stop printing object tpu print.STL id:11 copy 0
; printing object tpu print.STL id:13 copy 0
G1 E-2.24000
;WIPE_START
G1 F7200.000
G1 X21.565 Y82.850 E-0.26372
G1 X22.093 Y82.850 E-0.16703
G1 X21.142 Y83.801 E-0.42582
G1 X21.142 Y83.976 E-0.05543
;WIPE_END
G1 E-0.04800 F2400.000
G1 Z1.150 F9000.000
G1 X20.976 Y103.377
G1 Z0.950
G1 E3.20000 F1500.000
M204 S800
;TYPE:Perimeter
;WIDTH:0.45
G1 F2400.000
G1 X27.731 Y103.377 E0.17601
G1 X27.795 Y103.603 E0.00610
G1 X28.156 Y104.491 E0.02499
G1 X28.588 Y105.242 E0.02257
G1 X29.107 Y105.930 E0.02246
G1 X29.707 Y106.550 E0.02246
G1 X30.378 Y107.091 E0.02246
```

```
G1 X31.110 Y107.545 E0.02246
G1 X31.893 Y107.905 E0.02246
G1 X32.715 Y108.167 E0.02246
G1 X33.562 Y108.326 E0.02246
G1 X34.402 Y108.377 E0.02194
G1 X41.575 Y108.377 E0.18688
G1 X41.575 Y112.092 E0.09678
G1 X34.402 Y112.092 E0.18688
G1 X33.562 Y112.144 E0.02194
G1 X32.715 Y112.302 E0.02246
G1 X31.893 Y112.564 E0.02246
G1 X31.110 Y112.924 E0.02246
G1 X30.378 Y113.379 E0.02246
G1 X29.707 Y113.920 E0.02246
G1 X29.107 Y114.539 E0.02246
G1 X28.588 Y115.227 E0.02246
G1 X28.158 Y115.974 E0.02246
G1 X27.823 Y116.768 E0.02246
G1 X27.731 Y117.092 E0.00876
G1 X20.860 Y117.092 E0.17903
G1 X20.860 Y103.377 E0.35734
G1 X20.916 Y103.377 E0.00146
M204 S1250
G1 X20.442 Y102.960 F9000.000
M204 S800
;TYPE:External perimeter
G1 F1800.000
G1 X28.047 Y102.960 E0.19815
G1 X28.191 Y103.467 E0.01373
```

```
G1 X28.532 Y104.308 E0.02365
G1 X28.937 Y105.011 E0.02116
G1 X29.425 Y105.658 E0.02112
G1 X29.989 Y106.241 E0.02112
G1 X30.620 Y106.749 E0.02112
G1 X31.309 Y107.176 E0.02112
G1 X32.045 Y107.515 E0.02112
G1 X32.817 Y107.761 E0.02112
G1 X33.613 Y107.910 E0.02112
G1 X34.415 Y107.960 E0.02093
G1 X41.992 Y107.960 E0.19743
G1 X41.992 Y112.510 E0.11855
G1 X34.415 Y112.510 E0.19743
G1 X33.613 Y112.559 E0.02093
G1 X32.817 Y112.708 E0.02112
G1 X32.045 Y112.954 E0.02112
G1 X31.309 Y113.293 E0.02112
G1 X30.620 Y113.720 E0.02112
G1 X29.989 Y114.229 E0.02112
G1 X29.425 Y114.811 E0.02112
G1 X28.937 Y115.458 E0.02112
G1 X28.533 Y116.160 E0.02112
G1 X28.218 Y116.907 E0.02112
G1 X28.047 Y117.510 E0.01632
G1 X20.442 Y117.510 E0.19815
G1 X20.442 Y103.020 E0.37755
M204 S1250
G1 X20.816 Y103.102 F9000.000
G1 E-2.24000 F2400.000
```

```
;WIPE_START
G1 F7200.000
G1 X23.322 Y102.997 E-0.91200
;WIPE_END
G1 E-0.04800 F2400.000
G1 Z1.150 F9000.000
G1 X41.452 Y111.215
G1 Z0.950
G1 E3.20000 F1500.000
M204 S1000
;TYPE:Top solid infill
;WIDTH:0.405169
G1 F2400.000
G1 X40.863 Y111.804 E0.01937
G1 X40.335 Y111.804 E0.01227
G1 X41.286 Y110.853 E0.03128
G1 X41.286 Y110.325 E0.01227
G1 X39.808 Y111.804 E0.04863
G1 X39.280 Y111.804 E0.01227
G1 X41.286 Y109.798 E0.06598
G1 X41.286 Y109.270 E0.01227
G1 X38.753 Y111.804 E0.08333
G1 X38.225 Y111.804 E0.01227
G1 X41.286 Y108.743 E0.10068
G1 X41.286 Y108.666 E0.00179
G1 X40.836 Y108.666 E0.01048
G1 X37.698 Y111.804 E0.10321
G1 X37.171 Y111.804 E0.01227
G1 X40.308 Y108.666 E0.10321
```

```
G1 X39.781 Y108.666 E0.01227
G1 X36.643 Y111.804 E0.10321
G1 X36.116 Y111.804 E0.01227
G1 X39.253 Y108.666 E0.10321
G1 X38.726 Y108.666 E0.01227
G1 X35.588 Y111.804 E0.10321
G1 X35.061 Y111.804 E0.01227
G1 X38.198 Y108.666 E0.10321
G1 X37.671 Y108.666 E0.01227
G1 X34.533 Y111.804 E0.10321
G1 X33.980 Y111.829 E0.01288
G1 X37.143 Y108.666 E0.10405
G1 X36.616 Y108.666 E0.01227
G1 X33.401 Y111.880 E0.10574
G1 X32.752 Y112.002 E0.01536
G1 X36.088 Y108.666 E0.10974
G1 X35.561 Y108.666 E0.01227
G1 X31.717 Y112.510 E0.12645
M204 S1250
G1 E-2.24000
;WIPE_START
G1 F7200.000
G1 X33.753 Y110.473 E-0.91200
;WIPE_END
G1 E-0.04800 F2400.000
G1 Z1.150 F9000.000
G1 X27.858 Y116.368
G1 Z0.950
G1 E3.20000 F1500.000
```

```
M204 S1000
G1 F2400.000
G1 X27.423 Y116.804 E0.01431
G1 X26.896 Y116.804 E0.01227
G1 X27.961 Y115.738 E0.03505
G1 X28.347 Y115.068 E0.01799
G1 X28.888 Y114.351 E0.02088
G1 X29.512 Y113.706 E0.02088
G1 X30.211 Y113.143 E0.02088
G1 X31.077 Y112.622 E0.02350
G1 X35.034 Y108.666 E0.13015
G1 X34.506 Y108.666 E0.01227
G1 X26.368 Y116.804 E0.26769
G1 X25.841 Y116.804 E0.01227
G1 X34.003 Y108.642 E0.26848
G1 X33.508 Y108.609 E0.01153
G1 X25.313 Y116.804 E0.26956
G1 X24.786 Y116.804 E0.01227
G1 X33.064 Y108.526 E0.27230
G1 X32.622 Y108.440 E0.01047
G1 X24.258 Y116.804 E0.27511
G1 X23.731 Y116.804 E0.01227
G1 X32.222 Y108.313 E0.27930
G1 X31.822 Y108.185 E0.00977
G1 X23.204 Y116.804 E0.28349
G1 X22.676 Y116.804 E0.01227
G1 X31.457 Y108.022 E0.28886
G1 X31.096 Y107.856 E0.00925
G1 X22.149 Y116.804 E0.29433
```

```
G1 X21.621 Y116.804 E0.01227
G1 X30.759 Y107.666 E0.30057
G1 X30.433 Y107.464 E0.00891
G1 X21.149 Y116.749 E0.30541
G1 X21.149 Y116.221 E0.01227
G1 X30.118 Y107.252 E0.29504
G1 X29.826 Y107.016 E0.00873
G1 X21.149 Y115.694 E0.28544
M73 P85 R10
G1 X21.149 Y115.166 E0.01227
G1 X29.534 Y106.781 E0.27583
G1 X29.272 Y106.515 E0.00868
G1 X21.149 Y114.639 E0.26721
G1 X21.149 Y114.111 E0.01227
G1 X29.012 Y106.247 E0.25868
G1 X28.770 Y105.962 E0.00870
G1 X21.149 Y113.584 E0.25070
G1 X21.149 Y113.056 E0.01227
G1 X28.543 Y105.662 E0.24324
G1 X28.321 Y105.356 E0.00878
G1 X21.149 Y112.529 E0.23594
G1 X21.149 Y112.001 E0.01227
G1 X28.128 Y105.021 E0.22960
G1 X27.936 Y104.687 E0.00898
G1 X21.149 Y111.474 E0.22326
G1 X21.149 Y110.946 E0.01227
G1 X27.775 Y104.320 E0.21797
G1 X27.623 Y103.945 E0.00942
G1 X21.149 Y110.419 E0.21296
```

```
G1 X21.149 Y109.891 E0.01227
G1 X27.374 Y103.666 E0.20479
G1 X26.847 Y103.666 E0.01227
G1 X21.149 Y109.364 E0.18744
G1 X21.149 Y108.836 E0.01227
G1 X26.319 Y103.666 E0.17009
G1 X25.792 Y103.666 E0.01227
G1 X21.149 Y108.309 E0.15273
G1 X21.149 Y107.781 E0.01227
G1 X25.264 Y103.666 E0.13538
G1 X24.737 Y103.666 E0.01227
G1 X21.149 Y107.254 E0.11803
G1 X21.149 Y106.727 E0.01227
G1 X24.209 Y103.666 E0.10068
G1 X23.682 Y103.666 E0.01227
G1 X21.149 Y106.199 E0.08333
G1 X21.149 Y105.672 E0.01227
G1 X23.154 Y103.666 E0.06598
G1 X22.627 Y103.666 E0.01227
G1 X21.149 Y105.144 E0.04863
G1 X21.149 Y104.617 E0.01227
G1 X22.099 Y103.666 E0.03128
G1 X21.572 Y103.666 E0.01227
G1 X20.983 Y104.255 E0.01937
M204 S1250
; stop printing object tpu print.STL id:13 copy 0
; printing object tpu print.STL id:17 copy 0
G1 E-2.24000
;WIPE_START
```

```
G1 F7200.000
G1 X21.572 Y103.666 E-0.26372
G1 X22.099 Y103.666 E-0.16703
G1 X21.149 Y104.617 E-0.42582
G1 X21.149 Y104.792 E-0.05543
;WIPE_END
G1 E-0.04800 F2400.000
G1 Z1.150 F9000.000
G1 X20.983 Y122.031
G1 Z0.950
G1 E3.20000 F1500.000
M204 S800
;TYPE:Perimeter
;WIDTH:0.45
G1 F2400.000
G1 X27.716 Y122.031 E0.17544
G1 X27.780 Y122.257 E0.00610
G1 X28.141 Y123.145 E0.02499
G1 X28.573 Y123.896 E0.02257
G1 X29.092 Y124.584 E0.02246
G1 X29.692 Y125.204 E0.02246
G1 X30.363 Y125.745 E0.02246
G1 X31.095 Y126.199 E0.02246
G1 X31.878 Y126.559 E0.02246
G1 X32.700 Y126.821 E0.02246
G1 X33.547 Y126.980 E0.02246
G1 X34.387 Y127.031 E0.02194
G1 X41.560 Y127.031 E0.18688
G1 X41.560 Y130.746 E0.09678
```

```
G1 X34.387 Y130.746 E0.18688
G1 X33.547 Y130.798 E0.02194
G1 X32.700 Y130.956 E0.02246
G1 X31.878 Y131.218 E0.02246
G1 X31.095 Y131.578 E0.02246
G1 X30.363 Y132.033 E0.02246
G1 X29.692 Y132.573 E0.02246
G1 X29.092 Y133.193 E0.02246
G1 X28.573 Y133.881 E0.02246
G1 X28.143 Y134.628 E0.02246
G1 X27.808 Y135.422 E0.02246
G1 X27.716 Y135.746 E0.00876
G1 X20.845 Y135.746 E0.17903
G1 X20.845 Y122.031 E0.35734
G1 X20.923 Y122.031 E0.00203
M204 S1250
G1 X20.427 Y121.614 F9000.000
M204 S800
;TYPE:External perimeter
G1 F1800.000
G1 X28.032 Y121.614 E0.19815
G1 X28.176 Y122.121 E0.01373
G1 X28.517 Y122.962 E0.02365
G1 X28.922 Y123.665 E0.02116
G1 X29.410 Y124.312 E0.02112
G1 X29.974 Y124.895 E0.02112
G1 X30.605 Y125.403 E0.02112
G1 X31.294 Y125.830 E0.02112
G1 X32.030 Y126.169 E0.02112
```

G1 X32.802 Y126.415 E0.02112

G1 X33.598 Y126.564 E0.02112

G1 X34.400 Y126.614 E0.02093

G1 X41.977 Y126.614 E0.19743

G1 X41.977 Y131.164 E0.11855

G1 X34.400 Y131.164 E0.19743

G1 X33.598 Y131.213 E0.02093

G1 X32.802 Y131.362 E0.02112

G1 X32.030 Y131.608 E0.02112

G1 X31.294 Y131.947 E0.02112

G1 X30.605 Y132.374 E0.02112

G1 X29.974 Y132.883 E0.02112

G1 X29.410 Y133.465 E0.02112

G1 X28.922 Y134.112 E0.02112

G1 X28.518 Y134.814 E0.02112

G1 X28.203 Y135.561 E0.02112

G1 X28.032 Y136.164 E0.01632

G1 X20.427 Y136.164 E0.19815

G1 X20.427 Y121.674 E0.37755

M204 S1250

G1 X20.801 Y121.756 F9000.000

G1 E-2.24000 F2400.000

;WIPE_START

G1 F7200.000

G1 X23.307 Y121.651 E-0.91200

;WIPE_END

G1 E-0.04800 F2400.000

G1 Z1.150 F9000.000

G1 X41.437 Y129.868

```
G1 Z0.950
G1 E3.20000 F1500.000
M204 S1000
;TYPE:Top solid infill
;WIDTH:0.405169
G1 F2400.000
G1 X40.848 Y130.457 E0.01937
G1 X40.320 Y130.457 E0.01227
G1 X41.271 Y129.507 E0.03128
G1 X41.271 Y128.979 E0.01227
G1 X39.793 Y130.457 E0.04863
G1 X39.265 Y130.457 E0.01227
G1 X41.271 Y128.452 E0.06598
G1 X41.271 Y127.924 E0.01227
G1 X38.738 Y130.457 E0.08333
G1 X38.210 Y130.457 E0.01227
G1 X41.271 Y127.397 E0.10068
G1 X41.271 Y127.320 E0.00179
G1 X40.821 Y127.320 E0.01048
G1 X37.683 Y130.457 E0.10321
G1 X37.155 Y130.457 E0.01227
G1 X40.293 Y127.320 E0.10321
G1 X39.766 Y127.320 E0.01227
G1 X36.628 Y130.457 E0.10321
G1 X36.100 Y130.457 E0.01227
G1 X39.238 Y127.320 E0.10321
G1 X38.711 Y127.320 E0.01227
G1 X35.573 Y130.457 E0.10321
G1 X35.046 Y130.457 E0.01227
```

```
G1 X38.183 Y127.320 E0.10321
G1 X37.656 Y127.320 E0.01227
G1 X34.518 Y130.457 E0.10321
G1 X33.965 Y130.483 E0.01288
G1 X37.128 Y127.320 E0.10405
G1 X36.601 Y127.320 E0.01227
G1 X33.386 Y130.534 E0.10574
G1 X32.737 Y130.656 E0.01536
G1 X36.073 Y127.320 E0.10974
G1 X35.546 Y127.320 E0.01227
G1 X31.702 Y131.164 E0.12645
M204 S1250
G1 E-2.24000
;WIPE_START
G1 F7200.000
G1 X33.738 Y129.127 E-0.91200
;WIPE_END
G1 E-0.04800 F2400.000
G1 Z1.150 F9000.000
G1 X27.843 Y135.022
G1 Z0.950
G1 E3.20000 F1500.000
M204 S1000
G1 F2400.000
G1 X27.408 Y135.457 E0.01431
G1 X26.881 Y135.457 E0.01227
G1 X27.946 Y134.392 E0.03505
G1 X28.332 Y133.722 E0.01799
G1 X28.872 Y133.005 E0.02088
```

```
G1 X29.497 Y132.360 E0.02088
G1 X30.196 Y131.797 E0.02088
G1 X31.062 Y131.276 E0.02350
G1 X35.018 Y127.320 E0.13015
G1 X34.491 Y127.320 E0.01227
G1 X26.353 Y135.457 E0.26769
G1 X25.826 Y135.457 E0.01227
G1 X33.988 Y127.296 E0.26848
G1 X33.493 Y127.263 E0.01153
G1 X25.298 Y135.457 E0.26956
G1 X24.771 Y135.457 E0.01227
G1 X33.049 Y127.180 E0.27230
G1 X32.607 Y127.094 E0.01047
G1 X24.243 Y135.457 E0.27511
G1 X23.716 Y135.457 E0.01227
G1 X32.207 Y126.967 E0.27930
G1 X31.807 Y126.839 E0.00977
G1 X23.188 Y135.457 E0.28349
G1 X22.661 Y135.457 E0.01227
G1 X31.442 Y126.676 E0.28886
G1 X31.081 Y126.510 E0.00925
G1 X22.133 Y135.457 E0.29433
G1 X21.606 Y135.457 E0.01227
G1 X30.743 Y126.320 E0.30057
G1 X30.418 Y126.118 E0.00891
G1 X21.134 Y135.402 E0.30541
G1 X21.134 Y134.875 E0.01227
G1 X30.103 Y125.906 E0.29504
G1 X29.811 Y125.670 E0.00873
```

G1 X21.134 Y134.348 E0.28544

G1 X21.134 Y133.820 E0.01227

G1 X29.519 Y125.435 E0.27583

G1 X29.257 Y125.169 E0.00868

G1 X21.134 Y133.293 E0.26721

G1 X21.134 Y132.765 E0.01227

G1 X28.997 Y124.901 E0.25868

G1 X28.755 Y124.616 E0.00870

G1 X21.134 Y132.238 E0.25070

G1 X21.134 Y131.710 E0.01227

G1 X28.528 Y124.315 E0.24324

G1 X28.306 Y124.010 E0.00878

G1 X21.134 Y131.183 E0.23594

G1 X21.134 Y130.655 E0.01227

G1 X28.113 Y123.675 E0.22960

G1 X27.921 Y123.341 E0.00898

G1 X21.134 Y130.128 E0.22326

G1 X21.134 Y129.600 E0.01227

G1 X27.760 Y122.974 E0.21797

G1 X27.608 Y122.599 E0.00942

G1 X21.134 Y129.073 E0.21296

G1 X21.134 Y128.545 E0.01227

G1 X27.359 Y122.320 E0.20479

G1 X26.832 Y122.320 E0.01227

G1 X21.134 Y128.018 E0.18744

G1 X21.134 Y127.490 E0.01227

G1 X26.304 Y122.320 E0.17009

G1 X25.777 Y122.320 E0.01227

G1 X21.134 Y126.963 E0.15273

G1 X21.134 Y126.435 E0.01227

G1 X25.249 Y122.320 E0.13538

G1 X24.722 Y122.320 E0.01227

G1 X21.134 Y125.908 E0.11803

G1 X21.134 Y125.380 E0.01227

G1 X24.194 Y122.320 E0.10068

G1 X23.667 Y122.320 E0.01227

G1 X21.134 Y124.853 E0.08333

G1 X21.134 Y124.326 E0.01227

G1 X23.139 Y122.320 E0.06598

G1 X22.612 Y122.320 E0.01227

G1 X21.134 Y123.798 E0.04863

G1 X21.134 Y123.271 E0.01227

G1 X22.084 Y122.320 E0.03128

G1 X21.557 Y122.320 E0.01227

G1 X20.968 Y122.909 E0.01937

M204 S1250

; stop printing object tpu print.STL id:17 copy 0

; printing object Petg print.STL id:16 copy 0

; stop printing object Petg print.STL id:16 copy 0

; printing object Petg print.STL id:12 copy 0

; stop printing object Petg print.STL id:12 copy 0

; printing object tpu print.STL id:7 copy 0

G1 E-2.24000

;WIPE_START

G1 F7200.000

G1 X21.557 Y122.320 E-0.26372

G1 X22.084 Y122.320 E-0.16703

G1 X21.134 Y123.271 E-0.42582

```
G1 X21.134 Y123.446 E-0.05543
;WIPE_END
G1 E-0.04800 F2400.000
G1 Z1.150 F9000.000
G1 X69.635 Y122.612
G1 Z0.950
G1 E3.20000 F1500.000
M204 S800
;TYPE:Perimeter
;WIDTH:0.45
G1 F2400.000
G1 X76.506 Y122.612 E0.17903
G1 X76.569 Y122.837 E0.00610
G1 X76.930 Y123.726 E0.02499
G1 X77.362 Y124.477 E0.02257
G1 X77.881 Y125.165 E0.02246
G1 X78.481 Y125.785 E0.02246
G1 X79.152 Y126.325 E0.02246
G1 X79.885 Y126.780 E0.02246
G1 X80.668 Y127.140 E0.02246
G1 X81.489 Y127.402 E0.02246
G1 X82.336 Y127.560 E0.02246
G1 X83.177 Y127.612 E0.02194
G1 X90.349 Y127.612 E0.18688
G1 X90.349 Y131.327 E0.09678
G1 X83.177 Y131.327 E0.18688
G1 X82.336 Y131.379 E0.02194
G1 X81.489 Y131.537 E0.02246
G1 X80.668 Y131.799 E0.02246
```

```
G1 X79.885 Y132.159 E0.02246
G1 X79.152 Y132.613 E0.02246
G1 X78.481 Y133.154 E0.02246
G1 X77.881 Y133.774 E0.02246
G1 X77.362 Y134.462 E0.02246
G1 X76.932 Y135.209 E0.02246
G1 X76.597 Y136.003 E0.02246
G1 X76.506 Y136.327 E0.00876
G1 X69.635 Y136.327 E0.17903
G1 X69.635 Y122.672 E0.35578
M204 S1250
G1 X69.217 Y122.194 F9000.000
M204 S800
;TYPE:External perimeter
G1 F1800.000
G1 X76.822 Y122.194 E0.19815
G1 X76.965 Y122.702 E0.01373
G1 X77.306 Y123.542 E0.02365
G1 X77.712 Y124.246 E0.02116
G1 X78.199 Y124.893 E0.02112
G1 X78.763 Y125.475 E0.02112
G1 X79.394 Y125.984 E0.02112
G1 X80.083 Y126.411 E0.02112
G1 X80.819 Y126.750 E0.02112
G1 X81.591 Y126.996 E0.02112
G1 X82.388 Y127.145 E0.02112
G1 X83.190 Y127.194 E0.02093
G1 X90.767 Y127.194 E0.19743
G1 X90.767 Y131.744 E0.11855
```

```
G1 X83.190 Y131.744 E0.19743
G1 X82.388 Y131.794 E0.02093
G1 X81.591 Y131.943 E0.02112
G1 X80.819 Y132.189 E0.02112
G1 X80.083 Y132.528 E0.02112
G1 X79.394 Y132.955 E0.02112
G1 X78.763 Y133.463 E0.02112
G1 X78.199 Y134.046 E0.02112
G1 X77.712 Y134.693 E0.02112
G1 X77.307 Y135.395 E0.02112
G1 X76.992 Y136.142 E0.02112
G1 X76.822 Y136.744 E0.01632
G1 X69.217 Y136.744 E0.19815
G1 X69.217 Y122.254 E0.37755
M204 S1250
G1 X69.591 Y122.337 F9000.000
G1 E-2.24000 F2400.000
;WIPE_START
G1 F7200.000
G1 X72.097 Y122.232 E-0.91200
;WIPE_END
G1 E-0.04800 F2400.000
G1 Z1.150 F9000.000
G1 X90.226 Y130.449
G1 Z0.950
G1 E3.20000 F1500.000
M204 S1000
;TYPE:Top solid infill
;WIDTH:0.405169
```

```
G1 F2400.000
G1 X89.637 Y131.038 E0.01937
G1 X89.110 Y131.038 E0.01227
G1 X90.061 Y130.087 E0.03128
G1 X90.061 Y129.560 E0.01227
G1 X88.582 Y131.038 E0.04863
G1 X88.055 Y131.038 E0.01227
G1 X90.061 Y129.032 E0.06598
G1 X90.061 Y128.505 E0.01227
G1 X87.527 Y131.038 E0.08333
G1 X87.000 Y131.038 E0.01227
G1 X90.061 Y127.977 E0.10068
G1 X90.061 Y127.901 E0.00179
G1 X89.610 Y127.901 E0.01048
G1 X86.472 Y131.038 E0.10321
G1 X85.945 Y131.038 E0.01227
G1 X89.082 Y127.901 E0.10321
G1 X88.555 Y127.901 E0.01227
G1 X85.417 Y131.038 E0.10321
G1 X84.890 Y131.038 E0.01227
G1 X88.028 Y127.901 E0.10321
G1 X87.500 Y127.901 E0.01227
G1 X84.362 Y131.038 E0.10321
G1 X83.835 Y131.038 E0.01227
G1 X86.973 Y127.901 E0.10321
G1 X86.445 Y127.901 E0.01227
G1 X83.307 Y131.038 E0.10321
G1 X82.754 Y131.064 E0.01288
G1 X85.918 Y127.901 E0.10405
```

```
G1 X85.390 Y127.901 E0.01227
G1 X82.176 Y131.115 E0.10574
G1 X81.527 Y131.237 E0.01536
G1 X84.863 Y127.901 E0.10974
G1 X84.335 Y127.901 E0.01227
G1 X80.491 Y131.745 E0.12645
M204 S1250
G1 E-2.24000
;WIPE_START
G1 F7200.000
G1 X82.528 Y129.708 E-0.91200
;WIPE_END
G1 E-0.04800 F2400.000
G1 Z1.150 F9000.000
G1 X76.633 Y135.603
G1 Z0.950
G1 E3.20000 F1500.000
M204 S1000
G1 F2400.000
G1 X76.198 Y136.038 E0.01431
G1 X75.670 Y136.038 E0.01227
G1 X76.735 Y134.973 E0.03505
G1 X77.122 Y134.303 E0.01799
G1 X77.662 Y133.586 E0.02088
G1 X78.286 Y132.941 E0.02088
G1 X78.985 Y132.378 E0.02088
G1 X79.851 Y131.857 E0.02350
G1 X83.808 Y127.901 E0.13015
G1 X83.280 Y127.901 E0.01227
```

```
G1 X75.143 Y136.038 E0.26769
G1 X74.615 Y136.038 E0.01227
G1 X82.777 Y127.876 E0.26848
G1 X82.282 Y127.844 E0.01153
G1 X74.088 Y136.038 E0.26956
G1 X73.560 Y136.038 E0.01227
G1 X81.838 Y127.761 E0.27230
G1 X81.396 Y127.675 E0.01047
G1 X73.033 Y136.038 E0.27511
G1 X72.505 Y136.038 E0.01227
G1 X80.996 Y127.547 E0.27930
G1 X80.596 Y127.420 E0.00977
G1 X71.978 Y136.038 E0.28349
G1 X71.450 Y136.038 E0.01227
G1 X80.232 Y127.257 E0.28886
G1 X79.871 Y127.091 E0.00925
G1 X70.923 Y136.038 E0.29433
G1 X70.395 Y136.038 E0.01227
G1 X79.533 Y126.901 E0.30057
G1 X79.207 Y126.699 E0.00891
G1 X69.923 Y135.983 E0.30541
G1 X69.923 Y135.456 E0.01227
G1 X78.892 Y126.486 E0.29504
G1 X78.600 Y126.251 E0.00873
G1 X69.923 Y134.928 E0.28544
G1 X69.923 Y134.401 E0.01227
G1 X78.308 Y126.016 E0.27583
G1 X78.046 Y125.750 E0.00868
G1 X69.923 Y133.873 E0.26721
```

```
G1 X69.923 Y133.346 E0.01227
G1 X77.787 Y125.482 E0.25868
G1 X77.544 Y125.197 E0.00870
G1 X69.923 Y132.818 E0.25070
G1 X69.923 Y132.291 E0.01227
G1 X77.318 Y124.896 E0.24324
G1 X77.095 Y124.591 E0.00878
G1 X69.923 Y131.764 E0.23594
G1 X69.923 Y131.236 E0.01227
G1 X76.903 Y124.256 E0.22960
G1 X76.710 Y123.922 E0.00898
G1 X69.923 Y130.709 E0.22326
G1 X69.923 Y130.181 E0.01227
G1 X76.549 Y123.555 E0.21797
G1 X76.397 Y123.180 E0.00942
G1 X69.923 Y129.654 E0.21296
G1 X69.923 Y129.126 E0.01227
G1 X76.148 Y122.901 E0.20479
G1 X75.621 Y122.901 E0.01227
G1 X69.923 Y128.599 E0.18744
M73 P86 R10
G1 X69.923 Y128.071 E0.01227
G1 X75.093 Y122.901 E0.17009
G1 X74.566 Y122.901 E0.01227
G1 X69.923 Y127.544 E0.15273
G1 X69.923 Y127.016 E0.01227
G1 X74.039 Y122.901 E0.13538
G1 X73.511 Y122.901 E0.01227
G1 X69.923 Y126.489 E0.11803
```

```
G1 X69.923 Y125.961 E0.01227
G1 X72.984 Y122.901 E0.10068
G1 X72.456 Y122.901 E0.01227
G1 X69.923 Y125.434 E0.08333
G1 X69.923 Y124.906 E0.01227
G1 X71.929 Y122.901 E0.06598
G1 X71.401 Y122.901 E0.01227
G1 X69.923 Y124.379 E0.04863
G1 X69.923 Y123.851 E0.01227
G1 X70.874 Y122.901 E0.03128
G1 X70.346 Y122.901 E0.01227
G1 X69.757 Y123.490 E0.01937
M204 S1250
; stop printing object tpu print.STL id:7 copy 0
; printing object tpu print.STL id:3 copy 0
G1 E-2.24000
;WIPE_START
G1 F7200.000
G1 X70.346 Y122.901 E-0.26372
G1 X70.874 Y122.901 E-0.16703
G1 X69.923 Y123.851 E-0.42582
G1 X69.923 Y124.026 E-0.05543
;WIPE_END
G1 E-0.04800 F2400.000
G1 Z1.150 F9000.000
G1 X69.757 Y117.673
G1 Z0.950
G1 E3.20000 F1500.000
M204 S800
```

```
;TYPE:Perimeter
;WIDTH:0.45
G1 F2400.000
G1 X69.650 Y117.673 E0.00281
G1 X69.650 Y103.958 E0.35734
G1 X76.521 Y103.958 E0.17903
G1 X76.584 Y104.183 E0.00610
G1 X76.945 Y105.072 E0.02499
G1 X77.378 Y105.823 E0.02257
G1 X77.896 Y106.511 E0.02246
G1 X78.496 Y107.131 E0.02246
G1 X79.167 Y107.672 E0.02246
G1 X79.900 Y108.126 E0.02246
G1 X80.683 Y108.486 E0.02246
G1 X81.504 Y108.748 E0.02246
G1 X82.351 Y108.906 E0.02246
G1 X83.192 Y108.958 E0.02194
G1 X90.364 Y108.958 E0.18688
M73 P86 R9
G1 X90.364 Y112.673 E0.09678
G1 X83.192 Y112.673 E0.18688
G1 X82.351 Y112.725 E0.02194
G1 X81.504 Y112.883 E0.02246
G1 X80.683 Y113.145 E0.02246
G1 X79.900 Y113.505 E0.02246
G1 X79.167 Y113.960 E0.02246
G1 X78.496 Y114.500 E0.02246
G1 X77.896 Y115.120 E0.02246
G1 X77.378 Y115.808 E0.02246
```

G1 X76.947 Y116.555 E0.02246

G1 X76.612 Y117.349 E0.02246

G1 X76.521 Y117.673 E0.00876

G1 X69.817 Y117.673 E0.17466

M204 S1250

G1 X69.232 Y118.091 F9000.000

M204 S800

;TYPE:External perimeter

G1 F1800.000

G1 X69.232 Y103.541 E0.37911

G1 X76.837 Y103.541 E0.19815

G1 X76.980 Y104.048 E0.01373

G1 X77.322 Y104.889 E0.02365

G1 X77.727 Y105.592 E0.02116

G1 X78.215 Y106.239 E0.02112

G1 X78.778 Y106.822 E0.02112

G1 X79.409 Y107.330 E0.02112

G1 X80.098 Y107.757 E0.02112

G1 X80.834 Y108.096 E0.02112

G1 X81.606 Y108.342 E0.02112

G1 X82.403 Y108.491 E0.02112

G1 X83.205 Y108.541 E0.02093

G1 X90.782 Y108.541 E0.19743

G1 X90.782 Y113.091 E0.11855

G1 X83.205 Y113.091 E0.19743

G1 X82.403 Y113.140 E0.02093

G1 X81.606 Y113.289 E0.02112

G1 X80.834 Y113.535 E0.02112

G1 X80.098 Y113.874 E0.02112

```
G1 X79.409 Y114.301 E0.02112
G1 X78.778 Y114.810 E0.02112
G1 X78.215 Y115.392 E0.02112
G1 X77.727 Y116.039 E0.02112
G1 X77.322 Y116.741 E0.02112
G1 X77.007 Y117.488 E0.02112
G1 X76.837 Y118.091 E0.01632
G1 X69.292 Y118.091 E0.19659
M204 S1250
G1 X69.423 Y117.739 F9000.000
G1 E-2.24000 F2400.000
;WIPE_START
G1 F7200.000
G1 X69.280 Y115.211 E-0.91200
;WIPE_END
G1 E-0.04800 F2400.000
G1 Z1.150 F9000.000
G1 X90.241 Y111.795
G1 Z0.950
G1 E3.20000 F1500.000
M204 S1000
;TYPE:Top solid infill
;WIDTH:0.405169
G1 F2400.000
G1 X89.652 Y112.384 E0.01937
G1 X89.125 Y112.384 E0.01227
G1 X90.076 Y111.433 E0.03128
G1 X90.076 Y110.906 E0.01227
G1 X88.597 Y112.384 E0.04863
```

G1 X88.070 Y112.384 E0.01227

G1 X90.076 Y110.378 E0.06598

G1 X90.076 Y109.851 E0.01227

G1 X87.542 Y112.384 E0.08333

G1 X87.015 Y112.384 E0.01227

G1 X90.076 Y109.324 E0.10068

G1 X90.076 Y109.247 E0.00179

G1 X89.625 Y109.247 E0.01048

G1 X86.487 Y112.384 E0.10321

G1 X85.960 Y112.384 E0.01227

G1 X89.098 Y109.247 E0.10321

G1 X88.570 Y109.247 E0.01227

G1 X85.432 Y112.384 E0.10321

G1 X84.905 Y112.384 E0.01227

G1 X88.043 Y109.247 E0.10321

G1 X87.515 Y109.247 E0.01227

G1 X84.377 Y112.384 E0.10321

G1 X83.850 Y112.384 E0.01227

G1 X86.988 Y109.247 E0.10321

G1 X86.460 Y109.247 E0.01227

G1 X83.323 Y112.384 E0.10321

G1 X82.770 Y112.410 E0.01288

G1 X85.933 Y109.247 E0.10405

G1 X85.405 Y109.247 E0.01227

G1 X82.191 Y112.461 E0.10574

G1 X81.542 Y112.583 E0.01536

G1 X84.878 Y109.247 E0.10974

G1 X84.350 Y109.247 E0.01227

G1 X80.506 Y113.091 E0.12645

```
M204 S1250
G1 E-2.24000
;WIPE_START
G1 F7200.000
G1 X82.543 Y111.054 E-0.91200
;WIPE_END
G1 E-0.04800 F2400.000
G1 Z1.150 F9000.000
G1 X76.648 Y116.949
G1 Z0.950
G1 E3.20000 F1500.000
M204 S1000
G1 F2400.000
G1 X76.213 Y117.384 E0.01431
G1 X75.685 Y117.384 E0.01227
G1 X76.751 Y116.319 E0.03505
G1 X77.137 Y115.649 E0.01799
G1 X77.677 Y114.932 E0.02088
G1 X78.301 Y114.287 E0.02088
G1 X79.000 Y113.724 E0.02088
G1 X79.866 Y113.203 E0.02350
G1 X83.823 Y109.247 E0.13015
G1 X83.295 Y109.247 E0.01227
G1 X75.158 Y117.384 E0.26769
G1 X74.630 Y117.384 E0.01227
G1 X82.792 Y109.223 E0.26848
G1 X82.297 Y109.190 E0.01153
G1 X74.103 Y117.384 E0.26956
G1 X73.575 Y117.384 E0.01227
```

```
G1 X81.853 Y109.107 E0.27230
G1 X81.411 Y109.021 E0.01047
G1 X73.048 Y117.384 E0.27511
G1 X72.520 Y117.384 E0.01227
G1 X81.011 Y108.894 E0.27930
G1 X80.611 Y108.766 E0.00977
G1 X71.993 Y117.384 E0.28349
G1 X71.465 Y117.384 E0.01227
G1 X80.247 Y108.603 E0.28886
G1 X79.886 Y108.437 E0.00925
G1 X70.938 Y117.384 E0.29433
G1 X70.410 Y117.384 E0.01227
G1 X79.548 Y108.247 E0.30057
G1 X79.222 Y108.045 E0.00891
G1 X69.938 Y117.329 E0.30541
G1 X69.938 Y116.802 E0.01227
G1 X78.907 Y107.833 E0.29504
G1 X78.615 Y107.597 E0.00873
G1 X69.938 Y116.274 E0.28544
G1 X69.938 Y115.747 E0.01227
G1 X78.323 Y107.362 E0.27583
G1 X78.061 Y107.096 E0.00868
G1 X69.938 Y115.219 E0.26721
G1 X69.938 Y114.692 E0.01227
G1 X77.802 Y106.828 E0.25868
G1 X77.559 Y106.543 E0.00870
G1 X69.938 Y114.165 E0.25070
G1 X69.938 Y113.637 E0.01227
G1 X77.333 Y106.242 E0.24324
```

G1 X77.111 Y105.937 E0.00878

G1 X69.938 Y113.110 E0.23594

G1 X69.938 Y112.582 E0.01227

G1 X76.918 Y105.602 E0.22960

G1 X76.725 Y105.268 E0.00898

G1 X69.938 Y112.055 E0.22326

G1 X69.938 Y111.527 E0.01227

G1 X76.564 Y104.901 E0.21797

G1 X76.412 Y104.526 E0.00942

G1 X69.938 Y111.000 E0.21296

G1 X69.938 Y110.472 E0.01227

G1 X76.164 Y104.247 E0.20479

G1 X75.636 Y104.247 E0.01227

G1 X69.938 Y109.945 E0.18744

G1 X69.938 Y109.417 E0.01227

G1 X75.109 Y104.247 E0.17009

G1 X74.581 Y104.247 E0.01227

G1 X69.938 Y108.890 E0.15273

G1 X69.938 Y108.362 E0.01227

G1 X74.054 Y104.247 E0.13538

G1 X73.526 Y104.247 E0.01227

G1 X69.938 Y107.835 E0.11803

G1 X69.938 Y107.307 E0.01227

G1 X72.999 Y104.247 E0.10068

G1 X72.471 Y104.247 E0.01227

G1 X69.938 Y106.780 E0.08333

G1 X69.938 Y106.252 E0.01227

G1 X71.944 Y104.247 E0.06598

G1 X71.416 Y104.247 E0.01227

```
G1 X69.938 Y105.725 E0.04863
G1 X69.938 Y105.198 E0.01227
G1 X70.889 Y104.247 E0.03128
G1 X70.361 Y104.247 E0.01227
G1 X69.772 Y104.836 E0.01937
M204 S1250
; stop printing object tpu print.STL id:3 copy 0
; printing object Petg print.STL id:2 copy 0
; stop printing object Petg print.STL id:2 copy 0
; printing object Petg print.STL id:6 copy 0
; stop printing object Petg print.STL id:6 copy 0
; printing object tpu print.STL id:27 copy 0
G1 E-2.24000
;WIPE_START
G1 F7200.000
G1 X70.361 Y104.247 E-0.26372
G1 X70.889 Y104.247 E-0.16703
G1 X69.938 Y105.198 E-0.42582
G1 X69.938 Y105.373 E-0.05543
;WIPE_END
G1 E-0.04800 F2400.000
G1 Z1.150 F9000.000
G1 X120.285 Y122.787
G1 Z0.950
G1 E3.20000 F1500.000
M204 S800
;TYPE:Perimeter
;WIDTH:0.45
G1 F2400.000
```

```
G1 X127.156 Y122.787 E0.17903
G1 X127.220 Y123.012 E0.00610
G1 X127.580 Y123.901 E0.02499
G1 X128.013 Y124.652 E0.02257
G1 X128.532 Y125.340 E0.02246
G1 X129.131 Y125.960 E0.02246
G1 X129.802 Y126.501 E0.02246
G1 X130.535 Y126.955 E0.02246
G1 X131.318 Y127.315 E0.02246
G1 X132.139 Y127.577 E0.02246
G1 X132.986 Y127.735 E0.02246
G1 X133.827 Y127.787 E0.02194
G1 X140.999 Y127.787 E0.18688
G1 X140.999 Y131.502 E0.09678
G1 X133.827 Y131.502 E0.18688
G1 X132.986 Y131.554 E0.02194
G1 X132.139 Y131.712 E0.02246
G1 X131.318 Y131.974 E0.02246
G1 X130.535 Y132.334 E0.02246
G1 X129.802 Y132.789 E0.02246
G1 X129.131 Y133.329 E0.02246
G1 X128.532 Y133.949 E0.02246
G1 X128.013 Y134.637 E0.02246
G1 X127.583 Y135.384 E0.02246
G1 X127.247 Y136.178 E0.02246
G1 X127.156 Y136.502 E0.00876
G1 X120.285 Y136.502 E0.17903
G1 X120.285 Y122.847 E0.35578
M204 S1250
```

G1 X119.867 Y122.370 F9000.000

M204 S800

;TYPE:External perimeter

G1 F1800.000

G1 X127.472 Y122.370 E0.19815

G1 X127.615 Y122.877 E0.01373

G1 X127.957 Y123.718 E0.02365

G1 X128.362 Y124.421 E0.02116

G1 X128.850 Y125.068 E0.02112

G1 X129.413 Y125.651 E0.02112

G1 X130.044 Y126.159 E0.02112

G1 X130.733 Y126.586 E0.02112

G1 X131.469 Y126.925 E0.02112

G1 X132.241 Y127.171 E0.02112

G1 X133.038 Y127.320 E0.02112

G1 X133.840 Y127.370 E0.02093

G1 X141.417 Y127.370 E0.19743

G1 X141.417 Y131.920 E0.11855

G1 X133.840 Y131.920 E0.19743

G1 X133.038 Y131.969 E0.02093

G1 X132.241 Y132.118 E0.02112

G1 X131.469 Y132.364 E0.02112

G1 X130.733 Y132.703 E0.02112

G1 X130.044 Y133.130 E0.02112

G1 X129.413 Y133.639 E0.02112

G1 X128.850 Y134.221 E0.02112

G1 X128.362 Y134.868 E0.02112

G1 X127.957 Y135.570 E0.02112

G1 X127.642 Y136.317 E0.02112

```
G1 X127.472 Y136.920 E0.01632
G1 X119.867 Y136.920 E0.19815
G1 X119.867 Y122.430 E0.37755
M204 S1250
G1 X120.241 Y122.512 F9000.000
G1 E-2.24000 F2400.000
;WIPE_START
G1 F7200.000
G1 X122.747 Y122.407 E-0.91200
;WIPE_END
G1 E-0.04800 F2400.000
G1 Z1.150 F9000.000
G1 X140.876 Y130.624
G1 Z0.950
G1 E3.20000 F1500.000
M204 S1000
;TYPE:Top solid infill
;WIDTH:0.405169
G1 F2400.000
G1 X140.287 Y131.213 E0.01937
G1 X139.760 Y131.213 E0.01227
G1 X140.711 Y130.262 E0.03128
G1 X140.711 Y129.735 E0.01227
G1 X139.232 Y131.213 E0.04863
G1 X138.705 Y131.213 E0.01227
G1 X140.711 Y129.208 E0.06598
G1 X140.711 Y128.680 E0.01227
G1 X138.177 Y131.213 E0.08333
G1 X137.650 Y131.213 E0.01227
```

```
G1 X140.711 Y128.153 E0.10068
G1 X140.711 Y128.076 E0.00179
G1 X140.260 Y128.076 E0.01048
G1 X137.123 Y131.213 E0.10321
G1 X136.595 Y131.213 E0.01227
G1 X139.733 Y128.076 E0.10321
G1 X139.205 Y128.076 E0.01227
G1 X136.068 Y131.213 E0.10321
G1 X135.540 Y131.213 E0.01227
G1 X138.678 Y128.076 E0.10321
G1 X138.150 Y128.076 E0.01227
G1 X135.013 Y131.213 E0.10321
G1 X134.485 Y131.213 E0.01227
G1 X137.623 Y128.076 E0.10321
G1 X137.095 Y128.076 E0.01227
G1 X133.958 Y131.213 E0.10321
G1 X133.405 Y131.239 E0.01288
G1 X136.568 Y128.076 E0.10405
G1 X136.040 Y128.076 E0.01227
G1 X132.826 Y131.290 E0.10574
G1 X132.177 Y131.412 E0.01536
G1 X135.513 Y128.076 E0.10974
G1 X134.985 Y128.076 E0.01227
G1 X131.141 Y131.920 E0.12645
M204 S1250
G1 E-2.24000
;WIPE_START
G1 F7200.000
G1 X133.178 Y129.883 E-0.91200
```

```
;WIPE_END
G1 E-0.04800 F2400.000
G1 Z1.150 F9000.000
G1 X127.283 Y135.778
G1 Z0.950
G1 E3.20000 F1500.000
M204 S1000
G1 F2400.000
G1 X126.848 Y136.213 E0.01431
G1 X126.320 Y136.213 E0.01227
G1 X127.386 Y135.148 E0.03505
G1 X127.772 Y134.478 E0.01799
G1 X128.312 Y133.761 E0.02088
G1 X128.936 Y133.116 E0.02088
G1 X129.635 Y132.553 E0.02088
G1 X130.501 Y132.032 E0.02350
G1 X134.458 Y128.076 E0.13015
G1 X133.931 Y128.076 E0.01227
G1 X125.793 Y136.213 E0.26769
G1 X125.265 Y136.213 E0.01227
G1 X133.427 Y128.052 E0.26848
G1 X132.933 Y128.019 E0.01153
G1 X124.738 Y136.213 E0.26956
G1 X124.210 Y136.213 E0.01227
G1 X132.488 Y127.936 E0.27230
G1 X132.046 Y127.850 E0.01047
G1 X123.683 Y136.213 E0.27511
G1 X123.155 Y136.213 E0.01227
G1 X131.646 Y127.723 E0.27930
```

G1 X131.246 Y127.595 E0.00977

G1 X122.628 Y136.213 E0.28349

G1 X122.101 Y136.213 E0.01227

G1 X130.882 Y127.432 E0.28886

G1 X130.521 Y127.266 E0.00925

G1 X121.573 Y136.213 E0.29433

G1 X121.046 Y136.213 E0.01227

G1 X130.183 Y127.076 E0.30057

G1 X129.857 Y126.874 E0.00891

G1 X120.573 Y136.158 E0.30541

G1 X120.573 Y135.631 E0.01227

G1 X129.543 Y126.662 E0.29504

G1 X129.250 Y126.426 E0.00873

G1 X120.573 Y135.103 E0.28544

G1 X120.573 Y134.576 E0.01227

G1 X128.958 Y126.191 E0.27583

G1 X128.696 Y125.925 E0.00868

G1 X120.573 Y134.049 E0.26721

G1 X120.573 Y133.521 E0.01227

G1 X128.437 Y125.657 E0.25868

G1 X128.194 Y125.372 E0.00870

G1 X120.573 Y132.994 E0.25070

G1 X120.573 Y132.466 E0.01227

G1 X127.968 Y125.071 E0.24324

G1 X127.746 Y124.766 E0.00878

G1 X120.573 Y131.939 E0.23594

G1 X120.573 Y131.411 E0.01227

G1 X127.553 Y124.431 E0.22960

G1 X127.360 Y124.097 E0.00898

G1 X120.573 Y130.884 E0.22326

G1 X120.573 Y130.356 E0.01227

G1 X127.200 Y123.730 E0.21797

G1 X127.047 Y123.355 E0.00942

G1 X120.573 Y129.829 E0.21296

G1 X120.573 Y129.301 E0.01227

G1 X126.799 Y123.076 E0.20479

G1 X126.271 Y123.076 E0.01227

G1 X120.573 Y128.774 E0.18744

G1 X120.573 Y128.246 E0.01227

G1 X125.744 Y123.076 E0.17009

G1 X125.216 Y123.076 E0.01227

G1 X120.573 Y127.719 E0.15273

G1 X120.573 Y127.191 E0.01227

G1 X124.689 Y123.076 E0.13538

G1 X124.161 Y123.076 E0.01227

G1 X120.573 Y126.664 E0.11803

G1 X120.573 Y126.136 E0.01227

G1 X123.634 Y123.076 E0.10068

G1 X123.106 Y123.076 E0.01227

G1 X120.573 Y125.609 E0.08333

G1 X120.573 Y125.081 E0.01227

G1 X122.579 Y123.076 E0.06598

G1 X122.051 Y123.076 E0.01227

G1 X120.573 Y124.554 E0.04863

G1 X120.573 Y124.027 E0.01227

G1 X121.524 Y123.076 E0.03128

G1 X120.996 Y123.076 E0.01227

G1 X120.408 Y123.665 E0.01937

```
M204 S1250
; stop printing object tpu print.STL id:27 copy 0
; printing object tpu print.STL id:23 copy 0
G1 E-2.24000
;WIPE_START
G1 F7200.000
G1 X120.996 Y123.076 E-0.26372
G1 X121.524 Y123.076 E-0.16703
G1 X120.573 Y124.027 E-0.42582
G1 X120.573 Y124.202 E-0.05543
;WIPE_END
G1 E-0.04800 F2400.000
G1 Z1.150 F9000.000
G1 X120.408 Y117.848
G1 Z0.950
G1 E3.20000 F1500.000
M204 S800
;TYPE:Perimeter
;WIDTH:0.45
G1 F2400.000
G1 X120.300 Y117.848 E0.00281
G1 X120.300 Y104.133 E0.35734
G1 X127.171 Y104.133 E0.17903
G1 X127.235 Y104.359 E0.00610
G1 X127.595 Y105.247 E0.02499
G1 X128.028 Y105.998 E0.02257
G1 X128.547 Y106.686 E0.02246
G1 X129.146 Y107.306 E0.02246
G1 X129.817 Y107.847 E0.02246
```

```
G1 X130.550 Y108.301 E0.02246
G1 X131.333 Y108.661 E0.02246
G1 X132.154 Y108.923 E0.02246
G1 X133.002 Y109.082 E0.02246
G1 X133.842 Y109.133 E0.02194
G1 X141.014 Y109.133 E0.18688
G1 X141.014 Y112.848 E0.09678
G1 X133.842 Y112.848 E0.18688
M73 P87 R9
G1 X133.002 Y112.900 E0.02194
G1 X132.154 Y113.058 E0.02246
G1 X131.333 Y113.320 E0.02246
G1 X130.550 Y113.680 E0.02246
G1 X129.817 Y114.135 E0.02246
G1 X129.146 Y114.675 E0.02246
G1 X128.547 Y115.295 E0.02246
G1 X128.028 Y115.983 E0.02246
G1 X127.598 Y116.730 E0.02246
G1 X127.263 Y117.524 E0.02246
G1 X127.171 Y117.848 E0.00876
G1 X120.468 Y117.848 E0.17466
M204 S1250
G1 X119.882 Y118.266 F9000.000
M204 S800
;TYPE:External perimeter
G1 F1800.000
G1 X119.882 Y103.716 E0.37911
G1 X127.487 Y103.716 E0.19815
G1 X127.631 Y104.223 E0.01373
```

G1 X127.972 Y105.064 E0.02365

G1 X128.377 Y105.767 E0.02116

G1 X128.865 Y106.414 E0.02112

G1 X129.428 Y106.997 E0.02112

G1 X130.059 Y107.505 E0.02112

G1 X130.748 Y107.932 E0.02112

G1 X131.484 Y108.271 E0.02112

G1 X132.257 Y108.517 E0.02112

G1 X133.053 Y108.666 E0.02112

G1 X133.855 Y108.716 E0.02093

G1 X141.432 Y108.716 E0.19743

G1 X141.432 Y113.266 E0.11855

G1 X133.855 Y113.266 E0.19743

G1 X133.053 Y113.315 E0.02093

G1 X132.257 Y113.464 E0.02112

G1 X131.484 Y113.710 E0.02112

G1 X130.748 Y114.049 E0.02112

G1 X130.059 Y114.476 E0.02112

G1 X129.428 Y114.985 E0.02112

G1 X128.865 Y115.567 E0.02112

G1 X128.377 Y116.214 E0.02112

G1 X127.973 Y116.916 E0.02112

G1 X127.658 Y117.663 E0.02112

G1 X127.487 Y118.266 E0.01632

G1 X119.942 Y118.266 E0.19659

M204 S1250

G1 X120.073 Y117.914 F9000.000

G1 E-2.24000 F2400.000

;WIPE_START

```
G1 F7200.000
G1 X119.930 Y115.386 E-0.91200
;WIPE_END
G1 E-0.04800 F2400.000
G1 Z1.150 F9000.000
G1 X140.891 Y111.970
G1 Z0.950
G1 E3.20000 F1500.000
M204 S1000
;TYPE:Top solid infill
;WIDTH:0.405169
G1 F2400.000
G1 X140.302 Y112.559 E0.01937
G1 X139.775 Y112.559 E0.01227
G1 X140.726 Y111.609 E0.03128
G1 X140.726 Y111.081 E0.01227
G1 X139.248 Y112.559 E0.04863
G1 X138.720 Y112.559 E0.01227
G1 X140.726 Y110.554 E0.06598
G1 X140.726 Y110.026 E0.01227
G1 X138.193 Y112.559 E0.08333
G1 X137.665 Y112.559 E0.01227
G1 X140.726 Y109.499 E0.10068
G1 X140.726 Y109.422 E0.00179
G1 X140.275 Y109.422 E0.01048
G1 X137.138 Y112.559 E0.10321
G1 X136.610 Y112.559 E0.01227
G1 X139.748 Y109.422 E0.10321
G1 X139.220 Y109.422 E0.01227
```

```
G1 X136.083 Y112.559 E0.10321
G1 X135.555 Y112.559 E0.01227
G1 X138.693 Y109.422 E0.10321
G1 X138.165 Y109.422 E0.01227
G1 X135.028 Y112.559 E0.10321
G1 X134.500 Y112.559 E0.01227
G1 X137.638 Y109.422 E0.10321
G1 X137.110 Y109.422 E0.01227
G1 X133.973 Y112.559 E0.10321
G1 X133.420 Y112.585 E0.01288
G1 X136.583 Y109.422 E0.10405
G1 X136.056 Y109.422 E0.01227
G1 X132.841 Y112.636 E0.10574
G1 X132.192 Y112.758 E0.01536
G1 X135.528 Y109.422 E0.10974
G1 X135.001 Y109.422 E0.01227
G1 X131.157 Y113.266 E0.12645
M204 S1250
G1 E-2.24000
;WIPE_START
G1 F7200.000
G1 X133.193 Y111.229 E-0.91200
;WIPE_END
G1 E-0.04800 F2400.000
G1 Z1.150 F9000.000
G1 X127.298 Y117.124
G1 Z0.950
G1 E3.20000 F1500.000
M204 S1000
```

```
G1 F2400.000
G1 X126.863 Y117.559 E0.01431
G1 X126.335 Y117.559 E0.01227
G1 X127.401 Y116.494 E0.03505
G1 X127.787 Y115.824 E0.01799
G1 X128.327 Y115.107 E0.02088
G1 X128.951 Y114.462 E0.02088
G1 X129.650 Y113.899 E0.02088
G1 X130.516 Y113.378 E0.02350
G1 X134.473 Y109.422 E0.13015
G1 X133.946 Y109.422 E0.01227
G1 X125.808 Y117.559 E0.26769
G1 X125.280 Y117.559 E0.01227
G1 X133.442 Y109.398 E0.26848
G1 X132.948 Y109.365 E0.01153
G1 X124.753 Y117.559 E0.26956
G1 X124.226 Y117.559 E0.01227
G1 X132.503 Y109.282 E0.27230
G1 X132.061 Y109.196 E0.01047
G1 X123.698 Y117.559 E0.27511
G1 X123.171 Y117.559 E0.01227
G1 X131.661 Y109.069 E0.27930
G1 X131.261 Y108.941 E0.00977
G1 X122.643 Y117.559 E0.28349
G1 X122.116 Y117.559 E0.01227
G1 X130.897 Y108.778 E0.28886
G1 X130.536 Y108.612 E0.00925
G1 X121.588 Y117.559 E0.29433
G1 X121.061 Y117.559 E0.01227
```

```
G1 X130.198 Y108.422 E0.30057
G1 X129.873 Y108.220 E0.00891
G1 X120.588 Y117.504 E0.30541
G1 X120.588 Y116.977 E0.01227
G1 X129.558 Y108.008 E0.29504
G1 X129.266 Y107.772 E0.00873
G1 X120.588 Y116.450 E0.28544
G1 X120.588 Y115.922 E0.01227
G1 X128.973 Y107.537 E0.27583
G1 X128.712 Y107.271 E0.00868
G1 X120.588 Y115.395 E0.26721
G1 X120.588 Y114.867 E0.01227
G1 X128.452 Y107.003 E0.25868
G1 X128.210 Y106.718 E0.00870
G1 X120.588 Y114.340 E0.25070
G1 X120.588 Y113.812 E0.01227
G1 X127.983 Y106.417 E0.24324
G1 X127.761 Y106.112 E0.00878
G1 X120.588 Y113.285 E0.23594
G1 X120.588 Y112.757 E0.01227
G1 X127.568 Y105.777 E0.22960
G1 X127.375 Y105.443 E0.00898
G1 X120.588 Y112.230 E0.22326
G1 X120.588 Y111.702 E0.01227
G1 X127.215 Y105.076 E0.21797
G1 X127.062 Y104.701 E0.00942
G1 X120.588 Y111.175 E0.21296
G1 X120.588 Y110.647 E0.01227
G1 X126.814 Y104.422 E0.20479
```

```
G1 X126.286 Y104.422 E0.01227
G1 X120.588 Y110.120 E0.18744
G1 X120.588 Y109.592 E0.01227
G1 X125.759 Y104.422 E0.17009
G1 X125.231 Y104.422 E0.01227
G1 X120.588 Y109.065 E0.15273
G1 X120.588 Y108.537 E0.01227
G1 X124.704 Y104.422 E0.13538
G1 X124.176 Y104.422 E0.01227
G1 X120.588 Y108.010 E0.11803
G1 X120.588 Y107.482 E0.01227
G1 X123.649 Y104.422 E0.10068
G1 X123.121 Y104.422 E0.01227
G1 X120.588 Y106.955 E0.08333
G1 X120.588 Y106.428 E0.01227
G1 X122.594 Y104.422 E0.06598
G1 X122.067 Y104.422 E0.01227
G1 X120.588 Y105.900 E0.04863
G1 X120.588 Y105.373 E0.01227
G1 X121.539 Y104.422 E0.03128
G1 X121.012 Y104.422 E0.01227
G1 X120.423 Y105.011 E0.01937
M204 S1250
; stop printing object tpu print.STL id:23 copy 0
; printing object tpu print.STL id:21 copy 0
G1 E-2.24000
;WIPE_START
G1 F7200.000
G1 X121.012 Y104.422 E-0.26372
```

```
G1 X121.539 Y104.422 E-0.16703
G1 X120.588 Y105.373 E-0.42582
G1 X120.588 Y105.548 E-0.05543
;WIPE_END
G1 E-0.04800 F2400.000
G1 Z1.150 F9000.000
G1 X120.423 Y97.032
G1 Z0.950
G1 E3.20000 F1500.000
M204 S800
;TYPE:Perimeter
;WIDTH:0.45
G1 F2400.000
G1 X120.293 Y97.032 E0.00338
G1 X120.293 Y83.318 E0.35734
G1 X127.164 Y83.318 E0.17903
G1 X127.228 Y83.543 E0.00610
G1 X127.589 Y84.432 E0.02499
G1 X128.021 Y85.183 E0.02257
G1 X128.540 Y85.871 E0.02246
G1 X129.139 Y86.490 E0.02246
G1 X129.811 Y87.031 E0.02246
G1 X130.543 Y87.485 E0.02246
G1 X131.326 Y87.846 E0.02246
G1 X132.147 Y88.107 E0.02246
G1 X132.995 Y88.266 E0.02246
G1 X133.835 Y88.318 E0.02194
G1 X141.007 Y88.318 E0.18688
G1 X141.007 Y92.032 E0.09678
```

```
G1 X133.835 Y92.032 E0.18688
G1 X132.995 Y92.084 E0.02194
G1 X132.147 Y92.243 E0.02246
G1 X131.326 Y92.504 E0.02246
G1 X130.543 Y92.865 E0.02246
G1 X129.811 Y93.319 E0.02246
G1 X129.139 Y93.860 E0.02246
G1 X128.540 Y94.479 E0.02246
G1 X128.021 Y95.168 E0.02246
G1 X127.591 Y95.915 E0.02246
G1 X127.256 Y96.709 E0.02246
G1 X127.164 Y97.032 E0.00876
G1 X120.483 Y97.032 E0.17409
M204 S1250
G1 X119.875 Y97.450 F9000.000
M204 S800
;TYPE:External perimeter
G1 F1800.000
G1 X119.875 Y82.900 E0.37911
G1 X127.480 Y82.900 E0.19815
G1 X127.624 Y83.407 E0.01373
G1 X127.965 Y84.248 E0.02365
G1 X128.370 Y84.952 E0.02116
G1 X128.858 Y85.599 E0.02112
G1 X129.422 Y86.181 E0.02112
G1 X130.053 Y86.690 E0.02112
G1 X130.741 Y87.117 E0.02112
G1 X131.477 Y87.456 E0.02112
G1 X132.250 Y87.702 E0.02112
```

```
G1 X133.046 Y87.851 E0.02112
G1 X133.848 Y87.900 E0.02093
G1 X141.425 Y87.900 E0.19743
G1 X141.425 Y92.450 E0.11855
G1 X133.848 Y92.450 E0.19743
G1 X133.046 Y92.500 E0.02093
G1 X132.250 Y92.649 E0.02112
G1 X131.477 Y92.895 E0.02112
G1 X130.741 Y93.234 E0.02112
G1 X130.053 Y93.661 E0.02112
G1 X129.422 Y94.169 E0.02112
G1 X128.858 Y94.751 E0.02112
G1 X128.370 Y95.398 E0.02112
G1 X127.966 Y96.101 E0.02112
G1 X127.651 Y96.847 E0.02112
G1 X127.480 Y97.450 E0.01632
G1 X119.935 Y97.450 E0.19659
M204 S1250
G1 X120.066 Y97.099 F9000.000
G1 E-2.24000 F2400.000
;WIPE_START
G1 F7200.000
G1 X119.923 Y94.570 E-0.91200
;WIPE_END
G1 E-0.04800 F2400.000
G1 Z1.150 F9000.000
G1 X140.885 Y91.155
G1 Z0.950
G1 E3.20000 F1500.000
```

```
M204 S1000
;TYPE:Top solid infill
;WIDTH:0.405169
G1 F2400.000
G1 X140.296 Y91.744 E0.01937
G1 X139.768 Y91.744 E0.01227
G1 X140.719 Y90.793 E0.03128
G1 X140.719 Y90.266 E0.01227
G1 X139.241 Y91.744 E0.04863
G1 X138.713 Y91.744 E0.01227
G1 X140.719 Y89.738 E0.06598
M73 P87 R8
G1 X140.719 Y89.211 E0.01227
G1 X138.186 Y91.744 E0.08333
G1 X137.658 Y91.744 E0.01227
G1 X140.719 Y88.683 E0.10068
G1 X140.719 Y88.606 E0.00179
G1 X140.268 Y88.606 E0.01048
G1 X137.131 Y91.744 E0.10321
G1 X136.603 Y91.744 E0.01227
G1 X139.741 Y88.606 E0.10321
G1 X139.214 Y88.606 E0.01227
G1 X136.076 Y91.744 E0.10321
G1 X135.548 Y91.744 E0.01227
G1 X138.686 Y88.606 E0.10321
G1 X138.159 Y88.606 E0.01227
G1 X135.021 Y91.744 E0.10321
G1 X134.493 Y91.744 E0.01227
G1 X137.631 Y88.606 E0.10321
```

```
G1 X137.104 Y88.606 E0.01227
G1 X133.966 Y91.744 E0.10321
G1 X133.413 Y91.769 E0.01288
G1 X136.576 Y88.606 E0.10405
G1 X136.049 Y88.606 E0.01227
G1 X132.834 Y91.821 E0.10574
G1 X132.185 Y91.942 E0.01536
G1 X135.521 Y88.606 E0.10974
G1 X134.994 Y88.606 E0.01227
G1 X131.150 Y92.450 E0.12645
M204 S1250
G1 E-2.24000
;WIPE_START
G1 F7200.000
G1 X133.186 Y90.414 E-0.91200
;WIPE_END
G1 E-0.04800 F2400.000
G1 Z1.150 F9000.000
G1 X127.291 Y96.309
G1 Z0.950
G1 E3.20000 F1500.000
M204 S1000
G1 F2400.000
G1 X126.856 Y96.744 E0.01431
G1 X126.329 Y96.744 E0.01227
G1 X127.394 Y95.678 E0.03505
G1 X127.780 Y95.008 E0.01799
G1 X128.320 Y94.291 E0.02088
G1 X128.945 Y93.647 E0.02088
```

```
G1 X129.644 Y93.083 E0.02088
G1 X130.510 Y92.563 E0.02350
G1 X134.466 Y88.606 E0.13015
G1 X133.939 Y88.606 E0.01227
G1 X125.801 Y96.744 E0.26769
G1 X125.274 Y96.744 E0.01227
G1 X133.435 Y88.582 E0.26848
G1 X132.941 Y88.549 E0.01153
G1 X124.746 Y96.744 E0.26956
G1 X124.219 Y96.744 E0.01227
G1 X132.497 Y88.466 E0.27230
G1 X132.055 Y88.380 E0.01047
G1 X123.691 Y96.744 E0.27511
G1 X123.164 Y96.744 E0.01227
G1 X131.655 Y88.253 E0.27930
G1 X131.255 Y88.126 E0.00977
G1 X122.636 Y96.744 E0.28349
G1 X122.109 Y96.744 E0.01227
G1 X130.890 Y87.963 E0.28886
G1 X130.529 Y87.796 E0.00925
G1 X121.581 Y96.744 E0.29433
G1 X121.054 Y96.744 E0.01227
G1 X130.191 Y87.606 E0.30057
G1 X129.866 Y87.405 E0.00891
G1 X120.581 Y96.689 E0.30541
G1 X120.581 Y96.161 E0.01227
G1 X129.551 Y87.192 E0.29504
G1 X129.259 Y86.957 E0.00873
G1 X120.581 Y95.634 E0.28544
```

```
G1 X120.581 Y95.107 E0.01227
G1 X128.967 Y86.721 E0.27583
G1 X128.705 Y86.456 E0.00868
G1 X120.581 Y94.579 E0.26721
G1 X120.581 Y94.052 E0.01227
G1 X128.445 Y86.188 E0.25868
G1 X128.203 Y85.903 E0.00870
G1 X120.581 Y93.524 E0.25070
G1 X120.581 Y92.997 E0.01227
G1 X127.976 Y85.602 E0.24324
G1 X127.754 Y85.297 E0.00878
G1 X120.581 Y92.469 E0.23594
G1 X120.581 Y91.942 E0.01227
G1 X127.561 Y84.962 E0.22960
G1 X127.368 Y84.627 E0.00898
G1 X120.581 Y91.414 E0.22326
G1 X120.581 Y90.887 E0.01227
G1 X127.208 Y84.260 E0.21797
G1 X127.056 Y83.885 E0.00942
G1 X120.581 Y90.359 E0.21296
G1 X120.581 Y89.832 E0.01227
G1 X126.807 Y83.606 E0.20479
G1 X126.279 Y83.606 E0.01227
G1 X120.581 Y89.304 E0.18744
G1 X120.581 Y88.777 E0.01227
G1 X125.752 Y83.606 E0.17009
G1 X125.225 Y83.606 E0.01227
G1 X120.581 Y88.249 E0.15273
G1 X120.581 Y87.722 E0.01227
```

```
G1 X124.697 Y83.606 E0.13538
G1 X124.170 Y83.606 E0.01227
G1 X120.581 Y87.194 E0.11803
G1 X120.581 Y86.667 E0.01227
G1 X123.642 Y83.606 E0.10068
G1 X123.115 Y83.606 E0.01227
G1 X120.581 Y86.139 E0.08333
G1 X120.581 Y85.612 E0.01227
G1 X122.587 Y83.606 E0.06598
G1 X122.060 Y83.606 E0.01227
G1 X120.581 Y85.085 E0.04863
G1 X120.581 Y84.557 E0.01227
G1 X121.532 Y83.606 E0.03128
G1 X121.005 Y83.606 E0.01227
G1 X120.416 Y84.195 E0.01937
M204 S1250
; stop printing object tpu print.STL id:21 copy 0
; printing object Petg print.STL id:20 copy 0
; stop printing object Petg print.STL id:20 copy 0
; printing object Petg print.STL id:22 copy 0
; stop printing object Petg print.STL id:22 copy 0
; printing object Petg print.STL id:26 copy 0
; stop printing object Petg print.STL id:26 copy 0
; printing object Petg print.STL id:0 copy 0
; stop printing object Petg print.STL id:0 copy 0
; printing object tpu print.STL id:1 copy 0
G1 E-2.24000
;WIPE_START
G1 F7200.000
```

```
G1 X121.005 Y83.606 E-0.26372
G1 X121.532 Y83.606 E-0.16703
G1 X120.581 Y84.557 E-0.42582
G1 X120.581 Y84.732 E-0.05543
;WIPE_END
G1 E-0.04800 F2400.000
G1 Z1.150 F9000.000
G1 X90.357 Y88.143
G1 Z0.950
G1 E3.20000 F1500.000
M204 S800
;TYPE:Perimeter
;WIDTH:0.45
G1 F2400.000
G1 X90.357 Y91.857 E0.09678
G1 X83.185 Y91.857 E0.18688
G1 X82.344 Y91.909 E0.02194
G1 X81.497 Y92.068 E0.02246
G1 X80.676 Y92.329 E0.02246
G1 X79.893 Y92.690 E0.02246
G1 X79.160 Y93.144 E0.02246
G1 X78.489 Y93.685 E0.02246
G1 X77.890 Y94.304 E0.02246
G1 X77.371 Y94.993 E0.02246
G1 X76.941 Y95.739 E0.02246
G1 X76.606 Y96.534 E0.02246
G1 X76.514 Y96.857 E0.00876
G1 X69.643 Y96.857 E0.17903
G1 X69.643 Y83.143 E0.35734
```

```
G1 X76.514 Y83.143 E0.17903
G1 X76.578 Y83.368 E0.00610
G1 X76.938 Y84.257 E0.02499
G1 X77.371 Y85.007 E0.02257
G1 X77.890 Y85.696 E0.02246
G1 X78.489 Y86.315 E0.02246
G1 X79.160 Y86.856 E0.02246
G1 X79.893 Y87.310 E0.02246
G1 X80.676 Y87.671 E0.02246
G1 X81.497 Y87.932 E0.02246
G1 X82.344 Y88.091 E0.02246
G1 X83.185 Y88.143 E0.02194
G1 X90.297 Y88.143 E0.18531
M204 S1250
G1 X90.775 Y87.725 F9000.000
M204 S800
;TYPE:External perimeter
G1 F1800.000
G1 X90.775 Y92.275 E0.11855
G1 X83.198 Y92.275 E0.19743
G1 X82.396 Y92.325 E0.02093
G1 X81.599 Y92.474 E0.02112
G1 X80.827 Y92.719 E0.02112
G1 X80.091 Y93.059 E0.02112
G1 X79.402 Y93.486 E0.02112
G1 X78.771 Y93.994 E0.02112
G1 X78.208 Y94.576 E0.02112
G1 X77.720 Y95.223 E0.02112
G1 X77.315 Y95.926 E0.02112
```

```
G1 X77.000 Y96.672 E0.02112
G1 X76.830 Y97.275 E0.01632
G1 X69.225 Y97.275 E0.19815
G1 X69.225 Y82.725 E0.37911
G1 X76.830 Y82.725 E0.19815
G1 X76.973 Y83.232 E0.01373
G1 X77.315 Y84.073 E0.02365
G1 X77.720 Y84.777 E0.02116
G1 X78.208 Y85.424 E0.02112
G1 X78.771 Y86.006 E0.02112
G1 X79.402 Y86.514 E0.02112
G1 X80.091 Y86.941 E0.02112
G1 X80.827 Y87.281 E0.02112
G1 X81.599 Y87.526 E0.02112
G1 X82.396 Y87.675 E0.02112
G1 X83.198 Y87.725 E0.02093
G1 X90.715 Y87.725 E0.19586
M204 S1250
G1 X90.574 Y88.071 F9000.000
G1 E-2.24000 F2400.000
;WIPE_START
G1 F7200.000
G1 X90.753 Y90.605 E-0.91200
;WIPE_END
G1 E-0.04800 F2400.000
G1 Z1.150 F9000.000
G1 X90.234 Y90.980
G1 Z0.950
G1 E3.20000 F1500.000
```

```
M204 S1000
;TYPE:Top solid infill
;WIDTH:0.405169
G1 F2400.000
G1 X89.645 Y91.569 E0.01937
G1 X89.118 Y91.569 E0.01227
G1 X90.069 Y90.618 E0.03128
G1 X90.069 Y90.090 E0.01227
G1 X88.590 Y91.569 E0.04863
G1 X88.063 Y91.569 E0.01227
G1 X90.069 Y89.563 E0.06598
G1 X90.069 Y89.035 E0.01227
G1 X87.535 Y91.569 E0.08333
G1 X87.008 Y91.569 E0.01227
M73 P88 R8
G1 X90.069 Y88.508 E0.10068
G1 X90.069 Y88.431 E0.00179
G1 X89.618 Y88.431 E0.01048
G1 X86.481 Y91.569 E0.10321
G1 X85.953 Y91.569 E0.01227
G1 X89.091 Y88.431 E0.10321
G1 X88.563 Y88.431 E0.01227
G1 X85.426 Y91.569 E0.10321
G1 X84.898 Y91.569 E0.01227
G1 X88.036 Y88.431 E0.10321
G1 X87.508 Y88.431 E0.01227
G1 X84.371 Y91.569 E0.10321
G1 X83.843 Y91.569 E0.01227
G1 X86.981 Y88.431 E0.10321
```

```
G1 X86.453 Y88.431 E0.01227
G1 X83.316 Y91.569 E0.10321
G1 X82.763 Y91.594 E0.01288
G1 X85.926 Y88.431 E0.10405
G1 X85.398 Y88.431 E0.01227
G1 X82.184 Y91.646 E0.10574
G1 X81.535 Y91.767 E0.01536
G1 X84.871 Y88.431 E0.10974
G1 X84.344 Y88.431 E0.01227
G1 X80.499 Y92.275 E0.12645
M204 S1250
G1 E-2.24000
;WIPE_START
G1 F7200.000
G1 X82.536 Y90.239 E-0.91200
;WIPE_END
G1 E-0.04800 F2400.000
G1 Z1.150 F9000.000
G1 X76.641 Y96.134
G1 Z0.950
G1 E3.20000 F1500.000
M204 S1000
G1 F2400.000
G1 X76.206 Y96.569 E0.01431
G1 X75.678 Y96.569 E0.01227
G1 X76.744 Y95.503 E0.03505
G1 X77.130 Y94.833 E0.01799
G1 X77.670 Y94.116 E0.02088
G1 X78.294 Y93.472 E0.02088
```

```
G1 X78.993 Y92.908 E0.02088
G1 X79.859 Y92.388 E0.02350
G1 X83.816 Y88.431 E0.13015
G1 X83.289 Y88.431 E0.01227
G1 X75.151 Y96.569 E0.26769
G1 X74.623 Y96.569 E0.01227
G1 X82.785 Y88.407 E0.26848
G1 X82.291 Y88.374 E0.01153
G1 X74.096 Y96.569 E0.26956
G1 X73.568 Y96.569 E0.01227
G1 X81.846 Y88.291 E0.27230
G1 X81.404 Y88.205 E0.01047
G1 X73.041 Y96.569 E0.27511
G1 X72.514 Y96.569 E0.01227
G1 X81.004 Y88.078 E0.27930
G1 X80.604 Y87.951 E0.00977
G1 X71.986 Y96.569 E0.28349
G1 X71.459 Y96.569 E0.01227
G1 X80.240 Y87.787 E0.28886
G1 X79.879 Y87.621 E0.00925
G1 X70.931 Y96.569 E0.29433
G1 X70.404 Y96.569 E0.01227
G1 X79.541 Y87.431 E0.30057
G1 X79.216 Y87.229 E0.00891
G1 X69.931 Y96.514 E0.30541
G1 X69.931 Y95.986 E0.01227
G1 X78.901 Y87.017 E0.29504
G1 X78.608 Y86.782 E0.00873
G1 X69.931 Y95.459 E0.28544
```

```
G1 X69.931 Y94.931 E0.01227
G1 X78.316 Y86.546 E0.27583
G1 X78.054 Y86.281 E0.00868
G1 X69.931 Y94.404 E0.26721
G1 X69.931 Y93.876 E0.01227
G1 X77.795 Y86.013 E0.25868
G1 X77.553 Y85.728 E0.00870
G1 X69.931 Y93.349 E0.25070
G1 X69.931 Y92.822 E0.01227
G1 X77.326 Y85.427 E0.24324
G1 X77.104 Y85.122 E0.00878
G1 X69.931 Y92.294 E0.23594
G1 X69.931 Y91.767 E0.01227
G1 X76.911 Y84.787 E0.22960
G1 X76.718 Y84.452 E0.00898
G1 X69.931 Y91.239 E0.22326
G1 X69.931 Y90.712 E0.01227
G1 X76.558 Y84.085 E0.21797
G1 X76.405 Y83.710 E0.00942
G1 X69.931 Y90.184 E0.21296
G1 X69.931 Y89.657 E0.01227
G1 X76.157 Y83.431 E0.20479
G1 X75.629 Y83.431 E0.01227
G1 X69.931 Y89.129 E0.18744
G1 X69.931 Y88.602 E0.01227
G1 X75.102 Y83.431 E0.17009
G1 X74.574 Y83.431 E0.01227
G1 X69.931 Y88.074 E0.15273
G1 X69.931 Y87.547 E0.01227
```

```
G1 X74.047 Y83.431 E0.13538
G1 X73.519 Y83.431 E0.01227
G1 X69.931 Y87.019 E0.11803
G1 X69.931 Y86.492 E0.01227
G1 X72.992 Y83.431 E0.10068
G1 X72.464 Y83.431 E0.01227
G1 X69.931 Y85.964 E0.08333
G1 X69.931 Y85.437 E0.01227
G1 X71.937 Y83.431 E0.06598
G1 X71.409 Y83.431 E0.01227
G1 X69.931 Y84.909 E0.04863
G1 X69.931 Y84.382 E0.01227
G1 X70.882 Y83.431 E0.03128
G1 X70.355 Y83.431 E0.01227
G1 X69.766 Y84.020 E0.01937
M204 S1250
; stop printing object tpu print.STL id:1 copy 0
; printing object tpu print.STL id:5 copy 0
G1 E-2.24000
;WIPE_START
G1 F7200.000
G1 X70.355 Y83.431 E-0.26372
G1 X70.882 Y83.431 E-0.16703
G1 X69.931 Y84.382 E-0.42582
G1 X69.931 Y84.557 E-0.05543
;WIPE_END
G1 E-0.04800 F2400.000
G1 Z1.150 F9000.000
G1 X69.766 Y74.665
```

```
G1 Z0.950
G1 E3.20000 F1500.000
M204 S800
;TYPE:Perimeter
;WIDTH:0.45
G1 F2400.000
G1 X69.694 Y74.665 E0.00187
G1 X69.694 Y60.950 E0.35734
G1 X76.565 Y60.950 E0.17903
G1 X76.629 Y61.176 E0.00610
G1 X76.989 Y62.064 E0.02499
G1 X77.422 Y62.815 E0.02257
G1 X77.941 Y63.503 E0.02246
G1 X78.540 Y64.123 E0.02246
G1 X79.211 Y64.664 E0.02246
G1 X79.944 Y65.118 E0.02246
G1 X80.727 Y65.478 E0.02246
G1 X81.548 Y65.740 E0.02246
G1 X82.396 Y65.899 E0.02246
G1 X83.236 Y65.950 E0.02194
G1 X90.408 Y65.950 E0.18688
G1 X90.408 Y69.665 E0.09678
G1 X83.236 Y69.665 E0.18688
G1 X82.396 Y69.717 E0.02194
G1 X81.548 Y69.875 E0.02246
G1 X80.727 Y70.137 E0.02246
G1 X79.944 Y70.497 E0.02246
G1 X79.211 Y70.952 E0.02246
G1 X78.540 Y71.493 E0.02246
```

```
G1 X77.941 Y72.112 E0.02246
G1 X77.422 Y72.800 E0.02246
G1 X76.992 Y73.547 E0.02246
G1 X76.657 Y74.341 E0.02246
G1 X76.565 Y74.665 E0.00876
G1 X69.826 Y74.665 E0.17560
M204 S1250
G1 X69.276 Y75.083 F9000.000
M204 S800
;TYPE:External perimeter
G1 F1800.000
G1 X69.276 Y60.533 E0.37911
G1 X76.881 Y60.533 E0.19815
G1 X77.025 Y61.040 E0.01373
G1 X77.366 Y61.881 E0.02365
G1 X77.771 Y62.584 E0.02116
G1 X78.259 Y63.231 E0.02112
G1 X78.823 Y63.814 E0.02112
G1 X79.454 Y64.322 E0.02112
G1 X80.142 Y64.749 E0.02112
G1 X80.878 Y65.088 E0.02112
G1 X81.651 Y65.334 E0.02112
G1 X82.447 Y65.483 E0.02112
G1 X83.249 Y65.533 E0.02093
G1 X90.826 Y65.533 E0.19743
G1 X90.826 Y70.083 E0.11855
G1 X83.249 Y70.083 E0.19743
G1 X82.447 Y70.132 E0.02093
G1 X81.651 Y70.281 E0.02112
```

```
G1 X80.878 Y70.527 E0.02112
G1 X80.142 Y70.866 E0.02112
G1 X79.454 Y71.293 E0.02112
G1 X78.823 Y71.802 E0.02112
G1 X78.259 Y72.384 E0.02112
G1 X77.771 Y73.031 E0.02112
G1 X77.367 Y73.733 E0.02112
G1 X77.052 Y74.480 E0.02112
G1 X76.881 Y75.083 E0.01632
G1 X69.336 Y75.083 E0.19659
M204 S1250
G1 X69.467 Y74.731 F9000.000
G1 E-2.24000 F2400.000
;WIPE_START
G1 F7200.000
G1 X69.324 Y72.203 E-0.91200
;WIPE_END
G1 E-0.04800 F2400.000
G1 Z1.150 F9000.000
G1 X90.286 Y68.788
G1 Z0.950
G1 E3.20000 F1500.000
M204 S1000
;TYPE:Top solid infill
;WIDTH:0.405169
G1 F2400.000
G1 X89.697 Y69.376 E0.01937
G1 X89.169 Y69.376 E0.01227
G1 X90.120 Y68.426 E0.03128
```

```
G1 X90.120 Y67.898 E0.01227
G1 X88.642 Y69.376 E0.04863
G1 X88.114 Y69.376 E0.01227
G1 X90.120 Y67.371 E0.06598
G1 X90.120 Y66.843 E0.01227
G1 X87.587 Y69.376 E0.08333
G1 X87.059 Y69.376 E0.01227
G1 X90.120 Y66.316 E0.10068
G1 X90.120 Y66.239 E0.00179
G1 X89.669 Y66.239 E0.01048
G1 X86.532 Y69.376 E0.10321
G1 X86.004 Y69.376 E0.01227
G1 X89.142 Y66.239 E0.10321
G1 X88.614 Y66.239 E0.01227
G1 X85.477 Y69.376 E0.10321
G1 X84.949 Y69.376 E0.01227
G1 X88.087 Y66.239 E0.10321
G1 X87.559 Y66.239 E0.01227
G1 X84.422 Y69.376 E0.10321
G1 X83.894 Y69.376 E0.01227
G1 X87.032 Y66.239 E0.10321
G1 X86.505 Y66.239 E0.01227
G1 X83.367 Y69.376 E0.10321
G1 X82.814 Y69.402 E0.01288
G1 X85.977 Y66.239 E0.10405
G1 X85.450 Y66.239 E0.01227
G1 X82.235 Y69.453 E0.10574
G1 X81.586 Y69.575 E0.01536
G1 X84.922 Y66.239 E0.10974
```

```
G1 X84.395 Y66.239 E0.01227
G1 X80.551 Y70.083 E0.12645
M204 S1250
G1 E-2.24000
;WIPE_START
G1 F7200.000
G1 X82.587 Y68.046 E-0.91200
;WIPE_END
G1 E-0.04800 F2400.000
G1 Z1.150 F9000.000
G1 X76.692 Y73.941
G1 Z0.950
G1 E3.20000 F1500.000
M204 S1000
G1 F2400.000
G1 X76.257 Y74.376 E0.01431
G1 X75.729 Y74.376 E0.01227
G1 X76.795 Y73.311 E0.03505
G1 X77.181 Y72.641 E0.01799
G1 X77.721 Y71.924 E0.02088
G1 X78.345 Y71.279 E0.02088
G1 X79.044 Y70.716 E0.02088
G1 X79.911 Y70.195 E0.02350
G1 X83.867 Y66.239 E0.13015
G1 X83.340 Y66.239 E0.01227
G1 X75.202 Y74.376 E0.26769
G1 X74.675 Y74.376 E0.01227
G1 X82.836 Y66.215 E0.26848
G1 X82.342 Y66.182 E0.01153
```

```
G1 X74.147 Y74.376 E0.26956
G1 X73.620 Y74.376 E0.01227
G1 X81.897 Y66.099 E0.27230
G1 X81.456 Y66.013 E0.01047
G1 X73.092 Y74.376 E0.27511
G1 X72.565 Y74.376 E0.01227
G1 X81.055 Y65.886 E0.27930
G1 X80.655 Y65.758 E0.00977
G1 X72.037 Y74.376 E0.28349
G1 X71.510 Y74.376 E0.01227
G1 X80.291 Y65.595 E0.28886
G1 X79.930 Y65.429 E0.00925
G1 X70.982 Y74.376 E0.29433
G1 X70.455 Y74.376 E0.01227
G1 X79.592 Y65.239 E0.30057
G1 X79.267 Y65.037 E0.00891
G1 X69.982 Y74.321 E0.30541
G1 X69.982 Y73.794 E0.01227
G1 X78.952 Y64.825 E0.29504
G1 X78.660 Y64.589 E0.00873
G1 X69.982 Y73.267 E0.28544
G1 X69.982 Y72.739 E0.01227
G1 X78.368 Y64.354 E0.27583
G1 X78.106 Y64.088 E0.00868
G1 X69.982 Y72.212 E0.26721
G1 X69.982 Y71.684 E0.01227
G1 X77.846 Y63.820 E0.25868
G1 X77.604 Y63.535 E0.00870
G1 X69.982 Y71.157 E0.25070
```

```
G1 X69.982 Y70.629 E0.01227
G1 X77.377 Y63.235 E0.24324
G1 X77.155 Y62.929 E0.00878
G1 X69.982 Y70.102 E0.23594
G1 X69.982 Y69.574 E0.01227
G1 X76.962 Y62.594 E0.22960
G1 X76.769 Y62.260 E0.00898
G1 X69.982 Y69.047 E0.22326
G1 X69.982 Y68.519 E0.01227
G1 X76.609 Y61.893 E0.21797
G1 X76.456 Y61.518 E0.00942
G1 X69.982 Y67.992 E0.21296
G1 X69.982 Y67.464 E0.01227
G1 X76.208 Y61.239 E0.20479
G1 X75.680 Y61.239 E0.01227
G1 X69.982 Y66.937 E0.18744
G1 X69.982 Y66.409 E0.01227
G1 X75.153 Y61.239 E0.17009
G1 X74.625 Y61.239 E0.01227
G1 X69.982 Y65.882 E0.15273
G1 X69.982 Y65.354 E0.01227
G1 X74.098 Y61.239 E0.13538
G1 X73.570 Y61.239 E0.01227
G1 X69.982 Y64.827 E0.11803
G1 X69.982 Y64.300 E0.01227
G1 X73.043 Y61.239 E0.10068
G1 X72.516 Y61.239 E0.01227
G1 X69.982 Y63.772 E0.08333
G1 X69.982 Y63.245 E0.01227
```

```
G1 X71.988 Y61.239 E0.06598
G1 X71.461 Y61.239 E0.01227
G1 X69.982 Y62.717 E0.04863
G1 X69.982 Y62.190 E0.01227
G1 X70.933 Y61.239 E0.03128
G1 X70.406 Y61.239 E0.01227
G1 X69.817 Y61.828 E0.01937
M204 S1250
; stop printing object tpu print.STL id:5 copy 0
; printing object tpu print.STL id:9 copy 0
G1 E-2.24000
;WIPE_START
G1 F7200.000
G1 X70.406 Y61.239 E-0.26372
G1 X70.933 Y61.239 E-0.16703
G1 X69.982 Y62.190 E-0.42582
G1 X69.982 Y62.365 E-0.05543
;WIPE_END
G1 E-0.04800 F2400.000
G1 Z1.150 F9000.000
G1 X69.817 Y54.716
G1 Z0.950
G1 E3.20000 F1500.000
M204 S800
;TYPE:Perimeter
;WIDTH:0.45
G1 F2400.000
G1 X69.686 Y54.716 E0.00340
G1 X69.686 Y41.002 E0.35734
```

```
G1 X76.558 Y41.002 E0.17903
G1 X76.621 Y41.227 E0.00610
G1 X76.982 Y42.115 E0.02499
G1 X77.414 Y42.866 E0.02257
G1 X77.933 Y43.555 E0.02246
G1 X78.533 Y44.174 E0.02246
G1 X79.204 Y44.715 E0.02246
G1 X79.937 Y45.169 E0.02246
G1 X80.720 Y45.530 E0.02246
G1 X81.541 Y45.791 E0.02246
G1 X82.388 Y45.950 E0.02246
G1 X83.229 Y46.002 E0.02194
G1 X90.401 Y46.002 E0.18688
G1 X90.401 Y49.716 E0.09678
G1 X83.229 Y49.716 E0.18688
G1 X82.388 Y49.768 E0.02194
G1 X81.541 Y49.926 E0.02246
G1 X80.720 Y50.188 E0.02246
G1 X79.937 Y50.549 E0.02246
G1 X79.204 Y51.003 E0.02246
G1 X78.533 Y51.544 E0.02246
G1 X77.933 Y52.163 E0.02246
G1 X77.414 Y52.851 E0.02246
G1 X76.984 Y53.598 E0.02246
G1 X76.649 Y54.392 E0.02246
G1 X76.558 Y54.716 E0.00876
G1 X69.877 Y54.716 E0.17407
M204 S1250
G1 X69.269 Y55.134 F9000.000
```

```
M204 S800
;TYPE:External perimeter
G1 F1800.000
G1 X69.269 Y40.584 E0.37911
G1 X76.874 Y40.584 E0.19815
G1 X77.017 Y41.091 E0.01373
G1 X77.358 Y41.932 E0.02365
G1 X77.764 Y42.635 E0.02116
G1 X78.251 Y43.282 E0.02112
G1 X78.815 Y43.865 E0.02112
G1 X79.446 Y44.373 E0.02112
G1 X80.135 Y44.800 E0.02112
G1 X80.871 Y45.139 E0.02112
G1 X81.643 Y45.385 E0.02112
G1 X82.440 Y45.534 E0.02112
G1 X83.242 Y45.584 E0.02093
G1 X90.819 Y45.584 E0.19743
G1 X90.819 Y50.134 E0.11855
G1 X83.242 Y50.134 E0.19743
G1 X82.440 Y50.183 E0.02093
G1 X81.643 Y50.332 E0.02112
G1 X80.871 Y50.578 E0.02112
G1 X80.135 Y50.917 E0.02112
G1 X79.446 Y51.344 E0.02112
G1 X78.815 Y51.853 E0.02112
G1 X78.251 Y52.435 E0.02112
G1 X77.764 Y53.082 E0.02112
G1 X77.359 Y53.784 E0.02112
G1 X77.044 Y54.531 E0.02112
```

```
G1 X76.874 Y55.134 E0.01632
G1 X69.329 Y55.134 E0.19659
M204 S1250
G1 X69.460 Y54.782 F9000.000
G1 E-2.24000 F2400.000
;WIPE_START
G1 F7200.000
G1 X69.317 Y52.254 E-0.91200
;WIPE_END
G1 E-0.04800 F2400.000
G1 Z1.150 F9000.000
G1 X90.278 Y48.839
G1 Z0.950
G1 E3.20000 F1500.000
M204 S1000
;TYPE:Top solid infill
;WIDTH:0.405169
G1 F2400.000
G1 X89.689 Y49.428 E0.01937
G1 X89.162 Y49.428 E0.01227
G1 X90.112 Y48.477 E0.03128
G1 X90.112 Y47.949 E0.01227
G1 X88.634 Y49.428 E0.04863
G1 X88.107 Y49.428 E0.01227
G1 X90.112 Y47.422 E0.06598
G1 X90.112 Y46.894 E0.01227
G1 X87.579 Y49.428 E0.08333
G1 X87.052 Y49.428 E0.01227
G1 X90.112 Y46.367 E0.10068
```

```
G1 X90.112 Y46.290 E0.00179
G1 X89.662 Y46.290 E0.01048
G1 X86.524 Y49.428 E0.10321
G1 X85.997 Y49.428 E0.01227
G1 X89.134 Y46.290 E0.10321
G1 X88.607 Y46.290 E0.01227
G1 X85.469 Y49.428 E0.10321
G1 X84.942 Y49.428 E0.01227
G1 X88.079 Y46.290 E0.10321
G1 X87.552 Y46.290 E0.01227
G1 X84.414 Y49.428 E0.10321
G1 X83.887 Y49.428 E0.01227
G1 X87.025 Y46.290 E0.10321
G1 X86.497 Y46.290 E0.01227
G1 X83.359 Y49.428 E0.10321
G1 X82.806 Y49.453 E0.01288
G1 X85.970 Y46.290 E0.10405
G1 X85.442 Y46.290 E0.01227
G1 X82.227 Y49.505 E0.10574
G1 X81.579 Y49.626 E0.01536
G1 X84.915 Y46.290 E0.10974
G1 X84.387 Y46.290 E0.01227
G1 X80.543 Y50.134 E0.12645
M204 S1250
G1 E-2.24000
;WIPE_START
G1 F7200.000
G1 X82.580 Y48.097 E-0.91200
;WIPE_END
```

```
G1 E-0.04800 F2400.000
G1 Z1.150 F9000.000
G1 X76.685 Y53.992
G1 Z0.950
G1 E3.20000 F1500.000
M204 S1000
G1 F2400.000
G1 X76.249 Y54.428 E0.01431
G1 X75.722 Y54.428 E0.01227
G1 X76.787 Y53.362 E0.03505
G1 X77.173 Y52.692 E0.01799
G1 X77.714 Y51.975 E0.02088
G1 X78.338 Y51.330 E0.02088
G1 X79.037 Y50.767 E0.02088
G1 X79.903 Y50.247 E0.02350
G1 X83.860 Y46.290 E0.13015
G1 X83.332 Y46.290 E0.01227
G1 X75.195 Y54.428 E0.26769
G1 X74.667 Y54.428 E0.01227
G1 X82.829 Y46.266 E0.26848
G1 X82.334 Y46.233 E0.01153
G1 X74.140 Y54.428 E0.26956
M73 P89 R8
G1 X73.612 Y54.428 E0.01227
G1 X81.890 Y46.150 E0.27230
G1 X81.448 Y46.064 E0.01047
M73 P89 R7
G1 X73.085 Y54.428 E0.27511
G1 X72.557 Y54.428 E0.01227
```

G1 X81.048 Y45.937 E0.27930

G1 X80.648 Y45.809 E0.00977

G1 X72.030 Y54.428 E0.28349

G1 X71.502 Y54.428 E0.01227

G1 X80.284 Y45.646 E0.28886

G1 X79.922 Y45.480 E0.00925

G1 X70.975 Y54.428 E0.29433

G1 X70.447 Y54.428 E0.01227

G1 X79.585 Y45.290 E0.30057

G1 X79.259 Y45.088 E0.00891

G1 X69.975 Y54.373 E0.30541

G1 X69.975 Y53.845 E0.01227

G1 X78.944 Y44.876 E0.29504

G1 X78.652 Y44.640 E0.00873

G1 X69.975 Y53.318 E0.28544

G1 X69.975 Y52.790 E0.01227

G1 X78.360 Y44.405 E0.27583

G1 X78.098 Y44.139 E0.00868

G1 X69.975 Y52.263 E0.26721

G1 X69.975 Y51.735 E0.01227

G1 X77.839 Y43.871 E0.25868

G1 X77.596 Y43.586 E0.00870

G1 X69.975 Y51.208 E0.25070

G1 X69.975 Y50.680 E0.01227

G1 X77.369 Y43.286 E0.24324

G1 X77.147 Y42.980 E0.00878

G1 X69.975 Y50.153 E0.23594

G1 X69.975 Y49.625 E0.01227

G1 X76.955 Y42.646 E0.22960

G1 X76.762 Y42.311 E0.00898

G1 X69.975 Y49.098 E0.22326

G1 X69.975 Y48.570 E0.01227

G1 X76.601 Y41.944 E0.21797

G1 X76.449 Y41.569 E0.00942

G1 X69.975 Y48.043 E0.21296

G1 X69.975 Y47.515 E0.01227

G1 X76.200 Y41.290 E0.20479

G1 X75.673 Y41.290 E0.01227

G1 X69.975 Y46.988 E0.18744

G1 X69.975 Y46.460 E0.01227

G1 X75.145 Y41.290 E0.17009

G1 X74.618 Y41.290 E0.01227

G1 X69.975 Y45.933 E0.15273

G1 X69.975 Y45.406 E0.01227

G1 X74.090 Y41.290 E0.13538

G1 X73.563 Y41.290 E0.01227

G1 X69.975 Y44.878 E0.11803

G1 X69.975 Y44.351 E0.01227

G1 X73.036 Y41.290 E0.10068

G1 X72.508 Y41.290 E0.01227

G1 X69.975 Y43.823 E0.08333

G1 X69.975 Y43.296 E0.01227

G1 X71.981 Y41.290 E0.06598

G1 X71.453 Y41.290 E0.01227

G1 X69.975 Y42.768 E0.04863

G1 X69.975 Y42.241 E0.01227

G1 X70.926 Y41.290 E0.03128

G1 X70.398 Y41.290 E0.01227

```
G1 X69.809 Y41.879 E0.01937
M204 S1250
; stop printing object tpu print.STL id:9 copy 0
; printing object Petg print.STL id:8 copy 0
; stop printing object Petg print.STL id:8 copy 0
; printing object Petg print.STL id:4 copy 0
; stop printing object Petg print.STL id:4 copy 0
; printing object tpu print.STL id:29 copy 0
G1 E-2.24000
;WIPE_START
G1 F7200.000
G1 X70.398 Y41.290 E-0.26372
G1 X70.926 Y41.290 E-0.16703
G1 X69.975 Y42.241 E-0.42582
G1 X69.975 Y42.416 E-0.05543
;WIPE_END
G1 E-0.04800 F2400.000
G1 Z1.150 F9000.000
G1 X120.337 Y41.177
G1 Z0.950
G1 E3.20000 F1500.000
M204 S800
;TYPE:Perimeter
;WIDTH:0.45
G1 F2400.000
G1 X127.208 Y41.177 E0.17903
G1 X127.272 Y41.402 E0.00610
G1 X127.632 Y42.290 E0.02499
G1 X128.065 Y43.041 E0.02257
```

G1 X128.583 Y43.730 E0.02246

G1 X129.183 Y44.349 E0.02246

G1 X129.854 Y44.890 E0.02246

G1 X130.587 Y45.344 E0.02246

G1 X131.370 Y45.705 E0.02246

G1 X132.191 Y45.966 E0.02246

G1 X133.038 Y46.125 E0.02246

G1 X133.879 Y46.177 E0.02194

G1 X141.051 Y46.177 E0.18688

G1 X141.051 Y49.891 E0.09678

G1 X133.879 Y49.891 E0.18688

G1 X133.038 Y49.943 E0.02194

G1 X132.191 Y50.101 E0.02246

G1 X131.370 Y50.363 E0.02246

G1 X130.587 Y50.724 E0.02246

G1 X129.854 Y51.178 E0.02246

G1 X129.183 Y51.719 E0.02246

G1 X128.583 Y52.338 E0.02246

G1 X128.065 Y53.026 E0.02246

G1 X127.634 Y53.773 E0.02246

G1 X127.299 Y54.567 E0.02246

G1 X127.208 Y54.891 E0.00876

G1 X120.337 Y54.891 E0.17903

G1 X120.337 Y41.237 E0.35578

M204 S1250

G1 X119.919 Y40.759 F9000.000

M204 S800

;TYPE:External perimeter

G1 F1800.000

```
G1 X127.524 Y40.759 E0.19815
G1 X127.667 Y41.266 E0.01373
G1 X128.009 Y42.107 E0.02365
G1 X128.414 Y42.810 E0.02116
G1 X128.902 Y43.458 E0.02112
G1 X129.465 Y44.040 E0.02112
G1 X130.096 Y44.548 E0.02112
G1 X130.785 Y44.975 E0.02112
G1 X131.521 Y45.314 E0.02112
G1 X132.293 Y45.560 E0.02112
G1 X133.090 Y45.709 E0.02112
G1 X133.892 Y45.759 E0.02093
G1 X141.469 Y45.759 E0.19743
G1 X141.469 Y50.309 E0.11855
G1 X133.892 Y50.309 E0.19743
G1 X133.090 Y50.358 E0.02093
G1 X132.293 Y50.507 E0.02112
G1 X131.521 Y50.753 E0.02112
G1 X130.785 Y51.092 E0.02112
G1 X130.096 Y51.519 E0.02112
G1 X129.465 Y52.028 E0.02112
G1 X128.902 Y52.610 E0.02112
G1 X128.414 Y53.257 E0.02112
G1 X128.009 Y53.959 E0.02112
G1 X127.694 Y54.706 E0.02112
G1 X127.524 Y55.309 E0.01632
G1 X119.919 Y55.309 E0.19815
G1 X119.919 Y40.819 E0.37755
M204 S1250
```

```
G1 X120.293 Y40.901 F9000.000
G1 E-2.24000 F2400.000
;WIPE_START
G1 F7200.000
G1 X122.799 Y40.796 E-0.91200
;WIPE_END
G1 E-0.04800 F2400.000
G1 Z1.150 F9000.000
G1 X140.928 Y49.014
G1 Z0.950
G1 E3.20000 F1500.000
M204 S1000
;TYPE:Top solid infill
;WIDTH:0.405169
G1 F2400.000
G1 X140.339 Y49.603 E0.01937
G1 X139.812 Y49.603 E0.01227
G1 X140.763 Y48.652 E0.03128
G1 X140.763 Y48.124 E0.01227
G1 X139.284 Y49.603 E0.04863
G1 X138.757 Y49.603 E0.01227
G1 X140.763 Y47.597 E0.06598
G1 X140.763 Y47.069 E0.01227
G1 X138.229 Y49.603 E0.08333
G1 X137.702 Y49.603 E0.01227
G1 X140.763 Y46.542 E0.10068
G1 X140.763 Y46.465 E0.00179
G1 X140.312 Y46.465 E0.01048
G1 X137.174 Y49.603 E0.10321
```

G1 X136.647 Y49.603 E0.01227

G1 X139.785 Y46.465 E0.10321

G1 X139.257 Y46.465 E0.01227

G1 X136.120 Y49.603 E0.10321

G1 X135.592 Y49.603 E0.01227

G1 X138.730 Y46.465 E0.10321

G1 X138.202 Y46.465 E0.01227

G1 X135.065 Y49.603 E0.10321

G1 X134.537 Y49.603 E0.01227

G1 X137.675 Y46.465 E0.10321

G1 X137.147 Y46.465 E0.01227

G1 X134.010 Y49.603 E0.10321

G1 X133.457 Y49.628 E0.01288

G1 X136.620 Y46.465 E0.10405

G1 X136.092 Y46.465 E0.01227

G1 X132.878 Y49.680 E0.10574

G1 X132.229 Y49.801 E0.01536

G1 X135.565 Y46.465 E0.10974

G1 X135.037 Y46.465 E0.01227

G1 X131.193 Y50.309 E0.12645

M204 S1250

G1 E-2.24000

;WIPE_START

G1 F7200.000

G1 X133.230 Y48.273 E-0.91200

;WIPE_END

G1 E-0.04800 F2400.000

G1 Z1.150 F9000.000

G1 X127.335 Y54.168

```
G1 Z0.950
G1 E3.20000 F1500.000
M204 S1000
G1 F2400.000
G1 X126.900 Y54.603 E0.01431
G1 X126.372 Y54.603 E0.01227
G1 X127.438 Y53.537 E0.03505
G1 X127.824 Y52.867 E0.01799
G1 X128.364 Y52.150 E0.02088
G1 X128.988 Y51.505 E0.02088
G1 X129.687 Y50.942 E0.02088
G1 X130.553 Y50.422 E0.02350
G1 X134.510 Y46.465 E0.13015
G1 X133.982 Y46.465 E0.01227
G1 X125.845 Y54.603 E0.26769
G1 X125.317 Y54.603 E0.01227
G1 X133.479 Y46.441 E0.26848
G1 X132.984 Y46.408 E0.01153
G1 X124.790 Y54.603 E0.26956
G1 X124.262 Y54.603 E0.01227
G1 X132.540 Y46.325 E0.27230
G1 X132.098 Y46.239 E0.01047
G1 X123.735 Y54.603 E0.27511
G1 X123.207 Y54.603 E0.01227
G1 X131.698 Y46.112 E0.27930
G1 X131.298 Y45.984 E0.00977
G1 X122.680 Y54.603 E0.28349
G1 X122.152 Y54.603 E0.01227
G1 X130.934 Y45.821 E0.28886
```

G1 X130.573 Y45.655 E0.00925

G1 X121.625 Y54.603 E0.29433

G1 X121.098 Y54.603 E0.01227

G1 X130.235 Y45.465 E0.30057

G1 X129.909 Y45.263 E0.00891

G1 X120.625 Y54.548 E0.30541

G1 X120.625 Y54.020 E0.01227

G1 X129.594 Y45.051 E0.29504

G1 X129.302 Y44.815 E0.00873

G1 X120.625 Y53.493 E0.28544

G1 X120.625 Y52.965 E0.01227

G1 X129.010 Y44.580 E0.27583

G1 X128.748 Y44.314 E0.00868

G1 X120.625 Y52.438 E0.26721

G1 X120.625 Y51.910 E0.01227

G1 X128.489 Y44.046 E0.25868

G1 X128.246 Y43.761 E0.00870

G1 X120.625 Y51.383 E0.25070

G1 X120.625 Y50.855 E0.01227

G1 X128.020 Y43.461 E0.24324

G1 X127.798 Y43.155 E0.00878

G1 X120.625 Y50.328 E0.23594

G1 X120.625 Y49.800 E0.01227

G1 X127.605 Y42.821 E0.22960

G1 X127.412 Y42.486 E0.00898

G1 X120.625 Y49.273 E0.22326

G1 X120.625 Y48.745 E0.01227

G1 X127.251 Y42.119 E0.21797

G1 X127.099 Y41.744 E0.00942

```
G1 X120.625 Y48.218 E0.21296
G1 X120.625 Y47.690 E0.01227
G1 X126.851 Y41.465 E0.20479
G1 X126.323 Y41.465 E0.01227
G1 X120.625 Y47.163 E0.18744
G1 X120.625 Y46.636 E0.01227
G1 X125.796 Y41.465 E0.17009
G1 X125.268 Y41.465 E0.01227
G1 X120.625 Y46.108 E0.15273
G1 X120.625 Y45.581 E0.01227
G1 X124.741 Y41.465 E0.13538
G1 X124.213 Y41.465 E0.01227
G1 X120.625 Y45.053 E0.11803
G1 X120.625 Y44.526 E0.01227
G1 X123.686 Y41.465 E0.10068
G1 X123.158 Y41.465 E0.01227
G1 X120.625 Y43.998 E0.08333
G1 X120.625 Y43.471 E0.01227
G1 X122.631 Y41.465 E0.06598
G1 X122.103 Y41.465 E0.01227
G1 X120.625 Y42.943 E0.04863
G1 X120.625 Y42.416 E0.01227
G1 X121.576 Y41.465 E0.03128
G1 X121.048 Y41.465 E0.01227
G1 X120.460 Y42.054 E0.01937
M204 S1250
; stop printing object tpu print.STL id:29 copy 0
; printing object tpu print.STL id:25 copy 0
G1 E-2.24000
```

```
;WIPE_START
G1 F7200.000
G1 X121.048 Y41.465 E-0.26372
G1 X121.576 Y41.465 E-0.16703
G1 X120.625 Y42.416 E-0.42582
G1 X120.625 Y42.591 E-0.05543
;WIPE_END
G1 E-0.04800 F2400.000
G1 Z1.150 F9000.000
G1 X120.460 Y61.126
G1 Z0.950
G1 E3.20000 F1500.000
M204 S800
;TYPE:Perimeter
;WIDTH:0.45
G1 F2400.000
G1 X127.215 Y61.126 E0.17603
G1 X127.279 Y61.351 E0.00610
G1 X127.640 Y62.239 E0.02499
G1 X128.072 Y62.990 E0.02257
G1 X128.591 Y63.679 E0.02246
G1 X129.191 Y64.298 E0.02246
G1 X129.862 Y64.839 E0.02246
G1 X130.594 Y65.293 E0.02246
G1 X131.377 Y65.654 E0.02246
G1 X132.199 Y65.915 E0.02246
G1 X133.046 Y66.074 E0.02246
G1 X133.886 Y66.126 E0.02194
G1 X141.059 Y66.126 E0.18688
```

```
G1 X141.059 Y69.840 E0.09678
G1 X133.886 Y69.840 E0.18688
G1 X133.046 Y69.892 E0.02194
G1 X132.199 Y70.050 E0.02246
G1 X131.377 Y70.312 E0.02246
G1 X130.594 Y70.673 E0.02246
G1 X129.862 Y71.127 E0.02246
G1 X129.191 Y71.668 E0.02246
G1 X128.591 Y72.287 E0.02246
G1 X128.072 Y72.975 E0.02246
G1 X127.642 Y73.722 E0.02246
G1 X127.307 Y74.516 E0.02246
G1 X127.215 Y74.840 E0.00876
G1 X120.344 Y74.840 E0.17903
G1 X120.344 Y61.126 E0.35734
G1 X120.400 Y61.126 E0.00144
M204 S1250
G1 X119.926 Y60.708 F9000.000
M204 S800
;TYPE:External perimeter
G1 F1800.000
G1 X127.531 Y60.708 E0.19815
G1 X127.675 Y61.215 E0.01373
G1 X128.016 Y62.056 E0.02365
G1 X128.421 Y62.759 E0.02116
G1 X128.909 Y63.406 E0.02112
G1 X129.473 Y63.989 E0.02112
G1 X130.104 Y64.497 E0.02112
G1 X130.793 Y64.924 E0.02112
```

```
G1 X131.529 Y65.263 E0.02112
G1 X132.301 Y65.509 E0.02112
G1 X133.097 Y65.658 E0.02112
G1 X133.899 Y65.708 E0.02093
G1 X141.476 Y65.708 E0.19743
G1 X141.476 Y70.258 E0.11855
G1 X133.899 Y70.258 E0.19743
G1 X133.097 Y70.307 E0.02093
G1 X132.301 Y70.456 E0.02112
G1 X131.529 Y70.702 E0.02112
G1 X130.793 Y71.041 E0.02112
G1 X130.104 Y71.468 E0.02112
G1 X129.473 Y71.977 E0.02112
G1 X128.909 Y72.559 E0.02112
G1 X128.421 Y73.206 E0.02112
G1 X128.017 Y73.908 E0.02112
G1 X127.702 Y74.655 E0.02112
G1 X127.531 Y75.258 E0.01632
G1 X119.926 Y75.258 E0.19815
G1 X119.926 Y60.768 E0.37755
M204 S1250
G1 X120.300 Y60.850 F9000.000
G1 E-2.24000 F2400.000
;WIPE_START
G1 F7200.000
G1 X122.806 Y60.745 E-0.91200
;WIPE_END
G1 E-0.04800 F2400.000
G1 Z1.150 F9000.000
```

G1 X140.936 Y68.963

G1 Z0.950

G1 E3.20000 F1500.000

M204 S1000

;TYPE:Top solid infill

;WIDTH:0.405169

G1 F2400.000

G1 X140.347 Y69.552 E0.01937

G1 X139.819 Y69.552 E0.01227

G1 X140.770 Y68.601 E0.03128

G1 X140.770 Y68.073 E0.01227

G1 X139.292 Y69.552 E0.04863

G1 X138.764 Y69.552 E0.01227

G1 X140.770 Y67.546 E0.06598

G1 X140.770 Y67.018 E0.01227

G1 X138.237 Y69.552 E0.08333

G1 X137.709 Y69.552 E0.01227

G1 X140.770 Y66.491 E0.10068

G1 X140.770 Y66.414 E0.00179

G1 X140.320 Y66.414 E0.01048

G1 X137.182 Y69.552 E0.10321

G1 X136.654 Y69.552 E0.01227

G1 X139.792 Y66.414 E0.10321

G1 X139.265 Y66.414 E0.01227

G1 X136.127 Y69.552 E0.10321

G1 X135.600 Y69.552 E0.01227

G1 X138.737 Y66.414 E0.10321

G1 X138.210 Y66.414 E0.01227

G1 X135.072 Y69.552 E0.10321

```
G1 X134.545 Y69.552 E0.01227
G1 X137.682 Y66.414 E0.10321
G1 X137.155 Y66.414 E0.01227
G1 X134.017 Y69.552 E0.10321
G1 X133.464 Y69.577 E0.01288
G1 X136.627 Y66.414 E0.10405
G1 X136.100 Y66.414 E0.01227
G1 X132.885 Y69.629 E0.10574
G1 X132.236 Y69.750 E0.01536
G1 X135.572 Y66.414 E0.10974
G1 X135.045 Y66.414 E0.01227
G1 X131.201 Y70.258 E0.12645
M204 S1250
G1 E-2.24000
;WIPE_START
G1 F7200.000
G1 X133.237 Y68.221 E-0.91200
;WIPE_END
G1 E-0.04800 F2400.000
G1 Z1.150 F9000.000
G1 X127.342 Y74.116
G1 Z0.950
G1 E3.20000 F1500.000
M204 S1000
G1 F2400.000
G1 X126.907 Y74.552 E0.01431
G1 X126.380 Y74.552 E0.01227
G1 X127.445 Y73.486 E0.03505
G1 X127.831 Y72.816 E0.01799
```

```
G1 X128.371 Y72.099 E0.02088
G1 X128.996 Y71.454 E0.02088
G1 X129.695 Y70.891 E0.02088
G1 X130.561 Y70.371 E0.02350
G1 X134.517 Y66.414 E0.13015
G1 X133.990 Y66.414 E0.01227
G1 X125.852 Y74.552 E0.26769
G1 X125.325 Y74.552 E0.01227
G1 X133.487 Y66.390 E0.26848
G1 X132.992 Y66.357 E0.01153
G1 X124.797 Y74.552 E0.26956
G1 X124.270 Y74.552 E0.01227
G1 X132.548 Y66.274 E0.27230
G1 X132.106 Y66.188 E0.01047
G1 X123.742 Y74.552 E0.27511
G1 X123.215 Y74.552 E0.01227
G1 X131.706 Y66.061 E0.27930
G1 X131.306 Y65.933 E0.00977
G1 X122.687 Y74.552 E0.28349
G1 X122.160 Y74.552 E0.01227
G1 X130.941 Y65.770 E0.28886
G1 X130.580 Y65.604 E0.00925
G1 X121.632 Y74.552 E0.29433
G1 X121.105 Y74.552 E0.01227
G1 X130.243 Y65.414 E0.30057
G1 X129.917 Y65.212 E0.00891
G1 X120.633 Y74.497 E0.30541
G1 X120.633 Y73.969 E0.01227
G1 X129.602 Y65.000 E0.29504
```

```
G1 X129.310 Y64.764 E0.00873
G1 X120.633 Y73.442 E0.28544
M73 P90 R7
G1 X120.633 Y72.914 E0.01227
G1 X129.018 Y64.529 E0.27583
G1 X128.756 Y64.263 E0.00868
G1 X120.633 Y72.387 E0.26721
G1 X120.633 Y71.859 E0.01227
G1 X128.496 Y63.995 E0.25868
G1 X128.254 Y63.710 E0.00870
G1 X120.633 Y71.332 E0.25070
G1 X120.633 Y70.804 E0.01227
G1 X128.027 Y63.410 E0.24324
G1 X127.805 Y63.104 E0.00878
G1 X120.633 Y70.277 E0.23594
G1 X120.633 Y69.749 E0.01227
G1 X127.612 Y62.770 E0.22960
G1 X127.420 Y62.435 E0.00898
G1 X120.633 Y69.222 E0.22326
G1 X120.633 Y68.694 E0.01227
G1 X127.259 Y62.068 E0.21797
G1 X127.107 Y61.693 E0.00942
G1 X120.633 Y68.167 E0.21296
G1 X120.633 Y67.639 E0.01227
G1 X126.858 Y61.414 E0.20479
G1 X126.331 Y61.414 E0.01227
G1 X120.633 Y67.112 E0.18744
G1 X120.633 Y66.584 E0.01227
G1 X125.803 Y61.414 E0.17009
```

```
G1 X125.276 Y61.414 E0.01227
G1 X120.633 Y66.057 E0.15273
G1 X120.633 Y65.530 E0.01227
G1 X124.748 Y61.414 E0.13538
G1 X124.221 Y61.414 E0.01227
G1 X120.633 Y65.002 E0.11803
G1 X120.633 Y64.475 E0.01227
G1 X123.693 Y61.414 E0.10068
G1 X123.166 Y61.414 E0.01227
G1 X120.633 Y63.947 E0.08333
G1 X120.633 Y63.420 E0.01227
G1 X122.638 Y61.414 E0.06598
G1 X122.111 Y61.414 E0.01227
G1 X120.633 Y62.892 E0.04863
G1 X120.633 Y62.365 E0.01227
G1 X121.583 Y61.414 E0.03128
G1 X121.056 Y61.414 E0.01227
G1 X120.467 Y62.003 E0.01937
M204 S1250
; stop printing object tpu print.STL id:25 copy 0
; printing object Petg print.STL id:24 copy 0
; stop printing object Petg print.STL id:24 copy 0
; printing object Petg print.STL id:28 copy 0
; stop printing object Petg print.STL id:28 copy 0
G1 E-2.80000
;WIPE_START
G1 F7200.000
G1 X121.056 Y61.414 E-0.26372
G1 X121.583 Y61.414 E-0.16703
```

```
G1 X120.633 Y62.365 E-0.42582
G1 X120.633 Y62.892 E-0.16703
G1 X120.892 Y62.632 E-0.11639
;WIPE_END
G1 E-0.06000 F2400.000
G1 Z1.150 F9000.000
; Filament-specific end gcode
M600
M106 S127.5
T0
M900 K0 ; Filament gcode
; printing object tpu print.STL id:19 copy 0
; stop printing object tpu print.STL id:19 copy 0
; printing object Petg print.STL id:18 copy 0
G1 X61.611 Y54.135
G1 Z0.950
G1 E4.00000 F900.000
M204 S800
;TYPE:Perimeter
;WIDTH:0.45
G1 F1292.454
G1 X54.740 Y54.135 E0.20589
G1 X54.649 Y53.812 E0.01007
G1 X54.314 Y53.017 E0.02583
G1 X53.884 Y52.270 E0.02583
G1 X53.365 Y51.582 E0.02583
G1 X52.765 Y50.963 E0.02583
G1 X52.094 Y50.422 E0.02583
G1 X51.361 Y49.968 E0.02583
```

```
G1 X50.671 Y49.650 E0.02277
G1 X49.761 Y49.346 E0.02874
G1 X48.910 Y49.187 E0.02596
G1 X48.069 Y49.135 E0.02523
G1 X39.897 Y49.135 E0.24487
G1 X39.897 Y45.421 E0.11130
G1 X48.069 Y45.421 E0.24487
G1 X48.910 Y45.369 E0.02523
G1 X49.757 Y45.210 E0.02583
G1 X50.578 Y44.949 E0.02583
G1 X51.361 Y44.588 E0.02583
G1 X52.007 Y44.188 E0.02277
G1 X52.762 Y43.596 E0.02874
G1 X53.365 Y42.974 E0.02596
G1 X53.884 Y42.285 E0.02583
G1 X54.314 Y41.538 E0.02583
G1 X54.649 Y40.744 E0.02583
G1 X54.740 Y40.421 E0.01007
G1 X61.611 Y40.421 E0.20589
G1 X61.611 Y54.075 E0.40914
M204 S1250
G1 X62.029 Y54.553 F9000.000
M204 S800
;TYPE:External perimeter
G1 F1292.454
G1 X54.424 Y54.553 E0.22787
G1 X54.254 Y53.950 E0.01877
G1 X53.939 Y53.203 E0.02428
G1 X53.534 Y52.501 E0.02428
```

```
G1 X53.047 Y51.854 E0.02428
G1 X52.483 Y51.272 E0.02428
G1 X51.852 Y50.763 E0.02428
G1 X51.163 Y50.336 E0.02428
G1 X50.517 Y50.039 E0.02131
G1 X49.656 Y49.752 E0.02719
G1 X48.858 Y49.602 E0.02433
G1 X48.056 Y49.553 E0.02407
G1 X39.479 Y49.553 E0.25701
G1 X39.479 Y45.003 E0.13634
G1 X48.056 Y45.003 E0.25701
G1 X48.858 Y44.953 E0.02407
G1 X49.655 Y44.804 E0.02428
G1 X50.427 Y44.558 E0.02428
G1 X51.163 Y44.219 E0.02428
G1 X51.767 Y43.845 E0.02131
G1 X52.482 Y43.285 E0.02719
G1 X53.047 Y42.702 E0.02433
G1 X53.534 Y42.054 E0.02428
G1 X53.939 Y41.352 E0.02428
G1 X54.254 Y40.606 E0.02428
G1 X54.424 Y40.003 E0.01877
G1 X62.029 Y40.003 E0.22787
G1 X62.029 Y54.493 E0.43418
M204 S1250
G1 X61.655 Y54.411 F9000.000
G1 X61.489 Y53.259
M204 S1000
;TYPE:Top solid infill
```

```
;WIDTH:0.404487
G1 F1450.455
G1 X60.901 Y53.847 E0.02219
G1 X60.375 Y53.847 E0.01406
G1 X61.323 Y52.898 E0.03582
G1 X61.323 Y52.372 E0.01406
G1 X59.848 Y53.847 E0.05570
G1 X59.322 Y53.847 E0.01406
G1 X61.323 Y51.845 E0.07558
G1 X61.323 Y51.319 E0.01406
G1 X58.795 Y53.847 E0.09546
G1 X58.269 Y53.847 E0.01406
G1 X61.323 Y50.792 E0.11534
G1 X61.323 Y50.266 E0.01406
G1 X57.742 Y53.847 E0.13522
G1 X57.216 Y53.847 E0.01406
G1 X61.323 Y49.739 E0.15510
G1 X61.323 Y49.213 E0.01406
G1 X56.689 Y53.847 E0.17498
G1 X56.163 Y53.847 E0.01406
G1 X61.323 Y48.686 E0.19486
G1 X61.323 Y48.160 E0.01406
G1 X55.636 Y53.847 E0.21474
G1 X55.110 Y53.847 E0.01406
G1 X61.323 Y47.633 E0.23462
G1 X61.323 Y47.107 E0.01406
G1 X54.860 Y53.570 E0.24405
G1 X54.704 Y53.200 E0.01073
G1 X61.323 Y46.580 E0.24995
```

G1 X61.323 Y46.054 E0.01406

G1 X54.542 Y52.835 E0.25607

G1 X54.349 Y52.501 E0.01029

G1 X61.323 Y45.527 E0.26334

G1 X61.323 Y45.001 E0.01406

G1 X54.157 Y52.167 E0.27060

G1 X53.936 Y51.861 E0.01007

G1 X61.323 Y44.474 E0.27893

G1 X61.323 Y43.948 E0.01406

G1 X53.710 Y51.561 E0.28748

G1 X53.469 Y51.275 E0.00998

G1 X61.323 Y43.421 E0.29657

G1 X61.323 Y42.895 E0.01406

G1 X53.210 Y51.008 E0.30635

M73 P90 R6

G1 X52.950 Y50.741 E0.00994

G1 X61.323 Y42.368 E0.31617

G1 X61.323 Y41.841 E0.01406

G1 X52.658 Y50.506 E0.32718

G1 X52.367 Y50.271 E0.01000

G1 X61.323 Y41.315 E0.33819

G1 X61.323 Y40.788 E0.01406

G1 X52.054 Y50.058 E0.35000

G1 X51.729 Y49.856 E0.01021

G1 X60.876 Y40.709 E0.34540

G1 X60.350 Y40.709 E0.01406

G1 X51.394 Y49.665 E0.33818

G1 X51.033 Y49.499 E0.01060

G1 X59.823 Y40.709 E0.33191

```
G1 X59.297 Y40.709 E0.01406
G1 X50.663 Y49.343 E0.32602
G1 X50.268 Y49.211 E0.01111
G1 X58.770 Y40.709 E0.32104
G1 X58.244 Y40.709 E0.01406
G1 X49.873 Y49.080 E0.31607
G1 X49.434 Y48.992 E0.01195
G1 X57.717 Y40.709 E0.31275
G1 X57.191 Y40.709 E0.01406
G1 X48.991 Y48.909 E0.30962
G1 X48.500 Y48.873 E0.01313
G1 X56.664 Y40.709 E0.30826
G1 X56.138 Y40.709 E0.01406
G1 X48.000 Y48.847 E0.30728
G1 X47.473 Y48.847 E0.01406
G1 X51.460 Y44.860 E0.15053
G1 X52.172 Y44.424 E0.02230
G1 X52.955 Y43.811 E0.02656
G1 X53.584 Y43.161 E0.02414
G1 X54.125 Y42.445 E0.02397
G1 X54.463 Y41.857 E0.01810
G1 X55.611 Y40.709 E0.04335
G1 X55.085 Y40.709 E0.01406
G1 X54.587 Y41.206 E0.01878
M204 S1250
G1 X50.822 Y44.972 F9000.000
M204 S1000
G1 F1450.455
G1 X46.947 Y48.847 E0.14631
```

G1 X46.420 Y48.847 E0.01406

G1 X49.765 Y45.502 E0.12629

G1 X49.117 Y45.623 E0.01759

G1 X45.894 Y48.847 E0.12171

G1 X45.367 Y48.847 E0.01406

G1 X48.533 Y45.681 E0.11954

G1 X47.979 Y45.709 E0.01483

G1 X44.841 Y48.847 E0.11848

G1 X44.314 Y48.847 E0.01406

G1 X47.452 Y45.709 E0.11848

G1 X46.925 Y45.709 E0.01406

G1 X43.788 Y48.847 E0.11848

G1 X43.261 Y48.847 E0.01406

G1 X46.399 Y45.709 E0.11848

G1 X45.872 Y45.709 E0.01406

G1 X42.735 Y48.847 E0.11848

G1 X42.208 Y48.847 E0.01406

G1 X45.346 Y45.709 E0.11848

G1 X44.819 Y45.709 E0.01406

G1 X41.682 Y48.847 E0.11848

G1 X41.155 Y48.847 E0.01406

G1 X44.293 Y45.709 E0.11848

G1 X43.766 Y45.709 E0.01406

G1 X40.629 Y48.847 E0.11848

G1 X40.185 Y48.847 E0.01184

G1 X40.185 Y48.763 E0.00222

G1 X43.240 Y45.709 E0.11534

G1 X42.713 Y45.709 E0.01406

G1 X40.185 Y48.237 E0.09546

```
G1 X40.185 Y47.710 E0.01406
G1 X42.187 Y45.709 E0.07557
G1 X41.660 Y45.709 E0.01406
G1 X40.185 Y47.184 E0.05569
G1 X40.185 Y46.657 E0.01406
G1 X41.134 Y45.709 E0.03581
G1 X40.607 Y45.709 E0.01406
G1 X40.020 Y46.296 E0.02218
M204 S1250
; stop printing object Petg print.STL id:18 copy 0
; printing object Petg print.STL id:14 copy 0
G1 E-4.00000 F2400.000
G1 X40.020 Y65.370 F9000.000
G1 E4.00000 F900.000
M204 S800
;TYPE:Perimeter
;WIDTH:0.45
G1 F1292.454
G1 X48.077 Y65.370 E0.24142
G1 X48.917 Y65.318 E0.02523
G1 X49.765 Y65.159 E0.02583
G1 X50.586 Y64.898 E0.02583
G1 X51.369 Y64.537 E0.02583
G1 X52.015 Y64.137 E0.02277
G1 X52.770 Y63.545 E0.02874
G1 X53.372 Y62.923 E0.02596
G1 X53.891 Y62.234 E0.02583
G1 X54.321 Y61.487 E0.02583
G1 X54.656 Y60.693 E0.02583
```

```
G1 X54.748 Y60.370 E0.01007
G1 X61.619 Y60.370 E0.20589
G1 X61.619 Y74.084 E0.41094
G1 X54.748 Y74.084 E0.20589
G1 X54.656 Y73.760 E0.01007
G1 X54.321 Y72.966 E0.02583
G1 X53.891 Y72.219 E0.02583
G1 X53.372 Y71.531 E0.02583
G1 X52.773 Y70.912 E0.02583
G1 X52.101 Y70.371 E0.02583
G1 X51.369 Y69.917 E0.02583
G1 X50.679 Y69.599 E0.02277
G1 X49.769 Y69.295 E0.02874
G1 X48.917 Y69.136 E0.02596
G1 X48.077 Y69.084 E0.02523
G1 X39.905 Y69.084 E0.24487
G1 X39.905 Y65.370 E0.11130
G1 X39.960 Y65.370 E0.00166
M204 S1250
G1 X39.487 Y64.952 F9000.000
M204 S800
;TYPE:External perimeter
G1 F1292.454
G1 X48.064 Y64.952 E0.25701
G1 X48.866 Y64.902 E0.02407
G1 X49.662 Y64.753 E0.02428
G1 X50.435 Y64.507 E0.02428
G1 X51.171 Y64.168 E0.02428
G1 X51.775 Y63.794 E0.02131
```

```
G1 X52.489 Y63.234 E0.02719
G1 X53.054 Y62.651 E0.02433
G1 X53.542 Y62.003 E0.02428
G1 X53.946 Y61.301 E0.02428
G1 X54.261 Y60.554 E0.02428
G1 X54.432 Y59.952 E0.01877
G1 X62.037 Y59.952 E0.22787
G1 X62.037 Y74.502 E0.43598
G1 X54.432 Y74.502 E0.22787
G1 X54.261 Y73.899 E0.01877
G1 X53.946 Y73.152 E0.02428
G1 X53.542 Y72.450 E0.02428
G1 X53.054 Y71.803 E0.02428
G1 X52.490 Y71.221 E0.02428
G1 X51.859 Y70.712 E0.02428
G1 X51.171 Y70.285 E0.02428
G1 X50.525 Y69.988 E0.02131
G1 X49.664 Y69.701 E0.02719
G1 X48.866 Y69.551 E0.02433
G1 X48.064 Y69.502 E0.02407
G1 X39.487 Y69.502 E0.25701
G1 X39.487 Y65.012 E0.13454
M204 S1250
G1 X39.882 Y65.017 F9000.000
G1 E-4.00000 F2400.000
G1 X61.496 Y73.208 F9000.000
G1 E4.00000 F900.000
M204 S1000
;TYPE:Top solid infill
```

```
;WIDTH:0.404487
G1 F1450.455
G1 X60.909 Y73.796 E0.02219
G1 X60.382 Y73.796 E0.01406
G1 X61.331 Y72.847 E0.03582
G1 X61.331 Y72.321 E0.01406
G1 X59.856 Y73.796 E0.05570
G1 X59.329 Y73.796 E0.01406
G1 X61.331 Y71.794 E0.07558
G1 X61.331 Y71.268 E0.01406
G1 X58.803 Y73.796 E0.09546
G1 X58.276 Y73.796 E0.01406
G1 X61.331 Y70.741 E0.11534
G1 X61.331 Y70.215 E0.01406
G1 X57.750 Y73.796 E0.13522
G1 X57.223 Y73.796 E0.01406
G1 X61.331 Y69.688 E0.15510
G1 X61.331 Y69.162 E0.01406
G1 X56.697 Y73.796 E0.17498
M73 P91 R6
G1 X56.170 Y73.796 E0.01406
G1 X61.331 Y68.635 E0.19486
G1 X61.331 Y68.109 E0.01406
G1 X55.644 Y73.796 E0.21474
G1 X55.117 Y73.796 E0.01406
G1 X61.331 Y67.582 E0.23462
G1 X61.331 Y67.056 E0.01406
G1 X54.867 Y73.519 E0.24405
G1 X54.711 Y73.149 E0.01073
```

```
G1 X61.331 Y66.529 E0.24995
G1 X61.331 Y66.002 E0.01406
G1 X54.549 Y72.784 E0.25607
G1 X54.357 Y72.450 E0.01029
G1 X61.331 Y65.476 E0.26334
G1 X61.331 Y64.949 E0.01406
G1 X54.164 Y72.116 E0.27060
G1 X53.944 Y71.810 E0.01007
G1 X61.331 Y64.423 E0.27893
G1 X61.331 Y63.896 E0.01406
G1 X53.717 Y71.510 E0.28748
G1 X53.476 Y71.224 E0.00998
G1 X61.331 Y63.370 E0.29657
G1 X61.331 Y62.843 E0.01406
G1 X53.217 Y70.957 E0.30635
G1 X52.957 Y70.690 E0.00994
G1 X61.331 Y62.317 E0.31617
G1 X61.331 Y61.790 E0.01406
G1 X52.666 Y70.455 E0.32718
G1 X52.374 Y70.220 E0.01000
G1 X61.331 Y61.264 E0.33819
G1 X61.331 Y60.737 E0.01406
G1 X52.061 Y70.007 E0.35000
G1 X51.736 Y69.805 E0.01021
G1 X60.884 Y60.658 E0.34540
G1 X60.357 Y60.658 E0.01406
G1 X51.401 Y69.614 E0.33818
G1 X51.041 Y69.448 E0.01060
G1 X59.831 Y60.658 E0.33191
```

G1 X59.304 Y60.658 E0.01406

G1 X50.670 Y69.292 E0.32602

G1 X50.275 Y69.160 E0.01111

G1 X58.778 Y60.658 E0.32104

G1 X58.251 Y60.658 E0.01406

G1 X49.881 Y69.028 E0.31607

G1 X49.442 Y68.941 E0.01195

G1 X57.725 Y60.658 E0.31275

G1 X57.198 Y60.658 E0.01406

G1 X48.998 Y68.858 E0.30962

G1 X48.508 Y68.822 E0.01313

G1 X56.672 Y60.658 E0.30826

G1 X56.145 Y60.658 E0.01406

G1 X48.007 Y68.796 E0.30728

G1 X47.481 Y68.796 E0.01406

G1 X51.467 Y64.809 E0.15053

G1 X52.180 Y64.373 E0.02230

G1 X52.963 Y63.760 E0.02656

G1 X53.592 Y63.110 E0.02414

G1 X54.132 Y62.394 E0.02397

G1 X54.470 Y61.806 E0.01810

G1 X55.619 Y60.658 E0.04335

G1 X55.092 Y60.658 E0.01406

G1 X54.595 Y61.155 E0.01878

M204 S1250

G1 X50.829 Y64.921 F9000.000

M204 S1000

G1 F1450.455

G1 X46.954 Y68.796 E0.14631

G1 X46.428 Y68.796 E0.01406

G1 X49.772 Y65.451 E0.12629

G1 X49.125 Y65.572 E0.01759

G1 X45.901 Y68.796 E0.12171

G1 X45.375 Y68.796 E0.01406

G1 X48.541 Y65.630 E0.11954

G1 X47.986 Y65.658 E0.01483

G1 X44.848 Y68.796 E0.11848

G1 X44.322 Y68.796 E0.01406

G1 X47.459 Y65.658 E0.11848

G1 X46.933 Y65.658 E0.01406

G1 X43.795 Y68.796 E0.11848

G1 X43.269 Y68.796 E0.01406

G1 X46.406 Y65.658 E0.11848

G1 X45.880 Y65.658 E0.01406

G1 X42.742 Y68.796 E0.11848

G1 X42.216 Y68.796 E0.01406

G1 X45.353 Y65.658 E0.11848

G1 X44.827 Y65.658 E0.01406

G1 X41.689 Y68.796 E0.11848

G1 X41.163 Y68.796 E0.01406

G1 X44.300 Y65.658 E0.11848

G1 X43.774 Y65.658 E0.01406

G1 X40.636 Y68.796 E0.11848

G1 X40.193 Y68.796 E0.01184

G1 X40.193 Y68.712 E0.00222

G1 X43.247 Y65.658 E0.11534

G1 X42.721 Y65.658 E0.01406

G1 X40.193 Y68.186 E0.09546

```
G1 X40.193 Y67.659 E0.01406
G1 X42.194 Y65.658 E0.07557
G1 X41.668 Y65.658 E0.01406
G1 X40.193 Y67.133 E0.05569
G1 X40.193 Y66.606 E0.01406
G1 X41.141 Y65.658 E0.03581
G1 X40.615 Y65.658 E0.01406
G1 X40.027 Y66.245 E0.02218
M204 S1250
; stop printing object Petg print.STL id:14 copy 0
; printing object tpu print.STL id:15 copy 0
; stop printing object tpu print.STL id:15 copy 0
; printing object Petg print.STL id:10 copy 0
G1 E-4.00000 F2400.000
G1 X40.027 Y87.562 F9000.000
G1 E4.00000 F900.000
M204 S800
;TYPE:Perimeter
;WIDTH:0.45
G1 F1292.454
G1 X48.026 Y87.562 E0.23966
G1 X48.866 Y87.510 E0.02523
G1 X49.713 Y87.351 E0.02583
G1 X50.535 Y87.090 E0.02583
G1 X51.318 Y86.729 E0.02583
G1 X51.963 Y86.329 E0.02277
G1 X52.718 Y85.737 E0.02874
G1 X53.321 Y85.115 E0.02596
G1 X53.840 Y84.427 E0.02583
```

```
G1 X54.270 Y83.680 E0.02583
G1 X54.605 Y82.885 E0.02583
G1 X54.697 Y82.562 E0.01007
G1 X61.568 Y82.562 E0.20589
G1 X61.568 Y96.276 E0.41094
G1 X54.697 Y96.276 E0.20589
G1 X54.605 Y95.953 E0.01007
G1 X54.270 Y95.159 E0.02583
G1 X53.840 Y94.412 E0.02583
G1 X53.321 Y93.723 E0.02583
G1 X52.722 Y93.104 E0.02583
G1 X52.050 Y92.563 E0.02583
G1 X51.318 Y92.109 E0.02583
G1 X50.628 Y91.791 E0.02277
G1 X49.718 Y91.488 E0.02874
G1 X48.866 Y91.328 E0.02596
G1 X48.026 Y91.276 E0.02523
G1 X39.853 Y91.276 E0.24487
G1 X39.853 Y87.562 E0.11130
G1 X39.967 Y87.562 E0.00342
M204 S1250
G1 X39.436 Y87.144 F9000.000
M204 S800
;TYPE:External perimeter
G1 F1292.454
G1 X48.013 Y87.144 E0.25701
G1 X48.815 Y87.095 E0.02407
G1 X49.611 Y86.946 E0.02428
G1 X50.383 Y86.700 E0.02428
```

```
G1 X51.119 Y86.361 E0.02428
G1 X51.724 Y85.986 E0.02131
G1 X52.438 Y85.426 E0.02719
G1 X53.003 Y84.843 E0.02433
G1 X53.491 Y84.196 E0.02428
G1 X53.895 Y83.493 E0.02428
G1 X54.210 Y82.747 E0.02428
G1 X54.381 Y82.144 E0.01877
G1 X61.986 Y82.144 E0.22787
G1 X61.986 Y96.694 E0.43598
G1 X54.381 Y96.694 E0.22787
G1 X54.210 Y96.091 E0.01877
G1 X53.895 Y95.345 E0.02428
G1 X53.491 Y94.643 E0.02428
G1 X53.003 Y93.995 E0.02428
G1 X52.439 Y93.413 E0.02428
G1 X51.808 Y92.905 E0.02428
G1 X51.119 Y92.478 E0.02428
G1 X50.474 Y92.180 E0.02131
G1 X49.613 Y91.893 E0.02719
G1 X48.815 Y91.744 E0.02433
G1 X48.013 Y91.694 E0.02407
G1 X39.436 Y91.694 E0.25701
G1 X39.436 Y87.204 E0.13454
M204 S1250
G1 X39.830 Y87.209 F9000.000
G1 E-4.00000 F2400.000
G1 X61.445 Y95.400 F9000.000
G1 E4.00000 F900.000
```

```
M204 S1000
;TYPE:Top solid infill
;WIDTH:0.404487
G1 F1450.455
G1 X60.857 Y95.988 E0.02219
G1 X60.331 Y95.988 E0.01406
G1 X61.279 Y95.039 E0.03582
G1 X61.279 Y94.513 E0.01406
G1 X59.804 Y95.988 E0.05570
G1 X59.278 Y95.988 E0.01406
G1 X61.279 Y93.986 E0.07558
G1 X61.279 Y93.460 E0.01406
G1 X58.751 Y95.988 E0.09546
G1 X58.225 Y95.988 E0.01406
G1 X61.279 Y92.933 E0.11534
G1 X61.279 Y92.407 E0.01406
G1 X57.698 Y95.988 E0.13522
G1 X57.172 Y95.988 E0.01406
G1 X61.279 Y91.880 E0.15510
G1 X61.279 Y91.354 E0.01406
G1 X56.645 Y95.988 E0.17498
G1 X56.119 Y95.988 E0.01406
G1 X61.279 Y90.827 E0.19486
G1 X61.279 Y90.301 E0.01406
G1 X55.592 Y95.988 E0.21474
G1 X55.066 Y95.988 E0.01406
G1 X61.279 Y89.774 E0.23462
G1 X61.279 Y89.248 E0.01406
G1 X54.816 Y95.711 E0.24405
```

G1 X54.660 Y95.341 E0.01073

G1 X61.279 Y88.721 E0.24995

G1 X61.279 Y88.195 E0.01406

G1 X54.498 Y94.976 E0.25607

G1 X54.305 Y94.642 E0.01029

G1 X61.279 Y87.668 E0.26334

G1 X61.279 Y87.142 E0.01406

G1 X54.113 Y94.308 E0.27060

G1 X53.892 Y94.002 E0.01007

G1 X61.279 Y86.615 E0.27893

G1 X61.279 Y86.089 E0.01406

G1 X53.666 Y93.702 E0.28748

G1 X53.425 Y93.416 E0.00998

G1 X61.279 Y85.562 E0.29657

G1 X61.279 Y85.036 E0.01406

G1 X53.166 Y93.149 E0.30635

G1 X52.906 Y92.883 E0.00994

G1 X61.279 Y84.509 E0.31617

G1 X61.279 Y83.983 E0.01406

G1 X52.615 Y92.648 E0.32718

G1 X52.323 Y92.413 E0.01000

G1 X61.279 Y83.456 E0.33819

G1 X61.279 Y82.930 E0.01406

G1 X52.010 Y92.199 E0.35000

G1 X51.685 Y91.998 E0.01021

G1 X60.832 Y82.850 E0.34540

G1 X60.306 Y82.850 E0.01406

G1 X51.350 Y91.806 E0.33818

G1 X50.989 Y91.640 E0.01060

```
G1 X59.779 Y82.850 E0.33191
G1 X59.253 Y82.850 E0.01406
G1 X50.619 Y91.484 E0.32602
G1 X50.224 Y91.353 E0.01111
G1 X58.726 Y82.850 E0.32104
G1 X58.200 Y82.850 E0.01406
G1 X49.829 Y91.221 E0.31607
G1 X49.391 Y91.133 E0.01195
G1 X57.673 Y82.850 E0.31275
G1 X57.147 Y82.850 E0.01406
G1 X48.947 Y91.050 E0.30962
M73 P91 R5
G1 X48.457 Y91.014 E0.01313
G1 X56.620 Y82.850 E0.30826
G1 X56.094 Y82.850 E0.01406
G1 X47.956 Y90.988 E0.30728
G1 X47.430 Y90.988 E0.01406
G1 X51.416 Y87.001 E0.15053
G1 X52.129 Y86.566 E0.02230
G1 X52.912 Y85.952 E0.02656
G1 X53.541 Y85.303 E0.02414
G1 X54.081 Y84.586 E0.02397
G1 X54.419 Y83.998 E0.01810
G1 X55.567 Y82.850 E0.04335
G1 X55.041 Y82.850 E0.01406
G1 X54.543 Y83.348 E0.01878
M204 S1250
G1 X50.778 Y87.113 F9000.000
M204 S1000
```

```
G1 F1450.455
G1 X46.903 Y90.988 E0.14631
G1 X46.377 Y90.988 E0.01406
G1 X49.721 Y87.643 E0.12629
G1 X49.074 Y87.765 E0.01759
G1 X45.850 Y90.988 E0.12171
M73 P92 R5
G1 X45.324 Y90.988 E0.01406
G1 X48.489 Y87.822 E0.11954
G1 X47.935 Y87.850 E0.01483
G1 X44.797 Y90.988 E0.11848
G1 X44.271 Y90.988 E0.01406
G1 X47.408 Y87.850 E0.11848
G1 X46.882 Y87.850 E0.01406
G1 X43.744 Y90.988 E0.11848
G1 X43.218 Y90.988 E0.01406
G1 X46.355 Y87.850 E0.11848
G1 X45.829 Y87.850 E0.01406
G1 X42.691 Y90.988 E0.11848
G1 X42.165 Y90.988 E0.01406
G1 X45.302 Y87.850 E0.11848
G1 X44.776 Y87.850 E0.01406
G1 X41.638 Y90.988 E0.11848
G1 X41.112 Y90.988 E0.01406
G1 X44.249 Y87.850 E0.11848
G1 X43.723 Y87.850 E0.01406
G1 X40.585 Y90.988 E0.11848
G1 X40.142 Y90.988 E0.01184
G1 X40.142 Y90.905 E0.00222
```

```
G1 X43.196 Y87.850 E0.11534
G1 X42.670 Y87.850 E0.01406
G1 X40.142 Y90.378 E0.09546
G1 X40.142 Y89.852 E0.01406
G1 X42.143 Y87.850 E0.07557
G1 X41.617 Y87.850 E0.01406
G1 X40.142 Y89.325 E0.05569
G1 X40.142 Y88.799 E0.01406
G1 X41.090 Y87.850 E0.03581
G1 X40.564 Y87.850 E0.01406
G1 X39.976 Y88.438 E0.02218
M204 S1250
; stop printing object Petg print.STL id:10 copy 0
; printing object tpu print.STL id:11 copy 0
; stop printing object tpu print.STL id:11 copy 0
; printing object tpu print.STL id:13 copy 0
; stop printing object tpu print.STL id:13 copy 0
; printing object tpu print.STL id:17 copy 0
; stop printing object tpu print.STL id:17 copy 0
; printing object Petg print.STL id:16 copy 0
G1 E-4.00000 F2400.000
G1 X54.688 Y122.031 F9000.000
G1 E4.00000 F900.000
M204 S800
;TYPE:Perimeter
;WIDTH:0.45
G1 F1292.454
G1 X61.560 Y122.031 E0.20589
G1 X61.560 Y135.746 E0.41094
```

G1 X54.688 Y135.746 E0.20589

G1 X54.597 Y135.422 E0.01007

G1 X54.262 Y134.628 E0.02583

G1 X53.832 Y133.881 E0.02583

G1 X53.313 Y133.193 E0.02583

G1 X52.713 Y132.573 E0.02583

G1 X52.042 Y132.033 E0.02583

G1 X51.309 Y131.578 E0.02583

G1 X50.619 Y131.261 E0.02277

G1 X49.709 Y130.957 E0.02874

G1 X48.858 Y130.798 E0.02596

G1 X48.017 Y130.746 E0.02523

G1 X39.845 Y130.746 E0.24487

G1 X39.845 Y127.031 E0.11130

G1 X48.017 Y127.031 E0.24487

G1 X48.858 Y126.980 E0.02523

G1 X49.705 Y126.821 E0.02583

G1 X50.526 Y126.559 E0.02583

G1 X51.309 Y126.199 E0.02583

G1 X51.955 Y125.798 E0.02277

G1 X52.710 Y125.207 E0.02874

G1 X53.313 Y124.584 E0.02596

G1 X53.832 Y123.896 E0.02583

G1 X54.262 Y123.149 E0.02583

G1 X54.597 Y122.355 E0.02583

G1 X54.672 Y122.089 E0.00828

M204 S1250

G1 X54.373 Y121.614 F9000.000

M204 S800

```
;TYPE:External perimeter
G1 F1292.454
G1 X61.977 Y121.614 E0.22787
G1 X61.977 Y136.164 E0.43598
G1 X54.373 Y136.164 E0.22787
G1 X54.202 Y135.561 E0.01877
G1 X53.887 Y134.814 E0.02428
G1 X53.482 Y134.112 E0.02428
G1 X52.995 Y133.465 E0.02428
G1 X52.431 Y132.883 E0.02428
G1 X51.800 Y132.374 E0.02428
G1 X51.111 Y131.947 E0.02428
G1 X50.465 Y131.650 E0.02131
G1 X49.604 Y131.362 E0.02719
G1 X48.806 Y131.213 E0.02433
G1 X48.004 Y131.164 E0.02407
G1 X39.427 Y131.164 E0.25701
G1 X39.427 Y126.614 E0.13634
G1 X48.004 Y126.614 E0.25701
G1 X48.806 Y126.564 E0.02407
G1 X49.603 Y126.415 E0.02428
G1 X50.375 Y126.169 E0.02428
G1 X51.111 Y125.830 E0.02428
G1 X51.716 Y125.455 E0.02131
G1 X52.430 Y124.896 E0.02719
G1 X52.995 Y124.312 E0.02433
G1 X53.482 Y123.665 E0.02428
G1 X53.887 Y122.963 E0.02428
G1 X54.202 Y122.216 E0.02428
```

```
G1 X54.356 Y121.671 E0.01697
M204 S1250
G1 X54.694 Y121.852 F9000.000
G1 E-4.00000 F2400.000
G1 X61.437 Y134.870 F9000.000
G1 E4.00000 F900.000
M204 S1000
;TYPE:Top solid infill
;WIDTH:0.404487
G1 F1450.455
G1 X60.849 Y135.457 E0.02219
G1 X60.323 Y135.457 E0.01406
G1 X61.271 Y134.509 E0.03582
G1 X61.271 Y133.982 E0.01406
G1 X59.796 Y135.457 E0.05570
G1 X59.270 Y135.457 E0.01406
G1 X61.271 Y133.456 E0.07558
G1 X61.271 Y132.929 E0.01406
G1 X58.743 Y135.457 E0.09546
G1 X58.217 Y135.457 E0.01406
G1 X61.271 Y132.403 E0.11534
G1 X61.271 Y131.876 E0.01406
G1 X57.690 Y135.457 E0.13522
G1 X57.164 Y135.457 E0.01406
G1 X61.271 Y131.350 E0.15510
G1 X61.271 Y130.823 E0.01406
G1 X56.637 Y135.457 E0.17498
G1 X56.111 Y135.457 E0.01406
G1 X61.271 Y130.297 E0.19486
```

```
G1 X61.271 Y129.770 E0.01406
G1 X55.584 Y135.457 E0.21474
G1 X55.058 Y135.457 E0.01406
G1 X61.271 Y129.244 E0.23462
G1 X61.271 Y128.717 E0.01406
G1 X54.808 Y135.181 E0.24405
G1 X54.652 Y134.810 E0.01073
G1 X61.271 Y128.191 E0.24995
G1 X61.271 Y127.664 E0.01406
G1 X54.490 Y134.446 E0.25607
G1 X54.297 Y134.112 E0.01029
G1 X61.271 Y127.138 E0.26334
G1 X61.271 Y126.611 E0.01406
G1 X54.105 Y133.778 E0.27060
G1 X53.884 Y133.472 E0.01007
G1 X61.271 Y126.085 E0.27893
G1 X61.271 Y125.558 E0.01406
G1 X53.658 Y133.172 E0.28748
G1 X53.417 Y132.886 E0.00998
G1 X61.271 Y125.032 E0.29657
G1 X61.271 Y124.505 E0.01406
G1 X53.158 Y132.618 E0.30635
G1 X52.898 Y132.352 E0.00994
G1 X61.271 Y123.979 E0.31617
G1 X61.271 Y123.452 E0.01406
G1 X52.606 Y132.117 E0.32718
G1 X52.315 Y131.882 E0.01000
G1 X61.271 Y122.926 E0.33819
G1 X61.271 Y122.399 E0.01406
```

```
G1 X52.002 Y131.668 E0.35000
G1 X51.677 Y131.467 E0.01021
G1 X60.824 Y122.320 E0.34540
G1 X60.298 Y122.320 E0.01406
G1 X51.342 Y131.276 E0.33818
G1 X50.981 Y131.110 E0.01060
G1 X59.771 Y122.320 E0.33191
G1 X59.245 Y122.320 E0.01406
G1 X50.611 Y130.954 E0.32602
G1 X50.216 Y130.822 E0.01111
G1 X58.718 Y122.320 E0.32104
G1 X58.192 Y122.320 E0.01406
G1 X49.821 Y130.690 E0.31607
G1 X49.382 Y130.602 E0.01195
G1 X57.665 Y122.320 E0.31275
G1 X57.139 Y122.320 E0.01406
G1 X48.939 Y130.520 E0.30962
G1 X48.448 Y130.484 E0.01313
G1 X56.612 Y122.320 E0.30826
G1 X56.086 Y122.320 E0.01406
G1 X47.948 Y130.457 E0.30728
G1 X47.421 Y130.457 E0.01406
G1 X51.408 Y126.471 E0.15053
G1 X52.121 Y126.035 E0.02230
G1 X52.904 Y125.422 E0.02656
G1 X53.532 Y124.772 E0.02414
G1 X54.073 Y124.055 E0.02397
G1 X54.411 Y123.468 E0.01810
G1 X55.559 Y122.320 E0.04335
```

G1 X55.033 Y122.320 E0.01406

G1 X54.535 Y122.817 E0.01878

M204 S1250

G1 X50.770 Y126.583 F9000.000

M204 S1000

G1 F1450.455

G1 X46.895 Y130.457 E0.14631

G1 X46.368 Y130.457 E0.01406

G1 X49.713 Y127.113 E0.12629

G1 X49.065 Y127.234 E0.01759

G1 X45.842 Y130.457 E0.12171

G1 X45.315 Y130.457 E0.01406

G1 X48.481 Y127.292 E0.11954

G1 X47.927 Y127.320 E0.01483

G1 X44.789 Y130.457 E0.11848

G1 X44.262 Y130.457 E0.01406

G1 X47.400 Y127.320 E0.11848

G1 X46.874 Y127.320 E0.01406

G1 X43.736 Y130.457 E0.11848

G1 X43.209 Y130.457 E0.01406

G1 X46.347 Y127.320 E0.11848

G1 X45.821 Y127.320 E0.01406

G1 X42.683 Y130.457 E0.11848

G1 X42.156 Y130.457 E0.01406

G1 X45.294 Y127.320 E0.11848

G1 X44.768 Y127.320 E0.01406

G1 X41.630 Y130.457 E0.11848

G1 X41.103 Y130.457 E0.01406

G1 X44.241 Y127.320 E0.11848

G1 X43.715 Y127.320 E0.01406

G1 X40.577 Y130.457 E0.11848

G1 X40.134 Y130.457 E0.01184

G1 X40.134 Y130.374 E0.00222

G1 X43.188 Y127.320 E0.11534

G1 X42.662 Y127.320 E0.01406

G1 X40.134 Y129.848 E0.09546

G1 X40.134 Y129.321 E0.01406

G1 X42.135 Y127.320 E0.07557

G1 X41.608 Y127.320 E0.01406

G1 X40.134 Y128.795 E0.05569

G1 X40.134 Y128.268 E0.01406

G1 X41.082 Y127.320 E0.03581

G1 X40.555 Y127.320 E0.01406

G1 X39.968 Y127.907 E0.02218

M204 S1250

; stop printing object Petg print.STL id:16 copy 0

; printing object Petg print.STL id:12 copy 0

G1 E-4.00000 F2400.000

G1 X39.968 Y112.092 F9000.000

G1 E4.00000 F900.000

M204 S800

;TYPE:Perimeter

;WIDTH:0.45

G1 F1292.454

G1 X39.860 Y112.092 E0.00323

G1 X39.860 Y108.377 E0.11130

G1 X48.032 Y108.377 E0.24487

G1 X48.873 Y108.326 E0.02523

```
G1 X49.720 Y108.167 E0.02583
G1 X50.542 Y107.905 E0.02583
G1 X51.325 Y107.545 E0.02583
G1 X51.970 Y107.145 E0.02277
G1 X52.725 Y106.553 E0.02874
G1 X53.328 Y105.930 E0.02596
G1 X53.847 Y105.242 E0.02583
G1 X54.277 Y104.495 E0.02583
G1 X54.612 Y103.701 E0.02583
G1 X54.704 Y103.377 E0.01007
G1 X61.575 Y103.377 E0.20589
G1 X61.575 Y117.092 E0.41094
G1 X54.704 Y117.092 E0.20589
G1 X54.612 Y116.768 E0.01007
G1 X54.277 Y115.974 E0.02583
G1 X53.847 Y115.227 E0.02583
G1 X53.328 Y114.539 E0.02583
G1 X52.728 Y113.920 E0.02583
G1 X52.057 Y113.379 E0.02583
G1 X51.325 Y112.924 E0.02583
G1 X50.634 Y112.607 E0.02277
G1 X49.725 Y112.303 E0.02874
G1 X48.873 Y112.144 E0.02596
G1 X48.032 Y112.092 E0.02523
G1 X40.028 Y112.092 E0.23985
M204 S1250
G1 X39.442 Y112.510 F9000.000
M204 S800
;TYPE:External perimeter
```

G1 F1292.454

G1 X39.442 Y107.960 E0.13634

G1 X48.020 Y107.960 E0.25701

G1 X48.821 Y107.910 E0.02407

G1 X49.618 Y107.761 E0.02428

G1 X50.390 Y107.515 E0.02428

G1 X51.126 Y107.176 E0.02428

G1 X51.731 Y106.802 E0.02131

G1 X52.445 Y106.242 E0.02719

G1 X53.010 Y105.658 E0.02433

G1 X53.498 Y105.011 E0.02428

G1 X53.902 Y104.309 E0.02428

G1 X54.217 Y103.562 E0.02428

G1 X54.388 Y102.960 E0.01877

G1 X61.992 Y102.960 E0.22787

G1 X61.992 Y117.510 E0.43598

G1 X54.388 Y117.510 E0.22787

G1 X54.217 Y116.907 E0.01877

G1 X53.902 Y116.160 E0.02428

G1 X53.498 Y115.458 E0.02428

G1 X53.010 Y114.811 E0.02428

G1 X52.446 Y114.229 E0.02428

G1 X51.815 Y113.720 E0.02428

G1 X51.126 Y113.293 E0.02428

G1 X50.481 Y112.996 E0.02131

G1 X49.620 Y112.709 E0.02719

G1 X48.821 Y112.559 E0.02433

G1 X48.020 Y112.510 E0.02407

G1 X39.502 Y112.510 E0.25521

```
M204 S1250
G1 X39.643 Y112.164 F9000.000
G1 E-4.00000 F2400.000
G1 X61.452 Y116.216 F9000.000
G1 E4.00000 F900.000
M204 S1000
;TYPE:Top solid infill
;WIDTH:0.404487
G1 F1450.455
G1 X60.864 Y116.804 E0.02219
G1 X60.338 Y116.804 E0.01406
G1 X61.286 Y115.855 E0.03582
G1 X61.286 Y115.328 E0.01406
G1 X59.811 Y116.804 E0.05570
G1 X59.285 Y116.804 E0.01406
G1 X61.286 Y114.802 E0.07558
G1 X61.286 Y114.275 E0.01406
G1 X58.758 Y116.804 E0.09546
G1 X58.232 Y116.804 E0.01406
G1 X61.286 Y113.749 E0.11534
G1 X61.286 Y113.222 E0.01406
G1 X57.705 Y116.804 E0.13522
G1 X57.179 Y116.804 E0.01406
G1 X61.286 Y112.696 E0.15510
G1 X61.286 Y112.169 E0.01406
G1 X56.652 Y116.804 E0.17498
G1 X56.126 Y116.804 E0.01406
G1 X61.286 Y111.643 E0.19486
G1 X61.286 Y111.116 E0.01406
```

G1 X55.599 Y116.804 E0.21474

G1 X55.073 Y116.804 E0.01406

G1 X61.286 Y110.590 E0.23462

G1 X61.286 Y110.063 E0.01406

G1 X54.823 Y116.527 E0.24405

G1 X54.667 Y116.156 E0.01073

G1 X61.286 Y109.537 E0.24995

M73 P93 R5

G1 X61.286 Y109.010 E0.01406

G1 X54.505 Y115.792 E0.25607

G1 X54.312 Y115.458 E0.01029

G1 X61.286 Y108.484 E0.26334

G1 X61.286 Y107.957 E0.01406

G1 X54.120 Y115.124 E0.27060

G1 X53.899 Y114.818 E0.01007

G1 X61.286 Y107.431 E0.27893

G1 X61.286 Y106.904 E0.01406

G1 X53.673 Y114.518 E0.28748

G1 X53.432 Y114.232 E0.00998

G1 X61.286 Y106.378 E0.29657

G1 X61.286 Y105.851 E0.01406

G1 X53.173 Y113.964 E0.30635

G1 X52.913 Y113.698 E0.00994

G1 X61.286 Y105.325 E0.31617

G1 X61.286 Y104.798 E0.01406

G1 X52.622 Y113.463 E0.32718

G1 X52.330 Y113.228 E0.01000

G1 X61.286 Y104.272 E0.33819

G1 X61.286 Y103.745 E0.01406

G1 X52.017 Y113.015 E0.35000

G1 X51.692 Y112.813 E0.01021

G1 X60.839 Y103.666 E0.34540

G1 X60.313 Y103.666 E0.01406

G1 X51.357 Y112.622 E0.33818

G1 X50.996 Y112.456 E0.01060

G1 X59.786 Y103.666 E0.33191

G1 X59.260 Y103.666 E0.01406

G1 X50.626 Y112.300 E0.32602

G1 X50.231 Y112.168 E0.01111

G1 X58.733 Y103.666 E0.32104

G1 X58.207 Y103.666 E0.01406

G1 X49.836 Y112.036 E0.31607

G1 X49.398 Y111.949 E0.01195

G1 X57.680 Y103.666 E0.31275

G1 X57.154 Y103.666 E0.01406

G1 X48.954 Y111.866 E0.30962

G1 X48.463 Y111.830 E0.01313

G1 X56.627 Y103.666 E0.30826

G1 X56.101 Y103.666 E0.01406

G1 X47.963 Y111.804 E0.30728

G1 X47.437 Y111.804 E0.01406

G1 X51.423 Y107.817 E0.15053

G1 X52.136 Y107.381 E0.02230

G1 X52.919 Y106.768 E0.02656

G1 X53.547 Y106.118 E0.02414

G1 X54.088 Y105.401 E0.02397

G1 X54.426 Y104.814 E0.01810

G1 X55.574 Y103.666 E0.04335

```
G1 X55.048 Y103.666 E0.01406
G1 X54.550 Y104.163 E0.01878
M204 S1250
G1 X50.785 Y107.929 F9000.000
M204 S1000
G1 F1450.455
G1 X46.910 Y111.804 E0.14631
G1 X46.384 Y111.804 E0.01406
G1 X49.728 Y108.459 E0.12629
G1 X49.080 Y108.580 E0.01759
G1 X45.857 Y111.804 E0.12171
G1 X45.331 Y111.804 E0.01406
G1 X48.496 Y108.638 E0.11954
G1 X47.942 Y108.666 E0.01483
G1 X44.804 Y111.804 E0.11848
G1 X44.277 Y111.804 E0.01406
G1 X47.415 Y108.666 E0.11848
G1 X46.889 Y108.666 E0.01406
G1 X43.751 Y111.804 E0.11848
G1 X43.224 Y111.804 E0.01406
G1 X46.362 Y108.666 E0.11848
G1 X45.836 Y108.666 E0.01406
G1 X42.698 Y111.804 E0.11848
G1 X42.171 Y111.804 E0.01406
G1 X45.309 Y108.666 E0.11848
G1 X44.783 Y108.666 E0.01406
G1 X41.645 Y111.804 E0.11848
G1 X41.118 Y111.804 E0.01406
G1 X44.256 Y108.666 E0.11848
```

```
G1 X43.730 Y108.666 E0.01406
G1 X40.592 Y111.804 E0.11848
G1 X40.149 Y111.804 E0.01184
G1 X40.149 Y111.720 E0.00222
G1 X43.203 Y108.666 E0.11534
G1 X42.677 Y108.666 E0.01406
G1 X40.149 Y111.194 E0.09546
G1 X40.149 Y110.667 E0.01406
G1 X42.150 Y108.666 E0.07557
G1 X41.624 Y108.666 E0.01406
G1 X40.149 Y110.141 E0.05569
G1 X40.149 Y109.614 E0.01406
G1 X41.097 Y108.666 E0.03581
G1 X40.571 Y108.666 E0.01406
G1 X39.983 Y109.253 E0.02218
M204 S1250
; stop printing object Petg print.STL id:12 copy 0
; printing object tpu print.STL id:7 copy 0
; stop printing object tpu print.STL id:7 copy 0
; printing object tpu print.STL id:3 copy 0
; stop printing object tpu print.STL id:3 copy 0
; printing object Petg print.STL id:2 copy 0
G1 E-4.00000 F2400.000
G1 X89.650 Y108.958 F9000.000
G1 E4.00000 F900.000
M204 S800
;TYPE:Perimeter
;WIDTH:0.45
G1 F1292.454
```

```
G1 X97.822 Y108.958 E0.24487
M73 P93 R4
G1 X98.662 Y108.906 E0.02523
G1 X99.510 Y108.748 E0.02583
G1 X100.331 Y108.486 E0.02583
G1 X101.114 Y108.126 E0.02583
G1 X101.760 Y107.725 E0.02277
G1 X102.515 Y107.134 E0.02874
G1 X103.117 Y106.511 E0.02596
G1 X103.636 Y105.823 E0.02583
G1 X104.066 Y105.076 E0.02583
G1 X104.401 Y104.282 E0.02583
G1 X104.493 Y103.958 E0.01007
G1 X111.364 Y103.958 E0.20589
G1 X111.364 Y117.673 E0.41094
G1 X104.493 Y117.673 E0.20589
G1 X104.401 Y117.349 E0.01007
G1 X104.066 Y116.555 E0.02583
G1 X103.636 Y115.808 E0.02583
G1 X103.117 Y115.120 E0.02583
G1 X102.518 Y114.500 E0.02583
G1 X101.847 Y113.960 E0.02583
G1 X101.114 Y113.505 E0.02583
G1 X100.424 Y113.188 E0.02277
G1 X99.514 Y112.884 E0.02874
G1 X98.662 Y112.725 E0.02596
G1 X97.822 Y112.673 E0.02523
G1 X89.650 Y112.673 E0.24487
G1 X89.650 Y109.018 E0.10950
```

```
M204 S1250
G1 X89.232 Y108.541 F9000.000
M204 S800
;TYPE:External perimeter
G1 F1292.454
G1 X97.809 Y108.541 E0.25701
G1 X98.611 Y108.491 E0.02407
G1 X99.407 Y108.342 E0.02428
G1 X100.180 Y108.096 E0.02428
G1 X100.916 Y107.757 E0.02428
G1 X101.520 Y107.382 E0.02131
G1 X102.234 Y106.823 E0.02719
G1 X102.799 Y106.239 E0.02433
G1 X103.287 Y105.592 E0.02428
G1 X103.691 Y104.890 E0.02428
G1 X104.006 Y104.143 E0.02428
G1 X104.177 Y103.541 E0.01877
G1 X111.782 Y103.541 E0.22787
G1 X111.782 Y118.091 E0.43598
G1 X104.177 Y118.091 E0.22787
G1 X104.006 Y117.488 E0.01877
G1 X103.691 Y116.741 E0.02428
G1 X103.287 Y116.039 E0.02428
G1 X102.799 Y115.392 E0.02428
G1 X102.235 Y114.810 E0.02428
G1 X101.604 Y114.301 E0.02428
G1 X100.916 Y113.874 E0.02428
G1 X100.270 Y113.577 E0.02131
G1 X99.409 Y113.289 E0.02719
```

```
G1 X98.611 Y113.140 E0.02433
G1 X97.809 Y113.091 E0.02407
G1 X89.232 Y113.091 E0.25701
G1 X89.232 Y108.601 E0.13454
M204 S1250
G1 X89.627 Y108.605 F9000.000
G1 E-4.00000 F2400.000
G1 X111.241 Y116.797 F9000.000
G1 E4.00000 F900.000
M204 S1000
;TYPE:Top solid infill
;WIDTH:0.404487
G1 F1450.455
G1 X110.654 Y117.384 E0.02219
G1 X110.127 Y117.384 E0.01406
G1 X111.076 Y116.436 E0.03582
G1 X111.076 Y115.909 E0.01406
G1 X109.601 Y117.384 E0.05570
G1 X109.074 Y117.384 E0.01406
G1 X111.076 Y115.383 E0.07558
G1 X111.076 Y114.856 E0.01406
G1 X108.548 Y117.384 E0.09546
G1 X108.021 Y117.384 E0.01406
G1 X111.076 Y114.330 E0.11534
G1 X111.076 Y113.803 E0.01406
G1 X107.495 Y117.384 E0.13522
G1 X106.968 Y117.384 E0.01406
G1 X111.076 Y113.277 E0.15510
G1 X111.076 Y112.750 E0.01406
```

G1 X106.442 Y117.384 E0.17498

G1 X105.915 Y117.384 E0.01406

G1 X111.076 Y112.224 E0.19486

G1 X111.076 Y111.697 E0.01406

G1 X105.389 Y117.384 E0.21474

G1 X104.862 Y117.384 E0.01406

G1 X111.076 Y111.171 E0.23462

G1 X111.076 Y110.644 E0.01406

G1 X104.612 Y117.108 E0.24405

G1 X104.456 Y116.737 E0.01073

G1 X111.076 Y110.118 E0.24995

G1 X111.076 Y109.591 E0.01406

G1 X104.294 Y116.373 E0.25607

G1 X104.102 Y116.039 E0.01029

G1 X111.076 Y109.065 E0.26334

G1 X111.076 Y108.538 E0.01406

G1 X103.909 Y115.705 E0.27060

G1 X103.689 Y115.399 E0.01007

G1 X111.076 Y108.012 E0.27893

G1 X111.076 Y107.485 E0.01406

G1 X103.462 Y115.099 E0.28748

G1 X103.222 Y114.813 E0.00998

G1 X111.076 Y106.959 E0.29657

G1 X111.076 Y106.432 E0.01406

G1 X102.963 Y114.545 E0.30635

G1 X102.702 Y114.279 E0.00994

G1 X111.076 Y105.906 E0.31617

G1 X111.076 Y105.379 E0.01406

G1 X102.411 Y114.044 E0.32718

```
G1 X102.119 Y113.809 E0.01000
G1 X111.076 Y104.853 E0.33819
G1 X111.076 Y104.326 E0.01406
G1 X101.806 Y113.595 E0.35000
G1 X101.481 Y113.394 E0.01021
G1 X110.629 Y104.247 E0.34540
G1 X110.102 Y104.247 E0.01406
G1 X101.146 Y113.203 E0.33818
G1 X100.786 Y113.037 E0.01060
G1 X109.576 Y104.247 E0.33191
G1 X109.049 Y104.247 E0.01406
G1 X100.415 Y112.881 E0.32602
G1 X100.020 Y112.749 E0.01111
G1 X108.523 Y104.247 E0.32104
G1 X107.996 Y104.247 E0.01406
G1 X99.626 Y112.617 E0.31607
G1 X99.187 Y112.529 E0.01195
G1 X107.470 Y104.247 E0.31275
G1 X106.943 Y104.247 E0.01406
G1 X98.743 Y112.446 E0.30962
G1 X98.253 Y112.410 E0.01313
G1 X106.417 Y104.247 E0.30826
G1 X105.890 Y104.247 E0.01406
G1 X97.752 Y112.384 E0.30728
G1 X97.226 Y112.384 E0.01406
G1 X101.213 Y108.398 E0.15053
G1 X101.925 Y107.962 E0.02230
G1 X102.708 Y107.349 E0.02656
G1 X103.337 Y106.699 E0.02414
```

```
G1 X103.877 Y105.982 E0.02397
G1 X104.215 Y105.395 E0.01810
G1 X105.364 Y104.247 E0.04335
G1 X104.837 Y104.247 E0.01406
G1 X104.340 Y104.744 E0.01878
M204 S1250
G1 X100.574 Y108.510 F9000.000
M204 S1000
G1 F1450.455
G1 X96.699 Y112.384 E0.14631
G1 X96.173 Y112.384 E0.01406
G1 X99.517 Y109.040 E0.12629
G1 X98.870 Y109.161 E0.01759
G1 X95.646 Y112.384 E0.12171
G1 X95.120 Y112.384 E0.01406
G1 X98.286 Y109.219 E0.11954
G1 X97.731 Y109.247 E0.01483
G1 X94.593 Y112.384 E0.11848
G1 X94.067 Y112.384 E0.01406
G1 X97.205 Y109.247 E0.11848
G1 X96.678 Y109.247 E0.01406
G1 X93.540 Y112.384 E0.11848
G1 X93.014 Y112.384 E0.01406
G1 X96.152 Y109.247 E0.11848
G1 X95.625 Y109.247 E0.01406
G1 X92.487 Y112.384 E0.11848
G1 X91.961 Y112.384 E0.01406
G1 X95.099 Y109.247 E0.11848
G1 X94.572 Y109.247 E0.01406
```

```
G1 X91.434 Y112.384 E0.11848
M73 P94 R4
G1 X90.908 Y112.384 E0.01406
G1 X94.045 Y109.247 E0.11848
G1 X93.519 Y109.247 E0.01406
G1 X90.381 Y112.384 E0.11848
G1 X89.938 Y112.384 E0.01184
G1 X89.938 Y112.301 E0.00222
G1 X92.992 Y109.247 E0.11534
G1 X92.466 Y109.247 E0.01406
G1 X89.938 Y111.775 E0.09546
G1 X89.938 Y111.248 E0.01406
G1 X91.939 Y109.247 E0.07557
G1 X91.413 Y109.247 E0.01406
G1 X89.938 Y110.722 E0.05569
G1 X89.938 Y110.195 E0.01406
G1 X90.886 Y109.247 E0.03581
G1 X90.360 Y109.247 E0.01406
G1 X89.772 Y109.834 E0.02218
M204 S1250
; stop printing object Petg print.STL id:2 copy 0
; printing object Petg print.STL id:6 copy 0
G1 E-4.00000 F2400.000
G1 X89.772 Y127.612 F9000.000
G1 E4.00000 F900.000
M204 S800
;TYPE:Perimeter
;WIDTH:0.45
G1 F1292.454
```

```
G1 X97.807 Y127.612 E0.24074
G1 X98.647 Y127.560 E0.02523
G1 X99.495 Y127.402 E0.02583
G1 X100.316 Y127.140 E0.02583
G1 X101.099 Y126.780 E0.02583
G1 X101.745 Y126.379 E0.02277
G1 X102.500 Y125.788 E0.02874
G1 X103.102 Y125.165 E0.02596
G1 X103.621 Y124.477 E0.02583
G1 X104.051 Y123.730 E0.02583
G1 X104.386 Y122.936 E0.02583
G1 X104.478 Y122.612 E0.01007
G1 X111.349 Y122.612 E0.20589
G1 X111.349 Y136.327 E0.41094
G1 X104.478 Y136.327 E0.20589
G1 X104.386 Y136.003 E0.01007
G1 X104.051 Y135.209 E0.02583
G1 X103.621 Y134.462 E0.02583
G1 X103.102 Y133.774 E0.02583
G1 X102.503 Y133.154 E0.02583
G1 X101.831 Y132.613 E0.02583
G1 X101.099 Y132.159 E0.02583
G1 X100.409 Y131.841 E0.02277
G1 X99.499 Y131.538 E0.02874
G1 X98.647 Y131.379 E0.02596
G1 X97.807 Y131.327 E0.02523
G1 X89.635 Y131.327 E0.24487
G1 X89.635 Y127.612 E0.11130
G1 X89.712 Y127.612 E0.00234
```

```
M204 S1250
G1 X89.217 Y127.194 F9000.000
M204 S800
;TYPE:External perimeter
G1 F1292.454
G1 X97.794 Y127.194 E0.25701
G1 X98.596 Y127.145 E0.02407
G1 X99.392 Y126.996 E0.02428
G1 X100.164 Y126.750 E0.02428
G1 X100.901 Y126.411 E0.02428
G1 X101.505 Y126.036 E0.02131
G1 X102.219 Y125.477 E0.02719
G1 X102.784 Y124.893 E0.02433
G1 X103.272 Y124.246 E0.02428
G1 X103.676 Y123.544 E0.02428
G1 X103.991 Y122.797 E0.02428
G1 X104.162 Y122.194 E0.01877
G1 X111.767 Y122.194 E0.22787
G1 X111.767 Y136.744 E0.43598
G1 X104.162 Y136.744 E0.22787
G1 X103.991 Y136.142 E0.01877
G1 X103.676 Y135.395 E0.02428
G1 X103.272 Y134.693 E0.02428
G1 X102.784 Y134.046 E0.02428
G1 X102.220 Y133.463 E0.02428
G1 X101.589 Y132.955 E0.02428
G1 X100.901 Y132.528 E0.02428
G1 X100.255 Y132.231 E0.02131
G1 X99.394 Y131.943 E0.02719
```

```
G1 X98.596 Y131.794 E0.02433
G1 X97.794 Y131.744 E0.02407
G1 X89.217 Y131.744 E0.25701
G1 X89.217 Y127.254 E0.13454
M204 S1250
G1 X89.611 Y127.259 F9000.000
G1 E-4.00000 F2400.000
G1 X111.226 Y135.451 F9000.000
G1 E4.00000 F900.000
M204 S1000
;TYPE:Top solid infill
;WIDTH:0.404487
G1 F1450.455
G1 X110.639 Y136.038 E0.02219
G1 X110.112 Y136.038 E0.01406
G1 X111.061 Y135.090 E0.03582
G1 X111.061 Y134.563 E0.01406
G1 X109.586 Y136.038 E0.05570
G1 X109.059 Y136.038 E0.01406
G1 X111.061 Y134.037 E0.07558
G1 X111.061 Y133.510 E0.01406
G1 X108.533 Y136.038 E0.09546
G1 X108.006 Y136.038 E0.01406
G1 X111.061 Y132.984 E0.11534
G1 X111.061 Y132.457 E0.01406
G1 X107.480 Y136.038 E0.13522
G1 X106.953 Y136.038 E0.01406
G1 X111.061 Y131.931 E0.15510
G1 X111.061 Y131.404 E0.01406
```

```
G1 X106.426 Y136.038 E0.17498
G1 X105.900 Y136.038 E0.01406
G1 X111.061 Y130.878 E0.19486
G1 X111.061 Y130.351 E0.01406
G1 X105.373 Y136.038 E0.21474
G1 X104.847 Y136.038 E0.01406
G1 X111.061 Y129.825 E0.23462
G1 X111.061 Y129.298 E0.01406
G1 X104.597 Y135.762 E0.24405
G1 X104.441 Y135.391 E0.01073
G1 X111.061 Y128.772 E0.24995
G1 X111.061 Y128.245 E0.01406
G1 X104.279 Y135.027 E0.25607
G1 X104.087 Y134.693 E0.01029
G1 X111.061 Y127.719 E0.26334
G1 X111.061 Y127.192 E0.01406
G1 X103.894 Y134.359 E0.27060
G1 X103.674 Y134.053 E0.01007
G1 X111.061 Y126.666 E0.27893
G1 X111.061 Y126.139 E0.01406
G1 X103.447 Y133.752 E0.28748
G1 X103.206 Y133.467 E0.00998
G1 X111.061 Y125.613 E0.29657
G1 X111.061 Y125.086 E0.01406
G1 X102.947 Y133.199 E0.30635
G1 X102.687 Y132.933 E0.00994
G1 X111.061 Y124.560 E0.31617
G1 X111.061 Y124.033 E0.01406
G1 X102.396 Y132.698 E0.32718
```

G1 X102.104 Y132.463 E0.01000

G1 X111.061 Y123.507 E0.33819

G1 X111.061 Y122.980 E0.01406

G1 X101.791 Y132.249 E0.35000

G1 X101.466 Y132.048 E0.01021

G1 X110.614 Y122.901 E0.34540

G1 X110.087 Y122.901 E0.01406

G1 X101.131 Y131.857 E0.33818

G1 X100.771 Y131.691 E0.01060

G1 X109.561 Y122.901 E0.33191

G1 X109.034 Y122.901 E0.01406

G1 X100.400 Y131.535 E0.32602

G1 X100.005 Y131.403 E0.01111

G1 X108.508 Y122.901 E0.32104

G1 X107.981 Y122.901 E0.01406

G1 X99.610 Y131.271 E0.31607

G1 X99.172 Y131.183 E0.01195

G1 X107.455 Y122.901 E0.31275

G1 X106.928 Y122.901 E0.01406

G1 X98.728 Y131.100 E0.30962

G1 X98.238 Y131.064 E0.01313

G1 X106.401 Y122.901 E0.30826

G1 X105.875 Y122.901 E0.01406

G1 X97.737 Y131.038 E0.30728

G1 X97.211 Y131.038 E0.01406

G1 X101.197 Y127.052 E0.15053

G1 X101.910 Y126.616 E0.02230

G1 X102.693 Y126.003 E0.02656

G1 X103.322 Y125.353 E0.02414

```
G1 X103.862 Y124.636 E0.02397
G1 X104.200 Y124.049 E0.01810
G1 X105.348 Y122.901 E0.04335
G1 X104.822 Y122.901 E0.01406
G1 X104.325 Y123.398 E0.01878
M204 S1250
G1 X100.559 Y127.163 F9000.000
M204 S1000
G1 F1450.455
G1 X96.684 Y131.038 E0.14631
G1 X96.158 Y131.038 E0.01406
G1 X99.502 Y127.694 E0.12629
G1 X98.855 Y127.815 E0.01759
G1 X95.631 Y131.038 E0.12171
G1 X95.105 Y131.038 E0.01406
G1 X98.271 Y127.873 E0.11954
G1 X97.716 Y127.901 E0.01483
G1 X94.578 Y131.038 E0.11848
G1 X94.052 Y131.038 E0.01406
G1 X97.189 Y127.901 E0.11848
G1 X96.663 Y127.901 E0.01406
G1 X93.525 Y131.038 E0.11848
G1 X92.999 Y131.038 E0.01406
G1 X96.136 Y127.901 E0.11848
G1 X95.610 Y127.901 E0.01406
G1 X92.472 Y131.038 E0.11848
G1 X91.946 Y131.038 E0.01406
G1 X95.083 Y127.901 E0.11848
G1 X94.557 Y127.901 E0.01406
```

```
G1 X91.419 Y131.038 E0.11848
G1 X90.893 Y131.038 E0.01406
G1 X94.030 Y127.901 E0.11848
G1 X93.504 Y127.901 E0.01406
G1 X90.366 Y131.038 E0.11848
G1 X89.923 Y131.038 E0.01184
G1 X89.923 Y130.955 E0.00222
G1 X92.977 Y127.901 E0.11534
G1 X92.451 Y127.901 E0.01406
G1 X89.923 Y130.429 E0.09546
G1 X89.923 Y129.902 E0.01406
G1 X91.924 Y127.901 E0.07557
G1 X91.398 Y127.901 E0.01406
G1 X89.923 Y129.376 E0.05569
G1 X89.923 Y128.849 E0.01406
G1 X90.871 Y127.901 E0.03581
G1 X90.345 Y127.901 E0.01406
G1 X89.757 Y128.488 E0.02218
M204 S1250
; stop printing object Petg print.STL id:6 copy 0
; printing object tpu print.STL id:27 copy 0
; stop printing object tpu print.STL id:27 copy 0
; printing object tpu print.STL id:23 copy 0
; stop printing object tpu print.STL id:23 copy 0
; printing object tpu print.STL id:21 copy 0
; stop printing object tpu print.STL id:21 copy 0
; printing object Petg print.STL id:20 copy 0
G1 E-4.00000 F2400.000
G1 X141.293 Y92.032 F9000.000
```

```
G1 E4.00000 F900.000
M204 S800
;TYPE:Perimeter
;WIDTH:0.45
G1 F1292.454
G1 X141.293 Y88.318 E0.11130
G1 X149.465 Y88.318 E0.24487
G1 X150.306 Y88.266 E0.02523
G1 X151.153 Y88.107 E0.02583
G1 X151.974 Y87.846 E0.02583
G1 X152.757 Y87.485 E0.02583
G1 X153.403 Y87.085 E0.02277
G1 X154.158 Y86.493 E0.02874
G1 X154.761 Y85.871 E0.02596
G1 X155.279 Y85.183 E0.02583
G1 X155.710 Y84.436 E0.02583
G1 X156.045 Y83.641 E0.02583
G1 X156.136 Y83.318 E0.01007
G1 X163.007 Y83.318 E0.20589
G1 X163.007 Y97.032 E0.41094
G1 X156.136 Y97.032 E0.20589
G1 X156.045 Y96.709 E0.01007
G1 X155.710 Y95.915 E0.02583
G1 X155.279 Y95.168 E0.02583
G1 X154.761 Y94.479 E0.02583
G1 X154.161 Y93.860 E0.02583
G1 X153.490 Y93.319 E0.02583
G1 X152.757 Y92.865 E0.02583
G1 X152.067 Y92.547 E0.02277
```

```
G1 X151.157 Y92.244 E0.02874
G1 X150.306 Y92.084 E0.02596
G1 X149.465 Y92.032 E0.02523
G1 X141.353 Y92.032 E0.24307
M204 S1250
G1 X140.875 Y92.450 F9000.000
M204 S800
;TYPE:External perimeter
G1 F1292.454
G1 X140.875 Y87.900 E0.13634
G1 X149.452 Y87.900 E0.25701
G1 X150.254 Y87.851 E0.02407
G1 X151.051 Y87.702 E0.02428
G1 X151.823 Y87.456 E0.02428
G1 X152.559 Y87.117 E0.02428
G1 X153.163 Y86.742 E0.02131
G1 X153.878 Y86.182 E0.02719
G1 X154.443 Y85.599 E0.02433
G1 X154.930 Y84.952 E0.02428
G1 X155.335 Y84.249 E0.02428
G1 X155.650 Y83.503 E0.02428
G1 X155.820 Y82.900 E0.01877
G1 X163.425 Y82.900 E0.22787
G1 X163.425 Y97.450 E0.43598
G1 X155.820 Y97.450 E0.22787
G1 X155.650 Y96.847 E0.01877
G1 X155.335 Y96.101 E0.02428
G1 X154.930 Y95.398 E0.02428
G1 X154.443 Y94.751 E0.02428
```

```
G1 X153.879 Y94.169 E0.02428
G1 X153.248 Y93.661 E0.02428
G1 X152.559 Y93.234 E0.02428
G1 X151.913 Y92.936 E0.02131
G1 X151.052 Y92.649 E0.02719
G1 X150.254 Y92.500 E0.02433
G1 X149.452 Y92.450 E0.02407
G1 X140.935 Y92.450 E0.25521
M204 S1250
G1 X141.076 Y92.104 F9000.000
G1 E-4.00000 F2400.000
G1 X162.885 Y96.156 F9000.000
G1 E4.00000 F900.000
M204 S1000
;TYPE:Top solid infill
;WIDTH:0.404487
M73 P94 R3
G1 F1450.455
G1 X162.297 Y96.744 E0.02219
G1 X161.771 Y96.744 E0.01406
G1 X162.719 Y95.795 E0.03582
G1 X162.719 Y95.269 E0.01406
G1 X161.244 Y96.744 E0.05570
G1 X160.718 Y96.744 E0.01406
G1 X162.719 Y94.742 E0.07558
G1 X162.719 Y94.216 E0.01406
G1 X160.191 Y96.744 E0.09546
G1 X159.665 Y96.744 E0.01406
G1 X162.719 Y93.689 E0.11534
```

```
G1 X162.719 Y93.163 E0.01406
G1 X159.138 Y96.744 E0.13522
G1 X158.612 Y96.744 E0.01406
G1 X162.719 Y92.636 E0.15510
G1 X162.719 Y92.110 E0.01406
G1 X158.085 Y96.744 E0.17498
G1 X157.559 Y96.744 E0.01406
G1 X162.719 Y91.583 E0.19486
G1 X162.719 Y91.057 E0.01406
G1 X157.032 Y96.744 E0.21474
G1 X156.505 Y96.744 E0.01406
G1 X162.719 Y90.530 E0.23462
G1 X162.719 Y90.004 E0.01406
G1 X156.256 Y96.467 E0.24405
G1 X156.100 Y96.097 E0.01073
G1 X162.719 Y89.477 E0.24995
G1 X162.719 Y88.951 E0.01406
G1 X155.938 Y95.732 E0.25607
G1 X155.745 Y95.398 E0.01029
G1 X162.719 Y88.424 E0.26334
G1 X162.719 Y87.898 E0.01406
G1 X155.553 Y95.064 E0.27060
G1 X155.332 Y94.758 E0.01007
G1 X162.719 Y87.371 E0.27893
M73 P95 R3
G1 X162.719 Y86.845 E0.01406
G1 X155.106 Y94.458 E0.28748
G1 X154.865 Y94.172 E0.00998
G1 X162.719 Y86.318 E0.29657
```

```
G1 X162.719 Y85.792 E0.01406
G1 X154.606 Y93.905 E0.30635
G1 X154.346 Y93.638 E0.00994
G1 X162.719 Y85.265 E0.31617
G1 X162.719 Y84.739 E0.01406
G1 X154.054 Y93.404 E0.32718
G1 X153.763 Y93.169 E0.01000
G1 X162.719 Y84.212 E0.33819
G1 X162.719 Y83.686 E0.01406
G1 X153.450 Y92.955 E0.35000
G1 X153.125 Y92.753 E0.01021
G1 X162.272 Y83.606 E0.34540
G1 X161.746 Y83.606 E0.01406
G1 X152.790 Y92.562 E0.33818
G1 X152.429 Y92.396 E0.01060
G1 X161.219 Y83.606 E0.33191
G1 X160.693 Y83.606 E0.01406
G1 X152.059 Y92.240 E0.32602
G1 X151.664 Y92.108 E0.01111
G1 X160.166 Y83.606 E0.32104
G1 X159.640 Y83.606 E0.01406
G1 X151.269 Y91.977 E0.31607
G1 X150.830 Y91.889 E0.01195
G1 X159.113 Y83.606 E0.31275
G1 X158.587 Y83.606 E0.01406
G1 X150.387 Y91.806 E0.30962
G1 X149.896 Y91.770 E0.01313
G1 X158.060 Y83.606 E0.30826
G1 X157.534 Y83.606 E0.01406
```

G1 X149.396 Y91.744 E0.30728

G1 X148.869 Y91.744 E0.01406

G1 X152.856 Y87.757 E0.15053

G1 X153.568 Y87.322 E0.02230

G1 X154.351 Y86.708 E0.02656

G1 X154.980 Y86.059 E0.02414

G1 X155.520 Y85.342 E0.02397

G1 X155.859 Y84.754 E0.01810

G1 X157.007 Y83.606 E0.04335

G1 X156.480 Y83.606 E0.01406

G1 X155.983 Y84.104 E0.01878

M204 S1250

G1 X152.218 Y87.869 F9000.000

M204 S1000

G1 F1450.455

G1 X148.343 Y91.744 E0.14631

G1 X147.816 Y91.744 E0.01406

G1 X151.161 Y88.399 E0.12629

G1 X150.513 Y88.521 E0.01759

G1 X147.290 Y91.744 E0.12171

G1 X146.763 Y91.744 E0.01406

G1 X149.929 Y88.578 E0.11954

G1 X149.374 Y88.606 E0.01483

G1 X146.237 Y91.744 E0.11848

G1 X145.710 Y91.744 E0.01406

G1 X148.848 Y88.606 E0.11848

G1 X148.321 Y88.606 E0.01406

G1 X145.184 Y91.744 E0.11848

G1 X144.657 Y91.744 E0.01406

```
G1 X147.795 Y88.606 E0.11848
G1 X147.268 Y88.606 E0.01406
G1 X144.131 Y91.744 E0.11848
G1 X143.604 Y91.744 E0.01406
G1 X146.742 Y88.606 E0.11848
G1 X146.215 Y88.606 E0.01406
G1 X143.078 Y91.744 E0.11848
G1 X142.551 Y91.744 E0.01406
G1 X145.689 Y88.606 E0.11848
G1 X145.162 Y88.606 E0.01406
G1 X142.025 Y91.744 E0.11848
G1 X141.581 Y91.744 E0.01184
G1 X141.581 Y91.661 E0.00222
G1 X144.636 Y88.606 E0.11534
G1 X144.109 Y88.606 E0.01406
G1 X141.581 Y91.134 E0.09546
G1 X141.581 Y90.608 E0.01406
G1 X143.583 Y88.606 E0.07557
G1 X143.056 Y88.606 E0.01406
G1 X141.581 Y90.081 E0.05569
G1 X141.581 Y89.555 E0.01406
G1 X142.530 Y88.606 E0.03581
G1 X142.003 Y88.606 E0.01406
G1 X141.416 Y89.194 E0.02218
M204 S1250
; stop printing object Petg print.STL id:20 copy 0
; printing object Petg print.STL id:22 copy 0
G1 E-4.00000 F2400.000
G1 X141.416 Y109.133 F9000.000
```

```
G1 E4.00000 F900.000

M204 S800

;TYPE:Perimeter

;WIDTH:0.45

G1 F1292.454

G1 X149.472 Y109.133 E0.24140

G1 X150.313 Y109.082 E0.02523

G1 X151.160 Y108.923 E0.02583

G1 X151.981 Y108.661 E0.02583

G1 X152.764 Y108.301 E0.02583

G1 X153.410 Y107.900 E0.02277

G1 X154.165 Y107.309 E0.02874

G1 X154.768 Y106.686 E0.02596

G1 X155.286 Y105.998 E0.02583

G1 X155.717 Y105.251 E0.02583

G1 X156.052 Y104.457 E0.02583

G1 X156.143 Y104.133 E0.01007

G1 X163.014 Y104.133 E0.20589

G1 X163.014 Y117.848 E0.41094

G1 X156.143 Y117.848 E0.20589

G1 X156.052 Y117.524 E0.01007

G1 X155.717 Y116.730 E0.02583

G1 X155.286 Y115.983 E0.02583

G1 X154.768 Y115.295 E0.02583

G1 X154.168 Y114.675 E0.02583

G1 X153.497 Y114.135 E0.02583

G1 X152.764 Y113.680 E0.02583

G1 X152.074 Y113.363 E0.02277

G1 X151.164 Y113.059 E0.02874
```

```
G1 X150.313 Y112.900 E0.02596
G1 X149.472 Y112.848 E0.02523
G1 X141.300 Y112.848 E0.24487
G1 X141.300 Y109.133 E0.11130
G1 X141.356 Y109.133 E0.00168
M204 S1250
G1 X140.882 Y108.716 F9000.000
M204 S800
;TYPE:External perimeter
G1 F1292.454
G1 X149.459 Y108.716 E0.25701
G1 X150.261 Y108.666 E0.02407
G1 X151.058 Y108.517 E0.02428
G1 X151.830 Y108.271 E0.02428
G1 X152.566 Y107.932 E0.02428
G1 X153.170 Y107.557 E0.02131
G1 X153.885 Y106.998 E0.02719
G1 X154.449 Y106.414 E0.02433
G1 X154.937 Y105.767 E0.02428
G1 X155.342 Y105.065 E0.02428
G1 X155.657 Y104.318 E0.02428
G1 X155.827 Y103.716 E0.01877
G1 X163.432 Y103.716 E0.22787
G1 X163.432 Y118.266 E0.43598
G1 X155.827 Y118.266 E0.22787
G1 X155.657 Y117.663 E0.01877
G1 X155.342 Y116.916 E0.02428
G1 X154.937 Y116.214 E0.02428
G1 X154.449 Y115.567 E0.02428
```

```
G1 X153.886 Y114.985 E0.02428
G1 X153.255 Y114.476 E0.02428
G1 X152.566 Y114.049 E0.02428
G1 X151.920 Y113.752 E0.02131
G1 X151.059 Y113.464 E0.02719
G1 X150.261 Y113.315 E0.02433
G1 X149.459 Y113.266 E0.02407
G1 X140.882 Y113.266 E0.25701
G1 X140.882 Y108.776 E0.13454
M204 S1250
G1 X141.277 Y108.780 F9000.000
G1 E-4.00000 F2400.000
G1 X162.891 Y116.972 F9000.000
G1 E4.00000 F900.000
M204 S1000
;TYPE:Top solid infill
;WIDTH:0.404487
G1 F1450.455
G1 X162.304 Y117.559 E0.02219
G1 X161.777 Y117.559 E0.01406
G1 X162.726 Y116.611 E0.03582
G1 X162.726 Y116.084 E0.01406
G1 X161.251 Y117.559 E0.05570
G1 X160.724 Y117.559 E0.01406
G1 X162.726 Y115.558 E0.07558
G1 X162.726 Y115.031 E0.01406
G1 X160.198 Y117.559 E0.09546
G1 X159.671 Y117.559 E0.01406
G1 X162.726 Y114.505 E0.11534
```

G1 X162.726 Y113.978 E0.01406

G1 X159.145 Y117.559 E0.13522

G1 X158.618 Y117.559 E0.01406

G1 X162.726 Y113.452 E0.15510

G1 X162.726 Y112.925 E0.01406

G1 X158.092 Y117.559 E0.17498

G1 X157.565 Y117.559 E0.01406

G1 X162.726 Y112.399 E0.19486

G1 X162.726 Y111.872 E0.01406

G1 X157.039 Y117.559 E0.21474

G1 X156.512 Y117.559 E0.01406

G1 X162.726 Y111.346 E0.23462

G1 X162.726 Y110.819 E0.01406

G1 X156.263 Y117.283 E0.24405

G1 X156.106 Y116.912 E0.01073

G1 X162.726 Y110.293 E0.24995

G1 X162.726 Y109.766 E0.01406

G1 X155.944 Y116.548 E0.25607

G1 X155.752 Y116.214 E0.01029

G1 X162.726 Y109.240 E0.26334

G1 X162.726 Y108.713 E0.01406

G1 X155.560 Y115.880 E0.27060

G1 X155.339 Y115.574 E0.01007

G1 X162.726 Y108.187 E0.27893

G1 X162.726 Y107.660 E0.01406

G1 X155.113 Y115.274 E0.28748

G1 X154.872 Y114.988 E0.00998

G1 X162.726 Y107.134 E0.29657

G1 X162.726 Y106.607 E0.01406

G1 X154.613 Y114.720 E0.30635

G1 X154.353 Y114.454 E0.00994

G1 X162.726 Y106.081 E0.31617

G1 X162.726 Y105.554 E0.01406

G1 X154.061 Y114.219 E0.32718

G1 X153.770 Y113.984 E0.01000

G1 X162.726 Y105.028 E0.33819

G1 X162.726 Y104.501 E0.01406

G1 X153.457 Y113.770 E0.35000

G1 X153.132 Y113.569 E0.01021

G1 X162.279 Y104.422 E0.34540

G1 X161.752 Y104.422 E0.01406

G1 X152.796 Y113.378 E0.33818

G1 X152.436 Y113.212 E0.01060

G1 X161.226 Y104.422 E0.33191

G1 X160.699 Y104.422 E0.01406

G1 X152.065 Y113.056 E0.32602

G1 X151.671 Y112.924 E0.01111

G1 X160.173 Y104.422 E0.32104

G1 X159.646 Y104.422 E0.01406

G1 X151.276 Y112.792 E0.31607

G1 X150.837 Y112.704 E0.01195

G1 X159.120 Y104.422 E0.31275

G1 X158.593 Y104.422 E0.01406

G1 X150.394 Y112.621 E0.30962

G1 X149.903 Y112.586 E0.01313

G1 X158.067 Y104.422 E0.30826

G1 X157.540 Y104.422 E0.01406

G1 X149.403 Y112.559 E0.30728

```
G1 X148.876 Y112.559 E0.01406
G1 X152.863 Y108.573 E0.15053
G1 X153.575 Y108.137 E0.02230
G1 X154.358 Y107.524 E0.02656
G1 X154.987 Y106.874 E0.02414
G1 X155.527 Y106.157 E0.02397
G1 X155.866 Y105.570 E0.01810
G1 X157.014 Y104.422 E0.04335
G1 X156.487 Y104.422 E0.01406
G1 X155.990 Y104.919 E0.01878
M204 S1250
G1 X152.225 Y108.685 F9000.000
M204 S1000
G1 F1450.455
G1 X148.350 Y112.559 E0.14631
G1 X147.823 Y112.559 E0.01406
G1 X151.168 Y109.215 E0.12629
G1 X150.520 Y109.336 E0.01759
G1 X147.297 Y112.559 E0.12171
G1 X146.770 Y112.559 E0.01406
G1 X149.936 Y109.394 E0.11954
G1 X149.381 Y109.422 E0.01483
G1 X146.244 Y112.559 E0.11848
G1 X145.717 Y112.559 E0.01406
G1 X148.855 Y109.422 E0.11848
G1 X148.328 Y109.422 E0.01406
G1 X145.191 Y112.559 E0.11848
G1 X144.664 Y112.559 E0.01406
G1 X147.802 Y109.422 E0.11848
```

G1 X147.275 Y109.422 E0.01406

G1 X144.138 Y112.559 E0.11848

G1 X143.611 Y112.559 E0.01406

G1 X146.749 Y109.422 E0.11848

G1 X146.222 Y109.422 E0.01406

G1 X143.085 Y112.559 E0.11848

G1 X142.558 Y112.559 E0.01406

G1 X145.696 Y109.422 E0.11848

G1 X145.169 Y109.422 E0.01406

G1 X142.032 Y112.559 E0.11848

G1 X141.588 Y112.559 E0.01184

G1 X141.588 Y112.476 E0.00222

G1 X144.643 Y109.422 E0.11534

G1 X144.116 Y109.422 E0.01406

G1 X141.588 Y111.950 E0.09546

G1 X141.588 Y111.423 E0.01406

G1 X143.590 Y109.422 E0.07557

G1 X143.063 Y109.422 E0.01406

G1 X141.588 Y110.897 E0.05569

G1 X141.588 Y110.370 E0.01406

G1 X142.537 Y109.422 E0.03581

G1 X142.010 Y109.422 E0.01406

G1 X141.423 Y110.009 E0.02218

M204 S1250

; stop printing object Petg print.STL id:22 copy 0

; printing object Petg print.STL id:26 copy 0

G1 E-4.00000 F2400.000

G1 X141.423 Y127.787 F9000.000

G1 E4.00000 F900.000

```
M204 S800
;TYPE:Perimeter
;WIDTH:0.45
M73 P96 R3
G1 F1292.454
G1 X149.457 Y127.787 E0.24074
G1 X150.297 Y127.735 E0.02523
G1 X151.145 Y127.577 E0.02583
G1 X151.966 Y127.315 E0.02583
G1 X152.749 Y126.955 E0.02583
G1 X153.395 Y126.554 E0.02277
G1 X154.150 Y125.963 E0.02874
G1 X154.752 Y125.340 E0.02596
G1 X155.271 Y124.652 E0.02583
G1 X155.701 Y123.905 E0.02583
G1 X156.036 Y123.111 E0.02583
G1 X156.128 Y122.787 E0.01007
G1 X162.999 Y122.787 E0.20589
G1 X162.999 Y136.502 E0.41094
G1 X156.128 Y136.502 E0.20589
G1 X156.036 Y136.178 E0.01007
G1 X155.701 Y135.384 E0.02583
G1 X155.271 Y134.637 E0.02583
G1 X154.752 Y133.949 E0.02583
G1 X154.153 Y133.329 E0.02583
G1 X153.482 Y132.789 E0.02583
G1 X152.749 Y132.334 E0.02583
G1 X152.059 Y132.017 E0.02277
G1 X151.149 Y131.713 E0.02874
```

```
G1 X150.297 Y131.554 E0.02596
G1 X149.457 Y131.502 E0.02523
G1 X141.285 Y131.502 E0.24487
G1 X141.285 Y127.787 E0.11130
G1 X141.363 Y127.787 E0.00234
M204 S1250
G1 X140.867 Y127.370 F9000.000
M204 S800
;TYPE:External perimeter
G1 F1292.454
G1 X149.444 Y127.370 E0.25701
G1 X150.246 Y127.320 E0.02407
G1 X151.043 Y127.171 E0.02428
G1 X151.815 Y126.925 E0.02428
G1 X152.551 Y126.586 E0.02428
G1 X153.155 Y126.211 E0.02131
G1 X153.870 Y125.652 E0.02719
G1 X154.434 Y125.068 E0.02433
G1 X154.922 Y124.421 E0.02428
G1 X155.326 Y123.719 E0.02428
G1 X155.641 Y122.972 E0.02428
G1 X155.812 Y122.370 E0.01877
G1 X163.417 Y122.370 E0.22787
G1 X163.417 Y136.920 E0.43598
G1 X155.812 Y136.920 E0.22787
G1 X155.641 Y136.317 E0.01877
G1 X155.326 Y135.570 E0.02428
G1 X154.922 Y134.868 E0.02428
G1 X154.434 Y134.221 E0.02428
```

```
G1 X153.871 Y133.639 E0.02428
G1 X153.240 Y133.130 E0.02428
G1 X152.551 Y132.703 E0.02428
G1 X151.905 Y132.406 E0.02131
G1 X151.044 Y132.118 E0.02719
G1 X150.246 Y131.969 E0.02433
G1 X149.444 Y131.920 E0.02407
G1 X140.867 Y131.920 E0.25701
G1 X140.867 Y127.430 E0.13454
M204 S1250
G1 X141.262 Y127.434 F9000.000
G1 E-4.00000 F2400.000
G1 X162.876 Y135.626 F9000.000
G1 E4.00000 F900.000
M204 S1000
;TYPE:Top solid infill
;WIDTH:0.404487
G1 F1450.455
G1 X162.289 Y136.213 E0.02219
G1 X161.762 Y136.213 E0.01406
G1 X162.711 Y135.265 E0.03582
G1 X162.711 Y134.738 E0.01406
G1 X161.236 Y136.213 E0.05570
G1 X160.709 Y136.213 E0.01406
G1 X162.711 Y134.212 E0.07558
G1 X162.711 Y133.685 E0.01406
G1 X160.183 Y136.213 E0.09546
G1 X159.656 Y136.213 E0.01406
G1 X162.711 Y133.159 E0.11534
```

```
G1 X162.711 Y132.632 E0.01406
G1 X159.130 Y136.213 E0.13522
G1 X158.603 Y136.213 E0.01406
G1 X162.711 Y132.106 E0.15510
G1 X162.711 Y131.579 E0.01406
G1 X158.077 Y136.213 E0.17498
G1 X157.550 Y136.213 E0.01406
G1 X162.711 Y131.053 E0.19486
G1 X162.711 Y130.526 E0.01406
G1 X157.024 Y136.213 E0.21474
G1 X156.497 Y136.213 E0.01406
G1 X162.711 Y130.000 E0.23462
G1 X162.711 Y129.473 E0.01406
G1 X156.247 Y135.937 E0.24405
G1 X156.091 Y135.566 E0.01073
G1 X162.711 Y128.947 E0.24995
G1 X162.711 Y128.420 E0.01406
G1 X155.929 Y135.202 E0.25607
G1 X155.737 Y134.868 E0.01029
G1 X162.711 Y127.894 E0.26334
G1 X162.711 Y127.367 E0.01406
G1 X155.544 Y134.534 E0.27060
G1 X155.324 Y134.228 E0.01007
G1 X162.711 Y126.841 E0.27893
G1 X162.711 Y126.314 E0.01406
G1 X155.098 Y133.928 E0.28748
G1 X154.857 Y133.642 E0.00998
G1 X162.711 Y125.788 E0.29657
G1 X162.711 Y125.261 E0.01406
```

G1 X154.598 Y133.374 E0.30635

M73 P96 R2

G1 X154.338 Y133.108 E0.00994

G1 X162.711 Y124.735 E0.31617

G1 X162.711 Y124.208 E0.01406

G1 X154.046 Y132.873 E0.32718

G1 X153.754 Y132.638 E0.01000

G1 X162.711 Y123.682 E0.33819

G1 X162.711 Y123.155 E0.01406

G1 X153.442 Y132.424 E0.35000

G1 X153.117 Y132.223 E0.01021

G1 X162.264 Y123.076 E0.34540

G1 X161.737 Y123.076 E0.01406

G1 X152.781 Y132.032 E0.33818

G1 X152.421 Y131.866 E0.01060

G1 X161.211 Y123.076 E0.33191

G1 X160.684 Y123.076 E0.01406

G1 X152.050 Y131.710 E0.32602

G1 X151.656 Y131.578 E0.01111

G1 X160.158 Y123.076 E0.32104

G1 X159.631 Y123.076 E0.01406

G1 X151.261 Y131.446 E0.31607

G1 X150.822 Y131.358 E0.01195

G1 X159.105 Y123.076 E0.31275

G1 X158.578 Y123.076 E0.01406

G1 X150.379 Y131.275 E0.30962

G1 X149.888 Y131.239 E0.01313

G1 X158.052 Y123.076 E0.30826

G1 X157.525 Y123.076 E0.01406

```
G1 X149.388 Y131.213 E0.30728
G1 X148.861 Y131.213 E0.01406
G1 X152.848 Y127.227 E0.15053
G1 X153.560 Y126.791 E0.02230
G1 X154.343 Y126.178 E0.02656
G1 X154.972 Y125.528 E0.02414
G1 X155.512 Y124.811 E0.02397
G1 X155.851 Y124.224 E0.01810
G1 X156.999 Y123.076 E0.04335
G1 X156.472 Y123.076 E0.01406
G1 X155.975 Y123.573 E0.01878
M204 S1250
G1 X152.209 Y127.339 F9000.000
M204 S1000
G1 F1450.455
G1 X148.335 Y131.213 E0.14631
G1 X147.808 Y131.213 E0.01406
G1 X151.153 Y127.869 E0.12629
G1 X150.505 Y127.990 E0.01759
G1 X147.282 Y131.213 E0.12171
G1 X146.755 Y131.213 E0.01406
G1 X149.921 Y128.048 E0.11954
G1 X149.366 Y128.076 E0.01483
G1 X146.228 Y131.213 E0.11848
G1 X145.702 Y131.213 E0.01406
G1 X148.840 Y128.076 E0.11848
G1 X148.313 Y128.076 E0.01406
G1 X145.175 Y131.213 E0.11848
G1 X144.649 Y131.213 E0.01406
```

G1 X147.787 Y128.076 E0.11848

G1 X147.260 Y128.076 E0.01406

G1 X144.122 Y131.213 E0.11848

G1 X143.596 Y131.213 E0.01406

G1 X146.734 Y128.076 E0.11848

G1 X146.207 Y128.076 E0.01406

G1 X143.069 Y131.213 E0.11848

G1 X142.543 Y131.213 E0.01406

G1 X145.681 Y128.076 E0.11848

G1 X145.154 Y128.076 E0.01406

G1 X142.016 Y131.213 E0.11848

G1 X141.573 Y131.213 E0.01184

G1 X141.573 Y131.130 E0.00222

G1 X144.628 Y128.076 E0.11534

G1 X144.101 Y128.076 E0.01406

G1 X141.573 Y130.604 E0.09546

G1 X141.573 Y130.077 E0.01406

G1 X143.575 Y128.076 E0.07557

G1 X143.048 Y128.076 E0.01406

G1 X141.573 Y129.551 E0.05569

G1 X141.573 Y129.024 E0.01406

G1 X142.522 Y128.076 E0.03581

G1 X141.995 Y128.076 E0.01406

G1 X141.408 Y128.663 E0.02218

M204 S1250

; stop printing object Petg print.STL id:26 copy 0

; printing object Petg print.STL id:0 copy 0

G1 E-4.00000 F2400.000

G1 X111.357 Y96.857 F9000.000

```
G1 E4.00000 F900.000
M204 S800
;TYPE:Perimeter
;WIDTH:0.45
G1 F1292.454
G1 X104.486 Y96.857 E0.20589
G1 X104.394 Y96.534 E0.01007
G1 X104.059 Y95.739 E0.02583
G1 X103.629 Y94.993 E0.02583
G1 X103.110 Y94.304 E0.02583
G1 X102.511 Y93.685 E0.02583
G1 X101.840 Y93.144 E0.02583
G1 X101.107 Y92.690 E0.02583
G1 X100.417 Y92.372 E0.02277
G1 X99.507 Y92.068 E0.02874
G1 X98.656 Y91.909 E0.02596
G1 X97.815 Y91.857 E0.02523
G1 X89.643 Y91.857 E0.24487
G1 X89.643 Y88.143 E0.11130
G1 X97.815 Y88.143 E0.24487
G1 X98.656 Y88.091 E0.02523
G1 X99.503 Y87.932 E0.02583
G1 X100.324 Y87.671 E0.02583
G1 X101.107 Y87.310 E0.02583
G1 X101.753 Y86.910 E0.02277
G1 X102.508 Y86.318 E0.02874
G1 X103.110 Y85.696 E0.02596
G1 X103.629 Y85.007 E0.02583
G1 X104.059 Y84.261 E0.02583
```

```
G1 X104.394 Y83.466 E0.02583
G1 X104.486 Y83.143 E0.01007
G1 X111.357 Y83.143 E0.20589
G1 X111.357 Y96.797 E0.40914
M204 S1250
G1 X111.775 Y97.275 F9000.000
M204 S800
;TYPE:External perimeter
G1 F1292.454
G1 X104.170 Y97.275 E0.22787
G1 X104.000 Y96.672 E0.01877
G1 X103.685 Y95.926 E0.02428
G1 X103.280 Y95.223 E0.02428
G1 X102.792 Y94.576 E0.02428
G1 X102.229 Y93.994 E0.02428
G1 X101.598 Y93.486 E0.02428
G1 X100.909 Y93.059 E0.02428
G1 X100.263 Y92.761 E0.02131
G1 X99.402 Y92.474 E0.02719
G1 X98.604 Y92.325 E0.02433
G1 X97.802 Y92.275 E0.02407
G1 X89.225 Y92.275 E0.25701
G1 X89.225 Y87.725 E0.13634
G1 X97.802 Y87.725 E0.25701
G1 X98.604 Y87.675 E0.02407
G1 X99.401 Y87.526 E0.02428
G1 X100.173 Y87.281 E0.02428
G1 X100.909 Y86.941 E0.02428
G1 X101.513 Y86.567 E0.02131
```

```
G1 X102.228 Y86.007 E0.02719
G1 X102.792 Y85.424 E0.02433
G1 X103.280 Y84.777 E0.02428
G1 X103.685 Y84.074 E0.02428
G1 X104.000 Y83.328 E0.02428
G1 X104.170 Y82.725 E0.01877
G1 X111.775 Y82.725 E0.22787
G1 X111.775 Y97.215 E0.43418
M204 S1250
G1 X111.401 Y97.133 F9000.000
G1 X111.234 Y95.981
M204 S1000
;TYPE:Top solid infill
;WIDTH:0.404487
G1 F1450.455
G1 X110.647 Y96.569 E0.02219
G1 X110.120 Y96.569 E0.01406
G1 X111.069 Y95.620 E0.03582
G1 X111.069 Y95.094 E0.01406
G1 X109.594 Y96.569 E0.05570
G1 X109.067 Y96.569 E0.01406
G1 X111.069 Y94.567 E0.07558
G1 X111.069 Y94.041 E0.01406
G1 X108.541 Y96.569 E0.09546
G1 X108.014 Y96.569 E0.01406
G1 X111.069 Y93.514 E0.11534
G1 X111.069 Y92.988 E0.01406
G1 X107.488 Y96.569 E0.13522
G1 X106.961 Y96.569 E0.01406
```

```
G1 X111.069 Y92.461 E0.15510
G1 X111.069 Y91.935 E0.01406
G1 X106.435 Y96.569 E0.17498
G1 X105.908 Y96.569 E0.01406
G1 X111.069 Y91.408 E0.19486
G1 X111.069 Y90.882 E0.01406
G1 X105.382 Y96.569 E0.21474
G1 X104.855 Y96.569 E0.01406
G1 X111.069 Y90.355 E0.23462
G1 X111.069 Y89.829 E0.01406
G1 X104.606 Y96.292 E0.24405
G1 X104.449 Y95.922 E0.01073
G1 X111.069 Y89.302 E0.24995
G1 X111.069 Y88.776 E0.01406
G1 X104.287 Y95.557 E0.25607
G1 X104.095 Y95.223 E0.01029
G1 X111.069 Y88.249 E0.26334
G1 X111.069 Y87.723 E0.01406
G1 X103.902 Y94.889 E0.27060
G1 X103.682 Y94.583 E0.01007
G1 X111.069 Y87.196 E0.27893
G1 X111.069 Y86.670 E0.01406
G1 X103.456 Y94.283 E0.28748
G1 X103.215 Y93.997 E0.00998
G1 X111.069 Y86.143 E0.29657
G1 X111.069 Y85.617 E0.01406
G1 X102.956 Y93.730 E0.30635
G1 X102.696 Y93.463 E0.00994
G1 X111.069 Y85.090 E0.31617
```

```
G1 X111.069 Y84.564 E0.01406
G1 X102.404 Y93.228 E0.32718
M73 P97 R2
G1 X102.112 Y92.994 E0.01000
G1 X111.069 Y84.037 E0.33819
G1 X111.069 Y83.511 E0.01406
G1 X101.800 Y92.780 E0.35000
G1 X101.475 Y92.578 E0.01021
G1 X110.622 Y83.431 E0.34540
G1 X110.095 Y83.431 E0.01406
G1 X101.139 Y92.387 E0.33818
G1 X100.779 Y92.221 E0.01060
G1 X109.569 Y83.431 E0.33191
G1 X109.042 Y83.431 E0.01406
G1 X100.408 Y92.065 E0.32602
G1 X100.014 Y91.933 E0.01111
G1 X108.516 Y83.431 E0.32104
G1 X107.989 Y83.431 E0.01406
G1 X99.619 Y91.802 E0.31607
G1 X99.180 Y91.714 E0.01195
G1 X107.463 Y83.431 E0.31275
G1 X106.936 Y83.431 E0.01406
G1 X98.737 Y91.631 E0.30962
G1 X98.246 Y91.595 E0.01313
G1 X106.410 Y83.431 E0.30826
G1 X105.883 Y83.431 E0.01406
G1 X97.746 Y91.569 E0.30728
G1 X97.219 Y91.569 E0.01406
G1 X101.206 Y87.582 E0.15053
```

```
G1 X101.918 Y87.147 E0.02230
G1 X102.701 Y86.533 E0.02656
G1 X103.330 Y85.884 E0.02414
G1 X103.870 Y85.167 E0.02397
G1 X104.209 Y84.579 E0.01810
G1 X105.357 Y83.431 E0.04335
G1 X104.830 Y83.431 E0.01406
G1 X104.333 Y83.929 E0.01878
M204 S1250
G1 X100.567 Y87.694 F9000.000
M204 S1000
G1 F1450.455
G1 X96.693 Y91.569 E0.14631
G1 X96.166 Y91.569 E0.01406
G1 X99.511 Y88.224 E0.12629
G1 X98.863 Y88.345 E0.01759
G1 X95.640 Y91.569 E0.12171
G1 X95.113 Y91.569 E0.01406
G1 X98.279 Y88.403 E0.11954
G1 X97.724 Y88.431 E0.01483
G1 X94.587 Y91.569 E0.11848
G1 X94.060 Y91.569 E0.01406
G1 X97.198 Y88.431 E0.11848
G1 X96.671 Y88.431 E0.01406
G1 X93.534 Y91.569 E0.11848
G1 X93.007 Y91.569 E0.01406
G1 X96.145 Y88.431 E0.11848
G1 X95.618 Y88.431 E0.01406
G1 X92.480 Y91.569 E0.11848
```

```
G1 X91.954 Y91.569 E0.01406
G1 X95.092 Y88.431 E0.11848
G1 X94.565 Y88.431 E0.01406
G1 X91.427 Y91.569 E0.11848
G1 X90.901 Y91.569 E0.01406
G1 X94.039 Y88.431 E0.11848
G1 X93.512 Y88.431 E0.01406
G1 X90.374 Y91.569 E0.11848
G1 X89.931 Y91.569 E0.01184
G1 X89.931 Y91.486 E0.00222
G1 X92.986 Y88.431 E0.11534
G1 X92.459 Y88.431 E0.01406
G1 X89.931 Y90.959 E0.09546
G1 X89.931 Y90.433 E0.01406
G1 X91.933 Y88.431 E0.07557
G1 X91.406 Y88.431 E0.01406
G1 X89.931 Y89.906 E0.05569
G1 X89.931 Y89.380 E0.01406
G1 X90.880 Y88.431 E0.03581
G1 X90.353 Y88.431 E0.01406
G1 X89.766 Y89.019 E0.02218
M204 S1250
; stop printing object Petg print.STL id:0 copy 0
; printing object tpu print.STL id:1 copy 0
; stop printing object tpu print.STL id:1 copy 0
; printing object tpu print.STL id:5 copy 0
; stop printing object tpu print.STL id:5 copy 0
; printing object tpu print.STL id:9 copy 0
; stop printing object tpu print.STL id:9 copy 0
```

```
; printing object Petg print.STL id:8 copy 0
G1 E-4.00000 F2400.000
G1 X104.530 Y54.716 F9000.000
G1 E4.00000 F900.000
M204 S800
;TYPE:Perimeter
;WIDTH:0.45
G1 F1292.454
G1 X104.438 Y54.392 E0.01007
G1 X104.103 Y53.598 E0.02583
G1 X103.673 Y52.851 E0.02583
G1 X103.154 Y52.163 E0.02583
G1 X102.555 Y51.544 E0.02583
G1 X101.883 Y51.003 E0.02583
G1 X101.151 Y50.549 E0.02583
G1 X100.461 Y50.231 E0.02277
G1 X99.551 Y49.927 E0.02874
G1 X98.699 Y49.768 E0.02596
G1 X97.859 Y49.716 E0.02523
G1 X89.686 Y49.716 E0.24487
G1 X89.686 Y46.002 E0.11130
G1 X97.859 Y46.002 E0.24487
G1 X98.699 Y45.950 E0.02523
G1 X99.546 Y45.791 E0.02583
G1 X100.368 Y45.530 E0.02583
G1 X101.151 Y45.169 E0.02583
G1 X101.796 Y44.769 E0.02277
G1 X102.551 Y44.177 E0.02874
G1 X103.154 Y43.555 E0.02596
```

```
G1 X103.673 Y42.866 E0.02583
G1 X104.103 Y42.119 E0.02583
G1 X104.438 Y41.325 E0.02583
G1 X104.530 Y41.002 E0.01007
G1 X111.401 Y41.002 E0.20589
G1 X111.401 Y54.716 E0.41094
G1 X104.590 Y54.716 E0.20409
M204 S1250
G1 X104.214 Y55.134 F9000.000
M204 S800
;TYPE:External perimeter
G1 F1292.454
G1 X104.043 Y54.531 E0.01877
G1 X103.728 Y53.784 E0.02428
G1 X103.324 Y53.082 E0.02428
G1 X102.836 Y52.435 E0.02428
G1 X102.272 Y51.853 E0.02428
G1 X101.641 Y51.344 E0.02428
G1 X100.952 Y50.917 E0.02428
G1 X100.307 Y50.620 E0.02131
G1 X99.446 Y50.333 E0.02719
G1 X98.648 Y50.183 E0.02433
G1 X97.846 Y50.134 E0.02407
G1 X89.269 Y50.134 E0.25701
G1 X89.269 Y45.584 E0.13634
G1 X97.846 Y45.584 E0.25701
G1 X98.648 Y45.534 E0.02407
G1 X99.444 Y45.385 E0.02428
G1 X100.216 Y45.139 E0.02428
```

```
G1 X100.952 Y44.800 E0.02428
G1 X101.557 Y44.426 E0.02131
G1 X102.271 Y43.866 E0.02719
G1 X102.836 Y43.282 E0.02433
G1 X103.324 Y42.635 E0.02428
G1 X103.728 Y41.933 E0.02428
G1 X104.043 Y41.186 E0.02428
G1 X104.214 Y40.584 E0.01877
G1 X111.819 Y40.584 E0.22787
G1 X111.819 Y55.134 E0.43598
G1 X104.274 Y55.134 E0.22608
M204 S1250
G1 X104.204 Y54.734 F9000.000
G1 E-4.00000 F2400.000
G1 X111.278 Y53.840 F9000.000
G1 E4.00000 F900.000
M204 S1000
;TYPE:Top solid infill
;WIDTH:0.404487
G1 F1450.455
G1 X110.690 Y54.428 E0.02219
G1 X110.164 Y54.428 E0.01406
G1 X111.112 Y53.479 E0.03582
G1 X111.112 Y52.953 E0.01406
G1 X109.637 Y54.428 E0.05570
G1 X109.111 Y54.428 E0.01406
G1 X111.112 Y52.426 E0.07558
G1 X111.112 Y51.899 E0.01406
G1 X108.584 Y54.428 E0.09546
```

```
G1 X108.058 Y54.428 E0.01406
G1 X111.112 Y51.373 E0.11534
G1 X111.112 Y50.846 E0.01406
G1 X107.531 Y54.428 E0.13522
G1 X107.005 Y54.428 E0.01406
G1 X111.112 Y50.320 E0.15510
G1 X111.112 Y49.793 E0.01406
G1 X106.478 Y54.428 E0.17498
G1 X105.952 Y54.428 E0.01406
G1 X111.112 Y49.267 E0.19486
G1 X111.112 Y48.740 E0.01406
G1 X105.425 Y54.428 E0.21474
G1 X104.899 Y54.428 E0.01406
G1 X111.112 Y48.214 E0.23462
G1 X111.112 Y47.687 E0.01406
G1 X104.649 Y54.151 E0.24405
G1 X104.493 Y53.780 E0.01073
G1 X111.112 Y47.161 E0.24995
G1 X111.112 Y46.634 E0.01406
G1 X104.331 Y53.416 E0.25607
G1 X104.138 Y53.082 E0.01029
G1 X111.112 Y46.108 E0.26334
G1 X111.112 Y45.581 E0.01406
G1 X103.946 Y52.748 E0.27060
G1 X103.725 Y52.442 E0.01007
G1 X111.112 Y45.055 E0.27893
G1 X111.112 Y44.528 E0.01406
G1 X103.499 Y52.142 E0.28748
G1 X103.258 Y51.856 E0.00998
```

```
G1 X111.112 Y44.002 E0.29657
G1 X111.112 Y43.475 E0.01406
G1 X102.999 Y51.589 E0.30635
G1 X102.739 Y51.322 E0.00994
G1 X111.112 Y42.949 E0.31617
G1 X111.112 Y42.422 E0.01406
G1 X102.448 Y51.087 E0.32718
G1 X102.156 Y50.852 E0.01000
G1 X111.112 Y41.896 E0.33819
G1 X111.112 Y41.369 E0.01406
G1 X101.843 Y50.639 E0.35000
G1 X101.518 Y50.437 E0.01021
G1 X110.665 Y41.290 E0.34540
G1 X110.139 Y41.290 E0.01406
G1 X101.183 Y50.246 E0.33818
G1 X100.822 Y50.080 E0.01060
G1 X109.612 Y41.290 E0.33191
G1 X109.086 Y41.290 E0.01406
G1 X100.452 Y49.924 E0.32602
G1 X100.057 Y49.792 E0.01111
G1 X108.559 Y41.290 E0.32104
G1 X108.033 Y41.290 E0.01406
G1 X99.662 Y49.660 E0.31607
G1 X99.224 Y49.573 E0.01195
G1 X107.506 Y41.290 E0.31275
G1 X106.980 Y41.290 E0.01406
G1 X98.780 Y49.490 E0.30962
G1 X98.290 Y49.454 E0.01313
G1 X106.453 Y41.290 E0.30826
```

```
M73 P97 R1
G1 X105.927 Y41.290 E0.01406
G1 X97.789 Y49.428 E0.30728
G1 X97.263 Y49.428 E0.01406
G1 X101.249 Y45.441 E0.15053
G1 X101.962 Y45.005 E0.02230
G1 X102.745 Y44.392 E0.02656
G1 X103.374 Y43.742 E0.02414
G1 X103.914 Y43.026 E0.02397
G1 X104.252 Y42.438 E0.01810
G1 X105.400 Y41.290 E0.04335
G1 X104.874 Y41.290 E0.01406
G1 X104.376 Y41.787 E0.01878
M204 S1250
G1 X100.611 Y45.553 F9000.000
M204 S1000
G1 F1450.455
G1 X96.736 Y49.428 E0.14631
G1 X96.210 Y49.428 E0.01406
G1 X99.554 Y46.083 E0.12629
G1 X98.907 Y46.204 E0.01759
G1 X95.683 Y49.428 E0.12171
G1 X95.157 Y49.428 E0.01406
G1 X98.322 Y46.262 E0.11954
G1 X97.768 Y46.290 E0.01483
G1 X94.630 Y49.428 E0.11848
G1 X94.104 Y49.428 E0.01406
G1 X97.241 Y46.290 E0.11848
G1 X96.715 Y46.290 E0.01406
```

```
G1 X93.577 Y49.428 E0.11848
G1 X93.051 Y49.428 E0.01406
G1 X96.188 Y46.290 E0.11848
G1 X95.662 Y46.290 E0.01406
G1 X92.524 Y49.428 E0.11848
G1 X91.998 Y49.428 E0.01406
G1 X95.135 Y46.290 E0.11848
G1 X94.609 Y46.290 E0.01406
G1 X91.471 Y49.428 E0.11848
G1 X90.945 Y49.428 E0.01406
G1 X94.082 Y46.290 E0.11848
G1 X93.556 Y46.290 E0.01406
G1 X90.418 Y49.428 E0.11848
G1 X89.975 Y49.428 E0.01184
G1 X89.975 Y49.344 E0.00222
G1 X93.029 Y46.290 E0.11534
G1 X92.503 Y46.290 E0.01406
G1 X89.975 Y48.818 E0.09546
G1 X89.975 Y48.291 E0.01406
G1 X91.976 Y46.290 E0.07557
G1 X91.450 Y46.290 E0.01406
G1 X89.975 Y47.765 E0.05569
G1 X89.975 Y47.238 E0.01406
G1 X90.923 Y46.290 E0.03581
G1 X90.397 Y46.290 E0.01406
G1 X89.809 Y46.877 E0.02218
M204 S1250
; stop printing object Petg print.STL id:8 copy 0
; printing object Petg print.STL id:4 copy 0
```

```
G1 E-4.00000 F2400.000
G1 X89.809 Y65.950 F9000.000
G1 E4.00000 F900.000
M204 S800
;TYPE:Perimeter
;WIDTH:0.45
G1 F1292.454
G1 X97.866 Y65.950 E0.24142
G1 X98.707 Y65.899 E0.02523
G1 X99.554 Y65.740 E0.02583
G1 X100.375 Y65.478 E0.02583
G1 X101.158 Y65.118 E0.02583
G1 X101.804 Y64.718 E0.02277
G1 X102.559 Y64.126 E0.02874
G1 X103.162 Y63.503 E0.02596
G1 X103.680 Y62.815 E0.02583
G1 X104.111 Y62.068 E0.02583
G1 X104.446 Y61.274 E0.02583
G1 X104.537 Y60.950 E0.01007
G1 X111.408 Y60.950 E0.20589
G1 X111.408 Y74.665 E0.41094
G1 X104.537 Y74.665 E0.20589
G1 X104.446 Y74.341 E0.01007
G1 X104.111 Y73.547 E0.02583
G1 X103.680 Y72.800 E0.02583
G1 X103.162 Y72.112 E0.02583
M73 P98 R1
G1 X102.562 Y71.493 E0.02583
G1 X101.891 Y70.952 E0.02583
```

```
G1 X101.158 Y70.497 E0.02583
G1 X100.468 Y70.180 E0.02277
G1 X99.558 Y69.876 E0.02874
G1 X98.707 Y69.717 E0.02596
G1 X97.866 Y69.665 E0.02523
G1 X89.694 Y69.665 E0.24487
G1 X89.694 Y65.950 E0.11130
G1 X89.749 Y65.950 E0.00166
M204 S1250
G1 X89.276 Y65.533 F9000.000
M204 S800
;TYPE:External perimeter
G1 F1292.454
G1 X97.853 Y65.533 E0.25701
G1 X98.655 Y65.483 E0.02407
G1 X99.452 Y65.334 E0.02428
G1 X100.224 Y65.088 E0.02428
G1 X100.960 Y64.749 E0.02428
G1 X101.564 Y64.374 E0.02131
G1 X102.279 Y63.815 E0.02719
G1 X102.843 Y63.231 E0.02433
G1 X103.331 Y62.584 E0.02428
G1 X103.736 Y61.882 E0.02428
G1 X104.051 Y61.135 E0.02428
G1 X104.221 Y60.533 E0.01877
G1 X111.826 Y60.533 E0.22787
G1 X111.826 Y75.083 E0.43598
G1 X104.221 Y75.083 E0.22787
G1 X104.051 Y74.480 E0.01877
```

```
G1 X103.736 Y73.733 E0.02428
G1 X103.331 Y73.031 E0.02428
G1 X102.843 Y72.384 E0.02428
G1 X102.280 Y71.802 E0.02428
G1 X101.649 Y71.293 E0.02428
G1 X100.960 Y70.866 E0.02428
G1 X100.314 Y70.569 E0.02131
G1 X99.453 Y70.282 E0.02719
G1 X98.655 Y70.132 E0.02433
G1 X97.853 Y70.083 E0.02407
G1 X89.276 Y70.083 E0.25701
G1 X89.276 Y65.593 E0.13454
M204 S1250
G1 X89.671 Y65.597 F9000.000
G1 E-4.00000 F2400.000
G1 X111.286 Y73.789 F9000.000
G1 E4.00000 F900.000
M204 S1000
;TYPE:Top solid infill
;WIDTH:0.404487
G1 F1450.455
G1 X110.698 Y74.376 E0.02219
G1 X110.171 Y74.376 E0.01406
G1 X111.120 Y73.428 E0.03582
G1 X111.120 Y72.901 E0.01406
G1 X109.645 Y74.376 E0.05570
G1 X109.118 Y74.376 E0.01406
G1 X111.120 Y72.375 E0.07558
G1 X111.120 Y71.848 E0.01406
```

```
G1 X108.592 Y74.376 E0.09546
G1 X108.065 Y74.376 E0.01406
G1 X111.120 Y71.322 E0.11534
G1 X111.120 Y70.795 E0.01406
G1 X107.539 Y74.376 E0.13522
G1 X107.012 Y74.376 E0.01406
G1 X111.120 Y70.269 E0.15510
G1 X111.120 Y69.742 E0.01406
G1 X106.486 Y74.376 E0.17498
G1 X105.959 Y74.376 E0.01406
G1 X111.120 Y69.216 E0.19486
G1 X111.120 Y68.689 E0.01406
G1 X105.433 Y74.376 E0.21474
G1 X104.906 Y74.376 E0.01406
G1 X111.120 Y68.163 E0.23462
G1 X111.120 Y67.636 E0.01406
G1 X104.657 Y74.100 E0.24405
G1 X104.500 Y73.729 E0.01073
G1 X111.120 Y67.110 E0.24995
G1 X111.120 Y66.583 E0.01406
G1 X104.338 Y73.365 E0.25607
G1 X104.146 Y73.031 E0.01029
G1 X111.120 Y66.057 E0.26334
G1 X111.120 Y65.530 E0.01406
G1 X103.954 Y72.697 E0.27060
G1 X103.733 Y72.391 E0.01007
G1 X111.120 Y65.004 E0.27893
G1 X111.120 Y64.477 E0.01406
G1 X103.507 Y72.091 E0.28748
```

G1 X103.266 Y71.805 E0.00998

G1 X111.120 Y63.951 E0.29657

G1 X111.120 Y63.424 E0.01406

G1 X103.007 Y71.537 E0.30635

G1 X102.747 Y71.271 E0.00994

G1 X111.120 Y62.898 E0.31617

G1 X111.120 Y62.371 E0.01406

G1 X102.455 Y71.036 E0.32718

G1 X102.164 Y70.801 E0.01000

G1 X111.120 Y61.845 E0.33819

G1 X111.120 Y61.318 E0.01406

G1 X101.851 Y70.588 E0.35000

G1 X101.526 Y70.386 E0.01021

G1 X110.673 Y61.239 E0.34540

G1 X110.146 Y61.239 E0.01406

G1 X101.190 Y70.195 E0.33818

G1 X100.830 Y70.029 E0.01060

G1 X109.620 Y61.239 E0.33191

G1 X109.093 Y61.239 E0.01406

G1 X100.459 Y69.873 E0.32602

G1 X100.065 Y69.741 E0.01111

G1 X108.567 Y61.239 E0.32104

G1 X108.040 Y61.239 E0.01406

G1 X99.670 Y69.609 E0.31607

G1 X99.231 Y69.522 E0.01195

G1 X107.514 Y61.239 E0.31275

G1 X106.987 Y61.239 E0.01406

G1 X98.788 Y69.439 E0.30962

G1 X98.297 Y69.403 E0.01313

```
G1 X106.461 Y61.239 E0.30826
G1 X105.934 Y61.239 E0.01406
G1 X97.797 Y69.376 E0.30728
G1 X97.270 Y69.376 E0.01406
G1 X101.257 Y65.390 E0.15053
G1 X101.969 Y64.954 E0.02230
G1 X102.752 Y64.341 E0.02656
G1 X103.381 Y63.691 E0.02414
G1 X103.921 Y62.974 E0.02397
G1 X104.260 Y62.387 E0.01810
G1 X105.408 Y61.239 E0.04335
G1 X104.881 Y61.239 E0.01406
G1 X104.384 Y61.736 E0.01878
M204 S1250
G1 X100.619 Y65.502 F9000.000
M204 S1000
G1 F1450.455
G1 X96.744 Y69.376 E0.14631
G1 X96.217 Y69.376 E0.01406
G1 X99.562 Y66.032 E0.12629
G1 X98.914 Y66.153 E0.01759
G1 X95.691 Y69.376 E0.12171
G1 X95.164 Y69.376 E0.01406
G1 X98.330 Y66.211 E0.11954
G1 X97.775 Y66.239 E0.01483
G1 X94.638 Y69.376 E0.11848
G1 X94.111 Y69.376 E0.01406
G1 X97.249 Y66.239 E0.11848
G1 X96.722 Y66.239 E0.01406
```

```
G1 X93.585 Y69.376 E0.11848
G1 X93.058 Y69.376 E0.01406
G1 X96.196 Y66.239 E0.11848
G1 X95.669 Y66.239 E0.01406
G1 X92.532 Y69.376 E0.11848
G1 X92.005 Y69.376 E0.01406
G1 X95.143 Y66.239 E0.11848
G1 X94.616 Y66.239 E0.01406
G1 X91.479 Y69.376 E0.11848
G1 X90.952 Y69.376 E0.01406
G1 X94.090 Y66.239 E0.11848
G1 X93.563 Y66.239 E0.01406
G1 X90.426 Y69.376 E0.11848
G1 X89.982 Y69.376 E0.01184
G1 X89.982 Y69.293 E0.00222
G1 X93.037 Y66.239 E0.11534
G1 X92.510 Y66.239 E0.01406
G1 X89.982 Y68.767 E0.09546
G1 X89.982 Y68.240 E0.01406
G1 X91.984 Y66.239 E0.07557
G1 X91.457 Y66.239 E0.01406
G1 X89.982 Y67.714 E0.05569
G1 X89.982 Y67.187 E0.01406
G1 X90.931 Y66.239 E0.03581
G1 X90.404 Y66.239 E0.01406
G1 X89.817 Y66.826 E0.02218
M204 S1250
; stop printing object Petg print.STL id:4 copy 0
; printing object tpu print.STL id:29 copy 0
```

```
; stop printing object tpu print.STL id:29 copy 0
; printing object tpu print.STL id:25 copy 0
; stop printing object tpu print.STL id:25 copy 0
; printing object Petg print.STL id:24 copy 0
G1 E-4.00000 F2400.000
G1 X141.344 Y66.126 F9000.000
G1 E4.00000 F900.000
M204 S800
;TYPE:Perimeter
;WIDTH:0.45
G1 F1292.454
G1 X149.516 Y66.126 E0.24487
G1 X150.357 Y66.074 E0.02523
G1 X151.204 Y65.915 E0.02583
G1 X152.026 Y65.654 E0.02583
G1 X152.808 Y65.293 E0.02583
G1 X153.454 Y64.893 E0.02277
G1 X154.209 Y64.301 E0.02874
G1 X154.812 Y63.679 E0.02596
G1 X155.331 Y62.990 E0.02583
G1 X155.761 Y62.243 E0.02583
G1 X156.096 Y61.449 E0.02583
G1 X156.187 Y61.126 E0.01007
G1 X163.059 Y61.126 E0.20589
G1 X163.059 Y74.840 E0.41094
G1 X156.187 Y74.840 E0.20589
G1 X156.096 Y74.516 E0.01007
G1 X155.761 Y73.722 E0.02583
G1 X155.331 Y72.975 E0.02583
```

```
G1 X154.812 Y72.287 E0.02583
G1 X154.212 Y71.668 E0.02583
G1 X153.541 Y71.127 E0.02583
G1 X152.808 Y70.673 E0.02583
G1 X152.118 Y70.355 E0.02277
G1 X151.209 Y70.051 E0.02874
G1 X150.357 Y69.892 E0.02596
G1 X149.516 Y69.840 E0.02523
G1 X141.344 Y69.840 E0.24487
G1 X141.344 Y66.186 E0.10950
M204 S1250
G1 X140.926 Y65.708 F9000.000
M204 S800
;TYPE:External perimeter
G1 F1292.454
G1 X149.503 Y65.708 E0.25701
G1 X150.305 Y65.658 E0.02407
G1 X151.102 Y65.509 E0.02428
G1 X151.874 Y65.263 E0.02428
G1 X152.610 Y64.924 E0.02428
G1 X153.215 Y64.550 E0.02131
G1 X153.929 Y63.990 E0.02719
G1 X154.494 Y63.406 E0.02433
G1 X154.981 Y62.759 E0.02428
G1 X155.386 Y62.057 E0.02428
G1 X155.701 Y61.310 E0.02428
G1 X155.872 Y60.708 E0.01877
G1 X163.476 Y60.708 E0.22787
G1 X163.476 Y75.258 E0.43598
```

```
G1 X155.872 Y75.258 E0.22787
G1 X155.701 Y74.655 E0.01877
G1 X155.386 Y73.908 E0.02428
G1 X154.981 Y73.206 E0.02428
G1 X154.494 Y72.559 E0.02428
G1 X153.930 Y71.977 E0.02428
G1 X153.299 Y71.468 E0.02428
G1 X152.610 Y71.041 E0.02428
G1 X151.964 Y70.744 E0.02131
G1 X151.103 Y70.457 E0.02719
G1 X150.305 Y70.307 E0.02433
G1 X149.503 Y70.258 E0.02407
G1 X140.926 Y70.258 E0.25701
G1 X140.926 Y65.768 E0.13454
M204 S1250
G1 X141.321 Y65.772 F9000.000
G1 E-4.00000 F2400.000
G1 X162.936 Y73.964 F9000.000
G1 E4.00000 F900.000
M204 S1000
;TYPE:Top solid infill
;WIDTH:0.404487
G1 F1450.455
G1 X162.348 Y74.552 E0.02219
G1 X161.822 Y74.552 E0.01406
G1 X162.770 Y73.603 E0.03582
G1 X162.770 Y73.077 E0.01406
G1 X161.295 Y74.552 E0.05570
G1 X160.769 Y74.552 E0.01406
```

G1 X162.770 Y72.550 E0.07558

G1 X162.770 Y72.024 E0.01406

G1 X160.242 Y74.552 E0.09546

G1 X159.716 Y74.552 E0.01406

G1 X162.770 Y71.497 E0.11534

G1 X162.770 Y70.970 E0.01406

G1 X159.189 Y74.552 E0.13522

G1 X158.663 Y74.552 E0.01406

G1 X162.770 Y70.444 E0.15510

G1 X162.770 Y69.917 E0.01406

G1 X158.136 Y74.552 E0.17498

G1 X157.610 Y74.552 E0.01406

G1 X162.770 Y69.391 E0.19486

G1 X162.770 Y68.864 E0.01406

G1 X157.083 Y74.552 E0.21474

G1 X156.557 Y74.552 E0.01406

G1 X162.770 Y68.338 E0.23462

G1 X162.770 Y67.811 E0.01406

G1 X156.307 Y74.275 E0.24405

G1 X156.151 Y73.904 E0.01073

G1 X162.770 Y67.285 E0.24995

G1 X162.770 Y66.758 E0.01406

G1 X155.989 Y73.540 E0.25607

G1 X155.796 Y73.206 E0.01029

G1 X162.770 Y66.232 E0.26334

G1 X162.770 Y65.705 E0.01406

G1 X155.604 Y72.872 E0.27060

G1 X155.383 Y72.566 E0.01007

G1 X162.770 Y65.179 E0.27893

```
G1 X162.770 Y64.652 E0.01406
G1 X155.157 Y72.266 E0.28748
G1 X154.916 Y71.980 E0.00998
G1 X162.770 Y64.126 E0.29657
G1 X162.770 Y63.599 E0.01406
G1 X154.657 Y71.713 E0.30635
G1 X154.397 Y71.446 E0.00994
G1 X162.770 Y63.073 E0.31617
G1 X162.770 Y62.546 E0.01406
G1 X154.105 Y71.211 E0.32718
G1 X153.814 Y70.976 E0.01000
G1 X162.770 Y62.020 E0.33819
G1 X162.770 Y61.493 E0.01406
G1 X153.501 Y70.763 E0.35000
G1 X153.176 Y70.561 E0.01021
G1 X162.323 Y61.414 E0.34540
M73 P99 R1
G1 X161.797 Y61.414 E0.01406
G1 X152.841 Y70.370 E0.33818
G1 X152.480 Y70.204 E0.01060
G1 X161.270 Y61.414 E0.33191
G1 X160.744 Y61.414 E0.01406
G1 X152.110 Y70.048 E0.32602
G1 X151.715 Y69.916 E0.01111
G1 X160.217 Y61.414 E0.32104
G1 X159.691 Y61.414 E0.01406
G1 X151.320 Y69.784 E0.31607
G1 X150.881 Y69.697 E0.01195
G1 X159.164 Y61.414 E0.31275
```

```
G1 X158.638 Y61.414 E0.01406
G1 X150.438 Y69.614 E0.30962
G1 X149.947 Y69.578 E0.01313
G1 X158.111 Y61.414 E0.30826
G1 X157.585 Y61.414 E0.01406
G1 X149.447 Y69.552 E0.30728
G1 X148.920 Y69.552 E0.01406
G1 X152.907 Y65.565 E0.15053
G1 X153.620 Y65.129 E0.02230
G1 X154.403 Y64.516 E0.02656
G1 X155.031 Y63.866 E0.02414
G1 X155.572 Y63.150 E0.02397
G1 X155.910 Y62.562 E0.01810
G1 X157.058 Y61.414 E0.04335
G1 X156.532 Y61.414 E0.01406
G1 X156.034 Y61.911 E0.01878
M204 S1250
G1 X152.269 Y65.677 F9000.000
M204 S1000
G1 F1450.455
G1 X148.394 Y69.552 E0.14631
G1 X147.867 Y69.552 E0.01406
G1 X151.212 Y66.207 E0.12629
G1 X150.564 Y66.328 E0.01759
G1 X147.341 Y69.552 E0.12171
G1 X146.814 Y69.552 E0.01406
G1 X149.980 Y66.386 E0.11954
G1 X149.426 Y66.414 E0.01483
G1 X146.288 Y69.552 E0.11848
```

G1 X145.761 Y69.552 E0.01406

G1 X148.899 Y66.414 E0.11848

G1 X148.373 Y66.414 E0.01406

G1 X145.235 Y69.552 E0.11848

G1 X144.708 Y69.552 E0.01406

G1 X147.846 Y66.414 E0.11848

G1 X147.320 Y66.414 E0.01406

G1 X144.182 Y69.552 E0.11848

G1 X143.655 Y69.552 E0.01406

G1 X146.793 Y66.414 E0.11848

G1 X146.267 Y66.414 E0.01406

G1 X143.129 Y69.552 E0.11848

G1 X142.602 Y69.552 E0.01406

G1 X145.740 Y66.414 E0.11848

G1 X145.214 Y66.414 E0.01406

G1 X142.076 Y69.552 E0.11848

G1 X141.633 Y69.552 E0.01184

G1 X141.633 Y69.468 E0.00222

G1 X144.687 Y66.414 E0.11534

G1 X144.161 Y66.414 E0.01406

G1 X141.633 Y68.942 E0.09546

G1 X141.633 Y68.415 E0.01406

G1 X143.634 Y66.414 E0.07557

G1 X143.108 Y66.414 E0.01406

G1 X141.633 Y67.889 E0.05569

G1 X141.633 Y67.362 E0.01406

G1 X142.581 Y66.414 E0.03581

G1 X142.055 Y66.414 E0.01406

G1 X141.467 Y67.001 E0.02218

```
M204 S1250
; stop printing object Petg print.STL id:24 copy 0
; printing object Petg print.STL id:28 copy 0
G1 E-4.00000 F2400.000
G1 X141.467 Y49.891 F9000.000
G1 E4.00000 F900.000
M204 S800
;TYPE:Perimeter
;WIDTH:0.45
G1 F1292.454
G1 X141.337 Y49.891 E0.00390
G1 X141.337 Y46.177 E0.11130
G1 X149.509 Y46.177 E0.24487
G1 X150.349 Y46.125 E0.02523
G1 X151.197 Y45.966 E0.02583
G1 X152.018 Y45.705 E0.02583
M73 P99 R0
G1 X152.801 Y45.344 E0.02583
G1 X153.447 Y44.944 E0.02277
G1 X154.202 Y44.352 E0.02874
G1 X154.804 Y43.730 E0.02596
G1 X155.323 Y43.041 E0.02583
G1 X155.753 Y42.294 E0.02583
G1 X156.088 Y41.500 E0.02583
G1 X156.180 Y41.177 E0.01007
G1 X163.051 Y41.177 E0.20589
G1 X163.051 Y54.891 E0.41094
G1 X156.180 Y54.891 E0.20589
G1 X156.088 Y54.567 E0.01007
```

G1 X155.753 Y53.773 E0.02583

G1 X155.323 Y53.026 E0.02583

G1 X154.804 Y52.338 E0.02583

G1 X154.205 Y51.719 E0.02583

G1 X153.534 Y51.178 E0.02583

G1 X152.801 Y50.724 E0.02583

G1 X152.111 Y50.406 E0.02277

G1 X151.201 Y50.102 E0.02874

G1 X150.349 Y49.943 E0.02596

G1 X149.509 Y49.891 E0.02523

G1 X141.527 Y49.891 E0.23917

M204 S1250

G1 X140.919 Y50.309 F9000.000

M204 S800

;TYPE:External perimeter

G1 F1292.454

G1 X140.919 Y45.759 E0.13634

G1 X149.496 Y45.759 E0.25701

G1 X150.298 Y45.709 E0.02407

G1 X151.094 Y45.560 E0.02428

G1 X151.867 Y45.314 E0.02428

G1 X152.603 Y44.975 E0.02428

G1 X153.207 Y44.601 E0.02131

G1 X153.921 Y44.041 E0.02719

G1 X154.486 Y43.458 E0.02433

G1 X154.974 Y42.810 E0.02428

G1 X155.378 Y42.108 E0.02428

G1 X155.693 Y41.361 E0.02428

G1 X155.864 Y40.759 E0.01877

```
G1 X163.469 Y40.759 E0.22787
G1 X163.469 Y55.309 E0.43598
G1 X155.864 Y55.309 E0.22787
G1 X155.693 Y54.706 E0.01877
G1 X155.378 Y53.959 E0.02428
G1 X154.974 Y53.257 E0.02428
G1 X154.486 Y52.610 E0.02428
G1 X153.923 Y52.028 E0.02428
G1 X153.292 Y51.519 E0.02428
G1 X152.603 Y51.092 E0.02428
G1 X151.957 Y50.795 E0.02131
G1 X151.096 Y50.508 E0.02719
G1 X150.298 Y50.358 E0.02433
G1 X149.496 Y50.309 E0.02407
G1 X140.979 Y50.309 E0.25521
M204 S1250
G1 X141.120 Y49.963 F9000.000
G1 E-4.00000 F2400.000
G1 X162.928 Y54.015 F9000.000
G1 E4.00000 F900.000
M204 S1000
;TYPE:Top solid infill
;WIDTH:0.404487
G1 F1450.455
G1 X162.341 Y54.603 E0.02219
G1 X161.814 Y54.603 E0.01406
G1 X162.763 Y53.654 E0.03582
G1 X162.763 Y53.128 E0.01406
G1 X161.288 Y54.603 E0.05570
```

G1 X160.761 Y54.603 E0.01406

G1 X162.763 Y52.601 E0.07558

G1 X162.763 Y52.075 E0.01406

G1 X160.235 Y54.603 E0.09546

G1 X159.708 Y54.603 E0.01406

G1 X162.763 Y51.548 E0.11534

G1 X162.763 Y51.022 E0.01406

G1 X159.182 Y54.603 E0.13522

G1 X158.655 Y54.603 E0.01406

G1 X162.763 Y50.495 E0.15510

G1 X162.763 Y49.969 E0.01406

G1 X158.129 Y54.603 E0.17498

G1 X157.602 Y54.603 E0.01406

G1 X162.763 Y49.442 E0.19486

G1 X162.763 Y48.916 E0.01406

G1 X157.076 Y54.603 E0.21474

G1 X156.549 Y54.603 E0.01406

G1 X162.763 Y48.389 E0.23462

G1 X162.763 Y47.863 E0.01406

G1 X156.299 Y54.326 E0.24405

G1 X156.143 Y53.956 E0.01073

G1 X162.763 Y47.336 E0.24995

G1 X162.763 Y46.809 E0.01406

G1 X155.981 Y53.591 E0.25607

G1 X155.789 Y53.257 E0.01029

G1 X162.763 Y46.283 E0.26334

G1 X162.763 Y45.756 E0.01406

G1 X155.596 Y52.923 E0.27060

G1 X155.376 Y52.617 E0.01007

```
G1 X162.763 Y45.230 E0.27893
G1 X162.763 Y44.703 E0.01406
G1 X155.149 Y52.317 E0.28748
G1 X154.909 Y52.031 E0.00998
G1 X162.763 Y44.177 E0.29657
G1 X162.763 Y43.650 E0.01406
G1 X154.650 Y51.764 E0.30635
G1 X154.389 Y51.497 E0.00994
G1 X162.763 Y43.124 E0.31617
G1 X162.763 Y42.597 E0.01406
G1 X154.098 Y51.262 E0.32718
G1 X153.806 Y51.027 E0.01000
G1 X162.763 Y42.071 E0.33819
G1 X162.763 Y41.544 E0.01406
G1 X153.493 Y50.814 E0.35000
G1 X153.168 Y50.612 E0.01021
G1 X162.316 Y41.465 E0.34540
G1 X161.789 Y41.465 E0.01406
G1 X152.833 Y50.421 E0.33818
G1 X152.473 Y50.255 E0.01060
G1 X161.263 Y41.465 E0.33191
G1 X160.736 Y41.465 E0.01406
G1 X152.102 Y50.099 E0.32602
G1 X151.707 Y49.967 E0.01111
G1 X160.210 Y41.465 E0.32104
G1 X159.683 Y41.465 E0.01406
G1 X151.313 Y49.835 E0.31607
G1 X150.874 Y49.748 E0.01195
G1 X159.157 Y41.465 E0.31275
```

```
G1 X158.630 Y41.465 E0.01406
G1 X150.430 Y49.665 E0.30962
G1 X149.940 Y49.629 E0.01313
G1 X158.104 Y41.465 E0.30826
G1 X157.577 Y41.465 E0.01406
G1 X149.439 Y49.603 E0.30728
G1 X148.913 Y49.603 E0.01406
G1 X152.900 Y45.616 E0.15053
G1 X153.612 Y45.180 E0.02230
G1 X154.395 Y44.567 E0.02656
G1 X155.024 Y43.917 E0.02414
G1 X155.564 Y43.201 E0.02397
G1 X155.902 Y42.613 E0.01810
G1 X157.051 Y41.465 E0.04335
G1 X156.524 Y41.465 E0.01406
G1 X156.027 Y41.962 E0.01878
M204 S1250
G1 X152.261 Y45.728 F9000.000
M204 S1000
G1 F1450.455
G1 X148.386 Y49.603 E0.14631
G1 X147.860 Y49.603 E0.01406
G1 X151.205 Y46.258 E0.12629
G1 X150.557 Y46.379 E0.01759
G1 X147.333 Y49.603 E0.12171
G1 X146.807 Y49.603 E0.01406
G1 X149.973 Y46.437 E0.11954
G1 X149.418 Y46.465 E0.01483
G1 X146.280 Y49.603 E0.11848
```

G1 X145.754 Y49.603 E0.01406

G1 X148.892 Y46.465 E0.11848

G1 X148.365 Y46.465 E0.01406

G1 X145.227 Y49.603 E0.11848

G1 X144.701 Y49.603 E0.01406

G1 X147.839 Y46.465 E0.11848

G1 X147.312 Y46.465 E0.01406

G1 X144.174 Y49.603 E0.11848

G1 X143.648 Y49.603 E0.01406

G1 X146.786 Y46.465 E0.11848

G1 X146.259 Y46.465 E0.01406

G1 X143.121 Y49.603 E0.11848

G1 X142.595 Y49.603 E0.01406

G1 X145.733 Y46.465 E0.11848

G1 X145.206 Y46.465 E0.01406

G1 X142.068 Y49.603 E0.11848

G1 X141.625 Y49.603 E0.01184

G1 X141.625 Y49.519 E0.00222

G1 X144.680 Y46.465 E0.11534

G1 X144.153 Y46.465 E0.01406

G1 X141.625 Y48.993 E0.09546

G1 X141.625 Y48.466 E0.01406

G1 X143.627 Y46.465 E0.07557

G1 X143.100 Y46.465 E0.01406

G1 X141.625 Y47.940 E0.05569

G1 X141.625 Y47.413 E0.01406

G1 X142.574 Y46.465 E0.03581

G1 X142.047 Y46.465 E0.01406

G1 X141.460 Y47.052 E0.02218

M204 S1250

; stop printing object Petg print.STL id:28 copy 0

G1 E-4.00000 F2400.000

M107

;TYPE:Custom

; Filament-specific end gcode

; Filament-specific end gcode

G1 E-1 F2100 ; retract

G1 Z2.95 F720 ; Move print head up

G1 X178 Y178 F4200 ; park print head

G1 Z30.95 F720 ; Move print head further up

G4 ; wait

M104 S0 ; turn off temperature

M140 S0 ; turn off heatbed

M107 ; turn off fan

M221 S100 ; reset flow

M900 K0 ; reset LA

M84 ; disable motors

M73 P100 R0

; filament used [mm] = 1483.55, 1244.42

; filament used [cm3] = 3.57, 2.99

; filament used [g] = 4.35, 3.80

; filament cost = 0.36, 0.11

; total filament used [g] = 8.15

; total filament cost = 0.46

; estimated printing time (normal mode) = 1h 8m 14s

; avoid_crossing_perimeters = 0

; avoid_crossing_perimeters_max_detour = 0

; bed_custom_model =

; bed_custom_texture =

; bed_shape = 0x0,180x0,180x180,0x180

; bed_temperature = 50,90

; before_layer_gcode = ;BEFORE_LAYER_CHANGE\nG92 E0.0\n;[layer_z]\n\n

; between_objects_gcode =

; bottom_fill_pattern = monotonic

; bottom_solid_layers = 5

; bottom_solid_min_thickness = 0.5

; bridge_acceleration = 1000

; bridge_angle = 0

; bridge_fan_speed = 80,50

; bridge_flow_ratio = 0.85

; bridge_speed = 30

; brim_width = 0

; clip_multipart_objects = 1

; color_change_gcode = M600

; complete_objects = 0

; cooling = 0,1

; cooling_tube_length = 5

; cooling_tube_retraction = 91.5

; default_acceleration = 1250

; deretract_speed = 40,40

; disable_fan_first_layers = 4,3

; dont_support_bridges = 1

; draft_shield = 0

; duplicate_distance = 6

; elefant_foot_compensation = 0.2

; end_gcode = G1 E-1 F2100 ; retract\n{if max_layer_z < max_print_height}G1 Z{z_offset+min(max_layer_z+2, max_print_height)}{endif} F720 ; Move print head up\nG1 X178 Y178 F4200 ; park print head\n{if max_layer_z < max_print_height}G1 Z{z_offset+min(max_layer_z+30, max_print_height)}{endif} F720 ; Move print head further up\nG4 ; wait\nM104 S0 ; turn off temperature\nM140 S0 ; turn off heatbed\nM107 ; turn off fan\nM221 S100 ; reset flow\nM900 K0 ; reset LA\nM84 ; disable motors

; ensure_vertical_shell_thickness = 1

; external_perimeter_extrusion_width = 0.45

; external_perimeter_speed = 30

; external_perimeters_first = 0

; extra_loading_move = -2

; extra_perimeters = 0

; extruder_clearance_height = 20

; extruder_clearance_radius = 35

; extruder_colour = ;

; extruder_offset = 0x0,0x0

; extrusion_axis = E

; extrusion_multiplier = 1.15,1

; extrusion_width = 0.45

; fan_always_on = 1,1

; fan_below_layer_time = 100,20

; filament_colour = #F2F200;#FF8000

; filament_cooling_final_speed = 3.4,3.4

; filament_cooling_initial_speed = 2.2,2.2

; filament_cooling_moves = 4,4

; filament_cost = 82.26,27.82

; filament_density = 1.22,1.27

; filament_deretract_speed = 15,25

; filament_diameter = 1.75,1.75

; filament_load_time = 0,0

; filament_loading_speed = 28,28

; filament_loading_speed_start = 3,3

; filament_max_volumetric_speed = 1.35,7

; filament_minimal_purge_on_wipe_tower = 15,15

; filament_notes = ;

; filament_ramming_parameters = "120 100 6.6 6.8 7.2 7.6 7.9 8.2 8.7 9.4 9.9 10.0| 0.05 6.6 0.45 6.8 0.95 7.8 1.45 8.3 1.95 9.7 2.45 10 2.95 7.6 3.45 7.6 3.95 7.6 4.45 7.6 4.95 7.6";"120 100 6.6 6.8 7.2 7.6 7.9 8.2 8.7 9.4 9.9 10.0| 0.05 6.6 0.45 6.8 0.95 7.8 1.45 8.3 1.95 9.7 2.45 10 2.95 7.6 3.45 7.6 3.95 7.6 4.45 7.6 4.95 7.6"

; filament_retract_before_travel = 7,1

; filament_retract_length = 4,nil

; filament_retract_lift = 0,nil

; filament_retract_speed = 40,40

; filament_settings_id = "Fillamentum Flexfill 98A ";"Generic PETG "

; filament_soluble = 0,0

; filament_spool_weight = 230,0

; filament_toolchange_delay = 0,0

; filament_type = FLEX;PETG

; filament_unload_time = 0,0

; filament_unloading_speed = 90,90

; filament_unloading_speed_start = 100,100

; filament_vendor = Fillamentum

; filament_wipe = 0,nil

; fill_angle = 45

; fill_density = 15%

; fill_pattern = gyroid

; first_layer_acceleration = 800

; first_layer_bed_temperature = 50,85

; first_layer_extrusion_width = 0.42

; first_layer_height = 0.2

; first_layer_speed = 20

; first_layer_temperature = 240,230

; full_fan_speed_layer = 6,5

; gap_fill_speed = 40

; gcode_comments = 0

; gcode_flavor = marlin

; gcode_label_objects = 1

; high_current_on_filament_swap = 0

; host_type = octoprint

; infill_acceleration = 1000

; infill_anchor = 2.5

; infill_anchor_max = 12

; infill_every_layers = 1

; infill_extruder = 1

; infill_extrusion_width = 0.45

; infill_first = 0

; infill_only_where_needed = 0

; infill_overlap = 25%

; infill_speed = 80

; interface_shells = 0

; ironing = 0

; ironing_flowrate = 15%

; ironing_spacing = 0.1

; ironing_speed = 15

; ironing_type = top

; layer_gcode = ;AFTER_LAYER_CHANGE\n;[layer_z]

; layer_height = 0.15

; machine_limits_usage = emit_to_gcode

; machine_max_acceleration_e = 5000

; machine_max_acceleration_extruding = 1250

; machine_max_acceleration_retracting = 1250

; machine_max_acceleration_x = 1250

; machine_max_acceleration_y = 1250

; machine_max_acceleration_z = 400

; machine_max_feedrate_e = 80

; machine_max_feedrate_x = 180

; machine_max_feedrate_y = 180

; machine_max_feedrate_z = 12

; machine_max_jerk_e = 10

; machine_max_jerk_x = 8

; machine_max_jerk_y = 8

; machine_max_jerk_z = 2

; machine_min_extruding_rate = 0

; machine_min_travel_rate = 0

; max_fan_speed = 50,50

; max_layer_height = 0.25,0.25

; max_print_height = 180

; max_print_speed = 150

; max_volumetric_speed = 0

; min_fan_speed = 50,30

; min_layer_height = 0.07,0.07

; min_print_speed = 15,15

; min_skirt_length = 4

; notes =

; nozzle_diameter = 0.4,0.4

; only_retract_when_crossing_perimeters = 0

; ooze_prevention = 0

; output_filename_format =
{input_filename_base}_{layer_height}mm_{filament_type[0]}_{printer_model}_{print_time}.gcode

; overhangs = 0

; parking_pos_retraction = 92

; pause_print_gcode = M601

; perimeter_acceleration = 800

; perimeter_extruder = 1

; perimeter_extrusion_width = 0.45

; perimeter_speed = 40

; perimeters = 2

; physical_printer_settings_id =

; post_process =

; print_settings_id = 0.1mm QUALITY @cura

; printer_model = cura

; printer_technology = FFF

; printer_variant = 0.4

; printer_vendor =

; raft_layers = 0

; remaining_times = 1

; resolution = 0

; retract_before_travel = 1.5,1.5

; retract_before_wipe = 70%,70%

; retract_layer_change = 1,1

; retract_length = 3.2,3.2

; retract_length_toolchange = 4,4

; retract_lift = 0.2,0.2

; retract_lift_above = 0,0

; retract_lift_below = 179,179

; retract_restart_extra = 0,0

; retract_restart_extra_toolchange = 0,0

; retract_speed = 70,70

; seam_position = nearest

; silent_mode = 0

; single_extruder_multi_material = 0

; single_extruder_multi_material_priming = 1

; skirt_distance = 2

; skirt_height = 3

; skirts = 1

; slice_closing_radius = 0.049

; slowdown_below_layer_time = 15,15

; small_perimeter_speed = 25

; solid_infill_below_area = 0

; solid_infill_every_layers = 0

; solid_infill_extruder = 1

; solid_infill_extrusion_width = 0.45

; solid_infill_speed = 80

; spiral_vase = 0

; standby_temperature_delta = -5

; start_filament_gcode = "M900 K0 ; Filament gcode";"M900 K{if nozzle_diameter[0]==0.6}0.12{elsif nozzle_diameter[0]==0.8}0.06{else}0.2{endif} ; Filament gcode"

; start_gcode = G90 ; use absolute coordinates\nM83 ; extruder relative mode\nM104 S170 ; set extruder temp for bed leveling\nM140 S[first_layer_bed_temperature] ; set bed temp\nM109 R170 ; wait for bed leveling temp\nM190 S[first_layer_bed_temperature] ; wait for bed temp\nG28 ; home all without mesh bed level\nG29 ; mesh bed leveling \nM104 S[first_layer_temperature] ; set extruder temp\nG92 E0.0\nG1 Y-2.0 X179 F2400\nG1 Z3 F720\nM109 S[first_layer_temperature] ; wait for extruder temp\n\n; intro line\nG1 X170 F1000\nG1 Z0.2 F720\nG1 X110.0 E8.0 F900\nG1 X40.0 E10.0 F700\nG92 E0.0\n\nM221 S95 ; set flow

; support_material = 0

; support_material_angle = 0

; support_material_auto = 1

; support_material_buildplate_only = 0

; support_material_contact_distance = 0.1

; support_material_enforce_layers = 0

; support_material_extruder = 0

; support_material_extrusion_width = 0.35

; support_material_interface_contact_loops = 0

; support_material_interface_extruder = 0

; support_material_interface_layers = 2

; support_material_interface_spacing = 0.2

; support_material_interface_speed = 80%

; support_material_pattern = rectilinear

; support_material_spacing = 2

; support_material_speed = 40

; support_material_synchronize_layers = 0

; support_material_threshold = 55

; support_material_with_sheath = 0

; support_material_xy_spacing = 60%

; temperature = 240,240

; template_custom_gcode =

; thin_walls = 0

; threads = 8

; thumbnails = 16x16,220x124

; toolchange_gcode = M600

; top_fill_pattern = monotonic

; top_infill_extrusion_width = 0.4

; top_solid_infill_speed = 40

; top_solid_layers = 7

; top_solid_min_thickness = 0.7

; travel_speed = 150

; use_firmware_retraction = 0

; use_relative_e_distances = 1

; use_volumetric_e = 0

; variable_layer_height = 1

; wipe = 1,1

; wipe_into_infill = 0

; wipe_into_objects = 0

; wipe_tower = 0

; wipe_tower_bridging = 10

; wipe_tower_no_sparse_layers = 0

; wipe_tower_rotation_angle = 0

; wipe_tower_width = 60

; wipe_tower_x = 170

; wipe_tower_y = 140

; wiping_volumes_extruders = 70,70,70,70

; wiping_volumes_matrix = 0,140,140,0

; xy_size_compensation = 0

; z_offset = 0
